# Therapeutic roles of plants for 15 hypothesised causal bases of Alzheimer’s disease

**DOI:** 10.1007/s13659-022-00354-z

**Published:** 2022-08-23

**Authors:** Sheena E. B. Tyler, Luke D. K. Tyler

**Affiliations:** 1 John Ray Research Field Station, Cheshire, UK; 2 School of Natural Sciences, Bangor University, Gwynedd, UK

**Keywords:** Medicinal plants, Alzheimer’s, Causal basis, Ethnomedicine, Traditional knowledge

## Abstract

Alzheimer’s disease (AD) is progressive and ultimately fatal, with current drugs failing to reverse and cure it. This study aimed to find plant species which may provide therapeutic bioactivities targeted to causal agents proposed to be driving AD. A novel toolkit methodology was employed, whereby clinical symptoms were translated into categories recognized in ethnomedicine. These categories were applied to find plant species with therapeutic effects, mined from ethnomedical surveys. Survey locations were mapped to assess how this data is at risk. Bioactivities were found of therapeutic relevance to 15 hypothesised causal bases for AD. 107 species with an ethnological report of memory improvement demonstrated therapeutic activity for all these 15 causal bases. The majority of the surveys were found to reside within biodiversity hotspots (centres of high biodiversity under threat), with loss of traditional knowledge the most common threat. Our findings suggest that the documented plants provide a large resource of AD therapeutic potential. In demonstrating bioactivities targeted to these causal bases, such plants may have the capacity to reduce or reverse AD, with promise as drug leads to target multiple AD hallmarks. However, there is a need to preserve ethnomedical knowledge, and the habitats on which this knowledge depends.

**Graphical Abstract:**

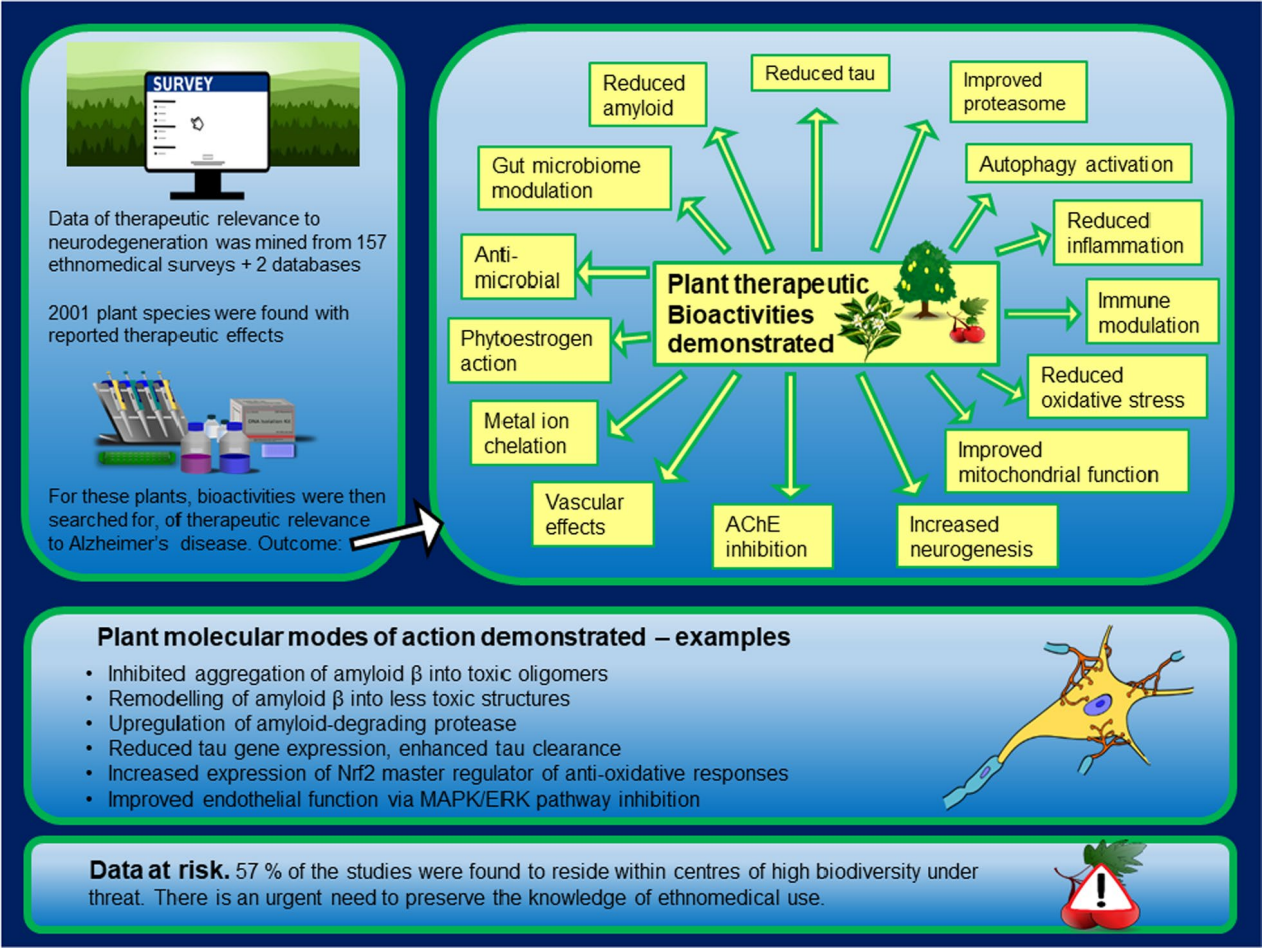

**Supplementary Information:**

The online version contains supplementary material available at 10.1007/s13659-022-00354-z.

## Introduction

The global incidence of Alzheimer’s disease (AD) and other dementias is 43·8 million and rising, and a cause of 2·4 million deaths annually [1]. AD is also recalcitrant against modern pharmacological interventions, with a failure of treatments to reverse and cure disease progression [2–6]. Therapeutic strategies remain limited due to a lack of knowledge of the precise mechanisms underlying the observed pathology [7]. For Alzheimer’s treatment from 2002 to 2012, 413 clinical trials were performed, assessing 221 agents, with none being found to show disease-modifying potential [8]. The US Food and Drug Administration licensed only one AD drug in that duration (memantine), which provides just a minor clinical benefit [9]. Subsequently in 2021 the monoclonal antibody (mAb) Aducanumab was licensed by the U.S. Food and Drug Administration (FDA) for AD treatment, based on demonstrating amyloid reduction, but the drug has limited impact on reducing cognitive decline and disease progression (reviewed by [10, 11]). Trials with other mAbs, vaccines and other agents are still ongoing, with some providing symptomatic relief, but none showing strong evidence of halting the disease (reviewed by [5]).

There is thus a search for more effective drugs, and evidence is mounting that plants may provide such a source. Of the new therapeutic drugs approved by the FDA and similar organizations in several of the years from 1981 to 2019, 50% of all approvals were derived from natural products, including plants [12, 13]. However, it is likely that the bioactivity of most plant species remains to be investigated [14], and this represents a huge untapped resource.

Of the most useful drugs derived from plants, 80% were discovered by follow-up of ethnomedical uses (plants used in traditional medical practices) [15]. Screening indigenous community ethnomedicine data can increase the “hit rate” for discovery of novel active compounds [16]. This is because it is the application of a knowledge-based strategy to detect therapeutic potential. This is in contrast to the screening of natural compounds at random, which has a low hit rate for identification of relevant bioactivity [14]. Moreover, for drugs derived from ethnomedicine, ethnomedical uses can provide insight of efficacy and safety [17], often long-established over many generations.

This study aimed firstly to find and document plant species with reported therapeutic effects of AD relevance. A toolkit methodology was applied, which involved construction of therapeutic categories which could be recognized by ethnomedical practitioners. These categories were then applied to mine ethnological data in search of therapeutic potential of relevance to neurodegenerative diseases (NDs). The rationale for this is to attain a wide set of relevant terms to maximise the mining of therapeutic data. Although some hallmarks and symptoms, such as memory impairment, are easily recognised by both clinicians and ethnomedical practitioners, certain hallmarks such as neurotoxicity, of central importance in numerous NDs, cannot be easily translated into terms in ethnomedical use. Anti-neurotoxic effects in plants may indeed exist, which could be revealed from a wider probing of the many medicinal effects reported. The relevance of the findings to a wider range of ND diseases are reported separately. This study focuses on the relevance of the findings to AD.

A second aim was to map the geographical locations of the mined ethnomedical surveys, to assess how this ethnomedical data may be at risk, since mapping anthropogenic threats is a key tool to guide management of these threats [18]. The world’s greatest biodiversity hotspots (BDH) are centres of high biodiversity that are under threat [19, 20], defined as having lost at least 70% of their primary native vegetation (https://www.cepf.net/our-work/biodiversity-hotspots/hotspots-defined). In most hotspots, it is estimated that less than 10% of natural intact vegetation remains [21]. The survey locations were mapped to establish how many were located in these BDHs and therefore at elevated risk. The surveys were examined systematically to discover what the threats were of most concern to the authors, to inform responses appropriate to how any valuable ethnomedical data of relevant therapeutic potential can be preserved.

The leading factor in AD pathogenesis remains unknown [22]. Numerous hypotheses have been proposed in which the disease is postulated to be initiated or driven by a particular causal agent (reviewed by [23, 24]). We were interested in finding what specific causal agents AD can be attributed to, since these provide a focus to be targeted therapeutically. Our third aim was thus to search the literature for various causal hypotheses for Alzheimer’s disease, for which plant species mined in this study may provide bioactivities of AD therapeutic potential. The implication is that if plants can be found which can target the underlying causes of neurodegeneration driving AD pathogenesis, this could be of fundamental importance in the search for more effective therapies, since to date no drugs halt and remediate disease progression.

## Results and discussion

### Overview

2001 plant species were identified with reported uses for alleviating pathologies relevant to NDs, by application of the toolkit methodology (Additional files 1: Additional Table S1, 2: Table S2). Bioactivities of therapeutic relevance were discovered from the literature for 1339 of these 2001 species (67%) (Additional file 3: Table S3). Bioactivities were found for every one of the toolkit categories, and also beyond the toolkit categories (i.e., species with an ethnomedical use demonstrating other bioactivities of ND therapeutic potential). This paper focuses on the relevance of the data to AD.

We found that plant bioactivities were found of therapeutic relevance to 15 hypothesised causal bases for AD (Table 1). This plant therapeutic potential for a wide range of causal agents implicated in driving AD pathogenesis is evidence that the toolkit methodology is useful for providing a wide reach in the search of this potential.

**Table 1:** Summary: number of medicinal plant species with therapeutic bioactivity vs. proposed AD causal agents

Causal hypothesis	Number of medicinal plant species with therapeutic bioactivity vs. hypothesised causal agent
Amyloid hypothesis	46
Tau hypothesis	18
Ubiquitin–proteasome hypothesis	8
Impaired autophagy hypothesis	7
Inflammation hypothesis	694
Immune dysregulation hypothesis	46
Oxidative Stress hypothesis	218
Mitochondria hypothesis	27
Neurogenic hypothesis	30
AChE inhibition	33
Vascular hypothesis	
Hypertension	124
Atherosclerosis	11
Dyslipidemia/high cholesterol	50
Platelet aggregation/thrombolytic	45
Metal ion hypothesis	29
Oestrogen hypothesis	19
Infection hypothesis	768
Gut microbiome hypothesis	3

### Plant bioactivities of relevance to AD causal hypotheses

The study found reports of plant therapeutic bioactivities potentially relevant to the following hypothesised causal bases and dysregulated processes. In this section the therapeutic effects are documented in more detail.

#### The amyloid hypothesis

The dominant model of AD pathogenesis is the amyloid hypothesis, in which the accumulation of Aβ is proposed to be causal [25, 26]. In the AD brain, lesions known as neuritic plaques are found, consisting of microscopic foci of amyloid protein deposition [27]. George Glenner identified a distinctive amyloid β (Aβ) peptide found in these deposits [28], and proposed that the Aβ causes destruction of neuronal fibres, which is intrinsic to the ensuing dementia of AD [29]. This pathology has come to be associated with aberrant metabolism of the amyloid precursor protein (APP) [30]. These findings have led to the amyloid cascade hypothesis, in which an imbalance between production and clearance of Aβ peptides initiates the complex pathological cascade of AD [31, 32]. A variant of the cascade view is the amyloid-β oligomer (AβO) hypothesis, which postulates that the brain damage of AD is instigated by toxic soluble amyloid oligomers [33]. Substantial evidence supports these hypotheses: for instance, mutations in APP lead to more aggressive AD; humans with Down’s syndrome have 3 copies of APP and invariably develop AD; patients with an APP mutation that decreases Aβ are associated with reduced AD and cognitive decline; and in animal and cell models Aβ induces tau hyperphosphorylation, reduces synapse density and impairs memory; and blocking AβO production reverses synapse loss and memory impairment in APP mice (reviewed by [25, 34]). Moreover, various Aβ monoclonal antibodies (mAb) such as Aducanumab reduce brain Aβ brain deposits and result in small clinical improvements (reviewed by [35]). Such evidence provides a rationale for the targeting of Aβ in AD disease pathology, but there is a scepticism that mAb treatments reduce cognitive decline, in spite of the Food and Drug Administration in the United States licencing Aducanumab for AD treatment [11, 36]. Clinical effects of mAb also significantly increase the risk of adverse events (https://www.eisai.com/news/2018/news201866.html accessed 25 Oct 2021).

The search thus remains for alternative, more effective treatments to target AD amyloid pathology. Of therapeutic relevance to this, 46 plant species (Table 2) and 42 phytochemicals (Additional file 5: Table S5) have been found to demonstrate anti-amyloid activity in a variety of human cell line, animal in vivo and in vitro studies. Seven of these species (*Allium sativum, Bacopa monnieri, Centella asiatica, Cocos nucifera, Convolvulus prostratus, Moringa oleifera* and *Rosmarinus officinalis* all demonstrated both memory/ cognitive improvement and anti-amyloid activities, providing evidence that the anti-amyloid activity may be of therapeutic effect in alleviating AD memory/cognitive dysfunction.

**Table 2:** A range of anti-amyloid activities demonstrated by medicinal plants

Treatment outcome	Species*	Model	References
Reduced CSF Aβ40	*Vitis vinifera* [resveratrol]	Clinical trial	[37]
APP processing toward non-amyloidogenic pathway	*Apium graveolens*	In vivo mouse	[38]
Suppressed amyloid protein precursor gene expression/ APP levels	*Convolvulus prostratus*	In vivo AD rat	[39]
	*Coptis chinensis*	In vivo AD mouse	[40]
	*Silybum marianum*	In vivo AD rat	[41]
	*Phyllanthus emblica*	In vivo AD rat	[42]
Inhibits amyloid Aβ aggregation	*Fragaria x ananassa*	Mouse microglia, in vitro	[43]
	*Capsicum annuum*	In vitro	[44]
	*Opuntia ficus-indica*	In vivo AD *Drosophila melanogaster, Saccharomyces cerevisiae*	[45]
	*Scoparia dulcis*	In vitro	[46]
	*Uncaria rhynchophylla*	In vivo AD mouse	[47]
	*Cornus officinalis, Cyperus rotundus, Myristica fragrans, Paeonia lactiflora, Prunella vulgaris*	In vitro	[48]
	*Mentha spicata, Satureja thymbra, Thymus vulgaris*	In vitro	[49]
Reduced Aβ production by reduced expression of β -site APP cleaving enzyme 1 (BACE1)	*Asparagus racemosus*	In vitro	[50]
	*Elsholtzia rugulosa*	In vivo AD mouse	[51]
	*Chromolaena odorata*	In vivo AD mouse	[52]
	*Moringa oleifera*	In vivo rat	[53]
Upregulation of amyloid-degrading protease	*Morus alba*	In vivo AD mouse	[54]
Inhibits β-secretase activity	*Capsicum annuum*	In vitro	[44]
Inhibition of fibrillogenesis	*Allium roseum*	Human cell line*, *in vitro	[55]
	*Bacopa monnieri*	In vitro	[56]
	*Cuminum cyminum*	Rat neuron	[57]
	*Pistacia lentiscus*	In vitro	[58]
	*Salvia miltiorrhiza*	Human neuron cell line; in vitro	[59]
inhibits aggregation of Aβ into toxic oligomers/ attenuated Aβ oligomer neurotoxicity/decreased oligomer deposition	*Cocos nucifera*	In vivo AD mouse	[60]
	*Elaeis guineensis*	In vitro*,* AD *Saccharomyces cerevisiae*	[61]
	*Garcinia mangostana*	Rat neuron	[62]
	*Olea europaea*	In vivo AD *Caenorhabditis elegans*	[63]
	*Rosmarinus officinalis*	In vivo AD mouse	[64]
	*Uncaria tomentosa*	a/vivo rat, mouse	[65]
Remodelling of Aβ fibrils into less toxic structures	*Caesalpinia sappan*	Human neuronal cell line; in vitro	[66]
	*Vitis vinifera*	In vitro	[67]
Reversal of plaque pathology	*Withania. somnifera*	In vivo AD mouse	[68]
Decreased plaque burden/ reduced Aβ accumulation or deposition	*Panax ginseng*	In vivo AD mouse; human cell line	[69]
	*Panax quinquefolius*	Hamster cell line, in vivo AD mouse	[70]
	*Camellia sinensis*	In vivo AD mouse	[71]
	*Centella asiatica*	In vivo AD mouse	[72]
	*Curcuma longa*	In vivo AD mouse, mouse microglia	[73]
	*Fibraurea recisa*	In vivo AD mouse, in vitro	[74]
Enhanced clearance of Aβ/ cathepsin B upregulation	*Vaccinium myrtillus*	In vivo AD mouse	[75]
	*Cajanus cajan*	In vivo AD mouse	[76]

*[or extracted phytochemical]. *Aβ* amyloid β, *CSF* cerebro-spinal fluid

The therapeutic effects result from a variety of mechanisms, which provide potential to target the various amyloid ligands and amyloidogenic processes. For instance, in a randomized, placebo-controlled, double-blind, multicentre 52-week phase 2 trial of resveratrol (Table 3) in individuals with mild to moderate AD, CSF Aβ40 levels significantly declined [37].

**Table 3:** Examples of phytochemicals with therapeutic bioactivity for AD, with molecular structure indicated

Phytochemical	CAS RN	Molecular structure
Allicin	539-86-6	
Apigenin	520-36-5	
Berberine	2086-83-1	
Betulinic acid	472-15-1	
Brasilin	474-07-7	
Butylphthalide	6066-49-5	
Cajaninstilbene acid	87402-84-4	
Carnosol	5957-80-2	
Curcumin	458-37-7	
Epigallocatechin-3-gallate	989-51-5	
Fibrauretine	10605-02-4	
Genistein	446-72-0	
Ginsenoside RG2	52286-74-5	
Morin	480-16-0	
Resveratrol	501-36-0	
Rosmarinic acid	20283-92-5	
Rutin	153-18-4	
Sarsasapogenin	126-19-2	
Silybin	22888-70-6	
Sominone	98569-64-3	
Sulforaphane	4478-93-7	
Urolithin A	1143-70-0	

*CAS RN* Chemical Abstracts Service Registry Number

In various pre-clinical studies, treatment with *Cornus officinalis, Cyperus rotundus, Fragaria x ananassa, Opuntia ficus-indica* and *Satureja hortensis* inhibited Aβ aggregation [43, 45, 48, 49]. Decrease in amyloid plaque deposition resulted from treatment with *Centella asiatica* [72] and *Coptis chinensis* [40]. Rosmarinic acid (Table 3) (from sources such as *Rosmarinus officinalis*) decreased brain deposition of A11-positive Aβ oligomers [64]. *Uncaria tomentosa* disaggregated preformed Aβ fibrils [65]. *Cajanus cajan* stimulated amyloid β clearance [76]. *Bacopa monnieri* inhibited Aβ42 fibrillogenesis [56]. In mouse neurons exposed with metal–associated Aβ, EGCG (Epigallocatechin-3-gallate) (Table 3) increased cell survival [77] (Additional file 5: Table S5).

Molecular mechanisms have also been elucidated. *Moringa oleifera* decreased amyloid production via β -site APP cleaving enzyme (BACE1) downregulation [53]. *Morus alba* reduced cerebral Aβ production and Aβ plaque burden via upregulation of amyloid-degrading protease (e.g., NEP, IDE) [54]. *Olea europaea* blocked formation of toxic Aβ oligomers [63]. *Phyllanthus emblica and* silymarin (a mixture containing mainly silybin) (Table 3) from *Silybum marianum* reduced expression of amyloid precursor protein [41, 42]. *Vaccinium myrtillus* inhibited aggregation of Aβ1–42 via suppression of p44/42 MAPK [75]. Resveratrol remodelled Aβ into non-toxic structures [67]. Similarly, brasilin (Table 3) (from *Caesalpinia sappan)* remodelled Aβ fibrils into less toxic aggregates [66]. *Apium graveolens* treatment led to amyloid precursor protein (APP) processing toward a non-amyloidogenic pathway [38].

However, there is a body of evidence that confutes the amyloid cascade hypothesis as the central event in AD pathogenesis (reviewed by [78, 79]). For instance, there are numerous human subjects who were cognitively normal (without clinical expression of AD) despite harbouring brain amyloid plaque deposits [80–82]. Moreover, mice genetically engineered to produce brain amyloid deposits do not demonstrate neurodegeneration or cognitive decline [83], suggesting that Aβ does not provide a sufficient cause for the complex symptoms of AD [84] (for counter-arguments to these and other objections, see [25]).

#### The tau hypothesis

An alternative framework is the tau hypothesis, which states that the principle causative substance of AD is tau, not Aβ (reviewed by [79]). Tau is a protein regulating the function of microtubules, its microtubule binding affinity being determined by its phosphorylation [85]. In AD, tau becomes hyperphosphorylated, aggregating into toxic neurofibrillary tangles (NFTs) within neurons [86, 87]. Moreover, tau may have a pathogenic role in mediating Aβ toxicity in AD [88]. Tau hyperphosphorylation may be induced by various factors, such as impaired glucose metabolism [89]. Evidence for a causative role for tau is suggested by an association between the spreading of pathological tau and the patterns of neurodegeneration, and that tau lesions occur prior to Aβ accumulation (reviewed by [79]).

Of therapeutic relevance, at least 20 plant species or their associated phytochemical extracts have demonstrated anti-tau bioactivity in various pre-clinical models (Table 4), via several mechanisms.

**Table 4:** A range of anti-tau activities demonstrated by medicinal plants

Treatment outcome	Species [or extracted phytochemical]	Model	References
Reduced tau phosphorylation	*Apium graveolens* [L-NBP]	Human cell line*, *in vivo mouse	[90]
	*Camellia sinensis* [EGCG]	In vivo AD mouse	[91]
	*Cinnamomum zeylanicum* [cinnamaldehyde]	In vitro	[92]
	Crataegus spp.* [Quercetin]	In vivo mouse	[93]
	*Fragaria x ananassa* [Fisetin]	mouse microglia, in vitro	[43]
	*Glycine max* [Genistein]	In vivo AD rat	[94]
	*Moringa oleifera*	In vivo rat	[53]
	*Morus alba* [Morin]	In vivo AD mouse	[54]
	*Olea europaea* [Oleocanthal]	In vitro	[95]
	*Psidium guajava*	In vitro	[96]
	*Rosmarinus officinalis*	In vitro	[97]
Reduced brain tau levels/reduced tau gene expression	*Cocos nucifera*	In vivo rat	[98]
	*Convolvulus prostratus*	In vivo rat	[39]
	*Curcuma longa*	In vivo AD mouse	[73]
	*Fibraurea recisa*	In vivo AD mouse, in vitro	[74]
	*Passiflora edulis*	In vivo mouse	[99]
	*Zataria multiflora*	In vivo rat	[100]
Disaggregates tau tangles/filaments	*Uncaria tomentosa*	In vivo rat, mouse	[65]
Reduced tau pathology	*Vitis vinifera* [Resveratrol]	In vivo mouse	[101]
Enhanced tau clearance	*Myrica cerifera* [Myricanol]	Human neural cell line	[102]

*Nominal species: good source of the phytochemical

For instance, in pre-clinical models, reduced tau hyperphosphorylation was demonstrated in treatments with either L-3-n-butylphthalide (L-NBP) (Table 3) (from *Apium graveolens*) [103], EGCG (Table 3) (from *Camellia sinensis*) [91] or genistein (Table 3) (from *Glycine max*) [94]. In all these examples, the anti-tau effects were associated with cognitive or memory improvements. Other species, such as *Convolvulus prostratus,* reduced tau gene expression [39]. *Uncaria tomentosa* disaggregated tau tangles/filaments [65]. Resveratrol (from species such as *Vitis vinifera*) reduced tau pathology [101] and myricanol (from *Myrica cerifera*) enhanced tau clearance [102].

Another possibility is the “dual pathway” model of causality, in which Aβ and tau may be linked to a common upstream driver [104], for which combined Aβ and tau-directed therapies have been proposed [105]. Plant species and derived phytochemicals demonstrating both anti-amyloid and anti-tau activity are attractive candidates for this combination strategy. Such species include *Curcuma longa, Fibraurea recisa, Fragaria ananassa, Moringa oleifera, Morus alba, Uncaria tomentosa,* and the phytochemicals berberine and resveratrol (Table 3) (Additional file 5: Table S5).

In some of the studies, anti-tau effects were associated with yet other activities of therapeutic relevance. For instance, with resveratrol treatment, reduced aberrant amyloid production and tau pathology were also associated with enhanced proteasome activity [101]. *Uncaria tomentosa* treatment led not only to anti-amyloid and anti-tau effects but also memory improvement and anti-inflammatory activity [65]. With *Moringa oleifera,* anti-amyloid and anti-tau effects were associated with rescued cognitive impairment and recovery of decreased synaptic proteins [53].

Molecular mechanisms have also been revealed. For example, mice treated with L-NBP led to reduced tau hyperphosphorylation at Ser199, Thr205, Ser396, and Ser404 sites. Also expressions of cyclin-dependent kinase and glycogen synthase kinase 3β, key kinases involved in tau phosphorylation, were reduced [90].

#### The ubiquitin–proteasome hypothesis

According to the ubiquitin–proteasome hypothesis, impairment of the ubiquitin–proteasome system, by which damaged proteins are dismantled, is at the root of neurodegenerative diseases such as AD [106]. A protein quality control (PQC) system consists chiefly of molecular chaperones such as heat shock proteins. These survey misfolded proteins, unfolding and refolding them into natively functional forms [107] (reviewed by [108]). Misfolded proteins that are unable to be refolded are degraded through two protein clearance pathways, the ubiquitin–proteasome system (UPS) and the autophagy-lysosome pathway (reviewed by [109]). In the UPS system, ubiquitin protein becomes conjugated to the misfolded protein, enabling the protein’s recognition and degradation within a multimeric enzyme cascade system known as the proteasome [110]. There is evidence for a central role of the UPS in AD pathology. For instance, in the AD brain, ubiquitinated proteins are found to accumulate, proteasome activity is decreased, and there is malfunction in the UPS pathway [111, 112], with a consequent impairment of neurotoxic protein clearance [113].

These systems are of intense interest for developing novel therapeutic interventions for AD [114, 115], and several plant species have demonstrated a role in the targeting of these pathways. Treatment with resveratrol enhanced mouse brain proteasome function, and this was associated with attenuation of aberrant amyloid production and reduced tau pathology [101]. Betulinic acid (Table 3) (from sources such as (*Betula pubescens* and *Ziziphus mauritiana*) [116] activated proteasome activity in human cell lines [117]. Sulforaphane (Table 3) (from sources such as *Brassica oleracea*) [118] mediated degradation of misfolded huntingtin protein in mice and human cell lines through the UPS pathway [119]. Sulforaphane was also found to ameliorate scopolamine-induced memory impairment in a rat model [120]. Mouse cell lines treated with sulforaphane protected cells from Aβ1–42-mediated cell death by upregulation of the 26S proteasome [121]. These evidences taken together suggest that these various phytochemicals have therapeutic potential for targeting proteasome impairment in AD.

#### Impaired autophagy hypothesis

Another hypothesis is that autophagy dysfunction plays an important role in AD pathophysiology [122]. If the UPS is impaired or cannot recognise the misfolded proteins, the misfolded proteins are destined for autophagy. This is a process of degradation and recycling of cell components within lysosomes [123], orchestrated by a complex network of proteins [124]. Autophagy dysfunction is implicated in AD (reviewed by [125]). Pharmacological agents acting to modulate autophagy are being explored for AD therapy [126, 127], and several plant species demonstrate this potential (Additional file 4: Table S4). For instance, in a clinical trial with resveratrol, the lysosomal/ phagosomal pathway was upregulated, indicating induction of autophagy [128]. Resveratrol also induced autophagy by directly inhibiting the mTOR-ULK1 pathway in an in vitro study [129]. Treatment of mice with the ginsenoside Rg2 (Table 3) (from *Panax ginseng*) induced autophagy, resulting in enhanced clearance of protein aggregates [69]. Berberine (from sources such as *Coptis chinensis, Phellodendron amurense* and *Hydrastis canadensis*) induced autophagy in numerous cell types including neurons, by mechanisms including AMPK/mTOR signaling upregulation [130, 131]. Phenolic acids from *Eucommia ulmoides* leaves may also activate autophagy via the autophagy regulators (Pink1, Beclin1, Ulk2, and Atg5) [132]. Urolithin A (Table 3) (from *Punica granatum*) induced autophagic flux in mouse and human neurons, which also contributed to inhibition of neuroinflammation [133].

#### The inflammation hypothesis

Inflammation is a normal host defence response triggered by damaging agents such as traumatic injury and invading pathogens, and is diminished once the tissue is repaired and resolved [134]. However, these normal mechanisms fail when there is an abnormal activation of inflammatory factors, leading to a chronic neuroinflammatory state, with harmful consequences [135]. The neuroinflammatory process involves the recruitment of numerous cellular and molecular immune components [136]. These include microglia and astrocytes, non-neuronal immune cells collectively known as glia, resident within the CNS. Microglia exhibit a surveillance function, with long processes in dynamic activity to constantly sense their surroundings [137]. This enables them to perform their housekeeping functions such as phagocytic engulfment of damaged tissue and elimination of pathogens [138]. During CNS damage or infection, microglia are activated and recruited to the site of insult, where they secrete small proteins called cytokines which can promote inflammation (pro-inflammatory) (e.g., IL-1, IL-6) or promote anti-inflammatory pathways (e.g., IL-4, IL-10) [134]. The secretion of proinflammatory cytokines can be beneficial, leading to the clearing of cell debris and promotion of regeneration [139]. However, disruption of microglial housekeeping (such as by persistent production of aberrant toxic proteins) leads to an exaggerated proinflammatory response [140]. This causes the microglia to shift to a reactive phenotype, secreting neurotoxins that kill neurons; hence correcting this maladaptive response may be a potential mode for disease-modifying therapy [141].

Astrocytes, comprising 25–50% of the brain volume, have a myriad of roles, such as ion homeostasis, neurotransmitter clearance, energy supply to neurons, synapse formation, remodelling of neural circuits [142], learning and memory [143], and the limiting of inflammation [144]. Astrocyte dysfunction has now been implicated in AD, associated with both loss-of-function and gain of toxicity phenotypes [145]. For instance, cytokine combinations such as TNF-α and IFN-γ stimulate astrocytes to generate Aβ, and since astrocytes outnumber neurons in the brain, astrocytes may be a significant source of Aβ during neuroinflammation in AD [146]. In an in vitro neuron-astrocyte co-culture, inhibition of astrocyte activation with an anti-inflammatory agent reduced the astrocytic inflammatory response and associated neuronal loss [147]. Astrocytes can thus be a therapeutic target for drug discovery [148].

The inflammation hypothesis for AD is based on the adverse effects of a pro-inflammatory brain microenvironment [149, 150], in which neuroinflammation (inflammation within the CNS) has a vital role in driving the pathogenesis and progression of AD [151]. A modification of this is the amyloid cascade—inflammation hypothesis, which envisages AD resulting from the inflammatory response induced by Aβ, later enhanced by aggregates of tau [152].

Supporting evidence for an inflammatory involvement in causality includes a reduced prevalence of AD in patients with rheumatoid arthritis treated with non-steroidal inflammatory drugs (NSAIDs) [153–155]; preceding clinical AD onset, an elevation of plasma inflammatory proteins [156] and microglial activation markers [157]; inflammatory markers co-localising with amyloid and tau deposition [158] (reviewed by [159]); and cognitively normal patients with profuse amyloid and tau deposits demonstrating lower levels of inflammation compared with AD patients [160].

Of relevance to the targeting of these neuroinflammatory processes, at least 21 plant species have been found to demonstrate anti-neuroinflammatory activity (Table 5).

**Table 5:** Examples of species with anti-neuroinflammatory bioactivity, which also validate reports of anti-inflammatory ethnological use

Treatment outcome	Model	Species [or phytochemical]	References
Reduced neuro-inflammation	In vivo rodent	*Fibraurea recisa*	[74]
		*Iresine diffusa*	[161]
		*Panax japonicus*	[162]
		*Peristrophe bicalyculata*	[163]
		*Withania somnifera*	[164]
		*Zingiber officinale*	[165]
	Mouse microglial cells	*Betula pendula*	[166]
		*Blumea balsamifera*	[167]
		*Capsella bursa-pastoris*	[168]
Reduced microglial + /astrocyte reactivity	Human neuronal cell line	*Camellia sinensis*	[169]
	In vivo mouse	*Pueraria montana var. lobata*	[170]
		*Cajanus cajan*	[76]
		*Olea europaea*	[171]
		*Vaccinium myrtillus*	[75]
	Mouse microglial cells	*Sambucus nigra*	[172]
NF-κB inhibition	Human cell line	*Lycium shawii*	[173]
	Mouse microglial cells	*Tussilago farfara*	[174]

For instance, rats treated with *Fibraurea recisa* showed anti-neuroinflammatory activity and also anti-amyloidogenic and anti-tau effects [74]. In another rat model, *Peristrophe bicalyculata* treatment led to anti-neuroinflammatory activity and reduced cognitive decline [163].

At least 9 species demonstrated reduced microglial or astrocyte activation (Additional file 4: Table S4), and this was associated with enhanced clearance of Aβ with *Vaccinium myrtillus* [75] (Table 5). Cajaninstilbene acid (Table 3) (from *Cajanus cajan*) reduced reactivity of both microglia and astrocytes, as well as stimulating Aβ clearance [76].

Evidence in favour of an anti-inflammatory involvement of AD causality based on therapeutic NSAID effects have been called into question. NSAIDs have failed to delay the onset of AD in adults with a family history of dementia [175]. There is also the possibility that AD does indeed develop less often in rheumatoid arthritis patients, but it is difficult to exclude the possibility that this is unrelated to anti-inflammatory drugs [176].

NSAIDs also increase the frequency of adverse health effects such as cardiotoxicity, upper gastrointestinal bleeding and perforation, notably with cyclooxygenase-2 (COX-2) inhibitors [177]. Medicinal plants demonstrating both COX and Lipoxygenase (LOX) inhibition bioactivity such as *Canarium patentinervium* may have less adverse effects, since there is evidence that dual COX and LOX inhibition reduces gastric and cardiovascular side effects [178].

Nuclear factor-κB (NF-κB) is a network hub consisting of a family of transcription factors [179, 180]. It serves as a pivotal mediator of inflammatory responses, inducing expression of various pro-inflammatory genes, and sustained NF-κB activation is integral to the persistence of inflammation [181]. Drug discovery units are searching for inhibitors of the NF-κB pathway as a pivotal target for AD pathologies [181, 182], thus medicinal plants demonstrating such activity are of therapeutic potential. For instance, treatment of mouse microglia with *Tussilago farfara* inhibited NF-κB inhibition and reduced microglial activation [174]. NF-κB inhibition was also demonstrated in a mouse microglia inflammation model treated with sulforophane [183]. However, targeting NF-κB may require cell-type specificity to preclude off-target deleterious effects [184].

#### The immune hypothesis

According to the immune hypothesis proposed by Fiala and colleagues [185, 186], a dysfunctional immune system may be the main player in the pathogenesis of AD [187]. In this view, a number of dysfunctional immune elements have been implicated. The innate immune response (which subjects are born with) primarily involves immune microglia cells within the brain, as described earlier. In AD, microglia change from a homeostatic state to disease-related pro-inflammatory phenotypes which cause neuronal damage [188]. There is also an adaptive immune system response, involving a proliferation of lymphocytes (types of white blood cells) circulating peripherally in the body outside the brain. T lymphocytes (the T denoting their thymus origin) have a major sub-set, T-helper (T_**H**_) cells, which “help” other immune cells, and can also be distinguished by their surface cluster of differentiation (CD) protein expression profile, notably ones expressing CD4, which once activated by antigens become CD4^+^ T cells. There are numerous CD4^+^ T cell subsets, such as T helper 1 (T_**H**_1), T helper 2 (T_**H**_2), T helper 17 (T_**H**_17), T helper 22 (T_**H**_22) and regulatory T cells (Treg) [189]. A number of research studies have implicated immune dysfunction in AD pathogenesis and clinical progression (reviewed by [190]). For instance, elevated peripheral immune-inflammatory markers are associated with future cognitive decline and phosphorylated tau [191, 192]. Also in AD, T cells invade the CNS when the blood–brain barrier (BBB) is disrupted, and localize in regions associated with AD neuropathology, where they are associated with neurotoxicity and enhanced inflammation (reviewed by [193]).

There is a lack of success with immunotherapy trials for AD to date, perhaps due to recruited patients being affected with the established disease that can no longer be halted [190]. Hence there is a search for novel immunomodulatory treatments which may alter the AD course [187], and a number of plants demonstrate this potential (Additional file 4: Table S4). For instance, in addition to microglial enhanced clearance of Aβ, sodium rutin (Table 3) (derived from sources such as *Ruta graveolens*) activated microglial phagocytosis of Aβ amyloid via up-regulation of phagocytosis-related receptors [194].

T_**H**_17 immune cells produce the cytokine interleukin-17A (IL-17A), and in AD patients there is an association between brain amyloid levels and elevated T_**H**_17 cytokine production (reviewed by [195]). IL-17 also inhibits hippocampal neurogenesis [196]. Extracts of *Allium sativum* inhibited IL-17 gene expression in human blood mononuclear cells [197]. In an autoimmune encephalomyelitis mouse model of multiple sclerosis, carnosol (Table 3) (originally extracted from *Rosmarinus officinalis*) promoted a microglial switch to an immunomodulatory phenotype and suppressed reactive T_**H**_17 cells [198].

In the T_**H**_1/T_**H**_2 paradigm first proposed by Mosmann and colleagues, T_**H**_1 and T_**H**_17 cells release pro-inflammatory and T_**H**_2 cells anti-inflammatory cytokines respectively [199]. This view has become expanded, in which both T_**H**_1 and T_**H**_2 cells together orchestrate a variety of adaptive immune responses to maintain a healthy CNS [200], with the T_**H**_1/T_**H**_2/T_**H**_17/T_reg_ cell balance resulting in either a tissue-protective or tissue-destructive immuno-inflammatory response [201]. A dysfunctional T_**H**_1/T_**H**_2 ratio has been regarded as a causative event in neurodegeneration. Several plants demonstrate a T_**H**_1 to T_**H**_2 shift. For instance, treatment with *Nigella sativa* favours a shift to a T_**H**_2 cytokine profile in mouse lymphocytes [202], and in human lymphocytes with *Sambucus nigra* [203].

Prostaglandin E_2_ (PGE_2_) is a downstream lipid product of the COX pathway, and a major modulator of inflammation [204]. In aging mice, inhibition of PGE_2_ in myeloid cells (non-lymphocyte peripheral immune cells e.g., monocytes, macrophages) promoted a more homeostatic anti-inflammatory state and reducing cognitive decline [205]. Since rejuvenating non-brain myeloid cells by reducing PGE_2_ signaling reverses age-related cognitive decline, this manipulation of the peripheral immune system can have a profound therapeutic effect within the brain [206]. Hence plants with this capacity for PGE_2_ /E_2_ reduction could also be of therapeutic potential. For instance, mangosteen (from *Garcinia mangostana*) inhibited E_2_ synthesis in rat glioma cells [207]. In mouse microglial cells curcumin (Table 3) (from *Curcuma longa*) reduced PGE_2_ and also reduced the inhibitory effect of PGE_2_ on Aβ42-induced microglial phagocytosis [208].

#### The oxidative stress hypothesis

According to the Oxidative Stress Hypothesis, free radical-associated oxidation appears to have a fundamental role in driving the pathogenesis of neuron degeneration and death in AD [209–211]. Reactive oxygen species (ROS) are oxygen-derived compounds with highly reactive free radicals, such as anion superoxide (O2·–). Reactive nitrogen species (RNS) are free radicals derived from nitrogen (e.g., peroxynitrite) [212]. Harmful effects of ROS/RNS are known as oxidative stress/ nitrosative stress respectively. Supporting evidence of a role for these stresses in AD progression includes a brain region correspondence between AD pathology and oxidative stress markers [213, 214] (reviewed by [215]). For instance, the oxidation marker 8-hydroxy-2’-deoxyguanosine (OH8dG) increases with aging and is further still increased in the AD brain [216]. Subjects with a diet high in fruits and vegetables had higher plasma anti-oxidants, lower oxidative stress biomarkers and better cognitive performance compared with subjects with a low fruit and vegetable consumption [217]. Hence a good anti-oxidant status appears to be protective against cognitive decline [22].

However, anti-oxidant treatments have failed to reduce oxidative damage (the ‘anti-oxidant paradox’) [218], suggesting that oxidative stress is a downstream effect. Another reason for this failure may be that in contrast to anti-oxidant supplements containing a single/ few anti-oxidants, plants contain phytochemicals with a wide range of anti-oxidant properties [219]. Particularly promising are plants with high anti-oxidant capacity associated with other therapeutic effects targeted to AD pathologies in various preclinical models. For instance, in an AD mouse model, treatment with apigenin (Table 3) (from sources such as *Elsholtzia rugulosa*) inhibited oxidative stress, lowered insoluble Aβ levels and amyloid plaque burden, and rescued learning and memory [51]. In other animal models, reduced oxidative stress was associated with heat shock protein modulation with allicin (from *Allium sativum*) (Table 3) [220], AChE inhibition with *Elettaria cardamomum* [221], memory improvement and anti-aging effects with *Polygonatum sibiricum* [222], anti-atherosclerotic activity with *Cynara scolymus* [223], reduced apoptotic cell death with *Moringa oleifera* [224], DNA damage protection with *Pilea microphylla* [225] and anti-hyperlipidemic effects with *Carthamus tinctorius* [226].

Oxidative stress and inflammation are interdependent, thus therapeutic agents may be required that target both inflammation and oxidative stress simultaneously [227]. Many plants demonstrate anti-inflammatory and anti-oxidant/reduced ROS activities (Additional file 3: Table S3) in studies associating the two, such as in a human study with *Campomanesia speciosa* treatment [228].

Nuclear factor erythroid 2-related factor 2 (Nrf2) is a master regulator of anti-oxidative responses, inducing expression of anti-oxidants, anti-inflammatory mediators and cytoprotective genes [229]. Its expression is decreased in AD patients [230]. Administration of Nrf2 activators reverses memory and synaptic impairments in AD rodent models [231], indicating that Nrf2 pathway activation is a therapeutic target for AD. Plants reported to demonstrate increased Nrf2 expression are thus also of potential therapeutic relevance. For instance, in human cell lines, quercetin (found in numerous plants such as *Crataegus* spp.) upregulated Nrf2 expression and subsequent expression of anti-oxidant enzymes [232]. Similarly, in other human cell line models, Nrf2 was activated with phenethyl isothiocyanate (from *Nasturtium officinale*) [233] and plumbagin (from *Plumbago zeylanica*) [234]. These examples suggest that such plants have therapeutic potential in targeting various oxidative stress effects that may be integral to numerous pathologies implicated in AD.

#### The mitochondrial cascade hypothesis

According to the “mitochondrial cascade hypothesis”, mitochondrial dysfunction triggers Aβ accumulation and AD pathogenesis [235, 236]. Evidence of impaired mitochondrial function is suggested by low brain glucose consumption, decreased oxygen utilization and impaired enzyme gene expression in AD (reviewed by [237]). Moreover, mitochondrial dysfunction precedes Aβ in a senescent AD rat model, suggesting that mitochondrial dysfunction may mediate or even initiate the development of AD pathology [238]. Treatment strategies aimed at boosting mitochondrial and bioenergetic function have shown some benefit in mainly animal models of AD, but clinical trials lag behind the more predominant target strategies such as amyloid [237]. Hence plants reported to enhance mitochondrial functions could provide novel treatment prospects (Table 6; Additional file 4: Table S4). For example, in a double blind RCT clinical study of 63 post-menopausal women, treatment with *Panax ginseng* resulted in increased mitochondrial DNA numbers, improved anti-oxidant status and reduced fatigue symptoms [239]. In a double blind RCT clinical trial enrolling 364 cancer patients, treatment with *Panax quinquefolius* led to a significant improvement in fatigue symptoms [240]. In various pre-clinical models, plant species demonstrated a number of activities, such as reduced mitochondrial dysfunction with *Boerhavia diffusa* [241], restored mitochondrial integrity with *Hippophae rhamnoides* [242] and increased mitochondrial biogenesis with *Paullinia cupana* [243]. A molecular mechanism for mitochondrial biogenesis was demonstrated in mouse muscle cells treated with *Cinnamomum cassia,* which stimulated energy expenditure via upregulation of mitochondrial biogenesis factors such as PGC1αα, NRF-1, and Tfam [244].

**Table 6:** Examples of plants demonstrating anti-fatigue/ improved mitochondrial function and biogenesis activities

Treatment outcome	Model	Species [or extracted phytochemical]	References
Increased mitochondrial DNA numbers	Clinical trial	*Panax ginseng*	[239]
Reduced mitochondrial dysfunction	In vivo mouse/rat	*Citrus paradisi*	[245]
		*Matricaria chamomilla*	[246]
		*Vitis vinifera*	[247]
	Rat cell line	*Boerhavia diffusa*	[241]
Maintained/ restored mitochondrial integrity	Rat glial cells	*Hippophae rhamnoides*	[242]
	Rat neuron	*Solanum indicum*	[248]
Mitochondrial biogenesis upregulation	In vivo mouse	*Paullinia cupana*	[243]
		*Theobroma cacao*	[249]
	Mouse muscle cells	*Cinnamomum* cassia	[244]
Improved mitochondrial energy metabolism	Rat brain mitochondria	*Carthamus tinctorius*	[250]
Reduced fatigue	Clinical trial	*Panax quinquefolius*	[240]

#### The neurogenesis hypothesis

New neurons continue to be generated in the adult human brain from endogenous neural stem cells, mainly in specialized niches within the hippocampus [251] (reviewed by [252]). Most brain areas also appear to possess progenitor cells capable of generating new neurons and glial cells [253, 254]. A neurogenesis hypothesis for AD has been raised as a possibility [255], based on experimentally-reduced neurogenesis resulting in impaired memory in animal models [256]. There is also evidence of impaired neurogenesis in AD (reviewed by [257]). For instance, Moreno-Jiménez et al. [258] demonstrated that adult hippocampal neurogenesis (AHN) persists into the ninth decade in healthy humans, but progressively declines in AD. AHN is also reduced in early stages of cognitive decline, suggesting that AHN deficits may proceed and even promote cognitive deficits in AD [259]. Thus, identifying drugs to stimulate AHN could provide novel therapeutic strategies for AD patients [260]. A number of medicinal plants demonstrating neurogenic activity could provide such sources. For instance, *Calotropis procera* root accelerated neuronal regeneration in a mouse nerve injury model [261]. Neurotrophic factors such as brain-derived neurotrophic factor (BDNF) and nerve growth factor (NGF) enhance the growth and survival of neurons (https://www.nature.com/subjects/neurotrophic-factors). The phytochemical morin (Table 3) (from sources such as *Morus alba* and *Acridocarpus orientalis*) demonstrated increased BDNF and NGF in a rat model [262].

Both neurogenic and memory/cognitive improvement activity were demonstrated in 9 species documented in this study (Additional file 3: Table S3)*.* For example, mice treated with *Prunella vulgaris* demonstrated improved cognitive performance, associated with up‐regulation of adult hippocampal neurogenesis [263]. Sominone (Table 3) (from *Withania somnifera*) enhanced memory in mice via activation of RET (a receptor for the glial cell line-derived neurotrophic factor) [264]. With oil palm phenolics (from *Elaeis guineensis*), treated mice showed improved learning and cognitive ability, associated with up-regulation of genes in the *Bdnf* network and synaptogenesis genes such as *Arc* and *Fos* [265].

#### The cholinergic hypothesis

In the cholinergic hypothesis, memory dysfunction and the cause of AD are attributed to disruption of the cholinergic neurotransmitter system within the brain [266–268]. Cholinergic neurons produce the neurotransmitter acetylcholine (ACh), which mediates its action within the synapse and is then inactivated by the enzyme, acetylcholinesterase (AChE) [269]. In AD acetylcholine is depleted, due to structural alterations in cholinergic synapses, loss of specific ACh receptors and death of ACh-generating neurons, all of which lead to a relative accumulation and activity of AChE [270]. Cholinesterase inhibitors (AChEIs) increase available ACh within the synapses of cholinergic neurons by inhibiting its degradation, but lead to only a modest improvement on cognition [271], with limited effects on the pathology and the disease progression [272]. However, AChEIs may have potentially disease-modifying effects [273]. Clinical trials with AChEIs on AD and VD patients have demonstrated a slowing of brain atrophy, which is implicated in AD pathology [274]. AChEIs are also associated with lower risk of stroke and death [275, 276]. A limitation is that AChEIs mediate adverse gastrointestinal symptoms at doses that are too low to be effective, and there are other adverse effects such as cardiac arrhythmia [268, 277]. Hence there remains much room for improvement in this drug class [278], and a search for drugs with more CNS-selective AChE inhibition profiles [279] would be desirable. From this study the 34 plant species with a documented AChEI activity are thus of prospective interest (Table 7; Additional file 4: Table S4).

**Table 7:** Examples of plant species demonstrating AChE inhibitory activity

Model	Species [or extracted phytochemical]	Bioactivities	References
In vivo rodent	*Albizia lebbeck*	AChE inhibition, improved memory + cognitive impairment	[280]
	*Carthamus tinctorius*	AChE inhibition, memory improvement	[281]
	*Evolvulus alsinoides*	AChE inhibition, anti-inflammatory, memory improvement	[282]
	*Leea indica*	AChE inhibition, reduced memory deficits	[283]
	*Peristrophe bicalyculata*	AChE inhibition, reversed memory impairment, anti-neuroinflammatory	[163]
	*Salvia officinalis*	AChE inhibition, cognitive improvement	[284]
	*Xylia xylocarpa*	AChE inhibition, cognitive improvement	[285]
In vitro	*Asparagus racemosus*	AChE inhibition, anti-amyloidogenic	[50]

For instance, AChE inhibition was associated with improved memory and/or cognition in rodent studies with extracts from *Carthamus tinctorius* [281], *Evolvulus alsinoides* [282] and *Xylia xylocarpa* [285]. In an in vitro and rat brain cell study, sarsasapogenin (Table 3) (from *Asparagus racemosus*) demonstrated AChE inhibition, anti-amyloidogenic activity, anti-oxidant and neuroprotective effects, suggesting a multi target directed ligand potential of sarsasapogenin for AD therapy [50].

#### The vascular hypothesis

The vascular hypothesis of AD (VHAD) proposes that an impaired vascular system is a major contributor to AD disease progression [286, 287]. In this view, vascular risk factors to AD result in chronic brain hypoperfusion, leading to oxidative stress and a neuronal energy crisis, with progressive neurodegeneration and eventually AD [288]. There are various supportive evidences for VHAD (reviewed by [288]). For instance, vascular dysfunction and reduced cerebral blood flow (CBF) occur before Aβ and hyperphosphorylated tau accumulation [289]. Patients exhibiting multiple vascular risk factors to AD demonstrate a faster rate of cognitive decline [290]. A chronic, ischemic-hypoxic state provoked by vascular dysfunction is sufficient to activate APP processing and thence brain Aβ deposition [291]. Positron emission tomography image assessments accurately predict conversion to AD in hypometabolic mild cognitive impairment patients, indicating that impaired cerebral blood flow reduces glucose supply for the brain’s metabolic needs [292]. A variant of the VHAD is the two-hit vascular hypothesis, which envisages that BBB damage allows leakage of neurotoxic molecules, resulting in neuronal dysfunction and impaired amyloid-β clearance (hit one). These processes lead to accumulation of Aβ in the brain (hit two), with neurotoxic effects [293].

Plants have been found to demonstrate therapeutic activities for various vascular risk factors including endothelial inflammation, atherosclerosis, hyperlipidemia, platelet agglutination and thrombotic components, which are next examined in more detail. An attractive characteristic of some of these species is that they provide multiple activities to target the diverse vascular pathologies implicated in AD.

##### Anti-hypertensive bioactivity

Patients developing AD and VD have been found to have higher blood pressure than cognitively normal individuals [294]. Hypertension leads to impairment of cerebral blood vessels and their occlusion, damaging the brain regions the vessels serve [295]. Hypertension also impairs vascular clearance of brain Aβ [296] and increases amyloid and tau deposition [297, 298]. In a meta-analysis of 12 RCTs, blood pressure lowering with antihypertensive agents was associated with a reduced risk of dementia [299], although data for patients with established AD are more scarce [300]. However, there is an incidence of adverse drug effects and adverse other outcomes [301]. A major issue is adherence with treatment (global average < 50%) [302]. Prospectively such limitations may be ameliorated by the many plants demonstrating anti-hypertensive activity.

In clinical trials, 17 plant species have significantly reduced hypertension (for examples, see Table 8). For instance, in treatment of hypertensive patients with aged garlic (*Allium sativum*) for 12 weeks, blood pressure was reduced by 12.5%, comparable to that achieved with common antihypertensive medication [303]. Similarly, in a review of 10 randomised double-blind placebo controlled trials, *Allium sativum* treatment was associated with blood pressure reductions in patients with an elevated systolic blood pressure (SBP) [312]. In a systematic review of the effects of beetroot juice on blood pressure of humans in 22 eligible studies, the overall SBP was significantly lower (3.55 mm Hg) in the beetroot juice–supplemented groups than in the control groups [313].

**Table 8:** Examples of plant species demonstrating improved vascular effects in clinical studies

Vascular dysfunction	Plant species	Subjects	N [C]	Type of study	Outcome	References
Hypertension	*Allium sativum*	Hypertensive patients	25 [25]	X2B RCT	SBP ↓12.5%	[303]
	*Beta vulgaris*	Hypertensive patients	16 [16]	X2B RCT	SBP ↓7.2%	[304]
	*Leonurus cardiaca*	Hypertensive patients	50	Pilot study	SBP ↓10.5%	[305]
	*Punica granatum*	Haemodialysis patients	41 [40]	Crossover RCT	SBP ↓5.1%	[306]
	*Solanum lycopersicum*	Hypertensive patients	130	X2B RCT	SBP ↓7.4%	[307]
Dyslipidemia/ elevated LDL cholesterol	Olea europaea*	Dyslipidemic [MetS] patients	26 [24]	X2B RCT	LDL-C ↓24%, Ox-LDL ↓20%	[308]
	*Punica granatum*	Haemodialysis patients	41 [40]	Crossover RCT	HDL ↑23.4%	[306]
	*Salvia officinalis*	Hyperlipidemic patients	34 [33]	X2B RCT	Total C ↓19.6%, TG ↓22.8%, VLDL ↓13.3%, LDL ↓19.7%, HDL ↑ 20.2%	[309]
Platelet aggreg-ation/endothelial dysfunction	Apium graveolens** [DL-3-n-butylphthalide]	Stroke patients	86 [84]	RCT	elevated circulating endothelial progenitor cells, stroke improvement	[310]
	*Malus pumila*	At risk CVD patients	30	RCT crossover	Improved endothelial function	(311)

*Treatment combined with red rice. **Treatment combined with anti-platelet and lipid-lowering therapy

*C* cholesterol, *CVD* cardio-vascular disease, *LDL* low-density lipoprotein, *HDL* high density lipoprotein, *MetS* metabolic syndrome, *N [C]* number of patients treated [number of untreated controls], *RCT* randomised controlled trial, *SBP* systolic blood pressure, *X2B RCT* double-blind randomised controlled trial, *TG* triglycerides, *Ox* oxidised, *VLDL* very low-density lipoprotein, *↓* lowered level, *↑* elevated level

In a double-blind, randomised parallel arm study in 90 elderly individuals with mild cognitive impairment, drinking high flavanol (≈ 990 mg) cocoa (from *Theobroma cacao*) was associated with significantly reduced SBP and diastolic blood pressure. The treatment was also associated with improved cognitive function [314].

##### Anti-atherosclerotic activity

AD patients also demonstrate atherosclerotic vascular wall thickening [315], reducing cerebral O_2_/nutrient supply which may lead to neuronal loss [316]. Atherosclerosis is associated with an elevation in triglyceride-rich lipoproteins and low-density lipoproteins (LDL), and low levels of protective high-density lipoprotein (HDL) [317]. The disease develops initially from injury to the vascular endothelium, such as from toxins (e.g., oxidized cholesterol). This results in an activated and leaky endothelium with increased cytokine expression, and monocytes and T‐lymphocyte recruitment. These migrate, along with various modified lipids such as oxidized low-density lipoprotein, through the leaky endothelium into the sub-endothelial space, and proliferate. An excessive inflammatory response of these various cells leads to atherosclerotic plaque formation, which can impede blood flow within the vessel, with ischaemic or fatal consequences (reviewed by [318, 319]). Medical interventions have majored on reduction of cholesterol and lipid levels with dietary modification and strategies employing intensive lipid-lowering agents including statins [320]. However, adverse side effects including muscle pain, fatigue and potentially life-threatening rhabdomyolysis, are reported in 10% to 25% of patients receiving statin therapy [321]. There can also be impaired mitochondrial function [322], which could aggravate AD-related mitochondrial dysfunction. In view of these factors, plants with lipid-lowering capability provide an alternative treatment option (Table 8; Additional file 4: Table S4). For instance, in a double blind RCT trial of 26 patients with metabolic syndrome treated with red yeast rice and olive extract supplement, LDL cholesterol was lowered by 24% [308]. Blood pressure was also significantly reduced. An RCT trial with 67 hyperlipidemic patients treated with *Salvia officinalis* resulted in a reduction of total cholesterol, triglyceride (TG), LDL and very low-density lipoprotein in the sage group compared with baseline [309]. In a prospective double blind study of 17 overweight menopausal women given a diet supplemented with *Chenopodium quinoa* (quinoa), there was a significant reduction in LDL cholesterol and TG compared with baseline [323].

##### Improved vascular endothelial function

However, there is now considerable evidence that atherosclerosis is a chronic inflammatory disease [324]. Clinical trials have shown that targeting inflammation can reduce cardiovascular events [317]. The healthy vascular endothelium inhibits platelet adhesion to the surface [325] but cardiovascular risk factors (e.g., hypertension) increase oxygen free radical production, causing the endothelium to switch to a pro-coagulant, pro-inflammatory and vasoconstrictor phenotype [326, 327].

The 38 species of plants with demonstrated anti-platelet aggregation/improved endothelial function have a bearing on these pathologies (Table 8; Additional file 4: Table S4). For instance, in a randomised controlled clinical trial, consumption of apple (*Malus pumila*) improved endothelial function in individuals with cardiovascular disease risk [311]. *Phyllanthus amarus* treatment improved endothelial function and prevented hypertension effects in a rat hypertension model [328]. Molecular mechanisms have been discovered: for instance, EGCG (from *Camellia sinensis*) demonstrated anti-inflammatory effects on endothelial cells, via inhibition of the MAPK/ERK pathway and downstream inflammatory markers such as TNF-α, IL-6, and ICAM-1 expression [329].

##### Thrombolytic activity

Atherosclerosis continues to develop to form atherosclerotic plaques, which are mainly unstable, and their rupture triggers thrombus formation, which can occlude the vessel [330], leading to the decreased cerebral blood flow associated with AD [316]. Seven plant species in this study were found to possess a thrombolytic activity (the capacity to dissolve thrombi), for instance in human and animal blood cell models with *Mauritia flexuosa* [331], and *Typha angustifolia* [332] (Table 8).

##### Anti-obesity activity

Obesity (particularly as indicated by increased waist circumference) increases the risk of AD and dementia [333, 334]. In obesity, excess circulating fatty acids cause adipose tissue cells to become dysfunctional, inducing dyslipidemia and inflammation, which contribute to atherosclerosis [335, 336]. Several plant species surveyed in this study demonstrated anti-obesity activities (Additional file 4: Table S4). For instance*,* obese rats treated with *Cyphomandra betacea* extracts raised high-density lipoprotein cholesterol and total anti-oxidant status, and lowered total cholesterol, body weight and pro-inflammatory TNF-α and IL-6 activities [337]. With *Cinnamomum cassia* treatment, weight gain in obese mice was reduced by increasing energy expenditure via up-regulation of mitochondrial biogenesis [244]. In mice fed a high-fat diet, *Alstonia scholaris* treatment attenuated lipogenesis by reducing expression of lipogenic enzymes ACC-1, PPARγ, LXRα and SCD-1, and upregulating expression of lipolytic enzymes CPT1A, PPARα and ACOX1 [338].

#### The metal ion hypothesis

Certain metals are essential nutrients for the body’s metabolism. For instance, iron has a role in a network of 151 components orchestrating respiration, energy metabolism, DNA synthesis and neurotransmission [339]. The metal ion hypothesis proposes a role of metal ions in AD pathogenesis [340], based on evidence of dysregulation in metal homeostasis (reviewed by [341]). For instance, elevated levels of zinc, copper and iron are found in the brains of AD patients [342]. Such metals interact with amyloid and tau, promoting their aggregation and neuronal toxicity [343]. These effects have been ameliorated by the application of iron, copper, zinc and nickel metal chelators in AD animal/ in vitro models [344–346]. Such chelators bind with the metal to form less toxic metal complexes which can be excreted [347]. It has thus been suggested that chelation therapy is a promising treatment strategy [348]. However, in clinical trials, synthetic chelators have shown limited efficacy for AD treatment and are associated with adverse side effects [349, 350] such as allergic reactions, along with liver, renal, eye and auditory toxicities [351, 352] and may even worsen AD pathology [353]. The plants in this study may provide more promising and safer alternatives, in which metal chelation or other metal-reduction activity was reported in 30 species (Additional file 4: Table S4). For instance, in several clinical studies with thalassemia patients, iron overload was reduced by treatment with *Nigella sativa* extract [354] and silymarin (from *Silybum marianum*) [355] (reviewed by [356]) and in a case report with *Camellia sinensis* [357]. In rodent models, iron overload was reduced by treatment with extracts from *Emblica officinalis* [358] and *Triticum aestivum* [359].

Iron chelation activity is demonstrated in in vitro models in 19 species (Additional file 4: Table S4). Moreover, these plants all demonstrate pleiotropic effects of AD therapeutic potential. For instance, *Cocos nucifera* demonstrated both iron chelation, anti-amyloid and anti-tau activity (Additional file 3: Table S3). Commonly, the active phytochemicals of such plants are polyphenols of dietary origin, which are considered safe, and thus represent strong candidates for metal chelation therapy in AD [360].

Exposure to non-essential metals (e.g., lead, arsenic, cadmium and aluminium) can also exacerbate AD brain pathogenesis. For instance, lead, arsenic and cadmium increase APP and BACE1 expression, leading to increased Aβ production, plaque formation and tau phosphorylation [361, 362]. Aluminium competes with iron binding sites, resulting in increased iron-mediated ROS, and accumulates in neurons [341]. Medicinal plants from the current survey have also demonstrated activities to reduce non-essential metal toxicity. For instance, in patients with chronic occupational lead poisoning, treatment with allicin (from *Allium sativum*) resulted in clinical improvement, which could be attributed to lead chelation, reduced oxidative stress and inhibition of lead absorption from the gut [363]. Curcumin (from *Curcuma longa*) reduced arsenic-induced oxidative stress and induced DNA repair expression in a chronically arsenic-exposed human population [364]. A reversal or reduction of arsenic-induced toxicity in rat models was demonstrated with silibinin (from *Silybum marianum*) [365] and *Ananas comosus* [366]. Lead induced toxicity in rats was ameliorated by treatment with *Zingiber officinale* [367] and *Moringa oleifera* [368]. In another rat model, treatment with *Cynara scolymus* leaf extract protected against cadmium toxicity [369]. In an aluminium exposed mouse model, *Allium cepa* treatment reduced brain aluminium deposition, possibly via PPAR-γ receptor agonism, to reduce aluminium transfer across the BBB [370]. In a rat model, aluminium toxicity-induced neurodegeneration was reduced by treatment with *Aloe vera* [371]. The effects were also associated with reduced memory deficits. *Solanum lycopersicum* extract protected mouse keratinocytes from nickel toxicity [372]. In a rat brain mitochondria model, the phytochemical EGCG protected against cadmium-induced damage, with in vitro evidence supporting metal chelating activity [373]. Further species demonstrating reduction of metal toxicity are reviewed by Amadi et al. [351] and Susan et al. [374].

#### The oestrogen hypothesis

Worldwide around 62% of persons with AD are women [375]. A major driver for this risk may be the precipitous decline in oestrogens with the menopause [376], which suggests that oestrogens have a neuroprotective role. Oestrogens have essential brain functions such as regulation of synaptic plasticity and learning [377] and also reduce oxidative injury, Aβ toxicity, and Aβ generation (reviewed by [378]). Thus oestrogen replacement therapy has been advocated, but from a review of nine clinical trials of oestrogen-containing hormone therapy, the findings suggested that hormone therapy (HT) fails to improve AD cognitive symptoms [379]. For instance, in an RCT treatment of 42 post-menopausal women with raloxifene, a selective oestrogen receptor modulator, no cognitive benefits were conferred in the treated group [380]. However, oestrogen provided a neuroprotective effect if administered to women under 50 years of age. This has been explained by the “critical window” or “window of opportunity” hypothesis, which suggests that the neuroprotective effects of oestrogen depend on age at the time of administration [381]. This is supported by clinical evidence that dementia risk is increased by surgical removal of ovaries prior to the menopause, which results in prematurely reducing sex steroid hormone production [382]. Opinion remains divided whether HT in postmenopausal women provides beneficial or harmful oncological and cardiovascular effects. Clinical data taken in total neither establishes nor refutes the possibility that HT causes breast cancer [383], and for cancer survivors the oncological risk of HT varies with the cancer type, with an increased risk associated with breast and brain cancers [384]. According to a revised global consensus statement, with menopausal HT there is an increased risk of venous thromboembolism and ischemic stroke, and if initiated over the age of 65 increased risk of dementia [385]. Medicinal plants rich in phytoestrogens provide a potentially safer therapeutic alternative. Phytoestrogens are a group of non-steroidal polyphenolic plant metabolites that induce the action of endogenous oestrogens, often by binding to oestrogen receptors [386]. Although almost ubiquitous in plant products, levels may be quite low or moderate in most foods, but are particularly high in soya-based foods and other legumes [387]. Of the plants documented in this study, 18 species demonstrated significant oestrogenic activity (Additional file 4: Table S4). For instance, in an RCT study of postmenopausal women treated with *Glycine max* (soy bean), therapeutic effects on reproductive system atrophy were attributed to oestrogenic action via an increased percentage of oestrogen receptor positive cells [388]. RCTs examining the effects of soy treatment have reported mixed results [389]. Beneficial effects in post-menopausal women included improved visual memory [390] and cognitive performance [391]. In men, enhanced working memory was reported, suggesting a role for oestrogen in mental processes in males [392]. A study reported negative effects [393] although it had limitations (e.g., short duration of 16 weeks). Moreover, the treatment given was soy milk, which is relatively low in phytoestrogen content [394] in contrast to high levels in other soy foods such as the soya bean and soya flour [387]. Overall, the data from RCTs indicate a need for further, much larger studies with more controlled methodological standards and mediating factors [389].

Oestrogenic activity or high phytoestrogen content was demonstrated on rodent and in vitro models with 19 plant species (Additional file 4: Table S4). For instance, in an ovariectomized rat model, phytoestrogens from *Glycine max* resulted in improved memory performance, which may be attributed to increased *bdnf* and synaptic protein gene expression [395]. In other ovariectomized rodent models *Pueraria lobata* extract promoted oestrogenic activity, by upregulating expression of oestrogen receptor α (ER-α) [396] and *Medicago sativa* prevented bone loss induced by oestrogen deficiency [397]. Many of these plant species also demonstrate other bioactivities of AD therapeutic potential. For instance, genistein (from *Glycine max*) alleviates hyperphosphorylation of tau protein through regulating CAMK4 [94].

Male sex hormones (androgens) in men, but also women, have roles in reproduction, cardiac health, bone remodelling, muscle mass, and brain function [398]. Reduced androgen levels in aged men and women may also be risk factors for cognitive impairments and AD, and thus testosterone therapy may have potential benefits [399]. Testosterone–raising activity is demonstrated in rat models with *Pistacia atlantica, Punica granatum, Tamarindus indica,* and *Zingiber officinale. Tamarindus indica* is noteworthy in its capacity to raise both oestrogen and testosterone levels [400], indicating a promising pleiotropic potential in targeting various hormone deficiencies implicated in AD.

#### The infectious agent hypothesis

Itzhaki and colleagues [401] have summarized evidence in favour of an infectious agent in AD which may have a causative role in the pathology. For instance, pathogen signatures specifically colocalize with AD pathology. Moreover, antivirals such as acyclovir block virus-induced Aβ and tau pathology in vitro [402]. Microbes implicated in AD include *Herpes simplex* virus type 1 (HSV1) and type 2 (HSV2), *Garcinia mangostana*, *Escherichia coli*, and several spirochaete and fungal species [401, 403]. Of therapeutic potential are the 769 species demonstrating anti-microbial activity, the most common bioactivity documented in this study (Additional file 3: Table S3; for examples, see Additional file 6: Table S6). For instance,* Alstonia scholaris* demonstrated effective anti-viral and anti-bacterial activity against HSV-1 [404] and *E. coli* [405], which are both implicated in AD brain pathology.

The studies were reported in various preclinical models, with anti-bacterial effects being predominantly in vitro*.* The anti-microbial inhibition ranged from highly potent to more moderate inhibition, according to the species. For instance, with *Gossypium barbadense* and *Ficus benghalensis,* HSV-1 inhibition was 99.9% and 96.6% respectively [406] and with *Coptis chinensis* it was 100% inactivated [407]. Another example of high potency is the anti-bacterial activity of *Dacryodes edulis*, which was higher than that of gentamicin, the standard reference drug [408]. *Morinda lucida* extracts were more active against all the tested bacteria than the standard antibiotics (chloramphenicol and ciprofloxacin) [409]. *Justicia gendarussa* inhibited HIV reverse transcription, by inhibition of both the early and late gene transcription at levels greater than the drug AZT [410].

Clinically approved antiviral drugs exist for only 10 of the 220 + viruses known to infect humans [411]. This highlights a crucial need for anti-viral drug discovery. The 202 plant species in this study documented with anti-viral activity could provide a source of novel anti-viral agents, with the capacity to act by a number of mechanisms. For example, *Phyllanthus amarus* targeted various stages of the HIV life cycle, thereby presenting multiple antiviral activities, and demonstrated significant anti-HIV activity in human-derived cells [412]. *Isatis tinctoria* acted on human influenza virus by targeting viral endocytosis, interfering with viral ribonuclear protein export from the nucleus [413]. *Urtica dioica* inhibited SARS-CoV infection in mice by targeting adsorption or penetration stages of the replication cycle, and by binding to the SARS-CoV spike glycoprotein [414]. An obstacle to the eradication of HIV is the persistence of latent virus in infected patients. *Euphorbia tirucalli* demonstrated a capacity to eliminate this latent viral reservoir. This is executed in a dual action, by upregulation of the pathway to reactivate the virus out of latency, and downregulation of the viral surface proteins essential for HIV replication [415]. This is of potential high relevance in targeting latent viruses in the brain, implicated in AD pathogenesis [416]. *Punica granatum* demonstrated multiple anti-viral activity against HSV, Sindbis virus, polio [417], influenza, [418] and HIV [419], indicating a broad spectrum anti-viral activity to target as yet unidentified viral pathogens involved in AD.

Another example of an infectious agent implicated in AD is *Porphyromonas gingivalis,* a key bacterium in chronic periodontitis. The pathogen was identified in the brain of AD patients, bacterial toxin levels correlating with tau and ubiquitin pathology [420]. Of therapeutic relevance are medicinal plants such as *Musa paradisiaca and Pistacia lentiscus*, which demonstrated anti-bacterial activity specifically against *Porphyromonas gingivalis* [421, 422] (Additional file 3: Table S3).

#### The gut microbiome hypothesis

Alterations in gut microbial communities in AD patients may result in pathophysiological changes in the brain [103, 423]. This hypothesis is supported by evidence of decreased microbial diversity in the gut microbiome of AD patients [424]; an increase in pro-inflammatory gut bacterial taxa is associated with brain amyloid pathology in AD patients [425]; and mice raised with germ-free gut conditions have less cerebral amyloid deposition [426]. Gut microbiome alterations may increase permeability of the gut barrier and result in immune activation, impaired BBB, neuroinflammation, and ultimately neurodegeneration [427]. In an RCT study of healthy older adults, a probiotics-rich diet was associated with reduced inflammatory-causing gut microbiota and improved mental flexibility [428]. Modulation of gut microbiota in AD mouse models led to restoration of an impaired ubiquitin proteasome system and autophagy, reduced cognitive decline and amyloid deposition [429, 430]. These findings suggest that targeting impaired gut microbiota is a promising therapeutic strategy. In the current study, reports of gut microbiota modulation activity were found for *Morus alba, Punica granatum* and *Vaccinium myrtillus.* For instance, in a randomised cross-over human trial with *Punica granatum* consumption, subjects were conferred with higher gut microbiome diversity and more favourable microbiota profile [431]. Resveratrol and quercetin administration in rat models also modified gut microbiota favourably (reviewed by [432]). Various species (e.g.,* Olea europaea, Solanum lycopersicum*) documented in this study are components of the Mediterranean diet. Frail adults’ adherence to a Mediterranean diet was associated with microbiome alterations, reduced inflammation and improved cognitive function, suggesting that such a diet has a beneficial impact on the gut microbiome, which in turn may promote healthier aging [433].

#### Other effects: apoptosis and aging

Apoptosis, a programme of controlled cell death [434], may have a role in the neuronal cell death associated with AD, based on evidence of increased apoptosis in the AD brain compared with the normal brain [435, 436]. 25 plant species in this study had reports of anti-apoptotic bioactivity (Additional file 4: Table S4). For instance, fibrauretine (Palmatine chloride) (Table 3) from *Fibraurea recisa* suppressed pro-apoptotic caspase-3 and *Bax* protein expression, and increased anti-apoptotic *Bcl-2* expression in an AD mouse model [74].

Age is the main risk factor for AD, with cellular senescence and other hallmarks of aging thought to contribute to AD pathology [437, 438]. 13 plant species were found to exhibit anti-aging activity (Additional file 4: Table S4), with molecular mechanisms such as upregulation of telomerase activity.

### Plants with ethnological use of memory improvement demonstrate bioactivities of therapeutic relevance to 15 causal bases for AD

The next part of our study focused more particularly on the subset of 107 species in which the ethnological report of memory improvement was validated by an AD-relevant bioactivity. We found that the various species within this subset together demonstrated therapeutic activity for all AD causal bases hypothesised in the previous Section (Additional file 7: Table S7). 69 of these species demonstrated multiple bioactivities of AD therapeutic relevance (for examples see Table 9). Some of the species or their phytochemical extracts demonstrated pleiotropic activity targeted to many of the causal bases. For three of these species (*Centella asiatica, Rosmarinus officinalis* and *Zingiber officinale*) the ethnomedical use of memory improvement is also validated by clinical studies confirming memory/cognitive improvement, along with multiple other bioactivities of relevance.

**Table 9:** Examples of plant species with multiple bioactivities of ND therapeutic relevance

Species [or extracted phytochemical]	Model	Bioactivity of ND therapeutic potential
*Centella asiatica*	Clinical studies	Memory improvement
	Human cell line	Mitochondrial biogenesis
	Anima in vivo studies	Anti-amyloidogenic; attenuated cognitive deficits; anti-inflammatory; neuronal growth stimulus; anti-hypertensive
	In vitro	Anti-bacterial
*Rosmarinus officinalis*	Clinical studies	Memory improvement
	Anima in vivo studies	Anti-amyloidogenic; reduced cognitive and mitochondrial dysfunction; promotes microglial switch to immunomodulatory phenotype; anti-hypercholesterolaemic; anti-inflammatory
	In vitro	Inhibition of tau aggregation; anti-oxidant; anti-viral, anti-bacterial, anti-fungal
*Zingiber officinale*	Clinical studies	Cognitive improvement; anti-viral; anti-inflammatory; anti-platelet aggregation
	Animal in vivo studies	Anti-neuroinflammatory, improved cognitive function
	Rat astrocyte cells	Reduced apoptosis, increased *bdnf* + *ngf* gene expression, attenuated mitochondrial impairment
	In vitro	Anti-bacterial; anti-oxidant

For references see Additional file 3: Table S3 and Table References

The implication is that the effectiveness of this set of plants is based on their action in targeting multiple key pathologies implicated in AD. If this is so, the plant species with ethnological reports of memory improvement could provide an attractive source of drug leads of AD therapeutic potential.

### Distribution of surveys

#### Overall distribution pattern of surveys with ND relevance

**Fig. 1:**
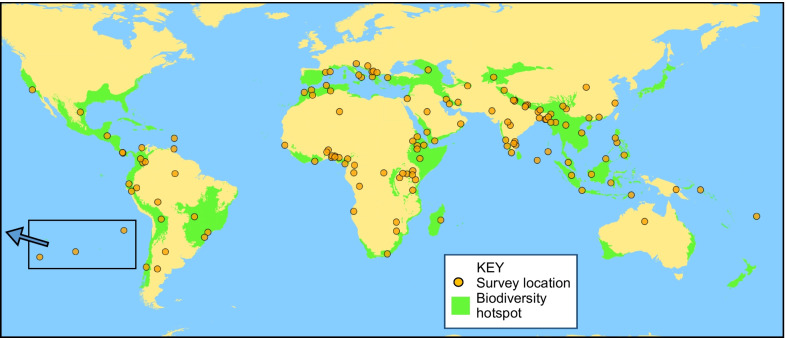
Distribution of ethnomedical surveys with potential therapeutic relevance for neurodegenerative diseases. The survey distribution on the map indicates that the surveys were located most commonly in Africa, Asia, Central and South America, suggesting an abundance of studies of ND relevance from those continents. There is also an abundance of surveys for certain countries (e.g., India, Nigeria), with the biggest cluster of surveys [40 in all] in India, Bangladesh and Pakistan combined. 90 out of 157 (57%) of the studies were found to reside within biodiversity hotspots and therefore are under threat. Inset: surveys located in Pacific islands

The distribution of the surveys from which data of ND relevance was mined are indicated in Fig. 1. Of the 67 countries represented by the surveys, these were located most commonly in Africa, Asia, Central and South America. This suggests a particular abundance of studies of ND relevance from those continents. More than one survey within a country was mined if there were sufficiently distinct habitat types or regional identity between them (e.g., Amazonian versus Atlantic Forest of Brazil) or where ND-relevant data richness was revealed in the literature searches. The latter is exemplified by India, in which 10 separate surveys were found that cited species with uses reported for memory improvement. The overall distribution pattern of the surveys across the world revealed by online search engines reflects the abundance of ethnomedical surveys for certain countries (e.g., India, Nigeria), but a paucity of surveys for other countries (e.g., Chad, Libya).

Figure 1 demonstrates that the biggest cluster of surveys [40 in total] occurs in India, Bangladesh and Pakistan. This cluster is concentrated particularly in the Himalayan ranges and environs, and although this may be associated with the very high species diversity there, there are other countries with even higher species diversity in which ethnomedical surveys are less common, such as parts of South America. For instance, the Pacific region of Columbia has one of the highest pockets of biodiversity in the world with 50,000 species of plants, and yet both scientific knowledge on Colombian flora and ethnomedical investigations are lacking [439]. The cluster of surveys yielding such high levels of data in the India-Bangladesh-Pakistan country block could be attributed to the philosophy of the Ayurvedic medicine system, in which every plant on earth is considered to have a medicinal property [440], which provides great motivation to search for novel medicinal plant uses, although this could return false positives.

#### Data at risk—an assessment of causes of concern

The surveys were then mapped in relation to the locations of biodiversity hotspots (BDH) (Fig. 1). 90 out of 157 (57%) of the studies were found to reside within BDHs and therefore are under threat. Since BDH regions have by definition lost at least 70% of their native vegetation, species with valuable therapeutic potential may already have been lost, and there is a threat to the survival of the remaining species. For instance, 40 of the included surveys reside in the Himalayan BDH. In a recent study of 12 Himalayan regions within this BDH, of 112 documented medicinal plant species, 19 species were found to be at risk of extinction, with seven species being critically endangered [441]. The threats are not limited to the BDH regions either. The BDH regions represent only 36 of the world’s most threatened areas, with numerous areas outside BDH status still being vulnerable, such as much of the Brazilian Amazon forest [442]. Another example outside a BDH is a study in Mizoram, India, in which 13 of the 81 species of therapeutic ND relevance listed were reported to be rare, vulnerable or endangered there [443].

Next, the 115 main surveys were examined systematically to determine what the specific threats of concern were to the authors. The threat of most common concern was loss of traditional knowledge (reported by 58% of authors). This was a problem reported in communities worldwide, ranging from South America to Europe, Asia and the Pacific. For instance, in Albania the knowledge erosion is due to urbanization and economic migration away from the villages [444]. In Ethiopia, India and Nepal the knowledge erosion is attributed to the younger generation’s disinterest in ethnomedical knowledge or a lack of knowledge flow [445–447]. In Fiji, Martinique and the Philippines its decline is the result of preferences for Western-type medicine [448–450]. Once such knowledge is lost, a major consequence reported in Italy is that the remedies that remain treat only unimportant pathologies [451].

The second-most common threat was habitat loss (reported by 34% of authors). For instance, the Atlantic Forest region of Brazil agriculture is now based on brazilwood, sugarcane, coffee and cattle. Relocating 50% of the Brazilian population to cities once covered by forest resulted in only 5% of the original forest remaining [452]. In Ethiopia, habitat loss resulted from various anthropogenic threats such as deforestation to expand agricultural land and for firewood collection [447]. Medicinal plants can also be under threat from invasive weeds [453] and grazing [454]. Over-harvesting is an issue in countries such as Peru [455]. This can lead to species with ND therapeutic potential becoming rare, as for *Sideritis athoa* in Turkey [454] and in Vietnam for *Aquilaria crassna*, which is now critically endangered [456].

Loss of medicinal knowledge and habitat loss are often intertwined. As Ji and colleagues comment regarding the Lisu people, in NW Yunnan, China, over-exploitation and deforestation have led to disappearance of some medicinal plants and the associated knowledge of their use [457]. Similar such associations were reported in Côte d'Ivoire [458] and Ethiopia [453].

Analysis of the knowledge erosion problem can be found in the study by Voeks and Leony [459], who attribute the key reason for this loss to formal education access, in which the healing properties of their forests and fields no longer find their way into the curriculum. In contrast to this, traditional knowledge (TK) is sustained in the Kenyan Masai tribe by children spending time with their parents, and this TK persists even with children’s enrolment into formal education [460].

Several remedial strategies are illustrated from the authors surveyed in this study. For instance, harvesting of medicinal plants which are introduced species have less impact on the local habitat, in order to preserve sites of native species under threat [461]. This low-impact harvesting of medicinal plants can bring economic benefits, such as the agro-industrial credit initiative in Panama for producers of medicinal plants that can be marketed [462]. In the Hakka communities of China there is already an awareness of which plants are endangered, and over-harvesting is prevented by using more common species [463]. Environmental education can be fostered by key individuals of a community being included into management programs [461]. The Nicobarese community harvest mainly the leaves of the plants, since these are the most renewable parts [464]. In Northern Peru, healers are open to new knowledge, watching international health trends to incorporate new species such as Noni (*Morinda citrifolia*) fruits into their own repertoire, the fruit products being harnessed in local plant pharmacies to benefit the local economy and population [455]. One of the most striking examples of an improvement in ethnomedical knowledge in recent years has occurred in Kyrgyzstan. Under the 70 years of Soviet rule, traditional medicinal practices in such Central Asian societies were neglected and suppressed, leading to a loss of TK [465]. However, in the post-Soviet era there has been a remarkable revival of ancestral TK [466].

### Future perspectives

The bioactivities documented in this study of therapeutic relevance to the various pathological causal bases for AD are mainly pre-clinical or in vitro studies. These provide a basis for further studies to ascertain clinical relevance, standardize dosage, ensure safety, and characterize deleterious off-target effects. State of the art technologies exist, such as sequencing, metabolomic and proteomic tools, to excavate undiscovered plant metabolites, improve yield and eliminate toxic compounds from valuable plant extracts [467, 468]. Plant tissue culture techniques can eliminate the reliance on wild plants under threat [469].

The ability of the plant products to cross the BBB can be tested with various in vitro and in vivo BBB models [470]. However, major challenges remain with current BBB models, which have limited ability to recapitulate barrier dysfunction and plaque deposition [471]. Promising next generation models apply tissue engineering technologies, which aim to more effectively replicate BBB architecture [472]. For instance, an in vitro 3D neurovascular model under development combines cells from the nervous system with a BBB endothelial cell interface, which could provide a platform to assess drug effects on neural function [473, 474].

There are yet other less known causal bases implicated in AD pathology [24], for which a plant therapeutic role is largely unexplored. For instance, according to the calcium homeostasis hypothesis, Aβ destabilizes neuronal calcium homeostasis, which renders neurons more vulnerable to environmental insults [475]. There is evidence, for example, of cognitive decline in AD associated with changes in calcineurin/nuclear factor of activated T-cells (NFAT) signaling [476]. Thus NFAT inhibition and application of other agents aiming to correct neuronal Ca^2+^ dysregulation are therapeutic strategies for AD treatments [477, 478]. However, here too there is potential from medicinal plants, with phytochemicals (e.g., gossypol, kaempferol and arctigenin) demonstrating NFAT and calcineurin inhibition [479], which could be promising candidates for further investigation.

Another underlying factor driving AD pathology may be meningeal lymphatic vessel dysfunction, which thus might be therapeutically targeted [480]. In peripheral tissues, the lymphatic system drains wastes from the spaces between cells, but no such system has been found within the CNS. However, waste fluids have been found to drain into spaces surrounding the blood vessels, a paravascular pathway or “glymphatic” system, so-called because of dependence on glial cells and function similar to the peripheral lymphatic system [481]. Since Aβ is transported along this route, its impairment may contribute to accumulation of amyloid [482], tau and lead to neurodegeneration [483]. Plants with therapeutic bioactivity in stimulating lymphatic drainage, such as *Aesculus hippocastanum* [484], could thus be explored for a possible similar role in improving glymphatic function.

The study excluded algae, in view of taxonomic placements outside the plant kingdom (Plantae), but there is a new consensus that red and green algae should be placed within Plantae [485]. Promising algal neuroprotective activities of relevance to AD have been reported. For instance in pre-clinical models, fucoxanthin (from the alga *Sargussum horneri*) inhibited Aβ assembly, reversed memory impairment and enhanced *bdnf* expression [486]. Future studies could investigate the potential of other algal species, which are underexplored [487].

The focus of the paper was the therapeutic potential of plants for the causal pathologies of AD, but there is also scope to explore a role for plants in relief of AD-related symptoms in addition to memory and cognitive improvement. For instance, subjects with AD show a higher prevalence of sarcopenia (degenerative loss of skeletal muscle mass and strength) [488]. The plants *Withania somnifera* and *Silybum marianum* rescued myotubes of sarcopenic subjects, indicating the potential of such plants to reverse the muscle functional decline in sarcopenia [489].

Some of the causal hypotheses are founded on evidence of the causal agent appearing early or earlier than other proposed causalities. An early chronology in the appearance of a pathology in pre-symptomatic individuals in itself does not prove its causality in the disease. There is a need to probe this further, to establish if AD clinical symptoms can convincingly be attributed to a specific causal agent. There is a need also for further clarity of the relationships between the various proposed causal factors: is it a linear and hierarchical one, with upstream and downstream effects, or is it a syndromic disease driven by multiple initial causes? The answers to these question are still needed, to inform more effective targeting of drugs to the key causal agents.

Initiatives to maintain and promote the vanishing ethnomedical knowledge are needed, which may provide valuable information of yet further novel plant species of therapeutic relevance. Gaps need to be filled in TK (such as effective dosage) that is fragmentary in the ethnological literature for species with important therapeutic potential. There is a need to document species that are vulnerable or at risk of extinction, and if possible, to reduce the risks. For instance, the survey of plants within the Himalayan BDH recommended prohibition of unmanaged harvesting of medicinally important threatened plants from the wild, encouraging instead their cultivation [441]. There are alternative modes of cultivation, such as micropropagation, which enable rapid regeneration of plantlets [490]. Another alternative is hydroponic technology, in which nutrients are supplied to the plant in irrigation water [491]. Several medicinal plant species grown with hydroponics have produced higher biomass in a much shorter time period, with a higher concentration of bioactive secondary metabolites, compared with field-grown plants [492]. There are challenges to such cultivation, such as unavailability of seeds, equipment costs, and difficulties of domesticating plants from the wild [493]. There can be a need too for crop management skills to monitor and modify nutrient solutions [494]. However, some of these obstacles are being overcome. For instance, simplified hydroponics, focused on low production costs, have produced promising results in rural communities throughout South America [495, 496].

Finally, this study was not exhaustive. Whilst around 46% of the angiosperm families are represented in this data, the number of species (1339) documented with therapeutic bioactivities is still quite small in relation to the estimated ≥ 300,000 species just within the angiosperms alone. Since many of these species still remain to be surveyed for their ND therapeutic potential, it is likely that the plant kingdom has an even greater repository of potential yet to be tapped. There is thus all the more need for TK and habitats to be preserved so that this resource is not lost.

## Conclusions

Our first aim was to search for plant species with reported therapeutic effects of AD relevance. Our findings suggest that the documented plants provide a large resource of AD therapeutic potential. The toolkit methodology was found useful for providing a wide reach in the search of this potential. The bioactivity reports of AD relevance were mainly pre-clinical. However, clinical studies were also found, conferring anti-amyloid effects, autophagy induction, increased mitochondrial biogenesis and improved energy metabolism, anti-hypertensive and anti-hyperlipidemic effects, improved endothelial function, reduced iron overload, reduced metal toxicities, oestrogenic activity, anti-microbial effects for pathogens implicated in AD, and gut microbiome modulation to a more favourable microbiota profile. These findings demonstrate that the capacity of plants to target AD pathologies can be translated across to humans. There is much scope for further exploration of the pre-clinical studies that mostly remain to be tested for clinical efficacy. Particularly promising are plant multiple molecular mechanisms targeted to a pathology. For instance, the plants can reduce Aβ via various mechanisms including BACE1 downregulation, upregulation of amyloid-degrading protease, blocking formation of toxic Aβ oligomers, reducing expression of amyloid precursor protein, and remodelling Aβ into non-toxic structures. There are prospects that at least some of the 46 plant species demonstrating these effects may possess phytochemicals that are BBB permeable, and reach their target pathology.

For our second aim, to assess how this ethnomedical data may be at risk, we found that 58% of the mined ethnomedical surveys reside within biodiversity hotspots and are thus under threat, with loss of traditional knowledge the threat most commonly reported. There is therefore an urgent need to preserve the knowledge of ethnomedical use, as well as the habitats on which this knowledge depends. Encouraging signs such as the reversal of such knowledge loss in Central Asian countries such as Kyrgystan indicate that this can indeed be possible.

Our third aim was to find AD causal hypotheses for which the mined plants may have therapeutic relevance. The outcome was that the documented plants in total demonstrated bioactivities targeted to 15 proposed causal pathologies. In particular, the species with an ethnological report of memory improvement as a subset, together demonstrated therapeutic activity for all these AD causal bases, from which it is concluded that this ethnological data is a very valuable resource of AD therapeutic relevance**.** The fact that there are a large number of AD causal hypotheses is an indication that multiple pathologies may be involved in a complex interplay, and that a primary causal agent (if it exists) remains to be unequivocally discerned. A number of the individual plant species also demonstrated pleiotropic therapeutic bioactivities for a range of pathologies implicated in the various causal hypotheses. These findings suggest that such plants have promise as drug leads to target these multiple hallmarks of pathology. By further probing of their molecular effects, the plants may also provide insight into delineating more clearly the causal basis or bases of AD, which is still crucially needed to inform therapeutic strategies.

## Materials and methods

### Toolkit methodology and data extraction

Three literature searches were performed using the databases PubMed and Google Scholar, conducted from October 2017 to March 2022, with no time limits on the years of publication. Additional sources of data included reference lists of included articles. The first search enabled an assessment of ND pathologies and symptoms, in order to construct therapeutic categories recognized by ethnomedical practitioners (Additional file 1: Table S1). The search terms of numerous neurodegenerative diseases were applied in relation to terms such as pathology and symptoms (e.g., Alzheimer’s disease AND pathology). A second search consisted of finding ethnomedical surveys containing species with reported ethnological uses which could also be of ND therapeutic potential. The search terms were: ethnomedical survey, ethnobotanical survey, medicinal plants, medical herb, ethnobotany OR indigenous tribe. These search term alternatives were then also combined with country-specific searches to find further surveys not revealed in the initial searches. The therapeutic categories were then applied as a toolkit to find plant species with reported therapeutic effects of ND relevance, mined from 115 ethnomedical surveys. A further 42 ethnomedical surveys were mined for therapeutic benefit of a single symptom only (e.g., memory improvement). Thus, 157 surveys were studied in total (Additional file 2: Table S2). In addition, two ethnomedical databases were accessed: the Prelude database of medicinal plants in Sub-Saharan Africa [497] (http://www.metafro.be/prelude) and the Native American Ethnobotany database (http://naeb.brit.org). The surveys were also analysed to determine how the data may be at risk, and the specific threats of concern to the authors were documented. A third search was to find various hypothesized causal bases for Alzheimer’s disease, and for bioactivities of the above documented plants which could be of therapeutic relevance to these causal bases. For method details with reference to inclusion/exclusion criteria and taxonomy, see S File 1.

### Mapping of survey locations

The RStudio package was used to map the locations of the studies, using the rgdal, rgeos, dplyr and ggplot2 packages. Biodiversity hotspots spatial data was obtained from Hoffman and colleagues [498]. Locations of the studies were determined from maps included in the surveys, and where necessary precise coordinates were obtained using LatLong (www.latlong.net).

## Supplementary Information

**Additional file 1. Table S1**. An ethnomedical toolkit.

**Additional file 2. Table S2**. List of species with ethnomedical use of neurodegenerative disease therapeutic potential.

**Additional file 3. Table S3**. Bioactivities listed by species.

**Additional file 4. Table S4**. Bioactivities listed by category.

**Additional file 5. Table S5**. Phytochemicals with anti-amyloid and tau activity.

**Additional file 6. Table S6**. Examples of plants with activity against infectious agents implicated in Alzheimer’s disease.

**Additional file 7. Table S7**. Plants with ethnological reports of memory improvement demonstrating bioactivities of therapeutic relevance to 15 causal hypotheses for AD.

**Additional file 8. Table S1–S7**. References.

**Additional file 9. File S1**. Materials and Methods further details.

## Abbreviations

ACh: Acetylcholine
AChE: Acetylcholinesterase
AChEIs: Acetylcholinesterase inhibitors
AD: Alzheimer’s disease
AHN: Adult hippocampal neurogenesis
APP: Amyloid precursor proteins
Aβ: Amyloid β
AβO: Amyloid- β oligomer
BACE1: β-Site APP cleaving enzyme
BBB: Blood brain barrier
BDH: Biodiversity hotspots
BDNF: Brain-derived neurotrophic factor
C: Cholesterol
[C]: Number of untreated controls
CBF: Cerebral blood flow
CD: Cluster of differentiation
COX-2: Cyclooxygenase-2
CSF: Cerebro-spinal fluid
CVD: Cardio-vascular disease
E2: Prostaglandin E2
EGCG: Epigallocatechin-3-gallate
ER-α: Oestrogen receptor α
FDA: Food and Drug Administration
HDL: High-density lipoproteins
HSV1: *Herpes simplex* Virus type 1
HSV2: *Herpes simplex* Virus type 2
HT: Hormone therapy
ICAM-1: Intercellular cell adhesion molecule-1
IL-6: IL-17, interleukin-6, interleukin-17 etc.
L-NBP: L-3-n-butylphthalide
LOX: Lipoxygenase
mAb: Monoclonal antibody
MetS: Metabolic syndrome
N: Number of patients treated
NDs: Neurodegenerative diseases
NFAT: Nuclear factor of activated T-cells
NFTs: Neurofibrillary tangles
NF-κB: Nuclear factor-κB
NGF: Nerve growth factor
RNS: Reactive nitrogen species
Nrf2: Nuclear factor erythroid 2-related factor 2
NSAIDs: Non-steroidal inflammatory drugs
OH8dG: 8-Hydroxy-2′-deoxyguanosine
Ox: Oxidised
PGE2: Prostaglandin E2
PQC: Protein quality control
RCT: Randomised control trial
ROS: Reactive oxygen species
SBP: Systolic blood pressure
TG: Triglyceride
T_**H**_: T-helper
T_**H**_1: T helper 1
T_**H**_2: T helper 2
T_**H**_17: T helper 17
T_**H**_22: T helper 22
TK: Traditional knowledge
TNF-α: Tumour necrosis factor alpha
Treg: Regulatory T cells
UPS: Ubiquitin–proteasome system
VHAD: Vascular hypothesis of AD
VLDL: Very low-density lipoprotein
