# Supplementary material for: Therapeutic roles of plants for 15 hypothesised causal bases of Alzheimer’s disease
Source: Nat Prod Bioprospect. 2022 Aug 23;12(1):34. doi: 10.1007/s13659-022-00354-z (PMC9395556; doi:10.1007/s13659-022-00354-z)
Supplement: Supplementary file 1 — Additional file 1. Table S1. An ethnomedical toolkit. [file 13659_2022_354_MOESM1_ESM.pdf]

Additional Table S1. An ethnomedical toolkit. Hallmarks of neurodegenerative pathology and clinical symptoms mapped to therapeutic categories used in ethnomedicine

**Disease category**	**Hallmarks of pathology and clinical symptoms**	**Therapeutic category [Ethnomedical category of therapeutic potential]**
Neurotoxicity	Misfolding and aggregation of proteins are involved in numerous NDs: e.g. amyloid β (Aβ) and tau in AD; alpha-synuclein (α-syn) in PD and MSA; superoxide dismutase 1 (SOD1) + TAR DNA binding protein (TDP-43) in ALS; Huntingtin in HD; prion proteins in Spongiform encephalopathies (Sweeney et al., 2017). These cause neurotoxic effects by disrupting synaptic transmission, damaging mitochondria and impeding proteasomal clearance (Boland et al., 2018). Malfunctioning microglia and astrocytes (neuron support cells) release neurotoxic factors that kill neurons (Heneka et al., 2015).	Detoxifying/poison antidote, anti-venom
Memory and cognitive impairment	Memory and cognitive impairment occurs in AD, A-H, BD, CS, FRDA, HD, MSA, MS, NPC, PD, VD, but also non-ND diseases + traumatic causes (Arlt, 2013). May result from neurotoxicity [see neurotoxicity box] and its effects, such as disrupted synaptic transmission + neuronal death.	Memory/cognitive improvement, anti-dementia
Paralysis/spasticity	Degeneration of neurons results in paralysis or spasticity in ALS (Robberecht & Philips, 2013); in autosomal dominant AD (Rujeedawa et al., 2021); MS (Trapp & Nave, 2008); FRDA (La Rosa et al., 2020); and polio virus infection and postpolio syndrome (Li Hi Shing et al., 2019).	Anti-paralytic/hemiplegia/ paraplegia/polio
Parkinson's disease/Parkinsonism	Associated with misfolded α-synuclein aggregation, resulting in dopaminergic neuron loss, leading to motor symptoms e.g. rigidity, resting tremor and postural instability (Wirdefeldt et al., 2011). Also relevant to dementia with Lewy Bodies (De Boni et al., 2022; Taylor et al., 2020). α-synuclein is also implicated in AD pathology (Twohig & Nielsen, 2019).	Anti-PD, anti-tremor
Neuroinflammation	Neuroinflammation may drive abnormal aggregation of misfolded proteins (Guzman-Martinez et al., 2019). A persistent inflammatory response, possibly induced by dysregulated cytokine proteins, perturbs microglia and astrocytes to become neurotoxic (Hickman et al., 2018).	Anti-inflammatory
Impaired neurogenesis	Neurogenesis (the production of new neurons) does occur in the adult brain but is impaired in NDs and may contribute to the disease process (Mu & Gage, 2011).	Wound healing, anti-ulcer
Immune involvement	Triggers of immune dysfunction can contribute to synaptic and neuron loss and drive ND progression (Hammond et al., 2019). Dysfunctional immune system: in MS attacks myelin sheath of CNS neurons; and is implicated in AD, HD, glaucoma + BD.	Boost immune system, immunomodulation
Mitochondrial dysfunction	Mitochondrial dysfunction or damage results in energy failure and fatigue. This is a pathological hallmark of NDs including AD and HD (Winner & Winkler, 2015), ALS (Robberecht & Philips, 2013), MSA (Monzio Compagnoni & Di Fonzo, 2019), BD (Saffari et al., 2017), Gangliosidosis (Brunetti-Pierri & Scaglia, 2008), peripheral neuropathies (Morena et al., 2019), FRDA (La Rosa et al., 2020) and A-H (Rahman & Copeland, 2019).	Anti-fatigue/weakness, /boosting energy/strength
Infectious agents	Microbial agents have been implicated in inducing hallmarks of ND such as misfolded protein aggregates, in AD and possibly ALS. Evidence includes pathogens co-localizing with AD pathology and anti-virals blocking Aβ and tau pathology (Itzhaki et al., 2016).	Anti-microbial
Aging	Risk factors associated with NDs include hallmarks of aging: e.g. genomic instability, telomere attrition, epigenetic alterations, impaired proteostasis, mitochondrial dysfunction, cellular senescence and stem cell exhaustion (López-Otín et al., 2013).	Anti-aging, promoting longevity
Vascular disease and hypertension	The majority of AD patients display vascular involvement. Atherosclerotic vascular wall thickening impedes O_2 and nutrient delivery to the brain, leading to neuronal loss (Lathe et al., 2014). Hypertension raises brain amyloid and tau deposition (Raz et al., 2019) and impairs cerebral blood vessels, leading to brain ischemic damage and impaired cognitive function (Iadecola et al., 2016).	Anti-hypertensive, cardiotonic, anti-thrombotic, anti-atherosclerotic, anti-stroke
Dyslipidemia/ hyperlipidemia/LSDs	Dyslipidemia-induced atherosclerosis is implicated in AD (Roher, 2015). In LSDs, enzyme deficiencies in lysosomes (which degrade cell waste) lead to toxic accumulation of macromolecules e.g. of unesterified cholesterol in NPC (Bräuer et al., 2019). Neurons are vulnerable to accumulating waste, leading to neurodegeneration (Toledano-Zaragoza & Ledesma, 2020).	Anti-hyperlipidemia; cholesterol-lowering
Non-ND neurological conditions	Agents alleviating these conditions can pass through the blood-brain barrier and have a therapeutic effect (Mendes et al., 2012) before being metabolized: thus may be of translational relevance in NDs.	Anti-epileptic, anxiolytic, alleviation of other neurological conditions

Abbreviations: AD, Alzheimer's disease; A-H, Alpers-Huttenlocher syndrome; ALS, Amyotrophic lateral sclerosis; ALS BD, Batten disease; CS, Cockayne syndrome; FRDA, Friedreich's ataxia; HD, Huntington's disease; LSD, MSA, Multiple system atrophy; MS, Multiple sclerosis; ND, neurodegenerative disease; NPC, Neimann-Pick disease type C;PD, Parkinson's disease; VD, Vascular dementia.

For references: See S8 Table references
