# Supplementary material for: Therapeutic roles of plants for 15 hypothesised causal bases of Alzheimer’s disease
Source: Nat Prod Bioprospect. 2022 Aug 23;12(1):34. doi: 10.1007/s13659-022-00354-z (PMC9395556; doi:10.1007/s13659-022-00354-z)
Supplement: Supplementary file 2 — Additional file 2. Table S2. List of species with ethnomedical use of neurodegenerative disease therapeutic potential. [file 13659_2022_354_MOESM2_ESM.pdf]

Additional Table S2. List of species with ethnomedical use of neurodegenerative disease therapeutic potential

Species	Family	Part of plant	Location(s) of ethnomedical use	Ethnomedical use	References
Aaronsohnia pubescens (Desf.) K.Bremer & Humphries Syn: *Matricaria pubescens* (Desf.) Sch.Bip.	Asteracea	AP, Le	Tassili N'Ajjer, Southern Algerian Sahara	Anti-microbial [measles]; anti-inflammatory [Fe]; anti-venom	Hammiche and Maiza, 2006
Abies pindrow (Royle ex D.Don) Royle	Pinaceae	Ba, Le, Ro	Kashmir, Pakistan; Jammu and Kashmir, India	Anti-microbial; [cough, bronc] anti-inflammatory [Fe, rheum]	Amjad et al., 2017; Kumar et al., 2009
Abuta grandifolia (Mart.) Sandw.	Menispermaceae	NS	Markets of Bogotá, Columbia	Anti-microbial; anti-inflammatory [Fe]	Bussmann et al., 2018
Abutilon hirsutum G. Don.	Malvaceae	Ro, WP	3 Kerala tribes, Western Ghats, India	Anti-microbial [TB]; anti-inflammatory [toothache]	Marjana et al., 2018
Abutilon indicum Sweet var. *welwitschii* E. G. Baker	Malvaceae	Le, St ba, WP	Andoman + Nicobar Is., India; Maonan people, Guangxi Zhuang, China; Orang Asli tribe, Malaysia; Rakhain people, Bangladesh; Kani people, Western Ghats, India	Anti-inflammatory [Fe, pain, rheum]; anti-microbial [STI, UTI]; anti-paralytic	Chander et al., 2014; Hong et al., 2015; Samuel et al., 2010; Hanif et al., 2009; Ayyanar and Ignacimuthu, 2011
Abutilon pannosum (G.Forst.) Schltdl. Syn: *Abutilon figarianum* Webb	Malvaceae	Ba, Ro, Se	Afar people, Rift Valley, Ethiopia; Jazan province, Saudi Arabia	Sedative, anti-microbial [STI, UTI, bronch, eye inf]	Teklehaymanot, 2017; Tounekti et al., 2019
Acacia ehrenbergiana Hayne	Leguminosae	Wo, Fr	Saudi Arabia	Anti-paralytic; anti-inflammatory	Tchatchedre et al., 2019
Acacia etbaïca (Schweinf.) Kyal. & Boatwr.	Leguminosae	Ro	Mana Angetu District, SE Ethiopia	Anti-paralytic [inability to walk properly]	Lulekal et al., 2008
Acacia farnesiana (L.) Willd. Syn: *Vachellia farnesiana* (L.) Wight & Arn.	Leguminosae	NS	Markets of Bogotá, Columbia	Anti-inflammatory [Fe]; anti-bacterial [UTI]	Bussmann et al., 2018
Acacia karroo Hayne	Leguminosae	Ro	Zimbabwe	Anti-convulsant	Maroyi, 2011
Acacia mellifera (M.Vahl) Benth.	Leguminosae	Ba, Le	Afar people, Rift Valley, Ethiopia	Anti-microbial [influenza]	Teklehaymanot, 2017
Acacia modesta Wall.	Leguminosae	Se	Himalayan region of Azad Jammu and Kashmir, Pakistan	Anti-paralytic	Rashid et al., 2015
Acacia nilotica (L.) Delile	Leguminosae	Ba, Ro, Le, Se, St	Satkhira District, Kalaroa, Bangladesh; Abyan territory, Yemen	Anti-microbial [dys, cold, pneum, ton'is]; boost immune system	Dulla and Jahan, 2017; Al-Fatimi, 2019
Acacia oerfota (Forssk.) Schweinf.	Leguminosae	Ba, Le	Afar people, Rift Valley, Ethiopia; Jazan province, Saudi Arabia	Anti-microbial [diphth, influenza]; anti-inflammatory [Fe]; anti-venom	Teklehaymanot, 2017; Tounekti et al., 2019
Acacia senegal (L.) Willd.	Leguminosae	Le	Afar people, Rift Valley, Ethiopia	Anti-microbial [mumps]	Teklehaymanot, 2017
Acacia seyal Delile Syn: *Acacia tortilis* (Forssk.) Hayne	Leguminosae	Ba, Le, Ro, Se	Tassili N'Ajjer, Southern Algerian Sahara	Anti-inflammatory [eye, Fe, rheum]; wound healing	Hammiche and Maiza, 2006
Acaena argentea Ruiz & Pav.	Rosaceae	Ba, Le	Mapuche people, Chile	Anti-microbial [STI]	Houghton and Manby, 1985
Acaena splendens Hook. & Arn.	Rosaceae	NS	Mapuche people, Argentina	Anti-inflammatory	Estomba et al., 2005

Acalypha fruticosa Forssk.	Euphorbiaceae	Le	Afar people, Rift Valley, Ethiopia	Anti-microbial [lung inf, *H. zoster*, ton'is]	Teklehaymanot, 2017
Acalypha indica L.	Euphorbiaceae	Le	Myanmar; Afar people, Rift Valley, Ethiopia; Satkhira District, Bangladesh; Jazan province, Saudi Arabia	Anti-inflammatory [rheum]; anti-microbial [RTI, cough, skin dis]; anti-venom	DeFilipps and Krupnick, 2018; Teklehaymanot, 2017; Dulla and Jahan, 2017; Tounekti et al., 2019
Acalypha wilkesiana Mull. Arg.	Euphorbiaceae	Le	South Nigeria	Anti-hypertensive; anti-microbial [skin inf]	Borokini et al., 2012
Acantholimon spp.	Plumbaginaceae	FL	Iran	Anti-microbial [pneum, cold]	Mosaddegh et al., 2013
Acanthospermum hispidum DC.	Compositae	Le	Benin; Togo	Memory improvement/ enhancement; anti-PD; anti-stroke	Adjanohoun et al., 1988; 1989: cited in Noe and Lehmann, 2012 [H168]; Kantati et al., 2016
Acanthus eminens C. B. Clarke	Acanthaceae	Le, Ro	Kakamega, Kenya	Anti-hypertensive	Odongo et al., 2018
Acanthus pubescens (Oliv.) Engl.	Acanthaceae	Ro	Mabira Forest, Uganda	Anti-microbial [measles]	Tugume et al., 2016
Acer monspessulanum subsp. *cinerascens* (Boiss.) Yalt. Syn: *Acer cinerascens* L.	Sapindaceae	Fr	Farashband nomadic tribe, Iran	Memory improvement/	Zali and Tahmasb, 2016
Achillea asiatica Serg.	Compositae	Le	Turkestan Range of south Kyrgystan	Wound healing	Pawera et al., 2016
Achillea millefolium L.	Compositae	FL, Le	Mestizo community, North Peru; Markets of Bogotá, Columbia; Chumash Indians, California, USA	Anti-microbil; wound dressing	Bussmann and Sharon, 2006; Bussmann et al., 2018; Timbrook, 1990
Achillea santolinoides subsp. *wilhelmsii* (K.Koch) Greuter. Syn: *Achillea wilhelmsii*	Compositae	FL	Iran	Anti-hypertensive	Mosaddegh et al., 2013
Achyranthes aspera L.	Amaranthaceae	Le, Ro, St	Satkhira District + Rakhain people Bangladesh; Uttarakhand, India; Trinidad; Jamaica; Tigrigna people, Central Eritrea; Tonga; Mizoram, India; Thari people of Nara Desert, Pakistan	Anti-paralytic; anti-inflammatory [Fe]; anti-microbial [ton'is, dys, tetanus, boil, pneum]; anti-epileptic; wound healing	Dulla and Jahan, 2017; Hanif et al., 2009; Sharma et al., 2013; Clement et al., 2015; Yemane et al., 2017; Croft and Tu'ipulotu, 1980; Rai and Lalramnghinglova, 2010; Qureshi and Bhatti, 2008
Achyranthes bidentata Blume	Amaranthaceae	Ro	Tian Mu Shan Biosphere Reserve, Zhejiang	Anti-inflammatory	Chaudhary et al., 2006
Achyrocline bogotensis (Kunth) DC	Compositae	NS	Columbia	Anti-microbial [skin dis, UTI]	Bussmann et al., 2018
Acmella caulirhiza Delile	Compositae	Le	Kenya; Burundi	Anti-microbial [mouth ulcer]; eye inflammation	Odongo et al., 2018; Baerts and Lehmann, 1989: cited in Noe and Lehmann, 2012 [H001]
Acmella oleracea Syn: *Spilanthes acmella var. oleracea* (L.) C.B.Clarke	Compositae	Le	Cataractes and Lukaya districts, D.R. Congo	Anti-microbial [throat /gum inf, dys]; enhance immune system	Latham and ku Mbuta, 2016

Acmella paniculata (Wall. ex DC.) R.K.Jansen Syn: *Spilanthes paniculata*	Compositae	WP	Lisu, Yunnan, China	Anti-microbial	Ji et al., 2005
Acokanthera schimperi (A.DC.) Schweinf.	Apocynaceae	Le, Ro	Afar people, Rift Valley, Ethiopia; Tigrigna people, Central Eritrea	Anti-microbial [polio, eye/kidney inf]; memory improvement; anti-inflammatory	Teklehaymanot, 2017; Tadeg et al., 2005: cited in Noe and Lehmann, 2012 [H168]; Yemane et al., 2017
Acorus gramineus Aiton	Ranunculaceae	Le	Ayta communities, Bataan, Philippines	Anti-microbial [UTI]	Tantengco et al., 2018
Acourtia microcephala DC. Syn: *Perezia microcephala* (DC.) A.Gray	Compositae	Ro	Chumash Indians, California, USA	Anti-microbial [cough]	Timbrook, 1990
Acridocarpus orientalis A.Juss	Malpighiaceae	NS	Middle East	Anti-paralytic	Abu-Rabia, 2012
Acridocarpus smeathmannii Engl.	Malpighiaceae	Le	Cataractes and Lukaya districts, D.R. Congo	Anti-inflammatory [Fe]	Latham and ku Mbuta, 2016
Acriopsis sp.	Orchidaceae	Bu	Seberida, Riau Province, Sumatra, Indonesia	Anti-inflammatory	Mahyar et al., 1991
Acrocomia aculeata (Jacq.) Lodd. ex R. Keith	Arecaceae	Le, Ro	Mato Grosso, Brazil	Anti-hypertensive; anxiolytic	Ribeiro et al., 2017
Actaea cimicifuga L. Syn: *Cimicifuga foetida* L.	Ranunculaceae	Rh	Lisu, Yunnan, China	Anti-microbial	Ji et al., 2005
Actiniopteris radiata (Sw.) Link	Pteridaceae	Le	Eastern Ghats, India	Anti-inflammatory [asthma]	Rao et al., 2006
Adansonia digitata L. Syn: *Adansonia sphaerocarpa*	Malvaceae	Ba, Fr, Le	3 states in SW Nigeria; Venda, South Africa; Senegal	Memory enhancement; anti-microbial [STI]; fortifying, anti-paralytic	Babawale et al., 2016; Arnold and Gulumian, 1984; Kerharo and. Adam, 1964, 1974: cited in Noe and Lehmann, 2012 [H130]
Adenocarpus bacquei Batt. & Pit.	Leguminosae	Le	High Atlas, Morocco	Anti-inflammatory	Fadili *et al*, 2017
Adenostoma sparsifolium Torr.	Rosaceae	NS	Chumash Indians, California, USA	Anti-paralytic	Timbrook, 1990
Adesmia boronioides Hook.f.	Leguminosae	NS	Mapuche people, Argentina	Anti-inflammatory	Estomba et al., 2005
Adiantum poiretii Wikstr.	Pteridaceae	FL, Le	Loja and Zamora-Chinchipe, Ecuador	Anti-inflammatory; anti-microbial [influenza, pneum, cough]	Tene et al., 2007
Aegle marmelos (L.) Corr.	Rutaceae	Ba, Fr, Le, Ro, Se	Rakhain people, Bangladesh; 3 Kerala tribes, Western Ghats, India; Eastern Ghats, India; Mizoram, India	Anti-inflammatory [Fe, headache]; anti-microbial [dys]	Hanif et al., 2009; Marjana et al., 2018; Rao et al., 2006; Rai and Lalramnghinglova, 2010
Aerva javanica (Burm.f.) Juss. ex Schult.	Amaranthaceae	AP, Fr, Le, Ro	Afar people, Rift Valley, Ethiopia; Jazan Region, Saudi Arabia; Abyan territory, Yemen; Tassili N'Ajjer, Southern Algerian Sahara	Anti-microbial [cough, STI, UTI]; neurological disorders; anti-inflammatory [headache]; anti-venom	Teklehaymanot, 2017; Tounekti et al., 2019; Al-Fatimi, 2019; Hammiche and Maiza, 2006
Aerva lanata (L.) Juss. ex Schult.	Amaranthaceae	Le, WP	Andoman + Nicobar Is., India; Kani people, Western Ghats, India; Eastern Ghats, India	Anti-inflammatory [Fe, asthma, headache]; anti-hypertensive; anti-venom	Chander et al., 2014; Ayyanar and Ignacimuthu, 2011; Rao et al., 2006
Aerva sanguinolenta (L.) Blume	Amaranthaceae	Ro	Eastern Ghats, India	Anti-inflammatory [Fe}	Rao et al., 2006

Aeschynanthus parasiticus (Roxb.) Wall.	Gesneriaceae	Le	Chin people, Myanmar	Anti-microbial [leprosy, boils, ton'is, TB]	Ong et al., 2018
Aeschynanthus sikkimensis (C.B. Clarke) Stapf.	Gesneriaceae	FL, Le	Magar and Majhi people, Nepal; Mizoram, India	Anti-inflammatory [Fe]; anti-microbial [ton'is]	Malla et al., 2015; Rai and Lalramnghinglova, 2010
Aesculus chinesis Bunge	Sapindaceae	Se	Shaanxi, China	Anti-inflammatory	Teng et al., 2011
Aesculus hippocastanum L.	Sapindaceae	Se	Markets of Bogotá, Columbia	Anti-inflammatory	Bussmann et al., 2018
Aframomum alboviolaceum (Ridl.) K.Schum.	Zingiberaceae	Le	Cataractes and Lukaya districts, D.R. Congo	Anti-inflammatory [Fe]	Latham and ku Mbuta, 2016
Aframomum angustifolium (Sonn.) K.Schum.	Zingiberaceae	Fr, Le, Ro	Maromizaha forest, Madagascar; Mabira Forest, Uganda	Anti-microbial [yell fev]; anti-inflammatory [Fe]; boost immune system	Riondato et al., 2019; Tugume et al., 2016
Aframomum melegueta (Roscoe) K.Schum.	Zingiberaceae	Fr, Se	Benin; Togo; 3 states in SW Nigeria; Baka Pygmies, Gabon	Hemiplegia/ paraplegia/ polio; memory improvement/ enhancement; anti-microbial [diarrh]; stroke; TBI	Adjanohoun et al., 1989; Adjanohoun et al., 1988; ku Mbuta 2012: cited in Noe and Lehmann, 2012 [H130, H168]; Babawale et al., 2016; Betti et al., 2013; Kantati et al., 2016
Afrostyrax lepidophyllum Mildbr.	Huaceae	Le	Masako Forest Reserve, D.R. Congo	Anti-hypertensive	Mbula et al., 2015
Agapetes mannii Hemsl.	Ericaceae	Ro	Chin people, Myanmar	Anti-microbial [boil]	Ong et al., 2018
Agarista salicifolia (Lam.) G.Don Syn: *Agauria salicifolia* (Lam.) Oliv.	Ericaceae	Ba, Le	NW Cameroon	Anti-microbial [STI]	Focho et al.,2009
Agave americana L.	Asparagaceae	Ju	Loja and Zamora-Chinchipe, Ecuador	Anti-inflammatory; anti-microbial [cold]	Tene et al., 2007
Agelaea pentagyna (Lam.) Baill.	Connaraceae	Ro	Cataractes and Lukaya districts, D.R. Congo	Anti-inflammatory [Fe], anti-microbial	Latham and ku Mbuta, 2016
Aglaia odorata Lour	Meliaceae	FL	Orang Asli tribe, Malaysia	Anti-inflammatory [Fe]	Samuel et al., 2010
Agrimonia eupatoria L.	Rosaceae	AP; FL	Catalonia, Spain; Pollino National Park, Southern Italy	Anti-inflammatory; improve brain functionality	Bonet et al., 1999; Di Sanzo et al., 2013
Ajuga austroiranica Rech.f.	Lamiaceae	AP	Iran	Anti-hypertensive	Mosaddegh et al., 2013
Ajuga integrifolia Buch.-Ham. ex D.Don Syn*: Ajuga bracteosa*	Lamiaceae	AP	Kashmir, Pakistan	Anti-inflammatory	Amjad et al., 2017
Albizia adianthifolia (Schumach) W. F. Wight	Fabaceae	Ro	Angola Congo-Brazaville	Anti-microbial [bronch]; eye inflammation	Adjanohoun et al., 1988: cited in Noe and Lehmann, 2012 [H037]; Bouquet, 1969: cited in Noe and Lehmann, 2012 [H001]
Albizia anthelmintica (A. Rich.) Brongn. R	Fabaceae	Ro	Venda, South Africa	Anti-microbial [STI]	Arnold and Gulumian, 1984
Albizia grandibracreata Taub.	Fabaceae	Le	Mabira Forest, Uganda	Anti-microbial [yell fev]	Tugume et al., 2016
Albizia lebbeck (L.) Benth	Fabaceae	Ba, FL, Le, Se	Saudi Arabia	Anti-paralytic, anti-venom, anti-microbial [dys, STI]	Youssef, 2013
Albizia versicolor Welw. ex Oliver	Fabaceae	Ba, Ro	Venda, South Africa; Kimboza forest, Tanzania	Anti-microbial [STI, boil]	Arnold and Gulumian, 1984; Amri and Kisangau, 2012

Albizia zygia (DC.) J.F.Macbr.	Fabaceae	Ba, Ro	Congo-Brazaville	Memory improvement/ enhancement	Bouquet, 1969: cited in Noe and Lehmann, 2012 [H168]
Alcea calvertii (Boiss.) Boiss.	Malvaceae	FL	Iran	Anti-viral [cold]	Mosaddegh et al., 2013
Alcea rosea (L.) Cavanilles	Malvaceae	WP	Mestizo community, North Peru; Markets of Bogotá, Columbia	Anti-inflammatory [throat]; anti-microbial [bronch]	Bussmann and Sharon, 2006; Bussmann et al., 2018
Alchornea cordifolia (Schumach. & Thonn.) Müll.Arg.	Euphorbiaceae	Ro	Cataractes and Lukaya districts, D.R. Congo	Anti-microbial [diarrh, dys]	Latham and ku Mbuta, 2016
Alchornea laxiflora (Benth.) Pax & K. Hoeffm	Euphorbiaceae	Le	South Nigeria	Anti-inflammatory	Borokini et al., 2012
Alibertia patinoi (Cuatrec.) Delprete & C.H. Perss.	Rubiaceae	NS	Markets of Bogotá, Columbia	Anti-microbial [UTI]	Bussmann et al., 2018
Allagoptera campestris (Mart.) Kuntze	Arecaceae	Le, St	Mato Grosso, Brazil	Anti-microbial; anti-inflammatory	Ribeiro et al., 2017
Allamanda cathartica L.	Apocynaceae	Le	Markets of Bogotá, Columbia; Antioquia, Columbia	Anti-microbial [influenza]; anti-venom; anti-inflammatory [headache]	Bussmann et al., 2018; Vasquez et al., 2013
Allanblackia gabonensis (Pellegr.) Bamps Syn: *Allanblackia floribunda* var.*gabonensis* Pellegr	Clusiceae	Fr, Le,	Cataractes and Lukaya districts, D.R. Congo	anti-microbial [bronch, cough]; anti-inflammatory [asthma]	Latham and ku Mbuta, 2014
Allium ascalonium L.	Amaryllidaceae	Le, Ro	Kimboza forest, Tanzania	Anti-fungal	Amri and Kisangau, 2012
Allium carolinianum DC.	Amaryllidaceae	Bu	Turkestan Range of south Kyrgystan	Anti-inflammatory	Pawera et al., 2016
Allium cepa L.	Amaryllidaceae	Bu, Le	Martinique; Bas-Congo Province, D.R. Congo; Carib people, Livingston, Guatamala; Ayta communities, Bataan, Philippines; Sundanese community, West Java; Agro Nocerino Sarnese, Southern Italy	Anti-inflammatory [Fe]; anti-hypertensive; anti-hypercholesterolaemia anti-PD; anti-microbial [measles]; wound healing [burn]	Girón et al., 1991; Longuefosse and Nossin, 1996; Kembelo, 2003: cited in Noe and Lehmann, 2012 [H130x]; Tantengco et al., 2018; Roosita et al., 2008; Motti and Motti, 2017
Allium fistulosum L.	Amaryllidaceae	Ro, WP	Maonan people, Guangxi Zhuang, China; Columbia	Anti-microbial [cold]; anti-memory loss	Hong et al., 2015; Cadena-González et al., 2013
Allium jesdianum Boiss. & Buhse	Amaryllidaceae	Le	Iran	Anti-microbial [UTI, cold]	Mosaddegh et al., 2013
Allium oreoprasum Schrenk	Amaryllidaceae	WP	Central Himalayas, Nepal	Anti-microbial [ton'is, cough, cold]; anti-inflammatory [headache]	Bhattarai et al., 2006
Allium rubellum M.Bieb.	Amaryllidaceae	Bu	Iran	Anti-hyperlipidemic	Mosaddegh et al., 2013
Allium sativum L.	Amaryllidaceae	Bu, Le	Carib people, Livingston, Guatamala; Antioquia, Columbia; Mato Grosso, Brazil; Kimboza forest, Tanzania; Nigeria; Beni-Sueif, Egypt; Ayta people, Philippines; Uttarakhand, India; Kosovo; Sundanese community, West	Anti-hypertensive; anti-venom; anti-inflammatory [Fe]; anti-viral [HIV, rabies, measles, influenza]; paralysis/ hemiplegia/ polio/ paraplegia; memory improvement/ enhancement; anti-epileptic; immunostimulant; anti-aging; cell regeneration; stroke	Girón et al., 1991; Vasquez et al., 2013; Amri and Kisangau, 2012; Ribeiro et al., 2017; Longuefosse and Nossin, 1996;; Tantengco et al., 2018; Ainslie, 1937; Sameh, 2011: cited in Noe and Lehmann, 2012 [H130, H168]; Sharma et al., 2013; Mustafa et al., 2015; Roosita et

			Java; Taounate, North Morocco; Edremit Gulf, Turkey; Togo		al., 2008; El-Hilaly et al., 2003; Polat and Satıl, 2012; Kantati et al., 2016
Allium schoenoprosum L.	Amaryllidaceae	WP	Martinique	Anti-inflammatory [Fe]	Longuefosse and Nossin, 1996
Alocasia macrorrhizos (L.) G.Don	Araceae	AP	Marquesas Islands, French Polynesia	Anti-microbial [boil]	Girardi et al., 2015
Aloe buettneri A.Berger Syn: *A. congolensis*	Xanthorrhoeaceae	Le	Cataractes and Lukaya districts, D.R. Congo	Anti-inflammatory	Latham and ku Mbuta, 2016
Aloe harlana Reynolds	Xanthorrhoeaceae	Le	Oromo people, Harla, Eastern Ethiopia	Anti-venom; anti-inflammatory; anti-fungal	Belayneh and Bussa, 2014
Aloe rubroviolacea Schweinf.	Xanthorrhoeaceae	Le	Abyan territory, Yemen	Anti-microbial, anti-inflammatory [Fe, psoriasis]	Al-Fatimi, 2019
Aloe sp.	Xanthorrhoeaceae	Ro	Maromizaha forest, Madagascar	Anti-fatigue	Riondato et al., 2019
Aloe littoralis Baker	Xanthorrhoeaceae	Le	Angola	Eye inflammation	Urso et al., 2016: cited in Noe and Lehmann, 2012 [H001]
Aloe trichosantha A.Berger	Xanthorrhoeaceae	Le	Afar people, Rift Valley, Ethiopia	Anti-microbial [diphth]	Teklehaymanot, 2017
Aloe vacillans Forssk	Xanthorrhoeaceae	Le	Abyan territory, Yemen	Anti-inflammatory [Fe. pain, rheum]; boost immune system	Al-Fatimi, 2019
Aloe vera (L.) Burm.f. Syn: *Aloe barbedensis* Mill.	Xanthorrhoeaceae	Le, sap	Arabian peninsula; Mato Grosso, Brazil; Trinidad; Oman; Columbia; Maharashtra, India	Anti-microbial [UTI, bronch, influenza]; anti-inflammatory [Fe, ext inflam]; anti-hypertensive; wound healing	Ribeiro et al., 2017; Longuefosse and Nossin, 1996; Clement et al., 2015; Cadena-González et al., 2013; Wadankar et al., 2011
Aloysia citriodora Paláu Syn: *Aloysia triphylla* (L'Hér.) Britton	Verbenaceae	FL, Le	Markets of Bogotá, Columbia; Loja and Zamora-Chinchipe, Ecuador	Anti-microbial [rabies]; anti-inflammatory [Fe	Bussmann et al., 2018; Tene et al., 2007
Aloysia virgata (Ruiz & Pav.) Juss	Verbenaceae	Le	Bolivian Amazon	Anti-microbial [cough]	Bourdy et al., 2000
Alpinia calcarata Rosc.	Zingiberaceae	Tu	3 Kerala tribes, Western Ghats, India	Anti-inflammatory [Fe]	Marjana et al., 2018
Alpinia galanga (L.) Willd.	Zingiberaceae	Rh	Kani people, Western Ghats, India	Anti-inflammatory [Fe, headache]; anti-microbial [cold]	Ayyanar and Ignacimuthu, 2011
Alpinia purpurata K. Schum	Zingiberaceae	Le	Caribbean coast of Columbia	Anti-inflammatory [headache]	Gómez-Estrada et al., 2011
Alpinia zerumbet (Pers.) B.L.Burtt & R.M.Sm.	Zingiberaceae	FL, Le, Ro	Martinique; Atlantic Forest, Brazil	Anti-inflammatory [Fe]; anti-microbial [influenza]	Longuefosse and Nossin, 1996; Gazzaneo et al., 2005
Alstonia boonei De Wild.	Apocynaceae	Ba, Ro	NW Cameroon	Memory improvement	Focho et al.,2009
Alstonia macrophylla Wall. ex G.Don	Apocynaceae	Le	Andoman + Nicobar Is., India	Anti-paralytic; anti-inflammatory [Fe]	Chander et al., 2014
Alstonia scholaris (L.) R. Br.	Apocynaceae	Ba, Le, Sh	Seberida, Riau Province, Sumatra, Indonesia, India; Myanmar; Chittagong Hill Tracts, Bangladesh	Anti-paralytic; cerebral palsy; anti-inflammatory; anti-viral; anti-hypertensive; anti-venom	Perry and Metzger, 1980: cited in DeFilipps and Krupnick, 2018; Mahyar et al., 1991; Rajakumar and Shivanna, 2009; Ong et al., 2018; Kadir et al., 2015

Alstonia spatulata Blume	Apocynaceae	sap	Dayaknese Communities, W Kalimantan, Indonesia	Anti-inflammatory [toothache]	Diba et al., 2013
Alternanthera brasiliana (L.) Kuntze	Amaranthaceae	Le, Ro	Mato Grosso, Brazil	Anti-microbial [influenza, pneum]; anti-inflammatory	Ribeiro et al., 2017
Alternanthera halmifolia (Lam.) Standl. ex Pittier	Amaranthaceae	WP	Mestizo community, North Peru	Anti-inflammatory	Bussmann and Sharon, 2006
Alternanthera lanceolata (Benth.) Schinz	Amaranthaceae	Le	Columbian Andes	Anti-inflammatory [Fe]	Cadena-González et al., 2013
Alternanthera porrigens var. *piurensis* (Standl.) Eliasson	Amaranthaceae	FL, Le	Loja and Zamora-Chinchipe, Ecuador	Anti-microbial [influenza]	Tene et al., 2007
Alternanthera ramosissima (Mart.) Chodat & Hassl.	Amaranthaceae	Le	Mato Grosso, Brazil	Anti-microbial	Ribeiro et al., 2017
Alternanthera sessilis (L.) R.Br. ex DC.	Amaranthaceae	Le, WP	Maonan people, Guangxi Zhuang, China; Kakamega County, Kenya; Cataractes and Lukaya districts, D.R. Congo	Anti-inflammatory; anti-microbial [eye inf, diarrh, dys]	Hong et al., 2015; Odongo et al., 2018; Latham and ku Mbuta, 2016
Althaea officinalis L.	Malvaceae	FL, Le	Mediterranean	Anti-inflammatory [Fe]	Cadena-González et al., 2013
Amaranthus hybridus L.	Amaranthaceae	Le, FL, St	Loja and Zamora-Chinchipe, Ecuador; Mestizo community, North Peru	Anti-inflammatory	Tene et al., 2007; Bussmann and Sharon, 2006
Amaranthus viridis L. Syn: *Amaranthus gracilis* Desf.	Amaranthaceae	WP	Maori people, Cook Islands; Fiji;	Anti-microbial [UTI]; wound healing [with coconut oil]; Anti-paralytic	Whistler, 1985; Singh, 1986
Amaranthus graecizans L.	Amaranthaceae	Le	Abyan territory, Yemen	Anti-inflammatory [rheum]	Al-Fatimi, 2019
Ambrosia peruviana Willd. Syn: *Ambrosia cumanensis* Kunth	Asteraceae	Le, St	Colombian Andes; Mestizo community, North Peru	Anti-inflammatory [rheum, pain]; anti-microbial	Cadena-González et al., 2013; Bussmann and Sharon, 2006
Ambrosia psilostachya DC.	Asteracea	NS	Chumash Indians, California, USA	Anti-inflammatory [Fe]	Timbrook, 1990
Ammannia baccifera L.	Lythraceae	WP	Eastern Ghats, India	Anti-venom	Rao et al., 2006
Amyema sp.	Loranthaceae		Conis Santana National Park, East Timor	Anti-mcrobial [UTI]	Collins et al., 2007
Amicia glandulosa Kunth	Fabaceae	Ro	Loja and Zamora-Chinchipe, Ecuador	Anti-inflammatory	Tene et al., 2007
Ampelodesmos mauritanicus (Poir.) T.Durand & Schinz	Poaceae	AP	M'Sila, North Algeria	Anti-hypertensive	Boudjelal et al., 2013
Amygdalus bucharica Korsh.	Rosaceae	Se	Turkestan Range of south Kyrgystan	Anti-inflammatory [asthma, pain]	Pawera et al., 2016
Anacardium giganteum Hancock ex Engl.	Anacardiaceae	Ba, Fr	Mato Grosso, Brazil	Anti-microbial	Ribeiro et al., 2017
Anacardium humile A.St.-Hil.	Anacardiaceae	Ba, Fr, Le, Ro	Mato Grosso, Brazil	Anti-microbial	Ribeiro et al., 2017
Anacardium occidentale L.	Anacardiaceae	Fr, St ba	Rio Jauaperi, Brazilian Amazon; Peru; Guinea-Bissau; Kani people, Western Ghats, India; Bolivian Amazon	Anti-microbial [dys]; anti-inflammatory [Fe, asthma, headache]; neurological disorders; wound	Pedrollo et al., 2016; Ribeiro et al., 2017; Odonne et al., 2013; Romeiras et al., 2012; Ayyanar

					and Ignacimuthu, 2011; Bourdy et al., 2000
Anacardium spruceanum Benth. ex Engl.	Anacardiaceae	St ba	Rio Jauaperi, Brazilian Amazon	Anti-inflammatory; anti-microbial [dys]	Pedrollo et al., 2016
Anacyclus pyrethrum (L.) Lag.	Asteraceae	Ro	Middle East	Anti-paralytic	Boulos, 1983: cited in Abu-Rabia, 2012
Ananas comosus (L.) Merr.	Bromeliaceae	Fr, Le	Kani people, Western Ghats, India; Madagascar	Anti-microbial [STI]; anti-inflammatory [asthma]	Ayyanar and Ignacimuthu, 2011; Riondato et al., 2019
Anastatica hierochuntica L.	Brassicaceae	WP	Tassili N'Ajjer, Southern Algerian Sahara	Anti-epileptic	Hammiche and Maiza, 2006
Anchietea pyrifolia (Mart.) G.Don	Violaceae	Ro, Vi	Mato Grosso, Brazil	Anti-microbial; anti-inflammatory	Ribeiro et al., 2017
Anchusa azurea Mill	Boraginaceae	AP, FL	M'Sila, North Algeria; Edremit Gulf, Turkey	Anti-inflammatory; anxiolytic	Boudjelal et al., 2013; Polat and Satıl, 2012
Anchusa italica Mill.	Boraginaceae	FL	Iran	Anti-viral [cold, influenza]	Mosaddegh et al., 2013
Andira cujabensis Benth.	Fabaceae	Ba, Le, St	Mato Grosso, Brazil	Anti-microbial; anti-inflammatory [Fe]	Ribeiro et al., 2017
Andira inermis (W.Wright) DC. Syn: *Geoffroea inermis* (Wright) Wright	Fabaceae	Ba	Martinique	Anti-microbial [Measles]	Longuefosse and Nossin, 1996
Andrographis paniculata (Burm.f.) Nees	Acanthaceae	Le	Ayta communities, Bataan, Philippines; Sundanese community, West Java	Anti-microbial [cough, diarrh]; anti-inflammatory [Fe]<br>*Toxicity Report	Tantengco et al., 2018; Roosita et al., 2008<br><br>* Ismael et al., 2017
Anemarrhena asphodeloides Bunge	Asparagaceae	Ro	Shaanxi, China	Anti-inflammatory; anti-microbial	Teng et al., 2011
Anemone multifida Poir.	Ranunculaceae	NS	Mapuche, Argentina	Anti-inflammatory	Estomba et al., 2005
Anemopsis californica (Nutt.) Hook. & Arn.	Saururaceae	Ro	Chumash Indians, California, USA	Wound healing; anti-mcrobial [cough]	Timbrook, 1990
Anethum graveolens L.	Apiaceae	Le	Martinique	Anti-microbial [influenza]	Longuefosse and Nossin, 1996
Angiopteris sp.	Marattiaceae	WP	Dayaknese Communities, W Kalimantan, Indonesia	Anti-inflammatory [headache	Diba et al., 2013
Angraecum eichlerianum Kraenzl.	Orchidaceae	Le	Sagamu, SW Nigeria	Memory enhancement	Elufioye et al., 2012
*Aniba canellila (*Kunth) Mez	Lauraceae	St ba	Rio Jauaperi, Brazilian Amazon	Anti-inflammatory [Fe, headache]	Pedrollo et al., 2016
Anisochilus carnosus (L.f.) Wall.	Lamiaceae	Le, RO	Eastern Ghats, India	Anti-inflammatory [Fe]	Rao et al., 2006
Anisomeles indica (L.) Kuntze Syns: *Epimeredi indica; Nepeta indica*	Lamiaceae	WP	Maonan people, Guangxi Zhuang, China	Anti-microbial	Hong et al., 2015
Anisotes trisulcus (Forssk.) Nees	Acanthaceae	Fr Ro St	Abyan territory, Yemen	Anti-microbial, anti-venom, boost immune system	Al-Fatimi, 2019
Annickia chlorantha (Oliv.) Setten & Maas Syn: *Enantia chlorantha* Oliv.	Annonaceae	St	Tropical rain forest, Central + S Cameroon	Anti-viral [hepatitis]	Ngono Ngane et al., 2011
Annona coriacea Mart.	Annonaceae	Ba	Mato Grosso, Brazil	Anti-thrombotic	Ribeiro et al., 2017
Annona montana Macfad. Syn: *Annona marcgravii* Mart.	Annonaceae	Le	Atlantic Forest, Brazil	Anti-inflammatory	Gazzaneo et al., 2005

Annona muricata L.	Annonaceae	Le	Orang Asli tribe, Malaysia; Martinique; Jamaica; Mestizo community, North Peru; Trinidad; Dayaknese Communities, W Kalimantan, Indonesia; Togo	Anti-hypertensive; anti-inflammatory [Fe]; anti-microbial [dys]; anti-AD, anti-dementia; TBI	Samuel et al., 2010; Clement et al., 2015; Longuefosse and Nossin, 1996; Bussmann and Sharon, 2006; Diba et al., 2013; Kantati et al., 2016
Annona senegalensis Pers. Syns: *Annona arenaria* Thonn., *Annona chrysophylla* Bojer	Annonaceae	AP, Le, Ro	Benin; D.R. Congo; Guinea-Bissau; Togo	Hemiplegia/ paraplegia/ polio; memory improvement/ enhancement/dementia; neurological disorders; epilepsy	Adjanohoun et al., 1988, 1989: cited in Noe and Lehmann, 2012 [H130, H168]; Romeiras et al., 2012; Kantati et al., 2016
Annona squamosa L.	Annonaceae	Le	Eastern Ghats, India	Anti-venom	Rao et al., 2006
Annonidium mannii (Oliv.) Engl. & Diels	Annonaceae	Ba, Fr, Ro, Se	Baka Pygmies, Gabon; Cataractes and Lukaya districts, D.R. Congo	Anti-microbial [STI, dys, pneum]	Betti et al., 2013; Latham and ku Mbuta, 2016
Anoda cristata (L.) Schl.	Malvaceae	Le, St	Mexico, Central America; Columbia	Anti-inflammatory [Fe]	Cadena-González et al., 2013
Anredera cordifolia Tenore	Basellaceae	Le, St	Columbian Andes	Anti-inflammatory [rheum, pain]; anti-microbial [skin inf]	Cadena-González et al., 2013
Antherotoma senegambiensis (Guill. & Perr.) Jacq.-Fél. Syn: *Dissotis senegambiensis* (Guill. & Perr.) Triana	Melastomataceae	NS	Rwanda	Eye inflammation	Kayonga and Habiyaremye, 1987: cited in Noe and Lehmann, 2012 [H001]
Anthocleista djalonensis A.Chev.	Gentianaceae	Le	Togo	Anti-epileptic; anti-paralytic	Kantati et al., 2016
Anthocleista madagascariensis Baker	Gentianaceae	Ba	Maromizaha forest, Madagascar	Anti-fatigue	Riondato et al., 2019
Anthurium sp.	Araceae	Ro	Guaymi indians, Panama	Anti-microbial [diarrh]	Joly et al., 1987
Antidesma membranaceum Müll.Arg.	Phyllanthaceae	Le, Ro	Cataractes and Lukaya districts, D.R. Congo	Anti-microbial	Latham and ku Mbuta, 2016
Aphelandra cirsioides Lindau	Acanthaceae	WP	Mestizo community, North Peru	Anti-microbial	Bussmann and Sharon, 2006
Aphelandra pilosa Leonard	Acanthaceae	NS	Markets of Bogotá, Columbia	Anti high cholesterol	Bussmann et al., 2018
Aphelandra tonduzii Leonard.	Acanthaceae	WP	Guaymi indians, Panama	Anti-microbial [mumps]; anti-inflammatory [Fe]	Joly et al., 1987
Apium graveolens L.	Apiaceae	Le	Martinique; Markets of Bogotá, Columbia	Anti-hypertensive; anti-inflammatory [Fe]	Longuefosse and Nossin, 1996; Bussmann et al., 2018
Apodanthera smilacifolia Cogn.	Cucurbitaceae		Mato Grosso, Brazil	Anti-microbial	Ribeiro et al., 2017
Aquilaria crassna Pierre ex Lecomte	Thymelaeaceae	Ro	Bac Huong Hoa nature reserve, Vietnam	Anti-inflammatory [Fe]	Lee et al., 2019
Aquilaria malaccensis Lam.	Thymelaeaceae	Wo	Myanmar	Anti-venom; anti-paralytic; anti-viral [s'pox]	Perry and Metzger, 1980: cited in DeFilipps and Krupnick, 2018
Araucaria araucana (Mol.) Koch (ind)	Araucariaceae	Fe	Mapuche people, Chile	Anti-inflammatory [Fe]	Houghton and Manby, 1985
Arctium lappa L.	Asteraceae	Fr	Shaanxi, China; Maonan people, Guangxi Zhuang, China; Naxi	Anti-inflammatory; anti-hypertensive; anti-microbial; anti-fatigue	Teng et al., 2011; Hong et al., 2015; Zhang et al., 2015

			people, Northwest Yunnan, China		
Ardisia crenata Sims	Primulaceae	Le, WP	Orang Asli tribe, Malaysia	Anti-inflammatory; anti-microbial [*H. zoster*, measles]	Samuel et al., 2010
Ardisia gigantifolia Stapf	Primulaceae	Rh, WP	Maonan people, Guangxi Zhuang, China	Anti-paralytic	Hong et al., 2015
Areca catechu L.	Arecaceae	Fr	Kani people, Western Ghats, India	Wound healing	Ayyanar and Ignacimuthu, 2011
Argemone mexicana L.	Papaveraceae	Le, FL, St	Loja and Zamora-Chinchipe, Ecuador; Mestizo community, North Peru; Abyan territory, Yemen	Anti-microbial [int inf]; anti-inflammatory; wound healing	Tene et al., 2007; Bussmann and Sharon, 2006; Al-Fatimi, 2019
Arisaema flavum (Forsk.) Schott.	Araceae	Rh	Himachal Pradesh, NW Himalaya, India	Anti-microbial [RTI]	Vidyarthi et al., 2013
Arisaema heterophyllum Blume	Araceae	Tu	Maonan people, Guangxi Zhuang, China	Anti-hypertensive	Hong et al., 2015
Aristolochia anguicida Jacq.	Aristolochiaceae	Le St	Caribbean coast of Columbia	Anti-inflammatory<br>* Toxicity Report	Gómez-Estrada et al., 2011<br>* Ding and Chen, 2012
Aristolochia bracteolata Lam.	Aristolochiaceae	Le, WP	Afar people, Rift Valley, Ethiopia; Abyan territory, Yemen	Anti-microbial [diphth, eye inf], HSV; anti-venom<br>* Toxicity Report	Teklehaymanot, 2017; Al-Fatimi, 2019<br>* Ding and Chen, 2012
Aristolochia esperanzae Kuntze	Aristolochiaceae	Le, Ro, Vi	Mato Grosso, Brazil	Anti-microbial; anti-inflammatory<br>* Toxicity Report	Ribeiro et al., 2017<br>* Ding and Chen, 2012
Aristolochia grandiflora Sw.	Aristolochiaceae	NS	Markets of Bogotá, Columbia	Muscular paralysis<br>* Toxicity Report	Bussmann et al., 2018<br>* Ding and Chen, 2012
Aristolochia indica L.	Aristolochiaceae	Ro, WP	Myanmar	Anti-venom; anti-inflammatory [Fe]; anti-microbial [cough, cold]<br>* Toxicity Report	DeFilipps and Krupnick, 2018<br>* Ding and Chen, 2012
Aristolochia ringens Vahl.	Aristolochiaceae	Le, St	Brazil; Columbia	Anti-inflammatory [rheum]; anti-microbial [skin inf]<br>* Toxicity Report	Cadena-González et al., 2013<br>* Ding and Chen, 2012
Aristolochia rugosa Lam.	Aristolochiaceae	Ro	Trinidad	Anti-inflammatory [Fe]; anti-microbial [cold]<br>* Toxicity Report	Clement et al., 2015<br>* Ding and Chen, 2012
Aristolochia tagala Cham.	Aristolochiaceae	Ro	Ayta communities, Bataan, Philippines	Anti-inflammatory [Fe]<br>* Toxicity Report	Tantengco et al., 2018<br>* Ding and Chen, 2012
Aristotelia chilensis (Molina) Stuntz Syn: *Aristotelia macqui* L'Hér	Elaeocarpaceae	Ba, Le	Chile	Anti-inflammatory<br>* Toxicity Report	Houghton and Manby, 1985<br>* Ding and Chen, 2012
Arnebia euchroma (Royle) I.M.Johnst.	Boraginaceae	Ro	Turkestan Range of south Kyrgystan	Anti-microbial [TB, cough, influenza]	Pawera et al., 2016
Arnica montana L.	Asteraceae	NS	Markets of Bogotá, Columbia	Anti-inflammatory	Bussmann et al., 2018
Artemisia absinthium L.	Asteraceae	AP, Le	Middle East; Martinique; Carib people, Livingston, Guatamala; Turkestan Range of south	Anti-paralytic; anti-inflammatory [Fe, pain]; anti-viral; anti-hypertensive	Girón et al., 1991; Ibn al-Baytar, 1992: cited in Abu-Rabia, 2012; Longuefosse and Nossin, 1996;

			Kyrgystan; M'Sila, North Algeria; Taounate, North Morocco		Pawera et al., 2016; Boudjelal et al., 2013; El-Hilaly et al., 2003
Artemisia annua L.	Asteraceae	Le, WP	Maonan people, Guangxi Zhuang, China; Cataractes and Lukaya districts, D.R. Congo; Georgia	Anti-inflammatory; anti-microbial [TB]; boost immunity; wound healing	Hong et al., 2015; Latham and ku Mbuta, 2016; Bussmann et al., 2016
Artemisia arborescens (Vaill.) L.	Asteraceae	WP	Abyan territory, Yemen	Anti-microbial [UTI]; anti-inflammatory [headache, Fe]; peripheral neuropathy	Al-Fatimi, 2019
Artemisia campestris L	Asteraceae	AP	M'Sila, North Algeria	Anti-hypertensive	Boudjelal et al., 2013
Artemisia gmelinii Weber ex Stechm.	Asteraceae	FL, Le, St	Central Himalayas, Nepal	Anti-microbial [cough, cold, sore throat]; anti-inflammatory [Fe]	Bhattarai et al., 2006
Artemisia indica Willd.	Asteraceae	Le	Chin people, Myanmar	Anti-microbial [influenza]	Ong et al., 2018
Artemisia judaica L	Asteraceae	AP	Tassili N'Ajjer, Southern Algerian Sahara	Anti-inflammatory [Fe]	Hammiche and Maiza, 2006
Artemisia scoparia Waldst. & Kitam.	Asteraceae	Le	Hakka people, Guangdong, China; Tian Mu Shan, Zhejiang, China	Anti-viral [cold]; anti-hypertensive	Au et al., 2008
Artemisia sodiroi Hieron. ex Sodiro	Asteraceae	Le	Loja and Zamora-Chinchipe, Ecuador	Anti-inflammatory; anti-microbial [int inf]	Tene et al., 2007
Artemisia stolonifera (Maxim) Kom.	Asteraceae	Ro	Tian Mu Shan, Zhejiang, China	Anti-microbial [leprosy]	Chaudhary et al., 2006
Artemisia vulgaris L.	Asteraceae	Le	Ayta communities, Bataan, Philippines; Markets of Bogotá, Columbia; Potosi, Bolivia	Anti-microbial [sore throat, cold, cough]; nervous disorders; anti-epileptic; anti-inflammatory [rheum]	Tantengco et al., 2018; Bussmann et al., 2018; Fernandez et al., 2003
Arthrostemma ciliatum Pav. Ex D. Don	Melastomataceae	NS	Markets of Bogotá, Columbia	Anti-inflammatory [Fe]	Bussmann et al., 2018
Artocarpus altilis (Parkinson ex F.A.Zorn) Fosberg Syn: *Artocarpus communis*	Moraceae	Le	Loja and Zamora-Chinchipe, Ecuador; Martinique, Trinidad; Markets of Bogotá, Columbia; Carib people, Livingston, Guatamala; Cataractes and Lukaya districts, D.R. Congo; Maori people, Cook Islands	cholesterol-reducing; anti-hypertensive; anti-microbial [wart]; anti-inflammatory [pain]	Tene et al., 2007; Longuefosse and Nossin, 1996; Clement et al., 2015; Girón et al., 1991; Latham and ku Mbuta, 2016; Bussmann et al., 2018; Whistler, 1985
Artocarpus heterophyllus Lam.	Moraceae	Fr, Se	Rakhain people, Bangladesh	Anti-inflammatory [Fe]	Hanif et al., 2009
Artocarpus integer (Thunb.) Merr.	Moraceae	Le	Seberida, Riau Province, Sumatra, Indonesia	Anti-inflammatory	Mahyar et al., 1991
Ascarina philippinensis C.B.Rob.	Chloranthaceae	Fr, Le	Eastern Highlands, Papua New Guinea	Anti-inflammatory [Fe]	Jorim et al., 2012
Asclepias curassavica L.	Apocynaceae	La, Le, Ro	Markets of Bogotá, Columbia; 3 Kerala tribes, Western Ghats, India; Maranao people, Philippines	Anti-microbial [skin inf, STI, dys, wart]; anti-inflammatory [Fe]	Bussmann et al., 2018; Marjana et al., 2018; Malawani et al., 2017

Asparagus africanus Lam.	Asparagaceae	Ro	Jazan province, Saudi Arabia; Oromo people, Harla, Eastern Ethiopia	Anti-inflammatory; anti-paralytic	Tounekti et al., 2019; Belayneh and Bussa, 2014
Asparagus cochinchinensis (Lour.) Merr.	Asparagaceae	Tu	Maonan people, Guangxi Zhuang, China	Anti-microbial [TB]	Hong et al., 2015
Asparagus filicinus Buch.-Ham. ex D.Don	Asparagaceae	Ro	Kashmir, Pakistan	Nervous stimulant	Amjad et al., 2017
Asparagus racemosus Willd.	Asparagaceae	Ro [in sp. comb]	Karnataka, India; Chittagong Hill Tracts, Bangladesh; Uttarakhand, India; Eastern Ghats, India	Anti-paralytic; anti-venom; anti-epileptic; weakness	Rajakumar and Shivanna, 2009; Kadir et al., 2015; Sharma et al., 2013; Rao et al., 2006
Asperula setosa Jaub. & Spach	Rubiaceae	AP	Iran	Anti-viral [cold]	Mosaddegh et al., 2013
Asphodelus aestivus Brot.	Xanthorrhoeaceae	Bu, FL, Le, Ro, Se	Middle East	Anti-paralytic	Boulos, 1983: cited in Abu-Rabia, 2012
Asphodelus tenuifolius Cav.	Xanthorrhoeaceae	AP	Tassili N'Ajjer, Southern Algerian Sahara	Anti-microbial [measles]; anti-inflammatory [Fe]	Hammiche and Maiza, 2006
Aspidosperma excelsum Benth.	Apocynaceae	St ba	Rio Jauaperi, Brazilian Amazon	Anti-inflammatory; anti-microbial [dys]	Pedrollo et al., 2016
Aspidosperma quebracho-blanco Schltdl.	Apocynaceae	Ba	Comechingones people, Argentina	Anti-inflammatory [Fe]	Goleniowski et al., 2006
Aspidosperma spruceanum Benth. ex Müll.Arg.	Apocynaceae	Ba	Mato Grosso, Brazil	Anti-microbial	Ribeiro et al., 2017
Aspidosperma subincanum Mart.	Apocynaceae	Ba	Mato Grosso, Brazil	Anti-hypertensive; cholesterol-reducing	Ribeiro et al., 2017
Asplenium nidus L.	Aspleniaceae	Le	Dayaknese Communities, W Kalimantan, Indonesia	Anti-inflammatory	Diba et al., 2013
Aster diplostephioides (DC.) C.B.Clarke	Asteraceae	FL	Central Himalayas, Nepal	Anti-microbial [cough, cold, ton'is]; anti-inflammatory [pain]; anti-venom	Bhattarai et al., 2006
Asteriscus graveolens (Forssk.) Less	Compositae	AP	Tassili N'Ajjer, Southern Algerian Sahara	Anti-inflammatory [rheum]; fatigue; anti-microbial [wound inf]	Hammiche and Maiza, 2006
Asteromyrtus symphyocarpa (F.Muell.) Craven	Myrtaceae	Le, Ro	Native Northern territory, Australia	Anti-microbial [cold, influenza]	Smith, 1991
Astragalus complanatus R. Br.	Leguminosae	Se	Shaanxi, China	Anti-inflammatory; support immune system	Teng et al., 2011
Astragalus fasciculifolius Boiss.	Leguminosae	FL, Ro	Iran	Anti-viral; wound healing	Mosaddegh et al., 2013
Astronium urundeuva Engl. Syn: *Myracrodruon urundeuva* Allemão	Anacardiaceae	Ba, Fr, Le, Ro	Bolivian Amazon; Mato Grosso, Brazil	Wound healing; Anti-microbial [bronch]; anti-inflammatory;	Bourdy et al., 2000; Ribeiro et al., 2017
Asystasia gangetica (L.) T. Anders.	Acanthaceae	Le	Congo-Brazaville	Memory improvement/ enhancement	Bouquet, 1969: cited in Noe and Lehmann, 2012 [H168]
Asystasia nemorum Nees	Acanthaceae	Le	Seberida, Riau Province, Sumatra, Indonesia	Anti-inflammatory	Mahyar et al., 1991

Ataenidia conferta (Benth.) A.C.Ley	Marantaceae	Le	Baka Pygmies, Gabon	Anti-inflammtory [Fe]; anti-microbial	Betti et al., 2013
Atalantia monophylla DC.	Rutaceae	Fr, St ba	Kani people, Western Ghats, India	Anti-viral [cold]; anti-venom	Ayyanar and Ignacimuthu, 2011
Atractylis aristata Batt.	Compositae	AP	Tassili N'Ajjer, Southern Algerian Sahara	Anti-inflammtory [Fe]; anti-microbial [sore]	Hammiche and Maiza, 2006
Attalea phalerata Mart. ex Spreng	Arecaceae	Ro, Se	Bolivian Amazon	Wound; anti-microbial [boil]	Bourdy et al., 2000
Austroeupatorium inulaefolium (Kunth) R.M.King & H.Rob. Syn: *Eupatorium inulaefolium* L.	Asteraceae	AP	Brazil; Markets of Bogotá, Columbia	Anti-hypertensive	Bussmann et al., 2018
Averrhoa bilimbi L.	Oxalidaceae	Fr, Le	Martinique; Dayaknese Communities, W Kalimantan, Indonesia	Anti-hypertensive	Longuefosse and Nossin, 1996; Diba et al., 2013
Averrhoa carambola L.	Oxalidaceae	Le	Bac Huong Hoa nature reserve, Vietnam	Anti-inflammatory [Fe]	Lee et al., 2019
Ayapana triplinervis (Vahl) R.M. King & H.Rob. Syn: *Eupatorium triplinerve* Blume	Asteraceae	Le	Trinidad; Martinique	Anti-inflammatory [Fe]; anti-microbial [influenza]; anti-hypertensive	Clement et al., 2015; Longuefosse and Nossin, 1996
Azadirachta indica A. Juss.	Meliaceae	Le, Ro, Se, St ba	Andoman + Nicobar Is., India; Kimboza forest, Tanzania; Cataractes and Lukaya districts, D.R. Congo; Conis Santana National Park, East Timor; Kani people, Western Ghats, India	Anti-inflammatory [Fe, headache, rheum]; anti-microbial [boil]; anti – venom; anti-hypertensive; anti-paralytic	Amri and Kisangau, 2012; Chander et al., 2014; Latham and ku Mbuta, 2016; Collins et al., 2007; Ayyanar and Ignacimuthu, 2011
Azara microphylla Hook.f.	Salicaceae	NS	Mapuche, Argentina	Anti-inflammatory	Estomba et al., 2005
Azolla imbricata (Roxb. ex Griff.) Nakai	Salviniaceae	WP	Maonan people, Guangxi Zhuang, China	Anti-microbial	Hong et al., 2015
Azorella diapensioides A.Gray	Apiaceae	AP	Potosi, Bolivia	Anti-microbial [STI pneum]; anti-inflammatory headache	Fernandez et al., 2003
Baccaurea lanceolata (Miq.) Müll.Arg.	Phyllanthaceae	St ba	Dayaknese Communities, W Kalimantan, Indonesia	Anti-inflammatory [Fe]	Diba et al., 2013
Baccharis genistelloides Lam. Pers.	Asteraceae	Le	Loja and Zamora-Chinchipe, Ecuador; Mestizo community, North Peru	Anti-inflammatory; anti-microbial [influenza; HIV]	Bussmann and Sharon, 2006; Tene et al., 2007
Baccharis latifolia (Ruiz & Pav.) Pers.	Asteraceae	Le, St	Loja and Zamora-Chinchipe, Ecuador	Anti-inflammatory; anti-microbial [gangr]	Tene et al., 2007
Baccharis nitida (Ruiz & Pav.) Pers Syn: *Baccharis obtusifolia*	Asteraceae	WP	Loja and Zamora-Chinchipe, Ecuador	Anti-microbial [anti-fungal, cold]	Tene et al., 2007
Baccharis pingraea DC. Syn: *Baccharis salicifolia* (Ruiz. & Pav.) Pers.	Asteraceae	Le, St	Comechingones people, Argentina	Anti-inflammatory; anti-microbial [STI]	Goleniowski et al., 2006
Baccharis punctulata DC.	Asteraceae/Compositae	AP	Potosi, Bolivia	Anti-inflammatory [asthma]	Fernandez et al., 2003

Baccharis trimera (Less.) DC.	Asteraceae	AP	Caiçaras people, Atlantic Forest, Brazil	Anti-hypertensive	Begossi et al., 2002
Baccharoides adoensis (Sch.Bip. ex Walp.) H.Rob. Syn: *Vernonia grantii* Oliv.	Asteraceae	Le	Mabira Forest, Uganda	Anti-microbial [influenza, skin inf]	Tugume et al., 2016
Baccharoides filigera (Oliv. & Hiern) "Isawumi, El-Ghazaly & B.Nord." Syn: *Vernonia hymenolepis* Vatke	Compositae	Le, Ro	Kimboza forest, Tanzania	Anti-inflammatory	Amri and Kisangau, 2012
Bacopa floribunda (R.Br.) Wettst.	Plantaginaceae	AP	Ondo State, SW Nigeria	Memory improvement	Mojisola et al., 2012
Bacopa monnieri (L.) Wettst	Plantaginaceae	WP	Terai forest, West Nepal; West Bengal, India	Memory improvement	Singh et al., 2012; Tariq and Ifham, 2013
Badilloa steetzii (B.L.Rob.) R.M.King & H.Rob. Syn: *Eupatorium subhastatum* Hook. & Arn.	Compositae	Le, St	Comechingones people, Argentina	Anti-inflammatory	Goleniowski et al., 2006
Balanites aegyptiaca (L.) Delile	Zygophyllaceae	Ba, Le	Afar people, Rift Valley, Ethiopia; Mabira Forest, Uganda; Tassili N'Ajjer, Southern Algerian Sahara	Anti-microbial [mumps, dys, *H. zoster*, HSV, lung inf]; anti-venom	Teklehaymanot, 2017; Tugume et al., 2016; Hammiche and Maiza, 2006
Bambusa bambos (L.) Voss Syn: *Bambusa arundinacea* Willd.	Poaceae	Se	Kani people, Western Ghats, India	Anti-inflammatory [rheum]	Ayyanar and Ignacimuthu, 2011
Bambusa multiplex (Lour.) Raeusch. ex Schult.f.	Poaceae	Le, Ro	Rakhain people, Bangladesh	Anti-inflammatory [Fe]	Hanif et al., 2009
Bambusa sp.	Poaceae	Le, Ro	Ayta communities, Bataan, Philippines	Anti-microbial [dengue, cold]	Tantengco et al., 2018
Bambusa vulgaris Schrad. ex J.C. Wendl	Poaceae	Le [in sp. comb]	Sagamu, SW Nigeria; Martinique	Memory enhancement; anti-inflammatory [Fe]	Elufioye et al., 2012; Babawale et al., 2016; Longuefosse and Nossin, 1996
Baphia nitida G.Lodd.	Fabaceae	Le, St ba	3 states in SW Nigeria; Ehotile people, Côte d'Ivoire	Memory enhancement; anti-microbial [boil]	Babawale et al., 2016; Malan et al., 2015
Barleria homoiotrichia C. B. Clarke	Acanthaceae	Ba, FL	Afar people, Rift Valley, Ethiopia	Anti-virall [HSV, mumps]	Teklehaymanot, 2017
Barleria prionitis L.	Acanthaceae	Le	Orang Asli tribe, Malaysia	Anti-inflammatory; eye inflammation	Samuel et al., 2010; Urso et al., 2016: cited in Noe and Lehmann, 2012 [H001]
Barnadesia arborea Kunth	Asteraceae	FL	Loja and Zamora-Chinchipe, Ecuador	Anti-microbial [influenza]; anti-paralytic	Tene et al., 2007
Barteria nigritiana Hook.f.	Passifloraceae	St ba	Baka Pygmies, Gabon	Anti-hypertensive	Betti et al., 2013
Bassia muricata (L.) Asch.	Amaranthaceae	Le	Tassili N'Ajjer, Southern Algerian Sahara	Anti-microbial [boil, wound inf]	Hammiche and Maiza, 2006
Bauhinia sp.	Fabaceae	Le	Dayaknese Communities, W Kalimantan, Indonesia	Anti-inflammatory [Fe]	Diba et al., 2013
Bauhinia variegata L.	Fabaceae	Ba, FL	Mizoram, India	Anxiolytic	Rai and Lalramnghinglova, 2010

Begonia humilis Aiton	Begoniaceae	Le	Trinidad	Anti-microbial [cold]	Clement et al., 2015
Bejaria aestuans Mutis	Ericaceae	FL, Le, St	Mestizo community, North Peru	Anti-inflammatory	Bussmann and Sharon, 2006
Benincasa hispida (Thunb.) Cogn.	Cucurbitaceae	Fr, Le	Magar and Majhi people, Nepal	Anti-microbial [TB]; nervous dis	Malla et al., 2015
Berberis actinacantha Mart. ex Schult. & Schult.f.	Berberidaceae	Le	Mapuche people, Chile	Anti-inflammatory [Fe]	Houghton and Manby, 1985
Berberis angulosa Wall. ex Hook.f. & Thomson	Berberidaceae	Ro	Central Himalayas, Nepal	Anti-microbial [dys, cold]; anti-inflammatory [Fe]	Bhattarai et al., 2006
Berberis darwinii Hook (ind)	Berberidaceae	Le	Mapuche people, Chile	Anti-inflammatory [Fe]	Houghton and Manby, 1985
Berberis goudotii Triana & Planch. ex Wedd.	Berberidaceae	NS	Markets of Bogotá, Columbia	Anti-inflammatory {Fe]	Bussmann et al., 2018
Berberis integerrima Bunge	Berberidaceae	FL, Ro, St	Iran	Anti-hyperlipidemic	Mosaddegh et al., 2013
Berberis lycium Royle	Berberidaceae	Ro, St	Himachal Pradesh, NW Himalaya, India	Anti-microbial [leprosy, cough, cold]	Vidyarthi et al., 2013
Berberis ruscifolia Lam.	Berberidaceae	Le, St	Comechingones people, Argentina	Anti-inflammatory	Goleniowski et al., 2006
Bergenia ciliata Sternb.	Saxifragaceae	Ro	Himachal Pradesh, NW Himalaya, India	Anti-inflammatory [Fe]; anti-paralytic	Vidyarthi et al., 2013; Phondani et a., 2010
Bertholletia excelsa Bonpl.	Lecythidaceae	St ba	Rio Jauaperi, Brazilian Amazon;	Anti-microbial [Dys, STI, UTI]	Pedrollo et al., 2016
Beta vulgaris L.	Amaranthaceae	Le, Ro	Cuba; Mato Grosso, Brazil; Agro Nocerino Sarnese, Southern Italy	Anti-microbial [abscess]	Ribeiro et al., 2017; Motti and Motti, 2017
Betula pendula Roth	Betulaceae	Le	Istro-Romanians of Croatia	Anti-inflammatory [rheum]	Pieroni et al., 2003
Bidens biternata (Lour.) Merr. & Sherff	Asteraceae/Compositae	Le	Haya people, Kagera, NW Tanzania	Anti-microbial [yell fev]	Moshi et al., 2009
Bidens pilosa L.	Asteraceae/Compositae	AP, FL, Le, WP	Mato Grosso, Brazil; Loja and Zamora-Chinchipe, Ecuador; Maromizaha forest, Madagascar; Martinique; Trinidad; Mizoram, India; Tonga; Maori people, Cook Islands	Anti-microbial [boil]; anti-hypertensive; anti-inflammatory [rheum]; wound healing	Ribeiro et al., 2017; Tene et al., 2007; Riondato et al., 2019; Longuefosse and Nossin, 1996; Clement et al., 2015; Rai and Lalramnghinglova, 2010; Croft and Tu'ipulotu, 1980; Whistler, 1985
Bidens schimperi Sch.Bip. ex Walp.	Asteraceae/Compositae	Le	Haya people, Kagera, NW Tanzania	Anti-microbial [yell fev]	Moshi et al., 2009
Bignonia aequinoctialis L. Syn*:* *Cydista aequinoctialis* (L.) Miers	Bignoniaceae	FL, Le, St	Mestizo community, North Peru	Anti-inflammatory	Bussmann and Sharon, 2006
Bignonia nocturna (Barb.Rodr.) L.G.Lohmann Syn: *Tanaecium nocturnum* (Barb.Rodr.) Bureau & K.Schum.	Bignoniaceae	Le	Tacana people, Bolivian Amazon	Anti-microbial [dys]; wound	Bourdy et al., 2000
Bischofia javanica Blume	Phyllanthaceae	Ba, Bd, Le	Mizoram, India	Anti-microbial [diphth, ton'is, cholera]	Rai and Lalramnghinglova, 2010

Biscutella didyma L.	Brassicaceae	Fr	Iran	Anti-fungal	Mosaddegh et al., 2013
Bistorta affinis (D.Don) Greene	Polygonaceae	Ro	Central Himalayas, Nepal	Anti-microbial [cough, cold, ton'is]; anti-inflammatory [Fe]	Bhattarai et al., 2006
Bistorta yunnanensis (Wall. ex Hook.f.) Yonek. & H.Ohashi	Polygonaceae	WP	Chin people, Myanmar	Anti-microbial [boil]	Ong et al., 2018
Bixa orellana L.	Bixaceae	Fr, La, Le, Ro, Se	Mato Grosso, Brazil; Trinidad; Mestizo community, North Peru; Chittagong Hill Tracts, Bangladesh; Eastern Ghats, India	Anti-inflammatory [Fe]; anti-microbial; anti-hypertensive; cholesterol-lowering; anti-venom	Bussmann and Sharon, 2006; Ribeiro et al., 2017; Clement et al., 2015; Odonne et al., 2013; Kadir et al., 2015; Rao et al., 2006
Bixa platycarpa Ruiz et Pav. ex G. Don.	Bixaceae	Ba	Peruvian Amazon	Anti-microbial [eye inf]	Odonne et al., 2013
Blepharis ciliaris (L.) B.L.Burtt	Acanthaceae	Le, Ro, Se	Jazan province, Saudi Arabia	Anti-inflammatory [eye], anti-microbial [cough]; wound healing	Tounekti et al., 2019
*Bletilla striata (*Thunb.) Rchb.f.	Orchidaceae	Bu	Maonan people, Guangxi Zhuang, China	Anti-microbial	Hong et al., 2015
Blumea balsamifera DC.	Asteraceae	Le, Ro, WP	Myanmar; Maonan people, Guangxi Zhuang, China; Maranao and Ayta people, Philippines	Limb paralysis, muscle spasms; anti-inflammatory [Fe, rheum]; Anti-microbial [coughs, cold]; anti-hypertensive	Nordal, 1963: cited in DeFilipps and Krupnick, 2018; Hong et al., 2015; Malawani et al., 2017; Tantengco et al., 2018
Blumea sinuata (Lour.) Merrill	Asteraceae	Le	Rakhain people, Bangladesh	weakness	Hanif et al., 2009
Bobgunnia madagascariensis (Desv.) J.H.Kirkbr. & Wiersema Syn: *Swartzia madagascariensis* Desv.	Leguminosae	Ro	Benin	Memory improvement/ enhancement	Adjanohoun et al., 1989 cited in Noe and Lehmann, 2012 [H168]
Boerhavia diffusa L.	Nyctaginaceae	AP, Le, Ro, WP	Myanmar; Bangladesh; Himachal Pradesh, NW Himalaya, India; Atlantic Forest, Brazil; Guinea-Bissau	Anti-paralytic, stimulates new tissue in wound healing; anti-venom; anti-microbial [leprosy]; anti-inflammatory; neurological disorders; anti-fatigue	Nordal, 1963: cited in DeFilipps and Krupnick, 2018; Ribeiro et al., 2017; Vidyarthi et al., 2013; Gazzaneo et al., 2005; Romeiras et al., 2012; Malzy et al., 1954: cited in Noe and Lehmann, 2012 [H091]
Boerhavia erecta L	Nyctaginaceae	Re	Eastern Ghats, India	Anti-inflammatory [Fe]	Rao et al., 2006
Boesenbergia rotunda (L.) Mansf.	Zingiberaceae	Le	Andoman + Nicobar Is., India	Anti-paralytic, anti-inflammatory [Fe, headache]	Chander et al., 2014
Bombax buonopozense P. Beauv.	Malvaceae	Le	South Nigeria	Anti-inflammatory [Fe]; anti-microbial [skin inf]	Borokini et al., 2012
Bombax ceiba Linn.	Malvaceae	Ba [in sp. comb]	India; Chittagong Hill Tracts, Bangladesh	Anti-paralytic; anti-venom	Mikawlrawng et al., 2017; Kadir et al., 2015
Bonafousia sp.	Apocynaceae	La	Mato Grosso, Brazil	Anti-inflammatory [pain]	Ribeiro et al., 2017
Bontia daphnoides L.	Scrophulariaceae	Le	Martinique; Trinidad	Anti-hypertensive; anti-microbial [cold]	Longuefosse and Nossin, 1996; Clement et al., 2015

Borago officinalis L.	Boraginaceae	FL., Le	Loja and Zamora-Chinchipe, Ecuador; Northeastern Dahra Mountains, NW Algeria; Agro Nocerino Sarnese, Southern Italy; Taounate, North Morocco	Anti-microbial [influenza, conj]; anti-hypertensive; hypercholesterolaemia; anxiolytic; anti-inflammatory; memory improvement	Tene et al., 2007; El-Hilaly et al., 2003; Senouci et al., 2019; Motti and Motti, 2017
Boscia arabica Pestal.	Capparaceae	Le	Abyan territory, Yemen	Anti-microbial [dental inf]	Al-Fatimi, 2019
Boscia coriacea Graells	Capparaceae	Le	Afar people, Rift Valley, Ethiopia	Anti-microbial [leprosy, lung inf]	Teklehaymanot, 2017
Boscia gossweileri Exell	Capparaceae	Le	Angola	Eye inflammation	Urso et al., 2016
Boscia senegalensis Lam. ex Poir Syn: *Boscia octandra* Hochst. ex Radlk.	Capparaceae	Le, Ro	Mana Angetu District, SE Ethiopia; Tassili N'Ajjer, Southern Algerian Sahara	Anti-inflammatory [headache, rheum, Fe]; anti-microbial [wound inf]	Lulekal et al., 2008; Hammiche and Maiza, 2006
Boswellia ovalifoliolata N.P.Balakr. & A.N.Henry	Burseraceae	Se	Eastern Ghats, India	Anti-bacterial [diarrh]	Rao et al., 2006
Boswellia sacra Flück.	Burseraceae	Re	N + S Oman; Mashhad markets, Iran	Anti-bacterial bronch]; anti-inflammatory [asthma]; wound healing; memory improvement	Divakar et al., 2016; Amiri and Joharchi, 2013
Boswellia serrata Roxb. ex Colebr.	Burseraceae	Le	Eastern Ghats, India	Anti-inflammatory [rheum]	Rao et al., 2006
Bouchea prismatica (L.) Kuntze	Verbenaceae	NS	Markets of Bogotá, Columbia	Anti-microbial [typhus]	Bussmann et al., 2018
Bowdichia virgilioides Kunth	Fabaceae	Ba, Ro	Mato Grosso, Brazil; Atlantic Forest, Brazil	Anti-microbial; anti-inflammatory	Ribeiro et al., 2017; Gazzaneo et al., 2005
Brassica nigra (L.) W.D.J. Koch	Brassicaceae	NS	Markets of Bogotá, Columbia	Anti-microbial [pneum]	Bussmann et al., 2018
Brassica oleracea L.	Brassicaceae	Le	Yörük people, SE Macedonia	Anti-inflammatory [rheum, toothache]	Nedelcheva et al., 2017
Brassica rapa L.	Brassicaceae	Ro	Mestizo community, North Peru; Markets of Bogotá, Columbia	Anti-inflammatory; anti-microbial; immunostimulant	Bussmann and Sharon, 2006; Bussmann et al., 2018; Mustafa et al., 2015
Breynia vitis-idaea (Burm.f.) C.E.C.Fisch.	Phyllanthaceae	Le	Andoman + Nicobar Is., India	Anti-paralytic, anti-inflammatory [Fe]; anti-hypertensive	Chander et al., 2014
Bridelia atroviridis Müll.Arg.	Phyllanthaceae	Ba	Cataractes and Lukaya districts, D.R. Congo	Anti-microbial [STI, cough, caries]	Latham and Konda ku Mbuta, 2016
Bridelia ferruginea Benth.	Phyllanthaceae	Le, St	Benin; D.R. Congo	Hemiplegia/ paraplegia/ polio; memory improvement/ enhancement; anti-microbial [dys]	Adjanohoun et al., 1989: cited in Noe and Lehmann, 2012 [H130, H168]; Latham and Konda ku Mbuta, 2016
Bridelia micrantha (Hochst.) Baill.	Phyllanthaceae	Le, Ro	Kakamega County, Kenya	Anti-microbial [dys]	Odongo et al., 2018
Brillantaisia owariensis P.Beauv Syn: *Brillantaisia patula* T. Anderson	Acanthaceae	Le, St	Cataractes and Lukaya districts, D.R. Congo	Dementia; anti-PD	Latham and Konda ku Mbuta, 2016; Kembelo, 2003: cited in Noe and Lehmann, 2012 [H130x]
Brocchia cinerea (Delile) Vis.	Asteraceae/ Compositae	AP	Tassili N'Ajjer, Southern Algerian Sahara	Anti-inflammatory [rheum]; anti-venom	Hammiche and Maiza, 2006
Brosimum gaudichaudii Trécul	Moraceae	Ro, Ba	Mato Grosso, Brazil	Anti-microbial; anti-inflammatory	Ribeiro et al., 2017
Brosimum parinarioides Ducke	Moraceae	St ba	Rio Jauaperi, Brazilian Amazon	Anti-microbial	Pedrollo et al., 2016

Brucea javanica (L.) Merr.	Simaroubaceae	Fr, Se	Cataractes and Lukaya districts, D.R. Congo	Anti-bacterial [boil]; anti-inflammatory [Fe]	Latham and Konda ku Mbuta, 2016
Brugmansia x candida Pers. Syn: *Datura arborea* Ruíz & Pav.	Solanaceae	Le	Madagascar	Anti-microbial/anti-paralytic [polio]	Randriamiharisoa et al., 2015: cited in Noe and Lehmann, 2012 [H130]
Bryonia cretica subsp. dioica (Jacq.) Tutin	Cucurbitaceae	Ro	Taounate, North Morocco	Anti-inflammatory [rheum]	El-Hilaly et al., 2003
Buchenavia tomentosa Eichler	Combretaceae	Ba, FL, Fr	Mato Grosso, Brazil	Anti-microbial; anti-hypertensive; cholesterol-reducing	Ribeiro et al., 2017
Buddleja asiatica Lour.	Scrophulariaceae	Le, St	Bac Huong Hoa nature reserve, Vietnam	Anti-inflammatory [headache]	Lee et al., 2019
Buddleja coriacea J.Rémy Syn: *Buddleja utilis* Kraenzl.	Scrophulariaceae	FL	Mestizo community, North Peru	Anti-inflammatory	Bussmann and Sharon, 2006
Buddleja mendozensis Gillet ex Benth.	Scrophulariaceae	Le	Comechingones people, Argentina	Anti-paralytic	Goleniowski et al., 2006
Bulbophyllum mutabile (Blume) Lindl.	Orchidaceae	Le	Orang Asli tribe, Malaysia	Anti-inflammatory	Samuel et al., 2010
Bunium persicum (Boiss.) B. Fedtsch.	Apiaceae	Se	Turkestan Range of south Kyrgystan; Kashmir Himalaya, India	Anti-inflammatory [headache]; anti-microbial [influenza, cold]; memory improvement	Pawera et al., 2016; Lone et al., 2014
Bupleurum abchasicum Manden. Syn: *Bupleurum chinense D.C.*	Apiaceae	Ro	Tian Mu Shan, Zhejiang, China	Anti-microbial	Chaudhary et al., 2006
Bupleurum longicaule Wall. ex DC.	Apiaceae	FL, Se	Central Himalayas, Nepal	Anti-microbial	Bhattarai et al., 2006
Bursera graveolens (Kunth) Triana & Planch.	Burseraceae	NS	Markets of Bogotá, Columbia	Anti-microbial [influenza]; anti-stress	Bussmann et al., 2018
Bursera simaruba (L.) Sarg.	Burseraceae	NS	Florida, Mexico, Venezuela; Markets of Bogotá, Columbia	Anti-microbial [gangr]	Bussmann et al., 2018
Butea monosperma (Lam.) Kuntze	Fabaceae	Ba, Le, Se	3 Kerala tribes, Western Ghats, Karnataka, Maharashtra, India	snake venom antidote; promoting strength and longevity; anti-microbial [sore throat, UTI]; wound healing	Ross, 1999: cited in DeFilipps and Krupnick, 2018; Rajakumar and Shivanna, 2009; Marjana et al., 2018; Wadankar et al., 2011
Byttneria pescapriifolia Britton	Malvaceae	Se	Mato Grosso, Brazil	Anti-microbial; anti-inflammatory	Ribeiro et al., 2017
Cadaba farinosa Forssk.	Capparidaceae	Le, Ro	Afar people, Rift Valley, Ethiopia	Anti-bacterial [lung inf, typhoid, anthrax]; anti-inflammatory [pain]	Teklehaymanot, 2017
Cadaba glandulosa Forssk.	Capparidaceae	Le	Afar people, Rift Valley, Ethiopia	Anti-microbial [meningitis, typhoid]	Teklehaymanot, 2017
Cadaba rotundifolia Forssk.	Capparidaceae	Le	Afar people, Rift Valley, Ethiopia; Abyan territory, Yemen	Anti-microbial [cough]; anti-inflammatory [nerve pain, rheum]	Teklehaymanot, 2017; Al-Fatimi, 2019
Caesalpinia crista L. Syn: *Caesalpinia nuga* (L.) W.T. Aiton	Leguminosae	Le, Ro	Rakhain people, Bangladesh	Anti-microbial [UTI]	Hanif et al., 2009
Caesalpinia spinosa (Molina) Kuntze	Leguminosae	Se	Mestizo community, North Peru	Anti-inflammatory; anti-microbial	Bussmann and Sharon, 2006
Caesalpinia violacea Standl.	Leguminosae	Ba, Le	Carib people, Livingston, Guatamala	Anti-inflammatory [Fe]	Girón et al., 1991

Cajanus cajan (L.) Millsp. Syn: *Cajanus indicus* Spreng. L	Leguminosae	Le	Ayta communities, Philippines; Mato Grosso, Brazil; Andoman + Nicobar Is., India; Chittagong Hill Tracts, Bangladesh; Trinidad; Cataractes and Lukaya districts, D.R. Congo; Guinea-Bissau	Anti-microbial [measles, dys, polio], thermal shock; anti-inflammatory [Fe]; anti-hypertensive; anti-venom; anti-epileptic	Tantengco et al., 2018; Ribeiro et al., 2017, Chander et al., 2014; Clement et al., 2015; Kadir et al., 2015; Kembelo, 2003: cited in Noe and Lehmann, 2012; Latham and Konda ku Mbuta, 2016; Romeiras et al., 2012
Caladium bicolor (Aiton) Vent.	Araceae	Rh	Cataractes and Lukaya districts, D.R. Congo	Anti-microbial [boil, abscess]	Latham and Konda ku Mbuta, 2016
Calceolaria rugulosa Edwin	Calceolariaceae	WP	Mestizo community, North Peru	Anti-inflammatory	Bussmann and Sharon, 2006
Calendula arvensis L.	Asteraceae/ Compositae	Le	Agro Nocerino Sarnese, Southern Italy	Wound healing [burn]	Motti and Motti, 2017
Calendula officinalis L.	Asteraceae/ Compositae	FL, Le	Markets of Bogotá, Columbia; Svaneti and Racha-Lechkumi, Georgia	Anti-inflammatory; anti-microbial; wound healing	Bussmann et al., 2018; Cadena-González et al., 2013; Bussmann et al., 2016
Calliandra parviflora Benth.	Leguminosae	Fr, Le	Mato Grosso, Brazil	Anti-microbial	Ribeiro et al., 2017
Callicarpa arborea Roxb.	Lamiaceae	Ba	Mizoram, India	Anti-microbial [dys]	Rai and Lalramnghinglova, 2010
Callicarpa longifolia Lam.	Lamiaceae	Le	Andoman + Nicobar Is., India	Anti-inflammatory [Fe]	Chander et al., 2014
Callicarpa tormentosa (L.) L.	Lamiaceae	Ba	3 Kerala tribes, Western Ghats, India	Anti-inflammatory [Fe]	Marjana et al., 2018
Callisia gracilis (Kunth) D.R.Hunt	Commelinaceae	FL, Le	Loja and Zamora-Chinchipe, Ecuador	Anti-hypertensive	Tene et al., 2007
Callisia monandra (Sw.) Schult. & Schult f.	Commelinaceae	Le, St	Columbian Andes	Anti-inflammatory [rheum]	Cadena-González et al., 2013
Callisia repens (Jacq.) L.	Commelinaceae	Le	Loja and Zamora-Chinchipe, Ecuador; Markets of Bogotá, Columbia	Anti-microbial [int inf, gangr]; anti-hypertensive; anti-inflammatory [Fe]	Tene et al., 2007; Bussmann et al., 2018
Calophyllum brasiliense Cambess.	Clusiaceae	Fr, La	Mato Grosso, Brazil	Anti-microbial [general inf]	Ribeiro et al., 2017
Calotropis procera (Aiton) Dryand.	Apocynaceae	Ba, FL, Le, sap	Myanmar; Ethiopia; Maharashtra, India; Abyan territory, Yemen; Tassili N'Ajjer, Southern Algerian Sahara; Golan Heights and West Bank, Israel; Togo	Anti-bacterial [boils]; antifungal, anti-viral [*H. zoster*]; anti-paralytic; anti-venom; wound healing; anti-inflammatory [asthma, Fe]; epilepsy; anti-stroke, anti-PD	DeFilipps and Krupnick, 2018; Teklehaymanot, 2017; Wadankar et al., 2011; Al-Fatimi, 2019; Hammiche and Maiza, 2006; Said et al., 2002; Kantati et al., 2016
Calystegia sepium (L.) R.Br.	Convolvulaceae	AP	Pollino National Park, Southern Italy	Anti-inflammatory [rheum]	Di Sanzo et al., 2013,
Calytrix brownii (Schauer) Craven	Myrtaceae	Le	Native Northern territory, Australia	Anti-microbial [cold, influenza)	Smith, 1991
Camellia sinensis (L.) Kuntze	Theaceae	FL, Le, Ro, St	Hakka people, Guangdong, China; Mashhad markets, Iran	Anti-hypertensive; anti-hyperlipidemia	Au et al., 2008; Amiri and Joharchi, 2013
Campomanesia speciosa (Diles) Mc Vaugh	Myrtaceae	Le	Peruvian Amazon	Anti-inflammatory [Fe]	Odonne et al., 2013
Campsiandra comosa Benth.	Fabaceae	St, Ba	Rio Jauaperi, Brazilian Amazon	Anti-microbial [dys]	Pedrollo et al., 2016

Camptostemon schultzii Mast.	Malvaceae	Ash	Native Northern territory, Australia	Anti-microbial [leprosy, skin inf]	Smith, 1991
Campyloneurum sp.	Polypodiaceae	WP	Loja and Zamora-Chinchipe, Ecuador	Anti-microbial [pneum]; anti-inflammatory	Tene et al., 2007
Campyloneurum fuscosquamatum Lellinger	Polypodiaceae	Le	Bolivian Amazon	Wound	Bourdy et al., 2000
Canarium schweinfurthii Engl.	Burseraceae	Ba	Mabira Forest, Uganda; Haya people, Kagera, NW Tanzania	Anti-hypertensive	Tugume et al., 2016; Moshi et al., 2009
Canna indica L. Syn: *C. bidentata*	Cannaceae	Le, Ro	Nuevo León, Mexico; Sagamu, Nigeria; Cataractes and Lukaya districts, D.R. Congo	Anti-inflammatory; anti-microbial [cough]; anti-aging	Estrada-Castillón et al., 2012; Latham and Konda ku Mbuta, 2016; Elufioye et al., 2012
Capparis cartilaginea Decne.	Capparaceae	Le, Ro	Abyan territory, Yemen	Anti-microbial [UTI]; anti-inflammatory [asthma, headache, toothache]	Al-Fatimi, 2019
Capparis erythrocarpus Isert Syn: *Capparis acuminata* De Wild.	Capparaceae	NS	Uganda	Memory improvement/ enhancement	Adjanohoun et al., 1993; cited in Noe and Lehmann, 2012 [H168]
Capparis grandis L.f.	Capparaceae	Ba, St	Telangana, India	Anti-paralytic	Nishteswar, 2015
Capparis spinosa L	Capparaceae	FL, Le, Ro, Sh	Western Ladakh, India; Tassili N'Ajjer, Southern Algerian Sahara; Taounate, North Morocco	Anti-paralytic; anti-inflammatory [rheum, headache]	Angmo et al., 2012; Hammiche and Maiza, 2006; El-Hilaly et al., 2003
Capparis zeylanica L	Capparaceae	Le, Ro	Eastern Ghats, India	Anti-inflammatory [Fe]	Rao et al., 2006
Capraria biflora L.	Scrophulariaceae	AP, Le	Martinique	Anti-inflammatory; anti-microbial [influenza]; anti-hypertensive	Longuefosse and Nossin, 1996
Capraria viana Benth.	Scrophulariaceae	WP	Mestizo community, North Peru	Anti-inflammatory	Bussmann and Sharon, 2006
Capsella bursa-pastoris (L.) Medic.	Brassicaceae	Le, WP	Mato Grosso, Brazil; Mestizo community, North Peru; Maonan people, Guangxi Zhuang, China	Anti-inflammatory; anti-microbial; anti-hypertensive	Ribeiro et al., 2017; Bussmann and Sharon, 2006; Hong et al., 2015
Capsicum annuum L.	Solanaceae	Fr	Markets of Bogotá, Columbia; Bac Huong Hoa nature reserve, Vietnam; Agro Nocerino Sarnese, Southern Italy	Anti-inflammatory [Fe, rheum, headache]; anti-microbial [influenza, laryngitis, ton'is, dys, boil]; anti-depressant; wound healing	Bussmann et al., 2018; Lee et al., 2019; Motti and Motti, 2017
Capsicum frutescens L.	Solanaceae	Fr [in sp. comb]	Mato Grosso, Brazil; Baka Pygmies, Gabon; Maori people, Cook Islands	Anti-microbial [inf, influenza]; anti-inflammatory; wound healing	Ribeiro et al., 2017; Betti et al., 2013; Whistler, 1985
Caragana brevispina Benth.	Fabaceae	St,	Central Himalayas, Nepal	Anti-microbial; vision defects; heart pain	Bhattarai et al., 2006
Caralluma penicillata (Deflers) N.E.Br.	Apocynaceae	St	Abyan territory, Yemen	Anti-inflammatory	Al-Fatimi, 2019
Caralluma speciosa (N.E.Br.) N.E.Br.	Apocynaceae	Sap, St	Oromo people, Harla, Eastern Ethiopia	Anti-poison; wound healing; anti-microbial [gangrene]	Belayneh and Bussa, 2014

Caralluma tuberculata N.E.Br.	Apocynaceae	WP	Sialkot, Pakistan	Ant-paralytic; anti-inflammatory [rheum, Fe]	Mahmood et al., 2011
Carapa guianensis Aubl.	Meliaceae	Se oil, Se	Rio Jauaperi, Brazilian Amazon + Brazil Mato Grosso; Trinidad	Anti-inflammatory; anti-microbial	Pedrollo et al., 2016; Ribeiro et al., 2017; Clement et al., 2015
Carapa procera DC.	Meliaceae	Ba	Cataractes and Lukaya districts, D.R. Congo	Anti-microbial [dys, cough], Anti-inflammatory [Fe]	Latham and Konda ku Mbuta, 2016
Cardiospermum grandiflorum Sw.	Sapindaceae	Le, Ro	Cataractes and Lukaya districts, D.R. Congo	Anti-inflammatory; anti-microbial [dys]	Latham and Konda ku Mbuta, 2016
Cardiospermum halicacabum L.	Sapindaceae	Le, Ro	Martinique; 3 Kerala and Kani people, Western Ghats, India	Anti-inflammatory [rheum]; anti-microbial [boil, abscess, polio]; anti-venom	Longuefosse and Nossin, 1996; Marjana et al., 2018; Ayyanar and Ignacimuthu, 2011
Careya arborea Roxb.	Lecythidaceae	Ba, WP	Karnataka and 3 Kerala tribes, Western Ghats, India	Anti-microbial [dys]; anti-inflammatory [Fe]	Rajakumar and Shivanna, 2009; Marjana et al., 2018
Carica papaya L.	Caricaceae	Fr, Le, Ro, Se	3 states in SW Nigeria; N + S Oman; Mato Grosso; Brazil; Mabira Forest, Uganda; Trinidad; Venda tribes, South Africa; Maranao people, Philippines; Maharashtra, India; Sundanese community, West Java; Maori people, Cook Islands; Fundong, NW Cameroon	Memory enhancement; anti-hypertensive; anti-microbial [dys, boil, sore]; boost immune system; anti-inflammatory [Fe, asthma]; wound healing	Babawale et al., 2016; Mojisola et al., 2012 Divakar et al., 2016, Ribeiro et al., 2017; Tugume et al., 2016; Clement et al., 2015; Arnold and Gulumian, 1984; Malawani et al., 2017; Wadankar et al., 2011; Roosita et al., 2008; Whistler, 1985; Focho et al.,2009
Cariniana estrellensis (Raddi) Kuntze	Lecythidaceae	Ba	Mato Grosso, Brazil	Anti-microbial	Ribeiro et al., 2017
Cariniana sp.	Lecythidaceae	Ba	Mato Grosso, Brazil	Anti-inflammatory	Ribeiro et al., 2017
Carissa carandas L.	Apocynaceae	Fr, Le	Dinajpur, Bangladesh	Anti-inflammatory [Fe, pain, headache], anti-epileptic; nervous disorders, wound healing, anti-microbial [leprosy]	Rahmatullah et al., 2009
Carissa edulis (Forssk.) Vahl	Apocynaceae	Le, Ro	Benin; Venda tribes, South Africa	Anti-microbial [TB]; memory improvement/ enhancement	Adjanohoun et al., 1989: cited in Noe and Lehmann, 2012 [H130, H168]; Arnold and Gulumian, 1984
Carissa spinarum L. Syn: *Carissa pubescens* A. DC.	Apocynaceae	Le, Ro	Kakamega County, Kenya	Anti-microbial	Odongo et al., 2018
Carpolobia alba G.Don.	Polygalaceae	Ro	Sagamu, Nigeria	Anti-aging	Elufioye et al., 2012
Carthamus tinctorius L.	Asteraceae	FL, Se	Myanmar	Anti-venom; energy-boosting; anti-inflammatory [Fe, rheum, inflam] *Toxicity Report	DeFilipps and Krupnick, 2018<br><br>* Louei Monfared and Salati, 2012
Caryocar brasiliense A.St.-Hil.	Caryocaraceae	Ba, FL, Fr, Le, Ro, St	Mato Grosso, Brazil	Anti-microbial	Ribeiro et al., 2017

Cascabela thevetia (L.) Lippold Syn: *Thevetia peruviana* (Pers.) K. Schum.	Apocynaceae	NS	Markets of Bogotá, Columbia	Anti-inflammatory [Fe]	Bussmann et al., 2018
Casearia sp.	Salicaceae	Le	Mato Grosso, Brazil	Anti-inflammatory; anti-hypertensive	Ribeiro et al., 2017
Casearia sylvestris Swartz	Salicaceae	Le	Brazil; Markets of Bogotá, Columbia	Anti-ulcer; anti-microbial [sore]	Bussmann et al., 2018
Cassia fistula L.	Fabaceae	Fr, Le	India; Martinique; 3 Kerala tribes, Western Ghats, India; Uttarakhand, India; Chittagong Hill Tracts, Bangladesh	Anti-paralytic; anti-microbial [ton'is]; anti-venom; anti-epileptic; anti-inflammatory	Mikawlrawng et al., 2017; Longuefosse and Nossin, 1996; Marjana et al., 2018; Kadir et al., 2015; Sharma et al., 2013
Cassia grandis L. f.	Fabaceae	NS	Markets of Bogotá, Columbia	Anti-inflammatory [Fe]; anti-microbial [STI]	Bussmann et al., 2018
Cassine transvaalensis (Burtt Davy) Codd	Celastraceae	Ba	Venda, South Africa	Anti-microbial [STI]	Arnold and Gulumian, 1984
Cassytha filiformis L.	Lauraceae	Se	Eastern Ghats, India	Anti-inflammatory [rheum]	Rao et al., 2006
Castanea sativa Mill.	Fagaceae	Le	Kashmir, Pakistan	Anti-inflammatory [Fe]; anti-microbial [sore throat]	Amjad et al., 2017
Castilleja coccinea (L.) Spreng.	Orobanchaceae	FL	Native America	Anti-paralytic	Densmore, 1928: cited in Native American Ethnobotany Database
Cattleya schroederae (Rchb.f.) Sander	Orchidaceae	Le	Columbian Andes	Anti-microbial [typhus, dengue]; anti-inflammatory [Fe]	Cadena-González et al., 2013
Caulophyllum robustum Maxim. Syn: *Leontice robustum* (Maxim.) Diels	Berberidaceae	Ro	Shaanxi, China;	Anti- hypertensive	Teng et al., 2011
Cayratia japonica (Thunb.) Gagnep.	Vitaceae	Ro, WP	Maonan people, Guangxi Zhuang, China	Anti-paralytic	Hong et al., 2015
Cecropia palmata Willd	Urticaceae	Ro	Atlantic Forest, Brazil	Anti-inflammatory	Gazzaneo et al., 2005
Cecropia pachystachya Trécul	Urticaceae	Ba, Fr, Le, Ro, Sh	Mato Grosso, Brazil; Cataractes and Lukaya districts, D.R. Congo	Anti-microbial [dys, caries, cough]; anti-inflammatory; anti-hypertensive; anti-PD	Ribeiro et al., 2017; Latham and Konda ku Mbuta, 2016
Cecropia peltata L.	Urticaceae	Le	Trinidad	Anti-inflammatory [Fe]	Clement et al., 2015
Cedrus deodara (Royle ex D. Don) G. Don	Pinaceae	Wo	Himachal Pradesh, NW Himalaya, India	Anti-microbial [leprosy]	Vidyarthi et al., 2013
Ceiba pentandra (L.) Gaertn.	Malvaceae	St ba	Martinique; Cataractes and Lukaya districts, D.R. Congo	Anti-microbial [measles, dys], anti-fungal; anti-inflammatory [Inf]	Longuefosse and Nossin, 1996; Latham and Konda ku Mbuta, 2016
Ceiba samauma (Mart.) K.Schum.	Malvaceae	St	Loja and Zamora-Chinchipe, Ecuador	Anti-inflammatory	Tene et al., 2007
Celastrus paniculatus Willd.	Celastraceae	Se	Philippines	Anti-paralytic	Perry and Metzger, 1980: cited in DeFilipps and Krupnick, 2018
Celosia argentea L.	Amaranthaceae	Le, Ro	Bac Huong Hoa nature reserve, Vietnam	Anti-paralytic	Lee et al., 2019

Celosia polystachia (Forssk.) C.C.Towns.	Amaranthaceae	WP	Afar people, Rift Valley, Ethiopia	Anti-microbial [typhoid, mumps, lung inf]	Teklehaymanot, 2017
Celtis australis L.	Cannabaceae	Le	Catalonia, Spain	Anti-hypercholesteremic	Bonet et al., 1999
Celtis philippensis Blanco	Cannabaceae	Ba	Native Northern territory, Australia	Anti-microbial	Smith, 1991
Celtis toka (Forssk.) Hepper & J.R.I.Wood Syn: *Celtis integrifolia* Lam.	Cannabaceae	Ro	Senegal	Limb paralysis	Kerharo and. Adam, 1974: cited in Noe and Lehmann, 2012 [H130]
Cenchrus echinatus L.	Poaceae	WP	Mestizo community, North Peru	Anti-inflammatory	Bussmann and Sharon, 2006
Centaurea benedicta (L.) L. Syn: *Cnicus benedictus* L.	Asteraceae/Compositae	AP	Edremit Gulf, Turkey	Sedative; anti-inflammatory [Fe]	Polat and Satıl, 2012
Centaurium cachanlahuen (Mol.) Rob. (ind) L	Gentianaceae	Le	Mapuche people, Argentina and Chile	Anti-inflammatory [Fe]; anti-microbial [pneum]	Estomba et al., 2005; Houghton and Manby, 1985
Centaurium erythraea Rafn.	Gentianaceae	AP, Le, St	Loja and Zamora-Chinchipe, Ecuador; Albanians, Bosniaks/Gorani + Turkish ethnic groups, Kosovo; Catalonia, Spain	Anti-microbial [influenza, int inf]; immunostimulant	Tene et al., 2007; Mustafa et al., 2015; Bonet et al., 1999
Centella asiatica (L.) Urb.	Apiaceae	Le	Fiji; Sama–Bajau Community, Sabah, Malaysia; Mizoram, India; Karnataka, India; Himachal Pradesh, NW Himalaya, India; Kakamega County, Kenya; Maranao people, Philippines; Bac Huong Hoa nature reserve, Vietnam; Kani people, Western Ghats, India; Eastern Ghats, India; Haya people, Kagera, NW Tanzania; Cataractes and Lukaya districts, D.R.Congo	Memory improvement; weakness; anti-inflammatory [asthma, Fe, headache]; anti-bacterial [leprosy, ear inf, STI, UTI]; anti-venom; anti-hypertensive; wound healing	Singh, 1986; Awang-Kanak et al., 2018; Rai and Lalramnghinglova, 2010; Arkinstall, 1979: cited in Noe and Lehmann, 2012 [H130]; Rajakumar and Shivanna, 2009; Vidyarthi et al., 2013; Odongo et al., 2018; Malawani et al., 2017; Lee et al., 2019; Ayyanar and Ignacimuthu, 2011; Rao et al., 2006; Moshi et al., 2009; Latham and Konda ku Mbuta, 2016
Ceropegia bulbosa Roxb.	Apocynaceae	St	Abyan territory, Yemen	Immunostimulatory	Al-Fatimi, 2019
Ceropegia variegata Decne.	Apocynaceae	St	Abyan territory, Yemen	Immunostimulatory	Al-Fatimi, 2019
Cestrum mariquitense Kunth Syn: *Cestrum sendtnerianum*	Solanaceae	FL, Le	Loja and Zamora-Chinchipe, Ecuador; Native Tropical America	Anti-inflammatory [Fe, headache]; anti-microbial [gangr, influenza, int inf]	Tene et al., 2007; Cadena-González et al., 2013
Cestrum mutisii Willd. ex Roem. & Schult.	Solanaceae	NS	Markets of Bogotá, Columbia	Anti-microbial [typhus]	Bussmann et al., 2018
Cestrum parqui (Lam.) L'Hér. Syn: *Cestrum foetidissimum* (Lam.) L'Hér.	Solanaceae	Le	Mapuche people, Argentina and Chile; Potosi, Bolivia	Anti-inflammatory [Fe]; anti-microbial [s'pox, TB, leprosy, *Herpes*, impetigo]	Estomba et al., 2005; Houghton and Manby, 1985; Fernandez et al., 2003
Cestrum racemosum Ruiz & Pav.	Solanaceae	WP	Loja and Zamora-Chinchipe, Ecuador	Anti-inflammatory [Fe]; anti-microbial [inf, influenza, cold]	Tene et al., 2007

Chamaecrista glandulosa (Michx.) Greene	Fabaceae	FL, Re	Martinique	Anti-inflammatory [Fe]	Longuefosse and Nossin, 1996
Chamaedorea angustisecta Burret	Arecaceae	Le	Bolivian Amazon	Anti-venom	Bourdy et al., 2000
*Chamaemelum nobile (*L.) All. Syn: *Anthemis nobilis* L.	Asteraceae/Compositae	FL	M'Sila, North Algeria	Anti-inflammatory	Boudjelal et al., 2013
Chamaesyce hypericifolia (L.) Millspaugh	Euphorbiaceae	WP	Mestizo community, North Peru	Anti-inflammatory	Bussmann and Sharon, 2006
Chaptalia nutans (L.) Polák	Asteraceae	WP	Trinidad	Anti-hypertensive	Clement et al., 2015
Chaptalia sinuata (Less.) Baker	Asteraceae	Le, Ro, St	Comechingones people, Argentina	Anti-inflammatory [Fe]	Goleniowski et al., 2006
Cheilocostus speciosus (J.Koenig) C.D.Specht Syn: *Costus speciosus* (J.Koenig) Sm.	Costaceae	Le, Ro	Martinique; Chittagong Hill Tracts, Bangladesh; Kani people, Western Ghats, India	Anti-inflammatory [Fe, inflam]; anti-venom; wound healing	Longuefosse and Nossin, 1996; Kadir et al., 2015; Ayyanar and Ignacimuthu, 2011
Chelidonium majus L.	Papaveraceae	La, Ro	Svaneti and Racha-Lechkumi, Georgia; Istro-Romanians, Northern Istria, Croatia; Agro Nocerino Sarnese, Southern Italy	Anti-viral [wart]; wound healing	Bussmann et al., 2016; Pieroni et al., 2003; Motti and Motti, 2017
Chenopodium album L.	Amaranthaceae	Le	Loja and Zamora-Chinchipe, Ecuador; Chittagong Hill Tracts, Bangladesh	Anti-inflammatory; anti-microbial; anti-venom	Tene et al., 2007; Kadir et al., 2015
Chenopodium quinoa Willd.	Amaranthaceae	NS	Markets of Bogotá, Columbia	Anti-inflammatory [Fe]; anti-microbial [TB]	Bussmann et al., 2018
Chiliadenus sericeus (Batt. & Trab.) Brullo Syn: *Varthemia sericea* (Batt. & Trab.) Diels	Asteraceae /Compositae	Le	Tassili N’Ajjer, Southern Algerian Sahara	Anti-inflammatory [headache]; wound healing	Hammiche and Maiza, 2006
Chiliadenus iphionoides (Boiss. & C.I.Blanche) Syn: *Varthemia iphionoides* Boiss. & Blanche	Asteraceae /Compositae	St	Golan Heights and West Bank, Israel	Nerve system [tremors]	Said et al., 2002
Chiococca alba (L.) Hitchc	Rubiaceae	Le, Ro	Mato Grosso, Brazil	Anti-microbial; anti-inflammatory	Ribeiro et al., 2017
Chlamydocola chlamydantha K.Schum.	Malvaceae	Fr	Cataractes and Lukaya districts, D.R. Congo	Anti-inflammatory [eye]	Latham and Konda ku Mbuta, 2016
Chlorophytum stolzii (K.Krause) Weim	Asparagaceae	Bu	Cataractes and Lukaya districts, D.R. Congo	Anti-fungal	Latham and Konda ku Mbuta, 2016
Chromolaena maximilianii (Schrad. ex DC.) R.M.King & H.Rob.	Compositae	Le, Ro, St	Mato Grosso, Brazil	Anti-microbial; anti-inflammatory	Ribeiro et al., 2017
Chromolaena odorata (L.) R.M.King & H.Rob. Syn: *Eupatorium odoratum* L.	Compositae	Le, Ro	Martinique,Trinidad; Myanmar; Satkhira District, Bangladesh	Anti-inflammatory [Fe]; anti-hypertensive; anti-microbial [cold, sore throat]	Longuefosse and Nossin, 1996; Clement et al., 2015; Ong et al., 2018; Dulla and Jahan, 2017; Hanif et al., 2009
Chromolaena scabra (L. f.) R.M. King & H. Rob.	Compositae	NS	Markets of Bogotá, Columbia	Anti-hypertensive	Bussmann et al., 2018
Chromolaena squalida (DC.) R.M.King & H.Rob.	Compositae	Le	Mato Grosso, Brazil	Anti-microbial	Ribeiro et al., 2017

Chrysactinia mexicana A.Gray	Asteraceae	FL, Le	Nuevo León, Mexico	Anti-inflammatory	Estrada-Castillón et al., 2012
Chrysanthemum morifolium Ramat.	Compositae	Le	Mao Naga people Manipur, India	Anti-microbial [dys]	Lokho, 2012
Chukrasia tabularis A.Juss.	Meliaceae	Ro, Se	Mizoram, India	Anti-microbial [dys]	Rai and Lalramnghinglova, 2010
Chuquiraga jussieui J.F.Gmel.	Compositae	WP	Loja and Zamora-Chinchipe, Ecuador	Anti-microbial [inf]	Tene et al., 2007
Chuquiraga spinosa Less.	Asteraceae	Le	Mestizo community, North Peru	Anti-inflammatory	Bussmann and Sharon, 2006
Chuquiragua weberbaueri Tovar	Asteraceae	WP	Mestizo community, North Peru	Anti-microbial	Bussmann and Sharon, 2006
Cichorium intybus L.	Asteraceae	FL, Le, St	Iran; Agro Nocerino Sarnese, Southern Italy	Anti- hyperlipidemia	Mosaddegh et al., 2013; Motti and Motti, 2017
Cichorium pumilum Jacq.	Asteraceae	WP, Le	Israel	Poison antidote, anti-inflammatory [rheum], anti-microbial	Said et al., 2002
Cinchona pubescens Vahl	Rubiaceae	NS	Markets of Bogotá, Columbia	Anti-inflammatory [Fe]	Bussmann et al., 2018
Cinnamomum spp.	Lauraceae	Ba	Eastern Highlands, Papua New Guinea	Weakness/fatigue	Jorim et al., 2012
Cinnamomum tamala (Buch.-Ham.) T.Nees & Eberm.	Lauraceae	Ba [in sp. comb]	Chin people, Myanmar; Mizoram, India	Anti-microbial [sore throat, STI]; anxiolytic; anti-inflammatory [rheum]	Ong et al., 2018; Rai and Lalramnghinglova, 2010
Cinnamomum verum J.Presl Syn*:* *Cinnamomum zeylanicum* Blume	Lauraceae	Ba, Le, Tw	Martinique; Morocco; Madagascar; Mizoram, India; Dinajpur District, Bangladesh	Anti-inflammatory; memory improvement/ enhancement; anti-inflammatory [Fe]; anti-microbial; anxiolytic	Longuefosse and Nossin, 1996; Bellakhdar et al., 1991: cited in Noe and Lehmann, 2012 [H168]; Riondato et al., 2019; Rai and Lalramnghinglova, 2010; Rahmatullah et al., 2009
Cissampelos owariensis P. Beauv. ex DC.	Menispermaceae	AP	Benin	Memory improvement/ enhancement	Adjanohoun et al., 1989: cited in Noe and Lehmann, 2012 [H168]
Cissampelos pareira L.	Menispermaceae	Le, St, Vi	Guaymi indians, Panama; Trinidad; Chittagong Hill Tracts, Bangladesh	Anti-microbial [cold]; anti-venom	Clement et al., 2015; Kadir et al., 2015
Cissus adnata Roxb.	Vitaceae	St	Dinajpur, Bangladesh	Anti-paralytic	Uddin et al., 2006
Cissus aralioides (Welw. ex Baker) Planch.	Vitaceae	NS	Cataractes and Lukaya districts, D.R. Congo; Togo	Anti-inflammatory [laryngitis, pain]; dementia; epilepsy, memory loss	Latham and Konda ku Mbuta, 2016; Kantati et al., 2016
Cissus quadrangularis L.	Vitaceae	WP, Le	Afar people, Rift Valley, Ethiopia; Abyan territory, Yemen	Anti-microbial [leprosy, lung inf, fungal skin inf, boils]	Teklehaymanot, 2017; Al-Fatimi, 2019
Cissus rotundifolia Vahl	Vitaceae	Le	Abyan territory, Yemen	Anti-microbial [cough]	Al-Fatimi, 2019
Cissus verticillata (L.) Nicolson & C.E.Jarvis Syn*:* *Cissus sicyoides* L.	Vitaceae	Le	Trinidad; Jamaica	Anti-microbial [cold, cough]	Clement et al., 2015;
Citharexylum spinosum L. Syn: *Citharexylum quadrangulare* Jacq.	Verbenaceae	Le	Martinique	Anti-inflammatory [Fe]; anti-microbial	Longuefosse and Nossin, 1996
Citrullus colocynthis (L.) Schrad.	Cucurbitaceae	Se	Iran	Anti-hyperlipidemic; anti-hypertensive	Mosaddegh et al., 2013
Citrus aurantifolia (Christm. & Panz.) Swingle	Rutaceae	Fr, Le, Se	Martinique; Trinidad; Carib people, Livingston, Guatamala;	Anti-inflammatory [Fe, eye inflammation]; anti-microbial	Girón et al., 1991; Longuefosse and Nossin, 1996; Amri and

			Kimboza forest, Tanzania; Benin; Congo-Brazzaville; Trinidad; Marquesas Islands, French Polynesia; Togo	[cold,TB,influenza, fungal skin inf, 'sorethroat]; hemiplegia; anti-PD	Kisangau, 2012; Clement et al., 2015; Girardi et al., 2015; Adjanohoun et al., 1989: cited in Noe and Lehmann, 2012; Diafouka, 1997: cited in Noe and Lehmann, 2012 [H001]; Kantati et al., 2016
Citrus x aurantiurn L.	Rutaceae	Fr, Ro	Carib people, Livingston, Guatamala; Congo-Brazaville; Bac Huong Hoa nature reserve, Vietnam; Maori people, Cook Islands; Edremit Gulf, Turkey	Anti-inflammatory [Fe, headache]; memory improvement/ enhancement; anti-microbial [influenza, UTI]	Girón et al., 1991; Adjanohoun et al., 1989: cited in Noe and Lehmann, 2012 [H168]; Lee et al., 2019; Whistler, 1985; Polat and Satıl, 2012
Citrus hystrix DC.	Rutaceae	NS	Conis Santana National Park, East Timor	Anti-inflammatory	Collins et al., 2007
Citrus limetta Riso	Rutaceae	FL, Fr	Mestizo community, North Peru	Anti-inflammatory	Bussmann and Sharon, 2006
Citrus limon (L.) N.L. Burm. f.	Rutaceae	Fr, Ro	Baka Pygmies, Gabon; Trinidad; Columbian Andes; Venda, South Africa	Anti-microbial [STI, cold]; anti-hypertensive; anti-inflammatory [Fe, rheum]; anti-fatigue	Betti et al., 2013; Tugume et al., 2016; Clement et al., 2015; Arnold and Gulumian, 1984; Cadena-González et al., 2013; York et al., 2011: cited in Noe and Lehmann, 2012
Citrus maxima (Burm.) Merr.	Rutaceae	Fr	Togo	Anti-paralytic; anti-epileptic	Kantati et al., 2016
Citrus paradisi Macfad	Rutaceae	Fr	Fundong, NW Cameroon	Anti-microbial [TB]; anti-fatigue	Focho et al.,2009
Citrus reticulata Blanco	Rutaceae	Le	Hakka people, Guangdong, China	Anti-microbial; anti-inflammatory	Au et al., 2008
Citrus sinensis (L.) Osbeck	Rutaceae	Le, FL, Fr, Ro	Caiçaras and Três Ladeiras people, Atlantic Forest, Brazil; Cataractes and Lukaya districts, D.R. Congo; Fundong, NW Cameroon	Anti-microbial [cold, influenza, dys, TB, STI]; anti-inflammatory [Fe]; anti-epileptic	Begossi et al., 2002; Gazzaneo et al., 2005; Latham and Konda ku Mbuta, 2016; Romeiras et al., 2012; Focho et al.,2009
Clausena anisata (Willd.) Hook.f. ex Benth.	Rutaceae	AP	Haya people, Kagera, NW Tanzania	Anti-convulsant, anti-hypertensive	Moshi et al., 2009
Cleistopholis glauca Pierre ex Engl. & Diels	Annonaceae	Ba	Cataractes and Lukaya districts, D.R. Congo	Anti-microbial [STI]	Latham and Konda ku Mbuta, 2016
Clematis acuminata DC.	Ranunculaceae	Ro	Chin people, Myanmar	Anti-microbial [influenza]	Ong et al., 2018
Clematis barbellata Edgew.	Ranunculaceae	FL, Le	Central Himalayas, Nepal	Anti-microbial [boil]; anti-inflammatory [pain]	Bhattarai et al., 2006
Clematis henryi Oliv.	Ranunculaceae	Ro	Tian Mu Shan Biosphere Reserve, Zhejiang	Anti-inflammatory	Chaudhary et al., 2006
Clematis heracleifolia Komarov.	Ranunculaceae	Ro	Tian Mu Shan, Zhejiang, China	Anti-microbial [HIV]	Chaudhary et al., 2006
Clematis tibetana Kuntze	Ranunculaceae	Le, St, FL	Central Himalayas, Nepal	Anti-microbial [ton'is, cough, cold]	Bhattarai et al., 2006
Cleome amblyocarpa Barratte & Murb.	Cleomaceae	AP	Tassili N'Ajjer, Southern Algerian Sahara	Anti-inflammatory [rheum]	Hammiche and Maiza, 2006

Cleome gynandra L.	Cleomaceae	Le	Mabira Forest, Uganda; Sagamu, Nigeria; Cataractes and Lukaya districts, D.R. Congo	Anti-microbial; anti-inflammatory [pain]; memory enhancement	Elufioye et al., 2012; Tugume et al., 2016; Latham and Konda ku Mbuta, 2016
Cleome viscosa L.	Cleomaceae	Le	Kani people, Western Ghats, India; Eastern Ghats, India	Anti-inflammatory [asthma]; wound healing	Ayyanar and Ignacimuthu, 2011; Rao et al., 2006
Clerodendrum cyrtophyllum Turcz.	Lamiaceae	Le	Maonan people, Guangxi Zhuang, China	Anti-inflammatory	Hong et al., 2015
Clerodendrum floribundum R.Br.	Lamiaceae	Le, Vap	Native Northern territory, Australia	Anti-microbial [cold, influenza]	Smith, 1991
Clerodendrum infortunatum L. Syn: *Clerodendrum viscosum* Vent.	Lamiaceae	Le Ro	Rakhain people, Bangladesh; Uttarakhand, India	Anti-inflammatory; anti-epileptic	Hanif et al., 2009; Sharma et al., 2013
Clerodendrum paniculatum L.	Lamiaceae	Le	Andoman + Nicobar Is., India	Anti-paralytic; anti-inflammatory [Fe]	Chander et al., 2014
Clerodendrum phlomidis L.f.	Lamiaceae	Le	Eastern Ghats, India	Anti-inflammatory [headache]	Rao et al., 2006
Clerodendrum sp.	Lamiaceae	Le	Chin people, Myanmar	Anti-inflammatory [headache]	Ong et al., 2018
Clibadium sylvestre (Aubl.) Baill.	Compositae	FL, Le, St	Mestizo community, North Peru	Anti-microbial	Bussmann and Sharon, 2006
Clidemia hirta (L.) D. Don	Melastomataceae	Le, Ro	Maromizaha forest, Madagascar	Anti-hypertensive	Riondato et al., 2019
Clinacanthus nutans (Burm.f.) Lindau	Acanthaceae	Le	Sundanese community, West Java	Anti-microbial [dys]	Roosita et al., 2008
Clinopodium nepeta subsp. *glandulosum* (Req.) Govaerts Syn: *Calamintha officinalis*	Lamiaceae	Le, St	Taounate, North Morocco	Anti-microbial [cough, cold]	El-Hilaly et al., 2003
Clinopodium taxifolium (Kunth) Govaerts	Lamiaceae	Le, St	Loja and Zamora-Chinchipe, Ecuador	Anti-inflammatory	Tene et al., 2007
Clinopodium umbrosum (M.Bieb.) K.Koch	Lamiaceae	Le, Ro, St Vap	Central Himalayas, Nepal	Anti-paralytic; anti-hypertensive; anti-inflammatory [pain]	Bhattarai et al., 2006
Clitoria ternatea L.	Fabaceae	Le, WP	Myanmar; Kani + Kamataka people, Western Ghats, India	Anti-venom; anti-microbial [cholera, sore throat]; anti-inflammatory [headache]	Nordal, 1963: cited in DeFilipps and Krupnick, 2018; Rajakumar and Shivanna, 2009; Ayyanar and Ignacimuthu, 2011
Clusia ellipticifolia Cuatrec.	Clusiaceae	Fr	Native Colombian Andes and Amazon	Anti-viral [wart]	Cadena-González et al., 2013
Cnestis platantha (Lour.) Merr.	Connaraceae	Le	Orang Asli tribe, Malaysia	Anti-inflammatory [Fe]	Samuel et al., 2010
Cnidoscolus urens (L.) Arthur	Euphorbiaceae	Ro	Mato Grosso, Brazil; Atlantic Forest, Brazil	Anti-microbial; anti-inflammatory	Ribeiro et al., 2017; Gazzaneo et al., 2005
Coccinia grandis (L.) Voigt Syn: *Coccinia indica* Wight & Arn.	Cucurbitaceae	Le, St	Abyan territory, Yemen; Kani people, Western Ghats, India	Anti-inflammatory; anti-fatigue	Al-Fatimi, 2019; Ayyanar and Ignacimuthu, 2011
Cochlospermum orinocense (Kunth) Steud.	Bixaceae	Ro	Mato Grosso, Brazil	Anti-microbial [UTI]	Ribeiro et al., 2017

Cochlospermum regium (Schrank) Pilg.	Bixaceae	Le, Ro	Mato Grosso, Brazil	Anti-microbial [STI, UTI]; anti-inflammatory	Ribeiro et al., 2017
Cochlospermum religiosum (L.) Alston	Bixaceae	Le	Gingee Hills, Tamil Nadu, India	Anti-microbial	Arulappan et al., 2015
Cocos nucifera L.	Arecaceae	Fr, Le, Ro	3 states in SW Nigeria; Mato Grosso, Brazil; Atlantic Forest, Brazil; Martinique; Mestizo community, North Peru; Cataractes and Lukaya districts, D.R. Congo; Sundanese community, West Java	Memory enhancement; anti-inflammatory; anti-microbial [dys, cholera]; wound healing	Babawale et al., 2016; Ribeiro et al., 2017; Gazzaneo et al., 2005; Longuefosse and Nossin, 1996; Bussmann and Sharon, 2006; Latham and Konda ku Mbuta, 2016; Roosita et al., 2008
Codonoboea crinita (Jack) C.L.Lim Syn: *Didymocarpus crinitus* Jack	Gesneriaceae	Le	Seberida, Riau Province, Sumatra, Indonesia	Anti-inflammatory	Mahyar et al., 1991
Codonopsis clematidea (Schrenk) C.B.Clarke	Campanulaceae	FL	Karakoram-Himalayan range, Pakistan	Memory retention	Bano et al., 2014
Codonopsis pilosula (Franch.) Nannf.	Campanulaceae	Ro	Shaanxi, China	Support immune system	Teng et al., 2011
Coffea arabica L.	Rubiaceae	Le, Se	Martinique	Anti-inflammatory [Fe]	Longuefosse and Nossin, 1996
Coffea mauritiana Lam.	Rubiaceae		Réunion	Eye inflammation	Lavergne and. Véra, 1989: in Noe and Lehmann, 2012 [H001]
Coix lacryma-jobi L.	Poaceae	WP	Tian Mu Shan Biosphere Reserve, Zhejiang	Anti-inflammatory	Chaudhary et al., 2006
Cola acuminata (P. Beauv.) Schott & Endl. Syn: *Sterculia acuminata* P. Beauv.	Malvaceae	Ba, Fr, Se	Benin; Sagamu, + Cataractes and Lukaya districts, SW Nigeria; Cataractes and Lukaya districts, D.R. Congo; Fundong, NW Cameroon	Hemiplegia/ paraplegia/ polio; anti-microbial [abscess, STI, cough]; Memory enhancement	Adjanohoun et al., 1989: cited in Noe and Lehmann, 2012 [H130]; Elufioye et al., 2012; Babawale et al., 2016; Mojisola et al., 2012; Latham and Konda ku Mbuta, 2016; Focho et al.,2009
Colletia spinosissima J.F. Gmel.	Rhamnaceae	Le, St	Comechingones people, Argentina	Anti-inflammatory [Fe]	Goleniowski et al., 2006
Colubrina asiatica (L.) Brongn.	Rhamnaceae	Le	Andoman + Nicobar Is., India	Anti-paralytic; anti-inflammatory [Fe]	Chander et al., 2014
Combretum apiculatum Sond.	Combretaceae	Ba, Ro	Kakamega County, Kenya	Anti-microbial [STI]	Odongo et al., 2018
Combretum hensii Engl. & Diels	Combretaceae	Ro	Cataractes and Lukaya districts, D.R. Congo	Anti-microbial [dys]	Latham and Konda ku Mbuta, 2016
Combretum micranthum G.Don	Combretaceae	Le	Togo	Dementia, memory loss	Kantati et al., 2016
Combretum molle R.Br. ex G.Don	Combretaceae	Ba, Le, Ro	Nigeria; Kakamega County, Kenya; Nyanza and Kamba people, Kenya; Tanzania	Anti-inflammatory [Fe]; anti-microbial [STI, whooping cough, cough]; anti-venom	Odongo et al., 2018; Amri and Kisangau, 2012; Owuor and Kisangau, 2006
Commelina benghalensis L.	Commelinaceae	St	Kani people, Western Ghats, India	Wound healing	Ayyanar and Ignacimuthu, 2011
Commelina communis L.	Commelinaceae	WP	Maonan people, Guangxi Zhuang, China	Anti-microbial [influenza, g- i inf, UTI]	Hong et al., 2015

Commelina dianthifolia Redouté	Commelinaceae	Le, St	Nuevo León, Mexico	Anti-inflammatory; anti-microbial [eye inf, diarrh]	Estrada-Castillón et al., 2012
Commelina diffusa Burm.f.	Commelinaceae	Le	Martinique; D. R. Congo	Anti-hypertensive; anti-microbial [boil, skin inf]; anti-inflammatory [eye]	Longuefosse and Nossin, 1996; Latham and Konda ku Mbuta, 2016
Commelina erecta L. Syn: *Commelina elegans* Kunth	Commelinaceae	FL	Markets of Bogotá, Columbia; Comechingones people, Argentina	Anti-inflammatory; anti-viral	Bussmann et al., 2018; Goleniowski et al., 2006;
Commicarpus helenae (Roem. & Schult.) Meikle	Nyctaginaceae	Ro, WP	Afar people, Rift Valley, Ethiopia	Anti-microbial [typhoid, *H. zoster]*	Teklehaymanot, 2017
Commiphora caudata (Wight & Arn.) Engl.	Burseraceae	Se	Eastern Ghats, India	Anti-venom	Rao et al., 2006
Commiphora foliacea Sprague	Burseraceae	St	Abyan territory, Yemen	Anti-microbial [cough, bronch, TB]	Al-Fatimi, 2019
Commiphora gileadensis (L.) C.Chr.	Burseraceae	Re	Jazan province, Saudi Arabia	Anti-inflammatory [toothache]; anti-venom; nervous system disorders	Tounekti et al., 2019
Commiphora habessinica (O.Berg) Engl.	Burseraceae	Ba	Abyan territory, Yemen	Anti-inflammatory [asthma]	Al-Fatimi, 2019
Commiphora mollis (Oliv.) Engl.	Burseraceae	Ba	Angola	Eye inflammation	Urso et al., 2016
Commiphora myrrha (T.Nees) Engl. Syn: *Commiphora molmol* (Engl.) Engl. ex Tschirch	Burseraceae	St	Abyan territory, Yemen	Anti-microbial [mouth + throat inf, influenza, cold]; boost immune system	Al-Fatimi, 2019
Convolvulus arvensis L.	Convolvulaceae	AP, Le	Turkestan Range of south Kyrgystan; Pollino National Park, Southern Italy; Thari people of Nara Desert, Pakistan	Wound healing; anti-inflammatory [rheum]	Pawera et al., 2016; Di Sanzo et al., 2013; Qureshi and Bhatti, 2008
Convolvulus prostratus Forssk Syn: *Convolvulus pluricaulis* Choisy	Convolvulaceae	Wp	Thari people, Nara Desert, Pakistan	Nerve tonic; anti-inflammatory [Fe]	Qureshi and Bhatti, 2008
Conyza sumatrensis (Retz.) E. H Walker Syn: *Erigeron floribundus* (Kunth) Sch.Bip.	Compositae	Le	Kakamega County, Kenya; Nyanza and Kamba people, Kenya; Cataractes and Lukaya districts, D.R. Congo	Anti-microbial [sore throat, leprosy]; anti-inflammatory [Fe]; anti-venom	Odongo et al., 2018; Latham and Konda ku Mbuta, 2016; Owuor and Kisangau, 2006
Conyza vernonioides (Sch.Bip. ex A.Rich.) Wild. Syn: *Conyza adolfi-fridericii* Muschl.	Compositae	Le	Mabira Forest, Uganda	Anti-microbial [eye inf]	Tugume et al., 2016
Copaifera langsdorffi Desf.	Fabaceae	Ba, Re, Se	Mato Grosso, Brazil	Anti-microbial; anti-inflammatory	Ribeiro et al., 2017
Copaifera malmei Harms	Leguminosae	Le, Se	Mato Grosso, Brazil	anti-inflammatory	Ribeiro et al., 2017
Copaifera multijuga Hayne	Fabaceae	St ba, St oil	Rio Jauaperi, Brazilian Amazon	Anti-microbial; anti-inflammatory	Pedrollo et al., 2016
Copaifera pubiflora Benth.	Fabaceae	NS	Markets of Bogotá, Columbia	anti-microbial [STI]	Bussmann et al., 2018
Copaifera reticulata Ducke	Fabaceae	Sap	Bolivian Amazon	Wound healing	Bourdy et al., 2000
Coptis teeta Wall.	Ranunculaceae	Ba, Ro	Myanmar	Anti-venom; anti-microbial [bronch, cough]	DeFilipps and Krupnick, 2018
Coptosperma sp.	Rubiaceae	Ba	Maromizaha forest, Madagascar	Anti-fatigue	Riondato et al., 2019

Corchorus aestuans L.	Malvaceae	Le	Andoman + Nicobar Is., India	Anti-inflammatory [Fe]	Chander et al., 2014
Corchorus olitorus L.	Malvaceae	Le	South Nigeria	Anti-inflammatory [Fe]; anti-microbial [STI]	Borokini et al., 2012
Cordia curassavica (Jacq.) Roem. & Schult. Syn: *Cordia verbenacea* A.DC.	Boraginaceae	Ju, Le	Trinidad	Anti-inflammatory [Fe]; anti-microbial [cold]	Clement et al., 2015
Cordia dichotoma G.Forst.	Boraginaceae	Le, PL	Ayta communities, Bataan, Philippines	Anti-microbial [inf]; anti-inflamatory [Fe]	Tantengco et al., 2018
Cordia lutea Lam.	Boraginaceae	FL	Mestizo community, North Peru	Anti-inflammatory	Bussmann and Sharon, 2006
Cordia millenii Baker.	Boraginaceae	Ro, Le, Se [in sp. comb]	Sagamu, SW Nigeria; Fundong, NW Cameroon	Anti-aging; anti-convulsant	Elufioye et al., 2012; Focho et al.,2009
Cordia subcordata Lam.	Boraginaceae	Ba, Fr, Le	Marquesas Islands, French Polynesia; Maori people, Cook Islands	Anti-microbial [cough, bronch, pneum, UTI]	Girardi et al., 2015; Whistler, 1985
Cordyline fruticosa (L.) A. Chev.	Asparagaceae	NS	Markets of Bogotá, Columbia	Anti-microbial [UTI]	Bussmann et al., 2018
Cornus mas L.	Cornaceae	Fr, Le	Albanians, Bosniaks/Gorani + Turkish ethnic groups, Kosovo; Gheg people, N-E Albania; Edremit Gulf, Turkey	Boost immunity; anti-hypertensive; anti-microbial [diarrh]; anti-ulcer	Mustafa et al., 2015; Pieroni and Sõukand, 2017; Polat and Satıl, 2012
Cornus officinalis Sieb. Et Zucc.	Cornaceae	Fr	Shaanxi, China	Anti-inflammatory; support immune system	Teng et al., 2011
Cornus sericea L.	Cornaceae	Ba	Native America	Weakness; paralysis	Smith, 1929; cited in Native American Ethnobotany Database
Corylus avellana L	Betulaceae	Le	Yörük people, SE Macedonia	Anti-microbial [sore throat]	Nedelcheva et al., 2017
Costus afer Ker-Gawler	Costaceae	St	Tropical rain forest, Central + S Cameroon; Cataractes and Lukaya districts, D.R. Congo	Anti-viral [c'pox, measles, influenza, *Herpes*], anti-inflammatory [periodontitis]	Ngono Ngane et al., 2011; Latham and Konda ku Mbuta, 2016
Costus comosus (Jacq.) Roscoe	Costaceae	St	Loja and Zamora-Chinchipe, Ecuador	Anti-microbial [influenza]	Tene et al., 2007
Costus lucanusianus J.Braun & K.Schum.	Costaceae	St	Cataractes and Lukaya districts, D.R. Congo	Anti-microbial [whooping cough, STI]	Latham and Konda ku Mbuta, 2016
Costus sericeus Blm.	Costaceae	Le	Mindanao, Philippines	Anti-inflammatory [Fe]	Malawani et al., 2017
Costus spicatus (Jacq.) Sw.	Costaceae	Fr, Le, Ro, St, WP	Mato Grosso, Brazil	Anti-microbial; anti-inflammatory	Ribeiro et al., 2017
Cotula anthemoides L.	Asteraceae	Le	Kashmir, Pakistan	Anti-microbial	Amjad et al., 2017
Coutarea hexandra (Jacq.) K.Schum.	Rubiaceae	Ba	Mato Grosso, Brazil	Anti-microbial	Ribeiro et al., 2017
Crassocephalum vitellinum (Benth.) S. Moore.	Asteraceae	Ju	Rwanda	Paralysis/ hemiplegia/ polio/ paraplegia	Van Puyvelde, 1977: cited in Noe and Lehmann, 2012 [H130]

Crataegus laciniata Ucria	Rosaceae	Le, Fr, FL	Iran	Anti-fungal; anti-hyperlipidemia; anti-hypertensive	Mosaddegh et al., 2013
Crataegus spp.	Rosaceae	Fr, Le	Taounate, North Morocco; High Atlas, Morocco	Anti-hypertensive, anxiolytic	El-Hilaly et al., 2003; Fadili *et al*, 2017
Crateva adansonii Oliv.	Capparaceae	Le, Ba	South Nigeria	Anti-hypertensive; anti-inflammatory [Fe]; anti-microbial [STI, UTI]	Borokini et al., 2012
Crescentia cujete L.	Bignoniaceae	Fr	Martinique; Trinidad; Columbian Andes; Bolicar, N Colombia	Anti-inflammatory [Fe, headache]; anti-hypertensive; anti-microbial [cold, cough, influenza]	Longuefosse and Nossin, 1996; Clement et al., 2015; Cadena-González et al., 2013; Bussmann et al., 2018; Gómez-Estrada et al., 2011
Crinum asiaticum L. Syn: *Crinum angustifolium*	Amaryllidaceae	Bu	Native Northern territory, Australia; Myanmar	Anti-microbial [leprosy], neutralise poisons	Smith, 1991; DeFilipps and Krupnick, 2018
Crinum nubicum Hannibal	Amaryllidaceae	Bu	Togo	Epilepsy; anti-paralytic	Kantati et al., 2016
Crinum sp.	Amaryllidaceae	Ro	Rakhain people, Bangladesh	Anti-inflammatory	Hanif et al., 2009
Cronquistianthus lavandulifolius D.C.	Compositae	FL, Le, St	Mestizo community, North Peru	Anti-microbial	Bussmann and Sharon, 2006
Crossopteryx febrifuga (Afzel. ex G. Don) Benth.	Rubiaceae	Ba, Le	3 states in SW Nigeria; Bas-Congo	Memory enhancement; anti-inflammatory [rheum]	Babawale et al., 2016; Latham and Konda ku Mbuta, 2016
Crotalaria eremaea F.Muell.	Fabaceae	Le	Native Northern territory, Australia	Anti-microbial [cold]	Smith, 1991
Crotalaria pallida Aiton. Hort.	Fabaceae	Ro	Kakamega County, Kenya	Anti-microbial [O inf]	Odongo et al., 2018
Crotalaria retusa L.	Fabaceae	FL	Martinique	Anti-microbial	Longuefosse and Nossin, 1996
Croton californicus Müll.Arg.	Euphorbiaceae	Ro	Chumash Indians, California, USA	Anti-inflammatory	Timbrook, 1990
Croton draconoides Müll. Arg.	Euphorbiaceae	Ba	Peruvian Amazon	Anti-fungal	Odonne et al., 2013
Croton grandivelum Baill.	Euphorbiaceae	Le, Ro	Mato Grosso, Brazil	Anti-microbial; anti-inflammatory [rheum, inflam]	Ribeiro et al., 2017
Croton hibiscifolius Kunth ex Spreng. Syn: *Croton funckianus* Müll. Arg.	Euphorbiaceae	NS	Markets of Bogotá, Columbia	Anti-inflammatory [Fe]	Bussmann et al., 2018
Croton macrostachyus Hochst. ex Delile	Euphorbiaceae	Ba, Le	Abyan territory, Yemen; Fundong, NW Cameroon	Anti-inflammatory [skin inflam]; anti-microbial [pneum]	Al-Fatimi, 2019; Focho et al.,2009
Croton mongue Baill.	Euphorbiaceae	Le	Maromizaha forest, Madagascar	Anti-inflammatory [Fe]	Riondato et al., 2019
Croton mubango Müll. Arg.	Euphorbiaceae	Ba, Le [in comb]	Bas-Congo Province, Cataractes and Lukaya districts, D.R. Congo; Angola	Anti-microbial/anti-paralytic [polio]; anti-inflammatory [pain]	Kembelo, 2003: cited in Noe and Lehmann, 2012 [H130]; Latham and Konda ku Mbuta, 2016
Croton mutisianus Kunth	Euphorbiaceae	La	Loja and Zamora-Chinchipe, Ecuador	Anti-inflammatory	Tene et al., 2007
Croton schiedeanus Schltdl.	Euphorbiaceae	NS	Markets of Bogotá, Columbia	Anti-atherosclerotic, anti-microbial [UTI]	Bussmann et al., 2018

Croton sylvaticus Hochst.	Euphorbiaceae	Ro, Ba	Cataractes and Lukaya districts, D.R. Congo	Anti-microbial; anti-inflammatory	Latham and ku Mbuta, 2016a
Croton tiglium L.	Euphorbiaceae	Le, Se, Se oil, Ro ba	Myanmar; Huanjiang Maonan people, Guangxi Zhuang, China	Anti-facial paralysis, anti-inflammatory; anti- venom, anti-microbial [*H. zoster*]	DeFilipps and Krupnick, 2018; Hong et al., 2015
Croton urucurana Baill.	Euphorbiaceae	Ba, La, Le	Mato Grosso, Brazil	Anti-microbial; anti-inflammatory	Ribeiro et al., 2017
Croton wagneri Müll.Arg.	Euphorbiaceae	Le	Loja and Zamora-Chinchipe, Ecuador	Anti-inflammatory [Fe]	Tene et al., 2007
Crudia amazonica Spruce ex Benth.	Fabaceae	St ba	Rio Jauaperi, Brazilian Amazon	Anti-microbial; anti-inflammatory	Pedrollo et al., 2016
Cryptocarya massoy Laur	Lauraceae	Ba	Maromizaha forest, Madagascar	Anti-inflammatory [Fe]	Riondato et al., 2019
Cryptolepis dubia (Burm.f.) M.R.Almeida Syn: *Cryptolepis buchananii* Roem. & Schult.	Apocynaceae	St	Magar and Majhi people, Nepal	Anti-inflammatory	Malla et al., 2015
Cucumeropsis mannii Naudin.	Curcubitaceae	Le, Se	Sagamu, SW Nigeria	Anti-aging	Elufioye et al., 2012
Cucumis anguria L.	Curcubitaceae	Fr	Mato Grosso, Brazil	Anti-hypertensive; cholesterol-reducing	Ribeiro et al., 2017
Cucumis ficifolium A.Rich.	Curcubitaceae	Ro ju	Debre Libanos Wereda, central Ethiopia	Anti-venom	Getaneh and, Girma, 2014
Cucumis myriocarpus E. Mey ex Naud	Curcubitaceae	Fr	3 Kerala tribes, Western Ghats, India	Anti-inflammatory	Marjana et al., 2018
Cucurbita maxima Duch	Curcubitaceae	Ba	Mestizo community, North Peru	Anti-inflammatory	Bussmann and Sharon, 2006
Cucurbita moschata Duchesne	Cucurbitaceae	Fr	Martinique	Anti-inflammatory; anti-microbial [measles]	Longuefosse and Nossin, 1996
Cucurbita pepo L.	Cucurbitaceae	Fr, Se	Abyan territory, Yemen; N + NE Bosnia and Herzegovina	Anti-hypertensive; anti-paralytic	Al-Fatimi, 2019; Saric-Kundalic et al., 2011
Cuminum cyminum L.	Apiaceae	Se	Middle East	Anti-paralytic	Abu-Rabia, 2012
Cunila spicata Benth.	Lamiaceae	AP	Caiçaras people, Atlantic Forest, Brazil	Anti-microbial [influenza, bronch	Begossi et al., 2002
Cupania sp.	Sapindaceae	St ba	Três Ladeiras people, Atlantic Forest, Brazil	Anti-inflammatory	Gazzaneo et al., 2005
Cuphea carthagenensis (Jacq.) J.F.Macbr	Lythraceae	Le, Ro, WP	Mato Grosso, Brazil	Anti-microbial; anti-hypertensive	Ribeiro et al., 2017
Cuphea dipetala (L.f.) Koehne	Lythraceae	NS	Markets of Bogotá, Columbia	Anti-inflammatory [throat], anti-microbial [STI]	Bussmann et al., 2018
Cuphea glutinosa Cham. & Schltdl.	Lythraceae	Le, St	Comechingones people, Argentina	Anti-hypertensive	Goleniowski et al., 2006
Cuphea strigulosa H.B.K.	Lythraceae	Le, St	Mestizo community, North Peru	Anti-inflammatory; anti-microbial	Bussmann and Sharon, 2006
Cupressus dupreziana A.Camus	Cupressaceae	Le	Tassili N'Ajjer, Southern Algerian Sahara	Anti-inflammatory [Fe]	Hammiche and Maiza, 2006
Cupressus lusitanica Mill.	Cupressaceae	Br	Europe, Mexico, Columbia	Anti-inflammatory [rheum]	Cadena-González et al., 2013

Curatella americana L.	Dilleniaceae	Bd	Markets of Bogotá, Columbia; Mato Grosso, Brazil; Bolivian Amazon	Anti-inflammatory; anti-hypertensive; anti-microbial [bronch, female inf]; anti-venom	Bussmann et al., 2018; Ribeiro et al., 2017; Bourdy et al., 2000
Curculigo pilosa (Schumach. & Thonn.) Engl.	Hypoxidaceae	Tu	Benin; Ibadan city, SW Nigeria	Memory improvement/ enhancement; anti-microbial [STI]	Adjanohoun et al., 1989: cited in Noe and Lehmann, 2012 [H168]; Gbadamosi and Egunyomi, 2014
Curcuma longa L.	Zingiberaceae	Rh	Mato Grosso, Brazil; Morocco; Nepal; Columbia	Anti-microbial [leprosy, bronch, pneum, c'pox, measles, influenza, cold]; anti-inflammatory; anti-thrombotic; memory improvement<br><br>*Toxicity Report	Ribeiro et al., 2017; Bellakhdar et al., 1991: cited in Noe and Lehmann, 2012 [H168]; Bhattarai et al., 2006; Bussmann et al., 2018<br>* Alafiatayo et al., 2019
Cuscuta americana L.	Convolvulaceae	WP	Martinique	Anti-inflammatory; memory improvement/ enhancement	Longuefosse and Nossin, 1996
Cuscuta australis R. Br.	Convolvulaceae	WP	Kakamega County, Kenya	Anti-microbial [typhoid]	Odongo et al., 2018
Cuscuta chinensis Lam.	Convolvulaceae	WP	Thari people of Nara Desert, Pakistan	Anti-inflammatory; wound healing	Qureshi and Bhatti, 2008
Cuscuta reflexa Roxb.	Convolvulaceae	Vi	Rakhain people, Bangladesh	Anti-inflammatory [Fe, pain]	Hanif et al., 2009
Cyanthillium cinereum (L.) H.Rob.	Compositae	Le, Ro	Ayta communities, Bataan, Philippines	Anti-microbial [measles]	Tantengco et al., 2018
Cyanthillium patulum (Dryand. ex Dryand.) H.Rob. Syn: *Vernonia patula* (Dryand.) Merr.	Asteraceae	Le	Andoman + Nicobar Is., India	Anti-paralytic, anti-inflammatory [Fe]	Chander et al., 2014
Cycas sp.	Cycadaceae		Conis Santana National Park, East Timor	Wound healing	Collins et al., 2007
Cyathula prostrata (L.) Blume	Amaranthaceae	WP	Mount Cameroon, Cameroon; Marquesas Islands, French Polynesia; Satkhira District, Bangladesh	Anti-microbial [dys, abscess,boil; anti-inflammatory [Fe, rheum fev]	Sandberg et al., 2005; Girardi et al., 2015; Dulla and Jahan, 2017
Cyclanthera pedata (L.) Schrad.	Cucurbitaceae	WP	Mestizo community, North Peru	Anti-inflammatory	Bussmann and Sharon, 2006
Cyclea hypoglauca (Schauer) Diels	Menispermaceae	Ro	Maonan people, Guangxi Zhuang, China	Anti-microbial [diphth]	Hong et al., 2015
Cyclea barbata Miers	Menispermaceae	Le	Sundanese community, West Java	Anti-inflammatory	Roosita et al., 2008
Cyclea peltata (Lam.) J. Hooker & Thoms.	Menispermaceae	Le	Karnataka, India	Anti-microbial [dys]	Rajakumar and Shivanna, 2009
Cydonia oblonga Mill.	Rosaceae	Le	Yörük people, SE Macedonia	Anti-microbial [sore throat]	Nedelcheva et al., 2017
Cymbopogon bombycinus (R.Br.) Domin	Poaceae	St, Le	Native Northern territory, Australia	Anti-microbial [cold]	Smith, 1991
Cymbopogon citratus (DC.) Stapf. Syn: *Andropogon citratus* DC.	Poaceae	Le [in comb]	Caiçaras and Três Ladeiras people, Atlantic Forest, Brazil; Carib people, Livingston,	Anti-microbial [polio, influenza]; anti-inflammatory [Fe]; memory	Girón et al., 1991; Kembelo, 2003, Adjanohoun et al., 1989: cited in Noe and Lehmann, 2012

			Guatamala; Philippines; Benin; D.R. Congo	improvement/ enhancement; anti-hypertensive	[H130, H168]; Begossi et al.,2002; Malawani et al., 2017
Cymbopogon densiflorus (Steud.) Stapf	Poaceae	FL	Cataractes and Lukaya districts, D.R. Congo	Dementia; anti-microbial [cough]; anti-inflammatory [rheum]	Latham and Konda ku Mbuta, 2016
Cymbopogon giganteus Chiov	Poaceae	Le, St, WP	Benin; Togo	Hemiplegia/ paraplegia/ polio; memory improvement/ enhancement	Adjanohoun et al., 1989; Adjanohoun et al., 1986: cited in Noe and Lehmann, 2012 [H130; H168]
Cymbopogon nardus (L.) Rendle	Poaceae	Ju, Le, Ro	Myanmar; Mabira Forest, Uganda	arm or leg paralysis;, anti-microbial [coughs and colds, eye inf]; anti-inflammatory [joint inflam, Fe]	Nordal, 1963: cited in DeFilipps and Krupnick, 2018; Tugume et al., 2016
Cymbopogon obtectus S.T.Blake	Poaceae	FL, Le, St	Native Northern territory, Australia	Anti-microbial [colds, influenza]; anti-inflammatory	Smith, 1991
Cymbopogon proximus (Hochst. ex A. Rich) Stapf. Syn: *Cymbopogon schoenanthus* (L.) Spreng. subsp. *proximum* (Hochst. ex A. Rich.) Maire & Weiller	Po**a**ceae	WP	Benin; Tassili N'Ajjer, Southern Algerian Sahara	Hemiplegia/ paraplegia/ polio; anti-inflammatory [Fe, rheum]	Adjanohoun et al., 1989: cited in Noe and Lehmann, 2012 [H130]; Hammiche and Maiza, 2006
Cynanchum lancifolium Hook. and Am.	Asclepiadaceae	Le	Mapuche people, Chile	Poisoning antidote	Houghton and Manby, 1985
Cynanchum vanlessenii (Lavranos) Goyder	Apocynaceae	La	Abyan territory, Yemen	Anti-microbial [boils, skin inf]	Al-Fatimi, 2019
Cynanchum viminale subsp. *Stipitaceum* (Forssk.) Meve & Liede	Apocynaceae	La, St	Abyan territory, Yemen	Anti-microbial [boils]; memory loss, wound healing	Al-Fatimi, 2019
Cynara cardunculus L.	Asteraceae	NS	Markets of Bogotá, Columbia	Anti-atherosclerotic	Bussmann et al., 2018
Cynara scolymus L.	Compositae	Le, Rc, Se	Mato Grosso, Brazil; Agro Nocerino Sarnese, Southern Italy	Anti-inflammatory; high cholesterol	Ribeiro et al., 2017; Motti and Motti, 2017
Cynodon dactylon (L.) Pers.	Poaceae	Fr, Le, Ro, Se, WP	Mato Grosso, Brazil; Karnataka, India; Satkhira District, Bangladesh; Pollino National Park, Southern Italy; Kathua, Jammu and Kashmir, India; Kani people, Western Ghats, India; Agro Nocerino Sarnese, Southern Italy	Anti-microbial [TB, UTI], anti-inflammatory [Fe, asthma]; memory improvement	Ribeiro et al., 2017; Rajakumar and Shivanna, 2009; Dulla and Jahan, 2017; Di Sanzo et al., 2013; Kumar and Bhagat, 2012; Ayyanar and Ignacimuthu, 2011; Motti and Motti, 2017
Cyperus rotundus L.	Cyperaceae	Tu	Maonan people, Guangxi Zhuang, China; Dinajpur, Bangladesh	Anti-microbial [cold]; anti-paralytic	Hong et al., 2015; Uddin et al., 2006
Cyphomandra betacea (Cav.) Sendtn. [unresolved]	Solanaceae	Fr	Loja and Zamora-Chinchipe, Ecuador	Cholesterol-reducing	Tene et al., 2007

Cyphostemma adenocaule (Steud. ex A.Rich.) Desc. ex Wild & R.B.Drumm.	Vitaceae	Le	Mabira Forest, Uganda	Anti-microbial [Measles, STI]	Tugume et al., 2016
Cyrtomium fortunei J. Sm.	Dryopteridaceae	WP	Maonan people, Guangxi Zhuang, China	Anti-viral [cold]	Hong et al., 2015
Dacryodes edulis (G.Don) H.J.Lam	Burseraceae	Ba, Le	Bas-Congo; Fundong, NW Cameroon	Anti-bacterial [dys]; anti-convulsant	Latham and Konda ku Mbuta, 2016; Focho et al.,2009
Dactylorhiza hatagirea D.Don	Orchidaceae	Rh	Himachal Pradesh, NW Himalaya, India	Anti-inflammatory [Fe]	Vidyarthi et al., 2013
Dalbergia lactea (Roxb) Vatke.	Fabaceae	Le Ba [in spp. comb]	Sagamu, SW Nigeria	Memory enhancement	Elufioye et al., 2012
Daniellia oliveri (Rolfe) Hutch. & Dalziel	Fabaceae	Le	Benin	Hemiplegia/ paraplegia/ polio	Adjanohoun et al., 1989: cited in Noe and Lehmann, 2012 [H130]
Daphne gnidium L.	Thymelaeaceae	AP	M'Sila, North Algeria	Anti-hypertensive	Boudjelal et al., 2013
Daphne mucronata Royle	Thymelaeaceae	AP, Ba, Br, Le	Iran	Anti-microbial	Mosaddegh et al., 2013
Daphniphyllum himalense (Benth.) Mull. Arg.	Daphniphyllaceae	Ba	Magar and Majhi people, Nepal	Anti-microbial [boil]	Malla et al., 2015
Dasymaschalon longiflorum (Roxb.) Finet & Gagnep	Annonaceae	Le, Ro	Mizoram, India	Chronic ulcer; anti-inflammatory [asthma]	Rai and Lalramnghinglova, 2010
Datisca glomerata (C.Presl) Baill	Datiscaceae	Ro	Chumash Indians, California, USA	Anti-microbial [sore throat]	Timbrook, 1990
Daucus montanus Humb. & Bonpl. ex Schult.	Apiaceae	FL, Le, St	Loja and Zamora-Chinchipe, Ecuador; Mestizo community, North Peru	Anti-microbial [int inf]; anti-inflammatory	Tene et al., 2007; Bussmann and Sharon, 2006
Davilla rugosa Poir.	Dilleniaceae	Le	Mato Grosso, Brazil	Anti-hypertensive	Ribeiro et al., 2017
Descurainia sophia (L.) Webb ex Prantl	Brassicaceae	Se	Iran	Ant-viral [measles]	Mosaddegh et al., 2013
Desmodium gangeticum (L.) DC.	Fabaceae	Ro	Cataractes and Lukaya districts, D.R. Congo; Chittagong Hill Tracts, Bangladesh	Anti-microbial; anti-inflammatory [Fe]; anti-venom	Latham and Konda ku Mbuta, 2016; Kadir et al., 2015
Desmodium heterocarpon (L.) DC.	Fabaceae	WP, Ro	Maonan people, Guangxi Zhuang, China; Toli Peer National Park, Kashmir, Pakistan	Anti-microbial [mumps, encephalitis B, dys]; anti-inflammatory [Fe]	Hong et al., 2015; Amjad et al., 2017
Desmodium incanum (Sw.) DC. Syns: *D. mauritianum* (Willd.) DC., *D. canum* (J.F.Gmel.) Schinz & Thell.	Fabaceae	Le	Cataractes and Lukaya districts, D.R. Congo	Anti-microbial [dys]	Latham and Konda ku Mbuta, 2016
Desmodium molliculum (H.B.K.) DC.	Fabaceae	WP	Mestizo community, North Peru	Anti-inflammatory	Bussmann and Sharon, 2006

Desmodium velutinum Syn: *D. lasiocarpum* (P.Beauv.) DC.	Fabaceae	Le	Cataractes and Lukaya districts, D.R. Congo	Anti-microbial [dys]	Latham and Konda ku Mbuta, 2016
Detarium microcarpum Guill. & Perr.	Fabaceae	St, Le [parts in comb]	Benin; 3 states in SW Nigeria	Memory improvement/ enhancement	Adjanohoun et al., 1989: cited in Noe and Lehmann, 2012 [H168]; Babawale et al., 2016; Mojisola et al., 2012
Deverra denudata (Viv.) Pfisterer & Podlech Syn: *Pituranthos chloranthus* (Coss. & Durieu) Schinz	Apiaceae	AP	Tassili N'Ajjer, Southern Algerian Sahara	Anti-inflammatory [Fe]	Hammiche and Maiza, 2006
Deverra scoparia Coss. & Durieu *Pituranthos scoparius* (Coss. & Durieu) Schinz	Apiaceae	AP	Tassili N'Ajjer, Southern Algerian Sahara	Anti-microbial [UTI]	Hammiche and Maiza, 2006
Dialium pachyphyllum Harms	Fabaceae	Le	Baka Pygmies, Gabon	Anti-hypertensive	Betti et al., 2013
Dichaea muricata (Sw.) Lindl.	Orchidaceae	WP	Guaymi indians, Panama	Anti-microbial [eye inf]	Joly et al., 1990
Dichrocephala integrifolia (Linn.f.) Kuntze	Compositae	Le	Mao Naga people Manipur, India; Kakamega County, Kenya	Anti-microbial [anti-fungal]	Lokho, 2012 Odongo et al., 2018
Dichrostachys cinerea (L.) Wight & Arn.	Fabaceae	Le	Eastern Ghats, India	Anti-inflammatory [headache]	Rao et al., 2006
Dicliptera bupleuroides Nees	Acanthaceae	Le	Himachal Pradesh, NW Himalaya, India	Anti-inflammatory	Vidyarthi et al., 2013
Dicliptera chinensis (L.) Juss.	Acanthaceae	WP	Maonan people, Guangxi Zhuang, China	Anti-inflammatory	Hong et al., 2015
Dictamnus albus L.	Rutaceae	AP	Catalonia, Spain	Anti-hypertensive	Raja et al., 1997
Diervilla lonicera Mill.	Caprifoliaceae	Ro	Native America	Anti-aging	Smith, 1923: cited in Native American Ethnobotany Database
Digitaria abyssinica (Hochst. ex A.Rich.) Stapf	Poaceae	Le	Mabira Forest, Uganda	Anti-microbial	Tugume et al., 2016
Digitaria debilis (Desf.) Willd.	Poaceae	Le	Sagamu, SW Nigeria	Memory enhancement	Elufioye et al., 2012
Digitaria insularis (L.) Mez ex Ekman	Poaceae	AP, Ro	Mato Grosso, Brazil	Anti-microbial	Ribeiro et al., 2017
Dillenia excelsa (Jack) Martelli ex Gilg.	Dilleniaceae	Ro	Dayaknese Communities, W Kalimantan, Indonesia	Anti-microbial [cough, influenza]	Diba et al., 2013
Dillenia indica L.	Dilleniaceae	Ba, Fr	Mizoram, India	Anti-microbial [dys]	Rai and Lalramnghinglova, 2010
Dillenia pentagyna Roxb.	Dilleniaceae	Ba, Wo	Mizoram, India	Anti-inflammatory [rheum]; ulcer	Rai and Lalramnghinglova, 2010
Dilodendron bipinnatum Radlk.	Sapindaceae	Ba	Mato Grosso, Brazil	Anti-inflammatory	Ribeiro et al., 2017
Dimorphandra mollis Benth.	Fabaceae	Le	Mato Grosso, Brazil	Anti-inflammatory [pain]	Ribeiro et al., 2017
Dioscorea alata L.	Dioscoreaceae	Ro	Bougainville, Solomon Islands	Anti-hypertensive, anti-transient paralysis, anti-inflammatiory [migraine]	Waruruai et al., 2011

Dioscorea bulbifera L.	Dioscoreaceae	Ae bu, Rh	Mato Grosso, Brazil; throughout Africa	Anti-microbial [eye inf, abscess]	Ribeiro et al., 2017
Dioscorea communis (L.) Caddick & Wilkin Syn: *Tamus communis* L.	Dioscoreaceae	Ro ju, Tu	Pollino National Park, Southern Italy; Edremit Gulf, Turkey	Anti-inflammatiory [rheum]	Di Sanzo et al., 2013; Polat and Satıl, 2012
Dioscorea deltoidea Wall. ex Kunth	Dioscoreaceae	Tu	Himachal Pradesh, NW Himalaya, India	Anti-microbial [dys]	Vidyarthi et al., 2013
Dioscorea hispida Dennst.	Dioscoreaceae	Ro	Ayta communities, Bataan, Philippines	Anti-microbial [rabies]	Tantengco et al., 2018
Dioscorea mangenotiana Meige.	Dioscoreaceae	Le, Tu	Sagamu and Ondo State, Nigeria,	Memory enhancement	Elufioye et al., 2012; Mojisola et al., 2012
Dioscorea tambillensis Kunth	Dioscoreaceae	Tu	Mestizo community, North Peru	Anti-inflammatory	Bussmann and Sharon, 2006
Dioscorea trifida L.f.	Dioscoreaceae	Tu	Mestizo community, North Peru	Anti-inflammatory	Bussmann and Sharon, 2006
Diospyros hispida A.DC	Ebenaceae	Ba, Se	Mato Grosso, Brazil	Stroke prevention/ therapy	Ribeiro et al., 2017
Diospyros melanoxylon Roxb.	Ebenaceae	Se	Eastern Ghats, India	Anti-inflammatory [asthma]; anti-microbial [bronc]	Rao et al., 2006
Diospyros mespiliformis Hochst. ex A.DC.	Ebenaceae	St ba	Togo	TBI	Kantati et al., 2016
Diplostephium gynoxyoides Cuatr.	Compositae	FL	Mestizo community, North Peru	Anti-inflammatory	Bussmann and Sharon, 2006
Dipteryx alata Vogel	Leguminosae	Ba, Fr, Le, Se, St	Mato Grosso, Brazil	Anti-microbial; anti-inflammatory [rheum]; cholesterol-reducing; anti-thrombotic; memory improvement; anti-venom	Ribeiro et al., 2017
Dirca palustris L.	Thymelaeaceae	Ro	Native America	Anti-inflammatory [int inflam]	Herrick, 1977; cited in Native American Ethnobotany Database
Discaria americana Gillies. & Hook.	Rhamnaceae	Ba, Ro	Comechingones people,	Anti-inflammatory [Fe]	Goleniowski et al., 2006
Donax canniformis (G.Forst.) K.Schum.	Marantaceae	Le	Seberida, Riau Province, Sumatra, Indonesia	Anti-inflammatory	Mahyar et al., 1991
Dovyalis abyssinica (A.Rich.) Warb.	Salicaceae	Ro	Debre Libanos Wereda, central Ethiopia	Anti-hypertensive; anti-microbial [periodontitis]	Getaneh and, Girma, 2014
Dovyalis macrocalyx (Oliv.) Warb.	Salicaceae	Le, Ro	Kakamega County, Kenya	Anti-microbial [boil]	Odongo et al., 2018
Dracaena spicata Roxb.	Asparagaceae	Le	Dinajpur District, Bangladesh	Anti-paralytic	Rahmatullah et al., 2009
Dracaena steudneri Engl.	Asparagaceae	Le	Mabira Forest, Uganda	Anti-microbial [skin inf, STI	Tugume et al., 2016
Dracocephalum stamineum Kar. & Kir.	Anacardiaceae	AP	Turkestan Range of south Kyrgystan	Anti-hypertensive	Pawera et al., 2016
Dracontomelon dao (Blanco) Merr. & Rolfe	Anacardiaceae	St	Ayta communities, Bataan, Philippines	Anti-microbial [wound inf]	Tantengco et al., 2018
Dregea volubilis (L.f.) Benth. ex Hook.f.	Apocynaceae	Le	Chin people, Myanmar	Anti-hypertensive	Ong et al., 2018
Drymis winteri J.R.Forst. & G.Forst.	Winteraceae	Le	Chile	Anti-virus [warts]	Houghton and Manby, 1985
Drymonia serrulata (Jacq.) Mart.	Gesneriaceae	St	Guaymi indians, Panama; Markets of Bogotá, Columbia	Anti-inflammatory [Fe]	Joly et al., 1987; Bussman et al., 2018

Drynaria quercifolia (L.) J. Sm.	Polypodiaceae	NS	Conis Santana National Park, East Timor	Anti-microbial [sore throat]	Collins et al., 2007
Duchesnea indica (Jacks.) Focke	Rosaceae	WP	Hakka people, Guangdong, China;	Anti-microbial [*H. zoster*]	Au et al., 2008
Duguetia furfuracea (A.St.-Hil.) Saff.	Annonaceae	AP, Le, Ro	Mato Grosso, Brazil	Anti-microbial; anti-hypertensive; anti-inflammatory	Ribeiro et al., 2017
Durio zibethinus L.	Malvaceae	Ba	Sundanese community, West Java	Anti-bacterial [dys]	Roosita et al., 2008
Dysosma versipellis (Hance) M.Cheng	Berberidaceae	Rh	Maonan people, Guangxi Zhuang, China	Anti-viral [mumps]	Hong et al., 2015
Dysphania ambrosioides (L.) Mosyakin & Clemants Syn: *Chenopodium ambrosioides* L.	Amaranthaceae	Le, Ro, St	Brazilian Amazon; Nuevo León, Mexico; Maranao people, Philippines; Loja and Zamora-Chinchipe, Ecuador; Martinique; Chile: Mao Naga people Manipur, India; High Atlas, Morocco; Bolivian Amazon; Potosi, Bolivia	Anti-microbial [pneum; measles, typhoid, UTI]; memory improvement; anti-inflammatory [asthma]; anti-hypertensive; anti-stroke	Pedrollo et al., 2016; Estrada-Castillón et al., 2012; Tene et al., 2007; Malawani et al., 2017; Longuefosse and Nossin, 1996; Houghton and Manby, 1985; Lokho, 2012; Fadili *et al*, 2017; Bourdy et al., 2000; Fernandez et al., 2003
Dysphania schraderiana (Schult.) Mosyakin & Clemants	Amaranthaceae	Le	Abyan territory, Yemen	Anti-microbial [UTI]	Al-Fatimi, 2019
Echeveria peruviana Meyen	Crassulaceae	Le	Mestizo community, North Peru	Anti-inflammatory	Bussmann and Sharon, 2006
Echinodorus scaber Rataj	Alismataceae	Le, Ro, St	Mato Grosso, Brazil	Anti-microbial; anti-hypertensive	Ribeiro et al., 2017
Echinops spinosissimus subsp. bovei (Boiss.) Greuter	Compositae	AP	Tassili N'Ajjer, Southern Algerian Sahara	Anti-inflammatory	Hammiche and Maiza, 2006
Ehretia cymosa Thonning	Boraginaceae	Le	Benin; South Nigeria	Memory improvement/ enhancement; anti-inflammatory; anti-paralytic; anti-microbial	Adjanohoun et al., 1989: cited in Noe and Lehmann, 2012 [H168]; Borokini et al., 2012
Elaeagnus angustifolia L.	Elaeagnaceae	Fr	Toli Peer National Park, Kashmir, Pakistan	Anti-microbial	Amjad et al., 2017
Elaeagnus umbellata Thunb.	Elaeagnaceae	Le, Se	Toli Peer National Park, Kashmir, Pakistan	Anti-microbial, immunomodulatory	Amjad et al., 2017
Elaeis guineensis Jacq.	Arecaceae	Fr, Le, Oil	Sagamu, SW Nigeria; Três Ladeiras people, Atlantic Forest, Brazil; Fundong, NW Cameroon	Memory enhancement; anti-microbial [boil, dys]; eye inflammation; anti-convulsant	Elufioye et al., 2012; Gazzaneo et al., 2005; Diafouka, 1997: cited in Noe and Lehmann, 2012 [H001]; Focho et al., 2009
Elaeocarpus subserratus Baker	Elaeocarpaceae	Le	Maromizaha forest, Madagascar	Anti-fatigue	Riondato et al., 2019
Elephantopus scaber L.	Asteraceae	Wp	Huanjiang Maonan people, Guangxi Zhuang, China	Anti-microbial	Hong et al., 2015
Elettaria cardamomum (L.) Maton	Zingiberaceae	Fr	Kani people, Western Ghats, India	Anti-inflammatory [headache, rheum asthma]	Ayyanar and Ignacimuthu, 2011
Eleusine indica (L.) Gaertn.	Poaceae	WP	Mount Cameroon, Cameroon	Anti-microbial	Sandberg et al., 2005
Eleutherine bulbosa (Mill.) Urb. Syns: *Eleutherine plicata* (Sw.)	Iridaceae	Ro	Três Ladeiras people, Atlantic Forest + Rio Jauaperi, Brazil	Anti-microbial [influenza]; memory improvement	Gazzaneo et al., 2005; Pedrollo et al., 2016

Herb.; *Eleutherine americana* (Aubl.) Merr. ex K.Heyne					
Elionurus sp.	Poaceae	Le	Mato Grosso, Brazil	Anti-microbial	Ribeiro et al., 2017
Elsholtzia blanda (Benth.) Benth.	Lamiaceae	Le	Mao Naga people, Manipur, India; Mizoram, India	Anti-hypertensive; anti-microbial [cholera]; anti-inflammatory [Fe]	Lokho, 2012; Rai and Lalramnghinglova, 2010
Embelia ribes Burm. f.	Primulaceae	Le [in spp. comb]	Karnataka, India	Anti-paralytic	Rajakumar and Shivanna, 2009
Embothrium coccineum J.R.Forst. & G.Forst.	Proteaceae	Ba, Le	Mapuche people, Chile	Anti-microbial [gland inf]	Houghton and Manby, 1985
Emilia abyssinica (Sch.Bip. ex A.Rich.) C.Jeffrey Syn*: Senecio abyssinicus* Sch.Bip. ex Hochst.	Asteraceae/Compositae	AP	Ondo State, SW Nigeria	Memory improvement	Mojisola et al., 2012
Emilia coccinea (Sims) G.Don	Asteraceae	Le	Cataractes and Lukaya districts, D.R. Congo	Anti-microbial; anti-oxidant; anti-inflammatory	Latham and Konda ku Mbuta, 2016
Emilia fosbergii Nicolson	Asteraceae	Le	Martinique	Anti-inflammatory [inflam]	Longuefosse and Nossin, 1996
Emilia sonchifolia DC.	Asteraceae	Le, WP	Maonan people, Guangxi Zhuang, China; Karnataka, India; 3 Kerala tribes, Western Ghats, India	Anti-microbial [leprosy, ton'is]	Hong et al., 2015; Rajakumar and Shivanna, 2009; Marjana et al., 2018
Enicostema axillare (Poir. ex Lam.) A.Raynal	Gentianaceae	WP	Gingee Hills, Villupuram, Tamil Nadu	Anti-inflammatory, anti-venom	Arulappan et al., 2015
Enicostema verticillatum (L.) Engl.	Gentianaceae	Le	Martinique; Trinidad	Anti-inflammatory [Fe]	Longuefosse and Nossin, 1996; Clement et al., 2015
Entada abyssinica Steud. ex A.Rich.	Fabaceae	Ba	Fundong, NW Cameroon	Anti-microbial [STI]	Focho et al.,2009
Entada pursaetha DC.	Fabaceae	Se	India	Anti-paralytic	Mikawlrawng et al., 2017
Entandrophragma utile (Dawe& Sprague) Sprague.	Meliaceae	St ba	Sagamu, SW Nigeria	Memory enhancement	Elufioye et al., 2012
Ephedra altissima Desf.	Ephedraceae	AP	Tassili N'Ajjer, Southern Algerian Sahara	Anti-inflammatory [asthma]; anti-microbial [bronch]; anti-hypertensive	Hammiche and Maiza, 2006
Ephedra americana Humb. & Bonpl. ex Willd.	Ephedraceae	AP	Potosi, Bolivia	Anti-inflammatory [asthma]; anti-microbial [cold, influenza]	Fernandez et al., 2003
Ephedra gerardiana Wall. ex Klotzsch & Garcke	Ephedraceae	Ro	Central Himalayas, Nepal	Anti-microbial	Bhattarai et al., 2006
Ephedra viridis Coville	Ephedraceae	NS	Chumash Indians, California, USA	Wound antiseptic wash	Timbrook, 1990
Epidendrum difforme Jacq.	Orchidaceae	Le	Guaymi indians, Panama	Anti-microbial [rubella]	Joly et al., 1987
Epidendrum sp.	Orchidaceae	FL, Le	Loja and Zamora-Chinchipe, Ecuador	Anti-microbial [influenza, conj]	Tene et al., 2007
Equisetum bogotense (H.B.K.)	Equisetaceae	St	Mestizo community, North Peru; Markets of Bogotá, Columbia	Anti-inflammatory; arteriosclerosis, anti-microbial [dys, UTI]	Bussmann and Sharon, 2006; Bussmann et al., 2018

Equisetum giganteum (Wedd.)	Equisetaceae	Le, St, WP	Mestizo community, North Peru; native Central and South America	Anti-inflammatory; Anti-microbial [skin infs]	Bussmann and Sharon, 2006; Cadena-González et al., 2013
*Equisetum hyemal*e L.	Equisetaceae	WP	Mato Grosso, Brazil	Anti-inflammatory; anti-hypertensive	Ribeiro et al., 2017
Erechtites hieracifolia (L.) Raf. ex DC. var. *cacalioides]* (Fisch. ex Spreng.) Griseb	Asteraceae	Le, St	Comechingones people, Argentina	Anti-microbial	Goleniowski et al., 2006
Eremophila alternifolia R.Br.	Scrophulariaceae	Le	Native Northern territory, Australia	Anti-microbial [cold, influenza]; anti-inflammatory [headache]	Smith, 1991
Eremophila bignoniiflora (Benth.) F.Muell.	Scrophulariaceae	Le	Native Northern territory, Australia	Anti-microbial; anti-inflammatory	Smith, 1991
Eremophila duttonii F.Muell.	Scrophulariaceae	Le	Native Northern territory, Australia	Anti-microbial [cold, influenza]	Smith, 1991
Eremophila latrobei F. Muell. subsp. *glabra* (L.S. Smith) Chinn.	Scrophulariaceae	Le, FL, St	Native Northern territory, Australia	Anti-viral [cold,influenza]	Smith, 1991
Erica arborea L.	Ericaceae	FL br	Edremit Gulf, Turkey	Anti-inflammatory [asthma]	Polat and Satıl, 2012
Eriobotrya japonica (Thunb.) Lindl.	Rosaceae	Le, FL	Loja and Zamora-Chinchipe, Ecuador; Maromizaha forest, Madagascar	Anti-microbial [pneum]; cholesterol-reducing	Tene et al., 2007; Riondato et al., 2019
Eriodictyon crassifolium Benth.	Boraginaceae	Le	Chumash Indians, California, USA	Anti-microbial [cough, cold]; anti-inflammatory [Fe]	Timbrook, 1990
Eriogonum fasciculatum Benth.	Polygonaceae	WP	Chumash Indians, California, USA	Anti-inflammatory [rheum]	Timbrook, 1990
Eriosema psoraleoides (Lam.) G.Don	Fabaceae	Fr, Le, RO	Cataractes and Lukaya districts, D.R. Congo	Anti-microbial [conj, STI	Latham and Konda ku Mbuta, 2016
Erodium cicutarium (L.) L'Herit.	Geraniaceae	Le, St, WP	Loja and Zamora-Chinchipe, Ecuador; Mestizo community, North Peru	Anti-microbial [influenza]	Tene et al., 2007; Bussmann and Sharon, 2006
Erodium moschatum (L.) L'Hér.	Geraniaceae	Le	Loja and Zamora-Chinchipe, Ecuador	Anti-microbial [pneum, influenza]	Tene et al., 2007
Eryngium caeruleum M. Bieb.	Apiaceae	Ro	Myanmar	Anti-paralytic	Nordal, 1963: cited in DeFilipps and Krupnick, 2018
Eryngium creticum Lam.	Apiaceae	Ro	Saudi Arabia	Anti-paralytic	Ghazanfar, 1994**:** cited in Abu-Rabia, 2012
Eryngium foetidum L.	Apiaceae	WP	Martinique; Markets of Bogotá, Columbia	Anti-inflammatory [Fe]; anti-microbial [s'pox]	Longuefosse and Nossin, 1996; Bussmann et al., 2018
Erythrina abyssinica Lam. ex DC.	Fabaceae	Le, Ro	Mabira Forest, Uganda; Benin; Cataractes and Lukaya districts, D.R. Congo	Anti-microbial [sore, abscess, yell fev]; memory improvement/ enhancement	Tugume et al., 2016; Adjanohoun et al., 1989: cited in Noe and Lehmann, 2012 [H168]; Latham and Konda ku Mbuta, 2016
Erythrina dominguezii Hassl.	Fabaceae	Ba	Bolivian Amazon	Wound healing	Bourdy et al., 2000

Erythrina edulis Triana ex Micheli	Fabaceae	Ba	Loja and Zamora-Chinchipe, Ecuador	Anti-hypertensive	Tene et al., 2007
Erythrina senegalensis A. DC.	Fabaceae	Le	Forest Regions, Guinea; Mali; Benin	Anti-microbial; memory improvement/ enhancement; paralysis/ hemiplegia/ polio/ paraplegia; eye inflammation	Adjanohoun et al., 1981, 1989: cited in Noe and Lehmann, 2012 [H001, H130, H168]
Erythrina stricta Roxb.	Fabaceae	Ba	Mizoram, India	Anti-venom, anti-ulcer	Rai and Lalramnghinglova, 2010
Erythrina variegata L.	Fabaceae	Le	Karnataka, India; Chittagong Hill Tracts, Bangladesh	Anti-microbial; anti-venom	Rajakumar and Shivanna, 2009; Kadir et al., 2015
Erythrina verna Vell.	Fabaceae	Ba, Le	Mato Grosso, Brazil	Anti-microbial; anxiolytic	Ribeiro et al., 2017
Erythrochiton fallax Kallunki	Rutaceae	FL	Bolivian Amazon	Anti-inflammatory [toothache]	Bourdy et al., 2000
Erythroxylum coca Lam.	Erythroxylaceae	Le	Colombia	Anti-microbial [conj]; anti-inflammatory [rheum, pain]	Bussmann et al., 2018; Cadena-González et al., 2013
*Escallon*ia *pendula* (Ruiz & Pav.) Pers.	Escalloniaceae	AP	Potosi, Bolivia	Anti-inflammatory [rheum, asthma]; anti-microbial [cough]	Fernandez et al., 2003
Eucalyptus camaldulensis Dehnh	Myrtaceae	Le	Native Northern territory, Australia; Abyan territory, Yemen	Anti-microbial [sore throat,RTI]; anti-inflammatory [headache]	Smith, 1991; Al-Fatimi, 2019
Eucalyptus citriodora Hook.	Myrtaceae	Le	Cataractes and Lukaya districts, D.R. Congo; Três Ladeiras people, Atlantic Forest, Brazil	Anti-bacterial; anti-inflammatory [Fe]	Latham and Konda ku Mbuta, 2016; Gazzaneo et al., 2005
Eucalyptus globulus Labill*.*	Myrtaceae	Le	M'Sila, North Algeria; Fundong, NW Cameroon; Bolicar, N Colombia	Anti-microbial [cold, influenza]; anti-inflammatory [asthma]	Boudjelal et al., 2013; Focho et al.,2009; Gómez-Estrada et al., 2011
Eucalyptus pruinosa Schauer	Myrtaceae	Ba	Native Northern territory, Australia	Anti-microbial [cold, influenza]	Smith, 1991
Eucalyptus robusta Smith	Myrtaceae	Le	Maonan people, Guangxi Zhuang, China	Anti-microbial	Hong et al., 2015
Eucalyptus tetrodonta F.Muell.	Myrtaceae	Le	Native Northern territory, Australia	Anti-microbial cold, influenza, bronc]; anti-inflammatory [headache]	Smith, 1991
Euclea racemosa subsp. *schimperi* (A. DC.) F. White	Ebenaceae	Ro ba, Ro	Tigray, Ethiopia	Anti-paralytic; anti-venom	Teklay et al., 2013
Eucommia ulmoides Oliv.	Eucommiaceae	Ba	China: Lisu people, Yunnan; Shaanxi; Maonan people, Guangxi Zhuang	Anti-hypertensive [Hong et al., 2015]	Ji et al., 2005; Teng et al., 2011
Eugenia dysenterica DC.	Myrtaceae	Ba, Le	Mato Grosso, Brazil	Anti-inflammatory; anti-hypertensive	Ribeiro et al., 2017
Eugenia obtusifolia Cambes.	Myrtaceae	Le	Mestizo community, North Peru	Anti-inflammatory	Bussmann and Sharon, 2006
Eugenia pitanga (O.Berg) Nied.	Myrtaceae	Le	Mato Grosso, Brazil	Anti-microbial	Ribeiro et al., 2017
Eugenia uniflora L.	Myrtaceae	Le	Três Ladeiras people, Atlantic Forest, Brazil	Anti-microbial [influenza, UTI, dys]	Begossi et al., 2002; Gazzaneo et al., 2005
Eupatorium cannabinum L.	Asteraceae/Compositae	AP	Catalonia, Spain	Anti-microbial [pneum]	Raja et al., 1997

Euphorbia abyssinica J.F.Gmel.	Euphorbiaceae	Ne	Oromo people, Harla, Eastern Ethiopia	Anti-paralytic	Belayneh et al., 2012: cited in Noe and Lehmann, 2012 [H130]
Euphorbia cactus Ehrenb. ex Boiss	Euphorbiaceae	St	Abyan territory, Yemen	Anti-microbial [dental inf, boils]	Al-Fatimi, 2019
Euphorbia calyptrata Coss. & Kralik	Euphorbiaceae	La	Tassili N'Ajjer, Southern Algerian Sahara	Anti-viral [wart]	Hammiche and Maiza, 2006
Euphorbia dendroides L.	Euphorbiaceae	La	Pollino National Park, Southern Italy	Anti-viral [wart]	Di Sanzo et al., 2013
Euphorbia glyptosperma Engelm.	Euphorbiaceae	Ro, WP	Nuevo León, Mexico	Anti-microbial	Estrada-Castillón et al., 2012
Euphorbia granulata Forssk.	Euphorbiaceae	AP, La	Tassili N'Ajjer, Southern Algerian Sahara	Anti-venom	Hammiche and Maiza, 2006
Euphorbia helioscopia L.	Euphorbiaceae	La	Agro Nocerino Sarnese, Southern Italy	Anti-microbial [wart]	Motti and Motti, 2017
Euphorbia hirta L. Syn: *Chamaesyce hirta* (L.) Millsp.	Euphorbiaceae	La, Le, FL, St, WP	Eastern Highlands, Papua New Guinea; Ehotile people, Côte d'Ivoire; Cataractes and Lukaya districts, D.R. Congo; Martinique; Guinea-Bissau; Maharashtra, India; Kani people, Western Ghats, India; Togo	Anti-microbial [measles, dys, UTI, wart]; anti-convulsive; wound healing; memory loss	Jorim et al., 2012; Malan et al., 2015; Latham and Konda ku Mbuta, 2016; Longuefosse and Nossin, 1996; Romeiras et al., 2012; Wadankar et al., 2011; Ayyanar and Ignacimuthu, 2011; Kantati et al., 2016
Euphorbia hypericifolia L. Syn: *Euphorbia glomerifera*	Euphorbiaceae	NS	Markets of Bogotá, Columbia	anti-microbial [gangr]	Bussmann et al., 2018
Euphorbia kamerunica Pax	Euphorbiaceae	WP	Benin	Hemiplegia	Adjanohoun et al., 1989: cited in Noe and Lehmann, 2012 [H130]
Euphorbia larica Boiss.	Euphorbiaceae	La, St	N + S Oman	Anti-inflammatory [burn]; anti-microbial [boil]	Divakar et al., 2016
Euphorbia mellifera Aiton Syn: *Euphorbia longifolia*	Euphorbiaceae	Ro	Central Himalayas, Nepal	Anti-microbial	Bhattarai et al., 2006
*Euphorbia monocyathium (*Prokh.) Prokh.	Euphorbiaceae	Ro	Turkestan Range of south Kyrgystan	Anti-inflammatory, anti-ulcer	Pawera et al., 2016
Euphorbia pallens Dillwyn	Euphorbiaceae	Le	Andoman + Nicobar Is., India	Anti-paralytic	Chander et al., 2014
Euphorbia prostrata Aiton	Euphorbiaceae	NS	Mauritius	Eye inflammation	Adjanohoun et al., 1983: cited in Noe and Lehmann, 2012 [H001]
Euphorbia retusa Forssk. Syn: *Euphorbia cornuta* Pers	Euphorbiaceae	La	Tassili N'Ajjer, Southern Algerian Sahara	Anti-microbial [wart]	Hammiche and Maiza, 2006
Euphorbia sikkimensis Boiss.Syn: *Euphorbia chrysocoma*	Euphorbiaceae	Ro	Maonan people, Guangxi Zhuang, China	Anti-microbial	Hong et al., 2015
Euphorbia terracina L.	Euphorbiaceae	WP	Saudi Arabia	Anti-paralytic	Al-Shanwani, 1996: cited in Aati et al., 2019
Euphorbia tirucalli L.	Euphorbiaceae	La, sap	Mato Grosso, Brazil; Mabira Forest, Uganda	Anti-microbial [wart]	Ribeiro et al., 2017; Tugume et al., 2016
Euphorbia tithymaloides L. Syn: *Pedilanthus tithymaloides* (L.) A.Poit.	Euphorbiaceae	Le	Markets of Bogotá, Columbia; Rakhain people, Bangladesh	Anti-microbial [STI]; anti-inflammatory [Fe]	Bussmann et al., 2018; Gómez-Estrada et al., 2011; Hanif et al., 2009

Euphorbia trigona Mill.	Euphorbiaceae	Le	Mabira Forest, Uganda	Anti-microbial [yell fev]	Tugume et al., 2016
Eurya sp.	Pentaphylacaceae	Le	Eastern Highlands, Papua New Guinea	Weakness/fatigue	Jorim et al., 2012
Eustephia coccinea Cav.	Amaryllidaceae	Ba	Mestizo community, North Peru	Anti-inflammatory	Bussmann and Sharon, 2006
Evodia lepta Merr. [unresolved]	Rutaceae	Le	Maonan people, Guangxi Zhuang, China	Anti- inflammatory	Hong et al., 2015
Evolvulus alsinoides L.	Convolvulaceae	WP	3 Kerala tribes, Western Ghats, India	Memory improvement	Marjana et al., 2018
Evolvulus nummularius (L) L.	Convolvulaceae	FL, Le, Ro	Kani people, Western Ghats, India	Anti-microbial [dys]	Ayyanar and Ignacimuthu, 2011
Eysenhardtia texana Scheele	Fabaceae	Ba	Nuevo León, Mexico	Anti-microbial	Estrada-Castillón et al., 2012
Fagara davyi Verdoorn [unresolved]	Rutaceae	Ro	Venda, South Africa	Anti-microbial [sore throat, STI]	Arnold and Gulumian, 1984
Fagonia bruguieri DC.	Zygophyllaceae	AP	Tassili N'Ajjer, Southern Algerian Sahara	Anxiolytic	Hammiche and Maiza, 2006
Fagonia paulayana J.Wagner & Vierh. Syn: *Fagonia schweinfurthii* (Hadidi) Nabil & Hadidi	Zygophyllaceae	Ro	Afar people, Rift Valley, Ethiopia	Anti-microbial	Teklehaymanot, 2017
Fagopyrum esculentum Moench	Polygonaceae	Fr	Myanmar	Anti-microbial [diarrh]	DeFilipps and Krupnick, 2018
Faidherbia albida (Delile) A.Chev. Syn: *Acacia albida* Delile	Fabaceae	Ba	Tassili N'Ajjer, Southern Algerian Sahara	Anti-microbial [bronch, cough]	Hammiche and Maiza, 2006
Faurea saligna Mutango Harvey	Proteaceae	Ro, Ba	Venda, South Africa	Anti-microbial [STI, cough]	Arnold and Gulumian, 1984
Ferula communis L.	Apiaceae	Le	Debre Libanos Wereda, central Ethiopia	Anti-hypertensive	Getaneh and, Girma, 2014
Ferula kokanica Regel & Schmalh.	Apiaceae	Sap, St, Br	Turkestan Range of south Kyrgystan	Anti-microbial [influenza, cough, cold, bronch]	Pawera et al., 2016
Fibraurea recisa Pierre	Menispermaceae	Ro	Maonan people, Guangxi Zhuang, China	Anti-inflammatory	Hong et al., 2015
Ficus americana subsp. *guianensis* (Desv. ex Ham.) C.C.Berg Syn*:* *Ficus guianensis*	Moraceae	Ba, Le, sap	Mato Grosso, Brazil	Anti-inflammatory	Ribeiro et al., 2017
Ficus benghalensis L.	Moraceae	Ba, La, Le, Ro, Se	Kani people, Western Ghats, India; Mizoram, India	Wound healing; anti-microbial [dys]; anti-inflammatory [rheum]	Ayyanar and Ignacimuthu, 2011; Rai and Lalramnghinglova, 2010
Ficus carica L.	Moraceae	Fr	Markets of Bogotá, Columbia; Farashband nomadic tribe, Iran; Agro Nocerino Sarnese, Southern Italy	Anti-microbial [wart, bronch, sore throat]; memory improvement	Bussmann et al., 2018; Zali and Tahmasb, 2016; Motti and Motti, 2017
Ficus cordata Thunb.	Moraceae	Sap	N + S Oman; Abyan territory, Yemen	Anti-inflammatory; anti-microbial [boil, dys, skin inf]; wound healing	Divakar et al., 2016; Al-Fatimi, 2019
Ficus cyathistipula Warb.	Moraceae	Le	Mabira Forest, Uganda	Anti-hypertensive	Tugume et al., 2016
Ficus exasperata Vahl.	Moraceae	Le	Sagamu, SW Nigeria	Memory enhancement	Elufioye et al., 2012
Ficus maxima Mill.	Moraceae	Le	Loja and Zamora-Chinchipe, Ecuador	Anti-inflammatory	Tene et al., 2007

Ficus microcarpa L.f.	Moraceae	Le	Maonan people, Guangxi Zhuang, China	Anti-microbial [inf]	Hong et al., 2015
Ficus natalensis Hochst.	Moraceae	Ba	Mabira Forest, Uganda	Anti-microbial [STI]	Tugume et al., 2016
Ficus obliqua G.Forst	Moraceae	Ba	Tonga	Anti-microbial [boil]	Croft and Tu'ipulotu, 1980
Ficus paraensis (Miq.) Miq.	Moraceae	Ba, La	Mato Grosso, Brazil	Anti-inflammatory	Ribeiro et al., 2017
Ficus platyphylla Del.	Moraceae	Le	Benin	Memory improvement/ enhancement	Adjanohoun et al., 1989: cited in Noe and Lehmann, 2012 [H168]
Ficus prostrata (Wall. ex Miq.) Buch.-Ham. ex Miq.	Moraceae	Ro	Mizoram, India	Anti-venom	Rai and Lalramnghinglova, 2010
Ficus pumila L.	Moraceae	Le	Eastern Highlands, Papua New Guinea	Anti-inflammatory [Fe]	Jorim et al., 2012
Ficus religiosa L.	Moraceae	Ba, Fr, Le, St	Maharashtra, India; Mizoram, India	Wound healing; anti-microbial [STI, dys]	Wadankar et al., 2011; Rai and Lalramnghinglova, 2010
Ficus septica Burm.f.	Moraceae	Le	Maranao people, Philippines	Numbness	Malawani et al., 2017
Ficus sp.	Moraceae	Le	Eastern Highlands, Papua New Guinea; Sao Tome; Dayaknese Communities, W Kalimantan, Indonesia	Anti-inflammatory [Fe, headache]; *Ficus kamerunensis* eye inflammation	Jorim et al., 2012; Sequeira, 1994: cited in Noe and Lehmann, 2012 [H001]; Diba et al., 2013
Ficus sur Forssk. Syn: *Ficus capensis* Thunb.	Moraceae	Ro, Le	Guinea-Bissau	Anti-epileptic	Romeiras et al., 2012
Ficus thonningii Blume	Moraceae	Fr, Le	Mali; Cataractes and Lukaya districts, D.R. Congo	Anti-paralytic; anti-microbial [cough, abscess]	Malgras, 1992: cited in Noe and Lehmann, 2012 [H130]; Latham and Konda ku Mbuta, 2016
Ficus vasta Forssk	Moraceae	Fr	Abyan territory, Yemen	Boost immune system; wound healing	Al-Fatimi, 2019
Flaveria bidentis (L.) Kuntze	Asteraceae	FL, Le, St	Mestizo community, North Peru	Anti-microbial	Bussmann and Sharon, 2006
Fleroya stipulosa (DC.) Y.F.Deng Syn: *Hallea stipulosa* (DC.) J.-F.Leroy	Rubiaceae	Ba	Cataractes and Lukaya districts, D.R. Congo	Anti-microbial [abscess], anti-inflammatory [Fe, rheum]	Latham and Konda ku Mbuta, 2016
Flourensia cernua DC.	Asteraceae	Le, St	Nuevo León, Mexico	Anti-microbial	Estrada-Castillón et al., 2012
Flueggea virosa (Roxb. ex Willd.) Voigt Syn: *Securinega virosa* (Roxb. ex Willd.) Baill.	Phyllanthaceae	Le, Ro, Tw	Benin; Zimbabwe; South Nigeria	Memory improvement/ enhancement; anti-microbial [STI, measles, dys, conj]; anti-inflammatory [headache, rheum]	Adjanohoun et al., 1989: cited in Noe and Lehmann, 2012 [H168]; Borokini et al., 2012
Foeniculum vulgare Mill.	Apiaceae	Ro	Markets of Bogotá, Columbia; Jazan Region, Saudi Arabia	Anti-inflammatory [Fe]; neurological disorders	Bussmann et al., 2018; Tounekti et al., 2019
Forsythia suspensa Thunb.) Vahl.	Oleaceae	Fr	Shaanxi, China	Anti-aging; anti-inflammatory [Fe]	Teng et al., 2011
Fragaria nubicola (Lindl. ex Hook.f.) Lacaita	Rosaceae	Fr, Ro	Himachal Pradesh, NW Himalaya, India; Rajouri ,Jammu and Kashmir, India	Anti-neuroinflammation; inflammation [Fe]; memory improvement	Vidyarthi et al., 2013; Dangwal and Singh, 2013
Fraxinus angustifolia Vahl Syn: *Fraxinus rotundifolia*	Oleaceae	Le, Se	Iran	Anti-microbial [cough]	Mosaddegh et al., 2013
Fraxinus chinensis Roxb.	Oleaceae	Ba	Shaanxi, China	Anti-microbial	Teng et al., 2011

Fuchsia canescens Benth	Onagraceae	Le, FL	Loja and Zamora-Chinchipe, Ecuador	Anti-hypertensive	Tene et al., 2007
Fuchsia hybrida hort. ex Siebert & Voss	Onagraceae	Le	Loja and Zamora-Chinchipe, Ecuador	Anti-hypertensive	Tene et al., 2007
Fuchsia magellanica Lam. (ind)	Onagraceae	Le	Mapuche people, Chile	Anti-hypertensive	Houghton and Manby, 1985
Fuerstia africana T. C. E. Fr.	Lamiaceae	Le	Kakamega County, Kenya	Anti-microbial [mouth inf]	Odongo et al., 2018
Fumaria officinalis L	Papaveraceae	AP	Edremit Gulf, Turkey	Anti-hypertensive	Polat and Satıl, 2012
Fumaria parviflora Lam.	Papaveraceae	AP, Le, St	Iran; Saudi Arabia	Anti-inflammatory [Fe]; anti-hypertensive; anti-viral	Mosaddegh et al., 2013
Furcraea cabuya Trel.	Asparagaceae	NS	Markets of Bogotá, Columbia	Anti-microbial [UTI, influenza, conj]	Bussmann et al., 2018
Furcraea foetida (L.) Haw.	Asparagaceae	Le	Maromizaha forest, Madagascar	Anti-microbial	Riondato et al., 2019
Furcraea macrophylla Baker, Hook	Asparagaceae	Le, Ro	Columbian Andes	Anti-inflammatory [rheum]	Cadena-González et al., 2013
Gaertnera paniculata Benth.	Rubiaceae	Ba, Le	D.C. Congo	Anti-microbial [cough]; anti-inflammatory [Fe]	Latham and Konda ku Mbuta, 2016
Gaiadendron punctatum (Ruiz & Pav.) G.Don	Loranthaceae	FL, Le	Loja and Zamora-Chinchipe, Ecuador	Anti-microbial [influenza]	Tene et al., 2007
Galenia africana L.	Aizoaceae		South Africa	Eye inflammation	Van Wyk, 2008: cited in Noe and Lehmann, 2012 [H001]
Galium verum L.	Rubiaceae	AP	Albanians, Bosniaks/Gorani + Turkish ethnic groups, Kosovo	Cell regeneration	Mustafa et al., 2015
Galinsoga parviflora Cav.	Asteraceae	Le, WP	Loja and Zamora-Chinchipe, Ecuador; Kibale, Uganda	Anti-inflammatory [Fe]); memory enhancement	Tene et al., 2007; Namukobe et al., 2011: cited in Noe and Lehmann, 2012 [H168]
Gamochaeta americana (Mill.) Wedd.	Asteraceae	NS	Columbia	Anti-microbial [UTI]	Bussmann et al., 2018
Ganophullum falcatum Blume	Sapindaceae	Le	Andoman + Nicobar Is., India	Anti-hypertensive	Chander et al., 2014
Garcinia cowa Roxb. ex Choisy	Clusiaceae	Ba	Mizoram, India	Anti-microbial [leprosy, dys]	Rai and Lalramnghinglova, 2010
Garcinia mangostana L.	Clusiaceae	Fr, Le	Seberida, Riau Province, Sumatra, Indonesia; Cataractes and Lukaya districts, D.R. Congo	Anti-inflammatory [Fe]; anti-microbial [dys]	Mahyar et al., 1991; Latham and Konda ku Mbuta, 2016
Garcinia gummi-gutta (L.) Roxb.	Clusiaceae	Fr, Le, Se	3 Kerala tribes, Western Ghats, India	Anti-microbial [ton'is]	Marjana et al., 2018
Garcinia huillensis Welw.	Clusiaceae	Ba, Ro	Bas-Congo	Anti-microbial [throat inf, sore, TB]; anti-inflammatory [pain]	Latham and Konda ku Mbuta, 2016
Garcinia kola Heckel.	Clusiaceae	Fr, Ro ba	Sagamu, SW Nigeria; Fundong, NW Cameroon	Anti-aging; Anti-microbial [TB]; anti-inflammatory [asthma]	Elufioye et al., 2012; Focho et al.,2009
Garcinia pedunculata Roxb. ex Buch.-Ham.	Clusiaceae	Fr	Mizoram, India	Anti-microbial [dys]	Rai and Lalramnghinglova, 2010
Garcinia sopsopia (Buch.-Ham.) Mabb.	Clusiaceae	Br	Mizoram, India	Anti-venom	Rai and Lalramnghinglova, 2010

Garcinia xanthochymus Hook.f.	Clusiaceae	Ba	Myanmar	Anti-microbial [dys, diarrh]	DeFilipps and Krupnick, 2018
Gardenia jasminoides J.Ellis Syn: *Gardenia augusta* (L.) Merr.	Rubiaceae	FL, Fr, Le, St	Maonan people, Guangxi Zhuang, China; Mestizo community, North Peru	Anti-inflammatory	Hong et al., 2015; Bussmann and Sharon, 2006
Gardenia taitensis DC.	Rubiaceae	Ba	Tonga; Maori people, Cook Islands	Anti-inflammatory [headache]	Croft and Tu'ipulotu, 1980; Whistler, 1985
Gardenia ternifolia subsp. *jovis-tonantis* (Welw.) Verdc.	Rubiaceae	Ba, Ro	Cataractes and Lukaya districts, D.R. Congo; Mana Angetu District, SE Ethiopia; Togo	Anti-microbial [dys, caries]; anti-inflammatory [rheum, headache]; anti-epileptic; anti-PD	Latham and Konda ku Mbuta, 2016; Lulekal et al., 2008; Kantati et al., 2016
Genipa sp.	Rubiaceae	Ba	Mato Grosso, Brazil	Anti-microbial	Ribeiro et al., 2017
*Gentiana macrophyll*a Pall.	Gentianaceae	Ro	Shaanxi, China	Anti-fungal	Teng et al., 2011
Gentianella bruneotricha (Gilg.) J.S. Pringle.	Gentianaceae	WP	Mestizo community, North Peru	Anti-microbial	Bussmann and Sharon, 2006
Gentianella graminea (H.B.K.) Fabris	Gentianaceae	WP	Mestizo community, North Peru	Anti-inflammatory	Bussmann and Sharon, 2006
*Gentianella multicaulis (*Gillies ex Griseb.) Fabris Syn: *Gentianella achalensis* (Hieron. ex Gilg.) T.N. Ho & S.W. Liu	Gentianaceae	Ro	Comechingones, Argentina	Anti-inflammatory [Fe]	Goleniowski et al., 2006
Geranium core-core Steud. (ind)	Geraniaceae	Ju	Mapuche people, Chile	Anti-inflammatory [Fe]	Houghton and Manby, 1985
Geranium rotundifolium L.	Geraniaceae	AP	Iran	Anti-viral [cold]	Mosaddegh et al., 2013
Geranium sesiliflorum Cavanilles	Geraniaceae	WP	Mestizo community, North Peru	Anti-inflammatory	Bussmann and Sharon, 2006
Gerbera gossypina (Royle) Beauverd	Compositae	AP	Toli Peer National Park, Kashmir, Pakistan	Nerve disorders	Amjad et al., 2017
Gerbera piloselloides (L.) Cass.	Compositae	WP	Maonan people, Guangxi Zhuang, China	Anti-inflammatory	Hong et al., 2015
Gilbertiodendron dewevrei (De Wild.) J.Léonard	Fabaceae	St ba	Baka Pygmies, Gabon	Anti-microbial; fatigue	Betti et al., 2013
Gladiolus italicus Mill	Iridaceae	Fr, Ro	Iran	Anti-microbial	Mosaddegh et al., 2013
Gliricidia sepium (Jacq.) Walp.	Fabaceae	Fr Le	Ayta communities, Bataan, Philippines; Martinique	Anti-microbial [skin inf]; anti-hypertensive	Tantengco et al., 2018; Longuefosse and Nossin, 1996
Globularia alypum L	Plantaginaceae	AP	Tassili N'Ajjer, Southern Algerian Sahara	Anti-fungal; anti-inflammatory [Fe]	Hammiche and Maiza, 2006
Glochidion oblatum Hook.f.	Phyllanthaceae	Ro, St	Mao Naga people Manipur, India	Anti-microbial [dys]	Lokho, 2012
Glycine max (L.) Merr.	Fabaceae	Se	Myanmar	Anti-inflammatory [Fe]	DeFilipps and Krupnick, 2018
Glycyrrhiza glabra L.	Fabaceae	Ro	3 Kerala tribes, Western Ghats, India	Anti-microbial [wart]	Marjana et al., 2018
Glycyrrhiza uralensis Fisch. ex DC.	Fabaceae	AP, Ro	Shaanxi, China;	Anti-viral [HIV]	Teng et al., 2011
Glyphaea brevis (Spren) Monochino	Malvaceae]	Le, Fr, Ro, Se	South Nigeria; Cataractes and Lukaya districts, D.R. Congo	Anti-inflammatory [Fe, eye inf]; anti-microbial [dys, STI, conj, cough]; anti-paralytic/anti-convulsant	Borokini et al., 2012; Latham and Konda ku Mbuta, 2016

Gnaphalium elegans Kunth	Asteraceae	NS	Markets of Bogotá, Columbia	Anti-inflammatory; anti-microbial [UTI]	Bussmann et al., 2018
Gnetum leptostachyum Blume	Gnetaceae	Le	Orang Asli tribe, Malaysia	Anti-inflammatory [Fe]; anti-microbial [influenza]	Samuel et al., 2010
Gomphrena globosa L.	Amaranthaceae	FL	Maonan people, Guangxi Zhuang, China; Martinique	Anti-microbial [TB]	Hong et al., 2015; Longuefosse and Nossin, 1996
Gomphrena perennis L.	Amaranthaceae	FL	Comechingones, Argentina	Anti-inflammatory [Fe]	Goleniowski et al., 2006
Gomphrena serrata L.	Amaranthaceae	NS	Markets of Bogotá, Columbia	Anti-inflammatory [Fe]; anti-microbial [bronch, pneum]	Bussmann et al., 2018
Gossypium barbadense L.	Malvaceae	Ba, Fr, Le, Ro, Se [In comb]	Martinique; Mato Grosso, Brazil; Cataractes and Lukaya districts, D.R. Congo	Anti-inflammatory [Fe, rheum]; anti-microbial/anti-paralytic [polio]; anxiolytic	Longuefosse and Nossin, 1996; Ribeiro et al., 2017; Latham and Konda ku Mbuta, 2016; Kembelo, 2003: cited in Noe and Lehmann, 2012 [H130]; Quinlan, 2010
Gossypium hirsutum L.	Malvaceae	NS	Markets of Bogotá, Columbia	Anti-inflammatory	Bussmann et al., 2018
Gossypium spp.	Malvaceae	NS	Burkina Faso	Eye inflammation	de la Pradilla et al., 1988: cited in Noe and Lehmann, 2012 [H001]
Goupia glabra Aubl.	Goupiaceae	NS	Rio Jauaperi, Brazilian Amazon	Anti-microbial [dys]	Pedrollo et al., 2016
Graptophyllum pictum (L.) Griff.	Acanthaceae	Le	Sundanese community, West Java	Anti-inflammatory	Roosita et al., 2008
Greenwayodendron suaveolens (Engl. & Diels) Verdc. Syn: *Polyalthia suaveolens*	Annonaceae	Le	Baka Pygmies, Gabon	Anti-microbial [influenza]	Betti et al., 2013
Grewia erythraea Schweinf	Malvaceae	Ba, Le, Ro	Afar people, Rift Valley, Ethiopia	Anti-microbial [leprosy, typhoid]	Teklehaymanot, 2017
Grewia retusifolia Kurz	Malvaceae	Ro	Native Northern territory, Australia	Anti-inflammatory [Fe]	Smith, 1991
Grindelia inuloides Willd.	Compositae	Le, FL	Nuevo León, Mexico	Anti-inflammatory	Estrada-Castillón et al., 2012
Grindelia pulchella Dunal	Compositae	Le, St	Comechingones, Argentina	Anti-inflammatory [Fe]	Goleniowski et al., 2006
Guarea pubescens (Rich.) A.Juss.	Meliaceae	St Ba	Rio Jauaperi, Brazilian Amazon	Anti-microbial	Pedrollo et al., 2016
Guazuma ulmifolia Lam.	Malvaceae	Fr, Le	Loja and Zamora-Chinchipe, Ecuador; Bolicar, N Colombia	Anti-microbial [influenza]; anti-inflammatory [pain]	Tene et al., 2007; Gómez-Estrada et al., 2011
Guiera senegalensis J.F.Gmel.	Combretaceae	Le	Guinea-Bissau	Anti-depressant	Romeiras et al., 2012
Guilandina bonduc L. Syns: *Caesalpinia bonducella* L. Fleming; *C. bonduc* (L.) Roxb.	Fabaceae	Le	Andoman + Nicobar Is., India	Anti-paralytic; anti-inflammatory [Fe]; anti-hypertensive	Chander et al., 2014
Gunnera tinctoria (Molina) Mirb. Syn: *Gunnera chilensis* Lam.	Gunneraceae	Ro	Mapuche people, Chile	Anti-microbial [dys]	Houghton and Manby, 1985

Gymnema sylvestre (Retz.) R.Br. ex Sm.	Apocynaceae	Le	Kani people, Western Ghats, India; Eastern Ghats, India	Anti-venom; anti-microbial [boil]	Ayyanar and Ignacimuthu, 2011; Rao et al., 2006
Gymnosporia senegalensis (Lam.) Loes Syn: *Maytenus senegalensis* (Lam.) Exell	Celastraceae	St	Tigrigna people, Central Eritrea	Anti-inflammatory [rheum]	Yemane et al., 2017
Gynerium sagittatum (Aubl.) P.Beauv.	Poaceae	Le, Bd	Bolivian Amazon	Wound	Bourdy et al., 2000
Gynostemma pentaphyllum (Thunb.) Makino	Cucurbitaceae	WP	Shaanxi, China	Anti-inflammatory; support immune system, anti-hypertensive	Teng et al., 2011
Gyrocarpus americanus Jacq.	Hernandiaceae	Le	Andoman + Nicobar Is., India	Anti-inflammatory [Fe]	Chander et al., 2014
Gynura procumbes Merr.	Asteraceae/Compositae	Le	Dayaknese Communities, W Kalimantan, Indonesia	Anti-inflammatory [headache]	Diba et al., 2013
Gynura scandens O.Hoffm.	Asteraceae/Compositae	Le	Haya people, Kagera, NW Tanzania	Anti-convulsant; anti-inflammatory	Moshi et al., 2009
Habenaria sp.	Orchidaceae	Rh	Chin people, Myanmar	Anti-microbial [boil]	Ong et al., 2018
Hancornia speciosa Gomes	Apocynaceae	Ba, Fr, La, Le	Mato Grosso, Brazil	Anti-microbial; anti-hypertensive	Ribeiro et al., 2017
Handroanthus impetiginosus (Mart. ex DC.) Mattos Syn: *Tabebuia avellanedae* Lorentz ex Griseb	Bignoniaceae	Ba, Ro, St	Mato Grosso, Brazil	Anti-microbial [leprosy, UTI]; anti-inflammatory [rheum]	Ribeiro et al., 2017
Haplophyllum tuberculatum Juss. Syn: *Ruta tuberculatum* Forsk.	Rutaceae	AP	Tassili N'Ajjer, Southern Algerian Sahara	Anti-inflammatory [rheum, Fe, headache]	Hammiche and Maiza, 2006
Harpullia sp.	Sapindaceae	Ba, Le	Bougainville, Solomon Islands	Paralysis, migraine, asthma	Waruruai et al., 2011
Harungana madagascariensis Lam. ex Poir.	Hypericaceae	Ba, Le, St	Maromizaha forest, Madagascar; Mabira Forest, Uganda; 3 states in SW Nigeria	Anti-inflammatory [Fe, eye inflam]; anti-microbial; memory enhancement	Riondato et al., 2019; Tugume et al., 2016; Babawale et al., 2016
Hedychium spicatum Sm.	Zingiberaceae	Rh	Mizoram, India	Anti-inflammatory; anxiolytic	Rai and Lalramnghinglova, 2010
Heinsia crinita (Wennberg) G.Taylor	Rubiaceae	Ro	Cataractes and Lukaya districts, D.R. Congo	Anti-inflammatory [periodontitis, rheum]; anti-microbial [TB, pneum]	Latham and Konda ku Mbuta, 2016
Helianthus annuus L.	Asteraceae	Se	Mato Grosso, Brazil; Hakka people, Guangdong, China; Mizoram, India	Anti-microbial [bronch, pneum, RTI]; anti-hypertensive	Ribeiro et al., 2017; Au et al., 2008; Rai and Lalramnghinglova, 2010
Helichrysum krausii Sch. Bip.	Asteraceae	Ro	Venda, South Africa	Anti-microbial [STI]	Arnold and Gulumian, 1984
Helichrysum mechowianum Klatt Syn: *Helichrysum ceres* S.Moore	Asteraceae	Le	Cataractes and Lukaya districts, D.R. Congo; Burundi	Anti-microbial [skin inf]; anti-inflammatory [headache, rheum, eye inflammation]	Latham and Konda ku Mbuta, 2016; Baerts and Lehmann, 1989: cited in Noe and Lehmann, 2012 [H001]
Helichrysum schimperi (Sch.Bip. ex A.Rich.) Moeser	Asteraceae	Le, St	Burundi	Eye inflammation	Baerts and Lehmann, 1989: cited in Noe and Lehmann, 2012 [H001]
Heliconia psittacorum L. f.	Heliconiaceae	NS	Markets of Bogotá, Columbia	Muscular paralysis	Bussmann et al., 2018

Helicteres isora L.	Malvaceae	Fr, Ro ba	3 Kerala tribes, Western Ghats, India; Chittagong Hill Tracts, Bangladesh; Eastern Ghats, India	Anti-microbial [dys]; anti-venom; Anti-inflammatory	Marjana et al., 2018; Kadir et al., 2015; Rao et al., 2006
Helinus integrifolius (Lam.) Kuntze	Rhamnaceae	Ro ba	Samburu, Kenya	Anti-inflammatory [rheum], anti-paralytic	Nanyingi et al., 2008
Heliotropium bacciferum Forssk.	Boraginaceae	Le	Tassili N'Ajjer, Southern Algerian Sahara	Anti-microbial [tons, boil]	Hammiche and Maiza, 2006
Heliotropium cinerascens Steud. ex DC. [unresolved]	Boraginaceae	Le	Afar people, Rift Valley, Ethiopia	Anti-microbial	Teklehaymanot, 2017
Heliotropium indicum L.	Boraginaceae	Le	Carib people, Livingston, Guatamala; Mato Grosso, Brazil; Madagascar; Togo	Anti-inflammatory; anti-microbial [wart, bronch, pneum]; paralysis/ hemiplegia/ polio/ paraplegia; dementia, memory loss	Girón et al., 1991; Ribeiro et al., 2017; Pernet and Meyer, 1957: cited in Noe and Lehmann, 2012 [H130]; Kantati et al., 2016
Heracleum sphondylium subsp. *montanum* (Schleich.ex Gaudin) Briq.	Apiaceae	Ro	Turkestan Range of south Kyrgystan	Anti-hypertensive	Pawera et al., 2016
Heracleum cachemiricum C.B. Clarke	Apiaceae	Ju	Toli Peer National Park, Kashmir, Pakistan	Nerve disorders	Amjad et al., 2017
Hernandia ovigera L.	Hernandiaceae	Le	Andoman + Nicobar Is., India	Anti-paralytic	Chander et al., 2014
Heteropterys obovata (Small) Cuatrec. & Croat	Malpighiaceae	St	Guaymi indians, Panama	Anti-inflammatory [Fe]	Joly et al., 1990
Heteropterys tomentosa A.Juss.	Malpighiaceae	Ro	Mato Grosso, Brazil	Memory improvement	Ribeiro et al., 2017
Heterotis rotundifolia (Sm.) Jacq.-Fél. Syn: *Dissotis rotundifolia* (Sm.) Triana	Melastomataceae	Ro, Le	Kimboza forest, Tanzania	Anti-microbial [STI]	Amri and Kisangau, 2012
Hibiscus acetosella Welw. ex Hiern	Malvaceae	Le	Cataractes and Lukaya districts, D.R. Congo	Anti-inflammatory [Fe]	Latham and Konda ku Mbuta, 2016
Hibiscus cannabinus L	Malvaceae	FL, Le, Ro, Se	Cataractes and Lukaya districts, D.R. Congo	Anti-microbial [conj, cough, STI, tetanus]	Latham and Konda ku Mbuta, 2016
Hibiscus fuscus Garcke	Malvaceae	Le	Haya people, Kagera, NW Tanzania	Anti-microbial [polio]	Moshi et al., 2009
Hibiscus lunariifolius Willd	Malvaceae	Le	South Nigeria	Anti-microbial/anti-inflammatory [typhoid Fe]	Borokini et al., 2012
Hibiscus rosa-sinensis L.	Malvaceae	FL, Le	Martinique; Trinidad; Ayta communities, Bataan, Philippines; Banglasesh; Dayaknese Communities, W Kalimantan, Indonesia; Kani people, Western Ghats, India	Anti-microbial [boil, STI]; anti-inflammatory [Fe, headache]	Longuefosse and Nossin, 1996; Tantengco et al., 2018; Clement et al., 2015; Hanif et al., 2009; Diba et al., 2013; Ayyanar and Ignacimuthu, 2011
Hibiscus sabdariffa L.	Malvaceae	FL	Congo-Brazzaville	Limb weakness, anti-inflammatory	Diafouka, 1997: cited in Noe and Lehmann, 2012 [H130]
Hibiscus tiliaceus L.	Malvaceae	FL, Le	Andoman + Nicobar Is., India	Anti-inflammatory [Fe]; anti-microbial [boil]; wound healing	Chander et al., 2014

Himatanthus drasticus (Mart.) Plumel	Apocynaceae	La	Rio Jauaperi, Brazilian Amazon	Anti-microbial [TB]	Pedrollo et al., 2016
Himatanthus obovatus (Müll.Arg.) Woodson	Apocynaceae	Le	Mato Grosso, Brazil	Anti-inflammatory; anti-microbial	Ribeiro et al., 2017
Himatanthus sucuuba (Spruce ex Müll.Arg.) Woodson	Apocynaceae	La	Bolivian Amazon	Anti-microbial; wound healing	Bourdy et al., 2000
Hippocratea myriantha Oliv.	Celastraceae	NS	Cataractes and Lukaya districts, D.R. Congo	Anti-inflammatory [headache, rheum]; anti-microbial [dys]	Latham and Konda ku Mbuta, 2016
Hippophae rhamnoides L.	Elaeagnaceae	FL, Le, St	Western Ladakh, India	Memory improvement, energy boosting	Angmo et al., 2012
Hippophae salicifolia D.Don	Elaeagnaceae	Fr	Central Himalayas, Nepal	Anti-microbial	Bhattarai et al., 2006
Hippophae tibetana Schltdl.	Elaeagnaceae	Fr	Central Himalayas, Nepal	Anti-microbial	Bhattarai et al., 2006
Hippophae turkestanica (Rousi) Tzvelev	Elaeagnaceae	Fr	Turkestan Range of south Kyrgystan	Anti-microbial [g-I infs]	Pawera et al., 2016
Holoptelea integrifolia (Roxb.) Planch.	Ulmaceae	Ba, Le	Karnataka, India; Andoman + Nicobar Is., India	Anti-microbial *[Herpes*]; anti-paralytic	Rajakumar and Shivanna, 2009; Chander et al., 2014
Hordeum vulgare L.	Poaceae	Se	Martinique	Anti-inflammatory; anti-microbial	Longuefosse and Nossin, 1996
Horkelia cuneata Lindl. Syn: *Horkelia californica* Cham. & Schltdl.	Rosaceae	NS In s. comb	Chumash Indians, California, USA	Anti-inflammatory [Fe]; anti-microbial [cold]	Timbrook, 1990
Hoya parasitica Wall. ex Traill [unresolved]	Asclepiadaceae	Le, Me, Ro	Rakhain people, Bangladesh	Anti-paralytic	Hanif et al., 2009
Humata heterophylla (Sm.) Desv. Syn: *Davallia heterophylla* Sm.	Davalliaceae	Le	Dayaknese Communities, W Kalimantan, Indonesia	Anti-inflammatory [Fe]	Diba et al., 2013
Hydnora abyssinica A.Br.	Aristolochiaceae	Fr	Abyan territory, Yemen	Boost immune system	Al-Fatimi, 2019
Hydrocotyle globiflora R. & P.	Araliaceae	WP	Mestizo community, North Peru	Anti-inflammatory	Bussmann and Sharon, 2006
Hygrophila auriculata (Schumach.) Heine	Acanthaceae	Le	Kani people, Western Ghats, India	Anti-microbial [cough]; anti-inflammatory [rheum]	Ayyanar and Ignacimuthu, 2011
Hymenaea courbaril L.	Fabaceae	Ba	Mato Grosso, Brazil	Anti-inflammatory; anxiolytic	Ribeiro et al., 2017
Hymenaea martiana Hayne	Fabaceae	St ba, Se	Três Ladeiras people, Atlantic Forest, Brazil	Anti-inflammatory	Gazzaneo et al., 2005
Hymenaea parvifolia Huber	Fabaceae	St Ba	Rio Jauaperi, Brazilian Amazon	Anti-microbial	Pedrollo et al., 2016
Hymenaea stigonocarpa Mart. ex Hayne	Fabaceae	Ba, Fr, Re, Ro, sap	Mato Grosso, Brazil	Anti-microbial; anti-inflammatory	Ribeiro et al., 2017
Hymenocardia acida Tul.	Phyllanthaceae	Fr, Ro, Sh	Togo; Cataractes and Lukaya districts, D.R. Congo; Ivory Coast	Memory improvement/ enhancement; anti-microbial [diarrh, cold, cough]; eye inflammation	Adjanohoun et al., 1986: cited in Noe and Lehmann, 2012 [H168]; Latham and Konda ku Mbuta, 2016; Adjanohoun and Ake Assi,

					1979: cited in Noe and Lehmann, 2012 [H001]
Hymenocardia ulmoides Oliv.	Phyllanthaceae	Ba	Cataractes and Lukaya districts, D.R. Congo	Anti-microbial [sore throat, diarrh, cough, urogenital inf]; anti-inflammatory [Fe, rheum]	Latham and Konda ku Mbuta, 2016
Hypericum perforatum L.	Hypericaceae	AP	Turkestan Range of south Kyrgystan	Anti-inflammatory [headache]; anti-hypertensive	Pawera et al., 2016
Hyptis suaveolens (L.) Poit.	Lamiaceae	Le	Trinidad; Cataractes and Lukaya districts, D.R. Congo; Chittagong Hill Tracts, Bangladesh	Anti-inflammatory [Fe, rheum]; anti-microbial [cold, catarrh, skin inf]; anti-venom	Clement et al., 2015; Latham and Konda ku Mbuta, 2016; Kadir et al., 2015
Hyptis verticillata Jacq.	Lamiaceae	Le	Martinique	Anti-inflammatory [Fe]	Longuefosse and Nossin, 1996
Illicium verum Hook. f.	Schisandraceae	Fr	3 Kerala tribes, Western Ghats, India; Martinique	Anti-microbial [bronc]; anti-inflammatory	Marjana et al., 2018; Longuefosse and Nossin, 1996
Impatiens balsamina L.	Balsaminaceae	FL, Le, WP	Hakka people, Guangdong, China; Maranao people, Philippines. Loja and Zamora-Chinchipe, Ecuador; Cataractes and Lukaya districts, D.R. Congo	Anti-microbial; anti-inflammatory	Au et al., 2008; Tene et al., 2007; Malawani et al., 2017; Latham and Konda ku Mbuta, 2016
Impatiens stuhlmannii Warb.	Balsaminaceae	Le	Rwanda	Paralysis/ hemiplegia/ polio/ paraplegia	Van Puyvelde, 1977: cited in Noe and Lehmann, 2012 [H130]
Imperata contracta (Humb., Bonpl. & Kunth) Hitchc.	Poaceae	NS	Markets of Bogotá, Columbia	Anti-microbial [UTI]	Bussmann et al., 2018
Imperata cylindrica (L.) P.Beauv.	Poaceae	Le, Ro, WP	Orang Asli tribe, Malaysia; Maromizaha forest, Madagascar; Ayta communities, Bataan, Philippines; Sundanese community, West Java; Dayaknese Communities, W Kalimantan, Indonesia	Anti-microbial [yell fev, TB]; anti-hypertensive; anti-inflammatory [Fe]	Samuel et al., 2010; Riondato et al., 2019; Tantengco et al., 2018; Roosita et al., 2008; Diba et al., 2013
Indigofera arrecta Hochst. ex A. Rich.	Fabaceae	Ro	Venda, South Africa; Burundi	Anti-microbial [TB]; eye inflammation	Arnold and Gulumian, 1984; Baerts and Lehmann, 1989: cited in Noe and Lehmann, 2012 [H001]
Indigofera oblongifolia Forssk	Fabaceae	Le	Afar people, Rift Valley, Ethiopia	Anti-microbial [diphth, lung inf, dys, *H. zoster*]	Teklehaymanot, 2017
Indigofera spinosa Forssk	Fabaceae	Le	Abyan territory, Yemen	Anti-microbial [UTI]; wound healing	Al-Fatimi, 2019
Indigofera suffruticosa Mill.	Fabaceae	NS	Markets of Bogotá, Columbia	Anti-microbial [STI]	Bussmann et al., 2018
Inga densiflora Benth.	Fabaceae	NS	Markets of Bogotá, Columbia	Anti-microbial [dys]	Bussmann et al., 2018
Inga ruiziana G. Don.	Fabaceae	Ba	Peruvian Amazon	Anti-fungal	Odonne et al., 2013
Inga ynga (Vell.) J.W. Moore	Fabaceae	NS	Markets of Bogotá, Columbia	Anti-microbial [dys]; anti-inflammatory	Bussmann et al., 2018

Inula japonica Thunb.	Asteraceae	Ca	Shaanxi, China	Anti-microbial	Teng et al., 2011
Inula orientalis Lam.	Asteraceae	Ro	Turkestan Range of South Kyrgystan	Anti-microbial [cough, cold, inf]	Pawera et al., 2016
Ipomoea batatas (L.) Lam.	Convolvulaceae	Tu	Mabira Forest, Uganda; Mato Grosso, Brazil; Philippines	Boost energy; memory Improvement; anti-microbial; anti-hypertensive	Tugume et al., 2016; Ribeiro et al., 2017; Tantengco et al., 2018
Ipomoea cairica (L.) Sweet	Convolvulaceae	Le	Kakamega County, Kenya	Anti-microbial [cold, measles]; anti-inflammatory	Odongo et al., 2018
Ipomoea gracilis R.Br. [unresolved]	Convolvulaceae	Le	Andoman + Nicobar Is., India	Anti-inflammatory [Fe]	Chander et al., 2014
Ipomoea mauritania Jacq.	Convolvulaceae	Le [in sp. comb]	Sagamu, SW Nigeria	Anti-aging	Elufioye et al., 2012
Ipomoea obscura (L.) Ker Gawl.	Convolvulaceae	Le	Andoman + Nicobar Is., India	Anti-inflammatory [Fe]	Chander et al., 2014
Ipomoea pes-caprae (L.) R.Br.	Convolvulaceae	Le	Andoman + Nicobar Is., India	Anti-paralytic; wound healing; anti-microbial [dys]	Chander et al., 2014
Iresine diffusa H.B.K. ex Willd. Syn: *Iresine celosia* L.	Amaranthaceae	Le, WP	Mestizo community, North Peru; Bolivian Amazon	Anti-inflammatory; leg paralysis	Bussmann and Sharon, 2006; Bourdy et al., 2000
Iresine herbstii Lindley	Amaranthaceae	Le	Mestizo community, North Peru	Anti-inflammatory	Bussmann and Sharon, 2006
Isatis raphanifolia Boiss.	Brassicaceae	Se	Iran	Anti-viral [cold]	Mosaddegh et al., 2013
Isatis tinctoria L. Syn: *Isatis indigotica* Fortune ex Lindl.	Brassicaceae	Ro	Shaanxi, China	Anti-viral [influenza]	Teng et al., 2011
Isodon coesta (Buch.- Ham. ex D. Don)	Lamiaceae	Le, Ro	Magar and Majhi people, Nepal	Anti-inflammatory [Fe]; anti-microbial [boil]	Malla et al., 2015
Isodon ternifolius (D.Don) Kudô Syn: *Rabdosia ternifolia*	Lamiaceae	Le, WP	Maonan people, Guangxi Zhuang, China	Anti-microbial	Hong et al., 2015
Ixora nigricans R.Br. ex Wight & Arn	Rubiaceae	Le	Mizoram, India	Anti-microbial [dys]	Rai and Lalramnghinglova, 2010
Ixora pavetta Andrews	Rubiaceae	Le	Eastern Ghats, India	Anti-inflammatory [headache]	Rao et al., 2006
Jacaranda caucana Pittier	Bignoniaceae	Ba, Le	Bogotá markets, Columbia; Columbian Andes; Mato Grosso, Brazil	Anti-microbial [mouth + skin inf, STI, UTI, dengue]	Bussmann et al., 2018; Cadena-González et al., 2013; Ribeiro et al., 2017
Jacaranda copaia (Aubl.) D. Don.	Bignoniaceae	Ba, Le, Ro	Mato Grosso, Brazil	Anti-inflammatory [rheum]; anti-microbial [leprosy, STI]	Ribeiro et al., 2017
Jacaranda cuspidifolia Mart.	Bignoniaceae	Le, Ro	Mato Grosso, Brazil	Anti-microbial [general inf, STI]	Ribeiro et al., 2017
Jasminum grandiflorum L.	Oleaceae	WP	India	Anti-paralytic	Mikawlrawng et al., 2017
Jasminum multiflorum (Burm.f.) Andrews	Oleaceae	Le	Martinique	Anti-microbial [eye inf]	Longuefosse and Nossin, 1996
Jasminum nervosum Lour.	Oleaceae	Le	Mizoram, India	Anti-inflammatory [Fe]	Rai and Lalramnghinglova, 2010
Jasminum officinale L.	Oleaceae	FL, Le	Himachal Pradesh, NW Himalaya, India	Anti-microbial [ear inf]	Vidyarthi et al., 2013
Jasminum syringifolium Wall. & G.Don	Oleaceae	Le	Andoman + Nicobar Is., India	Anti-paralytic; anti-hypertensive	Chander et al., 2014

Jatropha curcas L.	Euphorbiaceae	La, Le, Ro, Se	India; Mato Grosso, Brazil; Bogotá markets, Columbia; Loja and Zamora-Chinchipe, Ecuador; Benin; D.R. Congo; Guinea-Bissau; Mana Angetu District, SE Ethiopia; Bac Huong Hoa nature reserve, Vietnam; Conis Santana National Park, East Timor; Fundong, NW Cameroon	Anti-paralytic; anti-microbial [conj, *Herpes*, STI, UTI]; memory improvement/ enhancement; anti-convulsant; mental disorder; anti-inflammatory [headache, rheum]; anti-venom; wound healing; anti-toxin	Mikawlrawng et al., 2017; Ribeiro et al., 2017; Bussmann et al., 2018; Tene et al., 2007; Adjanohoun et al., 1989: cited in Noe and Lehmann, 2012 [H130, 168]; Staner and Boutique, 1937: cited in Noe and Lehmann, 2012 [H130]; Romeiras et al., 2012; Lulekal et al., 2008; Heller, 1996: cited in Thomas et al., 2008; Lee et al., 2019; Collins et al., 2007; Bourdy et al., 2000; Focho et al.,2009
Jatropha elliptica (Pohl) Oken	Euphorbiaceae	Le, Rh	Mato Grosso, Brazil	Anti-microbial; anti-inflammatory	Ribeiro et al., 2017
Jatropha glandulifera Roxb.	Euphorbiaceae	Ro, Se oil	Telangana, India	Anti-paralytic	Nishteswar, 2015
Jatropha gossypifolia L. var. elegans (Pohl) Müll.Arg	Euphorbiaceae	Fr, Le	India; Brazilian Amazon; Trinidad; Chittagong Hill Tracts, Bangladesh; Guinea-Bissau	Anti-paralytic; anti-microbial; anti-inflammatory [Fe]; anti-venom; anti-convulsant	Mikawlrawng et al., 2017; Pedrollo et al., 2016; Clement et al., 2015; Kadir et al., 2015; Romeiras et al., 2012
Jatropha pelargoniifolia Courbai	Euphorbiaceae	St	Abyan territory, Yemen	Anti-microbial [bronch, bois, fungal skin inf]	Al-Fatimi, 2019
Jatropha podagrica Hook.	Euphorbiaceae	Le	Kakamega County, Kenya	Anti-inflammatory [Fe]	Odongo et al., 2018
Jatropha spinosa Vahl	Euphorbiaceae	St	Abyan territory, Yemen	Wound healing; poison antidote	Al-Fatimi, 2019
Jodina rhombifolia (Hook. & Arn.) Reisseck	Santalaceae	Ba, Fr, Le	Comechingones, Argentina	Anti-inflammatory	Goleniowski et al., 2006
Juglans neotropica Diels	Juglandaceae	Ba	Markets of Bogotá, Columbia	Anti-microbial [mouth ulcer, UTI, antiseptic]; cholesterol-lowering	Bussmann et al., 2018
Juglans regia L. Syn: *Juglans sinensis*	Juglandaceae	Fr, Le, Se	Himachal Pradesh, NW Himalaya, India; Gheg people, N-E Albania; High Atlas, Morocco; Edremit Gulf, Turkey	Anti-microbial [sore throat, cough, anti-fungal]; anti-hypertensive	Vidyarthi et al., 2013; Pieroni and Sõukand, 2017; Fadili *et al*, 2017; Polat and Satıl, 2012
Juniperus communis L.	Cupressaceae	cone	Gheg people, NE Albania; Pollino National Park, Southern Italy; Istro-Romanians, Northern Istria, Croatia	Anti-microbial [cough]; anti-hypertensive; anti-inflammatory [rheum]	Pieroni and Sõukand, 2017; Di Sanzo et al., 2013; Pieroni et al., 2003
Juniperus indica Bertol.	Cupressaceae	Fr, Le	Central Himalayas, Nepal	Anti-microbial	Bhattarai et al., 2006
Juniperus oxycedrus L.	Cupressaceae	AP	M'Sila, North Algeria	Anti-inflammatory	Boudjelal et al., 2013
Juniperus phoenicea L	Cupressaceae	AP	M'Sila, North Algeria	Anti-inflammatory	Boudjelal et al., 2013
Juniperus recurva Buch.-Ham. ex D.Don	Cupressaceae	Bu	Western Ladakh, India	Anti-paralytic	Angmo et al., 2012

Justicia adhatoda L. Syns: *Adhatoda zeylanica* Medik, *Adhatoda vasica* Nees	Acanthaceae	Le	Karnataka, India; Kani people, Western Ghats, India; Eastern Ghats, India; Mizoram, India	Anti-inflammatory [Fe, asthma, headache, rheum]; anti-microbial [cold, cough, bronc]; anti-venom	Rajakumar and Shivanna, 2009; Ayyanar and Ignacimuthu, 2011; Rao et al., 2006; Rai and Lalramnghinglova, 2010
Justicia chaetocephala (Mildbr.) Leonard	Acanthaceae	Le	Caribbean coast of Columbia	Anti-inflammatory	Gómez-Estrada et al., 2011
Justicia filibracteolata Lindau	Acanthaceae	Ba, Le	Columbian Andes	Anti-inflammatory [rheum]	Cadena-González et al., 2013
Justicia gendarussa Burm.f. Syn: *Gendarussa vulgaris* Nees.	Acanthaceae	Ba, Le	India; Satkhira District, Kalaroa + Chittagong Hill Tracts, Bangladesh	Anti-paralytic; anti-inflammatory [pain]; anti-mcrobial [cough, cold, throat inf]; anti-venom	Mikawlrawng et al., 2017; Dulla and Jahan, 2017; Kadir et al., 2015
Justicia pectoralis Jacq.	Acanthaceae	Le, St	Loja and Zamora-Chinchipe, Ecuador; Trinidad	Anti-inflammatory [pain]; anti-microbial [cold, cough]	Tene et al., 2007; Clement et al., 2015;
Justicia schimperiana (Hochst. ex Nees) T. Anderson	Acanthaceae	WP	Afar people, Rift Valley, Ethiopia	Anti-microbial [diphth]	Teklehaymanot, 2017
Justicia secunda Vahl	Acanthaceae	Le	Martinique	Anti-hypertensive	Longuefosse and Nossin, 1996
Justicia xanthostachya Leonard	Acanthaceae	NS	Markets of Bogotá, Columbia	Anti-inflammatory [Fe]; anti-microbial [mouth inf]	Bussmann et al., 2018
Kaempferia galanga L.	Zingiberaceae	Tu	Ayta communities, Bataan, Philippines;	Anti-inflammatory [Fe]	Roosita et al., 2008
Kaempferia sp.		Le	Sundanese community, West Java	Anti-microbial [boil]	Tantengco et al., 2018;
Kageneckia lanceolata Ruiz & Pavon.	Rosaceae	Le, St	Comechingones, Argentina	Anti-inflammatory [Fe]	Goleniowski et al., 2006
Kalanchoe crenata (Andrews) Haw.	Crassulaceae	Le	Cataractes and Lukaya districts, D.R. Congo	Anti-inflammatory, eye inflammation; anti-microbial	Latham and Konda ku Mbuta, 2016; Bokdam and Droogers, 1975: cited in Noe and Lehmann, 2012 [H001]
Kalanchoe gastonis-bonnieri Raym.-Hamet & H. Perrier	Crassulaceae	NS	Markets of Bogotá, Columbia	Anti-inflammatory; anti-hypertensive; anti-microbial [abscess, UTI, conj]	Bussmann et al., 2018
Kalanchoe petitiana A. Rich.	Crassulaceae	Le	Ethiopia	Eye inflammation	Tuasha et al., 2018: cited in Noe and Lehmann, 2012 [H001]
Kalanchoe pinnata (Lam.) Pers Syn*: Bryophyllum pinnatum* (Lam.) Oken	Crassulaceae	Le, Ro, WP	Martinique; Maranao people, Philippines; Trinidad;Sierra Leone; Sagamu, SW Nigeria; Caiçaras people, Atlantic Forest, Brazil; Rio Jauaperi, Brazilian Amazon; Akha people, North Thailand; Dayaknese Communities, W Kalimantan, Indonesia; Bolivian Amazon	Anti-viral [influenza, cough, pneum, bronch, boil]; anti-aging; anti-hypertensive; anti-inflammatory [Fe, headache]; anti-paralytic	Pedrollo et al., 2016; Longuefosse and Nossin, 1996; Malawani et al., 2017; Latham and Konda ku Mbuta, 2016; Elufioye et al., 2012; Clement et al., 2015; Begossi et al., 2002; Anderson, 1986; Diba et al., 2013; Bourdy et al., 2000
Kalanchoe teretifolia Deflers	Crassulaceae	St	Abyan territory, Yemen	chronic wound	Al-Fatimi, 2019
Kanahia laniflora (Forssk.) R.Br.	Apocynaceae	Ro	Afar people, Rift Valley, Ethiopia	Anti-microbial	Teklehaymanot, 2017
Kedrostis foetidissima (Jacq.) Cogn.	Cucurbitaceae	WP	Mabira Forest, Uganda	Anti-microbial [measles]	Tugume et al., 2016

Khaya anthotheca (Welw.) C.DC.	Meliaceae	Ba, Le	Kimboza forest, Tanzania	Anti-microbial [STI]	Amri and Kisangau, 2012
Khaya ivorensis A.Chev.	Meliaceae	St ba [in sp. comb]	Sagamu, SW Nigeria	Anti-aging	Elufioye et al., 2012
Kigelia africana (Lam.) Benth.	Bignoniaceae	Ba, Fr, Le	Kimboza forest, Tanzania; Mabira Forest, Uganda; Guinea-Bissau; Fundong, NW Cameroon	Anti-inflammatory [Fe, rheum]; anti-hypertensive; anti-microbial [dys, pneum, STI, abscess ulcer]; anti-depressant; wound	Amri and Kisangau, 2012; Tugume et al., 2016; Romeiras et al., 2012; Focho et al.,2009
Kirkia acuminata Oliv.	Kirkiaceae	Ba, Fr	Angola	Anti-microbial [diarrh, cholera, dys]; anti-venom; eye inflammation	Urso et al., 2016
Kleinia longiflora DC. Syn: *Senecio longiflorus* (DC.) Sch.Bip.	Asteraceae	Le	Oromo people, Harla, Eastern Ethiopia	Anti-paralytic	Belayneh and Bussa, 2014
Kleinia odora (Forssk.) DC.	Asteraceae	St	Abyan territory, Yemen	chronic wound	Al-Fatimi, 2019
Kleinia squarrosa Cufod.	Asteraceae	Le	Afar people, Rift Valley, Ethiopia	Anti-microbial [eye inf]	Teklehaymanot, 2017
Koanophyllon solidaginoides (Kunth) R.M. King & H. Rob.	Compositae	NS	Markets of Bogotá, Columbia	Anti-microbial [influenza]	Bussmann et al., 2018
Kohleria spicata (Kunth) Oerst.	Gesneriaceae	NS	Markets of Bogotá, Columbia	anti-microbial [UTI]	Bussmann et al., 2018
Krameria lappacea (Dombey) Berdet & B. Simpson	Krameriaceae	Le, Ro	Mestizo community, North Peru	Anti-inflammatory	Bussmann and Sharon, 2006
Kyllinga brevifolia Rottb.	Cyperaceae	Le	Andoman + Nicobar Is., India	Anti-inflammatory [Fe]	Chander et al., 2014
Kyllinga nemoralis (J.R.Forst. & G.Forst.) Dandy ex Hutch. & Dalziel	Cyperaceae	AP	Marquesas Islands, French Polynesia	Anti-microbial	Girardi et al., 2015
Lablab purpureus (L.) Sweet Syn: *Dolichos lablab* L.	Leguminosae	Fr	Mestizo community, North Peru	Anti-inflammatory	Bussmann and Sharon, 2006
Lactuca serriola L. Syn: *Lactuca scariola* L.	Asteraceae	Le	Abyan territory, Yemen; High Atlas, Morocco	Anxiolytic; anti-venom	Al-Fatimi, 2019; Fadili *et al*, 2017
Lafoensia pacari A. St.-Hil.	Lythraceae	Ba, Le, Ro, sap	Mato Grosso, Brazil	Anti-microbial; anti-inflammatory	Ribeiro et al., 2017
Lagenaria siceraria (Molina) Standl.	Cucurbitaceae	Fr	Ayta communities, Bataan, Philippines	Anti-hypertensive	Tantengco et al., 2018
Lagerstroemia speciosa (L.) Pers.	Lythraceae	Ba, Le, Fr, PL, St	Maranao people, Philippines; Mizoram, India	Anti-microbial [dys, UTI]; anti-inflammatory [Fe]	Tantengco et al., 2018; Malawani et al., 2017; Rai and Lalramnghinglova, 2010
Landolphia camptoloba (K.Schum.) Pichon	Apocynaceae	Fr	Cataractes and Lukaya districts, D.R. Congo	Anti-microbial [conj, cough]	Latham and Konda ku Mbuta, 2016
Lannea acida A. Rich. Syn: *Lannea microcarpa* Engl. & K.Krause	Anacardiaceae	Le, St	Benin; Burkina Faso	Memory improvement/ enhancement; eye inflammation; anti-hypertensive	Adjanohoun et al., 1989: cited in Noe and Lehmann, 2012 [H168]; de la Pradilla: cited in Noe and Lehmann, 2012 [H001, H151]

Lannea antiscorbutica (Hiern) Engl.	Anacardiaceae	Ba	Cataractes and Lukaya districts, D.R. Congo	Anti-microbial [dental caries]	Latham and Konda ku Mbuta, 2016
Lannea coromandelica (Houtt.) Merr.	Anacardiaceae	Ro	Eastern Ghats, India	Anti-inflammatory [asthma]	Rao et al., 2006
Lantana canescens Kunth	Verbenaceae	NS	Markets of Bogotá, Columbia	Anti-inflammatory [Fe]	Bussmann et al., 2018
Lantana trifolia L.	Verbenaceae	Ro	Mabira Forest, Uganda	Anti-microbial [yell fev]	Tugume et al., 2016
Lapageria rosea Ruiz and Pav. (ind)	Philesiaceae	Le	Mapuche people, Chile	Energy-boosting	Houghton and Manby, 1985
Laurelia sempervirens Ruiz and Pav. (ind)	Atherospermataceae	Le	Mapuche people, Chile	Anti-paralytic	Houghton and Manby, 1985
Laurus nobilis L.	Lauraceae	NS	Mapuche people, Argentina	Anti-inflammatory	Estomba et al., 2005
Lavandula stoechas L.	Lamiaceae	FL, Le	Taounate, North Morocco; Edremit Gulf, Turkey	Anti-inflammatory [rheum, headache]; anti-hypertensive, anti-hyper cholesterolemia	El-Hilaly et al., 2003; Polat and Satıl, 2012
Lawsonia inermis L.	Lythraceae	Le	Abyan territory, Yemen; Sargodha region, Punjab, Pakistan	Anti-microbial [UTI]; poison antidote; memory enhancement	Al-Fatimi, 2019; Abdul et al., 2012
Leea guineensis G.Don	Vitaceae	Fr, Le	Cataractes and Lukaya districts, D.R. Congo; Guinea-Bissau	Anti-microbial [STI; anti-epileptic	Latham and Konda ku Mbuta, 2016; Romeiras et al., 2012
Leea indica (Burm.f.) Merr.	Vitaceae	Le	Andoman + Nicobar Is., India; Conis Santana National Park, East Timor	Anti-inflammatory [Fe]; anti-microbial [diarrh]	Chander et al., 2014; Collins et al., 2007
Lemna minuta Kunth	Araceae	WP	Mestizo community, North Peru	Anti-inflammatory	Bussmann and Sharon, 2006
Lens culinaris Medik.	Fabaceae	Se	Sargodha region, Punjab, Pakistan	Anti-paralytic, anti-PD; anti-microbial [measles]	Abdul et al., 2012
Leonotis nepetifolia (L.) R.Br.	Lamiaceae	AP, Bd, Le, Se, WP	Mato Grosso, Brazil; Martinique; Trinidad; Eastern Ghats, India	Anti-inflammatory [Fe]; anti-microbial [cold, cough]; anti-toxin	Ribeiro et al., 2017; Longuefosse and Nossin, 1996; Clement et al., 2015; Rao et al., 2006
Leonurus cardiaca L.	Lamiaceae	AP	Albanians, Bosniaks/Gorani + Turkish ethnic groups, Kosovo	Memory enhancement; cardiotonic, improved circulation	Mustafa et al., 2015
Leonurus sibiricus L.	Lamiaceae	Le	Mato Grosso, Brazil	stroke prevention	Ribeiro et al., 2017
Lepidium bipinnatifidum Desv.	Brassicaceae	NS	Markets of Bogotá, Columbia	Anti-microbial [UTI]	Bussmann et al., 2018
Lepidium draba L.	Brassicaceae	AP	Iran	Anti-viral [cold]; anti-inflammatory	Mosaddegh et al., 2013
Lepidium nitidum Nutt.	Brassicaceae	WP	Chumash Indians, California, USA	Anti-microbial	Timbrook, 1990
Lepidium sativum L.	Brassicaceae	FL	Afar people, Rift Valley, Ethiopia	Anti-microbial	Teklehaymanot, 2017
Lepidium thurberi Wooton	Brassicaceae	Le	Loja and Zamora-Chinchipe, Ecuador	Anti-inflammatory	Tene et al., 2007
Lepisanthes rubiginosa (Roxb.) Leenh.	Sapindaceae	Le	Andoman + Nicobar Is., India	Anti-inflammatory [Fe]; anti-hypertensive	Chander et al., 2014
Leptadenia pyrotechnica (Forssk.) Decne.	Apocynaceae]	AP	Tassili N'Ajjer, Southern Algerian Sahara	Anti-inflammatory [Fe]; anti-microbial [cough]	Hammiche and Maiza, 2006

Leptochloa chinensis (L.) Nees.	Poaceae	WP	Tian Mu Shan Biosphere Reserve, Zhejiang	Anti-inflammatory	Chaudhary et al., 2006
Leucaena leucocephala (Lam.) de Wit	Fabaceae	Ro, WP	Myanmar	Anti-venom/ anti-poison	Perry and Metzger, 1980: cited in DeFilipps and Krupnick, 2018
Leucas aspera (Willd.) Link	Lamiaceae	Le	Rakhain people, Bangladesh; Karnataka, India; Kani people, Western Ghats, India; Thari people of Nara Desert, Pakistan; Dinajpur District, Bangladesh	Anti-inflammatory [headache, asthma]; anti-microbial [dys, eye inf, sore throat]	Hanif et al., 2009; Rajakumar and Shivanna, 2009; Ayyanar and Ignacimuthu, 2011; Qureshi and Bhatti, 2008; Rahmatullah et al., 2009
Leucas cephalotes (Roth) Spreng.	Lamiaceae	WP	India, Myanmar	Anti-venom; anti-inflammatory; anti-microbial [cough, bronch]	Nordal, 1963: cited in DeFilipps and Krupnick, 2018
Leucas decemdentata (Willd.) Sm.	Lamiaceae	WP	Maori people, Cook Islands	Anti-microbial [anti-fungal, UTI]	Whistler, 1985
Leucas martinicensis (Jacq.) R.Br.	Lamiaceae	Le, Ju	Rwanda; Tigrigna people, Central Eritrea	Eye inflammation	Kayonga and Habiyaremye, 1987: cited in Noe and Lehmann, 2012 [H001]; Yemane et al., 2017
Libertia ixioides (G.Forst.) Spreng.	Iridaceae	Le	Mapuche people, Chile	Poisoning antidote	Houghton and Manby, 1985
Libidibia ferrea (Mart. ex Tul.) L.P.Queiroz Syn*: Caesalpinia ferrea*	Fabaceae	Ba, Fr, Se	Mato Grosso, Brazil	Anti-microbial	Ribeiro et al., 2017
Ligaria cuneifolia (Ruiz ex Pavon) Thiegh.	Loranthaceae	Le, St	Comechingones, Argentina	Anti-hypertensive	Goleniowski et al., 2006
Lindackeria laurina C.Presl	Achariaceae	St	Guaymi indians, Panama	Anti-microbial	Joly et al., 1990
Lindernia diffusa (L.) Wettst.	Linderniaceae	FL, Le	Martinique	Anti-microbial	Longuefosse and Nossin, 1996
Linum bienne Mill. Syn*: Linum angustifolium*	Linaceae	Se	Martinique	Anti-inflammatory; anti-microbial [measles]	Longuefosse and Nossin, 1996
Linum puberulum (Engelm.) Heller	Linaceae	Fr	Native America	Anti-inflammatory [eye inflam]	Train et al., 1941: cited in Native American Ethnobotany Database
Linum sativum L.	Linaceae	Se	Mestizo community, North Peru	Anti-inflammatory	Bussmann and Sharon, 2006
Linum selaginoides Lam. (ind)	Linaceae	Le	Mapuche people, Chile	Anti-inflammatory [Fe]	Houghton and Manby, 1985
Linum usitatissimum L.	Linaceae	Se	Mestizo community, North Peru; Markets of Bogotá, Columbia	Anti-inflammatory; anti-microbial [bronch, UTI]; Edremit Gulf, Turkey	Bussmann and Sharon, 2006; Bussmann et al., 2018; Polat and Satıl, 2012
Lippia alba (Mill.) N.E. Brown	Verbenaceae	Le	Rio Jauaperi, Brazilian Amazon; Martinique; Trinidad; Columbian Andes	Anti-viral [influenza], anti-hypertensive; anti-inflammatory [Fe]; fatigue, weakness	Pedrollo et al., 2016; Longuefosse and Nossin, 1996; Clement et al., 2015
Lippia integrifolia (Grieseb.) Hieron	Verbenaceae	Le, St	Mestizo community, North Peru	Anti-inflammatory	Bussmann and Sharon, 2006
Lippia javanica (Burm.f.) Spreng.	Verbenaceae	NS	Gingee Hills, Villupuram, Tamil Nadu, India	Anti-inflammatory [asthma]	Arulappan et al., 2015
Lippia multiflora Moldenke	Verbenaceae	St ba	Cataractes and Lukaya districts, D.R. Congo; Togo	Anti-hypertensive; anti-microbial; anti-AD; anti-PD; epilepsy; stroke	Latham and Konda ku Mbuta, 2016; Kantati et al., 2016

Lithospermum erythrorhizon Siebold & Zucc.	Boraginaceae	Ro	Maonan people, Guangxi Zhuang, China	Anti-microbial [measles]	Hong et al., 2015
Litsea cubeba (Lour.) Pers. Litsea	Lauraceae	WP	Mizoram, India	Anti-paralytic; memory improvement; anxioytic	Rai and Lalramnghinglova, 2010
Litsea glutinosa (Lour.) C.B.Rob. Syn: *Litsea chinensis* Lam.	Lauraceae	FL, Le	Dinajpur District, Bangladesh	Strengthening	Meera, 2010
Loeseneriella clematoides (Loes.) R.Wilczek	Celastraceae	Sap	Cataractes and Lukaya districts, D.R. Congo	Anti-inflammatory [Fe]	Latham and Konda ku Mbuta, 2016
Lomatia ferruginea R.Br. (int)	Proteaceae	Ba, Le	Mapuche people, Chile	Anti-microbial [abscess]	Houghton and Manby, 1985
Lomatia hirsuta (Lam.) Diels	Proteaceae	NS	Mapuche people, Argentina	Anti-inflammatory	Estomba et al., 2005
Lomatium californicum (Nutt.) Mathias & Constance	Apiaceae	Ro	Chumash Indians, California, USA	Anti-paralytic; anti-inflammatory [rheum]	Timbrook, 1990
Lonicera involucrata (Richardson) Banks ex Spreng.	Caprifoliaceae	St	Native America	Weakness/ paralysis	Smith, 1929: cited in Native American Ethnobotany Database
Lonicera japonica Thunb.	Caprifoliaceae	FL, St	Maonan people, Guangxi Zhuang, China	Anti-microbial [influenza, pneum]	Hong et al., 2015
Lonicera maackii (Rupr.) Maxim.	Caprifoliaceae	Ro	Tian Mu Shan Biosphere Reserve, Zhejiang	Anti-viral; anti-inflammatory	Chaudhary et al., 2006
Lophatherum gracile Brongn.	Poaceae	WP	Hakka people, Guangdong, China	Anti-inflammatory	Au et al., 2008
Lophira lanceolata Tiegh. ex Keay	Ochnaceae	Le	Benin	Eye inflammation; anti-hypertensive	Adjanohoun et al., 1989: cited in Noe and Lehmann, 2012 [H001]; Apema et al., 2011: cited in Noe and Lehmann, 2012 [H151]
Ludwigia abyssinica A.Rich	Onagraceae	Le	Haya people, Kagera, NW Tanzania	Anti-microbial [HIV]	Moshi et al., 2009
Ludwigia hyssopifolia (G.Don) Exell	Onagraceae	AP	Eastern Highlands, Papua New Guinea	Anti-inflammatory	Jorim et al., 2012
Ludwigia peruviana (L.) H. Hara	Onagraceae	NS	Markets of Bogotá, Columbia	Anti-microbial [RTI, pneum]	Bussmann et al., 2018
Ludwigia repens J.R. Forst.	Onagraceae	Le, St	Maromizaha forest, Madagascar	Anti-fatigue	Riondato et al., 2019
Luma chequen (Molina) A.Gray Syn: *Myrceugenella chequen* (Mol.) Kausel. (ind)	Myrtaceae	Le	Mapuche people, Chile	Anti-inflammatory [eye/renal inflam]	Houghton and Manby, 1985
Luma apiculata (DC.) Burret Syn: *Myrceugenella apiculuta* (DC.) Kausel. (ind)	Myrtaceae	Ro	Mapuche people, Chile	Anti-microbial [dys]	Houghton and Manby, 1985
Lupinus mutabilis Sweet	Fabaceae	Le	Loja and Zamora-Chinchipe, Ecuador	Anti-inflammatory [Fe]	Tene et al., 2007
Lycianthes asarifolia (Kunth & Bouché) Bitter	Solanaceae	Bd, Le	Bolivian Amazon	Anti-microbial [boil]	Bourdy et al., 2000
Lycium intricatum Boiss.	Solanaceae	NS	Western Sahara	Eye inflammation	Volpato et al., 2012: cited in Noe and Lehmann, 2012 [H001]

Lycium shawii Roem. & Schult.	Solanaceae	Le, St	N + S Oman; Abyan territory, Yemen	Anti-inflammatory; anti-microbial [boil]; wound healing	Divakar et al., 2016; Al-Fatimi, 2019
Lycopodiella cernua (L.) Pic. Serm.	Lycopodiaceae	Le	Martinique	Anti-hypertensive	Longuefosse and Nossin, 1996
Lycopus lucidus Turcz. var *hirtus* Regel.	Lamiaceae	WP	Tian Mu Shan Biosphere Reserve, Zhejiang	Anti-hypertensive	Chaudhary et al., 2006
Lygodium circinnatum (Burm. f.) Sw.	Lygodiaceae	Le	Andoman + Nicobar Is., India; Orang Asli tribe, Malaysia	Anti-hypertensive; anti-microbial ([eye inf]	Chander et al., 2014; Samuel et al., 2010
Lygodium japonicum (Thunb.) Sw.	Lygodiaceae	WP	Magar and Majhi people, Nepal	Anti-microbial [boil]	Malla et al., 2015
Lysimachia arvensis (L.) U.Manns & Anderb Syn: *Anagallis arvensis*	Primulaceae	AP	Iran	Anti-viral [cold]	Mosaddegh et al., 2013
Macaranga peltata (Roxb.) Müll.Arg.	Euphorbiaceae	Le	Andoman + Nicobar Is., India	Anti-inflammatory [Fe]	Chander et al., 2014
Macaranga perrieri Leandri [unresolved]	Euphorbiaceae	Le	Maromizaha forest, Madagascar	Anti-fatigue	Riondato et al., 2019
Macaranga triloba (Thunb.) Müll.Arg.	Euphorbiaceae	Le	Dayaknese Communities, W Kalimantan, Indonesia	Anti-inflammatory [Fe]	Diba et al., 2013
Machaerium stipitatum (DC.) Vogel	Leguminosae	Le	Mato Grosso, Brazil	Anti-microbial	Ribeiro et al., 2017
Maclura tinctoria (L.) D. Don. Ex Steud.	Moraceae	Ba, La	Mato Grosso, Brazil	Anti-inflammatory [rheum, toothache]	Ribeiro et al., 2017
Macropanax dispermus (Blume) Kuntze	Araliaceae	Le	Chin people, Myanmar	Anti-inflammatory [Fe]	Ong et al., 2018
Maerua crassifolia Forssk.	Capparaceae	Ba	Tassili N'Ajjer, Southern Algerian Sahara	Anti-inflammatory [Fe]	Hammiche and Maiza, 2006
Maesa lanceolata Forssk.	Primulaceae	Le, Ro	Cataractes and Lukaya districts, D.R. Congo; Nyanza and Kamba people, Kenya	Anti-microbial [s'pox, diarrh, g-i inf]; memory improvement; anti-venom	Latham and Konda ku Mbuta, 2017; Novy, 1997: cited in Noe and Lehmann, 2012 [H168]; Owuor and Kisangau, 2006
Maesobotrya vermeulenii (De Wild.) J.Léonard	Phyllanthaceae	Ba	Congo-Brazzaville	Anti-microbial [leprosy]	Latham and Konda ku Mbuta, 2017
Magnolia delavayi Franch.	Magnoliaceae	Ba	Lisu, Yunnan, China	Anti-microbial	Ji et al., 2005
Mahonia bealei (Fortune) Pynaert	Berberidaceae	Ro	Maonan people, Guangxi Zhuang, China	Anti-microbial [influenza, pneum]	Hong et al., 2015
Maianthemum stellatum (L.) Link	Asparagaceae	Ro	Native America	Anti-inflammatory [eye inflam]	Train et al., 1941: cited in Native American Ethnobotany Database
Malachra rudis Benth.	Malvaceae	NS	Markets of Bogotá, Columbia	Anti-microbial [influenza, bronch]	Bussmann et al., 2018
Mallotus apelta (Lour.) Müll.Arg.	Euphorbiaceae	Le, Ro	Maonan people, Guangxi Zhuang, China	Anti-microbial [hepatitis, g- i inf, fungal inf]	Hong et al., 2015
Mallotus philippensis (Lam.) Müll.Arg.	Euphorbiaceae	St ba	Mizoram, India	Anti-viral [HSV]	Rai and Lalramnghinglova, 2010
Mallotus polycarpus (Benth.) Kulju & Welzen Syn: *Trewia polycarpa* Benth.	Euphorbiaceae	Le	Gingee Hills, Tamil Nadu, India	Wound healing	Arulappan et al., 2015

Malpighia emarginata DC. Syn: *Malpighia glabra* L.	Malpighiaceae	NS	Markets of Bogotá, Columbia; Mato Grosso, Brazil Mato Grosso, Brazil	Anti-microbial [STI, UTI]; influenza	Bussmann et al., 2018; Ribeiro et al., 2017
Malus pumila Mill. Syn: *Malus domestica* (Suckow) Borkh.	Rosaceae	NS	Markets of Bogotá, Columbia	Anti-hypertensive	Bussmann et al., 2018
Malus sylvestris (L.) Mill.	Rosaceae	Fr	Gheg people, NE Albania	Anti-microbial [cold]	Pieroni and Sõukand, 2017
Malva parviflora L.	Malvaceae	Le, Ro	Nuevo León, Mexico; Mato Grosso, Brazil; Chumash Indians, California, USA	Anti-inflammatory [Fe]; anti-microbial	Estrada-Castillón et al., 2012; Ribeiro et al., 2017; Timbrook, 1990
Malva sylvestris L.	Malvaceae	AP	M'Sila, North Algeria	Anti-inflammatory	Boudjelal et al., 2013
Malva verticillata L.	Malvaceae	FL	Central Himalayas, Nepal	Anti-microbial	Bhattarai et al., 2006
Mandevilla velame (A.St.-Hil.) Pichon	Apocynaceae	Le, Ro, WP	Mato Grosso, Brazil	Anti-microbial; anti-inflammatory	Ribeiro et al., 2017
Mangifera indica L.	Anacardiaceae	Ba, Le	Martinique; Trinidad; Jamaica; Cataractes and Lukaya districts, D.R. Congo; Beninl Conis Santana National Park, East Timor; Fundong, NW Cameroon	Anti-microbial [dental caries, STI, typhoid]; anti-inflammatory; anti-hypertensive; memory improvement/ enhancement; boost immune system; wound healing	Longuefosse and Nossin, 1996; Kembelo, 2003, Adjanohoun et al., 1989: cited in Noe and Lehmann, 2012 [H168]; Clement et al., 2015; Latham and Konda ku Mbuta, 2017; Focho et al., 2009; Collins et al., 2007
Manihot esculenta Crantz	Euphorbiaceae	Le, Ro, Tu	Martinique; Maromizaha forest, Madagascar; Maranao people, Philippines; Mestizo community, North Peru	Anti-inflammatory [Fe, inflam]; anti-hypertensive; anti-microbial	Longuefosse and Nossin, 1996; Riondato et al., 2019; Malawani et al., 2017; Bussmann and Sharon, 2006
Manilkara zapota (L.) P. Royen	Sapotaceae	NS	Markets of Bogotá, Columbia	Anti-inflammatory [Fe]; anti-microbial {kidney inf]	Bussmann et al., 2018
Manotes expansa Sol. ex Planch.	Connaraceae	Ro, Sh	Bas-Congo; Rain forests from Guinea to Liberia	Anti-microbial [dys, conj]; anti-inflammatory [headache]	Latham and Konda ku Mbuta, 2017
Manotes longiflora Baker	Connaraceae	Le	Ehotile people, Côte d'Ivoire	Anti-microbial [dys]	Malan et al., 2015
*Mansoa alliacea (*Lam.) A.H.Gentry; *Mansoa* sp.	Bignoniaceae	Le	Loja and Zamora-Chinchipe, Ecuador; Tacana people, Bolivian Amazon	Anti-microbial [int inf, cold]; anti-inflammatory [Fe, rheum, pain]	Tene et al., 2007; Bourdy et al., 2000
Mansonia altissima (A. Chev.) A. Chev.	Malvaceae	Le	South Nigeria	Anti-microbial [leprosy]	Borokini et al., 2012
Mapania cuspidata (Miq.) Uittien	Cyperaceae	Le [in sp. comb]	Seberida, Riau Province, Sumatra, Indonesia	Anti-inflammatory	Mahyar et al., 1991
Maranta arundinacea L.	Marantaceae	Le, Ro	Martinique; Bas-Congo	Anti-inflammatory; detoxifying; wound healing	Longuefosse and Nossin, 1996; Latham and ku Mbuta, 2017
Markhamia lutea (Benth) K. Schum.	Bignoniaceae	Ba, Le, Ro	Kakamega County, Kenya	Anti-microbial [STI]; wound healing	Odongo et al., 2018
Marrubium deserti (de Noé) Coss	Lamiaceae	Le	Tassili N'Ajjer, Southern Algerian Sahara	Anti-inflammatory [Fe]	Hammiche and Maiza, 2006
Marrubium supinum L	Lamiaceae	AP	M'Sila, North Algeria	Anti-hypertensive	Boudjelal et al., 2013

Marrubium vulgare L.	Lamiaceae	FL br, Le, St, WP	Mestizo community, North Peru; Markets of Bogotá, Columbia; Taounate, North Morocco; Edremit Gulf, Turkey	Anti-inflammatory [Fe]; anti-microbial [TB, gangr, cough, cold]; anxiolytic	Bussmann and Sharon, 2006; Bussman et al., 2018; El-Hilaly et al., 2003; Polat and Satıl, 2012
Marsilea quadrifolia L.	Marsileaceae	WP	Maonan people, Guangxi Zhuang, China	Anti-inflammatory	Hong et al., 2015
Marsypianthes chamaedrys (Vahl) Kuntze	Lamiaceae	Le	Mato Grosso, Brazil	Anti-inflammatory	Ribeiro et al., 2017
Martinella obovata (Kunth) Bureau & K.Schum.	Bignoniaceae	Bu	Tacana people, Bolivian Amazon	Anti-microbial [conj]	Bourdy et al., 2000
Matricaria chamomilla L. Syn: *Matricaria recutita* L.	Asteraceae/Compositae	AP, FL, Le, St	Markets of Bogotá, Columbia; Nuevo León, Mexico; Mestizo community, North Peru; Svaneti and Racha-Lechkumi, Georgia; Potosi, Bolivia; Agro Nocerino Sarnese, Southern Italy; Yörük people, SE Macedonia	Anti-inflammatory [rheum]; anti-microbial [influenza, cough]; wound healing; anti-fatigue	Bussmann ans Sharon, 2006; Bussmann et al., 2016, 2018; Estrada-Castillón et al., 2012; Fernandez et al., 2003; Motti and Motti, 2017; Nedelcheva et al., 2017
Matricaria frigidum (HBK) Kunth	Asteraceae/Compositae	WP	Mestizo community, North Peru	Anti-inflammatory	Bussmann and Sharon, 2006
Mauria heterophylla H.B.K.	Anacardiaceae	Le	Mestizo community, North Peru	Anti-inflammatory	Bussmann and Sharon, 2006
Mauritia flexuosa L.f.	Arecaceae	Fr, Ro, Se	Mato Grosso, Brazil	Anti-inflammatory; anti-hypertensive	Ribeiro et al., 2017
Mauritiella armata (Mart.) Burret	Arecaceae	St	Mato Grosso, Brazil	Anti-microbial	Ribeiro et al., 2017
Maxillaria sp.	Orchidaceae	Le	Guaymi indians, Panama	Anti-fatigue	Joly et al., 1990
Maytenus boaria Molina	Celastraceae	Le	Comechingones, Argentina	Anti-inflammatory [Fe]	Goleniowski et al., 2006
Maytenus ilicifolia Mart. ex Reissek	Celastraceae	Le	Mato Grosso, Brazil	Anti-microbial; anti-inflammatory	Ribeiro et al., 2017
Maytenus laevis Reissek	Celastraceae	NS	Markets of Bogotá, Columbia	Anti-inflammatory	Bussmann et al., 2018
Medicago sativa L.	Fabaceae	AP	Markets of Bogotá, Columbia; Catalonia, Spain; Potosi, Bolivia	Anti-hypertensive; anti-microbial [TB, cough, UTI]; anti-inflammatory	Bussmann et al., 2018; Raja et al., 1997; Fernandez et al., 2003
Melaleuca kucadendra (L.) L.	Myrtaceae	Ba	Native Northern territory, Australia	Anti-inflammatory [Fe]	Smith, 1991
Melaleuca viridiflora Sol. ex Gaertner	Myrtaceae	Le	Native Northern territory, Australia	Anti-inflammatory [Fe]	Smith, 1991
Melanthera scandens (Schumach. & Thonn.) Roberty	Asteraceae	Le	Mabira Forest, Uganda	Anti-viral [yell fev]	Tugume et al., 2016
Melastoma sp.	Melastomataceae	Le	Dayaknese Communities, W Kalimantan, Indonesia	Anti-microbial [influenza, cough]	Diba et al., 2013
Melicoccus bijugatus Jacq.	Sapindaceae	Le	Martinique	Anti-inflammatory [Fe]; anti-microbial [influenza]	Longuefosse and Nossin, 1996
Melilotus alba Medikus	Fabaceae	Se	Mestizo community, North Peru	Anti-microbial	Bussmann and Sharon, 2006
Melilotus suaveolens Ledeb.	Fabaceae	Le	Rwanda	Anti-microbial/anti-paralytic [polio]	Hassan-Abdallah et al., 2013: cited in Noe and Lehmann, 2012 [H130]

Melinis minutiflora P.Beauv.	Poaceae	WP	Mato Grosso, Brazil	stroke	Ribeiro et al., 2017
Melissa officinalis L.	Lamiaceae	FL, FL br	Caiçaras people, Atlantic Forest, Brazil; Mashhad markets, Iran; Edremit Gulf, Turkey	Anti-hypertensive; anti-inflammatory; anti-microbial [influenza]; anxiolytic; memory improvement	Begossi et al., 2002; Amiri and Joharchi, 2013; Polat and Satıl, 2012
Mentha arvensis L.	Lamiaceae	AP, Le, St, WP	Budgam, Kashmir, India	Memory improvement	Hassan et al., 2013
Mentha canadensis L. Syn*: Mentha haplocalyx* Briq.	Lamiaceae	Le	Hakka people, Guangdong, China	Anti-microbial [cold]	Au et al., 2008
Mentha longifolia (L.) L	Lamiaceae	Le, AP	Central Himalayas, Nepal; Svaneti and Racha-Lechkumi, Georgia; Turkestan Range of south Kyrgystan	Anti-microbial; anxiolytic; anti-ulcer	Bhattarai et al., 2006; Bussmann et al., 2016; Pawera et al., 2016
Mentha pulegium L.	Lamiaceae	AP, Le, St, WP	M'Sila, North Algeria; Taounate, North Morocco; High Atlas, Morocco	Anti-hypertensive; anti-microbial [cold, cough]; anti-inflammatory [Fe]	Boudjelal et al., 2013; El-Hilaly et al., 2003; Fadili *et al*, 2017
Mentha × rotundifolia (L.) Huds.	Lamiaceae	AP	M'Sila, North Algeria	Anti-inflammatory	Boudjelal et al., 2013
Mentha royleana Wall. ex Benth. Syn: *Mentha royleana* subsp. *hymalaiensis* Briq	Lamiaceae	Le	Toli Peer National Park, Kashmir, Pakistan	Anti-microbial	Amjad et al., 2017
Mentha spicata L. Syn: *Mentha viridis*	Lamiaceae	Le, St	Balkan Peninsula, Turkey; Brazil Mato Grosso; Martinique; Markets of Bogotá, Columbia; Taounate, North Morocco; Edremit Gulf, Turkey; Yörük people, SE Macedonia	Anti-microbial; anti-viral [influenza, cold, cough]; anti-hypertensive; anti-inflammatory [headache]; fatigue; anxiolytic	Ribeiro et al., 2017; Longuefosse and Nossin, 1996; Bussmann et al., 2018; El-Hilaly et al., 2003; Polat and Satıl, 2012; Nedelcheva et al., 2017
Mentha x *piperita* L. [hybrid of *M. spicata* L. and *Mentha aquatica* L.]	Lamiaceae	AP, WP	Svaneti and Racha-Lechkumi, Georgia; Abyan territory, Yemen	Anxiolytic; anti-microbial [sore throat, influenza]	Bussmann et al., 2016; Al-Fatimi, 2019
Mercurialis annua L.	Euphorbiaceae	WP	Agro Nocerino Sarnese, Southern Italy	Anti-microbial [wart]	Motti and Motti, 2017
Meriandra dianthera (Roth ex Roem. & Schult.) Briq.	Lamiaceae	Le	Abyan territory, Yemen; Tigrigna people, Central Eritrea	Anti-microbial [cough]; anti-hypertensive	Al-Fatimi, 2019; Yemane et al., 2017
Mesua ferrea L.	Calophyllaceae	Ba, FL, Le, Se	Myanmar; Mizoram, India	Anti-venom; anti-microbial [cough]; anti-inflammatory [Fe]	Nordal, 1963: cited in DeFilipps and Krupnick, 2018; Rai and Lalramnghinglova, 2010
Microglossa pyrifolia (Lam.) Kuntze	Compositae	Le, St	Nyanza and Kamba people, East Kenya; Kakamega County, Western Kenya	Anti-venom; wound healing	Owuor and Kisangau, 2006; Odongo et al., 2018
Micromeria myrtifolia Boiss. & Hohen.	Lamiaceae	AP	Edremit Gulf, Turkey	Anti-microbial [cold, influenza]	Polat and Satıl, 2012
Microtea debilis Sw.	Phytolaccaceae	Le, Ro	Martinique; Trinidad	Anti-inflammatory; anti-microbial [cold]	Longuefosse and Nossin, 1996; Clement et al., 2015

Mikania cordata (Burm.f.) B.L.Rob.	Compositae	Le	D.R. Congo	Eye inflammation	Wome, 1985: cited in Noe and Lehmann, 2012 [H001]
Mikania cordifolia (L.f.) Willd.	Asteraceae/Compositae	AP	Tacana people, Bolivian Amazon	Anti-venom	Bourdy et al., 2000
Mikania guaco Kunth	Asteraceae/Compositae	Le	Antioquia, Columbia	Anti-venom	Vasquez et al., 2013 Antioquia, Columbia
Miliusia andamanica (King) Finet & Gagnep.	Annonaceae	Le	Andoman + Nicobar Is., India	Anti-inflammatory [Fe]	Chander et al., 2014
Millettia eetveldeana (Micheli) Hauman	Fabaceae	Ba	Bas-Congo Province, D.R. Congo	Anti-microbial/anti-paralytic [polio]	Kembelo, 2003: cited in Noe and Lehmann, 2012 [H130]
Millettia laurentii De Wild.	Fabaceae	NS	Cataractes and Lukaya districts, D.R. Congo	Anti-inflammatory [Fe]	Latham and Konda ku Mbuta, 2017
Milletia pinnata (L.) Panigrahi	Fabaceae	Se	Kani people, Western Ghats, India	wound	Ayyanar and Ignacimuthu, 2011
Millettia thonningii (Schum & Thonn) Bak	Fabaceae	Le	South Nigeria	Anti-microbial [dys, leprosy]	Borokini et al., 2012
Millettia versicolor Welw. Ex Baker	Fabaceae	Le	Masako Forest Reserve, D.R. Congo	Anti-microbial [influenza]	Mbula et al., 2015
Mimosa nothacacia Barneby	Leguminosae	Ba	Mestizo community, North Peru	Anti-inflammatory	Bussmann and Sharon, 2006
Mimosa pigra L.	Leguminosae	Le, Ro ash	Cataractes and Lukaya districts, D.R. Congo	Anti-inflammatory; anti-microbial [STI, septicaemia, diarrh, leprosy]	Latham and Konda ku Mbuta, 2017
Mimosa pudica L.	Leguminosae	Le, Ro, WP	Trinidad; Guaymi indians, Panama; Bogotá markets, Columbia; Maharashtra, India; Dayaknese Communities, W Kalimantan, Indonesia	Anti-microbial [influenza, cold]; anti-inflammatory; anti-epileptic; wound healing	Clement et al., 2015; Joly et al., 1987; Bussmann et al, 2018; Wadankar et al., 2011; Diba et al., 2013
Mimusops elengi L.	Sapotaceae	St ba	Eastern Ghats, India	Anti-inflammatory [Fe]	Rao et al., 2006
Minthostachys glabrescens (Benth.) Epling Syn: *Bystropogon glabrescens* Benth.	Lamiaceae	AP	Potosi, Bolivia	Tremor, anti-inflammatory [rheum]	Fernandez et al., 2003
Minthostachys mollis (Kunth) Griseb.	Lamiaceae	WP	Loja and Zamora-Chinchipe, Ecuador; Markets of Bogotá, Columbia; Potosi, Bolivia	Anti-microbial [influenza]; anti-inflammatory [throat inflam, rheum]	Tene et al., 2007; Bussmann et al., 2018; Fernandez et al., 2003
Mirabilis jalapa L.	Nyctaginaceae	Ro, WP	Myanmar; Chittagong Hill Tracts, Bangladesh; Benin; Maori people, Cook Islands	Anti-venom; blindness; anti-fungal	Kadir et al., 2015; Verger, 1995: cited in Noe and Lehmann, 2012 [H001; Whistler, 1985
Mitracarpus hirtus (L.) DC	Rubiaceae	Le, WP	Bas-Congo	Anti-fungal, anti-viral [*Herpes*]	Latham and Konda ku Mbuta, 2017
Mitragyna inermis (Willd.) K.Schum.	Rubiaceae	NS	Senegal	Memory improvement/ enhancement	Kerharo and Adam, 1974: cited in Noe and Lehmann, 2012 [H168]
Mitragyna parvifolia (Roxb.) Korth.	Rubiaceae	Le	Eastern Ghats, India	Anti-venom	Rao et al., 2006
Mitragyna rubrostipulata (Schum.) Hav.	Rubiaceae	Le	Rwanda	Anti-microbial/anti-paralytic [polio]	Van Puyvelde, 1977: cited in Noe and Lehmann, 2012 [H130]

Modiola caroliniana (L.) G.Don	Malvaceae	Ju	Mapuche people, Chile	Energy-boosting	Houghton and Manby, 1985
Mollugo cerviana (L.) Ser.	Molluginaceae	Le	Gingee Hills, Tamil Nadu, India	Anti-inflammatory	Arulappan et al., 2015
Mollugo nudicaulis Lam.	Molluginaceae	Le, WP	Kani people, Western Ghats, India	Anti-microbial [cough, STI]; anti-inflammatory [headache, Fe]	Ayyanar and Ignacimuthu, 2011
Momordica balsamina L.	Cucurbitaceae	AP	Benin	Memory improvement/ enhancement	Adjanohoun et al., 1989: cited in Noe and Lehmann, 2012 [H168]
Momordica charantia L.	Cucurbitaceae	FL, Le, St, WP	Mato Grosso, Brazil; Andoman + Nicobar Is., India; Tropical rain forest, Central + S Cameroon; Martinique; Trinidad; Benin; Cuba	Anti-microbial [c'pox, measles, *Herpes*, shingles, TB]; anti-hypertensive,; anti-inflammatory [Fe]; memory improvement/ enhancement	Ngono Ngane et al., 2011; Ribeiro et al., 2017; Longuefosse and Nossin, 1996; Clement et al., 2015; Chander et al., 2014; Adjanohoun et al., 1989: cited in Noe and Lehmann, 2012 [H168]
Monardella odoratissima Benth.	Lamiaceae	Br	Native America	Anti-inflammatory [eye inflam]	Train et al., 1941: Cited in Native American Ethnobotany Database
Mondia whitei (Hook.f.) Skeels	Apocynaceae	Se	Eastern Cape, South Africa	Memory improvement	Asowata-Ayodele et al., 2016
Moneses uniflora (L.) A.Gray	Ericaceae	NS	Native America	Anti-paralytic	Speck, 1917: cited in Native American Ethnobotany Database
Monodora myristica Dunal	Annonaceae	Se	Cataractes and Lukaya districts, D.R. Congo	Anti-bacterial; anti-inflammatory [Fe, pain, headache]	Latham and Konda ku Mbuta, 2017
Monolluma hexagona (Lavranos) Meve & Liede	Apocynaceae	St	Abyan territory, Yemen	Poison antidote; wound healing	Al-Fatimi, 2019
Monolluma quadrangula (Forssk.) Plowes	Apocynaceae	St	Abyan territory, Yemen	Anti-venom	Al-Fatimi, 2019
Monstera sp.	Araceae	NS	Conis Santana National Park, East Timor	Wound healing	Collins et al., 2007
Morina polyphylla Wall. ex DC.	Caprifoliaceae	Ro	Central Himalayas, Nepal	Limb numbness; anti-inflammatory [pain, headache]	Bhattarai et al., 2006
Morinda citrifolia L.	Rubiaceae	Fr, Le	Mato Grosso, Brazil; Andoman + Nicobar Is., India; Native Northern territory, Australia; Maori people, Cook Islands	Anti-microbial [sore throat, UTI]; anti-inflammatory; anti-paralytic; anti-hypertensive	Ribeiro et al., 2017; Chander et al., 2014; Smith, 1991; Whistler, 1985
Morinda lucida Benth.	Rubiaceae	St with Le	Togo; Cataractes and Lukaya districts, D.R. Congo	Memory improvement/ enhancement; anti-microbial [s'pox]; epilepsy; stroke	Adjanohoun et al., 1986: cited in Noe and Lehmann, 2012 [H168]; Latham and Konda ku Mbuta, 2017
Morinda pubescens Sm.		Le	Kani people, Western Ghats, India	Anti-inflammatory [rheum]; wound	Ayyanar and Ignacimuthu, 2011
Morinda umbellata L.	Rubiaceae	Le	Gingee Hills, Villupuram, Tamil Nadu	Anti-bacterial [dys]	Arulappan et al., 2015
Moringa oleifera Lam.	Moringaceae	Fr, Le, Ro, Se	Myanmar; Kimboza forest, Tanzania; 3 states in SW Nigeria; Nyere County, Centra	Anti-microbial [bronch, skin inf, STI]; tonic and strengthening; anti-inflammatory [rheum]; anti-	Maroyi, 2011; Potel, 2002: cited in Noe and Lehmann, 2012 [H130]; Amri and Kisangau,

			Kenya; Zimbabwe; Markets of Bogotá, Columbia; Maharashtra, India; Sundanese community, West Java; Ibadan city, SW Nigeria; Fiji	microbial/anti-paralytic; memory enhancement; anti-epileptic; wound healing	2012; Babawale et al., 2016; Kamau et al., 2016; Bussmann et al., 2018; DeFilipps and Krupnick, 2018; Wadankar et al., 2011; Roosita et al., 2008 Gbadamosi and Egunyomi, 2014; Singh, 1986
Morus alba L. Syn: *Morus multicaulis* (Perr.) Perr.	Moraceae	Ba, Fr, Le	Maonan people, Guangxi Zhuang, China; Hakka people, Guangdong, China; Trinidad; N + NE Bosnia and Herzegovina	Anti-hypertensive; anti-inflammatory [Fe, neuritis]; anti-microbial [sore throat]	Hong et al., 2015; Au et al., 2008; Clement et al., 2015; Saric-Kundalic et al., 2011
Morus nigra L.	Moraceae	Ba, Fr, Le, Ro	Mato Grosso, Brazil; Edremit Gulf, Turkey	Anti-microbial; anti-inflammatory [Fe]; cholesterol reducing	Ribeiro et al., 2017; Polat and Satıl, 2012
Mucuna pruriens (L.) DC.	Fabaceae	La, Ro, Se	Myanmar; Himalyas, India; South Nigeria; Cataractes and Lukaya districts, D.R. Congo; Bogotá markets, Columbia; Chittagong Hill Tracts, Bangladesh	Anti-microbial [cholera, dys, STI]; anti-paralytic; tissue regeneration + wound healing; anti-epileptic; anti-PD; anti-venom	Nordal, 1963: cited in DeFilipps and Krupnick, 2018; Mikawlrawng et al., 2017; Borokini et al., 2012; Latham and Konda ku Mbuta, 2017; Bussmann et al., 2018; Kadir et al., 2015
Mucuna stans Baker	Leguminosae	Le	Angola	Eye inflammation	Urso et al., 2016
Muehlenbeckia tamnifolia (Kunth) Meisn.	Polygonaceae	Le	Loja and Zamora-Chinchipe, Ecuador	Anti-microbial [influenza]	Tene et al., 2007
Multidentia fanshawei (Tennant) Bridson	Rubiaceae	Ro	Kimboza forest, Tanzania	Anti-inflammatory	Amri and Kisangau, 2012
Muntingia calabura L.	Muntingiaceae	NS	Markets of Bogotá, Columbia	Anti-microbial [mouth inf, dys]; anti-inflammatory [Fe]	Bussmann et al., 2018
Murraya paniculata (L.) Jack Syn: *Murraya exotica* L.	Rutaceae	Le	Caribbean coast of Columbia	Anti-inflammatory [Fe, pain]	Gómez-Estrada et al., 2011
Musa × paradisiaca L. Syn: *Musa × sapientum* L.	Musaceae	St	Angola; markets of Bogotá, Columbia; Ondo State, SW Nigeria; Maori people, Cook Islands	Memory improvement/ enhancement; anti-microbial [TB, diarrh, skin dis, *H. zoster*]; fatigue	Bossard, 1996: cited in Noe and Lehmann, 2012 [H168]; Bussmann et al., 2018; Mojisola et al., 2012; Whistler, 1985
Musa spp.	Musaceae	Fr, St	Mato Grosso, Brazil	Anti-microbial; anti-inflammatory	Ribeiro et al., 2017
Mussaenda philippica A.Rich	Rubiaceae	Le, Ro	Ayta communities, Bataan, Philippines	Anti-microbial [sore throat]	Tantengco et al., 2018
Muntingia calabura L.	Muntingiaceae	Fr	Mestizo community, North Peru	Anti-microbial	Bussmann and Sharon, 2006
Myrsine umbellata Mart	Primulaceae	Le	Tacana people, Bolivian Amazon	Anti-inflammatory [rheum];	Bourdy et al., 2000
Myrcianthes discolor (Kunth) McVaugh	Myrtaceae	WP	Mestizo community, North Peru	Anti-inflammatory	Bussmann and Sharon, 2006
Myrcianthes leucoxyla (Ortega) McVaugh.	Myrtaceae	NS	Markets of Bogotá, Columbia	Anti-inflammatory [rheum]; anti-microbial diarrh, dys]; nervous stimulant	Bussmann et al., 2018

Myrciaria dubia (Kunth) McVaugh	Myrtaceae	Le	Mato Grosso, Brazil	Anti-microbial	Ribeiro et al., 2017
Myrianthus arboreus P.Beauv.	Urticaceae	Le	Mount Cameroon, Cameroon	Anti-microbial [dys]	Sandberg et al., 2005
Myrica esculenta Buch.-Ham. Ex D.Don	Myricaceae	Ba, Fr	Himachal Pradesh, NW Himalaya, India	Anti-microbial [dys, bronch]	Vidyarthi et al., 2013
Myristica fragrans Houtt.	Myristicaceae	Se	Martinique; Mato Grosso, Brazil; Myanmar	Anti-inflammatory [Fe]	Longuefosse and Nossin, 1996; Ribeiro et al., 2017; DeFilipps and Krupnick, 2018
Myroxylon balsamum (L.) Harms	Leguminosae	Ba	Loja and Zamora-Chinchipe, Ecuador	Anti-microbial [int/ext inf]	Tene et al., 2007
Myroxylon peruiferum L. f.	Leguminosae	NS	Markets of Bogotá, Columbia	anti-microbial [TB]	Bussmann et al., 2018
Myrtus communis L.	Myrtaceae	AP, Le, FL, Fr	Mashhad markets, Iran; Oman; M'Sila, North Algeria	Anti-inflammatory [pain]; anti-microbial [*Herpes*]; anti-hypertensive	Amiri and Joharchi, 2013; Mosaddegh et al., 2013; Boudjelal et al., 2013
Myrtus nivelii Batt. & Trab.	Myrtaceae	Le	Tassili N'Ajjer, Southern Algerian Sahara	Anti-inflammatory [Fe]	Hammiche and Maiza, 2006
Nandina domestica Thunb.	Berberidaceae	Fr, Ro, St	Maonan people, Guangxi Zhuang, China	Anti-inflammatory	Hong et al., 2015
Napoleonaea vogelii Hook. & Planch.	Lecythidaceae	Ba, Fr, Le, Se	Bas-Congo	Anti-inflammatory [Fe]	Latham and Konda ku Mbuta, 2017
Nasa loxensis (Kunth) Weigend	Loasaceae	Ro	Loja and Zamora-Chinchipe, Ecuador	Anti-microbial [int inf]	Tene et al., 2007
Nasturtium officinale W.T. Aiton Syn: *Rorippa nasturtium-aquaticum* (L.) Hayek	Brassicaceae	St, WP	Loja and Zamora-Chinchipe, Ecuador; Três Ladeiras people, Atlantic Forest, Brazil; Mestizo community, North Peru; Potosi, Bolivia	Anti-microbial [pneum, influenza, bronch, TB]; anti-hypertensive	Tene et al., 2007; Gazzaneo et al., 2005; Bussmann and Sharon, 2006; Fernandez et al., 2003
Nauclea diderrichii (De Wild.) Merr.	Rubiaceae	Wo	Baka Pygmies, Gabon	Anti-microbial [diarrh]	Betti et al., 2013
Nauclea latifolia Sm. Syn: *Sarcocephalus latifolius*	Rubiaceae	Ba, Ro	Cataractes and Lukaya districts, D.R. Congo; Congo-Brazzaville; Togo	Paralysis/ hemiplegia/ polio/ paraplegia; memory improvement /enhancement; anti-inflammatory [Fe, pain]; anti-microbial [diarrh]; TBI	Diafouka, 1997, Adjanohoun et al., 1989: cited in Noe and Lehmann, 2012 [H130, H168]; Latham and Konda ku Mbuta, 2017; Kantati et al., 2016
Neea sp.	Nyctaginaceae	Le	Peruvian Amazon	Anti-microbial [abscess]	Odonne et al., 2013
Neopicrorhiza scrophulariiflora (Pennell) D.Y.Hong Syn*: Picrorhiza scrophulariiflora* Pennell	Plantaginaceae	Ro	Central Himalayas, Nepal	Anti-paralytic; anti-microbial [typhoid]; anti-inflammatory [Fe]; anti-venom	Bhattarai et al., 2006
Nepenthes ampullaria Jack	Nepenthaceae	Ro	Dayaknese Communities, W Kalimantan, Indonesia	Anti-microbial [yell fev]	Diba et al., 2013
Nepeta cataria L.	Lamiaceae	WP	Maonan people, Guangxi Zhuang, China	Anti-microbial	Hong et al., 2015

Nepeta erecta (Royle ex Benth.) Benth.	Lamiaceae	Le	Toli Peer National Park, Kashmir, Pakistan	Anti-microbial [cough, cold, influenza]; anti-inflammatory [Fe, toothache]	Amjad et al., 2017
Nepeta glomerulosa Boiss.	Lamiaceae	AP	Iran	Anti-viral [cold]	Mosaddegh et al., 2013
Nephrolepis auriculata (L.) Trimen [unresolved]	Nephrolepidaceae	Le, Tu	Magar and Majhi people, Nepal	Anti-inflammatory; anti-hypertensive	Malla et al., 2015
Nephrolepis biserrata (Sw.) Schott	Nephrolepidaceae	Le	Dayaknese Communities, W Kalimantan, Indonesia	Wound healing	Diba et al., 2013
Nephrolepis cordifolia (L.) C. Presl	Nephrolepidaceae	Le, Rh, WP	Maonan people, Guangxi Zhuang, China	Anti-inflammatory	Hong et al., 2015
Nervilia plicata (Andrews) Schltr.	Orchidaceae	Le	Chin people, Myanmar	Anti-microbial [*otitis media*]	Ong et al., 2018
Neurolaena lobata (L.) R.Br. ex Cass.	Asteraceae	Ba, Le	Trinidad; Guaymi indians, Panama; Antioquia, Columbia	Anti-inflammatory; anti-microbial [cold]; anti-hypertensive; anti-venom	Clement et al., 2015; Joly et al., 1987; Vasquez et al., 2013
Newbouldia laevis (P.Beauv.) Seem. ex Bureau	Bignoniaceae	Le	Cataractes and Lukaya districts, D.R. Congo	Anti-microbial [dental caries]	Latham and Konda ku Mbuta, 2017
Nigella sativa L.	Ranunculaceae	Se	Malaysia	Anti-inflammatory [rheum]; anti-microbial [abscess, septicaemia]	DeFilipps and Krupnick, 2018
Niphidium crassifolium (L.) Lellinger Syn: *Polypodium crassifolium* L.	Polypodiaceae	St	Mestizo community, North Peru	Anti-inflammatory	Bussmann and Sharon, 2006
Niphogeton ternata (Willd. Ex Schltr.) Mathias & Constance	Apiaceae	NS	Markets of Bogotá, Columbia	Anti-microbial [dys]	Bussmann et al., 2018
Nothofagus dombeyi (Mirb.) Oerst.	Nothofagaceae	NS	Mapuche people, Argentina	Anti-inflammatory	Estomba et al., 2005
Notopleura uliginosa (Sw.) Bremek. Syn: *Psychotria uliginosa* Sw.	Rubiaceae	Le	Guaymi indians, Panama	Anti-inflammatory [Fe]	Joly et al., 1987
Nyctanthes arbor-tristis L.	Oleaceae	Ba, FL, Le	Myanmar	Anti-venom; anti- inflammatory [Fe]	Nordal, 1963: cited in; DeFilipps and Krupnick, 2018
Nymphaea lotus L.	Nymphaeaceae	NS	D.R.Congo; Côte d'Ivoire	Leg weakness; delay of motor function	Arkinstall, 1979: cited in Noe and Lehmann, 2012 [H130]; Malan et al., 2015
Nymphaea nouchali Burm.f. Syn: *Nymphaea stellata* Burm. F.	Nymphaeaceae	FL, St, Tu [in spp. Comb]	3 Kerala tribes, Western Ghats, India	Anti-aging	Marjana et al., 2018
Ochanostachys amentacea Mast.	Olacaceae	Le	Seberida, Riau Province, Sumatra, Indonesia	Anti-inflammatory	Mahyar et al., 1991
Ochna afzelii R.Br. ex Oliv.	Ochnaceae	Ba, Ro, St	Cataractes and Lukaya districts, D.R. Congo	Anti-microbial [dys, skin inf], anti-inflammatory [periodontitis]	Latham and Konda ku Mbuta, 2017
Ochradenus baccatus Delile	Resedaceae	AP, Se	Iran	Anti-viral [cold]	Mosaddegh et al., 2013
Ochroma pyramidale (Cav. Ex Lam.) Urb.	Malvaceae	NS	Markets of Bogotá, Columbia	Anti-microbial [influenza]	Bussmann et al., 2018

Ocimum americanum L. Syn: *Ocimum canum* Sims	Lamiaceae	Le, St	D.R. Congo; Togo	Memory improvement/ enhancement; anti-epileptic; anti-stroke	Adjanohoun et al., 1986: cited in Noe and Lehmann, 2012 [H168]; Kantati et al., 2016
Ocimum basilicum L.	Lamiaceae	FL br; Le	Ecuador; Martinique; Marquesas Islands, French Polynesia; Abyan territory, Yemen; Tigrigna people, Central Eritrea; Maori people, Cook Islands; Agro Nocerino Sarnese, Southern Italy; Edremit Gulf, Turkey	Anti-microbial [int inf, UTI, cold, influenza]; anti-inflammatory [Fe, headache, asthma]; anti-hypertensive	Longuefosse and Nossin, 1996; Girardi et al., 2015; Al-Fatimi, 2019; Yemane et al., 2017; Motti and Motti, 2017; Polat and Satıl, 2012
Ocimum campechianum Mill.	Lamiaceae	Le	Rio Jauaperi, Brazilian Amazon	Anti-viral [influenza]	Pedrollo et al., 2016
Ocimum carnosum (Spreng.) Link & Otto ex Benth.	Lamiaceae	Le	Mato Grosso, Brazil	Anti-viral [influenza]	Ribeiro et al., 2017
Ocimum filamentosum Forssk. Syn*:* *Becium filamentosum*	Lamiaceae	Le, St	Afar people, Rift Valley, Ethiopia	Anti-microbial [eye/lung inf, STI]	Teklehaymanot, 2017
Ocimum campechianum Mill. Syn: *Ocimum micranthum* Willd	Lamiaceae	Le	Tacana people, Bolivian Amazon	Anti-inflammatory [Fe	Bourdy et al., 2000
Ocimum tenuiflorum L Syn: *Ocimum sanctum* L.	Lamiaceae	Le, Ro	India: Himachal Pradesh, Maharashtra,; Andoman + Nicobar Is., Western Ghats; Bataan, Philippines	Anti-inflammatory [Fe]; anti-microbial [dys]; enhanced memory; anti-paralytic; wound healing	Vidyarthi et al., 2013; Tantengco et al., 2018; Chander et al., 2014;; Wadankar et al., 2011
Ocotea usambarensis Engl.	Lauraceae	Ba, Ro	Kimboza forest, Tanzania	Anti-inflammatory	Amri and Kisangau, 2012
Odontonema tubaeforme (Bertol.) Kuntze.	Acanthaceae	Le	Guaymi indians, Panama	Anti-inflammatory	Joly et al., 1990
Oenanthe javanica (Blume) DC.	Apiaceae	WP	Maonan people, Guangxi Zhuang, China	Anti-hypertensive	Hong et al., 2015
Oenocarpus bataua Mart. Syn: *Jessenia bataua* (Mart.) Burret	Arecaceae	Se	Bolivian Amazon	Anti-microbial [measles, bronch]; anti-inflammatory [Fe]	Bourdy et al., 2000
Olax gambecola Baill.	Olacaceae	NS	Cataractes and Lukaya districts, D.R. Congo	Anti-microbial [pneum, leprosy]	Latham and Konda ku Mbuta, 2017
Olea europaea L.	Oleaceae	AP, Fr, Fr oil, Le	Iran; M'Sila, North Algeria; Taounate, North Morocco; High Atlas, Morocco; Catalonia, Spain; Agro Nocerino Sarnese, Southern Italy	Anti-hypertensive; hypercholesterolaemia; anti-microbial [Otitis, tons]; anxiolytic	Mosaddegh et al., 2018; Boudjelal et al., 2013; El-Hilaly et al., 2003; Fadili *et al*, 2017; Bonet et al., 1999; Raja et al., 1997; Motti and Motti, 2017
Operculina hamiltonii (G. Don) D.F. Austin & Staples Syn: *Operculina alata* (Ham.) Urb.	Convolvulaceae	Le, Rh	Três Ladeiras people, Atlantic Forest + Mato Grosso, Brazil	Anti-inflammatory; anti-microbial [influenza]	Gazzaneo et al., 2005; Ribeiro et al., 2017
Ophioglossum reticulatum L.	Ophioglossaceae	WP	Maonan people, Guangxi Zhuang, China	Anti-microbial	Hong et al., 2015
Ophryosporus axilliflorus (Griseb.) Hieron.	Asteraceae	Le	Comechingones, Argentina	Anti-microbial [STI]	Goleniowski et al., 2006
Opuntia albispinosa Mieckley ex Lindemuth & Dams	Cactaceae	Fr	Potosi, Bolivia	Anti-inflammatory [Fe]	Fernandez et al., 2003

Opuntia cochenillifera (L.) Mill.	Cactaceae	Le	Martinique	Anti-inflammatory	Longuefosse and Nossin, 1996
Opuntia dillenii (Ker Gawl.) Haw.	Cactaceae	Sh	Kani people, Western Ghats, India; Dinajpur District, Bangladesh	Wound healing; anti-paralytic	Ayyanar and Ignacimuthu, 2011; Rahmatullah et al., 2009
Opuntia ficus-indica (L.) Mill.	Cactaceae	Fr, Le, St	Nuevo León, Mexico; Abyan territory, Yemen; Agro Nocerino Sarnese, Southern Italy	Anti-inflammatory; cholesterol-lowering; wound healing; anti-microbial [whooping cough]	Estrada-Castillón et al., 2012; Al-Fatimi, 2019; Motti and Motti, 2017
Opuntia humifusa (Raf.) Raf. Syn: *Opuntia vulgaris* J. Miller	Cactaceae	Ro	Venda, South Africa	Anti-microbial [STI]	Arnold and Gulumian, 1984
Opuntia minor Müll. Hal.	Cactaceae	Fr	Potosi, Bolivia	Anti-microbial [pneum]	Fernandez et al., 2003
Orbivestus karaguensis (Oliv. & Hiern) H.Rob. Syn: *Vernonia karaguensis* Oliv. & Hiern	Compositae	Le	Burundi	Eye inflammation	Baerts and Lehmann, 1989: cited in Noe and Lehmann, 2012 [H001]
Oreocallis grandiflora (Lam.) R.Br.	Proteaceae	Ba, FL, Le	Loja and Zamora-Chinchipe, Ecuador; Mestizo community, North Peru	Anti-inflammatory [Fe]	Tene et al., 2007; Bussmann and Sharon, 2006
Oreopanax malacotrichus Harms Syn: *Oreopanax eriocephalus* Harms	Araliaceae	FL, Le	Mestizo community, North Peru	Anti-inflammatory	Bussmann and Sharon, 2006
Oreopanax sp.	Araliaceae	Le	Loja and Zamora-Chinchipe, Ecuador	Anti-microbial [int/ext inf]	Tene et al., 2007
Origanum majorana L	Lamiaceae	AP, Br	Taounate, North Morocco; Bolicar, N Colombia	Anti-inflammatory [Fe]; anti-microbial [cough]; anxiolytic	El-Hilaly et al., 2003; Gómez-Estrada et al., 2011
Origanum onites L	Lamiaceae	FL br	Edremit Gulf, Turkey	Anti-inflammatory [headache, toothache]	Polat and Satıl, 2012
Origanum vulgare L.	Lamiaceae	AP, Le, Ro, WP	Central Himalayas, Nepal; Naxi people, Northwest Yunnan, China; Mapuche people, Argentina; Middle East; Turkestan Range of S Kyrgystan; Gheg people, NE Albania; M'Sila, North Algeria; Yörük people, SE Macedonia	Anti-microbial [TB, boi, influenza; sore throat], cough; anti-inflammatory [headache]; anti-paralytic; anti-hypertensive; fatigue, weakness	Bhattarai et al., 2006; Zhang et el., 2015; Estomba et al., 2005; Vidyarthi et al., 2013; Abu-Rabia, 2012; Pawera et al., 2016; Pieroni and Sõukand, 2017; Boudjelal et al., 2013; Nedelcheva et al., 2017
Oroxylum indicum (L.) Kurz	Bignoniaceae	Ba, Fr, Le, Ro ba	Mao Naga people Manipur, India; Bac Huong Hoa nature reserve, Vietnam; Mizoram, India	Anti-microbial; anti-hypertensive; anti-inflammatory [Fe], rheum; anti-venom; anxiolytic	Lokho, 2012; Ong et al., 2018; Lee et al., 2019; Rai and Lalramnghinglova, 2010
Orthosiphon pallidus Royle ex Benth.	Lamiaceae	Le	Afar people, Rift Valley, Ethiopia	Anti-microbial [diphth]	Teklehaymanot, 2017
Oryctanthus alveolatus (Kunth) Kuijt	Loranthaceae	NS	Markets of Bogotá, Columbia	Anti-hypertensive; anti-microbial [ton'is]	Bussmann et al., 2018
Otoba parvifolia (Markgr.) A.H.Gentry	[Myristicaceae	Re	Tacana people, Bolivian Amazon	Anti-inflammatory [rheum]	Bourdy et al., 2000
Ottelia alismoides (L.) Pers.	Hydrocharitaceae	Le	Eastern Ghats, India	Anti-venom	Rao et al., 2006

Ottelia ulvifolia (Planch.) Walp.	Hydrocharitaceae	St	Bas-Congo Province, D.R. Congo	Anti-PD	Kembelo, 2003: cited in Noe and Lehmann, 2012 [H130x]
Oxalis adenophylla Gillies ex Hook. & Arn.	Oxalidaceae	NS	Mapuche people, Argentina	Anti-inflammatory	Estomba et al., 2005
Oxalis articulata Savigny	Oxalidaceae	Le	Satkhira District, Kalaroa, Bangladesh	Anti-microbial [dys]	Dulla and Jahan, 2017
Oxalis corniculata L.	Oxalidaceae	Le	Mapuche people, Chile; Markets of Bogotá, Columbia; 3 Kerala tribes, Western Ghats, India; Himachal Pradesh, NW Himalaya, India; Uttarakhand, India; Chittagong Hill Tracts, Bangladesh; Cataractes and Lukaya districts, D.R. Congo; Ivory Coast; Maori people, Cook Islands	Anti-inflammatory; anti-eye inflammation; anti-microbial [dys, boil, anti--fungal]; anti-venom; anti-epileptic	Houghton and Manby, 1985; Adjanohoun and Ake Assi, 1979: cited in Noe and Lehmann, 2012 [H001]; Bussmann et al., 2018; Marjana et al., 2018; Vidyarthi et al., 2013 Latham and Konda ku Mbuta, 2017; Kadir et al., 2015; Sharma et al., 2013; Whistler, 1985
Oxalis frutescens (Kunth) Lourteig	Oxalidaceae	Le	Martinique	Anti-microbial	Longuefosse and Nossin, 1996
Oxalis peduncularis Kunth	Oxalidaceae	Le	Loja and Zamora-Chinchipe, Ecuador	Anti-inflammatory [Fe]	Tene et al., 2007
Oxalis succulenta Barn.	Oxalidaceae	Ju	Mapuche people, Chile	Anti-inflammatory [Fe, headache]	Houghton and Manby, 1985
Oxygonum sinuatum (Hochst. & Steud ex Meisn.) Dammer	Polygonaceae	Le	Kenya	Eye inflammation	Kokwaro, 1976: cited in Noe and Lehmann, 2012 [H001]
Oxytenanthera abyssinica (A.Rich.) Munro	Poaceae	Le	Togo	Anti-stroke	Kantati et al., 2016
Ozoroa insignis Del.	Anacardiaceae	Ro	Zimbabwe	Anti-microbial [STI, diarrh]	Maroyi, 2011
Ozoroa pulcherrima (Schweinf.) R.Fern. & A.Fern.	Anacardiaceae	NS	Benin	Eye inflammation	Adjanohoun et al., 1989: in Noe and Lehmann, 2012 [H001]
Paederia foetida L.	Rubiaceae	Ju, Le	Myanmar; Mizoram, India	Anti-paralytic; anti-inflammatory [rheum]; anxiolytic; anti-microbial *[Herpes*]	Perry and Metzger, 1980; DeFilipps and Krupnick, 2018; Rai and Lalramnghinglova, 2010
Paeonia lactiflora Pall.	Paeoniaceae	Le, Ro	Tian Mu Shan Biosphere Reserve, Zhejiang, China	Anti-inflammatory	Chaudhary et al., 2006
Paeonia suffruticosa Andrews	Paeoniaceae	Ro ba	Huanjiang Maonan people, Guangxi Zhuang, China	Anti-microbial [pneumo]	Hong et al., 2015
Palicourea crocea (Sw.) Schult.	Rubiaceae	Le, Ro	Mato Grosso, Brazil	Anti-microbial; anti-hypertensive	Ribeiro et al., 2017
Paliurus spina-christi Mill.	[Rhamnaceae	Fr	Catalonia, Spain; Edremit Gulf, Turkey	Anti-hypercholesteremic; anti-microbial [cold, influenza]; anti-inflammatory [asthma]; anxiolytic	Bonet et al., 1999; Polat and Satıl, 2012
Panax japonicus (T.Nees) C.A.Mey.	Araliaceae	Ro	Shaanxi, China	support immune system; anti-inflammatory [rheum]; anti-hypertensive	Teng et al., 2011

Panax quinquefolius L.	Araliaceae	Ro	Shaanxi, China	support immune system, weakness/fatigue, anti-inflammatory [Fe]	Teng et al., 2011
Pandanus amaryllifolius Roxb.	Pandanaceae	Le	Ayta communities, Bataan, Philippines; Sundanese community, West Java	Anti-hypertensive; anti-bacterial [typhus]	Tantengco et al., 2018; Roosita et al., 2008
Pandanus sp.	Pandanaceae	Le	Ayta communities, Bataan, Philippines	Anti-microbial [UTI]	Tantengco et al., 2018
Pandanus tectorius Parkinson ex Du Roi	Pandanaceae	Fr	Maori people, Cook Islands	Anti-microbial [UTI]	Whistler, 1985
Papaver dubium L	Papaveraceae	FL	Jammu + Kashmir, India	Memory improvement	Lone and Bhardwaj, 2013
Papaver rhoeas L	Papaveraceae	AP	Northeastern Dahra Mountains, NW Algeria	Memory improvement	Senouci et al., 2019
Paranephelius uniflorus Poepp. & Endl.	Compositae	WP	Mestizo community, North Peru	Anti-inflammatory	Bussmann and Sharon, 2006
Parietaria judaica L.	Urticaceae	AP	Agro Nocerino Sarnese, Southern Italy	Anti-inflammatory [rheum]	Motti and Motti, 2017
Parietaria officinalis L.	Urticaceae	NS	Markets of Bogotá, Columbia	Anti-microbial [UTI]	Bussmann et al., 2018
Parinari curatellifolia Planch. ex Benth.	Chrysobalanaceae	Le, Ro, St	Togo; Venda, South Africa; Zimbabwe	Memory improvement/ enhancement; anti-microbial [STI]; anti-inflammatory [toothache]	Adjanohoun et al., 1986: cited in Noe and Lehmann, 2012 [H168]; Arnold and Gulumian, 1984; Maroyi, 2011
Parinari excelsa Sabine	Chrysobalanaceae	Ro	Senegal	Memory improvement/ enhancement	Kerharo and Adam, 1974: cited in Noe and Lehmann, 2012 [H168]
Paris polyphylla Smith.	Melanthiaceae	Ro	Shaanxi, China	Anti-fungal, anti-virus [influenza]	Teng et al., 2011
Parkia biglobosa (Jacq.) R.Br. ex G.Don	Fabaceae	St ba	Togo	Anti-epileptic	Kantati et al., 2016
Parkinsonia aculeata L.	Fabaceae	NS	Markets of Bogotá, Columbia	Anti-inflammatory [Fe]	Bussmann et al., 2018
Parnassia nubicola Hk. f.	Celastraceae	Ro	Himachal Pradesh, NW Himalaya, India	Anti-inflammatory	Vidyarthi et al., 2013
Parochetus communis Buch.-Ham. ex D. Don	Fabaceae	WP	Magar and Majhi people, Nepal	Anti-microbial [boil]	Malla et al., 2015
Parthenium hysterophorus L.	Asteraceae	AP, FL, Le	Comechingones, Argentina; Martinique	Anti-inflammatory	Goleniowski et al., 2006; Longuefosse and Nossin, 1996
Paspalum conjugatum P.J.Bergius	Poaceae	AP	Marquesas Islands, French Polynesia	Anti-microbial	Girardi et al., 2015
Paspalum melanospermum Desv. ex Poir.	Poaceae	Le	Martinique	Anti-inflammatory	Longuefosse and Nossin, 1996
Paspalum notatum Flüggé	Poaceae	Ro	Comechingones, Argentina	Anti-microbial [STI]	Goleniowski et al., 2006
Passiflora edulis Sims Syn: *Passiflora incarnata* L.	Passifloraceae	FR, Le	Martinique; Trinidad; Mao Naga people, Manipur, India; Uganda	Anti-hypertensive; anti-microbial [dys]; weakness	Longuefosse and Nossin, 1996; Clement et al., 2015; Lokho, 2012; Tugume et al., 2016: cited in Noe and Lehmann, 2012

Passiflora foetida L.	Passifloraceae	Le	Maromizaha forest, Madagascar; Guinea-Bissau	Anti-hypertensive; anti-depressant	Riondato et al., 2019; Romeiras et al., 2012
Passiflora ligularis Jus.	Passifloraceae	FL, Le	Loja and Zamora-Chinchipe, Ecuador	Anti-inflammatory; cholesterol-reducing; anti-hypertensive	Tene et al., 2007
Passiflora quadrangularis L.	Passifloraceae	Fr, Le	Mato Grosso, Brazi; Trinidad	Anti-hypertensive; cholesterol-reducing	Ribeiro et al., 2017; Clement et al., 2015
Paullinia cupana Kunth	Sapindaceae	Se	Rio Jauaperi, Brazilian Amazon	Anti-microbial	Pedrollo et al., 2016
Paullinia pinnata L.	Sapindaceae	Ro	Tanzania; Cataractes and Lukaya districts, D.R. Congo	Anti-paralytic, anti-microbial [STI, dys]	Kokwaro, 1976: cited in Noe and Lehmann, 2012 [H130]; Latham and Konda ku Mbuta, 2017
Pauridiantha paucinervis (Hiern) Bremek.	Rubiaceae	Ba	Maromizaha forest, Madagascar	Anti-fatigue	Riondato et al., 2019
Pavetta refractifolia K.Schum.	Rubiaceae	AP [in sp comb]	Haya people, Kagera, NW Tanzania	Mental confusion	Moshi et al., 2009
Peganum harmala L.	Nitrariaceae	AP, Le, Ro, Se	Abyan territory, Yemen; Turkestan Range of south Kyrgystan; M'Sila, North Algeria; Tassili N'Ajjer, Southern Algerian Sahara; High Atlas, Morocco	Anti-inflammatory [asthma, Fe, rheum, headache]; anti-hypertensive; anxiolytic	Al-Fatimi, 2019; Pawera et al., 2016; Boudjelal et al., 2013; Hammiche and Maiza, 2006; Fadili *et al*, 2017
Pelargonium graveolens L'Hér.	Geraniaceae	FL	Loja and Zamora-Chinchipe, Ecuador	Anti-microbial [conj]	Tene et al., 2007
Pelargonium odoratissimum (L.) L'Herit.	Geraniaceae	Le, WP	Loja and Zamora-Chinchipe, Ecuador; Mestizo community, North Peru; Taounate, North Morocco	Anti-inflammatory; anti-microbial [cough]	Tene et al., 2007; Bussmann and Sharon, 2006; El-Hilaly et al., 2003
Pelargonium peltatum (L.) L'Hér.	Geraniaceae	NS	Markets of Bogotá, Columbia	Anti-microbial [sore throat]	Bussmann et al., 2018
Pelargonium roseum Willd.	Geraniaceae	FL, Le	Mestizo community, North Peru	Anti-inflammatory; anti-microbial	Bussmann and Sharon, 2006
Pelargonium zonale (L.) L'Hér.	Geraniaceae	FL	Loja and Zamora-Chinchipe, Ecuador	Anti-microbial [influenza]	Tene et al., 2007
Peltogyne paniculata Benth	Leguminosae	St ba	Rio Jauaperi, Brazilian Amazon	Anti-microbial	Pedrollo et al., 2016
Peltophorum africanum Sonder	Euphorbiaceae	Ba, Ro	Venda, South Africa	Anti-microbial [STI, cough]	Arnold and Gulumian, 1984
Pennisetum glaucum (L.) R.Br.	Poaceae	Se	Abyan territory, Yemen	Boost immune system	Al-Fatimi, 2019
Pentacalia corymbosa (Benth.) Cuatrec.	Compositae	NS	Markets of Bogotá, Columbia	Anti-inflammatory	Bussmann et al., 2018
Pentacalia sp.	Compositae	Le, Ro, St	Loja and Zamora-Chinchipe, Ecuador	Anti-microbial [influenza]	Tene et al., 2007
Pentadesma butyracea Sabine	Clusiceae	Fr	Bassila + Toucountouna regions, Benin	Anti-inflammatory; anti-microbial	Avocèvou-Ayisso et al., 2013

Pentadiplandra brazzeana Baill.	Pentadiplandraceae	Ba	Congo-Brazzaville; Cataractes and Lukaya districts, D.R. Congo	Paralysis/ hemiplegia/ polio/ paraplegia, anti-inflammatory	Diafouka, 1997: cited in Noe and Lehmann, 2012 [H130]; Latham and Konda ku Mbuta, 2017
Pentas schimperiana subsp. *occidentalis* (Hook.f.) Verdc.	Rubiaceae	Ro ju	Debre Libanos Wereda, central Ethiopia	Anti-venom	Getaneh and, Girma, 2014
Pentasachme caudatum Wall. ex Wight Syn: *Pentasacme championii* Benth.	Apocynaceae	WP	Maonan people, Guangxi Zhuang, China	Anti-microbial	Hong et al., 2015
Peperomia garcia-barrigana Trel. & Yunck.	Piperaceae	NS	Markets of Bogotá, Columbia	Anti-microbial [influenza]	Bussmann et al., 2018
Peperomia pellucida (L.) Kunth	Piperaceae	Le	Martinique; Trinidad; Maranao people, Philippines; SW Nigeria; Chittagong Hill Tracts, Bangladesh	Anti-inflammatory; anti-microbial [influenza, UTI, eye inf]; anti-hypertensive; anti-aging; anti-venom	Longuefosse and Nossin, 1996; Clement et al., 2015; Malawani et al., 2017; Elufioye et al., 2012; Kadir et al., 2015
Perezia pungens Less.	Compositae	Le	Mestizo community, North Peru	Anti-microbial	Bussmann and Sharon, 2006
Pergularia daemia (Forssk.) Chiov. Syn: *Pergularia extensa* (Jacq.) N.E. Br.	Apocynaceae	Le	Togo	Anti-dementia, anti-epileptic	Kantati et al., 2016
Perilla frutescens (L.) Britton	Lamiaceae	Se, WP	Huanjiang Maonan people, Guangxi Zhuang, China	Anti-microbial [cough, cold]	Hong et al., 2015
*Peristrophe bicalyculata (*Retz.) Nees	Acanthaceae	Le	Eastern Ghats, India; Myanmar	Anti-inflammatory [asthma]; anti-venom	Rao et al., 2006; DeFilipps and Krupnick, 2018
Perovskia scrophulariifolia Bunge	Lamiaceae	Le	Turkestan Range of south Kyrgystan	Anti-hypertensive, wound healing	Pawera et al., 2016
Persea americana Mill.	Lauraceae	Fr, Le	Loja and Zamora-Chinchipe, Ecuador; Trinidad; Markets of Bogotá, Columbia; Três Ladeiras people, Atlantic Forest, Brazil; Congo-Brazzaville; Fundong, NW Cameroon; Togo	Anti-inflammatory [rheum]; anti-microbial [influenza, bronch, abscess, dys]; anti-hypertensive; paralysis/ hemiplegia/ polio/ paraplegia; stroke	Tene et al., 2007; Clement et al., 2015; Bussmann et al., 2018; Gazzaneo et al., 2005; Diafouka, 1997: cited in Noe and Lehmann, 2012 [H130]; Focho et al.,2009; Kantati et al., 2016
Persicaria acuminata (Kunth) M.Gómez Syn: *Polygonum acuminatum* Kunth.	Polygonaceae	Le	Comechingones, Argentina	Anti-microbial [STI]	Goleniowski et al., 2006
Persicaria bistorta (L.) Samp. Syn: *Polygonum bistorta* L.	Polygonaceae	Le	Yörük people, SE Macedonia	Anti-venom	Nedelcheva et al., 2017
Persicaria hydropiper (L.) Delarbre Syn: *Polygonum hydropiper* (L.) Delarbre	Polygonaceae	WP	Maonan people, Guangxi Zhuang, China	Anti-microbial [typhoid]; anti-inflammatory [rheum]	Hong et al., 2015
Persicaria orientalis (L.) Spach. *Syn: Polygonum orientale* L.	Polygonaceae	Le	Mao Naga people Manipur, India	Anti-microbial [dys]	Lokho, 2012
Persicaria punctata (Elliott) Small Syn: *Polygonum punctatum* Elliott	Polygonaceae	Le, WP	Mato Grosso, Brazil	Anti-inflammatory; anti-hypertensive	Ribeiro et al., 2017

Petiveria alliacea L.	Phytolaccaceae	Ro	Tacana people, Bolivian Amazon	Anti-inflammatory [rheum]	Bourdy et al., 2000
Petroselinum crispum (Miller) A.W. Hill	Apiaceae	Le, Wp	Mestizo community, North Peru; Markets of Bogotá, Columbia	Anti-microbial; anti-hypertensive; anti-inflammatory [dysmenorrhoea]	Bussmann and Sharon, 2006; Bussmann et al., 2018; Cadena-González et al., 2013
Peumus boldus Molina	Monimiacieae	NS	Mestizo community, North Peru; Chile; Markets of Bogotá, Columbia	Anti-inflammatory; anti-microbial [STI, kidney inf]	Bussmann and Sharon, 2006; Bussmann et al., 2018
Phenakospermum guyannense (A.Rich.) Endl. ex Miq.	Strelitziaceae	Le	Rio Jauaperi, Brazilian Amazon	Anti-microbial [dys]	Pedrollo et al., 2016
Philodendron camposportoanum G.M.Barroso	Araceae	Ro	Tacana people, Bolivian Amazon	Anti-venom	Bourdy et al., 2000
Philodendron imbe Schott ex Kunth	Araceae	Le, Ro	Mato Grosso, Brazil	Anti-microbial; anti-inflammatory	Ribeiro et al., 2017
Phlebodium aureum (L.) J. Sm.	Polypodiaceae	NS	Markets of Bogotá, Columbia	Anti- hypertensive	Bussmann et al., 2018
Phlogacanthus thyrsiformis (Roxb. ex Hardw.) Mabb	Acanthaceae	Le	Eastern Himalaya; Arunachal Pradesh, India	Anti-paralytic	Tangjang et al., 2011
Phoenix sylvestris (L.) Roxb.	Arecaceae	Fr, Ro, sap	Satkhira District, Kalaroa, Bangladesh	Nervous debility; anti-microbial [STI; anti-inflammatory [Fe, pain]	Dulla and Jahan, 2017
Pholidota chinensis Lindl.	Orchidaceae	WP	Hakka people, Guangdong, China; Maonan people, Guangxi Zhuang, China; Kani people, Western Ghats, India	Anti-inflammatory; anti-microbial TB]; anti-fatigue	Au et al., 2008; Hong et al., 2015; Ayyanar and Ignacimuthu, 2011
Phragmanthera usuiensis (Oliv.) M. G. Gilbert	Loranthaceae	Ba	Mont Elgon, Kenya	Anti-paralytic	Okelo et al., 2010: cited in Noe and Lehmann, 2012 [H130]
Phthirusa pyrifolia (Kunth) Eichler	Loranthaceae	Le	Três Ladeiras people, Atlantic Forest, Brazil	Anti-inflammatory	Gazzaneo et al., 2005
Phyla scaberrima (Juss. ex Pers.) Moldenke Syn: *Phyla dulcis* (Trevir.) Moldenke	Verbenaceae	NS	Markets of Bogotá, Columbia	Anti-inflammatory [Fe]	Bussmann et al., 2018
Phyllanthus acidus (L.) Skeels	Phyllanthaceae	Fr, Le	Martinique	Anti-hypertensive	Longuefosse and Nossin, 1996
Phyllanthus amarus Schumach. & Thonn.	Phyllanthaceae	St with Le, WP	Andoman + Nicobar Is., India; Martinique; Togo	Anti-paralytic; anti-hypertensive; anti-inflammatory [Fe]; anti-microbial [measles; influenza]; memory improvement/ enhancement; anti-epileptic	Chander et al., 2014; Longuefosse and Nossin, 1996; Adjanohoun et al., 1986: cited in Noe and Lehmann, 2012 [H168]; Kantati et al., 2016
Phyllanthus emblica L. Syn: *Emblica officinalis* Gaertn	Phyllanthaceae	Fr, Le, St	Hakka people, Guangdong, China; Chin people, Myanmar; Kani people, Western Ghats, India	Anti-hypertensive; anti-microbial [otitis media; dys, skin/ mouth inf]; anti-aging; anti-inflammatory; anti-fatigue	Au et al., 2008; Ong et al., 2018; DeFilipps and Krupnick, 2018; Ayyanar and Ignacimuthu, 2011
Phyllanthus fraternus G.L.Webster	Phyllanthaceae	WP	Mizoram, India	Anti-microbial [bronch, leprosy]; anti-inflammatory [asthma]	Rai and Lalramnghinglova, 2010
Phyllanthus niruri L.	Phyllanthaceae	AP, Le, WP	Mato Grosso, Brazil; Mestizo community, North Peru	Anti-inflammatory; anti-microbial	Ribeiro et al., 2017; Bussmann and Sharon, 2006

Phyllanthus parvifolius Buch.-Ham. ex D. Don	Phyllanthaceae	WP	Magar and Majhi people, Nepal	Anti-microbial [boil]	Malla et al., 2015
Phyllanthus stipulatus (Raf.) Webste	Phyllanthaceae	WP	Mestizo community, North Peru	Anti-inflammatory	Bussmann and Sharon, 2006
Phyllanthus ovalifolius Forssk Syn: *Phyllanthus guineensis*	Phyllanthaceae	Le	Mabira Forest, Uganda	Anti-microbial [measles]	Tugume et al., 2016
Phyllanthus urinaria L.	Phyllanthaceae	WP	Mestizo community, North Peru; Trinidad	Anti-inflammatory; anti-hypertensive	Bussmann and Sharon, 2006; Clement et al., 2015
Phyllodium pulchellum (L.) Desv. Syn: *Desmodium pulchellum* (L.) Benth.	Fabaceae	Le, Ro	Maonan people, Guangxi Zhuang, China	Anti-inflammatory	Hong et al., 2015
Physalis angulata L.	Solanaceae	Cx, Ro, WP	Hakka people, Guangdong, China; Tacana people, Bolivian Amazon	Anti-inflammatory [Fe, rheum]; anti-microbial [flu]	Au et al., 2008; Bourdy et al., 2000
Physalis minima L.	Solanaceae	Le	Andoman + Nicobar Is., India; Kakamega County, Kenya; Kani people, Western Ghats, India	Anti-paralytic; anti-inflammatory [Fe]; anti-microbial [boil, STI]	Chander et al., 2014; Odongo et al., 2018; Ayyanar and Ignacimuthu, 2011
Physalis peruviana L.	Solanaceae	Fr, Le	Mao Naga people Manipur, India; Markets of Bogotá, Columbia	Anti-microbial [eye inf]	Lokho, 2012; Bussmann et al., 2018
Phytolacca bogotensis Kunth	Phytolaccaceae	NS	Markets of Bogotá, Columbia	Anti-inflammatory	Bussmann et al., 2018
Phytolacca dioica L.	Phytolaccaceae	Le	Loja and Zamora-Chinchipe, Ecuador	Anti-hypertensive	Tene et al., 2007
Phytolacca dodecandra L'Hér.	Phytolaccaceae	Le, St	Maromizaha forest, Madagascar; Tigrigna people, Central Eritrea	Anti-fatigue; anti-microbial [TB]	Riondato et al., 2019; Yemane et al., 2017
Phytolacca rivinoides Kunth & C.D.Bouché	Phytolaccaceae	Br, Le	Colombian Andes	Anti-microbial [skin inf]; Anti-inflammatory [rheum]	Cadena-González et al., 2013
Picralima nitida (Stapf) T.Durand & H.Durand	Apocynaceae	R, Se	Cataractes and Lukaya districts, D.R. Congo; Ondo State, SW Nigeria	Anti-microbial [pneum]; memory improvement	Latham and Konda ku Mbuta, 2017; Mojisola et al., 2012
Pilea cavaleriei H.Lév.	Urticaceae	WP	Maonan people, Guangxi Zhuang, China	Antibacterial [TB]	Hong et al., 2015
Pilea elegans Gay	Urticaceae	Le	Mapuche people, Chile	Anti-inflammatory [Fe]	Houghton and Manby, 1985
Pilea microphylla (L.) Lieberman	Urticaceae	Le, WP	Martinique; Mestizo community, North Peru	Anti-inflammatory [Fe]	Longuefosse and Nossin, 1996; Bussmann and Sharon, 2006
Piliostigma thonningii (K. Schumacher) Milne-Redh.	Fabaceae	Ba, Ro	Venda, South Africa; Cataractes and Lukaya districts, D.R. Congo	Anti-microbial [STI]; anti-inflammatory [rheum]	Arnold and Gulumian, 1984; Latham and Konda ku Mbuta, 2017
Pilocarpus sp.	Rutaceae	Le, Ro	Três Ladeiras people, Atlantic Forest, Brazil	Antibacterial [dys]	Gazzaneo et al., 2005
Pimenta racemosa (Mill.) J.W.Moore	Myrtaceae	Le	Martinique; Trinidad	Anti-inflammatory [Fe]; anti-hypertensive; anti-microbial [cold]	Longuefosse and Nossin, 1996; Clement et al., 2015

Pimpinella anisum L.	Apiaceae	AP, FL, Le, Se	Middle East Carib people, Livingston, Guatamala; Atlantic Forest, Brazil; Potosi, Bolivia; Israel	Anti-paralytic; anti-inflammatory; anti-microbial [dys]; anti-venom	Krispil 2000: cited in Abu-Rabia, 2012; Girón et al., 1991; Fernandez et al., 2003
Pinellia ternata (Thunb.) Briet.	Araceae	FL, Le	Tian Mu Shan, Zhejiang, China	Anti-microbial [influenza, cold, cough]; anti-depressant	Chaudhary et al., 2006
Pinus contorta Dougl. ex Loud.	Pinaceae	Le	Native America	Anti-paralytic	Smith, 1929: cited in Native American Ethnobotany Database
Pinus kesiya Royle ex Gordon	Pinaceae	Re	Chin people, Myanmar	Anti-hypertensive; anti-microbial [ear inf, sore throat]	Ong et al., 2018
Piper aduncum L.	Piperaceae	FL, Le	Loja and Zamora-Chinchipe, Ecuador	Anti-microbial [influenza]; anti-inflammatory [rheum]	Tene et al., 2007
Piper aequale Vahl.	Piperaceae	Le, St	Mestizo community, North Peru	Anti-microbial	Bussmann and Sharon, 2006
Piper arboreum Aubl. Syn: *Piper verrucosum* Sw.	Piperaceae	NS	Markets of Bogotá, Columbia	Anti-microbial [dys]	Bussmann et al., 2018
Piper auritum Kunth	Piperaceae	Le	Carib population, Carib people, Livingston, Guatamala; Antioquia, Columbia	Anti-inflammatory [Fe]; anti-hypertensive; anti-venom	Girón et al., 1991; Vasquez et al., 2013
Piper betle L.	Piperaceae	Le	Maranao people, Philippines	Anti-inflammatory [Fe]	Malawani et al., 2017
Piper capense L.f.	Piperaceae	Ba	Venda, South Africa	Anti-microbial [sores, STI]	Arnold and Gulumian, 1984
Piper chaba Hunter	Piperaceae	Fr, Ro, St	Magar and Majhi people, Nepal	Anti-inflammatory	Malla et al., 2015
Piper guineense Schum. & Thonn.	Piperaceae	Fr	Mount Cameroon, Cameroon; Cataractes and Lukaya districts D. R. Congo; Sagamu, SW Nigeria; Guinea-Bissau	Anti-microbial [STI]; memory enhancement; anti-aging; anti-depressant	Sandberg et al., 2005; Latham and Konda ku Mbuta, 2017; Elufioye et al., 2012; Romeiras et al., 2012
Piper hispidum Sw.	Piperaceae	Le, St	Columbian Andes	Anti-inflammatory; anti-hypertensive	Cadena-González et al., 2013
Piper longum L.	Piperaceae	Ro	Eastern Ghats, India	Anti-inflammatory [headache]	Rao et al., 2006
Piper nigrum L.	Piperaceae	Fr	Kani people, Western Ghats, India; Yörük people, SE Macedonia	Anti-microbial [bronch, cough]; anti-inflammatory [rheum]	Ayyanar and Ignacimuthu, 2011; Nedelcheva et al., 2017
Piper obtusilimbum C. DC.	Piperaceae	NS	Markets of Bogotá, Columbia	Anti-inflammatory	Bussmann et al., 2018
Piper peepuloides Roxb.	Piperaceae	Le	Satkhira District, Kalaroa, Bangladesh	Anti-microbial [STI; anti-inflammatory [Fe]	Dulla and Jahan, 2017
Piper peltatum L.	Piperaceae	Le	Rio Jauaperi, Brazilian Amazon	Anti-microbial	Pedrollo et al., 2016
Piper sp.	Piperaceae	Le	Chin people, Myanmar	Anti-microbial [otitis media]	Ong et al., 2018
Piper puberulum (Benth.) Seem. Syn: *Piper hongkongense* C. DC	Piperaceae	Ba, Le	Tonga	Anti-microbial [boil]	Croft and Tu'ipulotu, 1980
Piper umbellatum L.	Piperaceae	Le, Ro	Cataractes and Lukaya districts, D.R. Congo; Congo Brazzaville; Burundi	Anti-microbial [STI, boil]; eye inflammation; anti-hypertensive	Latham and Konda ku Mbuta, 2017; Baerts and Lehmann, 1989: cited in Noe and Lehmann, 2012 [H001];

					Diafouka and Lejoly, 1993: cited in Noe and Lehmann, 2012 [H001]
Piptadeniastrum africanum (Hook.f.) Brenan	Fabaceae	St ba	Baka Pygmies, Gabon	Anti-microbial [influenza]	Betti et al., 2013
Pistacia atlantica Desf.	Anacardiaceae	Fr, Se, gum	Sirjan, SE Iran	Memory improvement	Nasab and Khosravi, 2014
Pistacia chinensis subsp. *integerrima* (J. L. Stewart ex Brandis) Rech. f. Syn*: Pistacia integerrima* L.	Anacardiaceae	Fr	Himachal Pradesh, NW Himalaya, India	Anti-microbial [dys]	Vidyarthi et al., 2013
Pisum sativum L.	Leguminosae	Se	Mestizo community, North Peru	Anti-inflammatory	Bussmann and Sharon, 2006
Pithecellobium jiringa (Jack) Merr.	Leguminosae	Ba	Sundanese community, West Java	Anti-bacterial [dys]	Roosita et al., 2008
Pittosporum mannii Hook. F.	Pittosporaceae	Ba, Le	Kakamega County, Kenya	Anti-microbial [measles, O inf]; anti-inflammatory [Fe]	Odongo et al., 2018
Pityrogramma chrysophylla (Sw.) Link	Pteridaceae	Le	Martinique	Anti-microbial	Longuefosse and Nossin, 1996
Plantago asiatica L.	Plantaginaceae	Le, WP	Maonan people, Guangxi Zhuang, China; Chin people, Myanmar	Anti-inflammatory; anti-hypertensive	Hong et al., 2015; Ong et al., 2018
Plantago lanceolata L.	Plantaginaceae	AP, Le	M'Sila, North Algeria; Agro Nocerino Sarnese, Southern Italy	Wound healing; anti-microbial [abscess]	Boudjelal et al., 2013; Motti and Motti, 2017
Plantago linearis H.B.K.	Plantaginaceae	Ro, WP	Mestizo community, North Peru	Anti-inflammatory	Bussmann and Sharon, 2006
Plantago major L.	Plantaginaceae	FL, Fr, Le, Ro, St	Martinique; Trinidad; Antioquia, Columbia; Svaneti and Racha-Lechkumi, Georgia; Turkestan Range, S Kyrgystan; Agro Nocerino Sarnese, S Italy	Neutralize venom; anti-microbial; anti-inflammatory [Fe]; anti-hypertensive; wound healing; Southern Italy	Longuefosse and Nossin, 1996; Clement et al., 2015; Vasquez et al., 2013; Bussmann et al., 2016; Pawera et al., 2016; Motti and Motti, 2017
Plantago sericea R. & P.	Plantaginaceae	Le	Mestizo community, North Peru	Anti-inflammatory	Bussmann and Sharon, 2006
Plantago sparsiflora Michx.	Plantaginaceae	Le, Se, WP	Mato Grosso, Brazil	Anti-microbial; anti-inflammatory	Ribeiro et al., 2017
Platostoma africanum P. Beauv.	Lamiaceae	Le	South Nigeria	Anti-microbial	Borokini et al., 2012
Platycodon grandiflorum (Jacq.) A. DC.	Campanulaceae	Ro, Spr	Shaanxi, China	Anti- atherosclerotic, anti-hypertensive, anti- hyperlipidemic	Teng et al., 2011
Plectranthus amboinicus (Lour.) Spreng. Syn*: Coleus amboinicus* Lour.	Lamiaceae	Le, Ro	Rio Jauaperi, Brazilian Amazon; Martinique; Eastern Ghats, India	Anti-viral [influenza]; anti-inflammatory [Fe]	Pedrollo et al., 2016; Longuefosse and Nossin, 1996; Rao et al., 2006
Plectranthus barbatus Andr.	Lamiaceae	Le	Três Ladeiras people, Atlantic Forest, Brazil	Anti-microbial [dys]	Gazzaneo et al., 2005

Plectranthus scutellarioides (L.) R.Br.	Lamiaceae	Le	Eastern Highlands, Papua New Guinea	Anti-microbial	Jorim et al., 2012
Pleioceras barteri Baill.	Apocynaceae	Le	Ehotile people, Côte d'Ivoire	Anti-microbial [boil]	Malan et al., 2015
Pleurospermum brunonis Benth. ex Cl.	Apiaceae	FL, Le	Himachal Pradesh, NW Himalaya, India	Anti-microbial [s'pox]	Vidyarthi et al., 2013
Plicosepalus curviflorus (Benth. ex Oliv.) Tiegh.	Loranthaceae	Le	Abyan territory, Yemen	Anti-microbial [abscess]; anti-hypertensive	Al-Fatimi, 2019
Plicosepalus robustus Wiens & Polhill	Loranthaceae	WP	Afar people, Rift Valley, Ethiopia	Anti-microbial	Teklehaymanot, 2017
Pluchea carolinensis (Jacq.) G.Don	Asteraceae	Le	Martinique; Trinidad	Anti-inflammatory [Fe]; anti-microbial [influenza, cold]; anti-hypertensive	Longuefosse and Nossin, 1996; Clement et al., 2015
Pluchea sagittalis (Lam.) Cabr.	Asteraceae	Le	Comechingones, Argentina	Anti-bacterial [STI	Goleniowski et al., 2006
Plumbago auriculata Lam.	Plumbaginaceae	Ro	3 Kerala tribes, Western Ghats, India	Anti-microbial [wart]; anti-inflammatory [headache]	Marjana et al., 2018
Plumbago indica L.	Plumbaginaceae	Ro, WP	Myanmar	Anti-aging/ promoting longevity; anti-partial paralysis	DeFilipps and Krupnick, 2018
Plumbago zeylanica L.	Plumbaginaceae	Le	Afar people, Rift Valley, Ethiopia; Kisangani, D.R. Congo; Ibadan city, SW Nigeria; Chittagong Hill Tracts, Bangladesh; Sundanese community, West Java	Anti-microbial [STI]; memory improvement; paralysis/ hemiplegia/ polio/ paraplegia; anti-venom; anti-inflammatory [headache]	Teklehaymanot, 2017; Wome, 1985: cited in Noe and Lehmann, 2012 [H168]; Ainslie, 1937: cited in Noe and Lehmann, 2012 [H130]; Kadir et al., 2015; Gbadamosi and Egunyomi, 2014; Roosita et al., 2008
Plumeria rubra L.	Apocynaceae	Ba, sap	Maori people, Cook Islands	Wound healing, anti-venom	Whistler, 1985
Pogostemon heyneanus Benth.	Lamiaceae	Le	Martinique	Anti-inflammatory; anti-microbial [cold, influenza]	Longuefosse and Nossin, 1996
Poincianella pluviosa (DC.) L.P.Queiroz	Fabaceae	Ba, sap	Mato Grosso, Brazil	Anti-inflammatory	Ribeiro et al., 2017
Polyalthia cerasoides (Roxb.) Bedd.	Annonaceae	Le	Gingee Hills, Tamil Nadu, India	Anti-fungal	Arulappan et al., 2015
Polygala acicularis Oliv.	Polygalaceae	Ba, Le, Ro, St	Bas-Congo	Anti-microbial [dys, sore throat, infected sore]	Latham and Konda ku Mbuta, 2017
Polygala elongata Klein ex Willd.	Polygalaceae	St ba	Eastern Ghats, India	Anti-inflammatory [headache]	Rao et al., 2006
Polygala paniculata L.	Polygalaceae	FL, Le	Eastern Highlands, Papua New Guinea	Anti-inflammatory	Jorim et al., 2012
Polygala tenuifolia Wild.	Polygalaceae	Ro	Shaanxi, China	Anti-microbial	Teng et al., 2011
Polygonatum cirrhifolium (Wall.) Royle	Asparagaceae	WP	Central Himalayas, Nepal	Anti-microbial [cough, cold]	Bhattarai et al., 2006
Polygonatum sibiricum Delar ex Redoute	Asparagaceae	Ro	Shaanxi, China; Maonan people, Guangxi Zhuang, China	Support immune system, anti-aging; anti-bacterial [TB]; anti-hypertensive; anti-fatigue	Teng et al., 2011; Hong et al., 2015

Polygonum hissaricum Popov	Polygonaceae	Le, St	Turkestan Range of south Kyrgystan	Immunostimulant	Pawera et al., 2016
Polymnia sonchifolia Poepp.	Compositae	Ro ju	Potosi, Bolivia	Anti-inflammatory [Fe]; anti-microbial [pneum]	Fernandez et al., 2003
Polypodium vulgare L.	Polypodiaceae	Ro	Edremit Gulf, Turkey	Anti-inflammatory [headache]; anxiolytic, anti-microbial [tons]	Polat and Satıl, 2012
Polyscias fulva (Hiern) Harms	Araliaceae	Le	Fundong, NW Cameroon	Anti-inflammatory [headache]	Focho et al.,2009
Polyscias guilfoylei (W.Bull) L.H.Bailey	Araliaceae	Le	Martinique	Anti-microbial [influenza]	Longuefosse and Nossin, 1996
Pongamia pinnata (L.) Pierre or Merr.	Leguminosae	Fr, Le, Se, St	Andoman + Nicobar Is.; India; Kani people, Western Ghats, India; Eastern Ghats, India	Anti-paralytic; wound healing; anti-inflammatory [headache]	Chander et al., 2014; Mikawlrawng et al., 2017; Ayyanar and Ignacimuthu, 2011; Rao et al., 2006
Portulaca grandiflora Hook.	Portulacaceae	Le	Martinique	Anti-inflammatory [Fe]	Longuefosse and Nossin, 1996
Portulaca oleracea L.	Portulacaceae	Le, Se, WP	Hakka people, Guangdong, China; Tian Mu Shan, Zhejiang, China; Loja and Zamora-Chinchipe, Ecuador; Martinique; Benin; Abyan territory, Yemen; Fiji	Anti-inflammatory [Fe]; anti-hypertensive; anti-microbial [measles, UTI]; hemiplegia; anti-paralytic	Au et al., 2008; Chaudhary et al., 2006 2006; Tene et al., 2007; Longuefosse and Nossin, 1996; Adjanohoun et al., 1989: cited in Noe and Lehmann, 2012 [H130]; Al-Fatimi, 2019; Singh, 1986
Potentilla polyphylla Wall. ex Lehm.	Rosaceae	Ro	Magar and Majhi people, Nepal	Anti-microbial [throat/tooth inf, cough, cold]	Malla et al., 2015
Potentilla freyniana Bornm.	Rosaceae	Ro	Maonan people, Guangxi Zhuang, China	Anti-microbial [rabies]	Hong et al., 2015
Pourouma bicolor Mart.	Urticaceae	Fr, St ba	Rio Jauaperi, Brazilian Amazon	Anti-viral [influenza]	Pedrollo et al., 2016
Pouteria ramiflora (Mart.) Radlk.	Sapotaceae	Ba, St	Mato Grosso, Brazil	Anti-microbial	Ribeiro et al., 2017
Pradosia sp.	Sapotaceae	St Ba	Três Ladeiras people, Atlantic Forest, Brazil	Anti-fatigue	Gazzaneo et al., 2005
Premna corymbosa Rottler	Lamiaceae	Le	Andoman + Nicobar Is., India	Anti-inflammatory [Fe]	Chander et al., 2014
Premna serratifolia L.	Lamiaceae	AP, Le	Marquesas Islands, French Polynesia	Anti-fungal	Girardi et al., 2015
Primula veris L.	Primulaceae	FL	Gheg people, NE. Albania	Anti-microbial [cough]	Pieroni and Sõukand, 2017
Primula vulgaris Huds.	Primulaceae		N + NE Bosnia and Herzegovina	Anti-paralytic; anti-inflammatory [rheum]; anti-microbial [pneum, bronch, influenza]	Saric-Kundalic et al., 2011
Priva curtisiae Kobuski	Verbenaceae	Le	Afar people, Rift Valley, Ethiopia	Anti-microbial [typhoid]	Teklehaymanot, 2017
Priva lappulacea (L.) Pers.	Verbenaceae	Le, Ro	Tacana people, Bolivian Amazon	Anti-venom	Bourdy et al., 2000
Protium heptaphyllum (Aubl.) March	Burseraceae	Re	Três Ladeiras people, Atlantic Forest, Brazil	Anti-microbial [influenza]; anti-inflammatory [headache, toothache]; anxiolytic	Gazzaneo et al., 2005; Ribeiro et al., 2017

Protium glabrescens Swart	Burseraceae	Re	Tacana people, Bolivian Amazon	Anti-paralytic [facial]; anti-inflammatory [headache, rheum]; anti-microbial [boil]	Bourdy et al., 2000
Prunella vulgaris L.	Lamiaceae	Se	Toli Peer National Park, Kashmir, Pakistan; Hakka people, Guangdong, China	Anti-inflammatory; anti-hypertensive	Amjad et al., 2017: Au et al., 2008
Prunus africana (Hook.f.) Kalkman	Rosaceae	Ba, Le	Nyeri County, Central Kenya; Fundong, NW Cameroon	Memory improvement; anti-inflammatory [rheum]; anti-hypertensive	Kamau et al., 2016; Focho et al.,2009
Prunus amygdalus Batsch Syn: *Amygdalus communis* L	Rosaceae	Fr	Golan Heights and West Bank, Israel	Anti-paralytic	Said et al., 2002
Prunus avium (L.) L.	Rosaceae	Fr	Agro Nocerino Sarnese, Southern Italy	Anti-microbial [cough]	Motti and Motti, 2017
Prunus cerasifera Ehrh.	Rosaceae	Fr	Edremit Gulf, Turkey	Anti-hypertensive	Polat and Satıl, 2012
Prunus cerasoides Buch.-Ham. ex D.Don	Rosaceae	Ba	Chin people, Myanmar	Anti-paralytic	Ong et al., 2018
Prunus dulcis Mill. ex Rchb.	Rosaceae	Fr	Agro Nocerino Sarnese, Southern Italy	Anti- hypercholesterolaemia	Motti and Motti, 2017
Prunus erythrocarpa (Nevski) Gilli	Rosaceae	Fr	Turkestan Range of south Kyrgystan	Anti-inflammatory [headache]	Pawera et al., 2016
Prunus persica (L.) Batsch	Rosaceae	Le	Abyan territory, Yemen; Oromo people, Harla, Eastern Ethiopia	Anti-microbial [cough]	Al-Fatimi, 2019; Belayneh and Bussa, 2014
Prunus spinosa L.	Rosaceae	Br, Fr	Pollino National Park, Southern Italy; Yörük people, SE Macedonia	Anti-microbial; anti-fatigue	Di Sanzo et al., 2013; Nedelcheva et al., 2017
Pseudelephantopus spicatus (B.Juss. ex Aubl.) Rohr ex C.F.Baker	Asteraceae	Le, Ro	Ayta communities, Bataan, Philippines	Anti-microbial [skin inf]	Tantengco et al., 2018
Pseuderanthemum latifolium B. Hansen	Acanthaceae	Le	Bac Huong Hoa nature reserve, Vietnam	Anti-hypertensive	Lee et al., 2019
Pseudocedrela kotschyi (Schweinf.) Harms	Meliaceae	Ro	Togo	Dementia, memory loss, epilepsy	Kantati et al., 2016
Pseudognaphalium canescens (DC.) Anderb. Syn: *Gnaphalium canescens*	Asteraceae	St	Nuevo León, Mexico	Anti-microbial	Estrada-Castillón et al., 2012
Pseudospondias microcarpa (A.Rich.) Engl.	Anacardiaceae	Ba	Mabira Forest, Uganda; Cataractes and Lukaya districts, D.R. Congo	Anti-microbial [yell fev, dys]	Tugume et al., 2016; Latham and Konda ku Mbuta, 2017
Pseudotsuga menziesii (Mirbel) Franco	Pinaceae	Le	Native America	Anti-paralytic	Jones, 1931: cited in Native American Ethnobotany Database
Psidium acutangulum Mart. ex DC.	Myrtaceae	St ba	Rio Jauaperi, Brazilian Amazon	Anti-microbial [dys]	Pedrollo et al., 2016
Psidium cattleianum Afzel. ex Sabine	Myrtaceae	Le	Três Ladeiras people, Atlantic Forest, Brazil	Anti-viral; anti-microbial [dys]	Gazzaneo et al., 2005

Psidium guajava L.	Myrtaceae	Ba, Fr, Le, Ro,	Rio Jauaperi, Brazilian Amazon; Caiçaras people, Brazil Atlantic forest; Venda, South Africa; Mao Naga people Manipur, India; Bangladesh; Guinea-Bissau; Tonga; Maori people, Cook Islands; Bolicar, N Colombia	Anti-microbial [boil, dys, bronch, STI]; anti-inflammatory [Fe]; anti-epileptic; wound; anxiolytic	Pedrollo et al., 2016; Begossi et al., 2002; Arnold and Gulumian, 1984; Lokho, 2012; Romeiras et al., 2012; Croft and Tu'ipulotu, 1980; Whistler, 1985; Gómez-Estrada et al., 2011
Psidium guineense Sw.	Myrtaceae	Le	Columbian Andes	Anti-microbial [skin inf]; anti-inflammatory [rheum]	Cadena-González et al., 2013
Psittacanthus calyculatus (DC.) G. Don	Loranthaceae	NS	Markets of Bogotá, Columbia	Anti-inflammatory; cholesterol-lowering; anti-epileptic	Bussmann et al., 2018
Psophocarpus scandens (Endl.) Verdc.	Leguminosae	sap	Cataractes and Lukaya districts, D.R. Congo	Anti-microbial [sore]	Latham and Konda ku Mbuta, 2017
Psoralea sp.	Leguminosae	Le, St	Eastern Highlands, Papua New Guinea	Anti-microbial	Jorim et al., 2012
Psychotria elata (Sw.) Hammel Syn: *Cephaelis elata Sw.*	Rubiaceae	St	Guaymi indians, Panama	Dementia treatment	Joly et al., 1987
Psychotria umbellata Thonn. Syn: *Psychotria calva* Hiern	Rubiaceae	Ro	Cataractes and Lukaya districts, D.R. Congo	Anti-microbial [whooping cough, cough]	Latham and Konda ku Mbuta, 2017
Pteleopsis hylodendron Mildbr.	Combretaceae	Ba	Mount Cameroon, Cameroon; Tropical rain forest, Central + S Cameroon	Anti-bacterial, anti-viral [C'pox]	Sandberg et al., 2005; Ngono Ngane et al., 2011
Pterocarpus angolensis DC.	Leguminosae	Ba	Venda, South Africa	Anti-microbial [STI]	Arnold and Gulumian, 1984
Pterocarpus indicus Willd.	Leguminosae	Ba, Le, Re, St	Ayta communities, Bataan, Philippines; Sundanese community, West Java; Conis Santana National Park, East Timor	Anti-microbial [TB, mouth sores]; anti-inflammatory [Fe, pain]	Tantengco et al., 2018; Roosita et al., 2008; Collins et al., 2007
Pterocarpus rohrii Vahl.	Leguminosae	Ba	Peruvian Amazon	Anti-microbial [TB]	Odonne et al., 2013
Pterocaulon alopecuroidum Chodat	Compositae	Le	Martinique	Anti-inflammatory [Fe]	Longuefosse and Nossin, 1996
Pterodon emarginatus Vogel	abaceae	Ba, Le, Se, St	Mato Grosso, Brazil	Anti-microbial; anti-inflammatory	Ribeiro et al., 2017
Pteromonnina pterocarpa (Ruiz & Pav.) B. Eriksen Syn: *Monnina pterocarpa* R. & P.	Polygalaceae	FL, Le	Mestizo community, North Peru	Anti-microbial	Bussmann and Sharon, 2006
Pueraria montana var. lobata (Willd.) Sanjappa & Pradeep Syn: *Pueraria thunbergiana*	Fabaceae	St, Tu	Hakka people, Guangdong, China; Huanjiang Maonan, Guangxi, China	Anti-inflammatory; anti-hypertensive	Au et al., 2008; Hong et al., 2015
Pulicaria mauritanica Batt.	Asteraceae/Compositae	AP	High Atlas, Morocco	Anti-inflammatory [Fe]; anti-hypertensive	Fadili *et al*, 2017
Pulicaria undulata (L.) C.A.Mey.	Asteraceae/Compositae	AP, Le	Abyan territory, Yemen	Anti-inflammatory; anti-microbial [abscess, boil]	Al-Fatimi, 2019

Punica granatum L.	Lythraceae	Fr	Toli Peer National Park, Kashmir, Pakistan, Brazil Mato Grosso; Uttarakhand, India; Abyan territory, Yemen; Tassili N'Ajjer, Southern Algerian Sahara; Satpuda forest, Maharashtra, India	Anti-microbial [dys; cough]; anti-inflammatory [asthma]; anti-epileptic; memory improvement	Amjad et al., 2017; Sharma et al., 2013; Al-Fatimi, 2019; Hammiche and Maiza, 2006; Jain et al., 2010
Pupalia lappacea (L.) Juss.	Amaranthaceae	WP	Benin	Hemiplegia/ paraplegia/ polio	Adjanohoun et al., 1989: cited in Noe and Lehmann, 2012 [H130]
Puya hamata L.B. Sm.	Bromeliaceae	Se	Mestizo community, North Peru	Anti-microbial	Bussmann and Sharon, 2006
Pycnobotria nitida Benth	Apocynaceae	Le	Baka Pygmies, Gabon	Anti-microbial [diarrh]	Betti et al., 2013
Pyracantha crenulata (D. Don) M.Roem.	Rosaceae	Fr	Magar and Majhi people, Nepal	Anti-microbial [dys]	Malla et al., 2015
Qualea parviflora Mart.	Vochysiaceae	Ba, Le, Ro	Mato Grosso, Brazil	Anti-microbial [diarrh, conj]; anti-inflammatory; wound healing	Ribeiro et al., 2017
Quassia africana (Baill.) Baill.	Simaroubaceae	Ro, Wo, WP	Cataractes and Lukaya districts, D.R. Congo	Anti-inflammatory [Fe]; anti-microbial [STI]	Latham and Konda ku Mbuta, 2017
Quassia amara L.	Simaroubaceae	Le	Martinique; Carib people, Livingston, Guatamala; Markets of Bogotá, Columbia	Anti-inflammatory [Fe]; anti-hypertensive; anti-microbial {kidney inf]	Longuefosse and Nossin, 1996; Girón et al., 1991; Bussmann et al., 2018
Quassia undulata (Guill. & Perr.) D.Dietr.	Simaroubaceae	Le	Ondo State, SW Nigeria	Memory improvement	Mojisola et al., 2012
Quercus humboldtii Bonpl.	Fagaceae	NS	Markets of Bogotá, Columbia	Anti-inflammatory [periodontitis]; anti-microbial [mouth inf]	Bussmann et al., 2018
Quinchamalium majus Brongn. (ind)	Schoepfiaceae	Le	Mapuche people, Chile	Anti-microbial	Houghton and Manby, 1985
Ranunculus nubigenus Kunth ex DC.	Ranunculaceae	NS	Markets of Bogotá, Columbia	Anti-microbial [wart]	Bussmann et al., 2018
Raphia vinifera P.Beauv.	Arecaceae	Fr	Mount Cameroon, Cameroon	Anti-microbial [leprosy]	Sandberg et al., 2005
Raphanus raphanistrum subsp. *sativus* (L.) Domin Syn: *Raphanus sativus* L.	Brassicaceae	Le, Ro	Abyan territory, Yemen; Fiji; Edremit Gulf, Turkey; Yörük people, SE Macedonia	Anti-inflammatory [asthma]; anti-paralytic; anxiolytic; anti-microbial [sore throat]	Al-Fatimi, 2019; Singh, 1986; Polat and Satıl, 2012; Nedelcheva et al., 2017
Rauvolfia mannii Stapf	Apocynaceae	Ro	Cataractes and Lukaya districts, D. R. Congo	Anti-inflammatory [Fe]; anti-microbial [STI	Latham and Konda ku Mbuta, 2017
Rauvolfia tetraphylla L.	Apocynaceae	Ro	Gingee Hills, Tamil Nadu, India; Markets of Bogotá, Columbia	Nervous disorders; anti-hypertensive	Arulappan et al., 2015; Bussmann et al., 2018
Rauvolfia verticillata (Lour.) Baill.	Apocynaceae	Ro	Maonan people, Guangxi Zhuang, China	Anti-hypertensive	Hong et al., 2015
Rauvolfia vomitoria Wennberg	Apocynaceae	Ro	Cataractes and Lukaya districts, D.R. Congo; Fundong, NW Cameroon	Anti-hypertensive; anti-microbial [abscess]	Latham and Konda ku Mbuta, 2017; Focho et al.,2009
Reseda villosa Coss.	Resedaceae	AP	Tassili N'Ajjer, Southern Algerian Sahara	Anti-inflammatory [rheum]	Hammiche and Maiza, 2006

Rhamnus alaternus L.	Rhamnaceae	Le	Catalonia, Spain	Anti-hypertensive	Raja et al., 1997
Rhamnus californica Esch. Syn: *Frangula californica* (Eschsch.) A.Gray	Rhamnaceae	Le	Chumash Indians, California, USA	Anti-inflammatory [rheum]	Timbrook, 1990
Rhamnus virgatus Roxb. [unresolved]	Rhamnaceae	Le	Himachal Pradesh, NW Himalaya, India	Anti-inflammatory [malarial Fe]	Vidyarthi et al., 2013
Rhaphidophora pertusa (Roxb.) Schott	Araceae	Le	Satkhira District, Kalaroa, Bangladesh	Anti-inflammatory	Dulla and Jahan, 2017
Rhaphithamnus spinosus (Juss.) Mold. (ind)	Verbenaceae	Fr	Mapuche people, Chile	Anti-microbial	Houghton and Manby, 1985
Rheum australe D. Don.	Polygonaceae	Ro	Himachal Pradesh, NW Himalaya, India	Anti-microbial [s'pox]	Vidyarthi et al., 2013
Rhipsalis baccifera (J.S.Muell.) Stearn	Cactaceae	NS	D.R. Congo	Anti-PD	Kembelo, 2003: cited in Noe and Lehmann, 2012 [H130x]
Rhizophora mangle L.	Rhizophoraceae	FL, St ba	Três Ladeiras people, Atlantic Forest, Brazil	Anti-inflammatory; anti-microbial [leprosy]	Gazzaneo et al., 2005
Rhodiola tibetica (Hook.f. & Thomson) S.H.Fu	Crassulaceae	Le, Sh	Karakoram wildlife sanctuary, Western Ladakh, India	Memory improvement; anti-inflammatory [headache]	Namtak and Sharma, 2018
Rhodobryum giganteum (Hook.) Par.	Bryaceae	WP	Lisu, Yunnan, China	Anti-hypertensive	Ji et al., 2005
Rhododendron anthopogon D. Don	Ericaceae	Sh, EO	Central Himalayas, Nepal	Anti-microbial	Bhattarai et al., 2006,
Rhododendron arboreum Sm.	Ericaceae	Le	Chin people, Myanmar	Anti-hypertensive	Ong et al., 2018
Rhus chinensis Mill. Syn: *Rhus semialata* Linn.	Anacardiaceae	Fr	Mao Naga people Manipur, India	Anti-microbial	Lokho, 2012
Rhus vulgaris Meikle	Anacardiaceae	Ba, Le, Ro, St Ba	Kakamega County, Kenya	Anti-microbial [STI cold]	Odongo et al., 2018
Rhynchosia hirta (Andrews) Meikle & Verdc	Fabaceae	Le	Mabira Forest, Uganda	Anti-microbial [*H zoster*].	Tugume et al., 2016
Rhynchospora colorata (L.) H.Pfeiff. Syn: *Cyperus kyllingia* Endl.	Cyperaceae	Rh	3 Kerala tribes, Western Ghats, India	Anti-microbial [dys]	Marjana et al., 2018
Rhynchospora nervosa (Vahl) Boeckeler	Cyperaceae	NS	Markets of Bogotá, Columbia	Anti-microbial [influenza]	Bussmann et al., 2018
Ribes magellanicum Poir.	Grossulariaceae	NS	Mapuche people, Argentina	Anti-inflammatory	Estomba et al., 2005
Rhytidocaulon macrolobum Lavranos	Apocynaceae	St	Abyan territory, Yemen	Memory loss	Al-Fatimi, 2019
Ribes sp.	Grossulariaceae	Ba	Native America	Anti-paralytic	Smith, 1929: cited in Native American Ethnobotany Database
Rinorea anguifera Kuntze [unresolved]	Violaceae	Le	Seberida, Riau Province, Sumatra, Indonesia	Anti-inflammatory	Mahyar et al., 1991
Rivea hypocrateriformis (Desr.) Choisy	Convolvulaceae	Re	Eastern Ghats, India	Anti-microbial [toothache]	Rao et al., 2006

Rodgersia aesculifolia Batal	Saxifragaceae	Ro	Shaanxi, China	Anti-microbial [sore throat, diarrh, fungal inf]	Teng et al., 2011
Rorippa sarmentosa (Sol. ex G.Forst. ex DC.) J.F.Macbr.	Brassicaceae	WP	Marquesas Islands, French Polynesia	Anti-inflammatory	Girardi et al., 2015
Rosa × *alba* L.	Rosaceae	FL	Mato Grosso, Brazil	Anti-microbial	Ribeiro et al., 2017
Rosa × *centifolia* L.	Rosaceae	FL	Loja and Zamora-Chinchipe, Ecuador	Anti-microbial [conj]	Tene et al., 2007
Rosa canina L.	Rosaceae	Fr	Iran; Potosi, Bolivia; Gheg people, NE Albania; Edremit Gulf, Turkey; Yörük people, SE Macedonia	Anti-hypertensive; anti-depressant; anti-microbial [cold, influenza, sore throat, cough]; anti-fatigue	Mosaddegh et al., 2013; Fernandez et al., 2003; Pieroni and Sõukand, 2017; Polat and Satıl, 2012; Nedelcheva et al., 2017
Rosa ecae Aitch.	Rosaceae	Le, FL, Fr	Turkestan Range of south Kyrgystan	Anti-microbial [bronch]	Pawera et al., 2016
Rosa fedtschenkoana Regel	Rosaceae	Fr	Turkestan Range of south Kyrgystan	Anti-microbial influenza, cough]; anti-ulcer	Pawera et al., 2016
Rosa macrophylla Lindl.	Rosaceae	Fr	Central Himalayas, Nepal	Anti-microbial [diarrh, dys]; anti-inflammatory [Fe]	Bhattarai et al., 2006
Rosa phoenicia Boiss.	Rosaceae	Fr	Edremit Gulf, Turkey	Anti-microbial [cold, influenza]	Polat and Satıl, 2012
Rosa sp.	Rosaceae	NS	Native America	Anti-paralytic	Smith, 1929: cited in Native American Ethnobotany Database
Rosmarinus officinalis L. Syn: *Salvia rosmarinus* Spenn.	Lamiaceae	AP, FL, Le	Loja and Zamora-Chinchipe, Ecuador; Bogota, Markets of Bogotá, Columbia; Northern cities + High Atlas, Morocco; Golan Heights and West Bank, Israel; North Algeria; Agro Nocerino Sarnese, Southern Italy; Catalonia, Spain; Edremit Gulf, Turkey	Anti-microbial [int inf, bronch, cough, cold, influenza, abscess]; anti-inflammatory [headache, migraine, asthma, rheum]; wound healing; anti-atherosclerotic; memory improvement; anti-hypercholesterol; anti-fatigue; anti-hypertensive	Tene et al., 2007; Bussmann et al., 2018; Moussi et al., 2015; Said et al., 2002; Boudjelal et al., 2013; Motti and Motti, 2017; Bonet et al., 1999; Polat and Satıl, 2012; El-Hilaly et al., 2003; Fadili *et al*, 2017
Rotheca myricoides (Hochst.) Steane & Mabb Syn: *Clerodendrum myricoides* (Hochst.) R. Br. ex Vatke	Lamiaceae	Le, Ro	Kakamega County, Kenya	Anti-microbial [polio, STI, eye inf]	Bussmann. 2006; Odongo et al., 2018
Rourea coccinea subsp. *coccinea* (Schumach. & Thonn.) Benth.	Connaraceae	Le, Ro	Cataractes and Lukaya districts, D.R. Congo	Anti-microbial [STI, sore throat]; anti-paralytic; anti- PD	Latham and Konda ku Mbuta, 2017
Rubia cordifolia L.	Rubiaceae	Le, Ro, St	Mabira Forest, Uganda; Himachal Pradesh, NW Himalaya, India; Kakamega County, Kenya; Burundi	Anti-microbial [TB, boil; O inf]; eye inflammation	Tugume et al., 2016; Vidyarthi et al., 2013; Odongo et al., 2018; Baert and Lehmann, 1989: cited in Noe and Lehmann, 2012 [H001]
Rubus buergeri Miq. Syn: *Rubus moluccanus* auct.	Rosaceae	Le	Eastern Highlands, Papua New Guinea	Anti-inflammatory	Jorim et al., 2012
Rubus caesius L.	Rosaceae	Fr	Turkestan Range of south Kyrgystan	Anti-inflammatory [headache]	Pawera et al., 2016

Rubus foliolosus Hal csy. [unresolved]	Rosaceae	Ro	Central Himalayas, Nepal	Anti-microbial [ton'is, cough]; anti-inflammatory [headache, Fe]	Bhattarai et al., 2006
Rubus glaucus Benth.	Rosaceae	Fr, Sh	Colombian Andes	Anti-microbial [skin inf]; anti-inflammatory [rheum]	Cadena-González et al., 2013
Rubus pinnatus Willd.	Rosaceae	Fr	Mabira Forest, Uganda	Boost energy	Tugume et al., 2016
Rubus robustus C. Presl.	Rosaceae	FL, Le	Mestizo community, North Peru	Anti-inflammatory	Bussmann and Sharon, 2006
Rubus sp.	Rosaceae	Ba	Native America	Anti-paralytic	Smith, 1929: cited in Native American EthnobotanyDatabase
Ruellia patula Jacq. *Dipteracanthus patulus* (Jacq.) Nees	Acanthaceae	WP	Afar people, Rift Valley, Ethiopia	Anti-microbial [diphth]	Teklehaymanot, 2017
Ruellia tuberosa L.	Acanthaceae	Le	Martinique; Carib people, Livingston, Guatamala	Anti-inflammatory [Fe]	Longuefosse and Nossin, 1996
Rumex abyssinicus Jacq	Polygonoceae	Le	Kakamega County, Kenya	Anti-microbial [eye inf]	Odongo et al., 2018
Rumex crispus L.	Polygonoceae	Le, WP	Europe; Mestizo community, North Peru; Loja and Zamora-Chinchipe, Ecuador; Columbia; West Asia	Anti-inflammatory; anti-microbial [gangr]	Bussmann and Sharon, 2006; Tene et al., 2007; Cadena-González et al., 2013
Rumex hastatus D. Don	Polygonoceae	Ro	Toli Peer National Park, Kashmir, Pakistan	Anti-microbial [cough]; anti-inflammatory [Fe]	Amjad et al., 2017
Rumex nepalensis Spreng.	Polygonoceae	Le, Ro	Central Himalayas, Nepal; Chin people, Myanmar; Himachal Pradesh, NW Himalaya, India	Anti-microbial [wart]; anti-hypertensive; wound healing	Bhattarai et al., 2006;; Ong et al., 2018; Vidyarthi et al., 2013
Rumex usambarensis (Dammer) Dammer	Polygonoceae	Le, Ro	Kakamega County, Kenya; Cataractes and Lukaya districts, D.R. Congo	Anti-microbial [UTI, conj]	Odongo et al., 2018; Latham and Konda ku Mbuta, 2017
Ruta chalepensis L.	Rutaceae	Le, WP	Native America; Jazan Region, Saudi Arabia; Abyan territory, Yemen; Tigrigna people, Central Eritrea	Anti-paralytic; neurological disorders; anti-venom; anti-microbial [cough, cold]	Bocek, 1984: cited in Native American Ethnobotany Database; Tounekti et al., 2019; Al-Fatimi, 2019; Yemane et al., 2017
Ruta montana L.	Rutaceae	AP	High Atlas, Morocco	Anti-paralytic, anti-inflammatory [headache]	Fadili et al., 2017
Ruta graveolens L.	Rutaceae	AP, Ba, FL, Le, St	Karnataka, India; Markets of Bogotá, Columbia	Anti-microbial [typhoid]; anti-inflammatory [rheum]; anti-epileptic	Rajakumar and Shivanna, 2009; Cadena-González et al., 2013; Bussmann et al., 2018
Rydingia integrifolia (Benth.) Scheen & V.A.Albert Syn: *Otostegia integrifolia* Benth.	Lamiaceae	Ba	Tigrigna people, Central Eritrea	Anti-hypertensive, anti-microbial [ton'is]	Yemane et al., 2017
Saba comorensis (Bojer ex A.DC.) Pichon	Apocynaceae	Le	Cataractes and Lukaya districts, D.R. Congo	Anti-hypertensive	Latham and Konda ku Mbuta, 2017
Saccharum officinarum L.	Poaceae	Le, St	Mato Grosso, Brazil Martinique; Angola; Togo	Anti-microbial; anti-hypertensive; anti-inflammatory [Fe]; memory	Ribeiro et al., 2017; Longuefosse and Nossin, 1996; Bossard, 1996: cited in Noe and

				improvement/ enhancement; anti-paralytic	Lehmann, 2012 [H168]; Kantati et al., 2016
Saccharum spontaneum L.	Poaceae	Le, Ro	Ayta and Maranao people, Philippines	Anti-microbial [dengue, TB]; anti-inflammatory [Fe]	Tantengco et al., 2018; Malawani et al., 2017
Salacia impressifolia (Miers) A.C.Sm.	Celastraceae	Ba	Tacana people, Bolivian Amazon	Anti-microbial [influenza]; anti-inflammatory [rheum]	Bourdy et al., 2000
Salix acmophylla Boiss.	Salicaceae	Le	Toli Peer National Park, Kashmir, Pakistan	Anti-microbial [boil]	Amjad et al., 2017
Salix denticulata Andersson	Salicaceae	Ro ba, St	Toli Peer National Park, Kashmir, Pakistan	Anti-paralytic	Amjad et al., 2017
Salix humboldtiana Willd.	Salicaceae	NS	Markets of Bogotá, Columbia	Anti-inflammatory [Fe]	Bussmann et al., 2018
Salsola imbricata Forssk. Syn: *Salsola baryosma* (Schult.) Dandy	Amaranthaceae	WP	Tassili N'Ajjer, Southern Algerian Sahara	Anti-hypertensive	Hammiche and Maiza, 2006
Salvadora persica L.	Salvadoraceae	Le, Fr	Abyan territory, Yemen; Tassili N'Ajjer, Southern Algerian Sahara	Anti-microbial [mouth inf, cough, boil], STI; poison antidote, anti-venom; low immunity; anti-inflammatory [Fe, asthma]	Al-Fatimi, 2019; Hammiche and Maiza, 2006
Salvertia convallariodora A. St.-Hil.	Vochysiaceae	Le	Mato Grosso, Brazil	Anti-microbial; anti-inflammatory	Ribeiro et al., 2017
Salvia aegyptiaca L.	Lamiaceae	AP, Fr	Western Sahara; Tassili N'Ajjer, Southern Algerian Sahara	Glaucoma; anti-inflammatory [Fe]; wound inf	Volpato et al., 2012: cited in Noe and Lehmann, 2012 [H001]; Hammiche and Maiza, 2006]
Salvia apiana Jeps.	Lamiaceae	Le	Chumash Indians, California, USA	Anti-inflammatory [headache]	Timbrook, 1990
Salvia ayavazensis Kunth	Lamiaceae	Le	Mestizo community, North Peru	Anti-inflammatory	Bussmann and Sharon, 2006
Salvia gilliesi Benth	Lamiaceae	Le	Comechingones, Argentina	Anti-inflammatory [Fe]	Goleniowski et al., 2006
Salvia hians Royle ex Benth.	Lamiaceae	Le	Toli Peer National Park, Kashmir, Pakistan	Anti-microbial [cough, cold]; anxiolytic	Amjad et al., 2017
Salvia merjamie Forssk.	Lamiaceae	Fr	Abyan territory, Yemen	Anti-microbial [skin inf, wart]	Al-Fatimi, 2019
Salvia officinalis L; and *Salvia officinalis* subsp. *lavandulifolia* (Vahl) Gams Syn: *Salvia rosmarinifolia* Hort. ex G. Don	Lamiaceae	AP, Le; WP	M'Sila, North Algeria; Taounate, North Morocco; Fadili *et al*, 2017 High Atlas, Morocco; Catalonia, Spain; Mestizo community, North Peru	Anti-hypertensive; arteriosclerosis; anti-inflammatory [rheum]; anti-microbial [cough]	Boudjelal et al., 2013; El-Hilaly et al., 2003; Fadili *et al*, 2017; Raja et al., 1997; Bussmann and Sharon, 2006
Salvia palifolia Kunth	Lamiaceae	NS	Markets of Bogotá, Columbia	Arteriosclerosis; anti-hypertensive	Bussmann et al., 2018
Salvia sagittata Ruiz & Pav.	Lamiaceae	AP	Potosi, Bolivia	Anti-microbial	Fernandez et al., 2003
Salvia scutellarioides Kunth	Lamiaceae	NS	Markets of Bogotá, Columbia	Arteriosclerosis; anti-inflammatory [periodontitis]; anti-microbial	Bussmann et al., 2018
Salvia tomentosa Mill.	Lamiaceae	Le	Edremit Gulf, Turkey	Anti-microbial [cold, influenza, tons]	Polat and Satıl, 2012
Salvia tubiflora Sm.	Lamiaceae	WP	Northern Mestizo community, North Peru	Anti-paralytic	Bussmann and Sharon, 2006
Salvia verbenaca L.	Lamiaceae	AP	M'Sila, North Algeria	Wound healing, tonic	Boudjelal et al., 2013

Sambucus australis Cham. & Schlecht	Adoxaceae	FL	Três Ladeiras people, Atlantic Forest, Brazil	Anti-microbial [measles]	Gazzaneo et al., 2005
Sambucus canadensis L. Syn: *Sambucus mexicana* C.Presl ex DC.	Adoxaceae	FL, Fr	Martinique; Nuevo León, Mexico; Magar and Majhi people, Nepal	Anti-microbial [bronch, cough, measles, influenza]; anti-inflammatory [Fe]	Longuefosse and Nossin, 1996; Malla et al., 2015
Sambucus ebulus L.	Adoxaceae	L, Le	Pollino National Park, Southern Italy	Wound healing; anti-microbial [bronc]	Di Sanzo et al., 2013
Sambucus javanica Reinw. ex Blume	Adoxaceae	Le	Orang Asli tribe, Malaysia	Anti-inflammatory	Samuel et al., 2010
Sambucus nigra L.	Adoxaceae	FL, Le	Mato Grosso, Brazil; Loja and Zamora-Chinchipe, Ecuador; Pollino National Park + Agro Nocerino Sarnese, Southern Italy; Istro-Romanians, Northern Istria, Croatia; Southern Italy; Edremit Gulf, Turkey	Anti-microbial [influenza, cold, bronch, cough, sore throat]; anti-inflammatory [Fe]	Ribeiro et al., 2017; Tene et al., 2007; Di Sanzo et al., 2013; Pieroni et al., 2003; Motti and Motti, 2017; Polat and Satıl, 2012
Sambucus peruviana H.B.K.	Adoxaceae	FL, Le, St	Mestizo community, North Peru, Markets of Bogotá, Columbia	Anti-inflammatory; anti-microbial [UTI, RTI, measles]	Bussmann and Sharon, 2006; Bussmann et al., 2018
Sandoricum koetjape (Burm.f.) Merr.	Meliaceae	Le	Ayta communities, Bataan, Philippines	Anti-inflammatory [Fe]	Tantengco et al., 2018
Sanguisorba minor Scop.	Rosaceae	AP	Iran	Anti-microbial [oral + gynaecological inf]	Mosaddegh et al., 2013
Sansevieria roxburghiana Schult. & Schult.f.	Asparagaceae	Le, Rh	Gingee Hills, Tamil Nadu, India; Dinajpur District, Bangladesh	Anti-microbial; anti-inflammatory	Arulappan et al., 2015; Rahmatullah et al., 2009
Sansevieria trifasciata Prain	Asparagaceae	NS	Markets of Bogotá, Columbia	Anti-microbial [*Herpes*]	Bussmann et al., 2018
Santalum insulare Bertero ex A.DC	Santalaceae	Wo	Maori people, Cook Islands	Anti-microbial [meningitis, earache, boil, skin inf]	Whistler, 1985
Santalum lanceolatum R. Br.	Santalaceae	St	Native Australia	Anti-microbial [cough, cold]	Smith, 1991
Santiria trimera (Oliv.) Aubrev.	Burseraceae	Ro	Masako Forest Reserve, D.R. Congo	Anti-hypertensive	Mbula et al., 2015
Santolina rosmarinifolia L.	Asteraceae	AP	M'Sila, North Algeria	Wound healing	Boudjelal et al., 2013
Sapium laurifolium (A.Rich.) Griseb.	Euphorbiaceae	La	Tacana people, Bolivian Amazon	Anti-microbial [boil; wound	Bourdy et al., 2000
Sapium marmierii Huber.	Euphorbiaceae	La	Peruvian Amazon	Anti-microbial [abscess]	Odonne et al., 2013
Saraca asoca (Roxb.) J.J.de Wilde	Fabaceae	Ba	Mizoram, India	Anti-microbial [STI]; anti-venom	Rai and Lalramnghinglova, 2010
Sarcostemma acidum (Roxb.)	Apocynaceae	WP	3 Kerala tribes, Western Ghats, India	Anti-microbial [rabies]	Marjana et al., 2018
Satureja bachtiarica Bunge	Lamiaceae	AP, Se	Iran	Anti-viral	Mosaddegh et al., 2013
Satureja pulchella (H.B.K.) Briquet	Lamiaceae	Le	Mestizo community, North Peru	Anti-microbial	Bussmann and Sharon, 2006
Satureja thymbra L.	Lamiaceae	AP	Golan Heights and West Bank, Israel	Anti-paralytic	Said et al., 2002

Saurauia sp.	Actinidiaceae	Ba, Le	Eastern Highlands, Papua New Guinea	Anti-inflammatory	Jorim et al., 2012
Saussurea costus (Falc.) Lipsch. Syn: *Saussurea lappa* (Decne.) Sch. Bip.	Compositae	Ro	3 Kerala tribes, Western Ghats, India	Anti-microbial [leprosy]	Marjana et al., 2018
Scaevola taccada (Gaertn.) Roxb.	Goodeniaceae	Le	Andoman + Nicobar Is., India	Anti-paralytic	Chander et al., 2014
Schefflera morototoni (Aubl.) Maguire, Steyerm. & Frodin	Araliaceae	Ro	Mato Grosso, Brazil	Anti-microbial [inf]; anti-inflammatory [rheum pain]	Ribeiro et al., 2017
Schima wallichii (DC.) Korth.	Theaceae	Fr, Le	Mizoram, India	Anxiolytic, anti-microbial [STI]; anti-venom	Rai and Lalramnghinglova, 2010
Schinus areira L.	Anacardiaceae	Le	Comechingones, Argentina	Anti-inflammatory	Goleniowski et al., 2006
Schinus molle L.	Anacardiaceae	Ba, Fr, La, Le, St, WP	Mestizo community, North Peru; Tigrigna people, Central Eritrea; Potosi, Bolivia	Anti-viral, anti-bacterial [bronchitis, cough, cold, TB] anti-inflammatory [rheum]	Bussmann and Sharon, 2006; Yemane et al., 2017; Fernandez et al., 2003
Schinus terebinthifolius Raddi	Anacardiaceae	Le	Três Ladeiras people, Atlantic Forest, Brazil	Anti-inflammatory	Gazzaneo et al., 2005
Schizostachyum lumampao (Blanco) Merr.	Poaceae	Le, Ro, Sh	Ayta communities, Bataan, Philippines	Anti-inflammatory [Fe]	Tantengco et al., 2018
Schkuhria pinnata (Lam.) Kuntze	Asteraceae	WP	Mestizo community, North Peru	Anti-microbial	Bussmann and Sharon, 2006
Schumanniophyton magnificum (K.Schum.) Harms	Rubiaceae	St ba	Baka Pygmies, Gabon	Anti-microbial [diarrh]	Betti et al., 2013
Schwenckia americana L.	Solanaceae	Le with St	Togo; Cataractes and Lukaya districts, D.R. Congo	Memory improvement/ enhancement; anti-inflammatory [rheum, asthma]; anti-microbial [conj, measles]	Adjanohoun et al., 1986: cited in Noe and Lehmann, 2012 [H168]; Latham and Konda ku Mbuta, 2017
Scleria gaertneri Raddi	Cyperaceae	Ro, WP	Mato Grosso, Brazil	Anti-microbial [UTI]; anti-inflammatory	Ribeiro et al., 2017
Scleria scrobiculata Nees & Meyen	Cyperaceae	Le, Ro	Ayta communities, Bataan, Philippines	Anti-microbial [sores]; anti-inflammatory [Fe]	Tantengco et al., 2018
Scolymus hispanicus L.	Asteraceae/Compositae	AP, St	Northeastern Dahra Mountains, NW Algeria	Memory improvement + neurological disorders; anti-inflammatory [rheum]	Senouci et al., 2019
Scoparia dulcis L.	Plantaginaceae	Le, Ro, St, WP	3 states in SW Nigeria; Mato Grosso, Brazil; Martinique; Markets of Bogotá + Antioquia, Columbia; Ayta communities, Bataan, Philippines; Cataractes and Lukaya districts, D.R. Congo; Bac Huong Hoa nature reserve, Vietnam	Memory enhancement; anti-microbial; [influenza, measles, dental caries]; anti-inflammatory [Fe]; anti-hypertensive; anti-venom	Babawale et al., 2016; Mojisola et al., 2012; Ribeiro et al., 2017; Longuefosse and Nossin, 1996; Bussmann et al., 2018; Tantengco et al.,2018; Latham and Konda ku Mbuta, 2017; Chander et al., 2014; Vasquez et al., 2013; Lee et al., 2019
Scrophularia ningpoensis Hemsl.	Scrophulariaceae	Ro	Shaanxi, China	Anti-fungal; anti-hypertensive	Teng et al., 2011
Scutellaria baicalensis Georgi	Labiatae	NS	Shaanxi, China	Anti-fungal	Teng et al., 2011
Scutia myrtina (Burm.f.) Kurz	Rhamnaceae	Le	Gingee Hills, Tamil Nadu, India	Nervous disorders	Arulappan et al., 2015

Sechium edule (Jacq.) Sw.	Cucurbitaceae	Fr	Martinique	Anti-hypertensive	Longuefosse and Nossin, 1996
Securidaca longipedunculata Fresen.	Polygalaceae	Ro	Bas-Congo Province, Cataractes and Lukaya districts, D.R. Congo; Venda, South Africa; Ibadan city, SW Nigeria	Anti-PD; anti-inflammatory; anti-microbial [TB, STI]; dementia, memory, epilepsy	Kembelo, 2003: cited in Noe and Lehmann, 2012 [H130x]; Arnold and Gulumian, 1984; Latham and ku Mbuta, 2017; Gbadamosi and Egunyomi, 2014
Semecarpus anacardium L.f.	Anacardiaceae	Fr, Re	Myanmar; Maharashtra, India	Motor paralysis; anti-inflammatory [neuralgia]; anti-microbial [leprosy]; wound healing	Perry and Metzger, 1980; DeFilipps and Krupnick, 2018; Wadankar et al., 2011
Senecio genisianus Cuatr.	Asteraceae	Le, St	Mestizo community, North Peru	Anti-microbial	Bussmann and Sharon, 2006
Senecio pseudotites Grieseb.	Asteraceae	Le, St	Mestizo community, North Peru	Anti-inflammatory	Bussmann and Sharon, 2006
Senecio rufinervis DC.	Asteraceae	WP	Himachal Pradesh, NW Himalaya, India	Anti-inflammatory [Fe]	Vidyarthi et al., 2013
Senecio scandens Buch.-Ham. ex D. Don	Asteraceae	WP	Maonan people, Guangxi Zhuang, China	Anti-microbial [mumps]	Hong et al., 2015
Senna alata (L.) Roxb. Syn: *Cassia alata* L.	Fabaceae	Le	Martinique; Cataractes and Lukaya districts, D.R. Congo; Ibadan city, SW Nigeria	anti-hypertensive; paralysis/ hemiplegia/ polio/ paraplegia; anti-microbial [skin inf, STI]; anti-inflammatory;	Longuefosse and Nossin, 1996; Adjanohoun et al., 1993: cited in Noe and Lehmann, 2012 [H130]; Latham and Konda ku Mbuta, 2017; Gbadamosi and Egunyomi, 2014
Senna crotalarioides (Kunth) H.S.Irwin & Barneby	Leguminosae	Le	Nuevo León, Mexico	Anti-inflammatory	Estrada-Castillón et al., 2012
Senna hirsuta (L.) H.S. Irwin and Barneby Syn: *Cassia hirsuta* L.	Leguminosae	Le	Peruvian Amazon; Chittagong Hill Tracts, Bangladesh	Anti-inflammatory; anti-venom	Odonne et al., 2013; Kadir et al., 2015
Senna italica Mill.	Leguminosae	Fr, Le	Abyan territory, Yemen; Thari people of Nara Desert, Pakistan	Anti-paralytic; wound healing; anti-inflammatory [Fe, asthma]; anti-microbial [bronch, cough]	Al-Fatimi, 2019; Qureshi and Bhatti, 2008
Senna notabilis (F. Muell.) Randell	Leguminosae	Le	Native Northern territory, Australia	Anti-inflammatory [Fe]	Smith, 1991
Senna pallida (Vahl) H.S. Irwin & Barneby	Leguminosae	NS	Markets of Bogotá, Columbia	Anti-microbial [STI]	Bussmann et al., 2018
Senna petersiana (Bolle) Lock Syn*:* *Cassia petersiana* Bolle	Leguminosae	Le, Ro	Kimboza forest, Tanzania	Anti-inflammatory	Amri and Kisangau, 2012
Senna reticulata (Willd.) H.S. Irwin and Barneby	Leguminosae	AP, Le	Peruvian Amazon; Caqueta Basin, Columbian Amazon	Anti-microbial [measles]	Odonne et al., 2013
Senna sophera (L.) Roxb Syn: *Cassia socotrana* Serrato	Leguminosae	Ba, Fr, Le, Se	Soqotra island, Yemen; Rakhain people, Bangladesh	Anti-viral; Anti-inflammatory [Fe]	Hanif et al., 2009
Senna tora (L.) Roxb. Syn*: Cassia tora*	Leguminosae	Le, Se	Maonan people, Guangxi Zhuang, China; Andoman + Nicobar Is., India; Bac Huong Hoa nature reserve, Vietnam	Anti-microbial [cough]; anti-inflammatory [Fe]	Hong et al., 2015; Chander et al., 2014; Lee et al., 2019
Sesbania sesban (L.) Merr.	Leguminosae	Ba, Le, Ro	Trinidad; Uganda	Anti-microbial [inf, boil]; anti-inflammatory; anti-venom;	Clement et al., 2015; Tugume et al., 2016;

				promote new tissue formation; anti-hypertensive	
Shorea tumbuggaia Roxb.	Dipterocarpaceae	Le	Eastern Ghats, India	Anti-microbial [dys]	Rao et al., 2006
Sida acuta Burm. f.	Malvaceae	Le, Ro	Andoman + Nicobar Is., India; Karnataka, India; Chittagong Hill Tracts, Bangladesh; Mizoram, India	Anti-inflammatory [Fe]; anti-microbial [dys, boil]; anti-paralytic; anti-venom; nervous diseases	Chander et al., 2014; Rajakumar and Shivanna, 2009;; Kadir et al., 2015; Rai and Lalramnghinglova, 2010
Sida cordata (Burm. f.) Borss. Waalk.	Malvaceae	Le	India	Anti-paralytic	Mikawlrawng et al., 2017
Sida rhombifolia L.	Malvaceae	Le, Ro	Andoman + Nicobar Is., India; Cataractes and Lukaya districts, D.R. Congo; Chittagong Hill Tracts, Bangladesh; Tacana people, Bolivian Amazon	Anti-hypertensive; anti-microbial [STI, diarrh, boil]; anti-venom	Chander et al., 2014; Latham and Konda ku Mbuta, 2017; Kadir et al., 2015; Bourdy et al., 2000
Sida tenuicarpa Vollesen	Malvaceae	Le, Ro	Kakamega County, Kenya	Anti-microbial [boil]	Odongo et al., 2018
Sida urens L.	Malvaceae	Le	South Nigeria	Anti-microbial [skin inf]	Borokini et al., 2012
Sideritis hirsuta L.	Lamiaceae	AP	Catalonia, Spain	Anti-hypertensive	Raja et al., 1997
Sideritis perfoliata subsp. athoa (Papan. & Kokkini) Baden Syn: *Sideritis athoa* Papan. & Kokkini	Lamiaceae	AP	Edremit Gulf, Turkey	Anti-microbial [cold, influenza]	Polat and Satıl, 2012
Sideritis raeseri Boiss. & Heldr.	Lamiaceae	AP, FL	Gheg people, NE Albania	Anti-microbial [sore throat, cough]	Pieroni and Sõukand, 2017
Sigesbeckia orientalis L.	Asteraceae	Le	Myanmar; Maonan people, Guangxi Zhuang, China	Anti- venom; anti-paralytic; anti-hypertensive	DeFilipps and Krupnick, 2018; Hong et al., 2015
Silene macrosolen Steud. ex A. Rich.	Caryophyllaceae	Le	Afar people, Rift Valley, Ethiopia	Anti-microbial [diphth]	Teklehaymanot, 2017
Silybum marianum (L.) Gaertn.	Asteraceae/Compositae	Ro	Agro Nocerino Sarnese, Southern Italy	Anti-inflammatory [rheum]	Motti and Motti, 2017
Simaba ferruginea A. St.-Hil.	Simaroubaceae	Ro	Mato Grosso	Anti-inflammatory [rheum, Fe]; cholesterol-reducing	Ribeiro et al., 2017
Simarouba amara Aubl.	Simaroubaceae	NS	Markets of Bogotá, Columbia	Anti-inflammatory [Fe]	Bussmann et al., 2018
Simarouba glauca DC.	Simaroubaceae	Le	3 Kerala tribes, Western Ghats, India	Boost immunity	Marjana et al., 2018
Sinomenium acutum (Thunb.) Rehder & E.H. Wilson	Menispermaceae	St	Shaanxi, China	Anti-inflammatory	Teng et al., 2011
Siparuna guianensis Aubl.	Siparunaceae	Le, Vi	Mato Grosso, Brazil; Caqueta Basin, Columbian Amazon	Anti-inflammatory [rheum, pain]; anti-microbial [influenza]	Ribeiro et al., 2017
Sisyrinchium tinctorium Kunth	Iridaceae	NS	Markets of Bogotá, Columbia	Anti-microbial [influenza, pneum, STI]	Bussmann et al., 2018
Skimmia laureola (DC.) Decne.	Rutaceae	EO, Le	Jammu, Kashmir + Pakistan	Anti-viral [s'pox]	Amjad et al., 2017
Smilax aspera Wall.	Smilacaceae	Ro	Himachal Pradesh, NW Himalaya, India	Anti-microbial [STI]	Vidyarthi et al., 2013
Smilax corbularia Kunth	Smilacaceae	Ro O	Bac Huong Hoa nature reserve, Vietnam	Anti-microbial [STI]	Lee et al., 2019

Smilax irrorata Mart. ex Griseb.	Smilacaceae	FL, Ro	Mato Grosso, Brazil	Anti-microbial; anti-inflammatory	Ribeiro et al., 2017
Smilax aristolochiifolia Mill. Syn: *Smilax medica* M.Martens & Galeotti	Smilacaceae	St	Mestizo community, North Peru	Anti-inflammatory	Bussmann and Sharon, 2006
Smilax rotundifolia L.	Smilacaceae	Le	Eastern Highlands, Papua New Guinea	Anti-inflammatory [Fe]	Jorim et al., 2012
Smilax rufescens Griseb.	Smilacaceae	Le, Rh	Mato Grosso, Brazil	Anti-microbial	Ribeiro et al., 2017
Smilax siphilitica Humb. & Bonpl. ex Willd.	Smilacaceae	NS	Markets of Bogotá, Columbia	Anti-microbial [STI]	Bussmann et al., 2018
Smilax sp.	Smilacaceae	WP	Três Ladeiras people, Atlantic Forest, Brazil	Anti-inflammatory	Gazzaneo et al., 2005
Socratea exorrhiza (Mart.) H.Wendl.	Arecaceae	Ro	Tacana people, Bolivian Amazon	Anti-venom	Bourdy et al., 2000
Solanecio mannii (Hook.f.) C. Jeffrey Syn*: Crassocephalum mannii* (Hook.f.) Milne-Redh.	Asteraceae/Compositae	AP, Le	Uganda; Nyanza and Kamba people, Kenya; Nyanza and Kamba people, Kenya; Haya people, Kagera, NW Tanzania	Anti-microbial [polio]; anti-venom; anti-convulsant	Adjanohoun et al., 1989: cited in Noe and Lehmann, 2012 [H130]; Owuor and Kisangau, 2006; Moshi et al., 2009
Solanum aethiopicum L.	Solanaceae	Fr	Benin; Cataractes and Lukaya districts, D.R. Congo	Hemiplegia/ paraplegia/ polio; anti-hypertensive	Adjanohoun et al., 1989: cited in Noe and Lehmann, 2012 [H130]; Latham and Konda ku Mbuta, 2017
Solanum caricaefolium Rusby	Solanaceae	Le	Peruvian Amazon	Anti-microbial [abscess]	Odonne et al., 2013
Solanum crispum Ruiz & Pav.Syn: *Solanum ligustrinum* Loddigges	Solanaceae	NS	Mapuche people, Argentina	Anti-inflammatory	Estomba et al., 2005
Solanum dasyphyllum Schumach. & Thonn.	Solanaceae	AP, Fr	Benin; Bas-Congo; Mabira Forest, Uganda	Hemiplegia/ paraplegia/ polio; anti-microbial [wart]	Adjanohoun et al., 1989: cited in Noe and Lehmann, 2012 [H130]; Tugume et al., 2016
Solanum huallagense Bitter.	Solanaceae	Le	Peruvian Amazon	Anti-microbial [abscess]	Odonne et al., 2013
Solanum inaequilaterale Merr.	Solanaceae	Le [In sp. comb]	Maranao people, Philippines	Anti-inflammatory [Fe]	Malawani et al., 2017
Solanum incanum L.	Solanaceae	Le, Fr, Ro	3 states in SW Nigeria; Mabira Forest, Uganda; Nyanza and Kamba people, Kenya; Mana Angetu District, SE Ethiopia; Burkina Faso	Memory enhancement; anti-microbial [influenza]; anti-venom; anti-inflammatory [headache, eye inflammation]	Babawale et al., 2016; Mojisola et al., 2012; Tugume et al., 2016; Owuor and Kisangau, 2006; Lulekal et al., 2008; de la Pradilla, 1981: cited in Noe and Lehmann, 2012 [H001]
Solanum indicum L. Syn: *Solanum anguivi* Lam.	Solanaceae	Fr, Ro, Se, WP	Mabira Forest, Uganda; Myanmar; Abyan territory, Yemen; Eastern Ghats, India	Anti-microbial [leprous sore, measles, RTI]; boost energy; anti-inflammatory [Fe]; boost immune system; anti-venom	Tugume et al., 2016; DeFilipps and Krupnick, 2018; Al-Fatimi, 2019; Rao et al., 2006
Solanum lycocarpum A. St.-Hil.	Solanaceae	Fr	Mato Grosso, Brazil	Anti-microbial [cough]; anti-epileptic; anti-inflammatory	Ribeiro et al., 2017

Solanum lycopersicum Syn: *Lycopersicon esculentum* Mill.	Solanaceae	Fr	Rio Jauaperi, Brazilian Amazon	Anti-inflammatory; anti-microbial [abscess]	Pedrollo et al., 2016
Solanum marginatum L. f.	Solanaceae	Le	Afar people, Rift Valley, Ethiopia	Anti-microbial [mumps, meningitis]	Teklehaymanot, 2017
Solanum melongena L.	Solanaceae	Ro	Markets of Bogotá, Columbia; Andoman + Nicobar Is., India; Ayta communities, Bataan, Philippines	Anti-hypertensive; anti-venom	Bussmann et al., 2018; Chander et al., 2014; Tantengco et al., 2018
Solanum mite Ruiz et Pav.	Solanaceae	AP	Peruvian Amazon	Anti-microbial [skin inf]	Odonne et al., 2013
Solanum nigrum Linn.	Solanaceae	Fr, Le	Orang Asli tribe, Malaysia; Mabira Forest, Uganda; Markets of Bogotá, Columbia; Maori people, Cook Islands	Anti-microbial [boil, UTI, *Herpes*]; boost immune system; anti-inflammatory	Samuel et al., 2010; Tugume et al., 2016; Bussmann et al., 2018; Whistler, 1985
Solanum peruvianum L. Syn: *Lycopersicon peruvianum* (L.) Mill.	Solanaceae	WP	Mestizo community, North Peru	Anti-inflammatory; anti-microbial	Bussmann and Sharon, 2006
Solanum sisymbriifolium Lam.	Solanaceae	Fr, Le, St	Comechingones people, Argentina; Pilaga Indians, Chaco, Northern Argentina	Anti-microbial [STI]	Goleniowski et al., 2006;
Solanum somalense Franch. in Revoil	Solanaceae	Le	Afar people, Rift Valley, Ethiopia	Anti-microbial [typhoid, dys]	Teklehaymanot, 2017
Solanum torvum Sw.	Solanaceae	Fr, Le, Ro	Martinique; Fundong, NW Cameroon	Anti-hypertensive; anti-microbial [influenza, pneum, TB]; anti-inflammatory [Fe]	Longuefosse and Nossin, 1996; Focho et al., 2009
Solanum tuberosum L.	Solanaceae	Fr, Tu	Mestizo community, North Peru; Yörük people, SE Macedonia	Anti-microbial [bronch]; anti-inflammatory [rheum]	Bussmann and Sharon, 2006; Nedelcheva et al., 2017
Solanum uporo Dunal [unresolved] Syn: *Solanum viride* G.Forst. ex Biehler	Solanaceae	Le	Tonga	Anti-microbial [boil]	Croft and Tu'ipulotu, 1980
Solenomelus segethi (Phil.) Kuntze Syn: *Susarium segethii* Phil. (ind)	Iridaceae	Ju	Mapuche people, Chile	Anti-inflammatory [Fe]	Houghton and Manby, 1985
Solenostemma oleifolium (Nectoux) Bullock & E.A.Bruce ex Maire	Apocynaceae	AP, Fr, Le	Tassili N'Ajjer, Southern Algerian Sahara	Anti-inflammatory [Fe]; anti-microbial [measles, sore]; wound healing	Hammiche and Maiza, 2006
Solidago californica Nutt.	Compositae	NS	Chumash Indians, California, USA	Anti-microbial [cough]	Timbrook, 1990
Solidago chilensis Meyen	Compositae	Le, Ro, WP	Mato Grosso, Brazil	Anti-inflammatory	Ribeiro et al., 2017
Soliva sessilis Ruiz and Pav.	Compositae	Le	Mapuche people, Chile	Anti-microbial [dys]	Houghton and Manby, 1985
Sonchus asper Wulf.	Asteraceae	Le	Mapuche people, Chile	Anti-microbial [abscess, boil]	Houghton and Manby, 1985
Sonchus oleraceus L.	Asteraceae	Le, Sh	Loja and Zamora-Chinchipe, Ecuador	Anti-viral	Tene et al., 2007
Sorbus aucuparia L.	Rosaceae	Fr	Edremit Gulf, Turkey	Anti-hypertensive	Polat and Satıl, 2012
Sorghum bicolor (L.) Moench Syn: *Holcus bicolor* L.	Poaceae	Fr, Ro	Ségué, Mopti region, Mali; Abyan territory, Yemen	Paralysis/ hemiplegia/ polio/ paraplegia; anti-microbial [UTI]	Somboro et al., 2011: cited in Noe and Lehmann, 2012 [H130]; Al-Fatimi, 2019

*Soymida febrifuga (*Roxb.) A.Juss.	Meliaceae	Le	Eastern Ghats, India	Anti-venom	Rao et al., 2006
Sparrmannia africana L. f.	Malvaceae	NS	Markets of Bogotá, Columbia	Anti-microbial [UTI]	Bussmann et al., 2018
Spartium junceum L.	Leguminosae	FL	Potosi, Bolivia	Anti-inflammatory [headache]	Fernandez et al., 2003
Spathodea campanulata P. Beauv.	Bignoniaceae	Le, Ro	Mabira Forest, Uganda; Kakamega County, Kenya; Fundong, NW Cameroon + Tropical rain forest, Central + S Cameroon	Anti-microbial [skin/ tongue inf]; anti-viral [HSV, c'pox]; mental disorders	Tugume et al., 2016; Odongo et al., 2018; Ngono Ngane et al., 2011; Focho et al.,2009
Spermacoce princeae (K. Schum.) Verdc.	Rubiaceae	Le, Ro	Kakamega County, Kenya	Anti-microbial [boil]	Odongo et al., 2018
Spermacoce verticillata L. Syn: *Borreria verticillata* (L.) G. Mey Rub WP	Rubiaceae	Le, WP	Trinidad: Três Ladeiras people, Atlantic Forest, Brazil	Anti-hypertensive; anti-inflammatory [Fe]	Clement et al., 2015; Gazzaneo et al., 2005
Spilanthes acmella (L.) L. Syn: *Blainvillea acmella* (L.) Philipson	Compositae	FL, Ro	India	Anti-paralytic	Mikawlrawng et al., 2017
Spilanthes oppositifolia (Lam.) D'Arcy	Compositae	NS	Markets of Bogotá, Columbia	Anti-microbial [mouth inf]	Bussmann et al., 2018
Spinacia oleracea L	Amaranthaceae	Le	M'Sila, North Algeria	Anti-inflammatory	Boudjelal et al., 2013
Spiranthera odoratissima A. St.-Hil.	Rutaceae	Ro, St	Mato Grosso, Brazil	Anti-inflammatory	Ribeiro et al., 2017
Spiranthes sinensis (Pers.) Ames BM	Orchidaceae	Rh	Magar and Majhi people, Nepal	Anti-microbial [sore throat, boil]	Malla et al., 2015
Spondias dulcis Parkinson Syn: *Spondias cytherea* Sonn	Anacardiaceae	Le	Andoman + Nicobar Is., India; Maori people, Cook Islands	Anti-inflammatory [Fe]; anti-microbial [UTI, anti-fungal]	Chander et al., 2014; Whistler, 1985
Spondias pinnata (L.f.) Kurz	Anacardiaceae	Ba	Mizoram, India	Anti-microbial [dys]; anti-inflammatory [rheum]	Rai and Lalramnghinglova, 2010
Spondias purpurea L.	Anacardiaceae	Fr	Ayta communities, Bataan, Philippines	Anti-microbial [sore]	Tantengco et al., 2018
Sporobolus indicus (L.) R. Br.	Poaceae	NS	Markets of Bogotá, Columbia	Anti-microbial [UTI]	Bussmann et al., 2018
Stachys obliqua Waldst. & Kit.	Lamiaceae	Br, FL	Edremit Gulf, Turkey	Anti-microbial [cold, influenza]	Polat and Satıl, 2012
Stachys lavandulifolia Vahl	Lamiaceae	AP	Iran	Anti-microbial	Mosaddegh et al., 2013
Stachys pilifera Benth	Lamiaceae	AP	Iran	Anti-microbial	Mosaddegh et al., 2013
Stachytarpheta cayennensis (Rich.) Vahl	Verbenaceae	AP, Le, Ro, WP	Mato Grosso, Brazil; Tacana people, Bolivian Amazon	Anti-microbial [measles]; anti-inflammatory	Ribeiro et al., 2017; Bourdy et al., 2000
Stachytarpheta elatior Schrad.	Verbenaceae	Ro	Três Ladeiras people, Atlantic Forest, Brazil	Anti-microbial [influenza]	Gazzaneo et al., 2005
Stachytarpheta jamaicensis (L.) Vahl	Verbenaceae	Le	Martinique; Trinidad	Anti-inflammatory; anti-eye inflammation; anti-hypertensive; anti-microbial [cold]	Longuefosse and Nossin, 1996; Lavergne and Véra, 1989: cited in Noe and Lehmann, 2012 [H001]; Clement et al., 2015
Staudtia kamerunensis var. gabonensis (Warb.) Fouilloy	Myristicaceae	Ba	Cataractes and Lukaya districts, D.R. Congo	Anti-microbial [skin/ mouth inf]	Latham and Konda ku Mbuta, 2017

Steganotaenia araliacea Hochst.	Apiaceae	AP	Haya people, Kagera, NW Tanzania	Anti-viral [*H. zoster*, HIV]	Moshi et al., 2009
Stellaria media (L.) Vill.	Caryophyllaceae	NS	Mapuche people, Argentina	Anti-inflammatory	Estomba et al., 2005
Stemona tuberosa Lour.	Stemonaceae	Tu	Maonan people, Guangxi Zhuang, China	Anti-microbial [TB]	Hong et al., 2015
Stenotaphrum secundatum (Walter) Kuntze	Poaceae	Le	Martinique	Anti-inflammatory	Longuefosse and Nossin, 1996
Stephania abyssinica (Dillon. & A. Rich.) Walp.	Menispermaceae	Le, Ro	Kakamega County, Kenya; Debre Libanos Wereda, central Ethiopia	Anti-microbial [STI]; wound healing	Odongo et al., 2018; Getaneh and, Girma, 2014
Sterculia guttata Roxb	Malvaceae	Le	Andoman + Nicobar Is., India	Anti-inflammatory [Fe]	Chander et al., 2014
Sterculia setigera Delile	Malvaceae	Le	Togo	Anti-epileptic	Kantati et al., 2016
Sterculia tragacantha Lindl.	Malvaceae	Br	Mount Cameroon, Cameroon; Cataractes and Lukaya districts, D.R. Congo	Anti-microbial [STI, cough]; anti-inflammatory [Fe]	Sandberg et al., 2005; Latham and Konda ku Mbuta, 2017
Sterculia urens Roxb.	Malvaceae	Re	Gingee Hills, Tamil Nadu, India	Anti-microbial	Arulappan et al., 2015
Stereospermum kunthianum Cham.	Bignoniaceae	Ro	Togo	Anti-epileptic; anti-paralytic; TBI	Kantati et al., 2016
Stevia rebaudiana (Bertoni) Bertoni	Asteraceae	NS	Markets of Bogotá, Columbia	Anti-hypertensive; anti-microbial [influenza]	Bussmann et al., 2018
Streblus asper Lour.	Moraceae	Fr, St	Ayta communities, Bataan, Philippines; Chittagong Hill Tracts, Bangladesh	Anti-microbial [sore throat]; anti-venom	Tantengco et al., 2018; Kadir et al., 2015
*Streblus dimepate (*Bureau) C.C. Berg	Moraceae	Ba	Maromizaha forest, Madagascar	Anti-fatigue	Riondato et al., 2019
Strobilanthes crispa Blume	Acanthaceae	Le	Orang Asli tribe, Malaysia	Enhance immune system	Samuel et al., 2010
Strobilanthes cusia (Nees) Kuntze Syn: *Baphicacanthus cusia* (Nees) Bremek.	Acanthaceae	WP	Maonan people, Guangxi Zhuang, China	Anti-microbial	Hong et al., 2015
Strychnos pseudoquina A.St.-Hil.	Loganiaceae	Ba	Mato Grosso, Brazil	Anti-microbial; anti-inflammatory	Ribeiro et al., 2017
Stryphnodendron adstringens (Mart.) Coville	Fabaceae	Ba, Le, sap, Se	Mato Grosso, Brazil	Anti-microbial; anti-inflammatory	Ribeiro et al., 2017
Stryphnodendron obovatum Benth.	Leguminosae	Ba, Le	Mato Grosso, Brazil	Anti-microbial; anti-inflammatory	Ribeiro et al., 2017
Sudamerlycaste gigantea (Lindl.) Syn: *Lycaste gigantea* Lindl.	Orchidaceae	St	Mestizo community, North Peru	Anti-inflammatory	Bussmann and Sharon, 2006
Swertia ciliata (D.Don) C.B.Clarke	Gentianaceae	AP	Toli Peer National Park, Kashmir, Pakistan	Anti-microbial	Amjad et al., 2017; Bhattarai et al., 2006
Swertia racemosa (Griseb.) Wall. ex C.B.Clarke	Gentianaceae	WP	Central Himalayas, Nepal	Anti-microbial [cough, cold]; anti-inflammatory [Fe, headache]	Bhattarai et al., 2006
Symmeria paniculata Benth.	Polygonaceae	Ro, St ba	Rio Jauaperi, Brazilian Amazon	Anti-microbial	Pedrollo et al., 2016
Symphonia sp.	Clusiaceae	Le	Três Ladeiras people, Atlantic Forest, Brazil	Anti-microbial [dys]	Gazzaneo et al., 2005

Symphyotrichum puniceum (L.) Á.Löve & D.Löve	Compositae	Ro	Native America	Anti-paralytic	Leighton, 1985: cited in Native American Ethnobotany Database
Symphytum officinale L.	Boraginaceae	Le, Ro	Mato Grosso, Brazil; Martinique	Anti-inflammatory [Fe, rheum, headache]	Ribeiro et al., 2017; Longuefosse and Nossin, 1996
Synsepalum cerasiferum (Welw.) T.D.Penn	Sapotaceae	St ba	Haya people, Kagera, NW Tanzania	Anti-microbial [STI UTI TB]	Moshi et al., 2009
Synedrella nodiflora (L.) Gaertn.	Asteraceae	NS	Cataractes and Lukaya districts, D.R. Congo	Anti-microbial [leprosy, mouth sore, conj]	Latham and Konda ku Mbuta, 2017
Syzygium aromaticum (L.) Merr. & L.M.Perry Syn: *Eugenia caryophyllus* (Spreng.) Bullock & S.G.Harrison	Myrtaceae	Bu, Le	Carib people, Livingston, Guatamala; N + S Oman; Yörük people, SE Macedonia	Anti-inflammatory [Fe, toothache]; anti-microbial [mouth sore]	Girón et al., 1991; Divakar et al., 2016; Nedelcheva et al., 2017
Syzygium cumini (L.) Skeels	Myrtaceae	Le, St ba, Se	Rio Jauaperi, Brazilian Amazon, Rakhain people, Bangladesh; Karnataka, India	Anti-microbial [dys]; anti-inflammatory	Pedrollo et al., 2016; Hanif et al., 2009; Rajakumar and Shivanna, 2009
Syzygium jambos (L.) Alston Syn: *Eugenia malaccensis* Blanco	Myrtaceae	Ba	Sundanese community, West Java	Anti-bacterial [dys]	Roosita et al., 2008
Syzygium malaccense (L.) Merr. & L.M.Perry	Myrtaceae	Le	Rio Jauaperi, Brazilian Amazon	Anti-microbial	Pedrollo et al., 2016
Tabebuia aurea (Silva Manso) Benth. & Hook.f. ex S.Moore	Bignoniaceae	Ba, FL, St	Mato Grosso, Brazil	Anti-microbial [inf, influenza], weakness	Ribeiro et al., 2017
Tabebuia rosea DC.	Bignoniaceae	Ba, St	Caribbean coast of Columbia	Anti-inflammatory [Fe]	Gómez-Estrada et al., 2011
Tabernaemontana crispa L.	Apocynaceae	Le	Andoman + Nicobar Is., India	Anti-inflammatory [Fe]	Chander et al., 2014
Tabernaemontana divaricata (L.) R.Br. ex Roem. & Schult. Syn: *Ervatamia coronaria* (Jacq.) Stapf	Apocynaceae	Ba, Le, Ro	Mizoram, India	Anti-venom; anti-microbial [toothache]	Rai and Lalramnghinglova, 2010
Tabernaenontana elegans Stapf	Apocynaceae	Ro	Venda, South Africa	Anti-microbial [STI]	Arnold and Gulumian, 1984
Tacca leontopetaloides (L.) Kuntze	Dioscoreaceae	Starch	Maori people, Cook Islands	Anti-microbial [boil]; wound	Whistler, 1985
Tadehagi triquetrum (L.) H.Ohashi	Fabaceae	Ro	Bac Huong Hoa nature reserve, Vietnam	Anti-microbial [cough]	Lee et al., 2019
Tagetes pusilla Kunth	Asteraceae/Compositae	AP	Potosi, Bolivia	Anti-microbial [bronch]; neuralgic pain	Fernandez et al., 2003
Tagetes erecta L.	Asteraceae/Compositae	FL, Le	Mestizo community, North Peru	Anti-inflammatory	Bussmann and Sharon, 2006
Tagetes terniflora Kunth Syn: *Tagetes graveolens* L'Hér.	Asteraceae/Compositae	AP, Le, Ro	Loja and Zamora-Chinchipe, Ecuador	Anti-fungal; Anti-inflammatory {Fe]	Tene et al., 2007
Tamarindus indica L.	Fabaceae	Ba, Fr, Le, Se	Martinique; Jamaica; Trinidad; Mauritius; Sundanese community, West Java; Abyan territory, Yemen; Tigrigna people, Central Eritrea; Conis Santana National Park, East Timor	Anti-venom; anti-microbial [dys, lung + mouth inf, typhoid, measles, cough, cold]; anti-hypertensive; memory improvement; anti-inflammatory [Fe, pain, rheum]	Nordal, 1963: cited in DeFilipps and Krupnick, 2018; Longuefosse and Nossin, 1996; Clement et al., 2015; Adjanohoun et al., 1983: cited in Noe and Lehmann, 2012 [H130, H168]; Roosita et al., 2008; Al-

					Fatimi, 2019; Yemane et al., 2017; Collins et al., 2007
Tamarix aphylla (L.) H.Karst.	Tamaricaceae	AP	N'Ajjer Southern Algerian Sahara	Anti-inflammatiory [Fe, eye]	Hammiche and Maiza, 2006
Tapinanthus globiferus (A. Rich) Van Tiegh	Loranthaceae	Le	South Nigeria	Anti-hypertensive	Borokini et al., 2012
Taraxacum mongolicum Hand.-Mazz.	Asteraceae	AP, Ro	Tian Mu Shan, Zhejiang, China	Anti-microbial [TB, measles, HSV]	Chaudhary et al., 2006
Tephrosia purpurea (L.) Pers.	Fabaceae	WP	Togo	Dementia, anti-epileptic, anti-paralytic	Kantati et al., 2016
Tephrosia villosa (L.) Pers.	Fabaceae	WP	Togo	Anti-stroke	Kantati et al., 2016
Terminalia argentea Mart.	Combretaceae	Ba, FL, Le, Ro	Mato Grosso, Brazil	Anti-microbial [influenza, bronch]; anti-inflammatory [inflam, rheum, pain]	Ribeiro et al., 2017
Terminalia arjuna (Roxb. ex DC.) Wight & Arn.	Combretaceae	Ba	Dinajpur District, Bangladesh	Heart disease, anti-inflammatory [rheum]	Rahmatullah et al., 2009
Terminalia catappa L.	Combretaceae	Bu	Carib people, Livingston, Guatamala; Ibadan city, SW Nigeria	Anti-hypertensive; anti-microbial {STI]	Girón et al., 1991; Gbadamosi and Egunyomi, 2014
Terminalia glabrata var. *brownii* Fosberg & Sachet	Combretaceae	Fr, Le	Marquesas Islands, French Polynesia	Anti-viral [measles]	Girardi et al., 2015
Terminalia sericea Burch. ex DC.	Combretaceae	Ro, Le	South Africa; Kimboza forest, Tanzania	Anti-microbial [STI, infected wound, anti-fungal]	Arnold and Gulumian, 1984; Amri and Kisangau, 2012
Tessaria integrifolia Ruiz & Pav.	Asteraceae	Bd, FL, Le	Mestizo community, North Peru; Tacana people, Bolivian Amazon	Anti-inflammatory; anti-venom	Bussmann and Sharon, 2006; Bourdy et al., 2000
Tetradenia riparia (Hochst.) Codd	Lamiaceae	Le	Mabira Forest, Uganda	Anti-microbial [eye, ear + g-i inf]	Tugume et al., 2016
Tetradium ruticarpum (A.Juss.) T.G.Hartley Syn: *Euodia rutaecarpa* (Juss.) Benth.	Rutaceae	WP	Tian Mu Shan, Zhejiang, China	Anti-microbial	Chaudhary et al., 2006
Tetraena simplex (L.) Beier & Thulin Syn: *Zygophyllum simplex* L. Mant. Pl.	Zygophyllaceae	Le, WP	Jazan province, Saudi Arabia; Tassili N'Ajjer, Southern Algerian Sahara	Anti-inflammatory [eye]; anti-microbial [abscess, boil]	Tounekti et al., 2019; Hammiche and Maiza, 2006
Tetragonia crystallina L'Herit	Aizoaceae	FL, Le, St	Mestizo community, North Peru	Anti-inflammatory	Bussmann and Sharon, 2006
Tetrapleura tetraptera (Schumach. & Thonn.) Taub	Fabaceae	Fr	Ondo State, SW Nigeria	Memory improvement	Mojisola et al., 2012
Tetrorchidium didymostemon (Baill.) Pax & K.Hoffm.	Euphorbiaceae	La, Le	Mabira Forest, Uganda; Cataractes and Lukaya districts, D.R. Congo	Anti-microbial [measles, leprosy]; anti-inflammatory [pain]	Tugume et al., 2016; Latham and Konda ku Mbuta, 2017
Teucrium mascatense Boiss	Lamiaceae	Le, St	N + S Oman	Anti-inflammatory [Fe]; anti-microbial	Divakar et al., 2016
Teucrium polium L.	Lamiaceae	AP	M'Sila, North Algeria;Tassili N'Ajjer, Southern Algerian Sahara	Wound healing, anti-hypertensive; anti-inflammatory [Fe]	Boudjelal et al., 2013; Hammiche and Maiza, 2006

Thalictrum foliolosum DC.	Ranunculaceae	WP	Mao Naga people Manipur, India	Anti-microbial [dys]	Lokho, 2012
Thapsia garganica L.	Apiaceae	AP	M'Sila, North Algeria	Anti-inflammatory	Boudjelal et al., 2013
Theobroma cacao L.	Malvaceae	Ba, Fr, St	Martinique; Mestizo community, North Peru; Sagamu, SW Nigeria; Mashhad markets, Iran	Anti-inflammatory [Fe]; anti-aging; tonic	Longuefosse and Nossin, 1996; Bussmann and Sharon, 2006; Elufioye et al., 2012; Amiri and Joharchi, 2013
Thespesia populnea (L.) Sol.ex Corrêa	Malvaceae	Fr	Marquesas Islands, French Polynesia; Maori people, Cook Islands	Anti-microbial [ringworm, wart, UTI]	Girardi et al., 2015; Whistler, 1985
Thonningia sanguinea Vahl	Balanophoraceae	WP	Cataractes and Lukaya districts, D.R. Congo	Anti-paralytic; anti-microbial [dys, STI, mouth inf, abscess]	Latham and Konda ku Mbuta, 2017
Thymbra capitata (L.) Cav. Syn: *Coridothymus capitatus* (L.) Rchb.f.	Lamiaceae	FL, Le	Golan Heights and West Bank, Israel	Anti-paralytic; anti-inflammatory	Krispil, 2000: cited in Abu-Rabia, 2012; Said et al., 2002
Thymbra spicata L.	Lamiaceae	FL Br, Le, St	Israel; Middle East; Edremit Gulf, Turkey	Anti-paralytic; anti-microbial [cold, influenza, cough]	Krispil, 2000: cited in Abu-Rabia, 2012; Polat and Satıl, 2012
Thymus linearis Benth.	Lamiaceae	AP	Nepal Himalayas	Anti-microbial [eye inf, gingivitis]	Bhattarai et al., 2006
Thymus longicaulis C.Presl	Lamiaceae	AP	Edremit Gulf, Turkey	Anti-microbial [cold, influenza]	Polat and Satıl, 2012
Thymus satureioides Coss.	Lamiaceae	AP	High Atlas, Morocco	Anti-microbial [cold]; anti-inflammatory [Fe, headache]	Fadili *et al*, 2017
Thymus serpyllum L. Syn: *Thymus ciliatus* Lam.	Lamiaceae	AP, WP	Algeria; Albanians, Bosniaks/Gorani + Turkish ethnic groups, Kosovo	Anti-microbial [bronc]; immunostimulant	Mustafa et al., 2015; Boudjelal et al., 2013
Thymus vulgaris L.	Lamiaceae	FL, Le	Markets of Bogotá, Columbia; N + S Oman; Catalonia, Spain	Anti-inflammatory [rheum]; anti-microbial; anxiolytic	Bussmann et al., 2018; Divakar et al., 2016; Raja et al., 1997
Thymus zygioides Griseb.	Lamiaceae	AP	Edremit Gulf, Turkey	Anti-microbial [cold, influenza]	Polat and Satıl, 2012
Tilia tomentosa Moench	Malvaceae)	FL	Edremit Gulf, Turkey	Anti-microbial [cold, influenza, cough]	Polat and Satıl, 2012
Tinospora caffra (Miers) Troupin	Menispermaceae	Le	Canhane, Massingir, Mozambique	Anti-paralytic	Ribeiro et al., 2010: cited in Noe and Lehmann, 2012 [H130]
Tinospora cordifolia (Thunb.) Miers	Menispermaceae	Le, St	3 Kerala tribes, Western Ghats, India; Mizoram, India; Myanmar	Anti-microbial [STI; anti-inflammatory [rheum]; poison antidote<br>* Toxicity Report	Marjana et al., 2018; Rai and Lalramnghinglova, 2010; DeFilipps and Krupnick, 2018<br>* Romagosa et al., 2016
Tinospora rumphii Boerl.	Menispermaceae	Le	Ayta communities, Bataan, Philippines	Anti-inflammatory [Fe]	Tantengco et al., 2018
Tinospora sinensis (Lour.) Merr.	Menispermaceae	St	Chin people, Myanmar; Huanjiang Maonan people, Guangxi Zhuang, China	Anti-hypertensive; anti-inflammatory [rheum]	Ong et al., 2018; Hong et al., 2015
Tinospora smilacina Benth.	Menispermaceae	NS	Conis Santana National Park, East Timor	Anti-venom	Collins et al., 2007
Tiquilia paronychioides (Phil.) A.T. Richardson	Boraginaceae	FL	Mestizo community, North Peru	Anti-inflammatory; anti-microbial	Bussmann and Sharon, 2006
Toddalia asiatica (L.) Lam.	Rutaceae	Le, Ro, St	Kakamega County, Kenya; Eastern Ghats, India	Anti-microbial [throat inf]; anti-epileptic	Odongo et al., 2018; Rao et al., 2006

Toona ciliata M.Roem.	Meliaceae	Ba, FL	Mizoram, India	Anti-microbial [dys]; anti-ulcer	Rai and Lalramnghinglova, 2010
Tournefortia hirsutissima L. Syn: *Heliotropium verdcourtii* Craven	Boraginacece	Le, St	Trinidad; Jamaica	Anti-microbial [cold, cough]	Clement et al., 2015;
Trachycarpus fortunei (Hook.) H.Wendl.	Arecaceae	Le	Hakka people, Guangdong, China	Anti-hypertensive	Au et al., 2008
Trachyphrynium braunianum (K.Schum.) Baker	Marantaceae	Le	Cataractes and Lukaya districts, D.R. Congo	Anti-inflammatory	Latham and Konda ku Mbuta, 2017
Tradescantia pallida (Rose) D.R.Hunt Syn: *Setcreasea purpurea* Boom	Commelinaceae	WP	Maonan people, Guangxi Zhuang, China	Anti-microbial [*Herpes*]	Hong et al., 2015
Tradescantia sp.	Commelinaceae	St	Loja and Zamora-Chinchipe, Ecuador	Anti-inflammatory	Tene et al., 2007
Tradescantia spathacea Sw. Syn: *Rhoeo spathacea* (Sw.) Stearn	Commelinaceae	FL, Le	Martinique	Anti-inflammatory	Longuefosse and Nossin, 1996
Tradescantia zebrina Heynh.	Commelinaceae	FL, Le	Loja and Zamora-Chinchipe, Ecuador; Columbia	Anti-microbial [int inf]; anti-inflammatory [rheum]	Tene et al., 2007; Cadena-González et al., 2013
Treculia africana Decne. ex Trécul	Moraceae	Se	Cataractes and Lukaya districts, D.R. Congo	Anti-microbial [dental caries]	Latham and Konda ku Mbuta, 2017
Trema orientalis (L.) Blume	Cannabaceae	Ba, Le	D.R.Congo; Cameroon, Gabon	Paralysis/ hemiplegia/ polio/ paraplegia; anti-inflammatory [toothache]; anti-microbial [cough]	De Graer, 1929: cited in Noe and Lehmann, 2012 [H130]; Latham and Konda ku Mbuta, 2017
Trevesia palmata (Roxb. ex Lindl.) Vis.	Araliaceae	Ro	Mizoram, India	Anti-hypertensive	Rai and Lalramnghinglova, 2010
Trianthema portulacastrum L.	Aizoaceae	WP	Afar people, Rift Valley, Ethiopia	Anti-microbial [H. zoster]; anti-venom	Teklehaymanot, 2017
Tribulus cistoides L.	Zygophyllaceae	Le	Martinique	Anti-inflammatory	Longuefosse and Nossin, 1996
Tribulus terrestris L.	Zygophyllaceae	WP	Mestizo community, North Peru; Turkestan Range of south Kyrgystan	Anti-inflammatory	Bussmann and Sharon, 2006; Pawera et al., 2016
Trichanthera gigantea (Bonpl.) Nees	Acanthaceae	Le, St	Markets of Bogotá, Columbia	Anti-inflammatory [Fe]; anti-microbial [STI, UTI, skin inf]	Bussmann et al., 2018; Cadena-González et al., 2013
Trichilia heudelotti Planch. ex Oliv.	Meliaceae	Fr	Mount Cameroon, Cameroon	Anti-microbial/anti-inflammatory [rheum fev]	Sandberg et al., 2005
Trichilia prieureana Juss.	Meliaceae	Ro	Masako Forest Reserve, D.R. Congo	Anti-microbial [Urogenital inf]	Mbula et al., 2015
Trichodesma indicum (L.) Sm.	Boraginaceae	Le	Toli Peer National Park, Kashmir, Pakistan	Anti-microbial; anti-viral	Amjad et al., 2017
Trichopus zeylanicus Gaertn.	Dioscoreaceae	FL, Fr	3 Kerala tribes and Kani tribes, Western Ghats, India	Boost immunity; anti-inflammatory [asthma]	Marjana et al., 2018; Ayyanar and Ignacimuthu, 2011
Trichospermum sp.	Malvaceae	Le	Papua New Guinea	Anti-inflammatory [headache]; anti-fatigue	Jorim et al., 2012
Trichuriella monsoniae (L.f.) Bennet	Amaranthaceae	Le	Eastern Ghats, India	Anti-inflammatory [rheum]	Rao et al., 2006

Tridax procumbens (L.) L.	Asteraceae	Le	Carib population, Carib people, Livingston, Guatamala; Jamaica	Anti-inflammatory [Fe] [but inflam at high dose]	Girón et al., 1991
Trifolium repens L.	Fabaceae	FL, Le, St	Mestizo community, North Peru; Jammu + Kashmir, India	Anti-inflammatory; memory improvement	Bussmann and Sharon, 2006; Dangwal and Singh, 2013
Tripodanthus flagellaris Cham. & Schltdl.	Loranthaceae	Le, St	Comechingones, Argentina	Anti-hypertensive	Goleniowski et al., 2006
Tristemma leiocalyx Cogn.	Melastomataceae	Le	Cataractes and Lukaya districts, D.R. Congo	Anti-microbial [STI]	Latham and Konda ku Mbuta, 2017
Triticum aestivum L. Syn: *Triticum sativum* L.	Poaceae	Se	Mestizo community, North Peru	Anti-microbial	Bussmann and Sharon, 2006
Triumfetta althaeoides Lam.	Malvaceae	FL, Le, Ro, St	Loja and Zamora-Chinchipe, Ecuador	Anti-inflammatory [Fe]; Anti-microbial int inf]	Tene et al., 2007
Trixis californica Kellogg	Asteraceae	WP	Nuevo León, Mexico	Anti-inflammatory	Estrada-Castillón et al., 2012
Tropaeolum majus L.	Tropaeolaceae	AP, Le	Mato Grosso, Brazil; Potosi, Bolivia	Anti-microbial [bronch, pneum*]*; anti-inflammatory	Ribeiro et al., 2017; Fernandez et al., 2003
Tropaeolum tuberosum Ruiz & Pav.	Tropaeolaceae	NS	Markets of Bogotá, Columbia	Anti-microbial [UTI]	Bussmann et al., 2018
Tussilago farfara L.	Asteraceae	AP, Le, Rh	Mashhad markets, Iran; Pollino National Park, Southern Italy	Anti-microbial; anti-inflammatory [asthma]	Amiri and Joharchi, 2013; Di Sanzo et al., 2013
Tylophora indica (Burm.f.) Merr.	Apocynaceae	Le	Andoman + Nicobar Is., India; Eastern Ghats, India	Anti-inflammatory [Fe]	Chander et al., 2014; Rao et al., 2006
Tynanthus cognatus (Cham.) Miers	Bignoniaceae	Fr	Tacana people, Bolivian Amazon	Anti-microbial [conj]	Bourdy et al., 2000
Typha angustifolia L.	Typhaceae	Se	M'Sila, North Algeria	Wound healing	Boudjelal et al., 2013
Uncaria africana G.Don	Rubiaceae	Le	Sierra Leone	Anti-inflammatory [toothache, lung inflam]	Latham and Konda ku Mbuta, 2017
Uncaria guianensis (Aubl.) J.F.Gmel.	Rubiaceae	Ro, Vi	Mato Grosso, Brazil	Anti-inflammatory	Ribeiro et al., 2017
Uncaria sp.	Rubiaceae	Le, St	Eastern Highlands, Papua New Guinea; Tacana people, Bolivian Amazon	Anti-microbial; anti-inflammatory	Jorim et al., 2012; Bourdy et al., 2000
Uncaria sessilifructus Roxb.	Rubiaceae	Le	Mizoram, India	Anti-microbial [diphth]	Rai and Lalramnghinglova, 2010
Uncaria tomentosa (Willdenow ex Roemer & Schultes) DC.	Rubiaceae	Ba, Ro	Loja and Zamora-Chinchipe, Ecuador; Mestizo community, North Peru; Tacana people, Bolivian Amazon	Anti-inflammatory [rheum]; anti-microbial [bronch, HIV]	Tene et al., 2007; Bussmann and Sharon, 2006; Bourdy et al., 2000
Uraria picta (Jacq.) Desv. ex DC.	Fabaceae	Le, WP	Ondo State, SW Nigeria	Memory improvement; anti- AD, dementia	Mojisola et al., 2012; Kantati et al., 2016
Urena lobata L.	Malvaceae	Le, Ro	Martinique; Andoman + Nicobar Is., India; Mizoram, India	Anti-inflammatory [Fe, rheum]	Longuefosse and Nossin, 1996; Chander et al., 2014; Rai and Lalramnghinglova, 2010

Urera baccifera (L.) Gaudich. Ex Wedd.	Urticaceae	NS	Markets of Bogotá, Columbia	Anti-microbial [bronch, UTI]	Bussmann et al., 2018
Urera caracasana (Jacq.) Gaudich. ex Griseb.	Urticaceae	Le, Ro, St	Loja and Zamora-Chinchipe, Ecuador	Anti-inflammatory	Tene et al., 2007
Urochloa mutica (Forssk.) T.Q.Nguyen Syn: *Brachiaria mutica* (Forsk.) Stapf	Poaceae	Ro	Três Ladeiras people, Atlantic Forest, Brazil	Anti-inflammatory	Gazzaneo et al., 2005
Urtica dioica L.	Urticaceae	AP, FL, Le, Ro, St, WP	India, Iran, Mao Naga people Manipur, India; Svaneti and Racha-Lechkumi, Georgia; Turkestan Range of south Kyrgystan; Gheg people, NE Albania; M'Sila, North Algeria; Pollino National Park, Southern Italy; Vikos-Aoos, NW Greece	Anti-paralytic; anti-microbial [measles, mumps, UTI]; anti-hypertensive; anxiolytic; anti-inflammatory [pain, rheum]	Mikawlrawng et al., 2017; Mosaddegh et al., 2013; Lokho, 2012; Bussmann et al., 2016; Pawera et al., 2016; Pieroni and Sõukand, 2017; Boudjelal et al., 2013; Di Sanzo et al., 2013; Vokou et al., 1993
Urtica flabellata Kunth	Urticaceae	WP	Potosi, Bolivia	Anti-inflammatory [rheum]	Fernandez et al., 2003
Urtica magellanica A. Jussieu ex Poiret	Urticaceae	WP	Mapuche people, Argentina; Mestizo community, North Peru	Anti-inflammatory	Estomba et al., 2005; Bussmann and Sharon, 2006
Uvaria brevistipitata De Wild.	Annonaceae	Ro [in sp. comb]	Bas-Congo	Anti-viral [HIV]	Latham and Konda ku Mbuta, 2017
Vaccinium arctostaphylos L.	Ericaceae	Fr	Mashhad markets, Iran	Anti-hypertensive	Amiri and Joharchi, 2013
Vaccinium myrtillus L.	Ericaceae	Fr, Le	Albanians, Bosniaks/Gorani + Turkish ethnic groups, Kosovo; Gheg people, N-E Albania.	Anti-microbial [g-i inf, cough]; anti-inflammatory	Mustafa et al., 2015; Pieroni and Sõukand, 2017
Valeriana jatamansi Jones ex Roxb.	Caprifoliaceae	Ro	Central Himalayas, Nepal	Anti-microbial [ton'is, wound inf]; anti-inflammatory [headache, eye pain]	Bhattarai et al., 2006
Valeriana officinalis L.	Caprifoliaceae	NS	Markets of Bogotá, Columbia; Svaneti and Racha-Lechkumi, Georgia	Anti-microbial [mouth inf]; anxiolytic; anti-epileptic	Bussmann et al., 2016, 2018
Vangueria apiculata K.Schum.	Rubiaceae	Ro	Mabira Forest, Uganda	Anti-hypertensive	Tugume et al., 2016
Vasconcellea pubescens A. DC.	Caricaceae	NS	Markets of Bogotá, Columbia	Anti-microbial [ton'is, wart]	Bussmann et al., 2018
Vellozia sp.	Velloziaceae	Ba	Mato Grosso, Brazil	Anti-microbial	Ribeiro et al., 2017
Verbascum thapsus L.	Scrophulariaceae	FL, Le	Himachal Pradesh, NW Himalaya, India; Pollino National Park, Southern Italy	Anti-inflammatory [Fe]; anti-microbial [cold]	Vidyarthi et al., 2013; Di Sanzo et al., 2013
Verbascum virgatum Stokes	Scrophulariaceae	NS	Markets of Bogotá, Columbia	Anti-microbial [influenza]; throat inflammation	Bussmann et al., 2018
Verbena litoralis Kunth	Verbenaceae	Le, Ro, St, WP	Loja and Zamora-Chinchipe, Ecuador, Carib people, Livingston, Guatamala; Mestizo community, North Peru; native western and central Andes	Anti-microbial [int inf]; cholesterol-reducing; anti-inflammatory [Fe, rheum]; fatigue/weakness	Tene et al., 2007; Girón et al., 1991; Bussmann and Sharon, 2006; Cadena-González et al., 2013

Verbena officinalis L.	Verbenaceae	WP	Maonan people, Guangxi Zhuang, China; Loja and Zamora-Chinchipe, Ecuador	Anti-inflammatory [Fe]; anti-hypertensive; anti-microbial [int inf]	Hong et al., 2015; Tene et al., 2007
Vernonanthura ferruginea (Less.) H.Rob	Asteraceae/Compositae	FL, Fr, Le, Ro	Mato Grosso, Brazil	Anti-microbial; anti-inflammatory	Ribeiro et al., 2017
Vernonanthura patens (Kunth) H.Rob.	Asteraceae/Compositae	St sap	Tacana people, Bolivian Amazon	Anti-microbial [conj]	Bourdy et al., 2000
Vernonia adoensis Sch. Bip. ex Walp. Var.	Asteraceae	Ba, Le, Ro	Kakamega County, Kenya	Anti-microbial [eye inf]	Odongo et al., 2018
Vernonia amygdalina Delile.	Asteraceae	Le	Sagamu + Ibadan city, SW Nigeria; Trinidad	Anti-aging; anti-hypertensive; anti-microbial [STI]	Elufioye et al., 2012; Clement et al., 2015; Gbadamosi and Egunyomi, 2014
Vernonia colorata (Willd.) Drake	Asteraceae	Le with St	Benin	Memory improvement/ enhancement	Adjanohoun et al., 1989: cited in Noe and Lehmann, 2012 [H168]
Vestia foetida (Ruiz & Pav.) Hoffmanns.	Solanaceae	Le	Mapuche people, Chile	Anti-microbial [dys]	Houghton and Manby, 1985
Viburnum grandiflorum Wall. ex DC.	Adoxaceae	Se	Toli Peer National Park, Kashmir, Pakistan	Anti-microbial [typhoid, whoopingcough]	Amjad et al., 2017
Vigna peduncularis (Kunth) Fawc. & Rendle.	Leguminosae	WP	Peruvian Amazon	Anti-microbial [infected skin ulcer, leprosy]	Odonne et al., 2013
Vigna racemosa (G. Don) Hutch	Leguminosae	Le	South Nigeria	Anti-microbial [skin inf]	Borokini et al., 2012
Vigna unguiculata (L.) Walp.	Leguminosae	Se	Abyan territory, Yemen	Boost immune system	Al-Fatimi, 2019
Viguiera lanceolata Britton Syn: *Viguiera mandonii* Sch.Bip. ex Rusby	Compositae	Ro	Potosi, Bolivia	Anti-microbial [dys]	Fernandez et al., 2003
Viola arguta Humb. & Bonpl. ex Schult.	Violaceae	FL	Loja and Zamora-Chinchipe, Ecuador	Anti-inflammatory [Fe]; anti-microbial [influenza]	Tene et al., 2007
Viola canescens Wall.	Violaceae	FL, Le	Himachal Pradesh, NW Himalaya, India; Toli Peer National Park, Kashmir, Pakistan	Anti-inflammatory [Fe, headache]; anti-microbial [cough, cold]	Vidyarthi et al., 2013; Amjad et al., 2017
Viola odorata L.	Violaceae	FL, Le	Loja and Zamora-Chinchipe, Ecuador	Anti-inflammatory; anti-microbial influenza, pneum, cough]	Tene et al., 2007
Viola pilosa Blume	Violaceae	Le	Toli Peer National Park, Kashmir, Pakistan	Anti-inflammatory [Fe, pain]	Amjad et al., 2017
Virectaria major (K.Schum.) Verdc.	Rubiaceae	Le	Burundi	Eye inflammation	Baerts and Lehmann, 1989: cited in Noe and Lehmann, 2012 [H001]
Virola elongata Benth.) Warb.	Myristicaceae	Ba, Re, St	Mato Grosso, Brazil	Anti-microbial; anti-inflammatory	Ribeiro et al., 2017
Viscum coloratum (Kom.) Nakai	Santalaceae	Le	Shaanxi, China	Anti-inflammatory [rheum]; support immune system	Teng et al., 2011

Vismia japurensis Rchb.f.	Hypericaceae	Re	Mato Grosso, Brazil	Anti-microbial; wound healing	Ribeiro et al., 2017
Vitellaria paradoxa C.F.Gaertn. Syn: *Butyrospermum paradoxum* (Gaertner f.) Hepper	Sapotacea	Ju, Se	Senegal	Memory improvement/ enhancement	Kerharo and Adam, 1974: cited in Noe and Lehmann, 2012 [H168]
Vitex agnus-castus L.	Lamiaceae	Le	Três Ladeiras people, Atlantic Forest, Brazil; Edremit Gulf, Turkey	Anti-inflammatory [Fe, rheum, headache]; anti-microbial [cough]	Gazzaneo et al., 2005; Polat and Satıl, 2012
Vitex doniana Sweet	Lamiaceae	Ba, Fr, Le	Cataractes and Lukaya districts, D.R. Congo; Fundong, NW Cameroon	Anti-microbial [dys, leprosy, STI]	Latham and Konda ku Mbuta, 2017; Focho et al.,2009
Vitex madiensis Oliv.	Lamiaceae	Le	Congo-Brazzaville; Cataractes and Lukaya districts, D.R. Congo	Anti-microbial [dys]; memory improvement/ enhancement	Latham and Konda ku Mbuta, 2017; Diafouka, 1997: cited in Noe and Lehmann, 2012 [H168]
Vitex negundo L.	Lamiaceae	Le, Ro, St, WP	Maonan people, Guangxi Zhuang, China; 3 Kerala tribes, Western Ghats, India; Himachal Pradesh, NW Himalaya, India; Uttarakhand, India; West Bengal, India; Ayta communities, Bataan, Philippines; Kani people, Western Ghats, India	Anti-microbial [TB]; anti-inflammatory [Fe]; anti-epileptic; memory improvement; anti-venom	Hong et al., 2015; Marjana et al., 2018; Vidyarthi et al., 2013; Tantengco et al., 2018; Sharma et al., 2013; Datta et al., 2014; Ayyanar and Ignacimuthu, 2011
Vitex peduncularis Wall. ex Schauer	Lamiaceae	Ba	Mizoram, India	Anti-microbial [typhoid]	Rai and Lalramnghinglova, 2010
Vitex simplicifolia Oliv.	Lamiaceae	Le	Burkina Faso	Memory improvement/ enhancement	Guinko, et al., 1989: cited in Noe and Lehmann, 2012 [H168]
Vitis vinifera L.	Vitaceae	Fr, La, Le, Se, Sh, sap	Gheg people, N-E Albania; Agro Nocerino Sarnese, Southern Italy; Edremit Gulf, Turley; Golan Heights and West Bank, Israel; Catalonia, Spain; Bandipora district, Kashmir, J&K, India	Anti-inflammatory [pain]; anti-hypertensive; memory improvement; anti-microbial [cough, conj, tons, cold, influenza, wart];	Pieroni and Sõukand, 2017; Motti and Motti, 2017; Polat and Satıl, 2012; Said et al., 2002; Bonet et al., 1999; Lone et al., 2013
Voacanga africana Stapf ex Scott Elliot	Apocynaceae	Ba, Ro, Se	Cataractes and Lukaya districts, D.R. Congo; Fundong, NW Cameroon	Anti-microbial [STI, abscess, fungal inf]; anti-hypertensive; anti-poison	Latham and Konda ku Mbuta, 2017; Focho et al., 2009
Vochysia rufa Mart.	Vochysiaceae	Ba, Le	Mato Grosso, Brazil	Anti-microbial; cholesterol-reducing	Ribeiro et al., 2017
Waltheria indica L. Syn: *Waltheria americana* L.	Malvaceae	Le	South Nigeria; Guinea-Bissau	Anti-inflammatory; immune booster; anti-depressant	Borokini et al., 2012; Romeiras et al., 2012
Waltheria ovata Cav.	Malvaceae	Ro	Loja and Zamora-Chinchipe, Ecuador	Anti-inflammatory	Tene et al., 2007
Warburgia ugandensis Sprague	Canellaceae	Ba, Ro	Kakamega County, Kenya; Mabira Forest, Uganda	Anti-inflammatory [Fe]; anti-microbial [influenza, measles]	Odongo et al., 2018; Tugume et al., 2016
Whitfieldia elongata (P.Beauv.) De Wild. & T.Durand	Acanthaceae	Le	Cataractes and Lukaya districts, D.R. Congo	Anti-microbial [food poisoning]	Latham and Konda ku Mbuta, 2017

Withania somnifera (L.) Dunal	Solanaceae	Ro	Afar people, Rift Valley, Ethiopia; Tigray, Ethiopia; Chittagong Hill Tracts, Bangladesh; Punjab-Pakistan; Abyan territory, Yemen; Odisha, India	Anti-microbial [typhoid]; anti-venom; anti-inflammatory [rheum]; wound healing; anti-paralytic; poison antidote	Teklehaymanot, 2017; Kadir et al., 2015; Umair et al., 2017; Teklay et al., 2013; Al-Fatimi, 2019; Sahoo and Mahalik, 2010
Wollastonia biflora (L.) DC. Syn: *Wedelia biflora* (L.) DC.	Compositae	Le	Andoman + Nicobar Is., India	Anti-inflammatory [Fe]	Chander et al., 2014
Woodfordia fruticosa (L.) Kurtz.	Lythraceae	FL	Himachal Pradesh, NW Himalaya, India	Anti-microbial [dys]	Vidyarthi et al., 2013
Wrightia laevis Hook.f.	Apocynaceae	Le, Ro	Maonan people, Guangxi Zhuang, China	Anti-viral [mumps]	Hong et al., 2015
Xanthium spinosum L. Syn: *Acanthoxanthium spinosum* (L.) Furreau	Asteraceae	WP	Mestizo community, North Peru	Anti-inflammatory	Bussmann and Sharon, 2006
Xanthosoma sagittifolium (L.) Schott	Araceae	Le	Mato Grosso, Brazil	Anti-microbial	Ribeiro et al., 2017
Ximenia caffra Sonder	Olacaceae	Ba, Le. Ro	Venda, South Africa; Debre Libanos Wereda, central Ethiopia	Anti-microbial [STI]; anti-venom; anti-inflammatory [headache]	Arnold and Gulumian, 1984; Getaneh and, Girma, 2014
*Xylia xylocarpa (*Roxb.) W.Theob.	Leguminosae	Ba, Se	Mizoram, India	Anti-microbial [STI, leprosy]; anti-inflammatory [rheum]	Rai and Lalramnghinglova, 2010
Xylopia aethiopica (Dunal) A.Rich	Annonaceae	Fr	Mount Cameroon, Cameroon; Masako Forest Reserve, Congo; Sagamu + Ibadan city, SW Nigeria	Anti-microbial [STI]; anti-inflammatory; anti-aging	Sandberg et al., 2005; Mbula et al., 2015; Elufioye et al., 2012; Gbadamosi and Egunyomi, 2014
Xylopia hypolampra Mildbr.	Annonaceae	St ba	Baka Pygmies, Gabon	Anti-microbial [diarrh]	Betti et al., 2013
Zanthoxylum armatum DC. Syn. *Zanthoxylum alatum* Roxb.	Rutaceae	Fr	Central and West Nepal Himalayas	Anti-microbial; limb numbness; anti-inflammatory [Fe, headache]	Bhattarai et al., 2006
Zanthoxylum capense (Thumb.) Harv.	Rutaceae	Fr	South Africa	Paralysis/ hemiplegia/ polio/ paraplegia	Kling, 1923: cited in Noe and Lehmann, 2012 [H130]
Zanthoxylum deremense (Engl.) Kokwaro	Rutaceae	Fr	Kimboza forest, Tanzania	Anti-inflammatory	Amri and Kisangau, 2012
Zanthoxylum ekmanii (Urb.) Alain.	Rutaceae	Le	Peruvian Amazon	Anti-microbial/anti-inflammatory [influenza Fe]	Odonne et al., 2013
Zanthoxylum gilletii (De Wild.) P.G.Waterman Syn: *Fagara macrophylla*	Rutaceae	Ro ba	Mount Cameroon, Cameroon	Anti-microbial [STI]	Sandberg et al., 2005
Zanthoxylum rhoifolium Lam.	Rutaceae	Ba, Fr	Mato Grosso, Brazil	Anti-inflammatory	Ribeiro et al., 2017
Zanthoxylum zanthoxyloides (Lam.) Zepernick & Timler Syn: *Fagara zanthoxyloides* Lam.	Rutaceae	Ro	Benin	Memory improvement/ enhancement	Adjanohoun et al., 1989: cited in Noe and Lehmann, 2012 [H168]
Zataria multiflora Boiss.	Lamiaceae	AP	Iran	Anti-viral	Mosaddegh et al., 2013

Zea mays L.	Poaceae	Fr, FL, Le	Iran; Martinique; Sagamu + 3 states in SW Nigeria; High Atlas, Morocco	Anti-microbial [measles, UTI]; anti-inflammatory; memory enhancement; paralysis/ hemiplegia/ polio/ paraplegia	Mosaddegh et al., 2013; Bussmann and Sharon, 2006; Longuefosse and Nossin, 1996; Elufioye et al., 2012; Babawale et al., 2016; Gbile, 1990: cited in Noe and Lehmann, 2012 [H130]; Fadili *et al*, 2017
Zehneria scabra Sond.	Cucurbitaceae	Le	Haya people, Kagera, NW Tanzania	Anti-microbial [STI]	Moshi et al., 2009
Zilla spinosa (L.) Prantl	Brassicaceae	AP	Tassili N'Ajjer, Southern Algerian Sahara	Anti-inflammatory [asthma]	Hammiche and Maiza, 2006
Zingiber montanum (J.Koenig) Link ex A.Dietr. Syns: *Zingiber cassumunar* Roxb; *Zingiber purpureum* Roscoe	Zingiberaceae	Rh	Sundanese community, West Java; SW Odissa, India	Anti-inflammatory [Fe]; anti-paralytic	Roosita et al., 2008; Pattanaik et al., 2007
Zingiber officinale Roscoe	Zingiberaceae	Rh	Mato Grosso, Brazil; Carib people, Livingston, Guatamala; Trinidad; Myanmar; Maharashtra, India; Sundanese community, West Java; Tigrigna people, Central Eritrea; Tunisia; Tacana people, Bolivian Amazon; To Manui, Menui Islands, Central Sulawesi, Indonesia	Anti-microbial [cold, cough, bronch, influenza]; anti-inflammatory [headache, pain, rheum]; memory improvement/enhancement; wound healing; anti-paralytic	Ribeiro et al., 2017; Girón et al., 1991; Clement et al., 2015; DeFilipps and Krupnick, 2018; Wadankar et al., 2011; Roosita et al., 2008; Yemane et al., 2017; Boulos, 1983: cited in Noe and Lehmann, 2012 [H168]; Bourdy et al., 2000; Rahmawati et al., 2020
Zingiber ottensii Valeton	Zingiberaceae	Rh	Orang Asli tribe, Malaysia	Anti-bacterial	Samuel et al., 2010
Zingiber zerumbet (L.) *Roscoe ex Sm.*	Zingiberaceae	Rh	Myanmar	Anti-microbial [bronchitis, cough, leprosy]	DeFilipps and Krupnick, 2018
Ziziphora clinopodioides Lam.	Lamiaceae	Le	Iran	Anti-viral [cold]	Mosaddegh et al., 2013
Ziziphora pamiroalaica Juz.	Lamiaceae	AP	Turkestan Range of south Kyrgystan	Anti-microbial [cold, cough, influenza]; anti-inflammatory [headache]	Pawera et al., 2016
Ziziphora taurica M.Bieb.	Lamiaceae	AP	Edremit Gulf, Turkey	Anti-microbial [cold, influenza]	Polat and Satıl, 2012
Ziziphus jujuba Mill.	Rhamnaceae	Fr	Iran; Edremit Gulf, Turkey	Anti-viral [cold]; anti-inflammatory [asthma]	Mosaddegh et al., 2013; Polat and Satıl, 2012
Ziziphus lotus (L.) Lam	Rhamnaceae	Fr, Le	M'Sila, North Algeria; Tassili N'Ajjer, Southern Algerian Sahara	Wound healing; anti-inflammatory [Fe]	Boudjelal et al., 2013; Hammiche and Maiza, 2006
Ziziphus mucronata Willd.	Rhamnaceae	Fr, Le	Benin	Memory improvement/ enhancement	Adjanohoun et al., 1989: cited in Noe and Lehmann, 2012 [H168]
Ziziphus nummularia (Burm.f.) Wight & Arn.	Rhamnaceae	Ba, Fr	Iran; Toli Peer National Park, Kashmir, Pakistan	Anti-hypertensive; anti-inflammatory [Fe]; anti-microbial [diarrh, dys, RTI]	Mosaddegh et al., 2013; Amjad et al., 2017
Ziziphus oenoplia (L.) Mill.	Rhamnaceae	Ro	Karnataka, India	mouth ulcer	Rajakumar and Shivanna, 2009

Ziziphus spina-christi (L.) Desf.	Rhamnaceae	Br, Fr, Le	Iran; Jazan province, Saudi Arabia; Abyan territory, Yemen	Anti-microbial skin inf, measles, rabies]; anti-inflammatory [Fe, pain]; weakness	Mosaddegh et al., 2013; Tounekti et al., 2019; Al-Fatimi, 2019
Ziziphus mauritiana Lam.	Rhamnaceae	Le	Martinique	Anti-microbial	Longuefosse and Nossin, 1996
Zygophyllum album L.f.	Zygophyllaceae	AP	Tassili N'Ajjer, Southern Algerian Sahara	Anti-inflammatory [rheum]	Hammiche and Maiza, 2006

Abbreviations
Plant parts: Ae bu, aerial bulbils; AP, aerial parts; Ba, bark; Bd, bud; Br, branch; Bu, bulb; Ca, capitulum; Cx, calyx; EO, essential oil; FL, flower; Fr, Fruit; Inflor, inflorescence; Ju, juice; La, latex; Le, leaf; Me, meristem; Ne, nectar; NS, not specified; Phy, phyllodes; PL, peel; Rc, receptacle; Re, resin; Rh, rhizome; Ro, root; Se, seed; Sh, shoot; SP, spine; Spr, sprout; St, stem; St ba, stem bark; Tu, tuber; Tw, Twig; Vap, vapour; Vi, vine; Wo, wood; WP, whole plant.

Symptoms/diseases: bronch, bronchitis**;** conj, conjunctivitis; CoV, coronavirus; c'pox, chickenpox; diarrh, diarrhoea**;** diphth, diphtheria; dis, disease; dys, dysentery; ext, external; gangr, gangrene; g- i inf, gastro-intestinal tract infection; inf, infection; inflam, inflammation; int, internal; O inf, oral infection; PD, Parkinson's disease; pneum, pneumonia; rheum, rheumatism; rheum fev, rheumatic fever; URTI, upper respiratory tract infection; s'pox, smallpox; STI, sexually transmitted infection; ton'is, tonsilitis; TBI, traumatic brain injury; UTI, urinary tract infection; yell fev, yellow fever.

For references: see S8 Table References File
