# Supplementary material for: Therapeutic roles of plants for 15 hypothesised causal bases of Alzheimer’s disease
Source: Nat Prod Bioprospect. 2022 Aug 23;12(1):34. doi: 10.1007/s13659-022-00354-z (PMC9395556; doi:10.1007/s13659-022-00354-z)
Supplement: Supplementary file 3 — Additional file 3. Table S3. Bioactivities listed by species. [file 13659_2022_354_MOESM3_ESM.pdf]

Additional Table S3. Bioactivities listed by species. List of species with confirmed bioactivities of therapeutic relevance to neurodegeneration, particularly for Alzheimer's disease [abbreviations at end]

**Species**	**Confirmed Bioactivity [reference] [model]**
Aaronsohnia pubescens (Desf.) K.Bremer & Humphries Syn: *Matricaria pubescens* (Desf.) Sch.Bip.	Anti-inflammatory [Metrouh-Amir and Amir, 2018] a/vivo mouse; anti-bacterial, anti-oxidant [Metrouh-Amir et al., 2015] [vit]
Abies pindrow (Royle ex D.Don) Royle	Anti-oxidant [Gupta D et al., 2011] [vit]; anxiolytic [Kumar V et al., 2000 [a/vivo rat]; anti-inflammatory [Singh RK and Pandey, 1997] [a/vivo rat]; anti-bacterial, anti-fungal [Ali M et al., 2020] [vit]
Abuta grandifolia (Mart.) Sandw.	Anti-microbial [Kloucek et al., 2007] [vit]
Abutilon hirsutum G. Don.	No records found
Abutilon indicum Sweet var. *welwitschii* E. G. Baker	Anti-inflammatory [Tripathi et al., 2012] [a/vivo rat]; mildly anti-microbial [Abdul et al., 2010]; anti-venom [Shrikanth et al., 2014] [vit]
Abutilon pannosum (G.Forst.) Schltdl. Syn: *Abutilon figarianum* Webb	Anti-bacterial [Aadesariya et al., 2017] [vit]
Acacia ehrenbergiana Hayne	Anti-inflammatory [Rizk et al., 1985] [a/vivo mouse]
Acacia etbaïca (Schweinf.) Kyal. & Boatwr.	No records found<br>*Acacia hydaspica, Acacia salicina* anti-oxidant, anxiolytic [Afsar et al., 2017] [a/vivo rat]; *Acacia catechu* [in sp. comb.] enhanced cognitive performance + memory improvement [Yimam et al., 2016] [h/c, a/vivo rat]
Acacia farnesiana (L.) Willd. Syn: *Vachellia farnesiana* (L.) Wight & Arn.	Anti-bacterial [Sánchez et al., 2013] [vit]; anti-inflammatory [Gabr et al., 2018] [vit]
Acacia karroo Hayne	Anti-inflammatory [Adedapo et al., 2008] [a/vivo rat]
Acacia mellifera (M.Vahl) Benth.	Anti-viral [HBV] [Arbab et al., 2015] [h/cell line], [HIV] [Rukunga et al., 2002] [vit]; anti-bacterial [Mutai et al., 2009] [vit]; anti-inflammatory [Veronica et al., 2017] [a/vivo mouse, rat]
Acacia modesta Wall.	Anti-inflammatory, anti-platelet aggregation [Bukhari et al., 2010] [a/vivo rat]; anti-bacterial [Khalid et al., 2011] [vit]
Acacia nilotica (L.) Delile	Anti-platelet aggregation [Shah BH et al., 1997] [h/cell platelet]; anti-bacterial [Banso, 2009] [vit]; immunomodulation [Koko et al., 2008] [vit]
Acacia oerfota (Forssk.) Schweinf.	Anti-viral [HBV] [Arbab et a., 2017] [vit]
Acacia senegal (L.) Willd.	Anti-bacterial [Okoro et al., 2012] [vit]
Acacia seyal Delile Syn: *Acacia tortilis* (Forssk.) Hayne	Anti-inflammatory, wound healing [Eldeen and Van Staden, 2008] [vit]
Acaena argentea Ruiz & Pav.	Anti-bacterial [Mølgaard et al., 2011] [vit]
Acaena splendens Hook. & Arn.	Anti-inflammatory [Backhouse et al., 2002] [a/vivo guinea pig, rabbit]
Acalypha fruticosa Forssk.	Anti-bacterial [Alasbahi et al., 1999] [vit]
Acalypha indica L.	Anti-inflammatory [Rahman MA et al., 2010] [a/vivo rat]; anti-fungal [Solomon et al., 2005] [vit]; anti-neurotoxic vs. snake venom [Shirwaikar et al., 2004] [a/isolated frog tissue]
Acalypha wilkesiana Mull. Arg.	Anti-hypertensive [Ikewuchi et al., 2011] [a/vivo rat]; anti-bacterial [Akinyemi et al., 2005] [vit]
Acantholimon spp.	No records found
Acanthospermum hispidum DC.	AChE inhibition [Elufioye and Machie, 2016] [vit]; anti-bacterial [Fleischer et al., 2003] vit]
Acanthus eminens C. B. Clarke	No records found
Acanthus pubescens (Oliv.) Engl.	Anti-viral [measles, polio] [Vlietinck et al., 1995] [vit, a/cell line monkey]
Acer monspessulanum subsp. *cinerascens* (Boiss.) Yalt. Syn: *Acer cinerascens* L.	Anti-oxidant [Ceylan et al., 2016] [vit]

Achillea asiatica Serg.	Wound healing, anti-inflammatory [Dorjsembe et al., 2017] [a/vivo rat, a/cell line mouse]
Achillea millefolium L.	Anti-bacterial [Kokoska et al., 2002] [vit]
Achillea santolinoides subsp. *wilhelmsii* (K.Koch) Greuter. Syn: *Achillea wilhelmsii*	Anti-hypertensive, anti-hyperlipidemic [Asgary et al., 2000] [h/c]
Achyranthes aspera L.	Anti-inflammatory [Vijaya Kumar et al., 2009] [a/vivo rat]; anti-epileptic [Viswanatha et al., 2017] [a/vivo mouse]; anti-bacterial, anti-fungal [Ndhlala et al., 2015] [vit]; neuroprotective, anti-oxidant [Viswanatha et al., 2019] [a/vivo rat, vit]; wound healing [Edwin et al., 2009] [a/vivo rat]
Achyranthes bidentata Blume	Anti-inflammatory [Vetrichelvan and Jegadeesan, 2002] [a/vivo rat]
Achyrocline bogotensis (Kunth) DC	Anti-viral [rotavirus, astrovirus] [Téllez et al., 2015] [vit]
Acmella caulirhiza Delile	Anti-bacterial [Sinei et al., 2013] [vit]
Acmella oleracea Syn: *Spilanthes acmella var. oleracea* (L.) C.B.Clarke	Anti-bacterial [de Alcantara et al., 2015] [vit]; immunostimulatory [Rajesh et al., 2011] [a/vivo rat, a/cell line rat]; anti-inflammatory [Matu and Staden, 2003] [vit]
Acmella paniculata (Wall. ex DC.) R.K.Jansen Syn: *Spilanthes paniculata*	Anti-bacterial [Mamidala and Gujjeti, 2013] [vit]
Acokanthera schimperi (A.DC.) Schweinf.	Anti-bacterial [Taye et al., 2011] [vit]
Acorus gramineus Aiton	Anti-fungal [Lee JY et al.,2004] [vit]; neuroprotection, BDNF upregulation [Gao N et al., 2019] [a/vivo mouse]; improved cognitive function [Chen Y et al., 2019] [a/vivo rat]
Acourtia microcephala DC. Syn: *Perezia microcephala* (DC.) A.Gray	No records found
Acridocarpus orientalis A.Juss	[morin] anti-amyloidogenic [Noor et al., 2012] [vit]; anti- tauopathic [Gong et al., 2011] [h/cell line neuron, a/vivo mouse, vit]; reduced oxidative stress + inflammation, enhanced neurotrophic support [Ola et al., 2014] [a/vivo rat]<br>*Acridocarpus orientalis* as source of morin: Hussain et al., 2014
Acridocarpus smeathmannii Engl.	No records found
Acriopsis sp.	No records found
Acrocomia aculeata (Jacq.) Lodd. ex R. Keith	Anti-inflammatory [Lescano et al., 2015] [a/vivo rat]
Actaea cimicifuga L. Syn: *Cimicifuga foetida* L.	No records found
Actiniopteris radiata (Sw.) Link	Anti-inflammatory [Vadnere et al., 2013] [a/vivo rat, mouse]
Adansonia digitata L. Syn: *Adansonia sphaerocarpa*	Anti-inflammatory [Ayele et al., 2013] [a/cell line mouse]; anti-microbial [Masola et al., 2009] [vit]; ROS scavenging [Talari et al., 2017]; [vit] anti-depressant, increased BDNF [Shehu et al., 2019] [a/vivo mouse]; energy source [Osman, 2004] [vit]
Adenocarpus bacquei Batt. & Pit.	No records found
Adenostoma sparsifolium Torr.	No records found
Adesmia boronioides Hook.f.	Anti-inflammatory [González et al., 2003] [a/cell line rat]
Adiantum poiretii Wikstr.	No records found<br>*Adiantum capillus veneris* anti-inflammatory [Haider S et al., 2011b] [a/vivo rat]; *Adiantum venustum* anti-bacterial, anti-fungal [Singh M et al., 2008] [vit]
Aegle marmelos (L.) Corr.	Anti-inflammatory [Arul et al., 2005] [a/vivo rat]; anti-bacterial [Pitre and Srivastava, 1988] [vit]
Aerva javanica (Burm.f.) Juss. ex Schult.	Anti-bacterial [Mufti et al., 2012] [vit]; neuroprotective [Roth et al., 1999] [h/cell line, a/cell line mouse neuron]
Aerva lanata (L.) Juss. ex Schult.	Anti-viral (HIV) [Gujjeti and Mamidala, 2017]; anti-inflammatory [Sharma A et al., 2011] [a/vivo rat]; anti-hyperlipidemic [Soundararajan et al., 2007] [a/vivo rat]

Aerva sanguinolenta (L.) Blume	Anti-inflammatory [Mandal et al., 2015] [a/vivo rat, mouse]
Aeschynanthus parasiticus (Roxb.) Wall.	No records found
Aeschynanthus sikkimensis (C.B. Clarke) Stapf.	No records found
Aesculus chinesis Bunge	Anti-viral [influenza, RSV] [Wei F et al., 2004] [vit]
Aesculus hippocastanum L.	Anti-inflammatory [Matsuda et al., 1997] [a/vivo rat, mouse]
Aframomum alboviolaceum (Ridl.) K.Schum.	No records found
Aframomum angustifolium (Sonn.) K.Schum.	Anti-bacterial [Anywar and Kirimuhuzya, 2015] [vit]
Aframomum melegueta (Roscoe) K.Schum.	Anti-viral [SARS-CoV-2] [Omotuyi et al., 2021] [vit]; anti-oxidant [Onoja et sl., 2014] [a/vivo rat, vit]; anti-inflammatory [Ilic et al., 2014] [a/vivo rat]; anti-bacterial [Alo et al., 2012] [vit]; reduced memory impairment [Ishola et al., 2016] [a/vivo mouse]
Afrostyrax lepidophyllum Mildbr.	Anti-oxidant [Ene-Obong et al., 2018] [vit]
Agapetes mannii Hemsl.	No records found
Agarista salicifolia (Lam.) G.Don Syn: *Agauria salicifolia* (Lam.) Oliv.	No records found
Agave americana L.	Anti-inflammatory [Monterrosas-Brisson et al., 2013] [a/vivo mouse]
Agelaea pentagyna (Lam.) Baill.	No records found
Aglaia odorata Lour	Anti-inflammatory [Yodsaoue et al., 2012] [a/mouse cell line]
Agrimonia eupatoria L.	Anti-inflammatory, improved lipid profile [Ivanova et al., 2013] [h/c]; anti-inflammatory, anti-oxidant [Santos TN et al., 2017] [a/vivo rat, mouse]
Ajuga austroiranica Rech.f.	No records found
Ajuga integrifolia Buch.-Ham. ex D.Don Syn: *Ajuga bracteosa*	Anti-inflammatory [Gautam et al., 2011] [a/vivo mouse]
Albizia adianthifolia (Schumach) W. F. Wight	Anti-bacterial, anti-fungal [Tamokou et al., 2012] [vit]; anti-inflammatory [Eldeen et al., 2005] [vit]; memory improvement [Beppe et al., 2014] [a/vivo PD rat]
Albizia anthelmintica (A. Rich.) Brongn. R	Anti-bacterial [Kareru et al., 2008] [vit]
Albizia grandibracreata Taub.	No records found
Albizia lebbeck (L.) Benth	Anti-inflammatory [Babu et al., 2009] [a/vivo rat]; anti-bacterial [Bobby and Wesely, 2012] [vit]; anxiolytic [Une et al., 2001] [a/vivo mouse]; anti-venom [Amog et al., 2016] [a/vivo mouse]; improved memory + cognitive impairment, AChE inhibition, neuroprotective [Saleem U et al., 2019] [a/vivo rat
Albizia versicolor Welw. ex Oliver	No records found
Albizia zygia (DC.) J.F.Macbr.	Anti-bacterial, anti-fungal, anti-oxidant [Oloyede et al., 2013] [vit]; anti-inflammatory [Abere et al., 2014] [a/vivo mouse]; anti-psychotic [Amoateng et al., 2017] [a/vivo mouse]
Alcea calvertii (Boiss.) Boiss.	No records found
Alcea rosea (L.) Cavanilles	Anti-bacterial [Seyyednejad et al., 2010] [vit]
Alchornea cordifolia (Schumach. & Thonn.) Müll.Arg.	Anti-bacterial [Ebi, 2001] [vit]
Alchornea laxiflora (Benth.) Pax & K. Hoeffm	Anti-inflammatory, anti-bacterial, anti-fungal [Ogundipe et al., 1998] [a/vivo rat, vit]; anti-venom [Molander et al., 2014] [vit]
Alibertia patinoi (Cuatrec.) Delprete & C.H. Perss.	No records found
Allagoptera campestris (Mart.) Kuntze	No records found
Allamanda cathartica L.	Anti-bacterial [Rajamanickam and Sudha, 2013] [vit]; anti-inflammatory [Hema, 2014] [vit]
Allanblackia gabonensis (Pellegr.) Bamps Syn:	Anti-bacterial [Fankam et al., 2015; Ajibesin et al., 2007] [vit]; anti-inflammatory [Ymele et al., 2013] [a/vivo mouse]

Allanblackia floribunda var.*gabonensis* Pellegr	
Allium ascalonium L.	Anti-fungal, anti-bacterial [Amin and Kapadnis, 2005] [vit]; *Allium* sp. Hsp70 modulation [Liu SG et al., 2015] [a/vivo rat]
Allium carolinianum DC.	No records found
Allium cepa L.	Anti-inflammatory [Dorsch et al., 1990] [h/cell line]; anti-bacterial [Zohri et al., 1995] [vit]; anti-viral [HSV] [Romeilah et al., 2010] [a/cell line monkey]; reduced oxidative stress [Prakash D et al., 2007] [vit]; anti-tauopathic; inhibits ER stress [Chen J et al., 2016] [h/cell line neuron] ;anti-venom [Asad et al., 2013] [vit]; memory improvement [Nakagawa et al., 2016] [h/c, a/vivo AD mouse]; anti- α-synucleinopathic [Caruana et al., 2011] [vit]; anti-hypertensive [Sakai et al., 2003] [a/vivo rat], [Brüll et al., 2015] [h/c]; anti-glaucoma [Miyamoto et al., 2011; Miyamoto and Kohno 2019] [h/cell trabecular meshwork]; wound healing [Tsala et al., 2005] [a/vivo mouse]; reduced cerebral injury, attenuated impairment in memory and motor coordination [Shri and Bora, 2008] [a/vivo mouse]; AChE inhibition [Park et al., 2015] [a/vivo mouse]
Allium fistulosum L.	Anti-oxidant [Štajner et al., 1998] [vit]; anti-inflammatory [Wang BS et al., 2013] [a/cell line mouse]; anti-viral [Lee JB et al., 2012] [a/vivo mouse]
Allium jesdianum Boiss. & Buhse	Anti- platelet aggregation [Lorigooini et al., 2015] [h/cell platelets]
Allium oreoprasum Schrenk	Anti-viral [influenza] [Rajbhandari et al., 2009] [a/cell line monkey]
Allium rubellum M.Bieb.	Anti-oxidant [Motamed and Naghibi, 2010] [vit]; anti-microbial [Bazzaz and Haririzadeh, 2003] [vit]
Allium sativum L.	Anti-hypertensive [Ried et al., 2010] [h/c]; anti-viral [Weber et al., 1992] [vit]; anti-inflammatory [Rabe et al., 2015] [a/cell line mouse]; anti-venom [Asad et al., 2013] [vit]; anti-amyloidogenic [Chauhan, 2003] [a/vivo mouse]; memory improvement [Semuyaba et al., 2017] [a/vivo rat]; reduced oxidative stress + Hsp modulation [Liu SG et al., 2015] [a/cell rat neuron]; anti-convulsant [Advani et al., 2011] [a/vivo mouse]; immunomodulation [IL-17 inhibition] [Moutia et al., 2016] [h/cell line]; anti-aging, increased lifespan and stress resistance [Ogawa T et al., 2016] [a/vivo *C. elegans*]
Allium schoenoprosum L.	Anti-inflammatory, reduced nitro-oxidative stress [Parvu et al., 2014] [a/vivo rat, vit]; anti-bacterial [Mnayer et al., 2014] [vit]
Alocasia macrorrhizos (L.) G.Don	Anti-microbial, thrombolytic [Banik et al., 2014] [h/blood cells, vit]
Aloe buettneri A.Berger Syn: *A. congolensis*	Anti-inflammatory, anti-ulcer, wound healing [Metowogo et al., 2008] [a/vivo rat]
Aloe harlana Reynolds	Anti-bacterial, anti-fungal, anti-oxidant [Asamenew et al., 2011] [vit]
Aloe rubroviolacea Schweinf.	No records found
Aloe sp.	No records found
Aloe littoralis Baker	Anti-inflammatory [Hajhashemi et al., 2012] [a/vivo rat]
Aloe trichosantha A.Berger	Anti-bacterial [Oumer et al., 2014] [vit]
Aloe vacillans Forssk	No records found
Aloe vera (L.) Burm.f. Syn: *Aloe barbedensis* Mill.	Anti-bacterial [Lorenzetti et al., 1964; Habeeb et al., 2007; Fani and Kohanteb] [vit]; anti-viral [influenza] [Choi JG et al., 2019] [a/cell line canine]; wound healing [Chithra et al., 1998] [a/vivo rat], Wahedi et al., 2017 [h/cell line, a/cell line]; anti-inflammatory [Reddy et al., 2012] [h/c]; raised BDNF [Malayeri et al., 2021] [h/c]; bone regeneration [Boonyagul et al., 2014] [a/vivo rat, a/cell line rat]; growth factor proliferation [Jettanacheawchankit et al., 2009] [a/vivo rat]; improved lipid profile, anti-atherogenic [Gupta A et al., 2013] [a/vivo rabbit]; reduced metal toxicity [Jakkala and Ali, 2015] [a/vivo rat]
Aloysia citriodora Paláu Syn: *Aloysia triphylla* (L'Hér.) Britton	Anti-inflammatory [Ponce-Monter et al., 2010] [vit, a/vivo]; anti-bacterial, anti-fungal [Oliva et al., 2010] [vit]

Aloysia virgata (Ruiz & Pav.) Juss	Anti-bacterial [Montanari et al., 2011] [vit]
Alpinia calcarata Rosc.	Anti-inflammatory [Arawwawalaet al., 2012] [a/vivo rat]
Alpinia galanga (L.) Willd.	Anti-inflammatory [Ghosh AK et al., 2011] [a/vivo rat]; anti-bacterial [Oonmetta-aree et al., 2006]; anti-viral [HIV] [Ye Y et al., 2006] [h/cell lines]; anti-fungal [Haraguchi et al., 1996] [vit]; T-bet inflammatory immune modulation [Min HJ et al., 2009] [a/vivo mouse]; reduced IFNγ [Yu et al., 2009] [a/cell line mouse CD4+ Th cells]; memory improvement, neuroprotective, anti-oxidant [Hanish Singh et al, 2011] [a/vivo mouse] *Alpinia katsumadai* BDNF modulation [Li H et al., 2011a] [a/vivo gerbil]
Alpinia purpurata K. Schum	Anti-bacterial [Santos GK et al., 2012] [vit]
Alpinia zerumbet (Pers.) B.L.Burtt & R.M.Sm.	Anti-viral [HIV, influenza] [Upadhyay et al., 2011] [vit]; anti-hypertensive and vasodilatory; anti-dyslipidemic [de Moura et al., 2005] [a/vivo rat]
Alstonia boonei De Wild.	Anti-inflammatory [Olajide et al., 2000] [a/vivo rat, mouse]
Alstonia macrophylla Wall. ex G.Don	Anti-inflammatory [Arunachalam et al., 2002] [a/vivo rat]
Alstonia scholaris (L.) R. Br.	Anti-viral [HSV, ADV] [Zhang L et al., 2014; Zhao YL et al., 2021] [vit]; anti-bacterial [Khan et al., 2003] [vit]; anti-convulsant [Quazi, 2015] [a/vivo mouse]; anti-inflammatory [Shang et al., 2010] [a/vivo mouse]; immunostimulatory [Iwo et al., 2000] [a/vivo mouse]; anti-hypertensive [Bhogayata et al., 2009] [h/c]; anti-venom [Ghosh et al., 2018] [a/vivo mouse]; memory improvement, anxiolytic [Kulkarni and Juvekar., 2009] [a/vivo mouse]; reduced lipogenesis [Sun et al., 2022] [a/vivo mouse]
Alstonia spatulata Blume	No records found
Alternanthera brasiliana (L.) Kuntze	Anti-inflammatory [Handique et al., 2017] [a/vivo rat]; anti-viral [HSV] [Lagrota et al, 1994] [h/cell line]
Alternanthera halmifolia (Lam.) Standl. ex Pittier	No records found
Alternanthera lanceolata (Benth.) Schinz	No records found
Alternanthera porrigens var. *piurensis* (Standl.) Eliasson	No records found
Alternanthera ramosissima (Mart.) Chodat & Hassl.	Anti-inflammatory [Dej-Adisai et al., 2018] [a/mouse cell line]
Alternanthera sessilis (L.) R.Br. ex DC.	Anti-inflammatory [Subhashini T et al., 2010] [a/vivo rat]
Althaea officinalis L.	Anti-inflammatory, anti-platelet aggregation, anti-ulcer [Hage-Sleiman et al., 2011] [a/vivo rat]; anti-oxidant [Elmastas et al., 2004] [vit]
Amaranthus hybridus L.	Anti-bacterial [Dahiya et al., 2010] [vit]; anti-inflammatory [Schröter et al., 2019] [a/cell line mouse]
Amaranthus viridis L. Syn: *Amaranthus gracilis* Desf.	Anti-bacterial [Ahmed SA et al., 2013] [vit]; wound healing [Sahoo et al., 2015] [a/vivo rat]
Amaranthus graecizans L.	Anti-inflammatory [Ishtiaq et al., 2017] [a/vivo rat]
Ambrosia peruviana Willd. Syn: *Ambrosia cumanensis* Kunth	Anti-microbial [Bussmann et al., 2009] [vit]
Ambrosia psilostachya DC.	Anti-inflammatory [Lastra et al., 2004] [a/cell line mouse]
Ammannia baccifera L.	Anti-inflammatory [Loganayaki et al., 2012] [a/vivo rat, mouse]
Amyema sp.	Anti-bacterial [Palombo and Semple, 2001] [vit]
Amicia glandulosa Kunth	No records found
Ampelodesmos mauritanicus (Poir.) T.Durand & Schinz	No records found
Amygdalus bucharica Korsh.	No records found
Anacardium giganteum Hancock ex Engl.	No records found
Anacardium humile A.St.-Hil.	Anti-bacterial [Ferreira PR et al, 2012] [vit]
Anacardium occidentale L.	Anti-bacterial [Himejima and Kubo, 1991] [vit]; anti-inflammatory [Olajide et al., 2004] [a/vivo];

	[Agathisflavone] anti-neuroinflammatory, reduced neurotoxicity [Velagapudi et al., 2018] [a/cell mouse microglia]
Anacardium spruceanum Benth. ex Engl.	No records found
Anacyclus pyrethrum (L.) Lag.	Improved memory and learning [Sujith et al., 2012] [a/vivo rat]; immunostimulatory [Bendjeddou et al., 2003] [a/vivo mouse]
Ananas comosus (L.) Merr.	Anti-bacterial [Ali et al., 2015] [vit]; anti-inflammatory [Akhtar et al., 2004] [h/c]; Ordesi et al., 2014] [h/c]; anti-hyperlipidemic [Xie et al., 2014] [a/vivo mouse]; cognitive enhancement [Momtazi-Borojeni et al., 2017] [mouse]; anti-inflammatory [Kargutkar and Brijesh, 2018] [a/vivo rat]
Anastatica hierochuntica L.	Anti-inflammatory [Alatshan et al., 2018] [a/vivo rat]; immunomodulatory [Abdulfattah, 2013] [a/vivo mouse]
Anchietea pyrifolia (Mart.) G.Don	No records found
Anchusa azurea Mill	Anti-inflammatory [Kuruuzum-Uz et al., 2012] [a/vivo rat]
Anchusa italica Mill.	Anti-viral [influenza] [Ketabchi et al., 2011] [a/cell line canine]
Andira cujabensis Benth.	No records found
Andira inermis (W.Wright) DC. Syn: *Geoffroea inermis* (Wright) Wright	No records found
Andrographis paniculata (Burm.f.) Nees	Anti-bacterial [Singha et al., 2003] [vit]; anti-inflammatory [Sandborn et al., 2010] [h/c]; anti-hypertensive [Yoopan et al., 2007] [a/vivo rat, isolated vessels rat]; anti-apoptotic [Lee MJ et al., 2010b] [a/cell line mouse]
Anemarrhena asphodeloides Bunge	Anti-inflammatory [Kim et al., 2009] [a/cell line mouse]; anti-fungal, anti-bacterial [Iida et al., 1999] [vit]
Anemone multifida Poir.	No records found
Anemopsis californica (Nutt.) Hook. & Arn.	Anti-microbial
Anethum graveolens L.	Anti-bacterial [Kaur and Arora, 2009] [vit]; anti-viral [Orhan et al., 2012] [a/cell line monkey]; anti-hyperlipidemic [Hajhashemi et al., 2008] [a/vivo rat]; anti-inflammatory, anti-oxidant [Kazemi, 2015] [vit]; memory improvement [Mesripour et al., 2016] [a/vivo mouse]
Angiopteris sp.	Anti-inflammatory [Lamichhane et al., 2020] [a/cell line mouse]
Angraecum eichlerianum Kraenzl.	No records found
*Aniba canellila (*Kunth) Mez	Anti-hypertensive [Lahlou et al., 2005] [a/vivo rat]
Anisochilus carnosus (L.f.) Wall.	Anti-oxidant [Bhagat et al., 2011] [vit]
Anisomeles indica (L.) Kuntze Syns: *Epimeredi indica; Nepeta indica*	Anti-bacterial [Rao et al., 2012] [vit, h/ cell line]
Anisotes trisulcus (Forssk.) Nees	Anti-inflammatory, anti-oxidant [El-Shanawany et al., 2012] [a/vivo rat]
Annickia chlorantha (Oliv.) Setten & Maas Syn: *Enantia chlorantha* Oliv.	Anti-viral [yell fev] [Fasola et al., 2011] [a/cell line monkey]
Annona coriacea Mart.	AChE inhibition [Formagio et al., 2015] [vit]
Annona montana Macfad. Syn*:* *Annona marcgravii* Mart.	Anti-inflammatory [Chuang et al., 2008] [a/cell line mouse]
Annona muricata L.	Anti-hypertensive [Adefegha et al., 2015] [vit]; anti-inflammatory; anti-bacterial [Pinto et al., 2017] [vit]; Hsp70 upregulation, anti-oxidant, wound healing [Moghadamtousi et al., 2014, 2015] [a/vivo rat]; anxiolytic [Okoronkwo et al., 2018] [a/vivo rat]
Annona senegalensis Pers. Syns: *Annona arenaria* Thonn., *Annona chrysophylla* Bojer	Anti-venom [Molander et al., 2014] [vit]; anti-inflammatory [Yeo et al., 2011] [a/vivo rat]
Annona squamosa L.	Anti-inflammatory [Yeh et al., 2004] [h/cell line]
Annonidium mannii (Oliv.) Engl. & Diels	No records found
Anoda cristata (L.) Schl.	Anti-oxidant [Juárez-Reyes et al., 2015] [vit]

Anredera cordifolia Tenore	Anti-inflammatory [Laksmitawati et al., 2017] [a/cell line mouse]; anti-bacterial [Tshikalange et al., 2005] [vit]
Antherotoma senegambiensis (Guill. & Perr.) Jacq.-Fél. Syn: *Dissotis senegambiensis* (Guill. & Perr.) Triana	Anti-oxidant, anti-microbial [Nzogong et al., 2018] [vit]
Anthocleista djalonensis A.Chev.	Anti-epileptic [Taiwe et al., 2017]; anti-inflammatory [Shorinwa et al., 2015] [a/vivo rat]
Anthocleista madagascariensis Baker	No records found
Anthurium sp.	*Anthurium andraeanum* anti-bacterial, anti-fungal [Shazhni et al., 2016] [vit; *[Anthurium cerrocampanense* anti-inflammatory [Segura et al., 1998] [a/vivo mouse, rat]
Antidesma membranaceum Müll.Arg.	No records found<br>*Antidesma venosum* anti-bacterial [Shengo et al., 2013] [vit]
Aphelandra cirsioides Lindau	No records found
Aphelandra pilosa Leonard	No records found
Aphelandra tonduzii Leonard.	No records found
Apium graveolens L.	[Anti-inflammatory [Mencherini et al., 2007] [a/vivo mouse]; improved learning and memory, anti-hypertensive [Samaha et al. et al., 2007] [a/vivo mouse, a/cell line mouse]. [DL-3-n-butylphthalide] reduced microglial activation, improved motor performance, extended survival [Feng X et al., 2012] [a/vivo ALS mouse]; anti-PD [Zhou H et al., 2019] [h/c]; improved mitochondrial function [Xiong N et al., 2012] [a/vivo rat]; reduced amyloid β [Peng et al., 2010] [a/vivo mouse]; anti-tau [Peng Y et al., 2012] [a/vivo mouse, h/cell line; endothelial progenitor cell mobilization [Zhao H et al., 2016] [h/c]; anti-depressant [Yang M et al., 2018] [a/vivo rat]
Apodanthera smilacifolia Cogn.	No records found
Aquilaria crassna Pierre ex Lecomte	Anti-inflammatory [Wongwad et al., 2019] [h/cell line]
Aquilaria malaccensis Lam.	Anti-bacterial, anti-oxidant [Hendra et al., 2016] [vit]
Araucaria araucana (Mol.) Koch (ind)	Anti-bacterial, anti-fungal [Céspedes et al., 2006] [vit]
Arctium lappa L.	Anti-inflammatory [Zhang WZ et al., 2015] [a/vivo mouse]; improved lipid profile, ameliorates endothelial dysfunction [Lee YJ et al., 2012] [a/vivo rat]; anti-bacterial [Kokoska et al., 2002] [vit]; anti-fatigue [Chen WC et al., 2017] [a/vivo mouse]
Ardisia crenata Sims	Antithrombin activity [Chistokhodova et al., 2002] [vit]; vasorelaxant [Zaima et al., 2013] [a/ isolated aortic artery rat]
Ardisia gigantifolia Stapf	Anti-inflammatory [Weibo et al., 2018] [a/vivo mouse]
Areca catechu L.	Wound healing [Bharat et al., 2014] [a/vivo rat]
Argemone mexicana L.	Anti-HIV [Sabde et al., 2011] [h/cell line]; anti-bacterial [Bhattacharjee I et al., 2006] [vit]; anti-inflammatory [Sourabie et al., 2012] [a/vivo mouse]; wound healing [Dash and Murthy, 2011] [a/vivo rat]
Arisaema flavum (Forsk.) Schott.	Moderately anti-bacterial [Bibi et al., 2011] [vit]
Arisaema heterophyllum Blume	No records found
Aristolochia anguicida Jacq.	No records found
Aristolochia bracteolata Lam.	Anti-bacterial, anti-fungal [Vaghasiya and Chanda , 2007][vit]; anti-inflammatory [Shirwaikar and Somashekar, 2003] [a/vivo rat]
Aristolochia esperanzae Kuntze	No records found
Aristolochia grandiflora Sw.	No records found
Aristolochia indica L.	Anti-venom; anti-inflammatory [Rakesh et al., 2010] [a/vivo rat]; anti-bacterial [Kamaraj et al., 2012]
Aristolochia ringens Vahl.	Anti-inflammatory [Ruth et al., 2014] [a/vivo rat, mouse]; anti-bacterial, anti-fungal [Fasola et al., 2015] [vit]
Aristolochia rugosa Lam.	No records found
Aristolochia tagala Cham.	Anti-inflammatory [Battu et al., 2011] [a/vivo rat, a/cell line mouse]

Aristotelia chilensis (Molina) Stuntz Syn: *Aristotelia macqui* L'Hér	Anti-inflammatory, anti-oxidant [Muñoz et al, 2011] [a/vivo guinea pigs, vit]
Arnebia euchroma (Royle) I.M.Johnst.	Anti-bacterial [Damianakos et al., 2012] [vit]
Arnica montana L.	Anti-inflammatory [Klaas et al., 2002] [a/vivo mouse]
Artemisia absinthium L.	Anti-viral [HIV] [Mohabatkar et al., 2015] [h/cell line]; wound healing [Gaspar-Pintiliescu et al., 2018] [h/cell line]; anti-depressant, anti-oxidant [*Mahmoudi et al., 2009] [a/vivo mouse];* anti-neuroinflammatory [Zeng KW et al., 2015] [a/cell line mouse]; anti-inflammatory [Moacă et al., 2019]; *Artemisia asiatica* Hsp modulation [Kim JM et al., 2009] [h/cell line]
Artemisia annua L.	Anti-virus [HSV] [Karamoddini et al., 2011] [vit], [SARS-CoV] [Li SY et al., 2005] [h/cell line, a/cell line monkey]; anti-inflammatory, antioxidant and anti-microbial [Kim WS et al., 2015] [a/cell line mouse, vit]; immunomodulation [Th1 to Th2] [Khakzad et al., 2017] [a/vivo MS mouse]; wound healing Mirbehbahani et al., 2020] [vit]; increased neuronal maturation, reduced paralysis, anti-adipogenic [Baek HK et al., 2017] [a/vivo obese mouse, *C. elegans* AD model]<br>*toxicity report [Ruperti-Repilado et al., 2019]
Artemisia arborescens (Vaill.) L.	Anti-viral [HSV] [Saddi et al., 2007] [a/cell line monkey]
Artemisia campestris L	Anti-hypertensive [Dib et al., 2017] [a/vivo rat]; anti-bacterial; anti-inflammatory, wound healing [Ghlissi et al., 2016] [a/vivo rat];
Artemisia gmelinii Weber ex Stechm.	No records found
Artemisia indica Willd.	Anti-microbial, anti-oxidant [Rashid S et al., 2013] [vit]
Artemisia judaica L	Anti-neuroinflammatory, anti-apoptotic, anti-oxidant, neuroprotective [Albasher et al., 2020] [a/vivo rat]
Artemisia scoparia Waldst. & Kitam.	Anti-viral [influenza] [Wang L et al., 2017] [vit]; anti-hypertensive [Cho JY et al., 2015] [a/vivo rat]
Artemisia sodiroi Hieron. ex Sodiro	No records found
Artemisia stolonifera (Maxim) Kom.	No records found
Artemisia vulgaris L.	Anti-microbial [Blagojević et al., 2006] [vit]; hypolipidemic, anti-inflammatory, antioxidant [El-Tantawy, 2015] [a/vivo rat]; anti-convulsant [de Almeida ER et al., 2013] [a/vivo mouse]
Arthrostemma ciliatum Pav. Ex D. Don	No records found
Artocarpus altilis (Parkinson ex F.A.Zorn) Fosberg Syn*:* *Artocarpus communis*	cholesterol-reducing [Adaramoye and Akanni, 2014] [a/vivo rat]; anti-hypertensive [Nwokocha et al., 2012] [a/vivo rat]
Artocarpus heterophyllus Lam.	Anti-viral [Hafid et al., 2017] [vit]; anti-inflammatory [Wei BL et al., 2005] [a/cell line rat]
Artocarpus integer (Thunb.) Merr.	Anti-inflammatory [Shah MK et al., 2016] [vit]
Ascarina philippinensis C.B.Rob.	No records found
Asclepias curassavica L.	Anti-bacterial [Reddy SH et al., 2012] [vit]
Asparagus africanus Lam.	Anti-inflammatory [Hassan HS et al., 2008] [a/vivo rat]
Asparagus cochinchinensis (Lour.) Merr.	Anti-neuro-inflammatory [Jian R et al., 2013] [a/microglial cell line mouse]
Asparagus filicinus Buch.-Ham. ex D.Don	Anti-viral [influenza] [Rajbhandari et al., 2009] [a/cell line monkey]; anti-hypolipidemic [Mishra et al., 2017] [a/vivo rat]
Asparagus racemosus Willd.	Anti-amyloidogenic, AChE inhibition [Kashyap et al., 2020] [vit]; neuroprotective, increased NGF + BDNF [Bhatnagar et al., 2009] [a/cell line rat neuron]; anti-epileptic [Jalalpure et al., 2009] [a/vivo rat]; anti-inflammatory [Mittal and Dixit, 2013] [a/vivo rat]; immunomodulatory [Gautam M et al., 2009] [a/vivo mouse]; anti-bacterial [Potduang et al., 2008] [vit]; anti-venom [Prashar et al., 2016] [a/vivo rat]

Asperula setosa Jaub. & Spach	No records found
Asphodelus aestivus Brot.	ROS activity [Peksel et al., 2013] [vit]; anti-ulcerogenic [Gürbüz et al., 2002] [a/vivo rat];
Asphodelus tenuifolius Cav.	Anti-inflammatory [Saleem M et al., 2020] [a/vivo rat]; anti-bacterial, anti-oxidant [Eddine et al., 2015] [vit]
Aspidosperma excelsum Benth.	No records found
Aspidosperma quebracho-blanco Schltdl.	Anti-inflammatory [Kaur M, 2013] [a/vivo rat]
Aspidosperma spruceanum Benth. ex Müll.Arg.	Anti-bacterial [Costa GM et al., 2019] [vit]
Aspidosperma subincanum Mart.	Anti-hypertensive [Bernardes et al., 2013] [a/vivo rat, isolated artery rat]
Asplenium nidus L.	Anti-inflammatory, anti-bacterial [Amoroso et al., 2014] [a/vivo rat]
Aster diplostephioides (DC.) C.B.Clarke	No records found
Asteriscus graveolens (Forssk.) Less	Anti-microbial [Al-Rimawi et al., 2018] [vit]; anti-hypertensive [El-Ouady F et al., 2020] [a/vivo rat]
Asteromyrtus symphyocarpa (F.Muell.) Craven	Anti-platelet aggregation [Rogers et al., 2000] [vit]
Astragalus complanatus R. Br.	Immunostimulatory [Qi et al., 2011] [a/vivo mouse]; *A. membranaceus* [in spp. Comb] reduced apoptosis + oxidative stress [Wang, S.-E. et al., 2015] [a/vivo mouse]
Astragalus fasciculifolius Boiss.	No records found<br>*A. lentiginosus* Hsp90 modulation [Dal Piaz et al., 2012] [vit]
Astronium urundeuva Engl. Syn: *Myracrodruon urundeuva* Allemão	Wound healing [Teixeira MC et al., 2020] [a/vivo rat]; anti-oxidant, AChE inhibition [Penido AB et al., 2017] [vit]; [Viana et al., 2003] [a/vivo mouse]
Asystasia gangetica (L.) T. Anders.	Anti-inflammatory [Adeyemi et al., 2011] [a/vivo rat, mouse]
Asystasia nemorum Nees	Anti-oxidant [Charoenchai et al., 2010] [vit]
Ataenidia conferta (Benth.) A.C.Ley	No records found
Atalantia monophylla DC.	Anti-viral [HSV] [Chansakaow et a., 1996]; anti-inflammatory [Rao BG et al., 2008] [a/vivo rat]
Atractylis aristata Batt.	No records found
Attalea phalerata Mart. ex Spreng	Anti-inflammatory [Freitas de Lima et al., 2018] [a/vivo rat]
Austroeupatorium inulaefolium (Kunth) R.M.King & H.Rob. Syn: *Eupatorium inulaefolium* L.	Anti-viral [HSV][Simoes et al., 1999] [a/cell line monkey]
Averrhoa bilimbi L.	Anti-hypertensive [Bipat et al., 2008] [a/isolated guinea pig atria]
Averrhoa carambola L.	Anti-inflammatory [Cabrini et al., 2011] [a/vivo mouse]; memory improvement, anti-apoptotic [Wei et al., 2018] [a/vivo mouse]
Ayapana triplinervis (Vahl) R.M. King & H.Rob. Syn: *Eupatorium triplinerve* Blume	Anti-inflammatory [Parimala et al.,2012] [a/vivo rat]
Azadirachta indica A. Juss.	Neuroprotective [Yanpallewar et al., 205] [a/vivo rat]; anti-inflammatory [Soares et al., 2014] [a/vivo mouse]; Hsp90 modulation [Gualtieri et al., 2014] [vit]; anti-bacterial [Joshi B et al., 2011] [vit]; anti- viral [polio] [Faccin-Galhardi et al., 2012] [vit], [HIV] [Awah et al., 2011] [h/cell line]; anti-hypertensive [Obiefuna and Young, 2005]; anti-venom [Sani et al., 2020b] [a//vivo rat]
Azara microphylla Hook.f.	No records found
Azolla imbricata (Roxb. ex Griff.) Nakai	No records found
Azorella diapensioides A.Gray	No records found
Baccaurea lanceolata (Miq.) Müll.Arg.	No records found<br>*Baccaurea courtallensis* anti-inflammatory [Muhammed Jasim et al., 2019] [a/vivo rat]

Baccharis genistelloides Lam. Pers.	Anti-viral [HIV] [Abad et al., 1999] [a/cell line monkey]
Baccharis latifolia (Ruiz & Pav.) Pers.	Anti-inflammatory [Abad et al., 2006] [a/cell line mouse]; anti-viral [HIV] [Abdel-Malek et al., 1996] [h/cell line]; moderately anti-bacterial [Bussmann et al., 2009] [vit]
Baccharis nitida (Ruiz & Pav.) Pers Syn: *Baccharis obtusifolia*	Moderately anti-bacterial, anti-fungal [Valarezo et al., 2015] [vit]
Baccharis pingraea DC. Syn: *Baccharis salicifolia* (Ruiz. & Pav.) Pers.	No records found
Baccharis punctulata DC.	Anti-inflammatory [Ascari et al., 2019] [a/vovo mouse]
Baccharis trimera (Less.) DC.	Anti-viral [polio] [Simoes et al., 1999] [a/cell line monkey]
Baccharoides adoensis (Sch.Bip. ex Walp.) H.Rob. Syn: *Vernonia grantii* Oliv.	No records found
Baccharoides filigera (Oliv. & Hiern) "Isawumi, El-Ghazaly & B.Nord." Syn: *Vernonia hymenolepis* Vatke	Anti-bacterial [Noumedem et al., 2013] [vit]
Bacopa floribunda (R.Br.) Wettst.	No records found
Bacopa monnieri (L.) Wettst	Cognitive and memory improvement [Kumar N et al., 2016] [h/c]; anti-neuroinflammatory [Nemetchek et al., 2017] [a/cell line mouse microglia]; anti-amyloidogenic [Malishev et al., 2017] [vit]; increased BDNF [Kwon et al., 2018] [a/vivo mouse]
Badilloa steetzii (B.L.Rob.) R.M.King & H.Rob. Syn: *Eupatorium subhastatum* Hook. & Arn.	Anti-inflammatory [Clavin et al., 2013] [a/vivo mouse]
Balanites aegyptiaca (L.) Delile	Anti-viral [HSV], anti-bacterial, antifungal [Al Ashaal et al., 2010] [vit]; anti-viral [HIV] [Hussein et al., 1999] [h/cell line]
Bambusa bambos (L.) Voss Syn: *Bambusa arundinacea* Willd.	No records found
Bambusa multiplex (Lour.) Raeusch. ex Schult.f.	No records found
Bambusa sp.	*Bambusa tuldoides* anti-fatigue [Zhang et al., 2006] [a/vivo mouse]; anti-hyperlipidemic, antihypertensive [Jiao et al., 2007] [a/vivo rat, rat aorta]; *B. vulgaris* anti-viral [measles] [Ojo et al., 2009]
Bambusa vulgaris Schrad. ex J.C. Wendl	Anti-inflammatory, wound healing [Lodhi et al., 2016] [a/vivo rat]
Baphia nitida G.Lodd.	Anti-inflammatory [Onwukaeme, 1995] [a/vivo rat, mouse]
Barleria homoiotrichia C. B. Clarke	No records found *Barleria lupulina* anti-viral [HSV] [Yoosook et al., 1999] [vit]; *B. lupulina* anti-inflammatory, improved microvascular endothelium [Senger et al., 2016] [h/ endothelial cells]
Barleria prionitis L.	Anti-inflammatory [Singh B et al., 2003] [a/vivo rat]
Barnadesia arborea Kunth	No records found
Barteria nigritiana Hook.f.	No records found
Bassia muricata (L.) Asch.	Anti-bacterial [Chemsa et al., 2016] [vit]
Bauhinia sp.	*Bauhinia racemosa* anti-inflammatory [Gupta M et al., 2005]
Bauhinia variegata L.	Anxiolytic [Khare et al., 2016] [a/vivo mouse]
Begonia humilis Aiton	No records found<br>*Begonia malabarica* anti-bacterial [Ramesh et al., 2002] [vit]
Bejaria aestuans Mutis	Moderately anti-bacterial [Bussmann et al., 2008] [vit]; *Bejaria resinosa* anti-inflammatory [Matulevich Peláez et al., 2016] [a/vivo mouse]
Benincasa hispida (Thunb.) Cogn.	Anxiolytic [Dhingra and Joshi, 2012] [a/vivo mouse]
Berberis actinacantha Mart. ex Schult. & Schult.f.	No records found

Berberis angulosa Wall. ex Hook.f. & Thomson	No records found
Berberis darwinii Hook (ind)	AChE inhibition [Habtemariam, 2011] [vit]
Berberis goudotii Triana & Planch. ex Wedd.	No records found<br>*Berberis vulgaris* anti-inflammatory [Ivanovska and Philipov, 1996] [a/vivo mouse]
Berberis integerrima Bunge	Anti-hyperlipidemic [Ashraf et al., 2014] [a/vivo rat]; immunomodulatory [Fateh et al., 2015] [a/cell line mouse]
Berberis lycium Royle	Anti-bacterial, anti-fungal [Singh et al., 2007] [vit]
Berberis ruscifolia Lam.	Anti-bacterial [Mattana et al., 2012] [vit]
Bergenia ciliata Sternb.	Anti-oxidant, anti-bacterial [Singh M et al., 2017 [vit]; anti-viral [HSV] [Rajbhandari et al., 2009] [a/cell line monkey]
Bertholletia excelsa Bonpl.	Reduced oxidative stress, anti-inflammatory [Stockler-Pinto et al., 2014] [h/c]; increased Nrf2 expression [Cardozo et al., 2016] [h/c]; anti-fungal [Mohamed et al., 1996] [vit]
Beta vulgaris L.	Anti-bacterial [Ahmad and Beg, 2001]; anti-oxidant [Wruss et al., 2015] [vit]; anti-hypertensive, anti-inflammatory [Raubenheimer et al., 2017] [h/c]
Betula pendula Roth	Anti-neuroinflammatory [Li C et al., 2018] [a/microglial cell line mouse]
Bidens biternata (Lour.) Merr. & Sherff	No records found
Bidens pilosa L.	Anti-viral [HSV] [Simoes et al., 1999] [a/cell line monkey]; anti-bacterial [Deba et al., 2008] [vit]; anti-hypertensive [Dimo et al., 2002] [a/vivo rat]; T_H cell modulation [Chiang et al., 2007] [a/cell line mouse]; wound healing [Kyakulaga et al., 2011] [a/vivo rat]
Bidens schimperi Sch.Bip. ex Walp.	No records found
Bignonia aequinoctialis L. Syn: *Cydista aequinoctialis* (L.) Miers	No records found
Bignonia nocturna (Barb.Rodr.) L.G.Lohmann Syn: *Tanaecium nocturnum* (Barb.Rodr.) Bureau & K.Schum.	No records found
Bischofia javanica Blume	Anti-bacterial [Khan MR et al., 2001] [vit]
Biscutella didyma L.	No records found
Bistorta affinis (D.Don) Greene	No records found
Bistorta yunnanensis (Wall. ex Hook.f.) Yonek. & H.Ohashi	No records found
Bixa orellana L.	Anti-bacterial, anti-oxidant, anti-convulsant [Shilpi et al., 2006] [a/vivo mouse, vit]; anti-hyperlipidemic [Ferreira JM et al., 2013] [a/vivo mouse]; anti-venom [Núñez V et al., 2004] [a/vivo mouse]; neuroprotective [Fontaine et al., 2020] [a/vivo AMD mouse]
Bixa platycarpa Ruiz et Pav. ex G. Don.	No records found
Blepharis ciliaris (L.) B.L.Burtt	Anti-inflammatory [El-Shanawany et al., 2015] [vit]; anti-bacterial, anti-fungal [Abdallah and Gamal, 2013] [vit]
*Bletilla striata (*Thunb.) Rchb.f.	Anti-bacterial [Qian et al., 2015] [vit]
Blumea balsamifera DC.	ROS-scavenging [Nessa et al., 2004] [vit]; anti-neuroinflammatory [Ma J et al., 2018] [a/microglial cell line mouse]; anti-bacterial, anti-fungal [Sakee et al., 2011] [vit]
Blumea sinuata (Lour.) Merrill	No records found
Bobgunnia madagascariensis (Desv.) J.H.Kirkbr. & Wiersema Syn: *Swartzia madagascariensis* Desv.	Anti-venom [Molander et al., 2014] [vit]
Boerhavia diffusa L.	Anti-inflammatory [Gharate and Kasture, 2013] [a/vivo rat]; reduced mitochondrial dysfunction [Prathapan et al., 2014] [a/cell line rat heart]; wound healing [Juneja et al., 2020] [h/cell line

	keratinocytes, a/vivo rat]; anti-bacterial [Abo and Ashidi, 1999] [vit]
Boerhavia erecta L	Anti-inflammatory, anti-oxidant [Compaore et al., 2018] [vit]
Boesenbergia rotunda (L.) Mansf.	Anti-viral [dengue-2 virus] [Kiat et al., 2006] [vit]; anti-inflammatory [Isa et al., 2012] [a/cell line mouse]; accelerated wound healing [Mahmood et al., 2010] [a/vivo rat]
Bombax buonopozense P. Beauv.	Anti-inflammatory [Akuodor et al., 2011] [a/vivo rat, mouse]; anti-bacterial, anti-fungal [Mann et al., 2011] [vit]
Bombax ceiba Linn.	Anti-inflammatory [Anandarajagopal et al., 2013] [vit]
Bonafousia sp.	No records found<br>*B. longituba*, *B. trinervis* anti-inflammatory [De las Heras et al., 1998] [vit]
Bontia daphnoides L.	No records found
Borago officinalis L.	Anti-bacterial [Brantne and Grein, 1994] [vit]; anti-hyperlipidemic [Navarro-Herrera et al., 2018] [a/vivo C. elegans, rat]; memory improvement [Ghahremanitamadon et al., 2014] [a/vivo rat]; anti-inflammatory, anti-oxidant [Conforti et al., 2008] [vit]; anxiolytic [Komaki et al., 2015] [a/vivo rat]
Boscia arabica Pestal.	No records found
Boscia coriacea Graells	No records found *Boscia albitrunca* anti-bacterial, anti-fungal [Pendota et al., 2015] [vit]
Boscia gossweileri Exell	No records found
Boscia senegalensis Lam. ex Poir Syn: *Boscia octandra* Hochst. ex Radlk.	Anti-viral [HIV] [Ali et al., 2002] [vit]
Boswellia ovalifoliolata N.P.Balakr. & A.N.Henry	Anti-bacterial [Anitha and Sudarsanam, 2013] [vit]
Boswellia sacra Flück.	Anti-bacterial [Hasson et al., 2011] [vit[; anti-inflammatory [Lee HY et al., 2008] [a/vivo mouse]; *Boswellia* spp. Immunomodulatory, reduced MS disease activity [Stürner et al., 2018] [h/c]
Boswellia serrata Roxb. ex Colebr.	Anti-inflammatory [Sontakke et al., 2007] [h/c]; memory improvement [Taghizadeh et al., 2018] [h/c]
Bouchea prismatica (L.) Kuntze	No records found<br>*Bouchea fluminensis* anti-inflammatory [Costa VB et al., 2003] [a/vivo mouse]
Bowdichia virgilioides Kunth	Anti-inflammatory [Thomazzi et al., 2010] [a/vivo rat]; anti-bacterial, wound healing [Agra IK et al., 2013] [a/vivo mouse]
Brassica nigra (L.) W.D.J. Koch	Anti-inflammatory [Alam et al., 2011a] [a/vivo rat]; anti-bacterial [Obi RK et al., 2009] [vit]
Brassica oleracea L.	Anti-inflammatory [Eren et al., 2018] [a/ microglial cells mouse]; protection against amyloid β-induced neurotoxicity [Roth et al., 1999] [h/cell line, a/cell line mouse neuron];<br>sulforophane: enhanced proteasome activity [Gan et al., 2010] [h/cell line; a cell line monkey]; induced mitochondrial biogenesis [Brose et al., 2012] [h/cell line].<br>Quercetin: anti-tauopathic; inhibits ER stress [Chen J et al., 2016] [h/cell line neuron]
Brassica rapa L.	Anti-inflammatory [Shin JS et al., 2011] [vit, a/vivo]; anti-bacterial [Jasim., 2021] [vit]; immunomodulation [Tanaka et al., 2016] [a/vivo mouse]
Breynia vitis-idaea (Burm.f.) C.E.C.Fisch.	Anti-hyperlipidemic [Nagar and Chauhan, 2016] [a/vivo rat]
Bridelia atroviridis Müll.Arg.	Anti-bacterial, anti-fungal [Agyare et al., 2006] [vit]
Bridelia ferruginea Benth.	Anti-inflammatory [Olajide et al., 2000] [a/vivo mouse, rat]; anti-microbial [Irobi et al., 1994] [vit]
Bridelia micrantha (Hochst.) Baill.	Anti-bacterial [Green et al., 2010; Adefuye AO et al., 2011] [vit]; anti-HIV [Bessong et al., 2006] [vit]]
Brillantaisia owariensis P.Beauv Syn: *Brillantaisia patula* T. Anderson	No records found
Brocchia cinerea (Delile) Vis.	Anti-inflammatory, anti-oxidant [Ghouti et al., 2018] [a/cell line mouse, vit]

Brosimum gaudichaudii Trécul	No records found
Brosimum parinarioides Ducke	No records found<br>*Brosimum gaudichaudii* anti-bacterial [Borges et al., 2017] [vit]
Brucea javanica (L.) Merr.	Anti-bacterial [Sornwatana et al., 2013] [vit]; [Yang J et al., 2013] [a/mouse celll line, a/vivo mouse]; spinal muscle atrophy improvement [Baek et al., 2019] [/vivo mouse]
Brugmansia x candida Pers. Syn: *Datura arborea* Ruíz & Pav.	No records found
Bryonia cretica subsp. dioica (Jacq.) Tutin	No records found<br>* toxicity report Bourhia et al., 2020
Buchenavia tomentosa Eichler	Anti-fungal [Teodoro et al., 2015] [vit]
Buddleja asiatica Lour.	Anti-inflammatory [Hien et al., 2018] [a/cell line mouse microglia]
Buddleja coriacea J.Rémy Syn: *Buddleja utilis* Kraenzl.	No records found
Buddleja mendozensis Gillet ex Benth.	Anti-oxidant [Borneo et al., 2009] [vit]
Bulbophyllum mutabile (Blume) Lindl.	No records found
Bunium persicum (Boiss.) B. Fedtsch.	Anti-inflammatory [Hajhashemi et al., 2011] [a/vivo rat]
Bupleurum abchasicum Manden. Syn: *Bupleurum chinense D.C.*	No records found<br>*Bupleurum falcatum* reduced demyelination, anti- inflammatory, inhibits microglial activation, attenuated motor paralysis [in spp. Comb] [Choi JH et al., 2015] [a/vivo MS mouse]
Bupleurum longicaule Wall. ex DC.	No records found
Bursera graveolens (Kunth) Triana & Planch.	Anti-bacterial, anti-fungal [Mendez et al., 2017] [vit]; *Bursera simaruba* as anti-viral [Álvarez et al., 2015] [vit]
Bursera simaruba (L.) Sarg.	Anti-inflammatory [Carretero et al., 2008] [a/vivo mouse]
Butea monosperma (Lam.) Kuntze	Anti-viral [HIV] [Sabde et al., 2011] [h/cell line]; wound healing and anti-oxidant [Sumitra et al., 2005] [a/vivo rat]; anti-venom [Tarannum et al., 2012] [a/vivo mouse]; anti-amnesic [Thirupathi et al., 2016] [a/vivo mouse]]
Byttneria pescapriifolia Britton	No records found<br>*Byttneria herbacea* anti-inflammatory [Sarkar et al., 2012] [a/vivo rat]; *Byttneria pilosa* anti-bacterial, anti-oxidant [Khine, 2019] [vit]
Cadaba farinosa Forssk.	Anti-bacterial [Al-Fatimi et al., 2007] [vit]
Cadaba glandulosa Forssk.	Anti-bacterial [Alothyqi et al., 2016] [vit]
Cadaba rotundifolia Forssk.	Anti-bacterial [Alothyqi et al., 2016] [vit]
Caesalpinia crista L. Syn: *Caesalpinia nuga* (L.) W.T. Aiton	Anti-bacterial [Kumar A et al., 2014] [vit]; inhibits Aβ aggregation from monomers and oligomers [Ramesh B et al., 2010] [vit].<br>*C. sappan* [Syn. *Biancaea sappan* (L.) Tod] remodeling of Aβ fibrils into less toxic structures [Du et al., 2015] [h/cell line; vit]
Caesalpinia spinosa (Molina) Kuntze	Anti-bacterial [Kloucek et al., 2005] [vit]
Caesalpinia violacea Standl.	No records found
Cajanus cajan (L.) Millsp. Syn: *Cajanus indicus* Spreng. L	Anti-bacterial [Okigbo and Omodamiro, 2007] [vit]; anti-viral [Nwodo UU et al., 2011] [a/vivo chicken embryo, h/cell line]; attenuated memory impairment, stimulated amyloid β clearance, reduced microglial and astrocyte reactivity [Wang LS et al., 2019] [a/vivo mouse]; anti-inflammatory [Patel NK et al., 2014] [a/vivo rat]; anti-convulsant [Kore et al., 2019] [a/vivo rat, mouse]; anti-hypertensive [Nawaz KA et al., 2017] [vit]
Caladium bicolor (Aiton) Vent.	Moderately anti-bacterial [Biswas MK et al., 2013] [vit]; anti-convulsant, anxiolytic [Akhigbemen et al., 2019] [a/vivo mouse]
Calceolaria rugulosa Edwin	No records found
Calendula arvensis L.	Wound healing [Lavagna et al., 2001] [h/c] [in sp. comb.]
Calendula officinalis L.	Improved memory impairment [Moradkhani et al., 2015] [a/vivo rat]; anti-inflammatory [Preethi et al., 2009] [a/vivo mouse];

	neuroprotective [Shivasharan et al., 2013] [a/vivo HD rat]; wound healing [Pommier et al., 2004] [h/c]; anti-bacterial, anti-fungal [Efstratiou et al., 2012] [vit
Calliandra parviflora Benth.	No records found
Callicarpa arborea Roxb.	No records found
Callicarpa longifolia Lam.	Anti-bacterial, wound healing [Susilawati et al., 2018] [a/vivo rabbit, vit]
Callicarpa tormentosa (L.) L.	No records found<br>*Callicarpa japonica* anti-inflammatory [Shin NR et al., 2015] [a/vivo mouse, a/mouse cell line]
Callisia gracilis (Kunth) D.R.Hunt	No records found
Callisia monandra (Sw.) Schult. & Schult f.	No records found
Callisia repens (Jacq.) L.	No records found
Calophyllum brasiliense Cambess.	Anti-viral [HIV] [Huerta-Reyes et al., 2004] [vit]
Calotropis procera (Aiton) Dryand.	Anti-inflammatory [Kumar VL and Basu, 1994] [a/vivo rat]; wound healing, anti-bacterial [Samy et al., 2012] [a/vivo mouse]; anti-venom [Sani et al., 2020a] [a//vivo rat]; promotes sensory + motor nerve regeneration [Zafar et al., 2020] [a/vivo mouse nerve injury model]<br><br>Latex toxic [Kadir ref 105]
Calystegia sepium (L.) R.Br.	No records found
Calytrix brownii (Schauer) Craven	No records found
Camellia sinensis (L.) Kuntze	Anti-hypertensive [Sagesaka-Mitane et al., 1996] [a/vivo rat]; anti-hyperlipidemic [Stepien et al., 2018] [a/vivo rat]; potentiated neurogenesis [Gundimeda et al., 2010] [a/neuronal cell line rat]; anti-inflammatory [Chen BT et al., 2012] [a/vivo mouse], Kumar B et al., 2012] [a/vivo rat]; anti-inflammatory [Chen BT et al., 2012] [a/vivo mouse], Kumar B et al., 2012] [a/vivo rat];]; increased longevity [Li YM et al., 2007] [a/vivo *Drosophila*].<br><br>[EGCG] metal chelation [Abib et al., 2011] [vit]; anti-amyloidogenic, anti-tauopathic; improved cognitive performance [Rezai-Zadeh et al., 2005, 2008] [a/vivo mouse]; suppressed microglial activation [Li R et al., 2004] [h/neuronal cell line, a/neuronal cell line rat]; improved retinal function [Falsini et al., 2009] [h/c]; α-synuclein + amyloid-β fibrillogenesis inhibition [Bieschke et al., 2010] [vit]; modulated huntingtin misfolding [Ehrnhoefer et al., 2006] [a/vivo *Drosophila* HD model, yeast HD model]; activated Nrf2, reduced oxidative stress [Romeo et al., 2009] [a/neuronal cell line rat]; reduced retinal inflammation [Zhang L et al., 2016] [h/cell retinal endothelium]; inhibits prionogenesis [EGCG + DAPH-12] [Roberts et al., 2009] [vit, in vivo yeast cell].
Campomanesia speciosa (Diles) Mc Vaugh	No records found<br>*Campomanesia xanthocarpa* anti-inflammatory [da Silva et al., 2016] [a/vivo rat]; anti-inflammatory, reduced cholesterol, anti-oxidant [Viecili et al., 2014] [h/c]; anti-platelet aggregation [Otero et al., 2017] [h/c]
Campsiandra comosa Benth.	Anti-fungal [Rodrigues K et al., 2014] [vit]
Camptostemon schultzii Mast.	No records found
Campyloneurum sp.	*Campyloneurum amphostenon* moderately anti-microbial [Castillo-Juárez et al., 2009] [vit]; *Campyloneurum amphostenon* anti-inflammatory [Vargas Maji, 2017] [a/vivo rat]
Campyloneurum fuscosquamatum Lellinger	No records found
Canarium schweinfurthii Engl.	Anti-bacterial [Dzotam et al., 2016]; *Canarium pimela* anti-hypertensive, anti-oxidant, vasorelaxant [Wu J et al., 2017] [a/rat

	aortic rings, vit]; *Canarium album* anti-viral [influenza] [Chen F et al., 2020] [vit]; *Canarium album* anti-neuroinflammatory [Zhang S et al., 2019] [a/mouse microglial cell line]; *Canarium patentinervium* LOX + COX anti-inflammatory [Mogana et al., 2013] [vit]
Canna indica L. Syn: *C. bidentata*	Anti-HIV [Woradulayapinij et al., 2005] [vit]; anti-inflammatory [Chen HJ et al., 2013] [cell line]
Capparis cartilaginea Decne.	Anti-bacterial, anti-inflammatory, anti-oxidant [Moharram et al., 2018] [a/vivo rat, vit]
Capparis erythrocarpus Isert Syn: *Capparis acuminata* De Wild.	Anti-inflammatory [Twumasi et al., 2019] [a/vivo rodent]
Capparis grandis L.f.	No records found
Capparis spinosa L	Anti-inflammatory [El Azhary et al., 2017] [a/vivo mouse]
Capparis zeylanica L	Anti-inflammatory [Ghule et al., 2007] [a/vivo rat]
Capraria biflora L.	Anti-inflammatory [Acosta et al., 2003] [a/vivo rodents]; anti-hypertensive [Rodríguez-García et al., 2019] [vit]
Capraria viana Benth.	No records found
Capsella bursa-pastoris (L.) Medic.	Anti-neuroinflammatory [Choi WJ et al., 2014] [a/cell line mouse microglia]; anti-bacterial [Soleimanpour et al., 2013] [vit]; T-helper cell modulation [Ghoreschi et al., 2011][a/vivo MS mouse]; [fumaric acid esters] reduced diseae symptoms [Schilling et al., 2006] [a/vivo MS mouse]<br>*Capsella bursa-pastoris* as source of fumaric acid: Calabrese et al., 2012
Capsicum annuum L.	Anti-amyloidogenic [Ogunruku et al., 2017] [vit]; anti-oxidant [Shan et al., 2005] [vit]; anti-inflammatory [Hernández-Ortega et al., 2012] [a/vivo mouse]; anti-bacterial Koffi-Nevry et al., 2012] [vit]; anti-viral [HSV] [Hafiz et al., 2017] [a/cell line monkey]
Capsicum frutescens L.	Anti-bacterial [Abdou et al., 1972] [vit]
Caragana brevispina Benth.	No records found
Caralluma penicillata (Deflers) N.E.Br.	Anti-inflammatory [Albaser et al., 2014] [a/vivo guinea pig]
Caralluma speciosa (N.E.Br.) N.E.Br.	No records found
Caralluma tuberculata N.E.Br.	Reduced cognitive impairment [Khan MZ et al., 2016] [a/vivo mouse]; anti-oxidant [Rauf et al., 2013] [vit]
Carapa guianensis Aubl.	Anti-inflammatory [Penido C et al., 2006] [a/vivo muse]
Carapa procera DC.	Anti-bacterial, wound healing [Udoumoh et al., 2011] [a/vivo rat, vit]
Cardiospermum grandiflorum Sw.	Anti-fungal and moderately anti-bacterial [Olaoluwa and Aiyelaagbe, 2015] [vit]
Cardiospermum halicacabum L.	Anti-inflammatory [Huang MH et al., 2011] [a/vivo mouse, a/cell line mouse]; anti-venom [Chandra et al., 2011] [vit]; anti-bacterial [Viji and Murugesan, 2010] vit]
Careya arborea Roxb.	Anti-inflammatory [Begum et al., 2015] [a/vivo rat]; anti-bacterial, anti-fungal [Kumar RS et al., 2006] [vit]
Carica papaya L.	Anti-inflammatory [Inam et al., 2017] [a/vivo mouse]; anti-hypertensive [Brasil et al., 2014] [a/vivo rat]; anti-oxidant [Guizani et al., 2011[[h/neuronal cell line]; Nrf2 activation, neuroprotective [Murakami et al., 2018] [a/vivo mouse, a/rat astrocyte cells]; anti-venom [Molander et al., 2014] [vit]; wound healing [Tiwari et al., 2011] [a/vivo rat]; immunomodulatory [Jayasinghe et al., 2017] [a/vivo rat]; anti-bacterial [Doughari et al., 2007] [vit]
Cariniana estrellensis (Raddi) Kuntze	No records found<br>*Cariniana rubra* anti-fungal [Silva Junior, 2009] [vit]
Cariniana sp.	*Cariniana rubra* anti-inflammatory [Santos EN et al., 2011] [a/vivo rat, mouse]
Carissa carandas L.	Anti-inflammatory [Hati et al., 2014] [a/vivo rat]; anti-bacterial [Agarwal T et al., 2012] [vit]; modulation of mitochondria oxidative

	damage [Prakash A et al., 2013] [a/vivo rat]; anti-convulsant [Hegde K et al., 2009] [a/vivo mouse].<br><br>[Naringin] neurogenic [Leem et al., 2014] [a/vivo rat PD model]; [reduced autophagic stress and microglial activation [Jeong et al., 2015b] [a/vivo mouse].
Carissa edulis (Forssk.) Vahl	Anti-inflammatory; antioxidant [Woode et al., 2007] [a/vivo chick]; anti-convulsant [Ya'u et al., 2015] [a/vivo mouse]; anti-bacterial [Abdu et al., 2008] [vit]
Carissa spinarum L. Syn: *Carissa pubescens* A. DC.	Anti-inflammatory [Beck et al., 2016] [a/vivo rat]; anti-bacterial, wound healing [Sanwal R, Chaudhary, 2011] [a/vivo mouse, vit]
Carpolobia alba G.Don.	Anti-inflammatory [Chukwujekwu et al., 2005] [vit]
Carthamus tinctorius L.	Memory improvement, AChE inhibition [Kim JH et al., 2019 [a/vivo mouse]; anti-hypertensive [Nie et al., 2012] [a/vivo rat]; reduced arterial stiffness [Suzuki et al. 2010] [h/c]; anti-hyperlipidemic, anti-oxidant [Koyama et al., 2006] [a/vivo mouse]; improved mitochondrial energy metabolism [Tian et al., 2008] [rat brain mitochondria]; anti-inflammatory [Wang Y et al., 2014] [a/vivo mouse]
Caryocar brasiliense A.St.-Hil.	Moderately anti-bacterial, anti-oxidant [Paula-Ju et al., 2006] [vit]
Cascabela thevetia (L.) Lippold Syn: *Thevetia peruviana* (Pers.) K. Schum.	No records found
Casearia sp.	*Casearia sylvestris* anti-inflammatory [Esteves et al., 2005] [a/vivo rat]; *Casearia esculenta* anti-hyperlipidemic [Chandramohan et al., 2010] [a/vivo rat]
Casearia sylvestris Swartz	Anti-viral [Simoes et al., 1999] [a/cell line monkey]; anti-paralytic vs neurotoxic venoms, wound healing [de Campos et al., 2015] [phrenic nerve-diaphragm mouse, a/vivo mouse]
Cassia fistula L.	Neuroprotective [Thabit et al., 2018] [a/vivo *C. elegans*]; anti-bacterial, anti-fungal [Bhalodia and Shukla , 2011] [vit]; anti-inflammatory and anti-oxidant [Ilavarasan et al., 2005] [a/vivo rat]; anti-convulsant, anxiolytic [Kalaiyarasia et al., 2015] [a/vivo mouse]; anti-venom [Pandey S et al., 2011] [a/vivo rat]
Cassia grandis L. f.	Anti-inflammatory [Rao BG and Ramadevi, 2018] [a/vivo rat]; anti-bacterial [Awal et al., 2009] [vit]
Cassine transvaalensis (Burtt Davy) Codd	Anti-bacterial, anti-viral [HIV] [Mthethwa et al., 2014] [vit]
Cassytha filiformis L.	Anti-inflammatory [Sahu et al., 2012] [a/vivo mouse]
Castanea sativa Mill.	Anti-inflammatory [Schink et al., 2018b] [h/cell line]; anti-bacterial [Basile et al., 2000] [vit]; neuroprotective, anti-oxidant [Brizi et al., 2016] [h/neural cell line]
Castilleja coccinea (L.) Spreng.	No records found
Cattleya schroederae (Rchb.f.) Sander	No records found
Caulophyllum robustum Maxim. Syn: *Leontice robustum* (Maxim.) Diels	Cardioprotective [Si et al., 2010] [a/cell line]; anti-inflammatory [Wang QH et al., 2017] [a/vivo + cell line mouse]
Cayratia japonica (Thunb.) Gagnep.	No records found
Cecropia palmata Willd	No records found
Cecropia pachystachya Trécul	Anti-inflammatory [Schinella et al., 2008] [a/vivo mouse]
Cecropia peltata L.	Wound healing [Nayak, 2006] [a/vivo rat]
Cedrus deodara (Royle ex D. Don) G. Don	Anti-venom [Asad et al., 2013] [vit]; anti-bacterial [Zeng et al. 2012] [vit]
Ceiba pentandra (L.) Gaertn.	Anti-inflammatory [Itou et al., 2014] [a/vivo rat, mouse]; anti-ulcerogenic, anti-oxidant [Anosike et al., 2014] [a/vivo rat]; anti-bacterial [Muñoz-Cazares et al., 2018] [vit]
Ceiba samauma (Mart.) K.Schum.	No records found

Celastrus paniculatus Willd.	Anti-HD, improved learning and memory, neuroprotection [Malik et al., 2017] [a/vivo HD rat]; anti-inflammatory [a/vivo rat]; [Kulkarni et al., 2015] [a/vivo rat
Celosia argentea L.	Anti-inflammatory [Bhujbal et al., 2008] [a/vivo rat]
Celosia polystachia (Forssk.) C.C.Towns.	No records found
Celtis australis L.	Anti-bacterial, anti-fungal [Ota et al., 2017] [vit]; anti-inflammatory [Semwal and Semwal, 2012] [a/vivo rat]
Celtis philippensis Blanco	No records found
Celtis toka (Forssk.) Hepper & J.R.I.Wood Syn: *Celtis integrifolia* Lam.	Anti-oxidant [Fall et al., 2017] [vit]
Cenchrus echinatus L.	Anti-inflammatory [Silva AA et al., 2012] [h/cell line]
Centaurea benedicta (L.) L. Syn: *Cnicus benedictus* L.	No records found
Centaurium cachanlahuen (Mol.) Rob. (ind) L	No records found
Centaurium erythraea Rafn.	Anti-bacterial [Kumarasamy et al., 2002] [vit]
Centella asiatica (L.) Urb.	Memory improvement [Wattanathorn et al., 2008] [h/c]; anti-amyloidogenic [Dhanasekaran et al, 2009] [a/vivo mouse]; attenuates cognitive deficits [Xu et al. 2012] [a/vivo mouse, h/cell line neuronal]; improved cognitive function, increased expression of mitochondrial and antioxidant response genes [Gray et al., 2016, 2017] [a/vivo mouse, h/cell line neural, a/cell line neuron]; memory enhancement, anti-inflammatory [Somchit et al., 2004] [a/vivo rat]; neuronal growth stimulus [Rao KG et al., 2006] [a/vivo rat, neuronal cell line rat]; increased RGC survival and function [Huang W et al., 2018] [a/vivo rat glaucoma; neuroprotective [Mook-Jung et al., 1999] [a/cell line rat]; anti-hypertensive [Intharachatorn and Srisawat, 2013] [a/vivo rat]; wound healing [Shukla et al., 1999] [a/vivo guinea pig]; anti-bacterial [Oyedeji and Afolayan, 2005; Idris and Nadzir, 2017 [vit]
Ceropegia bulbosa Roxb.	Immunostimulatory [Kanase et al., 2016] [a/cell line mouse]
Ceropegia variegata Decne.	No records found
Cestrum mariquitense Kunth Syn: *Cestrum sendtnerianum*	No records found
Cestrum mutisii Willd. ex Roem. & Schult.	No records found
Cestrum parqui (Lam.) L'Hér. Syn: *Cestrum foetidissimum* (Lam.) L'Hér.	Anti-inflammatory [Backhouse et al., 1996] [a/vivo guinea pig]; human anti-platelet agglutination [Falkenberg et al., 2012] [a/cell line sheep]; anti-fungal [Ahmed DB et al., 2012] [vit]
Cestrum racemosum Ruiz & Pav.	No records found
Chamaecrista glandulosa (Michx.) Greene	No records found
Chamaedorea angustisecta Burret	No records found
*Chamaemelum nobile (*L.) All. Syn: *Anthemis nobilis* L.	Anti-inflammatory [Aremu et al., 2018] [a/vivo rat]
Chamaesyce hypericifolia (L.) Millspaugh	No records found
Chaptalia nutans (L.) Polák	Anti-inflammatory [Badilla et al., 1999a] [a/vivo rat]
Chaptalia sinuata (Less.) Baker	No records found
Cheilocostus speciosus (J.Koenig) C.D.Specht Syn: *Costus speciosus* (J.Koenig) Sm.	Anti-bacterial [Malabadi, 2005] [vit]; anti-inflammatory [Binny et al., 2010] [a/vivo rat]
Chelidonium majus L.	Anti-bacterial, anti-fungal [Kokoska et al., 2002] [vit]<br>*toxicity report [Stickel et al., 2003]

Chenopodium album L.	Anti-inflammatory [Usman et al., 2010] [a/vivo mouse]; anti-bacterial [Singh KP et al., 2011] [vit]
Chenopodium quinoa Willd.	Anti-inflammatory {Yao Y et al., 2014] [a/cell line mouse]; cholesterol-lowering [De Carvalho et al., 2014] [h/c]
Chiliadenus sericeus (Batt. & Trab.) Brullo Syn: *Varthemia sericea* (Batt. & Trab.) Diels	No records found
Chiliadenus iphionoides (Boiss. & C.I.Blanche) Syn: *Varthemia iphionoides* Boiss. & Blanche	Anti-platelet activity [Afifi and Aburjai, 2004] [vit]; anti-inflammatory [Al-Bakheit et al., 2017] [h/cell line]
Chiococca alba (L.) Hitchc	Anti-inflammatory [Ruppelt et al., 1991] [a/vivo]
Chlamydocola chlamydantha K.Schum.	No records found
Chlorophytum stolzii (K.Krause) Weim	No records found
Chromolaena maximilianii (Schrad. ex DC.) R.M.King & H.Rob.	No records found
Chromolaena odorata (L.) R.M.King & H.Rob. Syn: *Eupatorium odoratum* L.	Anti-PAFR [Ling et al., 2007] [a/cell rabbit platelet]; wound healing [Phan et al., 2001] [h/cell line keratinocyte]; anti-amyloidogenic, attenuated memory impairment Pakdeepak et al., 2010] [a/vivo mouse]; anti-inflammatory [Dhar et al., 2018] [a/cell line mouse]; anti-bacterial [Stanley et al., 2014] [vit]
Chromolaena scabra (L. f.) R.M. King & H. Rob.	No records found
Chromolaena squalida (DC.) R.M.King & H.Rob.	Anti-bacterial [Taleb-Contini et al., 2003] [vit]
Chrysactinia mexicana A.Gray	Anti-inflammatory [Ku and Lin, 2013] [a/cell line]
Chrysanthemum morifolium Ramat.	Anti-bacterial [Kuang et al., 2018] [vit]
Chukrasia tabularis A.Juss.	Anti-bacterial, anti-fungal [Nagalakshmi et al., 2001] [vit]
Chuquiraga jussieui J.F.Gmel.	No records found
Chuquiraga spinosa Less.	Anti-inflammatory, anti-oxidant, anti-fungal [Casado et al., 2011] [a/vivo rat]; anti-bacterial [Bussmann et al., 2008] [vit]
Chuquiragua weberbaueri Tovar	No records found
Cichorium intybus L.	Anti-microbial [Kokoska et al., 2002] [vit]
Cichorium pumilum Jacq.	Anti-oxidant, anti-bacterial [Al Khateeb et al., 2012] [vit]
Cinchona pubescens Vahl	No records found<br>*Cinchona officinalis* anti-PAF [Shah et al., 1998] [h/cell line platelets]
Cinnamomum spp.	*Cinnamomum cassia* improved memory deficits, anti-neuroinflammatory, reduced tau hyperphosphorylation and microglial activation [Zhao Y et al., 2019] [a/vivo mouse] ; [anti-oxidant [Shan et al., 2005] [vit]; mitochondrial biogenesis up-regulation, energy-boosting, decreased weight [Song MY et al., 2017] [a/vivo obese mouse, a/cell mouse muscle]
Cinnamomum tamala (Buch.-Ham.) T.Nees & Eberm.	Anti-bacterial [Goyal et al., 2009] [vit]; anxiolytic [Upadhyay et al., 2016] [a/vivo rat]
Cinnamomum verum J.Presl Syn*: Cinnamomum zeylanicum* Blume	Anti-inflammatory [Schink et al., 2018a] [h/cell line]; Zareie et al., 2020] [h/c]; *C. cassia* anti-hypertensive [Akilen et al., 2010] [h/c]; inhibited tau aggregation, anti-oxidant [George et al., 2013] [vit]; raised Nrf2 [cinnamaldehyde] [Wang F et al., 2015] [a/cell line, isolated aorta mouse]; cognitive improvement [Pandit et al., 2018] [a/vivo rat]; anti-bacterial [Puangpronpitag and Sittiwet, 2009] [vit]; anxiolytic [Fadaei et al., 2017] [a/vivo rat]
Cissampelos owariensis P. Beauv. ex DC.	No records found<br>*Cissampelos mucronata* anti-venom [Molander et al., 2014]
Cissampelos pareira L.	Anti-bacterial [Ngoci et al., 2014] [vit]; anti-venom [Verrastro et al., 2018] [vit]; memory improvement, AChE inhibition [Kulkarni PD et al., 2011] [a/vivo mouse]

Cissus adnata Roxb.	Anti-bacterial, anti-oxidant [Shoibe et al., 2017] [vit]
Cissus aralioides (Welw. ex Baker) Planch.	No records found
Cissus quadrangularis L.	Anti-viral [HIV] [Ali et al., 2002] [vit]; anti-bacterial [Sánchez-Medina et al., 2001] [vit]; anti-inflammatory [Bhujade et al., 2012] [vit]
Cissus rotundifolia Vahl	Anti-bacterial [Al-Bukhaiti et al., 2020] [vit]
Cissus verticillata (L.) Nicolson & C.E.Jarvis Syn*: Cissus sicyoides* L.	Moderately anti-bacterial [Lozano et al., 2013] [vit]
Citharexylum spinosum L. Syn: *Citharexylum quadrangulare* Jacq.	Anti-inflammatory [Mohammed et al., 2016] [a/vivo rat]
Citrullus colocynthis (L.) Schrad.	Anti-inflammatory [Marzouk et al., 2010] [a/vivo rat]; anti-hyperlipidemic [Zamani et al., 2007] [a/vivo rabbit]
Citrus aurantifolia (Christm. & Panz.) Swingle	Anti-inflammatory [Dongmo et al., 2013] [vit]; *Citrus* spp: reduced apoptosis [Johnson et al., 2009] [h/cell line retinal]; anti-bacterial [Onyeagba et al., 2004] [vit]
Citrus x aurantium L.	Anti-apoptotic, anti-inflammatory, anti-oxidant [Youn et al., 2019] [a/cell line rat]; neurotrophic [Jeong et al., 2015a] [a/vivo rat PD]; anti-bacterial [Teneva et al., 2019] [vit]<br>[Naringin] reduced cognitive and mitochondrial dysfunction [Kumar A et al., 2010] [a/vivo mouse].<br>*Citrus aurantium* as source of naringin: Peterson et al., 2006b
Citrus hystrix DC.	Anti-inflammatory [Kidarn et al., 2018] [a/cell line mouse]
Citrus limetta Riso	Anti-inflammatory [Mohanty et al., 2015] [vit, a/vivo mouse]; anti-hypertensive [Perez et al., 2010] [a/vivo mouse]
Citrus limon (L.) N.L. Burm. f.	Anti-bacterial [Moosavy et al., 2017] [vit]; anti-viral [Hep A] [Battistini et al., 2019] [vit]; anti-inflammatory [Dongmo et al., 2013] [vit]; anti-hypertensive [Miyake et al., 1998] [a/vivo rat]; reduced apoptosis [Johnson et al., 2009] [h/cells retinal].
Citrus maxima (Burm.) Merr. Syn: *Citrus grandis* (L.) Osbeck	Anti-inflammatory [Shivananda et al., 2013] [a/vivo rat]; anti-epileptic [Sheik et al., 2014] [a/vivo rat, mouse]<br>[Naringin] reduced cognitive and mitochondrial dysfunction [Kumar A et al., 2010] [a/vivo mouse].<br>*Citrus maxima* as source of naringin: Diaconu et al., 2017.
Citrus paradisi Macfad	Anti-bacterial, anti-fungal [Cvetnić et al., 2004] [vit]; anti-bacterial [Brorson and Brorson, 2007] [vit].<br>[Naringin] reduced cognitive and mitochondrial dysfunction [Kumar A et al., 2010] [a/vivo mouse]; anti-viral [Hep A] [Battistini et al., 2019] [vit].<br>*Citrus paradisi* as source of naringin: Peterson et al., 2006a
Citrus reticulata Blanco †	Anti-bacterial [Jayaprakasha et al., 2000] [vit]; anti-inflammatory [Huang YS and Ho, 2010] [a/cell line mouse]
Citrus sinensis (L.) Osbeck	Anti-viral [HSV] [El-Serehy et al., 2014] [vit]; anti-oxidant [Abeysinghe et al., 2007] [vit]; anti-fungal [Ortuño et al., 2006] [vit]; anti-atherosclerotic [Parmar and Kar, 2007] [a/vivo rat] [anti-inflammatory [Pepe G et al., 2018] [a/cell line mouse], Leguizamón et al., 2019] [a/vivo rat]; anti-convulsant [Citraro et al., 2016[a/vivo rat, mouse]
Clausena anisata (Willd.) Hook.f. ex Benth.	Anti-microbial [Geyid et al., 2005] [vit]; anti-convulsant [Kenechukwu et al., 2012] [a/vivo mouse]; anti-hypertensive [Lechaba et al., 2016] [a/vivo rat]
Cleistopholis glauca Pierre ex Engl. & Diels	No records found
Clematis acuminata DC.	No records found
Clematis barbellata Edgew.	No records found
Clematis henryi Oliv.	Anti-inflammatory [Sun X et al ., 2016] [a/vivo rat]
Clematis heracleifolia Komarov.	Anti- HIV [Min et al., 2001] [vit]
Clematis tibetana Kuntze	No records found
Cleome amblyocarpa Barratte & Murb.	No records found

Cleome gynandra L.	Anti-inflammatory [Narendhirakannan et al., 2007] [a/vivo rat]
Cleome viscosa L.	Anti-inflammatory [Bawankule et al., 2008] [a/vivo mouse]; wound healing [Singh et al., 2017] [a/vivo rat]
Clerodendrum cyrtophyllum Turcz.	Anti-inflammatory , anti-oxidant [Liu H et al., 2011] [vit, a/cell line mouse]
Clerodendrum floribundum R.Br.	Anti-inflammatory [Sweeney et al., 2001] [vit]
Clerodendrum infortunatum L. Syn: *Clerodendrum viscosum* Vent.	Anti-bacterial [Choudhury et al., 2010] [vit]; *Clerodendron serratum* anti-inflammatory [Narayanan N. et al., 1999] [a/vivo rat]; anti-convulsant [Rath et al., 2018] [a/vivo rat]
Clerodendrum paniculatum L.	Anti-inflammatory [Phuneerub et al., 2015] [a/cell line mouse]
Clerodendrum phlomidis L.f.	Anti-inflammatory [Babu et al., 2014] [a/vivo rat]
Clerodendrum sp.	*C. glabrum* anti-venom [Molander et al., 2014] [vit]
Clibadium sylvestre (Aubl.) Baill.	No records found
Clidemia hirta (L.) D. Don	No records found
Clinacanthus nutans (Burm.f.) Lindau	Anti-bacterial, anti-fungal, anti-oxidant [Arullappan et al., 2014] [vit]
Clinopodium nepeta subsp. *glandulosum* (Req.) Govaerts Syn: *Calamintha officinalis*	Anti-bacterial [Monforte et al., 2011] [vit]
Clinopodium taxifolium (Kunth) Govaerts	No records found
Clinopodium umbrosum (M.Bieb.) K.Koch	Moderately anti-viral [Rajbhandari et al., 2009] [a/cell line monkey]
Clitoria ternatea L.	Anti-inflammatory [Devi BP et al., 2003] [a/vivo rat]
Clusia ellipticifolia Cuatrec.	No records found
Cnestis platantha (Lour.) Merr.	No records found
Cnidoscolus urens (L.) Arthur	No records found
Coccinia grandis (L.) Voigt Syn: *Coccinia indica* Wight & Arn.	Anti-viral [HBV] [Arbab et al., 2017] [h/cell line]<br>* toxicity report [Orech et al., 2005]
Cochlospermum orinocense (Kunth) Steud.	No records found
Cochlospermum regium (Schrank) Pilg.	Anti-oxidant, AChE inhibition [de Miranda Pedroso et al., 2019] [a/vivo rat]
Cochlospermum religiosum (L.) Alston	Anti-bacterial [Ponnamma et al., 2017] [vit]
Cocos nucifera L.	[scyllo-cyclohexanehexol] anti-amyloidogenic, ameliorates impaired cognition [McLaurin et al., 2006] [a/vivo AD mouse]; ALS enhanced motor performance, prolonged survival [Weerasekera et al., 2018] [a/vivo ALS mouse]; tau reduction [Radenahmad et al., 2011] [a/vivo rat]; anti-inflammatory, anti-oxidant [Padumadasa et al., 2016] [vit]; anti-viral [HSV], anti-bacterial [Esquenazi et al., 2002] [vit]; anti-fungal [Venkataraman et al., 1980]; wound healing [Srivastava and Durgaprasad, 2008] [a/vivo rat]; reduced mitochondrial lipid peroxidation [Sandhya et al., 2003] [a/vivo rat]<br>*C. nucifera* as abundant source of scyllo-cyclohexanehexol: Velander et al., 2017
Codonoboea crinita (Jack) C.L.Lim Syn: *Didymocarpus crinitus* Jack	No records found
Codonopsis clematidea (Schrenk) C.B.Clarke	No records found
Codonopsis pilosula (Franch.) Nannf.	Anti-inflammatory [Chu X et al., 2016] [a/vivo mouse]; immunostimulatory [Yongxu and Jicheng, 2008] [vit]
Coffea arabica L.	Anti-inflammatory [de Castro Moreira et al., 2013] a/vivo rat]; Reduced ROS, Nrf2 activation [Hwang YP and Jeong, 2008] [h/cell line neuronal]
Coffea mauritiana Lam.	No records found
Coix lacryma-jobi L.	Anti-inflammatory [Choi G et al., 2015] [a/cell line mouse]; anti-bacterial [Das S et al., 2017] [vit]

Cola acuminata (P. Beauv.) Schott & Endl. Syn: *Sterculia acuminata* P. Beauv.	Ameliorated memory impairment [Ishola et al., 2018] [a/vivo rat]; AChE inhibition, anti-oxidant [Oboh et al., 2014] [vit, a/cell rat brain]; anti-bacterial, anti-fungal [Dah-Nouvlessounon et al., 2015] [vit]
Colletia spinosissima J.F. Gmel.	No records found
Colubrina asiatica (L.) Brongn.	Anti-oxidant [Nivas et al., 2015]
Combretum apiculatum Sond.	Anti-bacterial [Eloff, 1999] [vit]
Combretum hensii Engl. & Diels	No records found
Combretum micranthum G.Don	Anti-viral [HSV] [Ferrea et al., 1993] [vit]; anti-inflammatory [Olajide et al., 2003] [a/vivo rat]
Combretum molle R.Br. ex G.Don	Anti-inflammatory [Ponou et al., 2008] [a/vivo rat]; anti-bacterial, anti-fungal [Asres et al., 2006] [vit]; anti-venom [Molander et al., 2014] [vit]
Commelina benghalensis L.	Wound healing [Sambrekar et al., 2011] [a/vivo rat]
Commelina communis L.	Anti-viral [influenza] [Bing et al., 2009] [a/vivo mouse, a/cell line]
Commelina dianthifolia Redouté	No records found
Commelina diffusa Burm.f.	Anti-inflammatory [Mensah et al., 2014] [a/vivo chick]; wound healing, anti-microbial [Mensah et al., 2006] [vit]
Commelina erecta L. Syn: *Commelina elegans* Kunth	Anti-bacterial [Fonkeng et al., 2015] [vit]
Commicarpus helenae (Roem. & Schult.) Meikle	No records found
Commiphora caudata (Wight & Arn.) Engl.	Anti-inflammatory [Mohan et al., 2009] [a/vivo rat]
Commiphora foliacea Sprague	Anti-bacterial [Al-Fatimi et al., 2007]
Commiphora gileadensis (L.) C.Chr.	Anti-inflammatory [Al-Howiriny et al., 2004] [a/vivo rat]; anti-convulsant [Zaidi et al., 2010] [a/vivo mouse]
Commiphora habessinica (O.Berg) Engl.	Anti-fungal [Hamedi et al., 2015] [vit]
Commiphora mollis (Oliv.) Engl.	Anti-inflammatory [Hassan HS et al., 2018] [a/vivo mouse]
Commiphora myrrha (T.Nees) Engl. Syn: *Commiphora molmol* (Engl.) Engl. ex Tschirch	Anti-bacterial, anti-fungal [Abd-Ulgadir, 2017] [vit]; anti-inflammatory [Atta and Alkofahi, 1998] [a/vivo mouse, rat]; immunomodulatory [Boual et al., 2020] [h/cell line]
Convolvulus arvensis L.	Anti-inflammatory [Saleem U et al., 2020] [a/vivo mouse, rat]
Convolvulus prostratus Forssk Syn: *Convolvulus pluricaulis* Choisy	Neuroprotective, reduced deficits in learning and memory [Bihaqi et al., 2011] [a/vivo rat]; memory enhancement [Nahata et al., 2008] [a/vivo rat]; reduced amyloid and tau, reduced neuronal loss [Bihaqi et al., 2012] [a/vivo rat]
Conyza sumatrensis (Retz.) E. H Walker Syn: *Erigeron floribundus* (Kunth) Sch.Bip.	Anti-inflammatory [Asongalem et al., 2004b] [a/vivo rat]; anti-bacterial, anti-fungal [Mabrouk et al., 2013] [vit]
Conyza vernonioides (Sch.Bip. ex A.Rich.) Wild. Syn: *Conyza adolfi-fridericii* Muschl.	No records found
Copaifera langsdorffi Desf.	Anti-inflammatory [Paiva et al., 200b2] [a/vivo rat]; wound healing [Paiva et al.,2002a] [a/vivo rat]; anti-bacterial, anti-fungal [Ribeiro VP et al., 2019] [vit]
Copaifera malmei Harms	Anti-ulcer [Adzu et al., 2015] [a/vivo mouse, rat]
Copaifera multijuga Hayne	Anti-inflammatory [Veiga et al., 2007] [vit, a/vivo mouse]
Copaifera pubiflora Benth.	Anti-bacterial [Fernández YA et al., 2018]
Copaifera reticulata Ducke	Wound healing [Feitosa et al., 2018] [a/vivo rat]
Coptis teeta Wall.	No records found<br>*C. chinensis* anti-amyloid, cognitive improvement [Durairajan et al., 2012] [a/vivo AD mouse]; reversesTDP-43 aggregation [Chang CF et al., 2016] [a/cell line mouse neuron]; reduced mutant huntingtin accumulation [Jiang W et al., 2015] [a/vivo HD mouse]; anti-PD: reduced neuronal damage, reduced apoptosis, improved motor functions and memory [Kim M et al., 2014] [a/vivo PD mouse]; anti-neuroinflammatory [Lu et al., 2010] [a/ cell line

	mouse microglia]; anti-viral [Xu et al., 1996] [vit]; *Coptis* spp.: autophagy regulation [Fan X et al., 2015] [a/cell line mouse]
Coptosperma sp.	No records found
Corchorus aestuans L.	Anti-inflammatory [Patel RP, 2011] [a/vivo rat]
Corchorus olitorus L.	Anti-bacterial [Yakoub et al., 2018] [vit]
Cordia curassavica (Jacq.) Roem. & Schult. Syn: *Cordia verbenacea* A.DC.	Anti-inflammatory [Fernandes ES et al., 2007] [a/vivo rat, mouse] *Cordia spinescens anti-viral* [HIV] [Matsuse et al., 1998] [h/cell line]
Cordia dichotoma G.Forst.	Anti-inflamatory [Sharma US et al., 2010] [a/vivo rat]
Cordia lutea Lam.	No records found
Cordia millenii Baker.	Anti-inflammatory [Avoseh et al., 2018] [a/vivo rat]
Cordia subcordata Lam.	No records found
Cordyline fruticosa (L.) A. Chev.	No records found
Cornus mas L.	Anti-oxidant, anti-inflammatory [Moldovan et al., 2016] [vit, a/vivo rat]; anti- ocular hypertension [Szumny et al., 2014] [a/vivo rabbit]; anti-hyperlipidemic [Asgary et al., 2014] [a/vivo rat]; immunomodulatory [Forman et al., 2016] [h/cell line]; anti-bacterial [Kyriakopoulos and Dinda, 2015] [vit]
Cornus officinalis Sieb. Et Zucc.	immunomodulatory [Yin L et al., 2014] [a/vivo MS rat]; anti-oxidant [Hwang KA et al., 2016] [a/cell line mouse]; anti-inflammatory [Akhavan et al., 2015] [a/cell line mouse]; anti- amyloidogenic [Lee JE et al., 2017] [vit]
Cornus sericea L.	Anti-inflammatory [Jiang Q et al., 2019] [h/cell line]
Corylus avellana L	Anti-bacterial, anti-oxidant [Oliveira et al., 2008] [vit]; memory improvement, anxiolytic [Bahaeddin et al., 2018] [a/vivo rat]
Costus afer Ker-Gawler	Anti-inflammatory, anti-oxidant [Anyasor et al., 2014] [a/vivo rat]
Costus comosus (Jacq.) Roscoe	No records found
Costus lucanusianus J.Braun & K.Schum.	Anti-bacterial [Baba and Onanuga, 2011] [vit]; anti-inflammatory [Owolabi and Nworgu, 2009] [a/vivo rat, mouse]
Costus sericeus Blm.	No records found
*Costus spicatu*s (Jacq.) Sw.	Anti-bacterial, anti-fungal [Uliana et al., 2015] [vit]; anti-inflammatory [Quintans et al., 2010] [a/vivo mouse, rat]
Cotula anthemoides L.	No records found
Coutarea hexandra (Jacq.) K.Schum.	No records found
Crassocephalum vitellinum (Benth.) S. Moore.	No records found
Crataegus laciniata Ucria	No records found
Crataegus spp.	*Crataegus curvisepala* anti-hypertensive [Asgary et a., 2004] [h/c]; [quercetin] attenuates tau hyperphosphorylation [Chen J et al., 2015]; [h/neuronal cell line]; [quercetin] anti-ATP synthase [Lang et al., 1974] [a/mitochondria bovine heart]; [quercetin] anti-atherosclerotic, raised Nrf2, anti-oxidant up-regulation [Li C et al., 2016] [h/cell line aortic endothelial]; *Crataegus pinnatifida* anti-inflammatory [Kao et al., 2005] [a/vivo rat, a/cell line mouse] *Crataegus* as source of quercetin: Alirezalu et al., 2020
Crateva adansonii Oliv.	Anti-hypertensive [Adjagba et al., 2017] [a/vivo rat]; anti-bacterial [Nounagnon et al., 2018] [vit]
Crescentia cujete L.	Anti-inflammatory, anti-bacterial [Parvin et al., 2015] [h/cell line, vit]; anti-venom [Molander et al., 2014] [vit]
Crinum asiaticum L. Syn: *Crinum angustifolium*	Anti-bacterial, anti-fungal [Iannello et al., 2014] [vit]; anti-inflammatory [Samud et al., 1999] [a/vivo mouse]
Crinum nubicum Hannibal	No records found
Crinum sp.	*Crinum glaucum* anti-inflammatory [Okpo et al., 2001] [a/vivo rat, mouse]
Cronquistianthus lavandulifolius D.C.	No records found

Crossopteryx febrifuga (Afzel. ex G. Don) Benth.	Anti-venom [Molander et al., 2014] [vit]; anti-inflammatory [Salawu et al., 2009] [a/vivo rat, mouse]
Crotalaria eremaea F.Muell.	No records found
Crotalaria pallida Aiton. Hort.	Anti-inflammatory, anti-oxidant, anti-bacterial, anti-fungal [Govindappa et al., 2011] [vit]
Crotalaria retusa L.	Anti-bacterial [Dhole et al., 2011] [vit]
Croton californicus Müll.Arg.	No records found
Croton draconoides Müll. Arg.	No records found
Croton grandivelum Baill.	No records found
Croton hibiscifolius Kunth ex Spreng. Syn: *Croton funckianus* Müll. Arg.	No records found
Croton macrostachyus Hochst. ex Delile	Anti-inflammatory [Matu and Van Staden, 2003] [vit]
Croton mongue Baill.	No records found
Croton mubango Müll. Arg.	Anti-inflammatory [Pompermaier et al., 2018] [a/cell line mouse]
Croton mutisianus Kunth	No records found
Croton schiedeanus Schltdl.	Anti-hypertensive, vasorelaxant [Guerrero MF et al., 2001] [a/vivo rat]
Croton sylvaticus Hochst.	No records found
Croton tiglium L.	Anti-viral [El-Mekkawy et al., 2000] [h/cell line]; anti-inflammatory [Wang JF et al., 2015] [vit]; neurite outgrowth upregulation [Nishina et al., 2017] [a/cell line rat brain]<br>* toxicity report [al-Mamun et al., 2010]
Croton urucurana Baill.	Anti-fungal [Gurgel LA et al., 2005] [vit]; anti-inflammatory [Cordeiro et al., 2016] [a/vivo mouse]
Croton wagneri Müll.Arg.	No records found
Crudia amazonica Spruce ex Benth.	No records found
Cryptocarya massoy Laur	No records found
Cryptolepis dubia (Burm.f.) M.R.Almeida Syn: *Cryptolepis buchananii* Roem. & Schult.	No records found
Cucumeropsis mannii Naudin.	No records found
Cucumis anguria L.	Anti-inflammatory [Gill et al., 2011] [a/vivo rat]
Cucumis ficifolium A.Rich.	No records found
Cucumis myriocarpus E. Mey ex Naud	No records found
Cucurbita maxima Duch	Anti-inflammatory [Lim and Choi, 2001] [a/vivo rat]
Cucurbita moschata Duchesne	Anti-inflammatory [Ko et al., 2013 [h/cell line]; anti-bacterial [Qian, 2014] [vit]
Cucurbita pepo L.	Anti-hypertensive [El-Mosallamy et al., 2012] [a/vivo rat]; anti-inflammatory [Karpagam et al., 2011] [a/vivo rat]; wound healing [Bardaa et al., 2016] [a/vivo rat]
Cuminum cyminum L.	Anti-amyloidogenic [Morshedi et al., 2014] [a/cell line rat neuron]; anti-oxidant [El-Ghorab et al., 2010] [vit]; anti-bacterial [Wongkattiya et al., 2019] [vit]
Cunila spicata Benth.	Anti-viral [HSV] [Simoes et al., 1999] [a/cell line monkey]
Cupania sp.	Anti-bacterial [De Lima et al., 2006] [vit]
Cuphea carthagenensis (Jacq.) J.F.Macbr	Cholesterol-lowering [Biavatti et al., 2004] [a/vivo rat]
Cuphea dipetala (L.f.) Koehne	No records found
Cuphea glutinosa Cham. & Schltdl.	No records found
Cuphea strigulosa H.B.K.	No records found
Cupressus dupreziana A.Camus	No records found
Cupressus lusitanica Mill.	Anti-bacterial, anti-fungal [Teke et al., 2013] [vit]
Curatella americana L.	Anti-inflammatory [Alexandre-Moreira et al., 1999] [a/vivo mouse]; anti-hyperlipidemic and anti-oxidant [Lopes et al., 2016] [a/vivo

	rat]; anti-viral [polio], anti-fungal [De Toledo et al., 2011] [a/cell line monkey, vit]
Curculigo pilosa (Schumach. & Thonn.) Engl.	Anti-oxidant [Sofidiya et al., 2011] [vit]
Curcuma longa L.	Inhibits amyloid aggregation, anti-tau, anti-inflammatory [Shytle et al., 2012] [a/vivo mouse, a/cell mouse microglia]; reduced retinal ganglion cell loss; [Davis et al., 2018] [a/vivo rat]; neurogenic, reversed cognitive deficits [Tiwari et al, 2013] [a/vivo rat, vit]; anti-viral [HBV] [Kim HJ et al., 2009] [h/cell line], [influenza] [Dao et al., 2012] [h/cell line]; *Curcuma* sp. anti-venom neurotoxin [Cherdchu and Karlsson, 1983] [vit]; reduced PGE2 [He et al., 2016] [a/cell line mouse microglia]
Cuscuta americana L.	No records found *Cuscuta* sp. [Luteolin] reduced inflammation and axonal damage [Hendriks et al., 2004] [a/vivo MS mouse]
Cuscuta australis R. Br.	No records found
Cuscuta chinensis Lam.	Anti-inflammatory [Liao JC et al., 2014] [a/vivo mouse]; memory improvement [Lin MK et al., 2018]; wound healing [Kim HJ et al., 2019] [a/vivo mouse]
Cuscuta reflexa Roxb.	No records found
Cyanthillium cinereum (L.) H.Rob.	Anti-bacterial [Tantengco et al., 2016] [vit]
Cyanthillium patulum (Dryand. ex Dryand.) H.Rob. Syn: *Vernonia patula* (Dryand.) Merr.	Anti-inflammatory [Hira et al., 2013] [a/vivo rat, vit]
Cycas sp.	No records found
Cyathula prostrata (L.) Blume	Anti-inflammatory [Ibrahim et al., 2012] [a/vivo rat]
Cyclanthera pedata (L.) Schrad.	Anti-inflammatory, anti-oxidant [Rivas et al., 2013] [vit]
Cyclea hypoglauca (Schauer) Diels	No records found
Cyclea barbata Miers	Anti-inflammatory [Santi et al. 2017] [a/vivo rat]
Cyclea peltata (Lam.) J. Hooker & Thoms.	Anti-bacterial [Abraham and Thomas, 2012] [vit]
Cydonia oblonga Mill.	Anti-bacterial [Fattouch et al., 2007] [vit]
Cymbopogon bombycinus (R.Br.) Domin	No records found
Cymbopogon citratus (DC.) Stapf. Syn: *Andropogon citratus* DC.	Anti-bacterial [Cimanga et al., 2002] [vit]; anti-viral [HIV] [Feriotto et al., 2018] [vit]; anti-oxidant [Shan et al., 2005] [vit]; anti-inflammatory [Mediesse et al., 2018] [a/cell line mouse]; anti-hypertensive [Carbajal et al., 1989] [a/vivo rat]; anxiolytic and anti-convulsant [Blanco et al., 2009] [a/vivo mouse]; vasorelaxant [Simões DM et al., 2020] [h/isolated artery]
Cymbopogon densiflorus (Steud.) Stapf	Anti-bacterial [Takaisi-Kikuni et al., 2000] [vit]
Cymbopogon giganteus Chiov	Anti-inflammatory [Sahouo et al., 2003] [vit]
Cymbopogon nardus (L.) Rendle	Anti-fungal [Nakahara et al., 2013] [vit]; anti-inflammatory, wound healing [Kandimalla et al., 2016] [a/vivo mouse]
Cymbopogon obtectus S.T.Blake	No records found
Cymbopogon proximus (Hochst. ex A. Rich) Stapf. Syn: *Cymbopogon schoenanthus* (L.) Spreng. subsp. *proximum* (Hochst. ex A. Rich.) Maire & Weiller	Anti-venom [Molander et al., 2014] [vit]
Cynanchum lancifolium Hook. and Am.	No records found
Cynanchum vanlessenii (Lavranos) Goyder	No records found
Cynanchum viminale subsp. *Stipitaceum* (Forssk.) Meve & Liede	Anti-inflammatory [Safari et al., 2016] [a/vivo mouse]

Cynara cardunculus L.	Anti-inflammatory [Tavares et al., 2013] [a/vivo mouse]
Cynara scolymus L.	Anti- atherosclerotic, anti-oxidant [Bogavac-Stanojevic et al., 2018] [a/vivo rat]
Cynodon dactylon (L.) Pers.	Anti-inflammatory [Garg and Paliwal, 2011a] [a/vivo rat]
Cyperus rotundus L.	Anti-bacterial [Parekh and Chanda, 2006] [vit]; anti-viral [Parvez et al., 2019] [h/cell line]; anti-amyloidogenic [Lee JE et al., 2017] [vit];
Cyphomandra betacea (Cav.) Sendtn. [unresolved]	Cholesterol-reducing, anti-adipogenic [Kadir et al., 2015] [a/vivo rat]
Cyphostemma adenocaule (Steud. ex A.Rich.) Desc. ex Wild & R.B.Drumm.	No records found
Cyrtomium fortunei J. Sm.	Anti-viral [Coxsackie virus, polio] [Guo JP et al., 2006] [vit]
Dacryodes edulis (G.Don) H.J.Lam	Anti-bacterial [Ajibesin et al., 2011] [vit]
Dactylorhiza hatagirea D.Don	Anti-inflammatory [Sirohi, 2019] [a/vivo rat]
Dalbergia lactea (Roxb) Vatke.	No records found
Daniellia oliveri (Rolfe) Hutch. & Dalziel	Anti-inflammatory [Jegede et al., 2006] [a/vivo mouse, rats]
Daphne gnidium L.	Anti-viral [Vidal et al., 2012] [vit]<br>* toxicity report [Bnouham et al., 2006]
Daphne mucronata Royle	Anti-bacterial [Javidnia et al., 2003] [vit]
Daphniphyllum himalense (Benth.) Mull. Arg.	Anti-bacterial, anti-oxidant [Majumdar and Roy, 2012] [vit]
Dasymaschalon longiflorum (Roxb.) Finet & Gagnep	No records found
Datisca glomerata (C.Presl) Baill	No records found
Daucus montanus Humb. & Bonpl. ex Schult.	No records found<br>*Daucus carrota* anti-oxidant [Kammerer and Schieber, 2004] [vit]
Davilla rugosa Poir.	Anti-bacterial [Roumy et al., 2015] [vit]
Descurainia sophia (L.) Webb ex Prantl	Anti-inflammatory [Mohamed et al., 2009] [a/vivo rat]
Desmodium gangeticum (L.) DC.	Memory improvement [Mahajan K et al., 2015] [a/vivo mouse]; anti-inflammatory [Govindarajan et al., 2007] [a/vivo rat]
Desmodium heterocarpon (L.) DC.	Mild anti-bacterial, antioxidant [Al Hasan et al., 2011] [vit]
Desmodium incanum (Sw.) DC. Syns: *D. mauritianum* (Willd.) DC., *D. canum* (J.F.Gmel.) Schinz & Thell.	Anti-bacterial [Delle Monache et al., 1996; Ezike et al., 2014] [vit]
Desmodium molliculum (H.B.K.) DC.	Anti-inflammatory [Lozano N et al., 20110] [a/vivo mouse]
Desmodium velutinum Syn: *D. lasiocarpum* (P.Beauv.) DC.	Anti-bacterial [Fomogne-Fodjo et al., 2014] [vit]
Detarium microcarpum Guill. & Perr.	Anti-viral [Olugbuyiro, 2009] [vit]
Deverra denudata (Viv.) Pfisterer & Podlech Syn: *Pituranthos chloranthus* (Coss. & Durieu) Schinz	Anti-inflammatory [Ben Nasr et al., 2020] [vit]
Deverra scoparia Coss. & Durieu *Pituranthos scoparius* (Coss. & Durieu) Schinz	Anti-bacterial [Boutaghane et al., 2004] [vit]
Dialium pachyphyllum Harms	No records found
Dichaea muricata (Sw.) Lindl.	No records found
Dichrocephala integrifolia (Linn.f.) Kuntze	Anti-bacterial [Mothana et al., 2009] [vit]

Dichrostachys cinerea (L.) Wight & Arn.	Anti-inflammatory [Hassan HS et al., 2012] [a/vivo rat]
Dicliptera bupleuroides Nees	Anti-bacterial [Riaz et al., 2019] [vit]
Dicliptera chinensis (L.) Juss.	No records found
Dictamnus albus L.	No records found
Diervilla lonicera Mill.	No records found
Digitaria abyssinica (Hochst. ex A.Rich.) Stapf	No records found
Digitaria debilis (Desf.) Willd.	No records found
Digitaria insularis (L.) Mez ex Ekman	No records found
Dillenia excelsa (Jack) Martelli ex Gilg.	Anti-bacterial [Abdulah et al., 2017] [vit]
Dillenia indica L.	Anti-bacterial, anti-fungal [Apu et al., 2010] [vit]
Dillenia pentagyna Roxb.	Anti-inflammatory [Puia and Kakoti, 2017] [a/vivo rat]
Dilodendron bipinnatum Radlk.	Anti-inflammatory [de Oliveira et al., 2017] [a/cell line mouse]
Dimorphandra mollis Benth.	No records found
Dioscorea alata L.	Anti-inflammatory [Chen T et al., 2017] [a/vivo mouse]; anti-hypertensive [Lin CL et al., 2006] [a/vivo rat]
Dioscorea bulbifera L.	Anti-viral [HIV] [Chaniad et al., 2016] [vit]; anti-venom [Molander et al. 2014] [vit]
Dioscorea communis (L.) Caddick & Wilkin Syn: *Tamus communis* L.	Anti-inflammatiory [Kupeli et al., 2007] [a/vivo mouse]
Dioscorea deltoidea Wall. ex Kunth	No records found<br>*Dioscorea japonica* induces NGF [Kim KH et al., 2011] [a/cell line rat]; induces neurite outgrowth, anti-neuro-inflammatory, neuroprotective [Woo et al., 2014] [a/cell line mouse microglia]
Dioscorea hispida Dennst.	No records found
Dioscorea mangenotiana Meige.	No records found
Dioscorea tambillensis Kunth	No records found
Dioscorea trifida L.f.	No records found
Diospyros hispida A.DC	No records found
Diospyros melanoxylon Roxb.	Anti-bacterial, anti-fungal [Rath SK et al., 2009] [vit]<br>*D. leucomelas* anti-inflammatory [del Carmen Recio et al., 2012] [a/vivo mouse]
Diospyros mespiliformis Hochst. ex A.DC.	Anti-inflammatory [Adzu et al., 2002] [a/vivo rat]
Diplostephium gynoxyoides Cuatr.	No records found
Dipteryx alata Vogel	Anti-paralytic vs neurotoxic venoms [Ferraz et al., 2014] [a/isolated phrenic nerve-diaphragm mouse]; anti-hyperlipidemic [Fiorini et al., 2017] [a/vivo rat]; anti-oxidant [de Almeida EM et al., 2012] [a/vivo rat]
Dirca palustris L.	Anti-oxidant [Ramsewak et al., 2001] [vit]
Discaria americana Gillies. & Hook.	Anxiolytic [Silva ER et al., 2012] [a/vivo mouse]
Donax canniformis (G.Forst.) K.Schum.	No records found
Dovyalis abyssinica (A.Rich.) Warb.	Anti-bacterial, anti-fungal [Geyid et al., 2005] [vit]
Dovyalis macrocalyx (Oliv.) Warb.	No records found
Dracaena spicata Roxb.	Anti-bacterial [Sharmin et al., 2014] [vit]
Dracaena steudneri Engl.	No records found
Dracocephalum stamineum Kar. & Kir.	No records found
Dracontomelon dao (Blanco) Merr. & Rolfe	Anti-bacterial, anti-fungal [Khan MR and Omoloso, 2002] [vit]
Dregea volubilis (L.f.) Benth. ex Hook.f.	No records found

Drymis winteri J.R.Forst. & G.Forst.	Anti-virus [HSV] [Pacheco et al., 1993] [a/cell line monkey]
Drymonia serrulata (Jacq.) Mart.	No records found
Drynaria quercifolia (L.) J. Sm.	Anti-bacterial [Kandhasamy et al., 2008] [vit]
Duchesnea indica (Jacks.) Focke	Anti-inflammatory [Zhao L et al., 2008] [a/cell line mouse]
Duguetia furfuracea (A.St.-Hil.) Saff.	Anti-inflammatory, anti-oxidant [do Santos et al., 2018] [vit, a/vivo mouse]
Durio zibethinus L.	Anti-bacterial [Lipipun et al., 2002] [vit]; anti-inflammatory [Chingsuwanrote et al., 2016] [h/cell line]
Dysosma versipellis (Hance) M.Cheng	Anti-viral [CBV, HSV] [Liyun and Liping, 1999] [vit]
Dysphania ambrosioides (L.) Mosyakin & Clemants Syn: *Chenopodium ambrosioides* L.	Anti-fungal [Kumar R et al., 2007] [vit]; anti-bacterial [Lall and Meyer, 1999] [vit]; anti-viral [Coxsackie B] [Mokni et al., 2019] [vit]; anti-inflammatory [Pereira WS et al., 2018] [a/vivo mouse]; anti-hypertensive [Assaidi et al., 2014] [a/vivo rat]
Dysphania schraderiana (Schult.) Mosyakin & Clemants	No records found
Echeveria peruviana Meyen	Anti-inflammatory [Vera Abanto and Zavaleta Minchola, 2019] [h/cell line]
Echinodorus scaber Rataj	No records found
Echinops spinosissimus subsp. bovei (Boiss.) Greuter	Anti-inflammatory [Rimbau et al., 1999] [a/vivo rat, mouse]
Ehretia cymosa Thonning	Anti-oxidant, anti-bacterial [Sarkodie et al., 2015] [vit]
Elaeagnus angustifolia L.	Anti-bacterial, anti-fungal [Khan SU et al., 2016] [vit]
Elaeagnus umbellata Thunb.	Anti-bacterial [Sabir et al., 2007] [vit]; anti-inflammatory [Yang et al., 2009] [a/cell line mouse]
Elaeis guineensis Jacq.	Anti-amyloid [Weinberg et al., 2018] [vit]; improved learning and cognitive ability, upregulation of neurogenic + neurotrophic genes, neuroprotective, anti-inflammatory [Leow et al., 2013] [a/vivo mouse]; anti-hypertensive [Jaffri et al., 2011] [a/vivo rat]; anti-convulsant [Alaribe et al., 2016] [a/vivo rat]
Elaeocarpus subserratus Baker	No records found
Elephantopus scaber L.	Anti-bacterial [Prusti, 2008] [vit]
Elettaria cardamomum (L.) Maton	Anti-inflammatory [Souissi et al., 2020] [h/cell line]; neuroprotective, anti-oxidant, AChE inhibition [Chowdhury S and Kumar, 2020] [a/cell line rat, vit]
Eleusine indica (L.) Gaertn.	Anti-bacterial, anti-oxidant [Al-Zubairi et al., 2011] [vit]; anti-inflammatory [Sagnia et al., 2014] [vit, h/cell line]
Eleutherine bulbosa (Mill.) Urb. Syns: *Eleutherine plicata* (Sw.) Herb.; *Eleutherine americana* (Aubl.) Merr. ex K.Heyne	Anti-viral [HIV] [Hara et al., 1997] [vit]; anti-bacterial [Padhi and Panda, 2015] [vit]; anti-inflammatory [Song SH et al., 2009 [a/cell line mouse]
Elionurus sp.	*Elionurus tristis* anti-bacterial [Yedomon et al., 2017] [vit]
Elsholtzia blanda (Benth.) Benth.	Reduced ischaemia [Haiyun et al., 2004] [a/vivo canine]; [*Elsholtzia rugulosa* anti-viral [influenza] [Liu AL et al., 2008] [vit; anti-amyloidogenic [Zhao et al., 2013] [a/vivo mouse]
Embelia ribes Burm. f.	Anti-viral [influenza] [Hossan et al., 2018] [a/cell line dog(MDCK)]; memory improvement, raised BDNF [Bhuvanendran et al., 2018] [a/vivo rat]; neuroprotective, anti-HD [Dhadde et al., 2016] [a/vivo HD rat]; anti-inflammatory [Mahendran et al., 2011] [a/vivo rat]
Embothrium coccineum J.R.Forst. & G.Forst.	No records found
Emilia abyssinica (Sch.Bip. ex A.Rich.) C.Jeffrey Syn*: Senecio abyssinicus* Sch.Bip. ex Hochst.	AChE inhibition, anti-oxidant [Odubanjo et al., 2018b] [vit]
Emilia coccinea (Sims) G.Don	Anti-bacterial [Teke et al., 2007] [vit]; neuroprotective, memory improvement [Simplice et al., 2014] [a/vivo rat]
Emilia fosbergii Nicolson	No records found
Emilia sonchifolia DC.	Anti-bacterial [Nwadinigwe, 2009] [vit]

Enicostema axillare (Poir. ex Lam.) A.Raynal	Anti-inflammatory [Leelaprakash G, Dass, 2011] [h/cell line]
Enicostema verticillatum (L.) Engl.	No records found
Entada abyssinica Steud. ex A.Rich.	Anti-bacterial [Dzoyem et al., 2017] [vit]
Entada pursaetha DC.	Anti-inflammatory [Kalpanadevi et al., 2012] [a/vivo rat]; wound healing [Vidya et al., 2012a] [a/vivo rat]; anti-bacterial [Vidya et al., 2012b] [vit]
Entandrophragma utile (Dawe& Sprague) Sprague.	Anti-ulcerogenic [John and Onabanjo, 2010] [a/vivo rat, a/isolated ileum guinea pig]
Ephedra altissima Desf.	No records found
Ephedra americana Humb. & Bonpl. ex Willd.	Anti-microbial [Bussmann et al., 2008] [vit]
Ephedra gerardiana Wall. ex Klotzsch & Garcke	Anti-bacterial, anti-oxidant [Khan A et al., 2017] [vit]
Ephedra viridis Coville	No records found
Epidendrum difforme Jacq.	No records found
Epidendrum sp.	No records found
Equisetum bogotense (H.B.K.)	No records found<br>*Equisetum arvanse* anti-inflammatory [Do Monte et al., 2004] [a/vivo mouse]
Equisetum giganteum (Wedd.)	Anti-inflammatory, immunomodulatory [Farinon et al., 2013] [a/vivo mouse]
*Equisetum hyemal*e L.	Anti-inflammatory [Park EY and Jeon, 2008] [a/cell line mouse]
Erechtites hieracifolia (L.) Raf. ex DC. var. *cacalioides]* (Fisch. ex Spreng.) Griseb	Anti-oxidant [Srianta et al., 2012] [vit]; anti-inflammatory [Epifano et al., 2014] [a/vivo mouse]
Eremophila alternifolia R.Br.	Anti-bacterial [Palombo and Semple, 2001] [vit]
Eremophila bignoniiflora (Benth.) F.Muell.	Anti-microbial [Sadgrove et al., 2013] [vit]
Eremophila duttonii F.Muell.	Anti-bacterial [Palombo and Semple, 2001] [vit]
Eremophila latrobei F. Muell. subsp. *glabra* (L.S. Smith) Chinn.	Anti-bacterial [Palombo and Semple, 2001] [vit]; anti-viral [Ross river virus] [Semple et al., 1998] [a/cell line hamster]
Erica arborea L.	
Eriobotrya japonica (Thunb.) Lindl.	Anti-bacterial, anti-oxidant [Lee KI et al., 2009] [vit]; reduced cholesterol [Kim AR et al., 2011] [a/vivo rat]; reversal of memory impairment, reduced ROS, anti-apoptotic [Kim MJ et al., 2011] [a/vivo mouse]; anti-inflammatory [Kuraoka-Oliveira et al., 2020] [a/vivo mouse]
Eriodictyon crassifolium Benth.	Anti-neuroinflammatory, neuroprotective [Maher et al., 2020] [a/cell line mouse neuron]
Eriogonum fasciculatum Benth.	No records found
Eriosema psoraleoides (Lam.) G.Don	Moderately anti-bacterial and modest anti-fungal [Khan MN et al., 2000] [vit]
Erodium cicutarium (L.) L'Herit.	Anti-bacterial, anti-fungal [Stojanović-Radić et al., 2010] [vit]
Erodium moschatum (L.) L'Hér.	No records found
Eryngium caeruleum M. Bieb.	Anti-bacterial [Dehghanzadeh et al., 2014] [vit]
Eryngium creticum Lam.	Anti-inflammatory [Küpeli et al., 2006] [a/vivo mouse]
Eryngium foetidum L.	Anti-inflammatory [Dawilai et al., 2013] [vit]; anti-microbial [Lingaraju DP et al., 2016] [vit]
Erythrina abyssinica Lam. ex DC.	Anti-viral [HIV] [Ali et al., 2002] [vit]; radical-scavenging [Yenesew et al., 2009] [vit]; *E. mildbraedii* anti-inflammatory [Njamen et al., 2003] [a/vivo mouse, a/cell line rat]
Erythrina dominguezii Hassl.	No records found
Erythrina edulis Triana ex Micheli	No records found
Erythrina senegalensis A. DC.	Anti-viral [HIV] [Lee JS et al., 2009] [vit]; anti-inflammatory [Saidu et al., 2000] [a/vivo rat]; anti-bacterial [Koné et al., 2004] [vit]

Erythrina stricta Roxb.	Anti-inflammatory [Subhashini N et al., 2011] [a/vivo rat]
Erythrina variegata L.	Anti-bacterial [Tanaka H et al., 2002] [vit]; anti-inflammatory [Mantena and Tejaswini, 2015] [a/vivo rat]
Erythrina verna Vell.	No records found
Erythrochiton fallax Kallunki	No records found
Erythroxylum coca Lam.	No records found
Escallonia pendula (Ruiz & Pav.) Pers.	Anti-bacterial [Bussmann et al., 2008] [vit]
Eucalyptus camaldulensis Dehnh	Anti-bacterial [Cimanga et al., 2002] [vit]; anti-inflammatory [Olawore and Ololade, 2017] [a/vivo rat]
Eucalyptus citriodora Hook.	Anti-bacterial [Cimanga et al., 2002] [vit]; anti-inflammatory [Silva J. et al., 2003] [a/vivo rat]
Eucalyptus globulus Labill.	Anti-inflammatory [Silva J. et al., 2003] [a/vivo rat]
Eucalyptus pruinosa Schauer	Anti-bacterial [Siddique et al., 2018] [vit]
Eucalyptus robusta Smith	Anti-bacterial [Cimanga et al., 2002] [vit]
Eucalyptus tetrodonta F.Muell.	No records found
Euclea racemosa subsp. *schimperi* (A. DC.) F. White	Anti-bacterial [Gebremariam et al., 2015] [vit]<br>*Euclea divinorum* anti-periodontopathic [Homer et al., 1990][vit]
Eucommia ulmoides Oliv.	Anti-viral [influenza] [Li J et al., 2019] [h/cell line, a/cell line canine]; increased Hsp 27 and Hsp70 [Nam et al., 2013] [h/cell line]; autophagy induction [Zhang S et al., 2020] [zebrafish PD model]
Eugenia dysenterica DC.	Wound healing, anti-inflammatory [Mazutti da Silva et al., 2019] [a/cell line mouse]; neuroprotective, anti-oxidant [Thomaz DV et al., 2018] [vit, a/vivo mouse]
Eugenia obtusifolia Cambes.	No records found
Eugenia pitanga (O.Berg) Nied.	Anti-bacterial [Alves et al., 2000] [vit]
Eugenia uniflora L.	Antioxidant, antibacterial, antifungal [Victoria et al., 2012] [vit]
Eupatorium cannabinum L.	Anti-bacterial [Senatore et al., 2001 [vit]
Euphorbia abyssinica J.F.Gmel.	Anti-fungal [El-Fiky et al., 2008] [vit]; anti-bacterial [Tarh and Iroegbu, 2017] [vit]
Euphorbia cactus Ehrenb. ex Boiss	No records found
Euphorbia calyptrata Coss. & Kralik	No records found
Euphorbia dendroides L.	Anti-viral [Chikungunya Virus] [Nothias-Scaglia et al., 2015] [a/cell line monkey]
Euphorbia glyptosperma Engelm.	No records found
Euphorbia granulata Forssk.	Anti-bacterial, anti-fungal [Awaad et al., 2017] [vit]
Euphorbia helioscopia L.	Anti-bacterial, anti-fungal [Awaad et al., 2017] [vit]
Euphorbia hirta L. Syn: *Chamaesyce hirta* (L.) Millsp.	Anti-viral [HIV [Gyuris et al., 2009] [h/cell line]; anti-bacterial, anti-fungal [Awaad et al., 2017] [vit; anti-venom [Gopi et al., 2016] [a/vivo mouse, vit]; wound healing [Rathnakumar et al., 2013] [a/vivo rat]; anti-convulsant [Bum et al., 2011] [a/vivo mouse]
Euphorbia hypericifolia L. Syn: *Euphorbia glomerifera*	No records found
Euphorbia kamerunica Pax	Anti-bacterial [Ogunnusi et al., 2010] [vit]
Euphorbia larica Boiss.	No records found
Euphorbia mellifera Aiton Syn: *Euphorbia longifolia*	Anti-fungal [Nim et al. 2016] [vit]
*Euphorbia monocyathium (*Prokh.) Prokh.	No records found
Euphorbia pallens Dillwyn	No records found
Euphorbia prostrata Aiton	Anti-inflammatory [Singla and Pathak, 1990] [a/vivo mouse]

Euphorbia retusa Forssk. Syn: *Euphorbia cornuta* Pers	Anti-inflammatory [Sdayria et al., 2018] [a/vivo mouse]
Euphorbia sikkimensis Boiss.Syn: *Euphorbia chrysocoma*	No records found
Euphorbia terracina L.	Moderately anti-bacterial and anti-fungal [Abdallah, 2014] [vit]
Euphorbia tirucalli L.	Anti-viral [HIV] [Abreu et al., 2014] [h/cell lines]; anti-inflammatory [Passos et al., 2013] [a/vivo mouse]
Euphorbia tithymaloides L. Syn: *Pedilanthus tithymaloides* (L.) A.Poit.	Anti-inflammatory [Abreu et al., 2006] [a/vivo rat]; wound healing [Ghosh S et al., 2012] [a/vivo rat]; anti-bacterial [Chaudhari et al., 2012] [vit]
Euphorbia trigona Mill.	No records found
Eurya sp.	*Eurya japonica* Anti-oxidant [Rosalind et al., 2013] [vit]
Eustephia coccinea Cav.	No records found
Evodia lepta Merr. [unresolved]	Anti- inflammatory [Yoon et al., 2013] [a/vivo mouse]
Evolvulus alsinoides L.	Memory improvement, neuroprotection, AChE inhibition, anti-inflammatory [5-LOX inhibition] [Sethiya et al., 2019] [vit, a/cell line mouse neuron, a/vivo rat]; anti-stress [Gupta P et al., 2013] [vit, a/vivo] [vit, a/vivo]
Evolvulus nummularius (L) L.	Anti-bacterial [Pavithra PS et al., 2009] [vit]
Eysenhardtia texana Scheele	Anti-bacterial, anti-fungal [Wächter et al., 1999] [vit]
Fagara davyi Verdoorn [unresolved]	No records found
Fagonia bruguieri DC.	Anti-fungal [Saleem R et al., 2019] [vit]
Fagonia paulayana J.Wagner & Vierh. Syn: *Fagonia schweinfurthii* (Hadidi) Nabil & Hadidi	No records found
Fagopyrum esculentum Moench	Anti-bacterial [Čabarkapa et al., 2008] [vit]; anti-Gaucher disease [enhanced GCase activity] [Khanna et al., 2010] [h/cell line Gaucher patient-derived] [Sun et al., 2011] [a/vivo mouse]; anti- α-synucleinopathic [Caruana et al., 2011] [vit]; anti-inflammatory [Ishii et al., 2008] [a/vivo mouse]; anti-hypertensive, anti-dyslipidaemic [Zhang HW et al., 2007] [h/c]; reduced DNA damage [Vogrinčič et al., 2013] [h/cell line]; memory improvement [Koda et al., 2008] [a/vivo rat]
Faidherbia albida (Delile) A.Chev. Syn: *Acacia albida* Delile	Anti-bacterial, anti-inflammatory [Eldeen et al., 2005] [vit]
Faurea saligna Mutango Harvey	No records found
Ferula communis L.	Anti-hypertensive [Ghanbari et al., 2012] [a/vivo rat]
Ferula kokanica Regel & Schmalh.	No records found
Fibraurea recisa Pierre	Anti-neuroinflammatory, anti-amyloidogenic, anti-tau, anti-apoptotic, [Xing Z et al., 2018] [a/vivo mouse, vit]
Ficus americana subsp. *guianensis* (Desv. ex Ham.) C.C.Berg Syn*: Ficus guianensis*	No records found
Ficus benghalensis L.	Wound healing [Garg and Paliwal, 2011b] [a/vivo rat]; anti-bacterial [Murti and Kumar , 2011] [vit]; anti-inflammatory [Mahajan MS et al., 2012] [a/vivo rat]
Ficus carica L.	Anti-bacterial [Duman et al., 2018] [vit]; anti-viral [HSV] [Ay and Duran, 2018] [h/cell line], wart [Bohlooli et al., 2007] [h/c]; memory improvement [Subash et al., 2016] [a/vivo mouse]; anti-inflammatory, anti-oxidant [Ali et al., 2012] [a/vivo rat]; anti-hypertensive [Alamgeer et al., 2017] [a/vivo rat]; anti-hyperlipidemic [Joerin et al., 2014] [a/vivo rat]; anti-platelet aggregation [Gilani et al., 2008] [h/cell line platelet]; AChE inhibition [Orhan et al., 2011] [vit]
Ficus cordata Thunb.	Anti-bacterial, anti-fungal [Kuete et al., 2008] [vit]; *Ficus bengalensis* anti-inflammatory [Patil V et al., 2009] [a/vivo rat]
Ficus cyathistipula Warb.	No records found

Ficus exasperata Vahl.	Anti-inflammatory [Nworu et al., 2013] [a/vivo] rod; anti-oxidant, anti-bacterial [Taiwo BJ and Igbeneghu, 2014] [vit]
Ficus maxima Mill.	No records found
Ficus microcarpa L.f.	Anti-bacterial, anti-oxidant [Ao et al., 2008] [vit]; hypolipidemic [Awad et al., 2011] [a/vivo rat]
Ficus natalensis Hochst.	Anti-bacterial, anti-oxidant [Ajaib et al., 2016] [vit]
Ficus obliqua G.Forst	No records found
Ficus paraensis (Miq.) Miq.	No records found
Ficus platyphylla Del.	Reduced cognitive deficits and neuronal cell loss, anti-convulsant [Chindo et al., 2009, 2015] [a/vivo mouse]; anti-venom [Molander et al., 2014] [vit]; anti-inflammatory [Amos et al., 2002] [a/vivo rat/mouse]
Ficus prostrata (Wall. ex Miq.) Buch.-Ham. ex Miq.	No records found
Ficus pumila L.	Anti-inflammatory [Liao et al., 2012] [a/vivo mouse]
Ficus religiosa L.	Memory improvement, modulation of serotonergic neurotransmission [Kaur et al., 2010] [a/vivo mouse]; anti-bacterial [Nair and Chanda, 2007] [vit]; anti-inflammatory [Sreelekshmi, et al., 2007] [a/vivo rat]; wound healing [Roy et al., 2009] [a/vivo rat]
Ficus septica Burm.f.	Anti-bacterial, anti-fungal [Vital et al., 2010] [vit]
Ficus sp.	*Ficus deltoidea* anti-inflammatory [Zakaria et al., 2012] [a/vivo rat]
Ficus sur Forssk. Syn: *Ficus capensis* Thunb.	Anti-inflammatory; anti-bacterial [Eldeen et al., 2005] [vit]; anti-convulsant Ishola et al., 2013] [a/vivo mouse]
Ficus thonningii Blume	Anti-inflammatory [Otimenyin et al., 2004] [a/vivo]
Ficus vasta Forssk	Anti-bacterial [Pękala-Safińska et al., 2019] [vit]
Flaveria bidentis (L.) Kuntze	No records found
Fleroya stipulosa (DC.) Y.F.Deng Syn: *Hallea stipulosa* (DC.) J.-F.Leroy	No records found
Flourensia cernua DC.	Anti-bacterial [Molina-Salinas et al., 2006] [vit]
Flueggea virosa (Roxb. ex Willd.) Voigt Syn: *Securinega virosa* (Roxb. ex Willd.) Baill.	Anti-venom [Molander et al., 2014] [vit]
Foeniculum vulgare Mill.	Anti-inflammatory [Choi EM et al., 2004] [a/vivo rat]; anti-bacterial [Kaur and Arora, 2009] [vit]
Forsythia suspensa Thunb.) Vahl.	Anti-microbial, anti-oxidant [Qu H et al., 2008] [vit]; anti-inflammatory [Ozaki et al., 2000] [a/vivo rat]; anti-aging [LI XT et al., 2009] [a/vivo mouse]
Fragaria nubicola (Lindl. ex Hook.f.) Lacaita	No records found<br>*Fragaria × ananassa* inhibits amyloid aggregation and tau phosphorylation, neuroprotective [Ma et al., 2018] [a/cell line mouse microglia, vit]; cognitive improvement [Joseph et al., 1999] [a/vivo rat]
Fraxinus angustifolia Vahl Syn: *Fraxinus rotundifolia*	No records found
Fraxinus chinensis Roxb.	Anti-oxidant [Lee BC et al., 2007] [vit]
Fuchsia canescens Benth	No records found
Fuchsia hybrida hort. ex Siebert & Voss	No records found
Fuchsia magellanica Lam. (ind)	Anti-hypertensive [Schmeda-Hirschmann et al., 1992] [vit]
Fuerstia africana T. C. E. Fr.	Anti-bacterial [Ngeny et al., 2013] [vit]
Fumaria officinalis L	AChE and POP inhibition [Chlebek et al., 2016] [vit]
Fumaria parviflora Lam.	Anti-viral [HBV] [Arbab et al., 2017] [vit]
Furcraea cabuya Trel.	No records found
Furcraea foetida (L.) Haw.	No records found
Furcraea macrophylla Baker, Hook	No records found

Gaertnera paniculata Benth.	No records found
Gaiadendron punctatum (Ruiz & Pav.) G.Don	No records found
Galenia africana L.	No records found
Galium verum L.	Anti-oxidant [Lakić et al., 2010] [vit]
Galinsoga parviflora Cav.	Anti-inflammatory [Studzińska-Sroka et al., 2018] [h/cell line; anti-viral [HSV] [Simoes et al., 1999] [a/cell line monkey]
Gamochaeta americana (Mill.) Wedd.	Anti-bacterial [Tamariz-Angeles et al., 2018] [vit]
Ganophullum falcatum Blume	No records found
Garcinia cowa Roxb. ex Choisy	Anti-bacterial [Negi PS et al., 2008] [vit]
Garcinia mangostana L.	Anti-viral HIV [Chen SX et al.,1996] [vit]; anti-amyloidogenic [Wang Y et al., 2012] [a/cell line rat neuron]
Garcinia gummi-gutta (L.) Roxb.	No records found
Garcinia huillensis Welw.	No records found
Garcinia kola Heckel.	No records found
Garcinia pedunculata Roxb. ex Buch.-Ham.	Anti-bacterial [Negi PS et al., 2008] [vit]
Garcinia sopsopia (Buch.-Ham.) Mabb.	No records found
Garcinia xanthochymus Hook.f.	Neuronal growth stimulus [Chanmahasathien et al. 2003] [a/cell line rat neuron] anti-inflammatory [Pal et al. 2005] [a/vivo rat]
Gardenia jasminoides J.Ellis Syn: *Gardenia augusta* (L.) Merr.	Anti-oxidant [Debnath et al., 2011] [vit]; anti-hyperlipidemic [Lee IA et al., 2005] [a/vivo mouse]; anti-inflammatory [Hong and Yang, 2013] [cell lines]
Gardenia taitensis DC.	No records found
Gardenia ternifolia subsp. *jovis-tonantis* (Welw.) Verdc.	Anti-inflammatory [Larsen et al., 2015] [vit]
Genipa sp.	Anti-oxidant [Náthia-Neves et al., 2017] [vit]
Gentiana macrophylla Pall.	Anti-fungal [Tan RX et al., 1996] [vit]; anti-viral [HIV] [Xu H-X et al., 2016] [vit]
Gentianella bruneotricha (Gilg.) J.S. Pringle.	Anti-bacterial [Bussmann et a., 2010] [vit]
Gentianella graminea (H.B.K.) Fabris	No records found
Gentianella multicaulis (Gillies ex Griseb.) Fabris Syn: *Gentianella achalensis* (Hieron. ex Gilg.) T.N. Ho & S.W. Liu	No records found
Geranium core-core Steud. (ind)	No records found
Geranium rotundifolium L.	No records found
Geranium sesiliflorum Cavanilles	No records found
Gerbera gossypina (Royle) Beauverd	No records found
Gerbera piloselloides (L.) Cass.	No records found
Gilbertiodendron dewevrei (De Wild.) J.Léonard	No records found
Gladiolus italicus Mill	No records found
Gliricidia sepium (Jacq.) Walp.	Anti-bacterial [Akharaiyi et al., 2012] [vit]; anti-fungal [Rahalison et al., 1993] [vit]
Globularia alypum L	Anti-bacterial, wound healing, anti-oxidant, anti-inflammatory [Ghlissi et al., 2016] [a/vivo rat, vit]
Glochidion oblatum Hook.f.	No records found
Glycine max (L.) Merr.	Free radical-scavenging, iron chelation [Prakash et al., 2007] [vit]; anti-inflammatory [Lin and Wu, 2021] [a/cell line mouse]; oestrogenic [Lima et al., 2014] [h/c]; increased BDNF, improved memory [Pan et al., 2010] [a/vivo rat]
Glycyrrhiza glabra L.	Anti-viral [HIV] [Mori et al., 1990] [h/c]; [Hsp90 downregulation [Nourazarian et al., 2016] [h/cell line]; anti-inflammatory, anti-bacterial [Nirmala and Selvaraj, 2011] [a/vivo rat, vit]; anti-PD

	[Petramfar et al., 2010] [h/c]; *Glycyrrhiza inflata* anti-inflammatory [Cui et al., 2008] [a/vivo mouse]
Glycyrrhiza uralensis Fisch. ex DC.	Anti-viral [influenza] [Ko et al, 2006] [h/cell line]; protects neuronal mitochondria [Yang EJ et al., 2012] [a/cell line mouse neuron]
Glyphaea brevis (Spren) Monochino	Anti-bacterial, anti-inflammatory, antioxidant [Dickson et al., 2011] [a/vivo *Gallus*]; anti-convulsant [Ogbonnia et al., 2003] [a/vivo mouse]
Gnaphalium elegans Kunth	No records found
Gnetum leptostachyum Blume	No records found
Gomphrena globosa L.	Anti-bacterial, anti-fungal, anti-oxidant [Hamiduzzaman and Azam, 2012] [vit]
Gomphrena perennis L.	No records found
Gomphrena serrata L.	No records found
Gossypium barbadense L.	Anti-hypertensive [Hasrat et al., 2004] [a/vivo rat]; anti-bacterial and wound healing [Ikobi et al., 2012 [a/vivo rat]; *Gossypium herbaceum* anti-inflammatory [Reddy and Raju, 2018] [a/vivo rat], Ji et al., 2012 [a/vivo rat]
Gossypium hirsutum L.	Anti-inflammatory [Cao H and Sethumadhavan, 2020] [a/cell line mouse]
Gossypium spp.	Anti-microbial [Essien et al., 2012] [vit]
Goupia glabra Aubl.	No records found
Graptophyllum pictum (L.) Griff.	Anti-inflammatory [Ozaki et al., 1989] [a/vivo rat]
Greenwayodendron suaveolens (Engl. & Diels) Verdc. Syn: *Polyalthia suaveolens*	Anti-bacterial [Idu M et al., 2017] [vit]
Grewia erythraea Schweinf	No records found<br>*G. mollis* anti-venom [Molander et al., 2014] [vit]
Grewia retusifolia Kurz	No records found
Grindelia inuloides Willd.	No records found
Grindelia pulchella Dunal	No records found
Guarea pubescens (Rich.) A.Juss.	No records found
Guazuma ulmifolia Lam.	Anti-bacterial [De Lima et al., 2006] [vit]; anti-inflammatory [Berengue et al., 2007] [vit, a/vivo rat]
Guiera senegalensis J.F.Gmel.	Anti-viral [HBV] [Arbab et al., 2017] [vit]; anti-venom [Molander et al., 2014] [vit]
Guilandina bonduc L. Syns: *Caesalpinia bonducella* L. Fleming; *C. bonduc* (L.) Roxb.	Anti-inflammatory [Gupta M et al., 2003] [a/vivo rat]; *C. sappan* anti-amyloidogenic [Du et al., 2015] [h/cell line neuronal, vit]
Gunnera tinctoria (Molina) Mirb. Syn*: Gunnera chilensis* Lam.	Anti-inflammatory Rodríguez-Díaz et al., 2013] [vit]; anti-fungal, anti-oxidant [Zamorano et al., 2017] [vit]
Gymnema sylvestre (Retz.) R.Br. ex Sm.	Anti-venom [Kini and Gowda , 1982] [vit]; anti-bacterial, anti-fungal [Khanna and Kannabiran, 2008] [vit]; anti-inflammatory [Malik et al., 2008] [a/vivo rat]; hypolipidemic [Daisy et al., 2009] [a/vivo rat]; neuroprotective [Fatani et al., 2015] [a/vivo rat]
Gymnosporia senegalensis (Lam.) Loes Syn: *Maytenus senegalensis* (Lam.) Exell	Anti-inflammatory, anti-oxidant, anti-bacterial [Makgatho et al., 2018] [a/cell line mouse, vit]
Gynerium sagittatum (Aubl.) P.Beauv.	No records found
Gynostemma pentaphyllum (Thunb.) Makino	Anti-inflammatory [Aktan et al., 2003] [a/cell line mouse]; anti-viral [HIV] [Okoye EL et al., 2012] [h/cell line]; anti-fatigue [Qi B et al., 2014] [a/vivo mouse]
Gyrocarpus americanus Jacq.	No records found
Gynura procumbes Merr.	No records found
Gynura scandens O.Hoffm.	No records found
Habenaria sp.	Anti-oxidant, anti-microbial [Jagtap et al., 2014] [vit]
Hancornia speciosa Gomes	Anti-hypertensive [Silva et al., 2011] [a/vivo mouse]; anti-oxidant [Assumpção et al., 2014] [vit]; AChE inhibition [Penido AB et al.,

	2017] [vit]; anti-inflammatory [Marinho et al., 2011] [a/vivo mouse, rat]
Handroanthus impetiginosus (Mart. ex DC.) Mattos Syn: *Tabebuia avellanedae* Lorentz ex Griseb	Anti-ulcerogenic [Twardowschy et al., 2008] [a/vivo rat]
Haplophyllum tuberculatum Juss. Syn: *Ruta tuberculatum* Forsk.	Anti-bacterial, anti-inflammatory, anti-oxidant [Sabri et al., 2016] [a/vivo rat, vit]
Harpullia sp.	*H. ramiflora* anti-bacterial [Khan MR et al., 2001] [vit]
Harungana madagascariensis Lam. ex Poir.	Anti-bacterial [Okoli AS et al., 2002] [vit]; anti-inflammatory [Nwodo, 1989] [a/vivomouse]; anti-hypertensive, vasodilatory [Tom et al., 2018] [isolated rat aorta]
Hedychium spicatum Sm.	Anti-inflammatory [Ghildiyal et al., 2012] [a/vivo rat]
Heinsia crinita (Wennberg) G.Taylor	Neuroprotective, AChE inhibition [Oboh et a., 2016] [vit]; anti-bacterial, anti-fungal [Morah and Ashipu, 2017] [vit]
Helianthus annuus L.	Anti-hypertensive [Megías et al., 2004] [vit]; anti-inflammatory [Odabasoglu et al., 2008] [a/vivo rat]
Helichrysum krausii Sch. Bip.	No records found
Helichrysum mechowianum Klatt Syn: *Helichrysum ceres* S.Moore	Anti-bacterial [Malolo et al., 2015] [vit]; anti-hypertensive [Musabayane et al., 2008] [a/vivo rat]
Helichrysum schimperi (Sch.Bip. ex A.Rich.) Moeser	No records found
Heliconia psittacorum L. f.	No records found
Helicteres isora L.	Anti-inflammatory, anti-oxidant [Rattanamaneerusmee et al., 2018] [h/cell line, vit]
Helinus integrifolius (Lam.) Kuntze	Anti-bacterial [Shai et al., 2013] [vit]
Heliotropium bacciferum Forssk.	Anti-bacterial, anti-oxidant [Ahmad S et al., 2016] [vit]
Heliotropium cinerascens Steud. ex DC. [unresolved]	No records found
Heliotropium indicum L.	Anti-venom [Molander et al., 2014] [vit]; anti-bacterial [Boominathan and Ramamurthy, 2009] [vit]; wound healing [Reddy JS et al., 2002] [a/vivo rat]; thrombolytic [Samira et al., 2016][vit]
Heracleum sphondylium subsp. *montanum* (Schleich.ex Gaudin) Briq.	Vasorelaxant [Senejoux et al., 2013] [a/aortic ring rat]
Heracleum cachemiricum C.B. Clarke	No records found
Hernandia ovigera L.	Anti-inflammatory [Jang DS et al., 2004] [vit]
Heteropterys obovata (Small) Cuatrec. & Croat	No records found
Heteropterys tomentosa A.Juss.	Memory improvement [Galvão et al., 2011] [a/vivo rat]
Heterotis rotundifolia (Sm.) Jacq.-Fél. Syn: *Dissotis rotundifolia* (Sm.) Triana	Anti-bacterial [Dougnon et al., 2017] [vit]
Hibiscus acetosella Welw. ex Hiern	Anti- DNA damage [Vilela TC et al., 2018] [a/vivo mouse]
Hibiscus cannabinus L	Moderate anti-microbial [Zakaria et al., 2011] [vit]
Hibiscus fuscus Garcke	Moderately anti-viral [Cos et al., 2002a] [a/cell line monkey]
Hibiscus lunariifolius Willd	No records found
Hibiscus rosa-sinensis L.	Anti-bacterial [Ruban and Gajalakshmi, 2012] [vit]; anti-inflammatory [Kandhare et al., 2012] [a/vivo rat]
Hibiscus sabdariffa L.	Anti-hypertensive [Nwachukwu et al., 2015] [h/c]; anti-inflammatory [Zhen J et al., 2015] [vit]
Hibiscus tiliaceus L.	Anti-inflammatory [Narender et al., 2009] [a/vivo mouse]; anti-hyperlipidemia [Kumar S et al., 2010] [a/vivo rat]; wound healing [Sunilson et al., 2012] [a/vivo rat]
Himatanthus drasticus (Mart.) Plumel	Anti-bacterial [Figueiredo et al., 2017] [vit]

Himatanthus obovatus (Müll.Arg.) Woodson	No records found
Himatanthus sucuuba (Spruce ex Müll.Arg.) Woodson	Anti-bacterial [Neto et al., 2002] [vit]; wound healing [Calero-Armijos et al., 2010] [a/vivo mouse]
Hippocratea myriantha Oliv.	No records found
Hippophae rhamnoides L.	Reduced cognitive impairment, anti-oxidant [Attrey et al., 2012] [a/vivo rat]; restored mitochondrial integrity [Narayanan S. et al., 2005] [a/cell line rat glia]; anti-fatigue [Ni et al., 2013] [a/vivo mouse]
Hippophae salicifolia D.Don	Anti-bacterial, anti-fungal [Gupta SM et al., 2011] [vit]
Hippophae tibetana Schltdl.	No records found
Hippophae turkestanica (Rousi) Tzvelev	No records found
Holoptelea integrifolia (Roxb.) Planch.	Anti-inflammatory [Lalan et al., 2015] [a/vivo rodents]; anti-hyperlipidemia [Subash and Augustine, 2013] [a/vivo rat]
Hordeum vulgare L.	Anti-inflammatory, potentiates anti-oxidant defence system, anti-platelet aggregation [Gul et al., 2014] [h/cell line platelet]
Horkelia cuneata Lindl. Syn: *Horkelia californica* Cham. & Schltdl.	No records found
Hoya parasitica Wall. ex Traill [unresolved]	Anti-bacterial [Ahmed F et al., 2008] [vit]
Humata heterophylla (Sm.) Desv. Syn: *Davallia heterophylla* Sm.	No records found
Hydnora abyssinica A.Br.	No records found
Hydrocotyle globiflora R. & P.	No records found
Hygrophila auriculata (Schumach.) Heine	Anti-microbial [Doss and An, 2013] [vit]
Hymenaea courbaril L.	Anti-inflammatory [Bezerra et al., 2013] [a/vivo rat, rat tracheal muscle]; antifungal [da Costa et al., 2014] [vit]; anti-viral [Cecílio et al., 2012] [vit]
Hymenaea martiana Hayne	Anti-inflammatory [Neves et al., 1993] [a/vivo mouse]
Hymenaea parvifolia Huber	No records found
Hymenaea stigonocarpa Mart. ex Hayne	Anti-bacterial [Dimech et al., 2013]
Hymenocardia acida Tul.	ROS-scavenging [Sofidiya et al., 2006] [vit]; anti-inflammatory [Sofidiya et al., 2010] [a/vivo rat]; anti-bacterial [Muanza et al., 1994] [vit]; anti-hypertensive, vasorelaxant [Manga et al., 2013] isolated rat aorta]
Hymenocardia ulmoides Oliv.	No records found
Hypericum perforatum L.	Anti-inflammatory [Raso et al., 2010] [a/vivo mouse]
Hyptis suaveolens (L.) Poit.	Anti-inflammatory [Shenoy and Shirwaikar, 2002] [a/vivo rat]; anti-bacterial, anti-fungal [Mondal KC et al., 2007] [vit]
Hyptis verticillata Jacq.	Anti-inflammatory, anti-bacterial [Kuhnt et al., 1993] [vit]
Illicium verum Hook. f.	Anti-bacterial, anti-fungal [De M et al., 2002] [vit]; anti-inflammatory [Sung et al., 2012] [h/cell line]
Impatiens balsamina L.	Anti-microbial [Tailor et al., 1997] [vit]; anti-inflammatory [Oku and Ishiguro 2002] [vit]
Impatiens stuhlmannii Warb.	No records found
Imperata contracta (Humb., Bonpl. & Kunth) Hitchc.	No records found
Imperata cylindrica (L.) P.Beauv.	Anti-bacterial [Parkavi et al., 2012] [vit]; anti-inflammatory [Huo et al., 2017] [vit]; anti-hypertensive [Mak-Mensah et al., 2010] [a/vivo cat, rabbit]
Indigofera arrecta Hochst. ex A. Rich.	Anti-bacterial [Ngule and Ndiku, 2014] [vit]; anti-inflammatory [Suleiman et al., 2015] [a/vivo rat]
Indigofera oblongifolia Forssk	Anti-bacterial, anti-fungal [Dahot, 1999] [vit]
Indigofera spinosa Forssk	Anti-bacterial [Al-Fatimi et al., 2007] [vit]

Indigofera suffruticosa Mill.	Anti-bacterial [Leite et al., 2006] [vit]
Inga densiflora Benth.	No records found
Inga ruiziana G. Don.	No records found
Inga ynga (Vell.) J.W. Moore	No records found
Inula japonica Thunb.	Anti-inflammatory [Choi JH et al., 2010] [a/mouse cell line]
Inula orientalis Lam.	No records found
Ipomoea batatas (L.) Lam.	Anti-inflammatory [Chen H et al., 2019] [a/vivo, vit] mouse; anti-neuroinflammation [Li J et al., 2018] [a/vivo mouse];anti-apoptotic [Adnyana et al., 2018] [a/vivo rat]; anti-fatigue [Li and Zhang, 2013] [a/vivo mouse]; anti-hypertensive [Kobayashi et al., 2005] [a/vivo rat]
Ipomoea cairica (L.) Sweet	Anti-bacterial [Choudhury et al., 2015] [vit]; anti-viral [HIV] Schröder et al., 1990 [vit]
Ipomoea gracilis R.Br. [unresolved]	No records found
Ipomoea mauritania Jacq.	No records found
Ipomoea obscura (L.) Ker Gawl.	Anti-inflammatory [Hamsa and Kuttan, 2011] [cell line]
Ipomoea pes-caprae (L.) R.Br.	Anti-bacterial [Leach et al., 1988] [vit]; anti-venom, anti-inflammatory [Pongprayoon et al., 1991a,b] [a/vivo rat, a/cell line guinea pig + vit]; wound healing [Eakwaropas et al., 2020] [vit]
Iresine diffusa H.B.K. ex Willd. Syn: *Iresine celosia* L.	Anti-neuroinflammatory [Kim N et al., 2019] [microglial cell line, a/vivo mouse]
Iresine herbstii Lindley	Anti-bacterial [Chaudhuri and Sevanan, 2012] [vit]
Isatis raphanifolia Boiss.	No records found
Isatis tinctoria L. Syn: *Isatis indigotica* Fortune ex Lindl.	Anti-viral [influenza] [Yang Z. et al., 2013] [vit]; anti-inflammatory [Recio et al., 2006] [a/vivo mouse], Danz et al., 2001 [vit]
Isodon coesta (Buch.- Ham. ex D. Don)	No records found
Isodon ternifolius (D.Don) Kudô Syn: *Rabdosia ternifolia*	No records found
Ixora nigricans R.Br. ex Wight & Arn	Anti-inflammatory [Alam MN et al., 2015] [vit]
Ixora pavetta Andrews	Anti-inflammatory [Mondal S et al., 2014] [a/vivo rat]
Jacaranda caucana Pittier	No records found
Jacaranda copaia (Aubl.) D. Don.	No records found
Jacaranda cuspidifolia Mart.	Moderately anti-bacterial + anti-fungal [Yuan et al., 2018] [vit]
Jasminum grandiflorum L.	Improved motor deficits, neuroprotective [Alabi et al., 2019] [a/vivo rat PD model]
Jasminum multiflorum (Burm.f.) Andrews	No records found
Jasminum nervosum Lour.	Anti-inflammatory, anti-oxidant [Guo ZY et al., 2014] [a/cell line mouse microglia [vit]
Jasminum officinale L.	Anti-viral [Zhao G. et al., 2009] cell line, a/vivo duck]; anti-bacterial, anti-fungal Hussain M. et al., 2013] [vit]
Jasminum syringifolium Wall. & G.Don	No records found
Jatropha curcas L.	Anti-viral [HIV] [Matsuse et al., 1998] [h/cell line]; anti-venom [Molander et al., 2014] [vit]; anti-inflammatory [Mujumdar and Misar, 2004] [a/vivo mouse, rat]; anti-bacterial [Igbinosa et al., 2009] [vit]; anti-convulsant [Obi BC et al., 2019] [a/vivo mouse]; wound healing [Esimone et al., 2008] [a/vivo rat]
Jatropha elliptica (Pohl) Oken	Anti-bacterial [De Lima et al., 2006] [vit]; anti-inflammatory [Ferreira-Rodrigues et al., 2016] [a/vivo mouse]
Jatropha glandulifera Roxb.	Anti-bacterial [Srinivasan et al., 2001] [vit]
Jatropha gossypifolia L. var. elegans (Pohl) Müll.Arg	Anti-venom, anti-bacterial, anti-inflammatory [Félix-Silva et al., 2018] [a/vivo mouse, vit]; anti-inflammatory [Panda et al., 2009] [a/vivo rat]; [orientin] reduced cognitive deficits [Yu L, et al., 2015] [a/vivo mouse]
Jatropha pelargoniifolia Courbai	Anti-inflammatory [Aati et al., 2018] [a/vivo rat, vit]

Jatropha podagrica Hook.	Anti-inflammatory [Lin CH et al., 2019] [a/cell line mouse]; anti-bacterial [Aiyelaagbe et al.,2007] [vit]
Jatropha spinosa Vahl	Anti-bacterial [Al-hood and Ali, 2015] [vit]
Jodina rhombifolia (Hook. & Arn.) Reisseck	Anti-inflammatory [Teves et al., 2015] [a/vivo rat]
Juglans neotropica Diels	Anti-bacterial, anti-fungal [Lopez et al., 2001] [vit]
Juglans regia L. Syn: *Juglans sinensis*	Cognitive improvement [Arab and Ang, 2015] [h/c]; memory enhancement [Haider S et al., 2011a] [a/vivo rat]; anti- α-synucleinopathic [Caruana et al., 2011] [vit]; enhanced mitochondrial function, energy-boosting [Liu R et al., 2019] [a/vivo mouse]; anti-hypertensive [Joukar et al., 2017] [a/vivo rat]
Juniperus communis L.	Anti-microbial [Glišić et al., 2007] [vit]; anti-inflammatory [Bais et al., 2017] [a/vivo rat]; anti-aging [Pandey S et al., 2018] [a/vivo *C. elegans*]
Juniperus indica Bertol.	Anti-oxidant [Bais and Prashar, 2015] [vit]; slightly anti-microbial [Mahajan B et al., 2012] [vit]
Juniperus oxycedrus L.	Anti-inflammatory [Moreno et al., 1998] [a/vivo rat]; neuroprotective [Tavares L et al., 2012] [h/cell line]
Juniperus phoenicea L	Anti-inflammatory [Zouari Bouassida et al., 2018] [a/vivo mouse]
Juniperus recurva Buch.-Ham. ex D.Don	No records found
Justicia adhatoda L. Syns: *Adhatoda zeylanica* Medik, *Adhatoda vasica* Nees	Anti-inflammatory [Mulla et al., 2010]; [a/vivo rat]; anti-bacterial [Shinwari et al., 2009] [vit]; anti-venom [Malathi et al., 2019] [vit]; anti-viral [Chavan and Chowdhary, 2014] [vit]
Justicia chaetocephala (Mildbr.) Leonard	No records found
Justicia filibracteolata Lindau	No records found
Justicia gendarussa Burm.f. Syn: *Gendarussa vulgaris* Nees.	Anti-inflammatory [Kumar KS et al., 2018] [a/vivo rat]; anti-viral [HIV] [Zhang HJ et al., 2017] [h/cell line, vit]
Justicia pectoralis Jacq.	Anti-inflammatory [Lino et al., 1997[[a/vivo rat]; anti-bacterial [Chariandy et al., 1999] [vit]
Justicia schimperiana (Hochst. ex Nees) T. Anderson	Anti-bacterial, anti-fungal [Tesfaye, 2017] [vit]
Justicia secunda Vahl	Anti-hypertensive [Manda et al., 2011] [a/vivo rabbit]
Justicia xanthostachya Leonard	No records found
Kaempferia galanga L.	Anti-bacterial, anti-fungal [Tewtrakul et al., 2005] [vit]; anti-inflammatory [Sulaiman et al., 2008] [a/vivo mouse, rat]
Kaempferia sp.	*Kaempferia pandurata* anti-bacterial [Park KM et al., 2005; Sukandar et al., 2014] [vit]
Kageneckia lanceolata Ruiz & Pavon.	No records found
Kalanchoe crenata (Andrews) Haw.	Anti-inflammatory [Dimo et al., 2006] [a/vivo rat]; anti-bacterial [Akinsulire et al., 2010] [vit]
Kalanchoe gastonis-bonnieri Raym.-Hamet & H. Perrier	Anti-inflammatory [Costa et al., 2015] [vit]; anti-bacterial [Abdalla et al., 2017] [a/vivo dog]
Kalanchoe petitiana A. Rich.	Wound healing [Mekonnen et al., 2013] [a/vivo mouse]
Kalanchoe pinnata (Lam.) Pers Syn*: Bryophyllum pinnatum* (Lam.) Oken	Anti-inflammatory [Ferreira RT et al., 2018] [a/vivo mouse]; anti-viral [Jaeger Greer et al., 2010] [vit]; anti-venom [Fernandes et al., 2016] [a/vivo mouse]; anti-hypertensive [Bopda et al., 2014] [a/vivo rat]
Kalanchoe teretifolia Deflers	No records found
Kanahia laniflora (Forssk.) R.Br.	Anti-bacterial [Mothana et al., 2009] [vit]
Kedrostis foetidissima (Jacq.) Cogn.	Anti-bacterial [Raja et al., 2019] [vit]
Khaya anthotheca (Welw.) C.DC.	Anti-bacteria, anti-fungall [Suleiman et al., 2010] [vit]
Khaya ivorensis A.Chev.	Anti-inflammatory [Agbedahunsi et al., 2004] [a/vivo rat]
Kigelia africana (Lam.) Benth.	Anti-inflammatory [Owolabi et al., 2007] [a/vivo guinea pig, mouse]; anti-venom [Molander et al., 2014] [vit]; vasorelaxant [Isah et al., 2020] [a/isolated artery rat]; wound healing [Agyare et al., 2013] [a/vivo rat]

Kirkia acuminata Oliv.	Anti-bacterial [Masoko, 2013] [vit]; anti-inflammatory [Recio et al., 1995] [a/vivo mouse]
Kleinia longiflora DC. Syn: *Senecio longiflorus* (DC.) Sch.Bip.	Anti-bacterial [Asong et al., 2019] [vit]
Kleinia odora (Forssk.) DC.	No records found
Kleinia squarrosa Cufod.	No records found
Koanophyllon solidaginoides (Kunth) R.M. King & H. Rob.	No records found
Kohleria spicata (Kunth) Oerst.	No records found
Krameria lappacea (Dombey) Berdet & B. Simpson	Anti-inflammatory [Baumgartner et al., 2011] [a/vivo mouse]
Kyllinga brevifolia Rottb.	Anti-oxidant [Ho et al., 2012] [vit]
Kyllinga nemoralis (J.R.Forst. & G.Forst.) Dandy ex Hutch. & Dalziel	Anti-bacterial, anti-oxidant [Sindhu T et al., 2014] [vit]
Lablab purpureus (L.) Sweet Syn: *Dolichos lablab* L.	Anti-inflammatory, anti-oxidant [Momin et al., 2012] [vit]
Lactuca serriola L. Syn: *Lactuca scariola* L.	Anxiolytic [Ghazala et al., 2009] [h/c]; anti-venom [Bouimeja et al., 2019] [a/vivo mouse]
Lafoensia pacari A. St.-Hil.	Anti-bacterial [Pereira et al., 2011] [vit]; anti-depressant [Galdino et al., 2009] [a/vivo mouse]
Lagenaria siceraria (Molina) Standl.	Anti-hypertensive [Mali et al., 2012] [a/vivo rat]
Lagerstroemia speciosa (L.) Pers.	Anti-bacterial, anti-fungal [Pavithra et al., 2013] [vit]; anti-inflammatory, anti-oxidant [Priya et al., 2008] [a/vivo mouse]
Landolphia camptoloba (K.Schum.) Pichon	No records found
Lannea acida A. Rich. Syn: *Lannea microcarpa* Engl. & K.Krause	Anti-inflammatory [Picerno et al., 2006] [a/vivo mouse, vit]
Lannea antiscorbutica (Hiern) Engl.	No records found
Lannea coromandelica (Houtt.) Merr.	Anti-inflammatory [Singh and Singh, 1994] [a/vivo rat]
Lantana canescens Kunth	No records found
Lantana trifolia L.	Anti-venom [Molander et al., 2014] [vit]
Lapageria rosea Ruiz and Pav. (ind)	No records found
Laurelia sempervirens Ruiz and Pav. (ind)	Anti-bacterial [Montenegro et al., 2012] [vit]; anti-hypertensive [Schmeda-Hirschmann et al., 1994] [a/vivo rat]
Laurus nobilis L.	Anti-oxidant [Shan et al., 2005] [vit]; anti-viral [SARS-CoV] [Loizzo et al., 2008] [vit]
Lavandula stoechas L.	Anti-inflammatory [Amira et al., 2012] [a/vivo mouse]
Lawsonia inermis L.	Anti-bacterial [Malekzadeh, 1968] [vit]; memory improvement [Rajesh V et al., 2017] [a/vivo mouse]
Leea guineensis G.Don	Anti-bacterial, anti-fungal [Neji et al., 2016 [vit]; anti-convulsive [Wode et al., 2011] [a/vivo mouse]
Leea indica (Burm.f.) Merr.	Anti-inflammatory [Shah SK et al., 2018] [a/vivo]; reduced memory deficits, AChE inhibition [Chen Q et al., 2019] [a/vivo rat]
Lemna minuta Kunth	Anti- bacterial, anti-fungal [Velichkova et al., 2018]; *Lemna minor* immunomodulatory [Ovodova et al., 2000] [vit]
Lens culinaris Medik.	Anti-PD muscle rigidity effects [Houshmand et al., 2016] [a/vivo rat]
Leonotis nepetifolia (L.) R.Br.	Anti-inflammatory [weak activity] [Pushpan et al., 2017] [a/vivo rat]; anti-bacterial [Oliveira et al., 2015] [vit]; anti-viral [polio] [Vlietinck et al., 1995] [a/cell line monkey]
Leonurus cardiaca L.	Memory improvement, improved cognitive dysfunction [Liu C et al., 2016] [a/vivo rat]; promotes neurite outgrowth and neurotrophic activity [Meng P et al., 2019] [a/cell line rat neuron]; anti-hypertensive, anxiolytic [Shikov et al., 2011] [h/c]; anti-platelet

	aggregation [Sadowska et al., 2017] [h/cell line platelet]; anti-inflammatory [Liu Y et al., 2018] [h/cell line, a/cell line]
Leonurus sibiricus L.	Anti-atherogenic, cholesterol-reducing, reduced cell adhesion [Lee MJ et al., 2010a] [h/cell line vascular endothelium]; anti-inflammatory [Islam MA et al., 2005] [a/vivo mouse]
Lepidium bipinnatifidum Desv.	No records found
Lepidium draba L.	Anti-bacterial [Al-Marzoqi et al., 2015] [vit]; anti-inflammatory [Chyad, 2017] [a/vivo mouse]
Lepidium nitidum Nutt.	No records found
Lepidium sativum L.	Anti- bacterial, anti-fungal, anti-inflammatory, anti-oxidant [Alqahtani et al., 2019] [vit]; anti-hypertensive [Maghrani et al., 2005] [a/vivo rat]
Lepidium thurberi Wooton	No records found
Lepisanthes rubiginosa (Roxb.) Leenh.	Anti-bacterial [Mohamad et al., 2011] [vit]
Leptadenia pyrotechnica (Forssk.) Decne.	Anti-inflammatory [Alqasoumi et al., 2012] [a/vivo rat]; anti-bacterial [Munazir et al., 2012] [vit]
Leptochloa chinensis (L.) Nees.	No records found
Leucaena leucocephala (Lam.) de Wit	Anti-inflammatory [Dzoyem and Eloff, 2015] [a/cell line mouse, vit]
Leucas aspera (Willd.) Link	Anti-venom, anti-oxidant, anti-inflammatory [Sakthivel et al., 2013] [a/vivo mouse, vit]
Leucas cephalotes (Roth) Spreng.	Anti-inflammatory, anti-oxidant [Baburao et al., 2010] [a/vivo rat]; anti-bacterial [Rulhania et al., 2021] [vit]
Leucas decemdentata (Willd.) Sm.	No records found
Leucas martinicensis (Jacq.) R.Br.	Anti-inflammatory [Twilley et al., 2017] [vit]; anti-microbial [cough, cold]
Libertia ixioides (G.Forst.) Spreng.	No records found
Libidibia ferrea (Mart. ex Tul.) L.P.Queiroz Syn*: Caesalpinia ferrea*	Anti-bacterial, anti-fungal [Sampaio et al., 2009] [vit]
Ligaria cuneifolia (Ruiz ex Pavon) Thiegh.	No records found
Lindackeria laurina C.Presl	Anti-fungal [Rahalison et al., 1993] [vit]
Lindernia diffusa (L.) Wettst.	No records found
Linum bienne Mill. Syn*: Linum angustifolium*	No records found
Linum puberulum (Engelm.) Heller	No records found
Linum sativum L.	No records found
Linum selaginoides Lam. (ind)	No records found
Linum usitatissimum L.	Anti-inflammatory [Kaithwas et al., 2011] [a/vivo mouse]; anti-bacterial [Bakht et al., 2011] [vit]
Lippia alba (Mill.) N.E. Brown	Anti-viral [HSV, polio] [Andrighetti-Fröhner et al., 2005] [vit]; vasorelaxant [Maynard et al., 2011] [vit]; anti-inflammatory [Viana et al., 1998] [a/vivo rat]; anti-convulsant [Zétola et al., 2002] [a/vivo mouse]
Lippia integrifolia (Grieseb.) Hieron	Anti-inflammatory, anti-oxidant [Marcial et al., 2014] [cell line]
Lippia javanica (Burm.f.) Spreng.	Anti-fungal, anti-bacterial [Viljoen et al., 2005] [vit]; anti-inflammatory [Dzoyem and Eloff, 2015] [cell line]
Lippia multiflora Moldenke	Anti-hypertensive [Noamesi et al., 1985] [a/vivo cat, rat, rabbit]; Anti-fungal, anti-bacterial [Kunle et al., 2003] [vit]
Lithospermum erythrorhizon Siebold & Zucc.	Anti-viral [HIV] [Xu HX et al., 1996] [vit]
Litsea cubeba (Lour.) Pers. Litsea	Anti-inflammatory [Yang X et al., 2018] [a/vivo mouse, rat]
Litsea glutinosa (Lour.) C.B.Rob. Syn: *Litsea chinensis* Lam.	Anti-inflammatory, wound healing [Devi and Meera, 2010] [a/vivo rat]

Loeseneriella clematoides (Loes.) R.Wilczek	No records found
Lomatia ferruginea R.Br. (int)	No records found
Lomatia hirsuta (Lam.) Diels	Anti-inflammatory [Erazo et al., 1997a] [a/vivo guinea pig]
Lomatium californicum (Nutt.) Mathias & Constance	Anti-bacterial [Chou SC et al., 2006] [vit]
Lonicera involucrata (Richardson) Banks ex Spreng.	Anti-bacterial [McCutcheon et al., 1992] [vit]; *Lonicera cerulea* anti-oxidant, cholesterol-reducing [Liu S et al., 2018] [a/vivo rat]
Lonicera japonica Thunb.	Anti-viral [RSV] [Li M et al., 2010] [vit]; anti-bacterial [Xiong et al., 2013] [vit]
Lonicera maackii (Rupr.) Maxim.	No records found
Lophatherum gracile Brongn.	Anti-inflammatory, anti-viral [RSV] [Chen LF et al., 2019] [a/vivo mouse, cell line]
Lophira lanceolata Tiegh. ex Keay	Anti-oxidant [Onyeto, 2014] [a/vivo mouse]; anti-hypertensive [Léandre et al., 2013] [a/vivo rabbit]
Ludwigia abyssinica A.Rich	Anti-bacterial [Oyedeji et al., 2011] [vit]
Ludwigia hyssopifolia (G.Don) Exell	Anti-inflammatory [Kundu et al., 2014] [a/vivo mouse]
Ludwigia peruviana (L.) H. Hara	Anti-oxidant [Armijos et al., 2018] [vit]
Ludwigia repens J.R. Forst.	Anti-inflammatory, sedative [Uddin ME et al., 2012] [a/vivo mouse]
Luma chequen (Molina) A.Gray Syn: *Myrceugenella chequen* (Mol.) Kausel. (ind)	Anti-oxidant [Simirgiotis et al., 2013] [vit]; anti- bacterial [Gonçalves et al., 2006] [vit]
Luma apiculata (DC.) Burret Syn: *Myrceugenella apiculuta* (DC.) Kausel. (ind)	Anti-platelet agglutination [Falkenberg et al., 2012] [h/cell platelet]; anti-virus [HSV] [Pacheco et al., 1993] [a/cell line monkey]; anti-bacterial [Viktorová et al., 2020] [vit]
Lupinus mutabilis Sweet	Anti-inflammatory [Gamarra et al., 2005] [a/vivo rat]
Lycianthes asarifolia (Kunth & Bouché) Bitter	No records found
Lycium intricatum Boiss.	Anti-oxidant [Abdennacer et al., 2015] [vit]
Lycium shawii Roem. & Schult.	Anti-inflammatory [Ahmed TA et al., 2012] [vit]; anti-bacterial [Dahech et al., 2013] [vit]; promotion of cell proliferation, NF-κB inhibition [Alkuwari et al., 2012] [h/cell line]
Lycopodiella cernua (L.) Pic. Serm.	Anti-bacterial [Ndip et al., 2008] [vit]
Lycopus lucidus Turcz. var *hirtus* Regel.	Anti- hypertensive [Yoon JJ et al., 2010] [h/cell line]; anti-inflammatory [Lee YJ et al., 2008] [h/cell line]
Lygodium circinnatum (Burm. f.) Sw.	No records found
Lygodium japonicum (Thunb.) Sw.	Anti-bacterial [Subba et al., 2016 [vit]
Lysimachia arvensis (L.) U.Manns & Anderb Syn: *Anagallis arvensis*	Anti-viral [HSV, polio] [Amoros et al., 1987] [vit]; anti-fungal [Ali-Shtayeh and Abu Ghdeib, 1999] [vit]; anti-inflammatory [López et al., 1999] [vit]
Macaranga peltata (Roxb.) Müll.Arg.	Anti-bacterial [Bijesh and Sebastian 2013] [vit]; anti-inflammatory [Gandhimathi, 2013] [a/vivo rat]
Macaranga perrieri Leandri [unresolved]	No records found
Macaranga triloba (Thunb.) Müll.Arg.	Anti-bacterial, anti-oxidant [Lim, TY et al., 2009] [vit]
Machaerium stipitatum (DC.) Vogel	No records found
Maclura tinctoria (L.) D. Don. Ex Steud.	Anti-inflammatory [Rivera et al., 2018] [a/cell line mouse]; anti-bacterial, anti-oxidant [Lamounier et al., 2012] [vit]; [morin] anti-ATP synthase [Chinnam et al., 2010] [vit]; anti- α-synucleinopathic [Caruana et al., 2011] [vit]
Macropanax dispermus (Blume) Kuntze	No records found
Maerua crassifolia Forssk.	Anti-inflammatory [Akuodor et al., 2016] [a/vivo rat]

Maesa lanceolata Forssk.	Anti-venom [Molander et al., 2014] [vit]; anti-viral [HIV] [Mengoni et al., 2002] [h/cell line]; anti-inflammatory [Elisha et al., 2016] [a/cell line mouse]
Maesobotrya vermeulenii (De Wild.) J.Léonard	No records found
Magnolia delavayi Franch.	No records found *Magnolia obovata* microglial NO inhibition; anti-neuroinflammation [Ock et al., 2010]
Mahonia bealei (Fortune) Pynaert	Anti-viral [influenza] [Zeng et al., 2006] [vit]; anti-bacterial [Li A. et al., 2008] [vit]; anti-inflammatory [Hu W. et al., 2016] [a/vivo mouse, cell line]
<u>*Maianthemum stellatum* (L.) Link</u>	No records found
Malachra rudis Benth.	No records found
Mallotus apelta (Lour.) Müll.Arg.	Anti- retroviral [Ono K. et al., 1989] [vit]
Mallotus philippensis (Lam.) Müll.Arg.	Anti-viral [polio, HSV] [Taylor et al., 1996] [a/cell line monkey]
Mallotus polycarpus (Benth.) Kulju & Welzen Syn: *Trewia polycarpa* Benth.	Anti-inflammatory [Chamundeeswari et al., 2004] [a/vivo rat]
Malpighia emarginata DC. Syn: *Malpighia glabra* L.	Anti-fungal [Cáceres et al., 1993a] [vit]; anti-inflammatory, reduced obesity-associated lipid metabolism defects [Dias et al., 2014] [a/vivo mouse]
Malus pumila Mill. Syn: *Malus domestica* (Suckow) Borkh.	Vasorelaxant [Matsui et al., 2009] [a/isolated aorta rat]; improved endothelial function; [Bondonno et al., 2018] [h/c].<br><br>[Quercetin] attenuated tau hyperphosphorylation [Chen J et al., 2016] [h/cell line neural]; anti- α-synucleinopathic [Caruana et al., 2011] [vit]; anti-tauopathic; inhibits ER stress [Chen J et al., 2016] [h/cell line neuron]; improved RGC survival [Gao et al., 2017] [a/vivo rat + rat RGC cell line rat].<br><br>[Fisetin] neuroprotective [Maher et al., 2011] [a/vivo HD mouse, *Drosophila*, a/cell line rat neuron].<br><br>[Kaempferol] autophagy induction [Che et al., 2017 [h/cell line], [Wu et al., 2017 [a/cell line rat neuron].<br>*Malus* as source of kaempferol: Stingl et al., 2002<br>*Malus* as source of fisetin: Kimira et al., 1998
Malus sylvestris (L.) Mill.	Anti-bacterial [Ihsan et al., 2018] [h/c]
Malva parviflora L.	Anti-inflammatory; anti-bacterial [Shale et al., 2005] [vit]
Malva sylvestris L.	Anti-inflammatory [Prudente et al., 2013] [a/vivo mouse]
Malva verticillata L.	Anti-bacterial [Jain P. et al., 2010] [vit]
Mandevilla velame (A.St.-Hil.) Pichon	No records found<br>*Mandevilla veraguasensis* anti-bacterial [Yaseen et al., 2017] [vit]
Mangifera indica L.	Anti-viral [HSV] [Yoosook et al., 2000] [a/cell line monkey]; anti-bacterial [Chitemerere and Mukanganyama, 2011] [vit]; anti-inflammatory [Garrido et al., 2001] [a/vivo mouse]; immunomodulatory [Makare et al., 2001] [a/vivo mouse]; anti-hypertensive [Ronchi et al., 2015] [a/vivo rat].<br>[Mangiferin] protects against mitochondrial dysfunction + neuronal apoptosis [Alberdi et al., 2018] [a/cell line rat neuron]memory improvement, raised NGF [Andreu et al., 2010] [a/vivo rat, h/cell line]; wound healing + growth factor promotion [Lwin et al., 2020] [a/vivo rat]
Manihot esculenta Crantz	Anti-bacterial [Noumedem et al., 2013] [vit]; anti-inflammatory [Adeyemi et al., 2008] [a/vivo rat, mouse]
Manilkara zapota (L.) P. Royen	Anti-inflammatory [Ganguly A. et al., 2013] [a/vivo rat]
Manotes expansa Sol. ex Planch.	No records found
Manotes longiflora Baker	No records found

*Mansoa alliacea (*Lam.) A.H.Gentry; *Mansoa* sp.	Anti-fungal [Freixa et al., 1998] [vit]; *M. hirsuita* anti-inflammatory [Campana et al., 2016] [cell line]
Mansonia altissima (A. Chev.) A. Chev.	No records found
Mapania cuspidata (Miq.) Uittien	No records found
Maranta arundinacea L.	Anti-ulcerogenic [Rajashekhara et al., 2014] [a/vivo rat]; immunostimulatory [Kumalasari et al., 2012] [a/vivo mouse]
Markhamia lutea (Benth) K. Schum.	Anti-viral [HSV] [Vlietinck et al., 1995] [vit, a/cell line monkey]
Marrubium deserti (de Noé) Coss	Anti-inflammatory [Saad et al., 2016] [a/vivo mouse]
Marrubium supinum L	No records found
Marrubium vulgare L.	Anti-inflammatory [Mascolo et al., 1987; Kanyonga et al., 2011] [a/vivo mouse]; anti-bacterial, anti-fungal [Zarai et al., 2011] [vit]
Marsilea quadrifolia L.	Anti-inflammatory, anti-venom [Subramanian et al., 2019] [vit]
Marsypianthes chamaedrys (Vahl) Kuntze	Anti-inflammatory [Ruppelt et al., 1991] [a/vivo mouse]
Martinella obovata (Kunth) Bureau & K.Schum.	No records found
Matricaria chamomilla L. Syn: *Matricaria recutita* L.	Anti-inflammatory [Al-Hindawi et al., 1989] [a/vivo rat]; anti-bacterial, anti-fungal [Nogueira et al.,2008] [vit]; anxiolytic [Amsterdam et al., 2009; Mao et al., 2016] [h/c]; inhibition of mitochondrial dysfunction, anti-apoptotic [Meeran et al., 2019] [a/vivo rat]; wound healing [Jarrahi , 2008] [a/vivo rat]; reversed memory impairment, raised BDNF [Ionita et al., 2018] [a/vivo rat}
Matricaria frigidum (HBK) Kunth	No records found
Mauria heterophylla H.B.K.	Anti-bacterial [Bussmann et al., 2008] [vit]
Mauritia flexuosa L.f.	Anti-inflammatory [Curimbaba et al., 2020] [a/vivo rat]; anti-oxidant, anti-bacterial [Koolen et al., 2013] [vit]; anti-platelet + anti-thrombotic [Fuentes et al., 2013] [a/vivo mouse, h/cell platelets]; improved lipid profile, reduced cholesterol [Aquino et al., 2015] [a/vivo rat]
Mauritiella armata (Mart.) Burret	No records found
Maxillaria sp.	Vasorelaxant [Rendón-Vallejo et al., 2012] [a/vivo rat]; anti-inflammatory [Déciga-Campos et al., 2007] [a/vivo mouse]
Maytenus boaria Molina	Anti-inflammatory [Backhouse et al., 1994] [a/vivo Guinea pig]
Maytenus ilicifolia Mart. ex Reissek	Anti-inflammatory [Jorge et al., 2004] [a/vivo rat, mouse]
Maytenus laevis Reissek	Anti-inflammatory [Moya and Olarte, 1977][a/vivo rat]
Medicago sativa L.	Anti- hypertensive [Martínez et al., 2016] [a/vivo rat]; anti-fungal [Sadowska et al., 2006] [vit]; anti-inflammatory [Hong YH et al., 2009] [a/vivo mouse]; oestrogenic-like activity [Jdidi et al., 2020] [a/vivo mouse]
Melaleuca kucadendra (L.) L.	Anti-inflammatory [Perera et al., 2016] [a/cell line]
Melaleuca viridiflora Sol. ex Gaertner	No records found
Melanthera scandens (Schumach. & Thonn.) Roberty	Anti-bacterial [Adesanwo et al., 2019] [vit]
Melastoma sp.	*Melastoma malabathricum* anti-bacterial [Choudhury et al., 2011; Omar et al., 2012] [vit]
Melicoccus bijugatus Jacq.	No records found
Melilotus alba Medikus	Anti-bacterial [Stefanović et al., 2015] [vit]
Melilotus suaveolens Ledeb.	Anti-inflammatory [Tao JY et al., 2009] [a/cell line mouse]
Melinis minutiflora P.Beauv.	No records found
Melissa officinalis L.	Anti-hypertensive [Ferreira A et al., 2006] [vit]; memory improvement [Soodi et al., 2014] [a/vivo rat]; anti-inflammatory [Bounihi et al., 2013] [a/vivo rat]; anti-viral [Allahverdiyev et al., 2004] [h/cell line]; improved delayed word recall [Perry NS et al., 2018] [h/c in spp. combn]; mood modulation [Kennedy et al., 2002]

	[h/c]; anti- α-synucleinopathic [Caruana et al., 2011] [vit]; anti-apoptotic [Soodi et al., 2017] [a/cell line mouse neuron]
Mentha arvensis L.	Anti-bacterial, anti-oxidant [Biswas NN et al., 2014] [vit]; DNA oxidative damage protection [Lin et al., 2013] [h/cell line]; anti-inflammatory [Malik et al., 2012] [vit]; *Mentha* × *villosa* anti-hypertensive [Pakdeechote et al., 2011] [a/vivo rat]
Mentha canadensis L. Syn*:* *Mentha haplocalyx* Briq.	Anti-bacterial [Jirovetz et al., 2009] [vit]; Anti- α-synucleinopathic [Caruana et al., 2011] [vit]
Mentha longifolia (L.) L	Anti-bacterial, anti-fungal [Mkaddem et al., 2009] [vit]; neuroprotective [López V et al., 2010] [cell line]; anti-hypertensive [Samaha et al., 2019] [h/c]; anxiolytic [López V et al., 2010] [vit]
Mentha pulegium L.	Anti-hypertensive, vasorelaxant [Ajebli and Eddouks, 2020] [a/vivo rat]; anti-bacterial [Mahboubi and Haghi, 2008] [vit; anti-inflammatory [Moussaid et al., 2011] [a/vivo mouse, vit]; anti-viral [HSV] [Parsania et al., 2017] [h/cell line]
Mentha × *rotundifolia* (L.) Huds.	Anti-inflammatory [Boussouf et al., 2017] [a/vivo mouse]
Mentha royleana Wall. ex Benth. Syn: *Mentha royleana* subsp. *hymalaiensis* Briq	Anti-bacterial [Hussain A et al., 2015] [vit]
Mentha spicata L. Syn: *Mentha viridis*	Anti-amyloidogenic [Ishigaki et al., 2013] [vit]; anti-bacterial, anti-fungal [Aggarwal et al., 2002] [vit]; anti-hypertensive [Cam et al., 2020] [vit]; anti-viral [Orhan et al., 2012] [a/cell line monkey]
Mentha x *piperita* L. [hybrid of *M. spicata* L. and *Mentha aquatica L.]*	Anti-bacteriial, anti-oxidant [Singh et al., 2015] [vit]; anti-vira [HSV] [Nolkemper et al., 2006] [a/cell line monkey]; anti-fatigue [Umezu et al., 2001] [a/vivo mouse]; anxiolytic [López V et al., 2010] [vit]; anti-aging [Sarikhani et al., 2021] [[a/cell line rat]
Mercurialis annua L.	Anxiolytic [Doukkali et al., 2016] [a/vivo mouse]
Meriandra dianthera (Roth ex Roem. & Schult.) Briq.	Anti-bacterial, anti-fungal [Mothana et al., 2019] [vit]
Mesua ferrea L.	Anti- venom [Uawonggul et al., 2006] [a/cell line *Gallus*]; anti-bacterial [Verotta et al., 2004] [vit]; anti-inflammatory [Tiwari et al., 2012] [a/vivo rat]
Microglossa pyrifolia (Lam.) Kuntze	No records found
Micromeria myrtifolia Boiss. & Hohen.	No records found
Microtea debilis Sw.	Anti-inflammatory [Bai et al., 2011] [h/cell line]
Mikania cordata (Burm.f.) B.L.Rob.	Anti-inflammatory [Bhattacharya et al., 1992] [a/vivo rat
Mikania cordifolia (L.f.) Willd.	No records found
Mikania guaco Kunth	No records found
Miliusia andamanica (King) Finet & Gagnep.	No records found
Millettia eetveldeana (Micheli) Hauman	No records found<br>*Millettia pachycarpa* anti-retroviral [Ono K. et al., 1989] [vit]
Millettia laurentii De Wild.	No records found
Milletia pinnata (L.) Panigrahi	No records found
Millettia thonningii (Schum & Thonn) Bak	Anti-bacterial [Harrison et al. 2019] [vit]
Millettia versicolor Welw. Ex Baker	No records found
Mimosa nothacacia Barneby	No records found
Mimosa pigra L.	Anti-microbial [Grosvenor et al., 1995] [vit]; anti-hypertensive, anti-oxidant [Rakotomalala et al., 2013] [h/cell line, a/vivo rat], a/cell line rat, vit]
Mimosa pudica L.	Anti-viral [mumps] [Malayan et al., 2013] [a/cell line monkey]; anti-venom [Meenatchisundaram et al., 2009] [a/vivo mouse]; wound healing [Kokane et al., 2009] [a/vivo rat]; anti-convulsant [Ngo Bum et al., 2004] [a/vivo mouse]; memory improvement, AChE inhibition, anxiolytic [Patro et al., 2016] [a/vivo mouse]
Mimusops elengi L*.*	Anti-inflammatory [Purnima et al., 2010] [a/vivo rat]

Minthostachys glabrescens (Benth.) Epling Syn: *Bystropogon glabrescens* Benth.	No records found
Minthostachys mollis (Kunth) Griseb.	Anti- viral [Brand et al., 2016] [vit]; anti-bacterial [Pellegrini et al., 2014] [vit]
Mirabilis jalapa L.	Anti-bacterial [Martinez et al., 1996] [vit]; anti-inflammatory [Singh M et al., 2010] [a/vivo rat]
Mitracarpus hirtus (L.) DC	No records found
Mitragyna inermis (Willd.) K.Schum.	Memory improvement [Pahaye et al., 2017] [a/vivo mouse]
Mitragyna parvifolia (Roxb.) Korth.	Anti-inflammatory [Sahu et al., 2016] [a/vivo rat]
Mitragyna rubrostipulata (Schum.) Hav.	No records found
Modiola caroliniana (L.) G.Don	No records found
Mollugo cerviana (L.) Ser.	Anti-inflammatory [Sadique et al., 1987] [a/vivo rat]
Mollugo nudicaulis Lam.	Anti-bacterial, anti-oxidant [Rameshkumar and Sivasudha, 2012] [vit]
Momordica balsamina L.	Anti-viral [Kaur I et al., 2011] [vit], [HIV] [Bot et al., 2007] [h/cell line]; anti-inflammatory [Karumi et al., 2003] [a/vivo rat]; anti-oxidant [Akula and Odhav, 2008] [vit]
Momordica charantia L.	Anti-venom [Asad et al., 2013] [vit]; anti-viral [influenza] [Pongthanapisith et al., 2013] [a/cell line canine]<br>*Momordica cochinchinensis* neurite outgrowth induction [Mazzio et al., 2015] [a/cell line rat neuron]
Monardella odoratissima Benth.	No records found
Mondia whitei (Hook.f.) Skeels	AChE inhibition, anti-bacterial [Baskaran et al., 2016] [vit]
Moneses uniflora (L.) A.Gray	Anti-bacterial, anti-fungal [McCutcheon et al., 1992, 1994] [vit]
Monodora myristica Dunal	Anti-bacterial [Cimanga et al., 2002] [vit]
Monolluma hexagona (Lavranos) Meve & Liede	No records found
Monolluma quadrangula (Forssk.) Plowes	No records found
Monstera sp.	*Monstera deliciosa* anti-bacterial, anti-oxidant [Rao VU et al., 2015] [vit]
Morina polyphylla Wall. ex DC.	No records found
Morinda citrifolia L.	Anti-bacterial [Jayaraman SK et al., 2008] [vit]; anti-inflammatory [Basar et al., 2010] [a/vivo mouse, h/cell line] anti-inflammatory, anti-oxidant [Wan Osman et al., 2019] [a/vivo rat]; neuroprotective, anti-apoptotic [Chen J et al., 2018] [h/cell line]; anti-atherosclerotic, reduced cholesterol, anti-hypertensive [Chong et al., 2018] [a/vivo rat]
Morinda lucida Benth.	Cognitive enhancement [Elufioye and Hameed, 2017] [a/vivo mouse]; anti-bacterial [Fakoya et al., 2014] [vit]
Morinda pubescens Sm.	Wound healing [Mathivanan et al., 2006] [a/vivo rat]; anti-inflammatory [Jeyabalan and Palayan, 2009] [a/vivo rat]
Morinda umbellata L.	Free radical scavenging [Krishnakumar et al., 2016] [vit]
Moringa oleifera Lam.	Anti-bacterial [Salau and Odeleye, 2007] [vit]; attenuates tau hyperphosphorylation, decreased amyloid production, rescues cognitive impairment [Mahaman et al., 2018] [a/vivo rat]; anti-glaucoma [Wulandari et al., 2019] [h/cell line trabecular]; anti-bacterial [Caceres et al., 1991] [vit]; anti-oxidant, anti-inflammatory, immunomodulatory, anti-apoptopic [Galuppo et al., 2014] [a/vivo MS mouse]; wound healing [Rathi et al., 2006] [a/vivo rat]; improved memory impairment [Omotoso et al., 2018] a/vivo MS rat], [Zhou J et al., 2018] [a/vivo AD mouse]; anti-convulsant [Bakre et al., 2013] [a/vivo mouse]; anti-fatigue [Lamou et al., 2016] [a/vivo rat]; promotes neurogenesis [Romeo et al., 2018] [h/cell line]; anti-atherosclerotic, promotes autophagy [Zhou Y et al., 2017] [h/cell line, a/vivo mouse]; delayed ALS disease phenotypes [Galuppo et al., 2015] [a/vivo ALS rat]; anti-

	aging [Im et al., 2016] [a/vivo *C. elegans*]; AChE inhibition [Nwidu et al., 2018] [vit]; reduced mitochondrial dysfunction [González-Burgos et al., 2021] [h/cell line]; metal chelation [Velaga et al., 2014] [a/vivo rat]
Morus alba L. Syn: *Morus multicaulis* (Perr.) Perr.	Anti-hypertensive, anti-hyperlipidemic [Lee YJ et al., 2011] [a/vivo rat]; anti-inflammatory [Chung et al., 2003] [a/vivo rat]; anti-viral [influenza] [Kim H et al., 2018] [a/cell line canine], [HSV] [Du J et al., 2003] [a/cell line monkey]; anti-bacterial, anti-fungal [De Oliveira AM et al., 2015] [vit]; attenuates tau hyperphosphorylation [Xia et al., 2019] [a/cell line mouse neuron + microglia]; anti-fatigue [Chen H et al., 2016] [a/vivo mouse]; gut microbiota modulation, lipogenesis downregulation [Li Y et al., 2020] [a/vivo mouse]<br>[morin] anti-amyloidogenic [Du et al., 2016] [AD mouse]<br><br>[miglustat/eliglustat] NPC disease increased survival [Nadjar et al., 2018; Walterfang et al., 2012] [h/c]; GM1 Gangliosidosis reversed disease progression [Deodato et al., 2017] [h/c]<br><br>*Morus alba* as source of morin: Rajput et al., 2021
Morus nigra L.	Anti-bacterial [Mazimba et al., 2011] [vit]; anti-inflammatory [Padilha et al., 2010] [a/vivo rat]; cholesterol reducing, lipolytic [Fan et al., 2020] [a/vivo porcine]
Mucuna pruriens (L.) DC.	Anti-neuroinflammation, reduced apoptosis of dopaminergic neurons [Rai et al., 2017] [a/vivo PD mouse]; anti-PD [Katzenschlager et al., 2004] [h/c], motor improvement [Cilia et al., 2017] [h/c]; reversed TDP-43 pathology [Maccioni et al., 2018] [a/vivo *Drosophila*]; anti-epileptic [Champatisingh et al., 2011] [a/vivo rat]; anti-venom [Tan NH et al., 2009] [a/vivo rat]; wound healing [Ohadoma SC, Lawal, 2019] [a/vivo mouse]
Mucuna stans Baker	No records found
Muehlenbeckia tamnifolia (Kunth) Meisn.	Anti-inflammatory [Jasbleidy et al., 2017] [a/vivo rat]
Multidentia fanshawei (Tennant) Bridson	No records found
Muntingia calabura L.	Anti-bacterial [Zakaria et al., 2010] [vit]; anti-inflammatory [Preethi et al., 2012] [a/vivo rat]
Murraya paniculata (L.) Jack Syn: *Murraya exotica* L.	Anti-inflammatory [Narkhede et al., 2012] [a/vivo rat]
Musa × paradisiaca L. Syn: *Musa × sapientum* L.	Memory improvement, anxiolytic [Samad et al., 2017] [a/vivo mouse]; anti-bacterial [Kapadia et al., 2015] [vit]; anti-bacterial, anti-fungal [Karadi et al., 2011] [vit]; anti-viral [HSV] [Subramaniam G et al., 2020] [a/cell line monkey]; anti-convulsant, anxiolytic [Reddy et al., 2017, 2018] [a/vivo mouse]; anti-hypertensive [Parmar and Kar, 2007] [a/vivo rat].<br><br>[tannic acid] prion protein inhibition [Kocisko et al., 2003] [a/cell line mouse neuron]<br>*M. paradisiaca*as source of tannin: Onyenekwe et al., 2013
Musa spp.	*M. acuminata* anti-Parkinson, anti-oxidant [Kanazawa and Sakakibara, 2000] [vit]; anti-viral [HIV] [Cheung et al., 2009] [vit]
Mussaenda philippica A.Rich	Anti-bacterial [Bungihan and Matias, 2011] [vit]
Muntingia calabura L.	Anti-bacterial [Boopath et al., 2017] [vit]
Myrsine umbellata Mart	No records found
Myrcianthes discolor (Kunth) McVaugh	Anti-bacterial [Bussmann et al., 2009] [vit]
Myrcianthes leucoxyla (Ortega) McVaugh.	Anti-bacterial [Ospina et al., 2016] [vit]
Myrciaria dubia (Kunth) McVaugh	Anti-bacterial [Fujita et al., 2015] [vit]
Myrianthus arboreus P.Beauv.	Anti-bacterial, anti-fungal, wound healing [Agyare et al., 2014] [vit, a/vivo rat]

Myrica esculenta Buch.-Ham. Ex D.Don	No records found<br>*Myrica cerifera* enhanced tau clearance [Jones JR et al., 2011] [h/cell line neural]
Myristica fragrans Houtt.	Anti-inflammatory [Ozaki Y et al., 1989] [a/vivo rat, mouse]; antioxidant, anti- amyloidogenic [Lee JE et al., 2017] [vit] reduced microglial activation, anti-convulsant [Ghorbanian et al., 2019] [a/vivo mouse]; memory improvement [Parle et al., 2004] [a/vivo mouse]
Myroxylon balsamum (L.) Harms	Anti-bacterial [Machado et al., 2005] [vit]
Myroxylon peruiferum L. f.	Anti-bacterial, anti-fungal, anti-oxidant [Pereira et al., 2019] [vit]
Myrtus communis L.	Anti-inflammatory [Rossi et al., 2009] [a/vivo mouse]; anti-bacterial [Mansouri et al., 2001] [vit]; anti-viral [Minaei et al., 2014] [h/c]; antifungal [Cannas et al., 2013] [vit]; anti-hypertensive [Cevikelli-Yakut et al., 2020] [a/vivo rat]
Myrtus nivelii Batt. & Trab.	Anti-inflammatory [Rached et al., 2017] [vit];
Nandina domestica Thunb.	Anti-inflammatory [Ueki et al., 2012] [h/cell line]; anti-HIV [Xu HX et al., 1996] [vit]
Napoleonaea vogelii Hook. & Planch.	No records found
Nasa loxensis (Kunth) Weigend	No records found
Nasturtium officinale W.T. Aiton Syn: *Rorippa nasturtium-aquaticum* (L.) Hayek	Hsp 90 inhibition, Nrf2 activation [Dayalan Naidu et al., 2016] [h/cell line]; anti-bacterial [Penecilla and Magno, 2011] [vit]; anti-inflammatory [Sadeghi et al., 2014] [a/vivo rat, mouse]
Nauclea diderrichii (De Wild.) Merr.	Anti-bacterial, antifungal, wound healing [Akunne et al., 2017] [a/vivo rat, mouse, vit]
Nauclea latifolia Sm. Syn: *Sarcocephalus latifolius*	Anti-inflammatory [Abbah et al., 2010] [a/vivo rat]; anti-bacterial [Deeni and Hussain, 1991] [vit]
Neea sp.	No records found
Neopicrorhiza scrophulariiflora (Pennell) D.Y.Hong Syn*:* *Picrorhiza scrophulariiflora* Pennell	Anti-inflammatory [Smit et al., 2000] [a/vivo mouse]
Nepenthes ampullaria Jack	No records found
Nepeta cataria L.	Anti-bacterial, anti-fungal [Adiguzel et al., 2009] [vit]
Nepeta erecta (Royle ex Benth.) Benth.	Anti-bacterial, anti-fungal [Bisht et al., 2010] [vit]
Nepeta glomerulosa Boiss.	Anti-bacterial [Nezhadali et al., 2011] [vit]
Nephrolepis auriculata (L.) Trimen [unresolved]	No records found
Nephrolepis biserrata (Sw.) Schott	No records found
Nephrolepis cordifolia (L.) C. Presl	Anti-inflammatory [Amoroso et al., 2014] [a/vivo rat]; Anti-bacterial, anti-fungal [Adebiyi, 2019] [vit]
Nervilia plicata (Andrews) Schltr.	Anti-bacterial, anti-fungal [Haridas et al., 2014] [vit]
Neurolaena lobata (L.) R.Br. ex Cass.	Anti-inflammatory [Lajter et al., 2014] [h/cell line]
Newbouldia laevis (P.Beauv.) Seem. ex Bureau	Anti-bacterial, anti-fungal [Gafner et al., 1996] [vit]
Nigella sativa L.	Anti-inflammatory, enhanced remyelination [Fahmy et al., 2014] [a/vivo MS rat]
Niphidium crassifolium (L.) Lellinger Syn: *Polypodium crassifolium* L.	No records found
Niphogeton ternata (Willd. Ex Schltr.) Mathias & Constance	No records found
Nothofagus dombeyi (Mirb.) Oerst.	No records found
Notopleura uliginosa (Sw.) Bremek. Syn: *Psychotria uliginosa* Sw.	No records found

Nyctanthes arbor-tristis L.	Anti-inflammatory [Saxena et al., 1984] [a/vivo rat]
Nymphaea lotus L.	Anti-inflammatory [Rege et al., 2020] [a/vivo mouse, rat]; anxiolytic [Fajemiroye et al., 2018] [a/vivo mouse]; anti-bacterail [Akinjogunla et al., 2009] [vit]
Nymphaea nouchali Burm.f. Syn: *Nymphaea stellata* Burm. F.	Anti-inflammatory, anti-oxidant, anti-ulcerogenic, anti-apoptotic [Antonisamy et al., 2014] [a/vivo rat]; anti-bacterial [Dash et al., 2013] [vit]
Ochanostachys amentacea Mast.	Anti-viral [HIV] [Rashid et al., 2001] [vit]
Ochna afzelii R.Br. ex Oliv.	Anti-inflammatory [Anuradha et al., 2006] [reviewed by Bandi et al., 2012][vit]; *Ochna integerrima* anti-viral [HIV] [Reutrakul et al., 2007] [vit]
Ochradenus baccatus Delile	No records found
Ochroma pyramidale (Cav. Ex Lam.) Urb.	No records found
Ocimum americanum L. Syn: *Ocimum canum* Sims	Anti-bacterial [Cimanga et al., 2002] [vit]; anti-oxidant, anti-inflammatory and AChE inhibition [Zengin et al., 2019] [vit, a/ex vivo rat colon]
Ocimum basilicum L.	Anti-viral [HSV, ADV, hep B] Chiang et al., 2005] [vit]; [Antioxidant and anti-microbial [Hussain AI et al., 2008]; anti-hypertensive [Umar et al., 2010] [a/vivo rat]; [carvacrol] anti-ATP synthase [Liu et al., 2016; Lee SJ et al., 2005] [vit]; anti- α-synucleinopathic [Caruana et al., 2011] [vit]
Ocimum campechianum Mill.	Anti-viral [HSV] [Brand et al., 2016] [vit]
Ocimum carnosum (Spreng.) Link & Otto ex Benth.	No records found
Ocimum filamentosum Forssk. Syn*: Becium filamentosum*	Anti-bacterial, anti-fungal [Qwarse et al., 2017] [vit]
Ocimum campechianum Mill. Syn: *Ocimum micranthum* Willd	No records found
Ocimum tenuiflorum L Syn: *Ocimum sanctum* L.	DNA-damage protection, anti-oxidant [Kaur P et al., 2018] [vit]; wound healing [Shetty et al., 2008] [a/vivo rat]; increased longevity, Hsp-70 + *sir.2.1* gene upregulation [Pandey et al., 2013] [a/vivo *C. elegans*]
Ocotea usambarensis Engl.	No records found<br>*Ocotea duckei* anti-PAF [Ribeiro R et al., 1996] [a/vivo rat]
Odontonema tubaeforme (Bertol.) Kuntze.	No records found
Oenanthe javanica (Blume) DC.	Anti-thrombotic [Ku et al., 2013] [h/cell line]; neuroprotective [Ma et al., 2010] [a/cell line rat neuron]; anti-viral [Han et al., 2008] [cell line]; anti-inflammatory Lee KH et al., 2011] [cell line]
Oenocarpus bataua Mart. Syn: *Jessenia bataua* (Mart.) Burret	Anti-viral [viral hepatitis] [Roumy et al., 2020] [vit]
Olax gambecola Baill.	Anti-hypertensive [Parry et al., 1986] [a/vivo rat]
Olea europaea L.	Anti-hypertensive [Susalit et al., 2011] [h/c]; proteasome stimulation [Katsiki et al., 2007] [h/cell line]; anti-platelet aggregation [Singh I et al., 2008] [h/cells blood]; anti-inflammatory, improved vascular function [Lockyer et al., 2015] [h/c]; anti-dyslipidemic, improved lipid profile [Verhoeven et al., 2015] [h/c]; anti-bacterial, anti-fungal [Pereira AP et al., 2007] [vit]; enhanced cognition [Valls-Pedret et al., 2015] [h/c]; ALS: improved survival + motor performance, larger muscle fibre area [Oliván et al., 2014] [a/vivo ALS mouse]; reduced ß-amyloid levels + plaque deposits, reduced astrocyte reaction [Grossi et al., 2013] [a/vivo mouse]; reduced amyloid β aggregation, decreased paralysis, increased lifespan [Diomede et al., 2013] [a/vivo AD *Caenorhabditis elegans*]; anti-tau [Monti et al., 2012] [vit]; anti-synuclein [Palazzi et al., 2018] [vit]; anti-DNA damage [Salvini et al., 2006] [h/c]; anti-apoptosis [Pasban-Aliabadi et al., 2013] [a/cell line rat neuron]; ATP synthase inhibition [Amini et al., 2017] [vit]; anti-oxidant [Oliveras-López et al., 2013] [h/c]; anxiolytic [Perveen et al., 2013] [a/vivo rat]

Operculina hamiltonii (G. Don) D.F. Austin & Staples Syn: *Operculina alata* (Ham.) Urb.	No records found
Ophioglossum reticulatum L.	No records found
Ophryosporus axilliflorus (Griseb.) Hieron.	Anti-bacterial [Sivakumar et al., 2008] [vit]; anti-inflammatory [Favier et al., 1998] [a/vivo mouse]
Opuntia albispinosa Mieckley ex Lindemuth & Dams	No records found
Opuntia cochenillifera (L.) Mill.	Anti-bacterial [Necchi et al. 2012] [vit]
Opuntia dillenii (Ker Gawl.) Haw.	Anti-inflammatory [Loro et al., 1999] [a/vivo rat]
Opuntia ficus-indica (L.) Mill.	Anti-inflammatory [Park et al., 1998,2001] [a/vivo rat, mouse]; endothelium modulation [Gentile et al., 2004] [h/cell endothelium]; wound healing [Khémiri et al., 2019] [a/vivo rat]; memory improvement, increased BDNF [Kim JM et al., 2010] [a/vivo mouse]
Opuntia humifusa (Raf.) Raf. Syn: *Opuntia vulgaris* J. Miller	Anti-bacterial, anti-oxidant [Lee and Lee, 2010] [vit]; anti-inflammatory [Cho et al., 2006] [cell line]
Opuntia minor Müll. Hal.	No records found
Orbivestus karaguensis (Oliv. & Hiern) H.Rob. Syn: *Vernonia karaguensis* Oliv. & Hiern	No records found
Oreocallis grandiflora (Lam.) R.Br.	Anti-inflammatory [Vinueza et al., 2018] [vit]
Oreopanax malacotrichus Harms Syn: *Oreopanax eriocephalus* Harms	Moderately anti-bacterial [Bussmann et al., 2008] [vit]
Oreopanax sp.	*Oreopanax ecuadorensis* anti-fungal [Noriega et al., 2019] [vit]
Origanum majorana L	Anti-inflammatory [Seoudi et al., 2009] [a/vivo rat]; anti-bacterial [Deans and Svoboda, 1990] [vit]; improved memory and cognitive function, elevated BDNF, anti-oxidant [Postu et al., 2020] [a/vivo rat]; anxiolytic [Rezaie et al., 2014] [a/vivo rat]
Origanum onites L	No records found
Origanum vulgare L.	Antioxidant, anti-bacterial, anti-fungal [Şahin et al., 2004] [vit]; anti-PD, AChE inhibition [Hanganu et al., 2020] [vit]; immunomodulatory [reduced activated $CD4^{+}CD25^{+}$ cells], anti-inflammatory, anti-apoptotic [Vujicic et al., 2015] [a/vivo mouse]; reduced cognitive impairment [Capatina et al., 2021] [a/vivo zebrafish].<br>[carvacrol] anti-ATP synthase [Liu et al., 2016].<br>*O. vulgare* as source of carvacrol: Teixeira et al., 2013
Oroxylum indicum (L.) Kurz	Anti-bacterial, anti-fungal, anti-inflammatory [Ali et al., 1998] [vit]
Orthosiphon pallidus Royle ex Benth.	Anti-microbial [Singh MK et al., 2017] [vit]
Oryctanthus alveolatus (Kunth) Kuijt	No records found
Otoba parvifolia (Markgr.) A.H.Gentry	No records found
Ottelia alismoides (L.) Pers.	No records found
Ottelia ulvifolia (Planch.) Walp.	No records found
Oxalis adenophylla Gillies ex Hook. & Arn.	No records found
Oxalis articulata Savigny	No records found
Oxalis corniculata L.	Anti-inflammatory [Sakat et al., 2010] [vit]; wound healing [Taranalli et al., 2004] [a/vivo rat]; anti-hypertensive [Rinayanti et al., 2013] [vit]; anti-oxidant [Alam et al., 2011b] [a/vivo rat];
Oxalis frutescens (Kunth) Lourteig	No records found
Oxalis peduncularis Kunth	No records found
Oxalis succulenta Barn.	No records found
Oxygonum sinuatum (Hochst. & Steud ex Meisn.) Dammer	Anti-inflammatory [Matu and Van Staden, 2003] [vit]

Oxytenanthera abyssinica (A.Rich.) Munro	No records found
Ozoroa insignis Del.	Anti-bacterial [Mathabe et al., 2006] [vit]
Ozoroa pulcherrima (Schweinf.) R.Fern. & A.Fern.	Anti-inflammatory [Jatsa et al., 2019] [a/vivo mouse]
Paederia foetida L.	Anti-hyperlipidemic [a/vivo rat]; anti-inflammatory [De S et al., 1994] [a/vivo rat]; anti-bacterial [Uddin SJ et al., 2008] [vit]; anxiolytic [Billah et al., 2015] [a/vivo mouse]; anti-viral [Hep E] [Roy K et al., 2017] [a/cell line porcine]
Paeonia lactiflora Pall.	Anti-inflammatory [Zhao M et al., 2019] [h/cell line]; anti-viral [HBV] [Lee SJ et al., 2006] [h/cell line], [influenza] [Ho JY et al., 2014] [a/vivo mouse, a/cell line canine]; anti- amyloidogenic [Lee JE et al., 2017] [vit]
Paeonia suffruticosa Andrews	No records found<br>*Paeonia delavayi* anti-viral [influenza] [Yang XY et al., 2016]
Palicourea crocea (Sw.) Schult.	No records found
Paliurus spina-christi Mill.	Anti-inflammatory, anti-oxidant [Sen, 2018] [vit]; cholesterol-reducing, anti-hyperlipidemic [Mosaddegh et al., 2010] [a/vivo rat]
Panax japonicus (T.Nees) C.A.Mey.	Immunostimulatory [Hyun et al., 2021] [h/c]; induces neurite outgrowth [Zou et al., 2002] [h/cell line neural]; reduced cognitive decline [Ruan et al., 2019] [a/vivo rat]; anti-neuroinflammatory [Deng et al., 2017] [a/vivo rat]; vasorelaxant [Yu et al., 2002] [a/vivo rat];<br>*Panax ginseng* enhanced autophagy, reduced HTT + amyoid aggregation [Fan et al., 2017] [h/cell line, a/vivo mouse]
Panax quinquefolius L.	Immunomodulatory, anti-inflammatory [Lui et al., 2012] [a/vivo rat, cell line]; antioxidant, metal chelation [Kitts et al., 2000]; anti-apoptotic [Luo and Luo, 2006] [rat cell line]; memory improvement [Scholey et al., 2010; Ossoukhova et al., 2015] [h/c]; raised NGF [Salim et al., 1997] [a/vivo rat]; anti-amyloidogenic [Chen F et al., 2006] [a/vivo mouse]; anti-hypertensive [Mucalo et al., 2013] [h/c]; protects dopaminergic neurons [Heng et al., 2016] [a/vivo mouse PD]; anti-fatigue [Barton et al., 2013] [h/c]
Pandanus amaryllifolius Roxb.	Anti-bacterial [Laluces *et al*., 2015] [vit]
Pandanus sp.	*Pandanus odoratissimus* anti-bacterial [Kumar D et a., 2010][vit]
Pandanus tectorius Parkinson ex Du Roi	Anti-bacterial [Andriani et al., 2019] [cit]
Papaver dubium L	No records found
Papaver rhoeas L	No records found
Paranephelius uniflorus Poepp. & Endl.	No records found
Parietaria judaica L.	No records found
Parietaria officinalis L.	No records found
Parinari curatellifolia Planch. ex Benth.	Anti-bacterial [Peni et al., 2010] [vit]; anti-venom [Omale et al., 2012] [a/vivo mouse]
Parinari excelsa Sabine	No records found
Paris polyphylla Smith.	Anti-fungal [Deng D et al., 2008] [vit]; anti-viral [influenza] [Pu X et al., 2015] [a/vivo mouse, vit]; anti-bacterial [Qin et al., 2012] [vit]
Parkia biglobosa (Jacq.) R.Br. ex G.Don	No records found
Parkinsonia aculeata L.	Anti-inflammatory [Marzouk MS et al., 2013] [vit]
Parnassia nubicola Hk. f.	No records found
Parochetus communis Buch.-Ham. ex D. Don	No records found
Parthenium hysterophorus L.	Anti-inflammatory [Pandey et al., 2012] [a/vivo rat, vit]
Paspalum conjugatum P.J.Bergius	No records found
Paspalum melanospermum Desv. ex Poir.	No records found
Paspalum notatum Flüggé	No records found

Passiflora edulis Sims Syn: *Passiflora incarnata* L.	Anti-hypertensive [Ichimura et al., 2006] [a/vivo rat]; anti-bacterial [López-Vargas et al., 2013] [vit]; neurogenic, memory improvement, decreased tau [Kim GH et al., 2019] [a/vivo mouse]; anti-inflammatory [Farid et al., 2010] [h/c], [Montanher et al., 2007] [a/vivo mouse]; antioxidant [Rudnicki et al., 2007] [vit]; anxiolytic [Deng J et al., 2010] [a/vivo mouse]; anti-fatigue [Hu M et al., 2020] [a/vivo mouse]
Passiflora foetida L.	Anti-bacterial [Mohanasundari et al., 2007] [vit]; anti-depressant [Santosh et al., 2011] [a/vivo mouse]
Passiflora ligularis Jus.	Anti-microbial, anti-oxidant [Saravanan et al., 2014] [vit]
Passiflora quadrangularis L.	No records found
Paullinia cupana Kunth	Anti-hyperlipidemic [Ruchel et al., 2017] [a/vivo rat]; increased mitochondrial biogenesis [Lima NDS et al., 2018] [a/vivo mouse]
Paullinia pinnata L.	Anti-venom [Molander et al., 2014] [vit]
Pauridiantha paucinervis (Hiern) Bremek.	No records found
Pavetta refractifolia K.Schum.	No records found
Peganum harmala L.	Anti-inflammatory [Khlifi et al., 2013] [a/cell line mouse]; anti-hypertensive: ACE inhibition [Kouchmeshky et al., 2012] [vit]; anxiolytic [Sassoui, 2012] [a/vivo rat]<br><br>*Toxic in high doses [Mahmoudian et al., 2002]
Pelargonium graveolens L'Hér.	Anti-bacterial [Ghannadi et al., 2012] [vit]
Pelargonium odoratissimum (L.) L'Herit.	Anti-fungal [Andrade et al., 2011] [vit]
Pelargonium peltatum (L.) L'Hér.	Anti-bacterial [Hurtado et al., 2013] [vit]
Pelargonium roseum Willd.	Anti-bacterial, anti-fungal [Carmen and Hancu, 2014] [vit]; wound healing [Panah et al., 2012] [h/c] [in sp. comb.]
Pelargonium zonale (L.) L'Hér.	No records found<br>*Pelargonium sidoides* anti-viral [cold] [Lizogub et al., 2007] [h/c]
Peltogyne paniculata Benth	No records found
Peltophorum africanum Sonder	Anti-viral [HIV] [Theo et al., 2009] [h/cell line]
Pennisetum glaucum (L.) R.Br.	Immune system activation [Nani et al., 2015] [a/cell line rat]
Pentacalia corymbosa (Benth.) Cuatrec.	Anti-fungal [Torrenegra et al., 2000] [vit]
Pentacalia sp.	No records found
Pentadesma butyracea Sabine	Anti-microbial [Tamokou et al., 2013] [vit]
Pentadiplandra brazzeana Baill.	Anti-inflammatory [Foe et al., 2016] [vit]; anti-bacterial [Nyegue et al., 2009] [vit]
Pentas schimperiana subsp. *occidentalis* (Hook.f.) Verdc.	No records found
Pentasachme caudatum Wall. ex Wight Syn: *Pentasacme championii* Benth.	No records found
Peperomia garcia-barrigana Trel. & Yunck.	No records found
Peperomia pellucida (L.) Kunth	Anti-inflammatory [Arrigoni-Blank et al., 2004] [a/vivo rat, mouse]; anti-hypertensive [Ahmad I, 2016] [vit]; anti-bacterial [Wei LS et al., 2011] [vit]
Perezia pungens Less.	No records found
Pergularia daemia (Forssk.) Chiov. Syn: *Pergularia extensa* (Jacq.) N.E. Br.	Anti-epileptic, neuroprotective [Kandeda et al., 2017] [a/vivo mouse]

Perilla frutescens (L.) Britton	Anti-inflammatory [Ueda H et al., 2002] [a/vivo mouse]; anti-bacterial, anti-fungal [Kang R et al., 1992] [vit]; anti-platelet aggregation, anti-thrombotic [Jang JY et al., 2014] [a/vivo rat]. [luteolin] reduced inflammation and axonal damage [Hendriks et al., 2004] [a/vivo MS rat]; [perillaldehyde] anti-apoptotic [Xu L et al., 2014] [a/vivo rat]
*Peristrophe bicalyculata (*Retz.) Nees	Reversed memory impairment, anti-neuroinflammatory, AChE inhibition [Njan et al., 2020] [a/vivo rat]
Perovskia scrophulariifolia Bunge	No records found
Persea americana Mill.	Anti-inflammatory [Adeyemi et al., 2002] [a/vivo mouse]; anti-bacterial [Raymond Chia and Dykes , 2010] [vit]; anti-viral [HSV, Aujeszky's disease virus, adenovirus 3] [De Almeida AP et al., 1998] [a/cell line monkey, porcine]; anti-hypertensive [Ojewole et al., 2007] [a/vivo rat]
Persicaria acuminata (Kunth) M.Gómez Syn: *Polygonum acuminatum* Kunth.	Anti-fungal [Derita et al., 2009] [vit]
Persicaria bistorta (L.) Samp. Syn: *Polygonum bistorta* L.	Anti-inflammatory [Klimczak et al., 2017] [h/cell line]
Persicaria hydropiper (L.) Delarbre Syn: *Polygonum hydropiper* (L.) Delarbre	Anti-bacterial, anti-fungal [Hasan et al., 2009] [vit]; anti-inflammatory [Furuta T et al., 1986] [vit]
Persicaria orientalis (L.) Spach. *Syn: Polygonum orientale* L.	Mild anti-bacterial [Islam et al., 2016] [vit]; anti-inflammatory [Gou et al., 2017] [a/vivo rat, mouse]
Persicaria punctata (Elliott) Small Syn: *Polygonum punctatum* Elliott	Anti-fungal [Alves et al., 2001] [vit]; anti-viral [HSV, RSV] [Kott et al., 1998] [h/cell line, a/cell line monkey]
Petiveria alliacea L.	Anti-inflammatory [Lopes-Martins et al., 2002] [a/vivo rat]
Petroselinum crispum (Miller) A.W. Hill	Anti-bacterial [Wong and Kitts, 2006] [vit]; anti-platelet aggregation [Mekhfi et al., 2004] [vit]; anti-hypertensive [Ajebli and Eddouks, 2019] [a/vivo rat]; anti-inflammatory [Al-Howiriny et al., 2003] [a/vivo rat]; immunomodulatory [Yousofi et al., 2012] [a/cell line mouse].<br><br>[Luteolin] reduced inflammation and axonal damage [Hendriks et al., 2004] [a/vivo MS rat].<br>*P. crispum* as souce of luteolin: Manzoor et al., 2017
Peumus boldus Molina	Anti-inflammatory [Lanhers et al., 1991] [a/vivo mouse, a/cell line rat]
Phenakospermum guyannense (A.Rich.) Endl. ex Miq.	No records found
Philodendron camposportoanum G.M.Barroso	No records found
Philodendron imbe Schott ex Kunth	No records found
Phlebodium aureum (L.) J. Sm.	No records found
Phlogacanthus thyrsiformis (Roxb. ex Hardw.) Mabb	Anti-inflammatory, anti-oxidant [Das P et al., 2017]
Phoenix sylvestris (L.) Roxb.	Anti-inflammatory [Mukherjee et al., 2001] [cell line]; anxiolytic [Shajib et al., 2015] [a/vivo mouse]
Pholidota chinensis Lindl.	Anti-inflammatory [Wang J et al., 2006] [cell line]; anti-fatigue [Liu J et al., 2006] [a/vivo mouse]
Phragmanthera usuiensis (Oliv.) M. G. Gilbert	No records found<br>*Phragmanthera austroarabica* neuroprotective [Aldawsari et al., 2017] [a/vivo mouse]
Phthirusa pyrifolia (Kunth) Eichler	Anti-oxidant [Costa RM et al., 2015] [vit]
Phyla scaberrima (Juss. ex Pers.) Moldenke Syn: *Phyla dulcis* (Trevir.) Moldenke	No records found

Phyllanthus acidus (L.) Skeels	Anti-hypertensive [Leeya et al., 2010] [a/vivo rat]
Phyllanthus amarus Schumach. & Thonn.	Memory improvement, anti-neuro-inflammatory [Alagan et al., 2019a,b] [a/vivo mouse, rat]; anti-inflammatory [Decha et al., 2019] [h/c]; anti-HIV [Notka et al.,2004] [h/cell line, vit]; anti-hypertensive, reduced cardiac hypertrophy, improved endothelial function [Yao NA et al., 2020] [a/vivo rat]; anti-convulsant [Tao Z et al., 2020] [a/vivo mouse]
Phyllanthus emblica L. Syn: *Emblica officinalis* Gaertn	Anti-oxidant, anti-inflammatory [Wang H –D et al., 2019] [a/cell line mouse]; anti-bacterial, anti-fungal [Liu X et al., 2009] [vit]; attenuated memory + learning impairments, anti-amyloidogenic [Justin Thenmozhi et al., 2016] [a/vivo rat]; anti-hypertensive [in comb] [Ghaffari et al., 2020] [h/c]; mitochondrial protection [Reddy VD et al., 2009] [a/vivo rat]; anti-aging [Pientaweeratch et al., 2016] [vit]
Phyllanthus fraternus G.L.Webster	Anti-bacterial [Ibrahim AM et al., 2017] [vit]; anti-inflammatory [Chopade et al., 2020] [a/vivo rat]
Phyllanthus niruri L.	Anti-hyperlipidemic [Khanna et al., 2002] [a/vivo rat]
Phyllanthus parvifolius Buch.-Ham. ex D. Don	No records found
Phyllanthus stipulatus (Raf.) Webste	No records found
Phyllanthus ovalifolius Forssk Syn: *Phyllanthus guineensis*	No records found
Phyllanthus urinaria L.	Anti-inflammatory [Fang SH et al., 2008] [cell line, vit]; anti-hypertensive [Lin SY et al., 2008] [a/vivo rat]
Phyllodium pulchellum (L.) Desv. Syn: *Desmodium pulchellum* (L.) Benth.	Anti-inflammatory [Noor S et al., 2013] [a/vivo rat]
Physalis angulata L.	Anti-inflammatory [Rivera et al., 2018] [a/cell line mouse]
Physalis minima L.	Anti-inflammatory [Khan MA et al., 2009] [a/vivo rat, mouse]; anti-bacterial [Gavimath et al., 2012] [vit]
Physalis peruviana L.	Anti-bacterial [Jaca and Kambizi, 2011] [vit]
Phytolacca bogotensis Kunth	Anti-bacterial [Bussmann et al., 2010] [vit]
Phytolacca dioica L.	Anti-bacterial, anti-viral, anti-fungal [Iglesias et al., 2016] [vit]
Phytolacca dodecandra L'Hér.	Anti-bacterial [Ogutu et al., 2012] [vit]
Phytolacca rivinoides Kunth & C.D.Bouché	No records found
Picralima nitida (Stapf) T.Durand & H.Durand	Anti-inflammatory [Duwiejua et al., 2002] [a/vivo rat]; anti-bacterial [Nkere and Iroegbu, 2005; Nwabor et al., 2014] [vit]
Pilea cavaleriei H.Lév.	Anti-bacterial [Ren HC et al., 2018] [vit]
Pilea elegans Gay	No records found
Pilea microphylla (L.) Lieberman	Anti-oxidant, DNA damage protection [Prabhakar et al., 2007; Bansal et al., 2011] [a/vivo mouse, vit]
Piliostigma thonningii (K. Schumacher) Milne-Redh.	Anti-bacterial, anti-inflammatory [Ibewuike et al., 1997] [vit]
Pilocarpus sp.	Anti-inflammatory [Silva et al., 2013] [a/vivo mouse]; anti-bacterial [Santos et al., 1997] [vit]
Pimenta racemosa (Mill.) J.W.Moore	Anti-inflammatory [Garcıa et al., 2004] [a/vivo mouse]; anti-bacterial, anti-fungal [Lowe et al., 2017] [vit]; anti-viral [Meneses et al., 2009] [vit]
Pimpinella anisum L.	Anti-inflammatory [Iannarelli et al., 2018] [a/cell line], Akhtar et al., 2008 [vit]; anti-oxidant, anti-microbial [Gülçın et al., 2003] [vit]
Pinellia ternata (Thunb.) Briet.	Anti-viral [Nagai et a l., 2002] [vit]; anti-bacterial, anti-fungal [Chen JH et al., 2003] [vit]; anti-inflammatory [Lee MY et al., 2013] [a/vivo mouse]
Pinus contorta Dougl. ex Loud.	No records found
Pinus kesiya Royle ex Gordon	No records found<br>*P. sylvestris* anti-bacterial [Mitić et al., 2018] [vit]
Piper aduncum L.	Anti-viral [West Nile Virus} [Radice et al., 2019]; anti-inflammatory [Thao et al., 2016] [a//cell line mouse]
Piper aequale Vahl.	Anti-bacterial [McFeeters et al., 2012] [vit]

Piper arboreum Aubl. Syn: *Piper verrucosum* Sw.	Anti-bacterial, anti-fungal [Nascimento et al., 2015] [vit]; anti–inflammatory [Finato et al., 2018] [h/cell line]
Piper auritum Kunth	Anti-bacterial, anti-fungal [Rahalison et al., 1993] [vit]; anti-venom [Rengifo-Rios et al., 2019] [vit]
Piper betle L.	Anti-inflammatory [Ganguly and Mula, 2007] [a/vivo rat]; immunomodulatory [Kanjwani et al., 2008] [a/vivo mouse]
Piper capense L.f.	Anti-bacterial, anti-fungal, anti-oxidant [Woguem et al., 2013] [vit]
Piper chaba Hunter	Anti-inflammatory [Sireeratawong et al., 2012] [a/vivo rat]; anti-bacterial [Vaghasiya et al., 2007] [vit]
Piper guineense Schum. & Thonn.	Memory improvement, increased neurogenesis [Go et al., 2018] [a/vivo mouse]
Piper hispidum Sw.	Anti-inflammatory [Benitez et al., 2009] [vit]
Piper longum L.	Anti-inflammatory [Kumar A et al., 2009] [a/vivo rat]
Piper nigrum L.	Anti-bacterial, anti-oxidant [Zarai et al., 2013] [vit]; anti-inflammatory [Tasleem et al., 2014] [a/vivo rat]; raises Nrf2 [Choi BM et al., 2007] [a/cell line mouse];
Piper obtusilimbum C. DC.	No records found
Piper peepuloides Roxb.	Anti-bacterial [Unni et al., 2009] [vit]
Piper peltatum L.	Anti-inflammatory, anti-bacterial [Michel et al., 2016] [a/cell line]
Piper sp.	*P. truncatum* vasorelaxant [Raimundo et al., 2009]; *P. sarmentosum* neuroprotective [Chan EW et al., 2019] [a/cell line microglia]
Piper puberulum (Benth.) Seem. Syn: *Piper hongkongense* C. DC	No records found
Piper umbellatum L.	Anti-inflammatory [Iwamoto et al., 2015] [a/vivo mouse]; anti-bacterial [Werka et al., 2007] [vit]
Piptadeniastrum africanum (Hook.f.) Brenan	Anti-bacterial [Brusotti et al., 2013]; anti-inflammatory [Mbiantcha et al., 2017] [a/vivo rat]; anti-ulcer [Ateufack et al., 2015] [a/vivo rat]
Pistacia atlantica Desf.	Anti-AChE, anti-oxidant [Peksel et al., 2010] [vit]<br><br>*P. lentiscus* anti-amyloid ß aggregation [Dhouafli Z et al., 2018] [vit]
Pistacia chinensis subsp. *integerrima* (J. L. Stewart ex Brandis) Rech. f. Syn*: Pistacia integerrima* L.	Anti-bacterial [Bibi et al., 2011] [vit]
Pisum sativum L.	Anti-inflammatory [Utrilla et al., 2015] [a/vivo mouse]; immunomodulatory, anti-inflammatory, anti-oxidant [Ndiaye et al., 2012] [mouse cell line]; anti-PAF [Zia-Ul-Haq et al., 2012] [vit]; anti-hypertensive [Li H et al., 2011b] [h/c, a/vivo]
Pithecellobium jiringa (Jack) Merr.	Anti-bacterial [Bakar et al., 2012] [vit]
Pittosporum mannii Hook. F.	No records found
Pityrogramma chrysophylla (Sw.) Link	No records found
Plantago asiatica L.	Anti-inflammatory [Kim BH et al., 2009] [vit]; reduced demyelination + motor paralysis [in spp. Comb] [Choi JH et al., 2015] [a/vivo MS mouse]; anti-hypertensive [Chou CC et al., 2018] [a/vivo rat]
Plantago lanceolata L.	Wound healing [Kurt et a., 2018] [a/vivo mouse]; anti-bacterial [Kassaw et al., 2018] [vit]
Plantago linearis H.B.K.	No records found
Plantago major L.	Anti-inflammatory [Núñez Guillén et al., 1997] [a/vivo mouse]; anti-microbial [Çitoğlu and Altanlar, 2003 [vit]; anti-hypertensive [Tong et al., 2019] [a/vivo rat]; wound healing [Kartini et al., 2021] [a/vivo rat]
Plantago sericea R. & P.	Anti-bacterial [Bussmann et al., 2011] [vit]
Plantago sparsiflora Michx.	No records found
Platostoma africanum P. Beauv.	No records found

Platycodon grandiflorum (Jacq.) A. DC.	Anti-atherosclerotic [Wu J et al., 2012] [h/cell line]; anti-hypertensive [Lin YC et al., 2017] [a/vivo rat]; anti-hypercholesterolemic, anti- hyperlipidemic [Kim KS et al., 1995] [a/vivo rat]; increased mitochondrial biogenesis [Shi C et al., 2020] [h/cell line]; anti-inflammatory [Kim M et al., 2019] [a/cell line]; immunomodulatory [Pang et al., 2019] [porcine cell line]; anti-apoptotic [Lin YC et al., 2017] [a/cell line rat]
Plectranthus amboinicus (Lour.) Spreng. Syn*: Coleus amboinicus* Lour.	Anti-viral [HIV] [Thayil and Thyagarajan, 2016] [vit]; anti-bacterial [Goncalves TB et al., 2012] [vit]; anti-inflammatory [Gurgel et al., 2009] [a/vivo mouse][anxiolytic [Tiwari DK et al., 2012] [a/vivo rat]
Plectranthus barbatus Andr.	Anti-bacterial [Matu et al., 2003][vit]; anti-viral [HIV], anti-inflammatory, anti-oxidant [Kapewangolo et al., 2013] [vit]
Plectranthus scutellarioides (L.) R.Br.	No records found
Pleioceras barteri Baill.	No records found
Pleurospermum brunonis Benth. ex Cl.	No records found
Plicosepalus curviflorus (Benth. ex Oliv.) Tiegh.	Anti-bacterial [Al-Fatimi et al., 2007] [vit]
Plicosepalus robustus Wiens & Polhill	No records found
Pluchea carolinensis (Jacq.) G.Don	Anti-bacterial [Córdova et al., 2006] [vit]; anti-inflammatory [Rosales Clares et al., 1999] [a/vivo rat]
Pluchea sagittalis (Lam.) Cabr.	Anti-viral [Simoes et al., 1999] [a/cell line monkey]
Plumbago auriculata Lam.	Anti-bacterial [van der Vijver and Lötter, 1971] [vit]; moderately anti-inflammatory [Adebayo et al. 2015] [vit]
Plumbago indica L.	Anti-viral [HIV] [Silprasit et al., 2011] [vit]; anti-oxidant [Eldhose et al., 2013] [vit]
Plumbago zeylanica L.	Raised Nrf2 [plumbagin] [Son et al., 2010] [h/cell line neuron, a/vivo mouse, a/cell line neuron]; anti-inflammatory [Arunachalam KD et al., 2010] [a/vivo rat]; anti-bacterial [Jeyachandran et al., 2009] [vit]
Plumeria rubra L.	Wound healing [Chanda et al., 2011] [a/vivo rat]
Pogostemon heyneanus Benth.	Anti-viral [HIV] [Kusumoto et al., 1995] [vit]; *Pogostemon cablin* PAF inhibition [Tsai et al., 2007] [vit]
Poincianella pluviosa (DC.) L.P.Queiroz	Wound healing, anti-oxidant [Bueno et al., 2016] [a/vivo rat]; anti-inflammatory [Domingos et al., 2019] [a/vivo mouse]
Polyalthia cerasoides (Roxb.) Bedd.	Anti-fungal [Surekha and Ram, 2011] [vit]; anti-bacterial [Kanokmedhakul et al., 2007] [vit]
Polygala acicularis Oliv.	No records found
Polygala elongata Klein ex Willd.	No records found
Polygala paniculata L.	No records found
Polygala tenuifolia Wild.	Autophagy induction [in spp. comb] [Bae et al., 2015] [a/cell line rat neuron]
Polygonatum cirrhifolium (Wall.) Royle	Anti-bacterial, anti-fungal [Xiaoming and Wei, 2007] [vit]
Polygonatum sibiricum Delar ex Redoute	Immunostimulatory [Liu et al., 2020] [a/vivo mouse]; anti-inflammatory [Zhao H et al., 2019] [a/cell line mouse]; anti-hyperlipidemic, anti-atherosclerotic [Yang JX et al., 2015] [a/vivo rabbit]; memory improvement, anti-aging, anti-oxidant [Zheng S, 2020] [a/vivo rat]; *Polygonatum sp*. antibacterial [Li ZT et al., 2017] [vit]; anti-fatigue [Liu S et al., 2009] [a/vivo rat]
Polygonum hissaricum Popov	No records found
Polymnia sonchifolia Poepp.	Anti-bacterial [Kim YS et al., 2005] [vit]
Polypodium vulgare L.	Anti-bacterial [Gleńsk et al., 2019] [vit]; anxiolytic [Ahmed M and Azmat, 2017] [a/vivo rat]
Polyscias fulva (Hiern) Harms	Anti-inflammatory [Sagnia et al., 2014] [vit, h/cell line]
Polyscias guilfoylei (W.Bull) L.H.Bailey	Anti-bacterial, anti-oxidant [Sundu et al., 2015] [vit]

Pongamia pinnata (L.) Pierre or Merr.	Anti-inflammatory [Srinivasan et al., 2001] [a/vivo rat]; wound healing, anti-microbial, anti-oxidant [Dwivedi et al., 2017] [a/vivo rat]
Portulaca grandiflora Hook.	No records found
Portulaca oleracea L.	Anti-viral [HSV [Dong et al., 2010] [vit]; anti-fungal [Oh et al., 2000] [vit]; neuroprotective, anti-PD [Abdel Moneim, 2013] [a/vivo rat]; reduced endothelial dysfunction [Lee AS et al., 2012] [a/vivo mouse]; anti-fatigue [Xu Z et al., 2014] [a/vivo mouse]
Potentilla polyphylla Wall. ex Lehm.	No records found<br>*Potentilla arguta* anti-viral [RSV] [McCutcheon et al., 1995] [vit]
Potentilla freyniana Bornm.	Anti-oxidant [Chen K et al., 2005] [vit]
Pourouma bicolor Mart.	No records found
Pouteria ramiflora (Mart.) Radlk.	No records found
Pradosia sp.	No records found
Premna corymbosa Rottler	Anti-inflammatory [Karthikeyan and Deepa, 2011] [a/vivo rat]
Premna serratifolia L.	Anti-fungal, anti-bacterial [Rajendran, 2010] [vit]
Primula veris L.	Anti-bacterial [Başbülbül et al., 2008] [vit]
Primula vulgaris Huds.	Anti-bacterial [Majid et al., 2014] [vit]
Priva curtisiae Kobuski	No records found
Priva lappulacea (L.) Pers.	No records found
Protium heptaphyllum (Aubl.) March	Anti-bacterial, anti-fungal, anti-oxidant [Bandeira et al., 2006] [vit]; anti-inflammatory [Oliveira FA et al., 2004] [a/vivo rat, mouse]; anxiolytic [Aragão et al., 2006] [a/vivo mouse]
Protium glabrescens Swart	No records found
Prunella vulgaris L.	Anti-inflammatory [Song YW et al., 2007] [h/c]; anti-viral [HSV] [Xu HX et al., 1999] [a/cell line] monkey], [HIV] [Yao XJ et al., 1992] [h/cell line]; anti- amyloidogenic [Lee JE et al., 2017] [vit]; improved cognitive performance, neurogenic [Park SJ et al., 2015] [a/vivo mouse]; immunomodulatory [Fang X et al., 2005] [a/cell line mouse]; anti-hypertensive [Hahm et al., 2009] [a/vivo rat], in comb [Zheng H, 2018] [h/c]
Prunus africana (Hook.f.) Kalkman	Anti-inflammatory, anti-bacterial [Eldeen et al., 2005] [vit]
Prunus amygdalus Batsch Syn: *Amygdalus communis* L	Wound healing [Bouaziz et al., 2014] [a/vivo rat]; memory improvement [Kulkarni et al., 2010] [a/vivo rat]
Prunus avium (L.) L.	Anti-bacterial [Abedini et al., 2020] [vit]
Prunus cerasifera Ehrh.	No records found
Prunus cerasoides Buch.-Ham. ex D.Don	Anti-bacterial, anti-fungal [Arora and Mahajan, 2018] [vit]; anti-inflammatory [Sharma A et al., 2018] [a/cell line mouse]; anti-oxidant [Malsawmtluangi et al., 2014] [vit]
Prunus dulcis Mill. ex Rchb.	Anti-hypercholesterolaemic, anti-hyperlipidemic [Harnafi et al., 2020] [a/vivo mouse]
Prunus erythrocarpa (Nevski) Gilli	No records found
Prunus persica (L.) Batsch	Anti-bacterial, anti-fungal [Aziz S. and Rahman, 2013] [vit]
Prunus spinosa L.	Anti-bacterial, anti-fungal, anti-inflammatory, anti-oxidant [Sabatini et al., 2020] [vit]
Pseudelephantopus spicatus (B.Juss. ex Aubl.) Rohr ex C.F.Baker	Moderately anti-bacterial [Facey et al., 2010] [vit]
Pseuderanthemum latifolium B. Hansen	No records found
Pseudocedrela kotschyi (Schweinf.) Harms	No records found
Pseudognaphalium canescens (DC.) Anderb. Syn: *Gnaphalium canescens*	No records found
Pseudospondias microcarpa (A.Rich.) Engl.	Anti-bacterial [Kisangau et al., 2007] [vit]; anti-viral [HIV] [Masalu et al., 2020] [vit]
Pseudotsuga menziesii (Mirbel) Franco	Anti-inflammatory, immunomodulatory [Han, 2017] [h/cell line]

Psidium acutangulum Mart. ex DC.	No records found
Psidium cattleianum Afzel. ex Sabine	No records found
Psidium guajava L.	Anti-bacterial, anti-fungal [Metwally et al., 2010] [vit]; anti-convulsant [Pushpa et al., 2014] [a/vivo mouse]; anti-oxidant [Penido AB et al., 2017] [vit]; anti-viral [influenza] [Khalil et al., 2019] [h/cell line]; anti-tau [Nguyen and Dai, 2017] [vit]; anti-oxidant [Jiménez-Escrig et al., 2001] [vit]; wound healing [Jayakumari, 2018] [a/vivo rat]; anxiolytic [Sahoo S et al., 2020] [a/vivo mouse].<br>[morin] anti- α-synucleinopathic [Caruana et al., 2011] [vit]; anti-ATP synthase [Chinnam et al., 2010] [vit]<br>*P. guajava* as source of morin: Kapoor et al., 2012
Psidium guineense Sw.	No records found
Psittacanthus calyculatus (DC.) G. Don	Vasorelaxant [Rodríguez-Cruz ME et al., 2003] [rat aortic rings]
Psophocarpus scandens (Endl.) Verdc.	No records found
Psoralea sp.	*Psoralea glandulosa* anti-bacterial [Erazo et al., 1997b] [vit]; anti-inflammatory [Backhouse et al., 1996] [a/vivo guinea pig]
Psychotria elata (Sw.) Hammel Syn: *Cephaelis elata Sw.*	No records found
Psychotria umbellata Thonn. Syn: *Psychotria calva* Hiern	No records found
Pteleopsis hylodendron Mildbr.	Anti-bacterial, anti-oxidant [Ngounou et al., 2001] [vit]
Pterocarpus angolensis DC.	Anti-bacterial [Abubakar and Majinda, 2016] [vit]
Pterocarpus indicus Willd.	Anti-bacterial [Khan MR and Omoloso, 2003] [vit]
Pterocarpus rohrii Vahl.	Anti-bacterial [Kloucek et al., 2007] [vit]
Pterocaulon alopecuroidum Chodat	No records found<br>*Pterocaulon alopecuroides* anti-viral [HSV] [Silveira et al., 2009] [vit]
Pterodon emarginatus Vogel	Anti-inflammatory [Carvalho et al., 1999] [a/vivo rat, mouse]
Pteromonnina pterocarpa (Ruiz & Pav.) B. Eriksen Syn: *Monnina pterocarpa* R. & P.	No records found
Pueraria montana var. lobata (Willd.) Sanjappa & Pradeep Syn: *Pueraria thunbergiana*	Neurogenic [Zhao J et al., 2015] [a/cell line rat neuron]; reduced microglial activation + neuroinflammation [Lim HS et al., 2018] [a/vivo mouse, a/cell line mouse microglia]; neurotrophic [Zhu G et al., 2010] [a/vivo rat PD]
Pulicaria mauritanica Batt.	No records found
Pulicaria undulata (L.) C.A.Mey.	Anti-PD [reduced α-synuclein gene expression], anti-inflammatory, anti-oxidant, neuroprotective [Issa et al., 2020] [a/vivo rat]; anti-bacterial [Ali et al., 2012] [vit]
Punica granatum L.	Anti-viral [HSV, Sindbis virus, polio] [Mouhajir et al., 2011] [a/cell line monkey], [influenza] [Haidari et al., 2009] [a/cell line canine, chick], [Moradi et al., 2017] [vit], [HIV] [Neurath et al., 2004] [h/cell line]; anti-inflammatory [Ghavipour et al., 2017] [h/c]; anti-oxidant [Singh RP et al., 2002] [vit]; improved memory and learning [Cambay et al., 2011] [a/vivo rat]; anti-convulsant [Mehrzadi et al., 2015] [a/vivo mouse]; neuroprotective, anti-PD [Kujawska et al., 2020] [a/vivo PD rat];.anti-hypertensive [Asgary et al., 2013] [h/c]; improved lipid profile [Kojadinovic et al., 2017] [h/c]; gut microbiota modulation [Singh et al., 2021] [h/c], [Larrosa et al., 2010] [a/vivo rat].<br>[urolithin] anti-neuro-inflammatory, autophagy modulation [Velagapudi et al., 2019] [h/cell neuron, a/cell neuron]
Pupalia lappacea (L.) Juss.	Anti-venom [Molander et al., 2014] [vit]
Puya hamata L.B. Sm.	No records found
Pycnobotria nitida Benth	No records found

Pyracantha crenulata (D. Don) M.Roem.	Anti-bacterial [Saklani and Chandra, 2014] [vit]
Qualea parviflora Mart.	Anti-bacterial, anti-inflammatory, anti-ulcerogenic [Mazzolin et al., 2010, 2013] [a/vivo rat]
Quassia africana (Baill.) Baill.	Anti-viral [Apers et al., 2002] [vit]
Quassia amara L.	Anti-inflammatory [Verma et al., 2010] [a/cell line mouse]
Quassia undulata (Guill. & Perr.) D.Dietr.	AChE inhibition, anti-oxidant [Odubanjo et al., 2018b] [vit]
Quercus humboldtii Bonpl.	No records found
Quinchamalium majus Brongn. (ind)	Anti-bacterial [Gu JQ et al., 2004] [vit]
Ranunculus nubigenus Kunth ex DC.	No records found
Raphia vinifera P.Beauv.	No records found
Raphanus raphanistrum subsp. *sativus* (L.) Domin Syn: *Raphanus sativus* L.	Anti-inflammatory [Park HJ and Song, 2017] [a/cell line mouse]; anti-bacterial [Iyda et al., 2019] [vit]
Rauvolfia mannii Stapf	No records found
Rauvolfia tetraphylla L.	Anti-microbial [Shariff et al., 2006] [vit]
Rauvolfia verticillata (Lour.) Baill.	Anti-hypertensive [Feng M et al., 2014 [a/vivo rat]
Rauvolfia vomitoria Wennberg	Anti-bacterial, anti-oxidant [Erasto et la., 2011] [vit]
Reseda villosa Coss.	No records found
Rhamnus alaternus L.	Anti-bacterial [Kosalec et al., 2013] [vit]; anti-viral [HSV] [Mouhajir et al., 2001] [a/cell line monkey]
Rhamnus californica Esch. Syn: *Frangula californica* (Eschsch.) A.Gray	No records found
Rhamnus virgatus Roxb. [unresolved]	No records found
Rhaphidophora pertusa (Roxb.) Schott	Anti-inflammatory [Linnet et al., 2010] [avivo rats]
Rhaphithamnus spinosus (Juss.) Mold. (ind)	No records found
Rheum australe D. Don.	Anti-bacterial [Hussain F et al., 2010] [vit]; *R. officinale* anti-viral [HSV, influenza] [Sydiskis et al., 1991] [vit]
Rhipsalis baccifera (J.S.Muell.) Stearn	No records found
Rhizophora mangle L.	Anti-inflammatory [Marrero et al. 2006] [vit]; anti-bacterial [Melchor et al., 2001] [vit]
Rhodiola tibetica (Hook.f. & Thomson) S.H.Fu	No records found
Rhodobryum giganteum (Hook.) Par.	No records found
Rhododendron anthopogon D. Don	Anti-bacterial, anti-fungal [Innocenti et al., 2010] [vit]
Rhododendron arboreum Sm.	Anti-oxidant [Acharya et al., 2011] [vit]; anti-inflammatory [Agarwal SS and Kalpana, 1988] [a/vivo rat]
Rhus chinensis Mill. Syn: *Rhus semialata* Linn.	[Tannic acid] anti- α-synucleinopathic [Ono and Yamada, 2006] [vit]; anti-prion [Kocisko et al., 2003] [mouse neural cell line] *R. chinensis* as source of tannin: Djakpo and Yao, 2010
Rhus vulgaris Meikle	Anti-bacterial, anti-fungal [Boily and Van Puyvelde, 1986] [vit]; anti-viral [Cocksackie, HSV] [Vlietinck et al., 1995] [a/cell line monkey]
Rhynchosia hirta (Andrews) Meikle & Verdc	No records found
Rhynchospora colorata (L.) H.Pfeiff. Syn: *Cyperus kyllingia* Endl.	Anti-bacterial, anti-fungal, [Pyne et al., 2011] [vit]
Rhynchospora nervosa (Vahl) Boeckeler	Anti-bacterial, anti-fungal, anti-oxidant [Bezerra et al., 2019] [vit]

Ribes magellanicum Poir.	Anti-inflammatory [Burgos-Edwards et al., 2019] [h/cell line]
Rhytidocaulon macrolobum Lavranos	No records found
<u>*Ribes* sp.</u>	*Ribes* spp. anti-oxidant [Benvenuti et al., 2004] [vit]; *R. fasciculatum* neuroprotective, memory improvement, raised BDNF [Park E et al., 2019] [a/vivo rat]; *Ribes nigrum* anti-fatigue [Tung et al., 2019]; *Ribes nigrum* anti-inflammatory [Lee Y and Lee, 2019] [h/cell line, a/cell line mouse]
Rinorea anguifera Kuntze [unresolved]	No records found
Rivea hypocrateriformis (Desr.) Choisy	Anti-bacterial, anti-fungal [Venkata et al., 2012] [vit]
Rodgersia aesculifolia Batal	Anti-bacterial [Shi Y et al., 2009] [vit]
Rorippa sarmentosa (Sol. ex G.Forst. ex DC.) J.F.Macbr.	Anti-inflammatory [Dunstan et al., 1997] [a/vivo rat]
Rosa × *alba* L.	Anti-bacterial [Gochev et al., 2010] [vit]
Rosa × *centifolia* L.	Anti-bacterial [Bayoub et al., 2010] [vit]
Rosa canina L.	Anti-inflammatory [Warholm et al., 2003] [h/c]; anti-depressant [Farajpour et al., 2017] [a/vivo mouse; anti-viral [cold] [Winther et al., 2018] [h/c]
Rosa ecae Aitch.	No records found
Rosa fedtschenkoana Regel	No records found
Rosa macrophylla Lindl.	No records found
Rosa phoenicia Boiss.	No records found
<u>*Rosa* sp.</u>	Anti-oxidant [Roman et al., 2013] [vit]; anti-inflammatory [Zhang GQ et al., 2008] [a/vivo mouse]
Rosmarinus officinalis L. Syn: *Salvia rosmarinus* Spenn.	Memory improvement [Nematolahi et al., 2018] [h/c], [Perry NS et al., 2018] [h/c]; memory improvement + AChE inhibition [Ozarowski et al., 2013] [a/vivo rat]; anti-oxidant [Aruoma et al., 1996] [vit]; anti-bacterial, anti-fungal [Bozin B et al., 2007] [vit]; anti-viral [Hep A] [Battistini et al., 2019] [vit]; anti-hypercholesterolemic [Belmouhoub et al., 2018] [a/vivo mouse]; wound healing [de Araujo et al., 2017] [a/vivo mouse].<br><br>[Rosmarinic acid] anti-amyloidogenic [Hamaguchi et al., 2009] [a/vivo mouse]; inhibition of tau aggregation [Cornejo et al., 2017] [vit]; extended survival, relieved motor function deficits, reduced neuronal loss, delayed disease onset + symptom progression [Seo JS et al., 2015; Shimojo et al., 2010] [a/vivo ALS mouse]; anti- α-synucleinopathic [Caruana et al., 2011] [vit]; improved subconjunctival healing [Ferreira et al., 2014] [a/vivo rabbit]; anti-inflammatory [Rocha et al., 2015] [a/vivo rat].<br><br>[Naringin] reduced cognitive and mitochondrial dysfunction [Kumar A et al., 2010] [a/vivo mouse]; alleviation of neurogenic deficits [Mirza et al., 2021] [a/vivo mouse].<br><br>[Carnosol] promotes microglial switch to immunomodulatory phenotype, promoting myelin regeneration; reactive Th17 cell suppression [Li et al., 2018] [a/vivo MS mouse].<br><br>[Carnosic acid] reduced p-tau + Aβ plaques, rescued synaptic loss, improved learning + memory [Lipton et al., 2016] [a/vivo AD mouse]<br>*R. officinalis* as source of rosmarinic acid + naringin: Zheng and Wang, 2001
*Rotheca myricoide*s (Hochst.) Steane & Mabb Syn: *Clerodendrum myricoides* (Hochst.) R. Br. ex Vatke	Anti-bacterial, anti-fungal [Njeru et al., 2016] [vit]; anti-venom [Molander et al., 2014] [vit];

Rourea coccinea subsp. *coccinea* (Schumach. & Thonn.) Benth.	No records found
Rubia cordifolia L.	Anti-viral [HIV] [Sabde et al., 2011] [h/cell line]; anti-inflammatory [Ghosh et al., 2010] [a/vivo rat]; anti-PAF [Tripathi et al., 1993] [a/cell rabbit platelet]; memory improvement [Chitra and Pavan Kumar, 2009] [a/vivo mouse]
Rubus buergeri Miq. Syn: *Rubus moluccanus* auct.	Anti-inflammatory [Yang EJ et al., 2009] [a/cell line mouse]; anti-oxidant [Bakar et al., 2016] [vit]
Rubus caesius L.	No records found
Rubus foliolosus Hal csy. [unresolved]	No records found
Rubus glaucus Benth.	Anti-oxidant [Alarcón-Barrera et al., 2018] [h/cell line]
Rubus pinnatus Willd.	No records found
Rubus robustus C. Presl.	No records found
<u>*Rubus* sp.</u>	*R. jamaicensis* anti-inflammatory; anti-oxidant [Bowen-Forbes et al., 2010] [vit]; *Rubus* spp. increased mitochondrial biogenesis, reduced lipid accumulation + lipid-induced inflammation [Zhao L et al., 2018] [a/vivo mouse]; *Rubus* spp. anti-inflammatory [Cuevas-Rodríguez et al., 2010] [a/cell line mouse]
Ruellia patula Jacq. *Dipteracanthus patulus* (Jacq.) Nees	Anti-bacterial, anti-fungal [Ramadevi et al., 2016] [vit]
Ruellia tuberosa L.	Anti-inflammatory [Alam MA et al., 2009] [a/vivo rat, mouse]; anti-bacterial [Arirudran et al., 2011] [vit]
Rumex abyssinicus Jacq	Anti-inflammatory, anti-bacterial, anti-viral [influenza] [Getie et al., 2003] [a/cell line canine]
Rumex crispus L.	Anti-inflammatory [Im et al., 2014] [a/cell line mouse]; anti-bacterial [Borchardt et al., 2008] [vit]
Rumex hastatus D. Don	Anti-microbial [Hussain F et al., 2010] [vit]; anti-inflammatory [Singh S et al., 2013] [a/vivo rat]
Rumex nepalensis Spreng.	Anti-bacterial, anti-fungal [Yadav S et al., 2011] [vit]; anti-viral [HIV] [Cos et al., 2002b] [h/cell line]
Rumex usambarensis (Dammer) Dammer	Anti-bacterial, anti-fungal [Boily and Van Puyvelde, 1986] [vit]
<u>*Ruta chalepensis* L.</u>	Anti-inflammatory [Al-Said et al., 1990] [a/vivo rat; anti-bacterial [Tedila and Shanmugam, 2019] [vit]
Ruta montana L.	No records found
Ruta graveolens L.	Anti-inflammatory [Raghav et al., 2006] [a/cell line mouse]; anti-convulsant [Keihanian et al., 2012] [a/vivo mouse]
Rydingia integrifolia (Benth.) Scheen & V.A.Albert Syn: *Otostegia integrifolia* Benth.	Anti-bacterial, anti-fungal [Tadesse et al., 2011] [vit]
Saba comorensis (Bojer ex A.DC.) Pichon	No records found
Saccharum officinarum L.	DNA damage protection, anti-oxidant [Abbas et al., 2014] [vit]
Saccharum spontaneum L.	Anti- microbial [Padalia et al., 2017] [vit]
Salacia impressifolia (Miers) A.C.Sm.	No records found
Salix acmophylla Boiss.	Anti-bacterial [Ali MR et al., 2017] [vit]
Salix denticulata Andersson	No records found
Salix humboldtiana Willd.	No records found
Salsola imbricata Forssk. Syn: *Salsola baryosma* (Schult.) Dandy	No records found
Salvadora persica L.	Anti-inflammatory [Ibrahim et al., 2011] [a/vivo rat]; anti-bacterial, anti-fungal [Al-Bayati and Sulaiman, 2008] [vit]
Salvertia convallariodora A. St.-Hil.	No records found
Salvia aegyptiaca L.	Anti-inflammatory [Al-Yousuf et al, 2002] [a/vivo mouse]; anti-oxidant [Mamache et al., 2020] [vit]

Salvia apiana Jeps.	Anti-bacterial [Cordova-Guerrero et al., 2016] [vit]
Salvia ayavazensis Kunth	No records found<br>*S. miltiorrhiza* anti-amyloidogenic [Wang Q et al., 2013] [h/cell line neuron]; anti-apoptotic [Zhang et al., 2009] [a/cell line rat neuron]; anti-inflammatory, memory improvement [Maione et al., 2018] [a/vivo AD mouse]; immunomodulation [Kang et al., 2000] [a/cell line mouse]; MS T cell downregulation, improved symptoms [Yan et al., 2016] [a/vivo MS rat]
Salvia gilliesi Benth	No records found
Salvia hians Royle ex Benth.	Anti-bacterial [Melkani et al., 2011] [vit]
Salvia merjamie Forssk.	Anti-bacterial [Gebrehiwot et al., 2009] [vit]
Salvia officinalis L; and *Salvia officinalis* subsp. *lavandulifolia* (Vahl) Gams Syn: *Salvia rosmarinifolia* Hort. ex G. Don	Cognitive improvement, AChE inhibition [Perry et al., 2003] [h/c, a/vivo rat], [Akhondzadeh et al., 2003] [h/c]; anti-hypertensive [Ferreira et al., 2006] [vit]; anti-hyperlipidemic [Kianbakht et al., 2011] [h/c]; anti-inflammatory [Mansourabadi et al., 2016] [a/vivo mouse, rat]; anti-bacterial [Horiuchi et al., 2007] [vit]; oestrogenic activity [Sabry et al., 2022] [a/vivo rat]
Salvia palifolia Kunth	No records found
Salvia sagittata Ruiz & Pav.	Anti-bacterial [Bussmann et al., 2011] [vit]
Salvia scutellarioides Kunth	Anti-hypertensive, vasodilatory [Ramírez JH et al., 2006, 2007] [a/vivo rat]
Salvia tomentosa Mill.	Anti-bacterial, anti-oxidant [Tepe et al., 2005] [vit]
Salvia tubiflora Sm.	No records found
Salvia verbenaca L.	Wound healing [Guaouguaou et al., 2018] [a/vivo rat]
Sambucus australis Cham. & Schlecht	Anti-inflammatory, anti-oxidant, anti-bacterial [Benevides Bahiense et al., 2017] [a/cell line mouse]
Sambucus canadensis L. Syn: *Sambucus mexicana* C.Presl ex DC.	Moderately anti-bacterial [Holetz et al., 2002] [vit]
Sambucus ebulus L.	Wound healing [Süntar et al., 2010] [a/vivo mouse, rat]; anti-bacterial [Rodino et al., 2015] [vit]; anti-inflammatory [Jabbari et al., 2016] [h/c]
Sambucus javanica Reinw. ex Blume	Anti-viral [HCoV-NL63] [Weng et al., 2019] [a/cell line monkey]; immunomodulatory [Putra and Rifa'I, 2019] [a/vivo mouse]
Sambucus nigra L.	Anti-viral [influenza] [Zakay-Rones et al., 2004[[h/c], [Hawkins et al., 2019] [h/c]; anti-bacterial [Hearst et al., 2010] [vit]; anti-inflammatory [Lin P et al., 2019] [h/cell line], [Olejnik et al., 2015] [a/cell line mouse]; inhibition of microglial activation [Simonyi et al., 2015] [a/cell line mouse]
Sambucus peruviana H.B.K.	Anti-inflammatory [Roxana et al., 2018] [a/vivo rat]; anti-bacterial [Hernández et al., 2000] [vit]
Sandoricum koetjape (Burm.f.) Merr.	Anti-inflammatory [Rasadah et al., 2004] [a/vivo mouse]
Sanguisorba minor Scop.	Anti-viral [HIV] [Bedoya et al., 2001] [h/cell line]
Sansevieria roxburghiana Schult. & Schult.f.	Anti-microbial [Philip et al., 2011] [vit]
Sansevieria trifasciata Prain	Anti-inflammatory, anti-oxidant [Pinky et al., 2020] [vit]; anti-bacterial [Sikder et al., 2011] [vit]
Santalum insulare Bertero ex A.DC	Anti-bacterial, anti-fungal[Butaud et al., 2007] [vit]
Santalum lanceolatum R. Br.	Anti-bacterial [Palombo and Semple, 2001] [vit]
Santiria trimera (Oliv.) Aubrev.	Anti-bacterial [Martins et al., 2003] [vit]
Santolina rosmarinifolia L.	Anti-bacterial [Chibani et al., 2005] [vit]
Sapium laurifolium (A.Rich.) Griseb.	No records found
Sapium marmierii Huber.	No records found
Saraca asoca (Roxb.) J.J.de Wilde	Anti-bacterial [Athiralakshmy et al., 2016] [vit]
Sarcostemma acidum (Roxb.)	No records found
Satureja bachtiarica Bunge	No records found<br>*S. montana* AChE inhibition [Vladimir-Knežević et al, 2014] [vit]

Satureja pulchella (H.B.K.) Briquet	No records found
Satureja thymbra L.	Anti-viral [HSV] [Loizzo et al., 2008] [vit]<br>*S. hortensis* anti-amyloidogenic [Ishigaki et al., 2013] [vit]
Saurauia sp.	*Saurauia vulcani,* wound healing [Ginting et al., 2018] [a/vivo rat]
Saussurea costus (Falc.) Lipsch. Syn: *Saussurea lappa* (Decne.) Sch. Bip.	No records found<br>*Saussurea pulvinata* + *Selaginella tamariscina* scavenging ROS + anti-apoptopic [Wang et al., 2010]
Scaevola taccada (Gaertn.) Roxb.	Anti-fungal [Suthiwong et al., 2017] [vit]
Schefflera morototoni (Aubl.) Maguire, Steyerm. & Frodin	No records found<br>*Schefflera heptaphylla* anti-viral [RSV] [Li Y. et al., 2005] [vit]
Schima wallichii (DC.) Korth.	Anti-bacterial, anti-fungal, anti-inflammatory [Dewanjee et al., 2008, 2009] [vit, a/vivo rat]
Schinus areira L.	Anti-inflammatory [Davicino et al., 2010] [a/cell line]
Schinus molle L.	Anti-inflammatory [Yueqin et al., 2003] [a/vivo mouse]
Schinus terebinthifolius Raddi	Anti-inflammatory [Rosas et al., 2015] [a/vivo mouse]; anti-bacterial [Martinez et al., 1996] [vit]
Schizostachyum lumampao (Blanco) Merr.	No records found
Schkuhria pinnata (Lam.) Kuntze	Anti-microbial [Bussmann et al., 2008] [vit]
Schumanniophyton magnificum (K.Schum.) Harms	Anti-bacterial [Tchouya et al., 2014] [vit]
Schwenckia americana L.	Anti-venom [Molander et al., 2014] [vit]; anti-inflammatory [Nwabunike et al., 2014] [a/vivo rat]
Scleria gaertneri Raddi	No records found
Scleria scrobiculata Nees & Meyen	No records found
Scolymus hispanicus L.	Anti-inflammatory [Kandil et al., 2020] [vit]
Scoparia dulcis L.	Anti-inflammatory [De Farias Freire et al., 1993] [a/vivo rat, mouse]; moderate anti-amyloidogenic [Kleinrichert and Alappat, 2019] [vit]; anti-viral [HSV] [Hayashi et a., 1988] [vit]
Scrophularia ningpoensis Hemsl.	Anti-bacterial [Li J et al., 2009] [vit]; anti-hypertensive [Zhang CC et al., 2016] [a/vivo rat]
Scutellaria baicalensis Georgi	Anti-fungal [Wong and Tsang, 2009] [vit]; anti-viral [influenza] Seong , 2018] [h/cell line, a/cell line canine], RSV [Ma SC et al., 2002] [h/cell line], dengue virus [Zandi et al., 2013] [a/cell line monkey]; inhibits α-synuclein fibril formation [Zhu M et al., 2004] [vit]; anti-inflammatory [Arweiler et al., 2011; Orzechowska et al., 2014] [h/c]
Scutia myrtina (Burm.f.) Kurz	Anti-inflammatory, anti-bacterial, anti-fungal [Kritheka et al., 2008] [a/vivo rat, vit]
Sechium edule (Jacq.) Sw.	Anti-hypertensive, anti-endothelial dysfunction [Trejo-Moreno et al., 2018] [a/vivo mouse]; anti-epileptic [Mumtaz et al., 2012] [a/vivo rat]; anti-bacterial [Ordoñez et al., 2003] [vit]
Securidaca longipedunculata Fresen.	Anti-inflammatory [Ojewole, 2008] [a/vivo rat, mouse]
Semecarpus anacardium L.f.	Anti-inflammatory [Selvam and Jachak, 2004] [vit]; wound healing [Lingaraju GM et al., 2012] [a/vivo rat]
Senecio genisianus Cuatr.	No records found
Senecio pseudotites Grieseb.	No records found
Senecio rufinervis DC.	Anti-inflammatory [Mishra et al., 2010] [a/vivo mouse]
Senecio scandens Buch.-Ham. ex D. Don	Anti-viral [HIV] [Chang RS, Yeung, 1988] [vit]
Senna alata (L.) Roxb. Syn: *Cassia alata* L.	Anti-microbial [Igwe and Onwu, 2015] [vit]; anti-inflammatory [Palanichamy and Nagarajan, 1990] [a/vivo mouse]; anti-bacterial and anti-fungal [Doughari and Okafor, 2007] [vit]

Senna crotalarioides (Kunth) H.S.Irwin & Barneby	Anti-inflammatory [García-Rodríguez et al., 2011] [a/vivo rat, mouse]
Senna hirsuta (L.) H.S. Irwin and Barneby Syn: *Cassia hirsuta* L.	Anti-venom [Prashar et al., 2015] [a/vivo mouse, rat, sheep]; anti-bacterial [Coolborn and Bolatito, 2010] [vit]
Senna italica Mill.	Anti-inflammatory, anti-bacterial [Mamba et al., 2016] [vit]
Senna notabilis (F. Muell.) Randell	No records found
Senna pallida (Vahl) H.S. Irwin & Barneby	No records found
Senna petersiana (Bolle) Lock Syn*:* *Cassia petersiana* Bolle	Anti-bacterial [Gatsing and Adoga, 2007] [vit]
Senna reticulata (Willd.) H.S. Irwin and Barneby	Anti-bacterial [Lopez et al., 2001] [vit]
Senna sophera (L.) Roxb Syn: *Cassia socotrana* Serrato	Anti-viral [HSV, influenza] [Mothana et al., 2006] [a/cell line monkey, canine]
Senna tora (L.) Roxb. Syn*:* *Cassia tora*	Anti-bacterial, anti-oxidant [Uddin SN et al., 2008] [vit]; anti-inflammatory [Samanta et al. et al., 2018] ; anti-oxidant, anti-apoptotic [Ravi et al., 2018] [h/cell neural cell line]
Sesbania sesban (L.) Merr.	Anti-bacterial, anti-fungal [Hossain et al., 2007] [vit]; anti-inflammatory [Dande et al., 2010] [a/vivo rat]
Shorea tumbuggaia Roxb.	No records found
Sida acuta Burm. f.	Anti-bacterial [Karou et al., 2006] [vit]; anti-inflammatory [Oboh IE and Onwukaeme, 2005] [a/vivo mouse, rat]; anti-venom [Otero R et al., 2000] [vit]; ameliorated neurotoxicity [Owoeye and Salami, 2017] [a/vivo rat]]
Sida cordata (Burm. f.) Borss. Waalk.	Anti-oxidant [Subramanya et al., 2015] [vit]
Sida rhombifolia L.	Moderately anti-bacterial [Assam Assam et al., 2010] [vit]
Sida tenuicarpa Vollesen	Moderately anti-bacterial [Amugune et al., 2017] [vit]
Sida urens L.	Anti-bacterial [Konaté et al., 2013] [vit]
Sideritis hirsuta L.	No records found
Sideritis perfoliata subsp. athoa (Papan. & Kokkini) Baden Syn: *Sideritis athoa* Papan. & Kokkini	Anti-inflammatory [Charami et al., 2008] [vit]
Sideritis raeseri Boiss. & Heldr.	Anti-bacterial [Stagos et al., 2012] [vit]
Sigesbeckia orientalis L.	Anti-neuroinflammatory [Chu J et al., 2018] [a/vivo mouse]
Silene macrosolen Steud. ex A. Rich.	No records found
Silybum marianum (L.) Gaertn.	Anti-inflammatory [Aghazadeh et al., 2011] [a/vivo rat]; memory improvement, anti-oxidant [Lu P et al., 2009] [a/vivo rat]; reduced a-synuclein, anti-aging [Srivastava et al., 2017] [a/vivo *C. elegans*]; anti-amyloidogenic via reduced APP gene expression [Yaghmaei et al., 2014] [a/vivo rat]
Simaba ferruginea A. St.-Hil.	Anti-ulcerogenic, anti-inflammatory [de Souza Almeida et al., 2011] [a/vivo rat, mouse]
Simarouba amara Aubl.	Anti-bacterial [Hegde V et al., 2019] [vit]
Simarouba glauca DC.	Anti-fungal [Mikawlrawng et al., 2014] [vit]
Sinomenium acutum (Thunb.) Rehder & E.H. Wilson	Anti-inflammatory [Kim TW et al., 2018] [a/vivo rat]
Siparuna guianensis Aubl.	Anti-inflammatory [Conegundes et al., 2020] [a/vivo mouse, a/cell line mouse]; anti-bacterial, moderately anti-viral [Lopez et al., 2001] [a/cell line monkey, vit]
Sisyrinchium tinctorium Kunth	AChE inhibition [Calderón et al., 2010] [vit]
Skimmia laureola (DC.) Decne.	Anti-bacterial, anti-fungal [Shah WA et al., 2013] [vit]
Smilax aspera Wall.	Anti-fungal [Belhouchet et al., 2008] [vit]; anti-inflammatory [Amira et al., 2012] [a/vivo mouse]
Smilax corbularia Kunth	Anti-bacterial [Itharat, 2010] [vit]
Smilax irrorata Mart. ex Griseb.	No records found
Smilax aristolochiifolia Mill. Syn: *Smilax medica* M.Martens & Galeotti	Anti-inflammatory, anti-hypertensive [Amaro et al., 2014] [a/vivo mouse]<br>*S. glabra* anti-inflammatory [Jiang J and Xu, 2003] [a/vivo rat]

Smilax rotundifolia L.	No records found
Smilax rufescens Griseb.	Anti-bacterial [Suffredini et al., 2006] [vit]
Smilax siphilitica Humb. & Bonpl. ex Willd.	No records found
Smilax sp.	*Smilax china, Smilax guianensis* anti-inflammatory [Shu et al., 2006; Kim et al., 2020] [a/cell line mouse]
Socratea exorrhiza (Mart.) H.Wendl.	No records found
Solanecio mannii (Hook.f.) C. Jeffrey Syn*: Crassocephalum mannii* (Hook.f.) Milne-Redh.	Anti-bacterial, anti-fungal [Mbosso et al., 2010] [vit]; [Hegazy et al., 2008] [vit]
Solanum aethiopicum L.	Anti-inflammatory [Anosike et al., 2015] [a/vivo rat]; anti-hypertensive [Mamyrbekova-Bekro et al., 2013] [a/vivo rabbit]
Solanum caricaefolium Rusby	No records found
Solanum crispum Ruiz & Pav.Syn: *Solanum ligustrinum* Loddigges	Anti-inflammatory [Delporte et al., 1998] [a/vivo guinea pig]
Solanum dasyphyllum Schumach. & Thonn.	Neuroprotective, maintains mitchondrial integrity Obade et al., 2018] [vit]; anti-bacterial, anti-fungal [Ajayi and Ojelere, 2014] [vit]
Solanum huallagense Bitter.	No records found
Solanum inaequilaterale Merr.	No records found
Solanum incanum L.	Anti-viral [HIV] [Shy et al., 2000] [vit]; anti-inflammatory [Mwonjoria et al., 2011] [a/vivo mouse]
Solanum i*ndicum* L. Syn: *Solanum anguivi* Lam.	Maintains mitochondrial integrity, reduce ROS [Elekofehinti et al., 2015] [a/cell rat neuron]; anti-viral [HBV] [Yin HL et al., 2013] [vit], Coxsackie virus [Vlietinck et al., 1995] [a/cell line monkey]
Solanum lycocarpum A. St.-Hil.	Anti-inflammatory, antioxidant, anti-bacterial [Da Costa GA et al., 2015] [a/vivo mouse, vit]
Solanum lycopersicum Syn: *Lycopersicon esculentum* Mill.	Anti-inflammatory, anti-oxidant [Li H et a., 2014] [a/vivo rat, vit]; anti-hypertensive [Paran et al., 2009] [h/c]; Nrf2 activation [Lian and Wang, 2008] [h/cell line]; metal chelation [Tito et al., 2011] [a/cell line mouse]
Solanum marginatum L. f.	Anti-bacterial [Colmenares and Corredor, 2011] [vit]; anti-hypertensive [Vidrio et al., 1988] [a/vivo rat]
Solanum melongena L.	Anti-oxidant [Hanson et al., 2006] [vit]; anti-inflammatory [Sun J et al., 2014] [a/cell line mouse]; anti-hypertensive [Yamaguchi et al., 2019] [a/vivo rat]
Solanum mite Ruiz et Pav.	No records found
Solanum nigrum Linn.	Anti-inflammatory [Gu XY et al., 2018] [a/cell line mouse]; anti-bacterial [Matasyoh et al., 2014] [vit]; anti-viral [Javed T et al., 2011] [h/cell line]; reduced memory impairment [Ogunsuyi et al., 2020] [a/vivo *Drosophila*]; immunomodulation [Razali et al., 2016] [a/vivo mouse]
Solanum peruvianum L. Syn*:* *Lycopersicon peruvianum* (L.) Mill.	No records found
Solanum sisymbriifolium Lam.	Anti-viral [HSV] [Simoes et al., 1999] [a/cell line monkey]; neurogenic [Lecanu et al., 2011] [a/vivo rat, a/cell line mouse]; anti-microbial; anti-oxidant [Gupta VK et al., 2014] [vit]
Solanum somalense Franch. in Revoil	No records found
Solanum torvum Sw.	Anti-inflammatory [Ndebia et al., 2007] [a/vivo rat]; anti-viral [HSV] [Arthan D et al., 2002] [vit]; anti-bacterial, anti-fungal [Balachandran et al., 2012] [vit]; anti-platelet aggregation, anti-hypertensive [Nguelefack et al., 2008] [a/vivo rat]; anti-oxidant [Loganayaki et al., 2010] [vit]

Solanum tuberosum L.	Anti-bacterial, anti-oxidant [Bontempo et al., 2013] [vit]; anti-hypertensive [Vinson et al., 2012] [h/c]; anti-apoptotic [Asokan et al., 2018] [a/cell line mouse]; anti-platelet aggregation [Pepe A et al., 2016] [h/cell erythrocyte]
Solanum uporo Dunal [unresolved] Syn: *Solanum viride* G.Forst. ex Biehler	No records found
*Solenomelus segethi (*Phil.) Kuntze Syn: *Susarium segethii* Phil. (ind)	No records found
Solenostemma oleifolium (Nectoux) Bullock & E.A.Bruce ex Maire	No records found
Solidago californica Nutt.	No records found
Solidago chilensis Meyen	Anti-inflammatory [da Silva et al., 2010] [h/c]
Soliva sessilis Ruiz and Pav.	No records found
Sonchus asper Wulf.	Anti-bacterial, anti-fungal [Khan RA et al., 2010] [vit]
Sonchus oleraceus L.	Anti-inflammatory [Vilela FC et al., 2010] [a/vivo rat]; anti-bacterial, antioxidant [Jimoh and Afolayan, 2011] [vit]; anti-viral [HSV, Sindbis virus] [Mouhajir et al., 2001] [a/cell line monkey]
Sorbus aucuparia L.	No records found
Sorghum bicolor (L.) Moench Syn: *Holcus bicolor* L.	Anti-inflammatory [Burdette et al., 2010] [a/vivo mouse]; anti-oxidant [Kamath et al., 2004] [vit]; anti-viral [HSV], anti-bacterial [Camargo Filho et al., 2008] [a/cell line monkey, vit]; immunomodulatory [Benson et al., 2013] [vit]
*Soymida febrifuga (*Roxb.) A.Juss.	Anti-inflammatory [Diwan and Singh, 1993] [a/vivo mouse, rat]
Sparrmannia africana L. f.	No records found
Spartium junceum L.	Anti-inflammatory [Menghini et al., 2006] [a/vivo rat]
Spathodea campanulata P. Beauv.	Anti-viral [HIV] [Niyonzima et al., 1999] [vit]; anti-bacterial, anti-oxidant [Akharaiyi et al., 2012] [vit]
Spermacoce princeae (K. Schum.) Verdc.	No records found
Spermacoce verticillata L. Syn: *Borreria verticillata* (L.) G. Mey Rub WP	Anti-bacterial [Maynart et al., 1980; Neto et al., 2002] [vit]; anti-inflammatory [Abdullahi-Gero et al., 2014] [a/vivo rat, mouse]
Spilanthes acmella (L.) L. Syn: *Blainvillea acmella* (L.) Philipson	Anti-inflammatory [Chakraborty et al., 2004] [a/vivo rat]; anti-oxidant, anti-microbial [Prachayasittikul et al., 2009] [vit]
Spilanthes oppositifolia (Lam.) D'Arcy	No records found
Spinacia oleracea L	Anti-inflammatory [Garg et al., 2009] [a/vivo rat]
Spiranthera odoratissima A. St.-Hil.	Anti-inflammatory [Barbosa et al., 2012] [a/vivo mouse]
Spiranthes sinensis (Pers.) Ames BM	Anti-inflammatory [Shie et al., 2015] [a/vivo, a/cell line mouse]
Spondias dulcis Parkinson Syn: *Spondias cytherea* Sonn	Anti-bacterial, anti-oxidant, thrombolytic [Islam SM et al., 2013] [vit]
Spondias pinnata (L.f.) Kurz	Anti-inflammatory, anti-fungal [Li R et al., 2020] [a/cell line mouse, vit]
Spondias purpurea L.	Anti-bacterial [Cáceres A et al., 1993b] [vit]
Sporobolus indicus (L.) R. Br.	No records found
Stachys obliqua Waldst. & Kit.	No records found
Stachys lavandulifolia Vahl	Anti-bacterial [Shahnama et al., 2015] [vit]
Stachys pilifera Benth	Mild anti-microbial [Farjam et al., 2011] [vit]
Stachytarpheta cayennensis (Rich.) Vahl	Anti-bacterial [Okoye et al., 2010] [vit]; anti-inflammatory [Schapoval et al., 1998] [a/vivo rat]
Stachytarpheta elatior Schrad.	No records found

Stachytarpheta jamaicensis (L.) Vahl	Anti-viral [HIV] [Woradulayapinij et al., 2005] [vit]; anti-inflammatory [Sulaiman et al., 2009] [a/vivo rat, mouse]; anti-hypertensive [Idu ME et al., 2006] [a/vivo rabbit]
Staudtia kamerunensis var. gabonensis (Warb.) Fouilloy	No records found
Steganotaenia araliacea Hochst.	Anti-viral [rhinovirus] [Beuscher et al., 1994] [h/cell line]
Stellaria media (L.) Vill.	Anti-inflammatory [Oyebanji et al., 2012] [a/vivo rat, mouse]
Stemona tuberosa Lour.	Anti-bacterial [Lin LG et al., 2008] [vit]
Stenotaphrum secundatum (Walter) Kuntze	No records found
Stephania abyssinica (Dillon. & A. Rich.) Walp.	No records found<br>*Stephania cephalantha* anti-viral [HIV] [Ma CM et al., 2002] [h/cell line].<br>*Stephania tetrandra* [tetrandrine] prion protein inhibition [Kocisko et al., 2003] [a/cell line mouse]; reduced ocular hypertension [Huang P et al., 2011] [a/vivo rat]; anti-platelet aggregation [Kim HS et al., 1999] [h/ cell platelet]; reduced microglial activaton, anti-inflammatory [Xue et al., 2008] [a/cell line rat microglia]; increased BDNF [Gao S et al., 2013] [a/vivo rat]
Sterculia guttata Roxb	No records found<br>*Sterculia setigera* anti-venom [Molander et al., 2014] [vit]
Sterculia setigera Delile	Anti-inflammatory [Henneh et al., 2018] [a/vivo rat]
Sterculia tragacantha Lindl.	Anti-inflammatory [Udegbunam et al., 2011] [a/vivo mouse]
Sterculia urens Roxb.	Moderately anti-bacterial [Padil et al., 2015] [vit]
Stereospermum kunthianum Cham.	Anti-epileptic [Ching et al., 2009] [a/vivo rat]
Stevia rebaudiana (Bertoni) Bertoni	Vasodilatory [Melis, 1996] [a/vivo rat; anti-bacterial, anti-fungal [Jayaraman S et al., 2008] [vit]
Streblus asper Lour.	Anti-bacterial [Wongkham et al., 2001] [vit]
*Streblus dimepate (*Bureau) C.C. Berg	No records found
Strobilanthes crispa Blume	No records found
Strobilanthes cusia (Nees) Kuntze Syn: *Baphicacanthus cusia* (Nees) Bremek.	Anti-bacterial [Shahni and Handique, 2015] [vit]
Strychnos pseudoquina A.St.-Hil.	Wound healing [Sarandy et al., 2017] [a/vivo rat]; anti-inflammatory, anti-viral [Boff et al., 2016] [vit]
Stryphnodendron adstringens (Mart.) Coville	Anti-inflammatory [Lima JC et al., 1998] [a/vivo rat]; anti-fungal [Fiori et al., 2013] [vit]
Stryphnodendron obovatum Benth.	Anti-fungal, anti-oxidant [Sanches et al., 2005]; anti-inflammatory [Henriques et al., 2016] [h/cell line]
Sudamerlycaste gigantea (Lindl.) Syn: *Lycaste gigantea* Lindl.	No records found
Swertia ciliata (D.Don) C.B.Clarke	Anti-bacterial, anti-fungal [Saeed et al., 1998] [vit].<br>[mangiferin] anti-oxidant [Khanal et al., 2015] [vit
Swertia racemosa (Griseb.) Wall. ex C.B.Clarke	[mangiferin] anti-inflammatory, anti-oxidant [Khanal et al., 2015] [vit]
Symmeria paniculata Benth.	No records found
Symphonia sp.	*Symphonia globulifera* as anti-bacterial [Lenta et al., 2004] [vit]
Symphyotrichum puniceum (L.) Á.Löve & D.Löve	No records found
Symphytum officinale L.	Anti-inflammatory [Petersen et al., 1993] [h/c], [Seigner et al., 2019] [h/cell line]
Synsepalum cerasiferum (Welw.) T.D.Penn	No records found
Synedrella nodiflora (L.) Gaertn.	Anti-bacterial, anti-fungal [Bhogaonkar et al., 2011] [vit]
Syzygium aromaticum (L.) Merr. & L.M.Perry Syn: *Eugenia caryophyllus* (Spreng.) Bullock & S.G.Harrison	Anti-bacterial, anti-oxidant [El-Maati et al., 2016] [vit]; anti-inflammatory [Daniel et al., 2009] [a/vivo rat]

Syzygium cumini (L.) Skeels	Anti-inflammatory [Muruganandan et al., 2001] [a/vivo rat], [Kumar A et al., 2008] [a/vivo rat], [Siani et al., 2013] [a/vivo mouse], [Jain A et al., 2010] [a/vivo rat]; anti-bacterial [Mohamed AA et al., 2013] [vit]
Syzygium jambos (L.) Alston Syn: *Eugenia malaccensis* Blanco	Anti-bacterial [Djipa et al., 2000] [vit]; anti-inflammatory [Sharma R et al., 2013] [vit]
Syzygium malaccense (L.) Merr. & L.M.Perry	Anti-bacterial [Bouzada et al., 2009] [vit]
Tabebuia aurea (Silva Manso) Benth. & Hook.f. ex S.Moore	Anti-bacterial, anti-fungal [Barbosa-Filho et al., 2004] [vit]
Tabebuia rosea DC.	Anti-inflammatory [Franco et al., 2013] [a/vivo mouse]
Tabernaemontana crispa L.	No records found
Tabernaemontana divaricata (L.) R.Br. ex Roem. & Schult. Syn: *Ervatamia coronaria* (Jacq.) Stapf	Anti-bacterial [Thombre et al., 2013] [vit]
Tabernaenontana elegans Stapf	No records found
Tacca leontopetaloides (L.) Kuntze	Anti-bacterial [Habila et al., 2011] [vit]
Tadehagi triquetrum (L.) H.Ohashi	No records found
Tagetes pusilla Kunth	Anti-inflammatory [De las Heras et al., 1998] [vit]
Tagetes erecta L.	Anti-inflammatory [Chatterjee et al., 2009] [a/vivo mouse]
Tagetes terniflora Kunth Syn: *Tagetes graveolens* L'Hér.	Anti-fungal [Galvez et al., 2018] [vit]
Tamarindus indica L.	Anti-venom [Molander et al., 2014] [vit]; anti-bacterial [Doughari, 2006] [vit]; anti-inflammatory [Bhadoriya et al., 2012] [a/vivo rat], [Rao PS et al., 2019] [h/c]; anti-hypercholesterolemic [Martinello et al., 2012] [a/vivo hamster]; neuroprotective, neurogenic [Tadtong et al., 2013] [a/cell line mouse]
Tamarix aphylla (L.) H.Karst.	Anti-inflammatory, anti-oxidant, wound healing [Yusufoglu and Alqasoumi, 2011] [a/vivo rat]
Tapinanthus globiferus (A. Rich) Van Tiegh	Anti-bacterial [Kabiru et al., 2017] [vit]
Taraxacum mongolicum Hand.-Mazz.	Anti-viral [HIV] [Xu et al., 1996] [vit], HBV [Jia et al., 2014] [h/cell line, a/cell line duck]; *Taraxacum coreanum* anti-oxidant, neuroprotective [Yoon et al., 2017] [a/cell line mouse neuron]
Tephrosia purpurea (L.) Pers.	AChE inhibition [Arjun et al., 2017] [vit]; anti-epileptic [Asuntha et al., 2010] [a/vivo rat]
Tephrosia villosa (L.) Pers.	No records found
Terminalia argentea Mart.	No records found<br>*Terminalia fagifolia* anti-venom [Tribuiani et al., 2017] [a/isolated phrenic nerve-muscle mouse + chick]
Terminalia arjuna (Roxb. ex DC.) Wight & Arn.	Cardioprotective [Dwivedi and Agarwal, 1994] [h/c]; congestive heart failure therapy [Bharani et al., 1995] [h/c]; anti-dyslipidemic, anti-oxidant [Chander R et al., 2004] [a/vivo rat, hamster, vit]; anti-hypertensive [Nammi et al., 2003] [a/vivo dog]; anti-platelet aggregation [Malik et al., 2009] [h/cell line platelet]; anti-inflammatory [Biswas M et al., 2011] [a/vivo rat]
Terminalia catappa L.	Anti-hypertensive [Braga et al., 2007] [vit]; anti-bacterial [Babayi et al., 2004] [vit]
Terminalia glabrata var. *brownii* Fosberg & Sachet	No records found
Terminalia sericea Burch. ex DC.	Anti-viral [HIV] [Chauke et al, 2016] [vit]; anti-bacterial [Tshikalange et al., 2005] [vit]
Tessaria integrifolia Ruiz & Pav.	Anti-inflammatory [Peluso et al., 1995] [vit]
Tetradenia riparia (Hochst.) Codd	Anti-bacterial, anti-fungal [Van Puyvelde et al., 1986]

Tetradium ruticarpum (A.Juss.) T.G.Hartley Syn: *Euodia rutaecarpa* (Juss.) Benth.	Moderately anti-bacterial [Wang XX et al., 2013] [vit]
Tetraena simplex (L.) Beier & Thulin Syn: *Zygophyllum simplex* L. Mant. Pl.	Anti-inflammatory [luteolin] [Qiao et al., 2012] [a/vivo rat]; [Tsilioni et al., 2015] [h/c]
Tetragonia crystallina L'Herit	No records found<br>*Tetragonia tetragonoides* anti-inflammatory [Ko EY et al., 2017] [a/cell line mouse]
Tetrapleura tetraptera (Schumach. & Thonn.) Taub	Memory improvement [Odubanjo et al., 2018a] [a/vivo rat]
Tetrorchidium didymostemon (Baill.) Pax & K.Hoffm.	Anti-bacterial [Tchouya et al., 2015] [vit]
Teucrium mascatense Boiss	Anti-bacterial, anti-fungal [Hisham et al., 2006] [vit]
Teucrium polium L.	Wound healing [Meguellati et al., 2019] [a/vivo rat]; anti-inflammatory [Capasso et al., 1983]; anti-hyperlipidemic [Rasekh et al., 2001] [a/vivo rat]<br>*Teucrium* spp. AChE inhibition, anti-oxidant [Vladimir-Knežević et al, 2014] [vit];
Thalictrum foliolosum DC.	Anti-bacterial, anti-fungal, anti-oxidant [Pandey G et al., 2018] [vit]
Thapsia garganica L.	Anti-inflammatory [Elmezogi et al., 2012] [a/vivo mouse]
Theobroma cacao L.	Enhanced mitochondrial biogenesis + function [Nogueira et al., 2011; Moreno-Ulloa et al., 2013] [a/vivo mouse, bovine]; anti-inflammatory [Zeng H et al., 2011] [h/cell line]; cognitive improvement, anti-hypertensive [Taubert et al., 2007; Desideri et al., 2012] [h/c]; anti-aging, extended lifespan [Baiges and Arola, 2016] [*Saccharomyces cerevisiae* model]
Thespesia populnea (L.) Sol.ex Corrêa	Anti-bacterial, anti-fungal [Shekshavali and Hugar, 2012] [vit]
Thonningia sanguinea Vahl	Free-radical-scavenging [Gyamfi et al., 1999] [a/vivo mouse, rat, vit]; anti-bacterial [N'guessan et al., 2007a,b] [vit]
Thymbra capitata (L.) Cav. Syn: *Coridothymus capitatus* (L.) Rchb.f.	Anti-inflammatory, AChE inhibition, anti-oxidant [Carrasco et al., 2016] [vit]
Thymbra spicata L.	Anti-bacterial [Baydar et al., 2004] [vit]; anti-fungal [Marković et al., 2011] [vit]; anti-viral [HSV] [Duran NI et al., 2012] [h/cell line]
Thymus linearis Benth.	Anti-inflammatory [Qadir et al., 2016] [a/vivo mouse]; anti-viral [HSV] [Rajbhandari et al., 2009] [a/cell line monkey]; anti-bacterial, anti-oxidant [Hussain AI. et al., 2013] [vit]
Thymus longicaulis C.Presl	Anti-viral [HSV] [Matta et al., 2007] [vit]; anti-bacterial [Vladimir-Knežević et al., 2012] [vit]
Thymus satureioides Coss.	Anti-viral [HSV, SINV] [Mouhajir et al., 2001] [a/cell line monkey]; anti-inflammatory, anti-oxidant [Ismaili et al., 2004] [a/vivo mouse, vit]
Thymus serpyllum L. Syn: *Thymus ciliatus* Lam.	Anti-fungal [Aziz S et al., 2010]; anti-bacterial, anti-oxidant [Nikolić et al., 2014] [vit]
Thymus vulgaris L.	Anti-amyloidogenic [Ishigaki et al., 2013] [vit]; anti-bacterial, anti-oxidant [Nikolić et al., 2014]; anti-hypertensive [Kensara et al., 2013] [a/vivo rat]; anti-fungal [Giordani et al., 2004] [vit]; anxiolytic [Komaki et al., 2016] [a/vivo rat].<br>[Thymol, carvacrol] immunomodulatory [Gholijani et al., 2015; Gholijani and Amirghofran, 2016] [a/vivo mouse].<br>[Thymol] anti-platelet aggregation [Okazaki et al., 2002] [vit].<br>[Luteolin] anti-inflammatory, reduced axonal damage [Hendriks et al., 2004] [a/vivo MS rat]
Thymus zygioides Griseb.	Anti-bacterial [Azaz et al., 2014] [vit]
Tilia tomentosa Moench	Anti-bacterial, anti-viral, anti-inflammatory, anti-oxidant [Frezza et al., 2020] [vit]
Tinospora caffra (Miers) Troupin	No records found
Tinospora cordifolia (Thunb.) Miers	Anti-bacterial [Mishra P et al., 2014] [vit]; memory improvement [Bairy et al., 2004] [h/c]; neurogenic [Sharma and Kaur, 2018]

	[a//cell line rat neuron]; anti-inflammatory [Sannegowda et al., 2015] [a/vivo rat] ; immunomodulatory [Bishayi et al., 2002] [a/vivo rat]; anti-platelet aggregation [Lugun et al., 2018] [a/vivo rat]; improved learning and memory] [Agarwal A, et al., 2002] [a/vivo rat]; anti-PD [Kosaraju et al., 2014] [a/vivo rat]; anxiolytic [Dhingra and Goyal, 2008] [a/vivo mouse]; anti-oxidant [Stanely Mainzen Prince and Menon, 2001] [a/vivo rat]
Tinospora rumphii Boerl.	Anti-inflammatory [Sy,1995] [a/vivo rat]
Tinospora sinensis (Lour.) Merr.	Moderately anti-inflammatory [Li RW et al., 2003] [vit]
Tinospora smilacina Benth.	Anti-inflammatory [Li RW et al., 2003] [vit]
Tiquilia paronychioides (Phil.) A.T. Richardson	No records found
Toddalia asiatica (L.) Lam.	Anti-bacterial, anti-fungal [Duraipandiyan and Ignacimuthu, 2009] [vit]; anti-viral [influenza] [Lu SY et al., 2005]; anti-HIV [Rashid et al., 1995] [vit]; anti-inflammatory [Kumagai et al., 2018] [a/cell line mouse]; anti-platelet aggregation [Tsai et al., 1998] [vit]
Toona ciliata M.Roem.	Anti-bacterial [Chowdhury R et al., 2003] [vit]; anti-ulcer [Malairajan et al., 2007] [a/vivo mouse, rat]
Tournefortia hirsutissima L. Syn: *Heliotropium verdcourtii* Craven	Anti-bacterial [Lozano et al., 2013] [vit]; anti-viral [HIV] [Antoun et al., 1999] [vit]; anti-inflammatory [Hurtado-Díaz et al., 2019] [a/cell line mouse]
Trachycarpus fortunei (Hook.) H.Wendl.	Anti-bacterial [Ahmed S et al., 2017] [vit]
Trachyphrynium braunianum (K.Schum.) Baker	No records found
Tradescantia pallida (Rose) D.R.Hunt Syn: *Setcreasea purpurea* Boom	Anti-bacterial [Tan JB et al., 2014] [vit]
Tradescantia sp.	*T. fluminensis* anti-inflammatory [Alaba et al., 2014] [vit]
Tradescantia spathacea Sw. Syn: *Rhoeo spathacea* (Sw.) Stearn	Anti-inflammatory [Pérez, 1996] [a/vivo mouse, rat]
Tradescantia zebrina Heynh.	Anti-bacterial [Tan JB et al., 2014] [vit]; anti-inflammatory [Alaba et al., 2014] [vit]
Treculia africana Decne. ex Trécul	Anti-bacterial [Ogbonnia et al., 2008] [vit]
Trema orientalis (L.) Blume	Anti-inflammatory [Uddin et al., 2009] [a/vivo mouse]; anti-oxidant, anti-bacterial [Uddin, 2008] [vit]
Trevesia palmata (Roxb. ex Lindl.) Vis.	Anti-inflammatory, thrombolytic [Sayeed et al., 2014] [vit]
Trianthema portulacastrum L.	Anti-hyperlipidemic [Anreddy et al., 2010] [a/vivo rat]; anti-fungal [Nawaz et al., 2001] [vit]; anti-inflammatory [Falade et al., 2019] [a/vivo rat]; wound healing, anti-oxidant [Yadav E et al., 2017] [a/vivo rat]; improved cognitive impairment [Yadav E et al., 2019] [a/vivo mouse]; anti-venom [Meenatchisundaram, 2007] [a/vivo mouse]
Tribulus cistoides L.	No records found
Tribulus terrestris L.	Anti-inflammatory [Baburao et al., 2009] [a/vivo rat]
Trichanthera gigantea (Bonpl.) Nees	No records found
Trichilia heudelotti Planch. ex Oliv.	No records found<br>*Trichilia emetica* Hsp90 modulation [Dal Piaz et al., 2012] [vit]
Trichilia prieureana Juss.	Anti-bacterial [Kuglerova et al., 2007] [vit]
Trichodesma indicum (L.) Sm.	Anti-bacterial [Perianayagam et al., 2012] [vit]
Trichopus zeylanicus Gaertn.	Immunostimulatory [Singh B et al., 2005] [a/vivo rat, mouse]
Trichospermum sp.	*Trichospermum galeottii* immunomodulatory [Barberena et al., 2004] [vit]
Trichuriella monsoniae (L.f.) Bennet	No records found

Tridax procumbens (L.) L.	Anti-inflammatory, anti-oxidant [Jachak et al., 2011] [a/vivo rat]; anti-inflammatory [Berlin Grace et al., 2019] [a/vivo mouse]; vasorelaxant [Salahdeen et al., 2015] [rat isolated artery]
Trifolium repens L.	No records found<br>*Trifolium pratense* anti-inflammatory [Fu X et al., 2019] [a/vivo mouse]
Tripodanthus flagellaris Cham. & Schltdl.	Anti-bacterial [Alcaráz et al., 2015] [vit]
Tristemma leiocalyx Cogn.	No records found
Triticum aestivum L. Syn: *Triticum sativum* L.	Moderately anti-bacterial [Jeong EY et al., 2010] [vit]; anti-inflammatory [Liu J et al., 2018] [a/cell line mouse]
Triumfetta althaeoides Lam.	No records found
Trixis californica Kellogg	No records found
Tropaeolum majus L.	Anti-inflammatory, anti-bacterial, anti-fungal [Butnariu and Bostan, 2011] [a/vivo mouse, vit]; anti-hypertensive [Gasparotto Junior et al., 2011] [a/vivo rat];
Tropaeolum tuberosum Ruiz & Pav.	Anti-bacterial, anti-fungal [Ticona et al., 2020] [vit]; anti-inflammatory [Apaza et al., 2019] [vit]
Tussilago farfara L.	PAF inhibition [Hwang et al., 1987] [a/vivo rat, a/cell line rabbit platelet]; NF-κB inhibition, reduced microglial activation [Lim HJ et al., 2015] [a/cell line mouse microglia]; anti-microbial [Kokoska et al., 2002] [vit]
Tylophora indica (Burm.f.) Merr.	Anti-venom, anti-oxidant, anti-inflammatory [Sakthivel et al., 2013] [a/vivo mouse, vit]
Tynanthus cognatus (Cham.) Miers	No records found
Typha angustifolia L.	Anti-inflammatory, anti-ulcer [Fruet et al., 2012] [a/vivo rat]; thrombolytic [Umesh et al., 2014] [vit]
Uncaria africana G.Don	No records found
Uncaria guianensis (Aubl.) J.F.Gmel.	Anti-inflammatory [Piscoya et al., 2001] [h/c, a/cell line]; anti-viral [Dengue] [Mello CD et al., 2017] [h/cell line]
Uncaria sp.	*U.perrottetii* anti-bacterial, anti-fungal [Vital and Rivera, 2009] [vit].<br><br>*U. rhynchophylla* anti-neuro-inflammatory, inhibited microglial activation, anti-amyloid aggregation, ameliorated neurogenic impairment [Shin et al., 2018] [a/vivo mouse]; anti-PD, Hsp modulation [Lan et al., 2018] [a/vivo mouse,h/neural cell line]; vasodilatory [Loh et al., 2017] [a/ isolated aorta rat]; anti-PD, autophagy induction [Lu JH et al., 2012; Chen LL et al., 2014] [h+a/neuronal cell lines]; anti-apoptotic [Xian et al., 2014] [a/vivo rat].<br><br>*U. sinensis* anti-hypertensive [Aisaka et al., 1985] [a/vivo dog]
Uncaria sessilifructus Roxb.	Anti-hypertensive [Le XT et al., 2020] [vit]
Uncaria tomentosa (Willdenow ex Roemer & Schultes) DC.	Anti-inflammatory [Piscoya et al., 2001] [a/cell line], [Mur E et al., 2002] [h/c]; anti-amyloid, anti-tau, memory improvement, anti-inflammatory [Snow et al., 2019] [a/vivo rat, mouse]; anti-viral [Dengue] [Reis et al., 2008] [h/cell line]; DNA repair [Sheng et al., 2001] [h/c]; immunomodulatory [Th1 to Th2] [Domingues et al., 2011] [a/vivo mouse]
Uraria picta (Jacq.) Desv. ex DC.	AChE inhibition, anti-oxidant [Odubanjo et al., 2013] [vit]; anti-bacterial, anti-fungal [Rahman MM et al., 2007] [vit]
Urena lobata L.	Anti-inflammatory [Rajagopal et al., 2019] [vit]
Urera baccifera (L.) Gaudich. Ex Wedd.	Anti-bacterial [Onofre SB, Herkert, 2012] [vit]; anti-viral [HSV] [Martins et al., 2009] [vit]; anti-inflammatory {Badilla et al., 1999b] [a/vivo rat]
Urera caracasana (Jacq.) Gaudich. ex Griseb.	No records found
Urochloa mutica (Forssk.) T.Q.Nguyen Syn: *Brachiaria mutica* (Forsk.) Stapf	No records found

Urtica dioica L.	Anti-inflammatory [Riehemann et al., 1999] [h/cell line], [Jacquet et al., 2009] [in spp. comb] [h/c]; anti-viral [SARS-CoV] [Kumaki et al., 2011] [a/cell line monkey]; anti-bacterial, anti-oxidant [Gülçin et al., 2004] [vit]; [anti-hypertensive [Samaha et al., 2019] [h/c]; anxiolytic [Patel SS et al., 2018] [a/vivo mouse]
Urtica flabellata Kunth	No records found
Urtica magellanica A. Jussieu ex Poiret	No records found
Uvaria brevistipitata De Wild.	No records found<br>*Uvaria angolensis* anti-viral [HIV] [Ngoutane Mfopa et al., 2018] [vit]
Vaccinium arctostaphylos L.	Anti-hypertensive [Khalili et al., 2011] [a/vivo rat]
Vaccinium myrtillus L.	*Vaccinium myrtillus* anti-bacterial [Kokoska et al., 2002] [vit]; anti-inflammatory [Lietti et al., 1976] [a/vivo rat]; reduced obesity-associated hypertension, anti-inflammatory [Mykkänen et al., 2014] [a/vivo mouse]; glaucoma patients improved visual acuity]Shim et al., 2012] [h/c]; memory improvement [Borowiec et al., 2019] [a/vivo rat, [a/cell rat neuron]; gut microbiota modulation, reduced body weight and cholesterol [Liu J. et al., 2021] [a/vivo mouse]; enhanced microglial clearance of Aβ, improved cognitive deficits [Zhu Y et al., 2008] [a/vivo AD mouse].<br><br>*V. angustifolium* memory improvement [Krikorian et al., 2010] [h/c].<br><br>*V. angustifolium, V. uliginosum* anti-oxidant [Grace et al., 2013] [vit]; *V. uliginosum* reduced α-synuclein expression [Maulik et al., 2018] [a/vivo *C. elegans*].<br><br>*Vaccinium* spp. memory improvement [Whyte et al., 2018] [h/c]; cognitve improvement [Miller MG et al., 2018] [h/c]; memory improvement + raised BDNF [Williams et al., 2008] [a/vivo rat]; anti-inflammatory [Lau et al, 2007] [a/cell line mouse microglia]; increased Hsp 70 activity [Galli et al., 2006] [a/vivo rat]; reduced retinal inflammation [Song Y et al., 2016] [a/vivo rat +rat retina]; reduced apoptosis, anti-oxidant [Huang WY et al., 2018] [h/cell line rpe]
Valeriana jatamansi Jones ex Roxb.	Anti-bacterial, anti-fungal, anti-inflammatory [Agnihotri et al., 2011] [vit, a/vivo mouse]
Valeriana officinalis L.	Anti-neuroinflammatory [Rodríguez-Cruz A et al., 2019] [a/vivo PD mouse]; anti-convulsive [Hiller KO, Zetler, 1996] [a/vivo mouse]; anxiolytic [Gharib et al., 2015] [h/c]
Vangueria apiculata K.Schum.	No records found
Vasconcellea pubescens A. DC.	Anti-bacterial [Vega-Gálvez et al., 2010] [vit]
Vellozia sp.	*Vellozia flavicans anti*-venom [Tribuiani et al., 2014] [a/isolated phrenic nerve-diaphragm mouse]
Verbascum thapsus L.	Anti-inflammatory [Speranza et al., 2010] [h/cell line]; anti-bacterial [Turker and Camper, 2002] [vit]; anti-viral [HSV] [McCutcheon et al., 1995] [vit]
Verbascum virgatum Stokes	No records found<br>*Verbascum chionophyllum* anti-inflammatory [Tatli et al., 2008] [a/vivo mouse]
Verbena litoralis Kunth	Anti-bacterial [Bussmann et al., 2010] [vit]; anti-inflammatory [Lima RD et al., 2020] [a/vivo rat]
Verbena officinalis L.	Anti-inflammatory [Deepak and Handa, 2000] [a/vivo rat]; anti-bacterial [Ahmed D et al., 2012] [vit]
Vernonanthura ferruginea (Less.) H.Rob	Anti-ulcerogenic [Oliveira et al., 2011] [a/vivo mouse]
Vernonanthura patens (Kunth) H.Rob.	Anti-fungal [Manzano et al., 2013] [vit]
Vernonia adoensis Sch. Bip. ex Walp. Var.	Anti-bacterial [Chitemerere and Mukanganyama, 2011] [vit]

Vernonia amygdalina Delile.	Anti-bacterial [Akinpelu, 1999] [vit]; anti-hypertensive [Taiwo IA et al., 2010] [a/vivo rat]; anti-inflammatory [Madzuki et al., 2019] [a/vivo rat]
Vernonia colorata (Willd.) Drake	Anti-microbial [Rabe et al., 2002]
Vestia foetida (Ruiz & Pav.) Hoffmanns.	No records found
Viburnum grandiflorum Wall. ex DC.	Anti-bacterial [Uddin et al., 2013] [vit]
Vigna peduncularis (Kunth) Fawc. & Rendle.	No records found
Vigna racemosa (G. Don) Hutch	No records found
Vigna unguiculata (L.) Walp.	Anti-inflammatory [Ojwang et al., 2015] [h/cell line]; immunomodulatory [Adjei-Fremah et al., 2016] [/cell line bovine]
Viguiera lanceolata Britton Syn: *Viguiera mandonii* Sch.Bip. ex Rusby	No records found
Viola arguta Humb. & Bonpl. ex Schult.	No records found<br>*Viola yedoensis* anti-influenza [Liu MZ et al., 2014] [vit]; *Viola betonicifolia* anti-inflammatory [Muhammad et al., 2012] [a/vivo mouse]
Viola canescens Wall.	Anti-bacterial [Prasad, 2014] [vit]
Viola odorata L.	Anti-inflammatory [Drozdova and Bubenchikov, 2005] [a/vivo mouse]; anti-viral :SARS- CoV] [Schwarz et al., 2014] [a/isolated *Xenopus* oocytes]; anti-bacterial [Gautam and Kumar, 2012] [vit]; anti-hypertensive [Samaha et al., 2019] [h/c]; decreased total cholesterol, anti-dyslipidemic] [Siddiqi et al., 2012] [a/vivo rat]
Viola pilosa Blume	Anti-bacterial [Panni and Bakht, 2018] [vit]
Virectaria major (K.Schum.) Verdc.	No records found
Virola elongata Benth.) Warb.	Anti-ulcerogenic [de Almeida et al., 2019] [a/vivo mouse, rat]; *Virola oleifera* anti-atherogenic [Coutinho et al., 2017] [a/vivo mouse]
Viscum coloratum (Kom.) Nakai	Anti-inflammatory [Hwang TL et al., 2006] [h/cell line]; PAF inhibition, anti- platelet aggregation [Chu W et al., 2008] [a/vivo rat, a/cell myocyte rat]; anti-fatigue, potentiating mitochondrial activity [Jung HY et al., 2012] [a/vivo mouse]
Vismia japurensis Rchb.f.	No records found<br>*Vismia rubescens* anti-bacterial, anti-fungal [Tamokou et al., 2009] [vit]
Vitellaria paradoxa C.F.Gaertn. Syn: *Butyrospermum paradoxum* (Gaertner f.) Hepper	Memory improvement [Foyet et al., 2016] [a/vivo rat]; anti-inflammatory [Akihisa et al., 2010] [a/vivo rat]
Vitex agnus-castus L.	Anti-inflammatory [Choudhary et al., 2009] [vit]; anti-bacterial [Ghannadi et al., 2012] [vit]
Vitex doniana Sweet	Anti-bacterial [Kilani, 2006] [vit]
Vitex madiensis Oliv.	No records found
Vitex negundo L.	Anti-bacterial [Panda et al., 2009] [vit]; anti-convulsant [Tandon and Gupta, 2005] [a/vivo mouse]; anti-venom [Alam and Gomes, 2003] [a/vivo mouse, vit]; neuroprotective, reduced cognitive impairment [Rahiman et al., 2015] [a/vivo rat]
Vitex peduncularis Wall. ex Schauer	Anti-bacterial [Prusti, 2008] [vit]
Vitex simplicifolia Oliv.	Anti-bacterial, wound healing [Ouoba et al., 2012] [vit]
Vitis vinifera L.	Anti-inflammatory [Aouey et al., 2016] [a/vivo mouse]; anti-hypertensive [Bomfim et al., 2019] [a/vivo rat]; memory improvement [Bensalem et al., 2019] [h/c]; anti-bacterial [Parekh and Chanda ,2006] [vit]; anti-viral [HSV, parainfluenza] [Orhan DD et al., 2009] [a/cell line monkey]; anti-oxidant, anti-lipid peroxidation, AChE inhibition [Local Food-Nutraceuticals Consortium, 2005] [vit].

	[Polydatin] attenuated dopaminergic neurodegeneration [Chen Y et al., 2015] [a/vivo rat]; reduced neuronal loss [Shindler et al., 2010] [a/vivo MS mouse]; raises Nrf2 [Ren et al., 2011] [a/vivo rat].<br><br>[Resveratrol] reduced CSF amyloid β [Turner et al., 2015] [h/c]; amyloid β remodelled into non-toxic structures [Ladiwala et al., 2010] [vit]; enhanced mitochondrial biogenesis [Mancuso et al., 2014] [a/vivo ALS mouse]; modulation of mitochondrial dysfunction + elevated Hsp 70 [Youssuf et al., 2009] [a/vivo rat]; improved mitochondrial function + biogenesis via raised AMPK, SIRT1 + mRNA PGC-1α gene expression [Ferretta et al., 2014] [h/cell line]; reversal of abnormal energy metabolism [Wei et al., 2011] [a/vivo mouse Batten disease]; improved visual acuity [Richer et al., 2014] [h/c]; reduced intraocular pressure [Pirhan et al., 2016] [a/vivo rat glaucoma]; improved neurological function [Yiu et al., 2015] [h/c Friedreich's ataxia patients]; immunomodulatory: modulating intestinal flora-mediated Th17/Tregs and Th1/Th2, decreased pro-inflammatory cytokines [Dou et al., 2019] [a/vivo ischaemic mouse]; enhanced neurogenesis [Yoo et al., 2011 [a/vivo mouse].<br><br>[Tannic acid] prion protein inhibition [Kocisko et al., 2003] [a/cell line mouse neuron]; increased SMN gene + protein expression [Sakla and Lorson, 2008] [h/cell line SMA patient fibroblasts]; anti-aging [Li J et al., 2017] [a/vivo rat, h/cell line]; enhanced proteasome activity [Corpas et al., 2019] [a/vivo mouse].<br>[Quercetin]: anti-tauopathic; inhibits ER stress [Chen J et al., 2016] [h/cell line neuron]
Voacanga africana Stapf ex Scott Elliot	Anti-bacterial, anti-fungal [Duru and Onyedineke, 2010]; neuroprotective [Currais et al., 2014] [vit]
Vochysia rufa Mart.	No records found<br>*Vochysia haenkeana* anti-venom [Harder et al., 2017] [a/ isolated phrenic nerve-diaphragm mouse]
Waltheria indica L. Syn: *Waltheria americana* L.	Anti-venom [Molander et al., 2014] [vit]; anti-inflammatory, anti-oxidant [Yougbare-Ziebrou et al., 2016] [a/vivo mouse]; anxioytic [GABA enhancement] [Mundo et al., 2015] [a/cell mouse neuron]
Waltheria ovata Cav.	Analgesic, anti-oxidant [Herrera-Calderon et al., 2016] [a/vivo mouse]
Warburgia ugandensis Sprague	Anti-viral [HIV] [Rukunga et al., 2002] [vit]; anti-bacterial [Wube et al., 2005] [vit]; immunostimulatory [Ngure et al., 2014] [a/vivo mouse]
Whitfieldia elongata (P.Beauv.) De Wild. & T.Durand	No records found
Withania somnifera (L.) Dunal	Anti-bacterial, anti-fungal [Mwitari et al., 2013] [vit]; anti-viral [HIV] [Rege AA et al., 2010] [vit]; improved memory and cognitive ability [Choudhary D et al., 2017] [h/c]; improved neuromuscular junction innervation + motor performance; reduced neuroinflammation, reversed TDP-43 pathology [Dutta et al., 2017] [a/vivo ALS mouse]; rescued motorneuron mitochondria and locomotory impairment, increased lifespan [De Rose at al., 2017] [a/vivo ALS *Drosophila*]; enhanced neural outgrowth + memory [Tohda and Joyashiki, 2009] [a/vivo AD mouse]; inhibits NF-κB activation + cell death [Akizuki et al., 2013] [a/cell mouse neuron]; reduced misfolded SOD, Hsp 25 induction [Patel P et al., 2015] [a/vivo ALS mouse]; anxiolytic [Lopresti et al., 2019] [h/c]; immunoregulatory, anti-stress [Khan B et al., 2006] [a/vivo mouse]; anti-venom neurotoxin [Machiah and Gowda, 2006] [a/vivo mouse]; wound healing [Sheoran et al., 2020] [h/c]
Wollastonia biflora (L.) DC. Syn: *Wedelia biflora* (L.) DC.	No records found<br>*W. chinensis* anti-inflammatory [Lin WC et al., 2015] [a/vivo mouse]
Woodfordia fruticosa (L.) Kurtz.	Anti-bacterial [Tambekar DH, Khante, 2011] [vit]; anti-inflammatory [Raj et al., 2019] [a/vivo rat]

Wrightia laevis Hook.f.	No records found<br>*Wrightia tinctoria* anti-viral [Hep C] [Sathyanarayanan et al., 2009] [h/cell line]
Xanthium spinosum L. Syn: *Acanthoxanthium spinosum* (L.) Furreau	Anti-inflammatory [Bader et al., 2013] [h/cell line]
Xanthosoma sagittifolium (L.) Schott	Moderately anti-bacterial [Obiukwu and Nwanekwu, 2010] [vit]; anti-inflammatory [Hossain MS et al., 2017] [a/vivo rat]
Ximenia caffra Sonder	Anti-bacterial [Munodawafa et al., 2013] [vit]; Anti-inflammatory [Zhen J et al., 2015] [cell line]
*Xylia xylocarpa (*Roxb.) W.Theob.	Cognitive improvement, AChE inhibition [Lam et al., 2016] [a/vivo mouse]
Xylopia aethiopica (Dunal) A.Rich	Anti-hypertensive [Somova et al., 2001] [vit]; anti-microbial [Tatsadjieu et al., 2003] [vit]
Xylopia hypolampra Mildbr.	No records found
Zanthoxylum armatum DC. Syn. *Zanthoxylum alatum* Roxb.	Anti-inflammatory [Guo T et al., 2011] [a/vivo mouse]; free-radical scavenging [Prakash B et al., 2012] [vit]]
Zanthoxylum capense (Thumb.) Harv.	Neuroprotective, anti-apoptotic [Seoposengwe et al., 2013] [h/ cell line neuron]; anti-inflammatory [Adebayo et al., 2015] [vit]; anti-venom [Molander et al., 2014] [vit]; anti-convulsant [Amabeoku and Kinyua, 2010] [a/vivo mouse]
Zanthoxylum deremense (Engl.) Kokwaro	No records found
Zanthoxylum ekmanii (Urb.) Alain.	No records found<br>*Zanthoxylum chalybeum* anti-viral [measles] [Olila et al., 2002] [vit]
Zanthoxylum gilletii (De Wild.) P.G.Waterman Syn: *Fagara macrophylla*	Anti-bacterial [Agyare et al., 2006] [vit]
Zanthoxylum rhoifolium Lam.	Anti-hypertensive, vasorelaxant [Ferreira-Filho et al., 2013] [a/vivo rat, a/isolated artery rat]; *Zanthoxylum riedelianum* anti-inflammatory [Lima LM et al., 2007] [a/vivo rat]
Zanthoxylum zanthoxyloides (Lam.) Zepernick & Timler Syn: *Fagara zanthoxyloides* Lam.	Anti-inflammatory [Oriowo, 1982] [a/vivo]
Zataria multiflora Boiss.	Anti-viral [HSV] [Arabzadeh et al., 2013] [a/monkey cell line]; improved learning + memory, reduced tau, reduced neuroinflammation [Ahmadi et al., 2019] [a/vivo rat]; anti-inflammatory [Ariaee et al., 2018] [h/c]; anti-aging [Sarikhani et al., 2021] [a/cell line rat]
Zea mays L.	Anti-bacterial [Nessa et al., 2012] [vit]; memory enhancement [in sp. comb. [Kirisattayakul et al., 2017] [a/vivo rat]; anti-inflammatory [Kim JY et al., 2018] [mouse cell line]; anti-hypertensive [Li CC et al., 2019] [a/vivo rat]
Zehneria scabra Sond.	No reports found
Zilla spinosa (L.) Prantl	Anti-inflammatory [Ullah et al., 2020] [a/vivo rat, mouse]
Zingiber montanum (J.Koenig) Link ex A.Dietr. Syns: *Zingiber cassumunar* Roxb; *Zingiber purpureum* Roscoe	Anti-inflammatory [Ozaki et al., 1991] [a/vivo rat]
Zingiber officinale Roscoe	Anti-viral [RSV] [San Chang et al., 2013] [h/c]; anti-bacterial [Karuppiah and Rajaram, 2012] [vit]; anti-inflammatory [Kulkarni RA and Deshpande, 2016] [h/c]; reduces apoptosis, increases BDNF + NGF expression, attenuated mitochondrial impairment [Kim S and Kwon, 2013] [a/cell line rat astrocyte]; anti-neuroinflammatory improves cognitive function [Moon et al., 2014] [a/vivo mouse]; anti-inflammatory, reduced MS-like symptoms [Jafarzadeh et al., 2014] [a/vivo MS mouse]; cognitive improvement [Saenghong et al., 2012] [h/c]; wound healing [Mohamed AH and Osman, 2017] [a/vivo rat]; anti-platelet aggregation [Young et al., 2006] [h/c]; anti-oxidant [Stoilova et al., 2007][vit]

Zingiber ottensii Valeton	Anti-bacterial [Habsah et al., 2000] [vit]
Zingiber zerumbet (L.) *Roscoe ex Sm.*	Nrf2 activation [Shin et al., 2011] [a/cell line mouse]; moderately anti-bacterial, anti-fungal [Kader et al., 2011] [vit]; anti-inflammatory [Sulaiman et al., 2010] [a/vivo mouse]
Ziziphora clinopodioides Lam.	Anti-bacterial [Shahla, 2012] [vit]
Ziziphora pamiroalaica Juz.	No records fund
Ziziphora taurica M.Bieb.	Anti-bacterial [Elgin et al., 2006] [vit]
Ziziphus jujuba Mill.	Anti-viral [influenza] [Hong EH et al., 2015] [h/cell line]; anti-inflammatory [Goyal R et al., 2011] [a/vivo rat]
Ziziphus lotus (L.) Lam	Anti-inflammatory [Hachimi, 2017] [a/vivo rat]; wound healing [Rais et al., 2020] [a/vivo mouse]
Ziziphus mucronata Willd.	Anti-venom [Molander et al., 2014] [vit]
Ziziphus nummularia (Burm.f.) Wight & Arn.	Anti-bacterial [Shahat et al., 2017] [vit]; anti-inflammatory, anti-atherosclerotic [Fardoun et al., 2017] [h/cells aorta]
Ziziphus oenoplia (L.) Mill.	Anti-ulcerogenic [Jadhav et al., 2011] [a/vivo rat]; anti-hyperlipidemic [Eswari et al., 2013] [a/vivo rat]
Ziziphus spina-christi (L.) Desf.	Anti-bacterial [Nazif, 2002] [vit]; anti-inflammatory [Adzu and Haruna, 2007] [a/vivo rat]; anti-venom [Molander et al., 2014] [vit]
Ziziphus mauritiana Lam.	Anti-bacterial [Abalaka et al., 2010] [vit]
Zygophyllum album L.f.	Anti-inflammatory, anti-oxidant [Ksouri et al., 2013] [a/cell line mouse]; AChE inhibition [Kchaou et al., 2016] [vit]

Abbreviations

Molecular target: AChE, acetylcholinesterase.

Tissue: rpe, retinal pigment epithelium.

Type of study: h/c, human clinical/epidemiological study; h/cell line, human cell line; a/vivo, animal *in vivo*; a/cell line, animal cell line; vit, *in vitro*; in sp. comb/spp. comb, in species combination.

† Treated as a separate species by Wu et al., 2018.

For references: see S8 Table References File.
