# Supplementary material for: Therapeutic roles of plants for 15 hypothesised causal bases of Alzheimer’s disease
Source: Nat Prod Bioprospect. 2022 Aug 23;12(1):34. doi: 10.1007/s13659-022-00354-z (PMC9395556; doi:10.1007/s13659-022-00354-z)
Supplement: Supplementary file 4 — Additional file 4. Table S4. Bioactivities listed by category. [file 13659_2022_354_MOESM4_ESM.pdf]

Additional Table S4. Bioactivities listed by category. List of species with confirmed bioactivities of AD relevance listed by therapeutic category

**Bioactivity category**	**Species**
Anti-amyloidogenic/protection vs amyloid-induced toxicity	*Allium roseum, Allium sativum, Apium graveolens, Asparagus racemosus, Bacopa monnieri, Cajanus cajan, Caesalpinia crista, Camellia sinensis, Capsicum annuum, Centella asiatica, Chromolaena odorata, Cocos nucifera*, *Convolvulus prostratus, Cornus officinalis, Cuminum cyminum, Curcuma longa, Cyperus rotundus, Elaeis guineensis, Fibraurea recisa, Garcinia mangostana, Mentha spicata, Moringa oleifera, Morus alba, Myristica fragrans, Olea europaea, Paeonia lactiflora, Panax quinquefolius, Phyllanthus emblica, Prunella vulgaris, Rosmarinus officinalis, Scoparia dulcis, Silybum marianum*, *Thymus vulgaris, Uncaria tomentosa, Vaccinium myrtillus, Vitis vinifera, Withania somnifera*<br><br>[sister spp*.]: *Caesalpinia sappan, Coptis chinensis, Elsholtzia rugulosa, Fragaria x ananassa, Panax ginseng, Pistacia lentiscus, Salvia miltiorrhiza, Satureja hortensis, Uncaria rhynchophylla*
Anti-tauopathic	*Acridocarpus orientalis, Allium cepa, Apium graveolens, Camellia sinensis, Cinnamomum cassia, Cinnamomum verum*, *Cocos nucifera, Convolvulus prostratus, Crataegus spp., Curcuma longa, Fibraurea recisa, Fragaria x ananassa, Malus pumila, Moringa oleifera, Morus alba, Myrica cerifera, Olea europaea, Passiflora edulis, Psidium guajava, Uncaria tomentosa, Zataria multiflora*
Enhanced proteasome activity	*Brassica oleracea, Olea europaea, Vitis vinifera*
Autophagy regulation, reduced autophagic stress	*Carissa carandas*, *Coptis spp., Eucommia ulmoides, Malus pumila, Moringa oleifera, Polygala tenuifolia, Punica granatum, Zingiber*<br><br>[sister spp.]: *Panax ginseng, Uncaria rhynchophylla*
Memory/cognitive improvement/reduced memory impairment/improved learning	*See* Table S7
Suppressed microglial activation/ reduced astrocyte reaction	*Apium graveolens, Cajanus cajan, Camellia sinensis, Carissa carandas, Myristica fragrans, Olea europaea, Pueraria montana var. lobata, Sambucus nigra, Tussilago farfara,*<br><br>[sister spp.]: *Acacia hydaspica, Acacia salicina, Bupleurum falcatum, Cinnamomum cassia, Rabdosa rubescens, Magnolia obovata, Stephania tetrandra, Uncaria rhynchophylla*
Neurogenic/neurotrophic/increases BDNF or NGF expression/ neuronal growth stimulus/ induces neurite outgrowth/ stimulates neural regeneration / reversed neurodegeneration	*Acorus gramineus, Acridocarpus orientalis, Adansonia digitata, Aloe vera, Anacardium occidentale, Artemisia annua, Asparagus racemosus, Bacopa monnieri, Boswellia sacra, Camellia sinensis, Calotropis procera, Carissa carandas*, *Centella asiatica, Citrus x aurantiurn, Croton tiglium, Curcuma longa, Elaeis guineensis, Embelia ribes, Garcinia xanthochymus, Leonurus cardiaca, Matricaria chamomilla, Moringa oleifera, Opuntia ficus-indica*, *Panax japonicus, Panax quinquefolius, Passiflora edulis, Prunella vulgaris, Pueraria montana, Rosmarinus officinalis*, *Solanum sisymbriifolium, Tinospora cordifolia, Vitis vinifera, Withania somnifera, Zingiber officinale*<br><br>[sister spp.]: *Alpinia katsumadai, Dioscorea japonica, Momordica cochinchinensis, Ribes fasciculatum, Stephania tetrandra, Uncaria rhynchophylla, Vaccinium spp.*
Anticholinergic/ AChE inhibition, POP inhibition	*Acanthospermum hispidum*, *Albizia lebbeck, Annona coriacea*, *Asparagus racemosus, Astronium urundeuva, Berberis darwinii*, *Carthamus tinctorius, Cissampelos pareira, Cochlospermum regium, Cola acuminata, Elettaria cardamomum, Emilia abyssinica, Evolvulus alsinoides, Ficus carica, Fumaria officinalis, Hancornia speciosa, Heinsia crinita, Leea indica, Mimosa pudica*, *Mondia whitei*, *Ocimum americanum, Origanum vulgare*, *Peristrophe bicalyculata, Pistacia atlantica, Quassia undulata, Rosmarinus officinalis*, *Salvia officinalis,*

	Sisyrinchium tinctorium, Tephrosia purpurea, Thymbra capitata, *Uraria picta, Vitis vinifera, Xylia xylocarpa, Zygophyllum album*<br>[sister spp.]: *Satureja montana, Teucrium* spp.
Anti-oxidant/ reduced oxidative stress/ free radical scavenging/ *reduced lipid peroxidation[reduced ROS/NO/* Fe2+ chelating	*Acer cinerascens, Achyranthes aspera, Adansonia digitata, Aframomum melegueta, Afrostyrax lepidophyllum, Allium cepa, Allium fistulosum, Allium rubellum, Allium schoenoprosum, Aloe harlana, Alpinia galanga, Anethum graveolens, Annona muricata, Anoda cristata, Antherotoma senegambiensis, Aquilaria malaccensis, Aristotelia chilensis, Artemisia absinthium, Artemisia annua, Artemisia indica, Artemisia judaica, Artemisia vulgaris, Asphodelus aestivus, Asphodelus tenuifolius, Astronium urundeuva, Asystasia nemorum*, *Bambusa vulgaris, Bergenia ciliata, Bertholletia excelsa, Beta vulgaris, Bixa orellana*, *Blumea balsamifera, Boerhavia erecta, Borago officinalis, Brocchia cinerea,Buddleja mendozensis, Butea monosperma, Camellia sinensis, Capparis cartilaginea, Capsicum annuum*, *Caralluma tuberculata, Carissa carandas*, *Carissa edulis, Carthamus tinctorius, Caryocar brasiliense, Cassia fistula, Castanea sativa, Ceiba pentandra, Celtis toka, Chuquiraga spinosa, Cissus adnata, Citrus sinensis, Citrus x aurantiurn, Clerodendrum cyrtophyllum, Cochlospermum regium, Cocos nucifera*, *Coffea arabica, Cola acuminata, Colubrina asiatica, Corchorus olitorus, Cornus mas, Cornus officinalis, Corylus avellana, Costus afer, Crataegus spp., Crotalaria pallida, Cuminum cyminum, Curatella americana*, *Curculigo pilosa, Cymbopogon citratus, Cynara scolymus, Daphniphyllum himalense, Desmodium heterocarpon, Dipteryx alata*, *Dirca palustris, Duguetia furfuracea, Ehretia cymosa, Elettaria cardamomum, Eleusine indica, Emilia abyssinica, Ephedra gerardiana, Erechtites hieracifolia, Erythrina abyssinica Eugenia dysenterica, Eugenia uniflora, Euphorbia tirucalli, Ficus carica, Ficus exasperata, Ficus microcarpa, Ficus natalensis, Foeniculum vulgare, Forsythia suspensa, Fraxinus chinensis, Galium verum, Gardenia jasminoides, Genipa americana, Glyphaea brevis, Globularia alypum, Gomphrena globosa, Gunnera tinctoria, Gymnosporia senegalensis, Hancornia speciosa, Haplophyllum tuberculatum, Helianthus annuus, Helicteres isora*, *Heliotropium bacciferum, Hibiscus tiliaceus, Hippophae rhamnoides, Hymenocardia acida*, *Jasminum nervosum, Kyllinga brevifolia, Kyllinga nemoralis*, *Lablab purpureus, Lagerstroemia speciosa, Laurus nobilis, Lepidium sativum, Leucas aspera, Leucas cephalotes, Lippia integrifolia, Lophira lanceolata, Ludwigia peruviana, Luma chequen, Lycium intricatum, Maclura tinctoria, Mauritia flexuosa, Mentha arvensis, Mentha* x *piperita, Mimosa pigra, Mollugo nudicaulis, Monstera deliciosa, Morinda citrifolia, Morinda umbellata, Moringa oleifera*, *Myristica fragrans, Myracrodruon urundeuva, Myroxylon peruiferum, Nymphaea nouchali, Ocimum americanum, Ocimum basilicum, Ocimum tenuiflorum*, *Olea europaea, Opuntia humifusa, Origanum vulgare, Oxalis corniculata, Paliurus spina-christi, Panax quinquefolius, Passiflora edulis, Passiflora ligularis, Phlogacanthus thyrsiformis, Phthirusa pyrifolia, Phyllanthus emblica, Pilea microphylla, Pimpinella anisum, Piper capense*, *Piper nigrum, Pisum sativum, Pistacia atlantica, Plectranthus barbatus, Plumbago indica, Poincianella pluviosa, Polygonatum sibiricum, Polyscias guilfoylei, Pongamia pinnata, Potentilla freyniana, Protium heptaphyllum, Prunus cerasoides, Prunus spinosa, Psidium guajava, Pulicaria undulata, Punica granatum, Quassia undulata, Rauvolfia vomitoria, Rhododendron arboreum*, *Rosa canina, Rosmarinus officinalis, Rubus buergeri, Rubus glaucus, Salvia aegyptiaca, Saccharum officinarum*, *Salvia tomentosa, Sambucus australis, Senna tora*, *Sansevieria trifasciata, Silybum marianum, Solanum lycocarpum, Solanum lycopersicum, Solanum melongena, Solanum sisymbriifolium, Solanum torvum, Solanum tuberosum, Sonchus oleraceus, Spathodea campanulata, Spondias dulcis, Stryphnodendron obovatum, Swertia ciliata, Swertia racemosa, Syzygium aromaticum, Tamarix aphylla*, *Thalictrum foliolosum, Thonningia sanguinea, Thymbra capitata, Thymus linearis, Thymus satureioides, Thymus serpyllumm, Thymus vulgaris, Tilia tomentosa, Tinospora cordifolia, Trema orientalis, Trianthema portulacastrum, Tridax procumbens, Trigonella foenum-graecum, Tylophora indica, Uraria picta, Urtica dioica, Vitis vinifera, Waltheria indica, Waltheria ovata*, *Zanthoxylum armatum, Zingiber officinale*

	[sister spp.]: *Acacia hydaspica, Acacia salicina, Byttneria pilosa, Campomanesia xanthocarpa, Cinnamomum cassia*, *Daucus carrota*, *Eurya japonica, Lonicera cerulea, Musa acuminata, Piper nigrum, Rubus jamaicensis*, *Saussurea pulvinata, Selaginella tamariscina*, *Taraxacum coreanum*, *Vaccinium angustifolium, Vaccinium uliginosum*
Immunomodulation/ immunostimulatory	*Acacia nilotica, Acmella oleracea, Allium sativum, Alpinia galanga, Alstonia scholaris, Anacyclus pyrethrum, Anastatica hierochuntica, Apium graveolens, Artemisia annua, Asparagus racemosus*, *Astragalus complanatus*, *Berberis integerrima, Bidens pilosa, Brassica rapa, Carica papaya, Codonopsis pilosula, Commiphora myrrha, Cornus mas, Cornus officinalis, Equisetum giganteum, Mangifera indica, Maranta arundinacea, Moringa oleifera, Origanum vulgare, Panax japonicus, Panax quinquefolius, Pennisetum glaucum, Petroselinum crispum, Piper betle, Pisum sativum, Platycodon grandiflorum, Polygonatum sibiricum, Prunella vulgaris, Sambucus javanica, Solanum muricatum, Solanum nigrum*, *Sorghum bicolor*, *Thymus serpyllum, Thymus vulgaris, Tinospora cordifolia, Trichopus zeylanicus, Uncaria tomentosa, Vigna unguiculata, Vitis vinifera, Warbugia ugandensis, Withania somnifera*<br><br>[sister spp.]: *Boswellia* spp., *Lemna minor Salvia miltiorrhiza, Trichospermum galeottii*
Reduced neural cell death/apoptosis/reduced neuronal loss	*Andrographis paniculata, Artemisia judaica, Averrhoa carambola, Citrus limon, Citrus x aurantiurn, Eriobotrya japonica, Fibraurea recisa, Ipomoea batatas, Mangifera indica, Matricaria chamomilla, Melissa officinalis, Morinda citrifolia, Moringa oleifera, Mucuna pruriens, Nymphaea nouchali, Olea europaea, Origanum vulgare, Panax quinquefolius, Perilla frutescens, Platycodon grandiflorum, Senna tora, Solanum tuberosum, Withania somnifera, Zanthoxylum capense*, *Zingiber officinale*<br><br>[sister spp.]: *Astragalus membranaceus*, *Citrus* spp., *Coptis* spp., *Salvia miltiorrhiza, Saussurea pulvinata, Selaginella tamariscina, Uncaria rhynchophylla, Vaccinium* spp.
Increased Nrf2 expression	*Bertholletia excelsa, Camellia sinensis, Carica papaya, Cinnamomum verum*, *Coffea arabica, Crataegus spp., Nasturtium officinale, Piper nigrum, Plumbago zeylanica, Solanum lycopersicum, Vitis vinifera, Zingiber zerumbet*
Supports or protects mitochondria/ maintain mitochondrial /function/enhanced mitochondrial biogenesis/ reversal of lost mitochondrial function.	*Apium graveolens, Boerhavia diffusa, Brassica oleracea, Carissa carandas, Carthamus tinctorius, Centella asiatica*, *Cinnamomum* spp., *Citrus x aurantium, Citrus maxima, Citrus paradisi, Cocos nucifera, Eurya japonica, Glycyrrhiza uralensis, Hippophae rhamnoides, Juglans regia, Mangifera indica, Matricaria chamomilla, Paullinia cupana, Platycodon grandiflorum, Rosmarinus officinalis, Rubus sp., Solanum dasyphyllum, Solanum* indicum, *Theobroma cacao, Viscum coloratum, Vitis vinifera, Withania somnifera*<br><br>[sister spp.]: *Rubus nigrum*
Anti-fatigue/energy-boosting	*Adansonia digitata, Aloe vera, Arctium lappa, Bambusa tuldoides, Carthamus tinctorius, Ipomoea batatas. Juglans regia, Mentha arvensis, Mentha* x *piperita, Moringa oleifera, Morus alba, Panax quinquefolius, Passiflora edulis, Pholidota chinensis, Phyllanthus emblica, Rosa canina, Viscum coloratum*<br><br>[sister spp.]: *Cinnamomum cassia, Polygonatum spp. Ribes nigrum*
DNA damage protection, reduced DNA damage	*Fagopyrum esculentum, Hibiscus acetosella*, *Mentha arvensis, Ocimum tenuiflorum, Olea europaea, Pilea microphylla, Saccharum officinarum, Uncaria tomentosa*
Anti-hypertensive/ vasodilatory/ vasorelaxant	*Acalypha wilkesiana, Achillea santolinoides, Allium cepa, Allium sativum, Alpinia zerumbet, Alstonia scholaris, Andrographis paniculata, Aniba canellila, Annona muricata, Apium graveolens, Arctium lappa, Ardisia crenata, Asteriscus graveolens, Artemisia scoparia*, *Artocarpus altilis, Aspidosperma subincanum, Averrhoa bilimbi, Azadirachta indica, Bambusa tuldoides, Beta vulgaris, Bidens pilosa,Camellia sinensis, Cajanus cajan, Camellia sinensis, Capraria biflora, Carica papaya, Centella asiatica, Citrus limetta, Citrus limon, Clausena anisata, Cornus mas, Crataegus spp., Crataeva adansonii, Croton schiedeanus, Cucurbita pepo,*

	Cymbopogon citratus, Dioscorea alata, Dysphania ambrosioides, Elaeis guineensis, Fagopyrum esculentum, Ferula communis, Ficus carica, Fuchsia magellanica, Hancornia speciosa, Harungana madagascariensis, Helianthus annuus, Helichrysum mechowianum, Heracleum sphondylium, Hibiscus sabdariffa, Hymenocardia acida, Imperata cylindrica, Ipomoea batatas, Juglans regia, Kalanchoe pinnata, Kigelia africana, Justicia secunda, Lagenaria siceraria, *Laurelia sempervirens, Leonurus cardiaca, Lepidium sativum*, *Lippia alba, Lippia multiflora, Lophira lanceolata, Lycopus lucidus, Mangifera indica, Medicago sativa, Melissa officinalis, Mentha arvensis, Mentha pulegium, Mentha spicata, Mimosa pigra, Morinda citrifolia, Morus alba, Musa x paradisiaca*, *Myrtus communis, Ocimum basilicum, Olea europaea, Oxalis corniculata, Panax japonicus, Panax quinquefolius, Peganum harmala, Peperomia pellucida, Persea americana, Petroselinum crispum, Phyllanthus acidus, Phyllanthus amarus, Phyllanthus emblica, Phyllanthus urinaria, Pisum sativum, Plantago asiatica, Platycodon grandiflorum, Prunella vulgaris, Psittacanthus calyculatus*, *Punica granatum, Rauvolfia verticillata, Salvia officinalis, Salvia scutellarioides, Scrophularia ningpoensis, Sechium edule, Smilax aristolochiifolia, Solanum aethiopicum, Solanum lycopersicum, Solanum marginatum, Solanum melongena, Solanum muricatum, Solanum tuberosum, Solanum torvum, Stachytarpheta jamaicensis, Stevia rebaudiana, Terminalia arjuna, Terminalia catappa, Theobroma cacao*, *Tridax procumbens, Uncaria sessilifructus, Uncaria sinensis, Urtica dioica, Vaccinium arctostaphylos, Vaccinium myrtillus, Vernonia amygdalina, Viola odorata*, *Vitis vinifera, Zanthoxylum rhoifolium, Zea mays, Zingiber officinale*<br><br>[sister spp.]: *Cinnamomum cassia, Canarium schweinfurthii, Crataegus curvisepala*, *Maxillaria densa, Mentha x villosa, Piper truncatum*
Anti-atherosclerotic/ reduced arterial stiffness/anti-atherogenic/cardioprotective	*Aloe vera, Carthamus tinctorius, Caulophyllum robustum, Citrus sinensis, Cynara scolymus, Leonurus sibiricu*s, *Morinda citrifolia, Moringa oleifera, Platycodon grandiflorum, Polygonatum sibiricum, Ziziphus nummularia*<br><br>[sister spp.]: *Virola oleifera*
Decreased total cholesterol/ anti-dyslipidemic/anti-hyperlipidemia/ improved lipid profile/ increased lipolysis	*Achillea santolinoides, Aerva lanata, Aloe vera, Alpinia zerumbet, Ananas comosus, Anethum graveolens, Arctium lappa, Artemisia vulgaris, Artocarpus altilis, Asparagus filicinus, Bambusa tuldoides, Berberis integerrima, Bixa orellana, Borago officinalis, Breynia vitis-idaea, Camellia sinensis, Carthamus tinctorius, Casearia esculenta, Chenopodium quinoa, Citrullus colocynthis, Cornus mas, Cuphea carthagenensis, Curatella americana*, *Cyphomandra betacea, Dipteryx alata, Fagopyrum esculentum, Ficus carica, Ficus microcarpa, Gardenia jasminoides, Gymnema sylvestre, Holoptelea integrifolia, Leonurus sibiricu*s, *Morinda citrifolia, Morus alba, Morus nigra, Olea europaea, Paederia foetida, Paliurus spina-christi, Paullinia cupana, Phyllanthus niruri*, *Polygonatum sibiricum, Punica granatum, Rosmarinus officinalis, Salvia officinalis, Tamarindus indica, Teucrium polium, Trianthema portulacastrum, Trigonella foenum-graecum, Viola odorata, Ziziphus oenoplia*<br><br>[sister spp.]: *Campomanesia xanthocarpa, Lonicera cerulea Senecio serratuloides*
Anti-platelet agglutination/aggregation/ Anti-PAF/ reduced increased vascular permeability/ Improved endothelial function/ thrombolytic/endothelial progenitor cell mobilization/ improved vascular function/cardioprotective	*Acacia modesta, Acacia nilotica, Allium jesdianum, Alocasia macrorrhizos, Althaea officinalis, Apium graveolens, Arctium lappa, Ardisia crenata, Asteromyrtus symphyocarp, Camellia sinensis, Cestrum parqui, Chiliadenus iphionoides, Chromolaena odorata, Crataegus spp., Ficus carica, Heliotropium indicum, Hordeum vulgare, Leonurus cardiaca, Leonurus sibiricu*s, *Luma apiculata, Malus pumila*, *Mauritia flexuosa, Oenanthe javanica, Olea europaea, Opuntia ficus-indica*, *Perilla frutescens, Petroselinum crispum, Phyllanthus amarus, Pisum sativum, Portulaca oleracea, Prunella vulgaris, Rubia cordifolia, Sechium edule, Solanum torvum, Solanum tuberosum, Spondias dulcis, Terminalia arjuna, Thymus vulgaris, Tinospora cordifolia, Toddalia asiatica, Trevesia palmata, Tussilago farfara, Typha angustifolia, Viscum coloratum, Zingiber officinale*<br><br>[sister spp.]: *Barleria lupulina, Campomanesia xanthocarpa, Ocotea duckei, Pogostemon cablin, Stephania tetrandra*

Anti-obesity/anti-adipogenic/ decreased weight	*Artemisia annua, Cyphomandra betacea, Cinnamomum* spp.
Anti-microbial	See Table S3 and S6
Gut microbiota modulation	*Morus alba, Punica granatum, Vaccinium myrtillus*
Anti-aging, increased longevity	*Allium sativum, Camellia sinensis, Forsythia suspense, Mentha piperata, Moringa oleifera, Ocimum tenuiflorum, Phyllanthus emblica, Polygonatum sibiricum Silybum marianum, Theobroma cacao, Vaccinium* spp.*, Vitis vinifera, Zataria multiflora*
Metal chelation	*Annona muricata, Annona* senegalensis, Cocos nucifera, Cola acuminata, *Combretum micranthum*, *Cymbopogon citratus, Dipteryx alata, Evolvulus alsinoides, Evolvulus alsinoides, Ipomoea batatas*, *Mentha arvensis, Moringa oleifera, Panax quinquefolius, Punica granatum*, *Quassia undulata, Rosmarinus officinalis, Saccharum officinarum, Triticum aestivum, Vitex negundo, Vitis vinifera, Zingiber officinale*
Reduced metal toxicity	*Allium sativum, Ananas comosus, Camellia sinensis, Curcuma longa, Cynara scolymus, Nigella sativa, Solanum lycopersicum, Silybum marianum, Zingiber officinale*
Oestrogenic activity/high phytoestrogen content	Oestrogenic<br>*Allium sativum, Erythrina spp., Euphorbia hirta, Ficus* sp., *Glycine max, Hippophae rhamnoides, Ipomoea batatas, Lannea acida, Medicago sativa, Momordica charantia, Piper guineense, Pueraria lobata, Punica granatum, Salvia officinalis, Scoparia dulcis, Tamarindus indica, Tephrosia purpurea.*<br>*Vitex negundo, Zingiber officinale*<br><br>Sister sp.: *Trifolium pratense*<br><br>Androgenic<br>*Pistacia atlantica, Punica granatum, Tamarindus indica, Zingiber officinale*

* sister species: species within the same genus. For further details see File S1.
