# Supplementary material for: Therapeutic roles of plants for 15 hypothesised causal bases of Alzheimer’s disease
Source: Nat Prod Bioprospect. 2022 Aug 23;12(1):34. doi: 10.1007/s13659-022-00354-z (PMC9395556; doi:10.1007/s13659-022-00354-z)
Supplement: Supplementary file 5 — Additional file 5. Table S5. Phytochemicals with anti-amyloid and tau activity. [file 13659_2022_354_MOESM5_ESM.pdf]

Additional Table S5. Phytochemicals with anti-amyloid and tau activity

Phytochemical	Plant sources	Model system	Activity	Reference
Apigenin	*Elsholtzia rugulosa*	*In vivo* AD mouse	Lowered insoluble Aβ1–40 and Aβ1–42 levels and amyloid plaque burden; improved learning and memory	Zhao et al., 2013
Bacoside-A	*Bacopa monnieri*	*In vitro*	Inhibited Aβ fibrillogenesis	Malishev et al., 2017
Baicalein	*Scutellaria baicalensis*	*In vitro*	Amylin amyloid inhibition	Velander et al., 2016
Berberine	*Coptis chinensis*	Mouse AD	Reduced A*β* accumulation and hyperphosphorylated tau, amelioration of learning deficits, reduced reactive astrocytes + microglia	Durairajan et al., 2012
Brazilin	*Caesalpinia sappan*	Human neuronal cell line; *in vitro*	Inhibition of Aβ42 fibril formation, remodelling of amyloid fibrils into less toxic species	Du et al., 2015
Cajaninstilbene acid	*Cajanus cajan*	*In vivo* AD mouse	Stimulated amyloid β clearance, attenuated memory impairment, reduced microglial and astrocyte reactivity	Wang LS et al., 2019
Catechin	*Acacia hydaspica, Acacia salicina, Camellia sinensis, Anethum graveolens, Myristica fragrans*	AD mice	Enhanced clearance of Aβ and inhibition of Aβ aggregation; suppressed microglial activation; enhanced cognitive performance	Zhu et al., 2008; Joseph et al., 1999
Cinnamaldehyde	*Cinnamomum zeylanicum*	*In vitro*	Inhibition of tau aggregation	George et al., 2013
Cryptotanshinone	*Salvia miltiorrhiza*	Human neural cell line	Inhibition of Aβ aggregation	Mei et al., 2012
Curcumin	*Curcuma longa*	*In vivo* AD mouse	Inhibition of Aβ aggregation, disaggregation of Aβ aggregates; reduced Aβ and phosphorylated tau protein	Shytle et al., 2012
EGCG	*Camellia sinensis*	*In vivo* AD mouse	Reduced Aβ deposition and tau phosphorylation, improved working memory	Rezai-Zadeh et al., 2005, 2008
EGCG	*Camellia sinensis*	*In vitro*	Remodelling of toxic amyloid aggregates into non-toxic off-pathway species	Bieschke et al., 2010
EGCG	*Camellia sinensis*	Mouse neurons*, in vitro*	Anti-amyloidogenic, reduced metal–Aβ toxicity	Hyung et al. 2013
Fibrauretine	*Fibraurea recisa*	*In vitro*	Reduced brain Aβ and tau levels; memory improvement, anti-neuroinflammatory	Xing Z et al., 2018
Fisetin	*Fragaria ananassa*	Mouse microglia, *in vitro*	Inhibition of Aβ aggregation, anti-Aβ fibrillation and tau phosphorylation	Ahmad et al., 2017
Ginsenosides	*Panax ginseng*	*In vivo* AD mouse	Decreased brain Aβ levels and plaque burden; improved cognitive function	Fan et al., 2017

	Panax quinquefolius	*In vivo* AD mouse; hamster cell line	Reduced brain $A\beta_{42}$ concentration	Chen F et al., 2006
Luteolin	*Perilla frutescens*	Mouse neurons and glia	Inhibition of Aβ-induced neuronal death	Choi et al., 2014
Mangostin	*Garcinia mangostana*	Rat neuron	Attenuated neurotoxicity induced by Aβ oligomers; inhibition of Aβ fibrillogenesis	Wang Y et al., 2012
Morin, morin hydrate	*Acridocarpus orientalis*	*In vitro*	Inhibition of Aβ aggregation and tau phosphorylation; Aβ fibril disaggregation	Noor et al., 2012;
Morin	*Morus alba*	*In vivo* AD mouse	Reduced Aβ production and Aβ plaque burden via upregulation of amyloid-degrading protease; up-regulation of synaptic proteins; decreased tau	Du et al., 2016
Myricanol	*Myrica cerifera*	Human IMR32 neural cell line	Enhanced tau clearance	Jones et al., 2011
Oleuropein aglycone	*Olea europaea*	AD *Caenorhabditis elegans*	Reduced Aβ plaque deposition, less abundant toxic Aβ oligomers	Diomede et al., 2013
Oleuropein	*Olea europaea*	*In vivo* AD mouse	Reduced Aβ levels and plaque deposits, reduced astrocyte reaction	Grossi et al., 2013
Oleocanthal	*Olea europaea*	*In vivo* AD mouse	Decreased hippocampal amyloid load, enhanced Aβ clearance	Qosa et al., 2015
Piceid [polydatin] [non-glycosylated derivative of resveratrol]	*Polygonum cuspidatum, Vitis vinifera*	*In vitro*	Inhibition of Aβ polymerization	Rivière et al., 2007; Romero-Pérez et al., 1999 [for source of piceid]
Quercetin	*Capparis spinosa, Mangifera indica, Crataegus spp., Brassica oleracea*	Human SH-SY5Y neural cell line	Attenuated tau hyperphosphorylation	Chen J et al., 2015
Resveratrol	*Vitis vinifera*	In vitro assay, Rat PC12 neural cell line	Remodelling of toxic amyloid aggregates into non-toxic off-pathway species	Ladiwala et al., 2010; Rivière et al., 2007
Rosmarinic acid	*Rosmarinus officinalis*	AD model transgenic mice (Tg2576).	Inhibition of Aβ aggregation, disaggregation of Aβ aggregates; reduced tau hyper-phosphorylation	Hamaguchi et al., 2009; Cornejo et al., 2017
Sarsasapogenin	*Asparagus racemosus*	*In vitro*	Inhibition of Aβ42 aggregation, BACE1 inhibition	Kashyap et al., 2020
Scyllo-cyclohexanehexol	*Cocos nucifera*	*In vivo* AD mouse	Blocked accumulation of Aβ oligomers, reduced brain Aβ levels and brain amyloid pathology; reduced synaptic loss and glial reactivity; improved cognitive performance	McLaurin et al., 2006
Silymarin	*Silybum marianum*	Human neural cell	Inhibition of Aβ aggregation	Yin et al., 2011; Murata et al., 2010

		line; *in vivo* AD mouse		
Tannic acid	*Rhus chinensis*	*In vitro*	Inhibition of Aβ fibrillation and Aβ peptide polymerization	Ono et al., 2004
Tannoid extract	*Phyllanthus emblica*	*In vivo* AD rat	Reduced expression of amyloid precursor protein and Aβ1-42; attenuated memory + learning impairments; AChE inhibition	Thenmozhi et al., 2016
Tanshinones	*Salvia miltiorrhiza*	*In vitro*	Retardation of Aβ aggregation, disassembly of fibrillar Aβ	Wang Q et al., 2013
Taxifolin	*Silybum marianum*	*In vitro*	Suppression of Aβ aggregation by targeting amyloid Lys-residues	Sato et al., 2013
Terpinolene and limonene	*Cuminum cyminum*	Rat neuron	Anti-amyloidogenic [interfering with amyloid fibrillation]	Morshedi et al., 2014
Tetramethoxyflav anone	*Chromolaena odorata*	*In vivo* AD mouse	Reduced Aβ production via reduced BACE1 expression	Pakdeepak et al., 2010
Verbascoside	*Olea europaea*	*In vitro*	Reduced aggregation and cytotoxicity of metal-free and/or metal-associated A*β*	Korshavn et al., 2015
Viniferin	*Vitis vinifera*	AD mouse neuron	Disaggregation of Aβ	Vion et al., 2018
Oil palm phenolics	*Elaeis guineensis*	*In vitro*	Inhibited aggregation of A*β* into oligomers, stacking of *β*-pleated sheets and fibrillary growth	Weinberg et al., 2018

For references: see Table References File
