# Supplementary material for: Therapeutic roles of plants for 15 hypothesised causal bases of Alzheimer’s disease
Source: Nat Prod Bioprospect. 2022 Aug 23;12(1):34. doi: 10.1007/s13659-022-00354-z (PMC9395556; doi:10.1007/s13659-022-00354-z)
Supplement: Supplementary file 6 — Additional file 6. Table S6. Examples of plants with activity against infectious agents implicated in Alzheimer’s disease. [file 13659_2022_354_MOESM6_ESM.pdf]

**Additional Table S6. Examples of plants with activity against infectious agents implicated in Alzheimer's disease**

| **1. Infectious agent implicated in AD** | | **2. Plant species with anti-microbial activity [against microbes in column 1.]** |
|---|---|---|
| Bacteria | *Borrelia spp.* | *Citrus paradisi* |
| | *Escherichia coli* | *Alstonia scholaris, Amaranthus viridis, Cissus rotundifolia, Heliotropium bacciferum, Thalictrum foliolosum, Treculia africana, Ziziphus mauritiana, Ziziphus spinachristi* |
| | *Porphyromonas gingivalis* | *Aloe vera, Musa paradisiaca, Pistacia lentiscus* |
| Fungi | *Alternaria* spp | *Thalictrum foliolosum* |
| | *Botrytis* | *Pentacalia corymbosa* |
| | *Cladosporium, Candida albicans* | *Luma chequen, Gentiana macrophylla* |
| Viruses | *Herpes simplex* virus (HSV) | *Allium cepa, Alstonia scholaris, Alternanthera brasiliana, Artemisia annua, Artemisia arborescens, Atalantia monophylla, Austroeupatorium inulaefolium, Balanites aegyptiaca, Barleria lupulina, Bergenia ciliata, Bidens pilosa, Capsicum annuum, Citrus sinensis, Cocos nucifera, Combretum micranthum, Cunila spicata, Drymis winteri, Dysosma versipellis, Ficus carica, Galinsoga parviflora, Lippia alba, Luma apiculata, Lysimachia arvensis, Mallotus philippensis, Mangifera indica, Markhamia lutea, Mentha pulegium, Mentha x piperita, Morus alba, Musa × paradisiaca, Ocimum basilicum, Ocimum campechianum, Persea americana, Pterocaulon alopecuroides, Portulaca oleracea, Prunella vulgaris, Punica granatum, Rhamnus alaternus, Rheum australe, Rhus vulgaris, Satureja thymbra, Scoparia dulcis, Senna sophera, Solanum sisymbriifolium, Solanum torvum, Sonchus oleraceus, Sorghum bicolor, Thymbra spicata, Thymus linearis, Thymus longicaulis, Thymus satureioides, Urera baccifera, Verbascum thapsus, Vitis vinifera, Zataria multiflora* |

**For references:** see Additional Table S3 and Additional Table References Files
