# Supplementary material for: Therapeutic roles of plants for 15 hypothesised causal bases of Alzheimer’s disease
Source: Nat Prod Bioprospect. 2022 Aug 23;12(1):34. doi: 10.1007/s13659-022-00354-z (PMC9395556; doi:10.1007/s13659-022-00354-z)
Supplement: Supplementary file 7 — Additional file 7. Table S7. Plants with ethnological reports of memory improvement demonstrating bioactivities of therapeutic relevance to 15 causal hypotheses for AD. [file 13659_2022_354_MOESM7_ESM.pdf]

Additional Table S7. Plants with ethnological reports of memory improvement demonstrating bioactivities of therapeutic relevance to 15 causal hypotheses for AD

Causal hypothesis	Plant species with therapeutic bioactivity
Amyloid hypothesis	*Allium sativum, Bacopa monnieri, Centella asiatica, Cocos nucifera*, *Convolvulus prostratus, Curcuma longa, Elaeis guineensis, Rosmarinus officinalis,*<br>Sister spp.* *Fragaria × ananassa, Pistacia lentiscus*
Tau hypothesis	*Cinnamomum verum*, *Cocos nucifera, Convolvulus prostratus, Crataegus spp., Curcuma longa, Moringa oleifera, Morus alba, Olea europaea, Passiflora edulis, Psidium guajava, Rosmarinus officinalis, Vitis vinifera*<br>Sister sp. *Fragaria × ananassa*
Ubiquitin–proteasome hypothesis	*Vitis vinifera*
Impaired autophagy hypothesis	*Moringa oleifera, Punica granatum, Zingiber officinale*
Inflammation hypothesis	*Adansonia digitata, Aframomum melegueta, Albizia zygia, Allium fistulosum, Allium sativum, Alstonia boonei*, *Annona muricata, Annona senegalensis, Asystasia gangetica, Bacopa monnieri, Bambusa vulgaris, Baphia nitida, Borago officinalis, Boswellia sacra, Bridelia ferruginea, Bunium persicum, Capparis erythrocarpus, Carica papaya, Carissa edulis, Centella asiatica, Cinnamomum verum*, *Citrus x aurantiurn, Cleome gynandra, Cocos nucifera, Combretum micranthum, Crossopteryx febrifuga, Curcuma longa, Cymbopogon citratus, Cymbopogon giganteus, Cynanchum viminale, Cynodon dactylon, Dysphania ambrosioides, Elaeis guineensis, Eleutherine bulbosa, Erythrina senegalensis, Evolvulus alsinoides, Ficus carica, Ficus exasperata, Ficus platyphylla, Flueggea virosa, Galinsoga parviflora, Harungana madagascariensis, Hymenocardia acida, Ipomoea batatas, Jatropha curcas, Lannea acida, Leonurus cardiaca, Litsea cubeba, Maesa lanceolata, Mangifera indica, Melissa officinalis, Mentha arvensis, Momordica balsamina, Moringa oleifera, Nauclea latifolia, Ocimum americanum, Phyllanthus amarus, Picralima nitida, Plumbago zeylanica, Prunus africana, Punica granatum, Rosmarinus officinalis, Schwenckia americana, Scolymus hispanicus, Scoparia dulcis, Securidaca longipedunculata, Solanum incanum, Tamarindus indica, Vitellaria paradoxa, Vitis vinifera, Zanthoxylum zanthoxyloides, Zea mays, Zingiber officinale, Ziziphus mucronata*<br>Sister sp.: *Erythrina mildbraedii*
Immune hyopothesis	*Allium sativum, Boswellia* spp., *Carica papaya, Mangifera indica, Moringa oleifera, Rosmarinus officinalis, Vitis vinifera*
Oxidative Stress Hypothesis	*Acer monspessulanum, Acokanthera schimperi, Aframomum melegueta, Albizia zygia, Allium fistulosum, Allium sativum, Annona muricata, Asystasia gangetica, Bambusa vulgaris, Borago officinalis, Carica papaya, Carissa edulis, Cinnamomum verum*, *Citrus x aurantiurn, Cocos nucifera, Cola acuminata, Curculigo pilosa, Cymbopogon citratus, Dipteryx alata, Ehretia cymosa, Emilia abyssinica, Entandrophragma utile, Erythrina abyssinica*, *Ficus carica, Ficus exasperata, Hippophae rhamnoides, Hymenocardia acida, Mentha arvensis, Momordica balsamina, Moringa oleifera, Ocimum americanum, Ocimum tenuiflorum, Pistacia atlantica, Punica granatum, Quassia undulata, Rosmarinus officinalis, Saccharum officinarum, Tephrosia purpurea, Uraria picta, Vitis vinifera, Zingiber officinale, Ziziphus mucronata*
Mitochondrial hypothesis	*Centella asiatica, Cocos nucifera, Heteropterys tomentosa, Hippophae rhamnoides, Mangifera indica, Moringa oleifera, Rosmarinus officinalis, Vitis vinifera, Zingiber officinale*
Neurogenesis hypothesis	*Adansonia digitata, Bacopa monnieri, Centella asiatica, Curcuma longa, Elaeis guineensis, Leonurus cardiaca, Mangifera indica, Moringa oleifera, Piper guineense, Rosmarinus officinalis, Tamarindus indica, Vitis vinifera, Zingiber officinale*
Cholinergic hypothesis	*Acanthospermum hispidum, Bambusa vulgaris, Cola acuminata, Emilia abyssinica, Evolvulus alsinoides, Ficus carica, Mondia whitei, Moringa oleifera, Ocimum americanum, Pistacia atlantica, Quassia undulata, Rosmarinus officinalis, Tephrosia purpurea, Uraria picta, Vitis vinifera, Ziziphus mucronata*
Vascular hypothesis	Anti-hypertensive: *Allium sativum, Annona muricata, Carica papaya, Centella asiatica, Cymbopogon citratus, Dysphania ambrosioides, Elaeis guineensis, Ficus carica, Harungana madagascariensis, Hymenocardia acida, Ipomoea batatas, Leonurus cardiaca, Mangifera indica, Melissa officinalis, Mitragyna inermis, Musa × paradisiaca, Phyllanthus amarus, Punica granatum, Vitis vinifera*<br>Anti-hyper/dyslipidemic: *Borago officinalis, Capparis erythrocarpus, Dipteryx alata, , Ficus carica, Punica granatum, Rosmarinus officinalis, Tamarindus indica*<br>Anti-atherosclerotic: *Moringa oleifera*<br>Anti-platelet aggregation: *Ficus carica, Leonurus cardiaca, Zingiber officinale*<br>Thrombolytic: *Heliotropium indicum*

	Improved endothelial function *Phyllanthus amarus*
Infection hypothesis	Anti-bacterial: *Acanthospermum hispidum, Acokanthera schimperi, Aframomum melegueta, Albizia zygia, Annona muricata, Baphia nitida, Bobgunnia madagascariensis, Borago officinalis, Boswellia sacra, Carica papaya, Carissa edulis, Centella asiatica, Cinnamomum verum*, *Citrus x aurantiurn, Cocos nucifera, Cola acuminata, Cymbopogon citratus, Cymbopogon densiflorus, Dysphania ambrosioides, Ehretia cymosa, Eleutherine bulbosa, Erythrina senegalensis, Euphorbia hirta, Ficus carica, Ficus exasperata, Harungana madagascariensis, Heliotropium indicum, Heteropterys tomentosa, Hymenocardia acida, Jatropha curcas, Lawsonia inermis Mangifera indica, Mentha arvensis, Mitragyna inermis, Mondia whitei, Morinda lucida, Moringa oleifera, Musa × paradisiaca, Nauclea latifolia, Ocimum americanum, Parinari curatellifolia, Picralima nitida, Plumbago zeylanica, Prunus africana, Rosmarinus officinalis, Tamarindus indica, Tetrapleura tetraptera, Uraria picta, Vitex negundo, Vitex simplicifolia, Vitis vinifera, Zea mays, Zingiber officinale, Ziziphus mucronata*<br><br>*Anti-viral: Acokanthera schimperi, Aframomum melegueta, Aframomum melegueta, Allium sativum, Bambusa vulgaris, Cocos nucifera, Combretum micranthum, Curcuma longa, Cymbopogon citratus, Detarium microcarpum, Dysphania ambrosioides, Eleutherine bulbosa, Erythrina abyssinica, Erythrina senegalensis, Euphorbia hirta, Ficus carica, Flueggea virosa, Galinsoga parviflora, Jatropha curcas, Maesa lanceolata, Mangifera indica, Melissa officinalis, Momordica balsamina, Momordica charantia, Musa × paradisiaca, Punica granatum, Rosmarinus officinalis, Scoparia dulcis, Solanum incanum, Vitis vinifera, Zingiber officinale*
Metal ion hypothesis	*Annona muricata, Annona senegalensis, Cocos nucifera, Cola acuminata, Combretum micranthum, Curcuma longa, Cymbopogon citratus, Evolvulus alsinoides, Hippophae rhamnoides, Ipomoea batatas, Mentha arvensis, Moringa oleifera, Punica granatum, Quassia undulata, Rosmarinus officinalis, Saccharum officinarum, Vitex negundo, Vitis vinifera, Zingiber officinale*
Oestrogen and androgen hypotheses	Oestrogenic: *Allium sativum, Erythrina abyssinica, Euphorbia hirta, Ficus* sp., *Hippophae rhamnoides, Ipomoea batatas, Lannea acida, Momordica charantia, Piper guineense, Punica granatum, Scoparia dulcis, Tamarindus indica, Vitex negundo, Zingiber officinale*<br>Sister spp.: *Erythrina excelsa*<br><br>Androgenic: *Tamarindus indica, Zingiber officinale*

* sister species: species within the same genus. For further details see File S1.
