# Supplementary material for: Therapeutic roles of plants for 15 hypothesised causal bases of Alzheimer’s disease
Source: Nat Prod Bioprospect. 2022 Aug 23;12(1):34. doi: 10.1007/s13659-022-00354-z (PMC9395556; doi:10.1007/s13659-022-00354-z)
Supplement: Supplementary file 8 — Additional file 8. Table S1–S7. References. [file 13659_2022_354_MOESM8_ESM.pdf]

## Additional Table references


Aati HY, El-Gamal AA, Kayser O, Ahmed AF. The phytochemical and biological investigation of *Jatropha pelargoniifolia* root native to the Kingdom of Saudi Arabia. Molecules. 2018 Aug;23(8):1892. https://doi.org/10.3390/molecules23081892

Abad MJ, Bermejo P, Gonzales E, Iglesias I, Irurzun A, Carrasco L. Antiviral activity of Bolivian plant extracts. General Pharmacology: The Vascular System. 1999 Apr 1;32(4):499-503. https://doi.org/10.1016/S0306-3623(98)00214-6

Abad MJ, Bessa AL, Ballarin B, Aragón O, Gonzales E, Bermejo P. Anti-inflammatory activity of four Bolivian *Baccharis* species (Compositae). Journal of ethnopharmacology. 2006 Feb 20;103(3):338-44. https://doi.org/10.1016/j.jep.2005.08.024

Abalaka ME, Daniyan SY, Mann A. Evaluation of the antimicrobial activities of two *Ziziphus* species (*Ziziphus mauritiana* L. and *Ziziphus spinachristi* L.) on some microbial pathogens. African Journal of Pharmacy and Pharmacology. 2010 Apr 1;4(4):135-9.

Abbah J, Amos S, Chindo B, Ngazal I, Vongtau HO, Adzu B, Farida T, Odutola AA, Wambebe C, Gamaniel KS. Pharmacological evidence favouring the use of *Nauclea latifolia* in malaria ethnopharmacy: effects against nociception, inflammation, and pyrexia in rats and mice. Journal of ethnopharmacology. 2010 Jan 8;127(1):85-90. https://doi.org/10.1016/j.jep.2009.09.045

Abbas SR, Sabir SM, Ahmad SD, Boligon AA, Athayde ML. Phenolic profile, antioxidant potential and DNA damage protecting activity of sugarcane (*Saccharum officinarum*). Food chemistry. 2014 Mar 15;147:10-6. https://doi.org/10.1016/j.foodchem.2013.09.113

Abdalla SL, Costa SS, Gioso MA, Casanova LM, Coutinho MA, Silva MF, Botelho MC, Dias RS. Efficacy of a *Kalanchoe gastonis-bonnieri* extract to control bacterial biofilms and dental calculus in dogs. Pesquisa Veterinária Brasileira. 2017 Aug;37(8):859-65. doi.org/10.1590/s0100-736x2017000800013

Abdallah EM, Gamal EG. Screening for antimicrobial activity of some plants from Saudi folk medicine. Global Journal of Research on Medicinal Plants & Indigenous Medicine. 2013 Apr 1;2(4):189.

Abdallah EM. Antimicrobial properties and phytochemical constituents of the methanol extracts of *Euphorbia retusa* Forssk. and *Euphorbia terracina* L. from Saudi Arabia. South Asian Journal of Experimental Biology. 2014 May 5;4(2):48-53.

Abdel Moneim A. The neuroprotective effects of purslane (*Portulaca oleracea*) on rotenone-induced biochemical changes and apoptosis in brain of rat. CNS & Neurological Disorders-Drug Targets (Formerly Current Drug Targets-CNS & Neurological Disorders). 2013 Sep 1;12(6):830-41.

Abdel-Malek S, Bastien JW, Mahler WF, Jia Q, Reinecke MG, Robinson Jr WE, Shu YH, Zalles-Asin J. Drug leads from the Kallawaya herbalists of Bolivia. 1. Background, rationale, protocol and anti-HIV activity. Journal of ethnopharmacology. 1996 Mar 1;50(3):157-66. https://doi.org/10.1016/0378-8741(96)01380-3

Abdennacer B, Karim M, Nesrine R, Mouna D, Mohamed B. Determination of phytochemicals and antioxidant activity of methanol extracts obtained from the fruit and leaves of Tunisian *Lycium intricatum* Boiss. Food chemistry. 2015 May 1;174:577-84. https://doi.org/10.1016/j.foodchem.2014.11.114

Abdou IA, Abou-Zeid AA, El-Sherbeeny MR, Abou-El-Gheat ZH. Antimicrobial activities of *Allium sativum, Allium cepa, Raphanus sativus, Capsicum frutescens, Eruca sativa, Allium kurrat* on bacteria. Qualitas Plantarum et Materiae Vegetabiles. 1972 Dec 1;22(1):29-35. doi.org/10.1007/BF01099735

Abdu KB, Khan ME, Rumah MM. Antimicrobial activity and phytochemical screening of extracts from the root bark of *Carissa edulis*, against human/animal pathogens. Continental Journal of Tropical Medicine. 2008 Jan 1;2:1.

Abdul G, Naqi H, Ishtiaq M. Ethnobotanical survey of medicinal plants of sargodha region and its vicinities. Global J Res Med Plants & Indig Med. 2012 June;1(6):242-246.

Abdul MM, Sarker AA, Saiful IM, Muniruddin A. Cytotoxic and antimicrobial activity of the crude extract of *Abutilon indicum*. International Journal of Pharmacognosy and Phytochemical Research. 2010;2(1):1-4.

Abdulah R, Milanda T, Sugijanto M, Barliana MI, Diantini A, Supratman U, Subarnas A. Antibacterial properties of selected plants consumed by primates against *Escherichia coli* and *Bacillus subtilis*. Southeast. Asian J. Trop. Med. Public Health. 2017 Jan 1;48:109-16.

Abdulfattah SY. Study of immunological effect of *Anastatica hierochuntica* (Kaff Maryam) plant methanolic extract on albino male mice. Jornal of Biotechnology Research Center. 2013;7(2):3-10.

Abd-Ulgadir KS. An in vitro antimicrobial potential of various extracts of *Commiphora myrrha*. Sudan Medical Laboratory Journal. 2017 Oct 1;5(2).

Abdullahi-Gero HS, Ahmed A, Zezi AU, Hussaini IM. Preliminary evaluation of ethanol leaf extract of *Borreria verticillata* Linn (Rubiaceae) for analgesic and anti-inflammatory effects. Journal of Medicinal Plants Research. 2014 May;8(20):736-47.

Abedini A, Colin M, Hubert J, Charpentier E, Angelis A, Bounasri H, Bertaux B, Kotland A, Reffuveille F, Nuzillard JM, Renault JH. Abundant Extractable Metabolites from Temperate Tree Barks: The Specific Antimicrobial Activity of *Prunus avium* Extracts. Antibiotics. 2020 Mar;9(3):111. https://doi.org/10.3390/antibiotics9030111

Abere TA, Ibanishuka P, Jesuorobo RI. Analgesic and toxicological evaluation of the stem bark of *Albizia zygia* Benth (Mimosoideae). IOSR Journal of Pharmacy and Biological Sciences. 2014;9(2):26-31.

Abeysinghe DC, Li X, Sun C, Zhang W, Zhou C, Chen K. Bioactive compounds and antioxidant capacities in different edible tissues of citrus fruit of four species. Food chemistry. 2007 Jan 1;104(4):1338-44.

Abib RT, Peres KC, Barbosa AM, Peres TV, Bernardes A, Zimmermann LM, Quincozes-Santos A, Fiedler HD, Leal RB, Farina M, Gottfried C. Epigallocatechin-3-gallate protects rat brain mitochondria against cadmium-induced damage. Food and chemical toxicology. 2011 Oct 1;49(10):2618-23. https://doi.org/10.1016/j.fct.2011.07.006

Abo KA, Ashidi JS. Antimicrobial screening of *Bridelia, micrantha, Alchormea cordifolia* and *Boerhavia diffusa*. African Journal of Medicine and Medical Sciences. 1999 Sep 1;28(3-4):167-9.

Abraham J, Thomas TD. Antibacterial activity of medicinal plant *Cyclea peltata* (Lam) Hooks & Thoms. Asian Pacific Journal of Tropical Disease. 2012 Jan 1;2:S280-4. https://doi.org/10.1016/S2222-1808(12)60166-2

Abreu CM, Price SL, Shirk EN, Cunha RD, Pianowski LF, Clements JE, Tanuri A, Gama L. Dual role of novel ingenol derivatives from *Euphorbia tirucalli* in HIV replication: inhibition of de novo infection and activation of viral LTR. PloS one. 2014 May 14;9(5):e97257. https://doi.org/10.1371/journal.pone.0097257

Abreu P, Matthew S, González T, Costa D, Segundo MA, Fernandes E. Anti-inflammatory and antioxidant activity of a medicinal tincture from *Pedilanthus tithymaloides*. Life sciences. 2006 Feb 28;78(14):1578-85. https://doi.org/10.1016/j.lfs.2005.07.037

Abubakar MN, Majinda RR. GC-MS analysis and preliminary antimicrobial activity of *Albizia adianthifolia* (Schumach) and *Pterocarpus angolensis* (DC). Medicines. 2016 Mar;3(1):3. https://doi.org/10.3390/medicines3010003

Abu-Rabia A. Ethno-botanic treatments for paralysis (falij) in the Middle East. Chinese Medicine. 2012 Dec 28;3(04):157. doi: 10.4236/cm.2012.34025

Acharya K, Giri S, Biswas G. Comparative study of antioxidant activity and nitric oxide synthase activation property of different extracts from *Rhododendron arboreum* flower. Int J Pharmtech Res. 2011;3(2):757-62.

Acosta SL, Muro LV, Sacerio AL, Monteagudo GL, Pena AR, Okwei SN. Anti-inflammatory effects of an aqueous extract of *Capraria biflora* L. Acta Farmacéutica Bonaerense. 2003;22(1):53-6.

Adaramoye OA, Akanni OO. Effects of methanol extract of breadfruit (*Artocarpus altilis*) on atherogenic indices and redox status of cellular system of hypercholesterolemic male rats. Advances in pharmacological sciences. 2014; Article ID 605425 https://doi.org/10.1155/2014/605425

Adebayo SA, Dzoyem JP, Shai LJ, Eloff JN. The anti-inflammatory and antioxidant activity of 25 plant species used traditionally to treat pain in southern African. BMC Complement Altern Med. 2015;15:159. Published 2015 May 27. doi:10.1186/s12906-015-0669-5

Adebiyi AO. Secondary Metabolites and Bioactivities of Two species of *Nephrolepis* in Ekiti State, Nigeria. International Journal of Life Sciences. 2019;8(3):95-9.

Adedapo AA, Sofidiya MO, Masika PJ, Afolayan AJ. Anti-inflammatory and analgesic activities of the aqueous extract of *Acacia karroo* stem bark in experimental animals. Basic & clinical pharmacology & toxicology. 2008 Nov;103(5):397-400. https://doi.org/10.1111/j.1742-7843.2008.00317.x

Adefegha SA, Oyeleye SI, Oboh G. Distribution of phenolic contents, antidiabetic potentials, antihypertensive properties, and antioxidative effects of soursop (*Annona muricata* L.) fruit parts in vitro. Biochemistry research international. 2015 Dec 15;2015. https://doi.org/10.1155/2015/347673

Adefegha SA, Oyeleye SI, Oboh G. Distribution of phenolic contents, antidiabetic potentials, antihypertensive properties, and antioxidative effects of soursop (*Annona muricata* L.) fruit parts in vitro. Biochemistry research international. 2015 Dec 15;2015. https://doi.org/10.1155/2015/347673

Adefuye AO, Samie A, Ndip RN. In-vitro evaluation of the antimicrobial activity of extracts of *Bridelia micrantha* on selected bacterial pathogens. Journal of Medicinal Plants Research. 2011 Sep 30;5(20):5116-22.

Adesanwo JK, Ajayi IS, Ajayi OS, Igbeneghu OA, McDonald AG. Identification of Chemical Constituents and Evaluation of the Antibacterial Activity of Methanol Extract and Fractions of the Leaf of *Melanthera scandens* (Schum. et Thonn.) Roberty. Journal of Exploratory Research in Pharmacology. 2019 Sep 30;4(3):31-40. doi: 10.14218/JERP.2019.00007

Adeyemi OO, Aigbe FR, Uyaiabasi NG. Analgesic and anti-inflammatory activities of the aqueous stem and leaf extract of *Asystasia gangetica* (Linn) T. Anderson. Nigerian quarterly journal of hospital medicine. 2011;21(2):129-34.

Adeyemi OO, Yemitan OK, Afolabi L. Inhibition of chemically induced inflammation and pain by orally and topically administered leaf extract of *Manihot esculenta* Crantz in rodents. Journal of Ethnopharmacology. 2008 Sep 2;119(1):6-11. https://doi.org/10.1016/j.jep.2008.05.019

Adiguzel AH, Ozer H, Sokmen M, Gulluce ME, Sokmen A, Kilic H, Sahin F, Baris O. Antimicrobial and antioxidant activity of the essential oil and methanol extract of *Nepeta cataria*. Polish Journal of Microbiology. 2009 Dec;58(1):69-76.

Adjagba M, Awede B, Osseni R, Hountondji C, Dougnon G, Lagnika L, Darboux R, Laleye A. Antihypertensive effect of extracts from *Crateva adansonii* DC. ssp. adansonii in the Wistar rats. International Journal of Biological and Chemical Sciences. 2017;11(6):2604-15. doi: 10.4314/ijbcs.v11i6.5

Adjei-Fremah S, Jackai LE, Schimmel K, Worku M. 0179 Immunomodulatory activities of polyphenol extract from cowpea (*Vigna unguiculata*) on bovine polymorphonuclear neutrophils. Journal of Animal Science. 2016 Oct 1;94(suppl_5):86-7. https://doi.org/10.2527/jam2016-0179

Adnyana IMO, Sudewi AR, Samatra DP, Suprapta DN. Neuroprotective Effects of Purple Sweet Potato Balinese Cultivar in Wistar Rats With Ischemic Stroke. *Open Access Maced J Med Sci*. 2018;6(11):1959–1964. Published 2018 Nov 15. doi:10.3889/oamjms.2018.435

Advani U, Anwar A, Menghani EK. Anticonvulsant potentials of *Sesamum indicum* and *Allium sativum* oil alone and in combination in animal models. Int J Pharm Pharm Sci. 2011 Jul;3(4):154-8.

Adzu B, Amos S, Dzarma S, Muazzam I, Gamaniel KS. Pharmacological evidence favouring the folkloric use of *Diospyros mespiliformis* Hochst in the relief of pain and fever. Journal of ethnopharmacology. 2002 Oct 1;82(2-3):191-5.https://doi.org/10.1016/S0378-8741(02)00179-4

Adzu B, Balogun SO, Pavan E, Ascêncio SD, Soares IM, Aguiar RW, Ribeiro RV, de Oliveira RG, da Silva LI, Damazo AS, de Oliveira Martins DT. Evaluation of the safety, gastroprotective activity and mechanism of action of standardised leaves infusion extract of *Copaifera malmei* Harms. Journal of ethnopharmacology. 2015 Dec 4;175:378-89. doi.org/10.1016/j.jep.2015.09.027

Adzu B, Haruna AK. Studies on the use of *Zizyphus spina-christi* against pain in rats and mice. African Journal of Biotechnology. 2007;6(11).

Afifi FU, Aburjai T. Antiplatelet activity of *Varthemia iphionoides*. Fitoterapia. 2004 Dec 1;75(7-8):629-33. https://doi.org/10.1016/j.fitote.2004.04.014

Afsar T, Razak S, Khan MR, Almajwal A. Anti-depressant and anxiolytic potential of *Acacia hydaspica* R. Parker aerial parts extract: Modulation of brain antioxidant enzyme status. BMC complementary and alternative medicine. 2017 Dec;17(1):1-2. http://doi.org/10.1186/s12906-017-1671-x

Agarwal A, Malini S, Bairy KL, Rao MS. Effect of *Tinospora cardifolia* on learning and memory in normal and memory deficit rats. Indian journal of pharmacology. 2002;34(5):339-49.

Agarwal SS, Kalpana S. Anti-inflammatory activity of flowers of *Rhododendron arboreum* (Smith) in Rat's hind paw oedema induced by various phlogistic agents. Indian Journal of Pharmacology. 1988 Apr 1;20(2):86.

Agarwal T, Singh R, Shukla AD, Waris I. In vitro study of antibacterial activity of *Carissa carandas* leaf extracts. Asian J. Plant Sci. Res. 2012;2(1):36-40.

Agbankpe AJ, Dougnon TV, Bankole SH, Houngbegnon O, Dah-Nouvlessounon D. In vitro antibacterial effects of *Crateva adansonii, Vernonia amygdalina* and *Sesamum radiatum* used for the treatment of infectious diarrhoeas in Benin. Journal of Infectious Diseases & Therapy. 2016 Jun 2;2016. http://dx.doi.org/10.4172/2332-0877.1000281

Agbedahunsi JM, Fakoya FA, Adesanya SA. Studies on the anti-inflammatory and toxic effects of the stem bark of *Khaya ivorensis* (Meliaceae) on rats. Phytomedicine. 2004 Sep 20;11(6):504-8. https://doi.org/10.1016/j.phymed.2003.07.009

Aggarwal KK, Khanuja SP, Ahmad A, Santha Kumar TR, Gupta VK, Kumar S. Antimicrobial activity profiles of the two enantiomers of limonene and carvone isolated from the oils of *Mentha spicata* and *Anethum sowa*. Flavour and Fragrance Journal. 2002 Jan;17(1):59-63.

Aghazadeh S, Amini R, Yazdanparast R, Ghaffari SH. Anti-apoptotic and anti-inflammatory effects of *Silybum marianum* in treatment of experimental steatohepatitis. Experimental and toxicologic pathology. 2011 Sep 1;63(6):569-74. https://doi.org/10.1016/j.etp.2010.04.009

Agnihotri S, Wakode S, Ali M. Chemical composition, antimicrobial and topical anti-inflammatory activity of *Valeriana jatamansi* Jones. essential oil. Journal of Essential Oil Bearing Plants. 2011 Jan 1;14(4):417-22. https://doi.org/10.1080/0972060X.2011.10643596

Agra IK, Pires LL, Carvalho PS, Silva-Filho EA, Smaniotto S, Barreto E. Evaluation of wound healing and antimicrobial properties of aqueous extract from *Bowdichia virgilioides* stem barks in mice. Anais da Academia Brasileira de Ciências. 2013 Sep;85(3):945-54.

Agyare C, Dwobeng AS, Agyepong N, Boakye YD, Mensah KB, Ayande PG, Adarkwa-Yiadom M. Antimicrobial, antioxidant, and wound healing properties of *Kigelia africana* (Lam.) Beneth. and *Strophanthus hispidus* DC. Advances in pharmacological sciences. 2013 Oct;2013. https://doi.org/10.1155/2013/692613

Agyare C, Mensah AY, Osei-Asante S. Antimicrobial activity and phytochemical studies of some medicinal plants from Ghana. Boletín Latinoamericano y del Caribe de Plantas Medicinales y Aromáticas 2006 Nov; 5(6):113 - 117.

Agyare C, Owusu-Ansah A, Ossei PP, Apenteng JA, Boakye YD. Wound healing and anti-infective properties of *Myrianthus arboreus* and *Alchornea cordifolia*. Medicinal chemistry, 2014, 4:7 doi.org/10.4172/2161-0444.1000191

Ahmad I, Beg AZ. Antimicrobial and phytochemical studies on 45 Indian medicinal plants against multi-drug resistant human pathogens. Journal of ethnopharmacology. 2001 Feb 1;74(2):113-23.

Ahmad I. Isolation of angiotensin converting enzyme (ACE) inhibitory activity quercetin from *Peperomia pellucida*. /International Journal of PharmTech Research, 2016, 9(7): 115-121.

Ahmad S, AbdEl-Salam NM, Ullah R. In vitro antimicrobial bioassays, DPPH radical scavenging activity, and FTIR spectroscopy analysis of *Heliotropium bacciferum*. BioMed research international. 2016 Aug 11;2016. https://doi.org/10.1155/2016/3818945

Ahmadi M, Taherianfard M, Shomali T. *Zataria multiflora* could improve hippocampal tau protein and TNFα levels and cognitive behavior defects in a rat model of Alzheimer's disease. *Avicenna J Phytomed*. 2019;9(5):465–473.

Ahmed D, Chaudhary MA, Raza A, Waheed A, Khan SR, Ikram M. Comparative Study of Antibacterial Activity and Mineral Contents of Various Parts of *Verbena officinalis* Linn. Asian Journal of Chemistry. 2012 Jan 1;24(1):68.

Ahmed DB, Chaieb I, Salah KB, Boukamcha H, Jannet HB, Mighri Z, Daami-Remadi M. Antibacterial and antifungal activities of *Cestrum parqui* saponins: possible interaction with membrane sterols. International Research Journal of Plant Science. 2012;3:001-7.

Ahmed F, Reza MS. Antibacterial and Antinociceptive Activity of *Hoya parasitica*. Hamdard Medicus. 2008;51(3).

Ahmed M, Amin S, Islam M, Takahashi M, Okuyama E, Hossain CF. Analgesic principle from *Abutilon indicum*. Die pharmazie. 2000 Apr;55(4):314-6.

Ahmed M, Azmat A. Decreased brain serotonin turnover rate following administration of Sharbat-e-Ahmed Shah produces antidepressant and anxiolytic effect in rats. Metabolic brain disease. 2017 Dec;32(6):1785-90. https://doi.org/10.1007/s11011-017-0065-6

Ahmed S, Liu H, Ahmad A, Akram W, Abdelrahman EK, Ran F, Ou W, Dong S, Cai Q, Zhang Q, Li X. Characterization of anti-bacterial compounds from the seed coat of Chinese windmill palm tree (*Trachycarpus fortunei*). Frontiers in microbiology. 2017 Oct 4;8:1894. https://doi.org/10.3389/fmicb.2017.01894

Ahmed SA, Hanif S, Iftkhar T. Phytochemical profiling with antioxidant and antimicrobial screening of *Amaranthus viridis* L. leaf and seed extracts. Open Journal of Medical Microbiology. 2013 Sep 18;2013. doi:10.4236/ojmm.2013.33025

Ahmed TA, Al Naemi H. Biological activities of *Lycium shawii* Leaves Extract. Int. J. Pharm. & Biol. Arch. 2012; 3(3):697-700.

Aires A, Carvalho R. Screening of polyphenol composition and antiradical capacity of wild *Erica arborea*: a native plant species from the Portuguese flora. J Chromatogr Sep Tech. 2017;8(379):2.

Aisaka K, Hattori Y, Kihara T, Ishihara T, Endo K, Hikino H. Hypotensive action of 3α-dihydrocadambine, an indole alkaloid glycoside of *Uncaria* hooks. Planta medica. 1985 Oct;51(05):424-7. doi: 10.1055/s-2007-969538

Aiyelaagbe OO, Adesogan K, Ekundayo O, Gloer JB. Antibacterial diterpenoids from *Jatropha podagrica* Hook. Phytochemistry. 2007 Oct 1;68(19):2420-5. https://doi.org/10.1016/j.phytochem.2007.05.021

Ajaib M, Almas M, Khan KM, Perveen S, Shah S. Phytochemical Screening, Antimicrobial and Antioxidant Activities of *Ficus natalensis*. Journal of the Chemical Society of Pakistan. 2016 Apr 1;38(2).

Ajani EO, Usman LA. *Tamarindus indica* Fruit Pulp Restores Reproductive Function in Sodium Fluoride Administered Rats. The FASEB Journal. 2020 Apr;34(S1):1-. https://doi.org/10.1096/fasebj.2020.34.s1.00285

Ajayi IA, Ojelere O. Evaluation of the Antimicrobial Properties of the Ethanolic Extracts of some Medicinal Plant Seeds from South-West Nigeria. IOSR J Pharm Biol Sci. 2014;9(4):80-5.

Ajebli M, Eddouks M. Antihypertensive activity of *Petroselinum crispum* through inhibition of vascular calcium channels in rats. Journal of ethnopharmacology. 2019 Oct 5;242:112039. https://doi.org/10.1016/j.jep.2019.112039

Ajebli M, Eddouks M. Vasorelaxant and Antihypertensive Effects of *Mentha pulegium* L. in Rats: An in vitro and in vivo Approach. Endocrine, Metabolic & Immune Disorders Drug Targets. 2020 Sep 8. doi: 10.2174/1871530320666200909093908

Ajibesin KK, Essien EE, Adesanya SA. Antibacterial constituents of the leaves of *Dacryodes edulis*. African Journal of Pharmacy and Pharmacology. 2011 Oct 1;5(15):1782-6. doi: 10.5897/AJPP11.371

Ajibesin KK, Rene N, Bala DN, Essiett UA. Antimicrobial activities of the extracts and fractions of *Allanblackia floribunda*. Biotechnology. 2008;7(1):129-33.

Akharaiyi FC, Boboye B, Adetuyi FC. Antibacterial, phytochemical and antioxidant activities of the leaf extracts of *Gliricidia sepium* and *Spathodea campanulata*. World Applied Sciences Journal. 2012;16(4):523-30.

Akhavan N, Feresin R, Johnson S, Pourafshar S, Elam M, Hsieh YH, Salazar G, Arjmandi B. *Cornus officinalis* Modulates the production of pro-inflammatory molecules in lipopolysaccharide-activated RAW264. 7 macrophages. The FASEB Journal. 2015 Apr;29(1_supplement):922-30.

Akhigbemen AM, Ozolua RI, Bafor EE, Okwuofu EO. Evaluation of some neuropharmacological effects of *Caladium bicolor* aiton (araceae) leaf extracts in mice. Metabolic brain disease. 2019 Apr 15;34(2):537-44. doi: 10.1007/s11011-019-0390-z

Akhondzadeh S, Noroozian M, Mohammadi M, Ohadinia S, Jamshidi AH, Khani M. *Salvia officinalis* extract in the treatment of patients with mild to moderate Alzheimer's disease: a double blind, randomized and placebo-controlled trial. J Clin Pharm Ther. 2003 Feb;28(1):53-9. doi: 10.1046/j.1365-2710.2003.00463.x.

Akhtar A, Deshmukh AA, Bhonsle AV, Kshirsagar PM, Kolekar MA. In vitro antibacterial activity of *Pimpinella anisum* fruit extracts against some pathogenic bacteria. Veterinary World. 2008 Sep 1;1(9):272-4.

Akhtar NM, Naseer R, Farooqi AZ, Aziz W, Nazir M. Oral enzyme combination versus diclofenac in the treatment of osteoarthritis of the knee--a double-blind prospective randomized study. Clin Rheumatol. 2004 Oct;23(5):410-5. doi: 10.1007/s10067-004-0902-y.

Akihisa T, Kojima N, Kikuchi T, Yasukawa K, Tokuda H, Masters ET, Manosroi A, Manosroi J. Anti-inflammatory and chemopreventive effects of triterpene cinnamates and acetates from shea fat. Journal of oleo science. 2010;59(6):273-80.

Akilen R, Tsiami A, Devendra D, Robinson N. Glycated haemoglobin and blood pressure-lowering effect of cinnamon in multi-ethnic Type 2 diabetic patients in the UK: a randomized, placebo-controlled, double-blind clinical trial. Diabetic Medicine. 2010 Oct;27(10):1159-67. doi.org/10.1111/j.1464-5491.2010.03079.x

Akinjogunla OJ, Adegoke AA, Udokang IP, Adebayo-Tayo BC. Antimicrobial potential of Nymphaea lotus (Nymphaeaceae) against wound pathogens. Journal of medicinal plants Research. 2009 Mar 31;3(3):138-41.

Akinpelu DA. Antimicrobial activity of *Vernonia amygdalina* leaves. Fitoterapia. 1999 Aug 1;70(4):432-4. https://doi.org/10.1016/S0367-326X(99)00061-1

Akinsulire OR, Aibin IE, Adenipekun T, Adelowotan T, Odugbemi T. In vitro antimicrobial activity of crude extracts from plants *Bryophyllum pinnatum* and *Kalanchoe crenata*. African Journal of Traditional, Complementary and Alternative Medicines. 2007;4(3):338-44.

Akinyemi KO, Oladapo O, Okwara CE, Ibe CC, Fasure KA. Screening of crude extracts of six medicinal plants used in South-West Nigerian unorthodox medicine for anti-methicillin resistant *Staphylococcus aureus* activity. BMC complementary and alternative medicine. 2005 Dec;5(1):6.

Akizuki M, Yamashita H, Uemura K, Maruyama H, Kawakami H, Ito H, Takahashi R. Optineurin suppression causes neuronal cell death via NF-κB pathway. Journal of neurochemistry. 2013 Sep;126(6):699-704. https://doi.org/10.1111/jnc.12326

Aktan F, Henness S, Roufogalis BD, Ammit AJ. Gypenosides derived from Gynostemma pentaphyllum suppress NO synthesis in murine macrophages by inhibiting iNOS enzymatic activity and attenuating NF-κB-mediated iNOS protein expression. Nitric oxide. 2003 Jun 1;8(4):235-42. https://doi.org/10.1016/S1089-8603(03)00032-6

Akula US, Odhav B. In vitro 5-lipoxygenase inhibition of polyphenolic antioxidants from undomesticated plants of South Africa. Journal of Medicinal Plants Research. 2008 Sep 1;2(9):207-12.

Akunne TC, Obi BC, Akpa PA, Udegbunam SO, Anaenugwu AJ. Antibacterial, antifungal and wound healing potentials of extract and fractions of *Nauclea diderrichii* root bark. International Journal of Traditional and Complementary Medicine. 2017;2:1.

Akuodor GC, Essien AD, Akpan JL, Chilaka KC, Uwaezuoke NJ, Nwadike KI, Nwobodo NN, Ezeokpo BC. Analgesic, anti-inflammatory and antipyretic activities of methanolic leaf extract of *Maerua crassifolia*. Journal of Coastal Life Medicine. 2016;4(3):225-30. doi: 10.12980/jclm.4.2016j5-153

Akuodor GC, Usman MI, Ibrahim JA, Chilaka KC, Akpan JL, Dzarma S, Muazzam I, Osunkwo UA. Anti-nociceptive, anti-inflammatory and antipyretic effects of the methanolic extract of *Bombax buonopozense* leaves in rats and mice. African Journal of Biotechnology. 2011;10(16):3191-6. doi: 10.5897/AJB09.1896

Al Hasan A, Hasan CM, Azam AT. Antimicrobial, cytotoxic and antioxidant activities of *Desmodium heterocarpon*. Bangladesh Pharm. J. 2011;14:0301-4606

Al Khateeb W, Hussein E, Qouta L, Alu'datt M, Al-Shara B, Abu-Zaiton A. In vitro propagation and characterization of phenolic content along with antioxidant and antimicrobial activities of *Cichorium pumilum* Jacq. Plant Cell, Tissue and Organ Culture (PCTOC). 2012 Jul;110(1):103-10. https://doi.org/10.1007/s11240-012-0134-9

Alaba CS, Chichioco-Hernandez CL. 15–Lipoxygenase inhibition of *Commelina benghalensis*, T*radescantia fluminensis*, *Tradescantia zebrina*. Asian Pacific journal of tropical biomedicine. 2014 Mar 1;4(3):184-8. https://doi.org/10.1016/S2221-1691(14)60229-X

Alabi AO, Ajayi AM, Ben-Azu B, Omorobge O, Umukoro S. Methyl jasmonate ameliorates rotenone-induced motor deficits in rats through its neuroprotective activity and increased expression of tyrosine hydroxylase immunopositive cells. Metabolic Brain Disease. 2019 Dec 1;34(6):1723-36.

Alagan A, Jantan I, Kumolosasi E, Azmi N. *Phyllanthus amarus* protects against spatial memory impairment induced by lipopolysaccharide in mice. Bioinformation. 2019a;15(8):535. doi:10.6026/97320630015535

Alagan A, Jantan I, Kumolosasi E, Ogawa S, Abdullah MA, Azmi N. Protective effects of *Phyllanthus amarus* against lipopolysaccharide-induced neuroinflammation and cognitive impairment in rats. Frontiers in pharmacology. 2019b Jun 4;10:632. doi:10.3389/fphar.2019.00632

Alam MA, Subhan N, Awal MA, Alam MS, Sarder M, Nahar L, Sarker SD. Antinociceptive and anti-inflammatory properties of *Ruellia tuberosa*. Pharmaceutical biology. 2009 Mar 1;47(3):209-14. https://doi.org/10.1080/13880200802434575

Alam MB, Hossain MS, Chowdhury NS, Mazumder ME, Haque ME, Islam A. In vitro and in vivo antioxidant and toxicity evaluation of different fractions of *Oxalis corniculata* Linn. J Pharmacol Toxicol. 2011b;6(4):337-48. doi: 10.3923/jpt.2011.337.348

Alam MB, Hossain MS, Haque ME. Antioxidant and anti-inflammatory activities of the leaf extract of *Brassica nigra*. International Journal of Pharmaceutical Sciences and Research. 2011a Feb 1;2(2):303.

Alam MI, Gomes A. Snake venom neutralization by Indian medicinal plants (*Vitex negundo* and *Emblica officinalis*) root extracts. Journal of Ethnopharmacology. 2003 May 1;86(1):75-80. https://doi.org/10.1016/S0378-8741(03)00049-7

Alam MN, Biozid MS, Chowdhury AI, Mazumdar MM, Chowdhury S, Chowdury MI. Anti-arthritic and cytotoxic effects of methanolic extract of *Ixora nigricans* leaf. Journal of Medicinal Plants Research. 2015 Jul 3;9(25):719-23. doi: 10.5897/JMPR2015.5867

Alamgeer, Iman S, Asif H, Saleem M. Evaluation of antihypertensive potential of *Ficus carica* fruit. Pharmaceutical biology. 2017 Jan 1;55(1):1047-53. https://doi.org/10.1080/13880209.2017.1278611

Alarcón-Barrera KS, Armijos-Montesinos DS, García-Tenesaca M, Iturralde G, Jaramilo-Vivanco T, Granda-Albuja MG, Giampieri F, Alvarez-Suarez JM. Wild Andean blackberry (*Rubus glaucus* Benth) and Andean blueberry (*Vaccinium floribundum* Kunth) from the Highlands of Ecuador: Nutritional composition and protective effect on human dermal fibroblasts against cytotoxic oxidative damage. Journal of Berry Research. 2018 Jan 1;8(3):223-36. doi: 10.3233/JBR-180316

Alaribe CS, Anyakora C, Emoghene E, Ota D, De Waard M. GC–MS profile, anti-seizure and anti-pyretic activities of palm kernel nut oil and its isolate, N-Octanoic acid from specially breed palm kernel *Elaeis guineensis*. Nigerian Journal of Pharmaceutical Research. 2016;12(2):87-94.

Alasbahi RH, Safiyeva S, Craker LE. Antimicrobial activity of some Yemeni medicinal plants. Journal of herbs, spices & medicinal plants. 1999 Sep 17;6(3):75-83.

Alatshan A, Qnais E, Wedyan M, Bseiso Y, Alzyoud E, Banat R, Alkhateeb H. Antinociceptive and Antiinflammatory Activities of *Anastatica hierochuntica* and Possible Mechanism of Action. Indian Journal of Pharmaceutical Sciences. 2018 Jul 31;80(4):637-46. doi: 10.4172/pharmaceutical-sciences.1000403

Al-Bakheit AA, Abu-Romman S, Sharab A, Shhab MA. Anti-inflammatory effect of *Varthemia iphionoides* extracts against prostate cancer in vitro. European Journal of Inflammation. 2017 Apr;15(1):8-14. https://doi.org/10.1177/1721727X17702151

Albaser N, Ghanem N, Shehab M, Al-Adhal A, Amood Al-Kamarany M. Investigation of Pharmacological Activity of *Caralluma penicillata*: Anti-Inflammatory Properties and Gastritis Protection against Indomethacin in Adult Guinea Pigs. Int Sch Res Notices. 2014 Dec 4;2014:738493. doi: 10.1155/2014/738493.

Albasher G, Aljarba N, Al Sultan N, Alqahtani WS, Alkahtani S. Evaluation of the neuro-protective effect of *Artemisia judaica* extract in a murine diabetic model. Journal of Food Biochemistry. 2020 Aug;44(8):e13337. https://doi.org/10.1111/jfbc.13337

Al-Bayati FA, Sulaiman KD. In vitro antimicrobial activity of *Salvadora persica* L. extracts against some isolated oral pathogens in Iraq. Turkish Journal of Biology. 2008 Feb 19;32(1):57-62.

Alberdi E, Sánchez-Gómez MV, Ruiz A, Cavaliere F, Ortiz-Sanz C, Quintela-López T, Capetillo-Zarate E, Solé-Domènech S, Matute C. Mangiferin and Morin Attenuate Oxidative Stress, Mitochondrial Dysfunction, and Neurocytotoxicity, Induced by Amyloid Beta Oligomers. Oxidative Medicine and Cellular Longevity. 2018;2018. Article ID 2856063, 13 pages https://doi.org/10.1155/2018/2856063

Al-Bukhaiti WQ, Noman A, AL-Maktary QA, Ali AH, Wang H. Evaluation of antibacterial and antioxidant activities of *Cissus rotundifolia* (Forssk.) leaves extract obtained by ultrasonic-assisted extraction conditions. Journal of Food Measurement and Characterization. 2020 Sep 28:1-8. https://doi.org/10.1007/s11694-020-00651-6y

Alcaráz LE, Fusco MR, Mattana CM, Satorres SE, Laciar AL. Antibacterial activity of methanolic and aqueous extracts of *Ligaria cuneifolia* and *Tripodanthus flagellaris*. Emirates Journal of Food and Agriculture. 2015 Oct 20:831-6. https://doi.org/10.9755/ejfa.2015-04-124

Aldawsari HM, Eid BG, Neamatallah T, Zaitone SA, Badr JM. Anticonvulsant and Neuroprotective Activities of *Phragmanthera austroarabica* Extract in Pentylenetetrazole-Kindled Mice. *Evid Based Complement Alternat Med*. 2017;2017:5148219. doi:10.1155/2017/5148219

Alexandre-Moreira MS, Piuvezam MR, Araújo CC, Thomas G. Studies on the anti-inflammatory and analgesic activity of *Curatella americana* L. Journal of Ethnopharmacology. 1999 Nov 1;67(2):171-7.

Al-Fatimi M, Wurster M, Schröder G, Lindequist U. Antioxidant, antimicrobial and cytotoxic activities of selected medicinal plants from Yemen. Journal of ethnopharmacology. 2007 May 22;111(3):657-66. https://doi.org/10.1016/j.jep.2007.01.018

Al-Fatimi M. Ethnobotanical survey of medicinal plants in central Abyan governorate, Yemen. Journal of ethnopharmacology. 2019 Sep 15;241:111973. https://doi.org/10.1016/j.jep.2019.111973

Al-hood FA, Ali KS. Antimicrobial activity of the *Jatropha spinosa* extracts on the selected pathogenic microorganisms. World Journal of Pharmaceutical Research 2015;4(11):2050-2056.

Al-Howiriny TA, Al-Sohaibani MO, El-Tahir KH, Rafatullah S. Preliminary evaluation of the anti-inflammatory and anti-hepatotoxic activities of'parsley *'Petroselinum crispum'* in rats. Journal of natural remedies. 2003 Jan 1;3(1):54-62.

Al-Howiriny TA, Al-Yahya MA, Al-Said MS, El-Tahir KE, Rafatullah S. Studies on the pharmacological activities of an ethanol extract of balessan (*Commiphora opobalsamum*). Pakistan Journal of Biological Sciences. 2004;7(11):1933-6. doi: 10.3923/pjbs.2004.1933.1936

Ali AA, Mohammed AM, Isa AG. Antimicrobial effects of crude bromelain extracted from pineapple fruit (*Ananas comosus* (Linn.) Merr.). 2015; 3(1): 1-4 doi: 10.11648/j.ab.20150301.11

Ali B, Mujeeb M, Aeri V, Mir SR, Faiyazuddin M, Shakeel F. Anti-inflammatory and antioxidant activity of *Ficus carica* Linn. leaves. Natural product research. 2012 Mar 1;26(5):460-5.

Ali H, König GM, Khalid SA, Wright AD, Kaminsky R. Evaluation of selected Sudanese medicinal plants for their in vitro activity against hemoflagellates, selected bacteria, HIV-1-RT and tyrosine kinase inhibitory, and for cytotoxicity. Journal of ethnopharmacology. 2002 Dec 1;83(3):219-28.

Ali M, Latif A, Zaman K, Mehsud S, Akbar N, Ahmad M, Arfan M. Evaluation of antioxidant and antimicrobial activities on various extracts of Himalayan medicinal plants. Pakistan journal of pharmaceutical sciences. 2020 Mar 1;33(2).

Ali MR, Aboud AS. Antimicrobial Activities of Aqueous and Methanolic Extracts from *Salvia officinalis* and *Salix acmophylla* Used in the treatment of wound infection isolates. Ibn AL-Haitham Journal For Pure and Applied Science. 2017 May 21;23(3):25-39.

Ali NA, Sharopov FS, Alhaj M, Hill GM, Porzel A, Arnold N, Setzer WN, Schmidt J, Wessjohann L. Chemical composition and biological activity of essential oil from *Pulicaria undulata* from Yemen. Natural product communications. 2012 Feb;7(2):1934578X1200700238. https://doi.org/10.1177/1934578X1200700238

Ali RM, Houghton PJ, Raman A, Hoult JR. Antimicrobial and antiinflammatory activities of extracts and constituents of *Oroxylum indicum* (L.) Vent. Phytomedicine. 1998 Oct 1;5(5):375-81. doi.org/10.1016/S0944-7113(98)80020-2

Alirezalu A, Ahmadi N, Salehi P, Sonboli A, Alirezalu K, Mousavi Khaneghah A, Barba FJ, Munekata PE, Lorenzo JM. Physicochemical characterization, antioxidant activity, and phenolic compounds of hawthorn (*Crataegus* spp.) fruits species for potential use in food applications. Foods. 2020 Apr;9(4):436.

Ali-Shtayeh MS, Abu Ghdeib SI. Antifungal activity of plant extracts against dermatophytes. Mycoses. 1999 Dec;42(11-12):665-72. https://doi.org/10.1046/j.1439-0507.1999.00499.x

Alkuwari A, Al-Naemi, MY, Vito P. Biological activities of *Lycium shawii* Leaves Extract. International Journal ofPharmaceutical & Biological Archives 2012; 3(3):697-700.

Allahverdiyev A, Duran N, Ozguven ME, Koltas S. Antiviral activity of the volatile oils of *Melissa officinalis* L. against *Herpes simplex* virus type-2. Phytomedicine. 2004 Nov 25;11(7-8):657-61. https://doi.org/10.1016/j.phymed.2003.07.014

Al-Mamun M, Jahan N, Chowdhury MH, Jahan R, Nasrin D. Brine shrimp toxicity study of different Bangladeshi medicinal plants. Advances in Natural and Applied Sciences. 2010;4(2):163-73.

Al-Marzoqi AH, Al-Khafaji KR, Kadhim AR. Influence of the crude Phenolic, Alkaloid and Terpenoid compounds extracts of *Cardaria draba* (*Lepidium draba* L.) on Human Pathogenic Bacteria. World J Pharm Res. 2015 Apr 6;4(6):456-60.

Alo MN, Anyim C, Igwe JC, Elom M, Uchenna DS. Antibacterial activity of water, ethanol and methanol extracts of *Ocimum gratissimum, Vernonia amygdalina and Aframomum melegueta*. Adv. Appl. Sci. Res. 2012;3(2):844-8.

Alothyqi N, Almalki M, Albqa'ai M, Alsamiri H, Alrashdi SM, Ibraheem F, Osman GH. In vitro antibacterial activity of four Saudi medicinal plants. J. Microb. Biochem. Technol.. 2016;8:083-9. http://dx.doi.org/10.4172/1948-5948.1000267

Alqahtani FY, Aleanizy FS, Mahmoud AZ, Farshori NN, Alfaraj R, Al-sheddi ES, Alsarra IA. Chemical composition and antimicrobial, antioxidant, and anti-inflammatory activities of *Lepidium sativum* seed oil. Saudi journal of biological sciences. 2019 Jul 1;26(5):1089-92. https://doi.org/10.1016/j.sjbs.2018.05.007

Alqasoumi SI, Soliman GA, Awaad AS, Donia AE. Anti-inflammatory activity, safety and protective effects of *Leptadenia pyrotechnica*, *Haloxylon salicornicum* and *Ochradenus baccatus* in ulcerative colitis. Phytopharmacology. 2012;2(1):58-71.

Al-Rimawi F, Alakhras F, Al-Zereini WA, Aldal'in HK, Abu-Lafi S, Al-Mazaideh GM. HPLC Analysis of Chemical Composition of Selected Jordanian Medicinal Plants and their Bioactive Properties. Oriental Journal of Chemistry. 2018 Oct 21;34(5):2397-403. http://dx.doi.org/10.13005/ojc/340522

Al-Said MS, Tariq M, Al-Yahya MA, Rafatullah S, Ginnawi OT, Ageel AM. Studies on *Ruta chalepensis*, an ancient medicinal herb still used in traditional medicine. Journal of Ethnopharmacology. 1990 Mar 1;28(3):305-12. https://doi.org/10.1016/0378-8741(90)90081-4

Álvarez ÁL, Habtemariam S, Parra F. Inhibitory effects of lupene-derived pentacyclic triterpenoids from *Bursera simaruba* on HSV-1 and HSV-2 in vitro replication. Natural product research. 2015 Dec 17;29(24):2322-7. https://doi.org/10.1080/14786419.2015.1007456

Alves TM, Ribeiro FL, Kloos H, Zani CL. Polygodial, the fungitoxic component from the Brazilian medicinal plant *Polygonum punctatum*. Memórias do Instituto Oswaldo Cruz. 2001 Aug;96(6):831-3. doi.org/10.1590/S0074-02762001000600016

Alves TM, Silva AF, Brandão M, Grandi TS, Smânia ED, Smânia Júnior A, Zani CL. Biological screening of Brazilian medicinal plants. Memórias do Instituto Oswaldo Cruz. 2000 Jun;95(3):367-73. https://doi.org/10.1590/S0074-02762000000300012

Al-Yousuf MH, Bashir AK, Ali BH, Tanira MO, Blunden G. Some effects of *Salvia aegyptiaca* L. on the central nervous system in mice. Journal of ethnopharmacology. 2002 Jun 1;81(1):121-7. https://doi.org/10.1016/S0378-8741(02)00057-0

Al-Zubairi AS, Abdul AB, Abdelwahab SI, Peng CY, Mohan S, Elhassan MM. *Eleucine indica* possesses antioxidant, antibacterial and cytotoxic properties. Evidence-Based Complementary and Alternative Medicine. 2011;2011. doi.org/10.1093/ecam/nep091

Amabeoku GJ, Kinyua CG. Evaluation of the anticonvulsant activity of *Zanthoxylum capense* (Thunb.) Harv.(Rutaceae) in mice. Int. J. Pharmacol. 2010 Nov 1;6(6):844-53.

Amaro CA, González-Cortazar M, Herrera-Ruiz M, Román-Ramos R, Aguilar-Santamaría L, Tortoriello J, Jiménez-Ferrer E. Hypoglycemic and hypotensive activity of a root extract of *Smilax aristolochiifolia*, standardized on N-trans-feruloyl-tyramine. Molecules. 2014 Aug;19(8):11366-84. https://doi.org/10.3390/molecules190811366

Amezouar F, Badri W, Hsaine M, Bourhim N, Fougrach H. Antioxidant and anti-inflammatory activities of Moroccan Erica arborea L. Pathologie-biologie. 2013 May 4;61(6):254-8.

Amin M, Kapadnis BP. Heat stable antimicrobial activity of *Allium ascalonicum* against bacteria and fungi. Indian Journal of Experimental Biology 2005 Aug; 43(08): 751-754.

Amini A, Liu M, Ahmad Z. Understanding the link between antimicrobial properties of dietary olive phenolics and bacterial ATP synthase. International journal of biological macromolecules. 2017 Aug 1;101:153-64. https://doi.org/10.1016/j.ijbiomac.2017.03.087

Amira S, Dade M, Schinella G, Ríos JL. Anti-inflammatory, anti-oxidant, and apoptotic activities of four plant species used in folk medicine in the Mediterranean basin. Pak J Pharm Sci. 2012 Jan 1;25(1):65-72.

Amiri MS, Joharchi MR. Ethnobotanical investigation of traditional medicinal plants commercialized in the markets of Mashhad, Iran. Avicenna Journal of Phytomedicine. 2013;3(3):254-71.

Amjad MS, Qaeem MF, Ahmad I, Khan SU, Chaudhari SK, Zahid Malik N, Shaheen H, Khan AM. Descriptive study of plant resources in the context of the ethnomedicinal relevance of indigenous flora: A case study from Toli Peer National Park, Azad Jammu and Kashmir, Pakistan. PloS one. 2017 Feb 13;12(2):e0171896.

Amoateng P, Osei-Safo D, Kukuia KK, Adjei S, Akure OA, Agbemelo-Tsomafo C, Adu-Poku SN, Agyeman-Badu KY. Psychotropic Effects of an Alcoholic Extract from the Leaves of *Albizia zygia* (Leguminosae-Mimosoideae). Evidence-Based Complementary and Alternative Medicine. 2017;2017. Article ID 9297808 https://doi.org/10.1155/2017/9297808

Amog PU, Manjuprasanna VN, Yariswamy M, Nanjaraj Urs AN, Joshi V, Suvilesh KN, Nataraju A, Vishwanath BS, Gowda TV. *Albizia lebbeck* seed methanolic extract as a complementary therapy to manage local toxicity of *Echis carinatus* venom in a murine model. Pharmaceutical biology. 2016 Nov 1;54(11):2568-74.

Amoros M, Fauconnier B, Girre RL. In vitro antiviral activity of a saponin from *Anagallis arvensis*, Primulaceae, against herpes simplex virus and poliovirus. Antiviral research. 1987 Aug 1;8(1):13-25. https://doi.org/10.1016/0166-3542(87)90084-2

Amoroso VB, Antesa DA, Buenavista DP, Coritico FP. Antimicrobial, antipyretic, and anti-inflammatory activities of selected Philippine medicinal pteridophytes. Asian Journal of Biodiversity Vol. 2014 Jan 5.

Amos S, Chindo B, Edmond I, Akah P, Wambebe C, Gamaniel K. Anti-inflammatory and anti-nociceptive effects of *Ficus platyphylla* extract in mice and rats. Journal of herbs, spices & medicinal plants. 2002 Feb 4;9(1):47-53.

Amri E, Kisangau DP. Ethnomedicinal study of plants used in villages around Kimboza forest reserve in Morogoro, Tanzania. Journal of ethnobiology and ethnomedicine. 2012 Dec;8(1):1-9. https://doi.org/10.1186/1746-4269-8-1

Amsterdam JD, Li Y, Soeller I, Rockwell K, Mao JJ, Shults J. A randomized, double-blind, placebo-controlled trial of oral *Matricaria recutita* (chamomile) extract therapy for generalized anxiety disorder. J Clin Psychopharmacol. 2009;29(4):378–382. doi:10.1097/JCP.0b013e3181ac935c AMA

Amugune BK, Mwangi JW, Thoithi GN, Kibwage IO. In Vitro Screening of Ten Selected Traditionally Used Medicinal Plants in Vihiga County, Kenya for Antibacterial and Antifungal Activity. International Journal of Medicinal Plants and Natural Products 2017; 3(2):37-44. http://dx.doi.org/10.20431/2454-7999.0302005

Anandarajagopal K, Sunilson JA, Ajaykumar TV, Ananth R, Kamal S. In-vitro anti-inflammatory evaluation of crude *Bombax ceiba* extracts. European Journal of Medicinal Plants. 2013 Jan 1;3(1):99.

Anderson EF. Ethnobotany of hill tribes of northern Thailand. I. Medicinal plants of Akha. Economic Botany. 1986 Jan 1;40(1):38-53.

Andrade MA, Cardoso MG, Batista LR, Freire JM, Nelson DL. Antimicrobial activity and chemical composition of essential oil of Pelargonium odoratissimum. Revista Brasileira de Farmacognosia. 2011 Feb;21(1):47-52. doi.org/10.1590/S0102-695X2011005000009

Andreu GL, Maurmann N, Reolon GK, de Farias CB, Schwartsmann G, Delgado R, Roesler R. Mangiferin, a naturally occurring glucoxilxanthone improves long-term object recognition memory in rats. European journal of pharmacology. 2010 Jun 10;635(1-3):124-8. doi:10.1016/j.ejphar.2010.03.011

Andriani Y, Ramli NM, Syamsumir DF, Kassim MN, Jaafar J, Aziz NA, Marlina L, Musa NS, Mohamad H. Phytochemical analysis, antioxidant, antibacterial and cytotoxicity properties of keys and cores part of *Pandanus tectorius* fruits. Arabian Journal of Chemistry. 2019 Dec 1;12(8):3555-64. https://doi.org/10.1016/j.arabjc.2015.11.003

Andrighetti-Fröhner C, Sincero TC, Da Silva AC, Savi LA, Gaido CM, Bettega JM, Mancini M, De Almeida MT, Barbosa RA, Farias MR, Barardi CR. Antiviral evaluation of plants from Brazilian atlantic tropical forest. Fitoterapia. 2005 Jun 1;76(3-4):374-8. https://doi.org/10.1016/j.fitote.2005.03.010

Angmo K, Adhikari BS, Rawat GS. Changing aspects of traditional healthcare system in Western Ladakh, India. Journal of ethnopharmacology. 2012 Sep 28;143(2):621-30.

Anitha G, Sudarsanam G. Studies on antimicrobial activity of *Boswellia ovalifoliolata* against *Xanthomonas citri* and *Salmonella typhynurium*. Int J Appl Biol Pharm. 2013;4(1):243-7.https://doi.org/10.1161/01.ATV.3.2.178

Anosike CA, Obidoa O, Ezeanyika LU. The anti—inflammatory activity of garden egg (*Solanum aethiopicum*) on egg albumin—induced oedema and granuloma tissue formation in rats. Asian Pacific journal of tropical medicine. 2012 Jan 1;5(1):62-6. https://doi.org/10.1016/S1995-7645(11)60247-2

Anreddy RN, Porika M, Yellu NR, Devarakonda RK. Hypoglycemic and hypolipidemic activities of *Trianthema portulacastrum* Linn. plant in normal and alloxan induced diabetic rats. Int J Pharmacol. 2010 Mar 1;6(2):129-33. doi: 10.3923/ijp.2010.129.133

Antonisamy P, Subash-Babu P, Alshatwi AA, Aravinthan A, Ignacimuthu S, Choi KC, Kim JH. Gastroprotective effect of nymphayol isolated from *Nymphaea stellata* (Willd.) flowers: contribution of antioxidant, anti-inflammatory and anti-apoptotic activities. Chemico-biological interactions. 2014 Dec 5;224:157-63. https://doi.org/10.1016/j.cbi.2014.09.020

Antoun MD, Martinez E, Caballero R, Oquendo I, Proctor GR, Weislow OS, McCloud TG, Kiser R, Staley P, Clanton D. Evaluation of the flora of Puerto Rico for in vitro cytotoxic and anti-HIV activities. Pharmaceutical biology. 1999 Jan 1;37(4):277-80.

Anuradha V, Srinivas PV, Rao RR, Manjulatha K, Purohit MG, Rao JM. Isolation and synthesis of analgesic and anti-inflammatory compounds from *Ochna squarrosa* L. Bioorganic & medicinal chemistry. 2006 Oct 15;14(20):6820-6. https://doi.org/10.1016/j.bmc.2006.06.048

Anyasor GN, Onajobi F, Osilesi O, Adebawo O, Oboutor EM. Anti-inflammatory and antioxidant activities of *Costus afer* Ker Gawl. hexane leaf fraction in arthritic rat models. Journal of ethnopharmacology. 2014 Aug 8;155(1):543-51. https://doi.org/10.1016/j.jep.2014.05.057

Anywar GU, Kirimuhuzya C. Phytochemical profile and antibacterial activity of crude extracts of the pod of *Aframomum angustifolium* (Sonn.) K. Schum. European Journal of biological research. 2015 Jun 26;5(2):36-41.

Ao C, Li A, Elzaawely AA, Xuan TD, Tawata S. Evaluation of antioxidant and antibacterial activities of *Ficus microcarpa* L. fil. extract. Food control. 2008 Oct 1;19(10):940-8. https://doi.org/10.1016/j.foodcont.2007.09.007

Aouey B, Samet AM, Fetoui H, Simmonds MS, Bouaziz M. Anti-oxidant, anti-inflammatory, analgesic and antipyretic activities of grapevine leaf extract (*Vitis vinifera*) in mice and identification of its active constituents by LC–MS/MS analyses. Biomedicine & pharmacotherapy. 2016 Dec 1;84:1088-98. https://doi.org/10.1016/j.biopha.2016.10.033

Apaza L, Pérez VT, Serban AM, Navarro MJ, Rumbero A. Alkamides from *Tropaeolum tuberosum* inhibit inflammatory response induced by TNF–α and NF–κB. Journal of ethnopharmacology. 2019 May 10;235:199-205. https://doi.org/10.1016/j.jep.2019.02.015

Apema R, Mozouloua D, Kosh-Komba E, Ngoule Y. Medicinal plants used in the treatment of high blood pressure by the traditional healers in Bangui. In Systematics and conservation of African plants. Proceedings of the 18th AETFAT Congress, Yaoundé, Cameroun, 26 February to 2 March 2007 2010 (pp. 305-311). Royal Botanic Gardens, Kew, UK

Apers S, Cimanga K, Berghe DV, Van Meenen E, Longanga AO, Foriers A, Vlietinck A, Pieters L. Antiviral activity of simalikalactone D, a quassinoid from *Quassia africana*. Planta medica. 2002 Jan;68(01):20-4. doi: 10.1055/s-2002-19870

Apu AS, Muhit MA, Tareq SM, Pathan AH, Jamaluddin AT, Ahmed M. Antimicrobial Activity and Brine Shrimp Lethality Bioassay of the Leaves Extract of *Dillenia indica* Linn. Journal of Young Pharmacists. 2010 Jan 1;2(1):50-3. https://doi.org/10.4103/0975-1483.62213

Aqil F, Ahmad I. Broad-spectrum antibacterial and antifungal properties of certain traditionally used Indian medicinal plants. World journal of microbiology and biotechnology. 2003 Aug 1;19(6):653-7.

Aquino JD, Soares JK, Magnani M, Stamford TC, Mascarenhas RD, Tavares RL, Stamford TL. Effects of dietary brazilian palm oil (*Mauritia flexuosa* L.) on cholesterol profile and vitamin A and E status of rats. Molecules. 2015 May;20(5):9054-70. https://doi.org/10.3390/molecules20059054

Arab L, Ang A. A cross sectional study of the association between walnut consumption and cognitive function among adult US populations represented in NHANES. The journal of nutrition, health & aging. 2015 Mar;19(3):284-90.

Arabzadeh AM, Ansari-Dogaheh M, Sharififar F, Shakibaie M, Heidarbeigi M. Anti *Herpes simplex*-1 activity of a standard extract of Zataria multiflora Boiss. Pak J Biol Sci. 2013 Feb 15;16(4):180-4. doi: 10.3923/pjbs.2013.180.184

Aragão GF, Carneiro LM, Junior AP, Vieira LC, Bandeira PN, Lemos TL, Viana GD. A possible mechanism for anxiolytic and antidepressant effects of alpha-and beta-amyrin from *Protium heptaphyllum* (Aubl.) March. Pharmacology Biochemistry and Behavior. 2006 Dec 1;85(4):827-34. https://doi.org/10.1016/j.pbb.2006.11.019

Araki R, Sasaki K, Onda H, Nakamura S, Kassai M, Kaneko T, Isoda H, Hashimoto K. Effects of Continuous Intake of Rosemary Extracts on Mental Health in Working Generation Healthy Japanese Men: Post-Hoc Testing of a Randomized Controlled Trial. Nutrients. 2020 Nov 20;12(11):3551. doi: 10.3390/nu12113551

Arawwawala LD, Arambewela LS, Ratnasooriya WD. *Alpinia calcarata* Roscoe: A potent antiinflammatory agent. Journal of ethnopharmacology. 2012 Feb 15;139(3):889-92.

Arbab AH, Parvez MK, Al-Dosari MS, Al-Rehaily AJ, Al-Sohaibani M, Zaroug EE, AlSaid MS, Rafatullah S. Hepatoprotective and antiviral efficacy of *Acacia mellifera* leaves fractions against hepatitis B virus. BioMed research international. 2015 Jan 1;2015. https://doi.org/10.1155/2015/929131

Arbab AH, Parvez MK, Al-Dosari MS, Al-Rehaily AJ. *In vitro* evaluation of novel antiviral activities of 60 medicinal plants extracts against hepatitis B virus. *Experimental and Therapeutic Medicine*. 2017;14(1):626-634. doi:10.3892/etm.2017.4530.

Aremu OO, Tata CM, Sewani-Rusike CR, Oyedeji AO, Oyedeji OO, Nkeh-Chungag BN. Phytochemical composition, and analgesic and antiinflammatory properties of essential oil of *Chamaemelum nobile* (Asteraceae L All) in rodents. Tropical Journal of Pharmaceutical Research. 2018;17(10):1939-45. doi: 10.4314/tjpr.v17i10.7

Ariaee N, Ghorbani J, Panahi M, et al. Oral Administration of *Zataria multiflora* Extract Decreases IL-17 Expression in Perennial Allergic Rhinitis. *Rep Biochem Mol Biol*. 2018;6(2):203–207.

Arirudran B, Saraswathy A, Krishnamurthy V. Antimicrobial activity of *Ruellia tuberosa* L.(whole plant). Pharmacognosy journal. 2011 Jul 1;3(23):91-5. https://doi.org/10.5530/pj.2011.23.14

Arjun P, Vincent SG, Kannan RR. HPLC-PDA isolation and LC-MS/MS detection of an acetylcholinesterase inhibitory flavonoid from *Tephrosia purpurea* (L.) Pers. in zebrafish brain. *Int. J. Biol. Biotech* 2017; 14 (2): 173-178.

Armijos CP, Meneses MA, Guamán-Balcázar MC, Cuenca M, Suárez AI. Antioxidant properties of medicinal plants used in the Southern Ecuador. Journal of Pharmacognosy and Phytochemistry. 2018;7(1):2803-12.

Arnold HJ, Gulumian M. Pharmacopoeia of traditional medicine in Venda. J Ethnopharmacol. 1984 Oct;12(1):35-74. doi: 10.1016/0378-8741(84)90086-2

Arora DS, Mahajan H. In vitro evaluation and statistical optimization of antimicrobial activity of *Prunus cerasoides* stem bark. Applied biochemistry and biotechnology. 2018 Mar 1;184(3):821-37. https://doi.org/10.1007/s12010-017-2571-8

Arrigoni-Blank M, Dmitrieva EG, Franzotti EM, Antoniolli AR, Andrade MR, Marchioro M. Anti-inflammatory and analgesic activity of *Peperomia pellucida* (L.) HBK (Piperaceae). Journal of Ethnopharmacology. 2004 Apr 1;91(2-3):215-8. doi.org/10.1016/j.jep.2003.12.030

Arthan D, Svasti J, Kittakoop P, Pittayakhachonwut D, Tanticharoen M, Thebtaranonth Y. Antiviral isoflavonoid sulfate and steroidal glycosides from the fruits of *Solanum torvum*. Phytochemistry. 2002 Feb 1;59(4):459-63. https://doi.org/10.1016/S0031-9422(01)00417-4

Arlt S. Non-Alzheimer's disease—related memory impairment and dementia. Dialogues in Clinical Neuroscience. 2022 Apr 1. 15:4, 465-473. DOI: 10.31887/DCNS.2013.15.4/sarlt

Arul V, Miyazaki S, Dhananjayan R. Studies on the anti-inflammatory, antipyretic and analgesic properties of the leaves of *Aegle marmelos* Corr. Journal of Ethnopharmacology. 2005 Jan 4;96(1-2):159-63.

Arulappan MT, Britto SJ, Ruckmani K, Kumar RM. An ethnobotanical study of medicinal plants used by ethnic people in Gingee hills, Villupuram district, Tamilnadu, India. American Journal of Ethnomedicine. 2015;2(2):2348-9502

Arullappan S, Rajamanickam P, Thevar N, Kodimani CC. In vitro screening of cytotoxic, antimicrobial and antioxidant activities of *Clinacanthus nutans* (Acanthaceae) leaf extracts. Tropical Journal of Pharmaceutical Research. 2014 Oct 10;13(9):1455-61. doi: 10.4314/tjpr.v13i9.11

Arulmozhi S, Mazumder PM, Sathiya NP, Thakurdesai A. Antianxiety and antidepressant activity of leaves of *Alstonia scholaris* Linn R. Br. Pharmacologia. 2012;3(8):239-48.

Arunachalam G, Chattopadhyay D, Chatterjee S, Mandal AB, Sur TK, Mandal SC. Evaluation of anti-inflammatory activity of *Alstonia macrophylla* Wall ex A. DC. leaf extract. Phytomedicine. 2002 Jan 1;9(7):632-5.

Arunachalam KD, Velmurugan P, Raja RB. Anti-inflammatory and cytotoxic effects of extract from *Plumbago zeylanica*. African journal of microbiology research. 2010 Jun 18;4(12):1239-45.

Aruoma OI, Spencer JP, Rossi R, Aeschbach R, Khan A, Mahmood N, Munoz A, Murcia A, Butler J, Halliwell B. An evaluation of the antioxidant and antiviral action of extracts of rosemary and Provencal herbs. Food and Chemical Toxicology. 1996 May 1;34(5):449-56. https://doi.org/10.1016/0278-6915(96)00004-X

Arweiler NB, Pergola G, Kuenz J, Hellwig E, Sculean A, Auschill TM. Clinical and antibacterial effect of an anti-inflammatory toothpaste formulation with Scutellaria baicalensis extract on experimental gingivitis. Clin Oral Investig. 2011 Dec;15(6):909-13. doi: 10.1007/s00784-010-0471-1.

Asad MH, Razi MT, Durr-e-Sabih, Najamus-Saqib Q, Nasim SJ, Murtaza G, Hussain I. Anti-venom potential of Pakistani medicinal plants: inhibition of anticoagulation activity of *Naja naja karachiensis* toxin. Current Science. 2013 Nov 25:1419-24.

Asamenew G, Bisrat D, Mazumder A, Asres K. In vitro antimicrobial and antioxidant activities of anthrone and chromone from the latex of *Aloe harlana* Reynolds. Phytotherapy Research. 2011 Dec;25(12):1756-60. https://doi.org/10.1002/ptr.3482

Ascari J, de Oliveira MS, Nunes DS, Granato D, Scharf DR, Simionatto E, Otuki M, Soley B, Heiden G. Chemical composition, antioxidant and anti-inflammatory activities of the essential oils from male and female specimens of *Baccharis punctulata* (Asteraceae). Journal of ethnopharmacology. 2019 Apr 24;234:1-7.https://doi.org/10.1016/j.jep.2019.01.005

Asgary S, Keshvari M, Sahebkar A, Hashemi M, Rafieian-Kopaei M. Clinical investigation of the acute effects of pomegranate juice on blood pressure and endothelial function in hypertensive individuals. *ARYA Atheroscler*. 2013;9(6):326-331.

Asgary S, Naderi GH, Sadeghi M, Kelishadi R, Amiri M. Antihypertensive effect of Iranian *Crataegus curvisepala* Lind.: a randomized, double-blind study. Drugs under experimental and clinical research. 2004;30(5-6).

Asgary S, Naderi GH, Sarrafzadegan N, Mohammadifard N, Mostafavi S, Vakili R. Antihypertensive and antihyperlipidemic effects of *Achillea wilhelmsii*. Drugs under experimental and clinical research. 2000 Jan 1;26(3):89-94.

Asgary S, Rafieian-Kopaei M, Shamsi F, Najafi S, Sahebkar A. Biochemical and histopathological study of the anti-hyperglycemic and anti-hyperlipidemic effects of cornelian cherry (*Cornus mas* L.) in alloxan-induced diabetic rats. Journal of Complementary and Integrative Medicine. 2014 Jun 1;11(2):63-9. doi.org/10.1515/jcim-2013-0022

Ashraf H, Heidari R, Nejati V. Antihyperglycemic and Antihyperlipidemic Effects of Fruit Aqueous Extract of *Berberis integerrima* Bge. in Streptozotocin-induced Diabetic Rats. *Iran J Pharm Res*. 2014;13(4):1313–1318.

Asokan SM, Yang JY, Lin WT. Anti-hypertrophic and anti-apoptotic effects of short peptides of potato protein hydrolysate against hyperglycemic condition in cardiomyoblast cells. Biomedicine & Pharmacotherapy. 2018 Nov 1;107:1667-73. doi: 10.1016/j.biopha.2018.08.070.

Asong JA, Amoo SO, McGaw LJ, Nkadimeng SM, Aremu AO, Otang-Mbeng W. Antimicrobial activity, antioxidant potential, cytotoxicity and phytochemical profiling of four plants locally used against skin diseases. Plants. 2019 Sep;8(9):350. doi.org/10.3390/plants8090350

Asongalem EA, Foyet HS, Ngogang J, Folefoc GN, Dimo TH, Kamtchouing PI. Analgesic and antiinflammatory activities of *Erigeron floribundus*. Journal of ethnopharmacology. 2004b Apr 1;91(2-3):301-8. doi.org/10.1016/j.jep.2004.01.010

Asowata-Ayodele AM, Afolayan AJ, Otunola GA. Ethnobotanical survey of culinary herbs and spices used in the traditional medicinal system of Nkonkobe Municipality, Eastern Cape, South Africa. South African Journal of Botany. 2016 May 1;104:69-75. http://dx.doi.org/10.1016/j.sajb.2016.01.001

Asres K, Mazumder A, Bucar F. Antibacterial and antifungal activities of extracts of *Combretum molle*. Ethiopian medical journal. 2006 Jul 1;44(3):269.

Assaidi A, Legssyer A, Berrichi A, Aziz M, Mekhfi H, Bnouham M, Ziyyat A. Hypotensive property of *Chenopodium ambrosioides* in anesthetized normotensive rats. Journal of Complementary and Integrative Medicine. 2014 Feb 20;11(1):1-7. https://doi.org/10.1515/jcim-2013-0045

Assumpção CF, Bachiega P, Morzelle MC, Nelson DL, Ndiaye EA, Rios AD, Souza ÉC. Characterization, antioxidant potential and cytotoxic study of mangaba fruits. Ciência Rural. 2014 Jul;44(7):1297-303. http://dx.doi.org/10.1590/0103-8478cr20130855

Asuntha G, Prasannaraju Y, Sujatha D, Prasad KV. Assessment of effect of ethanolic extract of *Tephrosia purpurea* (L.) Pers., Fabaceae, activity on lithium-pilocarpine induced Status epilepticus and oxidative stress in Wistar rats. Revista Brasileira de Farmacognosia. 2010 Nov;20(5):767-72. https://doi.org/10.1590/S0102-695X2010005000025

Ateufack G, Mokam EC, Mbiantcha M, Feudjio RB, David N, Kamanyi A. Gastroprotective and ulcer healing effects of *Piptadeniastrum africanum* on experimentally induced gastric ulcers in rats. BMC complementary and alternative medicine. 2015 Dec 1;15(1):214. https://doi.org/10.1186/s12906-015-0713-5

Athiralakshmy TR, Divyamol AS, Nisha P. Phytochemical screening of *Saraca asoca* and antimicrobial activity against bacterial species. Asian Journal of Plant Science and Research. 2016;6(2):30-6.

Atta AH, Alkofahi A. Anti-nociceptive and anti-inflammatory effects of some Jordanian medicinal plant extracts. Journal of ethnopharmacology. 1998 Mar 1;60(2):117-24.https://doi.org/10.1016/S0378-8741(97)00137-2

Attrey DP, Singh AK, Naved T, Roy B. Effect of seabuckthorn extract on scopolamine induced cognitive impairment. Indian J Exp Biol. 2012 Oct;50(10):690-5.

Au DT, Wu J, Jiang Z, Chen H, Lu G, Zhao Z. Ethnobotanical study of medicinal plants used by Hakka in Guangdong, China. Journal of Ethnopharmacology. 2008 Apr 17;117(1):41-50. https://doi.org/10.1016/j.jep.2008.01.016

Avocèvou-Ayisso C, Avohou TH, Omorou M, Dessou G, Sinsin B. Ethnobotany of Pentadesma butyracea in Benin: A quantitative approach. Ethnobotany Research and Applications. 2012 Apr 16;10:151-66.

Avoseh ON, Ogunwande IA, Afolabi PO, Lawal OA, Thang TD, Ascrizzi R, Guido F Essential oil of *Cordia millenii* from Nigeria. American Journal of Essential Oils and Natural Products 2018; 6(4): 13-17.

Awaad AS, Alothman MR, Zain YM, Zain GM, Alqasoumi SI, Hassan DA. Comparative nutritional value and antimicrobial activities between three *Euphorbia* species growing in Saudi Arabia. Saudi pharmaceutical journal. 2017 Dec 1;25(8):1226-30. https://doi.org/10.1016/j.jsps.2017.09.007

Awad NE, Seida AA, Hamed MA, Elbatanony MM. Hypolipidaemic and antioxidant activities of *Ficus microcarpa* (L.) in hypercholesterolemic rats. Natural product research. 2011 Jul 1;25(12):1202-7. https://doi.org/10.1080/14786419.2010.538015

Awah FM, Uzoegwu PN, Ifeonu P. In vitro anti-HIV and immunomodulatory potentials of *Azadirachta indica* (Meliaceae) leaf extract. African Journal of Pharmacy and Pharmacology. 2011 Sep 22;5(11):1353-9. https://doi.org/10.5897/AJPP11.173

Awal MA, Ashrafuzzaman S, Haque EE. Studies on antibacterial activity and brine shrimp toxicity of leaf extract of *Cassia grandis*. Bangladesh Journal of Medical Microbiology. 2009 Jul 28;3(1):17-9. https://doi.org/10.3329/bjmm.v3i1.2966

Awang-Kanak F, Bakar MF, Mohamed M. Ethnobotanical survey on plants used as traditional salad food (ulam) in Kampung Taun Gusi, Kota Belud Sabah, Malaysia. InAIP Conference Proceedings 2018 Aug 15 (Vol. 2002, No. 1, p. 020024). AIP Publishing LLC. https://doi.org/10.1063/1.5050120

Ay E, Duran N. Investigation of the antiviral activity of *Ficus carica* L. latex against HSV-2. International Conference on Advanced Materials and Systems (ICAMS): The National Research & Development Institute for Textiles and Leather INCDTP; 2018. p. 33-7

Ayele Y, Kim JA, Park E, et al. A Methanol Extract of *Adansonia digitata* L. Leaves Inhibits Pro-Inflammatory iNOS Possibly via the Inhibition of NF-κB Activation. Biomol Ther (Seoul). 2013;21(2):146–152. doi:10.4062/biomolther.2012.098

Ayyanar M, Ignacimuthu S. Ethnobotanical survey of medicinal plants commonly used by Kani tribals in Tirunelveli hills of Western Ghats, India. Journal of ethnopharmacology. 2011 Apr 12;134(3):851-64. https://doi.org/10.1016/j.jep.2011.01.029

Azaz AD, Irtem HA, Kurkcuoğlu M, Baser KH. Composition and the in vitro antimicrobial activities of the essential oils of some *Thymus* species. Zeitschrift für Naturforschung C. 2004 Feb 1;59(1-2):75-80. https://doi.org/10.1515/znc-2004-1-216

Aziz S, Habib-ur-Rehman, Irshad M, Asghar SF, Hussain H, Ahmed I. Phytotoxic and antifungal activities of essential oils of *Thymus serpyllum* grown in the State of Jammu and Kashmir. Journal of Essential Oil Bearing Plants. 2010 Jan 1;13(2):224-9.

Aziz S, Rahman, H. Biological activities of *Prunus persica* L. batch. Journal of Medicinal Plants Research. 2013 Apr 17;7(15):947-51. doi: 10.5897/JMPR12.232

Baba H, Onanuga A. Preliminary phytochemical screening and antimicrobial evaluation of three medicinal plants used in Nigeria. African Journal of Traditional, Complementary and Alternative Medicines. 2011;8(4).

Babawale OP, Taiye FR, Adetunji OS. Ethnobotanical survey of plants used as memory enhancer in three states of Southwestern Nigeria. Journal of applied pharmaceutical science. 2016 Sep;6(09):209-14.

Babayi H, Kolo I, Okogun JI, Ijah UJ. The antimicrobial activities of methanolic extracts of *Eucalyptus camaldulensis* and *Terminalia catappa* against some pathogenic microorganisms. Biokemistri. 2004 16(2):106-111.

Babu NP, Pandikumar P, Ignacimuthu S. Anti-inflammatory activity of *Albizia lebbeck* Benth., an ethnomedicinal plant, in acute and chronic animal models of inflammation. Journal of ethnopharmacology. 2009 Sep 7;125(2):356-60. https://doi.org/10.1016/j.jep.2009.02.041

Babu NP, Saravanan S, Pandikumar P, Bala Krishna K, Karunai Raj M, Ignacimuthu S. Anti-inflammatory and anti-arthritic effects of 3-hydroxy, 2-methoxy sodium butanoate from the leaves of *Clerodendrum phlomidis* L.f. Inflamm Res. 2014 Feb;63(2):127-38. doi: 10.1007/s00011-013-0681-5.

Baburao B, Rajyalakshmi G, Venkatesham A, Kiran G, Shyamsunder A, Gangarao B. Anti-inflammatory and antimicrobial activities of methanolic extract of *Tribulus terrestris* Linn plant. Int J Chem Sci. 2009 Jan;7(3):1867-72.

Baburao B, Reddy AR, Kiran G, Reddy YN, Mohan GK. Antioxidant, analgesic and anti-inflammatory activities of *Leucas cephalotes* (Roxb. ex Roth) Spreng. Brazilian Journal of Pharmaceutical Sciences. 2010 Sep;46(3):525-9. doi.org/10.1590/S1984-82502010000300016

Backhouse N, Delporte C, Negrete R, Feliciano SS, López-Pérez JL. Bioactive phenolic derivatives from *Acaena splendens* methanol extract. Phytotherapy Research: An International Journal Devoted to Pharmacological and Toxicological Evaluation of Natural Product Derivatives. 2002 Sep;16(6):562-6.

Backhouse N, Delporte C, Negrete R, Salinas P, Pinto A, Aravena S, Cassels BK. Antiinflammatory and Antipyretic Activities of *Cuscuta chilensis*, *Cestrum parqui*, and *Psoralea glandulosa*. International journal of pharmacognosy. 1996 Jan 1;34(1):53-7. https://doi.org/10.1076/phbi.34.1.53.13176

Bader A, Giner RM, Martini F, Schinella GR, Ríos JL, Braca A, Prieto JM. Modulation of COX, LOX and NFκB activities by *Xanthium spinosum* L. root extract and ziniolide. Fitoterapia. 2013 Dec 1;91:284-9.

Badilla B, Mora G, Lapa AJ, Silva Emim JA. Anti-inflammatory activity of *Urera baccifera* (Urticaceae) in Sprague-Dawley rats. Revista de biologia tropical. 1999b Sep;47(3):365-71.

Badilla B, Mora G, Poveda LJ. Anti-inflammatory activity of aqueous extracts of five Costa Rican medicinal plants in Sprague-Dawley rats. Revista de biologia tropical. 1999a Dec;47(4):723-7.

Bae N, Chung S, Kim HJ, Cha JW, Oh H, Gu MY, Oh MS, Yang HO. Neuroprotective effect of modified Chungsimyeolda-tang, a traditional Korean herbal formula, via autophagy induction in models of Parkinson′ s disease. Journal of ethnopharmacology. 2015 Jan 15;159:93-101.

Baek HK, Kim PS, Song JA, Choi DH, Kim DE, Oh SI, Park SK, Kim SJ, Song KD, Hwang IK, Seo HS. Neuronal maturation in the hippocampal dentate gyrus via chronic oral administration of *Artemisa annua* extract is independent of cyclooxygenase 2 signaling pathway in diet-induced obesity mouse model. Journal of veterinary science. 2017 Jun;18(2):119. https://doi.org/10.4142/jvs.2017.18.2.119

Baek J, Jeong H, Ham Y, Jo YH, Choi M, Kang M, Son B, Choi S, Ryu HW, Kim J, Shen H. Improvement of spinal muscular atrophy via correction of the SMN2 splicing defect by *Brucea javanica* (L.) Merr. extract and Bruceine D. Phytomedicine. 2019 Dec 1;65:153089. doi: 10.1016/j.phymed.2019.153089.

Bahaeddin Z, Yans A, Khodagholi F, Hajimehdipoor H, Sahranavard S. Hazelnut and neuroprotection: Improved memory and hindered anxiety in response to intra-hippocampal Aβ injection. Nutritional neuroscience. 2017 Jul 3;20(6):317-26. https://doi.org/10.1080/1028415X.2015.1126954

Bai N, He K, Roller M, Lai CS, Shao X, Pan MH, Bily A, Ho CT. Flavonoid glycosides from *Microtea debilis* and their cytotoxic and anti-inflammatory effects. Fitoterapia. 2011 Mar 1;82(2):168-72.

Baiges I, Arola L. COCOA (*Theobroma cacao*) Polyphenol-Rich Extract Increases the Chronological Lifespan of Saccharomyces cerevisiae. The Journal of frailty & aging. 2016 Jan 1;5(3):186-90.

Bairy KL, Rao Y, Kumar Das S, Kumar KB. Efficacy of *Tinospora cordifolia* on learning and memory in healthy volunteers: A double-blind, randomized, placebo controlled study. Iranian Journal of Pharmacology and Therapeutics. 2004 Nov 15;3(2):57-0.

Bais S, Abrol N, Prashar Y. Modulatory effect of standardised amentoflavone isolated from J*uniperus communis* L. agianst Freund's adjuvant induced arthritis in rats (histopathological and X Ray anaysis). Biomedicine & Pharmacotherapy. 2017 Feb 1;86:381-92. https://doi.org/10.1016/j.biopha.2016.12.027

Bais S, Prashar Y. Identification and characterization of amentoflavone from six species of Juniperus against H 2 O 2 induced oxidative damage in human erythrocytes and leucocytes. Res. J. Phytochem. 2015;9(2):41-55.

Bakar A, Fadzelly M, Ismail NA, Isha A, Ling M, Lee A. Phytochemical composition and biological activities of selected wild berries (*Rubus moluccanus* L., *R. fraxinifolius* Poir., and *R. alpestris* Blume). Evidence-Based Complementary and Alternative Medicine. 2016;2016. https://doi.org/10.1155/2016/2482930

Bakht J, Ali H, Khan MA, Khan A, Saeed M, Shafi M, Islam A, Tayyab M. Antimicrobial activities of different solvents extracted samples of *Linum usitatissimum* by disc diffusion method. African Journal of Biotechnology. 2011;10(85):19825-35. http://dx.doi.org/10.5897/AJB11.229

Bakre AG, Aderibigbe AO, Ademowo OG. Studies on neuropharmacological profile of ethanol extract of *Moringa oleifera* leaves in mice. Journal of Ethnopharmacology. 2013 Oct 7;149(3):783-9. https://doi.org/10.1016/j.jep.2013.08.006

Balachandran C, Duraipandiyan V, Al-Dhabi NA, Balakrishna K, Kalia NP, Rajput VS, Khan IA, Ignacimuthu S. Antimicrobial and Antimycobacterial Activities of Methyl Caffeate Isolated from *Solanum torvum* Swartz. Fruit. Indian J Microbiol. 2012 Dec;52(4):676-81. doi: 10.1007/s12088-012-0313-8.

Bandeira PN, Fonseca AM, Costa SM, Lins MU, Pessoa OD, Monte FJ, Nogueira NA, Lemos TL. Antimicrobial and antioxidant activities of the essential oil of resin of *Protium heptaphyllum*. Natural Product Communications. 2006 Feb;1(2):1934578X0600100207.

Banik S, Ibrahim M, Amin MN, Moghal MM, Majumder MS, Alam MK, Anonna SN, Rashed MS. Determination of biological properties of *Alocasia macrorrhizos*: A medicinal plant. World Journal of Pharmaceutical Research. 2014 Sep 9;3(9):193-210.

Bano A, Ahmad M, Hadda TB, Saboor A, Sultana S, Zafar M, Khan MP, Arshad M, Ashraf MA. Quantitative ethnomedicinal study of plants used in the skardu valley at high altitude of Karakoram-Himalayan range, Pakistan. Journal of ethnobiology and ethnomedicine. 2014 Dec;10(1):43.

Bansal P, Paul P, Nayak PG, Pannakal ST, Zou JH, Laatsch H, Priyadarsini KI, Unnikrishnan MK. Phenolic compounds isolated from *Pilea microphylla* prevent radiation-induced cellular DNA damage. Acta Pharmaceutica Sinica B. 2011 Dec 1;1(4):226-35. https://doi.org/10.1016/j.apsb.2011.10.006

Banso A. Phytochemical and antibacterial investigation of bark extracts of *Acacia nilotica*. Journal of medicinal plants research. 2009 Feb 28;3(2):082-5.

Barati Boldaji R, Akhlaghi M, Sagheb MM, Esmaeilinezhad Z. Pomegranate juice improves cardiometabolic risk factors, biomarkers of oxidative stress and inflammation in hemodialysis patients: A randomized crossover trial. Journal of the Science of Food and Agriculture. 2020 Jan 30;100(2):846-54.

Barberena I, Calderon A, Solís PN, Correa M, Risco E, Canigueral S, Alvarez E, Fernández T, Hajos S, Gupta MP. Screening of anticancer and immunomodulatory activities of Panamanian plants. Pharmaceutical biology. 2004 Jan 1;42(7):552-8. https://doi.org/10.3109/13880200490901230

Barbosa D, Nascimento MV, Lino RC, Magalhães MR, Florentino IF, Honório TC, Galdino PM, Bara MT, Paula JR, Costa EA. Mechanism involved in the anti-inflammatory effect of *Spiranthera odoratissima* (Manacá). Revista Brasileira de Farmacognosia. 2012 Feb;22(1):137-43. https://doi.org/10.1590/S0102-695X2011005000154

Barbosa-Filho JM, Lima SA, Camorim EL, de Sena KX, Almeida JR, da-Cunha VL, Silva MS, Agra MD, Braz-Filho R. Botanical study, phytochemistry and antimicrobial activity of *Tabebuia aurea*:(with 1 table & 1 figure). Phyton (Buenos Aires). 2004 Dec;73:221-8.

Bardaa S, Halima NB, Aloui F, Mansour RB, Jabeur H, Bouaziz M, Sahnoun Z. Oil from pumpkin (*Cucurbita pepo* L.) seeds: evaluation of its functional properties on wound healing in rats. Lipids in health and disease. 2016 Dec;15(1):1-2. ttps://doi.org/10.1186/s12944-016-0237-0

Barton DL, Liu H, Dakhil SR, Linquist B, Sloan JA, Nichols CR, McGinn TW, Stella PJ, Seeger GR, Sood A, Loprinzi CL. Wisconsin Ginseng (*Panax quinquefolius*) to improve cancer-related fatigue: a randomized, double-blind trial, N07C2. Journal of the National Cancer Institute. 2013 Aug 21;105(16):1230-8. https://doi.org/10.1093/jnci/djt181

Basak J, Majsterek I. miRNA-Dependent CD4+ T Cell Differentiation in the Pathogenesis of Multiple Sclerosis. Multiple Sclerosis International. 2021 Jan 8;2021. https://doi.org/10.1155/2021/8825588

Basar S, Uhlenhut K, Högger P, Schöne F, Westendorf J. Analgesic and antiinflammatory activity of *Morinda citrifolia* L.(Noni) fruit. Phytotherapy Research: An International Journal Devoted to Pharmacological and Toxicological Evaluation of Natural Product Derivatives. 2010 Jan;24(1):38-42. doi.org/10.1002/ptr.2863

Başbülbül G, Özmen A, Biyik HH, Şen Ö. Antimitotic and antibacterial effects of the *Primula veris* L. flower extracts. Caryologia. 2008 Jan 1;61(1):88-91. https://doi.org/10.1080/00087114.2008.10589614

Basile A, Sorbo S, Giordano S, Ricciardi L, Ferrara S, Montesano D, Cobianchi RC, Vuotto ML, Ferrara L. Antibacterial and allelopathic activity of extract from *Castanea sativa* leaves. Fitoterapia. 2000 Aug 1;71:S110-6. https://doi.org/10.1016/S0367-326X(00)00185-4

Baskaran P, Kumari A, Ncube B, Van Staden J. Acetylcholinesterase-Inhibition and Antibacterial Activity of *Mondia whitei* Adventitious Roots and *Ex vitro*-Grown Somatic Embryogenic-Biomass. Front Pharmacol. 2016 Oct 3;7:335. doi: 10.3389/fphar.2016.00335.

Battistini R, Rossini I, Ercolini C, Goria M, Callipo MR, Maurella C, Pavoni E, Serracca L. Antiviral activity of essential oils against hepatitis A virus in soft fruits. Food and environmental virology. 2019 Mar;11(1):90-5. https://doi.org/10.1007/s12560-019-09367-3

Battu GR, Parimi R, Chandra Shekar KB. In vivo and in vitro pharmacological activity of *Aristolochia tagala* (syn: Aristolochia acuminata) root extracts. Pharmaceutical biology. 2011 Nov 1;49(11):1210-4.

Baum L, Ng A. Curcumin interaction with copper and iron suggests one possible mechanism of action in Alzheimer's disease animal models. Journal of Alzheimer's disease. 2004 Jan 1;6(4):367-77. doi: 10.3233/JAD-2004-6403

Baumgartner L, Sosa S, Atanasov AG, Bodensieck A, Fakhrudin N, Bauer J, Favero GD, Ponti C, Heiss EH, Schwaiger S, Ladurner A. Lignan derivatives from *Krameria lappacea* roots inhibit acute inflammation in vivo and pro-inflammatory mediators in vitro. Journal of natural products. 2011 Jul 29;74(8):1779-86. doi.org/10.1021/np200343t

Bawankule DU, Chattopadhyay SK, Pal A, Saxena K, Yadav S, Faridi U, Darokar MP, Gupta AK, Khanuja SP. Modulation of inflammatory mediators by coumarinolignoids from *Cleome viscosa* in female swiss albino mice. Inflammopharmacology. 2008 Dec 1;16(6):272-7. https://doi.org/10.1007/s10787-008-8012-0

Baydar H, Sağdiç O, Özkan G, Karadoğan T. Antibacterial activity and composition of essential oils from *Origanum*, *Thymbra* and *Satureja* species with commercial importance in Turkey. Food control. 2004 Apr 1;15(3):169-72. doi.org/10.1016/S0956-7135(03)00028-8

Bayoub K, Baibai T, Mountassif D, Retmane A, Soukri A. Antibacterial activities of the crude ethanol extracts of medicinal plants against *Listeria monocytogenes* and some other pathogenic strains. African Journal of Biotechnology. 2010;9(27):4251-8.

Bazzaz BS, Haririzadeh G. Screening of Iranian plants for antimicrobial activity. Pharmaceutical Biology. 2003 Jan 1;41(8):573-83.

Beck NR, Namdeo KP. Evaluation of in-vivo anti-inflammatory activity of leaves Extract of *Carissa spinarum* on formalin induced paw edema in albino rats. UK J Pharm Biosci. 2016;4:36-40.

Bedoya LM, Sanchez-Palomino S, Abad MJ, Bermejo P, Alcami J. Anti-HIV activity of medicinal plant extracts. Journal of Ethnopharmacology. 2001 Sep 1;77(1):113-6.

Begossi A, Hanazaki N, Tamashiro JY. Medicinal plants in the Atlantic Forest (Brazil): knowledge, use, and conservation. Human ecology. 2002 Sep 1;30(3):281-99.

Begum R, Sharma M, Pillai KK, Aeri V, Sheliya MA. Inhibitory effect of *Careya arborea* on inflammatory biomarkers in carrageenan-induced inflammation. Pharmaceutical biology. 2015 Mar 4;53(3):437-45. doi.org/10.3109/13880209.2014.923005

Belayneh A, Bussa NF. Ethnomedicinal plants used to treat human ailments in the prehistoric place of Harla and Dengego valleys, eastern Ethiopia. Journal of ethnobiology and ethnomedicine. 2014 Dec;10(1):18.

Belhouchet Z, Sautour M, Miyamoto T, Lacaille-Dubois MA. Steroidal Saponins from the Roots of *Smilax aspera* subsp. mauritanica. Chemical and Pharmaceutical Bulletin. 2008 Sep 1;56(9):1324-7. https://doi.org/10.1248/cpb.56.1324

Belmouhoub M, Chebout I, Iguer-Ouada M. Antidiabetic and anti-hypercholesterolemic effects of flavonoid-rich fractions of *Rosmarinus officinalis* in streptozotocin-induced diabetes in mice. Phytothérapie. 2018 Aug 1;16(4):204-10. https://doi.org/10.3166/phyto-2018-0054

Ben Nasr S, Aazza S, Mnif W, Miguel M. In-vitro antioxidant and anti-inflamatory activities of *Pituranthos chloranthus* and *Artemisia vulgaris* from Tunisia. International Journal of Applied Pharmaceutical Sciences and Research. 2020;11(2):605-14. doi: 10.13040/IJPSR.0975-8232.11(2).605-14

Bendjeddou D, Lalaoui K, Satta D. Immunostimulating activity of the hot water-soluble polysaccharide extracts of *Anacyclus pyrethrum*, *Alpinia galanga* and *Citrullus colocynthis*. Journal of ethnopharmacology. 2003 Oct 1;88(2-3):155-60. https://doi.org/10.1016/S0378-8741(03)00226-5

Benevides Bahiense J, Marques FM, Figueira MM, et al. Potential anti-inflammatory, antioxidant and antimicrobial activities of *Sambucus australis*. Pharm Biol. 2017;55(1):991–997. doi:10.1080/13880209.2017.1285324

Benitez NP, Meléndez León EM, Stashenko EE. Essential oil composition from two species of Piperaceae family grown in Colombia. Journal of chromatographic science. 2009 Oct 1;47(9):804-7. https://doi.org/10.1093/chromsci/47.9.804

Bensalem J, Dudonné S, Etchamendy N, Pellay H, Amadieu C, Gaudout D, Dubreuil S, Paradis ME, Pomerleau S, Capuron L, Hudon C. Polyphenols from grape and blueberry improve episodic memory in healthy elderly with lower level of memory performance: A bicentric double-blind, randomized, placebo-controlled clinical study. The Journals of Gerontology: Series A. 2018 Jul 19.

Benson KF, Beaman JL, Ou B, Okubena A, Okubena O, Jensen GS. West African *Sorghum bicolor* leaf sheaths have anti-inflammatory and immune-modulating properties in vitro. Journal of medicinal food. 2013 Mar 1;16(3):230-8. doi.org/10.1089/jmf.2012.0214

Benvenuti S, Pellati F, Melegari MA, Bertelli D. Polyphenols, anthocyanins, ascorbic acid, and radical scavenging activity of *Rubus, Ribes,* and *Aronia*. Journal of Food Science. 2004 Apr;69(3):FCT164-9. doi.org/10.1111/j.1365-2621.2004.tb13352.x

Beppe GJ, Dongmo AB, Foyet HS, Tsabang N, Olteanu Z, Cioanca O, Hancianu M, Dimo T, Hritcu L. Memory-enhancing activities of the aqueous extract of *Albizia adianthifolia* leaves in the 6-hydroxydopamine-lesion rodent model of Parkinson's disease. BMC complementary and alternative medicine. 2014 Dec 1;14(1):142. https://doi.org/10.1186/1472-6882-14-142

Berenguer B, Trabadela C, Sánchez-Fidalgo S, Quílez A, Mino P, De la Puerta R, Martín-Calero MJ. The aerial parts of *Guazuma ulmifolia* Lam. protect against NSAID-induced gastric lesions. Journal of ethnopharmacology. 2007 Nov 1;114(2):153-60. https://doi.org/10.1016/j.jep.2007.07.019

Bernardes MJ, de Carvalho FS, Silveira LL, de Paula JR, Bara MT, Garrote CF, Pedrino GR, Rocha ML. Hypotensive effect of *Aspidosperma subincanum* Mart. in rats and its mechanism of vasorelaxation in isolated arteries. Journal of ethnopharmacology. 2013 Jan 9;145(1):227-32. https://doi.org/10.1016/j.jep.2012.10.057

Bessong PO, Rojas LB, Obi LC, Tshisikawe PM, Igunbor EO. Further screening of Venda medicinal plants for activity against HIV type 1 reverse transcriptase and integrase. African Journal of Biotechnology. 2006;5(6):526-8.

Betti JL, Yongo OD, Mbomio DO, Iponga DM, Ngoye A. An ethnobotanical and floristical study of medicinal plants among the Baka Pygmies in the periphery of the Ipassa-Biosphere reserve, Gabon. European Journal of Medicinal Plants. 2013 Mar 2:174-205. https://doi.org/10.9734/EJMP/2013/2550

Beuscher N, Bodinet C, Neumann-Haefelin D, Marston A, Hostettmann K. Antiviral activity of African medicinal plants. Journal of ethnopharmacology. 1994 Apr 1;42(2):101-9. https://doi.org/10.1016/0378-8741(94)90103-1

Bezerra GP, da Silva Góis RW, de Brito TS, de Lima FJ, Bandeira MA, Romero NR, Magalhães PJ, Santiago GM. Phytochemical study guided by the myorelaxant activity of the crude extract, fractions and constituent from stem bark of *Hymenaea courbaril* L. Journal of ethnopharmacology. 2013 Aug 26;149(1):62-9. https://doi.org/10.1016/j.jep.2013.05.052

Bezerra JJ, do Nascimento TG, Kamiya RU, do Nascimento Prata AP, de Medeiros PM, da Silva SA, de Mendonça CN. Phytochemical screening, chromatographic profile and evaluation of antimicrobial and antioxidant activities of three species of the Cyperaceae Juss. Family. Journal of Medicinal Plants Research 2019 July: Vol. 13(14), pp. 312-320. doi: 10.5897/JMPR2019.6796

Bhadoriya S, Mishra V, Raut S, Ganeshpurkar A, JAIN S. Anti-inflammatory and antinociceptive activities of a hydroethanolic extract of *Tamarindus indica* leaves. Scientia pharmaceutica. 2012 Apr 1;80(3):685-700.

Bhagat J, Lobo R, Parmar V, Ballal M. In vitro Free Radical Scavenging Potential of Indian Habitant *Anisochilus carnosus* (LF) Wall. Chinese journal of natural medicines. 2011 Nov 1;9(6):456-60. https://doi.org/10.3724/SP.J.1009.2011.00456

Bhalodia NR, Shukla VJ. Antibacterial and antifungal activities from leaf extracts of *Cassia fistula* l.: An ethnomedicinal plant. J Adv Pharm Technol Res. 2011 Apr;2(2):104-9. doi: 10.4103/2231-4040.82956.

Bharani A, Ganguly A, Bhargava KD. Salutary effect of *Terminalia arjuna* in patients with severe refractory heart failure. International Journal of Cardiology. 1995 May 1;49(3):191-9. https://doi.org/10.1016/0167-5273(95)02320-V

Bharat M, Verma DK, Shanbhag V, Rajput RS, Nayak D, Amuthan A. Ethanolic extract of oral *Areca catechu* promotes burn wound healing in rats. Int J Pharm Sci Rev Res. 2014 Jan 1;25(2):145-8.

Bhatnagar M, Meena P, Barbar S, Joshi C. Neuroprotective response of the hippocampus region of the brain to *Withania somnifera* and *Asparagus racemosus* root extract: An in vitro study. J Med Plants Res. 2013 Oct;7(13):2259-64.

Bhattacharjee I, Chatterjee SK, Chatterjee S, Chandra G. Antibacterial potentiality of Argemone mexicana solvent extracts against some pathogenic bacteria. Memórias do Instituto Oswaldo Cruz. 2006 Sep;101(6):645-8.

Bhattacharya S, Pal S, Chaudhuri AN. Pharmacological studies of the antiinflammatory profile of *Mikania cordata* (Burm) BL robinson root extract in rodents. Phytotherapy Research. 1992 Sep;6(5):255-60. https://doi.org/10.1002/ptr.2650060507

Bhattarai S, Chaudhary RP, Taylor RS. Ethnomedicinal plants used by the people of Manang district, central Nepal. Journal of Ethnobiology and Ethnomedicine. 2006;2:41. doi:10.1186/1746-4269-2-41.

Bhogaonkar PY, Dagawal MJ, Ghorpade DS. Pharmacognostic studies and antimicrobial activity of *Synedrella nodiflora* (L.) Gaertn. Bioscience Discovery. 2011;2(3):317-21.

Bhogayata K, Sharma PP, Patel BR. A clinical evaluation of Saptaparna (*Alstonia scholaris* L., R. Br.) on essential hypertension. AYU (An international quarterly journal of research in Ayurveda). 2009 Jul 1;30(3):318.

Bhujade AM, Talmale S, Kumar N, Gupta G, Reddanna P, Das SK, Patil MB. Evaluation of *Cissus quadrangularis* extracts as an inhibitor of COX, 5-LOX, and proinflammatory mediators. Journal of ethnopharmacology. 2012 Jun 14;141(3):989-96. https://doi.org/10.1016/j.jep.2012.03.044

Bhujbal SS, Chitlange SS, Suralkar A, Shinde DB, Patil MJ. Anti-inflammatory activity of an isolated flavonoid fraction from *Celosia argentea* Linn. Journal of medicinal plants research. 2008 Mar 1;2(3):052-4.

Bhuvanendran S, Kumari Y, Othman I, Shaikh MF. Amelioration of Cognitive Deficit by Embelin in a Scopolamine-Induced Alzheimer's Disease-Like Condition in a Rat Model. *Front Pharmacol*. 2018;9:665. Published 2018 Jun 25. doi:10.3389/fphar.2018.00665

Biavatti MW, Farias C, Curtius F, Brasil LM, Hort S, Schuster L, Leite SN, Prado SR. Preliminary studies on *Campomanesia xanthocarpa* (Berg.) and Cuphea *carthagenensis* (Jacq.) JF Macbr. aqueous extract: weight control and biochemical parameters. Journal of Ethnopharmacology. 2004 Aug 1;93(2-3):385-9.

Biber K, Owens T, Boddeke E. What is microglia neurotoxicity (Not)?. Glia. 2014 Jun;62(6):841-54. https://doi.org/10.1002/glia.22654

Bibi Y, Nisa S, Chaudhary FM, Zia M. Antibacterial activity of some selected medicinal plants of Pakistan. BMC complementary and alternative medicine. 2011 Dec 1;11(1):52. https://doi.org/10.1186/1472-6882-11-52

Bieschke J, Russ J, Friedrich RP, Ehrnhoefer DE, Wobst H, Neugebauer K, Wanker EE. EGCG remodels mature α-synuclein and amyloid-β fibrils and reduces cellular toxicity. Proceedings of the National Academy of Sciences. 2010 Apr 27;107(17):7710-5.

Bihaqi SW, Singh AP, Tiwari M. In vivo investigation of the neuroprotective property of *Convolvulus pluricaulis* in scopolamine-induced cognitive impairments in Wistar rats. Indian J Pharmacol. 2011;43(5):520–525. doi:10.4103/0253-7613.84958

Bihaqi SW, Singh AP, Tiwari M. Supplementation of *Convolvulus pluricaulis* attenuates scopolamine-induced increased tau and amyloid precursor protein (AβPP) expression in rat brain. Indian J Pharmacol. 2012 Sep-Oct;44(5):593-8. doi: 10.4103/0253-7613.100383.

Bijesh K, Sebastian D. Isolation and characterization of antibacterial compounds from *Macaranga peltata* against clinical isolates of Staphylococcus aureus. Int. J. Biol. Pharma Res. 2013;4:1196-203.

Billah MM, Hasan MR, Nawrin K, Mohiuddin, Habib MR. Evaluation of analgesic and sedative-anxiolytic potential of *Paederia foetida* leaf extract. Am J Biomed Sci. 2015;7(2):98-104.

Bing FH, Liu J, Li Z, Zhang GB, Liao YF, Li J, Dong CY. Anti-influenza-virus activity of total alkaloids from *Commelina communis* L. Archives of virology. 2009 Nov 1;154(11):1837. doi: 10.1007/s00705-009-0503-9

Binny K, Kumar SG, Dennis T. Anti-inflammatory and antipyretic properties of the rhizome of *Costus speciosus* (koen.) sm. *J Basic Clin Pharm*. 2010;1(3):177–181.

Bipat R, Toelsie J, Joemmanbaks R, Gummels J, Klaverweide J, Jhanjan N, Orie S, Ramjiawan K, BrusseL A, Soekhoe R, Mans D. Effects of plants popularly used against hypertension on norepinephrine-stimulated guinea pig atria. Pharmacognosy Magazine. 2008;4(13):12.

Bishayi B, Roychowdhury S, Ghosh S, Sengupta M. Hepatoprotective and immunomodulatory properties of *Tinospora cordifolia* in CCl4 intoxicated mature albino rats. The Journal of toxicological sciences. 2002;27(3):139-46. https://doi.org/10.2131/jts.27.139

Bisht DS, Padalia RC, Singh L, Pande V, Lal P, Mathela CS. Constituents and antimicrobial activity of the essential oils of six Himalayan *Nepeta* species. Journal of the Serbian Chemical Society. 2010;75(6):739-47. https://doi.org/10.2298/JSC091106052B

Biswas M, Biswas K, Karan TK, Bhattacharya S, Ghosh AK, Haldar PK. Evaluation of analgesic and anti-inflammatory activities of *Terminalia arjuna* leaf. Journal of Phytology. 2011 Jan 19; 3(1): 33-38.

Biswas MK, Mridha SA, Rashid MA, Sharmin T. Membrane stabilizing and antimicrobial activities of *Caladium bicolor* and *Chenopodium album.* IOSR J. Pharm. Biol. Sci. 2013;6:62-5.

Biswas NN, Saha S, Ali MK. Antioxidant, antimicrobial, cytotoxic and analgesic activities of ethanolic extract of *Mentha arvensis* L. Asian Pacific Journal of Tropical Biomedicine. 2014 Oct 1;4(10):792-7. https://doi.org/10.12980/APJTB.4.2014C1298

Blagojević P, Radulović N, Palić R, Stojanović G. Chemical composition of the essential oils of Serbian wild-growing *Artemisia absinthium* and *Artemisia vulgaris*. Journal of agricultural and food chemistry. 2006 Jun 28;54(13):4780-9.

Blanco MM, Costa CA, Freire AO, Santos Jr JG, Costa M. Neurobehavioral effect of essential oil of *Cymbopogon citratus* in mice. Phytomedicine. 2009 Mar 1;16(2-3):265-70. https://doi.org/10.1016/j.phymed.2007.04.007

Bnouham M, Merhfour FZ, Elachoui M, Legssyer A, Mekhfi H, Lamnaouer D, Ziyyat A. Toxic effects of some medicinal plants used in Moroccan traditional medicine. Moroccan Journal of Biology. 2006;2(3):21-30.

Bobby MN, Wesely EG. In vitro anti–bacterial activity of leaves extracts of *Albizia lebbeck* Benth against some selected pathogens. Asian Pacific Journal of Tropical Biomedicine. 2012 Feb 1;2(2):S859-62. https://doi.org/10.1016/S2221-1691(12)60324-4

Boff L, Silva IT, Argenta DF, Farias LM, Alvarenga LF, Pádua RM, Braga FC, Leite JP, Kratz JM, Simões CM. *Strychnos pseudoquina* A. St. Hil.: a Brazilian medicinal plant with promising in vitro antiherpes activity. Journal of applied microbiology. 2016 Dec;121(6):1519-29. https://doi.org/10.1111/jam.13279

Bogavac-Stanojevic N, Kotur Stevuljevic J, Cerne D, Zupan J, Marc J, Vujic Z, Crevar-Sakac M, Sopic M, Munjas J, Radenkovic M, Jelic-Ivanovic Z. The role of artichoke leaf tincture (*Cynara scolymus*) in the suppression of DNA damage and atherosclerosis in rats fed an atherogenic diet. Pharmaceutical biology. 2018 Jan 1;56(1):138-44.

Bohlooli S, Mohebipoor A, Mohammadi S, Kouhnavard M, Pashapoor S. Comparative study of fig tree efficacy in the treatment of common warts (*Verruca vulgaris*) vs. cryotherapy. International journal of dermatology. 2007 May;46(5):524-6. https://doi.org/10.1111/j.1365-4632.2007.03159.x

Boland B, Yu WH, Corti O, Mollereau B, Henriques A, Bezard E, Pastores GM, Rubinsztein DC, Nixon RA, Duchen MR, Mallucci GR. Promoting the clearance of neurotoxic proteins in neurodegenerative disorders of ageing. Nature reviews Drug discovery. 2018 Sep;17(9):660-88. https://doi.org/10.1038/nrd.2018.109

Bomfim GH, Musial DC, Miranda-Ferreira R, Nascimento SR, Jurkiewicz A, Jurkiewicz NH, de Moura RS. Antihypertensive effects of the *Vitis vinifera* grape skin (ACH09) extract consumption elicited by functional improvement of

P1 (A1) and P2 (P2X1) purinergic receptors in diabetic and hypertensive rats. PharmaNutrition. 2019 Jun 1;8:100146. https://doi.org/10.1016/j.phanu.2019.100146

Bondonno NP, Bondonno CP, Blekkenhorst LC, Considine MJ, Maghzal G, Stocker R, Woodman RJ, Ward NC, Hodgson JM, Croft KD. Flavonoid‐Rich Apple Improves Endothelial Function in Individuals at Risk for Cardiovascular Disease: A Randomized Controlled Clinical Trial. Molecular nutrition & food research. 2018 Feb;62(3):1700674. https://doi.org/10.1002/mnfr.201700674

Bonet MÀ, Parada M, Selga A, Valles J. Studies on pharmaceutical ethnobotany in the regions of L'Alt Emporda and Les Guilleries (Catalonia, Iberian Peninsula). Journal of Ethnopharmacology. 1999 Dec 15;68(1-3):145-68.

Bontempo P, Carafa V, Grassi R, Basile A, Tenore GC, Formisano C, Rigano D, Altucci L. Antioxidant, antimicrobial and anti-proliferative activities of *Solanum tuberosum* L. var. Vitelotte. Food and Chemical Toxicology. 2013 May 1;55:304-12. https://doi.org/10.1016/j.fct.2012.12.048

Boominathan M, Ramamurthy V. Antimicrobial activity of *Heliotropium indicum* and *Coldenia procumbens.* Journal of Ecobiology. 2009;24(1):11-5.

Boonyagul S, Banlunara W, Sangvanich P, Thunyakitpisal P. Effect of acemannan, an extracted polysaccharide from *Aloe vera*, on BMSCs proliferation, differentiation, extracellular matrix synthesis, mineralization, and bone formation in a tooth extraction model. Odontology. 2014 Jul 1;102(2):310-7.

Boopathi T, Gopalasatheeskumar K, Parthiban S, Sangeetha G, Thanga Kokila M, Manimaran T. Evaluation of Antimicrobial Activity of *Tecoma stans* and *Muntingia calabura*. World Journal of Pharmaceutical Research. 2017;6(3):617-27.

Bopda OS, Longo F, Bella TN, Edzah PM, Taïwe GS, Bilanda DC, Tom EN, Kamtchouing P, Dimo T. Antihypertensive activities of the aqueous extract of *Kalanchoe pinnata* (Crassulaceae) in high salt-loaded rats. Journal of ethnopharmacology. 2014 Apr 28;153(2):400-7. https://doi.org/10.1016/j.jep.2014.02.041

Borchardt JR, Wyse DL, Sheaffer CC, Kauppi KL, Fulcher RG, Ehlke NJ, Biesboer DD, Bey RF. Antioxidant and antimicrobial activity of seed from plants of the Mississippi river basin. Journal of Medicinal Plants Research. 2008 Apr 30;2(4):081-93. https://doi.org/10.5897/JMPR.9000210

Borges JD, Perim MC, de Castro RO, Araújo TA, Peixoto Sobrinho TJ, da Silva AC, Mariano SM, Carreiro SC, Pranchevicius MC. Evaluation of antibacterial activity of the bark and leaf extracts of *Brosimum gaudichaudii* Trecul against multidrug resistant strains. Natural product research. 2017 Dec 17;31(24):2931-5. https://doi.org/10.1080/14786419.2017.1305379

Borneo R, León AE, Aguirre A, Ribotta P, Cantero JJ. Antioxidant capacity of medicinal plants from the Province of Córdoba (Argentina) and their in vitro testing in a model food system. Food Chemistry. 2009 Feb 1;112(3):664-70.

Borokini TI, Omotayo FO. Phytochemical and ethnobotanical study of some selected medicinal plants from Nigeria. Journal of Medicinal Plants Research. 2012 Feb 23;6(7):1106-18. https://doi.org/10.5897/JMPR09.430

Borowiec K, Matysek M, Szwajgier D, Biała G, Kruk-Słomka M, Szalak R, Ziętek J, Arciszewski MB, Targoński Z. The influence of bilberry fruit on memory and the expression of parvalbumin in the rat hippocampus. Polish Journal of Veterinary Sciences. 2019:481-7. doi:  10.24425/pjvs.2019.129973

Borroto J, Salazar R, Pérez A, Quiros Y, Hernandez M, Waksman N, Trujillo R. Antimicrobial activity of the dichloromethane extract from in vitro cultured roots of *Morinda royoc* and its main constituents. Natural product communications. 2010 May;5(5):1934578X1000500526. https://doi.org/10.1177/1934578X1000500526

Bot YS, Mgbojikwe LO, Nwosu C, Abimiku A, Dadik J, Damshak D. Screening of the fruit pulp extract of *Momordica balsamina* for anti HIV property. African journal of Biotechnology. 2007;6(1).

Boual Z, Pierre G, Kemassi A, Mosbah S, Benaoun F, Delattre C, Michaud P, El Hadj MD. Chemical composition and biological activities of water-soluble polysaccharides from *Commiphora myrrha* (Nees) Engl. GUM. Analele Universităţii din Oradea, Fascicula Biologie. 2020;27(1):50-5.

Bouaziz F, Romdhane MB, Helbert CB, Buon L, Bhiri F, Bardaa S, Driss D, Koubaa M, Fakhfakh A, Sahnoun Z, Kallel F. Healing efficiency of oligosaccharides generated from almond gum (*Prunus amygdalus)* on dermal wounds of adult rats. Journal of Tissue Viability. 2014 Aug 1;23(3):98-108. https://doi.org/10.1016/j.jtv.2014.07.001

Boudjelal A, Henchiri C, Sari M, Sarri D, Hendel N, Benkhaled A, Ruberto G. Herbalists and wild medicinal plants in M'Sila (North Algeria): An ethnopharmacology survey. Journal of ethnopharmacology. 2013 Jul 9;148(2):395-402. https://doi.org/10.1016/j.jep.2013.03.082

Bouimeja B, Yetongnon KH, Touloun O, Berrougui H, Laaradia MA, Ouanaimi F, Chait A, Boumezzough A. Studies on antivenom activity of Lactuca serriola methanolic extract against *Buthus atlantis* scorpion venom by in vivo methods. South African Journal of Botany. 2019 Sep 1;125:270-9. https://doi.org/10.1016/j.sajb.2019.07.044

Bourdy G, DeWalt SJ, De Michel LC, Roca A, Deharo E, Muñoz V, Balderrama L, Quenevo C, Gimenez A. Medicinal plants uses of the Tacana, an Amazonian Bolivian ethnic group. Journal of ethnopharmacology. 2000 May 1;70(2):87-109. https://doi.org/10.1016/S0378-8741(99)00158-0

Bourdy G, DeWalt SJ, De Michel LC, Roca A, Deharo E, Muñoz V, Balderrama L, Quenevo C, Gimenez A. Medicinal plants uses of the Tacana, an Amazonian Bolivian ethnic group. Journal of ethnopharmacology. 2000 May 1;70(2):87-109. https://doi.org/10.1016/S0378-8741(99)00158-0

Bourhia M, Laasri FE, Aghmih K, Ullah R, Alqahtani AS, Mahmood HM, El Mzibri M, Said G, Khlil N, Benbacer L. Phytochemical composition, antioxidant activity, antiproliferative effect and acute toxicity study of *Bryonia dioica* roots used in North African alternative medicine. Intl J Agric Biol. 2020 Jan 1;23(3):597-602.

Boussouf L, Boutennoune H, Kebieche M, Adjeroud N, Al-Qaoud K, Madani K. Anti-inflammatory, analgesic and antioxidant effects of phenolic compound from Algerian *Mentha rotundifolia* L. leaves on experimental animals. South African Journal of Botany. 2017 Nov 1;113:77-83. https://doi.org/10.1016/j.sajb.2017.07.003

Boutaghane N, Nacer A, Kabouche Z, Ait-Kaki B. Comparative antibacterial activities of the essential oils of stems and seeds of *Pituranthos scoparius* from Algerian septentrional Sahara. Chemistry of Natural Compounds. 2004 Nov 1;40(6):606-7.

Bouzada ML, Fabri RL, Nogueira M, Konno TU, Duarte GG, Scio E. Antibacterial, cytotoxic and phytochemical screening of some traditional medicinal plants in Brazil. Pharmaceutical biology. 2009 Jan 1;47(1):44-52.

Bowen-Forbes CS, Zhang Y, Nair MG. Anthocyanin content, antioxidant, anti-inflammatory and anticancer properties of blackberry and raspberry fruits. Journal of food composition and analysis. 2010 Sep 1;23(6):554-60.

Bozin B, Mimica-Dukic N, Samojlik I, Jovin E. Antimicrobial and antioxidant properties of rosemary and sage (*Rosmarinus officinalis* L. and *Salvia officinalis* L., Lamiaceae) essential oils. Journal of agricultural and food chemistry. 2007 Sep 19;55(19):7879-85. https://doi.org/10.1021/jf0715323

Braga FC, Serra CP, Júnior NS, Oliveira AB, Côrtes SF, Lombardi JA. Angiotensin-converting enzyme inhibition by Brazilian plants. Fitoterapia. 2007 Jul 1;78(5):353-8. https://doi.org/10.1016/j.fitote.2007.02.007

Brand YM, Roa-Linares VC, Betancur-Galvis LA, Durán-García DC, Stashenko E. Antiviral activity of Colombian Labiatae and Verbenaceae family essential oils and monoterpenes on Human *Herpes* viruses. Journal of Essential Oil Research. 2016 Mar 3;28(2):130-7. https://doi.org/10.1080/10412905.2015.1093556

Brantner A, Grein E. Antibacterial activity of plant extracts used externally in traditional medicine. Journal of ethnopharmacology. 1994 Aug 1;44(1):35-40.

Bräuer AU, Kuhla A, Holzmann C, Wree A, Witt M. Current Challenges in Understanding the Cellular and Molecular Mechanisms in Niemann-Pick Disease Type C1. Int J Mol Sci. 2019;20(18). https://doi.org/10.3390/ijms20184392

Brizi C, Santulli C, Micucci M, Budriesi R, Chiarini A, Aldinucci C, Frosini M. Neuroprotective effects of *Castanea sativa* Mill. bark extract in human neuroblastoma cells subjected to oxidative stress. Journal of cellular biochemistry. 2016 Feb;117(2):510-20. doi: 10.1002/jcb.25302.

Brorson Ø, Brorson SH. Grapefruit seed extract is a powerful in vitro agent against motile and cystic forms of *Borrelia burgdorferi* sensu lato. Infection. 2007 Jun 1;35(3):206. doi: 10.1007/s15010-007-6105-0

Brose RD, Shin G, McGuinness MC, Schneidereith T, Purvis S, Dong GX, Keefer J, Spencer F, Smith KD. Activation of the stress proteome as a mechanism for small molecule therapeutics. Human molecular genetics. 2012 Oct 1;21(19):4237-52. https://doi.org/10.1093/hmg/dds247

Brüll V, Burak C, Stoffel-Wagner B, et al. Effects of a quercetin-rich onion skin extract on 24 h ambulatory blood pressure and endothelial function in overweight-to-obese patients with (pre-)hypertension: a randomised double-blinded placebo-controlled cross-over trial. Br J Nutr. 2015;114(8):1263–1277. doi:10.1017/S0007114515002950

Brunetti-Pierri N, Scaglia F. GM1 gangliosidosis: review of clinical, molecular, and therapeutic aspects. Mol Genet Metab. 2008;94(4):391-6. https://doi.org/10.1016/j.ymgme.2008.04.012

Brusotti G, Tosi S, Tava A, Picco AM, Grisoli P, Cesari I, Caccialanza G. Antimicrobial and phytochemical properties of stem bark extracts from *Piptadeniastrum africanum* (Hook f.) Brenan. Industrial Crops and Products. 2013 May 1;43:612-6. https://doi.org/10.1016/j.indcrop.2012.07.068

Bueno FG, Moreira EA, Morais GR, Pacheco IA, Baesso ML, Leite-Mello EV, Mello JC. Enhanced Cutaneous Wound Healing In Vivo by Standardized Crude Extract of *Poincianella pluviosa*. PLoS One. 2016 Mar 3;11(3):e0149223. doi: 10.1371/journal.pone.0149223.

Bukhari IA, Khan RA, Gilani AH, Ahmed S, Saeed SA. Analgesic, anti-inflammatory and anti-platelet activities of the methanolic extract of *Acacia modesta* leaves. Inflammopharmacology. 2010 Aug 1;18(4):187-96. https://doi.org/10.1007/s10787-010-0038-4

Bum EN, Taiwe GS, Moto FC, Ngoupaye GT, Vougat RR, Sakoue VD, Gwa C, Ayissi ER, Dong C, Rakotonirina A, Rakotonirina SV. Antiepileptic medicinal plants used in traditional medicine to treat epilepsy. In Clinical and genetic aspects of epilepsy 2011 Sep 15. IntechOpen. doi: 10.5772/18469

Bungihan ME, Matias CA. Determination of the antioxidant, phytochemical and antibacterial profiles of flowers from selected ornamental plants in Nueva Vizcaya, Philippines. Journal of Agricultural Science and Technology B. 2013 Dec 1;3:833-41.

Burdette A, Garner PL, Mayer EP, Hargrove JL, Hartle DK, Greenspan P. Anti-inflammatory activity of select sorghum (*Sorghum bicolor*) brans. Journal of medicinal food. 2010 Aug 1;13(4):879-87. doi.org/10.1089/jmf.2009.0147

Burgos-Edwards A, Martín-Pérez L, Jiménez-Aspee F, Theoduloz C, Schmeda-Hirschmann G, Larrosa M. Anti-inflammatory effect of polyphenols from Chilean currants (*Ribes magellanicum* and *R. punctatum*) after in vitro gastrointestinal digestion on Caco-2 cells: Anti-inflammatory activity of in vitro digested Chilean currants. Journal of Functional Foods. 2019 Aug 1;59:329-36. https://doi.org/10.1016/j.jff.2019.06.007

Bussmann RW, Ashley G, Sharon D, Chait G, Diaz D, Pourmand K, Jonat B, Somogy S, Guardado G, Aguirre C, Chan R. Proving that traditional knowledge works: the antibacterial activity of Northern Peruvian medicinal plants. Ethnobotany research and applications. 2011 Mar 2;9:067-96.

Bussmann RW, Glenn A, Meyer K, Rothrock A, Townesmith A, Sharon D, Díaz D, Castro M, Cardenas R, Regalado S. Antibacterial Activity of Medicinal Plants of Northern Peru—Part II. Arnaldoa. 2009;16(1):93-103.

Bussmann RW, Glenn A, Sharon D. Antibacterial activity of medicinal plants of Northern Peru–can traditional applications provide leads for modern science?. Ind J Trad Knowl 2010: 9(4):742-753.

Bussmann RW, Paniagua Zambrana NY, Sikharulidze S, Kikvidze Z, Kikodze D, Tchelidze D, Batsatsashvili K, Hart RE. Medicinal and food plants of Svaneti and Lechkhumi, Sakartvelo (Republic of Georgia), Caucasus. Med Aromat Plants. 2016b. 2016;5(266):2167-0412. Vanc doi: 10.4172/2167-0412.1000266

Bussmann RW, Sharon D, Perez F, Díaz D, Ford T, Rasheed T, Silva YB. Antibacterial activity of northern-peruvian medicinal plants. Arnaldoa. 2008;15(1):147-8.

Bussmann RW, Sharon D. Traditional medicinal plant use in Northern Peru: tracking two thousand years of healing culture. J Ethnobiol Ethnomed. 2006;2:47. doi: 10.1186/1746-4269-2-47.

Bussmann RW, Zambrana NY, Romero C, Hart RE. Astonishing diversity—the medicinal plant markets of Bogotá, Colombia. Journal of ethnobiology and ethnomedicine. 2018 Dec;14(1):43.

Butaud JF, Gaydou V, Bianchini JP, Faure R, Raharivelomanana P. Dihydroxysesquiterpenoids from *Santalum insulare* of french Polynesia. Natural Product Communications. 2007 Mar;2(3):1934578X0700200303. https://doi.org/10.1177/1934578X0700200303

Butnariu M, Bostan C. Antimicrobial and anti-inflammatory activities of the volatile oil compounds from *Tropaeolum majus* L.(Nasturtium). African journal of biotechnology. 2011;10(31):5900-9.

Čabarkapa IS, Sedej IJ, Sakač MB, Šarić LĆ, Plavšić DV. Antimicrobial activity of buckwheat (*Fagopyrum esculentum* Moench) hulls extract. Food and feed research. 2008;35(4):159-64.

Cabrini DA, Moresco HH, Imazu P, Silva CD, Pietrovski EF, Mendes DA, Prudente AD, Pizzolatti MG, Brighente IM, Otuki MF. Analysis of the potential topical anti-inflammatory activity of *Averrhoa carambola* L. in mice. Evidence-based Complementary and Alternative Medicine. 2011 Jan 1;2011. https://doi.org/10.1093/ecam/neq026

Caceres A, Cabrera O, Morales O, Mollinedo P, Mendia P. Pharmacological properties of *Moringa oleifera*. 1: Preliminary screening for antimicrobial activity. Journal of ethnopharmacology. 1991 Jul 1;33(3):213-6. https://doi.org/10.1016/0378-8741(91)90078-R

Cáceres A, Fletes L, Aguilar L, Ramirez O, Figueroa L, Taracena AM, Samayoa B. Plants used in Guatemala for the treatment of gastrointestinal disorders. 3. Confirmation of activity against enterobacteria of 16 plants. Journal of Ethnopharmacology. 1993b Jan 1;38(1):31-8. https://doi.org/10.1016/0378-8741(93)90076-H

Caceres A, Lopez B, Juarez X, Del Aguila J, Garcia S. Plants used in Guatemala for the treatment of dermatophytic infections. 2. Evaluation of antifungal activity of seven American plants. Journal of Ethnopharmacology. 1993a Dec 1;40(3):207-13. https://doi.org/10.1016/0378-8741(93)90070-L

Calabrese V, Cornelius C, Dinkova-Kostova AT, Iavicoli I, Di Paola R, Koverech A, Cuzzocrea S, Rizzarelli E, Calabrese EJ. Cellular stress responses, hormetic phytochemicals and vitagenes in aging and longevity. Biochimica et Biophysica Acta (BBA)-Molecular Basis of Disease. 2012 May 1;1822(5):753-83. https://doi.org/10.1016/j.bbadis.2011.11.002

Calderón AI, Cubilla M, Espinosa A, Gupta MP. Screening of plants of Amaryllidaceae and related families from Panama as sources of acetylcholinesterase inhibitors. Pharmaceutical biology. 2010 Sep 1;48(9):988-93. https://doi.org/10.3109/13880200903418514

Calero-Armijos LL, Herrera-Calderon O, Arroyo-Acevedo JL, Rojas-Armas JP, Hañari-Quispe RD, Figueroa-Salvador L. Histopathological evaluation of latex of Bellaco-Caspi, *Himatanthus sucuuba* (Spruce) Woodson on wound healing effect in BALB/C mice. Vet World. 2020 Jun;13(6):1045-1049. doi: 10.14202/vetworld.2020.1045-1049.

Cam M, Basyigit B, Alasalvar H, Yilmaztekin M, Ahhmed A, Sagdic O, Konca Y, Telci I. Bioactive properties of powdered peppermint and spearmint extracts: Inhibition of key enzymes linked to hypertension and type 2 diabetes. Food Bioscience. 2020 Jun 1;35:100577. https://doi.org/10.1016/j.fbio.2020.100577

Camargo Filho I, Cortez DA, Ueda-Nakamura T, Nakamura CV, Dias Filho BP. Antiviral activity and mode of action of a peptide isolated from *Sorghum bicolor*. Phytomedicine. 2008 Mar;15(3):202-8. doi: 10.1016/j.phymed.2007.07.059.

Cambay Z, Baydas G, Tuzcu M, Bal R. Pomegranate (*Punica granatum* L.) flower improves learning and memory performances impaired by diabetes mellitus in rats. Acta Physiologica Hungarica. 2011 Dec 1;98(4):409-20. https://doi.org/10.1556/aphysiol.98.2011.4.4

Campana PR, Coleman CM, Sousa LP, Teixeira MM, Ferreira D, Braga FC. Mansoins C–F, Oligomeric Flavonoid Glucosides Isolated from *Mansoa hirsuta* Fruits with Potential Anti-inflammatory Activity. Journal of natural products. 2016 Aug 22;79(9):2279-86. 10.1021/acs.jnatprod.6b00390

Cannas S, Molicotti P, Ruggeri M, Cubeddu M, Sanguinetti M, Marongiu B, Zanetti S. Antimycotic activity of *Myrtus communis* L. towards *Candida* spp. from clinical isolates. The Journal of Infection in Developing Countries. 2013 Mar 14;7(03):295-8.

Cao H, Sethumadhavan K. Regulation of Cell Viability and Anti-inflammatory Tristetraprolin Family Gene Expression in Mouse Macrophages by Cottonseed Extracts. Scientific reports. 2020 Jan 21;10(1):1-1. https://doi.org/10.1038/s41598-020-57584-9

Capasso F, Cerri R, Morrica P, Senatore F. Chemical composition and anti-inflammatory activity of an alcoholic extract of *Teucrium polium* L. Boll Soc Ital Biol Sper. 1983 Nov 30;59(11):1639-43.

Capatina L, Napoli EM, Ruberto G, Hritcu L. *Origanum vulgare* ssp. hirtum (Lamiaceae) Essential Oil Prevents Behavioral and Oxidative Stress Changes in the Scopolamine Zebrafish Model. Molecules. 2021 Jan;26(23):7085.

Carbajal D, Casaco A, Arruzazabala L, Gonzalez R, Tolon Z. Pharmacological study of *Cymbopogon citratus* leaves. Journal of Ethnopharmacology. 1989 Feb 1;25(1):103-7. https://doi.org/10.1016/0378-8741(89)90049-4

Cardozo LF, Stockler-Pinto MB, Mafra D. Brazil nut consumption modulates Nrf2 expression in hemodialysis patients: A pilot study. Molecular nutrition & food research. 2016 Jul;60(7):1719-24

Carmen G, Hancu G. Antimicrobial and antifungal activity of *Pelargonium roseum* essential oils. Advanced pharmaceutical bulletin. 2014 Dec;4(Suppl 2):511. doi: 10.5681/apb.2014.075

Carrasco A, Perez E, Cutillas AB, Martinez-Gutierrez R, Tomas V, Tudela J. *Origanum vulgare* and *Thymbra capitata* essential oils from Spain: determination of aromatic profile and bioactivities. Natural product communications. https://doi.org/10.1177/1934578X1601100133

Carretero ME, López-Pérez JL, Abad MJ, Bermejo P, Tillet S, Israel A, Noguera-P B. Preliminary study of the anti-inflammatory activity of hexane extract and fractions from *Bursera simaruba* (Linneo) Sarg.(Burseraceae) leaves. Journal of ethnopharmacology. 2008 Feb 28;116(1):11-5. https://doi.org/10.1016/j.jep.2007.10.034

Carrol DH, Chassagne F, Dettweiler M, Quave CL. Antibacterial activity of plant species used for oral health against *Porphyromonas gingivalis*. PLoS One. 2020 Oct 8;15(10):e0239316. doi: 10.1371/journal.pone.0239316.

Caruana M, Högen T, Levin J, Hillmer A, Giese A, Vassallo N. Inhibition and disaggregation of α-synuclein oligomers by natural polyphenolic compounds. FEBS letters. 2011 Apr 20;585(8):1113-20.

Carvalho JC, Sertié JA, Barbosa MV, Patrício KC, Caputo LR, Sarti SJ, Ferreira LP, Bastos JK. Anti-inflammatory activity of the crude extract from the fruits of *Pterodon emarginatus* Vog. Journal of Ethnopharmacology. 1999 Feb 1;64(2):127-33.

Casado R, Landa A, Calvo J, García-Mina JM, Marston A, Hostettmann K, Calvo MI. Anti-inflammatory, antioxidant and antifungal activity of *Chuquiraga spinosa*. Pharmaceutical biology. 2011 Jun 1;49(6):620-6. doi.org/10.3109/13880209.2011.577436

Castillo-Juárez I, González V, Jaime-Aguilar H, Martínez G, Linares E, Bye R, Romero I. Anti-*Helicobacter pylori* activity of plants used in Mexican traditional medicine for gastrointestinal disorders. Journal of ethnopharmacology. 2009 Mar 18;122(2):402-5.

Cecílio AB, de Faria DB, de Carvalho Oliveira P, Caldas S, de Oliveira DA, Sobral ME, Duarte MG, de Souza Moreira CP, Silva CG, de Almeida VL. Screening of Brazilian medicinal plants for antiviral activity against rotavirus. Journal of ethnopharmacology. 2012 Jun 14;141(3):975-81. https://doi.org/10.1016/j.jep.2012.03.031

Céspedes CL, Avila JG, Garcıa AM, Becerra J, Flores C, Aqueveque P, Bittner M, Hoeneisen M, Martinez M, Silva M. Antifungal and antibacterial activities of *Araucaria araucana* (Mol.) K. Koch heartwood lignans. Zeitschrift für Naturforschung C. 2006 Feb 1;61(1-2):35-43.

Cevik O, Akpinar H, Oba R, Cilingir OT, Ozdemir ZN, Cetinel S, Yoldemir T. The effect of *Momordica charantia* intake on the estrogen receptors ESRα/ESRβ gene levels and apoptosis on uterine tissue in ovariectomy rats. Molecular biology reports. 2015 Jan;42(1):167-77. https://doi.org/10.1007/s11033-014-3756-7

Cevikelli-Yakut ZA, Ertas B, Sen A, Koyuncuoglu T, Yegen BC, Sener G. *Myrtus communis* improves cognitive impairment in renovascular hypertensive rats. Journal of Physiology and Pharmacology: an Official Journal of the Polish Physiological Society. 2020 Oct 1;71(5). doi: 10.26402/jpp.2020.5.07

Ceylan O, Sahin MD, Akdamar G. Antioxidant and anti-quorum sensing potential of *Acer monspessulanum* subsp. *monspessulanum* extracts. Planta medica. 2016 Oct;82(15):1335-40. doi: 10.1055/s-0042-105294

Chakraborty AR, Devi RK, Rita S, Sharatchandra KH, Singh TI. Preliminary studies on antiinflammatory and analgesic activities of *Spilanthes acmella* in experimental animal models. Indian journal of pharmacology. 2004 May 1;36(3):148.

Champatisingh D, Sahu PK, Pal A, Nanda GS. Anticataleptic and antiepileptic activity of ethanolic extract of leaves of *Mucuna pruriens*: A study on role of dopaminergic system in epilepsy in albino rats. Indian J Pharmacol. 2011;43(2):197–199. doi:10.4103/0253-7613.77368

Champatisingh D, Sahu PK, Pal A, Nanda GS. Anticataleptic and antiepileptic activity of ethanolic extract of leaves of *Mucuna pruriens*: A study on role of dopaminergic system in epilepsy in albino rats. Indian J Pharmacol. 2011 Apr;43(2):197-9. doi: 10.4103/0253-7613.77368.

Chamundeeswari D, Vasantha J, Gopalakrishnan S, Sukumar E. Anti-inflammatory and antinociceptive activities of *Trewia polycarpa* roots. Fitoterapia. 2004 Dec 1;75(7-8):740-4. https://doi.org/10.1016/j.fitote.2004.07.001

Chan EW, Yeo ET, Wong KW, See ML, Wong KY, Gan SY. *Piper sarmentosum* Roxb. Root Extracts Confer Neuroprotection by Attenuating Beta Amyloid-Induced Pro-Inflammatory Cytokines Released from Microglial Cells. Current Alzheimer Research. 2019 Mar 1;16(3):251-60. doi: 10.2174/1567205016666190228124630.

Chanda I, Sarma U, Basu SK, Lahkar M, Dutta SK. A protease isolated from the latex of *Plumeria rubra* linn (apocynaceae) 2: Anti-inflammatory and wound-healing activities. Tropical Journal of Pharmaceutical Research. 2011;10(6):755-60. doi: 10.4314/tjpr.v10i6.8

Chander MP, Kartick C, Gangadhar J, Vijayachari P. Ethno medicine and healthcare practices among Nicobarese of Car Nicobar–An indigenous tribe of Andaman and Nicobar Islands. Journal of ethnopharmacology. 2014 Dec 2;158:18-24. https://doi.org/10.1016/j.jep.2014.09.046

Chander R, Singh K, Khanna AK, Kaul SM, Puri A, Saxena R, Bhatia G, Rizvi F, Rastogi AK. Antidyslipidemic and antioxidant activities of different fractions of *Terminalia arjuna* stem bark. Indian Journal of Clinical Biochemistry. 2004 Jul 1;19(2):141.https://doi.org/10.1007/BF02894274

Chandra DN, Prasanth GK, Singh N, Kumar S, Jithesh O, Sadasivan C, Sharma S, Singh TP, Haridas M. Identification of a novel and potent inhibitor of phospholipase A2 in a medicinal plant: crystal structure at 1.93 Å and surface plasmon resonance analysis of phospholipase A2 complexed with berberine. Biochimica et Biophysica Acta (BBA)-Proteins and Proteomics. 2011 May 1;1814(5):657-63. https://doi.org/10.1016/j.bbapap.2011.03.002

Chandramohan G, Al-Numair KS, Sridevi M, Pugalendi KV. Antihyperlipidemic activity of 3-hydroxymethyl xylitol, a novel antidiabetic compound isolated from *Casearia esculenta* (Roxb.) root, in streptozotocin-diabetic rats. Journal of biochemical and molecular toxicology. 2010 Mar;24(2):95-101. https://doi.org/10.1002/jbt.20317.

Chang CF, Lee YC, Lee KH, Lin HC, Chen CL, Shen CK, Huang CC. Therapeutic effect of berberine on TDP-43-related pathogenesis in FTLD and ALS. Journal of biomedical science. 2016 Dec;23(1):72. doi:10.1186/s12929-016-0290-z

Chang RS, Yeung HW. Inhibition of growth of human immunodeficiency virus in vitro by crude extracts of Chinese medicinal herbs. Antiviral Res. 1988;9(3):163-175. doi:10.1016/0166-3542(88)90001-0

Chang VH, Chiu TH, Fu SC. In vitro anti-inflammatory properties of fermented pepino (*Solanum muricatum*) milk by γ-aminobutyric acid-producing *Lactobacillus brevis* and an in vivo animal model for evaluating its effects on hypertension. Journal of the Science of Food and Agriculture. 2016 Jan 15;96(1):192-8.

Chaniad P, Wattanapiromsakul C, Pianwanit S, Tewtrakul S. Anti-HIV-1 integrase compounds from *Dioscorea bulbifera* and molecular docking study. Pharmaceutical biology. 2016 Jun 2;54(6):1077-85. https://doi.org/10.3109/13880209.2015.1103272

Chanmahasathien W, Li Y, Satake M, Oshima Y, Ruangrungsi N, Ohizumi Y. Prenylated xanthones with NGF-potentiating activity from *Garcinia xanthochymus*. Phytochemistry. 2003 Nov 1;64(5):981-6.

Chansakaow S, Ruangrungsi N, Ishikawa T. Isolation of pyropheophorbide a from the leaves of *Atalantia monophylla* (Roxb.) Corr.(Rutaceae) as a possible antiviral active principle against *Herpes simplex* virus type 2. Chemical and pharmaceutical bulletin. 1996 Jul 15;44(7):1415-7. https://doi.org/10.1248/cpb.44.1415

Charami MT, Lazari D, Karioti A, Skaltsa H, Hadjipavlou-Litina D, Souleles C. Antioxidant and antiinflammatory activities of *Sideritis perfoliata* subsp. *perfoliata* (Lamiaceae). Phytotherapy Research: An International Journal Devoted to Pharmacological and Toxicological Evaluation of Natural Product Derivatives. 2008 Apr;22(4):450-4. https://doi.org/10.1002/ptr.2333

Chariandy CM, Seaforth CE, Phelps RH, Pollard GV, Khambay BP. Screening of medicinal plants from Trinidad and Tobago for antimicrobial and insecticidal properties. Journal of ethnopharmacology. 1999 Mar 1;64(3):265-70. https://doi.org/10.1016/S0378-8741(98)00130-5

Charoenchai P, Vajrodaya S, Somprasong W, Mahidol C, Ruchirawat S, Kittakoop P. Part 1: Antiplasmodial, cytotoxic, radical scavenging and antioxidant activities of Thai plants in the family Acanthaceae. Planta medica. 2010 Nov;76(16):1940-3.

Chatterjee S, Rajarajan S, Sharm UR, Ramesh K. Evaluation of Antinociceptive and antiinflammatory activities of *Tagetes erecta* Linn. leaves. Archieve Phaarmacol. Sci. Res. 2009;2:207-11.

Chaudhari Y, Badhe M, Kumar EP, Mody H, Kokardekar R. An investigation of antibacterial activity of *Pedilanthus tithymaloides* on different strains of bacteria. Int. J. Pharm. Phytopharmacol. Res. 2012;1(6):385-6

Chaudhary MI, He Q, Cheng YY, Xiao PG. Ethnobotany of medicinal plants from tian mu Shan biosphere reserve, Zhejiang-province, China. Asian J Plant Sci. 2006;5(4):646-53

Chaudhuri D, Sevanan M. Investigation on phytochemicals and antibacterial activity of the leaf and stem extracts of *Iresine herbstii.* International Journal Pharma and Bio Sciences. 2012;3(4):697-705.

Chauhan NB. Anti-amyloidogenic effect of *Allium sativum* in Alzheimer's transgenic model Tg2576. Journal of herbal pharmacotherapy. 2003 Jan 1;3(1):95-107. https://doi.org/10.1080/J157v03n01_05

Chauke MA, Shai LJ, Mogale MA, Mokgotho MP. Antibacterial and anti HIV 1 reverse transcriptase activity of selected medicinal plants from Phalaborwa, South Africa. Res. J. Med. Plant. 2016;10:388-95. doi: 10.3923/rjmp.2016.388.395

Chavan R, Chowdhary A. In vitro inhibitory activity of *Justicia adhatoda* extracts against influenza virus infection and hemagglutination. Int. J. Pharm. Sci. Rev. Res. 2014;25(2):231-6.

Che J, Liang B, Zhang Y, Wang Y, Tang J, Shi G. Kaempferol alleviates ox-LDL-induced apoptosis by up-regulation of autophagy via inhibiting PI3K/Akt/mTOR pathway in human endothelial cells. Cardiovascular Pathology. 2017 Nov 1;31:57-62. https://doi.org/10.1016/j.carpath.2017.08.001

Chemsa AE, Derdouri S, Labbi Z, Acila S, Amara DG, Chouikh A, Kherraz K, Allali A, Zellagui A. Total phenolic and total flavonoid contents of different solvent extracts of *Bassia muricata* (L.) Asch. and evaluation of antibacterial and antioxidant activities. Journal of Chemical and Pharmaceutical Research. 2016;8(4):1317-21.

Chen BT, Li WX, He RR, Li YF, Tsoi B, Zhai YJ, Kurihara H. Anti-inflammatory effects of a polyphenols-rich extract from tea (*Camellia sinensis*) flowers in acute and chronic mice models. Oxidative medicine and cellular longevity. 2012 Jul 30;2012.

Chen F, Eckman EA, Eckman CB, Chen F, Eckman EA, Eckman CB. Reductions in levels of the Alzheimer's amyloid β peptide after oral administration of ginsenosides. The FASEB Journal. 2006 Jun;20(8):1269-71. https://doi.org/10.1096/fj.05-5530fje

Chen F, Yang L, Huang Y, Chen Y, Sang H, Duan W, Yang J. Isocorilagin, isolated from *Canarium album* (Lour.) Raeusch, as a potent neuraminidase inhibitor against influenza A virus. Biochemical and Biophysical Research Communications. 2020 Feb 26;523(1):183-9. doi: 10.1016/j.bbrc.2019.12.043

Chen H, He X, Liu Y, Li J, He Q, Zhang C, Wei B, Zhang Y, Wang J. Extraction, purification and anti-fatigue activity of γ-aminobutyric acid from mulberry (*Morus alba* L.) leaves. Sci Rep. 2016 Jan 8;6:18933. doi: 10.1038/srep18933.

Chen H, Sun J, Liu J, Gou Y, Zhang X, Wu X, Sun R, Tang S, Kan J, Qian C, Zhang N. Structural characterization and anti-inflammatory activity of alkali-soluble polysaccharides from purple sweet potato. International journal of biological macromolecules. 2019 Jun 15;131:484-94.

Chen HJ, Chen CN, Sung ML, Wu YC, Ko PL, Tso TK. *Canna indica* L. attenuates high-glucose-and lipopolysaccharide-induced inflammatory mediators in monocyte/macrophage. Journal of ethnopharmacology. 2013 Jun 21;148(1):317-21. doi.org/10.1016/j.jep.2013.04.037

Chen J, Deng X, Liu N, Li M, Liu B, Fu Q, Qu R, Ma S. Quercetin attenuates tau hyperphosphorylation and improves cognitive disorder via suppression of ER stress in a manner dependent on AMPK pathway. Journal of Functional Foods. 2016 Apr 1;22:463-76. https://doi.org/10.1016/j.jff.2016.01.036

Chen J, Shi X, Chen Y, Liang H, Cheng C, He Q. Neuroprotective effects of chloroform and aqueous fractions of noni juice against t-Butyl hydroperoxide-induced oxidative damage in SH-SY5Y cells. Food & nutrition research. 2018;62. doi: 10.29219/fnr.v62.1605

Chen JH, Cui GY, Liu JY, Tan RX. Pinelloside, an antimicrobial cerebroside from *Pinellia ternata*. Phytochemistry. 2003 Oct 1;64(4):903-6. https://doi.org/10.1016/S0031-9422(03)00421-7

Chen K, Plumb GW, Bennett RN, Bao Y. Antioxidant activities of extracts from five anti-viral medicinal plants. Journal of ethnopharmacology. 2005 Jan 4;96(1-2):201-5. https://doi.org/10.1016/j.jep.2004.09.020

Chen LF, Zhong YL, Luo D, Liu Z, Tang W, Cheng W, Xiong S, Li YL, Li MM. Antiviral activity of ethanol extract of *Lophatherum gracile* against respiratory syncytial virus infection. Journal of ethnopharmacology. 2019 Oct 5;242:111575. https://doi.org/10.1016/j.jep.2018.10.036

Chen LL, Song JX, Lu JH, Yuan ZW, Liu LF, Durairajan SS, Li M. Corynoxine, a natural autophagy enhancer, promotes the clearance of alpha-synuclein via Akt/mTOR pathway. Journal of Neuroimmune Pharmacology. 2014 Jun 1;9(3):380-7. doi: 10.1007/s11481-014-9528-2

Chen Q, Chen X, Fu Z, Bais S, Hou X. Anti-amnesic Effect of *Leea indica* Extract in Scopolamine-induced Amnesia of Alzheimer's Type in Rats. International Journal of Pharmacology. 2019 Jan 1;15(1):116-23. doi: 10.3923/ijp.2019.116.123

Chen SX, Wan M, Loh BN. Active constituents against HIV-1 protease from *Garcinia mangostana*. Planta medica. 1996 Aug;62(04):381-2. doi: 10.1055/s-2006-957916

Chen T, Hu S, Zhang H, Guan Q, Yang Y, Wang X. Anti-inflammatory effects of *Dioscorea alata* L. anthocyanins in a TNBS-induced colitis model. Food & function. 2017;8(2):659-69.

Chen WC, Hsu YJ, Lee MC, Li HS, Ho CS, Huang CC, Chen FA. Effect of burdock extract on physical performance and physiological fatigue in mice. J Vet Med Sci. 2017 Oct 7;79(10):1698-1706. doi: 10.1292/jvms.17-0345. Epub 2017 Sep 11.

Chen Y, Gao X, Liu Q, Zeng L, Zhang K, Mu K, Zhang D, Zou H, Wu N, Ou J, Wang Q. Alpha-asarone improves cognitive function of aged rats by alleviating neuronal excitotoxicity via GABAA receptors. Neuropharmacology. 2019 Nov 5:107843. doi: 10.1016/j.neuropharm.2019.107843.

Chen Y, Zhang DQ, Liao Z, Wang B, Gong S, Wang C, Zhang MZ, Wang GH, Cai H, Liao FF, Xu JP. Anti-oxidant polydatin (piceid) protects against substantia nigral motor degeneration in multiple rodent models of Parkinson's disease. Molecular neurodegeneration. 2015 Dec;10(1):4. https://doi.org/10.1186/1750-1326-10-4

Cherdchu C, Karlsson E. Proteolytic-independent cobra neurotoxin inhibiting activity of *Curcuma* sp.(Zingiberaceae). The Southeast Asian journal of tropical medicine and public health. 1983 Jun;14(2):176-80.

Cherng JM, Chiang W, Chiang LC. Immunomodulatory activities of common vegetables and spices of Umbelliferae and its related coumarins and flavonoids. Food chemistry. 2008 Feb 1;106(3):944-50. https://doi.org/10.1016/j.foodchem.2007.07.005

Cheung AH, Wong JH, Ng TB. *Musa acuminata* (Del Monte banana) lectin is a fructose-binding lectin with cytokine-inducing activity. Phytomedicine. 2009 Jun 1;16(6-7):594-600. https://doi.org/10.1016/j.phymed.2008.12.016

Chiang LC, Ng LT, Cheng PW, Chiang W, Lin CC. Antiviral activities of extracts and selected pure constituents of *Ocimum basilicum*. Clinical and Experimental Pharmacology and Physiology. 2005 Oct;32(10):811-6. https://doi.org/10.1111/j.1440-1681.2005.04270.x

Chiang YM, Chang CL, Chang SL, Yang WC, Shyur LF. Cytopiloyne, a novel polyacetylenic glucoside from *Bidens pilosa*, functions as a T helper cell modulator. Journal of Ethnopharmacology. 2007 Apr 4;110(3):532-8. https://doi.org/10.1016/j.jep.2006.10.007

Chibani S, Amira L, Kabouche A, Semra Z, Smati F, Aburjai T, Kabouche Z. Antibacterial activity and chemical composition of essential oil of *Santolina rosmarinifolia* L.(Asteraceae) from Algeria. Der Pharmacia Lettre. 2013;5(2):238-41.

Chindo BA, Anuka JA, McNeil L, Yaro AH, Adamu SS, Amos S, Connelly WK, Lees G, Gamaniel KS. Anticonvulsant properties of saponins from *Ficus platyphylla* stem bark. Brain Research Bulletin. 2009 Mar 30;78(6):276-82.

Chindo BA, Schröder H, Becker A. Methanol extract of *Ficus platyphylla* ameliorates seizure severity, cognitive deficit and neuronal cell loss in pentylenetetrazole-kindled mice. Phytomedicine. 2015 Jan 15;22(1):86-93. https://doi.org/10.1016/j.phymed.2014.10.005

Ching FP, Omogbai E, Otokiti I. Aqueous stem bark extract of *Stereospermum kunthianum* (Cham, Sandrine Petit) protects against generalized seizures in pentylenetetrazole and electro-convulsive models in rodents. African Journal of Traditional, Complementary and Alternative Medicines. 2009;6(4).

Chingsuwanrote P, Muangnoi C, Parengam K, Tuntipopipat S. Antioxidant and anti-inflammatory activities of durian and rambutan pulp extract. International Food Research Journal. 2016 May 1;23(3):939.

Chistokhodova N, Nguyen C, Calvino T, Kachirskaia I, Cunningham G, Miles DH. Antithrombin activity of medicinal plants from central Florida. Journal of ethnopharmacology. 2002 Jul 1;81(2):277-80.

Chitemerere TA, Mukanganyama S. *In vitro* antibacterial activity of selected medicinal plants from Zimbabwe. African J Pl Sci Biotech. 2011;5:1–7.

Chithra P, Sajithlal GB, Chandrakasan G. Influence of *Aloe vera* on the healing of dermal wounds in diabetic rats. Journal of ethnopharmacology. 1998 Jan 1;59(3):195-201.

Chitra V, Pavan Kumar K. Neuroprotective studies of *Rubia cordifolia* Linn. on β-amyloid induced cognitive dysfunction in mice. Int J Pharm Tech Res. 2009 Oct;1(4):1000-9.

Chlebek J, Novák Z, Kassemová D, Šafratová M, Kostelník J, Malý L, Ločárek M, Opletal L, Hošťálková A, Hrabinová M, Kuneš J. Isoquinoline alkaloids from *Fumaria officinalis* L. and their biological activities related to Alzheimer's disease. Chemistry & biodiversity. 2016 Jan;13(1):91-9. https://doi.org/10.1002/cbdv.201500033

Cho JY, Park KH, Hwang DY, Chanmuang S, Jaiswal L, Park YK, Park SY, Kim SY, Kim HR, Moon JH, Ham KS. Antihypertensive effects of *Artemisia scoparia* Waldst in spontaneously hypertensive rats and identification of angiotensin I converting enzyme inhibitors. Molecules. 2015 Nov;20(11):19789-804. https://doi.org/10.3390/molecules201119657

Cho JY, Park SC, Kim TW, Kim KS, Song JC, Lee HM, Sung HJ, Rhee MH, Kim SK, Park HJ, Song YB. Radical scavenging and anti-inflammatory activity of extracts from *Opuntia humifusa* Raf. Journal of pharmacy and pharmacology. 2006 Jan;58(1):113-9. ttps://doi.org/10.1211/jpp.58.1.0014

Choi BM, Kim SM, Park TK, Li G, Hong SJ, Park R, Chung HT, Kim BR. Piperine protects cisplatin-induced apoptosis via heme oxygenase-1 induction in auditory cells. The Journal of nutritional biochemistry. 2007 Sep 1;18(9):615-22. https://doi.org/10.1016/j.jnutbio.2006.11.012

Choi EM, Hwang JK. Antiinflammatory, analgesic and antioxidant activities of the fruit of *Foeniculum vulgare*. Fitoterapia. 2004 Sep 1;75(6):557-65. https://doi.org/10.1016/j.fitote.2004.05.005

Choi G, Han AR, Lee JH, Park JY, Kang U, Hong J, Kim YS, Seo EK. A comparative study on hulled adlay and unhulled adlay through evaluation of their LPS-induced anti-inflammatory effects, and isolation of pure compounds. Chemistry & biodiversity. 2015 Mar;12(3):380-7. https://doi.org/10.1002/cbdv.201400242

Choi JG, Lee H, Kim YS, Hwang YH, Oh YC, Lee B, Moon KM, Cho WK, Ma JY. *Aloe vera* and its components inhibit influenza A virus-induced autophagy and replication. The American journal of Chinese medicine. 2019 Sep 11;47(06):1307-24. https://doi.org/10.1142/S0192415X19500678

Choi JH, Lee MJ, Jang M, Kim EJ, Shim I, Kim HJ, Lee S, Lee SW, Kim YO, Cho IH. An oriental medicine, Hyungbangpaedok-San attenuates motor paralysis in an experimental model of multiple sclerosis by regulating the T cell response. PloS one. 2015 Oct 7;10(10):e0138592.

Choi WJ, Kim SK, Park HK, Sohn UD, Kim W. Anti-inflammatory and anti-superbacterial properties of sulforaphane from shepherd's purse. The Korean Journal of Physiology & Pharmacology. 2014 Feb 1;18(1):33-9. doi.org/10.3390/molecules22061023

Chong CLG, Othman F, Hussan F. Vascular Protective Effects of *Morinda citrifolia* Leaf Extract on Postmenopausal Rats Fed with Thermoxidized Palm Oil Diet: Evidence at Microscopic Level. *Int J Vasc Med*. 2018;2018:6317434. Published 2018 Sep 5. doi:10.1155/2018/6317434

Chopade AR, Patil PA, Mali SN. Pharmacological Aspects of *Phyllanthus fraternus* Standardized Extract (Rich in Lignans and Tannins) as a Pain Modulator. The Open Pain Journal. 2020 Sep 25;13(1). doi: 10.2174/1876386302013010022

Chou CC, Lue SI, Fu YS, Weng CF. *Plantago asiatica* seed Extracts Alleviated Blood Pressure of Phase I–Spontaneous Hypertension Rats. The FASEB Journal. 2018 Apr;32(1_supplement):715-16.

Chou SC, Everngam MC, Sturtz G, Beck JJ. Antibacterial activity of components *from Lomatium californicum*. Phytotherapy Research: An International Journal Devoted to Pharmacological and Toxicological Evaluation of Natural Product Derivatives. 2006 Feb;20(2):153-6.

Choudhary D, Bhattacharyya S, Bose S. Efficacy and safety of Ashwagandha (*Withania somnifera* (L.) Dunal) root extract in improving memory and cognitive functions. Journal of Dietary Supplements. 2017 Nov 2;14(6):599-612. doi: 10.1080/19390211.2017.1284970

Choudhary MI, Jalil S, Nawaz SA, Khan KM, Tareen RB. Antiinflammatory and lipoxygenase inhibitory compounds from *Vitex agnus‐castus.* Phytotherapy Research: An International Journal Devoted to Pharmacological and Toxicological Evaluation of Natural Product Derivatives. 2009 Sep;23(9):1336-9. https://doi.org/10.1002/ptr.2639

Choudhury MD, Nath D, Talukdar AD. Antimicrobial activity of *Melastoma malabathricum* L. Assam University Journal of Science and Technology. 2011 Apr 6;7(1):76-8.

Choudhury MD, Paul SB, Choudhury S, Choudhury S, Choudhury PP. Isolation, characterization and bio-activity screening of compound from *Clerodendrum viscosum* Vent. Assam University Journal of Science and Technology. 2010 Sep 8;4(1):29-34.

Choudhury S, Rahaman CH, Mandal S. Studies on *Ipomoea cairica* (L.) Sweet-A Promising Ethnomedicinally Important Plant. Journal of Innovations in Pharmaceuticals and Biological Sciences. 2015;2(4):378-95.

Chowdhury R, Hasan CM, Rashid MA. Antimicrobial activity of *Toona ciliata* and *Amoora rohituka.* Fitoterapia. 2003 Feb 1;74(1):155-8. doi: 10.1016/S0367-326X(02)00322-2 ·

Chowdhury S, Kumar S. Alpha-terpinyl acetate: A natural monoterpenoid from *Elettaria cardamomum* as multi-target directed ligand in Alzheimer's disease. Journal of Functional Foods. 2020 May 1;68:103892.https://doi.org/10.1016/j.jff.2020.103892

Chu JM, Xiong W, Linghu KG, Liu Y, Zhang Y, Zhao GD, Irwin MG, Wong GT, Yu H. *Siegesbeckia orientalis* L. extract attenuates postoperative cognitive dysfunction, systemic inflammation, and neuroinflammation. Experimental neurobiology. 2018 Dec;27(6):564. doi:10.5607/en.2018.27.6.564

Chu W, Qiao G, Bai Y, Pan Z, Li G, Piao X, Wu L, Lu Y, Yang B. Flavonoids from Chinese *Viscum coloratum* produce cytoprotective effects against ischemic myocardial injuries: inhibitory effect of flavonoids on PAF-Induced Ca2+ overload. Phytotherapy Research: An International Journal Devoted to Pharmacological and Toxicological Evaluation of Natural Product Derivatives. 2008 Jan;22(1):134-7. https://doi.org/10.1002/ptr.2267

Chu X, Liu XJ, Qiu JM, Zeng XL, Bao HR, Shu J. Effects of *Astragalus* and *Codonopsis pilosula* polysaccharides on alveolar macrophage phagocytosis and inflammation in chronic obstructive pulmonary disease mice exposed to PM2. 5. Environmental toxicology and pharmacology. 2016 Dec 1;48:76-84. doi.org/10.1016/j.etap.2016.10.006

Chuang PH, Hsieh PW, Yang YL, Hua KF, Chang FR, Shiea J, Wu SH, Wu YC. Cyclopeptides with anti-inflammatory activity from seeds of *Annona montana*. Journal of natural products. 2008 Aug 8;71(8):1365-70.

Chukwujekwu JC, Van Staden J, Smith P. Antibacterial, anti-inflammatory and antimalarial activities of some Nigerian medicinal plants. South African Journal of Botany. 2005 Nov 1;71(3/4):316.

Chung KO, Kim BY, Lee MH, Kim YR, Chung HY, Park JH, Moon JO. In-vitro and in-vivo anti-inflammatory effect of oxyresveratrol from *Morus alba* L. Journal of Pharmacy and Pharmacology. 2003 Dec;55(12):1695-700. https://doi.org/10.1211/0022357022313

Chyad AH. Evaluation of anticancer, analgesic and anti-inflammatory activities of the ethanolic extract of *Lepidium draba* Linn. leaves. Adv. Anim. Vet. Sci. 2017;5(1):7-13.

Cilia R, Laguna J, Cassani E, Cereda E, Pozzi NG, Isaias IU, Contin M, Barichella M, Pezzoli G. *Mucuna pruriens* in Parkinson disease: A double-blind, randomized, controlled, crossover study. Neurology. 2017 Aug 1;89(5):432-8. https://doi.org/10.1212/WNL.0000000000004175

Cimanga K, Kambu K, Tona L, Apers S, De Bruyne T, Hermans N, Totté J, Pieters L, Vlietinck AJ. Correlation between chemical composition and antibacterial activity of essential oils of some aromatic medicinal plants growing in the Democratic Republic of Congo. Journal of ethnopharmacology. 2002 Feb 1;79(2):213-20. https://doi.org/10.1016/S0378-8741(01)00384-1

Çitoğlu GS, Altanlar N. Antimicrobial activity of some plants used in folk medicine. Journal of Faculty of Pharmacy of Ankara University. 2003;32(3):159-63.

Citraro R, Navarra M, Leo A, Donato Di Paola E, Santangelo E, Lippiello P, Aiello R, Russo E, De Sarro G. The anticonvulsant activity of a flavonoid-rich extract from orange juice involves both NMDA and GABA-benzodiazepine receptor complexes. Molecules. 2016 Sep;21(9):1261. https://doi.org/10.3390/molecules21091261

Clavin ML, Redko F, Acevedo C, Martino VS, Gorzalczany S. In Vivo Anti-inflammatory Activity and Flavonoid Identification in Medicinal *Eupatorium* Species.

Clement YN, Baksh-Comeau YS, Seaforth CE. An ethnobotanical survey of medicinal plants in Trinidad. Journal of ethnobiology and ethnomedicine. 2015 Dec 1;11(1):67. DOI 10.1186/s13002-015-0052-0

Collins SW, Martins X, Mitchell A, Teshome A, Arnason JT. Fataluku medicinal ethnobotany and the East Timorese military resistance. Journal of Ethnobiology and Ethnomedicine. 2007 Dec 1;3(1):5. https://doi.org/10.1186/1746-4269-3-5

Colmenares MG, Corredor MC. Evaluación del efecto sanitizante de un extracto biodegradable obtenido de la especie *Solanum marginatum*, de uso etnobotánico en Boyacá. Revista Luna Azul. 2011(32):10-5.

Compaore M, Bakasso S, Meda RN, Nacoulma OG. Antioxidant and Anti-Inflammatory Activities of Fractions from *Bidens engleri* OE Schulz (Asteraceae) and *Boerhavia erecta* L.(Nyctaginaceae). Medicines. 2018 Jun;5(2):53. https://doi.org/10.3390/medicines5020053

Conegundes JL, da Silva JM, de Freitas Mendes R, Fernandes MF, Pinto ND, de Almeida MA, Dib PR, de Oliveira Andrade R, Rodrigues MN, Castañon MC, Macedo GC. Anti-inflammatory and antinociceptive activity of *Siparuna guianensis* Aublet, an amazonian plant traditionally used by indigenous communities. Journal of Ethnopharmacology. 2020 Sep 3;265:113344. https://doi.org/10.1016/j.jep.2020.113344

Conforti F, Sosa S, Marrelli M, Menichini F, Statti GA, Uzunov D, Tubaro A, Menichini F, Della Loggia R. In vivo anti-inflammatory and in vitro antioxidant activities of Mediterranean dietary plants. Journal of ethnopharmacology. 2008 Feb 28;116(1):144-51. https://doi.org/10.1016/j.jep.2007.11.015

Coolborn AF, Bolatito B. Antibacterial and phytochemical evaluation of three medicinal plants. J Nat Prod. 2010;3:27-34.

Cordeiro KW, Felipe JL, Malange KF, do Prado PR, de Oliveira Figueiredo P, Garcez FR, de Cássia Freitas K, Garcez WS, Toffoli-Kadri MC. Anti-inflammatory and antinociceptive activities of *Croton urucurana* Baillon bark. Journal of ethnopharmacology. 2016 May 13;183:128-35. https://doi.org/10.1016/j.jep.2016.02.051

Córdova WH, Mesa LG, Hill AL, Lima CN, Lamas GD, Suárez MO, Domínguez RS. Antimicrobial activity of crude extracts and flavonoids from leaves of *Pluchea carolinensis* (Jacq.) G. Don. Pharmacologyonline. 2006;3:757-61.

Cordova-Guerrero I, Aragon-Martinez OH, Diaz-Rubio L, Franco-Cabrera S, Serafin-Higuera NA, Pozos-Guillen A, Soto-Castro TA, Martinez-Morales F, Isiordia-Espinoza M. Antibacterial and antifungal activity of *Salvia apiana* against clinically important microorganisms. Revista Argentina de microbiologia. 2016;48(3):217-21. doi: 10.1016/j.ram.2016.05.007

Cornejo A, Aguilar Sandoval F, Caballero L, Machuca L, Muñoz P, Caballero J, Perry G, Ardiles A, Areche C, Melo F. Rosmarinic acid prevents fibrillization and diminishes vibrational modes associated to β sheet in tau protein linked to Alzheimer's disease. Journal of enzyme inhibition and medicinal chemistry. 2017 Jan 1;32(1):945-53. https://doi.org/10.1080/14756366.2017.1347783

Corpas R, Griñán-Ferré C, Rodríguez-Farré E, Pallàs M, Sanfeliu C. Resveratrol Induces Brain Resilience Against Alzheimer Neurodegeneration Through Proteostasis Enhancement. Mol Neurobiol. 2019 Feb;56(2):1502-1516. doi: 10.1007/s12035-018-1157-y.

Cos P, Hermans N, De Bruyne T, Apers S, Sindambiwe JB, Berghe DV, Pieters L, Vlietinck AJ. Further evaluation of Rwandan medicinal plant extracts for their antimicrobial and antiviral activities. Journal of ethnopharmacology. 2002a Feb 1;79(2):155-63.

Cos P, Hermans N, De Bruyne T, Apers S, Sindambiwe JB, Witvrouw M, De Clercq E, Berghe DV, Pieters L, Vlietinck AJ. Antiviral activity of Rwandan medicinal plants against human immunodeficiency virus type-1 (HIV-1). Phytomedicine. 2002b Jan 1;9(1):62-8. https://doi.org/10.1078/0944-7113-00083

Costa GM, Chaves AC, Júnior LJ, de Paula VF, de Macedo GE, Oliveira MN, Queiroz RF. Antioxidant and antibacterial activities of the stem bark of *Aspidosperma spruceanum* Benth. International Journal of Advanced Engineering Research and Science. 2019 Nov 6;6(11). https://dx.doi.org/10.22161/ijaers.611.2

Costa RM, Vaz AF, Xavier HS, Correia MT, Carneiro-da-Cunha MG. Phytochemical screening of *Phthirusa pyrifolia* leaf extracts: Free-radical scavenging activities and environmental toxicity. South African Journal of Botany. 2015 Jul 1;99:132-7. doi.org/10.1016/j.sajb.2015.03.193

Costa SS, Corrêa MF, Casanova LM. A new triglycosyl flavonoid isolated from leaf juice of *Kalanchoe gastonis-bonnieri* (Crassulaceae). Natural product communications. 2015 Mar;10(3):1934578X1501000314.

Costa VB, Coube CS, Marinho BG, Matheus ME, Leitão SG, Fernandes PD. Anti-inflammatory and analgesic activity of *Bouchea fluminensis*. Fitoterapia. 2003 Jun 1;74(4):364-71. https://doi.org/10.1016/S0367-326X(03)00049-2

Coutinho PN, Pereira BP, Pereira AC, Porto ML, de Assis AL, Destefani AC, Meyrelles SS, Vasquez EC, Nogueira BV, de Andrade TU, Endringer DC. Chronic administration of antioxidant resin from *Virola oleifera* attenuates atherogenesis in LDLr-/-mice. Journal of ethnopharmacology. 2017 Jul 12;206:65-72. doi: 10.1016/j.jep.2017.05.015.

Croft KD, Tu'ipulotu P. A survey of Tongan medicinal plants. South Pacific Journal of Natural Science. 1980;1:45-57.

Cuevas-Rodríguez EO, Dia VP, Yousef GG, García-Saucedo PA, López-Medina J, Paredes-López O, Gonzalez de Mejia E, Lila MA. Inhibition of pro-inflammatory responses and antioxidant capacity of Mexican blackberry (*Rubus* spp.) extracts. Journal of agricultural and food chemistry. 2010 Aug 17;58(17):9542-8. https://doi.org/10.1021/jf102590p

Cui Y, Ao M, Hu J, Yu L. Anti-inflammatory activity of licochalcone A isolated from *Glycyrrhiza inflata*. Zeitschrift für Naturforschung C. 2008 Jun 1;63(5-6):361-5. https://doi.org/10.1515/znc-2008-5-609

Curimbaba TF, Almeida-Junior LD, Chagas AS, Quaglio AE, Herculano AM, Di Stasi LC. Prebiotic, antioxidant and anti-inflammatory properties of edible Amazon fruits. Food Bioscience. 2020 Apr 22:100599. https://doi.org/10.1016/j.fbio.2020.100599

Curini M, Cravotto G, Epifano F, Giannone G. Chemistry and biological activity of natural and synthetic prenyloxycoumarins. Current medicinal chemistry. 2006 Jan 1;13(2):199-222.

Currais A, Chiruta C, Goujon-Svrzic M, Costa G, Santos T, Batista MT, Paiva J, do Céu Madureira M, Maher P. Screening and identification of neuroprotective compounds relevant to Alzheimer′ s disease from medicinal plants of S. Tomé e Príncipe. Journal of ethnopharmacology. 2014 Aug 8;155(1):830-40.

Cvetnić Z, Vladimir-Knežević S. Antimicrobial activity of grapefruit seed and pulp ethanolic extract. Acta pharmaceutica. 2004 Sep 1;54(3):243-50.

Da Costa GA, Morais MG, Saldanha AA, Assis Silva IC, Aleixo ÁA, Ferreira JM, Soares AC, Duarte-Almeida JM, Lima LA. Antioxidant, antibacterial, cytotoxic, and anti-inflammatory potential of the leaves of *Solanum lycocarpum* A. St. Hil.(Solanaceae). Evidence-Based Complementary and Alternative Medicine. 2015 Jan 1;2015. https://doi.org/10.1155/2015/315987

Da Costa MP, Bozinis MC, Andrade WM, Costa CR, da Silva AL, de Oliveira CM, Kato L, Fernandes OD, Souza LK, Silva MD. Antifungal and cytotoxicity activities of the fresh xylem sap of *Hymenaea courbaril* L. and its major constituent fisetin. BMC complementary and alternative medicine. 2014 Dec;14(1):245.

da Silva AG, de Sousa CP, Koehler J, Fontana J, Christo AG, Guedes-Bruni RR. Evaluation of an extract of Brazilian arnica (*Solidago chilensis* Meyen, Asteraceae) in treating lumbago. Phytotherapy Research: An International Journal Devoted to Pharmacological and Toxicological Evaluation of Natural Product Derivatives. 2010 Feb;24(2):283-7. https://doi.org/10.1002/ptr.2934

da Silva ÉR, Salmazzo GR, da Silva Arrigo J, Oliveira RJ, Kassuya CA, Cardoso CA. Anti-inflammatory evaluation and toxicological analysis of *Campomanesia xanthocarpa* Berg. Inflammation. 2016 Aug 1;39(4):1462-8. doi: 10.1007/s10753-016-0378-3.

da Silveira Vasconcelos M, Gomes-Rochette NF, de Oliveira ML, Nunes-Pinheiro DC, Tomé AR, Maia de Sousa FY, Pinheiro FG, Moura CF, Miranda MR, Mota EF, de Melo DF. Anti-inflammatory and wound healing potential of cashew apple juice (*Anacardium occidentale* L.) in mice. Experimental Biology and Medicine. 2015 Dec;240(12):1648-55. https://doi.org/10.1177/1535370215576299

Dahech I, Farah W, Trigui M, Hssouna AB, Belghith H, Belghith KS, Abdallah FB. Antioxidant and antimicrobial activities of *Lycium shawii* fruits extract. International journal of biological macromolecules. 2013 Sep 1;60:328-33.

Dahiya SS, Sheoran SS, Sharma SK. Antibacterial activity of *Amaranthus hybridus* linn. root extracts. Int J Appl Biol Pharm Tech 2010;1:46-9.

Dah-Nouvlessounon DU, Adoukonou-Sagbadja HU, Diarrassouba NA, Sina HA, Noumavo PA, Baba-Moussa FA, Adjanohoun AD, Gbenou JD, Baba-Moussa LA. Antimicrobial, antioxidant, cytotoxic activities and phytochemical assessment of *Cola acuminata* used in Benin. Int. J. Pharm. Pharm. Sci. 2015;7:102-9.

Dahot MU. Antibacterial and antifungal activity of small protein of *Indigofera oblongifolia* leaves. Journal of ethnopharmacology. 1999 Mar 1;64(3):277-82. https://doi.org/10.1016/S0378-8741(98)00136-6

Daisy P, Eliza J, Farook KA. A novel dihydroxy gymnemic triacetate isolated from Gymnema sylvestre possessing normoglycemic and hypolipidemic activity on STZ-induced diabetic rats. Journal of ethnopharmacology. 2009 Nov 12;126(2):339-44. https://doi.org/10.1016/j.jep.2009.08.018

Dal Piaz F, Malafronte N, Romano A, Gallotta D, Belisario MA, Bifulco G, Gualtieri MJ, Sanogo R, De Tommasi N, Pisano C. Structural characterization of tetranortriterpenes from *Pseudrocedrela kotschyi* and *Trichilia emetica* and study of their activity towards the chaperone Hsp90. Phytochemistry. 2012 Mar 1;75:78-89.

Dal Piaz F, Vassallo A, Chini MG, et al. Natural iminosugar (+)-lentiginosine inhibits ATPase and chaperone activity of hsp90. PLoS One. 2012;7(8):e43316. doi:10.1371/journal.pone.0043316

Damianakos H, Kretschmer N, Sykłowska-Baranek K, Pietrosiuk A, Bauer R, Chinou I. Antimicrobial and cytotoxic isohexenylnaphthazarins from *Arnebia euchroma* (Royle) Jonst. (Boraginaceae) callus and cell suspension culture. Molecules. 2012 Dec 3;17(12):14310-22. doi: 10.3390/molecules171214310.

Dande PR, Talekar VS, Chakraborthy GS. Evaluation of crude saponins extract from leaves of *Sesbania sesban* (L.) Merr. for topical anti-inflammatory activity. International Journal of Research in Pharmaceutical Sciences. 2010 Jul 25;1(3):296-9.

Dangwal LR, Singh T. Ethno-Botanical study of some forest medicinal plants used by Gujjar tribe of district Rajouri (J&K), India. J&K Academy of Art, Culture and Languages, Srinagar, Jammu, India. 2013. 255-261.

Dangwal LR, Singh T. Ethno-Botanical study of some forest medicinal plants used by Gujjar tribe of district Rajouri (J&K), India. J&K Academy of Art, Culture and Languages, Srinagar, Jammu, India. 2013. 255-261.

Daniel AN, Sartoretto SM, Schmidt G, Caparroz-Assef SM, Bersani-Amado CA, Cuman RK. Anti-inflammatory and antinociceptive activities A of eugenol essential oil in experimental animal models. Revista Brasileira de Farmacognosia. 2009 Mar;19(1B):212-7.

Danz H, Stoyanova S, Wippich P, Brattström A, Hamburger M. Identification and isolation of the cyclooxygenase-2 inhibitory principle in *Isatis tinctoria*. Planta medica. 2001;67(05):411-6.

Dao TT, Nguyen PH, Won HK, Kim EH, Park J, Won BY, Oh WK. Curcuminoids from *Curcuma longa* and their inhibitory activities on influenza A neuraminidases. Food chemistry. 2012 Sep 1;134(1):21-8. https://doi.org/10.1016/j.foodchem.2012.02.015

Das P, Himaja M, Vinodhini V, Manimegalai S. Evaluation of anti-arthritic, HRBC membrane stabilization and antioxidant properties of the edible *Phlogacanthus thyrsiformis* (Hardow) Mabb flower extracts and their correlation studies. Studies on Ethno-Medicine. 2017 Jan 2;11(1):1-7. https://doi.org/10.1080/09735070.2017.1311692

Das S, Akhter R, Khandaker S, Huque S, Das P, Anwar MR, Tanni KA, Shabnaz S, Shahriar M. Phytochemical screening, antibacterial and anthelmintic activities of leaf and seed extracts of *Coix lacryma-jobi* L. Journal of Coastal Life Medicine. 2017;5(8):360-4.

Dash BK, Sen MK, Alam K, Hossain K, Islam R, Banu NA, Rahman S, Jamal AM. Antibacterial activity of *Nymphaea nouchali* (Burm. f) flower. Annals of clinical microbiology and antimicrobials. 2013 Dec;12(1):27. doi.org/10.1186/1476-0711-12-27

Dash GK, Murthy PN. Evaluation of *Argemone mexicana* Linn. Leaves for wound healing activity. J Nat Prod Plant Resour. 2011;1(1):46-56.

Datta T, Patra AK, Dastidar SG. Medicinal plants used by tribal population of Coochbehar district, West Bengal, India-an ethnobotanical survey. Asian Pac J Trop Biomed. 2014 May;4(Suppl 1):S478-82. doi: 10.12980/APJTB.4.2014C1122.

Davicino R, Mattar A, Casali Y, Anesini C, Micalizzi B. Different activities of *Schinus areira* L.: anti-inflammatory or pro-inflammatory effect. Immunopharmacology and immunotoxicology. 2010 Dec 1;32(4):620-7. https://doi.org/10.3109/08923971003657305

Davis BM, Pahlitzsch M, Guo L, Balendra S, Shah P, Ravindran N, Malaguarnera G, Sisa C, Shamsher E, Hamze H, Noor A. Topical curcumin nanocarriers are neuroprotective in eye disease. Scientific reports. 2018 Jul 23;8(1):1-3. doi:10.1038/s41598-018-29393-8

Dawilai S, Muangnoi C, Praengamthanachoti P, Tuntipopipat S. Anti-inflammatory activity of bioaccessible fraction from *Eryngium foetidum* leaves. BioMed research international. 2013;2013. http://dx.doi.org/10.1155/2013/958567

Dayalan Naidu S, Sutherland C, Zhang Y, Risco A, de la Vega L, Caunt CJ, Hastie CJ, Lamont DJ, Torrente L, Chowdhry S, Benjamin IJ, Keyse SM, Cuenda A, Dinkova-Kostova AT. Heat Shock Factor 1 Is a Substrate for p38 Mitogen-Activated Protein Kinases. Mol Cell Biol. 2016 Aug 26;36(18):2403-17. doi: 10.1128/MCB.00292-16.

de Alcantara BN, Kobayashi YT, Barroso KF, da Silva ID, de Almeida MB, Barbosa WL. Pharmacognostic analyses and evaluation of the in vitro antimicrobial activity of *Acmella oleracea* (L.) RK Jansen (Jambu) floral extract and fractions. Journal of Medicinal Plants Research. 2015 Jan 25;9(4):91-6.

De Almeida AP, Miranda MM, Simoni IC, Wigg MD, Lagrota MH, Costa SS. Flavonol monoglycosides isolated from the antiviral fractions of *Persea americana* (Lauraceae) leaf infusion. Phytotherapy Research: An International Journal Devoted to Pharmacological and Toxicological Evaluation of Natural Product Derivatives. 1998 Dec;12(8):562-7. https://doi.org/10.1002/(SICI)1099-1573(199812)12:8<562::AID-PTR356>3.0.CO;2-6

de Almeida EM, Marin AM, da Cunha MD, Fustinoni AM, de Sant'Ana LP, Arruda SF. Consumption of baru seeds [*Dipteryx alata* Vog.], a Brazilian savanna nut, prevents iron-induced oxidative stress in rats. Food Research International. 2012 Jan 1;45(1):427-33.

de Almeida ER, da Silva AR, Aragão-Neto AC, dos Santos Soares PH, da Silva LL, e Silva RP, Quixabeira DC, Botelho TL, Marinho LA. Anticonvulsant and anxiolytic assessment of leaves from *Artemisia vulgaris* L. in mice. J Med Plant Res. 2013;7:3325-31. doi: 10.5897/JMPR12.873

de Almeida GV, Arunachalam K, Balogun SO, Pavan E, Ascêncio SD, Soares IM, Zanatta AC, Vilegas W, Macho A, de Oliveira Martins DT. Chemical characterization and evaluation of gastric antiulcer properties of the hydroethanolic extract of the stem bark of *Virola elongata* (Benth.) Warb. Journal of ethnopharmacology. 2019 Mar 1;231:113-24. https://doi.org/10.1016/j.jep.2018.11.011

De Amorim VC, Júnior MS, da Silva AB, David JM, David JP, de Fátima Dias Costa M, Butt AM, Da Silva VD, Costa SL. Agathisflavone modulates astrocytic responses and increases the population of neurons in an in vitro model of traumatic brain injury. Naunyn-Schmiedeberg's Archives of Pharmacology. 2020 Oct;393:1921-30. https://doi.org/10.1007/s00210-020-01905-2

de Araujo JT, de Oliveira Pantoja F, Sá PS, Távora NP, Pinheiro AV, Trindade TB, Hoyos V, Pinto F, Lima CS, Barcessat AR, Pinheiro MT. Effect of Essential Oil of *Rosmarinus officinalis* L.(Rosemary) on the Healing of Cutaneous Lesions in Mice. Journal of Chemical and Pharmaceutical Research. 2017;9(5):381-6.

de Campos EP, Trombini LN, Rodrigues R, Portella DL, Werner AC, Ferraz MC, de Oliveira RV, Cogo JC, Oshima-Franco Y, Aranha N, Gerenutti M. Healing activity of *Casearia sylvestris* Sw. in second-degree scald burns in rodents. BMC research notes. 2015 Dec;8(1):269.

De Carvalho FG, Ovídio PP, Padovan GJ, Jordao Junior AA, Marchini JS, Navarro AM. Metabolic parameters of postmenopausal women after quinoa or corn flakes intake–a prospective and double-blind study. International journal of food sciences and nutrition. 2014 May 1;65(3):380-5.

de Castro Moreira ME, Pereira RG, Dias DF, Gontijo VS, Vilela FC, de Moraes GD, Giusti-Paiva A, dos Santos MH. Anti-inflammatory effect of aqueous extracts of roasted and green *Coffea arabica* L. Journal of functional foods. 2013 Jan 1;5(1):466-74. https://doi.org/10.1016/j.jff.2012.12.002

De Farias Freire SM, Da Silva Emim JA, Lapa AJ, Souccar C, Torres LM. Analgesic and antiinflammatory properties of *Scoparia dulcis* L. extracts and glutinol in rodents. Phytotherapy Research. 1993 Nov;7(6):408-14. https://doi.org/10.1002/ptr.2650070605

De las Heras B, Slowing K, Benedı J, Carretero E, Ortega T, Toledo C, Bermejo P, Iglesias I, Abad MJ, Gómez-Serranillos P, Liso PA. Antiinflammatory and antioxidant activity of plants used in traditional medicine in Ecuador. Journal of ethnopharmacology. 1998 Jun 1;61(2):161-6.

De Lima MR, de Souza Luna J, Dos Santos AF, De Andrade MC, Sant'Ana AE, Genet JP, Marquez B, Neuville L, Moreau N. Anti-bacterial activity of some Brazilian medicinal plants. Journal of ethnopharmacology. 2006 Apr 21;105(1-2):137-47

De M, De AK, Sen P, Banerjee AB. Antimicrobial properties of star anise (*Illicium verum* Hook f). Phytotherapy Research. 2002 Feb;16(1):94-5. https://doi.org/10.1002/ptr.989

De Miranda Pedroso TF, Bonamigo TR, da Silva J, Vasconcelos P, Félix JM, Cardoso CA, Souza RI, dos Santos AC, Volobuff CR, Formagio AS, Trichez VD. Chemical constituents of *Cochlospermum regium* (Schrank) Pilg. root and its antioxidant, antidiabetic, antiglycation, and anticholinesterase effects in Wistar rats. Biomedicine & Pharmacotherapy. 2019 Mar 1;111:1383-92. https://doi.org/10.1016/j.biopha.2019.01.005

De Moura RS, Emiliano AF, de Carvalho LC, Souza MA, Guedes DC, Tano T, Resende AC. Antihypertensive and endothelium-dependent vasodilator effects of *Alpinia zerumbet*, a medicinal plant. Journal of cardiovascular pharmacology. 2005 Sep 1;46(3):288-94.

De Oliveira AM, Mesquita MD, da Silva GC, de Oliveira Lima E, de Medeiros PL, Paiva PM, Souza IA, Napoleão TH. Evaluation of toxicity and antimicrobial activity of an ethanolic extract from leaves of *Morus alba* L.(Moraceae). Evidence-Based Complementary and Alternative Medicine. 2015 Jan 1;2015. https://doi.org/10.1155/2015/513978

De Oliveira RG, de Campos Castilho GR, da Cunha AL, Miyajima F, de Oliveira Martins DT. *Dilodendron bipinnatum* Radlk. inhibits pro-inflammatory mediators through the induction of MKP-1 and the down-regulation of MAPKp38/JNK/NF-κB pathways and COX-2 in LPS-activated RAW 264.7 cells. Journal of ethnopharmacology. 2017 Apr 18;202:127-37.

De Rose F, Marotta R, Talani G, Catelani T, Solari P, Poddighe S, Borghero G, Marrosu F, Sanna E, Kasture S, Acquas E. Differential effects of phytotherapic preparations in the hSOD1 *Drosophila melanogaster* model of ALS. Scientific reports. 2017 Jan 19;7(1):1-2.http://doi.org/10.1038/srep41059

De S, Ravishankar B, Bhavsar GC. Investigation of the anti-inflammatory effects of *Paederia foetida.* Journal of ethnopharmacology. 1994 Jun 1;43(1):31-8. doi.org/10.1016/0378-8741(94)90113-9

de Souza Almeida ES, Cechinel Filho V, Niero R, Clasen BK, Balogun SO, de Oliveira Martins DT. Pharmacological mechanisms underlying the anti-ulcer activity of methanol extract and canthin-6-one of *Simaba ferruginea* A. St-Hil. in animal models. Journal of ethnopharmacology. 2011 Apr 12;134(3):630-6. https://doi.org/10.1016/j.jep.2011.01.009

De Toledo CE, Britta EA, Ceole LF, Silva ER, De Mello JC, Dias Filho BP, Nakamura CV, Ueda-Nakamura T. Antimicrobial and cytotoxic activities of medicinal plants of the Brazilian cerrado, using Brazilian cachaça as extractor liquid. Journal of Ethnopharmacology. 2011 Jan 27;133(2):420-5. https://doi.org/10.1016/j.jep.2010.10.021

Deans SG, Svoboda KP. The antimicrobial properties of marjoram (*Origanum majorana* L.) volatile oil. Flavour and fragrance journal. 1990 Sep;5(3):187-90. https://doi.org/10.1002/ffj.2730050311

Deba F, Xuan TD, Yasuda M, Tawata S. Chemical composition and antioxidant, antibacterial and antifungal activities of the essential oils from *Bidens pilosa* Linn. var. Radiata. Food control. 2008 Apr 1;19(4):346-52.

Debnath T, Park PJ, Nath NC, Samad NB, Park HW, Lim BO. Antioxidant activity of *Gardenia jasminoides* Ellis fruit extracts. Food Chemistry. 2011 Oct 1;128(3):697-703. https://doi.org/10.1016/j.foodchem.2011.03.090

De Boni L, Watson AH, Zaccagnini L, Wallis A, Zhelcheska K, Kim N, Sanderson J, Jiang H, Martin E, Cantlon A, Rovere M. Brain region-specific susceptibility of Lewy body pathology in synucleinopathies is governed by α-synuclein conformations. Acta Neuropathologica. 2022 Apr;143(4):453-69. https://doi.org/10.1007/s00401-022-02406-7

Decha P, Kanokwan K, Jiraporn T, Pichaya J, Pisittawoot A. Phonopheresis Associated with Nanoparticle Gel from *Phyllanthus amarus* Relieves Pain by Reducing Oxidative Stress and Proinflammatory Markers in Adults with Knee Osteoarthritis. Chinese journal of integrative medicine. 2019 Sep 1;25(9):691-5. doi: 10.1007/s11655-019-3202-8

Déciga-Campos M, Palacios-Espinosa JF, Reyes-Ramírez A, Mata R. Antinociceptive and anti-inflammatory effects of compounds isolated from *Scaphyglottis livida* and *Maxillaria densa.* Journal of ethnopharmacology. 2007 Nov 1;114(2):161-8. https://doi.org/10.1016/j.jep.2007.07.021

Deeni YY, Hussain HS. Screening for antimicrobial activity and for alkaloids of *Nauclea latifolia*. Journal of ethnopharmacology. 1991 Oct 1;35(1):91-6.

Deep A, Rana P, Soni G. Iron Chelation and Iron Reducing Activity of Tissue Cultured and Tissue Culture Derived *Mentha* Spp. Journal of Applied Pharmaceutical Science. 2017 May;7(05):078-83. doi: 10.7324/JAPS.2017.70514

Deepak M, Handa SS. Antiinflammatory activity and chemical composition of extracts of *Verbena officinalis*. Phytotherapy Research: An International Journal Devoted to Pharmacological and Toxicological Evaluation of Natural Product Derivatives. 2000 Sep;14(6):463-5. doi: 10.1002/1099-1573(200009)14:63.0.CO;2-G

DeFilipps RA, Krupnick GA. The medicinal plants of Myanmar. PhytoKeys. 2018;(102):1-341. doi:10.3897/phytokeys.102.24380

Dehghanzadeh N, Ketabchi S, Alizadeh A. Essential oil composition and antibacterial activity of *Eryngium caeruleum* grown wild in Iran. Journal of Essential Oil Bearing Plants. 2014 May 4;17(3):486-92.

Dej-Adisai S, Phoopha S, Puripattanavong J. Phytochemical investigation and bioactivities of *Alternanthera ramosissima* (Mart.) Chodat and Hassl. Pharmacognosy Magazine. 2018 Jul 1;14(57):346.

del Carmen Recio M, Giner RM, Manez S, Gueho J, Julien HR, Hostettmann K, Rios JL. Investigations on the steroidal anti-inflammatory activity of triterpenoids from *Diospyros leucomelas*. Planta medica. 1995 Feb;61(01):9-12. doi: 10.1055/s-2006-957988

Delle Monache G, Botta B, Vinciguerra V, de Mello J, de Andrade Chiappeta A. Antimicrobial isoflavanones from *Desmodium canum*. Phytochemistry. 1996 Feb 1;41(2):537-44. https://doi.org/10.1016/0031-9422(95)00653-2

Delporte C, Backhouse N, Negrete R, Salinas P, Rivas P, Cassels BK, Feliciano AS. Antipyretic, hypothermic and antiinflammatory activities and metabolites from *Solanum ligustrinum* Lood. Phytotherapy Research: An International Journal Devoted to Pharmacological and Toxicological Evaluation of Natural Product Derivatives. 1998 Mar;12(2):118-22.

Deng D, Lauren DR, Cooney JM, Jensen DJ, Wurms KV, Upritchard JE, Cannon RD, Wang MZ, Li MZ. Antifungal saponins from *Paris polyphylla* Smith. Planta medica. 2008 Sep;74(11):1397-402. doi: 10.1055/s-2008-1081345

Deng LL, Yuan D, Zhou ZY, Wan JZ, Zhang CC, Liu CQ, Dun YY, Zhao HX, Zhao B, Yang YJ, Wang T. Saponins from *Panax japonicus* attenuate age-related neuroinflammation via regulation of the mitogen-activated protein kinase and nuclear factor kappa B signaling pathways. Neural regeneration research. 2017 Nov;12(11):1877. doi: 10.4103/1673-5374.219047

Deodato F, Procopio E, Rampazzo A, Taurisano R, Donati MA, Dionisi-Vici C, Caciotti A, Morrone A, Scarpa M. The treatment of juvenile/adult GM1-gangliosidosis with Miglustat may reverse disease progression. Metab Brain Dis. 2017 Oct;32(5):1529-1536. doi: 10.1007/s11011-017-0044-y. Epub 2017 Jun 3.

Derita MG, Leiva ML, Zacchino SA. Influence of plant part, season of collection and content of the main active constituent, on the antifungal properties of *Polygonum acuminatum* Kunth. Journal of ethnopharmacology. 2009 Jul 30;124(3):377-83. https://doi.org/10.1016/j.jep.2009.05.029

Desideri G, Kwik-Uribe C, Grassi D, Necozione S, Ghiadoni L, Mastroiacovo D, Raffaele A, Ferri L, Bocale R, Lechiara MC, Marini C. Benefits in cognitive function, blood pressure, and insulin resistance through cocoa flavanol consumption in elderly subjects with mild cognitive impairment: the Cocoa, Cognition, and Aging (CoCoA) study. Hypertension. 2012 Sep;60(3):794-801. https://doi.org/10.1161/HYPERTENSIONAHA.112.193060

Devi BP, Boominathan R, Mandal SC. Anti-inflammatory, analgesic and antipyretic properties of *Clitoria ternatea* root. Fitoterapia. 2003 Jun 1;74(4):345-9. doi.org/10.1016/S0367-326X(03)00057-1

Devi P, Meera R. Study of antioxdant, antiinflammatory and woundhealing activity of extracts of *Litsea glutinosa*. Journal of Pharmaceutical Sciences and Research. 2010 Mar 1;2(3):155.

Dewanjee S, Maiti A, Majumdar R, Majumdar A, Mandal SC. Evaluation of antimicrobial activity of hydroalcoholic extract *Schima wallichii* bark. Pharmcolology online. 2008;1:523-8.

Dewanjee S, Maiti A, Sahu R, Dua TK, Mandal SC. Study of anti-inflammatory and antinociceptive activity of hydroalcoholic extract of *Schima wallichii* bark. Pharmaceutical Biology. 2009 May 1;47(5):402-7. https://doi.org/10.1080/13880200902758824

Dhadde SB, Nagakannan P, Roopesh M, Kumar SA, Thippeswamy BS, Veerapur VP, Badami S. Effect of embelin against 3-nitropropionic acid-induced Huntington's disease in rats. Biomedicine & Pharmacotherapy. 2016 Feb 1;77:52-8. doi.org/10.1016/j.biopha.2015.11.009

Dhanasekaran M, Holcomb LA, Hitt AR, Tharakan B, Porter JW, Young KA, Manyam BV. Centella asiatica extract selectively decreases amyloid β levels in hippocampus of Alzheimer's disease animal model. Phytotherapy Research: An International Journal Devoted to Pharmacological and Toxicological Evaluation of Natural Product Derivatives. 2009 Jan;23(1):14-9. https://doi.org/10.1002/ptr.2405

Dhar R, Kimseng R, Chokchaisiri R, Hiransai P, Utaipan T, Suksamrarn A, Chunglok W. 2′, 4-Dihydroxy-3′, 4′, 6′-trimethoxychalcone from *Chromolaena odorata* possesses anti-inflammatory effects via inhibition of NF-κB and p38 MAPK in lipopolysaccharide-activated RAW 264.7 macrophages. Immunopharmacology and immunotoxicology. 2018 Jan 2;40(1):43-51. doi: 10.1080/08923973.2017.1405437.

Dhingra D, Goyal PK. Evidences for the Involvement of Monoaminergic and GABAergic Systems in Antidepressant-like Activity of *Tinospora cordifolia* in Mice. *Indian J Pharm Sci*. 2008;70(6):761-767. doi:10.4103/0250-474X.49118

Dhingra D, Joshi P. Antidepressant-like activity of *Benincasa hispida* fruits in mice: Possible involvement of monoaminergic and GABAergic systems. J Pharmacol Pharmacother. 2012;3(1):60–62. doi:10.4103/0976-500X.92521

Dhole JA, Dhole NA, Lone KD, Bodke SS, Dhole JA. Preliminary phytochemical analysis and antimicrobial activity of some weeds collected from Marathwada region. Journal of research in Biology. 2011;1:19-23.

Dhouafli Z, Rigacci S, Leri M, Bucciantini M, Mahjoub B, Tounsi MS, Wannes WA, Stefani M, Hayouni EA. Screening for amyloid-β aggregation inhibitor and neuronal toxicity of eight Tunisian medicinal plants. Industrial Crops and Products. 2018 Jan 1;111:823-33. https://doi.org/10.1016/j.indcrop.2017.11.045

Di Sanzo P, De Martino L, Mancini E, De Feo V. Medicinal and useful plants in the tradition of Rotonda, Pollino National Park, Southern Italy. Journal of Ethnobiology and Ethnomedicine. 2013 Dec 1;9(1):19. ttps://doi.org/10.1186/1746-4269-9-19

Diaconu C, Vlase L, Cuciureanu M, Filip L. Assessment of flavonoids content in citrus juices using a LC/MS method. Farmacia. 2017 Jan 1;65(1):92-6.

Dias FM, Leffa DD, Daumann F, de Oliveira Marques S, Luciano TF, Possato JC, de Santana AA, Neves RX, Rosa JC, Oyama LM, Rodrigues B. Acerola (*Malpighia emarginata* DC.) juice intake protects against alterations to proteins involved in inflammatory and lipolysis pathways in the adipose tissue of obese mice fed a cafeteria diet. Lipids in health and disease. 2014 Dec;13(1):24.

Dib I, Tits M, Angenot L, Wauters JN, Assaidi A, Mekhfi H, Aziz M, Bnouham M, Legssyer A, Frederich M, Ziyyat A. Antihypertensive and vasorelaxant effects of aqueous extract of *Artemisia campestris* L. from Eastern Morocco. J Ethnopharmacol. 2017 Jul 12;206:224-235. doi: 10.1016/j.jep.2017.05.036.

Diba F, Yusro F, Mariani Y, Ohtani K. Inventory and biodiversity of medicinal plants from tropical rain forest based on traditional knowledge by ethnic dayaknese communities in West Kalimantan Indonesia. Kuroshio Science 2013;7(1), 75-80.

Dickson RA, Ekuadzi E, Annan K, Komlaga G. Antibacterial, anti-inflammatory, and antioxidant effects of the leaves and stem bark of *Glyphaea brevis* (Spreng) Monachino (Tiliaceae): A comparative study. *Pharmacognosy Res*. 2011;3(3):166–172. doi:10.4103/0974-8490.85001

Dimech GS, Soares LA, Ferreira MA, de Oliveira AG, Carvalho MD, Ximenes EA. Phytochemical and antibacterial investigations of the extracts and fractions from the stem bark of *Hymenaea stigonocarpa* Mart. ex Hayne and effect on ultrastructure of *Staphylococcus aureus* induced by hydroalcoholic extract. The Scientific World Journal. 2013;2013. http://dx.doi.org/10.1155/2013/862763

Dimo T, Fotio AL, Nguelefack TB, Asongalem EA, Kamtchouing P. Antiinflammatory activity of leaf extracts of *Kalanchoe crenata* Andr. Indian Journal of Pharmacology. 2006 Mar 1;38(2):115. doi: 10.4103/0253-7613.24617

Dimo T, Rakotonirina SV, Tan PV, Azay J, Dongo E, Cros G. Leaf methanol extract of *Bidens pilosa* prevents and attenuates the hypertension induced by high-fructose diet in Wistar rats. Journal of ethnopharmacology. 2002 Dec 1;83(3):183-91. https://doi.org/10.1016/S0378-8741(02)00162-9

Diomede L, Rigacci S, Romeo M, Stefani M, Salmona M. Oleuropein aglycone protects transgenic *C. elegans* strains expressing Aβ42 by reducing plaque load and motor deficit. PLoS one. 2013 Mar 8;8(3):e58893.

Divakar MC, Al-Siyabi A, Varghese SS, Rubaie MA. The Practice of Ethnomedicine in the Northern and Southern Provinces of Oman. Oman Medical Journal. 2016;31(4):245-252. doi:10.5001/omj.2016.49.

Diwan PV, Singh AK. Antiinflammatory activity of '*Soymida febrifuga*'(*Mansa rohini*) in rats and mice. Phytotherapy research. 1993 May;7(3):255-6.

Djakpo O, Yao W. *Rhus chinensis* and *Galla chinensis*--folklore to modern evidence: review. Phytother Res. 2010 Dec;24(12):1739-47. doi: 10.1002/ptr.3215.

Djemgou PC, Gatsing D, Hegazy ME, Mohamed AH, Ngandeu F, Tane P, Ngadjui BT, Fotso S, Laatsch H. Turrealabdane, turreanone and an antisalmonellal agent from *Turraeanthus africanus*. Planta medica. 2010 Jan;76(02):165-71.

Djipa CD, Delmée M, Quetin-Leclercq J. Antimicrobial activity of bark extracts of *Syzygium jambos* (L.) Alston (Myrtaceae). Journal of Ethnopharmacology. 2000 Jul 1;71(1-2):307-13. https://doi.org/10.1016/S0378-8741(99)00186-5

Do Monte FH, dos Santos Jr JG, Russi M, Lanziotti VM, Leal LK, de Andrade Cunha GM. Antinociceptive and anti-inflammatory properties of the hydroalcoholic extract of stems from *Equisetum arvense* L. in mice. Pharmacological research. 2004 Mar 1;49(3):239-43. https://doi.org/10.1016/j.phrs.2003.10.002

Do Santos RC, de Souza AV, Andrade-Silva M, Kassuya CA, Cardoso CA, do Carmo Vieira M, Formagio AS. Antioxidant, anti-rheumatic and anti-inflammatory investigation of extract and dicentrinone from *Duguetia furfuracea* (A. St.-Hil.) Benth. & Hook. f. Journal of ethnopharmacology. 2018 Jan 30;211:9-16. https://doi.org/10.1016/j.jep.2017.09.019

Dogani M, Askari N, Kalantari-Hesari A, Rahbar FH. The effects of *P. atlantica* as a libido booster and sexual enhancer on the reproductive system of male rats. Journal of Traditional and Complementary Medicine. 2021 Sep 25. https://doi.org/10.1016/j.jtcme.2021.09.007

Domingos OD, Alcântara BG, Santos MF, Maiolini T, Dias DF, Baldim JL, Lago JH, Soares MG, Chagas-Paula DA. Anti-Inflammatory Derivatives with Dual Mechanism of Action from the Metabolomic Screening of *Poincianella pluviosa*. Molecules. 2019 Jan;24(23):4375. https://doi.org/10.3390/molecules24234375

Domingues A, Sartori A, Valente LM, Golim MA, Siani AC, Viero RM. *Uncaria tomentosa* Aqueous-ethanol Extract Triggers an Immunomodulation toward a Th2 Cytokine Profile. Phytotherapy Research. 2011 Aug;25(8):1229-35. doi: 10.1002/ptr.3549.

Dongmo PJ, Tchoumbougnang F, Boyom FF, Sonwa ET, Zollo PA, Menut C. Antiradical, antioxidant activities and anti-inflammatory potential of the essential oils of the varieties of *Citrus limon* and *Citrus aurantifolia* growing in Cameroon. Journal of Asian Scientific Research. 2013;3(10):1046-57.

Dorjsembe B, Lee HJ, Kim M, Dulamjav B, Jigjid T, Nho CW. *Achillea asiatica* extract and its active compounds induce cutaneous wound healing. Journal of ethnopharmacology. 2017 Jul 12;206:306-14. https://doi.org/10.1016/j.jep.2017.06.006

Dorsch W, Schneider E, Bayer T, Breu W, Wagner H. Anti-inflammatory effects of onions: inhibition of chemotaxis of human polymorphonuclear leukocytes by thiosulfinates and cepaenes. International Archives of Allergy and Immunology. 1990;92(1):39-42.

Doss A, An SP. Antimicrobial activity of *Hygrophila auriculata* (Schumach.) Heine and *Pergularia daemia* Linn. African Journal of Plant Science. 2013 Apr 30;7(4):137-42. https://doi.org/10.5897/AJPS12.193

Dou Z, Rong X, Zhao E, Zhang L, Lv Y. Neuroprotection of Resveratrol Against Focal Cerebral Ischemia/Reperfusion Injury in Mice Through a Mechanism Targeting Gut-Brain Axis. Cell Mol Neurobiol. 2019 Aug;39(6):883-898. doi: 10.1007/s10571-019-00687-3.

Doughari JH, Elmahmood AM, Manzara S. Studies on the antibacterial activity of root extracts of *Carica papaya* L. African Journal of Microbiology Research. 2007 Aug 1;1(3):037-41.

Doughari JH, Okafor B. Antimicrobial Activity of *Senna alata* Linn. East and Central African Journal of Pharmaceutical Sciences. 2007;10(1):17-21.

Doughari JH. Antimicrobial activity of *Tamarindus indica* Linn. Tropical Journal of Pharmaceutical Research. 2006;5(2):597-603. doi: 10.4314/tjpr.v5i2.14637

Dougnon VT, Klotoé JR, Sènou M, Roko GO, Dougnon G, Fabiyi K, Amadou A, Aniambossou A, Assogba P, Bankolé H, Dougnon J. Chemical Composition, Cytotoxicity, and Antibacterial Activity of Selected Extracts of *Euphorbia hirta*, *Citrus aurantifolia*, and *Heterotis rotundifolia* on Enteropathogenic Bacteria. EC Microbiology. 2017;12(2):180-95.

Doukkali Z, Kamal R, El Jemelif M, Nadjmouddine M, Zellou A, Cherrah Y, Alaoui K, Taghzouti K. Anti-Anxiety Effects of *Mercurialis annua* Aqueous Extract in the Elevated Plus Maze Test. Pharmaceutical Bioprocessing. 2016;4(4):56-61.

Drozdova IL, Bubenchikov RA. Composition and antiinflammatory activity of polysaccharide complexes extracted from sweet violet and low mallow. Pharmaceutical Chemistry Journal. 2005 Apr 1;39(4):197-200. https://doi.org/10.1007/s11094-005-0116-x

Du J, He ZD, Jiang RW, Ye WC, Xu HX, But PP. Antiviral flavonoids from the root bark of *Morus alba* L. Phytochemistry. 2003 Apr 1;62(8):1235-8. https://doi.org/10.1016/S0031-9422(02)00753-7

Du WJ, Guo JJ, Gao MT, Hu SQ, Dong XY, Han YF, Liu FF, Jiang S, Sun Y. Brazilin inhibits amyloid β-protein fibrillogenesis, remodels amyloid fibrils and reduces amyloid cytotoxicity. Scientific reports. 2015 Jan 23;5:7992. https://doi.org/10.1038/srep07992

Du Y, Qu J, Zhang W, Bai M, Zhou Q, Zhang Z, Li Z, Miao J. Morin reverses neuropathological and cognitive impairments in APPswe/PS1dE9 mice by targeting multiple pathogenic mechanisms. Neuropharmacology. 2016 Sep 1;108:1-3. https://doi.org/10.1016/j.neuropharm.2016.04.008

Dulla O, Jahan FI. Ethnopharmacological survey on traditional medicinal plants at Kalaroa Upazila, Satkhira District, Khulna Division, Bangladesh. Journal of Intercultural Ethnopharmacology. 2017;6(3):316-325. doi:10.5455/jice.20170719010256.

Duman E, Şimşek M, Özcan MM. Monitoring of composition and antimicrobial activity of fig (*Ficus carica* L.) fruit and seed oil. J. Agric. Processes Techol. 2018;24(2):75-80.

Duraipandiyan V, Ignacimuthu S. Antibacterial and antifungal activity of Flindersine isolated from the traditional medicinal plant, *Toddalia asiatica* (L.) Lam. Journal of ethnopharmacology. 2009 Jun 25;123(3):494-8. https://doi.org/10.1016/j.jep.2009.02.020

Durairajan SS, Liu LF, Lu JH, Chen LL, Yuan Q, Chung SK, Huang L, Li XS, Huang JD, Li M. Berberine ameliorates β-amyloid pathology, gliosis, and cognitive impairment in an Alzheimer's disease transgenic mouse model. Neurobiology of Aging. 2012 Dec 1;33(12):2903-19. https://doi.org/10.1016/j.neurobiolaging.2012.02.016

Duran NI, Kaya AL, Gulbol Duran G, Eryilmaz NA. In vitro antiviral effect of the essential oils of *Thymbra spicata* L. on Herpes simplex virus type 2. ICAMS 2012 – 4th International Conference on Advanced Materials and Systems 2012. .

Durand E, Ellington EV, Feng PC, Haynes LJ, Magnus KE, Philip N. Simple hypotensive and hypertensive principles from some West Indian medicinal plants. Journal of Pharmacy and Pharmacology. 1962 Sep;14(1):562-6. https://doi.org/10.1111/j.2042-7158.1962.tb11140.x

Duru CM, Onyedineke NE. In vitro study on the antimicrobial activity and phytochemical analysis of ethanolic extracts of the mesocarp of *Voacanga africana*. American Journal of Plant Physiology. 2010 Jul;5(4):163-9. ]

Dutta K, Patel P, Rahimian R, Phaneuf D, Julien JP. *Withania somnifera* Reverses Transactive Response DNA Binding Protein 43 Proteinopathy in a Mouse Model of Amyotrophic Lateral Sclerosis/Frontotemporal Lobar Degeneration. Neurotherapeutics. 2017 Apr;14(2):447-462. doi: 10.1007/s13311-016-0499-2.

Duwiejua M, Woode E, Obiri DD. Pseudo-akuammigine, an alkaloid from *Picralima nitida* seeds, has anti-inflammatory and analgesic actions in rats. Journal of ethnopharmacology. 2002 Jun 1;81(1):73-9. https://doi.org/10.1016/S0378-8741(02)00058-2

Duwiejua M, Zeitlin IJ, Waterman PG, Gray AI. Anti-inflammatory Activity of *Polygonum bistorta*, *Guaiacum officinale* and in Rats. Journal of pharmacy and pharmacology. 1994 Apr;46(4):286-90. doi.org/10.1111/j.2042-7158.1994.tb03795.x

Dwivedi D, Dwivedi M, Malviya S, Singh V. Evaluation of wound healing, anti-microbial and antioxidant potential of *Pongamia pinnata* in wistar rats. Journal of traditional and complementary medicine. 2017 Jan 1;7(1):79-85.

Dwivedi S, Agarwal MP. Antianginal and cardioprotective effects of *Terminalia arjuna*, an indigenous drug, in coronary artery disease. J Assoc Physicians India. 1994 Apr;42(4):287-9.

Dzotam JK, Touani FK, Kuete V. Antibacterial activities of the methanol extracts of *Canarium schweinfurthii* and four other Cameroonian dietary plants against multi-drug resistant Gram-negative bacteria. Saudi journal of biological sciences. 2016 Sep 1;23(5):565-70. https://doi.org/10.1016/j.sjbs.2015.06.006

Dzoyem JP, Eloff JN. Anti-inflammatory, anticholinesterase and antioxidant activity of leaf extracts of twelve plants used traditionally to alleviate pain and inflammation in South Africa. Journal of Ethnopharmacology. 2015 Feb 3;160:194-201. https://doi.org/10.1016/j.jep.2014.11.034

Dzoyem JP, Melong R, Tsamo AT, Tchinda AT, Kapche DG, Ngadjui BT, McGaw LJ, Eloff JN. Cytotoxicity, antimicrobial and antioxidant activity of eight compounds isolated from *Entada abyssinica* (Fabaceae). BMC research notes. 2017 Dec 1;10(1):118. https://doi.org/10.1186/s13104-017-2441-z

Eakwaropas P, Ngawhirunpat T, Rojanarata T, Akkaramongkolporn P, Opanasopit P, Patrojanasophon P. Fabrication of electrospun hydrogels loaded with *Ipomoea pes-caprae* (L.) R. Br extract for infected wound. Journal of Drug Delivery Science and Technology. 2020 Feb 1;55:101478. https://doi.org/10.1016/j.jddst.2019.101478

Eddine LS, Segni L, Ridha OM. In vitro assays of the antibacterial and antioxidant properties of extracts from *Asphodelus tenuifolius* Cav and its main constituents: a comparative study. Int J Pharm Clin Res. 2015;7(2):119-25.

Edwin S, Jarald EE, Deb L, Jain A, Kinger H, Dutt KR, Raj AA. Wound healing and antioxidant activity of *Achyranthes aspera*. Pharmaceutical biology. 2008 Jan 1;46(12):824-8. https://doi.org/10.1080/13880200802366645

Efstratiou E, Hussain AI, Nigam PS, Moore JE, Ayub MA, Rao JR. Antimicrobial activity of *Calendula officinalis* petal extracts against fungi, as well as Gram-negative and Gram-positive clinical pathogens. Complementary Therapies in Clinical Practice. 2012 Aug 1;18(3):173-6. https://doi.org/10.1016/j.ctcp.2012.02.003

Ehrnhoefer DE, Duennwald M, Markovic P, Wacker JL, Engemann S, Roark M, Legleiter J, Marsh JL, Thompson LM, Lindquist S, Muchowski PJ. Green tea (−)-epigallocatechin-gallate modulates early events in huntingtin misfolding and reduces toxicity in Huntington's disease models. Human molecular genetics. 2006 Sep 15;15(18):2743-51. https://doi.org/10.1093/hmg/ddl210

El Azhary K, Jouti NT, El Khachibi M, Moutia M, Tabyaoui I, El Hou A, Achtak H, Nadifi S, Habti N, Badou A. Anti-inflammatory potential of *Capparis spinosa* L. in vivo in mice through inhibition of cell infiltration and cytokine gene expression. BMC complementary and alternative medicine. 2017 Dec;17(1):1-2.

Eldeen IM, Elgorashi EE, Van Staden J. Antibacterial, anti-inflammatory, anti-cholinesterase and mutagenic effects of extracts obtained from some trees used in South African traditional medicine. Journal of Ethnopharmacology. 2005 Dec 1;102(3):457-64. https://doi.org/10.1016/j.jep.2005.08.049

Eldeen IM, Van Staden J. Cyclooxygenase inhibition and antimycobacterial effects of extracts from Sudanese medicinal plants. South African Journal of Botany. 2008 Apr 1;74(2):225-9. https://doi.org/10.1016/j.sajb.2007.11.009

El-Desoky AH, Abdel-Rahman RF, Ahmed OK, El-Beltagi HS, Hattori M. Anti-inflammatory and antioxidant activities of naringin isolated from *Carissa carandas* L.: In vitro and in vivo evidence. Phytomedicine. 2018 Mar 15;42:126-34.

Eldhose B, Notario V, Latha MS. Evaluation of phytochemical constituents and In vitro antioxidant activities of *Plumbago indica* root extracts. Journal of Pharmacognosy and Phytochemistry. 2013 Nov 1;2(4).

Elekofehinti OO, Kamdem JP, Meinerz DF, Kade IJ, Adanlawo IG, Rocha JB. Saponin from the fruit of *Solanum anguivi* protects against oxidative damage mediated by Fe 2+ and sodium nitroprusside in rat brain synaptosome P2 fraction. Archives of pharmacal research. 2015 Jul 10:1-7. https://doi.org/10.1007/s12272-014-0536-9

El-Fiky F, Asres K, Gibbons S, Hammoda H, Badr J, Umer S. Phytochemical and antimicrobial investigation of latex from *Euphorbia abyssinica* Gmel. Natural Product Communications. 2008 Sep;3(9):1934578X0800300922.

El-Ghazali GE, Al-Khalifa KS, Saleem GA, Abdallah EM. Traditional medicinal plants indigenous to Al-Rass province, Saudi Arabia. Journal of Medicinal Plants Research. 2010 Dec 18;4(24):2680-3. doi: 10.5897/JMPR09.556

El-Ghorab AH, Nauman M, Anjum FM, Hussain S, Nadeem M. A comparative study on chemical composition and antioxidant activity of ginger (*Zingiber officinale*) and cumin (*Cuminum cyminum*). Journal of agricultural and food chemistry. 2010 Jul 28;58(14):8231-7. https://doi.org/10.1021/jf101202x

Elgin G, Yavaşoğlu NÜ, Öztürk B. Antimicrobial activity of endemic *Ziziphora taurica* subsp. cleonioides (Boiss) PH Davis essential oil. Acta Pharmaceutica Sciencia. 2006;48(1).

El-Hilaly J, Hmammouchi M, Lyoussi B. Ethnobotanical studies and economic evaluation of medicinal plants in Taounate province (Northern Morocco). J Ethnopharmacol. 2003 Jun;86(2-3):149-58. doi: 10.1016/s0378-8741(03)00012-6.

Elisha IL, Dzoyem JP, McGaw LJ, Botha FS, Eloff JN. The anti-arthritic, anti-inflammatory, antioxidant activity and relationships with total phenolics and total flavonoids of nine South African plants used traditionally to treat arthritis. *BMC* Complement Altern Med. 2016;16(1):307. Published 2016 Aug 23. doi:10.1186/s12906-016-1301-z AMA

El-Maati MF, Mahgoub SA, Labib SM, Al-Gaby AM, Ramadan MF. Phenolic extracts of clove (*Syzygium aromaticum*) with novel antioxidant and antibacterial activities. European Journal of Integrative Medicine. 2016 Aug 1;8(4):494-504. https://doi.org/10.1016/j.eujim.2016.02.006

Elmastas M, Ozturk L, Gokce I, Erenler R, Aboul-Enein HY. Determination of antioxidant activity of marshmallow flower (*Althaea officinalis* L.). Analytical letters. 2004 Dec 28;37(9):1859-69. https://doi.org/10.1081/AL-120039431

El-Mekkawy S, Meselhy MR, Nakamura N, Hattori M, Kawahata T, Otake T. Anti-HIV-1 phorbol esters from the seeds of *Croton tiglium*. Phytochemistry. 2000 Feb 9;53(4):457-64. https://doi.org/10.1016/S0031-9422(99)00556-7

Elmezogi J, Zetrini A, Ben-Hussein G, Anwair M, Gbaj A, El-Ashheb M, Nahar L, Sarker SD. Evaluation of anti-inflammatory activity of some Libyan medicinal plants in experimental animals. Archives of Biological Sciences. 2012;64(3):1059-63. doi: 10.2298/ABS1203059E

El-Mosallamy AE, Sleem AA, Abdel-Salam OM, Shaffie N, Kenawy SA. Antihypertensive and cardioprotective effects of pumpkin seed oil. Journal of medicinal food. 2012 Feb 1;15(2):180-9. https://doi.org/10.1089/jmf.2010.0299

Eloff JN. The antibacterial activity of 27 southern African members of the Combretaceae. South African Journal of Science. 1999 Mar 1;95(3):148-52.

El-Ouady F, Eddouks M. Asteriscus graveolens exhibits Antihypertensive Activity through Activation of Vascular KATP Channels Activation in Rats. Endocrine, Metabolic & Immune Disorders-Drug Targets (Formerly Current Drug Targets-Immune, Endocrine & Metabolic Disorders). 2020 Jun 1;20(5):736-44. https://doi.org/10.2174/1871530319666191016100851

El-Serehy HA, Al-Rasheid KA, Al-Misned F, Mortuza G. *Citrus sinensis* Peel Extract Induced In vitro Effects on *Herpes simplex* Virus. Journal of Pure and Applied Microbiology. 2014 Oct;8(5):3807-12.

El-Shanawany MA, Sayed HM, Ibrahim SR, Fayed MA. Chemical constituents, anti-inflammatory, and antioxidant activities of *Anisotes trisulcus*. Bulletin of Faculty of Pharmacy, Cairo University. 2014 Jun 1;52(1):9-14. https://doi.org/10.1016/j.bfopcu.2014.02.004

El-Shanawany MA, Sayed HM, Ibrahim SR, Fayed MA. Stigmasterol tetracosanoate, a new stigmasterol ester from the Egyptian *Blepharis ciliaris*. Drug research. 2015 Jul;65(07):347-53. doi: 10.1055/s-0034-1382064

El-Tantawy WH. Biochemical effects, hypolipidemic and anti-inflammatory activities of *Artemisia vulgaris* extract in hypercholesterolemic rats. Journal of clinical biochemistry and nutrition. 2015:14-1.

Elufioye TO, Hameed HA. Cognitive-enhancing properties of *Morinda lucida* (Rubiaceae) and *Peltophorum pterocarpum* (Fabaceae) in scopolamine-induced amnesic mice. African Journal of Traditional, Complementary and Alternative Medicines. 2017;14(3):136-41.

Elufioye TO, Machie SC. Anticholinesterase activities of methanol extract and partitioned fractions of *Acanthospermum hispidum* DC. Nigerian Journal of Natural Products and Medicine. 2016;20:67-72.

Elufioye TO, Oladele AT, Cyril-Olutayo CM, Agbedahunsi JM, Adesanya SA. Ethnomedicinal study and screening of plants used for memory enhancement and antiaging in Sagamu, Nigeria. European Journal of Medicinal Plants. 2012 Jul 13:262-75. https://doi.org/10.9734/EJMP/2012/1372

Ene-Obong H, Onuoha N, Aburime L, Mbah O. Chemical composition and antioxidant activities of some indigenous spices consumed in Nigeria. Food chemistry. 2018 Jan 1;238:58-64.

Epifano F, Genovese S, Fiorito S, Loggia RD, Tubaro A, Sosa S. In vivo anti-inflammatory activity of some naturally occurring O-and N-prenyl secondary metabolites. Natural product communications. 2014 Jan;9(1):1934578X1400900125.

Erasto P, Lubschagne A, Mbwambo ZH, Nondo RS, Lall N. Antimycobacterial, antioxidant activity and toxicity of extracts from the roots of *Rauvolfia vomitoria* and *R. caffra*. Spatula DD. 2011; 1(2): 73-80. doi: 10.5455/spatula.20110514043359

Erazo S, García R, Backhouse N, Lemus I, Delporte C, Andrade C. Phytochemical and biological study of Radal *Lomatia hirsuta* (Proteaceae). Journal of ethnopharmacology. 1997a Jul 1;57(2):81-3.

Erazo S, González V, Zaldivar M, Negrete R. Antimicrobial activity of *Psoralea glandulosa* L. International journal of pharmacognosy. 1997b Jan 1;35(5):385-7.

Eren E, Tufekci KU, Isci KB, Tastan B, Genc K, Genc S. Sulforaphane inhibits lipopolysaccharide-induced inflammation, cytotoxicity, oxidative stress, and miR-155 expression and switches to Mox phenotype through activating extracellular signal-regulated kinase 1/2–nuclear factor erythroid 2-related factor 2/antioxidant response element pathway in murine microglial cells. Frontiers in immunology. 2018 Jan 23;9:36. https://doi.org/10.3389/fimmu.2018.00036

Esimone CO, Nworu CS, Jackson CL. Cutaneous wound healing activity of a herbal ointment containing the leaf extract of *Jatropha curcas* L.(Euphorbiaceae). Int J Appl Res Nat Prod. 2008 Dec;1(4):1-4.

Esquenazi D, Wigg MD, Miranda MM, Rodrigues HM, Tostes JB, Rozental S, da Silva AJ, Alviano CS. Antimicrobial and antiviral activities of polyphenolics from *Cocos nucifera* Linn.(Palmae) husk fiber extract. Research in microbiology. 2002 Dec 1;153(10):647-52.

Essien EE, Aboaba SO, Ogunw IA. Constituents and antimicrobial properties of the leaf essential oil of *Gossypium barbadense* (Linn.). Journal of Medicinal Plants Research. 2011 Mar 4;5(5):702-5.

Esteves I, Souza IR, Rodrigues M, Cardoso LG, Santos LS, Sertie JA, Perazzo FF, Lima LM, Schneedorf JM, Bastos JK, Carvalho JC. Gastric antiulcer and anti-inflammatory activities of the essential oil from *Casearia sylvestris* Sw. Journal of Ethnopharmacology. 2005 Oct 3;101(1-3):191-6. doi.org/10.1016/j.jep.2005.04.020

Estomba D, Ladio A, Lozada M. Medicinal wild plant knowledge and gathering patterns in a Mapuche community from North-western Patagonia. J Ethnopharmacol. 2006 Jan 3;103(1):109-19. doi: 10.1016/j.jep.2005.07.015.

Estrada-Castillón E, Soto-Mata BE, Garza-López M, Villarreal-Quintanilla JÁ, Jiménez-Pérez J, Pando-Moreno M, Sánchez-Salas J, Scott-Morales L, Cotera-Correa M. Medicinal plants in the southern region of the State of Nuevo León, México. Journal of Ethnobiology and Ethnomedicine. 2012 Dec;8(1):45. https://doi.org/10.1016/j.jep.2005.07.015

Eswari ML, Bharathi RV, Jayshree N. Hypolipidemic activity on ethanolic extract of leaves of *Ziziphus oenoplia* (L) Mill. Gard. International Journal of Pharmaceutical & Biological Archives. 2013;4(1):136-41.

Ezike AC, Akah PA, Okoli CO, Ufere IK, Ezeudu E, Okoye CF, Ashara C, Igbokwe IN. Studies on gastrointestinal effects of *Desmodium velutinum*: a traditional remedy for diarrhea. American Journal of Pharmacology and Toxicology. 2014 Apr 1;9(2):114-24.

Fabunmi TB, Arotupin DJ. Antioxidant properties of fermented kolanut husk and testa of three species of kolanut*: Cola acuminata, Cola nitida* and *Cola verticillata*. Br Biotechnol J. 2015;8(2):1-3.

Faccin-Galhardi LC, Yamamoto KA, Ray S, Ray B, Linhares RE, Nozawa C. The in vitro antiviral property of *Azadirachta indica* polysaccharides for poliovirus. Journal of ethnopharmacology. 2012 Jun 26;142(1):86-90. https://doi.org/10.1016/j.jep.2012.04.018

Facey PC, Pascoe KO, Porter RB, Jones AD. Investigation of Plants used in Jamaican Folk Medicine for Anti-bacterial Activity. Journal of Pharmacy and Pharmacology. 1999 Dec;51(12):1455-60. https://doi.org/10.1211/0022357991777119

Fadaei S, Asle-Rousta M. Anxiolytic and antidepressant effects of cinnamon (*Cinnamomum verum*) extract in rats receiving lead acetate. Scientific Journal of Kurdistan University of Medical Sciences. 2017;22(6).

Fadili K, Sekkate C, Alistiqsa F, Haloui Z, Chakir S, Zair T. Ethnobotanical study of medicinal plants from Er-Rich region (Moroccan High Atlas). Advances in Environmental Biology. 2017 Jun 1;11(6):27-41.

Fahmy HM, Noor NA, Mohammed FF, Elsayed AA, Radwan NM. *Nigella sativa* as an anti-inflammatory and promising remyelinating agent in the cortex and hippocampus of experimental autoimmune encephalomyelitis-induced rats. The Journal of Basic & Applied Zoology. 2014 Oct 1;67(5):182-95. https://doi.org/10.1016/j.jobaz.2014.08.005

Fajemiroye JO, Adam K, Jordan K Z, Alves CE, Aderoju AA. Evaluation of Anxiolytic and Antidepressant-like Activity of Aqueous Leaf Extract of *Nymphaea lotus* Linn. in Mice. *Iran J Pharm Res*. 2018;17(2):613–626.

Fakoya A, Owojuyigbe OS, Fakoya S, Adeoye SO. Possible antimicrobial activity of *Morinda lucida* stem bark, leaf and root extracts. African Journal of Biotechnology. 2014;13(3). doi: 10.5897/AJB10.1472

Falade T, Ishola IO, Akinleye MO, Oladimeji-Salami JA, Adeyemi OO. Antinociceptive and anti-arthritic effects of aqueous whole plant extract of *Trianthema portulacastrum* in rodents: Possible mechanisms of action. Journal of ethnopharmacology. 2019 Jun 28;238:111831. doi: 10.1016/j.jep.2019.111831.

Falkenberg SS, Tarnow I, Guzman A, Mølgaard P, Simonsen HT. Mapuche herbal medicine inhibits blood platelet aggregation. Evidence-Based Complementary and Alternative Medicine. 2012;2012. http://dx.doi.org/10.1155/2012/647620

Fall AD, Dieng SI, Diatta-Badji K, Diatta W, Bassene E. Phytochemical screening, phenol content and antioxidant studies of ethanol leaf extract of *Celtis toka* (Forssk.) Hepper & JRI Wood. Journal of Pharmacognosy and Phytochemistry. 2017;6(1):488-92.

Falsini B, Marangoni D, Salgarello T, Stifano G, Montrone L, Di Landro S, Guccione L, Balestrazzi E, Colotto A. Effect of epigallocatechin-gallate on inner retinal function in ocular hypertension and glaucoma: a short-term study by pattern electroretinogram. Graefe's Archive for Clinical and Experimental Ophthalmology. 2009 Sep 1;247(9):1223-33.

Fan L, Peng Y, Wu D, Hu J, Yang G, Li X. Dietary supplementation of *Morus nigra* L. leaves decrease fat mass partially through elevating leptin-stimulated lipolysis in pig model. Journal of Ethnopharmacology. 2020 Mar 1;249:112416. https://doi.org/10.1016/j.jep.2019.112416

Fan X, Wang J, Hou J, Lin C, Bensoussan A, Chang D, Liu J, Wang B. Berberine alleviates ox-LDL induced inflammatory factors by up-regulation of autophagy via AMPK/mTOR signaling pathway. Journal of translational medicine. 2015 Dec 1;13(1):92. doi:10.1186/s12967-015-0450-z

Fan Y, Wang N, Rocchi A, Zhang W, Vassar R, Zhou Y, He C. Identification of natural products with neuronal and metabolic benefits through autophagy induction. Autophagy. 2017 Jan 2;13(1):41-56. doi: 10.1080/15548627.2016.1240855.

Fang SH, Rao YK, Tzeng YM. Anti-oxidant and inflammatory mediator's growth inhibitory effects of compounds isolated from *Phyllanthus urinaria*. Journal of Ethnopharmacology. 2008 Mar 5;116(2):333-40. https://doi.org/10.1016/j.jep.2007.11.040

Fang X, Chang RC, Yuen WH, Zee SY. Immune modulatory effects of *Prunella vulgaris* L. International journal of molecular medicine. 2005 Mar 1;15(3):491-6. https://doi.org/10.3892/ijmm.15.3.491

Fani M, Kohanteb J. Inhibitory activity of *Aloe vera* gel on some clinically isolated cariogenic and periodontopathic bacteria. Journal of oral science. 2012;54(1):15-21.

Fankam, A.G., Kuiate, J.R. and Kuete, V., 2015. Antibacterial and antibiotic resistance modifying activity of the extracts from *Allanblackia gabonensis, Combretum molle* and *Gladiolus quartinianus* against Gram-negative bacteria including multi-drug resistant phenotypes. BMC complementary and alternative medicine, *15*(1), p.206. https://doi.org/10.1186/s12906-015-0726-0

Farajpour R, Sadigh-Eteghad S, Ahmadian N, Farzipour M, Mahmoudi J, Majdi A. Chronic administration of *Rosa canina* hydro-alcoholic extract attenuates depressive-like behavior and recognition memory impairment in diabetic mice: a possible role of oxidative stress. Medical principles and practice. 2017;26(3):245-50. https://doi.org/10.1159/000464364

Fardoun M, Al-Shehabi T, El-Yazbi A, Issa K, Zouein F, Maaliki D, Iratni R, Eid AH. *Ziziphus nummularia* inhibits inflammation-induced atherogenic phenotype of human aortic smooth muscle cells. Oxidative medicine and cellular longevity. 2017;2017. Article ID 4134093. https://doi.org/10.1155/2017/4134093

Farid R, Rezaieyazdi Z, Mirfeizi Z, Hatef MR, Mirheidari M, Mansouri H, Esmaelli H, Bentley G, Lu Y, Foo Y, Watson RR. Oral intake of purple passion fruit peel extract reduces pain and stiffness and improves physical function in adult patients with knee osteoarthritis. Nutrition research. 2010 Sep 1;30(9):601-6. https://doi.org/10.1016/j.nutres.2010.08.010

Farinon M, Lora PS, Francescato LN, et al. Effect of Aqueous Extract of Giant Horsetail (*Equisetum giganteum* L.) in Antigen-Induced Arthritis. *Open Rheumatol J*. 2013;7:129–133. Published 2013 Dec 30. doi:10.2174/1874312901307010129

Farjam MH, Khalili M, Rustayian A, Javidnia K, Izadi S. Biological activity of the n-butanolic extract of *Stachys pilifera*. Afr J Microbiol Res. 2011 Nov 30;5(28):5115-9.

Fasola TR, Adeyemo FA, Adeniji JA, Okonko IO. Antiviral potentials of *Enantia chlorantha* extracts on yellow fever virus. J Nat Sci. 2011;9(9):99-105.

Fasola TR, Oluwole ME, Obatayo O, Obayagbo SE. The antimicrobial potential and phytochemical composition of *Aristolochia ringens* Vahl. Adv. Life Sci. Technol. 2015;29:5-12.

Fatani AJ, Al-Rejaie SS, Abuohashish HM, Al-Assaf A, Parmar MY, Ola MS, Ahmed MM. Neuroprotective effects of *Gymnema sylvestre* on streptozotocin-induced diabetic neuropathy in rats. Experimental and therapeutic medicine. 2015 May 1;9(5):1670-8. https://doi.org/10.3892/etm.2015.2305

Fateh, S., Dibazar, S. P., & Daneshmandi, S. (2015). Barberry's (*Berberis integerrima*) ingredients suppress T-cell response and shift immune responses toward Th2: an *in vitro* study. Future science OA, 1(4), FSO49. doi:10.4155/fso.15.49

Fattouch S, Caboni P, Coroneo V, Tuberoso CI, Angioni A, Dessi S, Marzouki N, Cabras P. Antimicrobial activity of Tunisian quince (*Cydonia oblonga* Miller) pulp and peel polyphenolic extracts. Journal of Agricultural and Food Chemistry. 2007 Feb 7;55(3):963-9. https://doi.org/10.1021/jf062614e

Favier L, Tonn C, Guerreiro E, Rotelli A, Pelzer L. Anti-inflammatory activity of acetophenones from Ophryosporus axilliflorus. Planta medica. 1998 Oct;64(07):657-9. doi: 10.1055/s-2006-957543.

Feitosa DJS Junior, de Carvalho LTF, Rocha IRO, de Brito CN, Moreira RA, de Barros CAV. Effects of Copaiba oil in the healing process of urinary bladder in rats. Int Braz J Urol. 2018 Mar-Apr;44(2):384-389. doi: 10.1590/S1677-5538.IBJU.2017.0143.

Félix-Silva J, Gomes JA, Fernandes JM, Moura AK, Menezes YA, Santos EC, Tambourgi DV, Silva-Junior AA, Zucolotto SM, Fernandes-Pedrosa MF. Comparison of two *Jatropha* species (Euphorbiaceae) used popularly to treat snakebites in Northeastern Brazil: Chemical profile, inhibitory activity against *Bothrops erythromelas* venom and antibacterial activity. Journal of ethnopharmacology. 2018 Mar 1;213:12-20.

Feng M, Zhao M, Wang Y, Xu S, Wang M, Zhao C. 1 H-NMR metabonomics study of the therapeutic mechanism of total alkaloids and ajmalicine from *Rauvolfia verticillata* in spontaneously hypertensive rats. Analytical Methods. 2014;6(16):6473-82.

Feng X, Peng Y, Liu M, Cui L. DL-3-n-butylphthalide extends survival by attenuating glial activation in a mouse model of amyotrophic lateral sclerosis. Neuropharmacology. 2012 Feb 1;62(2):1004-10. https://doi.org/10.1016/j.neuropharm.2011.10.009

Feriotto G, Marchetti N, Costa V, Beninati S, Tagliati F, Mischiati C. Chemical Composition of Essential Oils from *Thymus vulgaris*, *Cymbopogon citratus*, and *Rosmarinus officinalis*, and Their Effects on the HIV-1 Tat Protein Function. Chemistry & biodiversity. 2018 Feb;15(2):e1700436. https://doi.org/10.1002/cbdv.201700436

Fernandes ES, Passos GF, Medeiros R, da Cunha FM, Ferreira J, Campos MM, Pianowski LF, Calixto JB. Anti-inflammatory effects of compounds alpha-humulene and (−)-trans-caryophyllene isolated from the essential oil of *Cordia verbenacea*. European journal of pharmacology. 2007 Aug 27;569(3):228-36. https://doi.org/10.1016/j.ejphar.2007.04.059

Fernandes JM, Félix-Silva J, da Cunha LM, et al. Inhibitory Effects of Hydroethanolic Leaf Extracts of *Kalanchoe brasiliensis* and *Kalanchoe pinnata* (Crassulaceae) against Local Effects Induced by *Bothrops jararaca* Snake Venom [published correction appears in PLoS One. 2017 Feb 16;12 (2):e0172598]. *PLoS One*. 2016;11(12):e0168658. Published 2016 Dec 29. doi:10.1371/journal.pone.0168658

Fernandez EC, Sandi YE, Kokoska L. Ethnobotanical inventory of medicinal plants used in the Bustillo Province of the Potosi Department, Bolivia. Fitoterapia. 2003 Jun;74(4):407-16. doi: 10.1016/s0367-326x(03)00053-4.

Fernández Ó, Giovannoni G, Fox RJ, Gold R, Phillips JT, Potts J, Okwuokenye M, Marantz JL. Efficacy and safety of delayed-release dimethyl fumarate for relapsing-remitting multiple sclerosis in prior interferon users: an integrated analysis of DEFINE and CONFIRM. Clinical Therapeutics. 2017 Aug 1;39(8):1671-9.

Fernández YA, Damasceno JL, Abrao F, Silva TD, Cândido AD, Fregonezi NF, Resende FA, Ramos SB, Ambrosio SR, Veneziani RC, Bastos JK. Antibacterial, preservative, and mutagenic potential of *Copaifera* spp. oleoresins against causative agents of foodborne diseases. Foodborne pathogens and disease. 2018 Dec 1;15(12):790-7.

Ferraz MC, Yoshida EH, Tavares RV, Cogo JC, Cintra AC, Dal Belo CA, Franco LM, dos Santos MG, Resende FA, Varanda EA, Hyslop S. An isoflavone from *Dipteryx alata* Vogel is active against the in vitro neuromuscular paralysis of *Bothrops jararacussu* snake venom and bothropstoxin I, and prevents venom-induced myonecrosis. Molecules. 2014 May 6;19(5):5790-805.

Ferrea G, Canessa A, Sampietro F, Cruciani M, Romussi G, Bassetti D. In vitro activity of a *Combretum micranthum* extract against *Herpes simplex* virus types 1 and 2. Antiviral research. 1993 Aug 1;21(4):317-25. https://doi.org/10.1016/0166-3542(93)90010-G

Ferreira A, Proença C, Serralheiro ML, Araujo ME. The in vitro screening for acetylcholinesterase inhibition and antioxidant activity of medicinal plants from Portugal. Journal of ethnopharmacology. 2006 Nov 3;108(1):31-7. https://doi.org/10.1016/j.jep.2006.04.010

Ferreira JD, Chahud F, Ramalho LN, Modulo CM, Vieira LC, Reinach PS, Rodrigues MD, Cunha AS, Paula JS. Rosmarinic acid suppresses subconjunctival neovascularization in experimental glaucoma surgery. Current eye research. 2015 Nov 2;40(11):1134-40. ttps://doi.org/10.3109/02713683.2014.980911

Ferreira JM, Sousa DF, Dantas MB, Fonseca SG, Menezes DB, Martins AM, de Queiroz MG. Effects of *Bixa orellana* L. seeds on hyperlipidemia. Phytotherapy Research. 2013 Jan;27(1):144-7. https://doi.org/10.1002/ptr.4675

Ferreira PR, Mendes CS, Rodrigues CG, Rocha JC, Royo VD, Valério HM, Oliveira DA. Antibacterial activity tannin-rich fraction from leaves of *Anacardium humile*. Ciência Rural. 2012 Oct;42(10):1861-4. https://doi.org/10.1590/S0103-84782012005000080

Ferreira RT, Coutinho MA, Malvar DD, Costa EA, Florentino IF, Costa SS, Vanderlinde FA. Mechanisms underlying the antinociceptive, antiedematogenic, and anti-inflammatory activity of the main flavonoid from *Kalanchoe pinnata*. Evidence-Based Complementary and Alternative Medicine. 2014;2014. doi.org/10.1155/2014/429256

Ferreira-Filho ES, Arcanjo DD, Moura LH, Silva-Filho JC, Paulino ET, Ribeiro ÊA, Chaves MH, Oliveira RD, Oliveira AP. Antihypertensive and vasorelaxant effects of ethanol extract of stem barks from *Zanthoxylum rhoifolium* Lam. in rats. Indian J Exp Biol 2013, Aug; 51: 661-669.

Ferreira-Rodrigues SC, Rodrigues CM, Dos Santos MG, et al. Anti-Inflammatory and Antibothropic Properties of *Jatropha elliptica*, a Plant from Brazilian Cerrado Biome. *Adv Pharm Bull*. 2016;6(4):573–579. doi:10.15171/apb.2016.07+

Ferretta A, Gaballo A, Tanzarella P, Piccoli C, Capitanio N, Nico B, Annese T, Di Paola M, Dell'Aquila C, De Mari M, Ferranini E. Effect of resveratrol on mitochondrial function: implications in parkin-associated familiar Parkinson's disease. Biochimica et Biophysica Acta (BBA)-Molecular Basis of Disease. 2014 Jul 1;1842(7):902-15. https://doi.org/10.1016/j.bbadis.2014.02.010

Figueiredo C, Branco Santos J, Castro Junior J, Wakui V, Rodrigues J, Arruda M, Monteiro A, Monteiro-Neto V, Bomfim M, Kato L, Nascimento da Silva L. *Himatanthus drasticus* leaves: chemical characterization and evaluation of their antimicrobial, antibiofilm, antiproliferative activities. Molecules. 2017;22(6):910. doi.org/10.3390/molecules22060910

Finato AC, Fraga-Silva TF, Prati AU, de Souza Júnior AA, Mazzeu BF, Felippe LG, Pinto RA, de Assis Golim M, Arruda MS, Furlan M, Venturini J. Crude leaf extracts of Piperaceae species downmodulate inflammatory responses by human monocytes. PloS one. 2018;13(6). https:// doi.org/10.1371/journal.pone.0198682

Finger A, Engelhardt UH, Wray V. Flavonol glycosides in tea—kaempferol and quercetin rhamnodiglucosides. Journal of the Science of Food and Agriculture. 1991;55(2):313-21.

Fiori GM, Fachin AL, Correa VS, Bertoni BW, Giuliatti S, Amui SF, de Castro França S, Pereira AM. Antimicrobial activity and rates of tannins in *Stryphnodendron adstringens* Mart. accessions collected in the Brazilian Cerrado. American Journal of Plant Sciences. 2013 Nov 1;4(11):2193. http://dx.doi.org/10.4236/ajps.2013.411272

Fiorini AMR, Barbalho SM, Guiguer EL, Oshiiwa M, Mendes CG, Vieites RL, Chies AB, De Oliveira PB, De Souza MD, Nicolau CC. *Dipteryx alata* Vogel may improve lipid profile and atherogenic indices in Wistar rats *Dipteryx alata* and atherogenic indices. Journal of medicinal food. 2017 Nov 1;20(11):1121-6. https://doi.org/10.1089/jmf.2017.0052

Fleischer TC, Ameade EP, Sawer IK. Antimicrobial activity of the leaves and flowering tops of *Acanthospermum hispidum.* Fitoterapia. 2003 Feb 1;74(1-2):130-2.

Focho DA, Newu MC, Anjah MG, Nwana FA, Ambo FB. Ethnobotanical survey of trees in Fundong, Northwest Region, Cameroon. Journal of Ethnobiology and Ethnomedicine. 2009 Dec;5(1):17

Foe FM, Tchinang TF, Nyegue AM, Abdou JP, Yaya AJ, Tchinda AT, Essame JL, Etoa FX. Chemical composition, in vitro antioxidant and anti-inflammatory properties of essential oils of four dietary and medicinal plants from Cameroon. BMC complementary and alternative medicine. 2016 Dec;16(1):117. doi.org/10.1186/s12906-016-1096-y

Folasade Ehigie A, Amos Akinyemi O, Eunice Ayoade T, Funke Ogundola A, Faith Oyelere S, P Olabinri F. Evaluation of the Antioxidant Potential of Aqueous Extracts of *Moringa oleifera* Leaf and *Cocos nucifera* Husk: A Comparative Analysis. Pharmaceutical and Biomedical Research. 2021 Oct 10;7(4):295-302.

Fomogne-Fodjo MC, Van Vuuren S, Ndinteh DT, Krause RW, Olivier DK. Antibacterial activities of plants from Central Africa used traditionally by the Bakola pygmies for treating respiratory and tuberculosis-related symptoms. Journal of ethnopharmacology. 2014 Aug 8;155(1):123-31.

Fonkeng LS, Mouokeu RS, Tume C, Njateng GS, Kamcthueng MO, Ndonkou NJ, Kuiate JR. Anti-*Staphylococcus aureus* activity of methanol extracts of 12 plants used in Cameroonian folk medicine. BMC research notes. 2015 Dec;8(1):710. doi.org/10.1186/s13104-015-1663-1

Fontaine V, Monteiro E, Fournié M, Brazhnikova E, Boumedine T, Vidal C, Balducci C, Guibout L, Latil M, Dilda PJ, Veillet S, Sahel JA, Lafont R, Camelo S. Systemic administration of the di-apocarotenoid norbixin (BIO201) is neuroprotective, preserves photoreceptor function and inhibits A2E and lipofuscin accumulation in animal models of age-related macular degeneration and Stargardt disease. Aging (Albany NY). 2020 Apr 7;12(7):6151-6171. doi: 10.18632/aging.103014.

Formagio AS, Vieira MC, Volobuff CR, Silva MS, Matos AI, Cardoso CA, Foglio MA, Carvalho JE. In vitro biological screening of the anticholinesterase and antiproliferative activities of medicinal plants belonging to Annonaceae. Brazilian Journal of Medical and Biological Research. 2015 Apr;48(4):308-15.

Forman V, Bukovský M, Grančai D. Immunomodulatory activity of leaf infusions of selected Cornaceae species on human leukocytes. Natural product communications. 2016 May;11(5):1934578X1601100534. https://doi.org/10.1177/1934578X1601100534

Foyet HS, Asongalem AE, Oben EK, Cioanca O, Hancianu M, Hritcu L. Effects of the methanolic extract of *Vitellaria paradoxa* stem bark against scopolamine-induced cognitive dysfunction and oxidative stress in the rat hippocampus. Cellular and molecular neurobiology. 2016 Oct 1;36(7):1139-49. doi.org/10.1007/s10571-015-0310-7

Freitas de Lima F, Lescano CH, Arrigo JD, Cardoso CA, Coutinho JP, Moslaves IS, Ximenes TV, Kadri MC, Weber SS, Perdomo RT, Kassuya CA. Anti-inflammatory, antiproliferative and cytoprotective potential of the *Attalea phalerata* Mart. ex Spreng. pulp oil. PloS one. 2018 Apr 10;13(4):e0195678.

Freixa B, Vila R, Vargas L, Lozano N, Adzet T, Cañigueral S. Screening for antifungal activity of nineteen Latin American plants. Phytotherapy Research: An International Journal Devoted to Pharmacological and Toxicological Evaluation of Natural Product Derivatives. 1998 Sep;12(6):427-30. https://doi.org/10.1002/(SICI)1099-1573(199809)12:6<427::AID-PTR338>3.0.CO;2-X

Frezza C, De Vita D, Spinaci G, Sarandrea M, Venditti A, Bianco A. Secondary metabolites of *Tilia tomentosa* Moench inflorescences collected in Central Italy: chemotaxonomy relevance and phytochemical rationale of traditional use. Natural product research. 2020 Apr 17;34(8):1167-74. https://doi.org/10.1080/14786419.2018.1550487

Fruet AC, Seito LN, Rall VL, Di Stasi LC. Dietary intervention with narrow-leaved cattail rhizome flour (*Typha angustifolia* L.) prevents intestinal inflammation in the trinitrobenzenesulphonic acid model of rat colitis. BMC complementary and alternative medicine. 2012 Dec 1;12(1):62. https://doi.org/10.1186/1472-6882-12-62

Fuentes E, Rodríguez-Pérez W, Guzmán L, Alarcón M, Navarrete S, Forero-Doria O, Palomo I. *Mauritia flexuosa* presents in vitro and in vivo antiplatelet and antithrombotic activities. Evidence-Based Complementary and Alternative Medicine. 2013;2013. http://dx.doi.org/10.1155/2013/653257

Fujita A, Sarkar D, Wu S, Kennelly E, Shetty K, Genovese MI. Evaluation of phenolic-linked bioactives of camu-camu (*Myrciaria dubia* Mc. Vaugh) for antihyperglycemia, antihypertension, antimicrobial properties and cellular rejuvenation. Food Research International. 2015 Nov 1;77:194-203. https://doi.org/10.1016/j.foodres.2013.07.025

Furuta T, Fukuyama Y, Asakawa Y. Polygonolide, an isocoumarin from Polygonum hydropiper possessing anti-inflammatory activity. Phytochemistry. 1986 Jan 22;25(2):517-20. https://doi.org/10.1016/S0031-9422(00)85513-2

Gabr S, Nikles S, Wenzig EM, Ardjomand-Woelkart K, Hathout RM, El-Ahmady S, Motaal AA, Singab A, Bauer R. Characterization and optimization of phenolics extracts from *Acacia* species in relevance to their anti-inflammatory activity. Biochemical systematics and ecology. 2018 Jun 1;78:21-30. https://doi.org/10.1016/j.bse.2018.03.001

Gafner S, Wolfender JL, Nianga M, Stoeckli-Evans H, Hostettmann K. Antifungal and antibacterial naphthoquinones from *Newbouldia laevis* roots. Phytochemistry. 1996 Jul 1;42(5):1315-20. https://doi.org/10.1016/0031-9422(96)00135-5

Galdino PM, Nascimento MV, Sampaio BL, Ferreira RN, Paula JR, Costa EA. Antidepressant-like effect of *Lafoensia pacari* A. St.-Hil. ethanolic extract and fractions in mice. Journal of ethnopharmacology. 2009 Jul 30;124(3):581-5. https://doi.org/10.1016/j.jep.2009.05.001

Galli RL, Bielinski DF, Szprengiel A, Shukitt-Hale B, Joseph JA. Blueberry supplemented diet reverses age-related decline in hippocampal HSP70 neuroprotection. Neurobiology of aging. 2006 Feb 1;27(2):344-50. https://doi.org/10.1016/j.neurobiolaging.2005.01.017

Galuppo M, Giacoppo S, De Nicola GR, Iori R, Navarra M, Lombardo GE, Bramanti P, Mazzon E. Antiinflammatory activity of glucomoringin isothiocyanate in a mouse model of experimental autoimmune encephalomyelitis. Fitoterapia. 2014 Jun 1;95:160-74. https://doi.org/10.1016/j.fitote.2014.03.018

Galvão SM, Mendes FR, Oliveira MG, Mattei R, Mello JC, Roman Júnior WA, Carlini ED. Memory retrieval improvement by *Heteropterys aphrodisiaca* in aging rats. Brazilian Journal of Pharmaceutical Sciences. 2011 Dec;47(4):825-32.

Galvez CE, Jimenez CM, Gomez AD, Lizarraga EF, Sampietro DA. Chemical composition and antifungal activity of essential oils from *Senecio nutans*, *Senecio viridis*, *Tagetes terniflora* and *Aloysia gratissima* against toxigenic *Aspergillus* and *Fusarium* species. Natural product research. 2018 Sep 14:1-4. https://doi.org/10.1080/14786419.2018.1511555

Gamarra C, Castañeda C, Castillo B, Martinez H. Gastric anti-inflamatory and anti-secretory activities in rats treated with an aqueous *Lupinus mutabilis* extract. InMéxico, where old and new world lupins meet. Proceedings of the 11th International Lupin Conference, Guadalajara, Jalisco, Mexico, 4-9 May 2005 2006 (pp. 347-349). International Lupin Association.

Gan N, Wu YC, Brunet M, Garrido C, Chung FL, Dai C, Mi L. Sulforaphane activates heat shock response and enhances proteasome activity through up-regulation of Hsp27. Journal of Biological Chemistry. 2010 Nov 12;285(46):35528-36. doi: 10.1074/jbc.M110.152686

Gandhimathi R. Evaluation of anti-inflammatory activity of *Macaranga peltata* ROXB. J. Pharm. Biol. 2013; 3:36-39.

Gangoue-Pieboji J, Pegnyemb DE, Niyitegeka D, Nsangou A, Eze N, Minyem C, Mbing JN, Ngassam P, Tih RG, Sodengam BL, Bodo B. The in-vitro antimicrobial activities of some medicinal plants from Cameroon. Annals of tropical medicine and parasitology. 2006 Apr 1;100(3):237-43.

Ganguly A, Al Mahmud Z, Uddin MM, Rahman SA. In-vivo anti-inflammatory and anti-pyretic activities of *Manilkara zapota* leaves in albino Wistar rats. Asian Pacific Journal of Tropical Disease. 2013 Aug 1;3(4):301-7. https://doi.org/10.1016/S2222-1808(13)60073-0

Ganguly S, Mula S, Chattopadhyay S, Chatterjee M. An ethanol extract of *Piper betle* Linn. mediates its anti-inflammatory activity via down-regulation of nitric oxide. Journal of Pharmacy and Pharmacology. 2007 May;59(5):711-8.

Gao FJ, Zhang SH, Xu P, et al. Quercetin Declines Apoptosis, Ameliorates Mitochondrial Function and Improves Retinal Ganglion Cell Survival and Function in *In Vivo* Model of Glaucoma in Rat and Retinal Ganglion Cell Culture *In Vitro*. *Front Mol Neurosci*. 2017;10:285. Published 2017 Sep 7. doi:10.3389/fnmol.2017.00285

Gao N, Liu H, Li S, Tu X, Tian S, Liu J, Li G, Ma Y. Volatile Oil from *Acorus gramineus* ameliorates the Injury Neurons in the Hippocampus of Amyloid Beta 1–42 Injected Mice. The Anatomical Record. 2019 Aug. doi.org/10.1002/ar.24236

Gao S, Cui YL, Yu CQ, Wang QS, Zhang Y. Tetrandrine exerts antidepressant-like effects in animal models: role of brain-derived neurotrophic factor. Behavioural brain research. 2013 Feb 1;238:79-85. https://doi.org/10.1016/j.bbr.2012.10.015

Garcıa MD, Fernandez MA, Alvarez A, Saenz MT. Antinociceptive and anti-inflammatory effect of the aqueous extract from leaves of *Pimenta racemosa* var. ozua (Mirtaceae). Journal of ethnopharmacology. 2004 Mar 1;91(1):69-73. https://doi.org/10.1016/j.jep.2003.11.018

García-Rodríguez RV, Zavala-Sánchez MÁ, Notario AD. Anti-inflammatory evaluation and antioxidant potential of *Senna crotalarioides* and *Penstemon roseus*. Boletín Latinoamericano y del Caribe de Plantas medicinales y aromáticas. 2011;10(1):23-9.

Garg VK, Jain ME, Sharma PK, Garg GA. Anti inflammatory activity of *Spinacia oleracea*. Int J Pharma Prof Res. 2010 Jul;1(1):1-4.

Garg VK, Paliwal SK. Anti-inflammatory activity of aqueous extract of *Cynodon dactylon*. Int J pharmacol. 2011a Apr 1;7(3):370-5. doi: 10.3923/ijp.2011.370.375

Garg VK, Paliwal SK. Wound-healing activity of ethanolic and aqueous extracts of *Ficus benghalensis*. J Adv Pharm Technol Res. 2011b Apr;2(2):110-4. doi: 10.4103/2231-4040.82957.

Garrido G, González D, Lemus Y, Garcıa D, Lodeiro L, Quintero G, Delporte C, Núñez-Sellés AJ, Delgado R. In vivo and in vitro anti-inflammatory activity of *Mangifera indica* L. extract (VIMANG®). Pharmacological Research. 2004 Aug 1;50(2):143-9. https://doi.org/10.1016/j.phrs.2003.12.003

Gasparotto Junior A, Gasparotto FM, Lourenço EL, Crestani S, Stefanello ME, Salvador MJ, da Silva-Santos JE, Marques MC, Kassuya CA. Antihypertensive effects of isoquercitrin and extracts from *Tropaeolum majus* L.: evidence for the inhibition of angiotensin converting enzyme. Journal of ethnopharmacology. 2011 Mar 24;134(2):363-72. https://doi.org/10.1016/j.jep.2010.12.026

Gaspar-Pintiliescu A, Seciu AM, Miculescu F, Moldovan L, Ganea E, Craciunescu O. Enhanced extracellular matrix synthesis using collagen dressings loaded with *Artemisia absinthium* plant extract. Journal of Bioactive and Compatible Polymers. 2018 Sep;33(5):516-28.

Gatsing D, Adoga GI. Antisalmonellal activity and phytochemical screening of the various parts of *Cassia petersiana* Bolle (Caesalpiniaceae). Research Journal of Microbiology. 2007 Jan 1;2(11):876-80.

Gautam M, Saha S, Bani S, Kaul A, Mishra S, Patil D, Satti NK, Suri KA, Gairola S, Suresh K, Jadhav S. Immunomodulatory activity of *Asparagus racemosus* on systemic Th1/Th2 immunity: implications for immunoadjuvant potential. Journal of ethnopharmacology. 2009 Jan 21;121(2):241-7. https://doi.org/10.1016/j.jep.2008.10.028

Gautam R, Jachak SM, Saklani A. Anti-inflammatory effect of *Ajuga bracteosa* Wall Ex Benth. mediated through cyclooxygenase (COX) inhibition. Journal of ethnopharmacology. 2011 Jan 27;133(2):928-30

Gautam SS, Kumar S. The antibacterial and phytochemical aspects of *Viola odorata* Linn. extracts against respiratory tract pathogens. Proceedings of the National Academy of Sciences, India Section B: Biological Sciences. 2012 Dec;82(4):567-72. https://doi.org/10.1007/s40011-012-0064-7

Gavimath CC, Kulkarni SM, Raorane CJ, Kalsekar DP, Gavade BG, Ravishankar BE, Hooli RS. Antibacterial potentials of *Solanum indicum*, *Solanum xanthocarpum* and *Physalis minima*. International journal of pharmaceutical applications. 2012;3(4):414-8.

Gazzaneo LR, De Lucena RF, de Albuquerque UP. Knowledge and use of medicinal plants by local specialists in an region of Atlantic Forest in the state of Pernambuco (Northeastern Brazil). Journal of Ethnobiology and Ethnomedicine. 2005 Dec;1(1):9. https://doi.org/10.1186/1746-4269-1-9

Gbadamosi IT, Egunyomi, A. Ethnobotanical survey of plants used for the treatment and management of sexually transmitted infections in Ibadan, Nigeria. Ethnobotany Research and Applications. 2014 Dec 15;12:659-69.

Gebrehiwot T, Rezene T, Kiros T, Medhanie G, Tewolde B. Antibacterial screening and phytochemical study of nine medicinal plants from Eritrea. Pharmacology. 2009;3:546-55.

Gebremariam T, Abula T, Gebremariam MG. Antibacterial and phytochemical screening of root extracts of *Euclea racemosa* subsp. *schimper*i. International Journal of Pharmacognosy. 2015;2(2):66-70.

Gentile C, Tesoriere L, Allegra M, Livrea MA, D'alessio P. Antioxidant betalains from cactus pear (*Opuntia ficus-indica*) inhibit endothelial ICAM-1 expression. Annals of the New York Academy of Sciences. 2004 Dec;1028(1):481-6. doi: 10.1196/annals.1322.057

George RC, Lew J, Graves DJ. Interaction of cinnamaldehyde and epicatechin with tau: implications of beneficial effects in modulating Alzheimer's disease pathogenesis. Journal of Alzheimer's Disease. 2013 Jan 1;36(1):21-40.

Getaneh S, Girma Z. An ethnobotanical study of medicinal plants in Debre Libanos Wereda, Central Ethiopia. African Journal of Plant Science. 2014 Jul 31;8(7):366-79. DOI: 10.5897/AJPS2013.1041

Getie M, Gebre-Mariam T, Rietz R, Höhne C, Huschka C, Schmidtke M, Abate A, Neubert RH. Evaluation of the anti-microbial and anti-inflammatory activities of the medicinal plants *Dodonaea viscosa, Rumex nervosus* and *Rumex abyssinicus*. Fitoterapia. 2003 Feb 1;74(1-2):139-43. https://doi.org/10.1016/S0367-326X(02)00315-5

Geyid A, Abebe D, Debella A, Makonnen Z, Aberra F, Teka F, Kebede T, Urga K, Yersaw K, Biza T, Mariam BH. Screening of some medicinal plants of Ethiopia for their anti-microbial properties and chemical profiles. Journal of ethnopharmacology. 2005 Mar 21;97(3):421-7.

Ghaffari S, Navabzadeh M, Ziaee M, Ghobadi A, Ghods R, Hashem-Dabaghian F. A Randomized, Triple-Blind, Placebo-Controlled, Add-On Clinical Trial to Evaluate the Efficacy of *Emblica officinalis* in Uncontrolled Hypertension. Evidence-Based Complementary and Alternative Medicine. 2020 Oct 7;2020. https://doi.org/10.1155/2020/8592869

Ghahremanitamadon F, Shahidi S, Zargooshnia S, Nikkhah A, Ranjbar A, Soleimani Asl S. Protective effects of *Borago officinalis* extract on amyloid β-peptide (25–35)-induced memory impairment in male rats: a behavioral study. BioMed research international. 2014 Jan 1;2014. https://doi.org/10.1155/2014/798535

Ghanbari M, Zahedi Khorasani M, Vakili A. Acute and chronic effects of Ferula persica on blood pressure of hypertensive rats and its possible mechanism of action. Journal of Medical Plants. 2012 Summer;11(43):62-68.

Ghannadi A, Bagherinejad MR, Abedi D, Jalali M, Absalan B, Sadeghi N. Antibacterial activity and composition of essential oils from *Pelargonium graveolens* L'Her and Vitex agnus-castus L. Iranian journal of microbiology. 2012 Dec;4(4):171.

Gharate M, Kasture V. Evaluation of anti-inflammatory, analgesic, antipyretic and antiulcer activity of Punarnavasava: an Ayurvedic formulation of *Boerhavia diffusa*. Oriental Pharmacy and Experimental Medicine. 2013 Jun 1;13(2):121-6.

Gharib M, Samani LN, Panah ZE, Naseri M, Bahrani N, Kiani K. The effect of valeric on anxiety severity in women undergoing hysterosalpingography. Glob J Health Sci. 2015 Apr 2;7(3):358-63. doi: 10.5539/gjhs.v7n3p358.

Ghavipour M, Sotoudeh G, Tavakoli E, Mowla K, Hasanzadeh J, Mazloom Z. Pomegranate extract alleviates disease activity and some blood biomarkers of inflammation and oxidative stress in Rheumatoid Arthritis patients. European journal of clinical nutrition. 2017 Jan;71(1):92-6. doi: 10.1038/ejcn.2016.151.

Ghazala J, Akhtar S, Vimal K, Mohammad A. Efficacy of Tukhm-e-Kahu (seeds of *Lactuca scariola* Linn.) on mixed anxiety depressive disorder: a randomized placebo controlled double-blind trial. Hamdard Medicus. 2009;52(1):97-101.

Ghildiyal S, Gautam MK, Joshi VK, Goel RK. Pharmacological evaluation of extracts of *Hedychium spicatum* (Ham-ex-Smith) rhizome. Anc Sci Life. 2012 Jan;31(3):117-22. doi: 10.4103/0257-7941.103189.

Ghlissi Z, Kallel R, Sila A, Harrabi B, Atheymen R, Zeghal K, Bougatef A, Sahnoun Z. *Globularia alypum* methanolic extract improves burn wound healing process and inflammation in rats and possesses antibacterial and antioxidant activities. Biomedicine & Pharmacotherapy. 2016 Dec 1;84:1488-95. https://doi.org/10.1016/j.biopha.2016.11.051

Ghlissi Z, Sayari N, Kallel R, Bougatef A, Sahnoun Z. Antioxidant, antibacterial, anti-inflammatory and wound healing effects of *Artemisia campestris* aqueous extract in rat. Biomedicine & Pharmacotherapy. 2016 Dec 1;84:115-22. doi: 10.1016/j.biopha.2016.09.018

Gholijani N, Amirghofran Z. Effects of thymol and carvacrol on T-helper cell subset cytokines and their main transcription factors in ovalbumin-immunized mice. Journal of immunotoxicology. 2016 Sep 2;13(5):729-37. https://doi.org/10.3109/1547691X.2016.1173134

Gholijani N, Gharagozloo M, Kalantar F, Ramezani A, Amirghofran Z. Modulation of cytokine production and transcription factors activities in human Jurkat T cells by thymol and carvacrol. Advanced pharmaceutical bulletin. 2015 Dec;5(Suppl 1):653. doi: 10.15171/apb.2015.089

Ghorbanian D, Ghasemi-Kasman M, Hashemian M, Gorji E, Gol M, Feizi F, Kazemi S, Ashrafpour M, Moghadamnia AA. *Myristica fragrans* Houtt Extract Attenuates Neuronal Loss and Glial Activation in Pentylenetetrazol-Induced Kindling Model. Iran J Pharm Res. 2019 Spring;18(2):812-825. doi: 10.22037/ijpr.2019.1100670

Ghoreschi K, Brück J, Kellerer C, et al. Fumarates improve psoriasis and multiple sclerosis by inducing type II dendritic cells. J Exp Med. 2011;208(11):2291–2303. doi:10.1084/jem.20100977

Ghosh AK, Banerjee M, Bhattacharyya NK. Anti-inflammatory activity of root of *Alpinia galanga* willd. Chron Young Sci 2011;2:139-43. doi: 10.4103/2229-5186.90890

Ghosh R, Mana K, Sarkhel S. Ameliorating effect of *Alstonia scholaris* L. bark extract on histopathological changes following viper envenomation in animal models. Toxicology reports. 2018 Jan 1;5:988-93.https://doi.org/10.1016/j.toxrep.2018.10.004

Ghosh S, Das Sarma M, Patra A, Hazra B. Anti-inflammatory and anticancer compounds isolated from *Ventilago madraspatana* Gaertn., *Rubia cordifolia* Linn. and *Lantana camara* Linn. Journal of pharmacy and pharmacology. 2010 Sep;62(9):1158-66. https://doi.org/10.1111/j.2042-7158.2010.01151.x

Ghosh S, Samanta A, Mandal NB, Bannerjee S, Chattopadhyay D. Evaluation of the wound healing activity of methanol extract of *Pedilanthus tithymaloides* (L.) Poit leaf and its isolated active constituents in topical formulation. Journal of Ethnopharmacology. 2012 Aug 1;142(3):714-22. https://doi.org/10.1016/j.jep.2012.05.048

Ghouti D, Rached W, Abdallah M, Pires TC, Calhelha RC, Alves MJ, Abderrahmane LH, Barros L, Ferreira IC. Phenolic profile and in vitro bioactive potential of Saharan *Juniperus phoenicea* L. and *Cotula cinerea* (Del) growing in Algeria. Food & function. 2018;9(9):4664-72.

Ghule BV, Murugananthan G, Yeole PG. Analgesic and antipyretic effects of *Capparis zeylanica* leaves. Fitoterapia. 2007 Jul 1;78(5):365-9. https://doi.org/10.1016/j.fitote.2007.02.003

Gilani AH, Mehmood MH, Janbaz KH, Khan AU, Saeed SA. Ethnopharmacological studies on antispasmodic and antiplatelet activities of *Ficus carica*. Journal of ethnopharmacology. 2008 Sep 2;119(1):1-5. https://doi.org/10.1016/j.jep.2008.05.040

Gill NS, Bajwa J, Dhiman K, Sharma P, Sood S, Sharma PD, Singh B, Bali M. Evaluation of therapeutic potential of traditionally consumed *Cucumis melo* seeds. Asian Journal of Plant Sciences. 2011;10(1):86.

Ginting GA, Rosidah R, Sitorus P, Satria D. Wound Healing Activity of *Saurauia vulcani* Korth. Aqueous Leaves Extract Evaluation on Excision Wound in Hyperglycemia Rats. JIBS. 2018;5(3):52-7.

Giordani R, Regli P, Kaloustian J, Mikail C, Abou L, Portugal H. Antifungal effect of various essential oils against *Candida albicans*. Potentiation of antifungal action of amphotericin B by essential oil from *Thymus vulgaris*. Phytotherapy Research. 2004 Dec;18(12):990-5. https://doi.org/10.1002/ptr.1594

Girardi C, Butaud JF, Ollier C, Ingert N, Weniger B, Raharivelomanana P, Moretti C. Herbal medicine in the Marquesas Islands. Journal of ethnopharmacology. 2015 Feb 23;161:200-13. https://doi.org/10.1016/j.jep.2014.09.045

Girón LM, Freire V, Alonzo A, Cáceres A. Ethnobotanical survey of the medicinal flora used by the Caribs of Guatemala. Journal of Ethnopharmacology. 1991 Sep 1;34(2-3):173-87.

Gleńsk M, Tichaczek-Goska D, Środa-Pomianek K, Włodarczyk M, Wesolowski CA, Wojnicz D. Differing antibacterial and antibiofilm properties of *Polypodium vulgare* L. Rhizome aqueous extract and one of its purified active ingredients–osladin. Journal of Herbal Medicine. 2019 Sep 1;17:100261. https://doi.org/10.1016/j.hermed.2019.100261

Glišić SB, Milojević SŽ, Dimitrijević SI, Orlović AM, Skala DU. Antimicrobial activity of the essential oil and different fractions of *Juniperus communis* L. and a comparison with some commercial antibiotics. Journal of the Serbian Chemical Society. 2007;72(4):311-20.

Go J, Park TS, Han GH, Park HY, Ryu YK, Kim YH, Hwang JH, Choi DH, Noh JR, Hwang DY, Kim S. Piperlongumine decreases cognitive impairment and improves hippocampal function in aged mice. International journal of molecular medicine. 2018 Oct 1;42(4):1875-84.doi:10.3892/ijmm.2018.3782 AMA

Gochev V, Dobreva A, Girova T, Stoyanova A. Antimicrobial activity of essential oil from *Rosa alba*. Biotechnology & Biotechnological Equipment. 2010 Jan 1;24(sup1):512-5. https://doi.org/10.1080/13102818.2010.10817892

Goel RK, Banerjee RS, Acharya SB. Antiulcerogenic and antiinflammatory studies with shilajit. Journal of ethnopharmacology. 1990 Apr 1;29(1):95-103. https://doi.org/10.1016/0378-8741(90)90102-Y

Goleniowski ME, Bongiovanni GA, Palacio L, Nuñez CO, Cantero JJ. Medicinal plants from the “Sierra de Comechingones”, Argentina. Journal of ethnopharmacology. 2006 Oct 11;107(3):324-41. https://doi.org/10.1016/j.jep.2006.07.026

Gomathi Rajashyamala L, Elango V. Antioxidant activity of *Evolvulus alsinoides* Linn. Asian Journal of Innovative Research. 2016;1(2):39-43.

Gómez-Estrada H, Díaz-Castillo F, Franco-Ospina L, Mercado-Camargo J, Guzmán-Ledezma J, Medina JD, Gaitán-Ibarra R. Folk medicine in the northern coast of Colombia: an overview. Journal of Ethnobiology and Ethnomedicine. 2011 Dec;7(1):27.

Gonçalves MJ, Cavaleiro C, Da Cunha AP, Salgueiro LR. Chemical composition and antimicrobial activity of the commercially available oil of *Luma chequen* (Molina) A. Gray. Journal of Essential Oil Research. 2006 Jan 1;18(1):108-10. https://doi.org/10.1080/10412905.2006.9699402

Goncalves TB, Braga MA, de Oliveira FF, Santiago GM, Carvalho CB, e Cabral PB, de Melo Santiago T, Sousa JS, Barros EB, do Nascimento RF, Nagao-Dias AT. Effect of subinihibitory and inhibitory concentrations of *Plectranthus amboinicus* (Lour.) Spreng essential oil on Klebsiella pneumoniae. Phytomedicine. 2012 Aug 15;19(11):962-8. https://doi.org/10.1016/j.phymed.2012.05.013

Gong EJ, Park HR, Kim ME, Piao S, Lee E, Jo DG, Chung HY, Ha NC, Mattson MP, Lee J. Morin attenuates tau hyperphosphorylation by inhibiting GSK3β. Neurobiology of disease. 2011 Nov 1;44(2):223-30. https://doi.org/10.1016/j.nbd.2011.07.005

González J, Cuéllar A, Sylvius L, Verdeau F, Smith Ravin J, Marcelin O. Antibacterial and Antifungal Activities of Gossypitrin from *Talipariti elatum* Sw.(Fryxell). International Journal of Current Microbiology and Applied Sciences. 2016;5(11):860-6. doi.org/10.20546/ijcmas.2016.511.098

González SB, Houghton PJ, Hoult JR. The activity against leukocyte eicosanoid generation of essential oil and polar fractions of *Adesmia boronioides* Hook. f. Phytotherapy Research. 2003 Mar;17(3):290-3.

González-Burgos E, Ureña-Vacas I, Sánchez M, Gómez-Serranillos MP. Nutritional value of *Moringa oleifera* Lam. leaf powder extracts and their neuroprotective effects via antioxidative and mitochondrial regulation. Nutrients. 2021 Jul;13(7):2203. https://doi.org/10.3390/nu13072203

Gopi K, Anbarasu K, Renu K, Jayanthi S, Vishwanath BS, Jayaraman G. Quercetin-3-O-rhamnoside from *Euphorbia hirta* protects against snake Venom induced toxicity. Biochimica et Biophysica Acta (BBA)-General Subjects. 2016 Jul 1;1860(7):1528-40. https://doi.org/10.1016/j.jep.2015.02.044

Gou KJ, Zeng R, Dong Y, Hu QQ, Hu HW, Maffucci KG, Dou QL, Yang QB, Qin XH, Qu Y. Anti-inflammatory and analgesic effects of *Polygonum orientale* L. Extracts. Frontiers in pharmacology. 2017 Aug 30;8:562. https://doi.org/10.3389/fphar.2017.00562

Govindappa M, Bharath N, Shruthi HB, Sadananda TS, Sharanappa P. Antimicrobial, antioxidant and in vitro anti-inflammatory activity and phytochemical screening of *Crotalaria pallida* Aiton. African Journal of Pharmacy and Pharmacology. 2011 Dec 8;5(21):2359-71. doi: 10.5897/AJPP11.038

Govindarajan R, Vijayakumar M, Rao CV, Shirwaikar A, Kumar S, Rawat AK, Pushpangadan P. Antiinflammatory and antioxidant activities of *Desmodium gangeticum* fractions in carrageenan-induced inflamed rats. Phytotherapy Research. 2007 Oct;21(10):975-9. https://doi.org/10.1002/ptr.2199

Goyal P, Chauhan A, Kaushik P. Laboratory evaluation of crude extracts of *Cinnamomum tamala* for potential antibacterial activity. Electronic journal of Biology. 2009;5(4):75-9.

Goyal R, Sharma PL, Singh M. Possible attenuation of nitric oxide expression in anti-inflammatory effect of *Ziziphus jujuba* in rat. Journal of natural medicines. 2011 Jul 1;65(3-4):514-8. https://doi.org/10.1007/s11418-011-0531-0

Grace MH, Esposito D, Dunlap KL, Lila MA. Comparative analysis of phenolic content and profile, antioxidant capacity, and anti-inflammatory bioactivity in wild Alaskan and commercial *Vaccinium* berries. Journal of agricultural and food chemistry. 2013 Nov 19;62(18):4007-17. doi: 10.1021/jf403810y

Gray NE, Harris CJ, Quinn JF, Soumyanath A. *Centella asiatica* modulates antioxidant and mitochondrial pathways and improves cognitive function in mice. J Ethnopharmacol. 2016 Mar 2;180:78-86. doi: 10.1016/j.jep.2016.01.013. Epub 2016 Jan 16.

Gray NE, Zweig JA, Matthews DG, Caruso M, Quinn JF, Soumyanath A. *Centella asiatica* attenuates mitochondrial dysfunction and oxidative stress in Aβ-exposed hippocampal neurons. Oxidative medicine and cellular longevity. 2017 Oct;2017. https://doi.org/10.1155/2017/7023091

Green E, Samie A, Obi CL, Bessong PO, Ndip RN. Inhibitory properties of selected South African medicinal plants against *Mycobacterium tuberculosis*. Journal of ethnopharmacology. 2010 Jul 6;130(1):151-7. https://doi.org/10.1016/j.jep.2010.04.033

Grossi C, Rigacci S, Ambrosini S, Dami TE, Luccarini I, Traini C, Failli P, Berti A, Casamenti F, Stefani M. The polyphenol oleuropein aglycone protects TgCRND8 mice against Aß plaque pathology. PloS one. 2013 Aug 8;8(8):e71702. doi: 10.1371/journal.pone.0071702.

Grosvenor PW, Supriono A, Gray DO. Medicinal plants from Riau Province, Sumatra, Indonesia. Part 2: antibacterial and antifungal activity. Journal of ethnopharmacology. 1995 Feb 1;45(2):97-111.

Gu JQ, Wang Y, Franzblau SG, Montenegro G, Timmermann BN. Constituents of *Quinchamalium majus* with potential antitubercular activity. Zeitschrift für Naturforschung C. 2004 Dec 1;59(11-12):797-802. https://doi.org/10.1515/znc-2004-11-1206

Gu XY, Shen XF, Wang L, Wu ZW, Li F, Chen B, Zhang GL, Wang MK. Bioactive steroidal alkaloids from the fruits of *Solanum nigrum*. Phytochemistry. 2018 Mar 1;147:125-31.

Gualtieri MJ, Malafronte N, Vassallo A, Braca A, Cotugno R, Vasaturo M, De Tommasi N, Dal Piaz F. Bioactive limonoids from the leaves of *Azaridachta indica* (Neem). Journal of natural products. 2014 Mar 28;77(3):596-602. https://doi.org/10.1021/np400863d

Guaouguaou FE, Taghzouti K, Oukabli M, Es-Safi NE. The Effect of *Salvia verbenaca* Extracts for Healing of Second-Degree Burn Wounds in Rats. Current Bioactive Compounds. 2018 Dec 1;14(4):419-27. https://doi.org/10.2174/1573407213666170621074336

Guerrero MF, Carrón R, Martın ML, San Román L, Reguero MT. Antihypertensive and vasorelaxant effects of aqueous extract from *Croton schiedeanus* Schlecht in rats. Journal of ethnopharmacology. 2001 Apr 1;75(1):33-6.

Guizani N, Waly MI, Ali A, Al-Saidi G, Singh V, Bhatt N, Rahman MS. Papaya epicarp extract protects against hydrogen peroxide induced oxidative stress in human SH-SY5Y neuronal cells. Exp Biol Med 2011; 236 (10):1205-10.

Gujjeti RP, Mamidala E. Anti-HIV activity of phytosterol isolated from Aerva lanata roots. Pharmacognosy Journal. 2017;9(1).

Gul S, Ahmed S, Kifli N, Uddin QT, Tahir NB, Hussain A, Jaafar HZ, Moga M, Zia-Ul-Haq M. Multiple pathways are responsible for Anti-inflammatory and Cardiovascular activities of *Hordeum vulgare* L. Journal of translational medicine. 2014 Dec;12(1):316.

Gülçin I, Küfrevioğlu Öİ, Oktay M, Büyükokuroğlu ME. Antioxidant, antimicrobial, antiulcer and analgesic activities of nettle (*Urtica dioica* L.). Journal of ethnopharmacology. 2004 Feb 1;90(2-3):205-15. https://doi.org/10.1016/j.jep.2003.09.028

Gülçın İ, Oktay M, Kıreçcı E, Küfrevıoğlu Öİ. Screening of antioxidant and antimicrobial activities of anise (*Pimpinella anisum* L.) seed extracts. Food chemistry. 2003 Nov 1;83(3):371-82. doi.org/10.1016/S0308-8146(03)00098-0

Gundimeda U., McNeill T. H., Schiffman J. E., Hinton D. R., Gopalakrishna R. Green tea polyphenols potentiate the action of nerve growth factor to induce neuritogenesis: possible role of reactive oxygen species. *Journal of Neuroscience Research*. 2010;88(16):3644–3655. doi: 10.1002/jnr.22519.

Guo JP, Pang J, Wang XW, Shen ZQ, Jin M, Li JW. In vitro screening of traditionally used medicinal plants in China against enteroviruses. *World J Gastroenterol*. 2006;12(25):4078-4081. doi: 10.3748/wjg.v12.i25.4078

Guo T, Deng YX, Xie H, Yao CY, Cai CC, Pan SL, Wang YL. Antinociceptive and anti-inflammatory activities of ethyl acetate fraction from *Zanthoxylum armatum* in mice. Fitoterapia. 2011 Apr 1;82(3):347-51.

Guo ZY, Li P, Huang W, Wang JJ, Liu YJ, Liu B, Wang YL, Wu SB, Kennelly EJ, Long CL. Antioxidant and anti-inflammatory caffeoyl phenylpropanoid and secoiridoid glycosides from *Jasminum nervosum* stems, a Chinese folk medicine. Phytochemistry. 2014 Oct 1;106:124-33. https://doi.org/10.1016/j.phytochem.2014.07.011

Gupta A, Sethi J, Sood S, Dahiya K, Singh G, Gupta R. Evaluation of hypoglycemic and anti-atherogenic effect of *Aloe vera* in diabetes mellitus. Pharmacie Globale. 2011:1-4.

Gupta D, Bhardwaj R, Gupta RK. In Vitro antioxidant activity of extracts from the leaves of *Abies pindrow* Royle. African Journal of Traditional, Complementary and Alternative Medicines. 2011;8(4).

Gupta M, Mazumder UK, Kumar RS, Gomathi P, Rajeshwar Y, Kakoti BB, Selven VT. Anti-inflammatory, analgesic and antipyretic effects of methanol extract from *Bauhinia racemosa* stem bark in animal models. Journal of ethnopharmacology. 2005 Apr 26;98(3):267-73. https://doi.org/10.1016/j.jep.2005.01.018

Gupta M, Mazumder UK, Sambath KR, Siva KT. Studies on anti-inflammatory, analgesic and antipyretic properties of methanol extract of *Caesalpinia bonducella* leaves in experimental animal models. Iranian Journal Of Pharmacology and Therapeutics Fall 2003; 2,(2);30-34.

Gupta P, Sharma U, Gupta P, Siripurapu KB, Maurya R. Evolvosides C–E, flavonol-4-O-triglycosides from *Evolvulus alsinoides* and their anti-stress activity. Bioorganic & medicinal chemistry. 2013 Mar 1;21(5):1116-22.

Gupta SM, Gupta AK, Ahmed Z, Kumar A. Antibacterial and antifungal activity in leaf, seed extract and seed oil of seabuckthorn (*Hippophae salicifolia* D. Don) plant. J Plant Pathol Microbiol. 2011;2(2):1-4.

Gupta VK, Simlai A, Tiwari M, Bhattacharya K, Roy A. Phytochemical contents, antimicrobial and antioxidative activities of *Solanum sisymbriifolium*. Journal of Applied Pharmaceutical Science. 2014 Mar 1;4(3):75. doi: 10.7324/JAPS.2014.40315

Gürbüz I, Üstün O, Yeşilada E, Sezik E, Akyürek N. In vivo gastroprotective effects of five Turkish folk remedies against ethanol-induced lesions. Journal of Ethnopharmacology. 2002 Dec 1;83(3):241-4.

Gurgel AP, da Silva JG, Grangeiro AR, Oliveira DC, Lima CM, da Silva AC, Oliveira RA, Souza IA. In vivo study of the anti-inflammatory and antitumor activities of leaves from *Plectranthus amboinicus* (Lour.) Spreng (Lamiaceae). Journal of Ethnopharmacology. 2009 Sep 7;125(2):361-3. https://doi.org/10.1016/j.jep.2009.07.006

Gurgel LA, Sidrim JJ, Martins DT, Cechinel Filho V, Rao VS. In vitro antifungal activity of dragon's blood from *Croton urucurana* against dermatophytes. Journal of Ethnopharmacology. 2005 Feb 28;97(2):409-12.

Guzman-Martinez L, Maccioni RB, Andrade V, Navarrete LP, Pastor MG, Ramos-Escobar N. Neuroinflammation as a Common Feature of Neurodegenerative Disorders. Front Pharmacol. 2019;10:1008. https://doi.org/10.3389/fphar.2019.01008

Gyamfi MA, Yonamine M, Aniya Y. Free-radical scavenging action of medicinal herbs from Ghana: *Thonningia sanguinea* on experimentally-induced liver injuries. General Pharmacology: The Vascular System. 1999 Jun 1;32(6):661-7. https://doi.org/10.1016/S0306-3623(98)00238-9

Gyuris A, Szlavik L, Minarovits J, Vasas A, Molnar J, Hohmann J. Antiviral activities of extracts of *Euphorbia hirta* L. against HIV-1, HIV-2 and SIVmac251. in vivo. 2009 May 1;23(3):429-32.

Habeeb F, Shakir E, Bradbury F, Cameron P, Taravati MR, Drummond AJ, Gray AI, Ferro VA. Screening methods used to determine the anti-microbial properties of *Aloe vera* inner gel. Methods. 2007 Aug 1;42(4):315-20. https://doi.org/10.1016/j.ymeth.2007.03.004

Habila JD, Bello IA, Dzikwe AA, Ladan Z, Sabiu M. Comparative Evaluation of Phytochemicals, Antioxidant and Antimicrobial Activity of Four Medicinal Plants Native to Northern Nigeria. Australian *Journal of Basic and Applied Sciences*, 2011; 5(5): 537-543.

Habsah M, Amran M, Mackeen MM, Lajis NH, Kikuzaki H, Nakatani N, Rahman AA, Ali AM. Screening of Zingiberaceae extracts for antimicrobial and antioxidant activities. Journal of ethnopharmacology. 2000 Oct 1;72(3):403-10.

Habtemariam S. The therapeutic potential of *Berberis darwinii* stem-bark: quantification of berberine and in vitro evidence for Alzheimer's disease therapy. Natural product communications. 2011 Aug;6(8):1934578X1100600809.

Hachimi E. Anti-inflammatory activity of the seed oil of *Zizyphus lotus* (L.) Desf. Phytotherapy 2017;15, 147–154. https://doi.org/10.1007/s10298-016-1056-1

Hafid AF, Aoki-Utsubo C, Permanasari AA, Adianti M, Tumewu L, Widyawaruyanti A, Wahyuningsih SP, Wahyuni TS, Lusida MI, Hotta H. Antiviral activity of the dichloromethane extracts from *Artocarpus heterophyllus* leaves against hepatitis C virus. Asian Pacific Journal of Tropical Biomedicine. 2017 Jul 1;7(7):633-9.

Hafiz TA, Mubaraki M, Dkhil M, Al-Quraishy S. Antiviral activities of *Capsicum annuum* methanolic extract against *Herpes simplex* virus 1 and 2. Pak. J. Zool. 2017 Feb 1;49(1):251. http://dx.doi.org/10.17582/journal.pjz/2017.49.1.267.272

Hage-Sleiman R, Mroueh M, Daher CF. Pharmacological evaluation of aqueous extract of *Althaea officinalis* flower grown in Lebanon. Pharmaceutical biology. 2011 Mar 1;49(3):327-33. https://doi.org/10.3109/13880209.2010.516754

Hahm TS, Park SJ, Lo YM. Effects of Medicinal plant extracts on Blood pressure in Spontaneously Hypertensive Rats. The FASEB Journal. 2009 Apr;23:718-9. https://doi.org/10.1096/fasebj.23.1_supplement.718.9

Haidari M, Ali M, Casscells III SW, Madjid M. Pomegranate (*Punica granatum*) purified polyphenol extract inhibits influenza virus and has a synergistic effect with oseltamivir. Phytomedicine. 2009 Dec 1;16(12):1127-36. https://doi.org/10.1016/j.phymed.2009.06.002

Haider S, Batool Z, Tabassum S, Perveen T, Saleem S, Naqvi F, Javed H, Haleem DJ. Effects of walnuts (*Juglans regia*) on learning and memory functions. Plant foods for human nutrition. 2011a Nov 1;66(4):335-40.

Haider S, Nazreen S, Alam MM, Gupta A, Hamid H, Alam MS. Anti-inflammatory and anti-nociceptive activities of ethanolic extract and its various fractions from *Adiantum capillus veneris* Linn. Journal of ethnopharmacology. 2011b Dec 8;138(3):741-7.

Haiyun L, Yijia L, Honggang L, Honghai W. Protective effect of total flavones from *Elsholtzia blanda* (TFEB) on myocardial ischemia induced by coronary occlusion in canines. Journal of ethnopharmacology. 2004 Sep 1;94(1):101-7. https://doi.org/10.1016/j.jep.2004.04.016

Hajhashemi V, Abbasi N. Hypolipidemic activity of *Anethum graveolens* in rats. Phytotherapy Research: An International Journal Devoted to Pharmacological and Toxicological Evaluation of Natural Product Derivatives. 2008 Mar;22(3):372-5.https://doi.org/10.1002/ptr.2329

Hajhashemi V, Ghannadi A, Heidari AH. Anti-inflammatory and wound healing activities of *Aloe littoralis* in rats. *Res Pharm Sci*. 2012;7(2):73–78.

Hajhashemi V, Sajjadi SE, Zomorodkia M. Antinociceptive and anti-inflammatory activities o*f Bunium persicum* essential oil, hydroalcoholic and polyphenolic extracts in animal models. Pharmaceutical biology. 2011 Feb 1;49(2):146-51.

Halabi MF, Sheikh BY. Anti-proliferative effect and phytochemical analysis of *Cymbopogon citratus* extract. BioMed research international. 2014 Mar 27;2014. https://doi.org/10.1155/2014/906239

Hamaguchi T, Ono K, Murase A, Yamada M. Phenolic compounds prevent Alzheimer's pathology through different effects on the amyloid-beta aggregation pathway. *Am J Pathol*. 2009;175(6):2557-65. doi: 10.2353/ajpath.2009.090417

Hamedi A, Zomorodian K, Safari F. Antimicrobial activity of four medicinal plants widely used in Persian folk medicine. Research Journal of Pharmacognosy. 2015 Jan 1;2(1):25-33.

Hamiduzzaman M, Azam AZ. Antimicrobial, antioxidant and cytotoxic activities of *Gomphrena globosa* (L.). Bangladesh Pharmaceutical Journal. 2012 Nov 13;15(2):183-5.

Hammiche V, Maiza K. Traditional medicine in Central Sahara: pharmacopoeia of Tassili N'ajjer. Journal of ethnopharmacology. 2006 May 24;105(3):358-67.

Hammond TR, Marsh SE, Stevens B. Immune Signaling in Neurodegeneration. Immunity. 2019;50(4):955-74. https://doi.org/10.1016/j.immuni.2019.03.016

Hamsa TP, Kuttan G. Evaluation of the anti-inflammatory and anti-tumor effect of *Ipomoea obscura* (L) and its mode of action through the inhibition of pro inflammatory cytokines, nitric oxide and COX-2. Inflammation. 2011 Jun 1;34(3):171-83. doi.org/10.1007/s10753-010-9221-4

Han X. In vitro biological activities of Douglas fir essential oil in a human skin disease model. Cogent Biology. 2017 Jan 1;3(1):1336886. https://doi.org/10.1080/23312025.2017.1336886

Han YQ, Huang ZM, Yang XB, Liu HZ, Wu GX. In vivo and in vitro anti-hepatitis B virus activity of total phenolics from *Oenanthe javanica*. Journal of ethnopharmacology. 2008 Jun 19;118(1):148-53. https://doi.org/10.1016/j.jep.2008.03.024

Handique C, Lahkar M, Dutta A. Study of the effect of the extract of the leaves of *Alternanthera brasiliana* in experimentally induced inflammatory bowel disease in experimental animals. World Journal of Pharmacy and Pharmaceutical Sciences. 2017 Feb 12;6(4):1806-18. doi: 10.20959/wjpps20174-8993

Hanganu DA, Benedec DA, Olah NK, Ranga F, Mirel SI, Tiperciuc BR, Oniga I. Research on enzyme inhibition potential and phenolic compounds from *Origanum vulgare* ssp. *vulgare*. Farmacia. 2020 Nov 1;68:1075-80.

Hanif A, Hossan MS, Mia MM, Islam MJ, Jahan R, Rahamatullah M. Ethnobotanical survey of the Rakhian tribe inhabiting the Chittagong hill tracts region of Bangladesh. Am Eurasian J Sustaibn Agric. 2009;3(2):172-80.

Hanish Singh JC, Alagarsamy V, Sathesh Kumar S, Narsimha Reddy Y. Neurotransmitter metabolic enzymes and antioxidant status on Alzheimer's disease induced mice treated with *Alpinia galanga* (L.) Willd. Phytotherapy Research. 2011 Jul;25(7):1061-7. https://doi.org/10.1002/ptr.3364

Hanson PM, Yang RY, Tsou SC, Ledesma D, Engle L, Lee TC. Diversity in eggplant (*Solanum melongena*) for superoxide scavenging activity, total phenolics, and ascorbic acid. Journal of Food composition and Analysis. 2006 Sep 1;19(6-7):594-600. https://doi.org/10.1016/j.jfca.2006.03.001

Hara H, Maruyama N, Yamashita S, Hayashi Y, Lee KH, Bastow KF, Marumoto R, Imakura Y. Elecanacin, a novel new naphthoquinone from the bulb of *Eleutherine americana*. Chemical and pharmaceutical bulletin. 1997 Oct 15;45(10):1714-6. https://doi.org/10.1248/cpb.45.1714

Haraguchi H, Kuwata Y, Inada K, Shingu K, Miyahara K, Nagao M, Yagi A. Antifungal activity from *Alpinia galanga* and the competition for incorporation of unsaturated fatty acids in cell growth. Planta medica. 1996 Aug;62(04):308-13. doi: 10.1055/s-2006-957890

Harbilas D, Vallerand D, Brault A, Saleem A, Arnason JT, Musallam L, Haddad PS. *Larix laricina*, an antidiabetic alternative treatment from the Cree of Northern Quebec pharmacopoeia, decreases glycemia and improves insulin sensitivity in vivo. Evidence-Based Complementary and Alternative Medicine. 2012;2012. https://doi.org/10.1155/2012/296432

Harder C, de Oliveira AL, Scriboni AB, Cintra AC, Schezaro-Ramos R, dos Santos MG, Cogo-Müller K, Miura RY, Floriano RS, Rostelato-Ferreira S, Oshima-Franco Y. Pharmacological Properties of *Vochysia haenkeana* (Vochysiaceae) Extract to Neutralize the Neuromuscular Blockade Induced by Bothropstoxin-I (Lys49 Phospholipase $A_2$) Myotoxin. *Adv Pharm Bull*. 2017;7(3):433–439. doi:10.15171/apb.2017.052

Haridas R, Thangapandian V, Thomas B. International Journal of Pharmacology Research. 2014: 4 (3):144-147.

Harnafi M, Bekkouch O, Touiss I, Khatib S, Mokhtari I, Milenkovic D, Harnafi H, Amrani S. Phenolic-Rich Extract from Almond (*Prunus dulcis*) Hulls Improves Lipid Metabolism in Triton WR-1339 and High-Fat Diet-Induced Hyperlipidemic Mice and Prevents Lipoprotein Oxidation: A Comparison with Fenofibrate and Butylated Hydroxyanisole. Prev Nutr Food Sci. 2020 Sep 30;25(3):254–62. doi: 10.3746/pnf.2020.25.3.254.

Harrison JJ, Ayine-Tora MD, Appiagyei B, Mills-Robertson FC, Asomaning WA, Achel DG, Ishida H, Kingsford-Adaboh R. Crystal structure and in vitro antimicrobial activity studies of Robustic acid and other Alpinum isoflavones isolated from *Millettia thonningii*. Zeitschrift für Kristallographie-Crystalline Materials. 2019 Apr 24;234(4):229-35. doi.org/10.1515/zkri-2018-2052

Hasan MF, Das R, Khan A, Hossain MS, Rahman M. The determination of antibacterial and antifungal activities of *Polygonum hydropiper* (L.) root extract. Advances in Biological Research. 2009;3(1-2):53-6.

Hasrat JA, Pieters L, Vlietinck AJ. Medicinal plants in Suriname: hypotensive effect of *Gossypium barbadense*. Journal of pharmacy and Pharmacology. 2004 Mar;56(3):381-7. https://doi.org/10.1211/0022357022917

Hassan GA, Ahmad TB, Mohi-ud-din RA. An ethnobotanical study in Budgam district of Kashmir valley: an attempt to explore and document traditional knowledge of the area. International Research Journal of Pharmacy. 2013;4:201-4.

Hassan HS, Ahmadu AA, Hassan AS. Analgesic and anti-inflammatory activities of *Asparagus africanus* root extract. African Journal of Traditional, Complementary and Alternative Medicines. 2008;5(1):27-31.

Hassan HS, Sule MI, Musa AM, Musa KY, Abubakar MS, Hassan AS. Anti-inflammatory activity of crude saponin extracts from five Nigerian medicinal plants. African Journal of Traditional, Complementary and Alternative Medicines. 2012;9(2):250-5.

Hassan HS, Yau J, Abubakar J, AHMADU A. Phytochemical, Analgesic and Anti-Inflammatory Studies of the Methanol Leaf Extract of *Commiphora mollis* (Oliv.) Engl.(Burseraceae). Nigerian Journal of Pharmaceutical Research. 2018 Jan 24;13(2):137-46.

Hasson SS, Al-Balushi MS, Sallam TA, Idris MA, Habbal O, Al-Jabri AA. In vitro antibacterial activity of three medicinal plants-Boswellia (Luban) species. Asian Pacific Journal of Tropical Biomedicine. 2011 Oct 1;1(2):S178-82. https://doi.org/10.1016/S2221-1691(11)60151-2

Hati M, Jena BK, Kar S, Nayak AK. Evaluation of anti-inflammatory and anti-pyretic activity of *Carissa carandas* L. leaf extract in rats. J. Pharm. Chem. Biol. Sci. 2014;1:18-25.

Hawkins J, Baker C, Cherry L, Dunne E. Black elderberry (*Sambucus nigra*) supplementation effectively treats upper respiratory symptoms: A meta-analysis of randomized, controlled clinical trials. Complementary therapies in medicine. 2019 Feb 1;42:361-5. doi: 10.1016/j.ctim.2018.12.004.

Hayashi K, Niwayama S, Hayashi T, Nago R, Ochiai H, Morita N. In vitro and in vivo antiviral activity of scopadulcic acid B from *Scoparia dulcis*, Scrophulariaceae, against herpes simplex virus type 1. Antiviral research. 1988 Sep 1;9(6):345-54.

He GL, Luo Z, Yang J, Shen TT, Chen Y, Yang XS. Curcumin ameliorates the reduction effect of PGE2 on fibrillar β-amyloid peptide (1-42)-induced microglial phagocytosis through the inhibition of EP2-PKA signaling in N9 microglial cells. PLoS One. 2016 Jan 29;11(1):e0147721.

Hearst C, McCollum G, Nelson D, Ballard LM, Millar BC, Goldsmith CE, Rooney PJ, Loughrey A, Moore JE, Rao JR. Antibacterial activity of elder (*Sambucus nigra* L.) flower or berry against hospital pathogens. Journal of Medicinal Plants Research. 2010 Sep 4;4(17):1805-9. doi: 10.5897/JMPR10.147

Hegazy ME, Ohta S, Abdel-latif FF, Albadry HA, Ohta E, Paré PW, Hirata T. Cyclooxygenase (COX)-1 and-2 inhibitory labdane diterpenes from *Crassocephalum mannii*. Journal of natural products. 2008 Jun 27;71(6):1070-3. https://doi.org/10.1021/np800017x

Hegde K, Thakker SP, Joshi AB, Shastry CS, Chandrashekhar KS. Anticonvulsant activity of *Carissa carandas* Linn. root extract in experimental mice. Tropical Journal of Pharmaceutical Research. 2009;8(2). http://dx.doi.org/10.4314/tjpr.v8i2.44519

Hegde V, Nagaraj N, Gowda SK, Thippeswamy NB. Antibacterial and antidiarrheal activity of *Simarouba amara* (Aubl.) bark. J Appl Pharm Sci, 2019; 9(05):088–096. doi: 10.7324/JAPS.2019.90511

Hema K. In vitro anti-inflammatory activity of quercitrin isolated from *Allamanda cathartica.* Int. J. Pharm. Bio-Sci. 2014;5:440-5.

Hendra H, Moeljopawiro S, Nuringtyas TR. Antioxidant and antibacterial activities of agarwood (*Aquilaria malaccensis* Lamk.) leaves. InAIP Conference Proceedings 2016 Jul 21 (Vol. 1755, No. 1, p. 140004). AIP Publishing.

Hendriks JJ, Alblas J, van der Pol SM, van Tol EA, Dijkstra CD, de Vries HE. Flavonoids influence monocytic GTPase activity and are protective in experimental allergic encephalitis. Journal of Experimental Medicine. 2004 Dec 20;200(12):1667-72. doi: 10.1084/jem.20040819

Heneka MT, Carson MJ, El Khoury J, Landreth GE, Brosseron F, Feinstein DL, Jacobs AH, Wyss-Coray T, Vitorica J, Ransohoff RM, Herrup K. Neuroinflammation in Alzheimer's disease. The Lancet Neurology. 2015 Apr 1;14(4):388-405. https://doi.org/10.1016/S1474-4422(15)70016-5

Heng Y, Zhang QS, Mu Z, Hu JF, Yuan YH, Chen NH. Ginsenoside Rg1 attenuates motor impairment and neuroinflammation in the MPTP-probenecid-induced parkinsonism mouse model by targeting α-synuclein abnormalities in the substantia nigra. Toxicology letters. 2016 Jan 22;243:7-21. https://doi.org/10.1016/j.jsbmb.2015.09.040

Henneh IT, Akrofi R, Ameyaw EO, Konja D, Owusu G, Abane B, Acquah-Mills J, Edzeameh FJ, Tayman F. Stem Bark Extract of Sterculia setigera Delile Exhibits Anti-inflammatory Properties through Membrane Stabilization, Inhibition of Protein Denaturation and Prostaglandin E2 Activity. Journal of Pharmaceutical Research International. 2018 Jun 20:1-1. https://doi.org/10.9734/JPRI/2018/42030

Henriques BO, Corrêa O, Azevedo EP, Pádua RM, Oliveira VL, Oliveira TH, Boff D, Dias AC, Souza DG, Amaral FA, Teixeira MM. In vitro TNF-inhibitory activity of brazilian plants and anti-inflammatory effect of *Stryphnodendron adstringens* in an acute arthritis model. Evidence-based Complementary and Alternative Medicine. 2016;2016. http://dx.doi.org/10.1155/2016/9872598

Hernández NE, Tereschuk ML, Abdala LR. Antimicrobial activity of flavonoids in medicinal plants from Tafı del Valle (Tucuman, Argentina). Journal of Ethnopharmacology. 2000 Nov 1;73(1-2):317-22. https://doi.org/10.1016/S0378-8741(00)00295-6

Hernández-Ortega M, Ortiz-Moreno A, Hernández-Navarro MD, Chamorro-Cevallos G, Dorantes-Alvarez L, Necoechea-Mondragón H. Antioxidant, antinociceptive, and anti-inflammatory effects of carotenoids extracted from dried pepper (*Capsicum annuum* L.). BioMed Research International. 2012 Oct 2;2012. doi:10.1155/2012/524019

Herrera-Calderon O, Enciso-Roca E, Pari-Olarte B, Arroyo-Acevedo J. Phytochemical screening, antioxidant activity and analgesic effect of *Waltheria ovata* Cav. roots in mice. Asian Pacific Journal of Tropical Disease. 2016 Dec 1;6(12):1000-3. https://doi.org/10.1016/S2222-1808(16)61172-6

Hickman S, Izzy S, Sen P, Morsett L, El Khoury J. Microglia in neurodegeneration. Nat Neurosci. 2018;21(10):1359-69. https://doi.org/10.1038/s41593-018-0242-x

Hien TT, Quang TH, Tai BH, Nhiem NX, Yen PH, Yen DT, Cuong LC, Kim YC, Oh H, Van Minh C, Van Kiem P. Iridoid Glycosides and Phenolic Glycosides from *Buddleja asiatica* with Anti-inflammatory and Cytoprotective Activities. Natural Product Communications. 2018 Jan;13(1):1934578X1801300102. https://doi.org/10.1177/1934578X1801300102

Hiller KO, Zetler G. Neuropharmacological studies on ethanol extracts of *Valeriana officinalis* L.: behavioural and anticonvulsant properties. Phytotherapy research. 1996 Mar;10(2):145-51. https://doi.org/10.1002/(SICI)1099-1573(199603)10:2<145::AID-PTR793>3.0.CO;2-W

Himejima M, Kubo I. Antibacterial agents from the cashew *Anacardium occidentale* (Anacardiaceae) nut shell oil. Journal of Agricultural and Food Chemistry. 1991 Feb;39(2):418-21. https://doi.org/10.1021/jf00002a039

Hira A, Dey SK, Howlader MS, Ahmed A, Hossain H, Jahan IA. Anti-inflammatory and antioxidant activities of ethanolic extract of aerial parts of *Vernonia patula* (Dryand.) Merr. Asian Pacific journal of tropical biomedicine. 2013 Oct 1;3(10):798-805. https://doi.org/10.1016/S2221-1691(13)60158-6

Hisham A, Pathare N, Al-Saidi S, Al-Salmi A. The composition and antimicrobial activity of leaf essential oil of *Teucrium mascatense* Boiss. from Oman. Journal of Essential Oil Research. 2006 Jul 1;18(4):465-8. https://doi.org/10.1080/10412905.2006.9699142

Ho JY, Chang HW, Lin CF, Liu CJ, Hsieh CF, Horng JT. Characterization of the anti-influenza activity of the Chinese herbal plant *Paeonia lactiflora*. Viruses. 2014 Apr;6(4):1861-75. https://doi.org/10.3390/v6041861

Ho YL, Huang SS, Deng JS, Lin YH, Chang YS, Huang GJ. In vitro antioxidant properties and total phenolic contents of wetland medicinal plants in Taiwan. Botanical studies. 2012 Jan 1;53(1).

Holetz FB, Pessini GL, Sanches NR, Cortez DA, Nakamura CV, Dias Filho BP. Screening of some plants used in the Brazilian folk medicine for the treatment of infectious diseases. Memórias do Instituto Oswaldo Cruz. 2002 Oct;97(7):1027-31. https://doi.org/10.1590/S0074-02762002000700017

Homer KA, Manji F, Beighton D. Inhibition of protease activities of periodontopathic bacteria by extracts of plants used in Kenya as chewing sticks (mswaki). Archives of Oral Biology. 1990 Jan 1;35(6):421-4. https://doi.org/10.1016/0003-9969(90)90203-M

Hong EH, Song JH, Kang KB, Sung SH, Ko HJ, Yang H. Anti-Influenza Activity of Betulinic Acid from *Zizyphus jujuba* on Influenza A/PR/8 Virus. Biomol Ther (Seoul). 2015 Jul;23(4):345-9. doi: 10.4062/biomolther.2015.019

Hong L, Guo Z, Huang K, Wei S, Liu B, Meng S, Long C. Ethnobotanical study on medicinal plants used by Maonan people in China. Journal of ethnobiology and ethnomedicine. 2015 Dec 1;11(1):32.

Hong YH, Chao WW, Chen ML, Lin BF. Ethyl acetate extracts of alfalfa (*Medicago sativa* L.) sprouts inhibit lipopolysaccharide-induced inflammation in vitro and in vivo. Journal of Biomedical Science. 2009 Dec;16(1):64. https://doi.org/10.1186/1423-0127-16-64

Hong YJ, Yang KS. Anti-inflammatory activities of crocetin derivatives from processed Gardenia jasminoides. Archives of pharmacal research. 2013 Aug 1;36(8):933-40. https://doi.org/10.1007/s12272-013-0128-0

Horiuchi K, Shiota S, Hatano T, Yoshida T, Kuroda T, Tsuchiya T. Antimicrobial activity of oleanolic acid from *Salvia officinalis* and related compounds on vancomycin-resistant enterococci (VRE). Biological and Pharmaceutical Bulletin. 2007;30(6):1147-9. https://doi.org/10.1248/bpb.30.1147

Hossain MA, Rahman MS, Chowdhury AS, Rashid MA. Bioactivities of *Sesbania sesban* Extractives. Dhaka University Journal of Pharmaceutical Sciences. 2007;6(1):61-3.

Hossain MS, Uddin MS, Asaduzzaman M, Munira MS, Uddin MJ, Rafe MR, Mosiqur M. Inquiry of analgesic and anti-inflammatory activities of *Xanthosoma sagittifolium* L.: An effective medicinal plant. Journal of Coastal Life Medicine. 2017;5(1):22-6. doi: 10.12980/jclm.5.2017J6-229

Hossan MS, Fatima A, Rahmatullah M, Khoo TJ, Nissapatorn V, Galochkina AV, Slita AV, Shtro AA, Nikolaeva Y, Zarubaev VV, Wiart C. Antiviral activity of *Embelia ribes* Burm. f. against influenza virus in vitro. Archives of virology. 2018 Aug 1;163(8):2121-31. https://doi.org/10.1007/s00705-018-3842-6

Hou J, Gu Y, Zhao S, Huo M, Wang S, Zhang Y, Qiao Y, Li X. Anti-Inflammatory Effects of Aurantio-Obtusin from Seed of *Cassia obtusifolia* L. through Modulation of the NF-κB Pathway. Molecules. 2018 Nov 27;23(12):3093. doi: 10.3390/molecules23123093. Erratum in: Molecules. 2019 Feb 19;24(4).

Houghton PJ, Manby J. Medicinal plants of the Mapuche. Journal of Ethnopharmacology. 1985 Mar 1;13(1):89-103. https://doi.org/10.1016/0378-8741(85)90063-7

Houshmand G, Tarahomi S, Arzi A, Goudarzi M, Bahadoram M, Rashidi-Nooshabadi M. Red Lentil Extract: Neuroprotective Effects on Perphenazine Induced Catatonia in Rats. J Clin Diagn Res. 2016 Jun;10(6):FF05-8. doi: 10.7860/JCDR/2016/17813.7977.

Hritcu L, Noumedem JA, Cioanca O, Hancianu M, Kuete V, Mihasan M. Methanolic extract of *Piper nigrum* fruits improves memory impairment by decreasing brain oxidative stress in amyloid beta (1–42) rat model of Alzheimer's disease. Cellular and molecular neurobiology. 2014 Apr 1;34(3):437-49.

Hu M, Du J, Du L, Luo Q, Xiong J. Anti-fatigue activity of purified anthocyanins prepared from purple passion fruit (P. edulis Sim) epicarp in mice. Journal of Functional Foods. 2020 Feb 1;65:103725. https://doi.org/10.1016/j.jff.2019.103725

Hu W, Wu L, Qiang Q, Ji L, Wang X, Luo H, Wu H, Jiang Y, Wang G, Shen T. The dichloromethane fraction from *Mahonia bealei* (Fort.) Carr. leaves exerts an anti-inflammatory effect both in vitro and in vivo. Journal of ethnopharmacology. 2016 Jul 21;188:134-43. https://doi.org/10.1016/j.jep.2016.05.013

Hu Y, Zhang QY, Hou TT, Xin HL, Zheng HC, Rahman K, Qin LP. Estrogen-like activities in *Vitex* species from China determined by a cell based proliferation assay. Die Pharmazie. 2007 Nov 1;62(11):872-5

Huang M, Jiang X, Liang Y, Liu Q, Chen S, Guo Y. Berberine improves cognitive impairment by promoting autophagic clearance and inhibiting production of beta-amyloid in APP/tau/PS1 mouse model of Alzheimer's disease. Exp Gerontol. 2017;91:25–33. doi: 10.1016/j.exger.2017.02.004. [PubMed][Cross Ref]

Huang MH, Huang SS, Wang BS, Wu CH, Sheu MJ, Hou WC, Lin SS, Huang GJ. Antioxidant and anti-inflammatory properties of *Cardiospermum halicacabum* and its reference compounds ex vivo and in vivo. Journal of ethnopharmacology. 2011 Jan 27;133(2):743-50. doi.org/10.1016/j.jep.2010.11.005

Huang P, Xu Y, Wei R, Li H, Tang Y, Liu J, Zhang SS, Zhang C. Efficacy of tetrandrine on lowering intraocular pressure in animal model with ocular hypertension. Journal of glaucoma. 2011 Mar 1;20(3):183-8. doi: 10.1097/IJG.0b013e3181d7882a

Huang W, Gao F, Hu F, Huang J, Wang M, Xu P, Zhang R, Chen J, Sun X, Zhang S, Wu J. Asiatic acid prevents retinal ganglion cell apoptosis in a rat model of glaucoma. Frontiers in neuroscience. 2018 Jul 20;12:489. doi:10.3389/fnins.2018.00489

Huang WY, Wu H, Li DJ, Song JF, Xiao YD, Liu CQ, Zhou JZ, Sui ZQ. Protective effects of blueberry anthocyanins against H2O2-induced oxidative injuries in human retinal pigment epithelial cells. Journal of agricultural and food chemistry. 2018 Feb 21;66(7):1638-48. https://doi.org/10.1021/acs.jafc.7b06135

Huerta-Reyes M, del Carmen Basualdo M, Abe F, Jimenez-Estrada M, Soler C, Reyes-Chilpa R. HIV-1 inhibitory compounds from *Calophyllum brasiliense* leaves. Biological and Pharmaceutical Bulletin. 2004;27(9):1471-5. https://doi.org/10.1248/bpb.27.1471

Huo HX, Gu YF, Sun H, Zhang YF, Liu WJ, Zhu ZX, Shi SP, Song YL, Jin HW, Zhao YF, Tu PF. Anti-inflammatory 2-(2-phenylethyl) chromone derivatives from Chinese agarwood. Fitoterapia. 2017 Apr 1;118:49-55. https://doi.org/10.1016/j.fitote.2017.02.009

Hurtado JD, Rubio ZM, Berrospi LF, Azahuanche FR. Antibacterial activity of *Pelargonium peltatum* (L.) L'Her. against *Streptococcus mutans, Streptococcus sanguis* and *Streptococcus mitis* versus chlorhexidine. Revista Cubana de Plantas Medicinales. 2013;18(2):224-36.

Hurtado-Díaz I, Sánchez-Carranza JN, Romero-Estrada A, González-Maya L, González-Christen J, Herrera-Ruiz M, Alvarez L. 16-hydroxy-lycopersene, a polyisoprenoid alcohol isolated from *Tournefortia hirsutissima*, inhibits nitric oxide production in RAW 264.7 cells and induces apoptosis in Hep3B cells. Molecules. 2019 Jan;24(13):2366. doi.org/10.3390/molecules24132366

Hussain A, Ahmad N, Qarshi IA, Rashid M, Shinwari ZK. Inhibitory potential of nine *Mentha* species against pathogenic bacterial strains. Pak. J. Bot. 2015 Dec 1;47(6):2427-33.

Hussain AI, Anwar F, Chatha SA, Latif S, Sherazi ST, Ahmad A, Worthington J, Sarker SD. Chemical composition and bioactivity studies of the essential oils from two *Thymus* species from the Pakistani flora. LWT-Food Science and Technology. 2013 Jan 1;50(1):185-92.

Hussain AI, Anwar F, Sherazi ST, Przybylski R. Chemical composition, antioxidant and antimicrobial activities of basil (*Ocimum basilicum*) essential oils depends on seasonal variations. Food chemistry. 2008 Jun 1;108(3):986-95.

Hussain F, Ahmad B, Hameed I, Dastagir G, Sanaullah P, Azam S. Antibacterial, antifungal and insecticidal activities of some selected medicinal plants of polygonaceae. African Journal of Biotechnology. 2010;9(31):5032-6.

Hussain J, Ali L, Khan AL, Rehman NU, Jabeen F, Kim JS, Al-Harrasi A. Isolation and bioactivities of the flavonoids morin and morin-3-O-β-D-glucopyranoside from *Acridocarpus orientalis*—A wild Arabian medicinal plant. Molecules. 2014 Nov;19(11):17763-72. https://doi.org/10.3390/molecules191117763

Hussain J, Jamila N, Gilani SA, Abbas G, Ahmed S. Platelet aggregation, antiglycation, cytotoxic, phytotoxic and antimicrobial activities of extracts of *Nepeta juncea.* African Journal of Biotechnology. 2009;8(6).

Hussain M, Bakhsh H, Aziz A, Majeed A, Khan IA, Mujeeb A, Farooq U. Comparative In vitro study of antimicrobial activities of flower and whole plant of *Jasminum officinale* against some human pathogenic microbes. J Pharm Alternative Med. 2013;2:33-43.

Hwang KA, Hwang YJ, Song J. Antioxidant activities and oxidative stress inhibitory effects of ethanol extracts from *Cornus officinalis* on raw 264.7 cells. BMC complementary and alternative medicine. 2016 Dec;16(1):196. doi.org/10.1186/s12906-016-1172-3

Hwang SB, Chang MN, Garcia ML, Han QQ, Huang L, King VF, Kaczorowski GJ, Winquist RJ. L-652,469-a dual receptor antagonist of platelet activating factor and dihydropyridines from *Tussilago farfara* L. European journal of pharmacology. 1987 Sep 11;141(2):269-81. https://doi.org/10.1016/0014-2999(87)90272-X

Hwang YP, Jeong HG. The coffee diterpene kahweol induces heme oxygenase-1 via the PI3K and p38/Nrf2 pathway to protect human dopaminergic neurons from 6-hydroxydopamine-derived oxidative stress. Febs Letters. 2008 Jul 23;582(17):2655-62. https://doi.org/10.1016/j.febslet.2008.06.045

Hyun SH, Ahn HY, Kim HJ, Kim SW, So SH, In G, Park CK, Han CK. Immuno-enhancement effects of Korean Red Ginseng in healthy adults: a randomized, double-blind, placebo-controlled trial. Journal of ginseng research. 2021 Jan 1;45(1):191-8. https://doi.org/10.1016/j.jgr.2020.08.003

Hyun, S.H., Ahn, H.Y., Kim, H.J., Kim, S.W., So, S.H., In, G., Park, C.K. and Han, C.K., 2021. Immuno-enhancement effects of Korean Red Ginseng in healthy adults: a randomized, double-blind, placebo-controlled trial. Journal of ginseng research, *45*(1), pp.191-198.

Iannarelli R, Marinelli O, Morelli MB, Santoni G, Amantini C, Nabissi M, Maggi F. Aniseed (*Pimpinella anisum* L.) essential oil reduces pro-inflammatory cytokines and stimulates mucus secretion in primary airway bronchial and tracheal epithelial cell lines. Industrial crops and products. 2018 Apr 1;114:81-6. doi.org/10.1016/j.indcrop.2018.01.076

Iannello C, Bastida J, Bonvicini F, Antognoni F, Gentilomi GA, Poli F. Chemical composition, and in vitro antibacterial and antifungal activity of an alkaloid extract from *Crinum angustum* Steud. Natural product research. 2014 May 19;28(10):704-10. doi.org/10.1080/14786419.2013.877903

Ibewuike JC, Ogungbamila FO, Ogundaini AO, Okeke IN, Bohlin L. Antiinflammatory and antibacterial activities of C-methylflavonols from *Piliostigma thonningii*. Phytotherapy Research: An International Journal Devoted to Medical and Scientific Research on Plants and Plant Products. 1997 Jun;11(4):281-4. https://doi.org/10.1002/(SICI)1099-1573(199706)11:4<281::AID-PTR281>3.0.CO;2-9

Ibrahim AM, Lawal B, Abubakar AN, Tsado NA, Kontagora GN, Gboke JA, Berinyuy EB. Antimicrobial and free radical scavenging potentials of N-hexane and ethyl acetate fractions of *Phyllanthus fraternus*. Nigerian Journal of Basic and Applied Sciences. 2017;25(2):6-11.

Iadecola C, Yaffe K, Biller J, Bratzke LC, Faraci FM, Gorelick PB, et al. Impact of Hypertension on Cognitive Function: A Scientific Statement From the American Heart Association. Hypertension. 2016;68(6):e67-e94. https://doi.org/10.1161/HYP.0000000000000053

Ibrahim AY, El-Gengaihi SE, Motawea HM, Sleem AA. Anti-inflammatory activity of *Salvadora persica* L. against carrageenan induced paw oedema in rat relevant to inflammatory cytokines. Notulae Scientia Biologicae. 2011 Nov 17;3(4):22-8. https://doi.org/10.15835/nsb346378

Ibrahim B, Sowemimo A, van Rooyen A, Van de Venter M. Antiinflammatory, analgesic and antioxidant activities of *Cyathula prostrata* (Linn.) Blume (Amaranthaceae). Journal of ethnopharmacology. 2012 May 7;141(1):282-9. https://doi.org/10.1016/j.jep.2012.02.032

Ichimura T, Yamanaka A, Ichiba T, Toyokawa T, Kamada Y, Tamamura T, Maruyama S. Antihypertensive effect of an extract of *Passiflora edulis* rind in spontaneously hypertensive rats. Bioscience, biotechnology, and biochemistry. 2006;70(3):718-21. https://doi.org/10.1271/bbb.70.718

Idris FN, Nadzir MM. Antimicrobial activity of *Centella asiatica* on *Aspergillus niger* and *Bacillus subtilis.* Chemical Engineering Transactions. 2017 Mar 20;56:1381-6.

Idu M, Ovuakporie-Uvo O, Obayagbona NO, Ogiemudia O. Phytochemistry, Antimicrobial and Toxicological Studies of *Greenwayodendron suaveolens* Seed Extracts. Journal of Basic Pharmacology and Toxicology. 2017 Jan 16;1(1):8-12.

Idu ME, Omogbai EK, Amechina F, Ataman JE. Some cardiovascular effects of the acqeous exract of the leaves of Stachytarpheta jamaicensis. L. vahl. Int J Pharmacol. 2006;2:163-5. doi: 10.3923/ijp.2006.163.165

Igbinosa OO, Igbinosa EO, Aiyegoro OA. Antimicrobial activity and phytochemical screening of stem bark extracts from *Jatropha curcas* (Linn). African journal of pharmacy and pharmacology. 2009 Feb 28;3(2):058-62.

Iglesias R, Citores L, Ragucci S, Russo R, Di Maro A, Ferreras JM. Biological and antipathogenic activities of ribosome-inactivating proteins from *Phytolacca dioica* L. Biochimica et Biophysica Acta (BBA)-General Subjects. 2016 Jun 1;1860(6):1256-64. https://doi.org/10.1016/j.bbagen.2016.03.011

Igwe OU, Onwu FK. Leaf essential oil of *Senna alata* Linn from South East Nigeria and its Antimicrobial activity. International Journal of Research in Pharmacy and Chemistry. 2015;5(1):27-33.

Ihsan MF, Aspriyanto D, Erlita I. The comparison of plaque index before and after rinsing with fuji apple (*Malus sylvestris*) extract 100% and chlorhexidine 0, 2% in children aged 8-10 years. Dentino. 2018 Sep 28;3(2):156-61. http://dx.doi.org/10.20527/dentino.v3i2.5371

Iida Y, Oh KB, Saito M, Matsuoka H, Kurata H, Natsume M, Abe H. Detection of antifungal activity in *Anemarrhena asphodeloides* by sensitive BCT method and isolation of its active compound. Journal of agricultural and food chemistry. 1999 Feb 15;47(2):584-7.

Ikewuchi JC, Ikewuchi CC, Eriyamremu GE. Effect of *Acalypha wilkesiana* Muell Arg on the blood pressure and aorta contractility of salt-loaded rats. Pac J Sci Technol. 2009;10(2):829-34.

Ikobi EU, Igwilo CI, Awodele O, Azubuike PC. Antibacterial and wound healing properties of methanolic extract of dried fresh *Gossypium barbadense* leaves. Asian Journal of Biomedical and Pharmaceutical Sciences. 2012 Jan 1;2(13):32.

Ilavarasan R, Malika M, Venkataraman S. Anti-inflammatory and antioxidant activities of *Cassia fistula* Linn bark extracts. African Journal of Traditional, Complementary and Alternative Medicines. 2005;2(1):70-85.

Ilic NM, Dey M, Poulev AA, Logendra S, Kuhn PE, Raskin I. Anti-inflammatory activity of grains of paradise (*Aframomum melegueta* Schum) extract. Journal of agricultural and food chemistry. 2014 Oct 20;62(43):10452-7.

Im JS, Lee HN, Oh JW, Yoon YJ, Park JS, Park JW, Kim JH, Kim YS, Cha DS, Jeon H. *Moringa oleifera* prolongs lifespan via DAF-16/FOXO transcriptional factor in *Caenorhabditis elegans*. Natural Product Sciences. 2016;22(3):201-8. http://dx.doi.org/10.20307/nps.2016.22.3.201

Im NK, Jung YS, Choi JH, Yu MH, Jeong GS. Inhibitory effect of the leaves of Rumex crispus L. on LPS-induced nitric oxide production and the expression of iNOS and COX-2 in macrophages. Natural Product Sciences. 2014;20(1):51-7.

Inam A, Shahzad M, Shabbir A, Shahid H, Shahid K, Javeed A. *Carica papaya* ameliorates allergic asthma via down regulation of IL-4, IL-5, eotaxin, TNF-α, NF-κB, and iNOS levels. Phytomedicine. 2017 Aug 15;32:1-7. doi.org/10.1016/j.phymed.2017.04.009

Ingolfsdottir K, Bloomfield SF, Hylands PJ. In vitro evaluation of the antimicrobial activity of lichen metabolites as potential preservatives. Antimicrobial agents and chemotherapy. 1985 Aug 1;28(2):289-92. doi: 10.1128/AAC.28.2.289

Innocenti G, Dall'Acqua S, Scialino G, Banfi E, Sosa S, Gurung K, Barbera M, Carrara M. Chemical composition and biological properties of *Rhododendron anthopogon* essential oil. Molecules. 2010 Apr;15(4):2326-38. https://doi.org/10.3390/molecules15042326

Intharachatorn T, Srisawat R. Antihypertensive effects of *Centella asiatica* extract. InInternational Conference on Food and Agricultural Sciences 2013 55(23):122-126.

Ionita R, Postu PA, Mihasan M, Gorgan DL, Hancianu M, Cioanca O, Hritcu L. Ameliorative effects of *Matricaria chamomilla* L. hydroalcoholic extract on scopolamine-induced memory impairment in rats: A behavioral and molecular study. Phytomedicine. 2018 Aug 1;47:113-20. https://doi.org/10.1016/j.phymed.2018.04.049

Irobi ON, Moo-Young M, Anderson WA, Daramola SO. Antimicrobial activity of bark extracts of *Bridelia ferruginea* (Euphorbiaceae). Journal of Ethnopharmacology. 1994 Jul 22;43(3):185-90.

Isa NM, Abdelwahab SI, Mohan S, Abdul AB, Sukari MA, Taha MM, Syam S, Narrima P, Cheah SC, Ahmad S, Mustafa MR. In vitro anti-inflammatory, cytotoxic and antioxidant activities of boesenbergin A, a chalcone isolated from *Boesenbergia rotunda* (L.)(fingerroot). Brazilian Journal of Medical and Biological Research. 2012 Jun;45(6):524-30.

Isah AO, Idu M, Abdulrahman AA, Amaechina F. Evaluation of in-vitro vasorelaxant effect (potential antihypertensive) of *Kigelia africana* fruit methanol extract on potassium chloride and phenylephrine induced tension in wistar rat aorta. FUDMA Journal of Sciences. 2020 Sep 29;4(3):470-5.

Ishigaki Y, Tanaka H, Akama H, Ogara T, Uwai K, Tokuraku K. A microliter-scale high-throughput screening system with quantum-dot nanoprobes for amyloid-β aggregation inhibitors. PloS one. 2013 Aug 26;8(8):e72992. doi:10.1371/journal.pone.0072992

Ishii S, Katsumura T, Shiozuka C, Ooyauchi K, Kawasaki K, Takigawa S, Fukushima T, Tokuji Y, Kinoshita M, Ohnishi M, Kawahara M. Anti-inflammatory effect of buckwheat sprouts in lipopolysaccharide-activated human colon cancer cells and mice. Bioscience, biotechnology, and biochemistry. 2008 Dec 23;72(12):3148-57. https://doi.org/10.1271/bbb.80324

Ishola IO, Awoyemi AA, Afolayan GO. Involvement of antioxidant system in the amelioration of scopolamine-induced memory impairment by grains of paradise (*Aframomum melegueta* K. Schum.) extract. Drug research. 2016 Sep;66(09):455-63.

Ishola IO, Olayemi SO, Yemitan OK, Ekpemandudiri NK. Mechanisms of anticonvulsant and sedative actions of the ethanolic stem-bark extract of *Ficus sur* Forssk (Moraceae) in rodents. Pak J Biol Sci. 2013 Nov 1;16(21):1287-94. doi: 10.3923/pjbs.2013.1287.1294

Ishtiaq S, Ali T, Ahmad B, Anwar F, Afridi MS, Shaheen H. Phytochemical and biological evaluations of methanolic extract of *Amaranthus graecizans* subsp. *silvestris* (Vill.) Brenan. Journal of Pharmaceutical Research International. 2017 Mar 29:1-1.

Islam MA, Ahmed F, Das AK, Bachar SC. Analgesic and anti-inflammatory activity of *Leonurus sibiricus*. Fitoterapia. 2005 Jun 1;76(3-4):359-62.

Islam MT, Priyanka AK, Sultana T, Kawsar MH, Sumon MH, Sohel MD. In Vitro Antimicrobial, Antioxidant and Cytotoxic activities of *Polygonum orientale* (Bishkatali). Journal of Pharmacy and Nutrition Sciences. 2016;6:112-9.

Islam SM, Ahmed KT, Manik MK, Wahid MA, Kamal CS. A comparative study of the antioxidant, antimicrobial, cytotoxic and thrombolytic potential of the fruits and leaves of *Spondias dulcis*. Asian Pacific journal of tropical biomedicine. 2013 Sep 1;3(9):682-91. https://doi.org/10.1016/S2221-1691(13)60139-2

Ismaili H, Milella L, Fkih-Tetouani S, Ilidrissi A, Camporese A, Sosa S, Altinier G, Della Loggia R, Aquino R. In vivo topical anti-inflammatory and in vitro antioxidant activities of two extracts of *Thymus satureioides* leaves. Journal of ethnopharmacology. 2004 Mar 1;91(1):31-6. https://doi.org/10.1016/j.jep.2003.11.013

Issa MY, Ezzat MI, Sayed RH, Elbaz EM, Omar FA, Mohsen E. Neuroprotective effects of *Pulicaria undulata* essential oil in rotenone model of parkinson's disease in rats: Insights into its anti-inflammatory and anti-oxidant effects. South African Journal of Botany. 2020 Aug 1;132:289-98. https://doi.org/10.1016/j.sajb.2020.04.032

Itharat A. Comparative biological activities of five Thai medicinal plants called Hua-Khao-Yen. Thai Journal of Pharmacology. 2010;32(1):327-31.

Itou RD, Sanogo R, Ossibi AW, Ntandou FG, Ondelé R, Pénemé BM, Andissa NO, Diallo D, Ouamba JM, Abena AA. Anti-inflammatory and analgesic effects of aqueous extract of stem bark of *Ceiba pentandra* Gaertn. Pharmacology & Pharmacy. 2014 Nov 5;5(12):1113.

Itzhaki RF, Lathe R, Balin BJ, Ball MJ, Bearer EL, Braak H, et al. Microbes and Alzheimer's Disease. J Alzheimers Dis. 2016;51(4):979-84. https://doi.org/10.3233/JAD-160152

Ivanova D, Vankova D, Nashar M. *Agrimonia eupatoria* tea consumption in relation to markers of inflammation, oxidative status and lipid metabolism in healthy subjects. Arch Physiol Biochem. 2013 Feb;119(1):32-7. doi: 10.3109/13813455.2012.729844.

Ivanovska N, Philipov S. Study on the anti-inflammatory action of *Berberis vulgaris* root extract, alkaloid fractions and pure alkaloids. International journal of immunopharmacology. 1996 Oct 1;18(10):553-61. https://doi.org/10.1016/S0192-0561(96)00047-1

Iwamoto LH, Vendramini-Costa DB, Monteiro PA, Ruiz AL, Sousa IM, Foglio MA, de Carvalho JE, Rodrigues RA. Anticancer and anti-inflammatory activities of a standardized dichloromethane extract from *Piper umbellatum* L. leaves. Evidence-Based Complementary and Alternative Medicine. 2015;2015. https://doi.org/10.1155/2015/948737

Iwo MI, Soemardji AA, Retnoningrum DS. Immunostimulating effect of pule (*Alstonia scholaris* LR Br., Apocynaceae) bark extracts. Clinical hemorheology and microcirculation. 2000 Jan 1;23(2, 3, 4):177-83.

Iyda JH, Fernandes Â, Ferreira FD, Alves MJ, Pires TC, Barros L, Amaral JS, Ferreira IC. Chemical composition and bioactive properties of the wild edible plant *Raphanus raphanistrum* L. Food Research International. 2019 Jul 1;121:714-22. https://doi.org/10.1016/j.foodres.2018.12.046

Jabbari M, Hashempur MH, Razavi SZ, Shahraki HR, Kamalinejad M, Emtiazy M. Efficacy and short-term safety of topical Dwarf Elder (*Sambucus ebulus* L.) versus diclofenac for knee osteoarthritis: A randomized, double-blind, active-controlled trial. J Ethnopharmacol. 2016 Jul 21;188:80-6. doi: 10.1016/j.jep.2016.04.035.

Jaca TP, Kambizi L. Antibacterial properties of some wild leafy vegetables of the Eastern Cape Province, South Africa. Journal of Medicinal Plants Research. 2011 Jul 4;5(13):2624-8.

Jachak SM, Gautam R, Selvam C, Madhan H, Srivastava A, Khan T. Anti-inflammatory, cyclooxygenase inhibitory and antioxidant activities of standardized extracts of *Tridax procumbens* L. Fitoterapia. 2011 Mar 1;82(2):173-7. https://doi.org/10.1016/j.fitote.2010.08.016

Jacquet A, Girodet PO, Pariente A, Forest K, Mallet L, Moore N. Phytalgic, a food supplement, vs placebo in patients with osteoarthritis of the knee or hip: a randomised double-blind placebo-controlled clinical trial. *Arthritis Res Ther*. 2009;11(6):R192. doi:10.1186/ar2891

Jadhav SA, Prasanna SM. Evaluation of antiulcer activity of *Ziziphus oenoplia* (L) Mill. roots in rats. Asian J Pharm Clin Res. 2011;1(1):92-5.

Jaeger Greer MR, Cates RG, Johnson FB, Lamnaouer D, Ohai L. Activity of acetone and methanol extracts from thirty-one medicinal plant species against *Herpes simplex* virus types 1 and 2. Pharmaceutical biology. 2010 Sep 1;48(9):1031-7. doi: 10.3109/13880200903468873.

Jafarzadeh, A., Mohammadi-Kordkhayli, M., Ahangar-Parvin, R., Azizi, V., Khoramdel-Azad, H., Shamsizadeh, A., Ayoobi, A., Nemati, M., Hassan, Z.M., Moazeni, S.M. and Khaksari, M., 2014. Ginger extracts influence the expression of IL-27 and IL-33 in the central nervous system in experimental autoimmune encephalomyelitis and ameliorates the clinical symptoms of disease. *Journal of neuroimmunology*, *276*(1-2), pp.80-88.

Jaffri JM, Mohamed S, Rohimi N, Ahmad IN, Noordin MM, Manap YA. Antihypertensive and cardiovascular effects of catechin-rich oil palm (*Elaeis guineensis*) leaf extract in nitric oxide–deficient rats. Journal of medicinal food. 2011 Jul 1;14(7-8):775-83. https://doi.org/10.1089/jmf.2010.1170

Jagtap SS, Satpute RA, Rahatgaonkar AM, Lanjewar KR. Phytochemical screening, antioxidant, antimicrobial and quantitative multi-elemental analysis of *Habenaria longicorniculata* J. Graham. J Acad Ind Res. 2014 Aug;3:108-7.

Jain A, Sharma S, Goyal M, Dubey S, Jain S, Sahu J, Sharma A, Kaushik A. Anti-inflammatory activity of *Syzygium cumini* leaves. International journal of phytomedicine. 2010 Apr 1;2(2). doi:10.5138/ijpm.2010.0975.0185.02019

Jain DL, Baheti AM, Jain SR, Khandelwal KR. Use of medicinal plants among tribes in Satpuda region of Dhule and Jalgaon districts of Maharashtra—an ethnobotanical survey. Indian Journal of Traditional Knowledge2010;9:152-157.

Jain P, Bansal D, Bhasin PA, Anjali A. Antimicrobial activity and phytochemical screening of five wild plants against *Escherichia coli, Bacillus subtilis* and *Staphylococcus aureus*. J Pharm Res. 2010 Jun;3(6):1260-2.

Jakkala LK, Ali SA. Amelioration of the toxic effects of aluminium induced neurodegenerative changes in brain of albino rats by *Aloe vera*. Journal of Global Biosciences. 2015;4(8):3171-7.

Jalalpure SS, Bagewadi V, Shaikh I. Antiepileptic Effect of *Asparagus racemosus* Root Extracts. Journal of Tropical Medicinal Plants. 2009 Dec 1;10(2).

Jamkhande PG, Wattamwar AS, Pekamwar SS, Chandak PG. Antioxidant, antimicrobial activity and in silico PASS prediction of *Annona reticulata* Linn. root extract. Beni-Suef University Journal of Basic and Applied Sciences. 2014 Jun 1;3(2):140-8. https://doi.org/10.1016/j.bjbas.2014.05.008

Jang DS, Cuendet M, Su BN, Totura S, Riswan S, Fong HH, Pezzuto JM, Kinghorn AD. Constituents of the seeds of *Hernandia ovigera* with inhibitory activity against cyclooxygenase-2. Planta medica. 2004 Oct;70(10):893-6. doi: 10.1055/s-2004-832612

Jang JY, Kim TS, Cai J, Kim J, Kim Y, Shin K, Kim KS, Lee SP, Kang MH, Choi EK, Rhee MH. *Perilla* oil improves blood flow through inhibition of platelet aggregation and thrombus formation. Laboratory animal research. 2014 Mar 1;30(1):21-7. https://doi.org/10.5625/lar.2014.30.1.21

Jangid AK, Pooja D, Kulhari H. Determination of solubility, stability and degradation kinetics of morin hydrate in physiological solutions. RSC advances. 2018;8(50):28836-42.

Jarić S, Mačukanović-Jocić M, Djurdjević L, Mitrović M, Kostić O, Karadžić B, Pavlović P. An ethnobotanical survey of traditionally used plants on Suva planina mountain (south-eastern Serbia). Journal of ethnopharmacology. 2015 Dec 4;175:93-108. https://doi.org/10.1016/j.jep.2015.09.002

Jarrahi M. An experimental study of the effects of *Matricaria chamomilla* extract on cutaneous burn wound healing in albino rats. Natural product research. 2008 Mar 20;22(5):422-7. https://doi.org/10.1080/14786410701591713

Jasbleidy MB, Rubén TG, Luis PO, Vanessa CG. Anti-inflammatory effect of the hydroalcoholic extract of *Muehlenbeckia tamnifolia* (Kunth) meins leaves in a rat paw model. *Pharmacology*OnLine 2017 Archives December (3):13-22.

Jasim RA. Anti-Microbial Activity and Screening Analysis by FT-IR Spectroscopic of Methanolic Extract of Selected Medicinal plant of *Brassica rapa* and *Cichorium endivia*. Annals of the Romanian Society for Cell Biology. 2021 May 13:15779-90.

Jatsa HB, Feussom NG, Nkondo ET, Kenfack MC, Simo ND, Fassi JB, Femoe UM, Moaboulou C, Tsague CD, Dongo E, Kamtchouing P. Efficacy of *Ozoroa pulcherrima* Schweinf methanolic extract against *Schistosoma mansoni*-induced liver injury in mice. Journal of Traditional and Complementary Medicine. 2019 Oct 1;9(4):304-11. https://doi.org/10.1016/j.jtcme.2017.08.009

Javed T, Ashfaq UA, Riaz S, Rehman S, Riazuddin S. In-vitro antiviral activity of *Solanum nigrum* against Hepatitis C Virus. Virology Journal. 2011 Dec;8(1):1-7. https://doi.org/10.1186/1743-422X-8-26

Javidnia K, Miri R, Najafi RB, Jahromi NK. A preliminary study on the biological activity of *Daphne mucronata* royle. DARU J Pharm Sc 2003; 11(1): 28-31.

Jayakumari S. Formulation and evaluation of herbal gel from tannin-enriched fraction of *Psidium guajava* Linn. leaves for diabetic wound healing. International Journal of Green Pharmacy (IJGP). 2018 Nov 6;12(03). http://dx.doi.org/10.22377/ijgp.v12i03.2009

Jayaprakasha GK, Negi PS, Sikder S, Mohanrao LJ, Sakariah KK. Antibacterial activity of *Citrus reticulata* peel extracts. Zeitschrift für Naturforschung C. 2000 Dec 1;55(11-12):1030-4. doi.org/10.1515/znc-2000-11-1230

Jayaraman S, Manoharan MS, Illanchezian S. In-vitro antimicrobial and antitumor activities of *Stevia rebaudiana* (Asteraceae) leaf extracts. Tropical Journal of Pharmaceutical Research. 2008;7(4):1143-9. doi: 10.4314/tjpr.v7i4.14700

Jayaraman SK, Manoharan MS, Illanchezian S. Antibacterial, antifungal and tumor cell suppression potential of *Morinda citrifolia* fruit extracts. International Journal of Integrative Biology. 2008 Jun;3(1):44-9.

Jayasinghe CD, Gunasekera DS, De Silva N, Jayawardena KK, Udagama PV. Mature leaf concentrate of Sri Lankan wild type *Carica papaya* Linn. modulates nonfunctional and functional immune responses of rats. BMC complementary and alternative medicine. 2017 Dec;17(1):1-4. https://doi.org/10.1186/s12906-017-1742-z

Jdidi H, Ghorbel Koubaa F, Aoiadni N, Elleuch A, Makni-Ayadi F, El Feki A. Effect of *Medicago sativa* compared To 17β-oestradiol on osteoporosis in ovariectomized mice. Arch Physiol Biochem. 2020 Mar 20:1-8. doi: 10.1080/13813455.2020.1741644

Jegede IA, Nwinyi FC, Muazzam I, Akumka DD, Njan AA, Shok M. Micromorphological, anti-nociceptive and antiinflammatory investigations of stem bark of *Daniellia oliveri*. African Journal of Biotechnology. 2006;5(10).

Jeong EY, Sung BK, Song HY, Yang JY, Kim DK, Lee HS. Antioxidative and antimicrobial activities of active materials derived from *Triticum aestivum* sprouts. Journal of the Korean Society for Applied Biological Chemistry. 2010 Aug 1;53(4):519-24. https://doi.org/10.3839/jksabc.2010.080

Jeong KH, Jeon MT, Kim HD, Jung UJ, Jang MC, Chu JW, Yang SJ, Choi IY, Choi MS, Kim SR. Nobiletin protects dopaminergic neurons in the 1-methyl-4-phenylpyridinium-treated rat model of Parkinson's disease. Journal of medicinal food. 2015a Apr 1;18(4):409-14. https://doi.org/10.1089/jmf.2014.3241

Jeong KH, Jung UJ, Kim SR. Naringin Attenuates Autophagic Stress and Neuroinflammation in Kainic Acid-Treated Hippocampus *In Vivo* . *Evidence-based Complementary and Alternative Medicine : eCAM*. 2015b;2015:354326. doi: 10.1155/2015/354326.

Jettanacheawchankit S, Sasithanasate S, Sangvanich P, Banlunara W, Thunyakitpisal P. Acemannan stimulates gingival fibroblast proliferation; expressions of keratinocyte growth factor-1, vascular endothelial growth factor, and type I collagen; and wound healing. Journal of pharmacological sciences. 2009;109(4):525-31. https://doi.org/10.1254/jphs.08204FP

Jeyabalan S, Palayan M. Analgesic and anti-inflammatory activity of leaves of *Morinda tinctoria* Roxb. International Journal of Pharmaceutical Research. 2009;1(4): 74-80.

Jeyachandran R, Mahesh A, Cindrella L, Sudhakar S, Pazhanichamy K. Antibacterial activity of Plumbagin and root extracts of *Plumbago zeylanica*. Acta Biologica Cracoviensia Series Botanica. 2009 Jan 1;51(1):17-22.

Ji C, Song C, Akber Aisa H, Yang N, Liu YY, Li Q, Zhu HB, Zuo PP. *Gossypium herbaceum* L. extracts ameliorate disequilibrium of IL-1RA/IL-1β ratio to attenuate inflammatory process induced by amyloid β in rats. Current Alzheimer Research. 2012 Oct 1;9(8):953-61. https://doi.org/10.2174/156720512803251093

Ji H, Shengji P, Chunlin L. An ethnobotanical study of medicinal plants used by the Lisu people in Nujiang, northwest Yunnan, China. Economic botany. 2004 Dec 1;58(1):S253-64. https://doi.org/10.1663/0013-0001(2004)58[S253:AESOMP]2.0.CO;2

Jia YY, Guan RF, Wu YH, Yu XP, Lin WY, Zhang YY, Liu T, Zhao J, Shi SY, Zhao Y. *Taraxacum mongolicum* extract exhibits a protective effect on hepatocytes and an antiviral effect against hepatitis B virus in animal and human cells. Molecular Medicine Reports. 2014 Apr 1;9(4):1381-7. https://doi.org/10.3892/mmr.2014.1925

Jian R, Zeng KW, Li J, Li N, Jiang Y, Tu P. Anti-neuroinflammatory constituents from *Asparagus cochinchinensis.* Fitoterapia. 2013 Jan 1;84:80-4. https://doi.org/10.1016/j.fitote.2012.10.011

Jiang J, Xu Q. Immunomodulatory activity of the aqueous extract from rhizome of *Smilax glabra* in the later phase of adjuvant-induced arthritis in rats. Journal of Ethnopharmacology. 2003 Mar 1;85(1):53-9. https://doi.org/10.1016/S0378-8741(02)00340-9

Jiang Q, Zhang H, Yang R, Hui Q, Chen Y, Mats L, Tsao R, Yang C. Red-Osier Dogwood Extracts Prevent Inflammatory Responses in Caco-2 Cells and a Caco-2 BBe1/EA. hy926 Cell Co-Culture Model. Antioxidants. 2019 Oct;8(10):428. https://doi.org/10.3390/antiox8100428

Jiang W, Wei W, Gaertig MA, Li S, Li X-J (2015) Therapeutic Effect of Berberine on Huntington's Disease Transgenic Mouse Model. PLoS ONE 10(7): e0134142. https://doi.org/10.1371/journal.pone.0134142

Jiao J, Zhang Y, Lou D, Wu X, Zhang Y. Antihyperlipidemic and antihypertensive effect of a triterpenoid-rich extract from bamboo shavings and vasodilator effect of friedelin on phenylephrine-induced vasoconstriction in thoracic aortas of rats. Phytotherapy Research:. 2007 Dec;21(12):1135-41.

Jiménez-Escrig A, Rincón M, Pulido R, Saura-Calixto F. Guava fruit (*Psidium guajava* L.) as a new source of antioxidant dietary fiber. Journal of Agricultural and food Chemistry. 2001 Nov 19;49(11):5489-93. https://doi.org/10.1021/jf010147p

Jimoh FO, Afolayan AA. Comparison of the nutritive value, antioxidant and antibacterial activities of *Sonchus asper* and *Sonchus oleraceus*. Records of Natural Products. 2011;5(1):29.

Jirovetz L, Buchbauer G, Bail S, Denkova Z, Slavchev A, Stoyanova A, Schmidt E, Geissler M. Antimicrobial activities of essential oils of mint and peppermint as well as some of their main compounds. Journal of Essential oil research. 2009 Jul 1;21(4):363-6. https://doi.org/10.1080/10412905.2009.9700193

Joerin L, Kauschka M, Bonnländer B, Pischel I, Benedek B, Butterweck V. Ficus carica Leaf Extract Modulates the Lipid Profile of Rats Fed with a High-Fat Diet through an Increase of HDL-C. Phytotherapy Research. 2014 Feb;28(2):261-7.https://doi.org/10.1002/ptr.4994

John TA, Onabanjo AO. Effect of an aqueous extract of *Entandrophragma utile* bark on gastric acid secretion in rat and isolated ileum contractility in guinea pig. African Journal of Biomedical Research. 2010;13(3):197-206.

Johnson J, Maher P, Hanneken A. The flavonoid, eriodictyol, induces long-term protection in ARPE-19 cells through its effects on Nrf2 activation and phase 2 gene expression. Investigative ophthalmology & visual science. 2009 May 1;50(5):2398-406.

Joly LG, Guerra S, Septimo R, Solis PN, Correa M, Gupta M, Levy S, Sandberg F. Ethnobotanical inventory of medicinal plants used by the Guaymi Indians in Western Panama. Part I. Journal of Ethnopharmacology. 1987 Jul 1;20(2):145-71. https://doi.org/10.1016/0378-8741(87)90085-7

Joly LG, Guerra S, Septimo R, Solís PN, Gupta MP, Levy S, Sandberg F, Perera P. Ethnobotanical inventory of medicinal plants used by the Guaymi Indians in Western Panama. Part II. Journal of ethnopharmacology. 1990 Feb 1;28(2):191-206. https://doi.org/10.1016/0378-8741(90)90029-S

Jones JR, Lebar MD, Jinwal UK, Abisambra JF, Koren III J, Blair L, O'Leary JC, Davey Z, Trotter J, Johnson AG, Weeber E. The diarylheptanoid (+)-a R, 11 S-myricanol and two flavones from bayberry (*Myrica cerifera*) destabilize the microtubule-associated protein Tau. Journal of natural products. 2011 Jan 28;74(1):38-44. doi:10.1021/np100572z

Jorim RY, Korape S, Legu W, Koch M, Barrows LR, Matainaho TK, Rai PP. An ethnobotanical survey of medicinal plants used in the eastern highlands of Papua New Guinea. J Ethnobiol Ethnomed. 2012 Dec 18;8:47. doi: 10.1186/1746-4269-8-47.

Joseph JA. Shukitt-Hale B. Denisova NA. Bielinski D. Martin A. McEwen JJ. Bickford PC. Reversals of age-related declines in neuronal signal transduction, cognitive, and motor behavioral deficits with blueberry, spinach, or strawberry dietary supplementation. J Neurosci. 1999;19:8114–8121.

Joshi B, Sah GP, Basnet BB, Bhatt MR, Sharma D, Subedi K, Pandey J, Malla R. Phytochemical extraction and antimicrobial properties of different medicinal plants: Ocimum sanctum (Tulsi), *Eugenia caryophyllata* (Clove), *Achyranthes bidentata* (Datiwan) and *Azadirachta indica* (Neem). J Microbiol Antimicrob. 2011 Jan 30;3(1):1-7.

Joukar S, Ebrahimi S, Khazaei M, Bashiri A, Shakibi MR, Naderi V, Shahouzehi B, Alasvand M. Co-administration of walnut (*Juglans regia*) prevents systemic hypertension induced by long-term use of dexamethasone: a promising strategy for steroid consumers. Pharmaceutical biology. 2017 Jan 1;55(1):184-9. https://doi.org/10.1080/13880209.2016.1233570

Juárez-Reyes K, Brindis F, Medina-Campos ON, Pedraza-Chaverri J, Bye R, Linares E, Mata R. Hypoglycemic, antihyperglycemic, and antioxidant effects of the edible plant *Anoda cristata*. Journal of ethnopharmacology. 2015 Feb 23;161:36-45. https://doi.org/10.1016/j.jep.2014.11.052

Juneja K, Mishra R, Chauhan S, Gupta S, Roy P, Sircar D. Metabolite profiling and wound-healing activity of *Boerhavia diffusa* leaf extracts using in vitro and in vivo models. Journal of traditional and complementary medicine. 2020 Jan 1;10(1):52-9. https://doi.org/10.1016/j.jtcme.2019.02.002

Jung HY, Lee AN, Song TJ, An HS, Kim YH, Kim KD, Kim IB, Kim KS, Han BS, Kim CH, Kim KS. Korean mistletoe (*Viscum album coloratum*) extract improves endurance capacity in mice by stimulating mitochondrial activity. Journal of medicinal food. 2012 Jul 1;15(7):621-8. https://doi.org/10.1089/jmf.2010.1469

Justin Thenmozhi A, Dhivyabharathi M, William Raja TR, Manivasagam T, Essa MM. Tannoid principles of *Emblica officinalis* renovate cognitive deficits and attenuate amyloid pathologies against aluminum chloride induced rat model of Alzheimer's disease. Nutritional neuroscience. 2016 Jul 2;19(6):269-78. https://doi.org/10.1179/1476830515Y.0000000016

Kabiru M, Usman M, Manga SB, Opaluwa SA, Nura A, Spencer TH. Phytochemical screening and antibacterial activity of the crude extract and fractions of *Tapinanthus globiferus* leaves on the bacterial isolates of wound. World J. Pharmaceut. Res.. 2017 Apr 28;6(7):209-38.

Kader G, Nikkon F, Rashid MA, Yeasmin T. Antimicrobial activities of the rhizome extract of *Zingiber zerumbet* Linn. Asian Pacific Journal of Tropical Biomedicine. 2011 Oct 1;1(5):409-12. https://doi.org/10.1016/S2221-1691(11)60090-7

Kadir A, Aizan NA, Rahmat A, Jaafar HZ. Protective effects of tamarillo (*Cyphomandra betacea*) extract against high fat diet induced obesity in Sprague-Dawley rats. Journal of obesity. 2015;2015 https://doi.org/10.1155/2015/846041

Kadir, M.F., Karmoker, J.R., Alam, M.D., Jahan, S.R., Mahbub, S. and Mia, M.M.K., 2015. Ethnopharmacological survey of medicinal plants used by traditional healers and indigenous people in Chittagong Hill Tracts, Bangladesh, for the treatment of snakebite. Evidence-Based Complementary and Alternative Medicine, 2015. https://doi.org/10.1155/2015/871675

Kaithwas G, Mukherjee A, Chaurasia AK, Majumdar DK. Antiinflammatory, analgesic and antipyretic activities of *Linum usitatissimum* L.(flaxseed/linseed) fixed oil. Indian J Exp Biol. 2011 Dec;49(12):932-8.

Kalaiyarasia C, Kalivaradhan K, Govindharaj R. Anticonvulsant and anxiolyic activities of ethyl acetate fraction of *Cassia fistula* Linn. pods in mice. Pharmacognosy Communication. 2015 Jan 1;5(1):76-82. doi: 10.5530/pc.2015.1.8

Kalpanadevi V, Shanmugasundaram R, Mohan VR. Antiinflammatory activity of seed extract of *Entada pursaetha* DC against carrageenan induced Paw edema. Group. 2012;2(5):12-24.

Kamaraj C, Rahuman AA, Siva C, Iyappan M, Kirthi AV. Evaluation of antibacterial activity of selected medicinal plant extracts from south India against human pathogens. Asian Pacific Journal of Tropical Disease. 2012 Jan 1;2:S296-301.

Kamath VG, Chandrashekar A, Rajini PS. Antiradical properties of sorghum (*Sorghum bicolor* L. Moench) flour extracts. Journal of cereal science. 2004 Nov 1;40(3):283-8. doi.org/10.1016/j.foodchem.2009.01.032

Kamau LN, Mbaabu PM, Mbaria JM, Gathumbi PK, Kiama SG. Ethnobotanical survey and threats to medicinal plants traditionally used for the management of human diseases in Nyeri County, Kenya. CellMed. 2016;6(3):21-1.

Kameshwaran S, Suresh V, Arunachalam G, Frank PR, Manikandan V. Evaluation of antinociceptive and anti-inflammatory potential of flower extract *Tecoma stans*. *Indian J Pharmacol*. 2012;44(4):543–544. doi:10.4103/0253-7613.99352

Kammerer D, Carle R, Schieber A. Characterization of phenolic acids in black carrots (*Daucus carota* ssp. sativus var. atrorubens Alef.) by high-performance liquid chromatography/electrospray ionization mass spectrometry. Rapid Commun Mass Spectrom. 2004;18:1331–1340. doi: 10.1002/rcm.1496. [PubMed] [Cross Ref]

Kanase VG, Jainb BB, Yadavc P. Evaluation of In-Vitro Immunomodulatory Activity of Hydroalcoholic Extract of *Ceropegia bulbosa* Roxb. *Int. J. Res. Sci. Innov. (IJRSI)* 2016;) 3(10):36-40.

Kanazawa K, Sakakibara H. High content of dopamine, a strong antioxidant, in cavendish banana. Journal of agricultural and food chemistry. 2000 Mar 20;48(3):844-8.

Kandeda AK, Taiwe GS, Moto FCO, Ngoupaye GT, Nkantchoua GCN, Njapdounke JSK, Omam JPO, Pale S, Kouemou N, Ngo Bum E. Antiepileptogenic and Neuroprotective Effects of *Pergularia daemia* on Pilocarpine Model of Epilepsy. Front Pharmacol. 2017 Jun 30;8:440. doi: 10.3389/fphar.2017.00440.

Kandhare AD, Raygude KS, Ghosh P, Ghule AE, Gosavi TP, Badole SL, Bodhankar SL. Effect of hydroalcoholic extract of *Hibiscus rosa sinensis* Linn. leaves in experimental colitis in rats. Asian Pacific journal of tropical biomedicine. 2012 May 1;2(5):337-44. https://doi.org/10.1016/S2221-1691(12)60053-7

Kandhasamy, M, Arunachalam KD, Thatheyus AJ. *Drynaria quercifolia* (L.) J. Sm: A potential resource for antibacterial activity. African Journal of Microbiology Research. 2008 Aug 31;2(8):202-5. https://doi.org/10.5897/AJMR.9000244

Kandil ZA, Esmat A, El-Din RS, Ezzat SM. Anti-inflammatory activity of the lipophilic metabolites from *Scolymus hispanicus* L. South African Journal of Botany. 2020 Jul 1;131:43-50. https://doi.org/10.1016/j.sajb.2020.01.022

Kandimalla R, Kalita S, Choudhury B, Dash S, Kalita K, Kotoky J. Chemical composition and anti-candidiasis mediated wound healing property of *Cymbopogon nardus* essential oil on chronic diabetic wounds. Frontiers in pharmacology. 2016 Jun 30;7:198. doi.org/10.3389/fphar.2016.00198

Kang BY, Chung SW, Kim SH, Ryu SY, Kim TS. Inhibition of interleukin-12 and interferon-γ production in immune cells by tanshinones from *Salvia miltiorrhiza*. Immunopharmacology. 2000 Sep 1;49(3):355-61.

Kang R, Helms R, Stout MJ, Jaber H, Chen Z, Nakatsu T. Antimicrobial activity of the volatile constituents of *Perilla frutescens* and its synergistic effects with polygodial. Journal of agricultural and food chemistry. 1992 Nov;40(11):2328-30. https://doi.org/10.1021/jf00023a054

Kanjwani DG, Marathe TP, Chiplunkar SV, Sathaye SS. Evaluation of immunomodulatory activity of methanolic extract of *Piper betel*. Scandinavian journal of immunology. 2008 Jun;67(6):589-93. https://doi.org/10.1111/j.1365-3083.2008.02110.x

Kanokmedhakul S, Kanokmedhakul K, Lekphrom R. Bioactive constituents of the roots of *Polyalthia cerasoides*. Journal of natural products. 2007 Sep 28;70(9):1536-8. https://doi.org/10.1021/np070293a

Kantati YT, Kodjo KM, Dogbeavou KS, Vaudry D, Leprince J, Gbeassor M. Ethnopharmacological survey of plant species used in folk medicine against central nervous system disorders in Togo. Journal of ethnopharmacology. 2016 Apr 2;181:214-20. https://doi.org/10.1016/j.jep.2016.02.006

Kanyonga PM, Faouzi MA, Meddah B, Mpona M, Essassi EM, Cherrah Y. Assessment of methanolic extract of *Marrubium vulgare* for anti-inflammatory, analgesic and anti-microbiologic activities. J Chem Pharm Res. 2011;3(1):199-204.

Kao ES, Wang CJ, Lin WL, Yin YF, Wang CP, Tseng TH. Anti-inflammatory potential of flavonoid contents from dried fruit of *Crataegus pinnatifida* in vitro and in vivo. Journal of agricultural and food chemistry. 2005 Jan 26;53(2):430-6. https://doi.org/10.1021/jf040231f

Kapadia SP, Pudakalkatti PS, Shivanaikar S. Detection of antimicrobial activity of banana peel (*Musa paradisiaca* L.) on *Porphyromonas gingivalis* and *Aggregatibacter actinomycetemcomitans*: An in vitro study. *Contemp Clin Dent*. 2015;6(4):496–499. doi:10.4103/0976-237X.169864 AMA

Kapewangolo P, Hussein AA, Meyer D. Inhibition of HIV-1 enzymes, antioxidant and anti-inflammatory activities of *Plectranthus barbatus*. Journal of ethnopharmacology. 2013 Aug 26;149(1):184-90. https://doi.org/10.1016/j.jep.2013.06.019

Kapoor R, Kakkar P. Protective role of morin, a flavonoid, against high glucose induced oxidative stress mediated apoptosis in primary rat hepatocytes. PLoS One. 2012;7(8):e41663. doi: 10.1371/journal.pone.0041663.

Karadi RV, Shah A, Parekh P, Azmi P. Antimicrobial activities of *Musa paradisiaca* and *Cocos nucifera*. International Journal of Research in Pharmaceutical and Biomedical Sciences. 2011;2(1):264-7.

Karamoddini MK, Emami SA, Ghannad MS, Sani EA, Sahebkar A. Antiviral activities of aerial subsets of *Artemisia* species against *Herpes simplex* virus type 1 (HSV1) in vitro. Asian Biomedicine. 2011 Feb 1;5(1):63-8. https://doi.org/10.5372/1905-7415.0501.007

Kareru PG, Gachanja AN, Keriko JM, Kenji GM. Antimicrobial activity of some medicinal plants used by herbalists in eastern province, Kenya. African Journal of Traditional, Complementary and Alternative Medicines. 2008;5(1):51-5.

Kargutkar S, Brijesh S. Anti-inflammatory evaluation and characterization of leaf extract of *Ananas comosus*. Inflammopharmacology. 2018 Apr;26(2):469-77. https://doi.org/10.1007/s10787-017-0379-3

Karou D, Savadogo A, Canini A, Yameogo S, Montesano C, Simpore J, Colizzi V, Traore AS. Antibacterial activity of alkaloids from *Sida acuta*. African journal of biotechnology. 2006;5(2):195-200.

Karpagam T, Varalakshmi B, Bai JS, Gomathi S. Effect of different doses of *Cucurbita pepo* linn extract as an anti-Inflammatory and analgesic nutraceautical agent on inflamed rats. IJPRD. 2011;3(3):184-92.

Karthikeyan M, Deepa MK. Anti–inflammatory activity of *Premna corymbosa* (Burm. f.) Rottl. & Willd. leaves extracts in Wistar albino rats. Asian Pacific journal of tropical medicine. 2011 Jul 1;4(7):510-3. https://doi.org/10.1016/S1995-7645(11)60136-3

Kartini K, Wati N, Gustav R, Wahyuni R, Anggada YF, Hidayani R, Raharjo A, Islamie R, Putra SE. Wound healing effects of *Plantago major* extract and its chemical compounds in hyperglycemic rats. Food Bioscience. 2021 Feb 27:100937. https://doi.org/10.1016/j.fbio.2021.100937

Karumi Y, Onyeyili P, Ogugbuaja VO. Anti-inflammatory and antinociceptive (analgesic) properties of *Momordical balsamina* Linn.(Balsam apple) leaves in rats. Pakistan Journal of Biological Sciences (Pakistan). 2003.

Karuppiah P, Rajaram S. Antibacterial effect of *Allium sativum* cloves and *Zingiber officinale* rhizomes against multiple-drug resistant clinical pathogens. *Asian Pac J Trop Biomed*. 2012;2(8):597-601. doi:10.1016/S2221-1691(12)60104-X

Kashyap P, Muthusamy K, Niranjan M, Trikha S, Kumar S. Sarsasapogenin: a steroidal saponin from *Asparagus racemosus* as multi target directed ligand in Alzheimer's disease. Steroids. 2020 Jan 1;153:108529. https://doi.org/10.1016/j.steroids.2019.108529

Kassaw E, Yohannes T, Bizualem E. In-Vitro Antibacterial Activity of *Plantago lanceolata* Against Some Selected Standard Pathogenic Bacterial. The International Journal of Biotechnology. 2018 Dec 4;7(1):44-50. doi: 10.18488/journal.57.2018.71.44.50

Katsiki M, Chondrogianni N, Chinou I, Rivett AJ, Gonos ES. The olive constituent oleuropein exhibits proteasome stimulatory properties in vitro and confers life span extension of human embryonic fibroblasts. Rejuvenation Res. 2007 Jun;10(2):157-72. doi: 10.1089/rej.2006.0513.

Katzenschlager R, Evans A, Manson A, Patsalos PN, Ratnaraj N, Watt H, Timmermann L, Van der Giessen R, Lees AJ. *Mucuna pruriens* in Parkinson's disease: a double blind clinical and pharmacological study. Journal of Neurology, Neurosurgery and Psychiatry. 2004 Dec 1;75(12):1672-7. http://dx.doi.org/10.1136/jnnp.2003.028761

Kaur GJ, Arora DS. Antibacterial and phytochemical screening of *Anethum graveolens, Foeniculum vulgare* and *Trachyspermum ammi*. BMC complementary and alternative medicine. 2009 Dec;9(1):30.

Kaur H, Singh D, Singh B, Goel RK. Anti-amnesic effect of *Ficus religiosa* in scopolamine-induced anterograde and retrograde amnesia. Pharmaceutical biology. 2010 Feb 1;48(2):234-40.

Kaur I, Yadav SK, Hariprasad G, Gupta RC, Srinivasan A, Batra JK, Puri M. Balsamin, a novel ribosome-inactivating protein from the seeds of Balsam apple *Momordica balsamina*. Amino acids. 2012 Aug 1;43(2):973-81.

Kaur M. Evaluation of Anti-inflammatory activity of Ethanol Extract of Bark of *Aspidosperma quebraco-blanco*. Research Journal of Pharmacy and Technology. 2013 May 1;6(5):9.

Kaur P, Dhull SB, Sandhu KS, Salar RK, Purewal SS. Tulsi (*Ocimum tenuiflorum*) seeds: in vitro DNA damage protection, bioactive compounds and antioxidant potential. Journal of Food Measurement and Characterization. 2018 Sep 1;12(3):1530-8.

Kchaou M, Salah HB, Mhiri R, Allouche N. Anti-oxidant and anti-acetylcholinesterase activities of *Zygophyllum album*. *Bangladesh Journal of Pharmacology.* 2016;11(1):54-62. doi:10.3329/bjp.v11i1.25463.

Keihanian F, Vajari MR, Saeidynia A, Elmieh A. Effect of *Ruta graveolens* hydro-alcoholic extract on pentylenetetrazole-induced seizure in male mice. Planta Medica. 2012 Jul;78(11):PL2. doi: 10.1055/s-0032-1321336

Kenechukwu FC, Mbah CJ, Momoh MA, Chime SA, Umeyor CE, Ogbonna JD. Pharmacological justification for the ethnomedical use of *Clausena anisata* root-bark extract in the management of epilepsy. Journal of Applied Pharmaceutical Science Vol. 2 (9), pp. 036-040, September, 2012. doi: 10.7324/JAPS.2012.2907

Kennedy DO, Scholey AB, Tildesley NT, Perry EK, Wesnes KA. Modulation of mood and cognitive performance following acute administration of *Melissa officinalis* (lemon balm). Pharmacology Biochemistry and Behavior. 2002 Jul 1;72(4):953-64.

Kensara OA, ElSawy NA, El-Shemi AG, Header EA. *Thymus vulgaris* supplementation attenuates blood pressure and aorta damage in hypertensive rats. Journal of Medicinal Plants Research. 2013 Mar 17;7(11):669-76. https://doi.org/10.5897/JMPR12.910

Ketabchi S, Moatari A, Shadram M, Rostami Y. The anti influenza virus activity of *Anchusa italica*. Asian Journal of Experimental Biological Sciences. 2011;2(4):758-61.

Khakzad MR, Ganji A, Ariabod V, Farahani I. Artemisinin therapeutic efficacy in the experimental model of multiple sclerosis. Immunopharmacology and immunotoxicology. 2017 Nov 2;39(6):348-53. https://doi.org/10.1080/08923973.2017.1379087

Khalaj-Kondori M, Sadeghi F, Hosseinpourfeizi MA, Shaikhzadeh-Hesari F, Nakhlband A, Rahmati-Yamchi M. *Boswellia serrata* gum resin aqueous extract upregulatesBDNF but not CREB expression in adult male rat hippocampus. Turkish journal of medical sciences. 2016 Nov 17;46(5):1573-8.

Khalid A, Rehman U, Sethi A, Khilji S, Fatima U, Khan MI, Waqas MK, Saqib QN, Farzana K, Asad MH, Mahmood S. Antimicrobial activity analysis of extracts of *Acacia modesta*, *Artimisia absinthium*, *Nigella sativa* and *Saussurea lappa* against Gram positive and Gram negative microorganisms. African Journal of Biotechnology. 2011;10(22):4574-80.

Khalil H, Abd El Maksoud AI, Roshdey T, El-Masry S. Guava flavonoid glycosides prevent influenza A virus infection via rescue of P53 activity. Journal of medical virology. 2019 Jan;91(1):45-55. doi: 10.1002/jmv.25295

Khan A, Jan G, Khan A, Gul Jan F, Bahadur A, Danish M. In vitro antioxidant and antimicrobial activities of *Ephedra gerardiana* (root and stem) crude extract and fractions. Evidence-Based Complementary and Alternative Medicine. 2017;2017. https://doi.org/10.1155/2017/4040254

Khan B, Ahmad SF, Bani S, Kaul A, Suri KA, Satti NK, Athar M, Qazi GN. Augmentation and proliferation of T lymphocytes and Th-1 cytokines by *Withania somnifera* in stressed mice. International immunopharmacology. 2006 Sep 1;6(9):1394-403. https://doi.org/10.1016/j.intimp.2006.04.00

Khan MR, Omoloso AD, Kihara M. Antibacterial activity of *Alstonia scholaris* and *Leea tetramera*. Fitoterapia. 2003 Dec;74(7-8):736-40. doi: 10.1016/s0367-326x(03)00192-8.

Khan MA, Khan H, Khan S, Mahmood T, Khan PM, Jabar A. Anti-inflammatory, analgesic and antipyretic activities of *Physalis minima* Linn. Journal of Enzyme Inhibition and Medicinal Chemistry. 2009 Jun 1;24(3):632-7. https://doi.org/10.1080/14756360802321120

Khan MN, Ngassapa O, Matee MI. Antimicrobial activity of Tanzanian chewing sticks against oral pathogenic microbes. Pharmaceutical Biology. 2000 Jul 1;38(3):235-40. https://doi.org/10.1076/1388-0209(200007)3831-SFT235

Khan MR, Kihara M, Omoloso AD. Anti-microbial activity of *Bidens pilosa*, *Bischofia javanica*, *Elmerillia papuana* and *Sigesbekia orientalis*. Fitoterapia. 2001 Aug 1;72(6):662-5. https://doi.org/10.1016/S0367-326X(01)00261-1

Khan MR, Kihara M, Omoloso AD. Antimicrobial activity of *Harpullia ramiflora*. Fitoterapia. 2001 Mar 1;72(3):298-300. https://doi.org/10.1016/S0367-326X(00)00299-9

Khan MR, Omoloso AD. Antibacterial activity of *Pterocarpus indicus*. Fitoterapia. 2003 Sep 1;74(6):603-5. https://doi.org/10.1016/S0367-326X(03)00149-7

Khan MR, Omoloso AD. Antibacterial and antifungal activities of *Dracontomelon dao*. Fitoterapia. 2002 Jul 1;73(4):327-30. https://doi.org/10.1016/S0367-326X(02)00076-X

Khan MZ, Atlas N, Nawaz W. Neuroprotective effects of *Caralluma tuberculata* on ameliorating cognitive impairment in a d-galactose-induced mouse model. Biomed Pharmacother. 2016 Dec;84:387-394. doi: 10.1016/j.biopha.2016.09.055.

Khan RA, Khan MR, Sahreen S, Bokhari J. Antimicrobial and phytotoxic screening of various fractions of *Sonchus asper*. African Journal of Biotechnology. 2010;9(25):3883-7.

Khan SU, Khan AU, Shah AU, Shah SM, Hussain S, Ayaz M, Ayaz S. Heavy metals content, phytochemical composition, antimicrobial and insecticidal evaluation of *Elaeagnus angustifolia.* Toxicology and industrial health. 2016 Jan;32(1):154-61. https://doi.org/10.1177/0748233713498459

Khanal S, Shakya N, Thapa K, Pant DR. Phytochemical investigation of crude methanol extracts of different species of *Swertia* from Nepal. BMC research notes. 2015 Dec;8(1):1-9. https://doi.org/10.1186/s13104-015-1753-0

Khanna AK, Rizvi F, Chander R. Lipid lowering activity of *Phyllanthus niruri* in hyperlipemic rats. Journal of ethnopharmacology. 2002 Sep 1;82(1):19-22. https://doi.org/10.1016/S0378-8741(02)00136-8

Khanna VG, Kannabiran K . Antimicrobial activity of saponin fractions of the leaves of *Gymnema sylvestre* and *Eclipta prostrata*. World Journal of microbiology and Biotechnology. 2008 Nov 1;24(11):2737. https://doi.org/10.1007/s11274-008-9758-7

Khare P, Singh L, Chauhan S. Evaluation of antianxiety activity of *Bauhinia variegata* in mice. Journal of Advance Research in Pharmacy & Biological Science (ISSN: 2208-2360). 2016 Feb 29;2(2):02-9.

Khémiri I, Essghaier Hédi B, Sadfi Zouaoui N, Ben Gdara N, Bitri L. The antimicrobial and wound healing potential of *Opuntia ficus indica* L. inermis extracted oil from Tunisia. Evidence-Based Complementary and Alternative Medicine. 2019 Apr 14;2019. https://doi.org/10.1155/2019/9148782

Khine KK. Investigation of some bioactivities, phytochemical screening and nutritional values of *Byttneria pilosa* Roxb.(Sat-le-pyat). J. Myanmar Acad. Arts Sci. 2019 Vol. XVII. No.1B.

Khlifi D, Sghaier RM, Amouri S, Laouini D, Hamdi M, Bouajila J. Composition and anti-oxidant, anti-cancer and anti-inflammatory activities of *Artemisia herba-alba*, *Ruta chalpensis* L. and *Peganum harmala* L. Food and chemical toxicology. 2013 May 1;55:202-8. https://doi.org/10.1016/j.fct.2013.01.004

Kianbakht S, Abasi B, Perham M, Hashem Dabaghian F. Antihyperlipidemic Effects of *Salvia officinalis* L. leaf extract in patients with hyperlipidemia: a randomized double-blind placebo-controlled clinical trial. Phytotherapy Research. 2011 Dec;25(12):1849-53. https://doi.org/10.1002/ptr.3506

Kiat TS, Pippen R, Yusof R, Ibrahim H, Khalid N, Rahman NA. Inhibitory activity of cyclohexenyl chalcone derivatives and flavonoids of fingerroot, *Boesenbergia rotunda* (L.), towards dengue-2 virus NS3 protease. Bioorganic & medicinal chemistry letters. 2006 Jun 15;16(12):3337-40.

Kidarn S, Saenjum C, Hongwiset D, Phrutivorapongkul A. Furanocoumarins from Kaffir lime and their inhibitory effects on inflammatory mediator production. Cogent Chemistry. 2018 Jan 1;4(1):1529259. https://doi.org/10.1080/23312009.2018.1529259

Kilani AM. Antibacterial assessment of whole stem bark of *Vitex doniana* against some enterobactriaceae. African Journal of Biotechnology. 2006;5(10).

Kim AR, Hwang YG, Lee JJ, Jung HO, Lee MY. Effects of *Eriobotrya japonica* Lindl.(Loquat) leaf ethanol extract on cholesterol and antioxidative activity in rats fed a high-fat/high-cholesterol diet. Journal of the Korean Society of Food Science and Nutrition. 2011;40(5):673-81. https://doi.org/10.3746/jkfn.2011.40.5.673

Kim BH, Park KS, Chang IM. Elucidation of anti-inflammatory potencies of *Eucommia ulmoides* bark and *Plantago asiatica* seeds. Journal of medicinal food. 2009 Aug 1;12(4):764-9.  https://doi.org/10.1089/jmf.2008.1239

Kim GH, Lim K, Yang HS, Lee JK, Kim Y, Park SK, Kim SH, Park S, Kim TH, Moon JS, Hwang IK. Improvement in neurogenesis and memory function by administration of *Passiflora incarnata* L. extract applied to sleep disorder in rodent models. Journal of Chemical Neuroanatomy. 2019 Jul 1;98:27-40. https://doi.org/10.1016/j.jchemneu.2019.03.005

Kim H, Chung MS. Antiviral activities of mulberry (*Morus alba*) juice and seed against influenza viruses. Evidence-Based Complementary and Alternative Medicine. 2018;2018. Article
ID 2606583 | 10 pages | https://doi.org/10.1155/2018/2606583

Kim HJ, Yang H, Jung DH, Hwang JT, Ko BS. Ameliorating effects of *Cuscuta chinensis* Lamak extract on hind-limb ischemia, and angiogenic-or inflammatory associated factors in ovariectomized mice. Molecular medicine reports. 2019 Apr 1;19(4):3321-9. https://doi.org/10.3892/mmr.2019.9977

Kim HJ, Yoo HS, Kim JC, Park CS, Choi MS, Kim M, Choi H, Min JS, Kim YS, Yoon SW, Ahn JK. Antiviral effect of *Curcuma longa* Linn extract against hepatitis B virus replication. Journal of ethnopharmacology. 2009 Jul 15;124(2):189-96. https://doi.org/10.1016/j.jep.2009.04.046

Kim HS, Zhang YH, Fang LH, Yun YP, Lee HK. Effects of tetrandrine and fangchinoline on human platelet aggregation and thromboxane B2 formation. Journal of ethnopharmacology. 1999 Aug 1;66(2):241-6. https://doi.org/10.1016/S0378-8741(98)00237-2

Kim IG, Kang SC, Kim KC, Choung ES, Zee OP. Screening of estrogenic and antiestrogenic activities from medicinal plants. Environmental toxicology and pharmacology. 2008 Jan 1;25(1):75-82. https://doi.org/10.1016/j.etap.2007.09.002

Kim JH, He MT, Kim MJ, Yang CY, Shin YS, Yokozawa T, Park CH, Cho EJ. Safflower (*Carthamus tinctorius* L.) seed attenuates memory impairment induced by scopolamine in mice via regulation of cholinergic dysfunction and oxidative stress. Food & function. 2019;10(6):3650-9.

Kim JM, Kim DH, Park SJ, Park DH, Jung SY, Kim HJ, Lee YS, Jin C, Ryu JH. The n-butanolic extract of *Opuntia ficus-indica* var. *saboten* enhances long-term memory in the passive avoidance task in mice. Progress in Neuro-Psychopharmacology and Biological Psychiatry. 2010 Aug 16;34(6):1011-7. https://doi.org/10.1016/j.pnpbp.2010.05.015

Kim JM, Lee DH, Kim JS, Lee JY, Park HG, Kim YJ, Oh YK, Jung HC, Kim SI. 5, 7-dihydroxy-3, 4, 6-trimethoxyflavone inhibits the inflammatory effects induced by *Bacteroides fragilis* enterotoxin via dissociating the complex of heat shock protein 90 and IκBα and IκB kinase-γ in intestinal epithelial cell culture. Clinical & Experimental Immunology. 2009 Mar;155(3):541-51. https://doi.org/10.1111/j.1365-2249.2008.03849.x

Kim JY, Kim KH, Kwag EH, Seol YJ, Lee YM, Ku Y, Rhyu IC. Magnoliae Cortex and maize modulate *Porphyromonas gingivalis*-induced inflammatory reactions. Journal of Periodontal & Implant Science. 2018 Apr;48(2):70-83. doi:10.5051/jpis.2018.48.2.70

Kim JY, Shin JS, Ryu JH, Kim SY, Cho YW, Choi JH, Lee KT. Anti-inflammatory effect of anemarsaponin B isolated from the rhizomes of *Anemarrhena asphodeloides* in LPS-induced RAW 264.7 macrophages is mediated by negative regulation of the nuclear factor-κB and p38 pathways. Food and chemical toxicology. 2009 Jul 1;47(7):1610-7.

Kim KH, Kim MA, Moon E, Kim SY, Choi SZ, Son MW, Lee KR. Furostanol saponins from the rhizomes of *Dioscorea japonica* and their effects on NGF induction. Bioorganic & medicinal chemistry letters. 2011 Apr 1;21(7):2075-8. doi: 10.1016/j.bmcl.2011.02.003

Kim KS, Ezaki O, Ikemoto S, ITAKURA H. Effects of *Platycodon grandiflorum* feeding on serum and liver lipid concentrations in rats with diet-induced hyperlipidemia. Journal of nutritional science and vitaminology. 1995;41(4):485-91.https://doi.org/10.3177/jnsv.41.485

Kim M, Cho KH, Shin MS, Lee JM, Cho HS, Kim CJ, Shin DH, Yang HJ. Berberine prevents nigrostriatal dopaminergic neuronal loss and suppresses hippocampal apoptosis in mice with Parkinson's disease. International Journal of Molecular Medicine. 2014 Apr 1;33(4):870-8. https://doi.org/10.3892/ijmm.2014.1656

Kim M, Hwang IG, Kim SB, Choi AJ. Chemical characterization of balloon flower (*Platycodon grandiflorum*) sprout extracts and their regulation of inflammatory activity in lipopolysaccharide-stimulated RAW 264.7 murine macrophage cells. Food Sci Nutr. 2019 Dec 3;8(1):246-256. doi: 10.1002/fsn3.1297.

Kim MJ, Lee J, Seong AR, Lee YH, Kim YJ, Baek HY, Kim YJ, Jun WJ, Yoon HG. Neuroprotective effects of *Eriobotrya japonica* against β-amyloid-induced oxidative stress and memory impairment. Food and chemical toxicology. 2011 Apr 1;49(4):780-4. doi: 10.1016/j.fct.2010.11.043

Kim N, Martínez CC, Jang DS, Lee JK, Oh MS. Anti-neuroinflammatory effect of *Iresine celosia* on lipopolysaccharide-stimulated microglial cells and mouse. Biomedicine & Pharmacotherapy. 2019 Mar 1;111:1359-66. doi.org/10.1016/j.biopha.2019.01.017

Kim S. Kwon J. [6]-Shogaol attenuates neuronal apoptosis in hydrogen peroxide-treated astrocytes through the up-regulation of neurotrophic factors. *Phytotherapy Research*. 2013;27(12):1795–1799. doi: 10.1002/ptr.4946.

Kim WS, Choi WJ, Lee S, Kim WJ, Lee DC, Sohn UD, Shin HS, Kim W. Anti-inflammatory, Antioxidant and Antimicrobial Effects of Artemisinin Extracts from *Artemisia annua* L. The Korean Journal of Physiology & Pharmacology. 2015 Jan 1;19(1):21-7.

Kim YS. Antimicrobial activity of yacon K-23 and manufacture of functional yacon jam. Korean Journal of Food Science and Technology. 2005;37(6):1035-8.

Kimira M, Arai Y, Shimoi K, Watanabe S. Japanese intake of flavonoids and isoflavonoids from foods. Journal of Epidemiology. 1998;8(3):168-75.

Kini RM, Gowda TV. Studies on snake venom enzymes: Part I-purification of ATPase, a toxic component of *Naja naja* venom and its inhibition by potassium gymnemate. Indian Journal of Biochemistry & Biophysics. 1982;19(2):152-4.

Kirisattayakul W, Wattanathorn J, Iamsaard S, Jittiwat J, Suriharn B, Lertrat K. Neuroprotective and Memory-Enhancing Effect of the Combined Extract of Purple Waxy Corn Cob and Pandan in Ovariectomized Rats. Oxid Med Cell Longev. 2017;2017:5187102. doi:10.1155/2017/5187102

Kisangau DP, Hosea KM, Joseph CC, Lyaruu HV. In vitro antimicrobial assay of plants used in traditional medicine in Bukoba rural district, Tanzania. African Journal of Traditional, complementary and alternative medicines. 2007;4(4):510-23.

Kitts DD, Wijewickreme AN, Hu C. Antioxidant properties of a North American ginseng extract. Molecular and cellular biochemistry. 2000 Jan 1;203(1-2):1-0. https://doi.org/10.1023/A:1007078414639

Klaas CA, Wagner G, Laufer S, Sosa S, Della Loggia R, Bomme U, Pahl HL, Merfort I. Studies on the anti-inflammatory activity of phytopharmaceuticals prepared from Arnica flowers. Planta medica. 2002 May;68(05):385-91.

Kleinrichert K, Alappat B. Comparative Analysis of Antioxidant and Anti-Amyloidogenic Properties of Various Polyphenol Rich Phytoceutical Extracts. Antioxidants (Basel). 2019;8(1):13. Published 2019 Jan 1. https://doi.org/10.3390/antiox8010013

Klimczak U, Woźniak M, Tomczyk M, Granica S. Chemical composition of edible aerial parts of meadow bistort (*Persicaria bistorta* (L.) Samp.). Food chemistry. 2017 Sep 1;230:281-90. https://doi.org/10.1016/j.foodchem.2017.02.128

Kloucek P, Polesny Z, Svobodova B, Vlkova E, Kokoska L. Antibacterial screening of some Peruvian medicinal plants used in Calleria District. Journal of Ethnopharmacology. 2005 Jun 3;99(2):309-12. https://doi.org/10.1016/j.jep.2005.01.062

Kloucek P, Svobodova B, Polesny Z, Langrova I, Smrcek S, Kokoska L. Antimicrobial activity of some medicinal barks used in Peruvian Amazon. Journal of Ethnopharmacology. 2007 May 4;111(2):427-9. https://doi.org/10.1016/j.jep.2006.11.010

Ko HC, Wei BL, Chiou WF. The effect of medicinal plants used in Chinese folk medicine on RANTES secretion by virus-infected human epithelial cells. Journal of ethnopharmacology. 2006 Sep 19;107(2):205-10.

Ko YJ, Seol HG, Lee GR, Jeong GI, Ryu CH. Anti-inflammatory effect and antioxidative activities of ingredients used in Bibimbab. Journal of Life Science. 2013;23(2):213-21.

Kobayashi M, Oki T, Masuda M, Nagai S, Fukui K, Matsugano K, Suda I. Hypotensive effect of anthocyanin-rich extract from purple-fleshed sweet potato cultivar" Ayamurasaki" in spontaneously hypertensive rats. Journal of the Japanese Society for Food Science and Technology (Japan). 2005.

Kocisko DA, Baron GS, Rubenstein R, Chen J, Kuizon S, Caughey B. New inhibitors of scrapie-associated prion protein formation in a library of 2,000 drugs and natural products. Journal of virology. 2003 Oct 1;77(19):10288-94.

Koda T, Kuroda Y, Ueno Y, Kitadate K, Imai H. Protective effects of buckwheat hull extract against experimental hippocampus injury induced by trimethyltin in rats. Nihon eiseigaku zasshi. Japanese journal of hygiene. 2008 Jul;63(4):711-6. doi: 10.1265/jjh.63.711

Koffi-Nevry R, Kouassi KC, Nanga ZY, Koussémon M, Loukou GY. Antibacterial activity of two bell pepper extracts: *Capsicum annuum* L. and *Capsicum frutescens*. International journal of food properties. 2012 Sep 1;15(5):961-71. https://doi.org/10.1080/10942912.2010.509896

Kokane DD, More RY, Kale MB, Nehete MN, Mehendale PC, Gadgoli CH. Evaluation of wound healing activity of root of *Mimosa pudica*. Journal of ethnopharmacology. 2009 Jul 15;124(2):311-5.

Koko WS, Mesaik MA, Yousaf S, Galal M, Choudhary MI. In vitro immunomodulating properties of selected Sudanese medicinal plants. Journal of ethnopharmacology. 2008 Jun 19;118(1):26-34.

Kokoska L, Polesny Z, Rada V, Nepovim A, Vanek T. Screening of some Siberian medicinal plants for antimicrobial activity. Journal of ethnopharmacology. 2002 Sep 1;82(1):51-3.

Komaki A, Hoseini F, Shahidi S, Baharlouei N. Study of the effect of extract of *Thymus vulgaris* on anxiety in male rats. Journal of traditional and complementary medicine. 2016 Jul 1;6(3):257-61. https://doi.org/10.1016/j.jtcme.2015.01.001

Komaki A, Rasouli B, Shahidi S. Anxiolytic effect of *Borago officinalis* (boraginaceae) extract in male rats. Avicenna Journal of Neuro Psycho Physiology. 2015 Feb 10;2(1):34-8.

Konaté K, Zerbo P, Ouédraogo M, Dibala CI, Adama H, Sytar O, Brestic M, Barro N. Anti-nociceptive properties in rodents and the possibility of using polyphenol-rich fractions from *Sida urens* L.(Malvaceae) against of dental caries bacteria. Annals of clinical microbiology and antimicrobials. 2013 Dec;12(1):14. https://doi.org/10.1186/1476-0711-12-14

Koolen HH, da Silva FM, Gozzo FC, de Souza AQ, de Souza AD. Antioxidant, antimicrobial activities and characterization of phenolic compounds from buriti (*Mauritia flexuosa* L. f.) by UPLC–ESI-MS/MS. Food Research International. 2013 May 1;51(2):467-73.  https://doi.org/10.1016/j.foodres.2013.01.039

Kore AP, Naikwade NS, Ladda PL, Shikalgar TS, Patil SS. Evaluation of Anticonvulsant Activity of Ethanolic Extract of Leaves of *Cajanus cajan* (L) Millsp. In Rodents. International Journal of Pharmaceutical Sciences and Drug Research. 2019;11(6):330-6.  doi:  10.25004/IJPSDR.2019.110608

Kosalec I, Kremer D, Locatelli M, Epifano F, Genovese S, Carlucci G, Randić M, Končić MZ. Anthraquinone profile, antioxidant and antimicrobial activity of bark extracts of *Rhamnus alaternus, R. fallax, R. intermedia* and *R. pumila.* Food chemistry. 2013 Jan 15;136(2):335-41. https://doi.org/10.1016/j.foodchem.2012.08.026

Kosaraju J, Chinni S, Roy PD, Kannan E, Antony AS, Kumar MN. Neuroprotective effect of *Tinospora cordifolia* ethanol extract on 6-hydroxy dopamine induced Parkinsonism. Indian J Pharmacol. 2014;46(2):176-180. doi:10.4103/0253-7613.129312

Kott V, Barbini L, Cruanes M, de D Munoz J, Vivot E, Cruañes J, Martino V, Ferraro G, Cavallaro L, Campos R. Antiviral activity in Argentine medicinal plants. Journal of ethnopharmacology. 1998 Jan 1;64(1):79-84. https://doi.org/10.1016/S0378-8741(98)00098-1

Kouchmeshky A, Jameie SB, Amin G, Ziai SA. Investigation of angiotensin-convertings enzyme inhibitory effects of medicinal plants used in traditional Persian medicine for treatment of hypertension: screening study. Thrita journal of medical sciences. 2012;1(1):13-23.

Koyama N, Kuribayashi K, Seki T, Kobayashi K, Furuhata Y, Suzuki K, Arisaka H, Nakano T, Amino Y, Ishii K. Serotonin derivatives, major safflower (*Carthamus tinctorius* L.) seed antioxidants, inhibit low-density lipoprotein (LDL) oxidation and atherosclerosis in apolipoprotein E-deficient mice. Journal of agricultural and food chemistry. 2006 Jul 12;54(14):4970-6.

Krikorian R, Shidler MD, Nash TA, Kalt W, Vinqvist-Tymchuk MR, Shukitt-Hale B, Joseph JA. Blueberry supplementation improves memory in older adults. Journal of agricultural and food chemistry. 2010 Jan 4;58(7):3996-4000.

Krishnakumar NM, Latha PG, Suja SR, Rajasekharan S. A comparative study on in vitro free radical scavenging potential of different solvent extracts of *Morinda umbellata* Linn. and its quantitative phytochemical constituents. International Journal of Pharmaceutical Research. 2016 Jan;8(1):39-45.

Kritheka N, Kumar RS, Kumar SS, Murthy NV, Sundram RS, Perumal P. Anti-inflammatory and Antimicrobial activities of Petroleum ether and Ethanol extracts of *Scutia myrtina* (Rhamnaceae). Oriental Pharmacy and Experimental Medicine. 2008;8(4):400-7. https://doi.org/10.3742/OPEM.2008.8.4.400

Ksouri WM, Medini F, Mkadmini K, Legault J, Magné C, Abdelly C, Ksouri R. LC–ESI-TOF–MS identification of bioactive secondary metabolites involved in the antioxidant, anti-inflammatory and anticancer activities of the edible halophyte *Zygophyllum album* Desf. Food chemistry. 2013 Aug 15;139(1-4):1073-80. https://doi.org/10.1016/j.foodchem.2013.01.047

Ku CM, Lin JY. Anti-inflammatory effects of 27 selected terpenoid compounds tested through modulating Th1/Th2 cytokine secretion profiles using murine primary splenocytes. Food chemistry. 2013 Nov 15;141(2):1104-13. doi.org/10.1016/j.foodchem.2013.04.044

Ku SK, Kim TH, Lee S, Kim SM, Bae JS. Antithrombotic and profibrinolytic activities of isorhamnetin-3-O-galactoside and hyperoside. Food and chemical toxicology. 2013 Mar 1;53:197-204. https://doi.org/10.1016/j.fct.2012.11.040

Kuang CL, Lv D, Shen GH, Li SS, Luo QY, Zhang ZQ. Chemical composition and antimicrobial activities of volatile oil extracted from *Chrysanthemum morifolium* Ramat. Journal of food science and technology. 2018 Jul 1;55(7):2786-94. https://doi.org/10.1007/s13197-018-3203-1

Kuete V, Ngameni B, Simo CF, Tankeu RK, Ngadjui BT, Meyer JJ, Lall N, Kuiate JR. Antimicrobial activity of the crude extracts and compounds from *Ficus chlamydocarpa* and *Ficus cordata* (Moraceae). Journal of ethnopharmacology. 2008 Oct 30;120(1):17-24. https://doi.org/10.1016/j.jep.2008.07.026

Kuglerova M, Halamova K, Kokoska L, Van Damme P, Grade J. Antimicrobial activity of Ugandan medicinal plants. Planta Medica. 2007;73(09):P_113. doi:  10.1055/s-2007-986895

Kuhnle GG, Dell'Aquila C, Aspinall SM, Runswick SA, Joosen AM, Mulligan AA, Bingham SA. Phytoestrogen content of fruits and vegetables commonly consumed in the UK based on LC–MS and 13C-labelled standards. Food Chemistry. 2009 Sep 15;116(2):542-54. https://doi.org/10.1016/j.foodchem.2009.03.002

Kuhnt M, Pröbstle A, Bauer R, Heinrich M. Biological and pharmacological investigations of Hyptis verticillata. Planta Medica. 1993 Dec;59(S 1):A665.

Kujawska M, Jourdes M, Kurpik M, Szulc M, Szaefer H, Chmielarz P, Kreiner G, Krajka-Kuźniak V, Mikołajczak PŁ, Teissedre PL, Jodynis-Liebert J. Neuroprotective Effects of Pomegranate Juice against Parkinson's Disease and Presence of Ellagitannins-Derived Metabolite—Urolithin A—In the Brain. International Journal of Molecular Sciences. 2020 Jan;21(1):202. https://doi.org/10.3390/ijms21010202

Kulkarni KS, Kasture SB, Mengi SA. Efficacy study of *Prunus amygdalus* (almond) nuts in scopolamine-induced amnesia in rats. Indian J Pharmacol. 2010 Jun;42(3):168-73. doi: 10.4103/0253-7613.66841.

Kulkarni MP, Juvekar AR. Effect of *Alstonia scholaris* (Linn.) R. Br. on stress and cognition in mice. Indian J Exp Biol. 2009 Jan;47(1):47-52.

Kulkarni PD, Ghaisas MM, Chivate ND, Sankpal PS. Memory enhancing activity of *Cissampelos pariera* in mice. International Journal of Pharmacy and Pharmaceutical Sciences. 2011;3(2):206-11.

Kulkarni RA, Deshpande AR. Anti-inflammatory and antioxidant effect of ginger in tuberculosis. Journal of Complementary and Integrative Medicine. 2016 Jun 1;13(2):201-6. doi: 10.1515/jcim-2015-0032.

Kulkarni YA, Agarwal S, Garud MS. Effect of Jyotishmati (*Celastrus paniculatus*) seeds in animal models of pain and inflammation. *J Ayurveda Integr Med*. 2015;6(2):82–88. doi:10.4103/0975-9476.146540

Kumagai M, Watanabe A, Yoshida I, Mishima T, Nakamura M, Nishikawa K, Morimoto Y. Evaluation of Aculeatin and Toddaculin Isolated from *Toddalia asiatica* as Anti-inflammatory Agents in LPS-Stimulated RAW264 Macrophages. Biological and Pharmaceutical Bulletin. 2018 Jan 1;41(1):132-7. https://doi.org/10.1248/bpb.b17-00607

Kumaki Y, Wandersee MK, Smith AJ, Zhou Y, Simmons G, Nelson NM, Bailey KW, Vest ZG, Li JK, Chan PK, Smee DF. Inhibition of severe acute respiratory syndrome coronavirus replication in a lethal SARS-CoV BALB/c mouse model by stinging nettle lectin, *Urtica dioica* agglutinin. Antiviral research. 2011 Apr 1;90(1):22-32. https://doi.org/10.1016/j.antiviral.2011.02.003

Kumalasari ID, Harmayani E, Lestari LA, Raharjo S, Asmara W, Nishi K, Sugahara T. Evaluation of immunostimulatory effect of the arrowroot (*Maranta arundinacea*. L) in vitro and in vivo. Cytotechnology. 2012 Mar 1;64(2):131-7. https://doi.org/10.1007/s10616-011-9403-4

Kumar A, Gahlot K, Dora J, Singh P. Analgesic activity of methanolic extract of *Flemingia strobilifera* (r. br). International journal of research in pharmacy and chemistry. 2011;1(4).

Kumar A, Garg V, Chaudhary A, Jain PK, Tomar PK. Isolation, characterisation and antibacterial activity of new compounds from methanolic extract of seeds of *Caesalpinia crista* L.(Caesalpinaceae). Natural product research. 2014 Feb 16;28(4):230-8. https://doi.org/10.1080/14786419.2013.814054

Kumar A, Ilavarasan R, Jayachandran T, Deecaraman M, Kumar RM, Aravindan P, Padmanabhan N, Krishan MR. Anti-inflammatory activity of *Syzygium cumini* seed. African Journal of Biotechnology. 2008;7(8).

Kumar A, Panghal S, Mallapur SS, Kumar M, Ram V, Singh BK. Antiinflammatory Activity of *Piper longum* Fruit Oil. Indian J Pharm Sci. 2009 Jul;71(4):454-6. doi: 10.4103/0250-474X.57300.

Kumar A, Prakash A, Dogra S. Naringin alleviates cognitive impairment, mitochondrial dysfunction and oxidative stress induced by D-galactose in mice. Food and Chemical Toxicology. 2010 Feb 1;48(2):626-32. https://doi.org/10.1016/j.fct.2009.11.043

Kumar B, Gupta SK, Nag TC, Srivastava S, Saxena R. Green tea prevents hyperglycemia-induced retinal oxidative stress and inflammation in streptozotocin-induced diabetic rats. Ophthalmic Research. 2012;47(2):103-8. https://doi.org/10.1159/000330051

Kumar D, Kumar S, Kumar S, Singh J, Sharma C, Aneja KR. Antimicrobial and preliminary phytochemical screening of crude leaf extract of *Pandanus odoratissimus* L. Pharmacol Online 2010; 2: 600. 2010;610.

Kumar KS, Sabu V, Sindhu G, Rauf AA, Helen A. Isolation, identification and characterization of apigenin from *Justicia gendarussa* and its anti-inflammatory activity. International immunopharmacology. 2018 Jun 1;59:157-67. doi.org/10.1016/j.intimp.2018.04.004

Kumar M, Paul Y, Anand VK. An ethnobotanical study of medicinal plants used by the locals in Kishtwar, Jammu and Kashmir, India. Ethnobotanical Leaflets. 2009;2009(10):5.

Kumar N, Abichandani LG, Thawani V, Gharpure KJ, Naidu MU, Venkat Ramana G. Efficacy of Standardized Extract of *Bacopa monnieri* (Bacognize®) on Cognitive Functions of Medical Students: A Six-Week, Randomized Placebo-Controlled Trial. Evid Based Complement Alternat Med. 2016;2016:4103423. doi: 10.1155/2016/4103423.

Kumar R, Bhagat N. Ethnomedicinal plants of district Kathua (J&K). International journal of medicinal and aromatic plants. 2012;2(4):603-11.

Kumar R, Mishra AK, Dubey NK, Tripathi YB. Evaluation of *Chenopodium ambrosioides* oil as a potential source of antifungal, antiaflatoxigenic and antioxidant activity. International journal of food microbiology. 2007 Apr 30;115(2):159-64.

Kumar RS, Sivakumar T, Sundaram RS, Sivakumar P, Nethaji R, Gupta M. Antimicrobial and antioxidant activities of *Careya arborea* Roxb. stem bark. Iranian Journal of Pharmacology & Therapeutics. 2006 Sep 10;5(1):35-41.

Kumar S, Kumar V, Prakash O. Antidiabetic and hypolipidemic activities of *Hibiscus tiliaceus* (L.) flowers extract in streptozotocin induced diabetic rats. Pharmacologyonline. 2010;2:1037-44.

Kumar V, Singh RK, Jaiswal AK, Bhattacharya SK, Acharya SB. Anxiolytic activity of Indian *Abies pindrow* Royle leaves in rodents: an experimental study. Ind J Exp Biol 2000: 38: 343-346.

Kumar VL, Basu NO. Anti-inflammatory activity of the latex of *Calotropis procera*. Journal of Ethnopharmacology. 1994 Oct 1;44(2):123-5. https://doi.org/10.1016/0378-8741(94)90078-

Kumarasamy Y, Cox PJ, Jaspars M, Nahar L, Sarker SD. Screening seeds of Scottish plants for antibacterial activity. Journal of Ethnopharmacology. 2002 Nov 1;83(1-2):73-7. https://doi.org/10.1016/S0378-8741(02)00214-3

Kundu JK, Das B, Kundu J, Bachar SC. Anti-inflammatory, analgesic and diuretic activity of *Ludwigia hyssopifolia* Linn. Archives of Medical and Biomedical Research. 2014;1(4):139-46. http://dx.doi.org/10.4314/ambr.v1i4.3

Kunle O, Okogun J, Egamana E, Emojevwe E, Shok M. Antimicrobial activity of various extracts and carvacrol from *Lippia multiflora* leaf extract. Phytomedicine. 2003 Jan 1;10(1):59-61. https://doi.org/10.1078/094471103321648674

Küpeli E, Kartal M, Aslan S, Yesilada E. Comparative evaluation of the anti-inflammatory and antinociceptive activity of Turkish *Eryngium* species. Journal of ethnopharmacology. 2006 Aug 11;107(1):32-7. doi.org/10.1016/j.jep.2006.02.005

Kupeli E, Orhan I, Yesilada E. Evaluation of some plants used in Turkish folk medicine for their anti-inflammatory and antinociceptive activities. Pharmaceutical biology. 2007 Jan 1;45(7):547-55. https://doi.org/10.1080/13880200701498895

Kuraoka-Oliveira ÂM, Radai JA, Leitão MM, Cardoso CA, Silva-Filho SE, Kassuya CA. Anti-inflammatory and anti-arthritic activity in extract from the leaves of *Eriobotrya japonica*. Journal of Ethnopharmacology. 2020 Mar 1;249:112418. doi: 10.1016/j.jep.2019.112418

Kurt B, Bilge N, Sözmen M, Aydın U, Önyay T, Özaydın İ. Effects of *Plantago lanceolata* L. extract on full-thickness excisional wound healing in a mouse model. Biotechnic & Histochemistry. 2018 May 19;93(4):249-57. https://doi.org/10.1080/10520295.2017.1421773

Kuruuzum-Uz A, Suleyman H, Cadirci E, Guvenalp Z, Demirezer LO. Investigation on anti-inflammatory and antiulcer activities of *Anchusa azurea* extracts and their major constituent rosmarinic acid. Z Naturforsch C J Biosci. 2012 Jul-Aug;67(7-8):360-6. doi: 10.1515/znc-2012-7-802.

Kusumoto IT, Nakabayashi T, Kida H, Miyashiro H, Hattori M, Namba T, Shimotohno K. Screening of various plant extracts used in ayurvedic medicine for inhibitory effects on human immunodeficiency virus type 1 (HIV-1) protease. Phytotherapy Research. 1995 May;9(3):180-4. https://doi.org/10.1002/ptr.2650090305

Kwon HJ, Jung HY, Hahn KR, Kim W, Kim JW, Yoo DY, Yoon YS, Hwang IK, Kim DW. *Bacopa monnieri* extract improves novel object recognition, cell proliferation, neuroblast differentiation, brain-derived neurotrophic factor, and phosphorylation of cAMP response element-binding protein in the dentate gyrus. Lab Anim Res. 2018 Dec;34(4):239-247. doi: 10.5625/lar.2018.34.4.239.

Kyakulaga AH, Deogratius O, Nyafuono JF, Francis O, Engeu OP. Wound healing potential of the ethanolic extracts of *Bidens pilosa* and *Ocimum suave*. African Journal of Pharmacy and Pharmacology. 2011 Feb 28;5(2):132-6.

Kyriakopoulos AM, Dinda B. *Cornus mas* (Linnaeus) novel devised medicinal preparations: bactericidal effect against *Staphylococcus aureus* and *Pseudomonas aeruginosa*. Molecules. 2015 333Jun;20(6):11202-18. https://doi.org/10.3390/molecules200611202

Labuschagné A, Hussein AA, Rodríguez B, Lall N. Synergistic antimycobacterial actions of *Knowltonia vesicatoria* (Lf) Sims. Evidence-based complementary and alternative medicine. 2012;2012. incomplete

Ladiwala AR, Lin JC, Bale SS, Marcelino-Cruz AM, Bhattacharya M, Dordick JS, Tessier PM. Resveratrol selectively remodels soluble oligomers and fibrils of amyloid Aβ into off-pathway conformers. Journal of Biological Chemistry. 2010 Jul 30;285(31):24228-37.

Lagrota MH, Wigg MD, Santos MM, Miranda MM, Camara FP, Couceiro JN, Costa SS. Inhibitory activity of extracts of *Alternanthera brasiliana* (Amaranthaceae) against the herpes simplex virus. Phytotherapy Research. 1994 Sep;8(6):358-61. https://doi.org/10.1002/ptr.2650080609

Lahlou S, Magalhães PJ, de Siqueira RJ, Figueiredo AF, Interaminense LF, Maia JG, da Cunha Sousa PJ. Cardiovascular effects of the essential oil of *Aniba canelilla* bark in normotensive rats. Journal of cardiovascular pharmacology. 2005 Oct 1;46(4):412-21.

Lajter I, Vasas A, Béni Z, Forgo P, Binder M, Bochkov V, Zupkó I, Krupitza G, Frisch R, Kopp B, Hohmann J. Sesquiterpenes from *Neurolaena lobata* and their antiproliferative and anti-inflammatory activities. J Nat Prod. 2014 Mar 28;77(3):576-82. doi: 10.1021/np400834c.

Lakić N, Mimica-Dukić N, Isak J, Božin B. Antioxidant properties of *Galium verum* L.(Rubiaceae) extracts. Open Life Sciences. 2010 Jun 1;5(3):331-7. https://doi.org/10.2478/s11535-010-0022-4

Laksmitawati DR, Widyastuti A, Karami N, Afifah E, Rihibiha DD, Nufus H, Widowati W. Anti-inflammatory effects of *Anredera cordifolia* and *Piper crocatum* extracts on lipopolysaccharide-stimulated macrophage cell line. Bangladesh Journal of Pharmacology. 2017 Mar 2;12(1):35-40.

Lalan BK, Hiray RS, Ghongane BB. Evaluation of Analgesic and Anti-Inflammatory Activity of Extract of *Holoptelea integrifolia* and *Argyreia speciosa* in Animal Models. *J Clin Diagn Res*. 2015;9(7):FF01–FF4. doi:10.7860/JCDR/2015/12059.6200

Lall N, Meyer JJ. In vitro inhibition of drug-resistant and drug-sensitive strains of *Mycobacterium tuberculosis* by ethnobotanically selected South African plants. Journal of ethnopharmacology. 1999 Sep 1;66(3):347-54. doi.org/10.1016/S0378-8741(98)00185-8

Laluces HM, Nakayama A, Nonato MG, dela Cruz TE, Tan MA. Antimicrobial alkaloids from the leaves of *Pandanus amaryllifolius*. Journal of Applied Pharmaceutical Science. 2015 Oct;5(10):151-3. doi: 10.7324/JAPS.2015.501026

Lam LM, Nguyen MT, Nguyen HX, Dang PH, Nguyen NT, Tran HM, Nguyen HT, Nguyen NM, Min BS, Kim JA, Choi JS. Anti-cholinesterases and memory improving effects of Vietnamese *Xylia xylocarpa*. Chemistry Central Journal. 2016 Dec;10(1):1-0. https://doi.org/10.1186/s13065-016-0197-5

Lamichhane R, Pandeya PR, Lee KH, Kim SG, Devkota HP, Jung HJ. Anti-Adipogenic and Anti-Inflammatory Activities of (-)-*epi*-Osmundalactone and Angiopteroside from *Angiopteris helferiana* C.Presl. Molecules. 2020 Mar 15;25(6):1337. doi: 10.3390/molecules25061337.

Lamou B, Taiwe GS, Hamadou A, Houlray J, Atour MM, Tan PV. Antioxidant and antifatigue properties of the aqueous extract of *Moringa oleifera* in rats subjected to forced swimming endurance test. Oxidative medicine and cellular longevity. 2016;2016. https://doi.org/10.1155/2016/3517824

Lamounier KC, Cunha LC, de Morais SA, de Aquino FJ, Chang R, do Nascimento EA, de Souza MG, Martins CH, Cunha WR. Chemical Analysis and Study of Phenolics, Antioxidant Activity, and Antibacterial Effect of the Wood and Bark of *Maclura tinctoria* (L.) D. Don ex Steud. Evidence-based complementary and alternative medicine. 2012 Jan 1;2012. doi: 10.1155/2012/451039.

Lan YL, Zhou JJ, Liu J, Huo XK, Wang YL, Liang JH, Zhao JC, Sun CP, Yu ZL, Fang LL, Tian XG. *Uncaria rhynchophylla* ameliorates Parkinson’s disease by inhibiting HSP90 expression: insights from quantitative proteomics. Cellular Physiology and Biochemistry. 2018;47(4):1453-64. https://doi.org/10.1159/000490837

Lang DR, Racker E. Effects of quercetin and F1 inhibitor on mitochondrial ATPase and energy-linked reactions in submitochondrial particles. Biochimica et Biophysica Acta (BBA)-Bioenergetics. 1974 Feb 22;333(2):180-6. doi.org/10.1016/0005-2728(74)90002-4

Lanhers MC, Joyeux M, Soulimani R, Fleurentin J, Sayag M, Mortier F, Younos C, Pelt JM. Hepatoprotective and anti-inflammatory effects of a traditional medicinal plant of Chile, *Peumus boldus*. Planta medica. 1991 Apr;57(02):110-5.

La Rosa P, Bertini ES, Piemonte F. The NRF2 Signaling Network Defines Clinical Biomarkers and Therapeutic Opportunity in Friedreich's Ataxia. Int J Mol Sci. 2020;21(3). https://doi.org/10.3390/ijms21030916

Larrosa M, González-Sarrías A, Yáñez-Gascón MJ, Selma MV, Azorín-Ortuño M, Toti S, Tomás-Barberán F, Dolara P, Espín JC. Anti-inflammatory properties of a pomegranate extract and its metabolite urolithin-A in a colitis rat model and the effect of colon inflammation on phenolic metabolism. The Journal of nutritional biochemistry. 2010 Aug 1;21(8):717-25.

Larsen BH, Soelberg J, Jäger AK. COX-1 inhibitory effect of medicinal plants of Ghana. South African Journal of Botany. 2015 Jul 1;99:129-31. https://doi.org/10.1016/j.sajb.2015.04.004

Lastra AL, Ramirez TO, Salazar L, Martinez M, Trujillo-Ferrara J. The ambrosanolide cumanin inhibits macrophage nitric oxide synthesis: some structural considerations. Journal of ethnopharmacology. 2004 Dec 1;95(2-3):221-7. https://doi.org/10.1016/j.jep.2004.07.020

Latham P, Konda ku Mbuta A. Useful plants of Bas-Congo province, Democratic Republic of Congo, Vol. 1. 2016. Salvation Army, London, UK.

Latham P, Konda ku Mbuta A. Useful plants of Bas-Congo province, Democratic Republic of Congo, Vol. 2. 2017. Salvation Army, London, UK.

Lathe R, Sapronova A, Kotelevtsev Y. Atherosclerosis and Alzheimer-diseases with a common cause? Inflammation, oxysterols, vasculature. BMC geriatrics. 2014;14(1):1-30.

Lau FC, Bielinski DF, Joseph JA. Inhibitory effects of blueberry extract on the production of inflammatory mediators in lipopolysaccharide-activated BV2 microglia. Journal of neuroscience research. 2007 Apr;85(5):1010-7.

Lavagna SM, Secci D, Chimenti P, Bonsignore L, Ottaviani A, Bizzarri B. Efficacy of *Hypericum* and *Calendula* oils in the epithelial reconstruction of surgical wounds in childbirth with caesarean section. Farmaco. 2001 May-Jul;56(5-7):451-3. doi: 10.1016/s0014-827x(01)01060-6.

Le MH, Do TT, Phan VK, Chau VM, Nguyen TH, Nguyen XN, Bui HT, Pham QL, Bui KA, Kim SH, Hong HJ. Chemical constituents of the rhizome of *Eleutherine bulbosa* and their inhibitory effect on the pro-Inflammatory cytokines production in lipopolysaccharide-Stimulated Bone marrow-derived dendritic cells. Bulletin of the Korean Chemical Society. 2013;34(2):633-6. https://doi.org/10.5012/bkcs.2013.34.2.633

Le XT, Phi XT, Nguyen DT, Pham HT, Van TN, Matsumoto K. Angiotensin-converting enzyme inhibitory activity of some Vietnamese medicinal plants. Vietnam Journal of Science, Technology and Engineering. 2020 Jul 24;62(2):77-82. https://doi.org/10.31276/VJSTE.62(2).77-82

Leach AJ, Leach DN, Leach GJ. Antibacterial activity of some medicinal plants of Papua New Guinea. Sci New Guinea. 1988 Jan;14(1):1-7.

Léandre KK, Mathieu BN, Jean-Baptiste ON, André KB, Augustin AK, Claude AK, Paul YA, Etienne EE. Effects of leaf decoction from *Lophira lanceolata* Tiegh. Ex Keay (Ochnaceae) on arterial blood pressure and electrocardiogram in anesthetized rabbits. The Pharma Innovation. 2013 Nov 1;2(9, Part A):66.

Lecanu L, Hashim AI, McCourty A, Giscos-Douriez I, Dinca I, Yao W, Vicini S, Szabo G, Erdélyi F, Greeson J, Papadopoulos V. The naturally occurring steroid solasodine induces neurogenesis in vitro and in vivo. Neuroscience. 2011 Jun 2;183:251-64.

Lechaba NM, Schutte PJ, Hay L, Böhmer L, Govender MM. The effects of an aqueous leaf extract of *Clausena anisata* (Willd.) Hook. f. ex Benth. on blood pressure, urine output, angiotensin II levels and cardiac parameters in spontaneously hypertensive rats. Journal of Medicinal Plants Research. 2016 Jul 25;10(28):425-34. https://doi.org/10.5897/JMPR2016.6135

Lee AS, Lee YJ, Lee SM, Yoon JJ, Kim JS, Kang DG, Lee HS. *Portulaca oleracea* ameliorates diabetic vascular inflammation and endothelial dysfunction in db/db mice. Evidence-Based Complementary and Alternative Medicine. 2012 Oct;2012. https://doi.org/10.1155/2012/741824

Lee BC, Lee SY, Lee HJ, Sim GS, Kim JH, Kim JH, Cho YH, Lee DH, Pyo HB, Choe TB, Moon DC. Anti-oxidative and photo-protective effects of coumarins isolated from *Fraxinus chinensis.* Archives of Pharmacal Research. 2007 Oct 1;30(10):1293. doi:10.1007/BF02980270

Lee C, Kim SY, Eum S, Paik JH, Bach TT, Darshetkar AM, Choudhary RK, Hai DV, Quang BH, Thanh NT, Choi S. Ethnobotanical study on medicinal plants used by local Van Kieu ethnic people of Bac Huong Hoa nature reserve, Vietnam. J Ethnopharmacol. 2019 Mar 1;231:283-294. doi: 10.1016/j.jep.2018.11.006.

Lee HY, Yun MY, Kang SM. Anti-inflammatory effect of *Boswellia sacra* (Franckincense) essential oil in a mouse model of allergic asthma. Microbiology and Biotechnology Letters. 2008;36(4):343-52.

Lee IA, Lee JH, Baek NI, Kim DH. Antihyperlipidemic effect of crocin isolated from the fructus of *Gardenia jasminoides* and its metabolite crocetin. Biological and Pharmaceutical Bulletin. 2005;28(11):2106-10. https://doi.org/10.1248/bpb.28.2106

Lee JB, Miyake S, Umetsu R, Hayashi K, Chijimatsu T, Hayashi T. Anti-influenza A virus effects of fructan from Welsh onion (*Allium fistulosum* L.). Food chemistry. 2012 Oct 15;134(4):2164-8. https://doi.org/10.1016/j.foodchem.2012.04.016

Lee JE, Kim MS, Park SY. Effect of natural antioxidants on the aggregation and disaggregation of beta-amyloid. Tropical Journal of Pharmaceutical Research. 2017;16(11):2629-35. doi: 10.4314/tjpr.v16i11.9

Lee JY, Lee JY, Yun BS, Hwang BK. Antifungal activity of β-asarone from rhizomes of *Acorus gramineus.* Journal of agricultural and food chemistry. 2004 Feb 25;52(4):776-80.

Lee KH, Padzil AM, Syahida A, Abdullah N, Zuhainis SW, Maziah M, Sulaiman MR, Israf DA, Shaari K, Lajis NH. Evaluation of anti-inflammatory, antioxidant and anti-nociceptive activities of six Malaysian medicinal plants. Journal of Medicinal Plants Research. 2011 Oct 23;5(23):5555-63.

Lee KI, Kim SM. Antioxidative and antimicrobial activities of *Eriobotrya japonica* Lindl. leaf extracts. Journal of the Korean Society of Food Science and Nutrition. 2009;38(3):267-73. https://doi.org/10.3746/jkfn.2009.38.3.267

Lee KS, Lee KY. Biological activity of phenol compound from a cactus Cheonnyuncho (Opuntia humifusa) in Korea. Journal of the Korean Society of Food Science and Nutrition. 2010;39(8):1132-6. doi: 10.3746/jkfn.2010.39.8.1132

Lee MJ, Lee HS, Park SD, Moon HI, Park WH. *Leonurus sibiricus* herb extract suppresses oxidative stress and ameliorates hypercholesterolemia in C57BL/6 mice and TNF-α induced expression of adhesion molecules and lectin-like oxidized LDL receptor-1 in human umbilical vein endothelial cells. Bioscience, biotechnology, and biochemistry. 2010a Feb 23;74(2):279-84. https://doi.org/10.1271/bbb.90582

Lee MJ, Rao YK, Chen K, Lee YC, Chung YS, Tzeng YM. Andrographolide and 14-deoxy-11, 12-didehydroandrographolide from *Andrographis paniculata* attenuate high glucose-induced fibrosis and apoptosis in murine renal mesangeal cell lines. Journal of ethnopharmacology. 2010b Nov 11;132(2):497-505. https://doi.org/10.1016/j.jep.2010.07.057

Lee MY, Shin IS, Jeon WY, Lim HS, Kim JH, Ha H. *Pinellia ternata* Breitenbach attenuates ovalbumin-induced allergic airway inflammation and mucus secretion in a murine model of asthma. Immunopharmacology and immunotoxicology. 2013 Jun 1;35(3):410-8. https://doi.org/10.3109/08923973.2013.770522

Lee SJ, Lee HK, Jung MK, Mar W. In vitro antiviral activity of 1, 2, 3, 4, 6-penta-O-galloyl-β-D-glucose against hepatitis B virus. Biological and Pharmaceutical Bulletin. 2006;29(10):2131-4. https://doi.org/10.1248/bpb.29.2131

Lee SJ, Umano K, Shibamoto T, Lee KG. Identification of volatile components in basil (*Ocimum basilicum* L.) and thyme leaves (*Thymus vulgaris* L.) and their antioxidant properties. Food Chemistry. 2005 Jun 1;91(1):131-7. https://doi.org/10.1016/j.foodchem.2004.05.056

Lee Y, Lee JY. Blackcurrant (*Ribes nigrum*) Extract Exerts an Anti-Inflammatory Action by Modulating Macrophage Phenotypes. *Nutrients*. 2019;11(5):975. Published 2019 Apr 28. doi:10.3390/nu11050975

Lee YJ, Choi DH, Cho GH, Kim JS, Kang DG, Lee HS. *Arctium lappa* ameliorates endothelial dysfunction in rats fed with high fat/cholesterol diets. BMC complementary and alternative medicine. 2012 Dec;12(1):1-0. https://doi.org/10.1186/1472-6882-12-116

Lee YJ, Kang DG, Kim JS, Lee HS. *Lycopus lucidus* inhibits high glucose-induced vascular inflammation in human umbilical vein endothelial cells. Vascular pharmacology. 2008 Jan 1;48(1):38-46. https://doi.org/10.1016/j.vph.2007.11.004

Leelaprakash G, Dass SM. In vitro anti-inflammatory activity of methanol extract of *Enicostemma axillare*. International journal of drug development and research. 2011 Jul;3(3):189-96.

Leem E, Nam JH, Jeon MT, Shin WH, Won SY, Park SJ, Choi MS, Jin BK, Jung UJ, Kim SR. Naringin protects the nigrostriatal dopaminergic projection through induction of GDNF in a neurotoxin model of Parkinson's disease. The Journal of nutritional biochemistry. 2014 Jul 1;25(7):801-6. doi: 10.1016/j.jnutbio.2014.03.006.

Leeya Y, Mulvany MJ, Queiroz EF, Marston A, Hostettmann K, Jansakul C. Hypotensive activity of an n-butanol extract and their purified compounds from leaves of *Phyllanthus acidus* (L.) Skeels in rats. European journal of pharmacology. 2010 Dec 15;649(1-3):301-13. doi.org/10.1016/j.ejphar.2010.09.038

Leguizamón ND, Rodrigues EM, de Campos ML, Nogueira AV, Viola KS, Schneider VK, Neo-Justino DM, Tanomaru-Filho M, Zambuzzi WF, Henrique-Silva F, Soares-Costa A. In vivo and in vitro anti-inflammatory and pro-osteogenic effects of citrus cystatin CsinCPI-2. Cytokine. 2019 Nov 1;123:154760. doi: 10.1016/j.cyto.2019.154760.

Leite SP, Vieira JR, de Medeiros PL, Leite RM, de Menezes Lima VL, Xavier HS, de Oliveira Lima E. Antimicrobial activity of *Indigofera suffruticosa*. Evidence-Based Complementary and Alternative Medicine. 2006;3(2):261-5. http://dx.doi.org/10.1093/ecam/nel010

Lenta BN, Ngouela S, Noungoue DT, Tsamo E, Connolly JD. Symphonin: A new prenylated pyranoxanthone with antimicrobial activity from the seeds of *Symphonia globulifera* (Guttiferae). Bulletin of the chemical Society of Ethiopia. 2004;18(2). doi: 10.4314/bcse.v18i2.61439

Lenta BN, Weniger B, Antheaume C, Noungoue DT, Ngouela S, Assob JC, Vonthron-Sénécheau C, Fokou PA, Devkota KP, Tsamo E, Sewald N. Anthraquinones from the stem bark of *Stereospermum zenkeri* with antimicrobial activity. Phytochemistry. 2007 Jun 1;68(11):1595-9. https://doi.org/10.1016/j.phytochem.2007.03.037

Leow SS, Sekaran SD, Tan Y, Sundram K, Sambanthamurthi R. Oil palm phenolics confer neuroprotective effects involving cognitive and motor functions in mice. Nutritional neuroscience. 2013 Sep 1;16(5):207-17. doi:10.1179/1476830512Y.0000000047

Lescano CH, Iwamoto RD, Sanjinez-Argandoña EJ, Kassuya CA. Diuretic and Anti-Inflammatory Activities of the Microencapsulated *Acrocomia aculeata* (Arecaceae) Oil on Wistar Rats. *J Med Food*. 2015;18(6):656–662. doi:10.1089/jmf.2014.0077

Li A, Zhu Y, He X, Tian X, Xu L, Ni W, Jiang P. Evaluation of antimicrobial activity of certain Chinese plants used in folkloric medicine. World Journal of Microbiology and Biotechnology. 2008 Apr 1;24(4):569-72. https://doi.org/10.1007/s11274-007-9494-4

Li C, Guo XD, Lei M, Wu JY, Jin JZ, Shi XF, Zhu ZY, Rukachaisirikul V, Hu LH, Wen TQ, Shen X. *Thamnolia vermicularis* extract improves learning ability in APP/PS1 transgenic mice by ameliorating both Aβ and Tau pathologies. Acta Pharmacologica Sinica. 2017 Jan;38(1):9-28. https://doi.org/10.1038/aps.2016.94

Li C, Zhang C, Zhou H, et al. Inhibitory Effects of Betulinic Acid on LPS-Induced Neuroinflammation Involve M2 Microglial Polarization via CaMKKβ-Dependent AMPK Activation. *Front Mol Neurosci*. 2018;11:98. Published 2018 Apr 3. doi:10.3389/fnmol.2018.00098

Li C, Zhang L. In vivo anti-fatigue activity of total flavonoids from sweetpotato [*Ipomoea batatas* (L.) Lam.] leaf in mice. Indian Journal of Biochemistry and Biophysics 50(4) [August 2013]: 266-272.

Li C, Zhang WJ, Frei B. Quercetin inhibits LPS-induced adhesion molecule expression and oxidant production in human aortic endothelial cells by p38-mediated Nrf2 activation and antioxidant enzyme induction. Redox Biol. 2016;9:104–113. doi:10.1016/j.redox.2016.06.006

Li CC, Lee YC, Lo HY, Huang YW, Hsiang CY, Ho TY. Antihypertensive Effects of Corn Silk Extract and Its Novel Bioactive Constituent in Spontaneously Hypertensive Rats: The Involvement of Angiotensin-Converting Enzyme Inhibition. Molecules. 2019;24(10):1886. Published 2019 May 16. doi:10.3390/molecules24101886

Li H, Deng Z, Liu R, Loewen S, Tsao R. Bioaccessibility, in vitro antioxidant activities and in vivo anti-inflammatory activities of a purple tomato (*Solanum lycopersicum* L.). Food chemistry. 2014 Sep 15;159:353-60. https://doi.org/10.1016/j.foodchem.2014.03.02

Li H, Park JH, Yan B, Yoo KY, Lee CH, Choi JH, Hwang IK, Won MH. Neuroprotection of *Alpinia katsumadai* seed extract against neuronal damage in the ischemic gerbil hippocampus is linked to altered brain-derived neurotrophic factor. Laboratory animal research. 2011a Mar 1;27(1):67-71. https://doi.org/10.5625/lar.2011.27.1.67

Li H, Prairie N, Udenigwe CC, Adebiyi AP, Tappia PS, Aukema HM, Jones PJ, Aluko RE. Blood pressure lowering effect of a pea protein hydrolysate in hypertensive rats and humans. Journal of agricultural and food chemistry. 2011b Sep 28;59(18):9854-60. dx.doi.org/10.1021/jf201911p

Li Hi Shing S, Chipika RH, Finegan E, Murray D, Hardiman O, Bede P. Post-polio Syndrome: More Than Just a Lower Motor Neuron Disease. Front Neurol. 2019;10:773. https://doi.org/10.3389/fneur.2019.00773

Li J, Huang X, Du X, Sun W, Zhang Y. Study of chemical composition and antimicrobial activity of leaves and roots of *Scrophularia ningpoensis*. Natural product research. 2009 May 20;23(8):775-80. https://doi.org/10.1080/14786410802696247

Li J, Liang X, Zhou B, Chen X, Xie P, Jiang H, Jiang Z, Yang Z, Pan X. (+)-pinoresinol-O-β-D-glucopyranoside from *Eucommia ulmoides* Oliver and its anti-inflammatory and antiviral effects against influenza A (H1N1) virus infection. Molecular medicine reports. 2019 Jan 1;19(1):563-72. https://doi.org/10.3892/mmr.2018.9696

Li J, Shi Z, Mi Y. Purple sweet potato color attenuates high fat-induced neuroinflammation in mouse brain by inhibiting MAPK and NF-κB activation. Molecular medicine reports. 2018 Mar 1;17(3):4823-31.

Li J, Zhang CX, Liu YM, Chen KL, Chen G. A comparative study of anti-aging properties and mechanism: resveratrol and caloric restriction. Oncotarget. 2017 Aug 9;8(39):65717-65729. doi: 10.18632/oncotarget.20084.

Li M. In vitro anti-respiratory syncytial virus effect of the extraction of *Lonicera japonica* Thunb. Journal of Tropical Medicine (Guangzhou). 2010;10(4):420-2.

Li R, Huang YG, Fang D, Le WD. (−)-Epigallocatechin gallate inhibits lipopolysaccharide-induced microglial activation and protects against inflammation-mediated dopaminergic neuronal injury. Journal of neuroscience research. 2004 Dec 1;78(5):723-31. https://doi.org/10.1002/jnr.20315

Li R, Yang JJ, Song XZ, Wang YF, Corlett RT, Xu YK, Hu HB. Chemical Composition and the Cytotoxic, Antimicrobial, and Anti-Inflammatory Activities of the Fruit Peel Essential Oil from *Spondias pinnata* (Anacardiaceae) in Xishuangbanna, Southwest China. Molecules. 2020 Jan;25(2):343. https://doi.org/10.3390/molecules25020343

Li RW, Myers SP, Leach DN, Lin GD, Leach G. A cross-cultural study: anti-inflammatory activity of Australian and Chinese plants. Journal of Ethnopharmacology. 2003 Mar 1;85(1):25-32. https://doi.org/10.1016/S0378-8741(02)00336-7

Li SY, Chen C, Zhang HQ, Guo HY, Wang H, Wang L, Zhang X, Hua SN, Yu J, Xiao PG, Li RS. Identification of natural compounds with antiviral activities against SARS-associated coronavirus. Antiviral research. 2005 Jul 1;67(1):18-23. https://doi.org/10.1016/j.antiviral.2005.02.007

Li XT, Chen R, Gao MB. Protective Effects on Mitochondria and Anti-Aging Activity of Aqueous Extract of *Forsythia suspensa* Leavies [J]. Journal of Food Science and Biotechnology. 2009;6.

Li Y, But PP, Ooi VE. Antiviral activity and mode of action of caffeoylquinic acids from *Schefflera heptaphylla* (L.) Frodin. Antiviral Research. 2005 Oct 1;68(1):1-9. https://doi.org/10.1016/j.antiviral.2005.06.004

Li Y, Xu W, Zhang F, Zhong S, Sun Y, Huo J, Zhu J, Wu C. The Gut Microbiota-Produced Indole-3-Propionic Acid Confers the Antihyperlipidemic Effect of Mulberry-Derived 1-Deoxynojirimycin. mSystems. 2020 Oct 6;5(5):e00313-20. doi: 10.1128/mSystems.00313-20.

Li YM, Chan HY, Huang Y, Chen ZY. Green tea catechins upregulate superoxide dismutase and catalase in fruit flies. Molecular nutrition & food research. 2007 May;51(5):546-54.

Li ZT, Sun JX, Zhu HX, Chu ZF. Extracting of *Polygonatum* polysaccharides and its antimicrobial activity. Food Res. Dev. 2017;38:36-8.

Lian F, Wang XD. Enzymatic metabolites of lycopene induce Nrf2-mediated expression of phase II detoxifying/antioxidant enzymes in human bronchial epithelial cells. International journal of cancer. 2008 Sep 15;123(6):1262-8.

Liao CR, Kao CP, Peng WH, Chang YS, Lai SC, Ho YL. Analgesic and anti-inflammatory activities of methanol extract of *Ficus pumila* L. in mice. Evidence-Based Complementary and Alternative Medicine. 2012;2012. http://dx.doi.org/10.1155/2012/340141

Liao JC, Chang WT, Lee MS, Chiu YJ, Chao WK, Lin YC, Lin MK, Peng WH. Antinociceptive and anti-inflammatory activities of *Cuscuta chinensis* seeds in mice. The American journal of Chinese medicine. 2014;42(01):223-42. https://doi.org/10.1142/S0192415X14500153

Lietti A, Cristoni A, Picci M. Studies on *Vaccinium myrtillus* anthocyanosides. I. Vasoprotective and antiinflammatory activity. Arzneimittel-Forschung. 1976 Jan 1;26(5):829-32.

Light ME, McGaw LJ, Sparg SG, Jäger AK, Van Staden J. Screening of *Cenchrus ciliaris* L. for biological activity. South African journal of botany. 2002 Sep 1;68(3):411-3. https://doi.org/10.1016/S0254-6299(15)30410-5

Lim HJ, Dong GZ, Lee HJ, Ryu JH. In vitro neuroprotective activity of sesquiterpenoids from the flower buds of *Tussilago farfara*. Journal of enzyme inhibition and medicinal chemistry. 2015 Sep 3;30(5):852-6. https://doi.org/10.3109/14756366.2014.965701

Lim HS, Kim YJ, Kim BY, Park G, Jeong SJ. The anti-neuroinflammatory activity of tectorigenin pretreatment via downregulated NF-κB and ERK/JNK pathways in BV-2 microglial and microglia inactivation in mice with lipopolysaccharide. Frontiers in pharmacology. 2018 May 9;9:462. https://doi.org/10.3389/fphar.2018.00462

Lim JP, Choi H. Effects of the water extract from *Cucurbita maxima* Duchesne on inflammation and hyperlipidemia in rats. Korean Journal of Medicinal Crop Science. 2001;9(4):280-3.

Lim TY, Lim YY, Yule CM. Evaluation of antioxidant, antibacterial and anti-tyrosinase activities of four Macaranga species. Food Chemistry. 2009 May 15;114(2):594-9. doi:10.1016/j.foodchem.2008.09.093

Lima GR, Montenegro CD, Almeida CL, Athayde-Filho PF, Barbosa-Filho JM, Batista LM. Database survey of anti-inflammatory plants in South America: A review. International journal of molecular sciences. 2011 Apr;12(4):2692-749.

Lima JC, Martins DT, de Souza Jr PT. Experimental evaluation of stem bark of *Stryphnodendron adstringens* (Mart.) Coville for antiinflammatory activity. Phytotherapy Research: An International Journal Devoted to Pharmacological and Toxicological Evaluation of Natural Product Derivatives. 1998 May;12(3):218-20. https://doi.org/10.1002/(SICI)1099-1573(199805)12:3<218::AID-PTR220>3.0.CO;2-4

Lima LM, Perazzo FF, Carvalho JC, Bastos JK. Anti-inflammatory and analgesic activities of the ethanolic extracts from *Zanthoxylum riedelianum* (Rutaceae) leaves and stem bark. Journal of Pharmacy and Pharmacology. 2007 Aug;59(8):1151-8. https://doi.org/10.1211/jpp.59.8.0014

Lima NDS, Teixeira L, Gambero A, Ribeiro ML. Guarana (*Paullinia cupana*) Stimulates Mitochondrial Biogenesis in Mice Fed High-Fat Diet. *Nutrients*. 2018;10(2):165. Published 2018 Jan 31. doi:10.3390/nu10020165

Lima RD, Brondani JC, Dornelles RC, Lhamas CL, Faccin H, Silva CV, Dalmora SL, Manfron MP. Anti-inflammatory activity and identification of the *Verbena litoralis* Kunth crude extract constituents. Brazilian Journal of Pharmaceutical Sciences. 2020;56. https://doi.org/10.1590/s2175-97902019000417419

Lima SM, Bernardo BF, Yamada SS, Reis BF, da Silva GM, Galvão MA. Effects of *Glycine max* (L.) Merr. soy isoflavone vaginal gel on epithelium morphology and estrogen receptor expression in postmenopausal women: a 12-week, randomized, double-blind, placebo-controlled trial. Maturitas. 2014 Jul 1;78(3):205-11. https://doi.org/10.1016/j.maturitas.2014.04.007

Lin CH, Huang JL, Zhang L, Hai-Yan TI, Sheng YI. Jatrogricaine A: a new diterpenoid with a 5/6/6/4 carbon ring system from the stems of *Jatropha podagrica*. Chinese journal of natural medicines. 2019 Apr 1;17(4):298-302. https://doi.org/10.1016/S1875-5364(19)30033-0

Lin CL, Lin SY, Lin YH, Hou WC. Effects of tuber storage protein of yam (*Dioscorea alata* cv. Tainong No. 1) and its peptic hydrolyzates on spontaneously hypertensive rats. Journal of the Science of Food and Agriculture. 2006 Aug 15;86(10):1489-94.https://doi.org/10.1002/jsfa.2530

Lin KH, Yang YY, Yang CM, Huang MY, Lo HF, Liu KC, Lin HS, Chao PY. Antioxidant activity of herbaceous plant extracts protect against hydrogenperoxide-induced DNA damage in human lymphocytes. BMC research notes. 2013 Dec 1;6(1):490. https://doi.org/10.1186/1756-0500-6-490

Lin LG, Yang XZ, Tang CP, Ke CQ, Zhang JB, Ye Y. Antibacterial stilbenoids from the roots of *Stemona tuberosa.* Phytochemistry. 2008 Jan 1;69(2):457-63. https://doi.org/10.1016/j.phytochem.2007.07.012

Lin MK, Lee MS, Huang HC, Cheng TJ, Cheng YD, Wu CR. *Cuscuta chinensis* and *C. campestris* attenuate scopolamine-induced memory deficit and oxidative damage in mice. Molecules. 2018 Dec;23(12):3060.https://doi.org/10.3390/molecules23123060

Lin P, Hwang E, Ngo HT, Seo SA, Yi TH. *Sambucus nigra* L. ameliorates UVB-induced photoaging and inflammatory response in human skin keratinocytes. Cytotechnology. 2019 Oct 1;71(5):1003-17. doi: 10.1007/s10616-019-00342-1

Lin SY, Wang CC, Lu YL, Wu WC, Hou WC. Antioxidant, anti-semicarbazide-sensitive amine oxidase, and anti-hypertensive activities of geraniin isolated from *Phyllanthus urinaria*. Food and Chemical Toxicology. 2008 Jul 1;46(7):2485-92. https://doi.org/10.1016/j.fct.2008.04.007

Lin WC, Wen CC, Chen YH, Hsiao PW, Liao JW, Peng CI, Yang NS. Integrative approach to analyze biodiversity and anti-inflammatory bioactivity of *Wedelia* medicinal plants. PloS one. 2015;10(6). doi: 10.1371/journal.pone.0129067

Lin Y, Wu S. Vegetable soybean (*Glycine max* (L.) Merr.) leaf extracts: Functional components and antioxidant and anti-inflammatory activities. Journal of Food Science. 2021 Jun;86(6):2468-80. https://doi.org/10.1111/1750-3841.15765

Lin YC, Lin CH, Yao HT, Kuo WW, Shen CY, Yeh YL, Ho TJ, Padma VV, Lin YC, Huang CY. *Platycodon grandiflorum* (PG) reverses angiotensin II-induced apoptosis by repressing IGF-IIR expression. Journal of ethnopharmacology. 2017 Jun 9;205:41-50. https://doi.org/10.1016/j.jep.2017.04.028

Ling SK, Pisar MM, Man S. Platelet-Activating Factor (PAF) Receptor Binding Antagonist Activity of the Methanol Extracts and Isolated Flavonoids from *Chromolaena odorata* (L.) K ING and R OBINSON. Biological and Pharmaceutical Bulletin. 2007;30(6):1150-2.□https://doi.org/10.1248/bpb.30.1150

Lingaraju DP, Sudarshana MS, Mahendra C, Rao KP. Phytochemical screening and antimicrobial activity of leaf extracts of *Eryngium foetidum* L.(Apiaceae). Indo American Journal of Pharmaceutical Research. 2016;6(2):4339-44.

Lingaraju GM, Krishna V, Joy Hoskeri H, Pradeepa K, Venkatesh, Babu PS. Wound healing promoting activity of stem bark extract of *Semecarpus anacardium* using rats. Natural product research. 2012 Dec 1;26(24):2344-7. https://doi.org/10.1080/14786419.2012.656108

Linnet A, Latha PG, Gincy MM, Anuja GI, Suja SR, Shyamal S, Shine VJ. Anti-inflammatory, analgesic and anti-lipid peroxidative effects of *Rhaphidophora pertusa* (Roxb.) Schott. and *Epipremnum pinnatum* (Linn.) Engl. aerial parts. Indian Journal of Natural Products and Resources 2010 Mar, Vol.1(1): 5-10.

Lino CS, Taveira ML, Viana GS, Matos FJ. Analgesic and antiinflammatory activities of *Justicia pectoralis* Jacq and its main constituents: coumarin and umbelliferone. Phytotherapy Research: An International Journal Devoted to Medical and

Scientific Research on Plants and Plant Products. 1997 May;11(3):211-5. doi.org/10.1002/(SICI)1099-1573(199705)11:3<211::AID-PTR72>3.0.CO;2-W

Lipipun V, Nantawanit N, Pongsamart S. Antimicrobial activity (in vitro) of polysaccharide gel from durian fruit-hulls. Songklanakarin J Sci Technol. 2002;24(1):31-8.

Liu AL, Liu B, Qin HL, Lee SM, Wang YT, Du GH. Anti-influenza virus activities of flavonoids from the medicinal plant *Elsholtzia rugulosa*. Planta medica. 2008 Jun;74(08):847-51. doi: 10.1055/s-2008-1074558

Liu C, Yin H, Gao J, Xu X, Zhang T, Yang Z. Leonurine ameliorates cognitive dysfunction via antagonizing excitotoxic glutamate insults and inhibiting autophagy. Phytomedicine. 2016 Dec 1;23(13):1638-46. https://doi.org/10.1016/j.phymed.2016.10.005

Liu H, Wang L, Wang MH. Antioxidant and nitric oxide release inhibition activities of methanolic extract from *Clerodendrum cyrtophyllum* Turcz. Horticulture, Environment, and Biotechnology. 2011 Jun 1;52(3):309-14. doi: 10.1007/s13580-011-0204-7

Liu J, Hao Y, Wang Z, Ni F, Wang Y, Gong L, Sun B, Wang J. Identification, quantification, and anti-inflammatory activity of 5-n-Alkylresorcinols from 21 different wheat varieties. Journal of agricultural and food chemistry. 2018 Aug 14;66(35):9241-7. doi: 10.1021/acs.jafc.8b02911.

Liu J, Zhou L, Zhou Q, Lian Q. Animal experiment of the fatigue-resisting and anoxia-resisting actions of *Pholidota chinensis* lindl. Chinese Journal of Tissue Engineering Research. 2006 Jan 1;10(7):157-9.

Liu M, Amini A, Ahmad Z. Safranal and its analogs inhibit Escherichia coli ATP synthase and cell growth. *Int J Biol Macromol.* 2016;95:145-152.

Liu MZ, Yang Y, Zhang SX, Tang L, Wang HM, Chen CJ, Shen ZF, Cheng KD, Kong JQ, Wang W. A cyclotide against influenza A H1N1 virus from *Viola yedoensis*. Yao xue xue bao= Acta pharmaceutica Sinica. 2014 Jun;49(6):905-12.

Liu R, Wu L, Du Q, Ren JW, Chen QH, Li D, Mao RX, Liu XR, Li Y. Small Molecule Oligopeptides Isolated from Walnut (*Juglans regia* L.) and Their Anti-Fatigue Effects in Mice. Molecules. 2019 Jan;24(1):45. https://doi.org/10.3390/molecules24010045

Liu S, Qin X, LIi S. Effects of polysaccharides from *Polygonatum* on fatigue in mice [J]. China Modern Medicine. 2009; 16:31–32.

Liu S, You L, Zhao Y, Chang X. Wild Lonicera caerulea berry polyphenol extract reduces cholesterol accumulation and enhances antioxidant capacity in vitro and in vivo. Food Research International. 2018 May 1;107:73-83. https://doi.org/10.1016/j.foodres.2018.02.016

Liu SG, Ren PY, Wang GY, Yao SX, He XJ. Allicin protects spinal cord neurons from glutamate-induced oxidative stress through regulating the heat shock protein 70/inducible nitric oxide synthase pathway. Food & function. 2015;6(1):320-9.

Liu X, Zhao M, Luo W, Yang B, Jiang Y. Identification of volatile components in *Phyllanthus emblica* L. and their antimicrobial activity. Journal of Medicinal food. 2009 Apr 1;12(2):423-8. https://doi.org/10.1089/jmf.2007.0679

Liu Y, Duan C, Chen H, Wang C, Liu X, Qiu M, Tang H, Zhang F, Zhou X, Yang J. Inhibition of COX-2/mPGES-1 and 5-LOX in macrophages by leonurine ameliorates monosodium urate crystal-induced inflammation. Toxicology and Applied Pharmacology. 2018 Jul 15;351:1-1. https://doi.org/10.1016/j.taap.2018.05.010

Liu Z, Ni H, Yu L, Xu S, Bo R, Qiu T, Gu P, Zhu T, He J, Wusiman A, Zhu S. Adjuvant activities of CTAB-modified *Polygonatum sibiricum* polysaccharide cubosomes on immune responses to ovalbumin in mice. International Journal of Biological Macromolecules. 2020 Apr 1;148:793-801. doi: 10.1016/j.ijbiomac.2020.01.174

Lizogub VG, Riley DS, Heger M. Efficacy of a Pelargonium sidoides preparation in patients with the common cold: A randomized, double blind, placebo-controlled clinical trial. Explore (NY) 2007; 3:573–584.

Local Food-Nutraceuticals Consortium. Understanding local Mediterranean diets: a multidisciplinary pharmacological and ethnobotanical approach. Pharmacological Research. 2005 Oct 1;52(4):353-66. https://doi.org/10.1016/j.phrs.2005.06.005

Lockyer S, Corona G, Yaqoob P, Spencer JP, Rowland I. Secoiridoids delivered as olive leaf extract induce acute improvements in human vascular function and reduction of an inflammatory cytokine: a randomised, double-blind, placebo-controlled, cross-over trial. British Journal of Nutrition. 2015 Jul;114(1):75-83. doi: 10.1017/S0007114515001269.

Lodhi S, Jain AP, Rai G, Yadav AK. Preliminary investigation for wound healing and anti-inflammatory effects of *Bambusa vulgaris* leaves in rats. Journal of Ayurveda and integrative medicine. 2016 Mar 1;7(1):14-22.

Loganayaki N, Siddhuraju P, Manian S. Antioxidant activity of two traditional Indian vegetables: *Solanum nigrum* L. and *Solanum torvum* L. Food Science and Biotechnology. 2010 Feb 1;19(1):121-7. https://doi.org/10.1007/s10068-010-0017-y

Loganayaki N, Siddhuraju P, Manian S. Antioxidant, anti-inflammatory and anti-nociceptive effects of *Ammannia baccifera* L.(Lythracceae), a folklore medicinal plant. Journal of Ethnopharmacology. 2012 Mar 27;140(2):230-3. https://doi.org/10.1016/j.jep.2012.01.001

Loh YC, Ch'ng YS, Tan CS, Ahmad M, Asmawi MZ, Yam MF. Mechanisms of action of *Uncaria rhynchophylla* ethanolic extract for its vasodilatory effects. Journal of medicinal food. 2017 Sep 1;20(9):895-911. . doi: 10.1089/jmf.2016.3804.

Loizzo MR, Saab AM, Tundis R, Statti GA, Menichini F, Lampronti I, Gambari R, Cinatl J, Doerr HW. Phytochemical analysis and in vitro antiviral activities of the essential oils of seven Lebanon species. Chemistry & biodiversity. 2008 Mar;5(3):461-70. https://doi.org/10.1002/cbdv.200890045

Lokho A. The folk medicinal plants of the Mao Naga in Manipur, North East India. International Journal of Scientific and Research Publications. 2012;2(6):1-8.

Lone PA, Bhardwaj AK, Bahar FA. Traditional knowledge on healing properties of plants in Bandipora district of Jammu and Kashmir, India. International Journal of Recent Scientific Research. 2013;4(11):1755-65.

Lone PA, Bhardwaj AK, Shah KW, Tabasum S. Ethnobotanical survey of some threatened medicinal plants of Kashmir Himalaya, India. Journal of Medicinal Plants Research. 2014 Dec 17;8(47):1362-73.

Lone PA, Bhardwaj AK. Traditional herbal based disease treatment in some rural areas of Bandipora district of Jammu and Kashmir, India. Asian Journal of Pharmaceutical and Clinical Research. 2013;6(4):162-71.

Longuefosse JL, Nossin E. Medical ethnobotany survey in Martinique. J Ethnopharmacol. 1996 Sep;53(3):117-42. doi: 10.1016/0378-8741(96)01425-0.

Lopatkin N, Sivkov A, Schläfke S, Funk P, Medvedev A, Engelmann U. Efficacy and safety of a combination of *Sabal* and *Urtica* extract in lower urinary tract symptoms—long-term follow-up of a placebo-controlled, double-blind, multicenter trial. International urology and nephrology. 2007 Dec 1;39(4):1137-46.

Lopes RH, Macorini LF, Antunes KÁ, Espindola PP, Alfredo TM, Rocha PD, Pereira ZV, Santos EL, de Picoli Souza K. Antioxidant and hypolipidemic activity of the hydroethanolic extract of *Curatella americana* L. leaves. Oxidative medicine and cellular longevity. 2016;2016. Article ID 9681425, 6 pages doi.org/10.1155/2016/9681425

Lopes-Martins RA, Pegoraro DH, Woisky R, Penna SC, Sertié JA. The anti-inflammatory and analgesic effects of a crude extract of *Petiveria alliacea* L.(Phytolaccaceae). Phytomedicine. 2002 Jan 1;9(3):245-8. https://doi.org/10.1078/0944-7113-00118

Lopez A, Hudson JB, Towers GH. Antiviral and antimicrobial activities of Colombian medicinal plants. Journal of Ethnopharmacology. 2001 Oct 1;77(2-3):189-96.

López V, Jäger AK, Akerreta S, Cavero RY, Calvo MI. Pharmacological properties of *Anagallis arvensis* L.("scarlet pimpernel") and *Anagallis foemina* Mill.("blue pimpernel") traditionally used as wound healing remedies in Navarra (Spain). Journal of ethnopharmacology. 2011 Apr 12;134(3):1014-7. https://doi.org/10.1016/j.jep.2010.12.036

López V, Martín S, Gómez-Serranillos MP, Carretero ME, Jäger AK, Calvo MI. Neuroprotective and neurochemical properties of mint extracts. Phytotherapy Research. 2010 Jun;24(6):869-74. https://doi.org/10.1002/ptr.3037

López-Otín C, Blasco MA, Partridge L, Serrano M, Kroemer G. The hallmarks of aging. Cell. 2013;153(6):1194-217. https://doi.org/10.1016/j.cell.2013.05.039

López-Vargas JH, Fernández-López J, Pérez-Álvarez JA, Viuda-Martos M. Chemical, physico-chemical, technological, antibacterial and antioxidant properties of dietary fiber powder obtained from yellow passion fruit (*Passiflora edulis* var. flavicarpa) co-products. Food Research International. 2013 May 1;51(2):756-63. https://doi.org/10.1016/j.foodres.2013.01.055

Lopresti AL, Smith SJ, Malvi H, Kodgule R. An investigation into the stress-relieving and pharmacological actions of an ashwagandha (*Withania somnifera*) extract: A randomized, double-blind, placebo-controlled study. Medicine. 2019 Sep;98(37). doi: 10.1097/MD.0000000000017186.

Lorenzetti LJ, Salisbury R, Beal JL, Baldwin JN. Bacteriostatic property of *Aloe vera*. J Pharm Sci. 1964 Oct 1;53:1287.

Lorigooini Z, Ayatollahi SA, Amidi S, Kobarfard F. Evaluation of Anti-Platelet Aggregation Effect of Some Allium Species. Iran J Pharm Res. 2015;14(4):1225–1231.

Loro JF, Del Rio I, Perez-Santana L. Preliminary studies of analgesic and anti-inflammatory properties of *Opuntia dillenii* aqueous extract. Journal of ethnopharmacology. 1999 Nov 1;67(2):213-8. https://doi.org/10.1016/S0378-8741(99)00027-6

Lowe HI, Daley DK, Lindo J, Davis C, Rainford L, Hartley SA, Watson C, Chambers C, Reynolds-Campbell G, Foster SR, Bahadoosingh P. The antibacterial and antifungal analysis of crude extracts from the leaves and bark of *Pimenta* species found in Jamaica. Journal of Medicinal Plants Research Vol. 11(38), pp. 591-595, 10 October, 2017. doi: 10.5897/JMPR2017.6435

Lowe HI, Toyang NJ, Heredia A, Watson CT, Bryant J. Anti HIV-1 activity of the crude extracts of *Guaiacum officinale* L.(Zygophyllaceae). European Journal of Medicinal Plants. 2014 Apr 1;4(4):483-9.

Lozano CM, Vasquez-Tineo MA, Ramirez M, Jimenez F. In vitro antimicrobial activity screening of tropical medicinal plants used in Santo Domingo, Dominican Republic. Part I. Pharmacognosy Communications. 2013 Apr 1;3(2):64.

Lozano N, Bonilla P, Arroyo J, Arias G, Córdova A, Baldoceda F. Evaluación fitoquímica y actividad biológica de *Desmodium molliculum* (HBK) DC (Manayupa). Ciencia e Investigación. 2001;4(2):37-44.

Lu DY, Tang CH, Chen YH, Wei IH. Berberine suppresses neuroinflammatory responses through AMP-activated protein kinase activation in BV-2 microglia. Journal of cellular biochemistry. 2010 Jun 1;110(3):697-705.https://doi.org/10.1002/jcb.22580

Lu JH, Tan JQ, Durairajan SS, Liu LF, Zhang ZH, Ma L, Shen HM, Chan HE, Li M. Isorhynchophylline, a natural alkaloid, promotes the degradation of alpha-synuclein in neuronal cells via inducing autophagy. Autophagy. 2012 Jan 1;8(1):98-108. https://doi.org/10.4161/auto.8.1.18313

Lu P, Mamiya T, Lu LL, Mouri A, Zou LB, Nagai T, Hiramatsu M, Ikejima T, Nabeshima T. Silibinin prevents amyloid β peptide-induced memory impairment and oxidative stress in mice. British journal of pharmacology. 2009 Aug;157(7):1270-7. https://doi.org/10.1111/j.1476-5381.2009.00295.x

Lu SY, Qiao YJ, Xiao PG, Tan XH. Identification of antiviral activity of *Toddalia asiatica* against influenza type A virus. China Journal of Chinese Materia Medica. 2005;30(13):1000.

Lugun O, Bhoi S, Kujur P, Kumar DV, Surin WR. Evaluation of Antithrombotic Activities of *Solanum xanthocarpum* and *Tinospora cordifolia*. Pharmacognosy research. 2018 Jan;10(1):98.

Lui EM, Azike CG, Guerrero-Analco JA, Romeh AA, Pei H, Kaldas SJ, Arnason JT, Charpentier PA. Bioactive polysaccharides of American ginseng *Panax quinquefolius* L. in modulation of immune function: Phytochemical and pharmacological characterization. In The Complex World of Polysaccharides 2012 Oct 31. IntechOpen. doi: 10.5772/50741

Lulekal E, Kelbessa E, Bekele T, Yineger H. An ethnobotanical study of medicinal plants in Mana Angetu District, southeastern Ethiopia. Journal of ethnobiology and Ethnomedicine. 2008 Dec;4(1):1-0. http://www.ethnobiomed.com/content/4/1/10

Luo JZ, Luo L. American ginseng stimulates insulin production and prevents apoptosis through regulation of uncoupling protein-2 in cultured β cells. Evidence-Based Complementary and Alternative Medicine. 2006;3(3):365-72. https://doi.org/10.1093/ecam/nel026

Lwin OM, Giribabu N, Kilari EK, Salleh N. Topical administration of mangiferin promotes healing of the wound of streptozotocin-nicotinamide-induced type-2 diabetic male rats. J Dermatolog Treat. 2020 Feb 3:1-10. doi: 10.1080/09546634.2020.1721419.

Ma CJ, Lee KY, Jeong EJ, Kim SH, Park J, Choi YH, Kim YC, Sung SH. Persicarin from water dropwort (*Oenanthe javanica*) protects primary cultured rat cortical cells from glutamate-induced neurotoxicity. Phytotherapy research. 2010 Jun;24(6):913-8. doi.org/10.1002/ptr.3065

Ma CM, Nakamura N, Miyashiro H, Hattori M, Komatsu K, Kawahata T, Otake T. Screening of Chinese and Mongolian herbal drugs for anti-human immunodeficiency virus type 1 (HIV-1) activity. Phytotherapy Research: An International Journal Devoted to Pharmacological and Toxicological Evaluation of Natural Product Derivatives. 2002 Mar;16(2):186-9. https://doi.org/10.1002/ptr.922

Ma H, Johnson SL, Liu W, DaSilva NA, Meschwitz S, Dain JA, Seeram NP. Evaluation of polyphenol anthocyanin-enriched extracts of blackberry, black raspberry, blueberry, cranberry, red raspberry, and strawberry for free radical scavenging, reactive carbonyl species trapping, anti-glycation, anti-β-amyloid aggregation, and microglial neuroprotective effects. International journal of molecular sciences. 2018 Feb 3;19(2):461. http://doi.org/10.3390/ijms19020461

Ma J, Ren Q, Dong B, Shi Z, Zhang J, Jin DQ, Xu J, Ohizumi Y, Lee D, Guo Y. NO inhibitory constituents as potential anti-neuroinflammatory agents for AD from *Blumea balsamifera.* Bioorganic chemistry. 2018 Feb 1;76:449-57. doi.org/10.1016/j.bioorg.2017.12.008

Ma SC, Du J, But PP, Deng XL, Zhang YW, Ooi VE, Xu HX, Lee SH, Lee SF. Antiviral Chinese medicinal herbs against respiratory syncytial virus. Journal of ethnopharmacology. 2002 Feb 1;79(2):205-11. https://doi.org/10.1016/S0378-8741(01)00389-0

Mabrouk S, Salah KB, Elaissi A, Jlaiel L, Jannet HB, Aouni M, Harzallah-Skhiri F. Chemical composition and antimicrobial and allelopathic activity of Tunisian *Conyza sumatrensis* (Retz.) E. Walker essential oils. Chemistry & biodiversity. 2013 Feb;10(2):209-23. https://doi.org/10.1002/cbdv.201200117

Maccioni R, Setzu MD, Talani G, Solari P, Kasture A, Sucic S, Porru S, Muroni P, Sanna E, Kasture S, Acquas E. Standardized phytotherapic extracts rescue anomalous locomotion and electrophysiological responses of TDP-43 Drosophila melanogaster model of ALS. Scientific reports. 2018 Oct 30;8(1):1-0. https://doi.org/10.1038/s41598-018-34452-1

Machado TB, Leal IC, Kuster RM, Amaral AC, Kokis V, de Silva MG, dos Santos KR. Brazilian phytopharmaceuticals–evaluation against hospital bacteria. Phytotherapy Research. 2005 Jun;19(6):519-25. doi.org/10.1002/ptr.1696

Machiah DK, Gowda TV. Purification of a post-synaptic neurotoxic phospholipase A2 from *Naja naja* venom and its inhibition by a glycoprotein from *Withania somnifera*. Biochimie. 2006 Jun 1;88(6):701-10. https://doi.org/10.1016/j.biochi.2005.12.006

Madzuki IN, Lau SF, Abdullah R, Mohd Ishak NI, Mohamed S. *Vernonia amygdalina* inhibited osteoarthritis development by anti-inflammatory and anticollagenase pathways in cartilage explant and osteoarthritis-induced rat model. Phytotherapy Research. 2019 Jul;33(7):1784-93. Phytother Res. 2019 Jul;33(7):1784-1793. doi: 10.1002/ptr.6366. Epub 2019 Apr 29.

Maghrani M, Zeggwagh NA, Michel JB, Eddouks M. Antihypertensive effect of *Lepidium sativum* L. in spontaneously hypertensive rats. Journal of ethnopharmacology. 2005 Aug 22;100(1-2):193-7. https://doi.org/10.1016/j.jep.2005.02.024

Mahajan B, Shrestha TM, Gyawali R. Antibacterial and cytotoxic activity of *Juniperus indica* Bertol from Nepalese Himalaya. International Journal of Pharmaceutical Science and Research. 2012 Apr 1;3(4):1104-7

Mahajan K, Kumar D, Kumar S. Antiamnesic activity of extracts and fraction of *Desmodium gangeticum.* J Pharm Technol Res Manag. 2015;3:67–77.

Mahajan MS, Gulecha VS, Khandare RA, Upaganlawar AB, Gangurde HH, Upasani CD. Anti-edematogenic and analgesic activities of *Ficus benghalensis*. International Journal of Nutrition, Pharmacology, Neurological Diseases. 2012 May 1;2(2):100. doi: 10.4103/2231-0738.95936

Mahaman YA, Huang F, Wu M, Wang Y, Wei Z, Bao J, Salissou MT, Ke D, Wang Q, Liu R, Wang JZ. *Moringa oleifera* alleviates homocysteine-induced Alzheimer's Disease-like pathology and cognitive impairments. Journal of Alzheimer's Disease. 2018 Jan 1;63(3):1141-59. doi:  10.3233/JAD-180091

Mahboubi M, Haghi G. Antimicrobial activity and chemical composition of *Mentha pulegium* L. essential oil. Journal of ethnopharmacology. 2008 Sep 26;119(2):325-7. https://doi.org/10.1016/j.jep.2008.07.023

Mahendran S, Badami S, Ravi S, Thippeswamy BS, Veerapur VP. Synthesis and evaluation of analgesic and anti-inflammatory activities of most active free radical scavenging derivatives of Embelin—A Structure–Activity relationship. Chemical and Pharmaceutical Bulletin. 2011 Aug 1;59(8):913-9. doi.org/10.1248/cpb.59.913

Maher P, Dargusch R, Bodai L, Gerard PE, Purcell JM, Marsh JL. ERK activation by the polyphenols fisetin and resveratrol provides neuroprotection in multiple models of Huntington's disease. Human molecular genetics. 2011 Jan 15;20(2):261-70. https://doi.org/10.1093/hmg/ddq460

Maher P, Fischer W, Liang Z, Soriano-Castell D, Pinto AF, Rebman J, Currais A. The Value of Herbarium Collections to the Discovery of Novel Treatments for Alzheimer's Disease, a Case Made With the Genus *Eriodictyon*. Frontiers in Pharmacology. 2020 Mar 10;11: 208. https://doi.org/10.3389/fphar.2020.00208

Mähler A, Steiniger J, Bock M, Klug L, Parreidt N, Lorenz M, Zimmermann BF, Krannich A, Paul F, Boschmann M. Metabolic response to epigallocatechin-3-gallate in relapsing-remitting multiple sclerosis: a randomized clinical trial. The American journal of clinical nutrition. 2015 Jan 14;101(3):487-95. https://doi.org/10.3945/ajcn.113.075309

Mahmood A, Mahmood A, Tabassum A. Ethnomedicinal survey of plants from District Sialkot, Pakistan. J Appl Pharm. 2011;3:212-20.

Mahmood AA, Mariod AA, Abdelwahab SI, Ismail S, Al-Bayaty F. Potential activity of ethanolic extract of *Boesenbergia rotunda* (L.) rhizomes extract in accelerating wound healing in rats. Journal of Medicinal Plants Research. 2010 Aug 4;4(15):1570-6.

Mahmoudi M, Ebrahimzadeh MA, Ansaroudi F, Nabavi SF, Nabavi SM. Antidepressant and antioxidant activities of *Artemisia absinthium* L. at flowering stage. African journal of Biotechnology. 2009;8(24).

Mahmoudian M, Jalipour H, Salehian Dardashti P. Toxicity of *Peganum harmala*: review and a case report. Iranian Journal of Pharmacology and Therapeutics. 2002 Apr 10;1(1):1-0.

Mahyar UW, Burley JS, Gyllenhaal C, Soejarto DD. Medicinal plants of Seberida (Riau Province, Sumatra, Indonesia). J Ethnopharmacol. 1991 Feb;31(2):217-37. doi: 10.1016/0378-8741(91)90007-z.

Maione F, Piccolo M, De Vita S, Chini MG, Cristiano C, De Caro C, Lippiello P, Miniaci MC, Santamaria R, Irace C, De Feo V. Down regulation of pro-inflammatory pathways by tanshinone IIA and cryptotanshinone in a non-genetic mouse model of Alzheimer’s disease. Pharmacological research. 2018 Mar 1;129:482-90.

Majid A, Hassan S, Hussain W, Khan A, Hassan A, Khan A, Khan T, Ahmad T, Ur-Rehman M. In vitro Approaches of *Primula vulgaris* Leaves and Roots Extraction against Human Pathogenic Bacterial Strains. World Applied Sciences Journal. 2014;30(5):575-80. doi: 10.5829/idosi.wasj.2014.30.05.82264

Majumdar S, Roy S. Antibacterial and antioxidative activity of the leaves of *Daphniphyllum himalense* (Benth.) Muell. Arg. growing in Darjeeling hills. Journal of Traditional Medicines. 2012;7(2).

Makambila-Koubemba MC, Mbatchi B, Ardid D, Gelot A, Henrion C, Janisson R, Abena AA, Banzouzi JT. Pharmacological studies of ten medicinal plants used for analgesic purposes in Congo Brazzaville. Int. J. Pharmacol. 2011 Jul 1;7(5):608-15. doi: 10.3923/ijp.2011.608.615

Makare N, Bodhankar S, Rangari V. Immunomodulatory activity of alcoholic extract of *Mangifera indica* L. in mice. Journal of ethnopharmacology. 2001 Dec 1;78(2-3):133-7.https://doi.org/10.1016/S0378-8741(01)00326-9

Makgatho ME, Nxumalo W, Raphoko LA. Anti-mycobacterial,-oxidative,-proliferative and-inflammatory activities of dichloromethane leaf extracts of *Gymnosporia senegalensis* (Lam.) Loes. South African Journal of Botany. 2018 Jan 1;114:217-22. https://doi.org/10.1016/j.sajb.2017.11.002

Mak-Mensah EE, Komlaga G, Terlabi EO. Antiypertensive action of ethanolic extract of *Imperata cylindrica* leaves in animal models. Journal of Medicinal Plants Research. 2010 Jul 18;4(14):1486-91.

Malabadi RB. Antibacterial activity in the rhizome extracts of *Costus speciosus* (Koen.). Journal of Phytological Research. 2005;18(1):83-5.

Malairajan P, Gopalakrishnan G, Narasimhan S, Veni KJ, Kavimani S. Anti-ulcer activity of crude alcoholic extract of *Toona ciliata* Roemer (heart wood). Journal of ethnopharmacology. 2007 Mar 21;110(2):348-51. https://doi.org/10.1016/j.jep.2006.10.018

Malan DF, Neuba DF, Kouakou KL. Medicinal plants and traditional healing practices in Ehotile people, around the Aby Lagoon (eastern littoral of Côte d'Ivoire). J Ethnobiol Ethnomed. 2015 Mar 14;11:21. doi: 10.1186/s13002-015-0004-8.

Malathi R, Kaviyarasan D, Chandrasekar S. Evaluation of in vitro anti snake venom activity of *Justicia adhatoda* leaves extract against Russell’s viper snake venom. Journal of Drug Delivery and Therapeutics. 2019 Jul 15;9(4):116-22.

Malawani AD, Nuñeza OM, Uy MM, Senarath WTPSK. Ethnobotanical survey of the medicinal plants used by the Maranaos in Pualas, Lanao del Sur, Philippines. Bull Environ Pharmacol Life Sci. 2017;6(6):45–53.

Malayan J, Selvaraj B, Warrier A, Shanmugam S, Mathayan M, Menon T. Anti-mumps virus activity by extracts of *Mimosa pudica*, a unique Indian medicinal plant. Indian J Virol. 2013 Sep;24(2):166-73. doi: 10.1007/s13337-013-0143-2.

Malayeri SR, Rahimi H, Sadati SK, Behdari R. Brain-derived neurotrophic factor (BDNF) variation to aerobic exercise and aloe vera intake in women with type 2 diabetes. Journal of Exercise & Organ Cross Talk. 2021;1(1):1-7. doi: 10.22034/JEOCT.2021.281858.1001

Malekzadeh F. Antimicrobial activity of *Lawsonia inermis* L. Applied microbiology. 1968 Apr;16(4):663.

Mali VR, Mohan V, Bodhankar SL. Antihypertensive and cardioprotective effects of the *Lagenaria siceraria* fruit in N G-nitro-L-arginine methyl ester (L-NAME) induced hypertensive rats. Pharmaceutical biology. 2012 Nov 1;50(11):1428-35. doi.org/10.3109/13880209.2012.684064

Malik F, Hussain S, Sadiq A, Parveen G, Wajid A, Shafat S, Channa RA, Mahmood R, Riaz H, Ismail M, Raja FY. Phyto-chemical analysis, anti-allergic and anti-inflammatory activity of *Mentha arvensis* in animals. Afr J Pharm Pharmacol. 2012 Mar 8;6(9):613-9. doi: 10.5897/AJPP11.702

Malik J, Karan M, Dogra R. Ameliorating effect of *Celastrus paniculatus* standardized extract and its fractions on 3-nitropropionic acid induced neuronal damage in rats: possible antioxidant mechanism. *Pharm Biol*. 2017;55(1):980–990. doi:10.1080/13880209.2017.1285945

Malik JK, Manvi FV, Alagawadi KR, Noolvi M. Evaluation of anti-inflammatory activity of *Gymnema sylvestre* leaves extract in rats. International Journal of Green Pharmacy (IJGP). 2008;2(2). http://dx.doi.org/10.22377/ijgp.v2i2.42

Malik N, Dhawan V, Bahl A, Kaul D. Inhibitory effects of *Terminalia arjuna* on platelet activation in vitro in healthy subjects and patients with coronary artery disease. Platelets. 2009 Jan 1;20(3):183-90. https://doi.org/10.1080/09537100902809004

Malishev R, Shaham-Niv S, Nandi S, Kolusheva S, Gazit E, Jelinek R. Bacoside-A, an Indian traditional-medicine substance, inhibits β-amyloid cytotoxicity, fibrillation, and membrane interactions. ACS chemical neuroscience. 2017 Apr 19;8(4):884-91.

Malla B, Gauchan DP, Chhetri RB. An ethnobotanical study of medicinal plants used by ethnic people in Parbat district of western Nepal. J Ethnopharmacol. 2015 May 13;165:103-17. doi: 10.1016/j.jep.2014.12.057.

Malolo FA, Nouga AB, Kakam A, Franke K, Ngah L, Flausino O, Mpondo EM, Ntie-Kang F, Ndom JC, da Silva Bolzani V, Wessjohann L. Protease-inhibiting, molecular modeling and antimicrobial activities of extracts and constituents from *Helichrysum foetidum* and *Helichrysum mechowianum* (compositae). Chemistry Central Journal. 2015 Dec;9(1):32.

Malsawmtluangi C, Thanzami K, Lalhlenmawia H, Selvan V, Palanisamy S, Kandasamy R, Pachuau L. Physicochemical characteristics and antioxidant activity of *Prunus cerasoides* D. Don gum exudates. International journal of biological macromolecules. 2014 Aug 1;69:192-9.

Mamache W, Amira S, Ben Souici C, Laouer H, Benchikh F. In vitro antioxidant, anticholinesterases, anti-α-amylase, and anti-α-glucosidase effects of Algerian *Salvia aegyptiaca* and *Salvia verbenaca*. Journal of Food Biochemistry. 2020 Nov;44(11):e13472. https://doi.org/10.1111/jfbc.13472

Mamba P, Adebayo SA, Tshikalange TE. Anti-microbial, anti-inflammatory and HIV-1 reverse transcriptase activity of selected South African plants used to treat sexually transmitted diseases. *International Journal of Pharmacognosy and Phytochemical Research* 2016; 8(11); 1870-1876.

Mamidala E, Gujjeti RP. Phytochemical and antimicrobial activity of *Acmella paniculata* plant extracts. J. Bio Innov. 2013;1:17-22.

Mamyrbekova-Bekro JA, Boua BB, Kouassi KC, Békro YA. Qualitative and pharmacological analysis of 2 antihypertensive plants used in N'gramanssabo in Côte d'Ivoire. Nat. & Tech. 2013;8(11).

Mancuso R, Del Valle J, Modol L, Martinez A, Granado-Serrano AB, Ramirez-Núñez O, Pallás M, Portero-Otin M, Osta R, Navarro X. Resveratrol improves motoneuron function and extends survival in SOD1 G93A ALS mice. Neurotherapeutics. 2014 Apr 1;11(2):419-32. doi: 10.1007/s13311-013-0253-y

Manda P, Abrogoua DP, Bahi C, Dano DS, Gnahoui G, Kablan J.. Evaluation of the antihypertensive activity of total aqueous extract of *Justicia secunda* Valh (Acanthaceae). African Journal of Pharmacy and Pharmacology. 2011 Oct 29;5(16):1838-45.

Mandal A, Ojha D, Lalee A, Kaity S, Das M, Chattopadhyay D, Samanta A. Bioassay directed isolation of a novel anti-inflammatory cerebroside from the leaves *of Aerva sanguinolenta*. Medicinal Chemistry Research. 2015 May 1;24(5):1952-63. https://doi.org/10.1007/s00044-014-1261-0

Manga FN, El Khattabi C, Fontaine J, Berkenboom G, Duez P, Noyon C, Van Antwerpen P, Nzunzu JL, Pochet S. Vasorelaxant and antihypertensive effects of methanolic extracts from *Hymenocardia acida* Tul. Journal of ethnopharmacology. 2013 Mar 27;146(2):623-31. https://doi.org/10.1016/j.jep.2013.02.002

Mann A, Salawu FB, Abdulrauf I. Antimicrobial activity of *Bombax buonopozense* P. Beauv.(Bombacaceae) edible floral extracts. Eur. J. Sci. Res. 2011;48(4):627-30.

Mansourabadi AH, Sadeghi HM, Razavi N, Rezvani E. Anti-inflammatory and analgesic properties of salvigenin, *Salvia officinalis* flavonoid extracted. Advanced Herbal Medicine. 2016 Feb 1;2(1):31-41.

Mansouri S, Foroumadi A, Ghaneie T, Najar AG. Antibacterial activity of the crude extracts and fractionated constituents of *Myrtus communis*. Pharmaceutical biology. 2001 Jan 1;39(5):399-401. doi.org/10.1076/phbi.39.5.399.5889

Mantena VK, Tejaswini G. Anti inflammatory activity of *Erythrina variegata*. International Journal of Pharmacy and Pharmaceutical Sciences. 2015:386-8.

Manzano PI, Miranda M, Abreu-Payrol J, Silva M, Sterner O, Peralta EL. Pentacyclic triterpenoids with antimicrobial activity from the leaves of *Vernonanthura patens* (Asteraceae). Emirates Journal of Food and Agriculture. 2013 May 1:539-43. https://doi.org/10.9755/ejfa.v25i7.

Manzoor MF, Ahmad N, Manzoor A, Kalsoom A. Food based phytochemical luteolin their derivatives, sources and medicinal benefits. Int. J. Agric. Life Sci. IJAL. 2017;3(11):1.

Mao JJ, Xie SX, Keefe JR, Soeller I, Li QS, Amsterdam JD. Long-term chamomile (*Matricaria chamomilla* L.) treatment for generalized anxiety disorder: A randomized clinical trial. Phytomedicine. 2016 Dec 15;23(14):1735-1742. doi: 10.1016/j.phymed.2016.10.012. Epub 2016 Oct 24.

Marcial G, Sendker J, Brandt S, de Lampasona MP, Catalán CA, de Valdez GF, Hensel A. Gastroprotection as an example: Antiadhesion against Helicobacter pylori, anti-inflammatory and antioxidant activities of aqueous extracts from the aerial parts of *Lippia integrifolia* Hieron. Journal of ethnopharmacology. 2014 Sep 11;155(2):1125-33.

Marinho DG, Alviano DS, Matheus ME, Alviano CS, Fernandes PD. The latex obtained from *Hancornia speciosa* Gomes possesses anti-inflammatory activity. Journal of Ethnopharmacology. 2011 May 17;135(2):530-7. https://doi.org/10.1016/j.jep.2011.03.059

Marjana MP, Remyakrishnan CR, Baiju EC. Ethnomedicinal flowering plants used by Kurumas, Kurichiyas and Paniyas tribes of Wayanad district of Kerala, India. International Journal of Biology Research. 2018;3:01-8.

Marković T, Chatzopoulou P, Šiljegović J, Nikolić M, Glamočlija J, Ćirić A, Soković M. Chemical analysis and antimicrobial activities of the essential oils of *Satureja thymbra* L. and *Thymbra spicata* L. and their main components. Archives of Biological Sciences. 2011;63(2):457-64.

Maroyi A. An ethnobotanical survey of medicinal plants used by the people in Nhema communal area, Zimbabwe. Journal of ethnopharmacology. 2011 Jun 22;136(2):347-54. https://doi.org/10.1016/j.jep.2011.05.003.

Marrero E, Sanchez J, de Armas E, Escobar A, Melchor G, Abad MJ, Bermejo P, Villar AM, Megías J, Alcaraz MJ. COX-2 and sPLA2 inhibitory activity of aqueous extract and polyphenols of *Rhizophora mangle* (red mangrove). Fitoterapia. 2006 Jun 1;77(4):313-5.

Martinello F, Soares SM, Franco JJ, Santos AC, Sugohara A, Garcia SB, Curti C, Uyemura SA. Hypolipemic and antioxidant activities from *Tamarindus indica* L. pulp fruit extract in hypercholesterolemic hamsters. Food Chem Toxicol. 2006 Jun;44(6):810-8. doi: 10.1016/j.fct.2005.10.011.

Martinez MJ, Betancourt J, Alonso-Gonzalez N, Jauregui A. Screening of some Cuban medicinal plants for antimicrobial activity. Journal of ethnopharmacology. 1996 Jul 5;52(3):171-4. https://doi.org/10.1016/0378-8741(96)01405-5

Martínez R, Kapravelou G, Porres JM, Melesio AM, Heras L, Cantarero S, Gribble FM, Parker H, Aranda P, López-Jurado M. *Medicago sativa* L., a functional food to relieve hypertension and metabolic disorders in a spontaneously hypertensive rat model. Journal of Functional Foods. 2016 Oct 1;26:470-84. https://doi.org/10.1016/j.jff.2016.08.013

Martins AP, Salgueiro LR, Goncalves MJ, Da Cunha AP, Vila R, Canigueral S. Essential oil composition and antimicrobial activity of *Santiria trimera* bark. Planta medica. 2003 Jan;69(01):77-9. doi:  10.1055/s-2003-37025

Martins FO, da Rocha Gomes MM, Pereira FL, Nogueira GR, Romanos MT, Coelho MA. In vitro inibitory effect of *Urera baccifera* (L.) Gaudich. extracts against herpes simplex. Afr J Pharm Pharmacol. 2009 Nov 1;1(11).

Maryniuk M, Tkachenko H, Buyun L, Kurhaluk N, Góralczyk A, Tomin W, Osadowski Z. In Vitro Antibacterial Activity of Ethanolic Extract Derived from Leaves of *Sansevieria aethiopica* Thunb.(Asparagaceae). Agrobiodiversity for improving nutrition, health and life quality. 2019 Dec 3(3). https://doi.org/10.15414/agrobiodiversity.2019.2585-8246.165-177

Marzouk B, Marzouk Z, Haloui E, Fenina N, Bouraoui A, Aouni M. Screening of analgesic and anti-inflammatory activities of *Citrullus colocynthis* from southern Tunisia. Journal of ethnopharmacology. 2010 Mar 2;128(1):15-9. doi.org/10.1016/j.jep.2009.11.027

Marzouk MS, Moharram FA, El Dib RA, El-Hossary DG. Novel macrocyclic monoterpene glycosides from bioactive extract of *Parkinsonia aculeata* L. Cell biochemistry and biophysics. 2013 Apr 1;65(3):301-13.

Masalu R, Ngassa S, Kinunda G, Mpinda C. Antibacterial and Anti-HIV-1 Reverse Transcriptase Activities of Selected Medicinal Plants and Their Synthesized Zinc Oxide Nanoparticles. Tanzania Journal of Science. 2020 Oct 30;46(3):597-612.

Mascolo N, Autore G, Capasso F, Menghini A, Fasulo MP. Biological screening of Italian medicinal plants for anti-inflammatory activity. Phytotherapy research. 1987 Mar;1(1):28-31. https://doi.org/10.1002/ptr.2650010107

Masoko P. Ethnobotanical study of some selected medicinal plants used by traditional healers in Limpopo Province (South Africa). Am J Res Commun. 2013;1:8-23.

Masola SN, Mosha RD, Wambura PN. Assessment of antimicrobial activity of crude extracts of stem and root barks from *Adansonia digitata* (Bombacaceae)(African baobab). African Journal of Biotechnology. 2009;8(19).

Matasyoh LG, Murigi HM, Matasyoh JC. Antimicrobial assay and phyto-chemical analysis of *Solanum nigrum* complex growing in Kenya. African Journal of Microbiology Research. 2014 Dec 10;8(50):3923-30.

Mathabe MC, Nikolova RV, Lall N, Nyazema NZ. Antibacterial activities of medicinal plants used for the treatment of diarrhoea in Limpopo Province, South Africa. Journal of ethnopharmacology. 2006 Apr 21;105(1-2):286-93.

Mathivanan N, Surendiran G, Srinivasan K, Malarvizhi K. *Morinda pubescens* JE Smith (*Morinda tinctoria* Roxb.) fruit extract accelerates wound healing in rats. Journal of medicinal food. 2006 Dec 1;9(4):591-3. https://doi.org/10.1089/jmf.2006.9.591

Matsuda H, Li Y, Murakami T, NINOMIYA K, YAMAHARA J, YOSHIKAWA M. Effects of escins Ia, Ib, IIa, and IIb from horse chestnut, the seeds of *Aesculus hippocastanum* L., on acute inflammation in animals. Biological and Pharmaceutical Bulletin. 1997 Oct 15;20(10):1092-5.

Matsui T, Korematsu S, Byun EB, Nishizuka T, Ohshima S, Kanda T. Apple procyanidins induced vascular relaxation in isolated rat aorta through NO/cGMP pathway in combination with hyperpolarization by multiple K+ channel activations. Bioscience, biotechnology, and biochemistry. 2009 Oct 23;73(10):2246-51. https://doi.org/10.1271/bbb.90334

Matsuse IT, Lim YA, Hattori M, Correa M, Gupta MP. A search for anti-viral properties in Panamanian medicinal plants.: The effects on HIV and its essential enzymes. Journal of ethnopharmacology. 1998 Jan 1;64(1):15-22.

Matta MK, Paltatzidou K, Triantafyllidou H, Lazari DM, Karioti A, Skaltsa H et al. Abstracts of the 55th International Congress and Annual Meeting of the Society for Medicinal Plant Research, September 2-6, 2007, Graz, Austria. Planta Med. 2007 Aug;73(9): P_529.

Mattana CM, Satorres SE, Virginia JU, Cifuente D, Carlos TO, Laciar AL. Antibacterial activity study of single and combined extracts of *Berberis ruscifolia*, *Baccharis sagittalis*, *Euphorbia dentata and Euphorbia schikendanzii*, native plants from Argentina. Boletín Latinoamericano y del Caribe de Plantas Medicinales y Aromáticas. 2012;11(5):428-34.

Matu EN, Van Staden J. Antibacterial and anti-inflammatory activities of some plants used for medicinal purposes in Kenya. Journal of Ethnopharmacology. 2003 Jul 1;87(1):35-41. https://doi.org/10.1016/S0378-8741(03)00107-7

Matulevich Peláez JA, Gil Archila E, Ospina Giraldo LF. Estudio fitoquímico y actividad antiinflamatoria de hojas, flores y frutos de *Bejaria resinosa* Mutis ex L.(Pegamosco). Revista Cubana de Plantas Medicinales. 2016 Sep;21(3):332-45.

Maulik M, Mitra S, Hunter S, Hunstiger M, Oliver SR, Bult-Ito A, Taylor BE. Sir-2.1 mediated attenuation of α-synuclein expression by Alaskan bog blueberry polyphenols in a transgenic model of Caenorhabditis elegans. Scientific reports. 2018 Jul 5;8(1):10216.

Maynard LG, Santos KC, Cunha PS, et al. Chemical composition and vasorelaxant effect induced by the essential oil of *Lippia alba* (Mill.) N.E. Brown. (Verbenaceae) in rat mesenteric artery. *Indian J Pharmacol*. 2011;43(6):694–698. doi:10.4103/0253-7613.89828

Maynart G, Pousset JL, Mboup S, Denis F. Antibacterial effect of borreverine, an alkaloid isolated from *Borreria verticillata* (Rubiaceae). Comptes Rendus des Seances de la Societe de Biologie et de ses Filiales. 1980;174(5):925-8.

Mazimba O, Majinda RR, Motlhanka D Antioxidant and antibacterial constituents from *Morus nigra*. African Journal of Pharmacy and Pharmacology 2011 June 5(6): 751-754. doi: 10.5897/AJPP11.260

Mazutti da Silva S, Rezende Costa C, Martins Gelfuso G, Silva Guerra E, de Medeiros Nóbrega Y, Gomes S, Pic-Taylor A, Fonseca-Bazzo Y, Silveira D, Magalhães P. Wound Healing Effect of Essential Oil Extracted from *Eugenia dysenterica* DC (Myrtaceae) Leaves. Molecules. 2019 Jan;24(1):2. https://doi.org/10.3390/molecules24010002

Mazzio E, Georges B, McTier O, Soliman KFA. Neurotrophic Effects of *Mu Bie Zi* (*Momordica cochinchinensis*) Seed Elucidated by High-Throughput Screening of Natural Products for NGF Mimetic Effects in PC-12 Cells. Neurochemical research. 2015;40(10):2102-2112. doi:10.1007/s11064-015-1560-y.

Mazzolin LP, de Almeida Kiguti LR, da Maia EO, Fernandes LT, da Rocha LR, Vilegas W, Pupo AS, Di Stasi LC, Hiruma-Lima CA. Antidiarrheal and intestinal antiinflammatory activities of a methanolic extract of *Qualea parviflora* Mart. in experimental models. Journal of ethnopharmacology. 2013 Dec 12;150(3):1016-23. https://doi.org/10.1016/j.jep.2013.10.006

Mazzolin LP, Nasser AL, Moraes TM, Santos RC, Nishijima CM, Santos FV, Varanda EA, Bauab TM, da Rocha LR, Di Stasi LC, Vilegas W. Qualea parviflora Mart.: an integrative study to validate the gastroprotective, antidiarrheal, antihemorragic and mutagenic action. Journal of ethnopharmacology. 2010 Feb 3;127(2):508-14. https://doi.org/10.1016/j.jep.2009.10.005

Mbiantcha M, Almas J, Shabana SU, Nida D, Aisha F. Anti-arthritic property of crude extracts of *Piptadeniastrum africanum* (Mimosaceae) in complete Freund's adjuvant-induced arthritis in rats. BMC complementary and alternative medicine. 2017 Dec;17(1):111. https://doi.org/10.1186/s12906-017-1623-5

Mbosso EJ, Ngouela S, Nguedia JC, Beng VP, Rohmer M, Tsamo E. In vitro antimicrobial activity of extracts and compounds of some selected medicinal plants from Cameroon. Journal of ethnopharmacology. 2010 Mar 24;128(2):476-81. https://doi.org/10.1016/j.jep.2010.01.017

Mbula JP, Kwembe JT, Tshilanda DD, Asimonio J, Toegaho A, Tshibangu DS, Ngbolua KN, Bokota MT, Mpiana PT. Ethnobotanical Survey of Aromatic Plants of Masako Forest Reserve (Kisangani, DR Congo). J. of Advanced Botany and Zoology, V2I3. DOI: 10.15297/JABZ.V2I3.04.

McCutcheon AR, Ellis SM, Hancock RE, Towers GH. Antibiotic screening of medicinal plants of the British Columbian native peoples. Journal of Ethnopharmacology. 1992 Oct 1;37(3):213-23. doi.org/10.1016/0378-8741(92)90036-Q

McCutcheon AR, Ellis SM, Hancock RE, Towers GH. Antifungal screening of medicinal plants of British Columbian native peoples. Journal of ethnopharmacology. 1994 Dec 1;44(3):157-69. doi.org/10.1016/0378-8741(94)01183-4

McCutcheon AR, Roberts TE, Gibbons E, Ellis SM, Babiuk LA, Hancock RE, Towers GH. Antiviral screening of British Columbian medicinal plants. Journal of Ethnopharmacology. 1995 Dec 1;49(2):101-10. https://doi.org/10.1016/0378-8741(95)90037-3

McFeeters H, Gilbert MJ, Thompson RM, Setzer WN, Cruz-Vera LR, McFeeters RL. Inhibition of essential bacterial peptidyl-tRNA hydrolase activity by tropical plant extracts. Natural product communications. 2012 Aug;7(8):1934578X1200700836. https://doi.org/10.1177/1934578X1200700836

McLaurin J, Kierstead ME, Brown ME, Hawkes CA, Lambermon MH, Phinney AL, et al. Cyclohexanehexol inhibitors of amyloid β aggregation prevent and reverse Alzheimer phenotype in a mouse model. Nat Med. 2006;12(7):801–808.

Mediesse FK, Boudjeko T, Hasitha A, Gangadhar M, Mbacham WF, Yogeeswari P. Inhibition of lipopolysaccharide (LPS)-induced neuroinflammatory response by polysaccharide fractions of *Khaya grandifoliola* (C.D.C.) stem bark, Cryptolepis sanguinolenta (Lindl.) Schltr and *Cymbopogon citratus* Stapf leaves in raw 264.7 macrophages and U87 glioblastoma cells. *BMC Complement Altern Med*. 2018;18(1):86. Published 2018 Mar 12. doi:10.1186/s12906-018-2156-2

Meenatchisundaram S, Priyagrace S, Vijayaraghavan R, Velmurugan A, Parameswari G, Michael A. Antitoxin activity of *Mimosa pudica* root extracts against *Naja naja* and *Bangarus caerulus* venoms. Bangladesh Journal of Pharmacology. 2009 May 3;4(2):105-9.

Meenatchisundaram S. Antitoxin Activity of Selected Indigenous Medicinal Plants with Inhibitory Properties against Russell's viper and Saw scaled viper venoms in Coimbatore District. University Grants Commission, Bahadur Shah Zafar Marg. New Delhi. 2007: 110 002.

Meeran MF, Laham F, Azimullah S, Tariq S, Ojha S. α-Bisabolol abrogates isoproterenol-induced myocardial infarction by inhibiting mitochondrial dysfunction and intrinsic pathway of apoptosis in rats. Molecular and cellular biochemistry. 2019 Mar;453(1):89-102. https://doi.org/10.1007/s11010-018-3434-5

Megías C, Yust MD, Pedroche J, Lquari H, Girón-Calle J, Alaiz M, Millán F, Vioque J. Purification of an ACE inhibitory peptide after hydrolysis of sunflower (*Helianthus annuus* L.) protein isolates. Journal of Agricultural and Food Chemistry. 2004 Apr 7;52(7):1928-32. doi.org/10.1021/jf034707r

Meguellati H, Ouafi S, Saad S, Djemouai N. Evaluation of acute, subacute oral toxicity and wound healing activity of mother plant and callus of *Teucrium polium* L. subsp. *geyrii* Maire from Algeria. South African Journal of Botany. 2019 Dec 1;127:25-34. https://doi.org/10.1016/j.sajb.2019.08.023

Mehrzadi S, Sadr S, Hosseinzadeh A, Gholamine B, Shahbazi A, FallahHuseini H, Ghaznavi H. Anticonvulsant activity of the ethanolic extract of *Punica granatum* L. seed. Neurological research. 2015 Jun 1;37(6):470-5. https://doi.org/10.1179/1743132814Y.0000000460

Mekhfi H, El Haouari M, Legssyer A, Bnouham M, Aziz M, Atmani F, Remmal A, Ziyyat A. Platelet anti-aggregant property of some Moroccan medicinal plants. Journal of ethnopharmacology. 2004 Oct 1;94(2-3):317-22. https://doi.org/10.1016/j.jep.2004.06.005

Mekonnen A, Sidamo T, Asres K, Engidawork E. In vivo wound healing activity and phytochemical screening of the crude extract and various fractions of *Kalanchoe petitiana* A. Rich (Crassulaceae) leaves in mice. Journal of ethnopharmacology. 2013 Jan 30;145(2):638-46. https://doi.org/10.1016/j.jep.2012.12.002

Melchor G, Armenteros M, Fernández O, Linares E, Fragas I. Antibacterial activity of *Rhizophora mangle* bark. Fitoterapia. 2001 Aug 1;72(6):689-91. https://doi.org/10.1016/S0367-326X(01)00294-5

Melis MS. A crude extract of *Stevia rebaudiana* increases the renal plasma flow of normal and hypertensive rats. Brazilian journal of medical and biological research= Revista brasileira de pesquisas medicas e biologicas. 1996 May;29(5):669-75.

Mello CD, Valente LM, Wolff T, Lima-Junior RS, Fialho LG, Marinho CF, Azeredo EL, Oliveira-Pinto LM, Pereira RD, Siani AC, Kubelka CF. Decrease in Dengue virus-2 infection and reduction of cytokine/chemokine production by *Uncaria guianensis* in human hepatocyte cell line Huh-7. Memórias do Instituto Oswaldo Cruz. 2017 Jun;112(6):458-68. https://doi.org/10.1590/0074-02760160323

Mencherini T, Cau A, Bianco G, Loggia RD, Aquino RP, Autore G. An extract of *Apium graveolens* var. dulce leaves: Structure of the major constituent, apiin, and its anti-inflammatory properties. Journal of pharmacy and pharmacology. 2007 Jun;59(6):891-7. https://doi.org/10.1211/jpp.59.6.0016

Mendes FR, Negri G, Duarte-Almeida JM, Tabach R, Carlini EA. The action of plants and their constituents on the central nervous system. Plant bioactives and drug discovery: principles, practice, and perspectives. 2012;17:161.

Mendez AH, Cornejo CG, Coral MF, Arnedo M. Chemical composition, antimicrobial and antioxidant activities of the essential oil of *Bursera graveolens* (Burseraceae) from Peru. Indian J Pharm Educ Res. 2017;51(3):S429-35.

Meneses R, Torres FÁ, Stashenko E, Ocazionez RE. Aceites esenciales de plantas colombianas inactivan el virus del dengue y el virus de la fiebre amarilla. Revista de la Universidad Industrial de Santander. Salud. 2009;41(3):236-43.

Meng P, Zhu Q, Yang H, et al. Leonurine promotes neurite outgrowth and neurotrophic activity by modulating the GR/SGK1 signaling pathway in cultured PC12 cells. *Neuroreport*. 2019;30(4):247–254. doi:10.1097/WNR.0000000000001180 AMA

Menghini L, Massarelli P, Bruni G, Pagiotti R. Anti-inflammatory and analgesic effects of *Spartium junceum* L. flower extracts: a preliminary study. Journal of medicinal food. 2006 Sep 1;9(3):386-90. https://doi.org/10.1089/jmf.2006.9.386

Mengoni F, Lichtner M, Battinelli L, Marzi M, Mastroianni CM, Vullo V, Mazzanti G. In vitro anti-HIV activity of oleanolic acid on infected human mononuclear cells. Planta medica. 2002 Feb;68(02):111-4.

Mensah AY, Houghton PJ, Dickson RA, Fleischer TC, Heinrich M, Bremner P. In vitro evaluation of effects of two Ghanaian plants relevant to wound healing. Phytotherapy Research: An International Journal Devoted to Pharmacological and Toxicological Evaluation of Natural Product Derivatives. 2006 Nov;20(11):941-4. doi.org/10.1002/ptr.1978

Mensah AY, Mireku EA, Oppong-Damoah A, Amponsah IK. Anti-inflammatory and antioxidant activities of *Commelina diffusa* (Commelinaceae). World Journal of Pharmaceutical Sciences, 2014; 2(10): 1159-1165.

Mesripour A, Rafieian-Kopaei M, Bahrami B. The effects of *Anethum graveolens* essence on scopolamine-induced memory impairment in mice. *Res Pharm Sci*. 2016;11(2):145-151.

Metowogo K, Agbonon A, Eklu-Gadegbeku K, Aklikokou AK, Gbeassor M. Anti-ulcer and anti-inflammatory effects of hydroalcohol extract of *Aloe buettneri* A. Berger (Lilliaceae). Tropical journal of pharmaceutical research. 2008;7(1):907-12.

Metrouh-Amir H, Amir N. Evaluation in vivo of anti-inflammatory and analgesic properties of *Matricaria pubescens* alkaloids. South African Journal of Botany. 2018 May 1;116:168-74. https://doi.org/10.1016/j.sajb.2018.03.008

Metrouh-Amir H, Duarte CM, Maiza F. Solvent effect on total phenolic contents, antioxidant, and antibacterial activities of *Matricaria pubescens*. Industrial Crops and Products. 2015 May 1;67:249-56. https://doi.org/10.1016/j.indcrop.2015.01.049

Metwally AM, Omar AA, Harraz FM, El Sohafy SM. Phytochemical investigation and antimicrobial activity of *Psidium guajava* L. leaves. Pharmacogn Mag. 2010 Jul;6(23):212-8. doi: 10.4103/0973-1296.66939.

Michel T, Kerdudo A, Ellong EN, Gonnot V, Rocchi S, Tanti JF, Boyer L, Adenet S, Rochefort K, Fernandez X. Anti-inflammatory, cytotoxic and antimicrobial activities of *Piper peltatum* leaf extract. Planta medica. 2016 Dec;82(S 01):P487. doi: 10.1055/s-0036-1596571

Mikawlrawng K, Kaushik S, Pushker A, Kumar S, Kameshwor M, Sharma G. Comparative in vitro antifungal activities of *Simarouba glauca* against *Fusarium oxysporum* and *Aspergillus parasiticus*. J. Med. Plant Studies. 2014;2:1-7.

Mikawlrawng K, Rani R, Kumar S, Bhardwaj AR, Prakash G. Anti-paralytic medicinal plants - Review. J Tradit Complement Med. 2017 Mar 9;8(1):4-10. doi: 10.1016/j.jtcme.2017.02.001.

Miller MG, Hamilton DA, Joseph JA, Shukitt-Hale B. Dietary blueberry improves cognition among older adults in a randomized, double-blind, placebo-controlled trial. European journal of nutrition. 2018 Apr 1;57(3):1169-80. https://doi.org/10.1007/s00394-017-1400-8

Min BS, Kim YH, Tomiyama M, Nakamura N, Miyashiro H, Otake T, Hattori M. Inhibitory effects of Korean plants on HIV-1 activities. Phytotherapy research. 2001 Sep;15(6):481-6. https://doi.org/10.1002/ptr.751

Min HJ, Nam JW, Yu ES, Hong JH, Seo EK, Hwang ES. Effect of naturally occurring hydroxychavicol acetate on the cytokine production in T helper cells. International immunopharmacology. 2009 Apr 1;9(4):448-54. https://doi.org/10.1016/j.intimp.2009.01.008

Minaei MB, Ghadami Yazdi E, Ebrahim Zadeh Ardakani M, et al. First Case Report: Treatment of the Facial Warts by Using *Myrtus communis* L. Topically on the Other Part of the Body. Iran Red Crescent Med J. 2014;16(2):e13565. doi:10.5812/ircmj.13565

Mirbehbahani FS, Hejazi F, Najmoddin N, Asefnejad A. *Artemisia annua* L. as a promising medicinal plant for powerful wound healing applications. Prog Biomater. 2020 Sep;9(3):139-151. doi: 10.1007/s40204-020-00138-z. Epub 2020 Sep 28.

Mirza FJ, Amber S, Hassan D, Ahmed T, Zahid S. Rosmarinic acid and ursolic acid alleviate deficits in cognition, synaptic regulation and adult hippocampal neurogenesis in an Aβ1-42-induced mouse model of Alzheimer's disease. Phytomedicine. 2021 Mar 1;83:153490. https://doi.org/10.1016/j.phymed.2021.153490

Mishra D, Bisht G, Mazumdar PM, Sah SP. Chemical composition and analgesic activity of *Senecio rufinervis* essential oil. Pharmaceutical biology. 2010 Nov 1;48(11):1297-301.  https://doi.org/10.3109/13880209.2010.491083

Mishra P, Jamdar P, Desai S, Patel D, Meshram D. Phytochemical analysis and assessment of in vitro antibacterial activity of *Tinospora cordifolia*. International Journal of Current Microbiology and Applied Sciences. 2014;3(3):224-34. https://doi.org/10.1155/2013/292934

Mishra S, Mishra A, Thakur M, Sharma A, Alok S. Investigations on hypolipidemic activity of *Asparagus filicinus* Buch-Ham ex D. Don. International Journal of Pharmaceutical Sciences and Research. 2017 Feb 1;8(2):813-8.

Mitić ZS, Jovanović B, Jovanović SČ, Mihajilov-Krstev T, Stojanović-Radić ZZ, Cvetković VJ, Mitrović TL, Marin PD, Zlatković BK, Stojanović GS. Comparative study of the essential oils of four *Pinus* species: Chemical composition, antimicrobial and insect larvicidal activity. Industrial crops and products. 2018 Jan 1;111:55-62. https://doi.org/10.1016/j.indcrop.2017.10.004

Mittal S, Dixit PK. In-vivo anti-inflammatory and anti-arthritic activity of *Asparagus racemosus* roots. International Journal of Pharmaceutical Sciences and Research. 2013 Jul 1;4(7):2652.

Miyake Y, Kuzuya K, Ueno C, Katayama N, Hayakawa T, Tsuge H, Osawa T. Suppressive effect of components in lemon juice on blood pressure in spontaneously hypertensive rats. Food Science and Technology International, Tokyo. 1998 Feb 25;4(1):29-32. https://doi.org/10.3136/fsti9596t9798.4.29

Miyamoto N, Izumi H, Miyamoto R, Kondo H, Tawara A, Sasaguri Y, Kohno K. Quercetin induces the expression of peroxiredoxins 3 and 5 via the Nrf2/NRF1 transcription pathway. Investigative ophthalmology & visual science. 2011 Feb 1;52(2):1055-63. doi.org/10.1167/iovs.10-5777

Miyamoto N, Kohno K. Quercetin and Glaucoma. In Handbook of Nutrition, Diet, and the Eye 2019 Jan 1 (pp. 189-202). Academic Press.https://doi.org/10.1016/B978-0-12-815245-4.00011-9

Mkaddem M, Bouajila J, Ennajar M, Lebrihi A, Mathieu F, Romdhane M. Chemical composition and antimicrobial and antioxidant activities of *Mentha* (*longifolia* L. and *viridis*) essential oils. Journal of food science. 2009 Sep;74(7):M358-63. doi: 10.1111/j.1750-3841.2009.01272.x

Mnayer D, Fabiano-Tixier AS, Petitcolas E, Hamieh T, Nehme N, Ferrant C, Fernandez X, Chemat F. Chemical composition, antibacterial and antioxidant activities of six essentials oils from the Alliaceae family. Molecules. 2014;19(12):20034-53.

Moacă EA, Pavel IZ, Danciu C, et al. Romanian Wormwood (*Artemisia absinthium* L.): Physicochemical and Nutraceutical Screening. *Molecules*. 2019;24(17):3087. Published 2019 Aug 25. doi:10.3390/molecules24173087

Moghadamtousi SZ, Rouhollahi E, Hajrezaie M, Karimian H, Abdulla MA, Kadir HA. Annona muricata leaves accelerate wound healing in rats via involvement of Hsp70 and antioxidant defence. International Journal of Surgery. 2015 Jun 1;18:110-7.

Moghadamtousi SZ, Rouhollahi E, Karimian H, Fadaeinasab M, Abdulla MA, Kadir HA. Gastroprotective activity of *Annona muricata* leaves against ethanol-induced gastric injury in rats via Hsp70/Bax involvement. *Drug Des Devel Ther*. 2014;8:2099–2110. Published 2014 Oct 28. doi:10.2147/DDDT.S70096

Mohabatkar H, Behbahani M, Nejad MR. Effects of root, shoot, leaf and seed extracts of seven *Artemisia* species on HIV-1 replication and CD4 expression. Journal of Coastal Life Medicine. 2015;3(12):996-9.

Mohamad S, Zin NM, Wahab HA, Ibrahim P, Sulaiman SF, Zahariluddin AS, Noor SS. Antituberculosis potential of some ethnobotanically selected Malaysian plants. Journal of ethnopharmacology. 2011 Feb 16;133(3):1021-6.

Mohamed AA, Ali SI, El-Baz FK. Antioxidant and antibacterial activities of crude extracts and essential oils of *Syzygium cumini* leaves. Plos one. 2013 Apr 12;8(4):e60269. https://doi.org/10.1371/journal.pone.0060269

Mohamed AH, Osman AA. Antibacterial and wound healing potential of ethanolic extract of *Zingiber Officinale* in albino rats. Journal of Diseases and Medicinal Plants. 2017 Jan 24;3(1):1-6. doi: 10.11648/j.jdmp.20170301.11

Mohamed NH, Mahrous AE. Chemical constituents of *Descurainia sophia* L. and its biological activity. Records of natural products. 2009 Jan 1;3(1).

Mohamed S, Saka S, El-Sharkawy SH, Ali AM, Muid S. Antimycotic screening of 58 Malaysian plants against plant pathogens. Pesticide science. 1996 Jul;47(3):259-64.https://doi.org/10.1002/(SICI)1096-9063(199607)47:3<259::AID-PS413>3.0.CO;2-N

Mohammed MH, Hamed AN, Khalil HE, Kamel MS. Phytochemical and pharmacological studies of *Citharexylum quadrangulare* Jacq. leaves. Journal of Medicinal Plants Research. 2016 May 10;10(18):232-41. doi: 10.5897/JMPR2016.6053

Mohan CG, Gokavi S, Viswanatha GL, Shylaja H, Nandakumar K. Analgesic and antiinflammatory activity of leaf extracts of *Commiphora caudata* in Rodents. Pharmacologyonline. 2009;2:991-8.

Mohanasundari C, Natarajan D, Srinivasan K, Umamaheswari S, Ramachandran A. Antibacterial properties of *Passiflora foetida* L.–a common exotic medicinal plant. African Journal of Biotechnology. 2007;6(23).

Mohanty S, K Maurya A, Saxena A, Shanker K, Pal A, U Bawankule D. Flavonoids rich fraction of *Citrus limetta* fruit peels reduces proinflammatory cytokine production and attenuates malaria pathogenesis. Current pharmaceutical biotechnology. 2015 Jun 1;16(6):544-52.

Moharram BA, Al-Mahbashi HM, Saif-Ali RI, AliAqlan F. Phytochemical, anti-inflammatory, antioxidant, cytotoxic and antibacterial study of *Capparis cartilaginea* Decne from yemen. Int J Pharm Pharm Sci. 2018 Jun 1;10:38-4. http://dx.doi.org/10.22159/ijpps.2018v10i6.22905

Mojisola CO, Oladele AT, Elufioye TO. Ethnobotanical Survey of Plants Used as Memory Enhancer and Antiaging in Ondo State, Nigeria. International Journal of Pharmaceutics. 2012;2:26-32.

Mokni RE, Youssef FS, Jmii H, Khmiri A, Bouazzi S, Jlassi I, Jaidane H, Dhaouadi H, Ashour ML, Hammami S. The Essential oil of Tunisian *Dysphania ambrosioides* and its antimicrobial and antiviral properties. Journal of Essential Oil Bearing Plants. 2019 Jan 2;22(1):282-94. https://doi.org/10.1080/0972060X.2019.1588171

Molander M, Nielsen L, Søgaard S, Staerk D, Rønsted N, Diallo D, Chifundera KZ, van Staden J, Jäger AK. Hyaluronidase, phospholipase A2 and protease inhibitory activity of plants used in traditional treatment of snakebite-induced tissue necrosis in Mali, DR Congo and South Africa. Journal of ethnopharmacology. 2014 Nov 18;157:171-80. https://doi.org/10.1016/j.jep.2014.09.027

Moldovan B, Filip A, Clichici S, Suharoschi R, Bolfa P, David L. Antioxidant activity of Cornelian cherry (*Cornus mas* L.) fruits extract and the in vivo evaluation of its anti-inflammatory effects. Journal of Functional Foods. 2016 Oct 1;26:77-87. doi.org/10.1016/j.jff.2016.07.004

Mølgaard P, Holler JG, Asar B, Liberna I, Rosenbæk LB, Jebjerg CP, Jørgensen L, Lauritzen J, Guzman A, Adsersen A, Simonsen HT. Antimicrobial evaluation of Huilliche plant medicine used to treat wounds. Journal of ethnopharmacology. 2011 Oct 31;138(1):219-27.

Molina-Salinas GM, Ramos-Guerra MC, Vargas-Villarreal J, Mata-Cárdenas BD, Becerril-Montes P, Said-Fernández S. Bactericidal activity of organic extracts from *Flourensia cernua* DC against strains of Mycobacterium tuberculosis. Archives of Medical Research. 2006 Jan 1;37(1):45-9. https://doi.org/10.1016/j.arcmed.2005.04.010

Momin MA, Habib MR, Hasan MR, Nayeem J, Uddin N, Rana MS. Anti-inflammatory, antioxidant and cytotoxicity potential of ethanolic extract of two Bangladeshi bean *Lablab purpureus* (L.) sweet white and purple. International J of Pharmaceutical Sci and research. 2012 Mar 1;3(3):776-81.

Momtazi-Borojeni AA, Sadeghi-Aliabadi H, Rabbani M, Ghannadi A, Abdollahi E. Cognitive enhancing of pineapple extract and juice in scopolamine-induced amnesia in mice. Res Pharm Sci. 2017 Jun;12(3):257-264. doi: 10.4103/1735-5362.207198.

Mondal KC, Dey S, Pati BR. Antimicrobial activity of the leaf extracts of *Hyptis suaveolens* (L.) poit. Indian Journal of Pharmaceutical Sciences. 2007;69(4):568. doi: 10.4103/0250-474X.36946

Mondal S, Raja S, Prasad PN, Suresh P. Investigations of phytochemical, analgesic, anti-inflammatory and antipyretic effects of *Ixora pavetta* Andrews leaf. Journal of Nepal Pharmaceutical Association. 2014;27(1):20-7. https://doi.org/10.3126/jnpa.v27i1.12146

Monforte MT, Tzakou O, Nostro A, Zimbalatti V, Galati EM. Chemical composition and biological activities of *Calamintha officinalis* Moench essential oil. Journal of medicinal food. 2011 Mar 1;14(3):297-303. https://doi.org/10.1089/jmf.2009.0191

Montanari RM, Barbosa LC, Demuner AJ, Silva CJ, Carvalho LS, Andrade NJ. Chemical composition and antibacterial activity of essential oils from Verbenaceae species: Alternative sources of (E)-caryophyllene and germacrene-D. Química Nova. 2011 Sep;34(9):1550-5. https://doi.org/10.1590/S0100-40422011000900013

Montanher AB, Zucolotto SM, Schenkel EP, Fröde TS. Evidence of anti-inflammatory effects of *Passiflora edulis* in an inflammation model. Journal of ethnopharmacology. 2007 Jan 19;109(2):281-8. https://doi.org/10.1016/j.jep.2006.07.031

Montenegro I, Villegas AM, Zaror L, Martínez R, Werner E, Carrasco-Altamirano H, Fritis MC, Palma-Flemming H. Antimicrobial activity of ethyl acetate extract and essential oil from bark of *Laurelia sempervirens* against multiresistant bacteria. Boletín Latinoamericano y del Caribe de Plantas Medicinales y Aromáticas. 2012;11(4):306-15.

Monterrosas-Brisson N, Ocampo ML, Jiménez-Ferrer E, Jiménez-Aparicio AR, Zamilpa A, Gonzalez-Cortazar M, Tortoriello J, Herrera-Ruiz M. Anti-inflammatory activity of different *Agave* plants and the compound Cantalasaponin-1. Molecules. 2013 Jul;18(7):8136-46. https://doi.org/10.3390/molecules18078136

Monti MC, Margarucci L, Riccio R, Casapullo A. Modulation of tau protein fibrillization by oleocanthal. Journal of natural products. 2012 Sep 28;75(9):1584-8. https://doi.org/10.1021/np300384h

Monzio Compagnoni G, Di Fonzo A. Understanding the pathogenesis of multiple system atrophy: state of the art and future perspectives. Acta Neuropathol Commun. 2019;7(1):113. https://doi.org/10.1186/s40478-019-0730-6

Mook-Jung I, Shin JE, Yun SH, Huh K, Koh JY, Park HK, Jew SS, Jung MW. Protective effects of asiaticoside derivatives against beta-amyloid neurotoxicity. Journal of neuroscience research. 1999 Nov 1;58(3):417-25 https://doi.org/10.1002/(SICI)1097-4547(19991101)58:3<417::AID-JNR7>3.0.CO;2-G

Moon M, Kim HG, Choi JG, Oh H, Lee PK, Ha SK, Kim SY, Park Y, Huh Y, Oh MS. 6-Shogaol, an active constituent of ginger, attenuates neuroinflammation and cognitive deficits in animal models of dementia. Biochemical and biophysical research communications. 2014 Jun 20;449(1):8-13. doi: 10.1016/j.bbrc.2014.04.121.

Moosavy MH, Hassanzadeh P, Mohammadzadeh E, Mahmoudi R, Khatibi SA, Mardani K. Antioxidant and antimicrobial activities of essential oil of Lemon (*Citrus limon*) peel in vitro and in a food model. Journal of food quality and hazards control. 2017 Jun 10;4(2):42-8.

Moradi MT, Karimi A, Lorigooini Z, Pourgheysari B, Alidadi S, Hashemi L. In vitro anti influenza virus activity, antioxidant potential and total phenolic content of twelve Iranian medicinal plants. Marmara Pharmaceutical Journal. 2017 Jan 1;21(4):843-51. doi: 10.12991/mpj.2017.10

Moradkhani S, Salehi I, Abdolmaleki S, Komaki A. Effect of *Calendula officinalis* hydroalcoholic extract on passive avoidance learning and memory in streptozotocin-induced diabetic rats. Anc Sci Life. 2015;34(3):156–161. doi:10.4103/0257-7941.157160

Morah FN, Ashipu LB. Chemical composition and antimicrobial activity of essential oil from *Heinsia crinita* leaf. American Journal of Essential Oil and Natural Products. 2017;5(2):23-8.

Morakinyo AO, Adeniyi OS, Arikawe AP. Effects of *Zingiber officinale* on reproductive functions in the male rat. African Journal of biomedical research. 2008;11(3). doi: 10.4314/ajbr.v11i3.50750

Morena J, Gupta A, Hoyle JC. Charcot-Marie-Tooth: From Molecules to Therapy. Int J Mol Sci. 2019;20(14). https://doi.org/10.3390/ijms20143419

Moreno L, Bello R, Beltran B, Calatayud S, Primo-Yúfera E, Esplugues J. Pharmacological screening of different *Juniperus oxycedrus* L. extracts. Pharmacology & toxicology. 1998 Feb;82(2):108-12. https://doi.org/10.1111/j.1600-0773.1998.tb01407.x

Moreno-Ulloa A, Cid A, Rubio-Gayosso I, Ceballos G, Villarreal F, Ramirez-Sanchez I. Effects of (-)-epicatechin and derivatives on nitric oxide mediated induction of mitochondrial proteins. Bioorg Med Chem Lett. 2013;23(15):4441–4446. doi:10.1016/j.bmcl.2013.05.079

Mori K, Sakai H, Suzuki S, Akutsu Y, Ishikawa M, Imaizumi M, Tada K, Aihara M, Sawada Y, Yokoyama M, Sato Y. Effects of glycyrrhizin (SNMC: Stronger Neo-Minophagen C®) in hemophilia patients with HIV-1 infection. The Tohoku journal of experimental medicine. 1990;162(2):183-93. https://doi.org/10.1620/tjem.158.25

Morshedi D, Kesejini TS, Aliakbari F, Karami-Osboo R, Shakibaei M, Marvian AT, Khalifeh M, Soroosh M. Identification and characterization of a compound from *Cuminum cyminum* essential oil with antifibrilation and cytotoxic effect. *Res Pharm Sci*. 2014;9(6):431-443.

Mosaddegh M, Khoshnood MJ, Kamalinejad M, Alizadeh E. Study on the Effect of *Paliurus spina-christi* on cholesterol, triglyceride and HDL Levels in diabetic male rats fed a high cholesterol diet. Iranian Journal of Pharmaceutical Research. 2010 Nov 20(1):51-4. doi: 10.22037/IJPR.2010.297

Mosaddegh M, Naghibi F, Moazzeni H, Pirani A, Esmaeili S. Ethnobotanical survey of herbal remedies traditionally used in Kohghiluyeh va Boyer Ahmad province of Iran. J Ethnopharmacol. 2012 May 7;141(1):80-95. doi: 10.1016/j.jep.2012.02.004.

Moshi MJ, Innocent E, Otieno JN, Magadula JJ, Nondo RS, Otieno DF, Wensheit A, Mbabazi P. Antimicrobial and brine shrimp activity of *Acanthus pubescens* root extracts. Tanzania Journal of Health Research. 2010;12(2):155-8.

Moshi MJ, Otieno DF, Mbabazi PK, Weisheit A. The Ethnomedicine of the Haya people of Bugabo ward, Kagera Region, north western Tanzania. Journal of ethnobiology and ethnomedicine. 2009 Dec;5(1):24.

Motamed SM, Naghibi F. Antioxidant activity of some edible plants of the Turkmen Sahra region in northern Iran. Food Chemistry. 2010 Apr 15;119(4):1637-42.

Mothana RA, Gruenert R, Bednarski PJ, Lindequist U. Evaluation of the in vitro anticancer, antimicrobial and antioxidant activities of some Yemeni plants used in folk medicine. Die Pharmazie-An International Journal of Pharmaceutical Sciences. 2009 Apr 1;64(4):260-8.

Mothana, R.A., Mentel, R., Reiss, C. and Lindequist, U., 2006. Phytochemical screening and antiviral activity of some medicinal plants from the island Soqotra. Phytotherapy Research: An International Journal Devoted to Pharmacological and Toxicological Evaluation of Natural Product Derivatives, 20(4), pp.298-302.

Motti R, Motti P. An ethnobotanical survey of useful plants in the agro Nocerino Sarnese (Campania, southern Italy). Human Ecology. 2017 Dec 1;45(6):865-78. https://doi.org/10.1007/s10745-017-9946-x

Mouhajir F, Hudson JB, Rejdali M, Towers GH. Multiple antiviral activities of endemic medicinal plants used by Berber peoples of Morocco. Pharmaceutical biology. 2001 Jan 1;39(5):364-74. https://doi.org/10.1076/phbi.39.5.364.5892

Moussaid M, Elamrani AE, Bourhim N, Benaissa M. In vivo anti-inflammatory and in vitro antioxidant activities of Moroccan medicinal plants. Natural Product Communications. 2011 Oct;6(10):1934578X1100601007. https://doi.org/10.1177/1934578X1100601007

Moussi M, Filali H, Tazi A, Hakkou F. Ethnobotanical survey of healing medicinal plants traditionally used in the main Moroccan cities. Journal of Pharmacognosy and Phytotherapy. 2015 Aug 31;7(8):164-82. https://doi.org/10.5897/JPP2015.0355

Moutia M, Seghrouchni F, Abouelazz O, Elouaddari A, Al Jahid A, Elhou A, Nadifi S, Eddine JJ, Habti N, Badou A. *Allium sativum* L. regulates in vitro IL-17 gene expression in human peripheral blood mononuclear cells. BMC complementary and alternative medicine. 2016 Dec;16(1):1-0. https://doi.org/10.1186/s12906-016-1365-9

Mthethwa NS, Oyedeji BA, Obi LC, Aiyegoro OA. Anti-staphylococcal, anti-HIV and cytotoxicity studies of four South African medicinal plants and isolation of bioactive compounds from *Cassine transvaalensis* (Burtt. Davy) codd. BMC complementary and alternative medicine. 2014 Dec 1;14(1):512. https://doi.org/10.1186/1472-6882-14-512

Mu Y, Gage FH. Adult hippocampal neurogenesis and its role in Alzheimer's disease. Mol Neurodegener. 2011;6:85. https://doi.org/10.1186/1750-1326-6-85

Mucalo I, Jovanovski E, Rahelić D, Božikov V, Romić Ž, Vuksan V. Effect of American ginseng (*Panax quinquefolius* L.) on arterial stiffness in subjects with type-2 diabetes and concomitant hypertension. Journal of ethnopharmacology. 2013 Oct 28;150(1):148-53. doi: 10.1016/j.jep.2013.08.015.

Mufti FU, Ullah H, Bangash A, Khan N, Hussain S, Ullah F, Jamil M, Jabeen M. Antimicrobial activities of *Aerva javanica* and *Paeonia emodi* plants. Pak J Pharm Sci. 2012 Jul 1;25(3):565-9.

Muhammad N, Saeed M, Khan H. Antipyretic, analgesic and anti-inflammatory activity of *Viola betonicifolia* whole plant. BMC complementary and alternative medicine. 2012 Dec 1;12(1):59. https://doi.org/10.1186/1472-6882-12-59

Muhammed Jasim A, Suja SR, Anusha S, Nair RR. Analysis of anti-oxidant and anti-inflammatory potential of *Baccaurea courtallensis* (Wight) Mull. Arg. Journal of Pharmacognosy and Phytochemistry. 2019;8(3):3994-4000.

Mujumdar AM, Misar AV. Anti-inflammatory activity of *Jatropha curcas* roots in mice and rats. Journal of ethnopharmacology. 2004 Jan 1;90(1):11-5. https://doi.org/10.1016/j.jep.2003.09.019

Mukherjee K, Paul P, Banerjee ER. Anti inflammatory activities of date palm extracts (*Phoenix sylvestris*). Antioxidants. 2001;3(1):1-21. doi:10.3390/antiox30x000x

Mukherjee M, Das AS, Das D, Mukherjee S, Mitra S, Mitra C. Role of oil extract of garlic (*Allium sativum* Linn.) on intestinal transference of calcium and its possible correlation with preservation of skeletal health in an ovariectomized rat model of osteoporosis. Phytotherapy Research: An International Journal Devoted to Pharmacological and Toxicological Evaluation of Natural Product Derivatives. 2006 May;20(5):408-15. https://doi.org/10.1002/ptr.1888

Mulla WA, More SD, Jamge SB, Pawar AM, Kazi MS, Varde MR. Evaluation of antiinflammatory and analgesic activities of ethanolic extract of roots *Adhatoda vasica* Linn. International Journal of PharmTech Research. 2010;2(2):1364-8.

Mumtaz SM, Ahmed S, Dey S. Antiepileptic and central nervous system depressant activity of *Sechium edule* fruit extract. Bangladesh Journal of Pharmacology. 2012 Sep 25;7(3):199-202. https://doi.org/10.3329/bjp.v7i3.11275

Munazir M, Qureshi R, Arshad M, Gulfraz M. Antibacterial activity of root and fruit extracts of *Leptadenia pyrotechnica* (Asclepiadaceae) from Pakistan. Pak. J. Bot. 2012 Aug 1;44(4):1209-13.

Mundo J, Castillo-España P, Villafuerte MD, Camacho CA, León-Rivera I, Arellano-García J, Perea-Arango I. Methanolic extracts from roots and cell suspension cultures of *Waltheria americana* Linn induce GABA release in cerebral slices of mouse brain. Afr. J. Pharm. Pharmacol.. 2015;9:139-44. https://doi.org/10.5897/AJPP2014.4115

Munodawafa T, Chagonda LS, Moyo SR. Antimicrobial and phytochemical screening of some Zimbabwean medicinal plants. Journal of Biologically Active Products from Nature. 2013 Nov 2;3(5-6):323-30. https://doi.org/10.1080/22311866.2013.782759

Muñoz O, Christen P, Cretton S, Backhouse N, Torres V, Correa O, Costa E, Miranda H, Delporte C. Chemical study and anti-inflammatory, analgesic and antioxidant activities of the leaves of *Aristotelia chilensis* (Mol.) Stuntz, Elaeocarpaceae. Journal of Pharmacy and Pharmacology. 2011 Jun;63(6):849-59.  https://doi.org/10.1111/j.2042-7158.2011.01280.x

Muñoz-Cazares N, Aguilar-Rodríguez S, García-Contreras R, Soto-Hernández M, Martínez-Vázquez M, Palma-Tenango M, Prado-Galbarro FJ, Castillo-Juárez I. Phytochemical screening and anti-virulence properties of *Ceiba pentandra* and *Ceiba aesculifolia* (Malvaceae) bark extracts and fractions. Botanical Sciences. 2018 Sep;96(3):415-25.

Mur E, Hartig F, Eibl G, Schirmer M. Randomized double blind trial of an extract from the pentacyclic alkaloid-chemotype of *Uncaria tomentosa* for the treatment of rheumatoid arthritis. The Journal of Rheumatology. 2002 Apr 1;29(4):678-81. http://www.jrheum.org/content/29/4/678

Murakami S, Miyazaki I, Asanuma M. Neuroprotective effect of fermented papaya preparation by activation of Nrf2 pathway in astrocytes. Nutritional neuroscience. 2018 Mar 16;21(3):176-84. https://doi.org/10.1080/1028415X.2016.1253171

Murti K, Kumar U. Antimicrobial activity of *Ficus benghalensis* and *Ficus racemosa* roots L. Am. J. microbiol. 2011;2(1):21-4.

Muruganandan S, Srinivasan K, Chandra S, Tandan SK, Lal J, Raviprakash V. Anti-inflammatory activity of *Syzygium cumini* bark. Fitoterapia. 2001 May 1;72(4):369-75. https://doi.org/10.1016/S0367-326X(00)00325-7

Musabayane CT, Kamadyaapa DR, Gondwe M, Moodley K, Ojewole JA. Cardiovascular effects of *Helichrysum ceres* S Moore [Asteraceae] ethanolic leaf extract in some experimental animal paradigms. Cardiovasc J Afr. 2008;19(5):246–253.

Mustafa B, Hajdari A, Pieroni A, Pulaj B, Koro X, Quave CL. A cross-cultural comparison of folk plant uses among Albanians, Bosniaks, Gorani and Turks living in south Kosovo. Journal of ethnobiology and ethnomedicine. 2015 Dec;11(1):39. https://doi.org/10.1186/s13002-015-0023-5

Mutai C, Bii C, Rukunga G, Ondicho J, Mwitari P, Abatis D, Vagias C, Roussis V, Kirui J. Antimicrobial activity of pentacyclic triterpenes isolated from *Acacia mellifera*. African Journal of Traditional, Complementary and Alternative Medicines. 2009;6(1). https://doi.org/10.1016/j.jep.2009.02.007

Muwanga C. *An assessment of Hypoxis hemerocallidea extracts, and actives as natural antibiotic, and immune modulation phytotherapies. 2006.* (Doctoral dissertation, University of the Western Cape.

Mwitari PG, Ayeka PA, Ondicho J, Matu EN, Bii CC. Antimicrobial activity and probable mechanisms of action of medicinal plants of Kenya*: Withania somnifera, Warbugia ugandensis, Prunus africana* and *Plectrunthus barbatus*. PloS one. 2013 Jun 13;8(6):e65619. https://doi.org/10.1371/journal.pone.0065619

Mwonjoria JK, Kariuki HN, Waweru FN. The antinociceptive antipyretic effects of *Solanum incanum* (Linneaus) in animal models. International Journal. 2011;2229:7472.

Mykkänen OT, Huotari A, Herzig KH, Dunlop TW, Mykkänen H, Kirjavainen PV. Wild blueberries (*Vaccinium myrtillus*) alleviate inflammation and hypertension associated with developing obesity in mice fed with a high-fat diet. *PLoS One*. 2014;9(12):e114790. doi:10.1371/journal.pone.0114790

N'guessan JD, Dinzedi MR, Guessennd N, Coulibaly A, Dosso M, Djaman AJ, Guede-Guina F. Antibacterial activity of the aqueous extract of *Thonningia sanguinea* against Extended-Spectrum-b-Lactamases (ESBL) producing *Escherichia coli* and *Klebsiella pneumoniae* strains. Tropical Journal of Pharmaceutical Research. 2007;6(3):779-83.

Nagai T, Kiyohara H, Munakata K, Shirahata T, Sunazuka T, Harigaya Y, Yamada H. Pinellic acid from the tuber of *Pinellia ternata* Breitenbach as an effective oral adjuvant for nasal influenza vaccine. International immunopharmacology. 2002 Jul 1;2(8):1183-93. https://doi.org/10.1016/S1567-5769(02)00086-3

Nagalakshmi MA, Thangadurai D, Rao DM, Pullaiah T. Phytochemical and antimicrobial study of *Chukrasia tabularis* leaves. Fitoterapia. 2001 Jan 1;72(1):62-4. https://doi.org/10.1016/S0367-326X(00)00245-8

Nagar JC, Chauhan LS. Evaluation of Antihyperglycemic and Antihyperlipidemic Activity of Leaf Extracts of *Breynia vitis-idaea* in Alloxan Induced Diabetic Rats. Pharmacognosy Journal. 2016 May;8(3):259.

Nahata A, Patil UK, Dixit VK. Effect of *Convulvulus pluricaulis* Choisy. on learning behaviour and memory enhancement activity in rodents. Natural product research. 2008 Nov 10;22(16):1472-82.doi:10.1128/MCB.00292-16 https://doi.org/10.1080/14786410802214199

Nair R, Chanda S. Antibacterial activities of some medicinal plants of the western region of India. Turkish Journal of Biology. 2007 Dec 12;31(4):231-6.

Nakagawa T, Itoh M, Ohta K, Hayashi Y, Hayakawa M, Yamada Y, Akanabe H, Chikaishi T, Nakagawa K, Itoh Y, Muro T. Improvement of memory recall by quercetin in rodent contextual fear conditioning and human early-stage Alzheimer's disease patients. Neuroreport. 2016 Jun 15;27(9):671-6.. doi: 10.1097/WNR.0000000000000594

Nakahara K, Alzoreky NS, Yoshihashi T, Nguyen HT, Trakoontivakorn G. Chemical composition and antifungal activity of essential oil from *Cymbopogon nardus* (citronella grass). Japan Agricultural Research Quarterly: JARQ. 2013 Oct 31;37(4):249-52. doi.org/10.6090/jarq.37.249

Nam JW, Kim SY, Yoon T, Lee YJ, Kil YS, Lee YS, Seo EK. Heat shock factor 1 inducers from the bark of *Eucommia ulmoides* as cytoprotective agents. Chemistry & biodiversity. 2013 Jul;10(7):1322-7.

Nammi S, Gudavalli R, Babu BS, Lodagala DS, Boini KM. Possible mechanisms of hypotension produced 70% alcoholic extract of *Terminalia arjuna* (L.) in anaesthetized dogs. BMC complementary and alternative medicine. 2003 Dec 1;3(1):5. https://doi.org/10.1186/1472-6882-3-5

Namtak S, Sharma RC. Medicinal plant resources in Skuru watershed of Karakoram wildlife sanctuary and their uses in traditional medicines system of Ladakh, India. International journal of. 2018:294-302. DOI: 10.15406/ijcam.2018.11.00415

Nani A, Belarbi M, Ksouri-Megdiche W, Abdoul-Azize S, Benammar C, Ghiringhelli F, Hichami A, Khan NA. Effects of polyphenols and lipids from *Pennisetum glaucum* grains on T-cell activation: modulation of Ca 2+ and ERK1/ERK2 signaling. BMC complementary and alternative medicine. 2015 Dec;15(1):1-1. https://doi.org/10.1186/s12906-015-0946-3

Nanyingi MO, Mbaria JM, Lanyasunya AL, Wagate CG, Koros KB, Kaburia HF, Munenge RW, Ogara WO. Ethnopharmacological survey of Samburu district, Kenya. Journal of Ethnobiology and Ethnomedicine. 2008 Dec;4(1):14. https://doi.org/10.1186/1746-4269-4-14

Narayanan N, Thirugnanasambantham P, Viswanathan S, Vijayasekaran V, Sukumar E. Antinociceptive, anti-inflammatory and antipyretic effects of ethanol extract of *Clerodendron serratum* roots in experimental animals. Journal of Ethnopharmacology. 1999 Jun 1;65(3):237-41. doi.org/10.1016/S0378-8741(98)00176-7

Narayanan S, Ruma D, Gitika B, Sharma SK, Pauline T, Ram MS, Ilavazhagan G, Sawhney RC, Kumar D, Banerjee PK. Antioxidant activities of seabuckthorn (*Hippophae rhamnoides*) during hypoxia induced oxidative stress in glial cells. Mol Cell Biochem. 2005 Oct;278(1-2):9-14. doi: 10.1007/s11010-005-7636-2.

Narender KS, Kumar D, Kumar V. Antinociceptive and anti-inflammatory activity of *Hibiscus tiliaceus* leaves. International Journal of Pharmacognosy and Phytochemical Research. 2009;1(1).

Narendhirakannan RT, Subramanian S, Kandaswamy M. Anti-inflammatory and lysosomal stability actions of *Cleome gynandra* L. studied in adjuvant induced arthritic rats. Food and Chemical Toxicology. 2007 Jun 1;45(6):1001-12.

Narkhede MB, Ajmire PV, Wagh AE. Evaluation of antinociceptive and anti-inflammatory activity of ethanol extract of *Murraya paniculata* leaves in experimental rodents. Int J Pharm Pharm Sci. 2012;4(1):247-50.

Nasab FK, Khosravi AR. Ethnobotanical study of medicinal plants of Sirjan in Kerman Province, Iran. Journal of ethnopharmacology. 2014 May 28;154(1):190-7. https://doi.org/10.1016/j.jep.2014.04.003

Nascimento SA, Araujo EA, Da Silva JM, Ramos CS. Chemical study and antimicrobial activities of *Piper arboreum* (Piperaceae). Journal of the Chilean Chemical Society. 2015 Mar;60(1):2837-9. http://dx.doi.org/10.4067/S0717-97072015000100013

Naseri M, Ahmadi A, Gharegozli K, Nabavi M, Faghihzadeh S, Ashtarian N, Montazami F, Rezaeizadeh H. A double blind, placebo-controlled, crossover study on the effect of MS14, an herbal-marine drug, on quality of life in patients with multiple sclerosis. J Med Plant Res. 2009 Apr 1;3(4):271-5.

Náthia-Neves G, Tarone AG, Tosi MM, Júnior MR, Meireles MA. Extraction of bioactive compounds from genipap (*Genipa americana* L.) by pressurized ethanol: Iridoids, phenolic content and antioxidant activity. Food research international. 2017 Dec 1;102:595-604. https://doi.org/10.1016/j.foodres.2017.09.041

Navarro-Herrera D, Aranaz P, Eder-Azanza L, Zabala M, Romo-Hualde A, Hurtado C, Calavia D, López-Yoldi M, Martínez JA, González-Navarro CJ, Vizmanos JL. *Borago officinalis* seed oil (BSO), a natural source of omega-6 fatty acids, attenuates fat accumulation by activating peroxisomal beta-oxidation both in *C. elegans* and in diet-induced obese rats. Food & function. 2018;9(8):4340-51. https://doi.org/10.1039/C8FO00423D

Nawaz HR, Malik A, Ali MS. Trianthenol: an antifungal tetraterpenoid from *Trianthema portulacastrum* (Aizoaceae). Phytochemistry. 2001 Jan 1;56(1):99-102. https://doi.org/10.1016/S0031-9422(00)00270-3

Nawaz KA, David SM, Murugesh E, Thandeeswaran M, Kiran KG, Mahendran R, Palaniswamy M, Angayarkanni J. Identification and in silico characterization of a novel peptide inhibitor of angiotensin converting enzyme from pigeon pea (Cajanus cajan). Phytomedicine. 2017 Dec 1;36:1-7. https://doi.org/10.1016/j.phymed.2017.09.013

Nayak BS. *Cecropia peltata* L (Cecropiaceae) has wound-healing potential: a preclinical study in a Sprague Dawley rat model. The international journal of lower extremity wounds. 2006 Mar;5(1):20-6. doi.org/10.1177/1534734606286472

Nazif NM. Phytoconstituents of *Zizyphus spina-christi* L. fruits and their antimicrobial activity. Food Chem. 2002;76:77–81.

Ndebia EJ, Kamgang R, Nkeh-ChungagAnye BN. Analgesic and anti-inflammatory properties of aqueous extract from leaves of *Solanum torvum* (Solanaceae). African Journal of Traditional, Complementary and Alternative Medicines. 2007;4(2):240-4.

Ndhlala AR, Ghebrehiwot HM, Ncube B, Aremu AO, Gruz J, Šubrtová M, Doležal K, du Plooy CP, Abdelgadir HA, Van Staden J. Antimicrobial, anthelmintic activities and characterisation of functional phenolic acids of achyranthes aspera linn.: a medicinal plant used for the treatment of wounds and Ringworm in East Africa. Frontiers in pharmacology. 2015 Nov 23;6:274.

Ndiaye F, Vuong T, Duarte J, Aluko RE, Matar C. Anti-oxidant, anti-inflammatory and immunomodulating properties of an enzymatic protein hydrolysate from yellow field pea seeds. European journal of nutrition. 2012 Feb 1;51(1):29-37. https://doi.org/10.1007/s00394-011-0186-3

Ndip RN, Ajonglefac AN, Mbullah SM, Tanih NF, Akoachere JF, Ndip LM, Luma HN, Wirmum C, Ngwa F, Efange SM. In vitro anti-*Helicobacter pylori* activity of *Lycopodium cernuum* (Linn) Pic. Serm. African Journal of Biotechnology. 2008;7(22).

Necchi RM, Alves IA, Alves SH, Manfron MP. In vitro antimicrobial activity, total polyphenols and flavonoids contents of *Nopalea cochenillifera* (L.) Salm-Dyck (Cactaceae). Research in Pharmacy. 2012. http://updatepublishing.com/journal/index.php/rip/article/view/266

Nedelcheva A, Pieroni A, Dogan Y. Folk food and medicinal botanical knowledge among the last remaining Yörüks of the Balkans. Acta Soc Bot Pol. 2017;86(2):3522. https://doi. org/10.5586/asbp.3522

Negi JS, Bisht VK, Bhandari AK, Singh P, Sundriyal RC. Chemical constituents and biological activities of the genus *Zanthoxylum*: a review. African Journal of Pure and Applied Chemistry. 2011 Oct 30;5(12):412-6.

Negi PS, Jayaprakasha GK, Jena BS. Antibacterial activity of the extracts from the fruit rinds of *Garcinia cowa* and *Garcinia pedunculata* against food borne pathogens and spoilage bacteria. LWT-Food Science and Technology. 2008 Dec 1;41(10):1857-61. https://doi.org/10.1016/j.lwt.2008.02.009

Neji PA, Neji HA, Ushie OA, Ojong OO. Phytochemical screening and antimicrobial activity of leaf extracts of *Leea guineensis*. Trends in Science & Technology Journal 2016:1(2): 448 – 456.

Nematolahi P, Mehrabani M, Karami-Mohajeri S, Dabaghzadeh F. Effects of *Rosmarinus officinalis* L. on memory performance, anxiety, depression, and sleep quality in university students: a randomized clinical trial. Complementary therapies in clinical practice. 2018 Feb 1;30:24-8. https://doi.org/10.1016/j.ctcp.2017.11.004

Nemetchek MD, Stierle AA, Stierle DB, Lurie DI. The Ayurvedic plant *Bacopa monnieri* inhibits inflammatory pathways in the brain. J Ethnopharmacol. 2017 Feb 2;197:92-100. doi: 10.1016/j.jep.2016.07.073.

Nessa F, Ismail Z, Mohamed N, Haris MR. Free radical-scavenging activity of organic extracts and of pure flavonoids of *Blumea balsamifera* DC leaves. Food Chemistry. 2004 Nov 1;88(2):243-52. doi.org/10.1016/j.foodchem.2004.01.041

Nessa F, Ismail Z, Mohamed N. Antimicrobial activities of extracts and flavonoid glycosides of corn silk (*Zea mays* L). International Journal of Biotechnology for Wellness Industries. 2012 Jun 18;1(2):115-20.

Neto CC, Owens CW, Langfield RD, Comeau AB, Onge JS, Vaisberg AJ, Hammond GB. Antibacterial activity of some Peruvian medicinal plants from the Callejon de Huaylas. Journal of ethnopharmacology. 2002 Feb 1;79(1):133-8. https://doi.org/10.1016/S0378-8741(01)00398-1

Neto PA, Silva MV, Campos NV, Porfírio Z, Caetano LC. Antibacterial activity of *Borreria verticillata* roots. Fitoterapia. 2002 Oct 1;73(6):529-31. https://doi.org/10.1016/S0367-326X(02)00166-1

Neurath AR, Strick N, Li YY, Debnath AK. *Punica granatum* (Pomegranate) juice provides an HIV-1 entry inhibitor and candidate topical microbicide. BMC Infectious diseases. 2004 Dec 1;4(1):41. https://doi.org/10.1186/1471-2334-4-41

Neves MC, Neves PC, Zanini Jr JC, Medeiros YS, Yunes RA, Calixto JB. Analgesic and anti-inflammatory activities of the crude hydroalcoholic extract obtained from the bark of *Hymenaea martiana*. Phytotherapy Research. 1993 Sep;7(5):356-62. https://doi.org/10.1002/ptr.2650070507

Nezhadali A, Masrornia M, Bari H, Akbarpour M, Joharchi MR, Nakhaei-Moghadam M. Essential oil composition and antibacterial activity of *Nepeta glomerulosa* Boiss from Iran. Journal of Essential Oil Bearing Plants. 2011 Jan 1;14(2):241-4.

Ngeny LC, Magiri E, Mutai C, Mwikwabe N, Bii C. Antimicrobial properties and toxicity of *Hagenia abyssinica* (Bruce) JF Gmel, *Fuerstia africana* TCE Fries, *Asparagus racemosus* (Willd.) and *Ekebergia capensis* Sparrm. African Journal of Pharmacology and Therapeutics. 2013 Jan 10;2(3).

Ngo Bum E, Dawack DL, Schmutz M, Rakotonirina A, Rakotonirina SV, Portet C, Jeker A, Olpe HR, Herrling P. Anticonvulsant activity of *Mimosa pudica* decoction. Fitoterapia. 2004 Jun 1;75(3-4):309-14. https://doi.org/10.1016/j.fitote.2004.01.012

Ngoci NS, Ramadhan M, Ngari MS, Leonard OP. Screening for antimicrobial activity of *Cissampelos pareira* L. methanol root extract. European Journal of Medicinal Plants. 2014:45-51. https://doi.org/10.9734/EJMP/2014/5464

Ngono Ngane RA, Mogtomo MK, Tiabou AT, Nana HM, Chieffo PM, Bounou ZM, Etame RE, Ndifor F, Biyiti L, Zollo PA,. A Ethnobotanical survey of some Cameroonian plants used for the treatment of viral disease. African Journal of Plant Science. 2011;5(1):15-21.

Ngounou FN, Choudhary MI, Malik S, Makhmoor T, Nur-E-Alam M, Zareen S, Lontsi D, Ayafor JF, Sondengam BL. New antioxidant and antimicrobial ellagic acid derivatives from *Pteleopsis hylodendron*. Planta Medica. 2001;67(04):335-9. doi: 10.1055/s-2001-14306

Ngoutane Mfopa A, Corona A, Eloh K, Tramontano E, Frau A, Boyom FF, Caboni P, Tocco G. Uvaria angolensis as a promising source of inhibitors of HIV-1 RT-associated RNA-dependent DNA polymerase and RNase H functions. Natural product research. 2018 Mar 19;32(6):640-7. https://doi.org/10.1080/14786419.2017.1332615

Nguelefack TB, Mekhfi H, Dimo T, Afkir S, Nguelefack-Mbuyo EP, Legssyer A, Ziyyat A. Cardiovascular and anti-platelet aggregation activities of extracts from *Solanum torvum* (Solanaceae) fruits in rat. Journal of Complementary and Integrative Medicine. 2008 May 20;5(1).

N'guessan JD, Coulibaly A, Ramanou AA, Okou OC, Djaman AJ, Guédé-Guina F. Antibacterial activity of *Thonningia sanguinea* against some multi-drug resistant strains of *Salmonella enterica*. African health sciences. 2007;7(3).

Ngule CM, Ndiku HM. Phytochemical evaluation and in vitro antibacterial activity of methanolic-aqua extract of *Indigofera arrecta* leaves against selected pathogenic microorganisms. African Journal of Pharmacology and Therapeutics Vol. 2014;3(3):74-8.

Ngure P, Ng'ang'a Z, Kimutai A, et al. Immunostimulatory responses to crude extracts of *Warburgia ugandensis* (Sprague) subsp *ugandensis* (Canellaceae) by BALB/c mice infected with *Leishmania major*. Pan Afr Med J. 2014;17 Suppl 1(Suppl 1):15. Published 2014 Jan 18. doi:10.11694/pamj.supp.2014.17.1.3638

Nguyen DK, Dai TT. Study on tau-aggregation inhibitors in Alzheimer's disease of methanol extracts of several medicinal plants collected in the Mekong Delta, Vietnam. Science and Technology Development Journal-Natural Sciences. 2017 Jun 30;1(T2):21-8. https://doi.org/https://doi.org/10.32508/stdjns.v1iT2.455

Ni W, Gao T, Wang H, Du Y, Li J, Li C, Wei L, Bi H. Anti-fatigue activity of polysaccharides from the fruits of four Tibetan plateau indigenous medicinal plants. Journal of ethnopharmacology. 2013 Nov 25;150(2):529-35.

Nie PH, Zhang L, Zhang WH, Rong WF, Zhi JM. The effects of hydroxysafflor yellow A on blood pressure and cardiac function. Journal of ethnopharmacology. 2012 Feb 15;139(3):746-50. https://doi.org/10.1016/j.jep.2011.11.054

Nielsen TR, Kuete V, Jäger AK, Meyer JJ, Lall N. Antimicrobial activity of selected South African medicinal plants. BMC complementary and alternative medicine. 2012 Dec;12(1):74. https://doi.org/10.1186/1472-6882-12-74

Nikolić M, Glamočlija J, Ferreira IC, Calhelha RC, Fernandes Â, Marković T, Marković D, Giweli A, Soković M. Chemical composition, antimicrobial, antioxidant and antitumor activity of *Thymus serpyllum* L., *Thymus algeriensis* Boiss. and Reut and *Thymus vulgaris* L. essential oils. Industrial Crops and Products. 2014 Jan 1;52:183-90.

Nim S, Mónico A, Rawal MK, Duarte N, Prasad R, Di Pietro A, Ferreira MJ. Overcoming multidrug resistance in Candida albicans: macrocyclic diterpenes from *Euphorbia* species as potent inhibitors of drug efflux pumps. Planta medica. 2016 Aug;82(13):1180-5. doi: 10.1055/s-0042-106169

Nirmala P, Selvaraj T. Anti-inflammatory and anti-bacterial activities of *Glycyrrhiza glabra* L. Journal of Agricultural Technology. 2011;7(3):815-23.

Nishina A, Yoshii K, Fukatsu M, Kushi Y, Suzuki Y, Ukiya M. Induction of neuronal differentiation by extracts from 57 kinds of traditional medicinal plants in Myanmar. Journal of Research Institute of Science and Technology, College of Science and Technology, Nihon University. 2017;139:1-11.

Nishteswar K. Identification of New Medicinal Plants Enumerated In Telugu Cantos of Basavarajeeyam. Journal of Research Traditional Medicine. 2015 Nov;1(1):3-9.

Nivas D, Dethe UL, Gaikwad DK. In vitro Antioxidant Activities and Antimicrobial Efficacy of Asian Snakewood; *Colubrina asiatica* (L.) Brong. Research Journal of Medicinal Plant. 2015;9:307-20. doi: 10.3923/rjmp.2015.307.320

Niyonzima G, Laekeman G, Witvrouw M, Van Poel B, Pieters L, Paper D, De Clercq E, Franz G, Vlietinck AJ. Hypoglycemic, anticomplement and anti-HIV activities of *Spathodea campanulata* stem bark. Phytomedicine. 1999 Mar 1;6(1):45-9. https://doi.org/10.1016/S0944-7113(99)80034-8

Njamen D, Talla E, Mbafor JT, Fomum ZT, Kamanyi A, Mbanya JC, Cerdá-Nicolás M, Giner RM, Recio MC, Ríos JL. Anti-inflammatory activity of erycristagallin, a pterocarpene from *Erythrina mildbraedii*. European journal of pharmacology. 2003 May 2;468(1):67-74.

Njan AA, Adenuga FO, Ajayi AM, Sotunde O, Ologe MO, Olaoye SO, Erdogan ON, Iwalewa OE. Neuroprotective and memory-enhancing effects of methanolic leaf extract of *Peristrophe bicalyculata* in rat model of type 2 diabetes mellitus. Heliyon. 2020 May 22;6(5):e04011. doi: 10.1016/j.heliyon.2020.e04011.

Njeru SN, Obonyo M, Nyambati S, Ngari S, Mwakubambanya R, Mavura H. Antimicrobial and cytotoxicity properties of the organic solvent fractions of Clerodendrum myricoides (Hochst.) R. Br. ex Vatke: Kenyan traditional medicinal plant. Journal of intercultural ethnopharmacology. 2016 Jun;5(3):226.

Nkeh-Chungag BN, Tiya S, Mbafor JT, Ndebia EJ, Rusike S, Iputo JE. Effects of the methanol extract of *Erythrina abyssinica* on hot flashes in ovariectomized rats. African Journal of Biotechnology. 2013;12(6).

Nkere CK, Iroegbu CU. Antibacterial screening of the root, seed and stembark extracts of Picralima nitida. African Journal of Biotechnology. 2005;4(6):522-6.

Noamesi BK, Adebayo GI, Bamgbose SO. The vascular actions of aqueous extract of Lippia multiflora. Planta medica. 1985 Jun;51(03):256-8.

Nogueira JC, Diniz Mde F, Lima EO. *In vitro* antimicrobial activity of plants in Acute Otitis Externa. Braz J Otorhinolaryngol. 2008;74:118–24.

Nogueira L, Ramirez-Sanchez I, Perkins GA, Murphy A, Taub PR, Ceballos G, Villarreal FJ, Hogan MC, Malek MH. (–)-Epicatechin enhances fatigue resistance and oxidative capacity in mouse muscle. The Journal of physiology. 2011 Sep;589(18):4615-31.

Nolkemper S, Reichling J, Stintzing FC, Carle R, Schnitzler P. Antiviral effect of aqueous extracts from species of the Lamiaceae family against *Herpes simplex* virus type 1 and type 2 in vitro. Planta medica. 2006 Dec;72(15):1378-82

Noor H, Cao P, Raleigh DP. Morin hydrate inhibits amyloid formation by islet amyloid polypeptide and disaggregates amyloid fibers. Protein Science. 2012 Mar;21(3):373-82. https://doi.org/10.1002/pro.2023

Noor S, Rahman SA, Ahmed Z, Das A, Hossain MM. Evaluation of anti-inflammatory and antidiabetic activity of ethanolic extracts of *Desmodium pulchellum* Benth.(Fabaceae) barks on albino wistar rats. Journal of Applied Pharmaceutical Science. 2013 Jul 1;3(7):48. doi: 10.7324/JAPS.2013.3709

Noriega P, Vergara B, Carillo C, Mosquera T. Chemical Constituents and Antifungal Activity of Leaf Essential Oil from *Oreopanax ecuadorensis* Seem.(Pumamaki), Endemic Plant of Ecuador. Pharmacognosy Journal. 2019;11(6s). doi: 10.5530/pj.2019.11.236

Nothias-Scaglia LF, Dumontet V, Neyts J, Roussi F, Costa J, Leyssen P, Litaudon M, Paolini J. LC-MS²-Based dereplication of *Euphorbia* extracts with anti-Chikungunya virus activity. Fitoterapia. 2015 Sep;105:202-9. doi: 10.1016/j.fitote.2015.06.021.

Notka F, Meier G, Wagner R. Concerted inhibitory activities of *Phyllanthus amarus* on HIV replication in vitro and ex vivo. Antiviral research. 2004 Nov 1;64(2):93-102. https://doi.org/10.1016/j.antiviral.2004.06.010

Noumedem JA, Mihasan M, Lacmata ST, Stefan M, Kuiate JR, Kuete V. Antibacterial activities of the methanol extracts of ten Cameroonian vegetables against Gram-negative multidrug-resistant bacteria. BMC Complementary and Alternative Medicine. 2013 Dec;13(1):26. . https://doi.org/10.1186/1472-6882-13-26

Nourazarian SM, Nourazarian A, Majidinia M, Roshaniasl E. Effect of root extracts of medicinal herb *Glycyrrhiza glabra* on HSP90 gene expression and apoptosis in the HT-29 colon cancer cell line. Asian Pacific Journal of Cancer Prevention. 2016;16(18):8563-6.

Núñez Guillén ME, da Silva Emim JA, Souccar C, Lapa AJ. Analgesic and anti-inflammatory activities of the aqueous extract of *Plantago major* L. International Journal of Pharmacognosy. 1997 Jan 1;35(2):99-104. https://doi.org/10.1076/phbi.35.2.99.13288

Núñez V, Otero R, Barona J, Saldarriaga M, Osorio RG, Fonnegra R, Jiménez SL, Diaz A, Quintana JC. Neutralization of the edema-forming, defibrinating and coagulant effects of *Bothrops asper* venom by extracts of plants used by healers in Colombia. Brazilian Journal of Medical and Biological Research. 2004 Jul;37(7):969-77. https://doi.org/10.1590/S0100-879X2004000700005

Nwabor OF, Dibua UM, Ngwu GI, Onyenma NC, Odiachi O, Nnamonu EI, Okoro JO, Eze TR, Okeke IS. Evaluation of the antimicrobial and larvicidal potentials of seed extracts of *Picralima nitida*. International Research Journal of Natural Sciences Vol.2, No.2, pp.23-30, June 2014

Nwabunike IA, Ezike AC, Nwodo NJ, Udegbunam SU, Okoli CO. Bioactivity-guided studies on the antiinflammatory activity of extract of aerial parts of *Schwenckia americana* L.(Solanaceae). J. Med. Plant Res. 2014 Jun 10;8(22):794-801.

Nwachukwu DC, Aneke E, Nwachukwu NZ, Obika LF, Nwagha UI, Eze AA. Effect of *Hibiscus sabdariffa* on blood pressure and electrolyte profile of mild to moderate hypertensive Nigerians: A comparative study with hydrochlorothiazide. Nigerian journal of clinical practice. 2015;18(6):762-70. doi: 10.4103/1119-3077.163278.

Nwadinigwe AO. Antimicrobial activities of some fractions of the extract of *Emilia sonchifolia* (Linn.) DC (Asteraceae). Plant Products Research Journal. 2009;13(1). doi: 10.4314/pprj.v13i1.65813

Nwodo OF. Antibiotic and anti-inflammatory analgesic activities of *Harungana madagascariensis* stem bark. International Journal of Crude Drug Research. 1989 Jan 1;27(3):137-40.

Nwodo UU, Ngene AA, Iroegbu CU, Onyedikachi OA, Chigor VN, Okoh AI. In vivo evaluation of the antiviral activity of *Cajanus cajan* on measles virus. Archives of virology. 2011 Sep;156(9):1551-7. https://doi.org/10.1007/s00705-011-1032-x

Nwokocha CR, Owu DU, McLaren M, Murray J, Delgoda R, Thaxter K, McCalla G, Young L. Possible mechanisms of action of the aqueous extract of *Artocarpus altilis* (breadfruit) leaves in producing hypotension in normotensive Sprague–Dawley rats. Pharmaceutical biology. 2012 Sep 1;50(9):1096-102.

Nworu CS, Nwuke HC, Akah PA, Okoye FB, Esimone CO. Extracts of *Ficus exasperata* leaf inhibit topical and systemic inflammation in rodents and suppress LPS-induced expression of mediators of inflammation in macrophages. Journal of immunotoxicology. 2013 Sep 1;10(3):302-10.

Nyegue M, Ndoyé F, Amvam Zollo H, Etoa FX, Agnaniet H, Menut C. Chemical and biological evaluation of essential oil of *Pentadiplandra brazzeana* (Bail.) roots from Cameroon. Adv Phytotherapy Res. 2009:91-107.

Nzogong RT, Ndjateu FS, Ekom SE, Fosso JA, Awouafack MD, Tene M, Tane P, Morita H, Choudhary MI. Antimicrobial and antioxidant activities of triterpenoid and phenolic derivatives from two Cameroonian Melastomataceae plants: *Dissotis senegambiensis* and *Amphiblemma monticola*. BMC complementary and alternative medicine. 2018 Dec;18(1):159. https://doi.org/10.1186/s12906-018-2229-2

Obade, E., Ilesanmi, O.B., Crown, O., Akinmoladun, A.C., Olaleye, T.M. and Akindahunsi, A.A., 2018. Neuromodulatory effect of solvent fractions of Africa eggplant (*Solanium dasyphyllum*) against KCN-induced mitochondria damage, viz.

NADH-succinate dehydrogenase, NADH-cytochrome c reductase, and succinate-cytochrome c reductase. *Clinical Phytoscience*, *4*(1), p.9. https://doi.org/10.1186/s40816-018-0068-9

Obi BC, Igweze Z, Nwaogu V, Ben C, Akunne TC. Studies on the anticonvulsant and sedative effects of *Jatropha curcas* (Euphorbiaceae) and *Phragmanthera capitata* (Loranthaceae) in mice. Drug Discovery. 2019;13:95-102.

Obi RK, Nwanebu FC, Ndubuisi UU, Orji NM. Antibacterial qualities and phytochemical screening of the oils of *Curcubita pepo* and *Brassica nigra.* Journal of Medicinal Plants Research. 2009 May 31;3(5):429-32.

Obiefuna I, Young R. Concurrent administration of aqueous *Azadirachta indica* (neem) leaf extract with DOCA-salt prevents the development of hypertension and accompanying electrocardiogram changes in the rat. Phytotherapy Research: An International Journal Devoted to Pharmacological and Toxicological Evaluation of Natural Product Derivatives. 2005 Sep;19(9):792-5. https://doi.org/10.1002/ptr.1739

Obiukwu CE, Nwanekwu KE. Evaluation of the antimicrobial potentials of 35 medicinal plants from Nigeria. Int Sci Res J. 2010;2:48-51.

Oboh G, Akinyemi AJ, Omojokun OS, Oyeleye IS. Anticholinesterase and antioxidative properties of aqueous extract of *Cola acuminata* seed in vitro. International Journal of Alzheimer's Disease. 2014 Nov 18;2014. Article ID 498629. https://doi.org/10.1155/2014/498629

Oboh G, Nwanna EE, Oyeleye SI, Olasehinde TA, Ogunsuyi OB, Boligon AA. In vitro neuroprotective potentials of aqueous and methanol extracts from *Heinsia crinita* leaves. Food Science and Human Wellness. 2016 Jun 1;5(2):95-102. https://doi.org/10.1016/j.fshw.2016.03.001

Oboh IE, Onwukaeme DN. Analgesic, anti-inflammatory and anti-ulcer activities of *Sida acuta* in mice and rat. Nigerian Journal of Natural Products and Medicine. 2005;9(1):19-21.

Odabasoglu F, Halici Z, Cakir A, Halici M, Aygun H, Suleyman H, Cadirci E, Atalay F. Beneficial effects of vegetable oils (corn, olive and sunflower oils) and α-tocopherol on anti-inflammatory and gastrointestinal profiles of indomethacin in rats. European Journal of Pharmacology. 2008 Sep 4;591(1-3):300-6. https://doi.org/10.1016/j.ejphar.2008.06.075

Odongo E, Mungai N, Mutai P, Karumi E, Mwangi J, Omale J. Ethnobotanical survey of the medicinal plants used in Kakamega County, western Kenya. Applied Medical Research. 2018;4(1):22.

Odonne G, Valadeau C, Alban-Castillo J, Stien D, Sauvain M, Bourdy G. Medical ethnobotany of the Chayahuita of the Paranapura basin (Peruvian Amazon). J Ethnopharmacol. 2013 Mar 7;146(1):127-53. doi: 10.1016/j.jep.2012.12.014.

Odubanjo VO, Ibukun EO, Oboh G, Adefegha SA. Aqueous extracts of two tropical ethnobotanicals (*Tetrapleura tetraptera* and *Quassia undulata*) improved spatial and non-spatial working memories in scopolamine-induced amnesic rats: Influence of neuronal cholinergic and antioxidant systems. Biomedicine & Pharmacotherapy. 2018a Mar 1;99:198-204. https://doi.org/10.1016/j.biopha.2018.01.043

Odubanjo VO, Ibukun EO, Oboh G, Adefegha SA. Aqueous extracts of two tropical ethnobotanicals (*Tetrapleura tetraptera* and *Quassia undulata*) improved spatial and non-spatial working memories in scopolamine-induced amnesic rats: Influence of neuronal cholinergic and antioxidant systems. Biomedicine & Pharmacotherapy. 2018a Mar 1;99:198-204.

Odubanjo VO, Oboh G, Ibukun EO. Antioxidant and anticholinesterase activities of aqueous extract of *Uraria picta* (Jacq.) DC. African journal of pharmacy and pharmacology. 2013 Nov 8;7(41):2768-73. doi: 10.5897/AJPP2013.3899

Odubanjo VO, Oboh G, Oyeleye SI, Adefegha SA. Anticholinesterase activity and phenolic profile of two medicinal plants (*Quassia undulata* and *Senecio abyssinicus*) used in managing cognitive dysfunction in Nigeria. Journal of Food Biochemistry. 2018b Aug;42(4):e12497. https://doi.org/10.1111/jfbc.12497

Ogawa EM, Costa HB, Ventura JA, Caetano LC, Pinto FE, Oliveira BG, Barroso ME, Scherer R, Endringer DC, Romão W. Chemical profile of pineapple cv. Vitória in different maturation stages using electrospray ionization mass spectrometry. Journal of the Science of Food and Agriculture. 2018 Feb;98(3):1105-16. https://doi.org/10.1002/jsfa.8561

Ogawa T, Kodera Y, Hirata D, Blackwell TK, Mizunuma M. Natural thioallyl compounds increase oxidative stress resistance and lifespan in *Caenorhabditis elegans* by modulating SKN-1/Nrf. Scientific Reports. 2016 Feb 22;6(1):1-3.

Ogbonnia S, Van Staden J, Jager AK, Coker HA. Anticonvulsant effect of *Glyphaea brevis* (Speng) Moraches leaf extracts in mice and preliminary phytochemical tests. Nigerian Quarterly Journal of Hospital Medicine Association. 2003;13(3–4).

Ogbonnia SO, Enwuru NV, Onyemenem EU, Oyedele GA, Enwuru CA. Phytochemical evaluation and antibacterial profile of *Treculia africana* Decne bark extract on gastrointestinal bacterial pathogens. African journal of biotechnology. 2008;7(10).

Ogundipe OO, Moody JO, Odelola HA. Biological activities of *Alchornea laxiflora* extractives. In Standardization and utilization of herbal medicines: challenges of the 21st century. Proceedings of 1st International Workshop on Herbal Medicinal Products, Ibadan, Nigeria, 22-24 November, 1998: 201-208.

Ogunnusi TA, Oso BA, Dosumu OO. Isolation and antibacterial activity of triterpenes from *Euphorbia kamerunica* Pax. International Journal of Biological and Chemical Sciences. 2010;4(1). http://dx.doi.org/10.4314/ijbcs.v4i1.54241

Ogunruku OO, Oboh G, Passamonti S, Tramer F, Boligon AA. *Capsicum annuum* var. *grossum* (Bell Pepper) Inhibits β-Secretase Activity and β-Amyloid1–40 Aggregation. Journal of medicinal food. 2017 Feb 1;20(2):124-30. doi.org/10.1089/jmf.2016.0077

Ogunsuyi OB, Oboh G, Özek G, Göger F. *Solanum* vegetable-based diets improve impairments in memory, redox imbalance, and altered critical enzyme activities in *Drosophila melanogaster* model of neurodegeneration. Journal of Food Biochemistry 2020::e13150. https://doi.org/10.1111/jfbc.13150

Ogutu AI, Lilechi DB, Mutai C, Bii C. Phytochemical analysis and antimicrobial activity of *Phytolacca dodecandra*, Cucumis aculeatus and Erythrina excelsa. International journal of Biological and chemical sciences. 2012;6(2):692-704. doi: 10.4314/ijbcs.v6i2.13

Oh KB, Chang IM, Hwang KJ, Mar W. Detection of antifungal activity in *Portulaca oleracea* by a single-cell bioassay system. Phytotherapy research. 2000 Aug;14(5):329-32.

Ohadoma SC, Lawal BA. Dermatological pharmacology of aqueous and ethanol leaf extracts of *Mucuna pruriens*. European Journal of Biomedical and Pharmaceutical sciences 2019;6(3):104-6.

Ojewole J, Kamadyaapa DR, Gondwe MM, Moodley K, Musabayane CT. Cardiovascular effects of *Persea americana* Mill (Lauraceae)(avocado) aqueous leaf extract in experimental animals. Cardiovascular Journal of South Africa. 2007 Mar 1;18(2):69.

Ojewole JA. Analgesic, anti-inflammatory and hypoglycaemic effects of *Securidaca longepedunculata* (Fresen.)[Polygalaceae] root-bark aqueous extract. Inflammopharmacology. 2008 Aug 1;16(4):174-81. https://doi.org/10.1007/s10787-007-0016-7

Ojewole JA. Antinociceptive, anti-inflammatory and antidiabetic properties of *Hypoxis hemerocallidea* Fisch. & CA Mey.(Hypoxidaceae) corm [‘African Potato’] aqueous extract in mice and rats. Journal of ethnopharmacology. 2006b Jan 3;103(1):126-34.

Ojo OO, Oluyege JO, Famurewa O. Antiviral properties of two Nigerian plants. African Journal of Plant Science. 2009 Jul 31;3(7):157-9.

Ojwang LO, Banerjee N, Noratto GD, Angel-Morales G, Hachibamba T, Awika JM, Mertens-Talcott SU. Polyphenolic extracts from cowpea (*Vigna unguiculata*) protect colonic myofibroblasts (CCD18Co cells) from lipopolysaccharide (LPS)-induced inflammation–modulation of microRNA 126. Food & function. 2015;6(1):145-53.. doi: 10.1039/C4FO00459K

Okazaki K, Kawazoe K, Takaishi Y. Human platelet aggregation inhibitors from thyme (*Thymus vulgaris* L.). Phytotherapy Research. 2002 Jun;16(4):398-9. https://doi.org/10.1002/ptr.979

Okigbo RN, Omodamiro OD. Antimicrobial effect of leaf extracts of pigeon pea (*Cajanus cajan* (L.) Millsp.) on some human pathogens. Journal of herbs, spices & medicinal plants. 2007 Jan 4;12(1-2):117-27. https://doi.org/10.1300/J044v12n01_11

Okokon JE, Udoh AE, Frank SG, Udo NM. Anti-inflammatory and antipyretic activities of *Panicum maximum*. African Journal of Biomedical Research. 2011;14(2):125-30

Okoli AS, Okeke MI, Iroegbu CU, Ebo PU. Antibacterial activity of *Harungana madagascariensis* leaf extracts. Phytotherapy Research: An International Journal Devoted to Pharmacological and Toxicological Evaluation of Natural Product Derivatives. 2002 Mar;16(2):174-9.

Okoli CO, Akah PA, Onuoha NJ, Okoye TC, Nwoye AC, Nworu CS. *Acanthus montanus*: An experimental evaluation of the antimicrobial, anti-inflammatory and immunological properties of a traditional remedy for furuncles. BMC complementary and alternative medicine. 2008 Dec;8(1):27.

Okoro SO, Kawo AH, Arzai AH. Phytochemical screening, antibacterial and toxicological activities of *Acacia senegal* extracts. Bayero Journal of Pure and Applied Sciences. 2012;5(1):163-70.

Okoronkwo SO, Uchewa OO, Egwu EO, Okoronkwo AC. Evaluation of the anxiolytic activities of aqueous leaf extract of *Annona muricata* and its effect on the microanatomy of the cerebrum. Int J Biol Med Res. 2018;9(2):6366-70.

Okoye EL, Nworu CS, Ezeifeka GO, Esimone CO. Inhibition of HIV-1 lentiviral particles infectivity by *Gynostemma pentaphyllum* extracts in a viral vectorbased assay. African Journal of Biotechnology. 2012;11(7):1782-8.

Okoye TC, Akah PA, Okoli CO, Ezike AC, Mbaoji FN. Antimicrobial and antispasmodic activity of leaf extract and fractions of *Stachytarpheta cayennensis*. Asian Pacific Journal of Tropical Medicine. 2010 Mar 1;3(3):189-92. https://doi.org/10.1016/S1995-7645(10)60006-5

Okpo SO, Fatokun F, Adeyemi OO. Analgesic and anti-inflammatory activity of *Crinum glaucum* aqueous extract. Journal of ethnopharmacology. 2001 Dec 1;78(2-3):207-11. https://doi.org/10.1016/S0378-8741(01)00318-X

Ola MS, Aleisa AM, Al-Rejaie SS, Abuohashish HM, Parmar MY, Alhomida AS, Ahmed MM. Flavonoid, morin inhibits oxidative stress, inflammation and enhances neurotrophic support in the brain of streptozotocin-induced diabetic rats. Neurological sciences. 2014 Jul 1;35(7):1003-8. https://doi.org/10.1007/s10072-014-1628-5

Olajide OA, Aderogba MA, Adedapo AD, Makinde JM. Effects of *Anacardium occidentale* stem bark extract on in vivo inflammatory models. Journal of ethnopharmacology. 2004 Dec 1;95(2-3):139-42.

Olajide OA, Awe SO, Makinde JM, Ekhelar AI, Olusola A, Morebise O, Okpako DT. Studies on the anti-inflammatory, antipyretic and analgesic properties of *Alstonia boonei* stem bark. Journal of ethnopharmacology. 2000 Jul 1;71(1-2):179-86. https://doi.org/10.1016/S0378-8741(99)00200-7

Olajide OA, Makinde JM, Okpako DT, Awe SO. Studies on the anti-inflammatory and related pharmacological properties of the aqueous extract of *Bridelia ferruginea* stem bark. Journal of ethnopharmacology. 2000 Jul 1;71(1-2):153-60.

Olajide OA, Makinde JM, Okpako DT. Evaluation of the anti-inflammatory property of the extract of *Combretum micranthum* G. Don (Combretaceae). Inflammopharmacology. 2003 Sep 1;11(3):293-8. https://doi.org/10.1163/156856003322315631

Olaoluwa OO, Aiyelaagbe OO. Phytochemical investigation and antimicrobial screening of *Cardiospermun grandiflorum* (Sweet)[Sapindaceae]. International Journal of Pharma Sciences and Research. 2015;6(2):348-51.

Olawore NO Ololade, ZS. *Eucalyptus camaldulensis* var. nancy and *Eucalyptus camaldulensis* var. petford Seed Essential Oils: Phytochemicals and Therapeutic Potentials. Chem Sci J 2017 8:1. doi: 10.4172/2150-3494.1000148

Olejnik A, Kowalska K, Olkowicz M, Rychlik J, Juzwa W, Myszka K, Dembczyński R, Białas W. Anti-inflammatory effects of gastrointestinal digested *Sambucus nigra* L. fruit extract analysed in co-cultured intestinal epithelial cells and lipopolysaccharide-stimulated macrophages. Journal of Functional Foods. 2015 Dec 1;19:649-60. https://doi.org/10.1016/j.jff.2015.09.064

Olila D, Olwa-Odyek, Opuda-Asibo J. Screening extracts of *Zanthoxylum chalybeum* and *Warburgia ugandensis* for activity against measles virus (Swartz and Edmonston strains) in vitro. *Afr Health Sci*. 2002;2(1):2–10.

Oliva MD, Beltramino E, Gallucci N, Casero C, Zygadlo J, Mirta DE. Antimicrobial activity of essential oils of *Aloysia triphylla* (LHer.) Britton from different regions of Argentina. Boletín Latinoamericano y del Caribe de Plantas Medicinales y Aromáticas. 2010;9(1):29-37.

Oliván S, Martínez-Beamonte R, Calvo AC, Surra JC, Manzano R, Arnal C, Osta R, Osada J. Extra virgin olive oil intake delays the development of amyotrophic lateral sclerosis associated with reduced reticulum stress and autophagy in muscle of SOD1G93A mice. The Journal of nutritional biochemistry. 2014 Aug 1;25(8):885-92.

Oliveira DM, Melo FG, Balogun SO, Flach A, de Souza EC, de Souza GP, Rocha ID, da Costa LA, Soares IM, da Silva LI, Ascêncio SD. Antibacterial mode of action of the hydroethanolic extract of *Leonotis nepetifolia* (L.) R. Br. involves bacterial membrane perturbations. Journal of ethnopharmacology. 2015 Aug 22;172:356-63. https://doi.org/10.1016/j.jep.2015.06.027

Oliveira EM, Couto RO, Pinto MV, Martins JL, Costa EA, Conceição EC, Paula JR, Bara MT. Influence of spray-dryer operating variables on the quality of *Vernonanthura ferruginea* (Less.) H. Rob. Extracts with antiulcer potential. J Pharm Res. 2011 Oct;4:3251-5.

Oliveira FA, Vieira-Júnior GM, Chaves MH, Almeida FR, Florêncio MG, Lima Jr RC, Silva RM, Santos FA, Rao VS. Gastroprotective and anti-inflammatory effects of resin from *Protium heptaphyllum* in mice and rats. Pharmacological Research. 2004 Feb 1;49(2):105-11. https://doi.org/10.1016/j.phrs.2003.09.001

Oliveira I, Sousa A, Morais JS, Ferreira IC, Bento A, Estevinho L, Pereira JA. Chemical composition, and antioxidant and antimicrobial activities of three hazelnut (*Corylus avellana* L.) cultivars. Food Chem Toxicol. 2008 May;46(5):1801-7. doi: 10.1016/j.fct.2008.01.026

Oliveras-López MJ, Molina JJ, Mir MV, Rey EF, Martín F, de la Serrana HL. Extra virgin olive oil (EVOO) consumption and antioxidant status in healthy institutionalized elderly humans. Archives of gerontology and geriatrics. 2013 Sep 1;57(2):234-42. https://doi.org/10.1016/j.archger.2013.04.002

Oloyede GK, Ogunlade AO. Phytochemical screening, antioxidant, antimicrobial and toxicity activities of polar and non-polar extracts of *Albizia zygia* (DC) stem-bark. Annual Research & Review in Biology. 2013 Aug 9:1020-31.

Olugbuyiro JA. Inhibitory activity of *Detarium microcarpum* extract against hepatitis C virus. African Journal of Biomedical Research. 2009;12(2):149-51.

Omale S, Auta A, Amagon KI, Ighagbon MV. Anti-snake venom activity of flavonoids from the root bark extract of *Parinari curatellifolia* in Mice. IJPR. 2012: 4(2), 55-58

Omar SN, Abdullah JO, Khairoji KA, Chin SC, Hamid M. Potentials of *Melastoma malabathricum* Linn. flower and fruit extracts as antimicrobial infusions. American Journal of Plant Sciences. 2012 Aug 1;3(8):1127-34. http://dx.doi.org/10.4236/ajps.2012.38136 P

Omotoso GO, Gbadamosi IT, Afolabi TT, Abdulwahab AB, Akinlolu AA. Ameliorative effects of *Moringa* on cuprizone-induced memory decline in rat model of multiple sclerosis. *Anat Cell Biol*. 2018;51(2):119–127. doi:10.5115/acb.2018.51.2.119

Omotuyi IO, Nash O, Ajiboye BO, Olumekun VO, Oyinloye BE, Osuntokun OT, Olonisakin A, Ajayi AO, Olusanya O, Akomolafe FS, Adelakun N. *Aframomum melegueta* secondary metabolites exhibit polypharmacology against SARS-CoV-2 drug targets: in vitro validation of furin inhibition. Phytotherapy Research. 2021 Feb;35(2):908-19. doi: 10.1002/ptr.6843

Ong HG, Ling SM, Win TTM, Kang DH, Lee JH, Kim YD. Ethnomedicinal plants and traditional knowledge among three Chin indigenous groups in Natma Taung National Park (Myanmar). J Ethnopharmacol. 2018 Oct 28;225:136-158. doi: 10.1016/j.jep.2018.07.006.

Ono K, Hasegawa K, Naiki H, Yamada M. Anti-amyloidogenic activity of tannic acid and its activity to destabilize Alzheimer's β-amyloid fibrils *in vitro*. Biochimica et Biophysica Acta (BBA)-Molecular Basis of Disease. 2004 Nov 5;1690(3):193-202. https://doi.org/10.1016/j.bbadis.2004.06.008

Ono K, Nakane H, Meng Zm, Ose Y, Sakai Y, Mizuno M. Differential inhibitory effects of various herb extracts on the activities of reverse transcriptase and various deoxyribonucleic acid (DNA) polymerases. Chemical and Pharmaceutical Bulletin. 1989 Jul 25;37(7):1810-2. https://doi.org/10.1248/cpb.37.1810

Ono K, Yamada M. Antioxidant compounds have potent anti-fibrillogenic and fibril-destabilizing effects for α-synuclein fibrils in vitro. Journal of neurochemistry. 2006 Apr;97(1):105-15. https://doi.org/10.1111/j.1471-4159.2006.03707.x

Onofre SB, Herkert PF. Antimicrobial activity of extracts obtained from *Urera baccifera* (L.) Gaudich. Advances in Life Sciences. 2012;2(5):139-43. doi: 10.5923/j.als.20120205.03

Onoja SO, Omeh YN, Ezeja MI, Chukwu MN. Evaluation of the in vitro and in vivo antioxidant potentials of *Aframomum melegueta* methanolic seed extract. Journal of tropical medicine. 2014;2014. Article ID 159343 https://doi.org/10.1155/2014/159343

Onwukaeme ND. Anti-inflammatory activities of flavonoids of *Baphia nitida* Lodd. (Leguminosae) on mice and rats. Journal of ethnopharmacology. 1995 May 1;46(2):121-4.

Onyeagba RA, Ugbogu OC, Okeke CU, Iroakasi O. Studies on the antimicrobial effects of garlic (*Allium sativum* Linn), ginger (*Zingiber officinale* Roscoe) and lime (*Citrus aurantifolia* Linn). African Journal of Biotechnology. 2004;3(10):552-4. doi: 10.5897/AJB2004.000-2108

Onyenekwe PC, Okereke OE, Owolewa SO. Phytochemical screening and effect of *Musa paradisiaca* stem extrude on rat haematological parameters. Current Research Journal of Biological Sciences. 2013;5(1):26-9.

Onyeto CA. Anti-plasmodial and antioxidant activities of methanol extract of the fresh leaf of *Lophira lanceolata* (Ochnaceae). African Journal of Biotechnology. 2014;13(16). http://dx.doi.org/10.5897/AJB2014.13707

Oonmetta-aree J, Suzuki T, Gasaluck P, Eumkeb G. Antimicrobial properties and action of galangal (*Alpinia galanga* Linn.) on *Staphylococcus aureus*. LWT-Food Science and Technology. 2006 Dec 1;39(10):1214-20. https://doi.org/10.1016/j.lwt.2005.06.015

Oppermann C, Engel N, Ruth W, Nebe B, Kragl U. LC-MS analysis of extracts from beech and sea buckthorn to correlate the phytoestrogen content and anti-cancer activity. Journal of Chemical and Pharmaceutical Research. 2015;7(5):175-85.

Ordesi P, Pisoni L, Nannei P, Macchi M, Borloni R, Siervo S. Therapeutic efficacy of bromelain in impacted third molar surgery: a randomized controlled clinical study. Quintessence Int. 2014 Sep;45(8):679-84. doi: 10.3290/j.qi.a32237.

Ordoñez AA, Gomez JD, Cudmani NM, Vattuone MA, Isla MI. Antimicrobial activity of nine extracts of *Sechium edule* (Jacq.) Swartz. Microbial ecology in health and disease. 2003 Jan 1;15(1):33-9. https://doi.org/10.1080/0891060010015583

Orech FO, Akenga T, Ochora J, Friis H, Aagaard-Hansen J. Potential toxicity of some traditional leafy vegetables consumed in Nyang'oma Division, Western Kenya. African Journal of Food, Agriculture, Nutrition and Development. 2005;5(1).

Orhan DD, Orhan N, Ozcelik B, Ergun F. Biological activities of *Vitis vinifera* L. leaves. Turk J Biol. 2009 Jan 1;33(1):341-8. doi:10.3906/biy-0806-17

Orhan İE, ÖZÇELİK B, Kartal M, Kan Y. Antimicrobial and antiviral effects of essential oils from selected Umbelliferae and Labiatae plants and individual essential oil components. Turkish Journal of Biology. 2012 Apr 25;36(3):239-46. doi:10.3906/biy-0912-30

Orhan IE, Üstün O, Şener B. Estimation of cholinesterase inhibitory and antioxidant effects of the leaf extracts of Anatolian *Ficus carica* var. *domestica* and their total phenol and flavonoid contents. Natural product communications. 2011 Mar;6(3):1934578X1100600315. https://doi.org/10.1177/1934578X1100600315

Ortuño A, Báidez A, Gómez P, Arcas MC, Porras I, García-Lidón A, Del Río JA. *Citrus paradisi* and *Citrus sinensis* flavonoids: Their influence in the defence mechanism against Penicillium digitatum. Food Chemistry. 2006 Jan 1;98(2):351-8. https://doi.org/10.1016/j.foodchem.2005.06.017

Orzechowska B, Chaber R, Wiśniewska A, Pajtasz-Piasecka E, Jatczak B, Siemieniec I, Gulanowski B, Chybicka A, Błach-Olszewska Z. Baicalin from the extract of *Scutellaria baicalensis* affects the innate immunity and apoptosis in leukocytes of children with acute lymphocytic leukemia. International immunopharmacology. 2014 Dec 1;23(2):558-67. https://doi.org/10.1016/j.intimp.2014.10.005

Osman MA. Chemical and nutrient analysis of baobab (*Adansonia digitata*) fruit and seed protein solubility. Plant Foods Hum Nutr. 2004 Winter;59(1):29-33. doi: 10.1007/s11130-004-0034-1.

Ospina LM, Muñoz PB, Matulevich J, Teherán AA, Villamizar LB. Composition and antimicrobial activity of the essential oils of three plant species from the Sabana of Bogota (Colombia): *Myrcianthes leucoxyla*, *Vallea stipularis* and *Phyllanthus salviifolius*. Natural product communications. 2016 Dec;11(12):1934578X1601101234.

Ossoukhova A, Owen L, Savage K, Meyer M, Ibarra A, Roller M, Pipingas A, Wesnes K, Scholey A. Improved working memory performance following administration of a single dose of American ginseng (*Panax quinquefolius* L.) to healthy middle-age adults. Human Psychopharmacology: Clinical and Experimental. 2015 Mar;30(2):108-22. doi: 10.1002/hup.2463.

Ota A, Višnjevec AM, Vidrih R, Prgomet Ž, Nečemer M, Hribar J, Cimerman NG, Možina SS, Bučar-Miklavčič M, Ulrih NP. Nutritional, antioxidative, and antimicrobial analysis of the Mediterranean hackberry (*Celtis australis* L.). Food science & nutrition. 2017 Jan;5(1):160-70. https://doi.org/10.1002/fsn3.375

Otero JS, Hirsch GE, Klafke JZ, Porto FG, de Almeida AS, Nascimento S, Schmidt A, da Silva B, Pereira RL, Jaskulski M, Parisi MM. Inhibitory effect of *Campomanesia xanthocarpa* in platelet aggregation: Comparison and synergism with acetylsalicylic acid. Thrombosis research. 2017 Jun 1;154:42-9. https://doi.org/10.1016/j.thromres.2017.03.020

Otero R, Núñez V, Jiménez SL, Fonnegra R, Osorio RG, Garcıa ME, Dıaz A. Snakebites and ethnobotany in the northwest region of Colombia: Part II: neutralization of lethal and enzymatic effects of *Bothrops atrox* venom. Journal of Ethnopharmacology. 2000 Aug 1;71(3):505-11. https://doi.org/10.1016/S0378-8741(99)00197-X

Otimenyin SO, Uguru MO, Atang BL. Antiinflammatory and analgesic activities of *Ficus thonningii* and *Pseudocedrela kotschyi* extracts. Nigerian Journal of Pharmaceutical Research. 2004;3(1):82-5.

Oumarou MR, Zingue S, Bakam BY, Ateba SB, Foyet SH, Mbakop FT, Njamen D. *Lannea acida* A. Rich. Evidence-Based Complementary and Alternative Medicine. 2017 Jan 1;2017. https://doi.org/10.1155/2017/7829059

Oumer A, Bisrat D, Mazumder A, Asres K. A new antimicrobial anthrone from the leaf latex of *Aloe trichosantha*. Natural product communications. 2014 Jul;9(7):1934578X1400900717. https://doi.org/10.1177/1934578X1400900717

Ouoba MA, Koudou J, Some N, Ouedraogo S, Guissou IP. Wound healing and antibacterial properties of leaf essential oil of *Vitex simplicifolia* oliv. from Burkina Faso. Chromatography and its applications. 2012 Mar 16:109-18.

Ovodova RG, Golovchenko VV, Shashkov AS, Popov SV, Ovodov YS. Structural studies and physiological activity of lemnan, a pectin from *Lemna minor* L. Russian Journal of Bioorganic Chemistry. 2000 Nov 1;26(10): 669-76. doi.org/10.1007/BF02821835

Owoeye O, Salami OA. Monosodium glutamate toxicity: *Sida acuta* leaf extract ameliorated brain histological alterations, biochemical and haematological changes in wistar rats. African Journal of Biomedical Research. 2017;20(2):173-82.

Owolabi OJ, Nworgu Z. Antiinflammatory an anti-nociceptive ativities of *Costus lucanuscianus* (Costaceae). Pharmacologyonline 2009:1: 1230-1238.

Owolabi OJ, Omogbai EK. Analgesic and anti-inflammatory activities of the ethanolic stem bark extract of *Kigelia africana* (Bignoniaceae). African Journal of Biotechnology. 2007 Mar 5;6(5):582-5. https://doi.org/10.5897/AJB2007.000-2051

Owuor BO, Kisangau DP. Kenyan medicinal plants used as antivenin: a comparison of plant usage. Journal of ethnobiology and ethnomedicine. 2006 Dec;2(1):7.

Oyebanji BO, Saba AB, Oridupa OA. Anti-inflammatory and analgesic effects of methanol extract of *Stellaria media* (L.) Vill leaf. African Journal of Biomedical Research. 2012;15(1):29-34.

Oyedeji O, Oziegbe M, Taiwo FO. Antibacterial, antifungal and phytochemical analysis of crude extracts from the leaves of *Ludwigia abyssinica* A. Rich. and *Ludwigia decurrens* Walter. Journal of Medicinal Plants Research. 2011 Apr 4;5(7):1192-9. https://doi.org/10.5897/JMPR.9000134

Oyedeji OA, Afolayan AJ. Chemical composition and antibacterial activity of the essential oil of *Centella asiatica*. Growing in South Africa. Pharmaceutical biology. 2005 Jan 1;43(3):249-52. https://doi.org/10.1080/13880200590928843

Ozaki Y, Kawahara N, Harada M. Anti-inflammatory effect of *Zingiber cassumunar* Roxb. and its active principles. Chemical and Pharmaceutical Bulletin. 1991 Sep 25;39(9):2353-6. https://doi.org/10.1248/cpb.39.2353

Ozaki Y, Rui J, Tang YT. Antiinflammatory effect of *Forsythia suspensa* VAHL and its active principle. Biological and Pharmaceutical Bulletin. 2000 Mar 1;23(3):365-7. https://doi.org/10.1248/bpb.23.365

Ozaki Y, Soedigdo S, Wattimena YR, Suganda AG. Antiinflammatory effect of mace, aril of *Myristica fragrans* Houtt., and its active principles. The Japanese Journal of Pharmacology. 1989;49(2):155-63. https://doi.org/10.1254/jjp.49.155

Ozarowski M, Mikolajczak PL, Bogacz A, Gryszczynska A, Kujawska M, Jodynis-Liebert J, Piasecka A, Napieczynska H, Szulc M, Kujawski R, Bartkowiak-Wieczorek J. *Rosmarinus officinalis* L. leaf extract improves memory impairment and affects acetylcholinesterase and butyrylcholinesterase activities in rat brain. Fitoterapia. 2013 Dec 1;91:261-71. https://doi.org/10.1016/j.fitote.2013.09.012

Pacheco P, Sierra J, Schmeda-Hirschmann G, Potter CW, Jones BM, Moshref M. Antiviral activity of Chilean medicinal plant extracts. Phytotherapy research. 1993 Nov;7(6):415-8.

Padalia H, Rathod T, Chanda S. Evaluation of antimicrobial potential of different solvent extracts of some medicinal plants of semi-arid region. Asian Journal of Pharmaceutical and Clinical Research, 2017;10(11):295-9. doi: 10.22159/ajpcr.2017.v10i11.17662

Padhi L, Panda SK. Antibacterial activity of *Eleutherine bulbosa* against multidrug-resistant bacteria. Journal of Acute Medicine. 2015 Sep 1;5(3):53-61.https://doi.org/10.1016/j.jacme.2015.05.004

Padhi LP, Panda SK, Satapathy SN, Dutta SK. In vitro evaluation of antibacterial potential of *Annona squamosa* L. and *Annona reticulata* L. from Similipal Biosphere Reserve, Orissa, India. Journal of agricultural Technology. 2011;7(1):133-42.

Padil VV, Senan C, Černík M. Dodecenylsuccinic anhydride derivatives of gum karaya (Sterculia urens): Preparation, characterization, and their antibacterial properties. Journal of agricultural and food chemistry. 2015 Apr 15;63(14):3757-65. https://doi.org/10.1021/jf505783e

Padilha MM, Vilela FC, Rocha CQ, Dias MJ, Soncini R, dos Santos MH, Alves-da-Silva G, Giusti-Paiva A. Antiinflammatory properties of *Morus nigra* leaves. Phytotherapy Research. 2010 Oct;24(10):1496-500. https://doi.org/10.1002/ptr.3134

Padumadasa, C., Dharmadana, D., Abeysekera, A., & Thammitiyagodage, M. (2016). In vitro antioxidant, anti-inflammatory and anticancer activities of ethyl acetate soluble proanthocyanidins of the inflorescence of Cocos nucifera L. *BMC complementary and alternative medicine*, *16*(1), 345. doi:10.1186/s12906-016-1335-2

Pahaye DB, Bum EN, Taïwé GS, et al. Neuroprotective and Antiamnesic Effects of *Mitragyna inermis* Willd (Rubiaceae) on Scopolamine-Induced Memory Impairment in Mice. *Behav Neurol*. 2017;2017:5952897. doi:10.1155/2017/5952897

Paiva LA, de Alencar Cunha KM, Santos FA, Gramosa NV, Silveira ER, Rao VS. Investigation on the wound healing activity of oleo-resin from *Copaifera langsdorffi* in rats. Phytotherapy Research: An International Journal Devoted to Pharmacological and Toxicological Evaluation of Natural Product Derivatives. 2002a Dec;16(8):737-9. https://doi.org/10.1002/ptr.1049

Paiva LA, Gurgel LA, Silva RM, Tomé AR, Gramosa NV, Silveira ER, Santos FA, Rao VS. Anti-inflammatory effect of kaurenoic acid, a diterpene from *Copaifera langsdorffii* on acetic acid-induced colitis in rats. Vascular pharmacology. 2002b Dec 1;39(6):303-7. https://doi.org/10.1016/S1537-1891(03)00028-4

Pakdeechote P, Kukongviriyapan U, Berkban W, Prachaney P, Kukongviriyapan V, Nakmareong S. *Mentha cordifolia* extract inhibits the development of hypertension in L-NAME-induced hypertensive rats. J. Med. Plants Res. 2011 Apr 4;5:1175-83.

Pakdeepak K, Chokchaisiri R, Tocharus J, Jearjaroen P, Tocharus C, Suksamrarn A. 5,6,7,4'-Tetramethoxyflavanone protects against neuronal degeneration induced by dexamethasone by attenuating amyloidogenesis in mice. EXCLI J. 2020;19:16-32. doi:10.17179/excli2019-1940

Palanichamy S, Nagarajan S. Anti-inflammatory activity of *Cassia alata* leaf extract and kaempferol 3-O-sophoroside. Fitoterapia. 1990;61(1):44-7.

Palazzi L, Bruzzone E, Bisello G, Leri M, Stefani M, Bucciantini M, de Laureto PP. Oleuropein aglycone stabilizes the monomeric α-synuclein and favours the growth of non-toxic aggregates. Scientific reports. 2018 May 29;8(1):1-7.

Palombo EA, Semple SJ. Antibacterial activity of traditional Australian medicinal plants. Journal of ethnopharmacology. 2001 Oct 1;77(2-3):151-7. https://doi.org/10.1016/S0378-8741(01)00290-2

Pan M, Li Z, Yeung V, Xu RJ. Dietary supplementation of soy germ phytoestrogens or estradiol improves spatial memory performance and increases gene expression of BDNF, TrkB receptor and synaptic factors in ovariectomized rats. Nutrition & Metabolism. 2010 Dec;7(1):1-1. https://doi.org/10.1186/1743-7075-7-75

Panahi Y, Beiraghdar F, Akbari H, Bekhradi H, Taghizadeh M, Sahebkar A. A herbal cream consisting of *Aloe vera*, *Lavandula stoechas*, and *Pelargonium roseum* as an alternative for silver sulfadiazine in burn management. Asian Biomedicine. 2012 Apr 1;6(2):273-8. doi:  10.5372/1905-7415.0602.053

Panda BB, Gaur K, Kori ML, Tyagi LK, Nema RK, Sharma CS, Jain AK. Anti-inflammatory and analgesic activity of *Jatropha gossypifolia* in experimental animal models. Global Journal of Pharmacology. 2009;3(1):1-5.

Panda SK, Thatoi HN, Dutta SK. Antibacterial activity and phytochemical screening of leaf and bark extracts of *Vitex negundo* l. from similipal biosphere reserve, Orissa. Journal of medicinal plants research. 2009 Apr 30;3(4):294-300.https://doi.org/10.5897/JMPR.9000120

Pandey G, Khatoon S, Pandey MM, Rawat AK. Altitudinal variation of berberine, total phenolics and flavonoid content in *Thalictrum foliolosum* and their correlation with antimicrobial and antioxidant activities. Journal of Ayurveda and integrative medicine. 2018 Jul 1;9(3):169-76. https://doi.org/10.1016/j.jaim.2017.02.010

Pandey K, Sharma PK, Dudhe RU. Antioxidant and anti-inflammatory activity of ethanolic extract of *Parthenium hysterophorus* Linn. Asian Journal of Pharmaceutical and Clinical Research. 2012;5(4):28-31.

Pandey R, Gupta S, Shukla V, Tandon S, Shukla V. Antiaging, antistress and ROS scavenging activity of crude extract of *Ocimum sanctum* (L.) in *Caenorhabditis elegans* (Maupas, 1900). Ind J Exp Biol. 2013; 51:515-521.

Pandey S, Tiwari S, Kumar A, Niranjan A, Chand J, Lehri A, Chauhan PS. Antioxidant and anti-aging potential of Juniper berry (*Juniperus communis* L.) essential oil in *Caenorhabditis elegans* model system. Industrial Crops and Products. 2018 Sep 15;120:113-22.

Pandey S, Toppo E, Chauhan P. Comparative study of antitoxin activity of *Calotropis gigentea* Linn and *Cassia fistula* Linn against Naja-naja (cobra) venom. International Journal of Green Pharmacy (IJGP). 2011;5(4).

Pandit C, Sai Latha S, Usha Rani T, Anilakumar KR. Pepper and cinnamon improve cold induced cognitive impairment via increasing non-shivering thermogenesis; a study. International Journal of Hyperthermia. 2018 Dec 31;35(1):518-27. https://doi.org/10.1080/02656736.2018.1511835

Pang DJ, Huang C, Chen ML, Chen YL, Fu YP, Paulsen BS, Rise F, Zhang BZ, Chen ZL, Jia RY, Li LX, Song X, Feng B, Ni XQ, Yin ZQ, Zou YF. Characterization of Inulin-Type Fructan from *Platycodon grandiflorus* and Study on Its Prebiotic and Immunomodulating Activity. Molecules. 2019 Mar 27;24(7):1199. doi: 10.3390/molecules24071199.

Panni MK, Bakht J. In vitro antibacterial activity and phyto-chemistry of samples from the roots of *Viola pilosa*. Pakistan journal of pharmaceutical sciences. 2018 Nov 1;31(6).

Paran E, Novack V, Engelhard YN, Hazan-Halevy I. The effects of natural antioxidants from tomato extract in treated but uncontrolled hypertensive patients. Cardiovascular drugs and therapy. 2009 Apr 1;23(2):145-51. doi:  10.1007/s10557-008-6155-2

Parekh J, Chanda S. In-vitro antimicrobial activities of extracts of *Launaea procumbens* roxb.(Labiateae), *Vitis vinifera* l.(Vitaceae) and *Cyperus rotundus* l.(Cyperaceae. African Journal of Biomedical Research. 2006;9(2).

Parimala K, Cheriyan BV, Viswanathan S. Antinociceptive and anti-inflammatory activity of Petroleum-ether extract of *Eupatorium triplinerve* vahl. J Pharm Sci. 2012;2(3):12-8.

Park E, Ryu MJ, Kim NK, Bae MH, Seo Y, Kim J, Yeo S, Kanwal M, Choi CW, Heo JY, Jeong SY. Synergistic Neuroprotective Effect of *Schisandra chinensis* and *Ribes fasciculatum* on Neuronal Cell Death and Scopolamine-Induced Cognitive Impairment in Rats. International journal of molecular sciences. 2019 Jan;20(18):4517. doi:10.3390/ijms20184517

Park EH, Kahng JH, Lee SH, Shin KH. An anti-inflammatory principle from cactus. Fitoterapia. 2001 Mar 1;72(3):288-90. doi.org/10.1016/S0367-326X(00)00287-2

Park EH, Kahng JH, Paek EA. Studies on the pharmacological actions of cactus: identification of its anti-inflammatory effect. Archives of pharmacal research. 1998 Feb 1;21(1):30-4. doi.org/10.1007/BF03216749

Park EY, Jeon H. Antioxidant and Anti-inflammatory Activities of *Equisetum hyemale*. Natural Product Sciences. 2008;14(4):239-43.

Park HJ, Song M. Leaves of *Raphanus sativus* L. shows anti-inflammatory activity in LPS-stimulated macrophages via suppression of COX-2 and iNOS expression. Preventive nutrition and food science. 2017 Mar;22(1):50.

Park KM, Choo JH, Sohn JH, Lee SH, Hwang JK. Antibacterial activity of panduratin A isolated from *Kaempferia pandurata* against *Porphyromonas gingivalis*. Food Science and Biotechnology. 2005;14(2):286-9.

Park SJ, Ahn YJ, Lee HE, Hong E, Ryu JH. Standardized *Prunella vulgaris* var. *lilacina* extract enhances cognitive performance in normal naive mice. Phytotherapy research. 2015 Nov;29(11):1814-21. https://doi.org/10.1002/ptr.5449

Park SK, Jin DE, Park CH, Seung TW, Guo TJ, Song JW, Kim JH, Kim DO, Heo HJ. Ameliorating effects of ethyl acetate fraction from onion (*Allium cepa* L.) flesh and peel in mice following trimethyltin-induced learning and memory impairment. Food Research International. 2015 Sep 1;75:53-60.

Parkavi V, Vignesh M, Selvakumar K, Muthu JM, Joysa JR. Antibacterial activity of aerial parts of *Imperata cylindrica* (L) Beauv. Int J Pharm Sci Drug Res. 2012;4(3):209-12.

Parle M, Dhingra D, Kulkarni SK. Improvement of mouse memory by *Myristica fragrans* seeds. Journal of medicinal food. 2004 Jun 1;7(2):157-61. https://doi.org/10.1089/1096620041224193

Parmar HS, Kar A. Protective role of *Citrus sinensis, Musa paradisiaca*, and *Punica granatum* peels against diet-induced atherosclerosis and thyroid dysfunctions in rats. Nutrition Research. 2007 Nov 1;27(11):710-8.

Parry O, Okwuasaba FK, Ekpenyong KI, Ashraf CM. Effects of *Olax gambecola* methanol extract on smooth muscle and rat blood pressure. Journal of ethnopharmacology. 1986 Oct 1;18(1):63-88. https://doi.org/10.1016/0378-8741(86)90044-9

Parsania M, Rezaee MB, Hamidreza Monavari S, Jaimand K, Milad Mousavi-Jazayeri S, Razazian M, Nadjarha MH. Antiviral screening of four plant extracts against acyclovir resistant *Herpes simplex* virus type-1. Pakistan journal of pharmaceutical sciences. 2017 Jul 2;30.

Parvez MK, Al-Dosari MS, Arbab AH, Niyazi S. The in vitro and in vivo anti-hepatotoxic, anti-hepatitis B virus and hepatic CYP450 modulating potential of *Cyperus rotundus*. Saudi Pharmaceutical Journal. 2019 May 1;27(4):558-64. https://doi.org/10.1016/j.jsps.2019.02.003

Parvin MS, Das N, Jahan N, Akhter MA, Nahar L, Islam ME. Evaluation of in vitro anti-inflammatory and antibacterial potential of *Crescentia cujete* leaves and stem bark. BMC research notes. 2015 Dec 1;8(1):412. https://doi.org/10.1186/s13104-015-1384-5

Parvu AE, Parvu M, Vlase L, Miclea P, Mot AC, Silaghi-Dumitrescu R. Anti-inflammatory effects of *Allium schoenoprasum* L. leaves. J Physiol Pharmacol. 2014 Apr 1;65(2):309-15.

Pasban-Aliabadi H, Esmaeili-Mahani S, Sheibani V, Abbasnejad M, Mehdizadeh A, Yaghoobi MM. Inhibition of 6-hydroxydopamine-induced PC12 cell apoptosis by olive (*Olea europaea* L.) leaf extract is performed by its main component oleuropein. Rejuvenation research. 2013 Apr 1;16(2):134-42. https://doi.org/10.1089/rej.2012.1384

Passos GF, Medeiros R, Marcon R, Nascimento AF, Calixto JB, Pianowski LF. The role of PKC/ERK1/2 signaling in the anti-inflammatory effect of tetracyclic triterpene euphol on TPA-induced skin inflammation in mice. European journal of pharmacology. 2013 Jan 5;698(1-3):413-20. https://doi.org/10.1016/j.ejphar.2012.10.019

Patel NK, Bhutani KK. Pinostrobin and Cajanus lactone isolated from *Cajanus cajan* (L.) leaves inhibits TNF-α and IL-1β production: In vitro and in vivo experimentation. Phytomedicine. 2014 Jun 15;21(7):946-53. doi.org/10.1016/j.phymed.2014.02.011

Patel P, Julien JP, Kriz J. Early-stage treatment with Withaferin A reduces levels of misfolded superoxide dismutase 1 and extends lifespan in a mouse model of amyotrophic lateral sclerosis. *Neurotherapeutics*. 2015;12(1):217–233. doi:10.1007/s13311-014-0311-0

Patel RP. A study of anti-inflammatory activity of methanolic fraction of aerial parts of *Corchorus aestuans* Limm. International Research Journal of Pharmacy 2011; 2(5): 198-200.

Patel SS, Ray RS, Sharma A, Mehta V, Katyal A, Udayabanu M. Antidepressant and anxiolytic like effects of *Urtica dioica* leaves in streptozotocin induced diabetic mice. Metabolic brain disease. 2018 Aug 1;33(4):1281-92. doi: 10.1007/s11011-018-0243-1.

Patil V V, Pimprikar R B, Patil V R. Pharmacognostical studies and evaluation of anti-inflammatory activity of *Ficus bengalensis* linn. J Young Pharmacists 2009;1:49-53 doi: 10.4103/0975-1483.51879

Patro G, Bhattamisra SK, Mohanty BK. Effects of *Mimosa pudica* L. leaves extract on anxiety, depression and memory. Avicenna journal of phytomedicine. 2016 Nov;6(6):696-710.

Pattanaik C, Reddy CS, Murthy MS. An ethnobotanical survey of medicinal plants used by the Didayi tribe of Malkangiri district of Orissa, India. Fitoterapia. 2008 Jan;79(1):67-71. doi: 10.1016/j.fitote.2007.07.015. Epub 2007 Aug 11.

Paula-Ju WD, Rocha FH, Donatti L, Fadel-Picheth CM, Weffort-Santos AM. Leishmanicidal, antibacterial, and antioxidant activities of *Caryocar brasiliense* Cambess leaves hydroethanolic extract. Revista Brasileira de Farmacognosia. 2006 Dec;16:625-30. https://doi.org/10.1590/S0102-695X2006000500007

Pavithra GM, Siddiqua S, Naik AS, Vinayaka KS. Antioxidant and antimicrobial activity of flowers of *Wendlandia thyrsoidea*, *Olea dioica*, *Lagerstroemia speciosa* and *Bombax malabaricum*. Journal of Applied Pharmaceutical Science. 2013 Jun 1;3(6):114.

Pavithra PS, Sreevidya N, Verma RS. Antibacterial and antioxidant activity of methanol extract of *Evolvulus nummularius*. Indian J Pharmacol. 2009 Oct;41(5):233-6. doi: 10.4103/0253-7613.58514

Pawera L, Verner V, Termote C, Sodombekov I, Kandakov A, Karabaev N, Skalicky M, Polesny Z. Medical ethnobotany of herbal practitioners in the Turkestan Range, southwestern Kyrgyzstan. Acta Societatis Botanicorum Poloniae. 2016;85(1). DOI: 10.5586/asbp.3483

Pedrollo CT, Kinupp VF, Shepard Jr G, Heinrich M. Medicinal plants at Rio Jauaperi, Brazilian Amazon: ethnobotanical survey and environmental conservation. Journal of ethnopharmacology. 2016 Jun 20;186:111-24.

Pękala-Safińska A, Tkachenko H, Osadowski Z, Buyun L, Honcharenko V, Prokopiv A. The antibacterial activity of the ethanolic leaf extract of *Ficus vasta* Forssk. (Moraceae) against *Aeromonas* spp. Strains. Scientific and technical bulletin of the Institute of Animal Husbandry of NAAS. 2019 (121): 33-44.

Peksel A, Arisan-Atac IN, Yanardag R. Evaluation of antioxidant and antiacetylcholinesterase activities of the extracts of *Pistacia atlantica* Desf. Leaves. Journal of food biochemistry. 2010 Jun;34(3):451-76. https://doi.org/10.1111/j.1745-4514.2009.00290.x

Peksel A, Imamoglu S, Altas Kiymaz N, Orhan N. Antioxidant and radical scavenging activities of *Asphodelus aestivus* Brot. extracts. International journal of food properties. 2013 Aug 18;16(6):1339-50.

Pellegrini MC, Alvarez MV, Ponce AG, Cugnata NM, De Piano FG, Fuselli SR. Anti-quorum sensing and antimicrobial activity of aromatic species from South America. Journal of Essential Oil Research. 2014 Nov 2;26(6):458-65. doi.org/10.1080/10412905.2014.947387

Peluso G, De Feo V, De Simone F, Bresciano E, Vuotto ML. Studies on the inhibitory effects of caffeoylquinic acids on monocyte migration and superoxide ion production. Journal of natural products. 1995 May;58(5):639-46. https://doi.org/10.1021/np50119a001

Pendota SC, Aderogba MA, Van Staden J. In vitro antimicrobial activity of extracts and an isolated compound from *Boscia albitrunca* leaves. South African Journal of Botany. 2015 Jan 1;96:91-3. https://doi.org/10.1016/j.sajb.2014.11.005

Penecilla GL, Magno CP. Antibacterial activity of extracts of twelve common medicinal plants from the Philippines. Journal of Medicinal Plants Research. 2011 Aug 18;5(16):3975-81.

Peng Y, Hu Y, Xu S, Li P, Li J, Lu L, Yang H, Feng N, Wang L, Wang X. L-3-n-butylphthalide reduces tau phosphorylation and improves cognitive deficits in AβPP/PS1-Alzheimer's transgenic mice. Journal of Alzheimer's Disease. 2012 Jan 1;29(2):379-91. doi: 10.3233/JAD-2011-111577

Peng Y, Sun J, Hon S, Nylander AN, Xia W, Feng Y, Wang X, Lemere CA. L-3-n-butylphthalide improves cognitive impairment and reduces amyloid-β in a transgenic model of Alzheimer's disease. Journal of Neuroscience. 2010 Jun 16;30(24):8180-9. https://doi.org/10.1523/JNEUROSCI.0340-10.2010

Peni IJ, Elinge CM, Yusuf H, Itodo AU, Agaie BM, Mbongo AN, Chogo E. Phytochemical screening and antibacterial activity of *Parinari curatellifolia* stem extract. Journal of Medicinal Plants Research. 2010 Oct 18;4(20):2099-102.

Penido AB, De Morais SM, Ribeiro AB, Alves DR, Rodrigues AL, dos Santos LH, de Menezes JE. Medicinal plants from northeastern Brazil against Alzheimer's disease. Evidence-Based Complementary and Alternative Medicine. 2017;2017. https://doi.org/10.1155/2017/1753673

Penido C, Conte FP, Chagas MS, Rodrigues CA, Pereira JF, Henriques MG. Antiinflammatory effects of natural tetranortriterpenoids isolated from *Carapa guianensis* Aublet on zymosan-induced arthritis in mice. Inflammation Research. 2006 Nov 1;55(11):457-64. doi.org/10.1007/s00011-006-5161-8

Pepe A, Frey ME, Munoz F, Fernández MB, Pedraza A, Galbán G, García DN, Daleo GR, Guevara MG. Fibrin (ogen) olytic and antiplatelet activities of a subtilisin-like protease from *Solanum tuberosum* (StSBTc-3). Biochimie. 2016 Jun 1;125:163-70.

Pepe G, Sommella E, Cianciarulo D, et al. Polyphenolic Extract from Tarocco (*Citrus sinensis* L. Osbeck) Clone "Lempso" Exerts Anti-Inflammatory and Antioxidant Effects via NF-kB and Nrf-2 Activation in Murine Macrophages. *Nutrients*. 2018;10(12):1961. Published 2018 Dec 11. doi:10.3390/nu10121961

Pereira AP, Ferreira IC, Marcelino F, Valentão P, Andrade PB, Seabra R, Estevinho L, Bento A, Pereira JA. Phenolic compounds and antimicrobial activity of olive (*Olea europaea* L. Cv. Cobrançosa) leaves. Molecules. 2007 May;12(5):1153-62. https://doi.org/10.3390/12051153

Pereira EM, Gomes RT, Freire NR, Aguiar EG, Brandão MD, Santos VR. In vitro antimicrobial activity of Brazilian medicinal plant extracts against pathogenic microorganisms of interest to dentistry. Planta medica. 2011 Mar;77(04):401-4. doi: 10.1055/s-0030-1250354

Pereira R, Pereira AL, Ferreira MM, Fontenelle RO, Saker-Sampaio S, Santos HS, Bandeira PN, Vasconcelos MA, Queiroz JA, Braz-Filho R, Teixeira EH. Evaluation of the antimicrobial and antioxidant activity of 7-hydroxy-4', 6-dimethoxy-isoflavone and essential oil from *Myroxylon peruiferum* Lf. Anais da Academia Brasileira de Ciências. 2019;91(2). doi.org/10.1590/0001-3765201920180204

Pereira WS, da Silva GP, Vigliano MV, Leal NR, Pinto FA, Fernandes DC, Santos SV, Martino T, Nascimento JR, de Azevedo AP, Fonseca EN. Anti-arthritic properties of crude extract from *Chenopodium ambrosioides* L. leaves. Journal of Pharmacy and Pharmacology. 2018 Aug;70(8):1078-91. doi.org/10.1111/jphp.12926

Perera HD, Samarasekera JK, Handunnetti SM, Weerasena OV. In vitro anti-inflammatory and anti-oxidant activities of Sri Lankan medicinal plants. Industrial Crops and Products. 2016 Dec 30;94:610-20. https://doi.org/10.1016/j.indcrop.2016.09.009

Pérez G. Anti-inflammatory activity of *Ambrosia artemisiaefolia* and *Rhoeo spathacea*. Phytomedicine. 1996;3(2):163-7.

Perez YY, Jimenez-Ferrer E, Alonso D, Botello-Amaro CA, Zamilpa A. *Citrus limetta* leaves extract antagonizes the hypertensive effect of angiotensin II. Journal of ethnopharmacology. 2010 Apr 21;128(3):611-4. https://doi.org/10.1016/j.jep.2010.01.059

Perianayagam JB, Sharma SK, Pillai KK, Pandurangan A, Kesavan D. Evaluation of antimicrobial activity of ethanol extract and compounds isolated from *Trichodesma indicum* (Linn.) R. Br. root. Journal of ethnopharmacology. 2012 Jun 26;142(1):283-6. https://doi.org/10.1016/j.jep.2012.04.020

Perry LM, Metzger J. Medicinal plants of east and southeast Asia: attributed properties and uses. MIT press; 1980.

Perry NS, Bollen C, Perry EK, Ballard C. Salvia for dementia therapy: review of pharmacological activity and pilot tolerability clinical trial. Pharmacology biochemistry and behavior. 2003 Jun 1;75(3):651-9.

Perry NS, Menzies R, Hodgson F, Wedgewood P, Howes MJ, Brooker HJ, Wesnes KA, Perry EK. A randomised double-blind placebo-controlled pilot trial of a combined extract of sage, rosemary and melissa, traditional herbal medicines, on the enhancement of memory in normal healthy subjects, including influence of age. Phytomedicine. 2018 Jan 15;39:42-8.

Perveen T, Hashmi BM, Haider S, Tabassum S, Saleem S, Siddiqui MA. Role of monoaminergic system in the etiology of olive oil induced antidepressant and anxiolytic effects in rats. International Scholarly Research Notices. 2013;2013. http://dx.doi.org/10.1155/2013/615685

Petersen G, Lorkowski G, Kasper FR, Gottwald R, Lücker PW. Anti-inflammatory activity of a pyrrolizidine alkaloid-free extract of roots of *Symphytum officinale* in humans. Planta Medica. 1993 Dec;59(S 1):A703-4.

Peterson JJ, Beecher GR, Bhagwat SA, Dwyer JT, Gebhardt SE, Haytowitz DB, Holden JM. Flavanones in grapefruit, lemons, and limes: A compilation and review of the data from the analytical literature. Journal of food composition and analysis. 2006a Aug 1;19:S74-80.

Peterson JJ, Dwyer JT, Beecher GR, Bhagwat SA, Gebhardt SE, Haytowitz DB, Holden JM. Flavanones in oranges, tangerines (mandarins), tangors, and tangelos: a compilation and review of the data from the analytical literature. Journal of Food Composition and Analysis. 2006b Aug 1;19:S66-73.

Petramfar P, Hajari F, Yousefi G, Azadi S, Hamedi A. Efficacy of oral administration of licorice as an adjunct therapy on improving the symptoms of patients with Parkinson's disease, A randomized double blinded clinical trial. Journal of ethnopharmacology. 2020 Jan 30;247:112226.

Phan TT, Hughes MA, Cherry GW. Effects of an aqueous extract from the leaves of *Chromolaena odorata* (Eupolin) on the proliferation of human keratinocytes and on their migration in an in vitro model of reepithelialization. wound repair and regeneration. 2001 Jul;9(4):305-13. https://doi.org/10.1046/j.1524-475X.2001.00305.x

Philip D, Kaleena PK, Valivittan K, Kumar CG. Phytochemical screening and antimicrobial activity of *Sansevieria roxburghiana* Schult. and Schult. F. Middle-East J Sci Res. 2011;10(4):512-8.

Phuneerub P, Limpanasithikul W, Palanuvej C, Ruangrungsi N. In vitro anti-inflammatory, mutagenic and antimutagenic activities of ethanolic extract of *Clerodendrum paniculatum* root. Journal of advanced pharmaceutical technology & research. 2015 Apr;6(2):48. doi:10.4103/2231-4040.154529

Picerno P, Mencherini T, Loggia RD, Meloni M, Sanogo R, Aquino RP. An extract of *Lannea microcarpa*: composition, activity and evaluation of cutaneous irritation in cell cultures and reconstituted human epidermis. Journal of pharmacy and pharmacology. 2006 Jul;58(7):981-8. https://doi.org/10.1211/jpp.58.7.0014

Pientaweeratch S, Panapisal V, Tansirikongkol A. Antioxidant, anti-collagenase and anti-elastase activities of *Phyllanthus emblica, Manilkara zapota* and silymarin: An in vitro comparative study for anti-aging applications. Pharmaceutical biology. 2016 Sep 1;54(9):1865-72. https://doi.org/10.3109/13880209.2015.1133658

Pieroni A, Giusti ME, Münz H, Lenzarini C, Turković G, Turković A. Ethnobotanical knowledge of the Istro-Romanians of Žejane in Croatia. Fitoterapia. 2003 Dec 1;74(7-8):710-9. https://doi.org/10.1016/j.fitote.2003.06.002

Pieroni A, Sõukand R. The disappearing wild food and medicinal plant knowledge in a few mountain villages of North-Eastern Albania. Journal of Applied Botany and Food Quality. 2017 Feb 7;90. DOI:10.5073/JABFQ.2017.090.009

Pinky SS, Monira S, Hossain MA, Hossain A. Antioxidant, Anti-inflammatory, Cytotoxic and Analgesic Activities of *Sensevieria trifasciata*. Bangladesh Pharmaceutical Journal. 2020 Jul 23;23(2):195-200. https://doi.org/10.3329/bpj.v23i2.48341

Pinto NDCC, Campos LM, Evangelista AC, Lemos AS, Silva TP, Melo RC, De Lourenco CC, Salvador MJ, Apolônio AC, Scio E, Fabri RL. Antimicrobial *Annona muricata* L.(soursop) extract targets the cell membranes of Gram-positive and Gram-negative bacteria. Industrial crops and products. 2017 Nov 15;107:332-40. https://doi.org/10.1016/j.indcrop.2017.05.054

Pirhan D, Yüksel N, Emre E, Cengiz A, Kürşat Yıldız D. Riluzole-and resveratrol-induced delay of retinal ganglion cell death in an experimental model of glaucoma. Current eye research. 2016 Jan 2;41(1):59-69. doi.org/10.3109/02713683.2015.1004719

Piscoya J, Rodriguez Z, Bustamante SA, Okuhama NN, Miller MJ, Sandoval M. Efficacy and safety of freeze-dried cat's claw in osteoarthritis of the knee: mechanisms of action of the species Uncaria guianensis. Inflammation Research. 2001 Sep 1;50(9):442-8. https://doi.org/10.1007/PL00000268

Pitre S, Srivastava SK. Pharmacological, microbiological and phytochemical studies on roots of *Aegle marmelos*. Journal of Ethnopharmacology. 1988 Jul 1;23(2-3):356.

Polat R, Satıl F. An ethnobotanical survey of medicinal plants in Edremit Gulf (Balıkesir–Turkey). Journal of Ethnopharmacology. 2012 Jan 31;139(2):626-41. doi:10.1016/j.jep.2011.12.004

Pommier P, Gomez F, Sunyach MP, D'hombres A, Carrie C, Montbarbon X. Phase III randomized trial of *Calendula officinalis* compared with trolamine for the prevention of acute dermatitis during irradiation for breast cancer. Journal of clinical oncology. 2004 Apr 15;22(8):1447-53. doi: 10.1200/JCO.2004.07.063

Pompermaier L, Marzocco S, Adesso S, Monizi M, Schwaiger S, Neinhuis C, Stuppner H, Lautenschläger T. Medicinal plants of northern Angola and their anti-inflammatory properties. J Ethnopharmacol. 2018 Apr 24;216:26-36. doi: 10.1016/j.jep.2018.01.019.

Ponce-Monter H, Fernández-Martínez E, Ortiz MI, Ramírez-Montiel ML, Cruz-Elizalde D, Pérez-Hernández N, Cariño-Cortés R. Spasmolytic and anti-inflammatory effects of *Aloysia triphylla* and citral, in vitro and in vivo studies. Journal of smooth muscle research. 2010;46(6):309-19.

Pongprayoon U, Bohlin L, Soonthornsaratune P, Wasuwat S. Antiinflammatory activity of *Ipomoea pes-caprae* (L.) R. Br. Phytotherapy Research. 1991b Apr;5(2):63-6.

Pongprayoon U, Bohlin L, Wasuwat S. Neutralization of toxic effects of different crude jellyfish venoms by an extract of *Ipomoea pes-caprae* (L.) R. Br. Journal of ethnopharmacology. 1991a Oct 1;35(1):65-9. https://doi.org/10.1016/0378-8741(91)90133-X

Pongthanapisith V, Ikuta K, Puthavathana P, Leelamanit W. Antiviral protein of *Momordica charantia* L. inhibits different subtypes of Influenza A. Evidence-Based Complementary and Alternative Medicine. 2013 Jan 1;2013. https://doi.org/10.1155/2013/729081

Ponnamma P, Manasa G, Sudarshana MS, Murali M, Mahendra C. In vitro antioxidant, antibacterial and phytochemical screening of *Cochlospermum religiosum* (L.) Alston-A potent medicinal plant. Tropical Plant Research. 2017;4(1):13-9.

Ponou BK, Barboni L, Teponno RB, Mbiantcha M, Nguelefack TB, Park HJ, Lee KT, Tapondjou LA. Polyhydroxyoleanane-type triterpenoids from *Combretum molle* and their anti-inflammatory activity. Phytochemistry Letters. 2008 Dec 12;1(4):183-7. https://doi.org/10.1016/j.phytol.2008.09.002

Postu PA, Gorgan DL, Cioanca O, Russ M, Mikkat S, Glocker MO, Hritcu L. Memory-Enhancing Effects of *Origanum majorana* Essential Oil in an Alzheimer's Amyloid beta1-42 Rat Model: A Molecular and Behavioral Study. Antioxidants (Basel). 2020 Sep 26;9(10):919. doi: 10.3390/antiox9100919.

Potduang B, Meeploy M, Giwanon R, Benmart Y, Kaewduang M, Supatanakul W. Biological activities of *Asparagus racemosus*. African Journal of Traditional, Complementary and Alternative Medicines. 2008;5(3):230-7.

Prabhakar KR, Veerapur VP, Bansal P, Parihar VK, Kandadi MR, Kumar PB, Priyadarsini KI, Unnikrishnan MK. Antioxidant and radioprotective effect of the active fraction of *Pilea microphylla* (L.) ethanolic extract. Chemico-biological interactions. 2007 Jan 5;165(1):22-32. https://doi.org/10.1016/j.cbi.2006.10.007

Prachayasittikul S, Suphapong S, Worachartcheewan A, Lawung R, Ruchirawat S, Prachayasittikul V. Bioactive metabolites from *Spilanthes acmella* Murr. Molecules. 2009 Feb 19;14(2):850-67.

Prakash A, Shur B, Kumar A. Naringin protects memory impairment and mitochondrial oxidative damage against aluminum-induced neurotoxicity in rats. International Journal of Neuroscience. 2013 Sep 1;123(9):636-45.

Prakash B, Singh P, Mishra PK, Dubey NK. Safety assessment of *Zanthoxylum alatum* Roxb. essential oil, its antifungal, antiaflatoxin, antioxidant activity and efficacy as antimicrobial in preservation of *Piper nigrum* L. fruits. International journal of food microbiology. 2012 Feb 1;153(1-2):183-91. https://doi.org/10.1016/j.ijfoodmicro.2011.11.007

Prakash D, Singh BN, Upadhyay G. Antioxidant and free radical scavenging activities of phenols from onion (*Allium cepa*). Food chemistry. 2007 Jan 1;102(4):1389-93. https://doi.org/10.1016/j.foodchem.2006.06.063

Prakash D, Upadhyay G, Singh BN, Singh HB. Antioxidant and free radical-scavenging activities of seeds and agri-wastes of some varieties of soybean (*Glycine max*). Food chemistry. 2007 Jan 1;104(2):783-90.

Prasad D. Antimicrobial activities of whole plant of *Voila canescens* and *Bauhinia variegate*. Biosci Biotechnol Res Asia. 2014;11(1):357-8.

Prashar S, Swamy S, Shalavadi M. Anti-snake Venom Activities of Ethanol and Aqueous Extract of *Cassia hirsuta* against Indian Cobra (*Naja naja*) Venom Induced Toxicity. Science, Technology and Arts Research Journal. 2015;4(4):65-71.

Prashar S, Swamy S, Shalavadi M. Anti-snake venom activities of ethanol and aqueous extract of *Asparagus racernosus* against Indian cobra (*Naja naja*) venom induced toxicity. Asian Journal of Pharmacology and Toxicology, 04(16), 2016, 01-08.

Prashar S, Swamy S, Shalavadi M. Anti-snake Venom Activities of Ethanol and Aqueous Extract of *Cassia hirsuta* against Indian Cobra (*Naja naja*) Venom Induced Toxicity. Science, Technology and Arts Research Journal. 2015;4(4):65-71.

Prathapan A, Vineetha VP, Raghu KG. Protective effect of *Boerhaavia diffusa* L. against mitochondrial dysfunction in angiotensin II induced hypertrophy in H9c2 cardiomyoblast cells. PLoS One. 2014 Apr 30;9(4):e96220. https://doi.org/10.1371/journal.pone.0096220

Preethi K, Premasudha P, Keerthana K. Anti-inflammatory activity of *Muntingia calabura* fruits. Pharmacognosy Journal. 2012 Jul 1;4(30):51-6. https://doi.org/10.5530/pj.2012.30.10

Preethi KC, Kuttan G, Kuttan R. Anti-inflammatory activity of flower extract of *Calendula officinalis* Linn. and its possible mechanism of action. Ind J Exp Biol 2009;47:113-120.

Prescott TA, Kiapranis R, Maciver SK. Comparative ethnobotany and in-the-field antibacterial testing of medicinal plants used by the Bulu and inland Kaulong of Papua New Guinea. Journal of ethnopharmacology. 2012 Jan 31;139(2):497-503. https://doi.org/10.1016/j.jep.2011.09.058

Priya TT, Sabu MC, Jolly CI. Free radical scavenging and anti-inflammatory properties of *Lagerstroemia speciosa* (L). Inflammopharmacology. 2008 Aug 1;16(4):182-7. doi.org/10.1007/s10787-008-7002-6

Promprom W, Chatan W. Estrogenic effects of *Euphorbia hirta* l. Extract in ovariectomized rats. Pharmacognosy Journal. 2018;10(3).

Prudente AS, Loddi AM, Duarte MR, Santos AR, Pochapski MT, Pizzolatti MG, Hayashi SS, Campos FR, Pontarolo R, Santos FA, Cabrini DA. Pre-clinical anti-inflammatory aspects of a cuisine and medicinal millennial herb: *Malva sylvestris* L. Food and chemical toxicology. 2013 Aug 1;58:324-31. https://doi.org/10.1016/j.fct.2013.04.042

Prusti A. Antibacterial activity of some Indian medicinal plants. Ethnobotanical leaflets. 2008;2008(1):27. *Exp Med*. 2015 Oct 15;8(10):18963-71.

Pu X, Ren J, Ma X, Liu L, Yu S, Li X, Li H. *Polyphylla saponin* I has antiviral activity against influenza A virus. International journal of clinical and experimental medicine. 2015;8(10):18963.

Puangpronpitag D, Sittiwet C. Antimicrobial properties of *Cinnamomum verum* aqueous extract. Asian Journal of Biological Sciences. 2009;2(2):49-53. doi: 10.3923/ajbs.2009.49.53

Puia, Z, Kakoti, KB. In vitro and in vivo anti-inflammatory activity of *Dillenia pentagyna* Roxb. bark, a folklore medicine of Mizoram. Sci Vision. 2017;17(2):69-77. doi: 10.33493/scivis.17.02.03

Purnima A, Koti BC, Thippeswamy AH, Jaji MS, Swamy AH, Kurhe YV, Sadiq AJ. Antiinflammatory, Analgesic and Antipyretic Activities of *Mimusops elengi* Linn. Indian J Pharm Sci. 2010 Jul;72(4):480-5. doi: 10.4103/0250-474X.73908.

Pushpa VH, Shetty KP, Sushma N, Kalabaharathi HL, Satish AM. Evaluation of the anticonvulsant activity of ethanol extract of *Psidium guajava* (guava leaves) in albino mice. Int J Pharm Sci Res. 2014 Oct 1;5(10):4288-92. doi: 10.13040/IJPSR.0975-8232.5(10).4288-92

Pushpan R, Karra N, Nariya MB, Ashok BK. Evaluation of anti-arthritic potential of *Leonotis nepetifolia* (L.) R. Br. against Freund's adjuvant induced arthritis. Journal of Ayurveda and Integrated Medical Sciences (ISSN 2456-3110). 2017 Nov 10;2(5):59-66. https://doi.org/10.21760/jaims.v2i05.10255

Putra WE, Rifa'i M. Immunomodulatory Activities of *Sambucus javanica* Extracts in DMBA-Exposed BALB/c Mouse. Advanced pharmaceutical bulletin. 2019 Oct;9(4):619. doi:10.15171/apb.2019.071

Pyne SG, Liawruangrath B, Liawruangrath S, Garson M, Khamsan S, Teerawutkulrag A. Antimalarial, anticancer, antimicrobial activities and chemical constituents of essential oil from the aerial parts of *Cyperus kyllingia* Endl. Records of Natural Products. 2011: 5 (4), 324-327.

Qadir MI, Parveen A, Abbas K, Ali M. Analgesic, anti-inflammatory and anti-pyretic activities of *Thymus linearis*. Pakistan journal of pharmaceutical sciences. 2016 Mar 1;29(2):591-4.

Qi B, Huang H. Anti-fatigue effects of polysaccharides from *Gynostemma pentaphyllum* makino by forced swimming test. InAdvanced Materials Research 2014 (Vol. 881, pp. 426-429). Trans Tech Publications Ltd.

Qi L, Liu CY, Wu WQ, Gu ZL, Guo CY. Protective effect of flavonoids from *Astragalus complanatus* on radiation induced damages in mice. Fitoterapia. 2011 Apr 1;82(3):383-92.

Qian CD, Jiang FS, Yu HS, Shen Y, Fu YH, Cheng DQ, Gan LS, Ding ZS. Antibacterial Biphenanthrenes from the fibrous roots of *Bletilla striata*. Journal of natural products. 2015 Apr 24;78(4):939-43. https://doi.org/10.1021/np501012n

Qian ZG. Cellulase-assisted extraction of polysaccharides from *Cucurbita moschata* and their antibacterial activity. Carbohydrate polymers. 2014 Jan 30;101:432-4. https://doi.org/10.1016/j.carbpol.2013.09.071

Qiao H, Zhang X, Zhu C, Dong L, Wang L, Zhang X, Xing Y, Wang C, Ji Y, Cao X. Luteolin downregulates TLR4, TLR5, NF-κB and p-p38MAPK expression, upregulates the p-ERK expression, and protects rat brains against focal ischemia. Brain research. 2012 Apr 11;1448:71-81. https://doi.org/10.1016/j.brainres.2012.02.003

Qin XJ, Sun DJ, Ni W, Chen CX, Hua Y, He L, Liu HY. Steroidal saponins with antimicrobial activity from stems and leaves of *Paris polyphylla* var. *yunnanensis*. Steroids. 2012 Oct 1;77(12):1242-8. doi.org/10.1016/j.steroids.2012.07.007

Qu H, Zhang Y, Wang Y, Li B, Sun W. Antioxidant and antibacterial activity of two compounds (forsythiaside and forsythin) isolated from *Forsythia suspensa*. Journal of Pharmacy and Pharmacology. 2008 Feb;60(2):261-6. https://doi.org/10.1211/jpp.60.2.0016

Quintans Júnior LJ, Santana MT, Melo MS, de Sousa DP, Santos IS, Siqueira RS, Lima TC, Silveira GO, Antoniolli ÂR, Ribeiro LA, Santos MR. Antinociceptive and anti-inflammatory effects of *Costus spicatus* in experimental animals. Pharmaceutical biology. 2010 Oct 1;48(10):1097-102. doi.org/10.3109/13880200903501822

Qureshi R, Bhatti GR. Ethnobotany of plants used by the Thari people of Nara Desert, Pakistan. Fitoterapia. 2008 Sep 1;79(6):468-73.

Qwarse M, Sempombe J, Mihale MJ, Henry L, Mugoyela V, Sung'hwa F. Cytotoxicity, Antibacterial, and Antifungal Activities of Five Plant Species Used by Agro-pastoral Communities in Mbulu District. Tanzania Int J Res Pharm Chem. 2017;7:1-4.

Rabe SZ, Ghazanfari T, Siadat Z, Rastin M, Zamani Taghizadeh Rabe S, Mahmoudi M. Anti-inflammatory effect of garlic 14-kDa protein on LPS-stimulated-J774A. 1 macrophages. Immunopharmacology and immunotoxicology. 2015 Mar 4;37(2):158-64.

Rabe T, Mullholland D, Van Staden J. Isolation and identification of antibacterial compounds from *Vernonia colorata* leaves. Journal of Ethnopharmacology. 2002 Apr 1;80(1):91-4. doi.org/10.1016/S0378-8741(02)00010-7

Rached W, Bennaceur M, Barros L, Calhelha RC, Heleno S, Alves MJ, Carvalho AM, Marouf A, Ferreira IC. Detailed phytochemical characterization and bioactive properties of *Myrtus nivelii* Batt & Trab. Food & function. 2017;8(9):3111-9. doi: 10.1039/c7fo00744b

Radenahmad N, Saleh F, Sawangjaroen K, Vongvatcharanon U, Subhadhirasakul P, Rundorn W, Withyachumnarnkul B, Connor JR. Young coconut juice, a potential therapeutic agent that could significantly reduce some pathologies associated with Alzheimer's disease: novel findings. British Journal of Nutrition. 2011 Mar;105(5):738-46. https://doi.org/10.1017/S0007114510004241

Radice M, Pietrantoni A, Guerrini A, Tacchini M, Sacchetti G, Chiurato M, Venturi G, Fortuna C. Inhibitory effect of Ocotea quixos (Lam.) Kosterm. and *Piper aduncum* L. essential oils from Ecuador on West Nile virus infection. Plant Biosystems-An International Journal Dealing with all Aspects of Plant Biology. 2019 May 4;153(3):344-51. https://doi.org/10.1080/11263504.2018.1478902

Raghav SK, Gupta B, Agrawal C, Goswami K, Das HR. Anti-inflammatory effect of *Ruta graveolens* L. in murine macrophage cells. Journal of Ethnopharmacology. 2006 Mar 8;104(1-2):234-9.

Rahalison L, Hamburger M, Hostettmann K, Monod M, Frenk E, Gupta MP, Santana AI, Correa MD, Gonzalez AG. Screening for antifungal activity of Panamanian plants. International journal of pharmacognosy. 1993 Jan 1;31(1):68-76.

Rahiman RA, Rajan N, Sreekumaran E. Neuroprotective Effect of *Vitex negundo* Against Scopolamine Induced Cognitive Impairment and Oxidative Stress in Wistar albino Rats. Biosciences Biotechnology Research Asia, 2015 Sept; 12(Spl. Edn. 2):301-307. http://dx.doi.org/10.13005/bbra/2040

Rahman MA, Bachar SC, Rahmatullah M. Analgesic and antiinflammatory activity of methanolic extract of *Acalypha indica* Linn. Pak J Pharm Sci. 2010 Jul 1;23(3):256-8.

Rahman MM, Gibbons S, Gray AI. Isoflavanones from *Uraria picta* and their antimicrobial activity. Phytochemistry. 2007 Jun 1;68(12):1692-7. https://doi.org/10.1016/j.phytochem.2007.04.015

Rahman MR, Alam K, Mollah A, Islam T, Akhter S, Faruquee HM. In-Vitro Evaluation of Thrombolytic Activity of Five Medicinal Plants Available In Bangladesh. Biotechnological Research. 2018 Apr 6;4(2):69-73.

Rahman S, Copeland WC. POLG-related disorders and their neurological manifestations. Nat Rev Neurol. 2019;15(1):40-52. https://doi.org/10.1038/s41582-018-0101-0

Rahmatullah M, Noman A, Hossan MS, Rashid MH, Rahman T, Chowdhury MH, Jahan R. A survey of medicinal plants in two areas of Dinajpur district, Bangladesh including plants which can be used as functional foods. American Eurasian Journal of Sustainable Agriculture. 2009 Dec 1;3(4):862-76.

Rahmawati N, Mustofa Fi, Haryanti S. Diversity of medicinal plants utilized by To Manui ethnic of Central Sulawesi, Indonesia. Biodiversitas Journal of Biological Diversity. 2020 Jan 5;21(1). doi: 10.13057/biodiv/d210145

Rai PK, Lalramnghinglova H. Ethnomedicinal plant resources of Mizoram, India: Implication of traditional knowledge in health care system. Ethnobotanical Leaflets. 2010;2010(3):6.

Rai SN, Birla H, SINGH SS, Zahra W, Patil RR, Jadhav JP, Rao GM, Singh SP. *Mucuna pruriens* protects against MPTP intoxicated neuroinflammation in Parkinson’s disease through NF-κB/pAKT signaling pathways. Frontiers in aging neuroscience. 2017;9:421. doi: 10.3389/fnagi.2017.00421

Rais C, Slimani C, Benidir M, Elhanafi L, Zeouk I, Errachidi F, El Ghadraoui L, Louahlia S. Seeds of *Zizyphus lotus*: In Vivo Healing Properties of the Vegetable Oil. The Scientific World Journal. 2020 Jun 30;2020. https://doi.org/10.1155/2020/1724543

Raj H, Gupta A, Upmanyu N. Anti-Inflammatory Effect of *Woodfordia fructicosa* Leaves Ethanolic Extract on Adjuvant and Carragenan Treated Rats. Anti-inflammatory & anti-allergy agents in medicinal chemistry. 2019 Feb. doi: 10.2174/1871523018666190222120127.

Raja D, Blanché C, Vallès Xirau J. Contribution to the knowledge of the pharmaceutical ethnobotany of La Segarra region (Catalonia, Iberian Peninsula). J Ethnopharmacol. 1997 Aug;57(3):149-60. doi: 10.1016/s0378-8741(97)00059-7.

Raja MJ, Arivuchelvan A, Jagadeeswaran A. Evaluation of antibacterial activity of *Kedrostis foetidissima* (Jacq.) Cogn. plant extracts against pathogens causing bovine mastitis. J Pharmacogn Phytochem. 2019;8:452-5.

Rajagopal PL, Linsha KT, Sreejith KR, Kumar PS, Arthi I, Rahul K, Aneeshia S. Anti-Arthritic Activity of the Leaves of *Urena lobata* Linn. International Journal of Research and Review. 2019;6(1):86-9.

Rajakumar N, Shivanna MB. Ethno-medicinal application of plants in the eastern region of Shimoga District, Karnataka, India. J Ethnopharmacol. 2009 Oct 29;126(1):64-73. doi: 10.1016/j.jep.2009.08.010.

Rajamanickam KA, Sudha SS. In-vitro antimicrobial activity and in vivo toxicity of *Moringa oleifera* and *Allamanda cathartica* against multiple drug resistant clinical pathogens. International Journal of Pharma and Bio Sciences. 2013;4(1):768-5.

Rajbhandari M, Mentel R, Jha PK, Chaudhary RP, Bhattarai S, Gewali MB, Karmacharya N, Hipper M, Lindequist U. Antiviral activity of some plants used in Nepalese traditional medicine. Evidence-Based Complementary and Alternative Medicine. 2009;6(4):517-22.

Rajendran R. Antimicrobial activity of different bark and wood of *Premna serratifolia* Lin. International Journal of Pharma and Bio Sciences. 2010;1(1):1-9.

Rajesh V, Riju T, Venkatesh S, Babu G. Memory enhancing activity of *Lawsonia inermis* Linn. leaves against scopolamine induced memory impairment in Swiss albino mice. Oriental Pharmacy and Experimental Medicine. 2017 Jun 1;17(2):127-42. https://doi.org/10.1007/s13596-017-0268-8

Rajesh Y, Murli KD, Nita Y, Rudraprabhu S. Immunomodulatory potential of ethanol extract of *Spilanthus acmella* leaves. Int J Biol Med Res. 2011;2(3):631-5.

Rajput SA, Wang XQ, Yan HC. Morin hydrate: A comprehensive review on novel natural dietary bioactive compound with versatile biological and pharmacological potential. Biomedicine & Pharmacotherapy. 2021 Jun 1;138:111511. https://doi.org/10.1016/j.biopha.2021.111511

Rakesh D, Atul K, Pal TK. Anti-inflammatory activity study of antidote *Aristolochia indica* to the venom of Heteropneustes fossilis in rats. Journal of Chemical and Pharmaceutical Research. 2010;2(2):554-62.

Rakotomalala G, Agard C, Tonnerre P, Tesse A, Derbré S, Michalet S, Hamzaoui J, Rio M, Cario-Toumaniantz C, Richomme P, Charreau B. Extract from *Mimosa pigra* attenuates chronic experimental pulmonary hypertension. Journal of ethnopharmacology. 2013 Jun 21;148(1):106-16. https://doi.org/10.1016/j.jep.2013.03.075

Ramadevi S, Kaleeswaran B, Natarajan P. Phytochemicals analysis and antimicrobial activity of *Ruellia patula* L. against pathogenic microorganisms. South Indian Journal of Biological Sciences. 2016 Apr 1;2(2):306-13.

Ramesh BN, Indi SS, Rao KS. Anti-amyloidogenic property of leaf aqueous extract of *Caesalpinia crista*. Neuroscience letters. 2010 May 14;475(2):110-4.

Ramesh N, Viswanathan MB, Saraswathy A, Balakrishna K, Brindha P, Lakshmanaperumalsamy P. Phytochemical and antimicrobial studies of *Begonia malabarica*. Journal of ethnopharmacology. 2002 Feb 1;79(1):129-32. https://doi.org/10.1016/S0378-8741(01)00352-X

Rameshkumar A, Sivasudha T. In vitro antioxidant and antibacterial activity of aqueous and methanolic extract *of Mollugo nudicaulis* Lam. leaves. Asian Pacific Journal of Tropical Biomedicine. 2012 Feb 1;2(2):S895-900. https://doi.org/10.1016/S2221-1691(12)60332-3

Ramírez JH, Palacios M, Gutiérrez O. Estudio del efecto antihipertensivo de la *Salvia scutellarioides* en un modelo de ratas hipertensas. Colombia Médica. 2006;37(1):53-60. https://doi.org/10.25100/cm.v37i1.412

Ramírez JH, Palacios M, Gutiérrez O. Implementation of the isolated vascular tissue model as a device for the validation of medicinal plants: Study of the vasodilator activity of *Salvia scutellarioides*. Colombia Médica. 2007 Mar;38(1):28-33.

Ramsewak RS, Nair MG, DeWitt DL, Mattson WG, Zasada J. Phenolic glycosides from *Dirca palustris*. Journal of natural products. 1999 Nov 29;62(11):1558-61. https://doi.org/10.1021/np9903595

Rao BG, Nath MS, Kumar GS, Samuel M. Evaluation of anti-inflammatory activity of roots of *Atalantia monophylla*. Int*. J. Chem. Sci.* 2008;6(1):212-8.

Rao BG, Ramadevi D. Evaluation of Anti-Inflammatory and Anti Arthritic Activity for Different Extracts of Aerial Parts of *Cassia grandis* linn. Int J Pharma Res Health Sci. 2018;6(4):2723-8. doi: 10.21276/ijprhs.2018.04.14

Rao DM, Rao UV, Sudharshanam G. Ethno-medico-botanical studies from Rayalaseema region of southern Eastern Ghats, Andhra Pradesh, India. Ethnobotanical Leaflets. 2006;2006(1):21.

Rao KG, Rao S, Rao S. *Centella asiatica* (L.) leaf extract treatment during the growth spurt period enhances hippocampal CA3 neuronal dendritic arborization in rats. Evidence-Based Complementary and Alternative Medicine. 2006;3(3):349-57.

Rao PS, Ramanjaneyulu YS, Prisk VR, Schurgers LJ. A combination of *Tamarindus indica* seeds and *Curcuma longa* rhizome extracts improves knee joint function and alleviates pain in non-arthritic adults following physical activity. International journal of medical sciences. 2019;16(6):845-853. doi:10.7150/ijms.32505

Rao VU, Viteesha V, Suma K, Nagababu P. Evaluation of phytochemical constituents, antibacterial and antioxidant activities of *Monstera deliciosa* Liebm. stem extracts. World J Pharm Pharm Sci. 2015 Sep 5;4(11):1422-33.

Rao YK, Lien HM, Lin YH, Hsu YM, Yeh CT, Chen CC, Lai CH, Tzeng YM. Antibacterial activities of *Anisomeles indica* constituents and their inhibition effect on *Helicobacter pylori*-induced inflammation in human gastric epithelial cells. Food chemistry. 2012 May 15;132(2):780-7. https://doi.org/10.1016/j.foodchem.2011.11.037

Rasadah MA, Khozirah S, Aznie AA, Nik MM. Anti-inflammatory agents from *Sandoricum koetjape* Merr. Phytomedicine. 2004 Jan 1;11(2-3):261-3. https://doi.org/10.1078/0944-7113-00339

Rasekh HR, Khoshnood-Mansourkhani MJ, Kamalinejad M. Hypolipidemic effects of *Teucrium polium* in rats. Fitoterapia. 2001 Dec 1;72(8):937-9. https://doi.org/10.1016/S0367-326X(01)00348-3

Rashid MA, Gustafson KR, Cardellina JH, Boyd MR. Absolute Stereochemistry and Anti-HIV Activity of Minquartynoic Acid, A Polyacetylene from *Ochanostachys amentacea* 1a. Natural product letters. 2001 Jan 1;15(1):21-6. doi.org/10.1080/10575630108041253

Rashid MA, Gustafson KR, Kashman Y, Cardellina JH, McMahon JB, Boyd MR. Anti-HIV alkaloids from *Toddalia asiatica*. Natural product letters. 1995 Apr 1;6(2):153-6. https://doi.org/10.1080/10575639508044104

Rashid S, Ahmad M, Zafar M, Sultana S, Ayub M, Khan MA, Yaseen G. Ethnobotanical survey of medicinally important shrubs and trees of Himalayan region of Azad Jammu and Kashmir, Pakistan. J Ethnopharmacol. 2015 May 26;166:340-51. doi: 10.1016/j.jep.2015.03.042.

Rashid S, Rather MA, Shah WA, Bhat BA. Chemical composition, antimicrobial, cytotoxic and antioxidant activities of the essential oil of *Artemisia indica* Willd. Food chemistry. 2013 May 1;138(1):693-700. https://doi.org/10.1016/j.foodchem.2012.10.102

Raso GM, Pacilio M, Di Carlo G, Esposito E, Pinto L, Meli R. In-vivo and in-vitro anti-inflammatory effect of *Echinacea purpurea* and *Hypericum perforatum*. Journal of Pharmacy and Pharmacology. 2002 Oct;54(10):1379-83. https://doi.org/10.1211/002235702760345464

Rastogi N, Abaul J, Goh KS, Devallois A, Philogène E, Bourgeois P. Antimycobacterial activity of chemically defined natural substances from the Caribbean flora in Guadeloupe. FEMS Immunology & Medical Microbiology. 1998 Apr 1;20(4):267-73. https://doi.org/10.1111/j.1574-695X.1998.tb01136.x

Rath M, Bhattacharya A, Santra S, Rath K, Ghosh G, Nanda BB. Neuropharmacological effects of methanolic extract of *Clerodendrum viscosum* leaves on wistar albino rats. Pharmacognosy Magazine. 2018 Oct 1;14(59):507. doi: 10.4103/pm.pm_267_18

Rath SK, Mohapatra N, Dubey D, Panda SK, Thatoi HN, Dutta SK. Antimicrobial activity of *Diospyros melanoxylon* bark from similipal biosphere reserve, Orissa, India. African Journal of Biotechnology. 2009;8(9).

Rathi BS, Bodhankar SL, Baheti AM. Evaluation of aqueous leaves extract of *Moringa oleifera* Linn for wound healing in albino rats. Ind J Exp. Biol 2006: 44: 898-901.

Rathnakumar K, Ranbir V, Jaikumar S, Sengottuvelu S. Wound healing activity of ethanolic extract of *Euphorbia hirta* leaves on excision wound model in rats. Global Journal of Research on Medicinal Plants & Indigenous Medicine. 2013 Aug 1;2(8):571.

Rattanamaneerusmee A, Thirapanmethee K, Nakamura Y, Bongcheewin B, Chomnawang MT. Chemopreventive and biological activities of *Helicteres isora* L. fruit extracts. Research in pharmaceutical sciences. 2018 Dec;13(6):484. doi:10.4103/1735-5362.245960

Raubenheimer K, Hickey D, Leveritt M, Fassett R, Ortiz de Zevallos Munoz J, Allen JD, Briskey D, Parker TJ, Kerr G, Peake JM, Pecheniuk NM. Acute effects of nitrate-rich beetroot juice on blood pressure, hemostasis and vascular inflammation markers in healthy older adults: a randomized, placebo-controlled crossover study. Nutrients. 2017 Nov;9(11):1270. doi:10.3390/nu9111270

Rauf A, Jan MR, Rehman WU, Muhammad N. Phytochemical, phytotoxic and antioxidant profile of *Caralluma tuberculata* NE Brown. Wudpecker Journal of Pharmacy and Pharmacology. 2013;2(2):21-5.

Ravi SK, Narasingappa RB, Joshi CG, Girish TK, Vincent B. Neuroprotective effects of *Cassia tora* against paraquat-induced neurodegeneration: relevance for Parkinson's disease. Natural product research. 2018 Jun 18;32(12):1476-80. doi: 10.1080/14786419.2017.1353504

Raymond Chia TW, Dykes GA. Antimicrobial activity of crude epicarp and seed extracts from mature avocado fruit (*Persea americana*) of three cultivars. Pharmaceutical Biology. 2010 Jul 1;48(7):753-6. https://doi.org/10.3109/13880200903273922

Raz L, Bhaskar K, Weaver J, Marini S, Zhang Q, Thompson JF, et al. Hypoxia promotes tau hyperphosphorylation with associated neuropathology in vascular dysfunction. Neurobiol Dis. 2019;126:124-36. https://doi.org/10.1016/j.nbd.2018.07.009

Razali FN, Sinniah SK, Hussin H, Abidin NZ, Shuib AS. Tumor suppression effect of *Solanum nigrum* polysaccharide fraction on Breast cancer via immunomodulation. International journal of biological macromolecules. 2016 Nov 1;92:185-93. https://doi.org/10.1016/j.ijbiomac.2016.06.079

Razali, F.N., Sinniah, S.K., Hussin, H., Abidin, N.Z. and Shuib, A.S., 2016. Tumor suppression effect of *Solanum nigrum* polysaccharide fraction on Breast cancer via immunomodulation. *International journal of biological macromolecules*, *92*, pp.185-193.

Recio MC, Cerdá-Nicolás M, Potterat O, Hamburger M, Ríos JL. Anti-inflammatory and antiallergic activity in vivo of lipophilic *Isatis tinctoria* extracts and tryptanthrin. Planta medica. 2006 Apr;72(06):539-46. doi: 10.1055/s-2006-931562

Recio MC, Giner RM, Manez S, Rios JL, Marston A, Hostettmann K. Screening of tropical medicinal plants for antiinflammatory activity. Phytotherapy Research. 1995 Dec;9(8):571-4. https://doi.org/10.1002/ptr.2650090807

Reddy AJ, Dubey AK, Handu S, Sachin M, Mediratta PK, Mushtaq QA. Effects of *Musa sapientum* stem extract on experimental models of anxiety. Avicenna journal of phytomedicine. 2017 Nov;7(6):495-501.

Reddy AJ, Dubey AK, Handu SS, Sharma P, Mediratta PK, Ahmed QM, Jain S. Anticonvulsant and Antioxidant Effects of *Musa sapientum* Stem Extract on Acute and Chronic Experimental Models of Epilepsy. Pharmacognosy Res. 2018 Jan-Mar;10(1):49-54. doi: 10.4103/pr.pr_31_17.

Reddy JS, Rao PR, Reddy MS. Wound healing effects of *Heliotropium indicum, Plumbago zeylanicum* and *Acalypha indica* in rats. Journal of ethnopharmacology. 2002 Feb 1;79(2):249-51. https://doi.org/10.1016/S0378-8741(01)00388-9

Reddy NS, Raju AB. Antiarthritic and antioxidant activities of *Gossypium herbaceum* plant (cotton plant) leaves. Agricultural Science Digest-A Research Journal. 2018;38(2):88-94. doi: 10.18805/ag.D-4551

Reddy RL, Reddy RS, Ramesh T, Singh TR, Swapna LA, Laxmi NV. Randomized trial of *Aloe vera* gel vs triamcinolone acetonide ointment in the treatment of oral lichen planus. Quintessence international. 2012 Oct 1;43(9).

Reddy SH, Chakravarthi M, Chandrashekara KN, Naidu CV. Phytochemical Screening and Antibacterial Studies on Leaf and Root Extracts of *Asclepias curassavica* (L). Journal of Pharmacy and Biological Sciences. 2012;2:39-44.

Reddy VD, Padmavathi P, Varadacharyulu NC. *Emblica officinalis* protects against alcohol-induced liver mitochondrial dysfunction in rats. Journal of Medicinal Food. 2009 Apr 1;12(2):327-33. https://doi.org/10.1089/jmf.2007.0694

Rege AA, Ambaye RY, Deshmukh RA. In vitro testing of anti-HIV activity of some medicinal plants. Indian J Nat Prod Resour. 2010 Jun;1(2):193-9.

Rege MG, Ayanwuyi LO, Zezi AU, Odoma S. Anti-nociceptive, anti-inflammatory and possible mechanism of anti-nociceptive action of methanol leaf extract of *Nymphaea lotus* Linn (Nymphaeceae). Journal of Traditional and Complementary Medicine. 2020 Feb 28. https://doi.org/10.1016/j.jtcme.2020.02.010

Reis SR, Valente LM, Sampaio AL, Siani AC, Gandini M, Azeredo EL, D'Avila LA, Mazzei JL, Maria das Graças MH, Kubelka CF. Immunomodulating and antiviral activities of *Uncaria tomentosa* on human monocytes infected with Dengue Virus-2. International immunopharmacology. 2008 Mar 1;8(3):468-76. https://doi.org/10.1016/j.intimp.2007.11.010

Ren HC, Zhang J, Liang H. Two new p-coumaroylated sesquiterpenoids from *Pilea cavaleriei*. Journal of Asian natural products research. 2018 Feb 1;20(2):109-16. https://doi.org/10.1080/10286020.2017.1320990

Ren J, Fan C, Chen N, Huang J, Yang Q. Resveratrol pretreatment attenuates cerebral ischemic injury by upregulating expression of transcription factor Nrf2 and HO-1 in rats. Neurochemical research. 2011 Dec 1;36(12):2352.

Rendón-Vallejo P, Hernández-Abreu O, Vergara-Galicia J, Millán-Pacheco C, Mejía A, Ibarra-Barajas M, Estrada-Soto S. Ex vivo study of the vasorelaxant activity induced by phenanthrene derivatives isolated from *Maxillaria densa*. Journal of natural products. 2012 Dec 12;75(12):2241-5. https://doi.org/10.1021/np300508v

Rengifo-Rios AM, Muñoz-Gómez LM, Cabezas-Fajardo FA, Guerrero-Vargas JA. Edematic and coagulant effects caused by the venom of *Bothrops rhombeatus* neutralized by the ethanolic extract of *Piper auritum*. Journal of ethnopharmacology. 2019 Oct 5;242:112046. https://doi.org/10.1016/j.jep.2019.112046

Reutrakul V, Ningnuek N, Pohmakotr M, Yoosook C, Napaswad C, Kasisit J, Santisuk T, Tuchinda P. Anti HIV-1 flavonoid glycosides from *Ochna integerrima*. Planta medica. 2007 Jun;73(07):683-8. doi: 10.1055/s-2007-981538

Rezaie A, Jafari B, Mousavi G, Ebadi AR, Ahmadizadeh C, Shishegar R, Pashazadeh M. Study of anxiolytic effect of herbal extract of *Origanum majorana* L. in comparison with diazepam in rat. Iranian Journal of Medicinal and Aromatic Plants. 2014;30(1).

Rezai-Zadeh K, Arendash GW, Hou H, Fernandez F, Jensen M, Runfeldt M, Shytle RD, Tan J. Green tea epigallocatechin-3-gallate (EGCG) reduces β-amyloid mediated cognitive impairment and modulates tau pathology in Alzheimer transgenic mice. Brain research. 2008 Jun 12;1214:177-87.

Rezai-Zadeh K, Shytle D, Sun N, Mori T, Hou H, Jeanniton D, Ehrhart J, Townsend K, Zeng J, Morgan D, Hardy J. Green tea epigallocatechin-3-gallate (EGCG) modulates amyloid precursor protein cleavage and reduces cerebral amyloidosis in Alzheimer transgenic mice. Journal of Neuroscience. 2005 Sep 21;25(38):8807-14. https://doi.org/10.1523/JNEUROSCI.1521-05.2005

Riaz T, Abbasi MA, Shazadi T, Shahid M. Assessment of Fumaria indica, *Dicliptera bupleuroides* and *Curcuma zedoaria* for their antimicrobial and hemolytic effects. Pakistan journal of pharmaceutical sciences. 2019 Mar 1;32(2).

Ribeiro R, Carvalho FA, Barbosa-Filho JM, Cordeiro RS, Tibiriçá EV. Protective effects of Yangambin—a naturally occurring platelet-activating factor (PAF) receptor antagonist—on anaphylactic shock in rats. Phytomedicine. 1996 Nov 1;3(3):249-56. https://doi.org/10.1016/S0944-7113(96)80062-6

Ribeiro RV, Bieski IG, Balogun SO, de Oliveira Martins DT. Ethnobotanical study of medicinal plants used by Ribeirinhos in the North Araguaia microregion, Mato Grosso, Brazil. Journal of ethnopharmacology. 2017 Jun 9;205:69-102. https://doi.org/10.1016/j.jep.2017.04.023

Ribeiro VP, Arruda C, da Silva JJ, Aldana Mejia JA, Furtado NA, Bastos JK. Use of spinning band distillation equipment for fractionation of volatile compounds of *Copaifera* oleoresins for developing a validated gas chromatographic method and evaluating antimicrobial activity. Biomedical Chromatography. 2019 Feb;33(2):e4412. https://doi.org/10.1002/bmc.4412

Richer S, Patel S, Sockanathan S, Ulanski LJ, Miller L, Podella C. Resveratrol based oral nutritional supplement produces long-term beneficial effects on structure and visual function in human patients. Nutrients. 2014 Oct;6(10):4404-20.Nutrients. 2014 Oct; 6(10): 4404–4420. doi: 10.3390/nu6104404

Ried K, Frank OR, Stocks NP. Aged garlic extract lowers blood pressure in patients with treated but uncontrolled hypertension: a randomised controlled trial. Maturitas. 2010 Oct 1;67(2):144-50.

Riehemann K, Behnke B, Schulze-Osthoff K. Plant extracts from stinging nettle (*Urtica dioica*), an antirheumatic remedy, inhibit the proinflammatory transcription factor NF-κB. FEBS letters. 1999 Jan 8;442(1):89-94.

Rimbau V, Cerdan C, Vila R, Iglesias J. Antiinflammatory activity of some extracts from plants used in the traditional medicine of North-African countries (II). Phytotherapy Research: An International Journal Devoted to Pharmacological and Toxicological Evaluation of Natural Product Derivatives. 1999 Mar;13(2):128-32. https://doi.org/10.1002/(SICI)1099-1573(199903)13:2<128::AID-PTR399>3.0.CO;2-7

Rinayanti A, Radji M, Mun'im A, Suyatna FD. Screening angiotensin converting enzyme (ACE) inhibitor activity of antihypertensive medicinal plants from Indonesia. Int J Pharm Teach Pract. 2013;4:527-32.

Riondato I, Donno D, Roman A, Razafintsalama VE, Petit T, Mellano MG, Torti V, De Biaggi M, Rakotoniaina EN, Giacoma C, Beccaro GL. First ethnobotanical inventory and phytochemical analysis of plant species used by indigenous people living in the Maromizaha forest, Madagascar. J Ethnopharmacol. 2019 Mar 25;232:73-89. doi: 10.1016/j.jep.2018.12.002.

Rivera DE, Ocampo YC, Castro JP, Barrios L, Diaz F, Franco LA. A screening of plants used in Colombian traditional medicine revealed the anti-inflammatory potential of *Physalis angulata* calyces. Saudi journal of biological sciences. 2019 Nov 1;26(7):1758-66.

Rizk AM, Williamson EM, Evans FJ. Constituents of plants growing in Qatar VII an examination of certain plants for anti-inflammatory activity. International Journal of Crude Drug Research. 1985 Jan 1;23(1):1-4. https://doi.org/10.3109/13880208509070677

Robberecht W, Philips T. The changing scene of amyotrophic lateral sclerosis. Nat Rev Neurosci. 2013;14(4):248-64. https://doi.org/10.1038/nrn3430

Roberts BE, Duennwald ML, Wang H, Chung C, Lopreiato NP, Sweeny EA, Knight MN, Shorter J. A synergistic small-molecule combination directly eradicates diverse prion strain structures. Nature chemical biology. 2009 Dec;5(12):936. doi: 10.1038/nchembio.246

Rocha J, Eduardo-Figueira M, Barateiro A, Fernandes A, Brites D, Bronze R, Duarte CM, Serra AT, Pinto R, Freitas M, Fernandes E. Anti-inflammatory effect of rosmarinic acid and an extract of *Rosmarinus officinalis* in rat models of local and systemic inflammation. Basic & clinical pharmacology & toxicology. 2015 May;116(5):398-413. https://doi.org/10.1111/bcpt.12335

Rodino S, Butu A, Petrache PE, Butu M, Dinu-Pirvu CE, Cornea CP. Evaluation of the antimicrobial and antioxidant activity of *Sambucus ebulus* extract. Farmacia. 2015 Sep 1;63(5):751-4.

Rodrigues K, Ramos DF, Carrion LL, Cursino LM, Jefreys MF, Pedroza LS, Osório MI, Oliveira JL, Andrade JI, Fernandes CC, Nunez CV. Antifungal activity of brazilian amazon plants extracts against some species of *Candida* spp. International Journal of Phytopharmacology. 2014;5(6):445-53.

Rodríguez-Cruz A, Romo-Mancillas A, Mendiola-Precoma J, Escobar-Cabrera JE, García-Alcocer G, Berumen LC. Effect of valerenic acid on neuroinflammation in a MPTP-induced mouse model of Parkinson’s disease. IBRO reports. 2020 Jun 1;8:28-35. doi:10.1016/j.ibror.2019.12.002

Rodríguez-Cruz ME, Pérez-Ordaz L, Serrato-Barajas BE, Juárez-Oropeza MA, Mascher D, Paredes-Carbajal MC. Endothelium-dependent effects of the ethanolic extract of the mistletoe *Psittacanthus calyculatus* on the vasomotor responses of rat aortic rings. Journal of ethnopharmacology. 2003 Jun 1;86(2-3):213-8

Rodríguez-Díaz M, Delgado JM, Torres F, Sandoval C, Rodríguez S, Delporte C, Ross C. Phytochemical and pharmacological screening of extracts from *Gunnera tinctoria* Mol., a native chilean plant. Planta Medica. 2013 Aug;79(13):PN92. doi: 10.1055/s-0033-1352434

Rodríguez-García CM, Ruiz-Ruiz JC, Peraza-Echeverría L, Peraza-Sánchez SR, Torres-Tapia LW, Pérez-Brito D, Tapia-Tussell R, Herrera-Chalé FG, Segura-Campos MR, Quijano-Ramayo A, Ramón-Sierra JM. Antioxidant, antihypertensive, anti-hyperglycemic, and antimicrobial activity of aqueous extracts from twelve native plants of the Yucatan coast. PloS one. 2019 Mar 27;14(3):e0213493. https://doi.org/10.1371/journal.pone.0213493

Rogers KL, Grice ID, Griffiths LR. Inhibition of platelet aggregation and 5-HT release by extracts of Australian plants used traditionally as headache treatments. European Journal of Pharmaceutical Sciences. 2000 Feb 1;9(4):355-63. doi: 10.1016/s0928-0987(99)00074-3

Roher AE. Cardiovascular system participation in Alzheimer's disease pathogenesis. J Intern Med. 2015;277(4):426-8. https://doi.org/10.1111/joim.12311

Roman I, Stănilă A, Stănilă S. Bioactive compounds and antioxidant activity of *Rosa canina* L. biotypes from spontaneous flora of Transylvania. Chemistry Central Journal. 2013 Dec;7(1):73. doi.org/10.1186/1752-153X-7-73

Romeilah RM, Fayed SA, Mahmoud GI. Chemical compositions, antiviral and antioxidant activities of seven essential oils. J Appl Sci Res. 2010;6(1):50-62.

Romeiras MM, Duarte MC, Indjai B, Catarino L. Medicinal plants used to treat neurological disorders in West Africa: a case study with Guinea-Bissau flora. American Journal of Plant Sciences. 2012 Jul 1;3(7):1028. http://dx.doi.org/10.4236/ajps.2012.327122

Romeiras MM, Duarte MC, Indjai B, Catarino L. Medicinal plants used to treat neurological disorders in West Africa: a case study with Guinea-Bissau flora. American Journal of Plant Sciences. 2012 Jul 1;3(7):1028. http://dx.doi.org/10.4236/ajps.2012.327122

Romeo L, Diomede F, Gugliandolo A, Scionti D, Lo Giudice F, Lanza Cariccio V, Iori R, Bramanti P, Trubiani O, Mazzon E. Moringin Induces Neural Differentiation in the Stem Cell of the Human Periodontal Ligament. Sci Rep. 2018 Jun 14;8(1):9153. doi: 10.1038/s41598-018-27492-0.

Romeo L, Intrieri M, D'Agata V, Mangano NG, Oriani G, Ontario ML, Scapagnini G. The major green tea polyphenol,(-)-epigallocatechin-3-gallate, induces heme oxygenase in rat neurons and acts as an effective neuroprotective agent against oxidative stress. Journal of the American College of Nutrition. 2009 Aug 1;28(sup4):492S-9S.

Ronchi SN, Brasil GA, do Nascimento AM, de Lima EM, Scherer R, Costa HB, Romão W, Boëchat GA, Lenz D, Fronza M, Bissoli NS. Phytochemical and in vitro and in vivo biological investigation on the antihypertensive activity of mango leaves (*Mangifera indica* L.). Therapeutic advances in cardiovascular disease. 2015 Oct;9(5):244-56. https://doi.org/10.1177/1753944715572958

Roosita K, Kusharto CM, Sekiyama M, Fachrurozi Y, Ohtsuka R. Medicinal plants used by the villagers of a Sundanese community in West Java, Indonesia. Journal of ethnopharmacology. 2008 Jan 4;115(1):72-81. https://doi.org/10.1016/j.jep.2007.09.010

Rosales Clares V del Pilar, Gross Fernández MD, Rosales Clares RA, Díaz G, de la Caridad R, León Sarabia JE, Vidal M. Evaluación farmacológica de *Pluchea carolinensis* Jacq.(salvia de playa) en animales de experimentación. Revista Cubana de Plantas Medicinales. 1999 Aug;4(2):65-7.

Rosalind TH, Dutta BK, Paul SB. Evaluation of in vitro antioxidant activity, estimation of total phenolic and flavonoid content of leaf extract of *Eurya japonica* Thunb. Asian J. Pharm. Clin. Res. 2013;6:152-5.

Rosas EC, Correa LB, de Almeida Pádua T, Costa TE, Mazzei JL, Heringer AP, Bizarro CA, Kaplan MA, Figueiredo MR, Henriques MG. Anti-inflammatory effect of *Schinus terebinthifolius* Raddi hydroalcoholic extract on neutrophil migration in zymosan-induced arthritis. Journal of ethnopharmacology. 2015 Dec 4;175:490-8. https://doi.org/10.1016/j.jep.2015.10.014

Rossi A, Di Paola R, Mazzon E, Genovese T, Caminiti R, Bramanti P, Pergola C, Koeberle A, Werz O, Sautebin L, Cuzzocrea S. Myrtucommulone from *Myrtus communis* exhibits potent anti-inflammatory effectiveness in vivo. Journal of pharmacology and experimental therapeutics. 2009 Apr 1;329(1):76-86. doi.org/10.1124/jpet.108.143214

Roth A, Schaffner W, Hertel C. Phytoestrogen kaempferol (3, 4′, 5, 7-tetrahydroxyflavone) protects PC12 and T47D cells from β-amyloid–induced toxicity. Journal of neuroscience research. 1999 Aug 1;57(3):399-404.

Roumy V, Gutierrez-Choquevilca AL, Mesia JP, Ruiz L, Macedo JC, Abedini A, Landoulsi A, Samaillie J, Hennebelle T, Rivière C, Neut C. In vitro antimicrobial activity of traditional plant used in mestizo shamanism from the Peruvian amazon in case of infectious diseases. Pharmacognosy magazine. 2015 Oct;11(Suppl 4):S625-S633. doi:10.4103/0973-1296.172975

Roumy V, Ruiz L, Macedo JC, Gutierrez-Choquevilca AL, Samaillie J, Encinas LA, Mesia WR, Cotrina HE, Rivière C, Sahpaz S, Bordage S. Viral hepatitis in the Peruvian Amazon: Ethnomedical context and phytomedical resource. Journal of ethnopharmacology. 2020 Jun 12;255:112735.

Roxana J, Huaroc B, Trujillo Gutiérrez RP. Efecto antiinflamatorio del gel a base del extracto etanólico de las hojas de *Sambucus peruviana* kunth (sauco) en ratas albinas. Pharmaceutical thesis, Universidad Inca Garcilaso de la Vega, 2018. http://repositorio.uigv.edu.pe/handle/20.500.11818/2425

Roy K, Kanwar Jr, Langerholc T. Antiviral Activity Of Selected Indian Medicinal Herbs Against Hepatitis E Virus (Hev) In The Established Porcine Cell Model. Journal of Research and Education in Indian Medicine. 2017;23(1):3-12.

Roy K, Shivakumar H, Sarkar S. Wound healing potential of leaf extracts of *Ficus religiosa* on Wistar albino strain rats. Int J Pharm Tech Res. 2009;1:506-8.

Ruan B, Wang R, Yang YJ, Wang DF, Wang JW, Zhang CC, Yuan D, Zhou ZY, Wang T. Improved effects of saponins from *Panax japonicus* on decline of cognitive function in natural aging rats via NLRP3 inflammasome pathway. Zhongguo Zhong yao za zhi [China journal of Chinese materia medica] 2019 Jan;44(2):344-9. doi: 10.19540/j.cnki.cjcmm.20180921.001

Ruban P, Gajalakshmi K. In vitro antibacterial activity of *Hibiscus rosa–sinensis* flower extract against human pathogens. Asian pacific journal of tropical biomedicine. 2012 May 1;2(5):399-403. https://doi.org/10.1016/S2221-1691(12)60064-1

Ruchel JB, Braun JB, Adefegha SA, Manzoni AG, Abdalla FH, de Oliveira JS, Trelles K, Signor C, Lopes ST, da Silva CB, Castilhos LG. Guarana (*Paullinia cupana*) ameliorates memory impairment and modulates acetylcholinesterase activity in Poloxamer-407-induced hyperlipidemia in rat brain. Physiology & behavior. 2017 Jan 1;168:11-9. doi.org/10.1016/j.physbeh.2016.10.003

Rukunga GM, Kofi-Tsekpo MW, Kurokawa M, Kageyama S, Mungai GM, Muli JM, Tolo FM, Kibaya RM, Muthaura CN, Kanyara JN, Tukei PM. Evaluation of the HIV-1 reverse transcriptase inhibitory properties of extracts from some medicinal plants in Kenya. African journal of health sciences. 2002;9(1):81-90. doi: 10.4314/ajhs.v9i1.30758

Rujeedawa T, Carrillo Félez E, Clare ICH, Fortea J, Strydom A, Rebillat AS, Coppus A, Levin J, Zaman SH. The Clinical and Neuropathological Features of Sporadic (Late-Onset) and Genetic Forms of Alzheimer's Disease. J Clin Med. 2021 Oct 3;10(19):4582. doi: 10.3390/jcm10194582.

Rulhania R, Arya N, Medha K, Rani L. Anti-bacterial potency of weed plants. The Pharma Innovation Journal 2021; 10(6): 343-346.

Rumbaoa RG, Cornago DF, Geronimo IM. Phenolic content and antioxidant capacity of Philippine sweet potato (*Ipomoea batatas*) varieties. Food Chemistry. 2009 Apr 15;113(4):1133-8. https://doi.org/10.1016/j.foodchem.2008.08.088

Ruperti-Repilado FJ, Haefliger S, Rehm S, Zweier M, Rentsch KM, Blum J, Jetter A, Heim M, Leuppi-Taegtmeyer A, Terracciano L, Bernsmeier C. Danger of herbal tea: A case of acute cholestatic hepatitis due to *Artemisia annua* tea. Frontiers in medicine. 2019 Oct 11;6:221. https://doi.org/10.3389/fmed.2019.00221

Ruppelt BM, Pereira EF, Gonçalves LC, Pereira NA. Pharmacological screening of plants recommended by folk medicine as anti-snake venom: I. Analgesic and anti-inflammatory activities. Memórias do Instituto Oswaldo Cruz. 1991;86:203-5.

Ruth AF, Olaide AO, Oluwatoyin SM. The aqueous root extract of *Aristolochia ringens* (Vahl.) Aristolochiaceae inhibits chemically-induced inflammation in rodents. Pak. J. Pharm. Sci. 2014 Nov 1;27(6):1885-9.

Sabatini L, Fraternale D, Di Giacomo B, Mari M, Albertini MC, Gordillo B, Rocchi MB, Sisti D, Coppari S, Semprucci F, Guidi L. Chemical composition, antioxidant, antimicrobial and anti-inflammatory activity of *Prunus spinosa* L. fruit ethanol extract. Journal of Functional Foods. 2020 Apr 1;67:103885. https://doi.org/10.1016/j.jff.2020.103885

Sabde S, Bodiwala HS, Karmase A, Deshpande PJ, Kaur A, Ahmed N, Chauthe SK, Brahmbhatt KG, Phadke RU, Mitra D, Bhutani KK. Anti-HIV activity of Indian medicinal plants. Journal of natural medicines. 2011 Jul 1;65(3-4):662-9. https://doi.org/10.1007/s11418-011-0513-2

Sabir MS, Ahmad DS, Imtiaz H, Tahir KM. Antibacterial activity of *Elaeagnus umbellata* (Thunb.) a medicinal plant from Pakistan. Saudi medical journal. 2007 Feb 1;28(2):259.

Sabraoui T, Khider T, Nasser B, Eddoha R, Moujahid A, Benbachir M, Essamadi A. Determination of punicalagins content, metal chelating, and antioxidant properties of edible pomegranate (*Punica granatum* L) peels and seeds grown in Morocco. International Journal of Food Science. 2020 Sep 17;2020. https://doi.org/10.1155/2020/8885889

Sabry MM, Abdel-Rahman RF, El-Shenawy SM, Hassan AM, El-Gayed SH. Estrogenic activity of Sage (*Salvia officinalis* L.) aerial parts and its isolated ferulic acid in immature ovariectomized female rats. Journal of Ethnopharmacology. 2022 Jan 10;282:114579.

Sabry OM, El Sayed AM, Sleem A. Potential anti-microbial, anti-inflammatory and anti-oxidant activities of *Haplophyllum tuberculatum* growing in Libya. J. Pharmacogn. Nat. Prod. 2016;2(116):2472-0992.

Saddi M, Sanna A, Cottiglia F, Chisu L, Casu L, Bonsignore L, De Logu A. Antiherpevirus activity of *Artemisia arborescens* essential oil and inhibition of lateral diffusion in Vero cells. Ann Clin Microbiol Antimicrob. 2007 Sep 26;6:10. doi: 10.1186/1476-0711-6-10.

Sadeghi H, Mostafazadeh M, Sadeghi H, Naderian M, Barmak MJ, Talebianpoor MS, Mehraban F. In vivo anti-inflammatory properties of aerial parts of *Nasturtium officinale*. Pharmaceutical biology. 2014 Feb 1;52(2):169-74. doi.org/10.3109/13880209.2013.821138

Sadgrove NJ, Hitchcock M, Watson K, Jones GL. Chemical and biological characterization of novel essential oils from *Eremophila bignoniiflora* (F. Muell)(Myoporaceae): A traditional Aboriginal Australian bush medicine. Phytotherapy Research. 2013 Oct;27(10):1508-16.

Sadique J, Chandra T, Thenmozhi V, Elango V. The anti-inflammatory activity of *Enicostemma littorale* and *Mollugo cerviana*. Biochemical medicine and metabolic biology. 1987 Apr 1;37(2):167-76. https://doi.org/10.1016/0885-4505(87)90023-5

Sadowska B, Budzyńska A, Więckowska-Szakiel M, Paszkiewicz M, Stochmal A, Moniuszko-Szajwaj B, Kowalczyk M, Różalska B. New pharmacological properties of *Medicago sativa* and *Saponaria officinalis* saponin-rich fractions addressed to *Candida albicans*. J Med Microbiol. 2014 Aug;63(Pt 8):1076-1086. doi: 10.1099/jmm.0.075291-0.

Sadowska B, Micota B, Różalski M, Redzynia M, Różalski M. The immunomodulatory potential of *Leonurus cardiaca* extract in relation to endothelial cells and platelets. Innate Immunity. 2017 Apr;23(3):285-95. https://doi.org/10.1177/1753425917691116

Saeed MA, Ford MR. Antimicrobial potential of some xanthones from *Swertia ciliata* Buch. et Ham. Acta Pharmaceutica Sciencia. 1998;40(4).

Saenghong N, Wattanathorn J, Muchimapura S, Tongun T, Piyavhatkul N, Banchonglikitkul C, Kajsongkram T. *Zingiber officinale* improves cognitive function of the middle-aged healthy women. Evidence-Based Complementary and Alternative Medicine. 2012 Jan 1;2012. doi.org/10.1155/2012/383062

Safari VZ, Ngugi MP, Orinda G, Njagi EM. Anti-pyretic, Anti-inflammatory and Analgesic Activities of Aqueous Stem Extract of *Cynanchum viminale* (L.) in Albino Mice. Med Aromat Plants. 2016;5(236):2167-0412. http://dx.doi.org/10.4172/2167-0412.1000236

Saffari A, Kölker S, Hoffmann GF, Ebrahimi-Fakhari D. Linking mitochondrial dysfunction to neurodegeneration in lysosomal storage diseases. J Inherit Metab Dis. 2017;40(5):631-40. https://doi.org/10.1007/s10545-017-0048-0

Sagesaka-Mitane Y, Sugiura T, Miwa Y, Yamaguchi K, Kyuki K. Effect of tea-leaf saponin on blood pressure of spontaneously hypertensive rats. Yakugaku Zasshi: Journal of the Pharmaceutical Society of Japan. 1996 May 1;116(5):388-95. DOI: 10.1248/yakushi1947.116.5_388

Sagnia B, Fedeli D, Casetti R, Montesano C, Falcioni G, Colizzi V. Antioxidant and anti-inflammatory activities of extracts from *Cassia alata*, *Eleusine indica*, *Eremomastax speciosa*, *Carica papaya* and *Polyscias fulva* medicinal plants collected in Cameroon. PloS one. 2014 Aug 4;9(8):e103999. doi.org/10.1371/journal.pone.0103999

Şahin F, Güllüce M, Daferera D, Sökmen A, Sökmen M, Polissiou M, Agar G, Özer H. Biological activities of the essential oils and methanol extract of *Origanum vulgare* ssp. vulgare in the Eastern Anatolia region of Turkey. Food control. 2004 Oct 1;15(7):549-57.

Sahoo H, Mahalik G. Ethnobotanical survey of medicinal plants of Kantapada block of Cuttack district, Odisha, India. International Journal of Biosciences. 2020;16(5):284-92.

Sahoo HB, Sahoo SK, Mishra K, Sagar R. Evaluation of the wound-healing potential of *Amaranthus viridis (*Linn.) in experimentally induced diabetic rats. International Journal of Nutrition, Pharmacology, Neurological Diseases. 2015 Apr 1;5(2):50.

Sahoo S, Kharkar PS, Sahu NU. Anxiolytic activity of *Psidium guajava* in mice subjected to chronic restraint stress and effect on neurotransmitters in brain. Phytotherapy Research. 2020 Oct 9. https://doi.org/10.1002/ptr.6900

Sahouo GB, Tonzibo ZF, Boti B, Chopard C, Mahy JP, N'guessan YT. Anti-inflammatory and analgesic activities: Chemical constituents of essential oils of *Ocimum gratissimum, Eucalyptus citriodora* and *Cymbopogon giganteus* inhibited lipoxygenase L-1 and cyclooxygenase of PGHS. Bulletin of the Chemical Society of Ethiopia. 2003;17(2). doi: 10.4314/bcse.v17i2.61681

Sahu RK, Kumar H, Roy A. Antipyretic effect of the ethanol extract obtained from leaves of *Mitragyna parvifolia* on a pyretic model induced by brewer's yeast. Biosciences Biotechnology Research Asia. 2016 Apr 29;5(2):881-3.

Sahu RK, Roy A, Kothiya S, Maurya AK, Kumar R. Screening of Antipyretic and Analgesic Potential of Ethanol Extract of *Cassytha filiformis* Leaves. Research Journal of Science and Technology. 2012 May 1;4(3):IV.

Said O, Khalil K, Fulder S, Azaizeh H. Ethnopharmacological survey of medicinal herbs in Israel, the Golan Heights and the West Bank region. Journal of ethnopharmacology. 2002 Dec 1;83(3):251-65. https://doi.org/10.1016/S0378-8741(02)00253-2

Saidu K, Onah J, Orisadipe A, Olusola A, Wambebe C, Gamaniel K. Antiplasmodial, analgesic, and anti-inflammatory activities of the aqueous extract of the stem bark of *Erythrina senegalensis*. Journal of ethnopharmacology. 2000 Jul 1;71(1-2):275-80.

Sakai Y, Murakami T, Yamamoto Y. Antihypertensive effects of onion on NO synthase inhibitor-induced hypertensive rats and spontaneou  https://doi.org/10.1271/bbb.67.1305

Sakat S, Juvekar AR, Gambhire MN. In vitro antioxidant and anti-inflammatory activity of methanol extract of *Oxalis corniculata* Linn. Int J Pharm Pharm Sci. 2010;2(1):146-55.

Sakee U, Maneerat S, Cushnie TT, De-Eknamkul W. Antimicrobial activity of *Blumea balsamifera* (Lin.) DC. extracts and essential oil. Natural Product Research. 2011 Nov 1;25(19):1849-56. https://doi.org/10.1080/14786419.2010.485573

Saklani S, Chandra S. In vitro antimicrobial activity, nutritional value, antinutritional value and phytochemical screening of *Pyracantha crenulata* fruit. Int J Pharm Sci Rev Res. 2014;26(1):1-5.

Sakthivel G, Dey A, Nongalleima K, Chavali M, Rimal Isaac RS, Singh NS, Deb L. In vitro and in vivo evaluation of polyherbal formulation against Russell's viper and cobra venom and screening of bioactive components by docking studies. Evidence-Based Complementary and Alternative Medicine. 2013;2013. https://doi.org/10.1155/2013/781216

Salahdeen HM, Adebari AO, Murtala BA, Alada AR. Potassium channels and prostacyclin contribute to vasorelaxant activities of *Tridax procumbens* crude aqueous leaf extract in rat superior mesenteric arteries. African journal of medicine and medical sciences. 2015 Mar;44(1):5-19.

Salawu OA, Chindo BA, Tijani AY, Obidike IC, Salawu TA, Akingbasote AJ. Acute and sub-acute toxicological evaluation of the methanolic stem bark extract of *Crossopteryx febrifuga* in rats. African Journal of Pharmacy and Pharmacology. 2009 Dec 31;3(12):621-6.

Saleem M, Iftikhar A, Asif M, Hussain K, Alamgeer, Shah PA, Saleem A, Akhtar MF, Tanzeem M, Yaseen HS. *Asphodelus tenuifolius* extracts arrested inflammation and arthritis through modulation of TNF-α, NF-κB, ILs, and COX-2 activities in in vivo models. Inflammopharmacology. 2020 Oct 16. doi: 10.1007/s10787-020-00761-z.

Saleem R, Shinwari ZK, Ali A, Malik A, Shahzad MS, Butt M, Nadir H, Qureshi MK, Ali Q. Comparative in vitro anti-oxidant and anti-fungal potential profiles from methanol extract of *Fagonia indica, Fagonia bruguieri* and *Fagonia paulayana*. International Journal of Botany Studies 2019 Sept 4(5): 69-76.

Saleem U, Raza Z, Anwar F, Ahmad B, Hira S, Ali T. Experimental and Computational Studies to Characterize and Evaluate the Therapeutic Effect of *Albizia lebbeck* (L.) Seeds in Alzheimer's Disease. Medicina (Kaunas). 2019 May 21;55(5):184. doi: 10.3390/medicina55050184.

Saleem U, Zaib S, Khalid S, Anwar F, Akhtar MF, Ahmad B. Chemical characterization, docking studies, anti-arthritic activity and acute oral toxicity of *Convolvulus arvensis* L. leaves. Asian Pacific Journal of Tropical Biomedicine. 2020 Oct 1;10(10):442. doi: 10.4103/2221-1691.290869

Salim KN, McEwen BS, Chao HM. Ginsenoside Rb1 regulates ChAT, NGF and trkA mRNA expression in the rat brain. Molecular Brain Research. 1997 Jul 1;47(1-2):177-82. https://doi.org/10.1016/S0169-328X(97)00042-9

Salvini S, Sera F, Caruso D, Giovannelli L, Visioli F, Saieva C, Masala G, Ceroti M, Giovacchini V, Pitozzi V, Galli C. Daily consumption of a high-phenol extra-virgin olive oil reduces oxidative DNA damage in postmenopausal women. British journal of nutrition. 2006 Apr;95(4):742-51. doi: 10.1079/bjn20051674

Samad N, Muneer A, Zaman A, Ayaz MM, Ahmad I. Banana fruit pulp and peel involved in antianxiety and antidepressant effects while invigorate memory performance in male mice: Possible role of potential antioxidants. Pakistan journal of pharmaceutical sciences. 2017 May 3;30.

Samaha AA, Fawaz M, Salami A, Baydoun S, Eid AH. Antihypertensive indigenous lebanese plants: ethnopharmacology and a clinical trial. Biomolecules. 2019 Jul;9(7):292. doi:10.3390/biom9070292

Samanta A, Das G, Ghosh S, Ojha D. In vivo & in vitro anti-inflammatory activity of the methanolic extract and isolated compound from the leaves of *Cassia tora* L.(Leguminosae/caesalpinaceae). Journal of Pharmacy Research. 2011 Jul;4(7):1999-2002.

Sambrekar SN, Patil PA, Patil SA. Wound Healing Activity of Root Extracts of *Commelina benghalensis* Linn. Research Journal of Pharmacy and Technology. 2011;4(11):1772-6.

Samira K, Md K, Laboni FR, Julie AS, Jalal U, Labu ZK. Biological investigations of medicinal plants of *Heliotropium indicum* indigenous to Bangladesh. Journal of Coastal Life Medicine. 2016;4(11):874-8.

Sampaio FC, Maria do Socorro VP, Dias CS, Costa VC, Conde NC, Buzalaf MA. In vitro antimicrobial activity of *Caesalpinia ferrea* Martius fruits against oral pathogens. Journal of Ethnopharmacology. 2009 Jul 15;124(2):289-94.

Samud AM, Asmawi MZ, Sharma JN, Yusof AP. Anti-inflammatory activity of *Crinum asiaticum* plant and its effect on bradykinin-induced contractions on isolated uterus. Immunopharmacology. 1999 Sep 1;43(2-3):311-6. doi.org/10.1016/S0162-3109(99)00132-0

Samuel AJ, Kalusalingam A, Chellappan DK, Gopinath R, Radhamani S, Husain HA, Muruganandham V, Promwichit P. Ethnomedical survey of plants used by the Orang Asli in Kampung Bawong, Perak, West Malaysia. J Ethnobiol Ethnomed. 2010 Feb 7;6:5. doi: 10.1186/1746-4269-6-5.

Samy PR, Chow VT. Pilot Study with regard to the Wound Healing Activity of Protein from *Calotropis procera* (Ait.) R. Br. Evidence-Based Complementary and Alternative Medicine. 2012;2012. Article ID 294528 https://doi.org/10.1155/2012/294528

Samy RP, Rajendran P, Li F, Anandi NM, Stiles BG, Ignacimuthu S, Sethi G, Chow VT. Identification of a novel *Calotropis procera* protein that can suppress tumor growth in breast cancer through the suppression of NF-κB pathway. PloS one. 2012 Dec 20;7(12):e48514.

San Chang J, Wang KC, Yeh CF, Shieh DE, Chiang LC. Fresh ginger (*Zingiber officinale*) has anti-viral activity against human respiratory syncytial virus in human respiratory tract cell lines. Journal of ethnopharmacology. 2013 Jan 9;145(1):146-51. doi: 10.1016/j.jep.2012.10.043

Sanches ACC, Lopes GC, Nakamura CV, Dias Filho BP, Mello JCP 2005. Antioxidant and antifungal activities of extracts and condensed tannins from *Stryphnodendron obovatum* Benth. *Rev Bras Cienc Farm 41:* 101-107.

Sánchez E, Heredia N, Camacho-Corona MD, García S. Isolation, characterization and mode of antimicrobial action against *Vibrio cholerae* of methyl gallate isolated from *Acacia farnesiana*. Journal of applied microbiology. 2013 Dec;115(6):1307-16. https://doi.org/10.1111/jam.12328

Sánchez-Medina A, García-Sosa K, May-Pat F, Peña-Rodríguez LM. Evaluation of biological activity of crude extracts from plants used in Yucatecan Traditional Medicine Part I. Antioxidant, antimicrobial and β-glucosidase inhibition activities. Phytomedicine. 2001 Jan 1;8(2):144-51. https://doi.org/10.1078/0944-7113-00020

Sandberg F, Perera-Ivarsson P, El-Seedi HR. A Swedish collection of medicinal plants from Cameroon. J Ethnopharmacol. 2005 Dec 1;102(3):336-43. doi: 10.1016/j.jep.2005.06.032.

Sandborn WJ, Targan SR, Byers VS, Yan X, Tang T. 847x Double Blind Placebo Controlled Phase IIB Trial of HMPL-004 (*Andrographis paniculata* Extract) in Active Mild to Moderate Ulcerative Colitis (UC). Gastroenterology. 2010 May 1;138(5):S-115.

Sandhya VG, Rajamohan T. Beneficial effects of coconut water feeding on lipid metabolism in cholesterol-fed rats. J Med Food 2006; 9: 400–407. doi: 10.1089/jmf.2006.9.400.

Sani I, Bello F, Fakai IM, Abdulhamid A. Evaluation of antisnake venom activities of some medicinal plants using albino rats. Sch. Int. J. Tradit. Complement. Med. 2020a;3(6):111-7.

Sani I, Hassan SW, Faruq UZ, Bello F, Abdulhamid A. Inhibition of snake venom enzymes and antivenom adjuvant effects of *Azadirachta indica* A. Juss.(Meliaceae) leaf extracts. European Journal of Medicinal Plants. 2020b Jul 16:114-28. doi.org/10.30574/gscbps.2020.12.2.0244

Sannegowda KM, Venkatesha SH, Moudgil KD. Tinospora cordifolia inhibits autoimmune arthritis by regulating key immune mediators of inflammation and bone damage. International journal of immunopathology and pharmacology. 2015 Dec;28(4):521-31. https://doi.org/10.1177/0394632015608248

Santana-Méridas O, Polissiou M, Izquierdo-Melero ME, Astraka K, Tarantilis PA, Herraiz-Peñalver D, Sánchez-Vioque R. Polyphenol composition, antioxidant and bioplaguicide activities of the solid residue from hydrodistillation of *Rosmarinus officinalis* L. Industrial Crops and Products. 2014 Aug 1;59:125-34. https://doi.org/10.1016/j.indcrop.2014.05.008

Santi I, Putra B, Wahyuni S. uji efek ekstrak etanol daun cincau hijau (*Cyclea barbata* miers) sebagai antiinflamasi pada tikus putih yang diinduksi karagen. Jurnal Ilmiah As-Syifaa. 2017 Jun 19;9(1):58-66.

Santos EN, Lima J, Noldin VF, Cechinel-Filho V, Rao VS, Lima EF, Schmeda-Hirschmann G, Sousa Jr PT, Martins DT. Anti-inflammatory, antinociceptive, and antipyretic effects of methanol extract of *Cariniana rubra* stem bark in animal models. Anais da Academia Brasileira de Ciências. 2011 Jun;83(2):557-66. doi.org/10.1590/S0001-37652011005000006

Santos FA, Cunha GM, Viana GS, Rao VS, Manoel AN, Silveira ER. Antibacterial activity of essential oils from *Psidium* and *Pilocarpus* species of plants. Phytotherapy research. 1997 Feb;11(1):67-9. https://doi.org/10.1002/(SICI)1099-1573(199702)11:1<67::AID-PTR29>3.0.CO;2-I

Santos GK, Dutra KA, Barros RA, da Câmara CA, Lira DD, Gusmão NB, Navarro DM. Essential oils from *Alpinia purpurata* (Zingiberaceae): chemical composition, oviposition deterrence, larvicidal and antibacterial activity. Industrial Crops and Products. 2012 Nov 1;40:254-60. https://doi.org/10.1016/j.indcrop.2012.03.020

Santos TN, Costa G, Ferreira JP, Liberal J, Francisco V, Paranhos A, Cruz MT, Castelo-Branco M, Figueiredo IV, Batista MT. Antioxidant, anti-inflammatory, and analgesic activities of *Agrimonia eupatoria* L. Infusion. Evidence-Based Complementary and Alternative Medicine. 2017 Apr 12;2017. https://doi.org/10.1155/2017/8309894

Santosh P, Venugopl R, Nilakash AS, Kunjbihari S, Mangala L. Antidepressant activity of methanolic extract of Passiflora foetida leaves in mice. Int J Pharm Pharm Sci. 2011;3(1):112-5.

Sanwal R, Chaudhary AK. Wound healing and antimicrobial potential of *Carissa spinarum* Linn. in albino mice. Journal of ethnopharmacology. 2011 Jun 1;135(3):792-6. https://doi.org/10.1016/j.jep.2011.04.025

Sarandy MM, Novaes RD, Xavier AA, Vital CE, Leite JP, Melo FC, Gonçalves RV. Hydroethanolic extract of *Strychnos pseudoquina* accelerates skin wound healing by modulating the oxidative status and microstructural reorganization of scar tissue in experimental type I diabetes. BioMed research international. 2017;2017, Article ID 9538351, 11 pages. https://doi.org/10.1155/2017/9538351

Saravanan S, Parimelazhagan T. In vitro antioxidant, antimicrobial and anti-diabetic properties of polyphenols of *Passiflora ligularis* Juss. fruit pulp. Food science and human wellness. 2014 Jun 1;3(2):56-64. doi.org/10.1016/j.fshw.2014.05.001

Šarić-Kundalić B, Dobeš C, Klatte-Asselmeyer V, Saukel J. Ethnobotanical survey of traditionally used plants in human therapy of east, north and north-east Bosnia and Herzegovina. Journal of Ethnopharmacology. 2011 Feb 16;133(3):1051-76.

Sarikhani M, Deylam M, Alizadeh E, Hejazy M, Alizadeh-Salteh S, Moeini H, Firouzamandi M. Anti-aging effects of peppermint (*Mentha piperita* L.) and Shirazi thyme (*Zataria multiflora* Boiss.) plant extracts. Food Bioscience. 2021 Jun 1;41:100930. https://doi.org/10.1016/j.fbio.2021.100930

Sarkar L, Bhuvaneswari N, Samanta SK, Islam MN, Sen T, Fukui H, Mizuguchi H, Karmakar S. A report on anti-oedemogenic activity of *Byttneria herbacea* roots–Possible involvement of histamine receptor (type I). Journal of ethnopharmacology. 2012 Mar 27;140(2):443-6. Ethnopharmacological communication https://doi.org/10.1016/j.jep.2012.01.013

Sarkodie JA, Squire SA, Kretchy IA, Domozoro CY, Ahiagbe KM, Twumasi MA, Edoh DA, Sakyiama M, Lamptey VK, Obresi AS, Duncan JL. The antihyperglycemic, antioxidant and antimicrobial activities of *Ehretia cymosa*. Journal of Pharmacognosy and Phytochemistry. 2015 Sep 1;4(3).

Sassoui D, Seridi R, Azin K, Usai M. Evaluation of phytochemical constituents by GC-MS and antidepressant activity of *Peganum harmala* L. seeds extract. Asian Pacific Journal of Tropical Disease. 2015 Dec 1;5(12):971-4.

Sathyanarayanan S, Selvam P, Asha J, George RM, Revikumar KG, Neyts J. Preliminary phytochemical screening and study of antiviral activity and cytotoxicity of *Wrightia tinctoria*. Int J Chem Sci. 2009;7(1):1-5.

Šavikin K, Zdunić G, Menković N, Živković J, Ćujić N, Tereščenko M, Bigović D. Ethnobotanical study on traditional use of medicinal plants in South-Western Serbia, Zlatibor district. Journal of ethnopharmacology. 2013 Apr 19;146(3):803-10. https://doi.org/10.1016/j.jep.2013.02.006

Saxena RS, Gupta B, Saxena KK, Singh RC, Prasad DM. Study of anti-inflammatory activity in the leaves of *Nyctanthes arbor tristis* Linn.—an Indian medicinal plant. Journal of Ethnopharmacology. 1984 Aug 1;11(3):319-30. https://doi.org/10.1016/0378-8741(84)90077-1

Sayeed MA, Faruk M, Chowdhury AI, Rusti A. Thrombolytic and Anti-arthritic Activities of Methanolic Extract of *Trevesia palmata*. e-Journal of Science & Technology. 2014 Nov 1;9(5).

Schapoval EE, de Vargas MR, Chaves CG, Bridi R, Zuanazzi JA, Henriques AT. Antiinflammatory and antinociceptive activities of extracts and isolated compounds from *Stachytarpheta cayennensis*. Journal of ethnopharmacology. 1998 Feb 1;60(1):53-9. https://doi.org/10.1016/S0378-8741(97)00136-0

Schilling S, Goelz S, Linker R, Luehder F, Gold R. Fumaric acid esters are effective in chronic experimental autoimmune encephalomyelitis and suppress macrophage infiltration. Clinical & Experimental Immunology. 2006 Jul;145(1):101-7. https://doi.org/10.1111/j.1365-2249.2006.03094.x

Schinella G, Aquila S, Dade M, Giner R, del Carmen Recio M, Spegazzini E, de Buschiazzo P, Tournier H, Ríos JL. Anti-inflammatory and apoptotic activities of pomolic acid isolated from *Cecropia pachystachya*. Planta medica. 2008 Feb;74(03):215-20. doi: 10.1055/s-2008-1034301

Schink A, Naumoska K, Kitanovski Z, Kampf CJ, Fröhlich-Nowoisky J, Thines E, Pöschl U, Schuppan D, Lucas K. Anti-inflammatory effects of cinnamon extract and identification of active compounds influencing the TLR2 and TLR4 signaling pathways. Food & function. 2018a;9(11):5950-64. doi: 10.1039/C8FO01286E

Schink A, Neumann J, Leifke AL, Ziegler K, Fröhlich-Nowoisky J, Cremer C, Thines E, Weber B, Pöschl U, Schuppan D, Lucas K. Screening of herbal extracts for TLR2-and TLR4-dependent anti-inflammatory effects. PloS one. 2018b;13(10). doi:10.1371/journal.pone.0203907

Schmeda-Hirschmann G, Loyola JI, Rodríguez J, Dutra-Behrens M. Hypotensive effect of *Laurelia sempervirens* (Monimiaceae) on normotensive rats. Phytotherapy Research. 1994 Feb;8(1):49-51. doi.org/10.1002/ptr.2650080112

Schmeda-Hirschmann G, Loyola JI, Sierra J, Retamal R, Rodriguez J. Hypotensive effect and enzyme inhibition activity of Mapuche medicinal plant extracts. Phytotherapy Research. 1992 Jul;6(4):184-8.

Scholey A, Ossoukhova A, Owen L, Ibarra A, Pipingas A, He K, Roller M, Stough C. Effects of American ginseng (*Panax quinquefolius*) on neurocognitive function: an acute, randomised, double-blind, placebo-controlled, crossover study. Psychopharmacology. 2010 Oct 1;212(3):345-56. https://doi.org/10.1007/s00213-010-1964-y

Schröder HC, Merz H, Steffen R, Müller WE, Sarin PS, Trumm S, Schulz J, Eich E. Differential in vitro anti-HIV activity of natural lignans. Zeitschrift für Naturforschung C. 1990 Dec 1;45(11-12):1215-21. https://doi.org/10.1515/znc-1990-11-1222

Schröter D, Neugart S, Schreiner M, Grune T, Rohn S, Ott C. Amaranth's 2-Caffeoylisocitric Acid—An Anti-Inflammatory Caffeic Acid Derivative That Impairs NF-κB Signaling in LPS-Challenged RAW 264.7 Macrophages. Nutrients. 2019 Mar;11(3):571. https://doi.org/10.3390/nu11030571

Schwarz S, Sauter D, Wang K, Zhang R, Sun B, Karioti A, Bilia AR, Efferth T, Schwarz W. Kaempferol derivatives as antiviral drugs against the 3a channel protein of coronavirus. Planta Med. 2014 Feb;80(2-3):177-82. doi: 10.1055/s-0033-1360277.

Sdayria J, Rjeibi I, Feriani A, Ncib S, Bouguerra W, Hfaiedh N, Elfeki A, Allagui MS. Chemical composition and antioxidant, analgesic, and anti-inflammatory effects of methanolic extract of *Euphorbia retusa* in mice. Pain Research and Management. 2018 Jan 1;2018. https://doi.org/10.1155/2018/4838413

Segura L, Vila R, Gupta MP, Espósito-Avella M, Adzet T, Canigueral S. Antiinflammatory activity of *Anthurium cerrocampanense* Croat in rats and mice. Journal of ethnopharmacology. 1998 Jul 1;61(3):243-8. https://doi.org/10.1016/S0378-8741(98)00047-6

Seigner J, Junker-Samek M, Plaza A, D'Urso G, Masullo M, Piacente S, Holper-Schichl YM, de Martin R. A *Symphytum officinale* root extract exerts anti-inflammatory properties by affecting two distinct steps of NF-κB signaling. Frontiers in pharmacology. 2019 Apr 26;10:289. doi: 10.3389/fphar.2019.00289.

Selvam C, Jachak SM. A cyclooxygenase (COX) inhibitory biflavonoid from the seeds of *Semecarpus anacardium*. Journal of ethnopharmacology. 2004 Dec 1;95(2-3):209-12.

Semple SJ, Reynolds GD, O'leary MC, Flower RL. Screening of Australian medicinal plants for antiviral activity. Journal of Ethnopharmacology. 1998 Mar 1;60(2):163-72. https://doi.org/10.1016/S0378-8741(97)00152-9

Semuyaba I, Safiriyu AA, Tiyo EA, Niurka RF. Memory improvement effect of ethanol garlic (*A. sativum*) extract in streptozotocin-nicotinamide induced diabetic wistar rats is mediated through increasing of hippocampal sodium-potassium ATPase, glutamine synthetase, and calcium ATPase activities. Evidence-Based Complementary and Alternative Medicine. 2017 Dec 27;2017. doi:10.1155/2017/3720380

Semwal RB, K Semwal DK Analgesic and anti-inflammatory activities of extracts and fatty acids from *Celtis australis* L. The Natural Products Journal. 2012 Dec 1;2(4):323-7.

Şen A. Antioxidant and anti-inflammatory activity of fruit, leaf and branch extracts of *Paliurus spina-christ*i P. Mill. Marmara Pharmaceutical Journal. 2018 May 1;22(2).

Senatore F, De Fusco R, Napolitano F. *Eupatorium cannabinum* L. ssp. *cannabinum* (Asteraceae) essential oil: chemical composition and antibacterial activity. Journal of Essential Oil Research. 2001 Nov 1;13(6):463-6. https://doi.org/10.1080/10412905.2001.9699730

Senejoux F, Demougeot C, Cuciureanu M, Miron A, Cuciureanu R, Berthelot A, Girard-Thernier C. Vasorelaxant effects and mechanisms of action of *Heracleum sphondylium* L.(Apiaceae) in rat thoracic aorta. Journal of ethnopharmacology. 2013 May 20;147(2):536-9. https://doi.org/10.1016/j.jep.2013.03.030

Senger DR, Hoang MV, Kim KH, Li C, Cao S. Anti-inflammatory activity of *Barleria lupulina*: Identification of active compounds that activate the Nrf2 cell defense pathway, organize cortical actin, reduce stress fibers, and improve cell junctions in microvascular endothelial cells. *J Ethnopharmacol*. 2016;193:397–407. doi:10.1016/j.jep.2016.09.017

Senouci F, Ababou A, Chouieb M. Ethnobotanical Survey of the Medicinal Plants used in the Southern Mediterranean. Case Study: The Region of Bissa (Northeastern Dahra Mountains, Algeria). Pharmacognosy Journal. 2019;11(4), 647-659. DOI : 10.5530/pj.2019.11.103

Seo JS, Choi J, Leem YH, Han PL. Rosmarinic acid alleviates neurological symptoms in the G93A-SOD1 transgenic mouse model of amyotrophic lateral sclerosis. Experimental neurobiology. 2015 Dec 1;24(4):341-50. https://doi.org/10.5607/en.2015.24.4.341

Seong RK, Kim JA, Shin OS. Wogonin, a flavonoid isolated from *Scutellaria baicalensis*, has anti-viral activities against influenza infection via modulation of AMPK pathways. Acta virologica. 2018 Jan 1;62(1):78-85. D
doi: 10.4149/av_2018_109

Seoposengwe K, Van Tonder JJ, Steenkamp V. In vitro neuroprotective potential of four medicinal plants against rotenone-induced toxicity in SH-SY5Y neuroblastoma cells. BMC complementary and alternative medicine. 2013 Dec;13(1):1-1. doi:10.1186/1472-6882-13-353

Seoudi DM, Medhat AM, Hewedi IH, Osman SA, Mohamed MK, Arbid MS. Evaluation of the anti-inflammatory, analgesic, and anti-pyretic effects of *Origanum majorana* ethanolic extract in experimental animals. Journal of Radiation Research and Applied Sciences. 2009;2(3):513-34.

Sethiya NK, Nahata A, Singh PK, Mishra SH. Neuropharmacological evaluation on four traditional herbs used as nervine tonic and commonly available as Shankhpushpi in India. Journal of Ayurveda and integrative medicine. 2019 Jan 1;10(1):25-31. doi:10.1016/j.jaim.2017.08.012

Seyyednejad SM, Koochak H, Darabpour E, Motamedi H. A survey on Hibiscus rosa—sinensis, *Alcea rosea* L. and *Malva neglecta* Wallr as antibacterial agents. Asian Pacific Journal of Tropical Medicine. 2010 May 1;3(5):351-5.

Shah BH, Nawaz Z, Virani SS, Ali IQ, Saeed SA, Gilani AH. The inhibitory effect of cinchonine on human platelet aggregation due to blockade of calcium influx. Biochemical pharmacology. 1998 Oct 15;56(8):955-60.

Shah BH, Safdar B, Virani SS, Nawaz Z, Saeed SA, Gilani AH. The antiplatelet aggregatory activity of *Acacia nilotica* is due to blockade of calcium influx through membrane calcium channels. General pharmacology. 1997 Aug;29(2):251-5. doi:: 10.1016/s0306-3623(96)00413-2

Shah MK, Sirat HM, Jamil S, Jalil J. Flavonoids from the Bark of *Artocarpus integer* var. *silvestris* and their Anti-inflammatory Properties. Natural product communications. 2016 Sep;11(9): 1275 – 1278. https://doi.org/10.1177/1934578X1601100921

Shah SK, Patel KM, Rathod NP. Evaluation of the Activity of *Leea indica* Merrill in Inflammatory Bowel Disease using Experimental Models. International Journal of Pharmaceutical Sciences and Nanotechnology. 2018 Jul – Aug 11(4): 4219.

Shah WA, Dar MY, Zagar MI, Agnihotri VK, Qurishi MA, Singh B. Chemical composition and antimicrobial activity of the leaf essential oil of *Skimmia laureola* growing wild in Jammu and Kashmir, India. Natural product research. 2013 Jun 1;27(11):1023-7.

Shahat AA, Mahmoud EA, Al-Mishari AA, Alsaid MS. Antimicrobial activities of some Saudi Arabian herbal plants. African Journal of Traditional, Complementary and Alternative Medicines. 2017;14(2):161-5.

Shahla SN. Chemical composition and in vitro antibacterial activity of *Ziziphora clinopodioides* Lam. essential oil against some pathogenic bacteria. African Journal of Microbiology Research. 2012 Feb 23;6(7):1504-8.

Shahnama M, Azami S, Mohammadhosseini M. Characterization of the essential oil and evaluation of antibacterial activity of methanolic extract of *Stachys lavandulifolia* Vahl. International Journal of Current Microbiology and Applied Sciences. 2015;4(3):275-83.

Shahni R, Handique PJ. Antibacterial properties of leaf extracts of *Strobilanthes cusia* (Nees) Kuntze, a rare ethno-medicinal plant of Manipur, India. Int J PharmTech Res. 2013;5:1281-5.

Shai LJ, Chauke MA, Magano SR, Mogale AM, Eloff JN. Antibacterial activity of sixteen plant species from Phalaborwa, Limpopo Province, South Africa. Journal of Medicinal Plants Research. 2013 Jul 10;7(26):1899-906. doi: 10.5897/JMPR2013.2598

Shajib M, Akter S, Ahmed T, Imam MZ. Antinociceptive and neuropharmacological activities of methanol extract of *Phoenix sylvestris* fruit pulp. Frontiers in pharmacology. 2015 Oct 2;6:212. doi.org/10.3389/fphar.2015.00212

Shale TL, Stirk WA, Van Staden J. Variation in antibacterial and anti-inflammatory activity of different growth forms of *Malva parviflora* and evidence for synergism of the anti-inflammatory compounds. Journal of ethnopharmacology. 2005 Jan 4;96(1-2):325-30. https://doi.org/10.1016/j.jep.2004.09.032

Shan B, Cai YZ, Sun M, Corke H. Antioxidant capacity of 26 spice extracts and characterization of their phenolic constituents. Journal of agricultural and food chemistry. 2005 Oct 5;53(20):7749-59. doi: 10.1021/jf051513y

Shang JH, Cai XH, Feng T, Zhao YL, Wang JK, Zhang LY, Yan M, Luo XD. Pharmacological evaluation of *Alstonia scholaris*: Anti-inflammatory and analgesic effects. Journal of ethnopharmacology. 2010 May 27;129(2):174-81. https://doi.org/10.1016/j.jep.2010.02.011

Shang Y, Xie C, Meng L, Cui F, Lu H, Li W, Li K. Polyphenol Profiles and Antioxidant Activities of Non-centrifugal Sugars Derived from Different Varieties of Membrane-Clarified Sugarcane Juice. Sugar Tech. 2021 May 24:1-2. https://doi.org/10.1007/s12355-021-00989-1

Shariff N, Sudarshana MS, Umesha S, Hariprasad P. Antimicrobial activity of *Rauvolfia tetraphylla* and *Physalis minima* leaf and callus extracts. African Journal of Biotechnology. 2006;5(10).

Sharma A, Joshi R, Kumar S, Sharma R, Padwad Y, Gupta M. *Prunus cerasoides* fruit extract ameliorates inflammatory stress by modulation of iNOS pathway and Th1/Th2 immune homeostasis in activated murine macrophages and lymphocytes. Inflammopharmacology. 2018a Dec 1;26(6):1483-95. https://doi.org/10.1007/s10787-018-0448-2

Sharma A, Kaur G. *Tinospora cordifolia* as a potential neuroregenerative candidate against glutamate induced excitotoxicity: an in vitro perspective. BMC complementary and alternative medicine. 2018 Dec 1;18(1):268. https://doi.org/10.1186/s12906-018-2330-6

Sharma A, Tanwar M, Nagar N, Sharma AK. Analgesic and anti-inflammatory activity of flowers extract of *Aerva lanata*. Advances in Pharmacology and Toxicology. 2011 Dec 1;12(3):13.

Sharma J, Gairola S, Gaur RD, Painuli RM, Siddiqi TO. Ethnomedicinal plants used for treating epilepsy by indigenous communities of sub-Himalayan region of Uttarakhand, India. Journal of ethnopharmacology. 2013 Oct 28;150(1):353-70. https://doi.org/10.1016/j.jep.2013.08.052

Sharma R, Kishore N, Hussein A, Lall N. Antibacterial and anti-inflammatory effects of *Syzygium jambos* L.(Alston) and isolated compounds on acne vulgaris. BMC complementary and alternative medicine. 2013 Dec 1;13(1):292. https://doi.org/10.1186/1472-6882-13-292

Sharma US, Sharma UK, Sutar N, Singh A, Shukla DK. Anti-inflammatory activity of *Cordia dichotoma* forst f. seeds extracts. International Journal of Pharmaceuticals Analysis. 2010 Jan 1;2(1):1.

Sharmin T, Chowdhury SR, Mian MY, Hoque M, Sumsujjaman M, Nahar F. Evaluation of antimicrobial activities of some Bangladeshi medicinal plants. World Journal of Pharmaceutical Sciences. 2014;2(2):170-5.

Shathish K, Guruvayoorappan C. *Solanum muricatum* Ait. Inhibits inflammation and cancer by modulating the immune system. Journal of cancer research and therapeutics. 2014 Jul 1;10(3):623.

Shazhni JA, Renu A, Murugan M. Phytochemical screening and in vitro antimicrobial activity of ornamental plant *Anthurium andraeanum*. Journal of Pharmaceutical Sciences and Research. 2016 Jul 1;8(7):668.

Shehu A, Magaji MG, Yau J, Mahmud B, Ahmed A. Antidepressant Effect of Methanol Stem Bark Extract of *Adansonia digitata* L. Malvaceae) in Mice. Trop J Nat Prod Res. 2018;2(2):87-91.

Sheik HS, Vedhaiyan N, Singaravel S. Evaluation of central nervous system activities of *Citrus maxima* leaf extract on rodents. Journal of Applied Pharmaceutical Science. 2014 Sep 1;4(9):77. doi: 10.7324/JAPS.2014.40914

Shekshavali T, Hugar S. Antimicrobial activity of *Thespesia populnea* Soland. ex Correa bark extracts. Indian Journal of Natural Products and Resources. 2012 Mar; 03(1): 128-130.

Sheng Y, Li L, Holmgren K, Pero RW. DNA repair enhancement of aqueous extracts of *Uncaria tomentosa* in a human volunteer study. Phytomedicine. 2001 Jan 1;8(4):275-82. https://doi.org/10.1078/0944-7113-00045

Shengo LM, Mundongo TH, Kasamba IE, Malonga KF, Kapend AK. A survey of the antibacterial activity of three plants used in the Congolese herbal medicine practiced by the healers in the city of Lubumbashi. African Journal of Pharmacy and Pharmacology. 2013;7(27):1870-5.

Shenoy R, Shirwaikar A. Anti inflammatory and free radical scavenging studies of *Hyptis suaveolens* (Labiatae). Indian drugs. 2002;39(11):574-7.

Sheoran S, Khanam B, Mahanta V, Gupta SK. Efficacy of Ashwagandha [*Withania somnifera* (Linn.) dunal] leaf paste in the management of chronic non-healing wound: A case report. Journal of Ayurveda Case Reports. 2020 Jul 1;3(3):95.

Shetty S, Udupa S, Udupa L. Evaluation of antioxidant and wound healing effects of alcoholic and aqueous extract of *Ocimum sanctum* Linn in rats. Evidence-Based Complementary and Alternative Medicine. 2008;5(1):95-101. doi:10.1093/ecam/nem004

Shi C, Li Q, Zhang X. Platycodin D Protects Human Fibroblast Cells from Premature Senescence Induced by H2O2 through Improving Mitochondrial Biogenesis. Pharmacology. 2020 Jan 31:1-1. doi: 10.1159/000505593.

Shi Y, Xi Y, Shi Y, Hu Z, Luo Y. The antimicrobial activity of *Rodgersia aesculifolia*. Indian Veterinary Journal. 2009;86(11):1182-3.

Shie PH, Huang SS, Deng JS, Huang GJ. *Spiranthes sinensis* suppresses production of pro-inflammatory mediators by down-regulating the NF-κB signaling pathway and up-regulating HO-1/Nrf2 anti-oxidant protein. The American journal of Chinese medicine. 2015 Jul 29;43(05):969-89. https://doi.org/10.1142/S0192415X15500561

Shikov AN, Pozharitskaya ON, Makarov VG, Demchenko DV, Shikh EV. Effect of *Leonurus cardiaca* oil extract in patients with arterial hypertension accompanied by anxiety and sleep disorders. Phytotherapy Research. 2011 Apr;25(4):540-3. https://doi.org/10.1002/ptr.3292

Shilpi JA, Taufiq-Ur-Rahman M, Uddin SJ, Alam MS, Sadhu SK, Seidel V. Preliminary pharmacological screening of *Bixa orellana* L. leaves. Journal of Ethnopharmacology. 2006 Nov 24;108(2):264-71.

Shim SH, Kim JM, Choi CY, Kim CY, Park KH. *Ginkgo biloba* extract and bilberry anthocyanins improve visual function in patients with normal tension glaucoma. Journal of medicinal food. 2012 Sep 1;15(9):818-23.

Shimojo Y, Kosaka K, Noda Y, Shimizu T, Shirasawa T. Effect of rosmarinic acid in motor dysfunction and life span in a mouse model of familial amyotrophic lateral sclerosis. Journal of neuroscience research. 2010 Mar;88(4):896-904.

Shin JS, Noh YS, Lee YS, Cho YW, Baek NI, Choi MS, Jeong TS, Kang E, Chung HG, Lee KT. Arvelexin from *Brassica rapa* suppresses NF-κB-regulated pro-inflammatory gene expression by inhibiting activation of IκB kinase. British journal of pharmacology. 2011 Sep;164(1):145-58.

Shin JW, Ohnishi K, Murakami A, Lee JS, Kundu JK, Na HK, Ohigashi H, Surh YJ. Zerumbone induces heme oxygenase-1 expression in mouse skin and cultured murine epidermal cells through activation of Nrf2. Cancer Prevention Research. 2011 Jun 1;4(6):860-70.

Shin NR, Shin IS, Song HH, Hong JM, Kwon OK, Jeon CM, Kim JH, Lee SW, Lee JK, Jin H, Li WY. *Callicarpa japonica* Thunb. reduces inflammatory responses: a mouse model of lipopolysaccharide-induced acute lung injury. International immunopharmacology. 2015 May 1;26(1):174-80. doi: 10.1016/j.intimp.2015.01.025.

Shin SJ, Jeong Y, Jeon SG, Kim S, Lee SK, Choi HS, Im CS, Kim SH, Kim SH, Park JH, Kim JI. *Uncaria rhynchophylla* ameliorates amyloid beta deposition and amyloid beta-mediated pathology in 5XFAD mice. Neurochemistry international. 2018 Dec 1;121:114-24. https://doi.org/10.1016/j.neuint.2018.10.003

Shindler KS, Ventura E, Dutt M, Elliott P, Fitzgerald DC, Rostami A. Oral resveratrol reduces neuronal damage in a model of multiple sclerosis. Journal of Neuro-Ophthalmology. 2010 Dec;30(4):328-339. doi:10.1097/WNO.0b013e3181f7f833.

Shinwari ZK, Khan I, Naz S, Hussain A. Assessment of antibacterial activity of three plants used in Pakistan to cure respiratory diseases. African Journal of Biotechnology. 2009;8(24).

Shirwaikar A, Rajendran K, Bodla R, Kumar CD. Neutralization potential of Viper *Russelli russelli* (Russell's viper) venom by ethanol leaf extract of *Acalypha indica*. Journal of Ethnopharmacology. 2004 Oct 1;94(2-3):267-73. https://doi.org/10.1016/j.jep.2004.05.010

Shirwaikar A, Somashekar AP. Antiinflammatory activity and free radical scavenging studies of *Aristolochia bracteolata* Lam. Indian journal of pharmaceutical sciences. 2003;65(1):67.

Shivananda A, Rao DM, Jayaveera KN. Analgesic and anti-inflammatory activities of *Citrus maxima* (J. Burm) Merr in animal models. Research Journal of Pharmaceutical, Biological and Chemical Sciences. 2013;4(2):1800-10.

Shivasharan BD, Nagakannan P, Thippeswamy BS, Veerapur VP, Bansal P, Unnikrishnan MK. Protective effect of *Calendula officinalis* Linn. flowers against 3-nitropropionic acid induced experimental Huntington's disease in rats. Drug and chemical toxicology. 2013 Oct 1;36(4):466-73. doi.org/10.3109/01480545.2013.776583

Shoibe M, Chy M, Uddin N, Alam M, Adnan M, Islam M, Nihar SW, Rahman N, Suez E. In vitro and in vivo biological activities of *Cissus adnata* (Roxb.). Biomedicines. 2017 Dec;5(4):63. doi:10.3390/biomedicines5040063

Shorinwa OA, Enemuoh AO, Uche FI. Anti-inflammatory and analgesic activities of methanol extracts of stem bark of *Anthocleista djalonensis* in wistar rats. Journal of Applied Pharmaceutical Science. 2015 Nov;5(11):117-20.

Shri R, Bora KS. Neuroprotective effect of methanolic extracts of *Allium cepa* on ischemia and reperfusion-induced cerebral injury. Fitoterapia. 2008 Feb 1;79(2):86-96. https://doi.org/10.1016/j.fitote.2007.06.013

Shrikanth VM, Janardhan B, More SS, MMuddapur U, KMirajkar K. In vitro anti snake venom potential of *Abutilon indicum* Linn leaf extracts against *Echis carinatus* (Indian saw scaled viper). Journal of Pharmacognosy and phytochemistry. 2014 May 1;3(1).

Shu XS, Gao ZH, Yang XL. Anti-inflammatory and anti-nociceptive activities of *Smilax china* L. aqueous extract. Journal of Ethnopharmacology. 2006 Feb 20;103(3):327-32. https://doi.org/10.1016/j.jep.2005.08.004

Shukla A, Rasik AM, Jain GK, Shankar R, Kulshrestha DK, Dhawan BN. In vitro and in vivo wound healing activity of asiaticoside isolated from *Centella asiatica*. Journal of ethnopharmacology. 1999 Apr 1;65(1):1-1.https://doi.org/10.1016/S0378-8741(98)00141-X

Shy SN, Chang WT, Lee SS, Liu KC. Production of triterpenes from cell suspension cultures of *Solanum incanum* L. The Chinese Pharmaceutical Journal. 2000 Feb 1;52(1):35-42.

Shytle DR, Tan J, C Bickford P, Rezai-Zadeh K, Hou L, Zeng J, R Sanberg P, D Sanberg C, S Alberte R, C Fink R, Roschek J. Optimized turmeric extract reduces β-amyloid and phosphorylated tau protein burden in Alzheimer's transgenic mice. Current Alzheimer Research. 2012 May 1;9(4):500-6. https://doi.org/10.2174/156720512800492459

Si KW, Liu JT, He LC, Li XK, Gou W, Liu CH, Li XQ. Effects of Caulophine on Caffeine-induced Cellular Injury and Calcium Homeostasis in Rat Cardiomyocytes. Basic & clinical pharmacology & toxicology. 2010 Dec;107(6):976-81. https://doi.org/10.1111/j.1742-7843.2010.00618.x

Siani AC, Souza MC, Henriques MG, Ramos MF. Anti-inflammatory activity of essential oils from *Syzygium cumini* and *Psidium guajava*. Pharmaceutical biology. 2013 Jul 1;51(7):881-7. https://doi.org/10.3109/13880209.2013.768675

Siddiqi HS, Mehmood MH, Rehman NU, Gilani AH. Studies on the antihypertensive and antidyslipidemic activities of Viola odorata leaves extract. Lipids in health and disease. 2012 Dec 1;11(1):6. https://doi.org/10.1186/1476-511X-11-6

Siddique S, Perveen Z, Firdaus-e-Baren S, Chaudhary MN. Antibacterial and antioxidant activities of leaves essential oils of seven *Eucalyptus* species grown in Pakistan. Journal of Animal & Plant Sciences, 28(1): 2018: 222-230.

Sikder MA, Hossian AN, Siddique AB, Ahmed M, Kaisar MA, Rashid MA. In vitro antimicrobial screening of four reputed Bangladeshi medicinal plants. Pharmacognosy Journal. 2011 Aug 1;3(24):72-6. https://doi.org/10.5530/pj.2011.24.14

Silprasit K, Seetaha S, Pongsanarakul P, Hannongbua S, Choowongkomon K. Anti-HIV-1 reverse transcriptase activities of hexane extracts from some Asian medicinal plants. Journal of Medicinal Plants Research. 2011 Sep 23;5(19):4899-4906.

Silva AA, Haraguchi SK, Cellet TS, Schuquel IT, Sarragiotto MH, Vidotti GJ, de Melo JO, Bersani-Amado CA, Zanoli K, Nakamura CV. Resveratrol-derived stilbenoids and biological activity evaluation of seed extracts of *Cenchrus echinatus* L. Natural product research. 2012 May 1;26(9):865-8.

Silva ER, Diedrich D, Bolzan RC, Giacomelli SR. Toxicological and pharmacological evaluation of *Discaria americana* Gillies & Hook (Rhamnaceae) in mice. Brazilian Journal of Pharmaceutical Sciences. 2012 Jun;48(2):273-80. http://dx.doi.org/10.1590/S1984-82502012000200011

Silva GC, Braga FC, Lima MP, Pesquero JL, Lemos VS, Cortes SF. *Hancornia speciosa* Gomes induces hypotensive effect through inhibition of ACE and increase on NO. Journal of Ethnopharmacology. 2011 Sep 1;137(1):709-13.

Silva J, Abebe W, Sousa SM, Duarte VG, Machado MI, Matos FJ. Analgesic and anti-inflammatory effects of essential oils of *Eucalyptus*. Journal of ethnopharmacology. 2003 Dec 1;89(2-3):277-83. https://doi.org/10.1016/j.jep.2003.09.007

Silva Junior IE, Cechinel Filho V, Zacchino SA, Lima JC, Martins DT. Antimicrobial screening of some medicinal plants from Mato Grosso Cerrado. Revista Brasileira de Farmacognosia. 2009 Mar;19(1B):242-8. https://doi.org/10.1590/S0102-695X2009000200011

Silva VG, Silva RO, Damasceno SR, Carvalho NS, Prudêncio RS, Aragão KS, Guimarães MA, Campos SA, Véras LM, Godejohann M, Leite JR. Anti-inflammatory and antinociceptive activity of epiisopiloturine, an imidazole alkaloid isolated from *Pilocarpus microphyllus*. Journal of natural products. 2013 Jun 28;76(6):1071-7. https://doi.org/10.1021/np400099m

Silveira CS, Martins FO, Costa CD, Romanos MT, Kaplan MA, Menezes FD. In vitro cytotoxic, antioxidant and antiviral effects of *Pterocaulon alopecuroides* and *Bidens segetum* extracts. Revista Brasileira de Farmacognosia. 2009 Jun;19(2A):343-8. https://doi.org/10.1590/S0102-695X2009000300001

Simirgiotis MJ, Bórquez J, Schmeda-Hirschmann G. Antioxidant capacity, polyphenolic content and tandem HPLC–DAD–ESI/MS profiling of phenolic compounds from the South American berries *Luma apiculata* and *L. chequén*. Food chemistry. 2013 Aug 15;139(1-4):289-99. https://doi.org/10.1016/j.foodchem.2013.01.089

Simões DM, Malheiros J, Antunes PE, Figueirinha A, Cotrim MD, Fonseca DA. Vascular activity of infusion and fractions of *Cymbopogon citratus* (DC) Stapf. in human arteries. Journal of ethnopharmacology. 2020 Aug 10;258:112947. https://doi.org/10.1016/j.jep.2020.112947

Simonyi A, Chen Z, Jiang J, Zong Y, Chuang DY, Gu Z, Lu CH, Fritsche KL, Greenlief CM, Rottinghaus GE, Thomas AL, Lubahn DB, Sun GY. Inhibition of microglial activation by elderberry extracts and its phenolic components. Life Sci. 2015 May 1;128:30-8. doi: 10.1016/j.lfs.2015.01.037. Epub 2015 Mar 2.

Simplice FH, Abdou BA, Abaissou N, Hervé H, Lucy MF, Annabel MN, Nyenti S, Neh P, Acha AE. Neuroprotective and memory improvement effects of a standardized extract of *Emilia coccinea* (SIMS) G. on animal models of anxiety and depression. Journal of Pharmacognosy and Phytochemistry. 2014;3(3):146-54.

Sindhu T, Rajamanikandan S, Srinivasan P. In vitro antioxidant and antibacterial activities of methanol extract of *Kyllinga nemoralis*. Indian journal of pharmaceutical sciences. 2014 Mar;76(2):170.

Sinei KA, Okalebo FA, Mugo HN, Mwalukumbi JM. An Investigation of the Antimicrobial Activity of *Acmella caulirhiza*. African Journal of Pharmacology and Therapeutics. 2013 Dec 31;2(4).

Singh A, D'Amico D, Andreux PA, Dunngalvin G, Kern T, Blanco-Bose W, Auwerx J, Aebischer P, Rinsch C. Direct supplementation with Urolithin A overcomes limitations of dietary exposure and gut microbiome variability in healthy adults to achieve consistent levels across the population. European Journal of Clinical Nutrition. 2021 Jun 11:1-2. https://doi.org/10.1038/s41430-021-00950-1

Singh AG, Kumar A, Tewari DD. An ethnobotanical survey of medicinal plants used in Terai forest of western Nepal. Journal of ethnobiology and ethnomedicine. 2012 Dec 1;8(1):19. https://doi.org/10.1186/1746-4269-8-19

Singh B, Bani S, Gupta DK, Chandan BK, Kaul A. Anti-inflammatory activity of 'TAF'an active fraction from the plant *Barleria prionitis* Linn. Journal of Ethnopharmacology. 2003 Apr 1;85(2-3):187-93.

Singh B, Chandan BK, Sharma N, Singh S, Khajuria A, Gupta DK. Adaptogenic activity of glyco-peptido-lipid fraction from the alcoholic extract of *Trichopus zeylanicus* Gaerten (part II). Phytomedicine. 2005 Jun 15;12(6-7):468-81. https://doi.org/10.1016/j.phymed.2005.01.009

Singh C, Singh S, Pande C, Tewari G, Pande V, Sharma P. Exploration of antimicrobial potential of essential oils of *Cinnamomum glanduliferum, Feronia elephantum, Bupleurum hamiltonii* and *Cyclospermum leptophyllum* against foodborne pathogens. Pharmaceutical biology. 2013 Dec 1;51(12):1607-10. doi.org/10.3109/13880209.2013.805234

Singh H, Ali SS, Khan NA, Mishra A, Mishra AK. Wound healing potential of *Cleome viscosa* Linn. seeds extract and isolation of active constituent. South African Journal of Botany. 2017 Sep 1;112:460-5. https://doi.org/10.1016/j.sajb.2017.06.026

Singh I, Mok M, Christensen AM, Turner AH, Hawley JA. The effects of polyphenols in olive leaves on platelet function. Nutrition, metabolism and cardiovascular diseases. 2008 Feb 1;18(2):127-32. https://doi.org/10.1016/j.numecd.2006.09.001

Singh JH, Alagarsamy V, Diwan PV, Kumar SS, Nisha JC, Reddy YN. Neuroprotective effect of *Alpinia galanga* (L.) fractions on Aβ (25–35) induced amnesia in mice. Journal of ethnopharmacology. 2011 Oct 31;138(1):85-91. doi: 10.1016/j.jep.2011.08.048.

Singh KP, Dwevedi AK, Dhakre G. Evaluation of antibacterial activities of *Chenopodium album* L. Int J Applied Biol and Pharm Tech 2011: 2(3): 398-401.

Singh M, Kumar V, Singh I, Gauttam V, Kalia AN. Anti-inflammatory activity of aqueous extract of *Mirabilis jalapa* Linn. leaves. *Pharmacognosy Res*. 2010;2(6):364–367. doi:10.4103/0974-8490.75456

Singh M, Pandey N, Agnihotri V, Singh KK, Pandey A. Antioxidant, antimicrobial activity and bioactive compounds of *Bergenia ciliata* Sternb.: A valuable medicinal herb of Sikkim Himalaya. Journal of traditional and complementary medicine. 2017 Apr 1;7(2):152-7. https://doi.org/10.1016/j.jtcme.2016.04.002

Singh M, Singh N, Khare PB, Rawat AK. Antimicrobial activity of some important *Adiantum* species used traditionally in indigenous systems of medicine. Journal of ethnopharmacology. 2008 Jan 17;115(2):327-9. https://doi.org/10.1016/j.jep.2007.09.018

Singh M, Srivastava S, Rawat AK. Antimicrobial activities of Indian *Berberis* species. Fitoterapia. 2007 Dec 1;78(7-8):574-6.

Singh MK, Dhongade H, Tripathi DK. Phytochemical analysis and antimicrobial activity of different extracts of *Orthosiphon pallidus*. Bangladesh J Pharmacol. 2017; 12: 93-94. doi: 10.3329/bjp.v12i1.30863

Singh R, Shushni MA, Belkheir A. Antibacterial and antioxidant activities of *Mentha piperita* L. Arabian Journal of Chemistry. 2015 May 1;8(3):322-8. https://doi.org/10.1016/j.arabjc.2011.01.019

Singh RK, Pandey BL. Further study of antiinflammatory effects of *Abies pindrow*. Phytotherapy Research: An International Journal Devoted to Medical and Scientific Research on Plants and Plant Products. 1997 Nov;11(7):535-7.

Singh RP, Chidambara Murthy KN, Jayaprakasha GK. Studies on the antioxidant activity of pomegranate (*Punica granatum*) peel and seed extracts using in vitro models. Journal of agricultural and food chemistry. 2002 Jan 2;50(1):81-6. https://doi.org/10.1021/jf010865b

Singh S, Kaur R, Sharma SK. Antinociceptive, antiinflammatory and antipyretic activities of *Rumex hastatus* D. don stem and roots. Der Pharmacia Sinica. 2013;4(3):95-102.

Singh S, Singh GB. Anti-inflammatory activity of *Lannea coromandelica* bark extract in rats. Phytotherapy Research. 1994 Aug;8(5):311-3. https://doi.org/10.1002/ptr.2650080513

Singh YN. Traditional medicine in Fiji: some herbal folk cures used by Fiji Indians. Journal of ethnopharmacology. 1986 Jan 1;15(1):57-88.

Singha PK, Roy S, Dey S. Antimicrobial activity of *Andrographis paniculata.* Fitoterapia. 2003 Dec 1;74(7-8):692-4. https://doi.org/10.1016/S0367-326X(03)00159-X

Singla AK, Pathak K. Topical antiinflammatory effects of *Euphorbia prostrata* on carrageenan-induced footpad oedema in mice. Journal of ethnopharmacology. 1990 Jul 1;29(3):291-4.

Sireeratawong S, Itharat A, Lerdvuthisopon N, Piyabhan P, Khonsung P, Boonraeng S, Jaijoy K. Anti-Inflammatory, Analgesic, and Antipyretic Activities of the Ethanol Extract of Piper interruptum Opiz. and Piper chaba Linn. ISRN pharmacology. 2012 Mar 18;2012. doi:10.5402/2012/480265

Sirohi B. Evaluation of the Anti-Inflammatory Activity of Hydroalcoholic Extract of *Dactylorhiza hatagirea* Roots and *Lavandula stoechas* Flower in Rats. EC Pharmacology and Toxicology. 2019;7:110-8.

Sivakumar PM, Sheshayan G, Doble M. Experimental and QSAR of acetophenones as antibacterial agents. Chemical biology & drug design. 2008 Oct;72(4):303-13. https://doi.org/10.1111/j.1747-0285.2008.00702.x

Smit HF, Kroes BH, Van den Berg AJ, Van der Wal D, Van den Worm E, Beukelman CJ, Van Dijk H, Labadie RP. Immunomodulatory and anti-inflammatory activity of *Picrorhiza scrophulariiflora*. Journal of Ethnopharmacology. 2000 Nov 1;73(1-2):101-9. https://doi.org/10.1016/S0378-8741(00)00268-3

Smita SS, Raj Sammi S, Laxman TS, Bhatta RS, Pandey R. Shatavarin IV elicits lifespan extension and alleviates Parkinsonism in *Caenorhabditis elegans*. Free radical research. 2017 Dec 2;51(11-12):954-69. https://doi.org/10.1080/10715762.2017.1395419

Smith NM. Ethnobotanical field notes from the Northern Territory, Australia. J Adelaide Bot Gard. 1991;14:1–65.

Soares DG, Godin AM, Menezes RR, Nogueira RD, Brito AM, Melo IS, Coura GM, Souza DG, Amaral FA, Paulino TP, Coelho MM. Anti-inflammatory and antinociceptive activities of azadirachtin in mice. Planta medica. 2014 Jun;80(08/09):630-6.

Sofidiya MO, Odukoya OA, Adedapo AA, Mbagwu HO, Afolayan AJ, Familoni OB. Investigation of the anti-inflammatory and antinociceptive activities of *Hymenocardia acida* Tul.(Hymenocardiaceae). African Journal of Biotechnology. 2010;9(49):8454-9. DOI: 10.5897/AJB10.744

Sofidiya MO, Odukoya OA, Familoni OB, Inya-Agha SI. Free radical scavenging activity of some Nigerian medicinal plant extracts. Pak J Biol Sci. 2006;9(8):1438-41.

Sofidiya MO, Oduwole B, Bamgbade E, Odukoya O, Adenekan S. Nutritional composition and antioxidant activities of *Curculigo pilosa* (Hypoxidaceae) rhizome. African Journal of Biotechnology. 2011;10(75):17275-81.

Soleimanpour S, Sedighinia FS, Safipour Afshar A, Zarif R, Asili J, Ghazvini K. Synergistic antibacterial activity of *Capsella bursa-pastoris* and *Glycyrrhiza glabra* against oral pathogens. Jundishapur Journal of Microbiology. 2013 Oct 1;6(8). doi: 10.5812/jjm.7262

Solomon RJ, Kallidass S, Vimalan J. Isolation, identification and study of antimicrobial property of a bioactive compound in an Indian medicinal plant *Acalypha indica* (Indian-nettle). World Journal of microbiology and biotechnology. 2005 Oct 1;21(6-7):1231-6.

Somchit MN, Sulaiman MR, Zuraini A, Samsuddin L, Somchit N, Israf DA, Moin S. Antinociceptive and antiinflammatory effects of *Centella asiatica*. Indian Journal of Pharmacology. 2004 Nov 1;36(6):377.

Somova LI, Shode FO, Moodley K, Govender Y. Cardiovascular and diuretic activity of kaurene derivatives of *Xylopia aethiopica* and *Alepidea amatymbica*. Journal of ethnopharmacology. 2001 Oct 1;77(2-3):165-74.

Son TG, Camandola S, Arumugam TV, Cutler RG, Telljohann RS, Mughal MR, Moore TA, Luo W, Yu QS, Johnson DA, Johnson JA. Plumbagin, a novel Nrf2/ARE activator, protects against cerebral ischemia. Journal of neurochemistry. 2010 Mar;112(5):1316-26. https://doi.org/10.1111/j.1471-4159.2009.06552.x

Song MY, Kang SY, Kang A, Hwang JH, Park YK, Jung HW. *Cinnamomum cassia* prevents high-fat diet-induced obesity in mice through the increase of muscle energy. The American journal of Chinese medicine. 2017 Jun 29;45(05):1017-31. https://doi.org/10.1142/S0192415X17500549

Song SH, Min HY, Han AR, Nam JW, Seo EK, Park SW, Lee SH, Lee SK. Suppression of inducible nitric oxide synthase by (−)-isoeleutherin from the bulbs of Eleutherine americana through the regulation of NF-κB activity. International immunopharmacology. 2009 Mar 1;9(3):298-302. https://doi.org/10.1016/j.intimp.2008.12.003

Song Y, Huang L, Yu J. Effects of blueberry anthocyanins on retinal oxidative stress and inflammation in diabetes through Nrf2/HO-1 signaling. Journal of neuroimmunology. 2016 Dec 15;301:1-6. https://doi.org/10.1016/j.jneuroim.2016.11.001

Song YW, Lee EY, Koh EM, Cha HS, Yoo B, Lee CK, Baek HJ, Kim HA, Suh II Y, Kang SW, Lee YJ. Assessment of comparative pain relief and tolerability of SKI306X compared with celecoxib in patients with rheumatoid arthritis: a 6-week, multicenter, randomized, double-blind, double-dummy, phase III, noninferiority clinical trial. Clinical therapeutics. 2007 May 1;29(5):862-73. doi: 10.1016/j.clinthera.2007.05.006

Sontakke S, Thawani V, Pimpalkhute S, Kabra P, Babhulkar S, Hingorani L. Open, randomized, controlled clinical trial of *Boswellia serrata* extract as compared to valdecoxib in osteoarthritis of knee. Indian Journal of Pharmacology. 2007 Jan 1;39(1):27.

Soodi M, Dashti A, Hajimehdipoor H, Akbari S, Ataei N. *Melissa officinalis* acidic fraction protects cultured cerebellar granule neurons against beta amyloid-induced apoptosis and oxidative stress. Cell Journal (Yakhteh). 2017;18(4):556.

Soodi M, Naghdi N, Hajimehdipoor H, Choopani S, Sahraei E. Memory-improving activity of *Melissa officinalis* extract in naïve and scopolamine-treated rats. Research in Pharmaceutical Sciences. 2014 Mar;9(2):107.

Sornwatana T, Roytrakul S, Wetprasit N, Ratanapo S. Brucin, an antibacterial peptide derived from fruit protein of Fructus Bruceae, *Brucea javanica* (L.) Merr. Letters in applied microbiology. 2013 Aug;57(2):129-36. https://doi.org/10.1111/lam.12085

Souissi M, Azelmat J, Chaieb K, Grenier D. Antibacterial and anti-inflammatory activities of cardamom (*Elettaria cardamomum*) extracts: potential therapeutic benefits for periodontal infections. Anaerobe. 2020 Feb 1;61:102089. https://doi.org/10.1016/j.anaerobe.2019.102089

Soundararajan P, Mahesh R, Ramesh T, Begum VH. Hypolipidemic activity of Aerva lanata on ethylene glycol induced calcium oxalate urolithiasis in rats. Pharmacology online 2007; 1:557-63.

Sourabie TS, Ouedraogo N, Sawadogo WR, Nikiema JB, Guissou IP, Nacoulma OG. Biological evaluation of anti-inflammatory and analgesic activities of *Argemone mexicana* Linn.(Papaveraceae) aqueous leaf extract. Int J Pharm Sci Res. 2012;3(9):451-8.

Speranza L, Franceschelli S, Pesce M, Reale M, Menghini L, Vinciguerra I, De Lutiis MA, Felaco M, Grilli A. Antiinflammatory effects in THP-1 cells treated with verbascoside. Phytotherapy Research. 2010 Sep;24(9):1398-404. doi: 10.1002/ptr.3173

Sreelekshmi R, Latha PG, Arafat MM, Arafat MM, Shyamal S, Shine VJ, Anuja GI, Suja SR, Rajasekharan S. 2007. Anti-inflammatory, analgesic and anti-lipid peroxidation studies on stem bark of *Ficus religiosa* Linn. Natural Product Radiance 6, 377–381.

Srianta I, Patria HD, Arisasmita JH, Epriliati I. Ethnobotany, nutritional composition and DPPH radical scavenging of leafy vegetables of wild *Paederia foetida* and *Erechtites hieracifolia*. International Food Research Journal. 2012;19(1):245-50.

Srinivasan D, Nathan S, Suresh T, Perumalsamy PL. Antimicrobial activity of certain Indian medicinal plants used in folkloric medicine. Journal of ethnopharmacology. 2001 Mar 1;74(3):217-20. https://doi.org/10.1016/S0378-8741(00)00345-7

Srinivasan K, Muruganandan S, Lal J, Chandra S, Tandan SK, Prakash VR. Evaluation of anti-inflammatory activity of *Pongamia pinnata* leaves in rats. Journal of ethnopharmacology. 2001 Dec 1;78(2-3):151-7. https://doi.org/10.1016/S0378-8741(01)00333-6

Srivastava P, Durgaprasad S. Burn wound healing property of *Cocos nucifera*: An appraisal. Indian J Pharmacol 2008;40:144-6. doi: 10.4103/0253-7613.43159

Srivastava S, Sammi SR, Laxman TS, Pant A, Nagar A, Trivedi S, Bhatta RS, Tandon S, Pandey R. Silymarin promotes longevity and alleviates Parkinson's associated pathologies in *Caenorhabditis elegans.* Journal of functional foods. 2017 Apr 1;31:32-43. https://doi.org/10.1016/j.jff.2017.01.029

Sriwiroch W, Chungsamarnyart N, Chantakru S, Pongket P, Saengprapaitip K, Pongchairerk U. The effect of *Pedilanthus tithymaloides* (L.) Poit crude extract on wound healing stimulation in mice. Agriculture and Natural Resources. 2010 Dec 30;44(6):1121-7.

Stagos D, Portesis N, Spanou C, Mossialos D, Aligiannis N, Chaita E, Panagoulis C, Reri E, Skaltsounis L, Tsatsakis AM, Kouretas D. Correlation of total polyphenolic content with antioxidant and antibacterial activity of 24 extracts from Greek domestic Lamiaceae species. Food and Chemical Toxicology. 2012 Nov 1;50(11):4115-24. https://doi.org/10.1016/j.fct.2012.08.033

Štajner D, Milić N, Lazić B, Mimica‐Dukić N. Study on antioxidant enzymes in *Allium cepa* L. and *Allium fistulosum* L. Phytotherapy Research: An International Journal Devoted to Pharmacological and Toxicological Evaluation of Natural Product Derivatives. 1998;12(S1):S15-7.

Stanely Mainzen Prince P, Menon VP. Antioxidant action of *Tinospora cordifolia* root extract in alloxan diabetic rats. Phytotherapy Research: An International Journal Devoted to Pharmacological and Toxicological Evaluation of Natural Product Derivatives. 2001 May;15(3):213-8. https://doi.org/10.1002/ptr.707

Stanisavljević IT, Stojičević SS, Veličković DT, Lazić ML, Veljković VB. Screening the antioxidant and antimicrobial properties of the extracts from plantain (*Plantago major* L.) leaves. Separation Science and Technology. 2008 Oct 8;43(14):3652-62. https://doi.org/10.1080/01496390802219091

Stanley MC, Ifeanyi OE, Nwakaego CC, Esther IO. Antimicrobial effects of *Chromolaena odorata* on some human pathogens. International Journal of current microbiology and applied sciences. 2014;3(3):1006-12.

Stefanović OD, Tešić JD, Čomić LR. *Melilotus albus* and *Dorycnium herbaceum* extracts as source of phenolic compounds and their antimicrobial, antibiofilm, and antioxidant potentials. journal of food and drug analysis. 2015 Sep 1;23(3):417-24. https://doi.org/10.1016/j.jfda.2015.01.003

Stepien M, Kujawska-Luczak M, Szulinska M, Kregielska-Narozna M, Skrypnik D, Suliburska J, Skrypnik K, Regula J, Bogdanski P. Beneficial dose-independent influence of *Camellia sinensis* supplementation on lipid profile, glycemia, and insulin resistance in an NaCl-induced hypertensive rat model. J Physiol Pharmacol. 2018 Apr 1;69(2). DOI: 10.26402/jpp.2018.2.13

Stickel F, Pöschl G, Seitz HK, Waldherr R, Hahn EG, Schuppan D. Acute hepatitis induced by Greater Celandine (*Chelidonium majus*). Scandinavian journal of gastroenterology. 2003 Jan 1;38(5):565-8. https://doi.org/10.1080/00365520310000942

Stingl C, Knapp H, Winterhalter P. 3, 4-Dihydroxy-7, 8-dihydro-β-ionone 3-O-β-D-glucopyranoside and other glycosidic constituents from apple leaves. Natural product letters. 2002 Jan 1;16(2):87-93.

Stockler-Pinto MB, Mafra D, Moraes C, Lobo J, Boaventura GT, Farage NE, Silva WS, Cozzolino SF, Malm O. Brazil nut (*Bertholletia excelsa*, HBK) improves oxidative stress and inflammation biomarkers in hemodialysis patients. Biological trace element research. 2014 Apr 1;158(1):105-12.

Stoilova I, Krastanov A, Stoyanova A, Denev P, Gargova S. Antioxidant activity of a ginger extract (Zingiber officinale). Food chemistry. 2007 Jan 1;102(3):764-70. https://doi.org/10.1016/j.foodchem.2006.06.023

Stojanović-Radić Z, Čomić L, Radulović N, Dekić M, Ranđelović V, Stefanović O. Chemical composition and antimicrobial activity of *Erodium* species: *E. ciconium* L., *E. cicutarium* L., and *E. absinthoides* Willd.(Geraniaceae). Chemical Papers. 2010 Jun 1;64(3):368-77. DOI: 10.2478/s11696-010-0014-x

Studzińska-Sroka E, Dudek-Makuch M, Chanaj-Kaczmarek J, Czepulis N, Korybalska K, Rutkowski R, Łuczak J, Grabowska K, Bylka W, Witowski J. Anti-inflammatory Activity and Phytochemical Profile of *Galinsoga parviflora* Cav. Molecules. 2018 Sep;23(9):2133. doi:10.3390/molecules23092133.

Stürner KH, Stellmann JP, Dörr J, Paul F, Friede T, Schammler S, Reinhardt S, Gellissen S, Weissflog G, Faizy TD, Werz O. A standardised frankincense extract reduces disease activity in relapsing-remitting multiple sclerosis (the SABA phase IIa trial). Journal of Neurology, Neurosurgery & Psychiatry. 2018 Apr 1;89(4):330-8. http://dx.doi.org/10.1136/jnnp-2017-317101

Subash AK, Augustine A. Hypolipidaemic effects of methanol extract of *Holoptelea integrifolia* (Roxb.) Planchon bark in diet-induced obese rats. Applied biochemistry and biotechnology. 2013 Jan 1;169(2):546-53.

Subash S, Essa MM, Braidy N, Al-Jabri A, Vaishnav R, Al-Adawi S, Al-Asmi A, Guillemin GJ. Consumption of fig fruits grown in Oman can improve memory, anxiety, and learning skills in a transgenic mice model of Alzheimer's disease. Nutritional neuroscience. 2016 Dec 12;19(10):475-83. https://doi.org/10.1179/1476830514Y.0000000131

Subashini R, Rakshitha SU. Phytochemical screening, antimicrobial activity and in vitro antioxidant investigation of methanolic extract of seeds from *Helianthus annuus* L. Chem Sci Rev Lett. 2012 Jan;1(1):30-4.

Subba B, Sharma A, Budhathoki A. Assessment of phytochemical content, antioxidant and antibacterial activities of three medicinal plants of Nepal. Journal of Medicinal Plants Research. 2016 Dec 10;10(45):829-37.

Subhashini N, Purnima S, Devi JA, Thirupathi AT, Lavanya N. Anti-inflammatory activity of *Erythrina stricta* Roxb in albino rats. Int J PharmTech Res. 2011 Apr;3(2):1014-8.

Subhashini T, Krishnaveni B, Srinivas Reddy C. Anti-inflammatory activity of leaf extracts of *Alternanthera sessilis*. Group. 2010 Mar;2(1):54-56.

Subramaniam G, Batcha AT, Wadhwani A. In vitro antiviral activity of BanLec against *Herpes simplex* viruses type 1 and 2. Bangladesh Journal of Pharmacology. 2020 Mar 8;15(1):11-8.

Subramanian M, Balakrishnan S. In vitro Marsilea anti-inflammatory and anti-venom activities of aerial parts of quadrifolia Linn. Asian Journal of Pharmacy and Pharmacology. 2019;5(1):73-7.

Subramanya MD, Pai SR, Upadhya V, Ankad GM, Bhagwat SS, Hegde HV. Total polyphenolic contents and in vitro antioxidant properties of eight *Sida* species from Western Ghats, India. *J Ayurveda Integr Med*. 2015;6(1):24–28. doi:10.4103/0975-9476.146544

Suffredini IB, Paciencia ML, Varella AD, Younes RN. Antibacterial activity of Brazilian Amazon plant extracts. Brazilian journal of Infectious diseases. 2006 Dec;10(6):400-2. https://doi.org/10.1590/S1413-86702006000600008

Sujith K, Darwin CR, Suba V. Memory-enhancing activity of *Anacyclus pyrethrum* in albino Wistar rats. Asian pacific journal of tropical disease. 2012 Aug 1;2(4):307-11. https://doi.org/10.1016/S2222-1808(12)60067-X

Sukandar EY, Sunderam N, Fidrianny I. Activity of *Kaempferia pandurata* (Roxb.) Rhizome Ethanol Extract Against MRSA, MIRCNS, MISSA, *Bacillus subtilis* and *Salmonella typhi*. Pakistan Journal of Biological Sciences. 2014;17(1):49-55.

Sulaiman MR, Perimal EK, Akhtar MN, Mohamad AS, Khalid MH, Tasrip NA, Mokhtar F, Zakaria ZA, Lajis NH, Israf DA. Anti-inflammatory effect of zerumbone on acute and chronic inflammation models in mice. Fitoterapia. 2010 Oct 1;81(7):855-8. https://doi.org/10.1016/j.fitote.2010.05.009

Sulaiman MR, Zakaria ZA, Chiong HS, Lai SK, Israf DA, Shah TA. Antinociceptive and anti-inflammatory effects of *Stachytarpheta jamaicensis* (L.) Vahl (Verbenaceae) in experimental animal models. Medical Principles and Practice. 2009;18(4):272-9.

Sulaiman MR, Zakaria ZA, Daud IA, Ng FN, Ng YC, Hidayat MT. Antinociceptive and anti-inflammatory activities of the aqueous extract of *Kaempferia galanga* leaves in animal models. Journal of natural medicines. 2008 Apr 1;62(2):221-7. https://doi.org/10.1007/s11418-007-0210-3

Suleiman MM, McGaw LI, Naidoo V, Eloff J. Detection of antimicrobial compounds by bioautography of different extracts of leaves of selected South African tree species. African Journal of Traditional, Complementary and Alternative Medicines. 2010;7(1).

Suleiman MM, Sulaiman MH, Iheme IS, Sukuntuni FS. Anti-inflammatory and Antinociceptive Effects of the Methanol Extract of *Indigofera arrecta* Hochst-Holl. in Rodents. African Journal of Pharmacology and Therapeutics. 2015 Jul 27;4(3).

Sumitra M, Manikandan P, Suguna L. Efficacy of *Butea monosperma* on dermal wound healing in rats. The International Journal of Biochemistry & Cell Biology. 2005 Mar 1;37(3):566-73. https://doi.org/10.1016/j.biocel.2004.08.003

Sun J, Gu YF, Su XQ, Li MM, Huo HX, Zhang J, Zeng KW, Zhang Q, Zhao YF, Li J, Tu PF. Anti-inflammatory lignanamides from the roots of *Solanum melongena* L. Fitoterapia. 2014 Oct 1;98:110-6.

Sun SF, Zhong HJ, Zhao YL, Ma XY, Luo JB, Zhu L, Zhang YT, Wang WX, Luo XD, Geng JW. Indole alkaloids of *Alstonia scholaris* (L.) R. Br. alleviated nonalcoholic fatty liver disease in mice fed with high-fat diet. Nat Prod Bioprospect. 2022 Apr 2;12(1):14. doi: 10.1007/s13659-022-00335-2.

Sun XB, Liu YP, Yang YY, Liu XY, Xiang DX. Anti-arthritic effect of total saponins from *Clematis henryi* Oliv. on collagen-induced arthritis rats. European Journal of Inflammation. 2016 Aug;14(2):71-7.

Sun Y, Ran H, Liou B, Quinn B, Zamzow M, Zhang W, Bielawski J, Kitatani K, Setchell KD, Hannun YA, Grabowski GA. Isofagomine in vivo effects in a neuronopathic Gaucher disease mouse. PLoS One. 2011 Apr 20;6(4):e19037. doi: 10.1371/journal.pone.0019037.

Sundu R, Mingvanish W, Arung ET, Kuspradini H, Khownium K. Antioxidant and antimicrobial activities of crude methanolic extract of *Polyscias guilfoylei* leaves. In Pure Applied Chemistry International Conference (PACCON), Thailand 2015 (pp. 161-165).

Sung YY, Kim YS, Kim HK. *Illicium verum* extract inhibits TNF-α-and IFN-γ-induced expression of chemokines and cytokines in human keratinocytes. Journal of ethnopharmacology. 2012 Oct 31;144(1):182-9. https://doi.org/10.1016/j.jep.2012.08.049

Sunilson AJ, Annuar F, Inez N, Asikin NS. Wound healing activity of *Hibiscus tiliaceus* L. Archives of Pharmacy Practice. 2012;3(1):70.

Süntar IP, Akkol EK, Yalçın FN, Koca U, Keleş H, Yesilada E. Wound healing potential of Sambucus ebulus L. leaves and isolation of an active component, quercetin 3-O-glucoside. Journal of ethnopharmacology. 2010 May 4;129(1):106-14. doi: 10.1016/j.jep.2010.01.051.

Surekha C, Ram TD. Antimicrobial potentiality of *Polyalthia cerasoides* leaf extracts and separation of compounds. Asian Journal of Chemistry. 2011 Apr 1;23(4):1661.

Susalit E, Agus N, Effendi I, Tjandrawinata RR, Nofiarny D, Perrinjaquet-Moccetti T, Verbruggen M. Olive (*Olea europaea*) leaf extract effective in patients with stage-1 hypertension: comparison with Captopril. Phytomedicine. 2011 Feb 15;18(4):251-8. doi: 10.1016/j.phymed.2010.08.016

Susilawati E, Aligita W, Adnyana IK, Sukmawati IK. Activity of Karehau (*Callicarpa longifolia* Lamk.) Leaves Ethanolic Extract as a Wound Healing. Journal of Pharmaceutical Sciences and Research. 2018 May 1;10(5):1243-7.

Suthiwong J, Thongsri Y, Yenjai C. A new furanocoumarin from the fruits of *Scaevola taccada* and antifungal activity against *Pythium insidiosum*. Natural product research. 2017 Feb 16;31(4):453-9.

Suzuki K, Tsubaki S, Fujita M, Koyama N, Takahashi M, Takazawa K. Effects of safflower seed extract on arterial stiffness. Vascular health and risk management. 2010;6:1007-1014. doi:10.2147/VHRM.S13998.

Swaroop A, Sinha AK, Chawla R, Arora R, Sharma RK, Kumar JK. Isolation and characterization of 1, 3-dicapryloyl-2-linoleoylglycerol: a novel triglyceride from berries of *Hippophae rhamnoides*. Chemical and pharmaceutical bulletin. 2005;53(8):1021-4.

Sweeney AP, Wyllie SG, Shalliker RA, Markham JL. Xanthine oxidase inhibitory activity of selected Australian native plants. Journal of Ethnopharmacology. 2001 May 1;75(2-3):273-7. https://doi.org/10.1016/S0378-8741(01)00176-3

Sweeney P, Park H, Baumann M, Dunlop J, Frydman J, Kopito R, et al. Protein misfolding in neurodegenerative diseases: implications and strategies. Transl Neurodegener. 2017;6:6. https://doi.org/10.1186/s40035-017-0077-5

Sydiskis RJ, Owen DG, Lohr JL, Rosler KH, Blomster RN. Inactivation of enveloped viruses by anthraquinones extracted from plants. Antimicrobial agents and chemotherapy. 1991 Dec 1;35(12):2463-6.

Szumny D, Kucharska AZ, Piorecki N, Szumny A, Sozanski T, Dziewiszek W, Chlebda E, Szelag A. Ocular hypotensive properties of *Cornus mas* extract and loganic acid. Acta Ophthalmologica. 2014 Sep;92. doi.org/10.1111/j.1755-3768.2014.F009.x

Tadesse S, Messele B, Seyoum A, Mazumder A, Bucar F, Asres K. Essential oil of *Otostegia integrifolia* benth: composition, antimicrobial and antioxidant activities. Ethip pharm J. 2011;29:1. http://dx.doi.org/10.4314/epj.v29i2.1

Tadtong S, Kanlayavattanakul M, Lourith N. Neuritogenic and neuroprotective activities of fruit residues. Natural product communications. 2013 Nov;8(11):1934578X1300801121. https://doi.org/10.1177/1934578X1300801121

Taghizadeh M, Maghaminejad F, Aghajani M, Rahmani M. The effect of tablet containing *Boswellia serrata* and *Melisa officinalis* extract on older adults' memory: A randomized controlled trial. Archives of gerontology and geriatrics. 2018 Mar 1;75:146-50. https://doi.org/10.1016/j.archger.2017.12.008

Tailor RH, Acland DP, Attenborough S, Cammue BP, Evans IJ, Osborn RW, Ray JA, Rees SB, Broekaert WF. A novel family of small cysteine-rich antimicrobial peptides from seed of *Impatiens balsamina* is derived from a single precursor protein. Journal of Biological Chemistry. 1997 Sep 26;272(39):24480-7. doi: 10.1074/jbc.272.39.244805.

Taiwe GS, Kouamou AL, Ambassa AR, Menanga JR, Tchoya TB, Dzeufiet PD. Evidence for the involvement of the GABA-ergic pathway in the anticonvulsant activity of the roots bark aqueous extract of *Anthocleista djalonensis* A. Chev.(Loganiaceae). Journal of Basic and Clinical Physiology and Pharmacology. 2017 Sep 26;28(5):425-35. https://doi.org/10.1515/jbcpp-2017-0048

Taiwo BJ, Igbeneghu OA. Antioxidant and antibacterial activities of flavonoid glycosides from *Ficus exasperata* Vahl-Holl (moraceae) leaves. African Journal of Traditional, Complementary and Alternative Medicines. 2014 Jul 9;11(3):97-101. doi:10.4314/ajtcam.v11i3.14

Taiwo IA, Odeigah PG, Jaja S, Mojiminiyi F. Cardiovascular effects of *Vernonia amygdalina* in rats and the implications for treatment of hypertension in diabetes. Researcher. 2010;2(1):76-9.

Tajadini H, Saifadini R, Choopani R, Mehrabani M, Kamalinejad M, Haghdoost AA. Herbal medicine Davaie Loban in mild to moderate Alzheimer's disease: A 12-week randomized double-blind placebo-controlled clinical trial. Complementary therapies in medicine. 2015 Dec 1;23(6):767-72. https://doi.org/10.1016/j.ctim.2015.06.009

Takaisi-Kikuni NB, Krüger D, Gnann W, Wecke J. Microcalorimetric and electron microscopic investigation on the effects of essential oil from *Cymbopogon densiflorus* on *Staphylococcus aureus*. Microbios. 1996;88(354):55-62. doi.org/10.1016/S0367-326X(99)00097-0

Talari S, Gundu C, Koila T, Nanna RS. In vitro free radical scavenging activity of different extracts of *Adansonia digitata* L. International Journal of Environment, Agriculture and Biotechnology. 2017;2(3).

Taleb-Contini SH, Salvador MJ, Watanabe E, Ito IY, Oliveira DC. Antimicrobial activity of flavonoids and steroids isolated from two *Chromolaena* species. Revista Brasileira de Ciências Farmacêuticas. 2003 Dec;39(4):403-8.

Tamariz-Angeles C, Olivera-Gonzales P, Santillán-Torres M. Antimicrobial, antioxidant and phytochemical assessment of wild medicinal plants from Cordillera Blanca (Ancash, Peru). Boletín Latinoamericano y del Caribe de Plantas Medicinales y Aromáticas. 2018 May 1;17(3):270-85.

Tambekar DH, Khante BS. Antibacterial evaluation of medicinal plants used by korkus in melghat forest against gastrointestinal infections. International Journal of Pharmaceutical Sciences and Research. 2011 Mar 1;2(3):577.

Tamokou deDJ, Chouna JR, Fischer-Fodor E, Chereches G, Barbos O, Damian G, Benedec D, Duma M, Efouet AP, Wabo HK, Kuiate JR. Anticancer and antimicrobial activities of some antioxidant-rich Cameroonian medicinal plants. PLoS One. 2013 Feb 11;8(2):e55880. https://doi.org/10.1371/journal.pone.0055880

Tamokou JD, Tala MF, Wabo HK, Kuiate JR, Tane P. Antimicrobial activities of methanol extract and compounds from stem bark of *Vismia rubescens*. Journal of ethnopharmacology. 2009 Jul 30;124(3):571-5. https://doi.org/10.1016/j.jep.2009.04.062

Tamokou JdeD, Mpetga DJ, Lunga PK, Tene M, Tane P, Kuiate JR. Antioxidant and antimicrobial activities of ethyl acetate extract, fractions and compounds from stem bark of *Albizia adianthifolia* (Mimosoideae). BMC complementary and alternative medicine. 2012 Dec;12(1):99.

Tan JB, Yap WJ, Tan SY, Lim YY, Lee SM. Antioxidant content, antioxidant activity, and antibacterial activity of five plants from the commelinaceae family. Antioxidants. 2014 Dec;3(4):758-69. doi:10.3390/antiox3040758

Tan NH, Fung SY, Sim SM, Marinello E, Guerranti R, Aguiyi JC. The protective effect of *Mucuna pruriens* seeds against snake venom poisoning. Journal of ethnopharmacology. 2009 Jun 22;123(2):356-8. https://doi.org/10.1016/j.jep.2009.03.025

Tanaka H, Sato M, Fujiwara S, Hirata M, Etoh H, Takeuchi H. Antibacterial activity of isoflavonoids isolated from *Erythrina variegata* against methicillin-resistant Staphylococcus aureus. Letters in applied microbiology. 2002 Dec;35(6):494-8. https://doi.org/10.1046/j.1472-765X.2002.01222.x

Tanaka J, Kadekaru T, Ogawa K, Hitoe S, Shimoda H, Hara H. Maqui berry (*Aristotelia chilensis*) and the constituent delphinidin glycoside inhibit photoreceptor cell death induced by visible light. Food chemistry. 2013 Aug 15;139(1-4):129-37. https://doi.org/10.1016/j.foodchem.2013.01.036

Tanaka S, Yamamoto K, Yamada K, Furuya K, Uyeno Y. Relationship of enhanced butyrate production by colonic butyrate-producing bacteria to immunomodulatory effects in normal mice fed an insoluble fraction of *Brassica rapa* L. Applied and environmental microbiology. 2016 May 1;82(9):2693-9. DOI: 10.1128/AEM.03343-15

Tandon VR, Gupta RK. An experimental evaluation of anticonvulsant activity of *Vitex-negundo*. Indian journal of physiology and pharmacology. 2005 Apr 21;49(2):199.

Tangjang S, Namsa ND, Aran C, Litin A. An ethnobotanical survey of medicinal plants in the Eastern Himalayan zone of Arunachal Pradesh, India. J Ethnopharmacol. 2011 Mar 8;134(1):18-25. doi: 10.1016/j.jep.2010.11.053.

Tantengco OA, Condes ML, Estadilla HH, Ragragio EM. Antibacterial activity of *Vitex parviflora* A. Juss. and *Cyanthillium cinereum* (L.) H. Rob. against human pathogens. Asian Pacific Journal of Tropical Disease. 2016 Dec 1;6(12):1004-6. https://doi.org/10.1016/S2222-1808(16)61173-8

Tantengco OA, Condes ML, Estadilla HH, Ragragio EM. Ethnobotanical Survey of Medicinal Plants used by Ayta Communities in Dinalupihan, Bataan, Philippines. Pharmacognosy Journal. 2018;10(5).

Tao JY, Zheng GH, Zhao L, Wu JG, Zhang XY, Zhang SL, Huang ZJ, Xiong FL, Li CM. Anti-inflammatory effects of ethyl acetate fraction from *Melilotus suaveolens* Ledeb on LPS-stimulated RAW 264.7 cells. Journal of ethnopharmacology. 2009 May 4;123(1):97-105. doi.org/10.1016/j.jep.2009.02.024

Tao Z, Chun-Yan H, Hua P, Bin-Bin Y, Xiaoping T. Phyllathin From Phyllanthus Amarus Ameliorates Epileptic Convulsion and Kindling Associated Post-Ictal Depression in Mice via Inhibition of NF-κB/TLR-4 Pathway. Dose-Response. 2020 Jul 31;18(3):1559325820946914. https://doi.org/10.1177/1559325820946914

Taranalli AD, Tipare SV, Kumar S, Torgal SS. Wound Healing Activity Of *Oxalis corniculata* Whole Plant Extract In Rats. Indian Journal of pharmaceutical sciences. 2004;66(4):444.

Tarannum S, Mohamed R, Vishwanath BS. Inhibition of testicular and *Vipera russelli* snake venom hyaluronidase activity by *Butea monosperma* (Lam) Kuntze stem bark. Natural product research. 2012 Sep 1;26(18):1708-11. https://doi.org/10.1080/14786419.2011.602829

Tarh JE, Iroegbu CU. In-vitro anti-bacterial activity of extracts of *Euphorbia abyssinica* (Desert Candle) Stem-Bark and Latex. Journal of Advances in Microbiology. 2017 May 1:1-3. doi.org/10.9734/JAMB/2017/32277

Tariq NP, Ifham S. Ethnobotanical Survey of Medicinal Plants in Yelagiri Hills of Tamil Nadu. Research Journal of Pharmacy and Technology. 2013;6(6):652-654.

Tasleem F, Azhar I, Ali SN, Perveen S, Mahmood ZA. Analgesic and anti-inflammatory activities of *Piper nigrum* L. Asian Pac J Trop Med. 2014 Sep;7S1:S461-8. doi: 10.1016/S1995-7645(14)60275-3.

Tata CM, Sewani-Rusike CR, Oyedeji OO, Gwebu ET, Mahlakata F, Nkeh-Chungag BN. Antihypertensive effects of the hydro-ethanol extract of *Senecio serratuloides* DC in rats. BMC Complement Altern Med. 2019 Feb 28;19(1):52. doi: 10.1186/s12906-019-2463-2.

Tatli II, Akkol EK, Yesilada E, Akdemir ZS. Antinociceptive and anti-inflammatory activities of seven endemic *Verbascum* species growing in Turkey. Pharmaceutical biology. 2008 Jan 1;46(10-11):781-8. https://doi.org/10.1080/13880200802315758

Tatsadjieu LN, Ngang JE, Ngassoum MB, Etoa FX. Antibacterial and antifungal activity of *Xylopia aethiopica*, *Monodora myristica*, *Zanthoxylum xanthoxyloıdes* and *Zanthoxylum leprieurii* from Cameroon. Fitoterapia. 2003 Jul 1;74(5):469-72. https://doi.org/10.1016/S0367-326X(03)00067-4

Taubert D, Roesen R, Lehmann C, Jung N, Schömig E. Effects of low habitual cocoa intake on blood pressure and bioactive nitric oxide: a randomized controlled trial. Jama. 2007 Jul 4;298(1):49-60. doi:10.1001/jama.298.1.49

Tavares L, McDougall GJ, Fortalezas S, Stewart D, Ferreira RB, Santos CN. The neuroprotective potential of phenolic-enriched fractions from four J*uniperus* species found in Portugal. Food chemistry. 2012 Nov 15;135(2):562-70. https://doi.org/10.1016/j.foodchem.2012.05.023

Tavares TG, Spindola H, Longato G, Pintado ME, Carvalho JE, Malcata FX. Antinociceptive and anti-inflammatory effects of novel dietary protein hydrolysate produced from whey by proteases of *Cynara cardunculus*. International dairy journal. 2013 Oct 1;32(2):156-62. doi.org/10.1016/j.idairyj.2013.05.010

Taye B, Giday M, Animut A, Seid J. Antibacterial activities of selected medicinal plants in traditional treatment of human wounds in Ethiopia. Asian Pacific Journal of Tropical Biomedicine. 2011 Oct 1;1(5):370-5.

Taylor JP, McKeith IG, Burn DJ, Boeve BF, Weintraub D, Bamford C, Allan LM, Thomas AJ, T O'Brien J. New evidence on the management of Lewy body dementia. The Lancet Neurology. 2020 Feb 1;19(2):157-69. https://doi.org/10.1016/S1474-4422(19)30153-X

Taylor RS, Hudson JB, Manandhar NP, Towers GH. Antiviral activities of medicinal plants of southern Nepal. Journal of Ethnopharmacology. 1996 Aug 1;53(2):105-10.

Tchouya GR, Foundikou H, Lebibi J. Phytochemical and in vitro antimicrobial evaluation of the stem bark of *Schumanniophyton magnificum* (Rubiaceae). J. Pharmacogn. Phytochem. 2014;3:185-9.

Tchouya GR, Souza A, Tchouankeu JC, Yala JF, Boukandou M, Foundikou H, Obiang GD, Boyom FF, Mabika RM, Zeuko E, Ndinteh DT. Ethnopharmacological surveys and pharmacological studies of plants used in traditional medicine in the treatment of HIV/AIDS opportunistic diseases in Gabon. Journal of ethnopharmacology. 2015 Mar 13;162:306-16.

Tedila H, Shanmugam AA. In-vitro antibacterial activity of *Ruta chalepensis* (Tenadam) and *Justicia shimperiana* (Senel) plants against some bacterial human pathogens. In-Vitro. 2019;63. DOI: 10.7176/JHMN

Teixeira B, Marques A, Ramos C, Serrano C, Matos O, Neng NR, Nogueira JM, Saraiva JA, Nunes ML. Chemical composition and bioactivity of different oregano (*Origanum vulgare*) extracts and essential oil. Journal of the Science of Food and Agriculture. 2013 Aug 30;93(11):2707-14. https://doi.org/10.1002/jsfa.6089

Teixeira MC, Lopes MJ, de Sousa-Júnior DL, Ribeiro AE, Pereira BS, de Aquino PE, de Aquino NC, Silveira ER, Leal LK, Viana GS. Evaluation of the Healing Potential of *Myracrodruon urundeuva* in Wounds Induced in Male Rats. Revista Brasileira de Farmacognosia. 2020 Mar 9; 30.:214–223. https://doi.org/10.1007/s43450-020-00025-5

Teke GN, Elisée KN, Roger KJ. Chemical composition, antimicrobial properties and toxicity evaluation of the essential oil of *Cupressus lusitanica* Mill. leaves from Cameroon. BMC complementary and alternative medicine. 2013 Dec;13(1):130.

Teke GN, Kuiate JR, Ngouateu OB, Gatsing D. Antidiarrhoeal and antimicrobial activities of *Emilia coccinea* (Sims) G. Don extracts. Journal of ethnopharmacology. 2007 Jun 13;112(2):278-83. https://doi.org/10.1016/j.jep.2007.03.007

Teklay A, Abera B, Giday M. An ethnobotanical study of medicinal plants used in Kilte Awulaelo District, Tigray Region of Ethiopia. Journal of ethnobiology and ethnomedicine. 2013 Dec 1;9(1):65. https://doi.org/10.1186/1746-4269-9-65

Teklehaymanot T. An ethnobotanical survey of medicinal and edible plants of Yalo Woreda in Afar regional state, Ethiopia. Journal of ethnobiology and ethnomedicine. 2017 Dec 1;13(1):40.

Téllez MA, Téllez AN, Vélez F, Ulloa JC. In vitro antiviral activity against rotavirus and astrovirus infection exerted by substances obtained from *Achyrocline bogotensis* (Kunth) DC.(Compositae). BMC complementary and alternative medicine. 2015 Dec;15(1):428.

Tene V, Malagón O, Finzi PV, Vidari G, Armijos C, Zaragoza T. An ethnobotanical survey of medicinal plants used in Loja and Zamora-Chinchipe, Ecuador. J Ethnopharmacol. 2007 Apr 20;111(1):63-81. doi: 10.1016/j.jep.2006.10.032.

Teneva D, Denkova-Kostova R, Goranov B, Hristova-Ivanova Y, Slavchev A, Denkova Z, Kostov G. Chemical composition, antioxidant activity and antimicrobial activity of essential oil from *Citrus aurantium* L zest against some pathogenic microorganisms. Zeitschrift für Naturforschung C. 2019 May 27;74(5-6):105-11.

Teng Y, Guo H, Liang Z, Shu Z, Li Z, Wu W. Ethnobotanical survey of medicinal plants and their utilization in Shaanxi Province, China. Journal of Medicinal Plants Research. 2011 May 4;5(9):1762-78. https://doi.org/10.5897/JMPR.9000595

Teodoro GR, Brighenti FL, Delbem AC, Delbem ÁC, Khouri S, Gontijo AV, Pascoal AC, Salvador MJ, Koga-Ito CY. Antifungal activity of extracts and isolated compounds from *Buchenavia tomentosa* on *Candida albicans* and *non-albicans*. Future microbiology. 2015 Jun;10(6):917-27.

Tepe B, Daferera D, Sokmen A, Sokmen M, Polissiou M. Antimicrobial and antioxidant activities of the essential oil and various extracts of *Salvia tomentosa* Miller (Lamiaceae). Food chemistry. 2005 May 1;90(3):333-40. https://doi.org/10.1016/j.foodchem.2003.09.013

Tesfaye M. Evaluation of in-vitro Antibacterial and Antifungal Activities of Crude extract and Solvent Fractions of the leaves of *Justicia schimperiana* Hochst. Ex Nees (Acanthaceae) (Doctoral dissertation, Addis Ababa University), 2017.

Teves MR, Wendel GH, Pelzer LE. *Jodina rhombifolia* leaves lyophilized aqueous extract decreases ethanol intake and preference in adolescent male Wistar rats. Journal of ethnopharmacology. 2015 Nov 4;174:11-6. https://doi.org/10.1016/j.jep.2015.07.046

Tewtrakul S, Yuenyongsawad S, Kummee S, Atsawajaruwan L. Chemical components and biological activities of volatile oil of *Kaempferia galanga* Linn. Songklanakarin J. Sci. Technol. 2005;27(2):53-07.

Thabit S, Handoussa H, Roxo M, El Sayed NS, Cestari de Azevedo B, Wink M. Evaluation of antioxidant and neuroprotective activities of *Cassia fistula* (L.) using the *Caenorhabditis elegans* model. *PeerJ*. 2018;6:e5159. Published 2018 Jul 13. doi:10.7717/peerj.5159

Thao NP, Luyen BT, Widowati W, Fauziah N, Maesaroh M, Herlina T, Manzoor Z, Ali I, Koh YS, Kim YH. Anti-inflammatory flavonoid C-glycosides from *Piper aduncum* leaves. Planta medica. 2016 Nov;82(17):1475-81. DOI: 10.1055/s-0042-108737

Thayil SM, Thyagarajan SP. PA-9: A Flavonoid Extracted from Plectranthus amboinicus Inhibits HIV-1 Protease. International Journal of Pharmacognosy and Phytochemical Research. 2016;8(6):1020-4.

Theo A, Masebe T, Suzuki Y, Kikuchi H, Wada S, Obi CL, Bessong PO, Usuzawa M, Oshima Y, Hattori T. *Peltophorum africanum*, a traditional South African medicinal plant, contains an anti HIV-1 constituent, betulinic acid. The Tohoku journal of experimental medicine. 2009;217(2):93-9. https://doi.org/10.1620/tjem.217.93

Thirupathi G, Pavan Kumar A, Narsimha Reddy Y. Effect of methanolic leaf extract and ethyl acetate fraction of *Butea monosperma* on β-amyloid (25–35) induced amnesia in laboratory animals. International Journal of Pharmaceutical and Biology Science. 2016;6:78-84.

Thomaz DV, Peixoto LF, de Oliveira TS, Fajemiroye JO, da Silva Neri HF, Xavier CH, Costa EA, dos Santos FC, de Souza Gil E, Ghedini PC. Antioxidant and Neuroprotective Properties of *Eugenia dysenterica* Leaves. Oxidative medicine and cellular longevity. 2018;2018. https://doi.org/10.1155/2018/3250908

Thomazzi SM, Silva CB, Silveira DC, Vasconcellos CL, Lira AF, Cambui EV, Estevam CS, Antoniolli AR. Antinociceptive and anti-inflammatory activities of *Bowdichia virgilioides* (sucupira). Journal of Ethnopharmacology. 2010 Feb 3;127(2):451-6.

Thombre R, Jagtap R, Patil N. Evaluation of phytoconstituents, antibacterial, antioxidant and cytotoxic activity of *Vitex negundo* L. and *Tabernaemontana divaricata* L. Int J Pharm Bio Sci. 2013;4(1):389-96.

Tian J, Li G, Liu Z, Fu F. Hydroxysafflor yellow A inhibits rat brain mitochondrial permeability transition pores by a free radical scavenging action. Pharmacology. 2008;82(2):121-6. https://doi.org/10.1159/000141653

Ticona LA, Sánchez ÁR, Gonzáles ÓO, Doménech MO. Antimicrobial compounds isolated from *Tropaeolum tuberosum*. Natural Product Research. 2020 Jan 7:1-5. https://doi.org/10.1080/14786419.2019.1710700

Timbrook J. Ethnobotany of Chumash indians, California, based on collections by John P. Harrington. Economic Botany. 1990 Apr 1;44(2):236-53. https://doi.org/10.1007/BF02860489

Tito A, Carola A, Bimonte M, Barbulova A, Arciello S, de Laurentiis F, Monoli I, Hill J, Gibertoni S, Colucci G, Apone F. A tomato stem cell extract, containing antioxidant compounds and metal chelating factors, protects skin cells from heavy metal-induced damages. International journal of cosmetic science. 2011 Dec;33(6):543-52. doi: 10.1111/j.1468-2494.2011.00668.x

Tiwari DK, Nagar HE, Dwivedi GA, Tripathi RK, Jena JI. Evaluation of anti-anxiety activity of *Plectranthus amboinicus* (Lour.) on rats. Asian Journal of Pharmaceutical and Clinical Research. 2012;5(4):110-3.

Tiwari OP, Tripathi YB. Antioxidant properties of different fractions of *Vitex negundo* Linn. Food Chemistry. 2007 Jan 1;100(3):1170-6. https://doi.org/10.1016/j.foodchem.2005.10.069

Tiwari P, Kumar K, Panik R, Pandey A, Pandey A, Sahu PK. Evaluation of aqueous extract of Roots of *Carica papaya* on wound healing activity in albino Rats. Journal of Chemical and Pharmaceutical Research. 2011;3(4):291-5.

Tiwari P, Nandy S. Screening of anti-inflammatory activity of *Mesua ferrea* Linn. Flower. International Journal of Biomedical Research. 2012;3(5).

Tiwari SK, Agarwal S, Seth B, Yadav A, Nair S, Bhatnagar P, Karmakar M, Kumari M, Chauhan LK, Patel DK, Srivastava V. Curcumin-loaded nanoparticles potently induce adult neurogenesis and reverse cognitive deficits in Alzheimer's disease model via canonical Wnt/β-catenin pathway. ACS nano. 2013 Dec 10;8(1):76-103.

Tohda C, Joyashiki E. Sominone enhances neurite outgrowth and spatial memory mediated by the neurotrophic factor receptor, RET. *Br J Pharmacol*. 2009;157(8):1427-1440. doi:10.1111/j.1476-5381.2009.00313.x

Tohma H, Gülçin İ, Bursal E, Gören AC, Alwasel SH, Köksal E. Antioxidant activity and phenolic compounds of ginger (*Zingiber officinale* Rosc.) determined by HPLC-MS/MS. Journal of food measurement and characterization. 2017 Jun;11(2):556-66. https://doi.org/10.1007/s11694-016-9423-z

Toledano-Zaragoza A, Ledesma MD. Addressing neurodegeneration in lysosomal storage disorders: Advances in Niemann Pick diseases. Neuropharmacology. 2020;171:107851.

Tom ENL, Mimb JRB, Nyunaï N, Bekono YF, Longo F, et al. (2018) Vasodilatory Effects of Aqueous Extract from *Harungana madagascariensis* Stem Bark in Isolated Rat Aorta: The Roles of Endothelium and K+ Channels. Am J Ethnomed Vol.5 No.1:8 DOI: 10.21767/2348-9502.10008

Tong R, Qi M, Yang Q, Li P, Wang D, Lan J, Wang Z, Yang L. Extract of *Plantago asiatica* L. seeds ameliorates hypertension in spontaneously hypertensive rats by inhibition of angiotensin converting enzyme. Frontiers in pharmacology. 2019;10:403. doi: 10.3389/fphar.2019.00403

Torrenegra RD, Pedrozo JA, Téllez AN, Cabeza G, Granados A, Méndez D. Chemistry and antifungal activity of *Pentacalia corymbosa* (Asteraceae-Senecioneae). Revista Latinoamericana de Química. 2000;28(1):31-4.

Tounekti T, Mahdhi M, Khemira H. Ethnobotanical Study of Indigenous Medicinal Plants of Jazan Region, Saudi Arabia. Evid Based Complement Alternat Med. 2019 Jun 2;2019:3190670. https://doi.org/10.1155/2019/3190670.

Touré A, Xu X, Michel T, Bangoura M. In vitro antioxidant and radical scavenging of Guinean kinkeliba leaf (*Combretum micranthum* G. Don) extracts. Natural Product Research. 2011 Jul 1;25(11):1025-36.

Trapp BD, Nave KA. Multiple sclerosis: an immune or neurodegenerative disorder? Annu Rev Neurosci. 2008;31:247-69. https://doi.org/10.1146/annurev.neuro.30.051606.094313

Trejo-Moreno C, Castro-Martínez G, Méndez-Martínez M, Jiménez-Ferrer JE, Pedraza-Chaverri J, Arrellín G, Zamilpa A, Medina-Campos ON, Lombardo-Earl G, Barrita-Cruz GJ, Hernández B. Acetone fraction from *Sechium edule* (Jacq.) Sw edible roots exhibits anti-endothelial dysfunction activity. Journal of ethnopharmacology. 2018 Jun 28;220:75-86. https://doi.org/10.1016/j.jep.2018.02.036

Tribuiani N, da Silva AM, Ferraz MC, Silva MG, Bentes AP, Graziano TS, dos Santos MG, Cogo JC, Varanda EA, Groppo FC, Cogo K. *Vellozia flavicans* Mart. ex Schult. hydroalcoholic extract inhibits the neuromuscular blockade induced by *Bothrops jararacussu* venom. BMC complementary and alternative medicine. 2014 Dec;14(1):1-9. https://doi.org/10.1186/1472-6882-14-48

Tribuiani N, Tavares MO, Santana MN, Fontana Oliveira IC, Amaral Filho JD, Silva MG, dos Santos MG, Cogo JC, Floriano RS, Cogo-Müller K, Oshima-Franco Y. Neutralising ability of *Terminalia fagifolia* extract (Combretaceae) against the in vitro neuromuscular effects of *Bothrops jararacussu* venom. Natural product research. 2017 Dec 2;31(23):2783-7.

Tripathi YB, Pandey SA, Shukla SD. Anti-platelet activating factor property of *Rubia cordifolia* Linn. Indian journal of experimental biology. 1993 Jun;31(6):533-5.

Tsai IL, Wun MF, Teng CM, Ishikawa T, Chen IS. Anti-platelet aggregation constituents from Formosan *Toddalia asiatica*. Phytochemistry. 1998 Aug 1;48(8):1377-82. DOI: 10.1016/s0031-9422(97)00678-x

Tsala DE, Mendimi NJ, Tatsimo S, Nnanga N, Edmond J. Effect of a methanol extract of *Allium cepa* Linn. on incisional wound healing in alloxan-induced diabetic mice. Appl Med Res. 2005;1(3):90-3. DOI: 10.5455/amr.20150427021827

Tshikalange TE, Meyer JJ, Hussein AA. Antimicrobial activity, toxicity and the isolation of a bioactive compound from plants used to treat sexually transmitted diseases. Journal of Ethnopharmacology. 2005 Jan 15;96(3):515-9. https://doi.org/10.1016/j.jep.2004.09.057

Tsilioni I, Taliou A, Francis K, Theoharides TC. Children with autism spectrum disorders, who improved with a luteolin-containing dietary formulation, show reduced serum levels of TNF and IL-6. Translational psychiatry. 2015 Sep;5(9):e647-. https://doi.org/10.1038/tp.2015.142

Tugume P, Kakudidi EK, Buyinza M, Namaalwa J, Kamatenesi M, Mucunguzi P, Kalema J. Ethnobotanical survey of medicinal plant species used by communities around Mabira Central Forest Reserve, Uganda. Journal of ethnobiology and ethnomedicine. 2016 Dec;12(1):5. doi.org/10.1186/s13002-015-0077-4

Tung YT, Wu MF, Lee MC, Wu JH, Huang CC, Huang WC. Antifatigue Activity and Exercise Performance of Phenolic-Rich Extracts from *Calendula officinalis*, *Ribes nigrum*, and *Vaccinium myrtillus*. Nutrients. 2019;11(8):1715. Published 2019 Jul 25. doi:10.3390/nu11081715

Turker AU, Camper ND. Biological activity of common mullein, a medicinal plant. Journal of ethnopharmacology. 2002 Oct 1;82(2-3):117-25. https://doi.org/10.1016/S0378-8741(02)00186-1

Turner RS, Thomas RG, Craft S, Van Dyck CH, Mintzer J, Reynolds BA, Brewer JB, Rissman RA, Raman R, Aisen PS. A randomized, double-blind, placebo-controlled trial of resveratrol for Alzheimer disease. Neurology. 2015 Oct 20;85(16):1383-91. DOI: https://doi.org/10.1212/WNL.0000000000002035

Twardowschy A, Freitas CS, Baggio CH, Mayer B, dos Santos AC, Pizzolatti MG, Zacarias AA, dos Santos EP, Otuki MF, Marques MC. Antiulcerogenic activity of bark extract of *Tabebuia avellanedae,* Lorentz ex Griseb. Journal of ethnopharmacology. 2008 Aug 13;118(3):455-9.

Twilley D, Langhansová L, Palaniswamy D, Lall N. Evaluation of traditionally used medicinal plants for anticancer, antioxidant, anti-inflammatory and anti-viral (HPV-1) activity. South African Journal of Botany. 2017 Sep 1;112:494-500. https://doi.org/10.1016/j.sajb.2017.05.021

Twohig D, Nielsen HM. α-synuclein in the pathophysiology of Alzheimer's disease. Molecular neurodegeneration. 2019 Dec;14(1):1-9. https://doi.org/10.1186/s13024-019-0320-x

Twumasi MA, Tandoh A, Mante PK, Ekuadzi E, Boakye-Gyasi ME, Benneh CK, Kumadoh D, Woode E. Leaves and stems of *Capparis erythrocarpos*, more sustainable than roots, show antiarthritic effects. Journal of ethnopharmacology. 2019 Jun 28;238:111890. doi.org/10.1016/j.jep.2019.111890

Uawonggul N, Chaveerach A, Thammasirirak S, Arkaravichien T, Chuachan C, Daduang S. Screening of plants acting against *Heterometrus laoticus* scorpion venom activity on fibroblast cell lysis. Journal of ethnopharmacology. 2006 Jan 16;103(2):201-7

Uddin B, Nahar T, Khalil MI, Hossain S. In vitro antibacterial activity of the ethanol extract of *Paederia foetida* L.(Rubiaceae) leaves. Bangladesh J. life Sci. 2007 Dec;19(2):141-3.

Uddin G, Alam M, Siddiqui BS, Rauf A. Preliminary phytochemical profile and antibacterial evaluation of *Viburnum grandiflorum* Wall. Glob J Pharm. 2013;7(2):133-7.

Uddin ME, Islam AM, Chowdhury MA, Rahman MK, Islam MS, Islam MR. Sedative and analgesic activities of *Ludwigia repens*. Phytopharmacology. 2012;2(2):202-11.

Uddin MZ, Hassan MA, Sultana M. Ethnobotanical survey of medicinal plants in Phulbari Upazila of Dinajpur District, Bangladesh. Bangladesh Journal of Plant Taxonomy. 2006;13(1):63-8. https://doi.org/10.3329/bjpt.v13i1.596

Uddin SJ, Rouf R, Shilpi JA, Alamgir M, Nahar L, Sarker SD. Screening of some Bangladeshi medicinal plants for in vitro antibacterial activity. Oriental Pharmacy and Experimental Medicine. 2008;8(3):316-21.

Uddin SN, Ali ME, Yesmin MN. Antioxidant and antibacterial activities of *Senna tora* Roxb. American Journal of Plant Physiology. 2008;3(2):096-100. DOI: 10.3923/ajpp.2008.96.100

Uddin SN, Yesmin MN, Pramanik MK, Akond MA. Anti-inflammatory, antinociceptive and diuretic activities of *Trema orientalis* Linn. Oriental Pharmacy and Experimental Medicine. 2009;9(4):320-5.

Uddin SN. Antioxidant and antibacterial activities of *Trema orientalis* Linn: an indigenous medicinal plant of indian subcontinent. Oriental Pharmacy and Experimental Medicine. 2008;8(4):395-9. doi.org/10.3742/OPEM.2008.8.4.395

Udegbunam RI, Asuzu UI, Kene RO, Udegbunam SO, Nwaehujor C. Anti-nociceptive, anti-inflammatory and anti-oxidant effects of the methanol leaf extract of *Sterculia tragacantha* lindl. Journal of Pharmacology and Toxicology. 2011;12(6):516-24. DOI: 10.3923/jpt.2011.516.524

Udoh FV. Uterine muscle reactivity to repeated administration and phytochemistry of the leaf and seed extracts of *Piper guineense*. Phytotherapy Research: An International Journal Devoted to Pharmacological and Toxicological Evaluation of Natural Product Derivatives. 1999 Feb;13(1):55-8. https://doi.org/10.1002/(SICI)1099-1573(199902)13:1<55::AID-PTR401>3.0.CO;2-3

Udoumoh AF, Eze CA, Chah KF, Etuk EU. Antibacterial and surgical wound healing properties of ethanolic leaf extracts of *Swietenia mahogoni* and *Carapa procera*. Asian J Trad Med. 2011 Dec 20;6(6).

Ueda H, Yamazaki C, Yamazaki M. Luteolin as an anti-inflammatory and anti-allergic constituent of *Perilla frutescens*. Biological and Pharmaceutical Bulletin. 2002;25(9):1197-202. https://doi.org/10.1248/bpb.25.1197

Ueki T, Akaishi T, Okumura H, Abe K. Extract from *Nandina domestica* inhibits lipopolysaccharide-induced cyclooxygenase-2 expression in human pulmonary epithelial A549 cells. Biological and Pharmaceutical Bulletin. 2012 Jul 1;35(7):1041-7. https://doi.org/10.1248/bpb.b110709

Uliana MP, Da Silva AG, Fronza M, Scherer R. In vitro antioxidant and antimicrobial activities of *Costus spicatus* swartz used in folk medicine for urinary tract infection in Brazil. Lat Am J Pharm. 2015 Jan 1;34:766-72.

Ullah R, Shahat AA, Alqahtani AS, Almarfadi OM, Alharbi MS, Ahamad SR, Ali SS. Anti-Inflammatory, Antipyretic and Analgesic Potential of *Zilla spinosa* in Animal Model. Indian Journal of Animal Research. 2020 Jul 1;54(7).

Umar A, Imam G, Yimin W, Kerim P, Tohti I, Berké B, Moore N. Antihypertensive effects of *Ocimum basilicum* L.(OBL) on blood pressure in renovascular hypertensive rats. Hypertension research. 2010 Jul;33(7):727-30. https://doi.org/10.1038/hr.2010.64

Umesh MK, Sanjeevkumar CB, Hanumantappa BN, Ramesh L. Evaluation of in vitro anti-thrombolytic activity and cytotoxicity potential of *Typha angustifolia* L leaves extracts. Int J Pharm Pharm Sci. 2014;6(5):81-5.

Umezu T, Sakata A, Ito H. Ambulation-promoting effect of peppermint oil and identification of its active constituents. Pharmacology Biochemistry and Behavior. 2001 Jul 8;69(3-4):383-90. https://doi.org/10.1016/S0091-3057(01)00543-3

Une HD, Sarveiya VP, Pal SC, Kasture VS, Kasture SB. Nootropic and anxiolytic activity of saponins of *Albizzia lebbeck* leaves. Pharmacology Biochemistry and Behavior. 2001 Jul 8;69(3-4):439-44. https://doi.org/10.1016/S0091-3057(01)00516-0

Unni BG, Borah A, Wann SB, Singh HR, Devi B, Bhattacharjee M. Phytochemical and antibacterial study of traditional medicinal plants of north east India on *Escherichia coli*. Asian J Exp Sci. 2009;23(1):103-8.

Upadhyay A, Chompoo J, Kishimoto W, Makise T, Tawata S. HIV-1 integrase and neuraminidase inhibitors from *Alpinia zerumbet*. Journal of agricultural and food chemistry. 2011 Apr 13;59(7):2857-62. https://doi.org/10.1021/jf104813k

Upadhyay G, Khoshla S, Kosuru R, Singh S. Anxiolytic, antidepressant, and antistress activities of the aqueous extract of *Cinnamomum tamala* Nees and Eberm in rats. Indian J Pharmacol. 2016 Sep-Oct;48(5):555-561. doi: 10.4103/0253-7613.190752.

Urso V, Signorini MA, Tonini M, Bruschi P. Wild medicinal and food plants used by communities living in Mopane woodlands of southern Angola: Results of an ethnobotanical field investigation. J Ethnopharmacol. 2016 Jan 11;177:126-39. doi: 10.1016/j.jep.2015.11.041.

Usman LA, Hamid AA, Muhammad NO, Olawore NO, Edewor TI, Saliu BK. Chemical constituents and anti-inflammatory activity of leaf essential oil of Nigerian grown *Chenopodium album* L. *EXCLI J*. 2010;9:181–186. Published 2010 Dec 7.

Vadnere GP, Pathan AR, Singhai AK, Kulkarni BU, Hundiwale JC. Anti-stress and anti-allergic effect of *Actiniopteris radiata* in some aspects of asthma. Pakistan journal of pharmaceutical sciences. 2013 Jan 1;26(1).

Vaghasiya Y, Chanda S. Screening of methanol and acetone extracts of fourteen Indian medicinal plants for antimicrobial activity. Turkish Journal of Biology. 2007 Dec 12;31(4):243-8.

Vaghasiya Y, Nair R, Chanda S. Investigation of Some Piper Species for Anti—Bacterial and Anti—Inflammatory Property. International journal of Pharmacology. 2007;3(5):400-5.

Valarezo E, Rosales J, Morocho V, Cartuche L, Guaya D, Ojeda-Riascos S, Armijos C, González S. Chemical composition and biological activity of the essential oil of *Baccharis obtusifolia* Kunth from Loja, Ecuador. Journal of Essential Oil Research. 2015 May 4;27(3):212-6. https://doi.org/10.1080/10412905.2015.1007217

Valls-Pedret C, Sala-Vila A, Serra-Mir M, Corella D, De la Torre R, Martínez-González MÁ, Martínez-Lapiscina EH, Fitó M, Pérez-Heras A, Salas-Salvadó J, Estruch R. Mediterranean diet and age-related cognitive decline: a randomized clinical trial. JAMA internal medicine. 2015 Jul 1;175(7):1094-103. doi:10.1001/jamainternmed.2015.1668

Van Puyvelde L, Nyirankuliza S, Panebianco R, Boily Y, Geizer I, Sebikali B, De Kimpe N, Schamp N. Active principles of *Tetradenia riparia*. I. Antimicrobial activity of 8 (14), 15-sandaracopimaradiene-7α, 18-diol. Journal of ethnopharmacology. 1986 Sep 1;17(3):269-75. https://doi.org/10.1016/0378-8741(86)90115-7

Vargas Maji NA. Determinación de la actividad antiinflamatoria de *Campyloneurum amphostenon* mediante inhibición de edema plantar inducido por carragenina en ratas *Rattus norvegicus.* Escuela Superior Politécnica de Chimborazo, Riobamba, Ecuador, 2017. http://dspace.espoch.edu.ec/handle/123456789/6692

Vasquez J, Jiménez SL, Gómez IC, Rey JP, Henao AM, Marín DM, Romero JO, Alarcón JC. Snakebites and ethnobotany in the eastern region of Antioquia, Colombia—the traditional use of plants. Journal of Ethnopharmacology. 2013 Mar 27;146(2):449-55.

Vega-Gálvez A, Stucken K, Cantuarias C, Lamas F, García V, Pastén A. Antimicrobial properties of papaya (*Vasconcellea pubescens*) subjected to low-temperature vacuum dehydration. Innovative Food Science & Emerging Technologies. 2020 Nov 18:102563. https://doi.org/10.1016/j.ifset.2020.102563

Veiga VF, Rosas EC, Carvalho MV, Henriques MD, Pinto AC. Chemical composition and anti-inflammatory activity of copaiba oils from *Copaifera cearensis* Huber ex Ducke, *Copaifera reticulata* Ducke and *Copaifera multijuga* Hayne—A comparative study. Journal of Ethnopharmacology. 2007 Jun 13;112(2):248-54. doi.org/10.1016/j.jep.2007.03.005

Velaga MK, Daughtry LK, Jones AC, Yallapragada PR, Rajanna S, Rajanna B. Attenuation of lead-induced oxidative stress in rat brain, liver, kidney and blood of male Wistar rats by *Moringa oleifera* seed powder. Journal of Environmental Pathology, Toxicology and Oncology. 2014;33(4).

Velagapudi R, Ajileye OO, Okorji U, Jain P, Aderogba MA, Olajide OA. Agathisflavone isolated from *Anacardium occidentale* suppresses SIRT1-mediated neuroinflammation in BV2 microglia and neurotoxicity in APPS we-transfected SH-SY5Y cells. Phytotherapy Research. 2018 Oct;32(10):1957-66. https://doi.org/10.1002/ptr.6122.

Velagapudi R, Lepiarz I, El-Bakoush A, Katola FO, Bhatia H, Fiebich BL, Olajide OA. Induction of Autophagy and Activation of SIRT-1 Deacetylation Mechanisms Mediate Neuroprotection by the Pomegranate Metabolite Urolithin A in BV2 Microglia and Differentiated 3D Human Neural Progenitor Cells. Molecular nutrition & food research. 2019 May;63(10):1801237. doi: 10.1002/mnfr.201801237.

Velander P, Wu L, Henderson F, Zhang S, Bevan DR, Xu B. Natural product-based amyloid inhibitors. *Biochem Pharmacol*. 2017;139:40-55. doi: 10.1016/j.bcp.2017.04.004.

Velichkova K, Sirakov I, Rusenova N, Beev G, Denev S, Valcheva N, Dinev T. In Vitro Antimicrobial Activity on *Lemna minuta*, *Chlorella vulgaris* and *Spirulina* Sp. extracts. Fresenius Environmental Bulletin. 2018 Jan 1;27(8):5736-41.

Venkata SP, Murali MC, da Silva JA, Raju BA, Sravani R. Screening the antimicrobial and antioxidant potential of *Ventilago denticulata*, *Scolopia crenata* and *Rivea hypocrateriformis* from maredumilli forest, india. Med. Aromat. Plant Sci. Biotechnol. 2012;6:58-62.

Venkataraman S, Ramanujam TR, Venkatasubbu VS. Antifungal activity of the alcoholic extract of coconut shell-Cocos nucifera Linn. Journal of ethnopharmacology. 1980;2(3):291-3. DOI: 10.1016/S0378-8741(80)81007-5.

Vera Abanto MN, Zavaleta Minchola MM. Comparación de la actividad antiinflamatoria in vitro de los extractos de hojas y flores de *Echeveria peruviana* Meyen. Bachelor Thesis, Universidad Nacional de Trujillo.Facultad de Farmacia y Bioquímica, Peru. 2019. http://dspace.unitru.edu.pe/handle/UNITRU/14259.

Verhoeven V, Van der Auwera A, Van Gaal L, Remmen R, Apers S, Stalpaert M, Wens J, Hermans N. Can red yeast rice and olive extract improve lipid profile and cardiovascular risk in metabolic syndrome?: a double blind, placebo controlled randomized trial. BMC complementary and alternative medicine. 2015 Dec 1;15(1):52. doi: 10.1186/s12906-015-0576-9.

Verma N, Tripathi SK, Sahu D, Das HR, Das RH. Evaluation of inhibitory activities of plant extracts on production of LPS-stimulated pro-inflammatory mediators in J774 murine macrophages. Molecular and cellular biochemistry. 2010 Mar 1;336(1-2):127-35. https://doi.org/10.1007/s11010-009-0263-6.

Veronica SA, Cheruiyot KS, Bosibori MJ, Munene IM, Murugi NJ, Piero NM. Antiinflammatory, analgesic and antipyretic effects of dichloromethane stem bark extract of *Acacia mellifera*. J. Phytopharmacol. 2017 Sep;6(4):239-46.

Verotta L, Lovaglio E, Vidari G, Finzi PV, Neri MG, Raimondi A, Parapini S, Taramelli D, Riva A, Bombardelli E. 4-Alkyl- and 4-phenylcoumarins from *Mesua ferrea* as promising multidrug resistant antibacterials. Phytochemistry. 2004 Nov 1;65(21):2867-79.

Verrastro BR, Torres AM, Ricciardi G, Teibler P, Maruñak S, Barnaba C, Larcher R, Nicolini G, Dellacassa E. The effects of *Cissampelos pareira* extract on envenomation induced by *Bothropsdiporus* snake venom. Journal of ethnopharmacology. 2018 Feb 15;212:36-42. https://doi.org/10.1016/j.jep.2017.09.015.

Vetrichelvan T, Jegadeesan M. Effect of alcoholic extract of *Achyranthes bidentata* blume on acute and sub acute inflammation. Indian journal of pharmacology. 2002 Mar 1;34(2):115-8.

Viana GS, Bandeira MA, Matos FJ. Analgesic and antiinflammatory effects of chalcones isolated from *Myracrodruon urundeuva* Allemão. Phytomedicine. 2003 Jan 1;10(2-3):189-95. https://doi.org/10.1078/094471103321659924.

Viana GS, do Vale TG, Rao VS, Matos FJ. Analgesic and antiinflammatory effects of two chemotypes of *Lippia alba*: a comparative study. Pharmaceutical biology. 1998 Jan 1;36(5):347-51. doi.org/10.1076/phbi.36.5.347.4646.

Victoria FN, Lenardão EJ, Savegnago L, Perin G, Jacob RG, Alves D, da Silva WP, da Motta AD, da Silva Nascente P. Essential oil of the leaves of *Eugenia uniflora* L.: antioxidant and antimicrobial properties. Food and chemical toxicology. 2012 Aug 1;50(8):2668-74. https://doi.org/10.1016/j.fct.2012.05.002.

Vidal V, Potterat O, Louvel S, Hamy F, Mojarrab M, Sanglier JJ, Klimkait T, Hamburger M. Library-based discovery and characterization of daphnane diterpenes as potent and selective HIV inhibitors in *Daphne gnidium*. Journal of natural products. 2012 Mar 23;75(3):414-9. https://doi.org/10.1021/np200855d.

Vidrio H, Soto B, García F, Méndez S. Hypotensive activity of extracts of *Solanum marginatum* in the rat. Planta medica. 1988 Apr;54(02):111-3. DOI: 10.1055/s-2006-962363.

Vidya SM, Krishna V, Manjunatha BK, Bharath BR, Rajesh KP, Manjunatha H, Mankani KL. Wound healing phytoconstituents from seed kernel of *Entada pursaetha* DC. and their molecular docking studies with glycogen synthase kinase 3-β. Medicinal Chemistry Research. 2012a Oct 1;21(10):3195-203.

Vidya SM, Krishna V, Manjunatha BK, Rajesh KP, Bharath BR, Manjunatha H. Antibacterial and molecular docking studies of entagenic acid, a bioactive principle from seed kernel of *Entada pursaetha* DC. Medicinal Chemistry Research. 2012b Jul 1;21(7):1016-22.

Vidyarthi S, Samant SS, Sharma P. Traditional and indigenous uses of medicinal plants by local residents in Himachal Pradesh, North Western Himalaya, India. International Journal of Biodiversity Science, Ecosystem Services & Management. 2013 Sep 1;9(3):185-200.

Viecili PR, Borges DO, Kirsten K, Malheiros J, Viecili E, Melo RD, Trevisan G, da Silva MA, Bochi GV, Moresco RN, Klafke JZ. Effects of *Campomanesia xanthocarpa* on inflammatory processes, oxidative stress, endothelial dysfunction and lipid biomarkers in hypercholesterolemic individuals. Atherosclerosis. 2014 May 1;234(1):85-92. https://doi.org/10.1016/j.atherosclerosis.2014.02.010.

Vieira Jr G, Ferreira PM, Matos LG, Ferreira EC, Rodovalho W, Ferri PH, Ferreira HD, Costa EA. Anti-inflammatory effect of *Solanum lycocarpum* fruits. Phytotherapy Research: An International Journal Devoted to Pharmacological and Toxicological Evaluation of Natural Product Derivatives. 2003 Sep;17(8):892-6.

Vigh S, Cziaky Z, Sinka LT, Pribac C, Moş L, Turcuş V, Remenyik J, Mathe E. Comparative chemomapping of phytoconstituents from different extracts of globe artichoke-*Cynara scolymus* L. Studia Universitatis Babes-Bolyai, Chemia. 2017 Jan 1;62.

Vijaya Kumar S, Sankar P, Varatharajan R. Anti-inflammatory activity of roots of *Achyranthes aspera*. Pharmaceutical Biology. 2009 Oct 1;47(10):973-5. https://doi.org/10.1080/13880200902967979

Viji M, Murugesan S. Phytochemical analysis and antibacterial activity of medicinal plant *Cardiospermum halicacabum* Linn. J Phytol. 2010 Oct 5;2(1):68-77.

Viktorová J, Kumar R, Řehořová K, Hoang L, Ruml T, Figueroa CR, Valdenegro M, Fuentes L. Antimicrobial Activity of Extracts of Two Native Fruits of Chile: Arrayan (*Luma apiculata*) and Peumo (*Cryptocarya alba*). Antibiotics. 2020 Aug;9(8):444. https://doi.org/10.3390/antibiotics9080444

Vilela FC, Bitencourt AD, Cabral LD, Franqui LS, Soncini R, Giusti-Paiva A. Anti-inflammatory and antipyretic effects of *Sonchus oleraceus* in rats. Journal of ethnopharmacology. 2010 Feb 17;127(3):737-41.

Vilela TC, Leffa DD, Damiani AP, Damazio DD, Manenti AV, Carvalho TJ, Ramlov F, Amaral PA, ANDRADE VM. *Hibiscus acetosella* extract protects against alkylating agent-induced DNA damage in mice. Anais da Academia Brasileira de Ciências. 2018 Sep;90(3):3165-74. doi.org/10.1590/0001-3765201820180144

Viljoen AM, Subramoney SV, Van Vuuren SF, Başer KH, Demirci B. The composition, geographical variation and antimicrobial activity of *Lippia javanica* (Verbenaceae) leaf essential oils. Journal of ethnopharmacology. 2005 Jan 4;96(1-2):271-7.

Vinson JA, Demkosky CA, Navarre DA, Smyda MA. High-antioxidant potatoes: acute in vivo antioxidant source and hypotensive agent in humans after supplementation to hypertensive subjects. Journal of agricultural and food chemistry. 2012 Jul 11;60(27):6749-54. https://doi.org/10.1021/jf2045262

Vinueza D, Yanza K, Tacchini M, Grandini A, Sacchetti G, Chiurato MA, Guerrini A. Flavonoids in Ecuadorian *Oreocallis grandiflora* (Lam.) R. Br.: Perspectives of Use of This Species as a Food Supplement. Evidence-Based Complementary and Alternative Medicine. 2018;2018. doi.org/10.1155/2018/1353129

Viswanatha GL, Venkataranganna MV, Prasad NB, Godavarthi A. *Achyranthes aspera* Attenuates epilepsy in experimental animals: possible involvement of GABAergic mechanism. Metabolic brain disease. 2017 Jun 1;32(3):867-79.

Vital PG, Rivera WL. Antimicrobial activity and cytotoxicity of *Chromolaena odorata* (L. f.) King and Robinson and *Uncaria perrottetii* (A. Rich) Merr. Extracts. Journal of medicinal plants Research. 2009 Jul 1;3(7):511-8.

Vital PG, Velasco Jr RN, Demigillo JM, Rivera WL. Antimicrobial activity, cytotoxicity and phytochemical screening of *Ficus septica* Burm and *Sterculia foetida* L. leaf extracts. Journal of Medicinal Plants Research. 2010 Jan 4;4(1):058-63.

Vladimir-Knežević S, Blažeković B, Kindl M, Vladić J, Lower-Nedza AD, Brantner AH. Acetylcholinesterase inhibitory, antioxidant and phytochemical properties of selected medicinal plants of the Lamiaceae family. Molecules. 2014 Jan 9;19(1):767-82.

Vladimir-Knežević S, Kosalec I, Babac M, Petrović M, Ralić J, Matica B, Blažeković B. Antimicrobial activity of *Thymus longicaulis* C. Presl essential oil against respiratory pathogens. Open Life Sciences. 2012 Dec 1;7(6):1109-15. https://doi.org/10.2478/s11535-012-0088-2

Vlietinck AJ, Van Hoof L, Totte J, Lasure A, Berghe DV, Rwangabo PC, Mvukiyumwami J. Screening of hundred Rwandese medicinal plants for antimicrobial and antiviral properties. Journal of ethnopharmacology. 1995 Apr 1;46(1):31-47. https://doi.org/10.1016/0378-8741(95)01226-4

Vogrinčič M, Kreft I, Filipič M, Žegura B. Antigenotoxic effect of Tartary (*Fagopyrum tataricum*) and common (*Fagopyrum esculentum*) buckwheat flour. Journal of medicinal food. 2013 Oct 1;16(10):944-52. https://doi.org/10.1089/jmf.2012.0266

Vokou D, Katradi K, Kokkini S. Ethnobotanical survey of Zagori (Epirus, Greece), a renowned centre of folk medicine in the past. J Ethnopharmacol. 1993 Aug;39(3):187-96. doi: 10.1016/0378-8741(93)90035-4.

Volpato G, Kourková P, Zelený V. Healing war wounds and perfuming exile: the use of vegetal, animal, and mineral products for perfumes, cosmetics, and skin healing among Sahrawi refugees of Western Sahara. Journal of ethnobiology and ethnomedicine. 2012 Dec 1;8(1):49.

Vujicic M, Nikolic I, Kontogianni VG, Saksida T, Charisiadis P, Orescanin-Dusic Z, Blagojevic D, Stosic-Grujicic S, Tzakos AG, Stojanovic I. Methanolic extract of *Origanum vulgare* ameliorates type 1 diabetes through antioxidant, anti-inflammatory and anti-apoptotic activity. British Journal of Nutrition. 2015 Mar;113(5):770-82. https://doi.org/10.1017/S0007114514004048

Wächter GA, Hoffmann JJ, Furbacher T, Blake ME, Timmermann BN. Antibacterial and antifungal flavanones from *Eysenhardtia texana*. Phytochemistry. 1999 Dec 1;52(8):1469-71. https://doi.org/10.1016/S0031-9422(99)00221-6

Wadankar GD, Malode SN, Sarambekar SL. Traditionally used medicinal plants for wound healing in the Washim District, Maharashtra (India). International Journal of PharmTech Research. 2011;3(4):2080-4.

Wahedi HM, Jeong M, Chae JK, Do SG, Yoon H, Kim SY. Aloesin from *Aloe vera* accelerates skin wound healing by modulating MAPK/Rho and Smad signaling pathways in vitro and in vivo. Phytomedicine. 2017 May 15;28:19-26. doi: 10.1016/j.phymed.2017.02.005.

Walterfang M, Chien YH, Imrie J, Rushton D, Schubiger D, Patterson MC. Dysphagia as a risk factor for mortality in Niemann-Pick disease type C: systematic literature review and evidence from studies with miglustat. Orphanet journal of rare diseases. 2012 Dec 1;7(1):76. https://doi.org/10.1186/1750-1172-7-76

Wan Osman WN, Che Ahmad Tantowi NA, Lau SF, Mohamed S. Epicatechin and scopoletin rich *Morinda citrifolia* (Noni) leaf extract supplementation, mitigated Osteoarthritis via anti-inflammatory, anti-oxidative, and anti-protease pathways. Journal of food biochemistry. 2019 Mar;43(3):e12755. doi.org/10.1111/jfbc.12755

Wang BS, Huang GJ, Lu YH, Chang LW. Anti-inflammatory effects of an aqueous extract of Welsh onion green leaves in mice. Food chemistry. 2013 Jun 1;138(2-3):751-6.

Wang CJ, Hu CP, Xu KP, Yuan Q, Li FS, Zou H, Tan GS, Li YJ. Protective effect of selaginellin on glutamate-induced cytotoxicity and apoptosis in differentiated PC12 cells. Naunyn-Schmiedeberg's archives of pharmacology. 2010 Jan 1;381(1):73.

Wang F, Pu C, Zhou P, Wang P, Liang D, Wang Q, Hu Y, Li B, Hao X. Cinnamaldehyde prevents endothelial dysfunction induced by high glucose by activating Nrf2. Cellular Physiology and Biochemistry. 2015;36(1):315-24. https://doi.org/10.1159/000374074

Wang H -D, Fu L, Cheng CC, Gao R, Lin MY, Su HL, Belinda NE, Nguyen TH, Lin W-H, Lee PC, Hsieh LP. Inhibition of LPS-Induced Oxidative Damages and Potential Anti-Inflammatory Effects of *Phyllanthus emblica* Extract via Down-Regulating NF-κB, COX-2, and iNOS in RAW 264.7 Cells. Antioxidants. 2019; 8(8):270. https://doi.org/10.3390/antiox8080270

Wang H, Liu J, Li T, Liu RH. Blueberry extract promotes longevity and stress tolerance via DAF-16 in *Caenorhabditis elegans*. Food & function. 2018;9(10):5273-82.https://doi.org/10.1039/C8FO01680A

Wang J, Matsuzaki K, Kitanaka S. Stilbene derivatives from *Pholidota chinensis* and their anti-inflammatory activity. Chemical and pharmaceutical bulletin. 2006;54(8):1216-8. doi.org/10.1248/cpb.54.1216

Wang JF, Yang SH, Liu YQ, Li DX, He WJ, Zhang XX, Liu YH, Zhou XJ. Five new phorbol esters with cytotoxic and selective anti-inflammatory activities from *Croton tiglium*. Bioorganic & medicinal chemistry letters. 2015 May 1;25(9):1986-9.

Wang L, Si L, Li Y, Wang H, Xu F, Bian H, Shi Y, Huang H. Study on mechanism and active ingredient of *Artemisia scoparia* extracts against influenza virus. Lat. Am. J. Pharm. 2017 Jan 1;36(7):1355-60.

Wang LS, Tao X, Liu XM, et al. Cajaninstilbene Acid Ameliorates Cognitive Impairment Induced by Intrahippocampal Injection of Amyloid-$\beta_{1-42}$ Oligomers. *Front Pharmacol*. 2019;10:1084. Published 2019 Sep 24. doi:10.3389/fphar.2019.01084

Wang Q, Yu X, Patal K, Hu R, Chuang S, Zhang G, Zheng J. Tanshinones inhibit amyloid aggregation by amyloid-β peptide, disaggregate amyloid fibrils, and protect cultured cells. ACS chemical neuroscience. 2013 Jun 19;4(6):1004-15. doi: 10.1021/cn400051ev

Wang QH, Lv SW, Guo YY, Duan JX, Dong SY, Wang QS, Yu FM, Su H, Kuang HX. Pharmacological effect of *Caulophyllum robustum* on collagen-induced arthritis and regulation of nitric oxide, NF-κB, and proinflammatory cytokines in vivo and in vitro. Evidence-Based Complementary and Alternative Medicine. 2017;2017. Article ID 8134321. doi: 10.1155/2017/8134321.

Wang SE, Lin CL, Hsu CH, Sheu SJ, Wu CH. Oral treatment with the herbal formula B401 protects against aging-dependent neurodegeneration by attenuating oxidative stress and apoptosis in the brain of R6/2 mice. Clinical Interventions in Aging. 2015;10:1825. http://doi.org/10.2147/CIA.S93819

Wang XX, Zan K, Shi SP, Zeng KW, Jiang Y, Guan Y, Xiao CL, Gao HY, Wu LJ, Tu PF. Quinolone alkaloids with antibacterial and cytotoxic activities from the fruits of *Evodia rutaecarpa*. Fitoterapia. 2013 Sep 1;89:1-7. https://doi.org/10.1016/j.fitote.2013.04.007

Wang Y, Chen P, Tang C, Wang Y, Li Y, Zhang H. Antinociceptive and anti-inflammatory activities of extract and two isolated flavonoids of *Carthamus tinctorius* L. Journal of ethnopharmacology. 2014 Feb 3;151(2):944-50. https://doi.org/10.1016/j.jep.2013.12.003

Wang Y, Xia Z, Xu JR, Wang YX, Hou LN, Qiu Y, Chen HZ. α-Mangostin, a polyphenolic xanthone derivative from mangosteen, attenuates β-amyloid oligomers-induced neurotoxicity by inhibiting amyloid aggregation. Neuropharmacology. 2012 Feb 1;62(2):871-81. https://doi.org/10.1016/j.neuropharm.2011.09.016

Wangsa K, Sarma I, Saikia P, Ananthakrishnan D, Sarma HN, Velmurugan D. Estrogenic Effect of *Scoparia dulcis* (Linn) Extract in Mice Uterus and In Silico Molecular Docking Studies of Certain Compounds with Human Estrogen Receptors. J Reprod Infertil. 2020 Oct-Dec;21(4):247-258. doi: 10.18502/jri.v21i4.4329.

Warholm O, Skaar S, Hedman E, Mølmen HM, Eik L. The effects of a standardized herbal remedy made from a subtype of *Rosa canina* in patients with osteoarthritis: a double-blind, randomized, placebo-controlled clinical trial. Current therapeutic research. 2003 Jan 1;64(1):21-31. doi:10.1016/S0011-393X(03)00004-3

Waruruai J, Sipana B, Koch M, Barrows LR, Matainaho TK, Rai PP. An ethnobotanical survey of medicinal plants used in the Siwai and Buin districts of the Autonomous Region of Bougainville. *J Ethnopharmacol*. 2011;138(2):564-577. doi:10.1016/j.jep.2011.09.052

Wattanathorn J, Mator L, Muchimapura S, Tongun T, Pasuriwong O, Piyawatkul N, Yimtae K, Sripanidkulchai B, Singkhoraard J. Positive modulation of cognition and mood in the healthy elderly volunteer following the administration of *Centella asiatica*. Journal of ethnopharmacology. 2008 Mar 5;116(2):325-32. https://doi.org/10.1016/j.jep.2007.11.038

Weber ND, Andersen DO, North JA, Murray BK, Lawson LD, Hughes BG. In vitro virucidal effects of *Allium sativum* (garlic) extract and compounds. Planta medica. 1992 Oct;58(05):417-423. DOI: 10.1055/s-2006-961504

Weerasekera A, Sima DM, Dresselaers T, Van Huffel S, Van Damme P, Himmelreich U. Non-invasive assessment of disease progression and neuroprotective effects of dietary coconut oil supplementation in the ALS $SOD1^{G93A}$ mouse model: A $^{1}H$-magnetic resonance spectroscopic study. Neuroimage Clin. 2018;20:1092-1105. doi: 10.1016/j.nicl.2018.09.011

Wei BL, Weng JR, Chiu PH, Hung CF, Wang JP, Lin CN. Antiinflammatory flavonoids from *Artocarpus heterophyllus* and *Artocarpus communis*. Journal of agricultural and food chemistry. 2005 May 18;53(10):3867-71.

Wei F, Ma SC, Ma LY, But PP, Lin RC, Khan IA. Antiviral Flavonoids from the Seeds of *Aesculus chinensis*. Journal of natural Products. 2004 Apr 23;67(4):650-3. https://doi.org/10.1021/np030470h

Wei H, Zhang Z, Saha A, Peng S, Chandra G, Quezado Z, Mukherjee AB. Disruption of adaptive energy metabolism and elevated ribosomal p-S6K1 levels contribute to INCL pathogenesis: partial rescue by resveratrol. Hum Mol Genet. 2011 Mar 15;20(6):1111-21. doi: 10.1093/hmg/ddq555.

Wei LS, Wee W, Siong JY, Syamsumir DF. Characterization of anticancer, antimicrobial, antioxidant properties and chemical compositions of *Peperomia pellucida* leaf extract. Acta Medica Iranica. 2011:670-4.

Wei X, Xu X, Chen Z, Liang T, Wen Q, Qin N, Huang W, Huang X, Li Y, Li J, He J, Wei J, Huang R. Protective Effects of 2-Dodecyl-6-Methoxycyclohexa-2,5 -Diene-1,4-Dione Isolated from *Averrhoa carambola* L. (Oxalidaceae) Roots on Neuron Apoptosis and Memory Deficits in Alzheimer's Disease. Cell Physiol Biochem. 2018;49(3):1064-1073. doi: 10.1159/000493289.

Weibo DA, Hongnian LI, Pengpeng DO, Quanxi ME. Screening of Anti-inflammatory and Analgesic Parts of *Ardisia gigantifolia* stapf. and Its Toxicological Safety Study. Medicinal Plant. 2018 Dec 1;9(6).

Weinberg RP, Koledova VV, Shin H, Park JH, Tan YA, Sinskey AJ, Sambanthamurthi R, Rha C. Oil Palm Phenolics Inhibit the *In Vitro* Aggregation of *β*-Amyloid Peptide into Oligomeric Complexes. Int J Alzheimers Dis. 2018;2018:7608038. Published 2018 Jan 31. doi:10.1155/2018/7608038

Wen W, Lin Y, Ti Z. Antidiabetic, Antihyperlipidemic, Antioxidant, Anti-inflammatory Activities of Ethanolic Seed Extract of *Annona reticulata* L. in Streptozotocin Induced Diabetic Rats. Front Endocrinol *(Lausanne)*. 2019;10:716. Published 2019 Oct 23. doi:10.3389/fendo.2019.00716

Weng JR, Lin CS, Lai HC, Lin YP, Wang CY, Tsai YC, Wu KC, Huang SH, Lin CW. Antiviral activity of Sambucus FormosanaNakai ethanol extract and related phenolic acid constituents against human coronavirus NL63. Virus Research. 2019 Nov 1;273:197767.

Werka JS, Boehme AK, Setzer WN. Biological activities of essential oils from Monteverde, Costa Rica. Natural Product Communications. 2007 Dec;2(12):1934578X0700201204.

Whistler WA. Traditional and herbal medicine in the Cook Islands. J Ethnopharmacol. 1985 Jul;13(3):239-80. doi: 10.1016/0378-8741(85)90072-8.

Whyte AR, Cheng N, Fromentin E, Williams CM. A Randomized, Double-Blinded, Placebo-Controlled Study to Compare the Safety and Efficacy of Low Dose Enhanced Wild Blueberry Powder and Wild Blueberry Extract (ThinkBlue™) in Maintenance of Episodic and Working Memory in Older Adults. Nutrients. 2018 May 23;10(6):660. https://doi.org/10.3390/nu10060660

Wightman EL, Jackson PA, Forster J, Khan J, Wiebe JC, Gericke N, Kennedy DO. Acute effects of a polyphenol-rich leaf extract of *Mangifera indica* l.(zynamite) on cognitive function in healthy adults: A double-blind, placebo-controlled crossover study. Nutrients. 2020 Aug;12(8):2194. https://doi.org/10.3390/nu12082194

Williams CM, El Mohsen MA, Vauzour D, Rendeiro C, Butler LT, Ellis JA, Whiteman M, Spencer JP. Blueberry-induced changes in spatial working memory correlate with changes in hippocampal CREB phosphorylation and brain-derived neurotrophic factor (BDNF) levels. Free Radical Biology and Medicine. 2008 Aug 1;45(3):295-305. https://doi.org/10.1016/j.freeradbiomed.2008.04.008

Winner B, Winkler J. Adult neurogenesis in neurodegenerative diseases. Cold Spring Harb Perspect Biol. 2015;7(4):a021287. https://doi.org/10.1101/cshperspect.a021287

Wirdefeldt K, Adami H-O, Cole P, Trichopoulos D, Mandel J. Epidemiology and etiology of Parkinson's disease: a review of the evidence. European journal of epidemiology. 2011;26(1):1.

Woguem V, Maggi F, Fogang HP, Tapondjou LA, Womeni HM, Quassinti L, Bramucci M, Vitali LA, Petrelli D, Lupidi G, Papa F. Antioxidant, antiproliferative and antimicrobial activities of the volatile oil from the wild pepper *Piper capense* used in Cameroon as a culinary spice. Natural product communications. 2013 Dec;8(12):1934578X1300801234. https://doi.org/10.1177/1934578X1300801234

Wong K, Tsang W. In vitro antifungal activity of the aqueous extract of *Scutellaria baicalensis* Georgi root against *Candida albicans*. International journal of antimicrobial agents. 2009;34(3):284-5. DOI: 10.1016/j.ijantimicag.2009.03.007

Wong PY, Kitts DD. Studies on the dual antioxidant and antibacterial properties of parsley (*Petroselinum crispum*) and cilantro (*Coriandrum sativum*) extracts. Food chemistry. 2006 Aug 1;97(3):505-15. https://doi.org/10.1016/j.foodchem.2005.05.031

Wongkattiya N, Sanguansermsri P, Fraser IH, Sanguansermsri D. Antibacterial activity of cuminaldehyde on food-borne pathogens, the bioactive component of essential oil from *Cuminum cyminum* L. collected in Thailand. Journal of Complementary and Integrative Medicine. 2019 May 25. doi: 10.1515/jcim-2018-0195.

Wongkham S, Laupattarakasaem P, Pienthaweechai K, Areejitranusorn P, Wongkham C, Techanitiswad T. Antimicrobial activity of *Streblus asper* leaf extract. Phytotherapy Research. 2001 Mar;15(2):119-21. https://doi.org/10.1002/ptr.705

Wongwad E, Pingyod C, Saesong T, Waranuch N, Wisuitiprot W, Sritularak B, Temkitthawon P, Ingkaninan K. Assessment of the bioactive components, antioxidant, antiglycation and anti-inflammatory properties of *Aquilaria crassna* Pierre ex Lecomte leaves. Industrial Crops and Products. 2019 Oct 5;138:111448.

Woo KW, Kwon OW, Kim SY, Choi SZ, Son MW, Kim KH, Lee KR. Phenolic derivatives from the rhizomes of *Dioscorea nipponica* and their anti-neuroinflammatory and neuroprotective activities. Journal of ethnopharmacology. 2014 Sep 11;155(2):1164-70.

Woode E, Alagpulinsa DA, Abotsi WK. Anti-nociceptive, anxiolytic and anticonvulsant effects of an aqueous leaf extract of *Leea guineensis* G. Don (Family: Leeaceae). African Journal of Pharmacy and Pharmacology. 2011 Aug 1;5(8):1132-44. DOI: 10.5897/AJPP10.407

Woode E, Ansah C, Ainooson GK, Abotsi WM, Mensah AY, Duweijua M. Anti-inflammatory and antioxidant properties of the root extract of *Carissa edulis* (Forsk.) Vahl (Apocynaceae). Journal of Science and Technology (Ghana). 2007;27(3):5-15. DOI: 10.4314/just.v27i3.33054

Woradulayapinij W, Soonthornchareonnon N, Wiwat C. In vitro HIV type 1 reverse transcriptase inhibitory activities of Thai medicinal plants and *Canna indica* L. rhizomes. Journal of ethnopharmacology. 2005 Oct 3;101(1-3):84-9. doi.org/10.1016/j.jep.2005.03.030

Wruss J, Waldenberger G, Huemer S, Uygun P, Lanzerstorfer P, Müller U, Höglinger O, Weghuber J. Compositional characteristics of commercial beetroot products and beetroot juice prepared from seven beetroot varieties grown in Upper Austria. J Food Comp Anal. 2015;42:46–55. doi: 10.1016/j.jfca.2015.03.005.

Wu GA, Terol J, Ibanez V, López-García A, Pérez-Román E, Borredá C, Domingo C, Tadeo FR, Carbonell-Caballero J, Alonso R, Curk F. Genomics of the origin and evolution of *Citrus*. Nature. 2018 Feb;554(7692):311-6

Wu J, Fang XA, Yuan Y, Dong Y, Liang Y, Xie Q, Ban J, Chen Y, Lv Z. UPLC/Q-TOF-MS profiling of phenolics from *Canarium pimela* leaves and its vasorelaxant and antioxidant activities. Revista Brasileira de Farmacognosia. 2017 Dec;27(6):716-23. https://doi.org/10.1016/j.bjp.2017.10.005

Wu J, Yang G, Zhu W, Wen W, Zhang F, Yuan J, An L. Anti-atherosclerotic activity of platycodin D derived from roots of *Platycodon grandiflorum* in human endothelial cells. Biological and Pharmaceutical Bulletin. 2012 Aug 1;35(8):1216-21. https://doi.org/10.1248/bpb.b-y110129

Wube AA, Bucar F, Gibbons S, Asres K. Sesquiterpenes from *Warburgia ugandensis* and their antimycobacterial activity. Phytochemistry. 2005 Oct 1;66(19):2309-15. https://doi.org/10.1016/j.phytochem.2005.07.018

Wulandari LR, Umiati S, Sujuti H. Protective effect of methanol extract of Kelor (*Moringa oleifera*) leaves on Glutathione Peroxidase (GPx) levels in trabecular meshwork cell culture of primary congenital glaucoma patients. EurAsian Journal of BioSciences. 2019 Jul 7;13(2):839-44.

Xia CL, Tang GH, Guo YQ, Xu YK, Huang ZS, Yin S. Mulberry Diels-Alder-type adducts from *Morus alba* as multi-targeted agents for Alzheimer's disease. Phytochemistry. 2019 Jan 1;157:82-91.

Xian YF, Mao QQ, Wu JC, Su ZR, Chen JN, Lai XP, Ip SP, Lin ZX. Isorhynchophylline treatment improves the amyloid-β-induced cognitive impairment in rats via inhibition of neuronal apoptosis and tau protein hyperphosphorylation. Journal of Alzheimer's Disease. 2014 Jan 1;39(2):331-46. DOI: 10.3233/JAD-131457

Xiaoming WD, Wei LJ. Steroid Saponins of *Polygonatum cirrhifolium* Root and Their Antisepsis Activity [J]. Scientia Silvae Sinicae. 2007;8.

Xie W, Zhang S, Lei F, Ouyang X, Du L. *Ananas comosus* L. Leaf Phenols and p-Coumaric Acid Regulate Liver Fat Metabolism by Upregulating CPT-1 Expression. Evid Based Complement Alternat Med. 2014;2014:903258. doi: 10.1155/2014/903258

Xing Z, He Z, Wang S, Yan Y, Zhu H, Gao Y, Zhao Y, Zhang L. Ameliorative effects and possible molecular mechanisms of action of fibrauretine from *Fibraurea recisa* Pierre on d-galactose/AlCl 3-mediated Alzheimer's disease. RSC advances. 2018;8(55):31646-57. DOI: 10.1039/C8RA05356A

Xiong J, Li S, Wang W, Hong Y, Tang K, Luo Q. Screening and identification of the antibacterial bioactive compounds from *Lonicera japonica* Thunb. leaves. Food chemistry. 2013 May 1;138(1):327-33. https://doi.org/10.1016/j.foodchem.2012.10.127

Xiong N, Huang J, Chen C, Zhao Y, Zhang Z, Jia M, Zhang Z, Hou L, Yang H, Cao X, Liang Z. Dl-3-n-butylphthalide, a natural antioxidant, protects dopamine neurons in rotenone models for Parkinson's disease. Neurobiology of aging. 2012 Aug 1;33(8):1777-91. https://doi.org/10.1016/j.neurobiolaging.2011.03.007

Xu HX, Lee SH, Lee SF, White RL, Blay J. Isolation and characterization of an anti-HSV polysaccharide from *Prunella vulgaris*. Antiviral research. 1999 Nov 1;44(1):43-54. https://doi.org/10.1016/S0166-3542(99)00053-4

Xu HX, Wan M, Loh BN, Kon OL, Chow PW, Sim KY. Screening of Traditional Medicines for their Inhibitory Activity Against HIV-1 Protease. Phytotherapy Research. 1996 May;10(3):207-10.

Xu L, Li Y, Fu Q, Ma S. Perillaldehyde attenuates cerebral ischemia–reperfusion injury-triggered overexpression of inflammatory cytokines via modulating Akt/JNK pathway in the rat brain cortex. Biochemical and biophysical research communications. 2014 Nov 7;454(1):65-70. https://doi.org/10.1016/j.bbrc.2014.10.025

Xu MF, Xiong YY, Liu JK, Qian JJ, Zhu L, Gao J. Asiatic acid, a pentacyclic triterpene in *Centella asiatica*, attenuates glutamate-induced cognitive deficits in mice and apoptosis in SH-SY5Y cells. Acta Pharmacologica Sinica. 2012 May;33(5):578-87. https://doi.org/10.1038/aps.2012.3

Xu Z, Shan Y. Anti-fatigue effects of polysaccharides extracted from *Portulaca oleracea* L. in mice. Indian Journal of Biochemistry & Biophysics. 2014 Aug 1;51(4):321-5.

Xue Y, Wang Y, Feng DC, Xiao BG, Xu LY. Tetrandrine suppresses lipopolysaccharide-induced microglial activation by inhibiting NF-κB pathway. Acta Pharmacologica Sinica. 2008 Feb;29(2):245-51. https://doi.org/10.1111/j.1745-7254.2008.00734.x

Ya'u J, Yaro AH, Malami S, Musa MA, Abubakar A, Yahaya SM, Chindo BA, Anuka JA, Hussaini IM. Anticonvulsant activity of aqueous fraction of *Carissa edulis* root bark. Pharmaceutical biology. 2015 Sep 2;53(9):1329-38. https://doi.org/10.3109/13880209.2014.981280

Yadav E, Singh D, Debnath B, Rathee P, Yadav P, Verma A. Molecular Docking and Cognitive Impairment Attenuating Effect of Phenolic Compound Rich Fraction of *Trianthema portulacastrum* in Scopolamine Induced Alzheimer's Disease Like Condition. Neurochemical Research. 2019 Jul 1;44(7):1665-77. https://doi.org/10.1007/s11064-019-02792-7

Yadav E, Singh D, Yadav P, Verma A. Attenuation of dermal wounds via downregulating oxidative stress and inflammatory markers by protocatechuic acid rich n-butanol fraction of *Trianthema portulacastrum* Linn. in wistar albino rats. Biomedicine & Pharmacotherapy. 2017 Dec 1;96:86-97. doi: 10.1016/j.biopha.2017.09.125.

Yadav S, Kumar S, Jain P, Pundir RK, Jadon S, Sharma A, Khetwal KS, Gupta KC. Antimicrobial activity of different extracts of roots of *Rumex nepalensis* Spreng. Indian Journal of Natural Products and Resources. 2011 March; 2(1): 65-69.

Yaghmaei P, Azarfar K, Dezfulian M, Ebrahim-Habibi A. Silymarin effect on amyloid-β plaque accumulation and gene expression of APP in an Alzheimer's disease rat model. DARU Journal of Pharmaceutical Sciences. 2014 Dec;22(1):24. https://doi.org/10.1186/2008-2231-22-24

Yakoub AR, Abdehedi O, Jridi M, Elfalleh W, Nasri M, Ferchichi A. Flavonoids, phenols, antioxidant, and antimicrobial activities in various extracts from Tossa jute leave (*Corchorus olitorus* L). Industrial Crops and Products. 2018 Aug 1;118:206-13. doi.org/10.1016/j.indcrop.2018.03.047

Yamaguchi S, Matsumoto K, Koyama M, Tian S, Watanabe M, Takahashi A, Miyatake K, Nakamura K. Antihypertensive effects of orally administered eggplant (*Solanum melongena*) rich in acetylcholine on spontaneously hypertensive rats. Food chemistry. 2019 Mar 15;276:376-82. https://doi.org/10.1016/j.foodchem.2018.10.017

Yan J, Yang X, Han D, Feng J. Tanshinone IIA attenuates experimental autoimmune encephalomyelitis in rats. Molecular medicine reports. 2016 Aug 1;14(2):1601-9. https://doi.org/10.3892/mmr.2016.5431

Yang EJ, Min JS, Ku HY, Choi HS, Park MK, Kim MK, Song KS, Lee DS. Isoliquiritigenin isolated from *Glycyrrhiza uralensis* protects neuronal cells against glutamate-induced mitochondrial dysfunction. Biochemical and biophysical research communications. 2012 May 18;421(4):658-64. https://doi.org/10.1016/j.bbrc.2012.04.053

Yang EJ, Yim EY, Song G, Kim GO, Hyun CG. Inhibition of nitric oxide production in lipopolysaccharide-activated RAW 264.7 macrophages by Jeju plant extracts. Interdisciplinary toxicology. 2009 Dec 1;2(4):245-9.

Yang J, Li S, Xie C, Ye H, Tang H, Chen L, Peng A. Anti-inflammatory activity of ethyl acetate fraction of the seeds of *Brucea javanica*. Journal of ethnopharmacology. 2013 May 20;147(2):442-6. https://doi.org/10.1016/j.jep.2013.03.034

Yang JX, Wu S, Huang XL, Hu XQ, Zhang Y. Hypolipidemic Activity and Antiatherosclerotic Effect of Polysaccharide of *Polygonatum sibiricum* in Rabbit Model and Related Cellular Mechanisms. Evid Based Complement Alternat Med. 2015;2015:391065. doi: 10.1155/2015/391065. Epub 2015 May 18. doi: 10.1155/2015/391065

Yang M, Dang R, Xu P, Guo Y, Han W, Liao D, Jiang P. Dl-3-n-Butylphthalide improves lipopolysaccharide-induced depressive-like behavior in rats: involvement of Nrf2 and NF-κB pathways. Psychopharmacology. 2018 Sep 1;235(9):2573-85. DOI: 10.1007/s00213-018-4949-x

Yang X, Gao X, Cao Y, Guo Q, Li S, Zhu Z, Zhao Y, Tu P, Chai X. Anti-Inflammatory effects of boldine and reticuline isolated from *Litsea cubeba* through JAK2/STAT3 and NF-κB signaling pathways. Planta medica. 2018 Jan;84(01):20-5. DOI: 10.1055/s-0043-113447

Yang XY, Liu AL, Liu SJ, Xu XW, Huang LF. Screening for neuraminidase inhibitory activity in traditional Chinese medicines used to treat influenza. Molecules. 2016 Sep;21(9):1138.

Yang Z, Wang Y, Zheng Z, Zhao S, Zhao JI, Lin Q, Li C, Zhu Q, Zhong N. Antiviral activity of *Isatis indigotica* root-derived clemastanin B against human and avian influenza A and B viruses in vitro. International journal of molecular medicine. 2013 Apr 1;31(4):867-73. doi.org/10.3892/ijmm.2013.1274

Yanpallewar S, Rai S, Kumar M, Chauhan S, Acharya SB. Neuroprotective effect of *Azadirachta indica* on cerebral post-ischemic reperfusion and hypoperfusion in rats. Life sciences. 2005 Feb 4;76(12):1325-38. https://doi.org/10.1016/j.lfs.2004.06.029

Yao NA, Niazi ZR, Najmanová I, Kamagaté M, Said A, Chabert P, Auger C, Die-Kakou H, Schini-Kerth V. Preventive Beneficial Effect of an Aqueous Extract of *Phyllanthus amarus* Schum. and Thonn.(Euphorbiaceae) on DOCA-Salt–Induced Hypertension, Cardiac Hypertrophy and Dysfunction, and Endothelial Dysfunction in Rats. Journal of cardiovascular pharmacology. 2020 Jun 1;75(6):573-83. doi: 10.1097/FJC.0000000000000825

Yao XJ, Wainberg MA, Parniak MA. Mechanism of inhibition of HIV-1 infection in vitro by purified extract of *Prunella vulgaris*. Virology. 1992 Mar 1;187(1):56-62. https://doi.org/10.1016/0042-6822(92)90294-Y

Yao Y, Yang X, Shi Z, Ren G. Anti-inflammatory activity of saponins from quinoa (*Chenopodium quinoa* Willd.) seeds in lipopolysaccharide-stimulated RAW 264.7 macrophages cells. Journal of food science. 2014 May;79(5):H1018-23.

Yaseen R, Branitzki-Heinemann K, Moubasher H, Setzer WN, Naim HY, Köckritz-Blickwede V. In vitro testing of crude natural plant extracts from Costa Rica for their ability to boost innate immune cells against *Staphylococcus aureus*. Biomedicines. 2017 Sep;5(3):40. https://doi.org/10.3390/biomedicines5030040

Ye S, Wang TT, Cai B, Wang Y, Li J, Zhan JX, Shen GM. Genistein protects hippocampal neurons against injury by regulating calcium/calmodulin dependent protein kinase IV protein levels in Alzheimer's disease model rats. Neural Regen Res. 2017 Sep;12(9):1479-1484. doi: 10.4103/1673-5374.215260.

Ye Y, Li B. 1′ S-1′-acetoxychavicol acetate isolated from *Alpinia galanga* inhibits human immunodeficiency virus type 1 replication by blocking Rev transport. Journal of general virology. 2006 Jul 1;87(7):2047-53. https://doi.org/10.1099/vir.0.81685-0

Yedomon BH, Saves I, Mtimet N, Raoelison EG, Constant P, Daffé M, Bouajila J. *Elionurus tristis* essential oil: GC-MS analysis and antioxidant and antituberculosis activities. Natural product communications. 2017 Apr;12(4):1934578X1701200436.

Yeh SH, Chang FR, Wu YC, Yang YL, Zhuo SK, Hwang TL. An anti-inflammatory ent-kaurane from the stems of *Annona squamosa* that inhibits various human neutrophil functions. Planta Med. 2005 Oct;71(10):904-9. doi: 10.1055/s-2005-871234.

Yemane B, Andebrhan M, Surender Reddy K. Traditional medicinal plants used by Tigrigna Ethnic Group in Central Region Of Eritrea. IOSR J Pharm Biol Sci. 2017;12(3):40-6. doi: 10.9790/3008-1203034046

Yenesew A, Twinomuhwezi H, Kiremire BT, Mbugua MN, Gitu PM, Heydenreich M, Peter MG. 8-Methoxyneorautenol and radical scavenging flavonoids from *Erythrina abyssinica*. Bulletin of the Chemical Society of Ethiopia. 2009;23(2). DOI: 10.4314/bcse.v23i2.44963

Yeo D, Dinica R, Yapi HF, Furdui B, Praisler M, Djaman AJ, N'Guessan JD. Evaluation of the anti-inflammatory activity and phytochemical screening of *Annona senegalensis* leaves. Therapie. 2011;66(1):73-80. DOI: 10.2515/therapie/2010076

Yimam M, Burnett BP, Brownell L, Jia Q. Clinical and preclinical cognitive function improvement after oral treatment of a botanical composition composed of extracts from *Scutellaria baicalensis* and *Acacia catechu*. Behavioural neurology. 2016;2016. Article ID 7240802 https://doi.org/10.1155/2016/7240802

Yin HL, Li JH, Li J, Li B, Chen L, Tian Y, Liu SJ, Zhang T, Dong JX. Four new coumarinolignoids from seeds of *Solanum indicum*. Fitoterapia. 2013 Jan 1;84:360-5. https://doi.org/10.1016/j.fitote.2012.09.002

Yin L, Chen Y, Qu Z, Zhang L, Wang Q, Zhang Q, Li L. Involvement of JAK/STAT signaling in the effect of cornel iridoid glycoside on experimental autoimmune encephalomyelitis amelioration in rats. Journal of neuroimmunology. 2014 Sep 15;274(1-2):28-37. https://doi.org/10.1016/j.jneuroim.2014.06.022

Yiu EM, Tai G, Peverill RE, Lee KJ, Croft KD, Mori TA, Scheiber-Mojdehkar B, Sturm B, Praschberger M, Vogel AP, Rance G. An open-label trial in Friedreich ataxia suggests clinical benefit with high-dose resveratrol, without effect on frataxin levels. Journal of Neurology. 2015 May 1;262(5):1344-53. https://doi.org/10.1007/s00415-015-7719-2

Ymele EV, Dongmo AB, Dimo T. Analgesic and anti-inflammatory effect of aqueous extract of the stem bark of *Allanblackia gabonensis* (Guttiferae). Inflammopharmacology. 2013 Feb 1;21(1):21-30.

Yodsaoue O, Sonprasit J, Karalai C, Ponglimanont C, Tewtrakul S, Chantrapromma S. Diterpenoids and triterpenoids with potential anti-inflammatory activity from the leaves of *Aglaia odorata*. Phytochemistry. 2012 Apr 1;76:83-91.

Yongxu S, Jicheng L. Structural characterization of a water-soluble polysaccharide from the roots of *Codonopsis pilosula* and its immunity activity. International Journal of Biological Macromolecules. 2008 Oct 1;43(3):279-82. https://doi.org/10.1016/j.ijbiomac.2008.06.009

Yoo DY, Kim W, Yoo KY, Lee CH, Choi JH, Yoon YS, Kim DW, Won MH, Hwang IK. Grape seed extract enhances neurogenesis in the hippocampal dentate gyrus in C57BL/6 mice. Phytotherapy Research. 2011 May;25(5):668-74. https://doi.org/10.1002/ptr.3319

Yoon CS, Ko W, Lee DS, Kim DC, Kim J, Choi M, Beom JS, An RB, Oh H, Kim YC. *Taraxacum coreanum* protects against glutamate-induced neurotoxicity through heme oxygenase-1 expression in mouse hippocampal HT22 cells. Molecular medicine reports. 2017 Apr 1;15(4):2347-52.

Yoon JJ, Lee YJ, Kim JS, Kang DG, Lee HS. Protective role of betulinic acid on TNF-α-induced cell adhesion molecules in vascular endothelial cells. Biochemical and biophysical research communications. 2010 Jan 1;391(1):96-101. https://doi.org/10.1016/j.bbrc.2009.11.009

Yoon JY, Jeong HY, Kim SH, Kim HG, Nam G, Kim JP, Yoon DH, Hwang H, Kimc TW, Hong S, Cho JY. Methanol extract of *Evodia lepta* displays Syk/Src-targeted anti-inflammatory activity. Journal of ethnopharmacology. 2013 Jul 30;148(3):999-1007. https://doi.org/10.1016/j.jep.2013.05.030

Yoopan N, Thisoda P, Rangkadilok N, Sahasitiwat S, Pholphana N, Ruchirawat S, Satayavivad J. Cardiovascular effects of 14-deoxy-11, 12-didehydroandrographolide and *Andrographis paniculata* extracts. Planta medica. 2007 Jun;73(06):503-11. DOI: 10.1055/s-2007-967181

Yoosook C, Bunyapraphatsara N, Boonyakiat Y, Kantasuk C. Anti-*Herpes simplex* virus activities of crude water extracts of Thai medicinal plants. Phytomedicine. 2000 Jan 1;6(6):411-9. https://doi.org/10.1016/S0944-7113(00)80068-9

Yoosook C, Panpisutchai Y, Chaichana S, Santisuk T, Reutrakul V. Evaluation of anti-HSV-2 activities of *Barleria lupulina* and *Clinacanthus nutans*. Journal of ethnopharmacology. 1999 Nov 1;67(2):179-87. https://doi.org/10.1016/S0378-8741(99)00008-2

Yougbare-Ziebrou MN, Lompo M, Ouedraogo N, Yaro B, Guissoun IP. Antioxidant, analgesic and anti-inflammatory activities of the leafy stems of *Waltheria indica* L.(Sterculiaceae). Journal of Applied Pharmaceutical Science. 2016 Feb;6(02):124-9. DOI: 10.7324/JAPS.2016.60219

Youn K, Lee S, Jun M. Discovery of Nobiletin from Citrus Peel as a Potent Inhibitor of β-Amyloid Peptide Toxicity. Nutrients. 2019 Nov;11(11):2648. doi.org/10.3390/nu11112648

Young HY, Liao JC, Chang YS, Luo YL, Lu MC, Peng WH. Synergistic effect of ginger and nifedipine on human platelet aggregation: a study in hypertensive patients and normal volunteers. The American journal of Chinese medicine. 2006;34(04):545-51. https://doi.org/10.1142/S0192415X06004089

Yousofi A, Daneshmandi S, Soleimani N, Bagheri K, Karimi MH. Immunomodulatory effect of Parsley (*Petroselinum crispum*) essential oil on immune cells: mitogen-activated splenocytes and peritoneal macrophages. Immunopharmacology and Immunotoxicology. 2012 Apr 1;34(2):303-8. https://doi.org/10.3109/08923973.2011.603338

Youssef RS. Medicinal and non-medicinal uses of some plants found in the middle region of Saudi Arabia. J Med Plants Res. 2013;7(34):2501-13.

Yousuf S, Atif F, Ahmad M, Hoda N, Ishrat T, Khan B, Islam F. Resveratrol exerts its neuroprotective effect by modulating mitochondrial dysfunctions and associated cell death during cerebral ischemia. Brain research. 2009 Jan 23;1250:242-53.

Yu ES, Min HJ, Lee K, Lee MS, Nam JW, Seo EK, Hong JH, Hwang ES. Anti-inflammatory activity of p-coumaryl alcohol-γ-O-methyl ether is mediated through modulation of interferon-γ production in Th cells. British journal of pharmacology. 2009 Apr;156(7):1107-14. https://doi.org/10.1111/j.1476-5381.2009.00114.x

Yu KD, Kim HH, Park HK, Ahn DK, Choi HY. The Effects of Water Extract of Genus *Panax* on Rat Blood Vessels. Journal of Korean Medicine.;2002;3(1):71-75.

Yu L, Wang S, Chen X, Yang H, Li X, Xu Y, Zhu X. Orientin alleviates cognitive deficits and oxidative stress in Aβ1–42-induced mouse model of Alzheimer's disease. Life sciences. 2015 Jan 15;121:104-9. http://dx.doi.org/10.1016/j.lfs.2014.11.021

Yuan J, Gan T, Liu Y, Gao H, Xu W, Zhang T, Tan R, Cai Z, Jiang H. Composition and antimicrobial activity of the essential oil from the branches of *Jacaranda cuspidifolia* Mart. growing in Sichuan, China. Natural product research. 2018 Jun 18;32(12):1451-4. doi.org/10.1080/14786419.2017.1346644

Yueqin Z, Recio MC, Máñez S, Giner RM, Cerdá-Nicolás M, Ríos JL. Isolation of two triterpenoids and a biflavanone with anti-inflammatory activity from *Schinus molle* fruits. Planta medica. 2003 Oct;69(10):893-8. DOI: 10.1055/s-2003-45096

Yusufoglu HS, Alqasoumi SI. Anti-inflammatory and wound healing activities of herbal gel containing an antioxidant *Tamarix aphylla* leaf extract. International Journal of Pharmacology. 2011 Nov 15;7(8):829-35. DOI: 10.3923/ijp.2011.829.835

Zafar S, Anwar H, Qasim M, Irfan S, Maqbool J, Sajid F, Naqvi SA, Hussain G. *Calotropis procera* (root) escalates functions rehabilitation and attenuates oxidative stress in a mouse model of peripheral nerve injury. Pak. J. Pharm. Sci. 2020 Nov;33(6):2801-7. doi.org/10.36721/PJPS.2020.33.6.SUP.2801-2807.1

Zaidi SA, Pathan SA, Ahmad FJ, Surender S, Jamil S, Khar RK. Anticonvulsant and Neurotoxicity Profile of *Commiphora gileadensis* (L.) C. Chr. Planta Medica. 2010 Mar;76(05):P84. DOI: 10.1055/s-0030-1251846

Zaima K, Deguchi J, Matsuno Y, Kaneda T, Hirasawa Y, Morita H. Vasorelaxant effect of FR900359 from *Ardisia crenata* on rat aortic artery. Journal of natural medicines. 2013 Jan 1;67(1):196-201.

Zakaria ZA, Hussain MK, Mohamad AS, Abdullah FC, Sulaiman MR. Anti-inflammatory activity of the aqueous extract of *Ficus deltoidea*. Biological research for nursing. 2012 Jan;14(1):90-7. https://doi.org/10.1177/1099800410395378

Zakaria ZA, Sufian AS, Ramasamy K, Ahmat N, Sulaiman MR, Arifah AK, Zuraini A, Somchit MN. In vitro antimicrobial activity of *Muntingia calabura* extracts and fractions. African Journal of Microbiology Research. 2010 Feb 18;4(4):304-8.

Zakaria ZA, Zakaria ML, Amom Z, Desa MN. Antimicrobial activity of the aqueous extract of selected Malaysian herbs. African Journal of Microbiology Research. 2011 Dec 16;5(30):5379-83.

Zakay-Rones Z, Thom E, Wollan T, Wadstein J. Randomized study of the efficacy and safety of oral elderberry extract in the treatment of influenza A and B virus infections. Journal of International Medical Research. 2004 Apr;32(2):132-40. https://doi.org/10.1177/147323000403200205

Zali SH, Tahmasb R. Medicinal plants of Farashband tribe's winter pastures and their traditional uses. J Adv Health Med Sci. 2016;2(1):18-27. DOI: 10.20474/jahms-2.1.3

Zamani M, Rahimi AO, Mahdavi R, Nikbakhsh M, Jabbari MV, Rezazadeh H, Delazar A, Nahar L, Sarker SD. Assessment of anti-hyperlipidemic effect of *Citrullus colocynthis*. Revista Brasileira de Farmacognosia. 2007 Dec;17(4):492-6. doi.org/10.1590/S0102-695X2007000400003

Zamorano P, Rojano BI, Morales M, Magariños H, Godoy P, Muñoz O. Biological and antioxidant activity of *Gunnera tinctoria* (Nalca). Journal of Medicinal Plants Research. 2017 May 3;11(17):318-30.

Zandi K, Lim TH, Rahim NA, Shu MH, Teoh BT, Sam SS, Danlami MB, Tan KK, Abubakar S. Extract of *Scutellaria baicalensis* inhibits dengue virus replication. BMC complementary and alternative medicine. 2013 Dec;13(1):1-0. https://doi.org/10.1186/1472-6882-13-91

Zarai Z, Boujelbene E, Salem NB, Gargouri Y, Sayari A. Antioxidant and antimicrobial activities of various solvent extracts, piperine and piperic acid from *Piper nigrum*. Lwt-Food science and technology. 2013 Mar 1;50(2):634-41.https://doi.org/10.1016/j.lwt.2012.07.036

Zarai Z, Kadri A, Chobba IB, Mansour RB, Bekir A, Mejdoub H, Gharsallah N. The in-vitro evaluation of antibacterial, antifungal and cytotoxic properties of *Marrubium vulgare* L. essential oil grown in Tunisia. Lipids in health and disease. 2011 Dec;10(1):161. https://doi.org/10.1186/1476-511X-10-161

Zareie A, Sahebkar A, Khorvash F, Bagherniya M, Hasanzadeh A, Askari G. Effect of cinnamon on migraine attacks and inflammatory markers: A randomized double-blind placebo-controlled trial. Phytother Res. 2020 Nov;34(11):2945-2952. doi: 10.1002/ptr.6721.

Zeng H, Locatelli M, Bardelli C, Amoruso A, Coisson JD, Travaglia F, Arlorio M, Brunelleschi S. Anti-inflammatory properties of clovamide and *Theobroma cacao* phenolic extracts in human monocytes: evaluation of respiratory burst, cytokine release, NF-κB activation, and PPARγ modulation. Journal of agricultural and food chemistry. 2011 May 25;59(10):5342-50. https://doi.org/10.1021/jf2005386

Zeng KW, Liao LX, Song XM, Lv HN, Song FJ, Yu Q, Dong X, Jiang Y, Tu PF. Caruifolin D from *Artemisia absinthium* L. inhibits neuroinflammation via reactive oxygen species-dependent c-jun N-terminal kinase and protein kinase c/NF-κB signaling pathways. European journal of pharmacology. 2015 Nov 15;767:82-93. https://doi.org/10.1016/j.ejphar.2015.10.011

Zeng WC, Zhang Z, Gao H, Jia LR, He Q. Chemical composition, antioxidant, and antimicrobial activities of essential oil from pine needle (*Cedrus deodara*). Journal of food science. 2012 Jul;77(7):C824-9. https://doi.org/10.1111/j.1750-3841.2012.02767.x

Zeng X, Dong Y, Sheng G, Dong X, Sun X, Fu J. Isolation and structure determination of anti-influenza component from *Mahonia bealei*. Journal of ethnopharmacology. 2006 Dec 6;108(3):317-9. https://doi.org/10.1016/j.jep.2006.05.014

Zengin G, Ferrante C, Gnapi DE, Sinan KI, Orlando G, Recinella L, Diuzheva A, Jekő J, Cziáky Z, Chiavaroli A, Leone S. Comprehensive approaches on the chemical constituents and pharmacological properties of flowers and leaves of American basil (*Ocimum americanum* L). Food Research International. 2019 Nov 1;125:108610. https://doi.org/10.1016/j.foodres.2019.108610

Zétola M, De Lima TC, Sonaglio D, González-Ortega G, Limberger RP, Petrovick PR, Bassani VL. CNS activities of liquid and spray-dried extracts from *Lippia alba*—Verbenaceae (Brazilian false melissa). Journal of Ethnopharmacology. 2002 Oct 1;82(2-3):207-15. https://doi.org/10.1016/S0378-8741(02)00187-3

Zhang CC, Gu WL, Wu XM, Li YM, Chen CX, Huang XY. Active components from Radix Scrophulariae inhibits the ventricular remodeling induced by hypertension in rats. SpringerPlus. 2016 Dec 1;5(1):358. https://doi.org/10.1186/s40064-016-1985-z

Zhang F, Zheng W, Pi R, Mei Z, Bao Y, Gao J, Tang W, Chen S, Liu P. Cryptotanshinone protects primary rat cortical neurons from glutamate-induced neurotoxicity via the activation of the phosphatidylinositol 3-kinase/Akt signaling pathway. Experimental brain research. 2009 Feb 1;193(1):109-18.

Zhang GQ, Huang XD, Wang H, Leung AK, Chan CL, Fong DW, Yu ZL. Anti-inflammatory and analgesic effects of the ethanol extract of *Rosa multiflora* Thunb. hips. Journal of ethnopharmacology. 2008 Jul 23;118(2):290-4. doi.org/10.1016/j.jep.2008.04.014

Zhang HJ, Rumschlag-Booms E, Guan YF, Wang DY, Liu KL, Li WF, Nguyen VH, Cuong NM, Soejarto DD, Fong HH, Rong L. Potent inhibitor of drug-resistant HIV-1 strains identified from the medicinal plant *Justicia gendarussa*. Journal of natural products. 2017 Jun 14;80(6):1798-807. doi: 10.1021/acs.jnatprod.7b00004.

Zhang HW, Zhang YH, Lu MJ, Tong WJ, Cao GW. Comparison of hypertension, dyslipidaemia and hyperglycaemia between buckwheat seed-consuming and non-consuming Mongolian-Chinese populations in Inner Mongolia, China. Clinical and experimental pharmacology & physiology. 2007 Sep;34(9):838-44. DOI: 10.1111/j.1440-1681.2007.04614.x

Zhang L, Zhang Y, Pei S, Geng Y, Wang C, Yuhua W. Ethnobotanical survey of medicinal dietary plants used by the Naxi People in Lijiang Area, Northwest Yunnan, China. Journal of ethnobiology and ethnomedicine. 2015 Dec;11(1):1-1. https://doi.org/10.1186/s13002-015-0030-6

Zhang L, Zhang ZK, Liang S. Epigallocatechin-3-gallate protects retinal vascular endothelial cells from high glucose stress in vitro via the MAPK/ERK-VEGF pathway. Genet Mol Res. 2016 Jun 10;15(2):10-4238. http://dx.doi.org/10.4238/gmr.15027874

Zhang S, Huang Y, Li Y, Wang Y, He X. Anti-neuroinflammatory and antioxidant phenylpropanoids from Chinese olive. Food chemistry. 2019 Jul 15;286:421-7. doi: 10.1016/j.foodchem.2019.02.031

Zhang S, Yu Z, Xia J, Zhang X, Liu K, Sik A, Jin M. Anti-Parkinson's disease activity of phenolic acids from *Eucommia ulmoides* Oliver leaf extracts and their autophagy activation mechanism. Food & Function. 2020. https://doi.org/10.1039/C9FO02288K

Zhang WZ, Jiang ZK, He BX, Liu XB. Arctigenin Protects against Lipopolysaccharide-Induced Pulmonary Oxidative Stress and Inflammation in a Mouse Model via Suppression of MAPK, HO-1, and iNOS Signaling. Inflammation. 2015;38(4):1406-1414. doi:10.1007/s10753-015-0115-3

Zhang Y, Yao X, Bao B, Zhang Y. Anti-fatigue activity of a triterpenoid-rich extract from Chinese bamboo shavings (*Caulis bamfusae in taeniam*). Phytotherapy Research: An International Journal Devoted to Pharmacological and Toxicological Evaluation of Natural Product Derivatives. 2006 Oct;20(10):872-6. https://doi.org/10.1002/ptr.1965

Zhao G, Yin Z, Dong J. Antiviral efficacy against hepatitis B virus replication of oleuropein isolated from *Jasminum officinale* L. var. *grandiflorum*. Journal of Ethnopharmacology. 2009 Sep 7;125(2):265-8. https://doi.org/10.1016/j.jep.2009.06.030

Zhao H, Wang QL, Hou SB, Chen G. Chemical constituents from the rhizomes of *Polygonatum sibiricum* Red. and anti-inflammatory activity in RAW264. 7 macrophage cells. Natural product research. 2019 Aug 18;33(16):2359-62. https://doi.org/10.1080/14786419.2018.1440220

Zhao H, Yun W, Zhang Q, Cai X, Li X, Hui G, Zhou X, Ni J. Mobilization of circulating endothelial progenitor cells by dl-3-n-butylphthalide in acute ischemic stroke patients. Journal of Stroke and Cerebrovascular Diseases. 2016 Apr 1;25(4):752-60. https://doi.org/10.1016/j.jstrokecerebrovasdis.2015.11.018

Zhao J, Cheng YY, Fan W, Yang CB, Ye SF, Cui W, Wei W, Lao LX, Cai J, Han YF, Rong JH. Botanical drug puerarin coordinates with nerve growth factor in the regulation of neuronal survival and neuritogenesis via activating ERK 1/2 and PI 3K/Akt signaling pathways in the neurite extension process. CNS neuroscience & therapeutics. 2015 Jan;21(1):61-70. doi: 10.1111/cns.12334.

Zhao L, Wang JL, Liu R, Li XX, Li JF, Zhang L. Neuroprotective, anti-amyloidogenic and neurotrophic effects of apigenin in an Alzheimer’s disease mouse model. Molecules. 2013 Aug;18(8):9949-65. doi:10.3390/molecules18089949

Zhao L, Zhang SL, Tao JY, Jin F, Pang R, Guo YJ, Ye P, Dong JH, Zheng GH. Anti-inflammatory mechanism of a folk herbal medicine, *Duchesnea indica* (Andr) Focke at RAW264. 7 cell line. Immunological investigations. 2008 Jan 1;37(4):339-57. https://doi.org/10.1080/08820130802111589

Zhao L, Zou T, Gomez NA, Wang B, Zhu MJ, Du M. Raspberry alleviates obesity-induced inflammation and insulin resistance in skeletal muscle through activation of AMP-activated protein kinase (AMPK) α1. Nutrition & diabetes. 2018 Jul 2;8(1):1-8. https://doi.org/10.1038/s41387-018-0049-6

Zhao M, Liu L, Zheng Y, Liu G, Che B, Li P, Chen H, Dong C, Lin L, Du Z. Anti-inflammatory effects of paeoniflorin from *Paeonia lactiflora* Pall. on human corneal epithelial cells and a mouse model of dry eye disease. RSC advances. 2019;9(23):12998-3006. DOI: 10.1039/C8RA09060B

Zhao Y, Deng H, Li K, Wang L, Wu Y, Dong X, Wang X, Chen Y, Xu Y. Trans-cinnamaldehyde improves neuroinflammation-mediated NMDA receptor dysfunction and memory deficits through blocking NF-κB pathway in presenilin1/2 conditional double knockout mice. Brain, behavior, and immunity. 2019 Nov 1;82:45-62. doi.org/10.1016/j.bbi.2019.07.032

Zhao YL, Gou ZP, Shang JH, Li WY, Kuang Y, Li MY, Luo XD. Anti-microbial Effects In Vitro and In Vivo of *Alstonia scholaris*. Nat Prod Bioprospect. 2021 Feb;11(1):127-135. doi: 10.1007/s13659-020-00294-6.

Zhen J, Guo Y, Villani T, Carr S, Brendler T, Mumbengegwi DR, Kong AN, Simon JE, Wu Q. Phytochemical analysis and anti-inflammatory activity of the extracts of the African medicinal plant *Ximenia caffra*. Journal of analytical methods in chemistry. 2015;2015. https://doi.org/10.1155/2015/948262

Zhen J, Villani TS, Guo Y, Qi Y, Chin K, Pan MH, Ho CT, Simon JE, Wu Q. Phytochemistry, antioxidant capacity, total phenolic content and anti-inflammatory activity of *Hibiscus sabdariffa* leaves. Food chemistry. 2016 Jan 1;190:673-80.

Zheng H. Effect of Selfheal soup combined benzene sulfonic acid amlodipine on elderly patients with hypertension and its effect on plasma endothelin-1. Chinese Journal of Primary Medicine and Pharmacy. 2018 Jan 1;25(20):2624-7. doi: 10.3760/cma.j.issn.1008－6706.2018.20.010

Zheng S. Protective effect of *Polygonatum sibiricum* Polysaccharide on D-galactose-induced aging rats model. Scientific Reports. 2020 Feb 10;10(1):1-3.https://doi.org/10.1038/s41598-020-59055-7

Zhou H, Ye M, Xu W, Yu M, Liu X, Chen Y. DL-3-n-butylphthalide therapy for Parkinson's disease: A randomized controlled trial. Experimental and therapeutic medicine. 2019 May 1;17(5):3800-6. doi: 10.3892/etm.2019.7397.

Zhou J, Yang WS, Suo DQ, Li Y, Peng L, Xu LX, Zeng KY, Ren T, Wang Y, Zhou Y, Zhao Y. *Moringa oleifera* seed extract alleviates scopolamine-induced learning and memory impairment in mice. Frontiers in pharmacology. 2018 Apr 24;9:389. doi:10.3389/fphar.2018.00389

Zhou Y, Cao ZQ, Wang HY, Cheng YN, Yu LG, Zhang XK, Sun Y, Guo XL. The anti-inflammatory effects of Morin hydrate in atherosclerosis is associated with autophagy induction through cAMP signaling. Molecular nutrition & food research. 2017 Sep;61(9):1600966. https://doi.org/10.1002/mnfr.201600966

Zhu G, Wang X, Chen Y, Yang S, Cheng H, Wang N, Li Q. Puerarin protects dopaminergic neurons against 6-hydroxydopamine neurotoxicity via inhibiting apoptosis and upregulating glial cell line-derived neurotrophic factor in a rat model of Parkinson's disease. Planta medica. 2010 Nov;76(16):1820-6. DOI: 10.1055/s-0030-1249976

Zhu M, Rajamani S, Kaylor J, Han S, Zhou F, Fink AL. The flavonoid baicalein inhibits fibrillation of α-synuclein and disaggregates existing fibrils. Journal of Biological Chemistry. 2004 Jun 25;279(26):26846-57.

Zhu Y, Bickford PC, Sanberg P, Giunta B, Tan J. Blueberry opposes beta-amyloid peptide-induced microglial activation via inhibition of p44/42 mitogen-activation protein kinase. *Rejuvenation Res*. 2008;11(5):891-901. doi: 10.1089/rej.2008.0757.

Zia-Ul-Haq M, Khan BA, Landa P, Kutil Z, Ahmed S, Qayum M, Ahmad S. Platelet aggregation and anti-inflammatory effects of garden pea, Desi chickpea and Kabuli chickpea. Acta poloniae pharmaceutica. 2012;69(4):707-11.

Zohri AN, Abdel-Gawad K, Saber S. Antibacterial, antidermatophytic and antitoxigenic activities of onion (*Allium cepa* L.) oil. Microbiological research. 1995 May 1;150(2):167-72.https://doi.org/10.1016/S0944-5013(11)80052-2

Zou K, Zhu S, Meselhy MR, Tohda C, Cai S, Komatsu K. Dammarane-Type Saponins from *Panax japonicus* and Their Neurite Outgrowth Activity in SK-N-SH Cells. Journal of natural products. 2002 Sep 27;65(9):1288-92. doi.org/10.1021/np0201117

Zouari Bouassida K, Makni S, Tounsi A, Jlaiel L, Trigui M, Tounsi S. Effects of *Juniperus phoenicea* Hydroalcoholic Extract on Inflammatory Mediators and Oxidative Stress Markers in Carrageenan-Induced Paw Oedema in Mice. Biomed Res Int. 2018 Jul 9;2018:3785487. doi: 10.1155/2018/3785487.

**Databases**

Africa Prelude Medicinal Plant Database http://www.metafro.be/prelude Noe N, Lehmann J (2012). Prelude Medicinal Plants Database. Belgian Biodiversity Platform. Checklist dataset https://doi.org/10.15468/t3kua6 accessed via GBIF.org on 2020-10-08.

Native American Ethnobotany http://herb.umd.umich.edu/
