# Supplementary material for: Therapeutic roles of plants for 15 hypothesised causal bases of Alzheimer’s disease
Source: Nat Prod Bioprospect. 2022 Aug 23;12(1):34. doi: 10.1007/s13659-022-00354-z (PMC9395556; doi:10.1007/s13659-022-00354-z)
Supplement: Supplementary file 9 — Additional file 9. File S1. Materials and Methods further details. [file 13659_2022_354_MOESM9_ESM.pdf]

Additional File S1. Materials and Methods further details.

Inclusion/exclusion criteria

Survey publications were evaluated for inclusion using the Preferred Reporting Items for Systematic Reviews and Meta-Analyses tool (PRISMA) (Moher et al., 2009). Publications were included which contained the following: data of potential relevance to ND treatment; evidence of accurate identification of plant specimens by botanical specialists and/or collection and comparison with species identified in herbaria; and published in the English language. Publications excluded were studies of primarily veterinary significance; surveys with no data of relevance for ND therapeutic potential; studies in which species were selected according to validation by bioactivity reports; and studies without evidence of correct identification of species.

Species within publications were excluded from the surveys for any of the following: reports of serious toxicity; no data of relevance for ND therapeutic potential; and vague/ambiguous description of symptoms. Since the aim was to determine plants with ND therapeutic potential, species used in therapies including animal and/or mineral parts were also excluded. Species with toxicity reports which were less serious (indicated in Supplementary Table 1) were included if the species had potentially important therapeutic effects.

Taxonomy

Species names, attributions and current family status were checked according to the Kew science online medicinal plants checklist (https://mpns.science.kew.org), the Kew Plant list (http://www.theplantlist.org), Kew Plants of the World Online (https://powo.science.kew.org) and PlantZAfrica (http://pza.sanbi.org/) in order to establish the current accepted scientific name. However, species reported in the literature which are now synonyms according to the above checklists were also included in the results (Supplementary Table 1), according to their currently accepted species name, with the synonym added. Potential errors were resolved according to the most plausible accepted name. For instance, *Alternanthera brasivine* (L.) Kuntze (as reported by Ribeiro et al., 2017) was not traceable in the checklists, but the common name (in this case, Terramicina, is suggestive of the currently accepted species *Alternanthera brasiliana* (L.) Kuntze. If bioactivity reports were not found to validate the ethnological use for a species, ND-relevant bioactivities found for sister species (species within the same genus) were included, on the basis that targeting close relatives of plants with a demonstrated bioactivity is a useful screening strategy to find novel plants with high therapeutic potential (Atanasov et al., 2015).
